# Geographic differentiation of the gut microbiota in *Lissorhoptrus oryzophilus* (Coleoptera: Curculionidae): associations with environmental gradients and predicted functional potential

**DOI:** 10.1093/jisesa/ieag111

**Published:** 2026-10-09

**Authors:** Jian-Xue Jin, Zhao-Chun Ye, Gui-Fen Zhang, Yu Wang, Zhao-Chun Jiang, Fei-Li Lan, Yong-Fu He

**Affiliations:** Guizhou Key Laboratory of Agricultural Biosecurity, The Institute of Plant Protection, Guizhou Academy of Agricultural Sciences, Guiyang, China; Guizhou Branch of State Key Laboratory for Biology of Plant Diseases and Insect Pests, The Institute of Plant Protection, Guizhou Academy of Agricultural Sciences, Guiyang, China; Guizhou Provincial Laboratory of Green Technology and Application Engineering of Plant Protection, The Institute of Plant Protection, Guizhou Academy of Agricultural Sciences, Guiyang, China; Guizhou Key Laboratory of Agricultural Biosecurity, The Institute of Plant Protection, Guizhou Academy of Agricultural Sciences, Guiyang, China; Guizhou Branch of State Key Laboratory for Biology of Plant Diseases and Insect Pests, The Institute of Plant Protection, Guizhou Academy of Agricultural Sciences, Guiyang, China; Guizhou Provincial Laboratory of Green Technology and Application Engineering of Plant Protection, The Institute of Plant Protection, Guizhou Academy of Agricultural Sciences, Guiyang, China; State Key Laboratory for Biology of Plant Diseases and Insect Pests, Key Laboratory for Prevention and Control of Invasive Alien Species of Ministry of Agriculture and Rural Affairs, Institute of Plant Protection, Chinese Academy of Agricultural Sciences, Beijing, China; Guizhou Key Laboratory of Agricultural Biosecurity, The Institute of Plant Protection, Guizhou Academy of Agricultural Sciences, Guiyang, China; Guizhou Branch of State Key Laboratory for Biology of Plant Diseases and Insect Pests, The Institute of Plant Protection, Guizhou Academy of Agricultural Sciences, Guiyang, China; Guizhou Provincial Laboratory of Green Technology and Application Engineering of Plant Protection, The Institute of Plant Protection, Guizhou Academy of Agricultural Sciences, Guiyang, China; Guizhou Plant Protection Station, Guiyang, China; College of Biological and Agricultural Sciences (College of Food Science and Technology), Zunyi Normal University, Zunyi, China; Guizhou Key Laboratory of Agricultural Biosecurity, The Institute of Plant Protection, Guizhou Academy of Agricultural Sciences, Guiyang, China; Guizhou Branch of State Key Laboratory for Biology of Plant Diseases and Insect Pests, The Institute of Plant Protection, Guizhou Academy of Agricultural Sciences, Guiyang, China; Guizhou Provincial Laboratory of Green Technology and Application Engineering of Plant Protection, The Institute of Plant Protection, Guizhou Academy of Agricultural Sciences, Guiyang, China

**Keywords:** *Lissorhoptrus oryzophilus*, gut microbiota, metagenome, predictive functional profiling, environmental gradient

## Abstract

The rice water weevil, *Lissorhoptrus oryzophilus* (Kuschel), is a major invasive pest native to rice that has expanded far beyond its native North American range to establish widespread populations across Asia and Europe. However, how altitudinal gradients shape this microbial ecosystem remains unclear. We performed shotgun metagenomics to profile the gut microbiota of 3 geographically distinct populations—Pingba District (PB), Yibin City (SC), and Mudanjiang (MDJ) City—of *L. oryzophilus* across elevational gradients in China. Though low-abundance archaeal, fungal, and viral sequences were detected, bacterial taxa (>89% of annotated genes) dominated functional profiles. All populations were Proteobacteria-dominated, but PB was enriched in Erwiniaceae (*Pantoea deleyi*), while SC/MDJ were dominated by Rickettsiaceae (*Rickettsia/Wolbachia*). Population-specific taxa were most abundant in MDJ (19 species), followed by PB (15), with SC harboring only 1 unique species. Predicted functional profiles aligned with taxonomy: PB showed elevated amino acid/carbohydrate metabolism, carbohydrate-active enzyme, and efflux pump gene abundances. SC and MDJ shared similar functional profiles. Principal coordinates analysis revealed distinct clustering of PB across taxonomic and functional dimensions, while SC/MDJ partially overlapped. These correlative findings suggest microbial metabolic plasticity may support the weevil’s persistence across heterogeneous environments, though causal links require experimental validation.

## Graphical Abstract

**Graphical Abstract:**
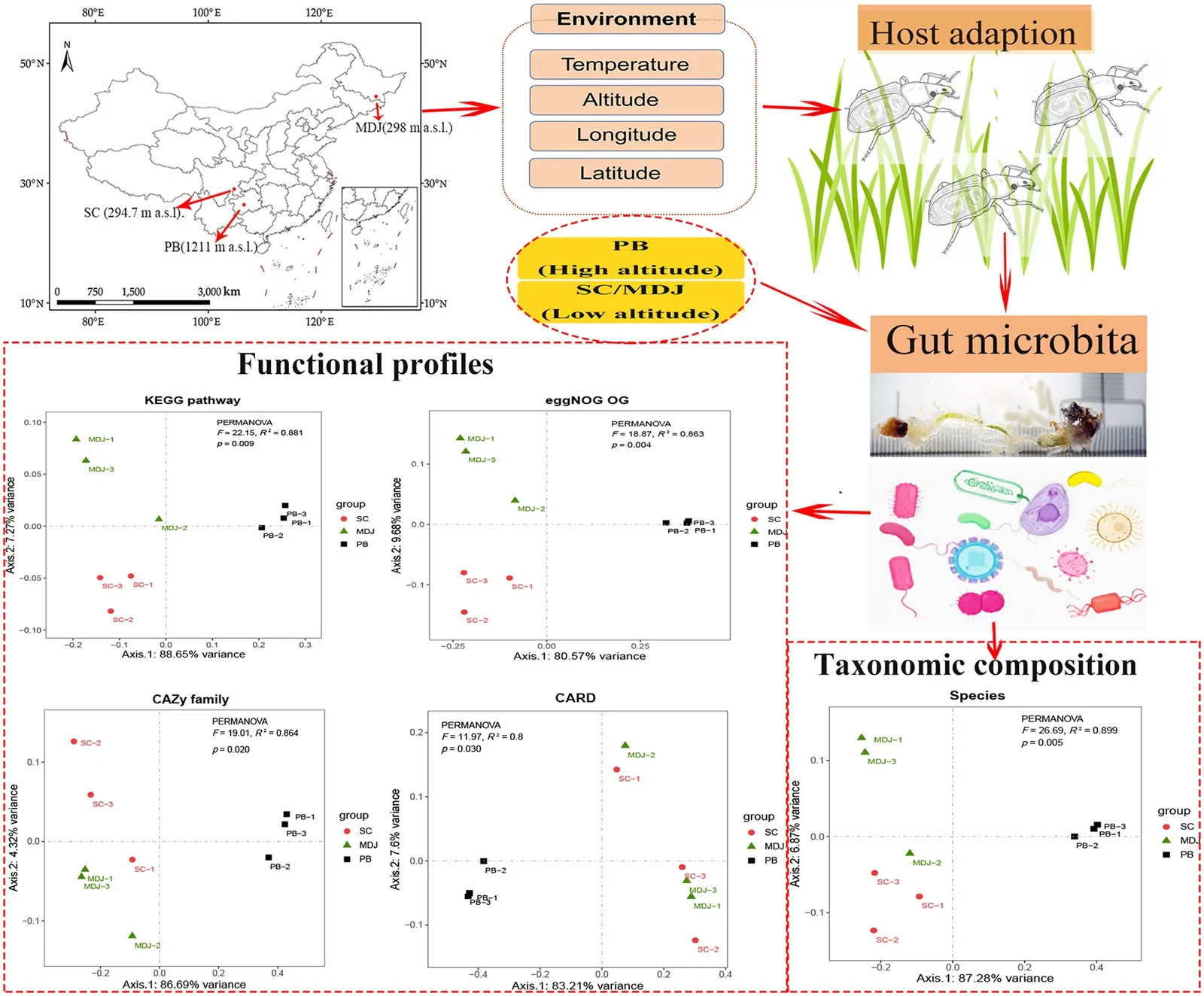

## Introduction

The gut microbiome, acting as a “second genome” plays a pivotal role in host nutrient metabolism, immune regulation, and stress resistance—a perspective supported by numerous studies (Brune 2014, Douglas 2015, Huerta-García and Álvarez-Cervantes 2024). In recent years, research on insect–microbe interactions has emerged as a core focus in invasion biology (Chen et al. 2023, 2025, Coolen et al. 2024, Li et al. 2024). Geographic variation in gut microbiota contributes to host adaptation to environmental gradients, acting in concert with host genetic divergence and phenotypic plasticity. In insects, microbial communities can rapidly respond to local selection pressures (e.g. temperature, diet), providing metabolic flexibility that enhances host fitness (Wang et al. 2023, Dong et al. 2024, Li et al. 2024).

Many studies indicate that invasive insects frequently enhance host adaptability via the enrichment of functional genes (e.g. metabolic enzymes, resistance genes) in their gut microbiota to overcome novel environmental filters. The functional diversity of gut microbiota is predominantly governed by the repertoire of functional genes. For instance, the expansion of carbohydrate-active enzyme (CAZy) gene families (e.g. glycoside hydrolase [GH] families GH28 and GH45) in the Colorado potato beetle (*Leptinotarsa decemlineata*) enables efficient degradation of complex carbohydrates (e.g. cellulose, pectin) in Solanaceous plant foliage, providing a critical carbon acquisition strategy for hosts feeding on Solanaceous plants. Such functional gene expansion is not restricted to host genomes; gut microbiota may also acquire adaptive genes via horizontal gene transfer (HGT) (Schoville et al. 2018). Empirical evidence across diverse taxa underscores the prevalence of this phenomenon. In the invasive woodwasp, *Sirex noctilio*, gut microbiome diversity and functional profiles (e.g. carbohydrate and terpenoid metabolism) shift significantly across latitudes, e.g. Daqing City and Hegang City of Heilongjiang Province, Tongliao City of the Inner Mongolia Autonomous Region, and Yushu City of Jilin Province, indicating that other environmental factors, such as temperature, precipitation, are positively correlated with most of the highly abundant bacterial and fungal genera. The study reveals the potential role of changes in *S. noctilio* larval gut microbiome in the successful colonization and better adaptation of the environment (Li et al. 2024). Similarly, for *Hylurgus ligniperda*, temperature variations induced specific structural changes in the gut bacterial community. Notably, the relative abundance of *Rahnella* increased by more than 10% during the cold period, and it maintained stable production of proteases and lipases under low temperatures—a trait that may be crucial for supporting host development in cold environments (Chen et al. 2025). Analogous patterns are observed in the different populations of *Ceracris kiangsu* (yellow-spined bamboo locust), where geographic location overrides host sex as the primary determinant of core microbiota composition (e.g. *Lactococcus* vs. Enterobacter dominance), mediated by environmental factors like humidity and atmospheric pressure (Yang et al. 2023). At broader spatial scales, *Apis cerana* (Chinese honey bee) and *Apis mellifera* (European honey bee) across a 1,000-km range in China revealed that host species shape distinct microbial community structures by modifying assembly processes (e.g. homogeneous selection, random drift). Honey bee gut microbiota exhibit a clear geographic gradient, underscoring geographic distance as a key driver of large-scale microbial differentiation (Ge et al. 2021). Even within social corbiculate bees in the subfamily Apinae like carpenter bees (*Xylocopa* spp.), long-read sequencing reveals significant population-level differentiation in core genera (e.g. *Bombilactobacillus* and *Bombiscardovia*), linking microbial biogeography directly to host adaptation (Handy et al. 2023). Collectively, these studies suggest that differentiation of gut microbiota among geographic populations represents a key manifestation of insect adaptation to environmental heterogeneity.

*Lissorhoptrus oryzophilus* (Kuschel), a major invasive rice pest native to North America (Kuschel 1951), ranks as a key pest across its native North American range and invaded agricultural systems throughout Asia and Europe (Heinrichs and Quisenberry 1999). It has since become widely established in all major rice-producing regions of China, as well as in Japan and South Korea, causing average annual rice yield losses of 10% to 25% and up to 70% in severe infestations, thereby posing a significant threat to global food security (Zou et al. 2004, Reay-Jones et al. 2008). Larvae of this pest bore into rice roots, while adults feed on leaves. Since its introduction to Tangshan, Hebei Province, China, in 1988, *L. oryzophilus* has rapidly expanded its range. As of July 2025, its distribution has extended to 25 provinces (autonomous regions and municipalities directly under the central government) and 516 counties (cities, districts, and banners) (Ministry of Agriculture and Rural Affairs of the People’s Republic of China 2025), impacting 6 major rice-cultivation regions in Northeast, Northwest, North, Southwest, Central, and South China. China spans ∼50° of latitude, encompassing 5 temperature zones (frigid temperate, mid-temperate, warm temperate, subtropical, and tropical) and the Qinghai-Tibet Plateau alpine region. The distribution of *L. oryzophilus* covers all these zones except the frigid temperate zone and the Qinghai-Tibet Plateau, ranging from the mid-temperate zone (Heilongjiang Province) in the north to tropical regions (Yunnan Province, Guangdong Province, and Taiwan Province) in the south. Emerging evidence further suggests that gut microbiota-mediated functional plasticity acts as a critical facilitative mechanism for this host-level adaptation, enabling populations to buffer environmental stress and exploit variable nutritional resources (Hodkinson et al. 1999, Jenkins and Hoffmann 1999, Lee 2002, Chen and Kang 2002).

Building on early culture-dependent studies, Lu et al. (2013) identified γ-Proteobacteria and Bacilli as the dominant cultivable taxa within the midgut of *L. oryzophilus*, reporting relatively low diversity and notable geographic variation between Chinese and US populations. Subsequent culture-independent surveys (Lu et al. 2014) corroborated these biogeographic patterns, detecting *Pantoea* and *Wolbachia* across all examined weevil populations while documenting significant geographic variation in community richness, diversity, and evenness. Recent shotgun metagenomic analyses corroborate these structural findings while extending insights into functional potential. Jin et al. (2025) confirmed the persistence of a *Pantoea*-dominated Proteobacteria community and revealed that these symbionts are primarily enriched in metabolic pathways crucial for herbivory, including carbohydrate and amino acid digestion, as well as the biodegradation of xenobiotics. However, the extent to which these functional profiles diverge across geographic populations—and the underlying mechanisms driving such differentiation—remain insufficiently understood. We hypothesize that altitude-driven environmental filtering (e.g. low temperature, hypoxia) shapes gut microbiota composition and function, promoting high-altitude host adaptation by enhancing metabolic flexibility. *L. oryzophilus*, with its broad latitudinal range in China, offers an ideal model to investigate these processes. To test this hypothesis, we focused on 3 representative geographic populations in China: Pingba (PB) District (Guizhou Province, 1,211 m a.s.l. high-altitude plateau), Yibin City (Sichuan Province, SC, 294.7 m a.s.l. basin hinterland), and Mudanjiang (MDJ) City (Heilongjiang Province, 298 m a.s.l. northeastern plain). Integrating metagenomic sequencing, multi-database functional annotation (Kyoto Encyclopedia of Genes and Genomes [KEGG], evolutionary genealogy of genes: Non-supervised Orthologous Groups [eggNOG], CAZy, CARD [Comprehensive Antibiotic Resistance Database]), and principal coordinates analysis (PCoA), we addressed 3 questions: (i) Do gut microbial compositions differ among geographic populations? (ii) How do the abundance and type of functional genes (e.g. metabolic, resistance) change with altitude? (iii) Does this differentiation reflect host evolutionary adaptation to the environment? Our results provide new evidence for the geographic pattern of insect–microbe coevolution and deepen understanding of invasive pest adaptation mechanisms.

## Materials and Methods

### Study Sites and Environmental Gradients

Three sampling sites were selected to capture a steep environmental gradient spanning latitude and altitude in China (Fig. 1). PB, Guizhou (26.4244°N, 106.3303°E): Located on the Yunnan-Guizhou Plateau, this site is characterized by a subtropical plateau monsoon climate with a Mean Annual Temperature (MAT) of 14.81 °C and mild winters (mean January temperature: 4.55 °C). SC, Sichuan Basin (29.0117°N, 104.5795°E): This site features a subtropical humid basin climate (MAT: 17.72 °C; mean January temperature: 7.5 °C). MDJ, Heilongjiang (44.4946°N, 129.7117°E): Situated in Northeast China, this site has a temperate continental climate with a MAT of 4.08 °C and harsh winters (mean January temperature: −16.98 °C). MAT and mean January temperature were obtained from WorldClim (www.worldclim.org; Fick and Hijmans 2017; Table 1).

**Fig. 1.**
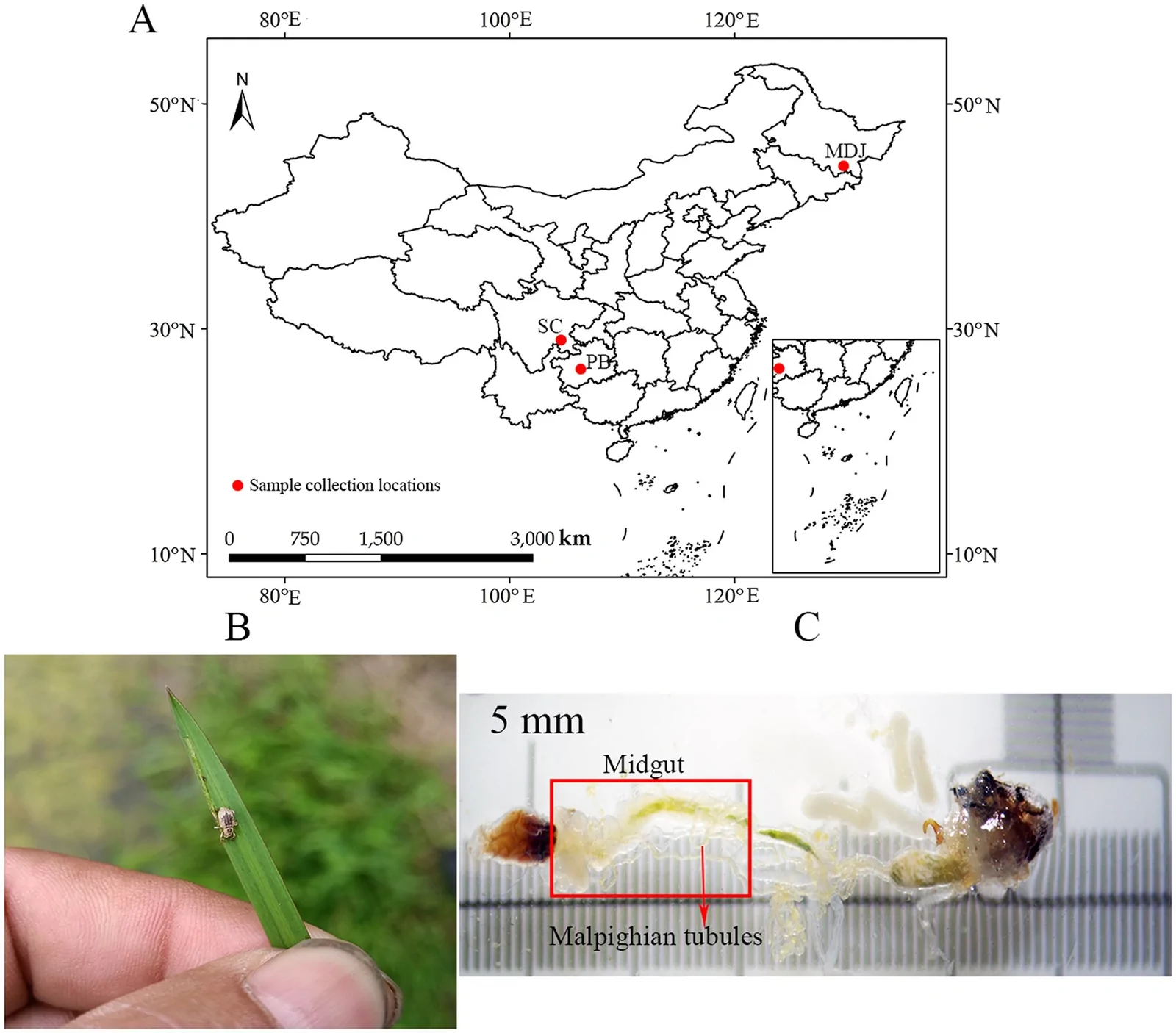
Geographic locations of *Lissorhoptrus oryzophilus* adult collection sites in natural habitats. (A) Collection sites in 3 different locations. (B) Adult *L. oryzophilus* feeding on rice leaves in the field. (C) Midgut of *L. oryzophilus* (Scale bar = 5 mm, the midgut is approximately 2 mm long inside the red rectangle. The midgut region used in this experiment excludes the Malpighian tubules).

**Table 1.** Environmental characteristics of the 3 sampling locations for *Lissorhoptrus oryzophilus*

Parameter	PB (Pingba, Guizhou)	SC (Yibin, Sichuan)	MDJ (Mudanjiang, Heilongjiang)
**Altitude (m.a.s.l.)**	1,211	294.7	298
**Mean Annual Temp.**	14.81 °C	17.72 °C	4.05 °C
**January mean temp.**	4.55 °C	7.5 °C	−16.98 °C
**The warmest quarter mean temp.**	22.43 °C	26.02 °C	20.35 °C
**The coldest quarter mean temp.**	6.02 °C	8.60 °C	−14.42 °C
**Growing degree days**	4,500 (effective)	5,200 (effective)	2,500 (effective)
**Atmospheric O_2_ pressure**	87 kPa (approx. 87% sea level)	95 kPa (approx. 95% sea level)	98 kPa (near sea level)
**Climate type**	Subtropical highland/humid monsoon	Subtropical humid monsoon	Temperate continental monsoon
**Key environmental stressors**	Low temp. variability, hypoxia, intense UV	High humidity, heat stress	Extreme winter cold, seasonal diapause

### Insect Collection

Overwintering adults of *L. oryzophilus* were collected from rice nursery seedlings at the 3 geographically distinct locations. Upon collection, adults were transferred to the laboratory and maintained in climate-controlled cages (30 × 30 × 30 cm) at ambient temperature (∼25 °C). Adults were provided with fresh, potted rice seedlings ad libitum for 2 to 3 days. To maintain hydration, seedling roots were wrapped in moist absorbent cotton and placed in water-saturated trays. Prior to gut dissection, approximately 600 adults from each population were starved for 24 h to evacuate the alimentary tract contents.

### Gut Dissection and Sample Preparation

All dissection procedures were performed under strict aseptic conditions. Dissection instruments (fine forceps and insect pins) were sterilized by flaming after immersion in 100% ethanol. Phosphate-buffered saline (PBS; 0.01 M, pH 7.4) was pre-chilled to 0 °C. Individual adults were surface-sterilized by sequential immersion in 75% ethanol for 1 min, followed by 2 rinses in sterile distilled water (Chen et al. 2023, Siddiqui et al. 2025). Each adult was then placed in a drop of pre-chilled PBS on a sterile Petri dish. Under a stereomicroscope (model: ZSA400), the abdomen was carefully opened, and the midgut was excised intact and transferred to a sterile microcentrifuge tube containing 500 μl of pre-chilled PBS. All dissections were conducted on ice to minimize nucleic acid degradation.

For each geographical population (SC, MDJ, PB), 3 independent biological replicates were prepared (Coolen et al. 2024, Siddiqui et al. 2025). Each replicate consisted of a pool of midguts dissected from 200 adults, yielding a total of 9 samples (SC-1 to SC-3, MDJ-1 to MDJ-3, and PB-1 to PB-3). Following dissection, PBS was aspirated from the microcentrifuge tube using a pipette. Gut samples were immediately flash-frozen in liquid nitrogen and stored at −80 °C until DNA extraction. Metagenomic DNA extraction, library construction, sequencing, and primary bioinformatics analyses were performed by Wuhan Generead Biotechnologies Co., Ltd. (Wuhan, China). Throughout the procedure, operators wore surgical masks and gloves, and instruments were promptly disinfected with 75% ethanol following any contact with non-sterile surfaces to preclude contamination from human skin microbiota or saliva.

### Gut Microbiota DNA Extraction

Metagenomic DNA was extracted using a cetyltrimethylammonium bromide (CTAB)-based standard protocol. Briefly, samples were homogenized in 2% (w/v) CTAB solution (Solarbio, Beijing, China) using grinding beads (in the absence of proteinase K) and incubated at 65 °C for 1 h. An equal volume of phenol:chloroform:isoamyl alcohol (25:24:1, v/v/v; Solarbio, Beijing, China) was added to facilitate phase separation and purification. After centrifugation at 12,000 × *g* for 10 min at 4 °C, the upper aqueous layer was transferred to a new microcentrifuge tube. DNA was precipitated by adding an equal volume of isopropanol, incubating at ambient temperature for 10 min, and centrifuging at 12,000 × *g* for 10 min at 4 °C. The resulting pellet was washed twice with 75% (v/v) ethanol, briefly air-dried, and resuspended in 50 μl of TE buffer (containing RNase A; Solarbio, Beijing, China). Samples were then incubated at 37 °C for 30 min to degrade RNA contaminants.

DNA concentration and purity were assessed using a NanoDrop spectrophotometer (Thermo Fisher Scientific, Waltham, Massachusetts, United States). High-quality DNA suitable for downstream sequencing was defined by 260/280 nm absorbance ratios of 1.8 to 2.0 and 260/230 nm ratios of 1.6 to 2.4 (Supplementary Table S1). DNA integrity was further verified by 1.0% (w/v) agarose gel electrophoresis, and all were classified as grade “A.”

### Metagenomic Sequencing and Bioinformatics Pipeline

Sequencing libraries were constructed using the VAHTS Universal Plus DNA Library Prep Kit (Vazyme, Nanjing, China; Cat. NDM627, lot WF2024052310) following the manufacturer’s protocol. In brief, high-quality metagenomic DNA was fragmented mechanically sheared by ultrasonication according to the kit protocol, followed by combined end-repair and 3′-adenylation (A-tailing). Indexed adapters supplied with the kit were then ligated to the A-tailed fragments prior to PCR amplification. The ligation products were enriched via PCR using index primers provided in the kit. To ensure high library quality, size selection was performed by excising the target band (250 to 350 bp) from a 1.5% low-melting-point agarose gel, followed by gel extraction using the E.Z.N.A. Gel Extraction Kit (Omega Bio-tek). Final library quality was assessed using an Agilent Bioanalyzer 2100 to verify insert size distribution and the absence of adapter dimers before sequencing on the Illumina NovaSeq X Plus platform.

Raw reads were quality-filtered using Trimmomatic (v0.39) to remove adapters and low-quality bases, generating high-quality clean reads. Clean reads were aligned to the host (*L. oryzophilus*) reference genome (Accession: PRJNA1474746) using Bowtie2 (v2.5.4) to eliminate host-derived contamination. De novo assembly of filtered reads was performed with MEGAHIT (v1.2.9) to generate contigs, and assembly quality was evaluated using QUAST (v5.3.0). Open reading frames (ORFs) were predicted using MetaGeneMark (v3.26), and predicted gene sequences were clustered with CD-HIT (v4.6.6) (90% coverage, 95% similarity threshold) to yield a non-redundant (Nr) gene catalog.

### Taxonomic and Functional Profiling

Taxonomic annotation was carried out by aligning high-quality clean reads to the NCBI Nr protein database via DIAMOND BLASTP (v2.17.0). Taxonomic annotation was performed by aligning predicted protein sequences against the NCBI Nr protein database using DIAMOND BLASTP (v2.17.0). To ensure annotation stringency and mitigate false positives arising from the variable quality of Nr entries, we applied a multi-tiered filtering criterion: an *E*-value threshold of ≤1 × 10^−5^. For queries returning multiple qualified hits, only the alignment with the highest bit-score was retained for taxonomic assignment. Sequences failing to meet these thresholds, or those mapping exclusively to “unclassified,” were categorized as “Unclassified” to prevent misleading ecological inferences.

Functional annotation utilized a composite database comprising KEGG, eggNOG, CAZy (Carbohydrate-Active enZYmes, CAZymes), and CARD. For KEGG pathway annotation, protein sequences were aligned to the KEGG database using DIAMOND blastp (*E*-value ≤1 × 10^−5^). And the top hit was assigned as the KEGG Orthology (KO) identifiers. For eggNOG annotation, sequences were aligned against the eggNOG database (DIAMOND blastp; *E*-value ≤1 × 10^−5^) to infer broader functional categories (e.g. COG classes). CAZymes were identified by scanning protein sequences against the CAZy database using HMMER (hmmscan, v3.3.2) with stringent thresholds (*E*-value <1 × 10^−15^, coverage ≥35%). Antibiotic resistance genes (ARGs) were annotated by aligning sequences to the CARD database (DIAMOND blastp; *E*-value ≤1 × 10^−5^), retaining only the best hit for high-confidence assignment.

### Statistical Analysis

Prior to multivariate ordination, all abundance matrices (taxonomic, KEGG, eggNOG, CAZy, and CARD) were normalized to Transcripts Per Million (TPM) values to correct for inter-sample variation in sequencing depth and reference gene length. To mitigate skewness from zero-inflated metagenomic data, TPM values were log_10_-transformed using the formula log_10_ (TPM + 1). Features present in fewer than 2 samples were filtered out to reduce technical noise. For relative abundance bar plots, the top 20/30 features were similarly selected from the relative abundance table and visualized using the ggplot2 package in R. Heatmaps were generated based on the TPM-normalized abundance table; the top 30 features were selected for visualization using the pheatmap package in R, with data scaled by *Z*-score scaled by row to standardize expression levels across samples. The core microbiome at the functional level was defined as unigenes shared across all 3 geographic populations. Venn diagrams were constructed using the VennDiagram package in R to delineate the distribution of shared and population-specific (unique) unigenes. To ensure robustness, unigenes were required to have a minimum count of 2 within at least 1 sample group, and only those with significant NR database hits (E-value <1e^−5^) were retained for visualization.

To resolve variations in gut microbial taxonomic and functional profiles across geographic populations of *L. oryzophilus*, we adopted PCoA (also referred to as metric multidimensional scaling), a distance-based ordination method capable of capturing non-linear patterns inherent to sparse, zero-inflated metagenomic data, as the primary approach for beta diversity analysis. All abundance matrices were first normalized to TPM values to correct for inter-sample variation in sequencing depth. To further mitigate skewness from zero-inflated distributions and reduce the influence of extremely high-abundance features, TPM values were log^10^ (TPM + 1) transformed prior to distance calculation. Bray–Curtis dissimilarity, a metric that accounts for both the presence/absence and relative abundance of features, was selected as the distance measure and computed from the transformed abundance matrix using the vegdist() function in the vegan package (v2.6.4) in R (v4.3.0). Eigen decomposition of the resulting Bray–Curtis distance matrix was performed using the pcoa() function in the ape package (v5.7.1) to derive orthogonal coordinate axes (PCo1, PCo2) that explain the maximum proportion of total multivariate variation. Ordination results were visualized as scatterplots using the ggplot2 package (v3.4.4). To statistically validate the observed ordination patterns, permutational multivariate analysis of variance (PERMANOVA) was implemented using the adonis2() function in vegan with 999 random permutations, which outputs pseudo-*F* values, *R*² (the proportion of variation explained by geographic population), and associated *P-values* to assess the significance of group differences.

## Results

### Metagenomic Sequencing, Assembly, and Gene Annotation

Paired-end sequencing of 9 gut metagenomic libraries generated 485,172 to 792,488 raw reads per sample. Following quality filtering with Trimmomatic, >95.86% of reads were retained as high-quality clean reads across all samples. Sequencing quality was uniformly high: Phred quality scores ≥ Q20 and ≥ Q30 accounted for >98.70% and >95.28% of total bases, respectively, with a low per-base error rate (∼0.1%) and GC content ranging from 33.68% to 47.08%—consistent with typical microbial genome profiles (Supplementary Table S2). All raw sequencing data have been deposited in the NCBI Sequence Read Archive under the following BioProject accessions: PB, PRJNA1439573; SC, PRJNA1442719; and MDJ, PRJNA1442367.

Metagenomic assembly of quality-filtered reads yielded 2,900 to 4,966 genes per sample, with total assembly lengths of 2,997,412 to 7,109,211 bp. Assembly continuity was robust, with an N50 > 900 bp, and GC content ranged from 30.57% to 53.75%, aligning with expected values for insect gut microbial consortia (Supplementary Table S3). ORF prediction identified 2,242 to 7,246 protein-coding genes per sample, with GC content of 35.07% to 55.85%.

To eliminate cross-population redundancy, a global Nr gene catalog was constructed via CD-HIT clustering, comprising 11,651 unigenes. Of these, 64.29% harbored complete ORFs with both start and stop codons, while 1.49% lacked both. The catalog had a total gene length of 7,024,143 bp, with an average length of 602.88 bp (range: 102 to 17,046 bp). Assembly completeness metrics for the catalog were favorable: N50 = 966 bp, L50 = 2,332, and overall GC content = 49.00% (Supplementary Table S4). Collectively, these quality-control metrics confirm the reliability of the dataset for downstream functional profiling of the *L. oryzophilus* gut microbiome.

### Taxonomic Composition of Gut Microbiota

With the high-quality Nr gene catalog established, we next characterized the taxonomic and functional profiles of the *L. oryzophilus* gut microbiome across the 3 geographic populations. A total of 7,184 high-quality sequences yielded significant matches for taxonomic assignment against the NCBI NR database. Of these 6,728 were classified into 4 canonical domains: Archaea, Bacteria, Fungi, and Viruses, with Bacteria overwhelmingly dominating the community. Taxonomic assignment was resolved across 6 hierarchical levels: phylum, class, order, family, genus, and species. The remaining 456 unigenes lacked clear taxonomic affiliation and were categorized as “unclassified” (Fig. 2A; Supplementary Table S5).

**Fig. 2.**
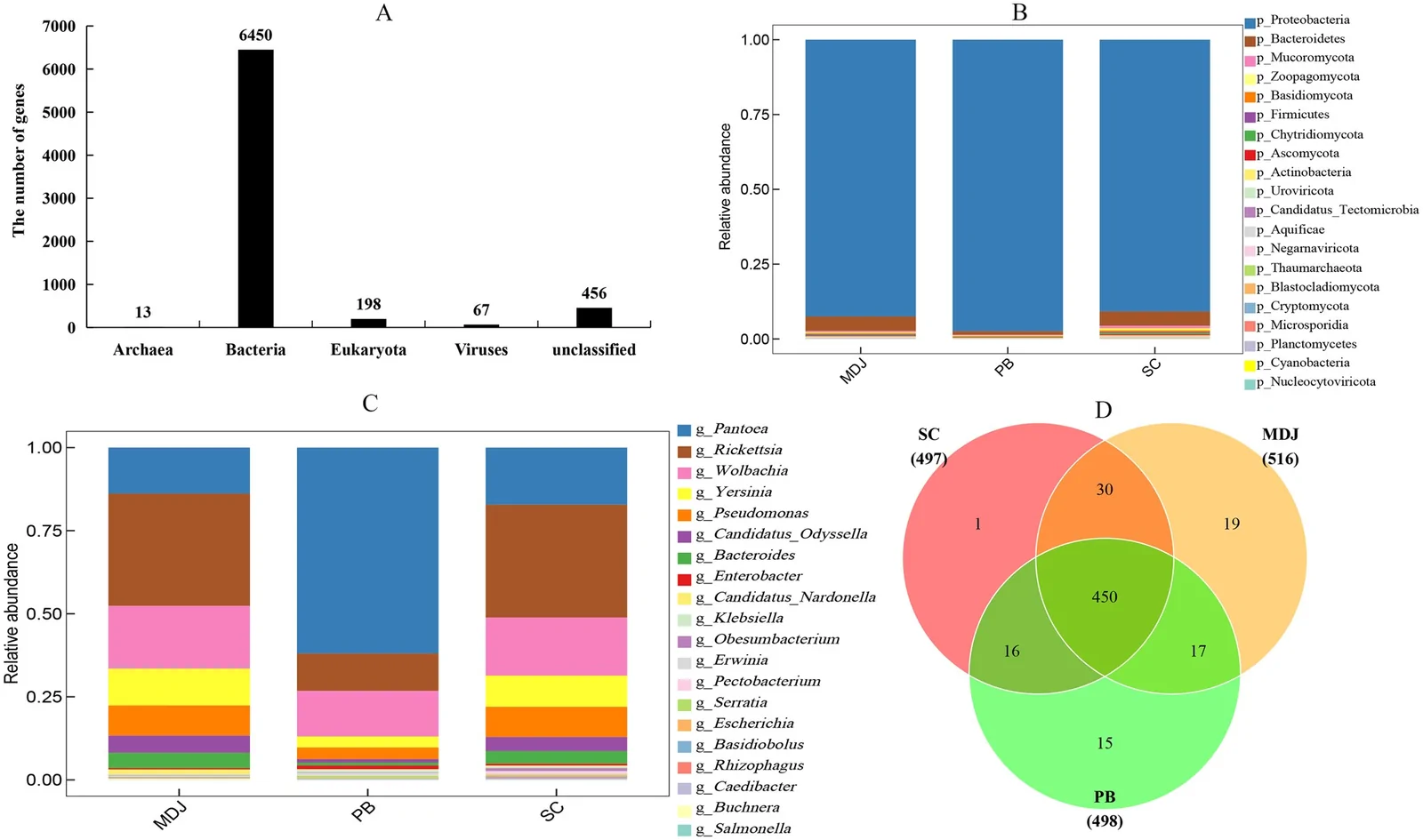
Relative taxonomic composition of the gut microbiota across geographic populations of *L. oryzophilus*. (A) Bar chart showing the number of Nr-annotated genes across the 4 canonical domains (Archaea, Bacteria, Eukaryota, and Viruses) and unclassified sequences. (B) Relative abundance of the top 20 phyla in 3 populations (MDJ, PB, and SC). (C) Relative abundance of the top 20 genera in 3 populations (MDJ, PB, and SC); each stacked bar segment represents the percentage abundance of a specific taxon within the gut microbiota of each population. (D) Venn diagram illustrating the overlap of bacterial species detected across the 3 geographic populations (MDJ, PB, and SC). Taxonomic profiles were resolved across 4 domains and 5 hierarchical levels: Phylum, Order, Family, Genus, and Species.

#### Phylum Level

The number of annotated phyla varied among the 3 geographic populations (SC, MDJ, PB): 26 in PB, 27 in SC, and 32 in MDJ. The top 20 most abundant taxa were largely consistent across populations. Proteobacteria was the dominant phylum in all populations, accounting for 82.64% (PB), 61.64% (SC), and 70.15% (MDJ). This was followed by Bacteroidetes (0.99% in PB, 3.25% in SC, 3.66% in MDJ) and Mucoromycota (0.21% in PB, 0.57% in SC, 0.35% in MDJ) (Fig. 2B).

#### Class Level

Within the Proteobacteria, Gammaproteobacteria and Alphaproteobacteria were the most abundant classes, though their rank order shifted geographically. In PB, Gammaproteobacteria predominated (61.10%), followed by Alphaproteobacteria (21.40%), Bacteroidia (0.79%), and Sphingobacteriia (0.17%). Conversely, Alphaproteobacteria dominated in SC (35.41%) and MDJ (41.78%), with Gammaproteobacteria occupying the second rank (26.00% in SC, 28.19% in MDJ). Bacteroidia (2.51% in SC, 3.41% in MDJ) and Sphingobacteriia (0.67% in SC, 0.20% in MDJ) were consistently the third and fourth most abundant classes, respectively.

#### Order Level

Community composition at the order level revealed a distinct shift in dominance between PB and the other populations. Enterobacterales and Rickettsiales were the primary drivers of community structure. In PB, Enterobacterales accounted for the largest proportion (57.78%), followed by Rickettsiales (20.50%). In contrast, SC and MDJ were characterized by a predominance of Rickettsiales (32.70% and 38.02%, respectively), with Enterobacterales ranking second (19.25% in SC, 19.90% in MDJ). Pseudomonadales and Holosporales exhibited higher relative abundances in SC (5.69% and 2.63%, respectively) and MDJ (6.56% and 3.71%, respectively) compared to PB (2.89% and 0.86%).

#### Family Level

Dominant families reflected the trends observed at the order level. In PB, Erwiniaceae (51.11%), Anaplasmataceae (11.18%), and Rickettsiaceae (9.32%) were most prevalent, with notable contributions from Yersiniaceae (3.25%), Pseudomonadaceae (2.82%), Enterobacteriaceae (2.61%), Candidatus Paracaedibacteraceae (0.84%), and Bacteroidaceae (0.73%). In SC, Rickettsiaceae ­dominated (21.87%), followed by Erwiniaceae (10.86%), Anaplasmataceae (10.82%), Yersiniaceae (5.96%), Pseudomonadaceae (5.66%), Candidatus Paracaedibacteraceae (2.62%), Bacteroidaceae (2.37%), and Enterobacteriaceae (1.23%). In MDJ, Rickettsiaceae remained dominant (24.47%), followed by Anaplasmataceae (13.55%), Erwiniaceae (10.36%), Yersiniaceae (8.08%), Pseudomonadaceae (6.52%), Candidatus Paracaedibacteraceae (3.70%), Bacteroidaceae (3.32%), and Enterobacteriaceae (1.07%).

#### Genus Level

The core gut microbiota was characterized by a consortium of *Pantoea*, *Wolbachia*, *Rickettsia*, *Yersinia*, *Pseudomonas*, *Candidatus Odyssella*, *Bacteroides*, and *Enterobacter*. A clear geographic dichotomy emerged. In PB, *Pantoea* was the most abundant genus (50.35%), followed by *Wolbachia* (11.17%), *Rickettsia* (9.13%), *Pseudomonas* (2.82%), and *Yersinia* (2.70%). Conversely, in SC and MDJ, *Rickettsia* dominated (21.09% and 24.28%, respectively), followed by *Wolbachia* (10.82% in SC, 13.54% in MDJ) and *Pantoea* (10.65% in SC, 9.91% in MDJ) as the third most abundant (Fig. 2C).

#### Species Level

At the species level, *Pantoea deleyi* was the dominant bacterium in PB, accounting for 27.17%, followed by *Pantoea* sp. ARC607 (12.78%) and *Rickettsia asembonensis* (5.22%). In contrast, SC and MDJ exhibited higher relative abundances of *R. asembonensis* (7.85% and 12.21%, respectively). *Yersinia pestis* was also notably enriched in SC (5.82%) and MDJ (7.91%) compared to PB.

To resolve the taxonomic overlap and specificity among the 3 geographic populations (MDJ, PB, SC), we performed a Venn diagram analysis based on species-level annotations derived from alignments against the NR Database. The Venn diagram enumerates only unigenes meeting the strict “presence” criterion (detected in ≥2 biological replicates per population), rather than all annotated unigenes (Fig. 2D). A total of 450 unigenes constituted the core microbiome, shared universally across all 3 populations. This core set represented the predominant fraction (∼87%) of the annotated gene catalog in each population, underscoring a conserved taxonomic backbone. In contrast, population-specific (unique) taxonomic markers were comparatively limited: MDJ harbored 19 unique unigenes, PB contained 15, and SC possessed only 1. Pairwise comparisons further revealed distinct sharing patterns: 17 unigenes were uniquely shared between MDJ and PB, 16 between PB and SC, whereas MDJ and SC shared a comparatively larger subset of 30 unigenes. These data delineate a gut microbial landscape characterized by high conservation punctuated by subtle population-level differentiation.

### KEGG Pathway Analysis of Gut Microbiota

To characterize the metabolic potential of the *L. oryzophilus* gut microbiome, the 11,651-gene Nr catalog was aligned to the KEGG database, retaining only the top hit per query to ensure annotation stringency. Two complementary statistical frameworks were applied to contextualize functional abundance: (i) relative proportion within KEGG-annotated genes: used to evaluate the internal distribution of functionally characterized sequences (summing to 100% across all level 1 categories); (ii) relative proportion within total predicted protein-coding genes: used to quantify the absolute contribution of functional pathways to the whole metagenomic gene pool (i.e. the 11,651-gene catalog).

Of the Nr genes, a total of 4,608 (39.5%) genes were successfully annotated and mapped to the KEGG database and it should be noted that a substantial proportion of the metagenomic catalog (60.5%) remained unannotated. These annotated genes mapped to 6 KEGG level 1 functional categories, 45 KEGG level 2 functional subcategories, and 369 KEGG level 3 pathways. At level 1, the distribution summed to 100% as expected: Metabolism (32.2%), Human Diseases (21.6%, comprising primarily microbial pathogenesis and stress–response pathways rather than host disease processes, consistent with insect gut microbiome profiles), Organismal Systems (21.4%), Environmental Information Processing (10.3%), Cellular Processes (8.9%), and Genetic Information Processing (5.4%). It is noteworthy that the high proportion of “Human Diseases” primarily reflects microbial pathogenesis and stress-response pathways (e.g. bacterial secretion systems, 2-component systems) rather than host disease processes, a common feature in insect gut microbiome profiles (Fig. 3A).

**Fig. 3.**
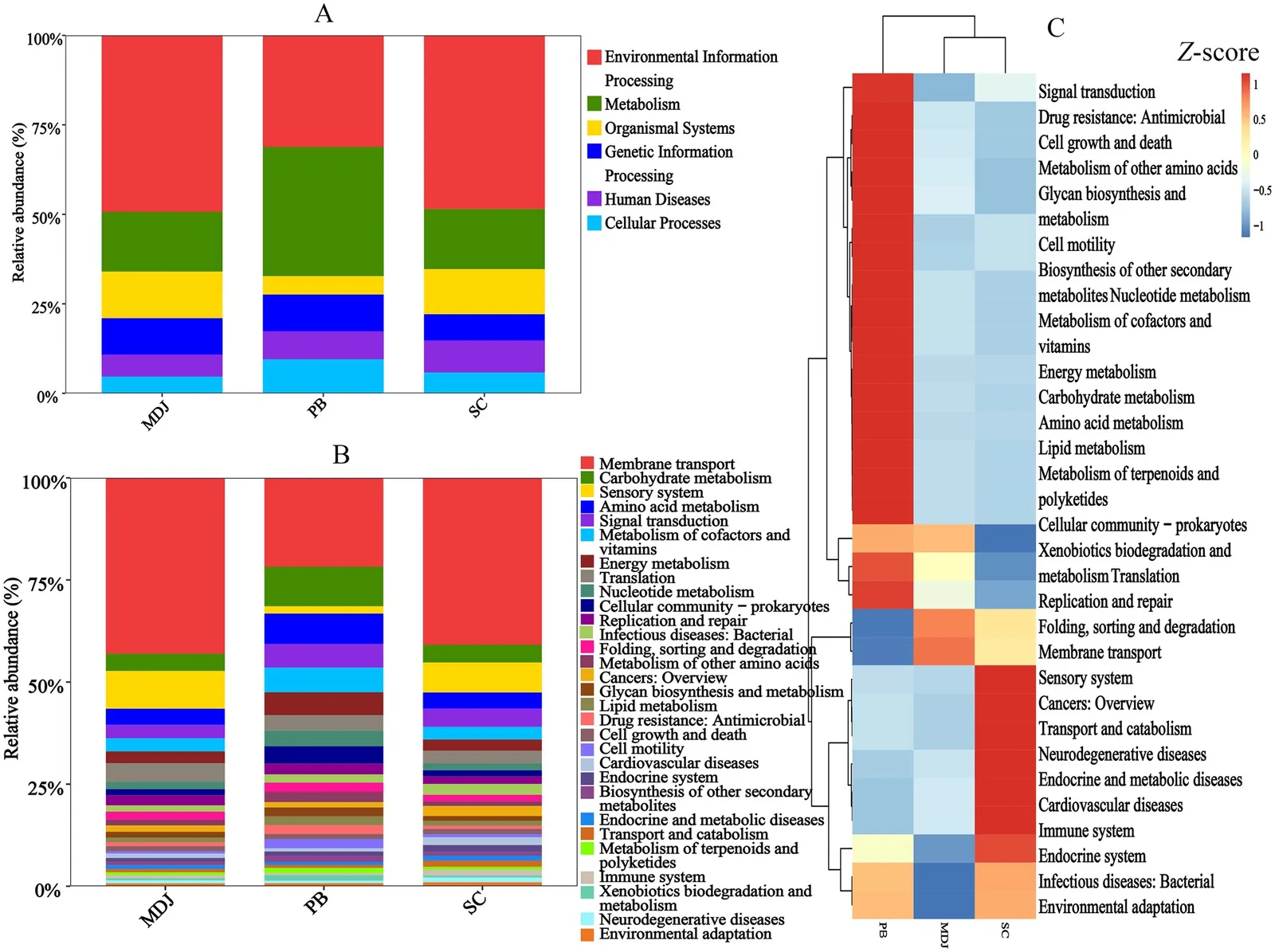
KEGG metabolic pathway analysis of gut microbial functional genes in *L. oryzophilus*. (A) Relative abundance of KEGG level 1 metabolic pathways. Percentages of relative abundance represent when restricted to KEGG-annotated genes, the 6 level 1 categories collectively account for 100% of assignments. (B) Relative abundance of KEGG level 2 pathways. Percentages of relative abundance represent when restricted to KEGG-annotated genes, the 45 level 2 categories collectively account for 100% of assignments. Sequences with no significant match to the KEGG database (accounting for ∼70% of the total gene pool) are not shown. (C) Heatmap of the top 30 most variable KEGG level 2 pathway abundances in the gut microbiota across geographic populations (SC, MDJ, and PB). Values are per-row *Z*-scores derived from TPM (Transcripts Per Million)-normalized KO gene counts. Briefly, raw read counts mapped to KEGG Orthology (KO) entries were first converted to TPM to correct for differences in sequencing depth and KO gene length, then each pathway (row) was *Z*-score transformed across the 3 populations to highlight relative enrichment/depletion. Color scale: red = enrichment relative to the pathway mean across populations; blue = depletion. Population keys: PB, Pingba, Guizhou; SC, Yibin, Sichuan; MDJ, Mudanjiang, Heilongjiang.

When normalized within each population’s respective set of annotated genes, distinct geographic patterns were evident in the KEGG level 1 profiles. The PB population exhibited the highest relative abundance in Metabolism (36.09%), followed by Environmental Information Processing (31.17%), Genetic Information Processing (10.25%), and Cellular Processes (9.49%). In contrast, the SC and MDJ populations showed higher relative abundances in Environmental Information Processing (48.51% and 49.31%, respectively), followed by Metabolism (16.81% and 16.70%, respectively) and Organismal Systems (12.58% and 12.99%, respectively) (Fig. 3B; Supplementary Table S6).

At level 2, Membrane Transport was the most enriched subcategory across all 3 geographic populations for 21.74% (PB), 40.86% (SC), and 43.07% (MDJ), respectively, of annotated genes. In PB, Carbohydrate Metabolism ranked second (9.69%), followed by Amino Acid Metabolism (7.43%), Metabolism of Cofactors and Vitamins (6.03%), Signal Transduction (5.82%), and Energy Metabolism (5.60%). In contrast, in the SC and MDJ populations, the Sensory System ranked second (7.34% and 9.24%, respectively). Subsequent rankings differed: in SC, Signal Transduction ranked third (4.46%), whereas in MDJ, Translation ranked third (4.54%) (Fig. 3C; Supplementary Table S7).

### eggNOG Analysis of Gut Microbiota

To functionally characterize the gut microbial gene repertoire, eggNOG-mapper was employed to assign orthology to the 6,533 genes (derived from the NR-based Nr catalog) against the eggNOG (v5.0) database. A total of 22 functional categories were represented within the annotated dataset. The most abundant categories reflected core microbial metabolic and cellular processes: “Amino acid transport and metabolism” (431 genes), “Transcription” (422 genes), “Replication, recombination, and repair” (413 genes), “Translation, ribosomal structure and biogenesis” (397 genes), and “Carbohydrate transport and metabolism” (373 genes). Other significant categories were “Cell wall/membrane/envelope biogenesis” (332 genes) and “Inorganic ion transport and metabolism” (316 genes). Notably, 2,311 genes (35.4% of annotated sequences) were categorized as “(S) function unknown” (Fig. 4A).

**Fig. 4.**
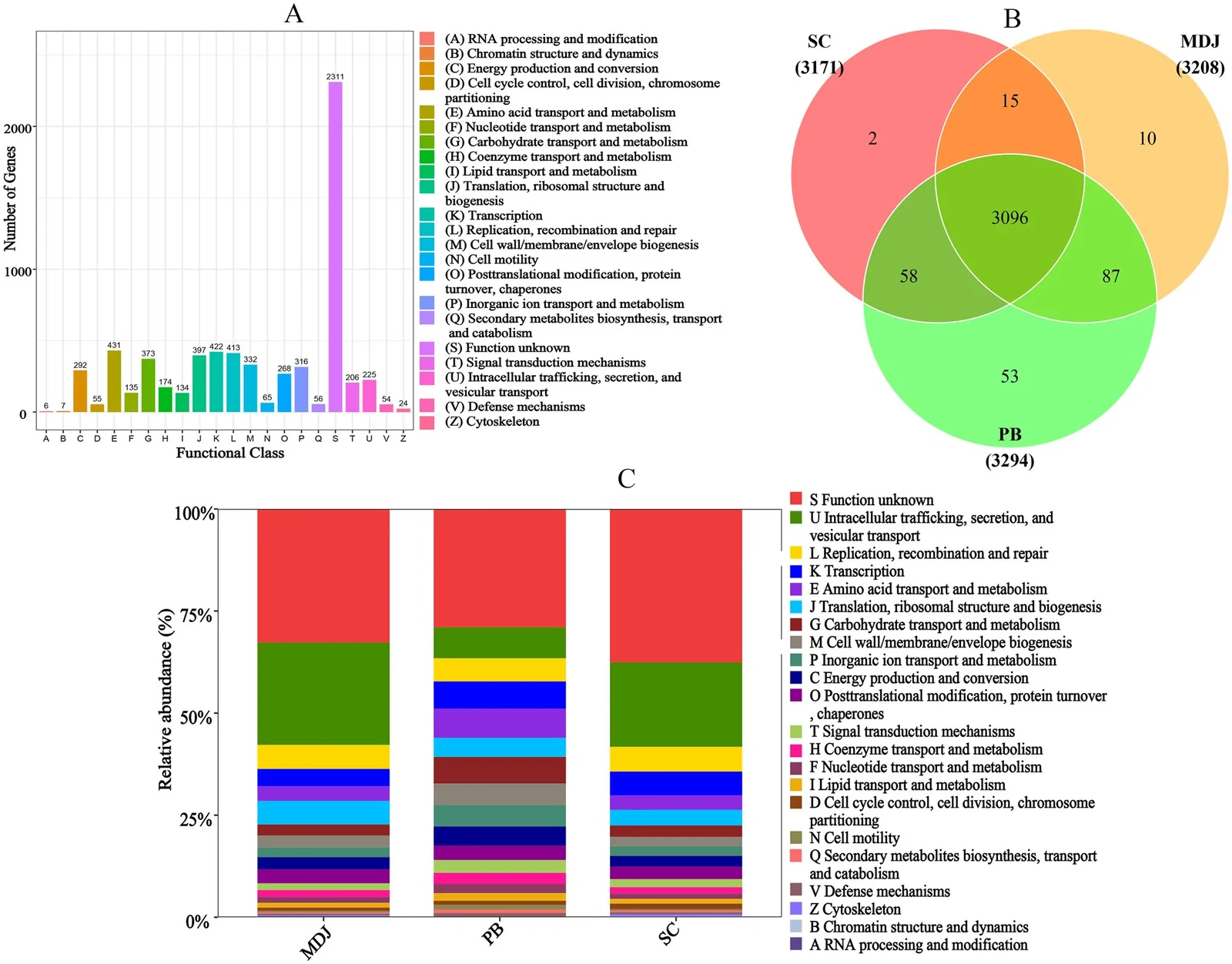
eggNOG functional annotation of gut microbiota in *L. oryzophilus*. (A) Overview of gene distribution across eggNOG functional categories. The analysis was based on 6,533 unigenes that were successfully annotated against the eggNOG database. The *x*-axis displays the 22 standard COG categories, and the *y*-axis shows the count of annotated unigenes per category. (B) Venn diagram of orthologous groups (OGs) identified in the gut microbiota across populations (SC: 3,171 OGs; MDJ: 3,208 OGs; and PB: 3,294 OGs). The diagram illustrates shared and unique OGs among the 3 populations. (C) Relative abundance of eggNOG-annotated functional categories across geographic populations (MDJ, PB, and SC). Abundances were normalized to 1 (100%) per sample to facilitate cross-population comparison of functional profiles. Population keys: PB, Pingba, Guizhou; SC, Yibin, Sichuan; MDJ, Mudanjiang, Heilongjiang.

Across the 3 geographic populations, the number of genes annotated with clusters of orthologous groups (OGs) was 3,294 in PB, 3,171 in SC, and 3,208 in MDJ, respectively (Fig. 4B). Despite these quantitative differences, all populations exhibited a distinct functional profile when normalized to their respective annotated gene pools. “Intracellular trafficking, secretion, and vesicular transport” constituted the most enriched category in SC (20.69%) and MDJ (25.06%), whereas it ranked lower in PB (7.58%). The functional landscape was dominated by metabolic and informational processing: “Amino acid transport and metabolism” (7.17%), “Transcription” (6.64%), “Carbohydrate transport and metabolism” (6.51%), and “Replication, recombination, and repair” (5.69%). Conversely, SC and MDJ prioritized cellular maintenance and replication. In SC, “Replication, recombination, and repair” (6.06%) ranked second, followed by “Transcription” (5.82%), “Translation, ribosomal structure and biogenesis” (3.80%), and “Amino acid transport and metabolism” (3.57%). MDJ exhibited a similar trend, with “Translation, ribosomal structure, and biogenesis” (5.77%) as the second most abundant category, followed by “Transcription” (4.25%) and “Amino acid transport and metabolism” (3.57%). Notably, the proportions of genes assigned to the eggNOG category (S) “Function unknown” were 28.98% in PB, 37.57% in SC, and 32.73% in MDJ, (normalized to the total number of eggNOG-annotated genes per population [Fig. 4C; Supplementary Table S8]).

### CAZy Analysis of Gut Microbiota

Annotation against the CAZy database revealed that CAZymes constitute a minor fraction of the total predicted gene repertoire. Of the 11,651 Nr genes, only 106 (0.91%) were identified as CAZymes. These sequences were distributed across 5 distinct classes: GHs (48 genes, 45.28%), Glycosyltransferases (GTs, 44 genes, 41.51%), Carbohydrate Esterases (CEs, 5.66%), Auxiliary Activities (AAs, 4.72%), and Carbohydrate-Binding Modules (CBMs, 2.83%) (Fig. 5A).

**Fig. 5.**
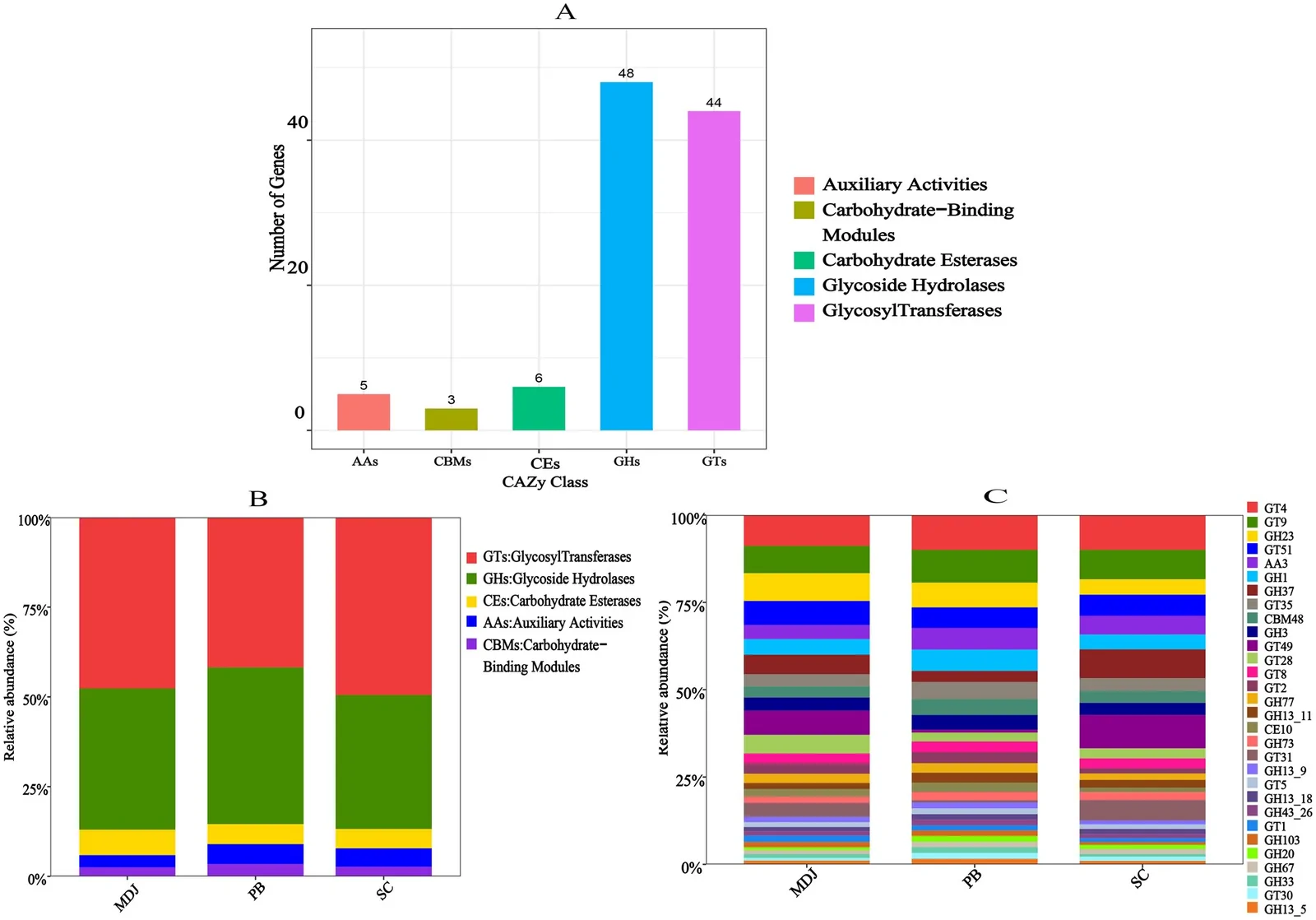
Distribution of carbohydrate-active enzymes (CAZymes) annotation in the gut microbiota of *L. oryzophilus*. (A) Bar chart illustrating the absolute number of genes annotated to 5 major CAZy classes: Auxiliary Activities (AAs), Carbohydrate-Binding Modules (CBMs), Carbohydrate Esterases (CEs), Glycoside Hydrolases (GHs), and Glycosyltransferases (GTs). The data indicate a predominant abundance of GHs (*n* = 48) and GTs (*n* = 44), which together constitute the majority of the identified CAZymes. (B) Relative abundance of CAZy classes across geographic populations (SC, MDJ, and PB). Stacked bar charts depict the proportional distribution of the 5 major CAZy classes. Values represent the proportion of genes annotated to each class relative to the total number of CAZy-annotated genes per sample. The *Y*-axis is scaled to 100% to illustrate the compositional structure within each sample. (C) Proportional composition of the top 30 CAZy subfamilies (including GHs and GTs) across populations. Stacked bars represent the relative abundance of specific CAZy families (e.g. GH13_5, GH3, GT4, etc.) within each geographic population (MDJ, PB, and SC). The *Y*-axis is scaled to 100% to facilitate comparison of the CAZyme repertoire composition among populations. Population keys: PB, Pingba, Guizhou; SC, Yibin, Sichuan; MDJ, Mudanjiang, Heilongjiang.

Across the 3 geographic populations, GTs and GHs were the 2 dominant classes across all samples, jointly constituting the core of microbial carbohydrate degradation and synthesis capabilities. While the PB population exhibited a higher relative abundance of GHs (43.63% of CAZymes) compared to GTs (41.81%), SC and MDJ displayed the inverse pattern, with GTs predominating (49.48% and 47.66%, respectively), followed by GHs (37.34% in SC, 39.35% in MDJ). CEs ranked third in all populations (5.57% in PB, 5.38% in SC, 7.09% in MDJ) (Fig. 5B; Supplementary Table S9). At the family level, GT4 was the most abundant CAZy family across all populations (9.98% in PB, 9.97% in SC, 8.83% in MDJ). GT4 encompasses key enzymes such as sucrose synthase (EC 2.4.1.13) and various GTs (EC 2.4.1.52, EC 2.4.1.56, EC 2.4.1.57), which are critical for sucrose metabolism and cell wall biogenesis. However, the rank order of secondary families varied markedly among populations. In PB, GT9 (9.38%) and GH23 (7.07%) were the second and third most abundant families. GT9 includes lipopolysaccharide (LPS) N-acetylglucosaminyltransferase (EC 2.4.1.56) and heptosyltransferase (EC 2.4.99.-), implicated in LPS and peptidoglycan biosynthesis. GH23, a muramidase associated with cell wall hydrolysis (e.g. lysozyme type G, EC 3.2.1.17; peptidoglycan lytic transglycosylase, EC 4.2.2.-), was also prevalent. The high abundance of GT4, GT9, and GH23 in PB corroborates the class-level data, suggesting an environment conducive to extensive cell wall remodeling and biofilm formation. In contrast, SC was characterized by high abundances of GT49 (9.55%) and GH37 (8.28%, an alpha-glucosidase), while MDJ exhibited enrichment in GH23 (7.96%) and GT51 (6.91%). Despite lower overall CAZyme loads, SC and MDJ maintained consistent proportional reliance on these core families, underscoring their fundamental role in basal cellular physiology (Supplementary Table S10).

These genes lacking significant similarity to any known CAZy family (the “Others” category) comprised over 98% of the total annotated genes, accounting for 99.65% (SC), 98.66% (MDJ), and 99.64% (PB). This high proportion of “Others” is consistent with the fact that the majority of microbial genes are involved in core cellular processes (e.g. transcription, translation, and metabolism) rather than polysaccharide degradation or modification. Minor classes, including AAs and CBMs, were present at very low abundances, reflecting a limited capacity for oxidative cleavage and substrate targeting in these environments.

### CARD Analysis of Gut Microbiota

Of the 11,651 Nr genes, only 325 (2.79%) returned significant alignments to entries in the CARD database, representing a minor fraction of the gut microbial functional repertoire. Within this CARD-annotated gene pool, the most prevalent determinant was *macB* (ARO: 3000535), an ATP-binding cassette (ABC) transporter specific for exporting 14- and 15-membered macrolides, accounting for 18.11% (PB), 15.90% (SC), and 13.11% (MDJ) of CARD-annotated genes. This was followed by *msbA* (ARO: 3003950), a conserved ABC superfamily nucleotide-binding domain transporter homologous to *Escherichia coli* multidrug resistance efflux pumps, with consistent representation across populations: 9.14% (PB), 9.77% (SC), and 9.72% (MDJ). The third most abundant determinant was *tetA(48)* (ARO: 3003980), a tetracycline-specific efflux pump originally isolated from *Paenibacillus* sp. LC231 in the oligotrophic, undisturbed Lechuguilla Cave ecosystem, with significantly higher enrichment in PB (7.27%) compared to SC (5.70%) and MDJ (4.41%) (Fig. 6A and B; Supplementary Table S11).

**Fig. 6.**
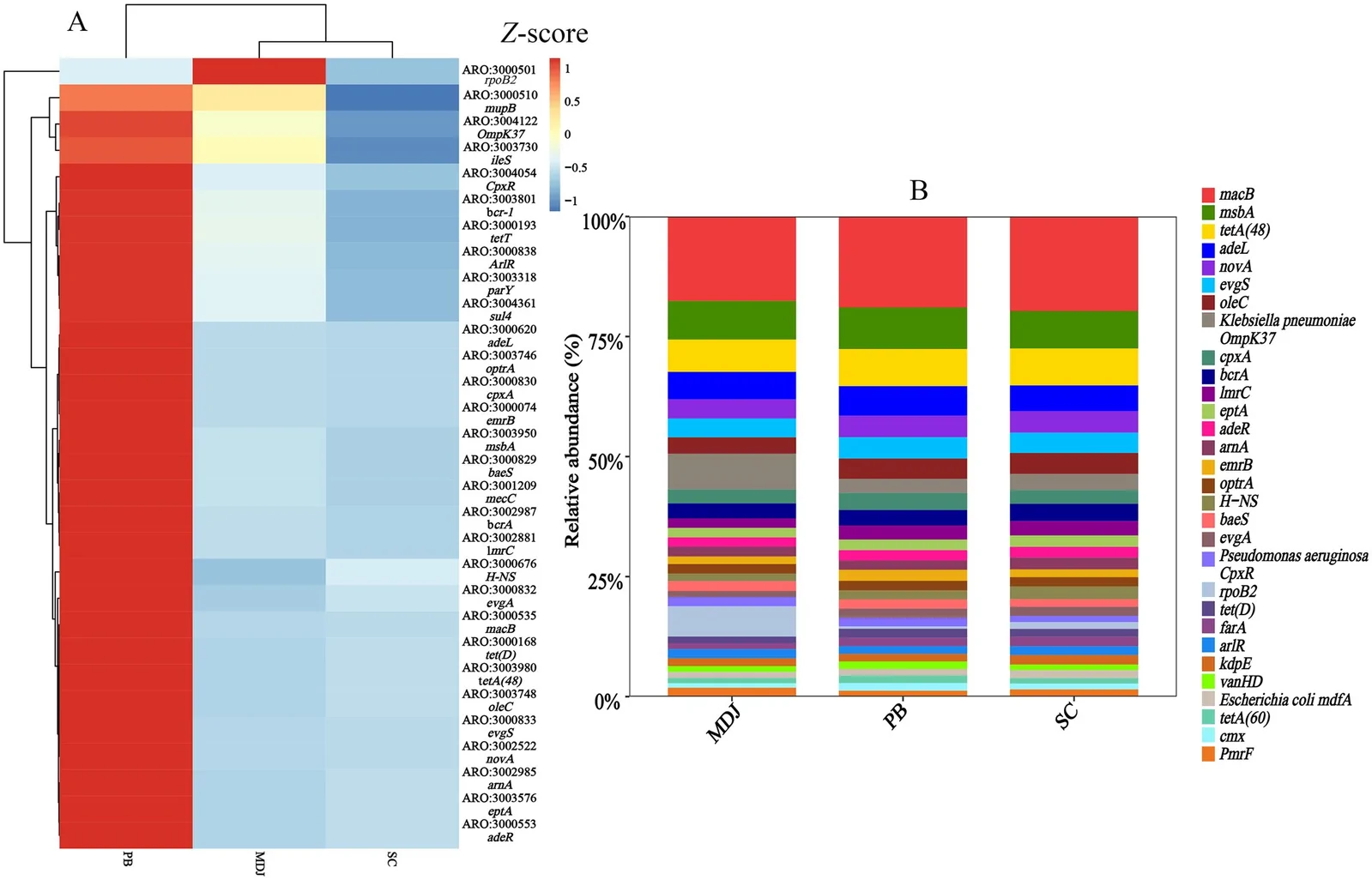
Profiling of antibiotic resistance genes (ARGs) in *L. oryzophilus* gut microbiota based on the Comprehensive Antibiotic Resistance Database (CARD). (A) Heatmap displaying the relative abundance of the top 30 most variable ARGs across populations (SC, MDJ, and PB). Data were normalized to TPM and transformed to *Z*-scores to highlight population-specific enrichment or depletion patterns. Red color indicates higher-than-average abundance, while blue indicates lower-than-average abundance. (B) Stacked bar charts displaying the relative abundance composition of ARG types across the same populations. Genes were classified using the Antibiotic Resistance Ontology (ARO) system, with each color representing a distinct resistance mechanism. The *Y*-axis is scaled to 100% to enable direct comparison of the resistance gene repertoire among populations. Population keys: PB, Pingba, Guizhou; SC, Yibin, Sichuan; MDJ, Mudanjiang, Heilongjiang.

### Multivariate Analysis of Gut Microbial Composition and Functional Potential

To mitigate the confounding influence of *Rickettsiaceae* and *Wolbachia* on the native gut community structure, all reads annotated as these endosymbionts were excluded from the abundance matrices prior to performing PCoA.

At the order level, the first 2 PCoA axes explained 93.08% (PCoA1) and 4.67% (PCoA2) of total variance. PERMANOVA confirmed highly significant inter-population differences (*F *= 39.14, *R²* = 0.929, *P* = 0.009): MDJ and SC overlapped on the negative PCoA1 axis, while PB was distinctly separated on the positive axis, indicating a markedly divergent taxonomic profile (Fig. 7A). This pattern persisted across lower taxonomic ranks (Family, Genus, Species). PCoA1 retained dominant explanatory power (87.28% to 90.94% variance), and PERMANOVA supported significant group differences (Family: *P *= 0.015; Genus: *P *= 0.015; Species: *P *= 0.009; all *R²* > 0.89). PB maintained its unique positioning, confirming geographic location as the primary driver of taxonomic variation (Fig. 7B to Dss).

**Fig. 7.**
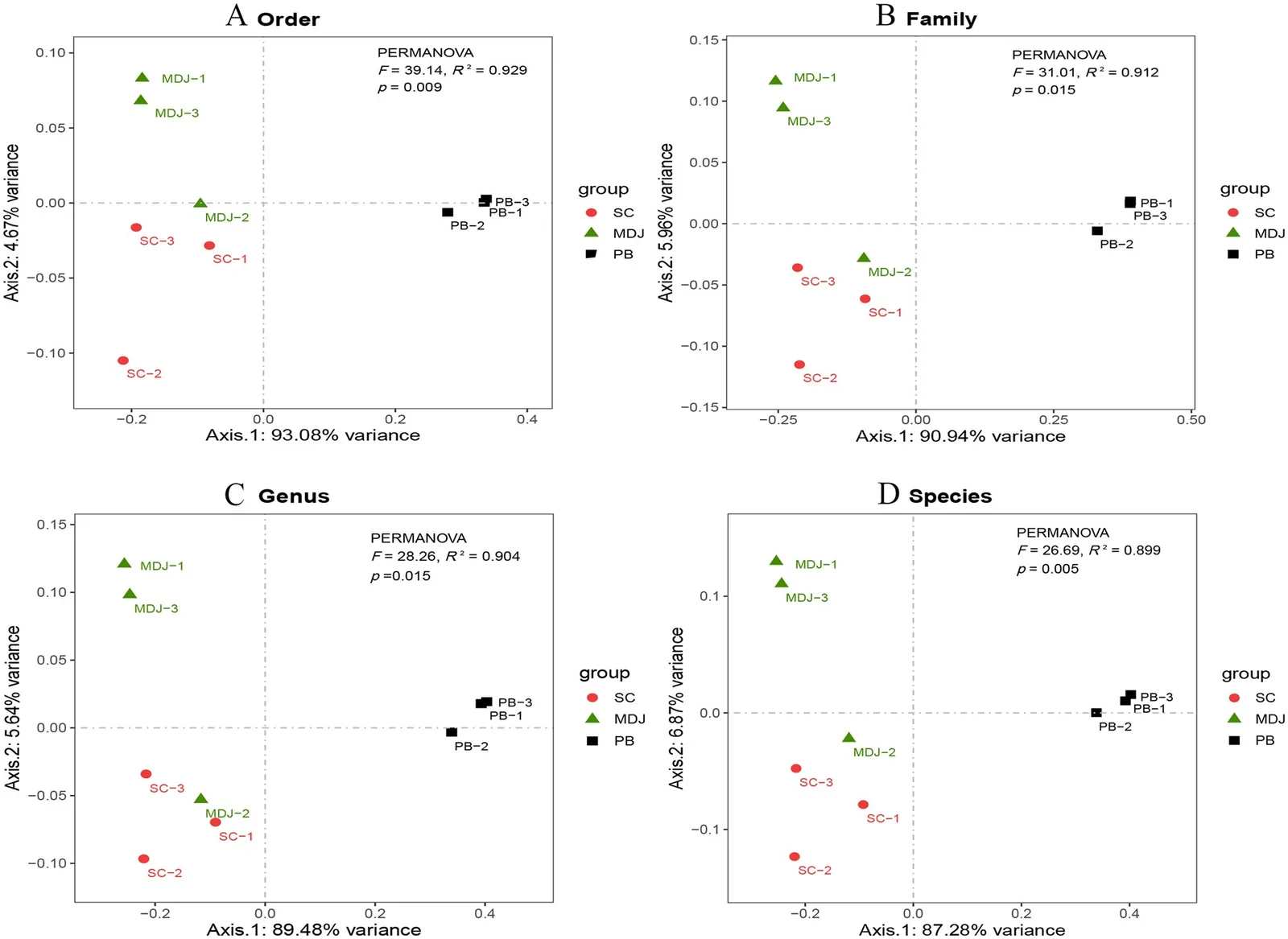
Principal Coordinates Analysis (PCoA) of gut microbiota taxonomic composition across geographic populations of *L. oryzophilus*. PCoA was performed based on Bray–Curtis dissimilarity at 4 taxonomic resolutions: (A) Order, (B) Family, (C) Genus, and (D) Species. The percentage of variation explained by each principal coordinate (axis 1, axis 2) is indicated in parentheses on the axis labels. Data points represent individual biological replicates, with symbols denoting population origin: ● SC (Yibin, Sichuan, Red), ▲ MDJ (Mudanjiang, Heilongjiang, Green), ■ PB (Pingba, Guizhou, Black). At the order level (A), PB samples clustered separately along the positive PCo1 axis, while SC and MDJ samples overlapped extensively. This distinct separation of the PB population persisted across lower taxonomic ranks (Family to Species; B to D), although the overlap between SC and MDJ diminished progressively at finer taxonomic resolutions. PERMANOVA confirmed significant inter-population differences across all levels (Order: *R²* = 0.929, *P *= 0.009; Family: *R²* = 0.912, *P *= 0.015; Genus: *R²* = 0.904, *P *= 0.015; Species: *R²* = 0.899, *P *= 0.009), indicating that geographic origin is the primary driver of gut microbial community structure. Reads mapped to intracellular endosymbionts (Rickettsiales) were excluded to better represent the gut-luminal microbial community.

To elucidate functional differences, PCoA was applied to 4 functional gene databases (KEGG, eggNOG, CAZy, and CARD). At the KEGG pathway (level 3), PCoA1 (88.65% variance) cleanly separated PB (positive axis) from SC/MDJ (negative axis), while PCoA2 (7.27% variance) distinguished MDJ from SC (Fig. 8A). PERMANOVA confirmed strong group effects (*R²* = 0.881, *P *= 0.009). For eggNOG OGs, a nearly identical pattern emerged (Fig. 8B). PCoA1 (80.57% variance) segregated PB, and PCoA2 (9.68% variance) separated MDJ/SC (*R²* = 0.863, *P *= 0.004). PCoA of CAZy families (Fig. 8C) mirrored taxonomic and broad functional trends. PCoA1 (86.69% variance) separated PB from SC/MDJ, while SC/MDJ (both on the negative PCoA1 axis) exhibited dispersed clustering along PCoA2. PERMANOVA supported significant group differences (*R²* = 0.864, *P *= 0.020), suggesting distinct (yet partially overlapping) carbohydrate degradation potentials between SC and MDJ—even as both diverged from PB. CARD-based PCoA revealed the most striking segregation; PB samples clustered exclusively on the negative PCoA1 axis (no overlap with SC/MDJ), while SC/MDJ occupied the positive axis (with MDJ overlapping from SC along PCoA2). PERMANOVA confirmed significant resistome divergence (*R²*= 0.80, *F *= 11.97, *P *= 0.030), implying PB-specific selective pressures (e.g. local pathogen communities, host immunity) shape its ARG pool (Fig. 8D).

**Fig. 8.**
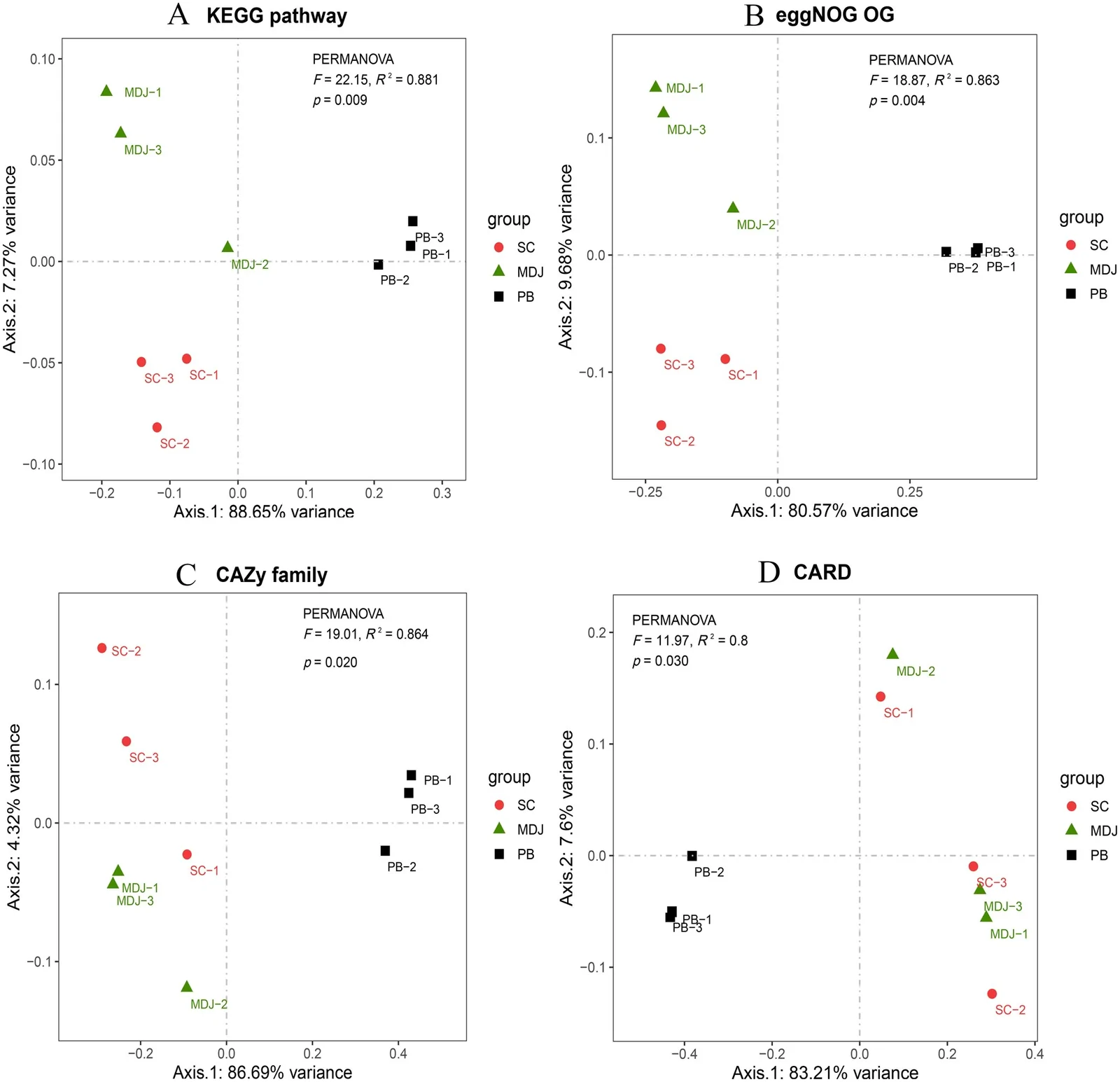
PCoA of gut microbial functional profiles in *L. oryzophilus* populations. Symbols follow Fig. 7. (A) KEGG level 3 pathways, (B) eggNOG orthologous groups (OGs), (C) CAZy families, (D) CARD-annotated antibiotic resistance genes (ARGs). Axis 1 (80.6% to 86.7% variance) consistently separated the high-altitude PB population from lowland SC/MDJ across all datasets; axis 2 (7.6% to 9.7% variance) discriminated SC and MDJ, particularly in metabolic and resistance profiles. PERMANOVA supported significant geographic structuring (*R*² > 0.80, all *P *< 0.05). Data points represent individual biological replicates, with symbols denoting population origin: ● SC (Yibin, Sichuan, Red), ▲ MDJ (Mudanjiang, Heilongjiang, Green), ■ PB (Pingba, Guizhou, Black). Reads mapped to intracellular endosymbionts (Rickettsiales) were excluded to better represent the gut-luminal microbial community.

## Discussion

The gut microbiota of insects constitutes a dynamic interface between the host and its environment, influencing nutrient assimilation, immune homeostasis, and tolerance to environmental stressors (Muhammad et al. 2019, Tobias et al. 2020, Moriyama and Fukatsu 2022). While the gut microbial communities of phytophagous insects often exhibit taxonomic convergence dominated by Proteobacteria (Yun et al. 2014, Wei et al. 2019), mounting evidence suggests that local environmental filtering can drive significant functional divergence among geographic populations. Examples include insect groups such as *Ceracris kiangsu* (Yang et al. 2023), *Holotrichia parallela* (Huang and Zhang 2013), *Nilaparvata lugens* (Wang et al. 2019), and *Dactylispa xanthospila* (Cui et al. 2021), and so on. Gut microbiomes profoundly influence the environmental adaptability of phytophagous insects and their strategies for coping with host resistance through multidimensional functional integration, revealing the complexity and plasticity of the “Environment-Insect-Microbe” tripartite interaction network (Shukla and Beran 2020, Sorokan et al. 2020, Jordan et al. 2021). In this study, we employed a metagenomic approach to characterize the taxonomic and functional landscapes of the gut microbiota across 3 representative Chinese populations of the invasive rice water weevil, *L. oryzophilus*, spanning a steep altitudinal gradient. Our findings reveal distinct patterns of microbial community assembly and functional potential, offering insights into the potential role of microbiota in mediating host adaptation to heterogeneous environments.

### Taxonomic Differentiation and Population-Specific Lineages

Consistent with previous reports on *L. oryzophilus* (Lu et al. 2013, Lu et al. 2014, Jin et al. 2025), Proteobacteria constituted the predominant phylum across all populations. However, significant taxonomic restructuring occurred along the altitudinal gradient. The high-altitude PB (1,211 m a.s.l.) population was characterized by a striking enrichment of the family Erwiniaceae, particularly the genus *Pantoea*, with *P. deleyi* serving as the dominant species. In contrast, the lowland populations of SC (294.7 m) and MDJ (298 m) exhibited a marked prevalence of Rickettsiales, specifically *R. asembonensis* and *Wolbachia* spp.

We identified a subset of population-specific (endemic) taxa—defined here as sequence variants detected exclusively within a single geographic population at the species level. The MDJ population harbored the highest richness of such taxa (19 species), followed by PB (15 species), while SC possessed only a single unique species. These endemic taxa likely represent either local environmental acquisitions or highly specialized symbionts undergoing host population-specific diversification. Notably, the detection of *Crenarchaeota* (Domain Archaea) exclusively in the MDJ population is intriguing. While Archaea are traditionally associated with extreme environments, recent studies confirm their presence in mesophilic habitats, including insect guts (Klammsteiner et al. 2020, Brayley et al. 2026). The functional role of *Crenarchaeota* in *L. oryzophilus* remains speculative but may involve ammonia oxidation or stress tolerance mechanisms distinct from those of bacteria. Furthermore, while our study focused primarily on bacterial communities due to their biomass dominance, we acknowledge the presence of fungal and viral sequences in our metagenomic assemblies. Future studies employing mycobiome- or virome-specific enrichment strategies are warranted to elucidate the potential roles of these non-bacterial constituents in shaping host–microbe interactions.

Across all taxonomic ranks from order to species, the PB population occupied a discrete, non-overlapping cluster, while the SC and MDJ populations exhibited extensive overlap. These findings are consistent with earlier work by Lu et al. (2013, 2014), who documented comparable geographic differentiation in *L. oryzophilus* gut microbiomes between Chinese and US populations, collectively supporting geographic divergence as an important determinant of gut microbiome assembly in this invasive weevil. These results align with established expectations theory, positing that broad-scale environmental filters govern the distribution of dominant microbial taxa, whereas localized abiotic variables primarily shape rare taxonomic fractions. We note, however, that as an observational study, our data do not permit definitive causal inference linking individual environmental parameters to the observed taxonomic shifts.

### Functional Plasticity in Response to Altitudinal Gradients

Functional profiling largely paralleled the observed taxonomic restructuring across populations, hinting at potential metabolic adjustments to altitudinal gradients rather than definitive adaptive reprogramming. Previous studies indicate that functional diversity of gut microbial communities in high-altitude insects correlates with overwintering strategies. In high-altitude environments (low temperature, hypoxia, high radiation), a genus-level replacement of *Snodgrassella* in the gut bacteria of *Bombus przewalskii* (high altitude, >4,200 m a.s.l.) versus *Bombus friseanus* (low altitude, 2,700 to 3,200 m a.s.l.) was observed, accompanied by higher abundance of genes involved in butyrate metabolism. Butyrate, a short-chain fatty acid, regulates host energy metabolism (e.g. promoting fat breakdown) and immune function (e.g. enhancing antioxidant capacity). Coadaptation between the host genome and gut microbiome in high-altitude bumblebees primarily involves genes related to energy metabolism and immune response—key mechanisms for adapting to extreme altitudes (Dong et al. 2024).

In our study, the PB population exhibited consistent enrichment trends in genes associated with carbohydrate and amino acid metabolism, as inferred from KEGG pathway and eggNOG orthology annotations. We note these differences reflect shifts in sequence representation rather than confirmed changes in enzymatic activity, as short-read metagenomic predictions capture genetic potential but do not resolve transcriptional or post-translational regulation. This metabolic signal was further supported by CAZy annotation, which highlighted higher relative abundances of GTs (e.g. GT4 family encoding sucrose synthases) and GHs in PB. The GT4 enrichment may indicate a greater potential for sucrose metabolism and exopolysaccharide biosynthesis, which could plausibly support physiological tolerance to colder high-altitude temperatures. CARD analysis further revealed elevated abundances of ABC transporter-encoding efflux pump genes (e.g. *macB*, *msbA*) in PB. It is critical to emphasize that these represent evolutionarily conserved intrinsic resistance determinants typical of environmental microbiomes, rather than indicators of clinically relevant antibiotic resistance. Their higher prevalence in PB likely reflects a generalized constitutive response to prevalent high-altitude abiotic stressors, including elevated UV-induced oxidative stress and naturally occurring antimicrobial compounds within high-altitude rice agroecosystem ecotones.

PCoA based on Bray–Curtis dissimilarities corroborated these trends. PB occupied a discrete, non-overlapping cluster across all 4 functional databases, aligning with its unique taxonomic composition and co-varying with altitudinal gradients. We stress that, as an observational study, these data preclude direct causal inference between elevation and functional shifts. In contrast, SC and MDJ occupied overlapping or adjacent spaces in CAZy and CARD ordinations, consistent with the stability of core enzymatic (CAZy) and defensive (CARD) modules across lowland populations. This pattern echoes earlier culture- and 16S rRNA-based surveys by Lu et al. (2013, 2014), who reported conserved gut microbial profiles among *L. oryzophilus* populations alongside broader divergence between Chinese and US invasive lineages. Subtle discrimination between SC and MDJ was largely restricted to PCoA 2 in KEGG and eggNOG profiles, where dispersed clustering was observed. These nuanced differences, despite partial taxonomic overlap across ranks, likely reflect fine-scale variation in local abiotic conditions (e.g. microclimate, water chemistry) rather than fundamental rewiring of functional architecture, as evidenced by the conserved CAZy and CARD profiles in these 2 populations. Collectively, these findings delineate a functional landscape wherein core nutrient catabolism and stress defense modules are maintained across lowland invasive ranges, while more flexible metabolic pathways exhibit minor variation in response to local environmental heterogeneity.

### Integrating Taxonomy With Functional Predictions

The congruence between taxonomic restructuring and functional potential provides insight into the potential mechanisms underpinning *L. oryzophilus* adaptation to altitudinal gradients. While functional redundancy often decouples taxonomic composition from metabolic output, our data reveal a tightly correlated “taxonomy-function” axis, particularly within the high-altitude PB population.

Specifically, the dominance of the order Enterobacterales—spearheaded by the genus *Pantoea* in PB—aligned with the significant enrichment of carbohydrate metabolism and amino acid biosynthesis (KEGG, eggNOG). This observation is consistent with established roles for *Pantoea* and related Enterobacteriaceae as metabolic hubs in insect guts, facilitating nutrient assimilation and complementary synthesis of essential metabolites (Anand et al. 2010, Feng et al. 2011, Li et al. 2017, Bhuyan et al. 2018). KEGG Orthology (KO) annotation revealed 18 unique functional genes in the PB population, encoding proteins involved in diverse biological processes. These included enzymes such as xylulose-5-phosphate/fructose-6-phosphate phosphoketolase and L-serine/L-threonine ammonia-lyase (metabolism); components of the Tat translocation and phosphotransferase systems (transport); phage shock proteins and restriction endonucleases (stress response/defense); Tra, Trb, and Icm/Dot protein systems (HGT and secretion); and E3 ubiquitin ligases (protein modification). Notably, enzyme analysis identified 2 unique enzymes in PB: fructose-6-phosphate phosphoketolase (EC 4.1.2.22) and phosphoketolase (EC 4.1.2.9), both involved in the pentose phosphate pathway per KEGG annotation. Comparative pathway analysis indicated that while SC and MDJ shared 369 KEGG pathways, PB lacked 2 specific pathways: KO04623 (cytosolic DNA-sensing pathway, mediating antiviral adaptation, endosymbiont regulation, and immunity to entomopathogens) and KO04978 (mineral absorption, responding to abiotic stress and host plant adaptation). However, as these correlations are derived from metagenomic inference, functional validation remains requisite to confirm these biochemical phenotypes.

These taxonomic and functional patterns resonate with the “environment-dominated, host-modulated” assembly framework observed in the widespread amphibian *Duttaphrynus melanostictus*, wherein large-scale environmental gradients (e.g. altitude/temperature) appear to set the primary template for microbiome restructuring (Zhu et al. 2026). Similarly, in *L. oryzophilus*, this manifests as substantial taxonomic and functional shifts in PB driven by environmental filtering (especially altitude factor), contrasted with the conservation of core enzymatic (CAZy) and defensive (CARD) repertoires in the lowland SC and MDJ populations despite subtle taxonomic drift. This supports a hierarchical assembly principle: while broad-scale environmental variables dictate the primary functional architecture, localized factors, and host traits fine-tune community composition. Collectively, these findings suggest that core metabolic functions, such as carbohydrate catabolism, remain taxonomically anchored within Proteobacteria-associated taxa across heterogeneous environments—a conservative strategy potentially widespread among herbivorous insects for balancing metabolic stability with ecological flexibility.

### Correlation, Causation, and Ecological Implications

The consistent congruence between environmental gradients (e.g. altitude, and temperature) and the compositional and functional architecture of the gut microbiota observed in this study supports the hypothesis that microbial symbionts contribute to the ecological plasticity of *L. oryzophilus*. The pronounced functional divergence between high-altitude (PB) and lowland (SC, MDJ) populations parallels recent findings in other invasive insects, wherein microbiota facilitate adaptation to novel climatic conditions or host resources (Lu et al. 2013, Dong et al. 2024, Zhu and Xu 2025). However, these correlative patterns must be interpreted with caution. While our data demonstrate a robust association between geographic origin and microbiota profiles, they do not establish direct causality. The observed differentiation may arise from the direct filtering of microbial taxa by abiotic environmental parameters, genotype-by-environment interactions of the host, or—most likely—a complex interplay of both factors. Furthermore, the “environment-microbe-host” axis represents a simplified view of a more intricate holobiont system. Our current focus on bacteria overlooks the potential contributions of fungi, viruses, and archaea, as well as the synergistic or antagonistic interactions occurring across these microbial kingdoms. Future studies incorporating these neglected components and employing controlled experiments (e.g. fecal transplants or axenic insect bioassays) are required to disentangle the specific mechanisms driving these ecological patterns.

### Limitations and Future Directions

Several limitations of the current study warrant consideration and highlight avenues for future research:

#### Sample Size and Replication

The modest sample size (*n* = 3 biological replicates per population) may limit the detection of subtle variations in low-abundance taxa or rare functional pathways. While the robust statistical separation observed in PERMANOVA (*P *< 0.05) supports our major conclusions, future studies with increased replication (*n* ≥ 6) are necessary to capture finer-scale intra-population variability.

#### Functional Potential vs. Activity

Our metagenomic approach identifies the potential for specific functions (e.g. CAZy, CARD, KEGG pathways) based on gene presence. It does not confirm whether these genes are transcriptionally active, translated into functional proteins, or biochemically active in situ. Complementary multi-omics approaches, such as metatranscriptomics, metaproteomics, or metabolomics, are required to validate the functional activity of these microbial genes and their physiological impact on the host.

#### Taxonomic Assignment Challenges

As discussed in the Materials and Methods, reliance on the NCBI Nr database presents challenges for species-level classification of insect-specific symbionts. Future work incorporating insect-symbiont-specific databases (e.g. iSymBase, RefSeq, GTDB, or insect-associated symbiont databases) or generating metagenome-assembled genomes would refine taxonomic resolution, particularly for *Wolbachia* and *Rickettsia* clades.

#### Core vs. Transient Microbiome

Distinguishing between resident core symbionts and transient environmental microbes (e.g. ingested with food) remains challenging in field-collected specimens. Controlled laboratory rearing experiments would help disentangle these components.

## Conclusion

In conclusion, this study provides a comprehensive metagenomic atlas of the gut microbiota of *L. oryzophilus* across a steep altitudinal gradient in China. We demonstrate that geographic isolation is associated with significant restructuring of both the taxonomic composition and functional potential of the gut microbiome. The high-altitude PB population exhibits a unique microbial signature, characterized by *Pantoea* dominance and enrichment in metabolic pathways related to carbohydrate utilization and stress resistance, potentially representing a microbial-mediated adaptive strategy. Conversely, lowland populations (SC, MDJ) share a more convergent functional profile dominated by intracellular symbionts. While these findings highlight the potential of gut microbiota to act as a flexible component of the host’s adaptive toolkit, they underscore the need for cautious interpretation regarding causality. Our work lays the groundwork for future hypothesis-driven experiments to disentangle the complex interplay between environment, host genetics, and microbial function in driving the invasive success of this global agricultural pest.

## Supplementary Material

ieag111_Supplementary_Data

## Data Availability

All data generated or analyzed during the current study are included in this article and are available from the corresponding author or the first author on reasonable request.

## References

[ieag111-B1] Anand AAP , Vennison SJ, Sankar SG, et al 2010. Isolation and characterization of bacteria from the gut of *Bombyx mori* that degrade cellulose, xylan, pectin and starch and their impact on digestion. J. Insect Sci. 10:107–120. 10.1673/031.010.10701 PMC3016902 20874394

[ieag111-B2] Bhuyan PM , Sandilya SP, Nath PK, et al 2018. Optimization and characterization of extracellular cellulase produced by Bacilluspumilus MGB05 isolated from midgut of muga silkworm (*Antheraea assamensis* Helfer). J. Asia-Pacific Entomol. 21:1171–1181. 10.1016/j.aspen.2018.08.004

[ieag111-B3] Brayley ODM , McCready K, Liu S, et al 2026. The microbiome of an invasive antarctic insect, *Eretmoptera murphyi* (Diptera: Chironomidae), and its potential role in nutrient cycling. Microbial. Ecol. 89:66. 10.1007/s00248-026-02706-5 PMC12966229 41764142

[ieag111-B4] Brune A. 2014. Symbiotic digestion of lignocellulose in termite guts. Nat. Rev. Microbiol. 12:168–180. 10.1038/nrmicro3182 24487819

[ieag111-B6] Chen B , Kang L. 2002. Cold hardiness and supercooling capacity in the pea leafminer *Liriomyza huidobrensis*. Cryo Letters. 23:173–182. 12148020

[ieag111-B7] Chen DF , Huang ZH, Li YB, et al 2023. The comparison of surface sterilization methods for the analysis of insect gut microbiota: *Solenopsis invicta* (Formicidae) as an example. Sociobiol 70:e8263. 10.13102/sociobiology.v70i3.8263

[ieag111-B8] Chen HW , Xie D, Jiang LH, et al 2025. Gut bacteria strategies of *Hylurgus ligniperda* F. (Coleoptera Scolytidae) in adapting to temperature changes. Microorganisms 13:2502. 10.3390/microorganisms13112502 PMC12654366 41304188

[ieag111-B9] Coolen S , Rogowska-van der Molen MA, Kwakernaak I, et al 2024. Microbiota of pest insect *Nezara viridula* mediate detoxification and plant defense repression. ISME J. 18:wrae097. 10.1093/ismejo/wrae097 PMC11195473 38836495

[ieag111-B10] Cui LX , Guo QY, Wang XX, et al 2021. Midgut bacterial diversity of a leaf-mining beetle, *Dactylispa xanthospila* (Gestro) (Coleoptera: Chrysomelidae: Cassidinae). Biodivers. Data J. 9:e62843. 10.3897/BDJ.9.E62843 PMC8128845 34012315

[ieag111-B11] Dong JH , Xu X, Ren ZX, et al 2024. The adaptation of bumblebees to extremely high elevation associated with their gut microbiota. mSystems 9:e0121923. 10.1128/msystems.01219-23 PMC10949452 38329353

[ieag111-B12] Douglas AE. 2015. Multiorganismal insects: diversity and function of resident microorganisms (Review). Annu. Rev. Entomol. 60:17–34. 10.1146/annurev-ento-010814-020822 PMC4465791 25341109

[ieag111-B13] Fick SE , Hijmans RJ. 2017. WorldClim 2: new 1‐km spatial resolution climate surfaces for global land areas. Int. J. Climatol. 37:4302–4315. 10.1002/joc.5086

[ieag111-B14] Feng W , Wang XQ, Zhou W, et al 2011. Isolation and characterization of lipase-producing bacteria in the intestine of the silkworm, *Bombyx mori*, reared on different forage. J. Insect Sci. 11:135. 10.1673/031.011.13501 PMC3391909 22243438

[ieag111-B15] Ge Y , Jing ZW, Diao QY, et al 2021. Host species and geography differentiate honeybee gut bacterial communities by changing the relative contribution of community assembly processes. mBio 12:e00751-21. 10.1128/mbio.00751-21 PMC8262996 34061602

[ieag111-B16] Handy MY , Sbardellati DL, Yu M, et al 2023. Incipiently social carpenter bees (Xylocopa) host distinctive gut bacterial communities and display geographical structure as revealed by full-length PacBio 16S rRNA sequencing. Mol. Ecol. 32:1530–1543. 10.1111/mec.16736 36239475

[ieag111-B17] Heinrichs EA , Quisenberry SS. 1999. Germplasm evaluationand utilization for insect resistance in rice. In: Clement SL, Quisenberry SS, editors. Global plant genetic resources for insect-resistance crops. CRC Press. p. 3–23.

[ieag111-B18] Hodkinson DI , Bird J, Miles EJ, et al 1999. Climatic signals in the life histories of insects: the distribution and abundance of heather psyllids (*Strophingia* spp.) in the UK. Funct. Ecol. 13:83–95. 10.1046/j.1365-2435.1999.00011.x

[ieag111-B19] Huang SW , Zhang HY. 2013. The impact of environmental heterogeneity and life stage on the hindgut microbiota of *Holotrichia parallela* larvae (Coleoptera: Scarabaeidae). PLoS One. 8:e57169. 10.1371/journal.pone.0057169 PMC3578786 23437336

[ieag111-B20] Huerta-García A , Álvarez-Cervantes J. 2024. The gut microbiota of insects: a potential source of bacteria and metabolites. Int. J. Trop. Insect Sci. 44:13–30. 10.1007/s42690-023-01147-8

[ieag111-B21] Jenkins NL , Hoffmann AA. 1999. Limits to the southern border of *Drosophila serrata*: cold resistance, heritable variation, and trade-offs. Evolution 53:1823–1834. 10.1111/j.1558-5646.1999.tb04565.x 28565454

[ieag111-B22] Jordan C , de Carvalho VR, Mascarin GM, et al 2021. First record of a new microsporidium pathogenic to *Gonipterus platensis* in Brazil. Sci. Rep. 11:10971. 10.1038/s41598-021-90041-9 PMC8155060 34040020

[ieag111-B23] Jin JX , Wang Y, Zhang GF, et al 2025. Diversity and functional analysis of gut microbiota in the adult of *Lissorhoptrus oryzophilus* (Coleoptera: Curculionidae) by metagenome sequencing. Insects 16:1260. 10.3390/insects16121260 PMC12733467 41465696

[ieag111-B24] Klammsteiner T , Walter A, Bogataj T, et al 2020. The core gut microbiome of black soldier fly (*Hermetia illucens*) larvae raised on low-bioburden diets. Front. Microbiol. 11:933. 10.3389/fmicb.2020.00993 PMC7253588 32508795

[ieag111-B25] Kuschel G. 1951. Revisión de *Lissorhoptrus* LeConte y géneros vecinos de América. *Rev. Chil. Ent.* 1:23–74.

[ieag111-B5] Lee C E. 2002. Evolutionary genetics of invasive species. Trends Eco. Evol. 17:386–391. 10.1016/S0169-5347(02)02554-5

[ieag111-B26] Li JL , Fu NN, Wang M, et al 2024. Functional and compositional changes in *Sirex noctilio* gut microbiome in different habitats: unraveling the complexity of invasive adaptation. IJMS. 25:2526. 10.3390/ijms25052526 PMC10931295 38473774

[ieag111-B27] Li WH , Jin DC, Li FL, et al 2017. Metabolic phenomics of bacterium *Pantoea* sp. from larval gut of the diamondback moth, *Plutella xylostella* (Lepidoptera: Plutellidae). Symbiosis 72:135–142. 10.1007/s13199-016-0453-4

[ieag111-B28] Lu F , Kang XY, Jiang C, et al 2013. Isolation and characterization of bacteria from midgut of the rice water weevil (Coleoptera: Curculionidae). Environ. Entomol. 42:874–881. 10.1603/EN13111 PMID: 24331600. 24331600

[ieag111-B29] Lu F , Kang XY, Lorenz G, et al 2014. Culture-independent analysis of bacterial communities in the gut of rice water weevil (Coleoptera: Curculionidae). Ann. Entomol. Soc. Am. 107:592–600. 10.1603/AN13145

[ieag111-B31] Ministry of Agriculture and Rural Affairs of the People’s Republic of China. 2025. List of administrative regions for the distribution of national agricultural quarantine pests. https://zzys.moa.gov.cn/gdxw/202507/t20250730_6476125.htm

[ieag111-B32] Moriyama M , Fukatsu T. 2022. Host’s demand for essential amino acids is compensated by an extracellular bacterial symbiont in ahemipteran insect model. Front. Physiol. 13:1028409. 10.3389/fphys.2022.1028409 PMC9561257 36246139

[ieag111-B33] Muhammad A , Habineza P, Ji TL, et al 2019. Intestinal microbiota confer protection by priming the immune system of red palm weevil *Rhynchophorus ferrugineus* Olivier (Coleoptera: Dryophthoridae). Front. Physiol. 10:1303. 10.3389/fphys.2019.01303 PMC6805723 31681013

[ieag111-B34] Reay-Jones FPF , Way MO, Tarpley L. 2008. Nitrogen fertilization at the rice panicle differentiation stage to compensate for rice water weevil (Coleoptera: Curculionidae) injury. Crop Prot 27:84–89. 10.1016/j.cropro.2007.04.009

[ieag111-B35] Schoville SD , Chen YH, Andersson MN, et al 2018. A model species for agricultural pest genomics: the genome of the Colorado potato beetle, *Leptinotarsa decemlineata* (Coleoptera: Chrysomelidae). Sci. Rep. 8:1931. 10.1038/s41598-018-20154-1 PMC5792627 29386578

[ieag111-B36] Shukla SP , Beran F. 2020. Gut microbiota degrades toxic isothiocyanates in a flea beetle pest. Mol. Ecol. 29:4692–4705. 10.1111/mec.15657 33006166

[ieag111-B37] Siddiqui JA , Fan RD, Liu YJ, et al 2025. The larval gut of *Spodoptera frugiperda* harbours culturable bacteria with metabolic versatility after insecticide exposure. Insect Mol. Biol. 34:452–469. 10.1111/IMB.12983 39952648

[ieag111-B38] Sorokan AV , Burkhanova GF, Benkovskaya GV, et al 2020. Colorado potato beetle microsymbiont Enterobacter BC-8 inhibits defense mechanisms of potato plants using crosstalk between jasmonate- and salicylate-mediated signaling pathways. Arthropod-Plant Inte 14:161–168. 10.1007/s11829-019-09732-w

[ieag111-B39] Tobias NJ , Eberhard FE, Guarneri AA. 2020. Enzymatic biosynthesis of B-complex vitamins is supplied by diverse microbiota in the *Rhodnius prolixus* anterior midgut following *Trypanosoma cruzi* infection. Comput. Struct. Biotechnol. J. 18:3395–3401. 10.1016/j.csbj.2020.10.031 PMC7691439 33294135

[ieag111-B40] Wang TZ , Wang ZL, Zhu HF, et al 2019. Analysis of the gut microbial diversity of the brown planthopper, *Nilaparvata lugens* (Hemiptera: Delphacidae) by high-throughput sequencing. Acta Entomol. Sin. 62:323–333. https://link.oversea.cnki.net/doi/10.16380/j.kcxb.2019.03.006. (In Chinese)

[ieag111-B41] Wang YD , Li ZH, Zhao ZH. 2023. Population mixing mediates the intestinal flora composition and facilitates invasiveness in a globally invasive fruit fly. Microbiome 11:213. 10.1186/s40168-023-01664-1 PMC10538247 37759251

[ieag111-B42] Wei XY , Guo CL, Chu D. 2019. Research progress on the factors influencing microbiota diversity in insect. J. Biosaf 28:170. http://www.jbscn.net/swuaqxbcn/article/abstract/20190302. (In Chinese)

[ieag111-B44] Yang LJ , Li HW, Li XY, et al 2023. Diversity analysis of gut microbes of yellow spined bamboo locust *Ceracris kiangsu* Tsai. J. Environ. Entomol. 45:952–964. https://hjkcxb.alljournals.net/hjkcxb/ch/reader/create_pdf.aspx?file_no=202304012&flag=1&journal_id=hjkcxb&year_id=2023. (In Chinese)

[ieag111-B45] Yun JH , Roh SW, Whon TW, et al 2014. Insect gut bacterial diversity determined by environmental habitat, diet, developmental stage, and phylogeny of host. Appl. Environ. Microbiol. 80:5254–5264. 10.1128/AEM.01226-14 PMC4136111 24928884

[ieag111-B46] Zhu DK , Wen N, Peng KY, et al 2026. Geographical variation in the gut microbiota of *Duttaphrynus melanostictus*: relative contributions of environmental filtering and host physiological modulation. Ecol. Evol. 16:e73993. 10.1002/ece3.73993 PMC13348828 42428325

[ieag111-B47] Zhu PP , Xu LT. 2025. Friends or foes? The indispensable role of gut microbiota in plant–fungus–herbivore interactions. Integr. Zool. 20:1093–1095. 10.1111/1749-4877.13019 40673794

[ieag111-B48] Zou L , Stout MJ, Dunand RT. 2004. The effects of feeding by the rice water weevil, *Lissorhoptrus oryzophilus* Kushel, on the growth and yield components of rice, *Oryza sativa*. Agri. Forest Entomol. 6:47–54. 10.1111/j.1461-9555.2004.00203.x

