# Supplementary material for: Geographic differentiation of the gut microbiota in Lissorhoptrus oryzophilus (Coleoptera: Curculionidae): associations with environmental gradients and predicted functional potential
Source: J Insect Sci. 2026 Oct 9;26(5):ieag111. doi: 10.1093/jisesa/ieag111 (PMC13651693; doi:10.1093/jisesa/ieag111)
Supplement: Supplementary file 1 [file ieag111_supplementary_data.pdf]

Supplementary Tables Legends

Table S1 Metagenomic DNA analysis of gut microbiota from different geographic populations of *Lissorhoptrus oryzophilus.*

Table S2 Metagenomic sequencing data quality evaluation for gut microbiota from different geographic populations of *Lissorhoptrus oryzophilus.*

Table S3 Statistics of metagenomic assembly for gut microbiota of different geographic populations of *Lissorhoptrus oryzophilus.*

Table S4 Statistics of predicted genes and non-redundant gene set for gut microbiota in different geographic populations of *Lissorhoptrus oryzophilus.*

Table S5 Taxonomic annotation of unigenes derived from the *Lissorhoptrus oryzophilus* gut microbiota aligned to the NCBI Non-Redundant (NR) protein database

Table S6 Relative abundance of KEGG Level 1 pathways across geographic populations of *Lissorhoptrus oryzophilu* , normalized within each group

Table S7 Relative abundance of genes assigned to 45 KEGG Level 2 pathways across geographic populations of *Lissorhoptrus oryzophilus*

Table S8 Relative abundance of eggNOG functional categories across geographic populations of *Lissorhoptrus oryzophilus* , normalized within each group

Table S9 Relative abundance of five CAZy classes across geographic populations of *Lissorhoptrus oryzophilus* , normalized within each group

Table S10 Relative abundance of all identified CAZy families across geographic populations of *Lissorhoptrus oryzophilus*

Table S11 Relative abundance of all identified Antimicrobial Resistance Orthology (ARO)-annotated genes across geographic populations of *Lissorhoptrus oryzophilus*

Table S1 Metagenomic DNA analysis of gut microbiota from different geographic populations of *Lissorhoptrus oryzophilus*

**Serial Number**	**Sample Name**	**Qubit Concentration （ng /uL）**	**Total amount （ug）**	**260/280**	**260/230**	**Sample Grade***	**Test Result**
1	PB-1	152	5.32	2.062	2.384	A	eligible
2	PB-2	148.1	5.19	2.068	2.356	A	eligible
3	PB-3	148.9	5.21	2.062	2.416	A	eligible
4	SC-1	180	8.64	1.82	1.678	A	eligible
5	SC-2	173	8.3	1.835	1.633	A	eligible
6	SC-3	184	8.83	1.856	1.878	A	eligible
7	MDJ-1	183	8.78	1.846	1.82	A	eligible
8	MDJ-2	176	8.45	1.818	1.649	A	eligible
9	MDJ-3	178	8.54	1.851	1.742	A	eligible

Note: * Sample Grades A: Sample quality meets the requirements for library construction.

Table S2 Metagenomic sequencing data quality evaluation for gut microbiota from different geographic populations of *Lissorhoptrus oryzophilus*

Sample	Raw Reads	Raw Bases	Clean Reads	Clean Bases	Q20	Q30	GC	Effective
PB-1	765,994	114,899,100	764,622	112,788,770	99.72%	98.79%	47.00%	98.16%
PB-2	675,668	101,350,200	673,830	97,153,533	99.44%	98.17%	45.77%	95.86%
PB-3	792,488	118,873,200	790,040	114,780,156	99.40%	98.03%	47.08%	96.56%
MDJ-1	485,172	72,061,351	483,464	71,579,255	98.98%	96.31%	34.49%	99.38%
MDJ-2	570,490	84,778,775	568,654	84,270,774	98.89%	95.95%	35.51%	99.32%
MDJ-3	503,218	74,588,777	501,198	74,012,167	98.70%	95.28%	34.40%	99.30%
SC-1	498,184	73,368,844	496,516	72,916,531	98.72%	95.40%	34.75%	99.33%
SC-2	562,534	83,444,371	560,528	82,873,484	98.98%	96.34%	33.69%	99.40%
SC-3	554,780	82,354,503	552,760	81,777,298	98.83%	95.67%	33.68%	99.23%

Note: Raw Reads: number of raw sequencing reads; Raw Bases: total number of bases in raw reads; Clean Reads: number of reads after quality trimming; Clean Bases: total number of bases after quality trimming; Q20: Q20 percentage after quality trimming (%); Q30: Q30 percentage of reads remaining after quality trimming (%); GC: GC content after quality trimming (%); Effective: effective rate of the sequencing data (%).

Table S3 Statistics of metagenomic assembly for gut microbiota of different geographic populations of *Lissorhoptrus oryzophilus*

**Sample**	**Number**	**Total Len (bp)**	**Average Len (bp)**	**Max Len (bp)**	**Min Len (bp)**	**N50**	**L50**	**N90**	**L90**	**GC (%)**
PB-1	3141	4,999,651	1591.74	13,320	600	2024	732	753	2394	53.75
PB-2	3079	6,522,469	2118.37	19,046	502	3401	543	832	2151	49.68
PB-3	2900	7,109,211	2451.45	70,421	507	5740	268	818	1888	49.13
SC-1	3904	3,928,101	1006.17	6486	505	987	1379	683	3292	31.66
SC-2	4966	5,696,184	1147.04	9738	519	1156	1567	711	4099	30.64
SC-3	4139	4,369,384	1055.66	6808	523	1060	1411	693	3464	30.57
MDJ-1	3056	2,997,412	980.83	8407	524	976	1105	676	2587	31.36
MDJ-2	3615	4,483,961	1240.38	15,121	517	1180	904	702	2927	32.94
MDJ-3	3696	4,061,675	1098.94	9738	519	1087	1179	698	3070	31.57

Note: Sample: Sample ID; Number: Number of assembled contigs; Total Len: Total length of all contigs (bp); Average Len: Average length of contigs (bp); Max Len: Length of the longest contig (bp); Min Len: Length of the shortest contig (bp); N50: The length of the shortest contig in the set of largest contigs whose combined length represents at least 50% of the total assembly length.L50: The smallest number of contigs whose combined length represents at least 50% of the total assembly length. N90: The length of the shortest contig in the set of largest contigs whose combined length represents at least 90% of the total assembly length. L90: The smallest number of contigs whose combined length represents at least 90% of the total assembly length. GC%: The percentage of guanine (G) and cytosine (C) bases among the total bases in the assembly.

Table S4 Statistics of predicted genes and non-redundant gene set for gut microbiota in different geographic populations of *Lissorhoptrus oryzophilus*

Sample	Number	Integrity:Start	Integrity:End	Integrity:All	ntegrity:Non	Total Len(bp)	verage Len(b	Max Len(bp)	Min Len(bp)	N50	L50	GC (%)
PB-1	6493	1241<br>-19.11%	1970<br>-30.34%	3056<br>-47.07%	226<br>-3.48%	3,919,968	603.72	3,807	102	831	1599	55.85
PB-2	7246	1000<br>-13.80%	1657<br>-22.87%	4440<br>-61.28%	149<br>-2.06%	4,924,452	679.61	6,732	102	939	1741	52.61
PB-3	7189	766<br>-10.66%	1279<br>-17.79%	5043<br>-70.15%	101<br>-1.40%	5,225,364	726.86	17,046	102	1,011	1707	52.94
SC-1	2413	395<br>-16.37%	777<br>-32.20%	1163<br>-48.20%	78<br>-3.23%	834,804	345.96	5,919	102	471	534	39.7
SC-2	3489	536<br>-15.36%	950<br>-27.23%	1936<br>-55.49%	67<br>-1.92%	1,474,740	422.68	5,781	102	627	699	35.07
SC-3	2544	400<br>-15.72%	777<br>-30.54%	1302<br>-51.18%	65<br>-2.56%	909,375	357.46	5,220	102	477	559	35.76
MDJ-1	2242	459<br>-20.47%	723<br>-32.25%	975<br>-43.49%	85<br>-3.79%	857,925	382.66	2,925	102	543	498	35.51
MDJ-2	3784	570<br>-15.06%	1030<br>-27.22%	2103<br>-55.58%	81<br>-2.14%	1,888,278	499.02	4,989	102	717	776	37.16
MDJ-3	3077	564<br>-18.33%	905<br>-29.41%	1535<br>-49.89%	73<br>-2.37%	1,363,872	443.25	3,813	102	651	647	35.47
Unigenes	11,651	1057<br>-9.07%	2929<br>-25.14%	7491<br>-64.29%	174<br>-1.49%	7,024,143	602.88	17,046	102	966	2332	49

Note: Sample: sample ID; Number: predicted number of genes; Integrity: proportion of gene integrity (Start: genes with only a start codon; End: genes with only a stop codon; All: genes with both start and stop codons; None: genes lacking both start and stop codons); Total Len: total base pairs of all predicted genes; Average Len: average base pairs of predicted genes; Max Len: length of the longest gene; Min Len: length of the shortest gene; N50: the length of the shortest contig in the set of largest contigs whose combined length represents at least 50% of the total assembly length; L50: the smallest number of contigs whose combined length represents at least 50% of the total assembly length; GC (%): the percentage of guanine (G) and cytosine (C) bases among the total bases in the assembly.

Table S5 Taxonomic annotation of unigenes derived from the *Lissorhoptrus oryzophilus* gut microbiota aligned to the NCBI Non-Redundant (NR) protein database

Query_ID	Query_length	Subject_ID	Subject_length	Identity	*E* _value	Description	Taxonomy_ID	Taxonomy
MDJ-1_gene_2	700	WP_114517515.1	767	105/163(64.42)	5.65e-43	hypothetical protein [Wolbachia endosymbiont of Cylisticus convexus]	118728	d__Bacteria;k__norank;p__Proteobacteria;c__Alphaproteobacteria;o__Rickettsiales;f__Anaplasmataceae;g__Wolbachia;s__Wolbachia_endosymbiont_of_Cylisticus_convexus
MDJ-1_gene_3	648	OJH31309.1	1214	426/648(65.74)	7.54e-287	Phosphocholine transferase AnkX [Wolbachia endosymbiont of Armadillidium vulgare]	77039	d__Bacteria;k__norank;p__Proteobacteria;c__Alphaproteobacteria;o__Rickettsiales;f__Anaplasmataceae;g__Wolbachia;s__Wolbachia_endosymbiont_of_Armadillidium_vulgare
MDJ-1_gene_5	334	RMS48285.1	385	95/205(46.34)	1.43e-49	hypothetical protein ALP66_200078 [Pseudomonas amygdali pv. photiniae]	251724	d__Bacteria;k__norank;p__Proteobacteria;c__Gammaproteobacteria;o__Pseudomonadales;f__Pseudomonadaceae;g__Pseudomonas;s__Pseudomonas_amygdali
MDJ-1_gene_6	287	RMP03281.1	305	124/305(40.66)	2.39e-71	hypothetical protein ALQ30_200320 [Pseudomonas syringae pv. persicae]	237306	d__Bacteria;k__norank;p__Proteobacteria;c__Gammaproteobacteria;o__Pseudomonadales;f__Pseudomonadaceae;g__Pseudomonas;s__Pseudomonas_syringae_group_genomosp._3
MDJ-1_gene_7	228	ROZ98597.1	228	85/232(36.64)	1.05e-37	type IV secretion system protein [Yersinia pestis]	632	d__Bacteria;k__norank;p__Proteobacteria;c__Gammaproteobacteria;o__Enterobacterales;f__Yersiniaceae;g__Yersinia;s__Yersinia_pestis
MDJ-1_gene_10	261	WP_127087886.1	238	75/244(30.74)	2.70e-24	type IV secretion system protein VirB5 [Pectobacterium	1905730	d__Bacteria;k__norank;p__Proteobacteria;c__Gammaproteobacteria;o__Enterobacterales;f__Pectobacteriaceae;g__Pectobacterium;s__Pectobacterium_parmentieri
MDJ-1_gene_11	61	WP_131019408.1	67	27/56(48.21)	2.43e-11	hypothetical protein, partial [Obesumbacterium proteus]	82983	d__Bacteria;k__norank;p__Proteobacteria;c__Gammaproteobacteria;o__Enterobacterales;f__Hafniaceae;g__Obesumbacterium;s__Obesumbacterium_proteus
MDJ-1_gene_13	74	AGJ98567.1	74	70/74(94.59)	9.07e-41	Putative phage head stabilizing protein GpW [Wolbachia endosymbiont of Drosophila simulans wNo]	1236908	d__Bacteria;k__norank;p__Proteobacteria;c__Alphaproteobacteria;o__Rickettsiales;f__Anaplasmataceae;g__Wolbachia;s__Wolbachia_endosymbiont_of_Drosophila_simulans
MDJ-1_gene_14	188	WP_077190439.1	323	178/186(95.70)	3.94e-117	IS110 family transposase [Wolbachia pipientis]	955	d__Bacteria;k__norank;p__Proteobacteria;c__Alphaproteobacteria;o__Rickettsiales;f__Anaplasmataceae;g__Wolbachia;s__Wolbachia_pipientis
MDJ-1_gene_15	111	WP_017532054.1	323	106/109(97.25)	2.32e-67	IS110 family transposase [Wolbachia endosymbiont of	116598	d__Bacteria;k__norank;p__Proteobacteria;c__Alphaproteobacteria;o__Rickettsiales;f__Anaplasmataceae;g__Wolbachia;s__Wolbachia_endosymbiont_of_Diaphorina_citri
MDJ-1_gene_16	83	AOV87842.1	110	63/69(91.30)	1.34e-40	transposase [Wolbachia endosymbiont of Drosophila incompta]	1633785	d__Bacteria;k__norank;p__Proteobacteria;c__Alphaproteobacteria;o__Rickettsiales;f__Anaplasmataceae;g__Wolbachia;s__Wolbachia_endosymbiont_of_Drosophila_incompta
MDJ-1_gene_17	144	PBD15915.1	205	94/112(83.93)	4.43e-54	ankyrin repeat domain-containing protein [Wolbachia endosymbiont of Drosophila subpulchrella]	2033655	d__Bacteria;k__norank;p__Proteobacteria;c__Alphaproteobacteria;o__Rickettsiales;f__Anaplasmataceae;g__Wolbachia;s__Wolbachia_endosymbiont_of_Drosophila_subpulchrella
MDJ-1_gene_18	467	KEK20477.1	398	158/424(37.26)	3.31e-64	dihydropteridine reductase [Bacillus manliponensis]	574376	d__Bacteria;k__norank;p__Firmicutes;c__Bacilli;o__Bacillales;f__Bacillaceae;g__Bacillus;s__Bacillus_manliponensis
MDJ-1_gene_20	462	OAM03955.1	596	449/462(97.19)	0	hypothetical protein TV41_04265 [Wolbachia endosymbiont of Dactylopius coccus]	1605993	d__Bacteria;k__norank;p__Proteobacteria;c__Alphaproteobacteria;o__Rickettsiales;f__Anaplasmataceae;g__Wolbachia;s__Wolbachia_endosymbiont_of_Dactylopius_coccus
MDJ-1_gene_23	219	ONI57218.1	404	203/205(99.02)	8.51e-146	DNA methylase family protein [Wolbachia pipientis wVitA]	1379786	d__Bacteria;k__norank;p__Proteobacteria;c__Alphaproteobacteria;o__Rickettsiales;f__Anaplasmataceae;g__Wolbachia;s__Wolbachia_pipientis
MDJ-1_gene_25	502	PBQ25495.1	492	447/492(90.85)	9.27e-286	hypothetical protein BTO27_05300 [Wolbachia	2049495	d__Bacteria;k__norank;p__Proteobacteria;c__Alphaproteobacteria;o__Rickettsiales;f__Anaplasmataceae;g__Wolbachia;s__Wolbachia_pipientis
MDJ-1_gene_26	211	PBQ25496.1	229	202/209(96.65)	1.24e-146	hypothetical protein BTO27_05305, partial	2049495	d__Bacteria;k__norank;p__Proteobacteria;c__Alphaproteobacteria;o__Rickettsiales;f__Anaplasmataceae;g__Wolbachia;s__Wolbachia_pipientis
MDJ-1_gene_27	348	ONI58176.1	336	298/318(93.71)	6.85e-219	phage terminase large subunit family protein [Wolbachia	1379786	d__Bacteria;k__norank;p__Proteobacteria;c__Alphaproteobacteria;o__Rickettsiales;f__Anaplasmataceae;g__Wolbachia;s__Wolbachia_pipientis
MDJ-1_gene_29	290	PBQ29234.1	290	289/290(99.66)	2.37e-186	hypothetical protein BTO27_00580 [Wolbachia	2049495	d__Bacteria;k__norank;p__Proteobacteria;c__Alphaproteobacteria;o__Rickettsiales;f__Anaplasmataceae;g__Wolbachia;s__Wolbachia_pipientis

MDJ-1_gene_30	123	PBQ29235.1	123	121/123(98.37)	2.19e-84	glutaredoxin [Wolbachia pipientis wAus]	2049495	d__Bacteria;k__norank;p__Proteobacteria;c__Alphaproteobacteria;o__Rickettsiales;f__Anaplasmataceae;g__Wolbachia;s__Wolbachia_pipientis
MDJ-1_gene_31	974	WP_100551500.1	999	617/975(63.28)	0	Tn3 family transposase [Caedibacter taeniospiralis]	28907	d__Bacteria;k__norank;p__Proteobacteria;c__Gammaproteobacteria;o__Thiotrichales;f__Fastidiosibacteraceae;g__Caedibacter;s__Caedibacter_taeniospiralis
MDJ-1_gene_32	237	WP_018300619.1	236	179/232(77.16)	9.12e-130	hypothetical protein [Fangia hongkongensis]	270495	d__Bacteria;k__norank;p__Proteobacteria;c__Gammaproteobacteria;o__Thiotrichales;f__Fastidiosibacteraceae;g__Fangia;s__Fangia_hongkongensis
MDJ-1_gene_36	107	WP_095742865.1	297	107/107(100.00)	2.72e-59	helix-turn-helix domain-containing protein [Wolbachia endosymbiont of Drosophila	77038	d__Bacteria;k__norank;p__Proteobacteria;c__Alphaproteobacteria;o__Rickettsiales;f__Anaplasmataceae;g__Wolbachia;s__Wolbachia_endosymbiont_of_Drosophila_simulans
MDJ-1_gene_38	588	WP_088415204.1	629	480/589(81.49)	0	DNA mismatch repair endonuclease MutL [Wolbachia endosymbiont of Wuchereria	96496	d__Bacteria;k__norank;p__Proteobacteria;c__Alphaproteobacteria;o__Rickettsiales;f__Anaplasmataceae;g__Wolbachia;s__Wolbachia_endosymbiont_of_Wuchereria_bancrofti
MDJ-1_gene_40	381	WP_063631094.1	513	165/411(40.15)	8.23e-46	hypothetical protein [Wolbachia endosymbiont of Laodelphax striatella]	368602	d__Bacteria;k__norank;p__Proteobacteria;c__Alphaproteobacteria;o__Rickettsiales;f__Anaplasmataceae;g__Wolbachia;s__Wolbachia_endosymbiont_of_Laodelphax_striatellus
MDJ-1_gene_41	201	POG50125.1	1120	138/201(68.66)	7.30e-79	hypothetical protein BK222_02020 [Wolbachia sp.	1912095	d__Bacteria;k__norank;p__Proteobacteria;c__Alphaproteobacteria;o__Rickettsiales;f__Anaplasmataceae;g__Wolbachia;s__Wolbachia_sp._wMel_AMD
MDJ-1_gene_42	290	WP_077188055.1	291	277/289(95.85)	1.84e-197	Rpn family recombination-promoting nuclease/putative transposase [Wolbachia pipientis]	955	d__Bacteria;k__norank;p__Proteobacteria;c__Alphaproteobacteria;o__Rickettsiales;f__Anaplasmataceae;g__Wolbachia;s__Wolbachia_pipientis
MDJ-1_gene_44	337	WP_065095215.1	337	299/337(88.72)	5.34e-218	MULTISPECIES: IS110 family transposase [Wolbachia]	2640676	d__Bacteria;k__norank;p__Proteobacteria;c__Alphaproteobacteria;o__Rickettsiales;f__Anaplasmataceae;g__Wolbachia;s__norank
MDJ-1_gene_47	105	WP_114517895.1	105	104/105(99.05)	8.60e-69	hypothetical protein [Wolbachia endosymbiont of Cylisticus convexus]	118728	d__Bacteria;k__norank;p__Proteobacteria;c__Alphaproteobacteria;o__Rickettsiales;f__Anaplasmataceae;g__Wolbachia;s__Wolbachia_endosymbiont_of_Cylisticus_convexus
MDJ-1_gene_8	324	WP_001401675.1	359	80/268(29.85)	9.08e-38	trbL/VirB6 plasmid conjugal transfer family protein	562	d__Bacteria;k__norank;p__Proteobacteria;c__Gammaproteobacteria;o__Enterobacterales;f__Enterobacteriaceae;g__Escherichia;s__Escherichia_coli
MDJ-1_gene_48	170	WP_127463623.1	170	152/169(89.94)	1.69e-97	phage tail protein [Wolbachia endosymbiont of Bemisia tabaci]	215173	d__Bacteria;k__norank;p__Proteobacteria;c__Alphaproteobacteria;o__Rickettsiales;f__Anaplasmataceae;g__Wolbachia;s__Wolbachia_endosymbiont_of_Bemisia_tabaci
MDJ-1_gene_49	158	WP_064124761.1	158	148/158(93.67)	1.83e-102	hypothetical protein [Wolbachia endosymbiont of Dactylopius coccus]	None	Unclassified
MDJ-1_gene_50	154	WP_077188260.1	154	141/154(91.56)	9.87e-96	phage baseplate assembly protein V [Wolbachia pipientis]	955	d__Bacteria;k__norank;p__Proteobacteria;c__Alphaproteobacteria;o__Rickettsiales;f__Anaplasmataceae;g__Wolbachia;s__Wolbachia_pipientis
MDJ-1_gene_52	111	WP_065095147.1	111	99/111(89.19)	2.92e-63	MULTISPECIES: baseplate assembly protein W [Wolbachia]	2640676	d__Bacteria;k__norank;p__Proteobacteria;c__Alphaproteobacteria;o__Rickettsiales;f__Anaplasmataceae;g__Wolbachia;s__norank
MDJ-1_gene_53	264	WP_077188545.1	261	248/264(93.94)	2.09e-164	baseplate assembly protein J [Wolbachia pipientis]	953	d__Bacteria;k__norank;p__Proteobacteria;c__Alphaproteobacteria;o__Rickettsiales;f__Anaplasmataceae;g__Wolbachia;s__norank
MDJ-1_gene_58	100	WP_081178591.1	100	92/100(92.00)	1.12e-55	hypothetical protein [Rickettsia bellii]	33990	d__Bacteria;k__norank;p__Proteobacteria;c__Alphaproteobacteria;o__Rickettsiales;f__Rickettsiaceae;g__Rickettsia;s__Rickettsia_bellii
MDJ-1_gene_59	210	EER20867.1	210	207/210(98.57)	1.58e-142	ParA1 (plasmid) [Rickettsia endosymbiont of Ixodes	444612	d__Bacteria;k__norank;p__Proteobacteria;c__Alphaproteobacteria;o__Rickettsiales;f__Rickettsiaceae;g__Rickettsia;s__Rickettsia_endosymbiont_of_Ixodes_scapularis
MDJ-1_gene_60	161	EER20868.1	161	148/161(91.93)	4.64e-102	helix-turn-helix domain protein (plasmid) [Rickettsia endosymbiont of Ixodes	444612	d__Bacteria;k__norank;p__Proteobacteria;c__Alphaproteobacteria;o__Rickettsiales;f__Rickettsiaceae;g__Rickettsia;s__Rickettsia_endosymbiont_of_Ixodes_scapularis
MDJ-1_gene_64	46	WP_063630706.1	330	42/46(91.30)	1.00e-18	ankyrin repeat domain-containing protein [Wolbachia endosymbiont of Laodelphax striatella]	368602	d__Bacteria;k__norank;p__Proteobacteria;c__Alphaproteobacteria;o__Rickettsiales;f__Anaplasmataceae;g__Wolbachia;s__Wolbachia_endosymbiont_of_Laodelphax_striatellus
MDJ-1_gene_65	314	WP_017532282.1	314	313/314(99.68)	2.12e-217	IS110 family transposase [Wolbachia endosymbiont of	116598	d__Bacteria;k__norank;p__Proteobacteria;c__Alphaproteobacteria;o__Rickettsiales;f__Anaplasmataceae;g__Wolbachia;s__Wolbachia_endosymbiont_of_Diaphorina_citri

MDJ-1_gene_66	319	WP_064085970.1	389	247/266(92.86)	1.23e-180	phage tail protein [Wolbachia endosymbiont of Dactylopius coccus]	None	Unclassified
MDJ-1_gene_67	141	STX61066.1	144	93/136(68.38)	1.45e-71	Uncharacterised protein [Legionella israelensis]	454	d__Bacteria;k__norank;p__Proteobacteria;c__Gammaproteobacteria;o__Legionellales;f__Legionellaceae;g__Legionella;s__Legionella_israelensis
MDJ-1_gene_68	391	WP_017443570.1	392	243/400(60.75)	2.78e-156	hypothetical protein [Rickettsia gravesii]	354585	d__Bacteria;k__norank;p__Proteobacteria;c__Alphaproteobacteria;o__Rickettsiales;f__Rickettsiaceae;g__Rickettsia;s__Rickettsia_gravesii
MDJ-1_gene_69	181	WP_017443569.1	184	97/183(53.01)	3.08e-52	hypothetical protein [Rickettsia gravesii]	354585	d__Bacteria;k__norank;p__Proteobacteria;c__Alphaproteobacteria;o__Rickettsiales;f__Rickettsiaceae;g__Rickettsia;s__Rickettsia_gravesii
MDJ-1_gene_71	253	PCG17143.1	253	247/253(97.63)	9.89e-181	cytochrome c1 [Wolbachia	None	Unclassified
MDJ-1_gene_73	396	RUM45435.1	583	195/397(49.12)	8.03e-137	hypothetical protein DSY80_03320, partial	2066021	d__Bacteria;k__norank;p__Proteobacteria;c__Deltaproteobacteria;o__Desulfobacterales;f__Desulfobulbaceae;g__Desulfocapsa;s__Desulfocapsa_sp.
MDJ-1_gene_74	257	POS82328.1	811	68/243(27.98)	2.30e-14	hypothetical protein EPUL_005100, partial [Erysiphe	225359	d__Eukaryota;k__Fungi;p__Ascomycota;c__Leotiomycetes;o__Erysiphales;f__Erysiphaceae;g__Erysiphe;s__Erysiphe_pulchra
MDJ-1_gene_79	793	WP_019078827.1	888	118/162(72.84)	2.07e-58	MULTISPECIES: hypothetical protein [Wolbachia]	2640676	d__Bacteria;k__norank;p__Proteobacteria;c__Alphaproteobacteria;o__Rickettsiales;f__Anaplasmataceae;g__Wolbachia;s__norank
MDJ-1_gene_88	535	AGJ99782.1	534	488/534(91.39)	0	CTP synthase [Wolbachia endosymbiont of Drosophila simulans wHa]	1236909	d__Bacteria;k__norank;p__Proteobacteria;c__Alphaproteobacteria;o__Rickettsiales;f__Anaplasmataceae;g__Wolbachia;s__Wolbachia_endosymbiont_of_Drosophila_simulans
MDJ-1_gene_93	158	ONI57217.1	155	141/156(90.38)	1.07e-98	holliday junction resolvasome [Wolbachia pipientis wVitA]	1379786	d__Bacteria;k__norank;p__Proteobacteria;c__Alphaproteobacteria;o__Rickettsiales;f__Anaplasmataceae;g__Wolbachia;s__Wolbachia_pipientis
MDJ-1_gene_94	208	AGJ99828.1	406	196/207(94.69)	1.15e-138	DNA methylase [Wolbachia endosymbiont of Drosophila simulans wHa]	1236909	d__Bacteria;k__norank;p__Proteobacteria;c__Alphaproteobacteria;o__Rickettsiales;f__Anaplasmataceae;g__Wolbachia;s__Wolbachia_endosymbiont_of_Drosophila_simulans
MDJ-1_gene_96	119	WP_077188192.1	119	116/118(98.31)	2.51e-70	molecular chaperone DnaJ [Wolbachia pipientis]	955	d__Bacteria;k__norank;p__Proteobacteria;c__Alphaproteobacteria;o__Rickettsiales;f__Anaplasmataceae;g__Wolbachia;s__Wolbachia_pipientis
MDJ-1_gene_97	194	ONI55953.1	194	192/194(98.97)	1.96e-135	nifU-like domain protein [Wolbachia pipientis wUni]	1379787	d__Bacteria;k__norank;p__Proteobacteria;c__Alphaproteobacteria;o__Rickettsiales;f__Anaplasmataceae;g__Wolbachia;s__Wolbachia_pipientis
MDJ-1_gene_98	408	WP_096616101.1	408	399/408(97.79)	4.45e-287	histidine--tRNA ligase [Wolbachia pipientis]	955	d__Bacteria;k__norank;p__Proteobacteria;c__Alphaproteobacteria;o__Rickettsiales;f__Anaplasmataceae;g__Wolbachia;s__Wolbachia_pipientis
MDJ-1_gene_101	325	PCG11436.1	325	317/325(97.54)	2.73e-230	quinone oxidoreductase [Wolbachia pipientis]	None	Unclassified
MDJ-1_gene_102	176	PBQ26867.1	176	141/175(80.57)	9.63e-100	hypothetical protein BTO27_04305 [Wolbachia	2049495	d__Bacteria;k__norank;p__Proteobacteria;c__Alphaproteobacteria;o__Rickettsiales;f__Anaplasmataceae;g__Wolbachia;s__Wolbachia_pipientis
MDJ-1_gene_108	207	WP_064085635.1	207	206/207(99.52)	1.00e-148	heme ABC exporter ATP-binding protein CcmA [Wolbachia endosymbiont of Dactylopius coccus]	None	Unclassified
MDJ-1_gene_111	511	RGV57858.1	324	42/126(33.33)	3.66e-11	ankyrin repeat domain-containing protein [Bacteroides fragilis]	817	d__Bacteria;k__norank;p__Bacteroidetes;c__Bacteroidia;o__Bacteroidales;f__Bacteroidaceae;g__Bacteroides;s__Bacteroides_fragilis
MDJ-1_gene_112	151	WP_068042359.1	382	127/150(84.67)	3.30e-79	hypothetical protein [Rickettsia endosymbiont of Bemisia tabaci]	337479	d__Bacteria;k__norank;p__Proteobacteria;c__Alphaproteobacteria;o__Rickettsiales;f__Rickettsiaceae;g__Rickettsia;s__Rickettsia_endosymbiont_of_Bemisia_tabaci
MDJ-1_gene_119	113	PWS21318.1	80	26/43(60.47)	1.10e-10	hypothetical protein DKP78_24225, partial	1352	d__Bacteria;k__norank;p__Firmicutes;c__Bacilli;o__Lactobacillales;f__Enterococcaceae;g__Enterococcus;s__Enterococcus_faecium
MDJ-1_gene_124	252	KDB19167.1	249	228/255(89.41)	3.05e-121	ankyrin repeat-containing prophage LambdaW1 [Wolbachia endosymbiont of Glossina morsitans morsitans]	1150948	d__Bacteria;k__norank;p__Proteobacteria;c__Alphaproteobacteria;o__Rickettsiales;f__Anaplasmataceae;g__Wolbachia;s__Wolbachia_endosymbiont_of_Glossina_morsitans_morsitans
MDJ-1_gene_125	157	CAQ54448.1	157	147/157(93.63)	4.68e-61	Ankyrin repeat domain protein [Wolbachia endosymbiont of Culex quinquefasciatus Pel]	570417	d__Bacteria;k__norank;p__Proteobacteria;c__Alphaproteobacteria;o__Rickettsiales;f__Anaplasmataceae;g__Wolbachia;s__Wolbachia_endosymbiont_of_Culex_quinquefasciatus

MDJ-1_gene_132	208	WP_016682675.1	276	57/139(41.01)	1.44e-24	type IV secretion system protein, partial [Yersinia pestis]	632	d__Bacteria;k__norank;p__Proteobacteria;c__Gammaproteobacteria;o__Enterobacterales;f__Yersiniaceae;g__Yersinia;s__Yersinia_pestis
MDJ-1_gene_133	228	ROZ98597.1	228	80/218(36.70)	9.26e-36	type IV secretion system protein [Yersinia pestis]	632	d__Bacteria;k__norank;p__Proteobacteria;c__Gammaproteobacteria;o__Enterobacterales;f__Yersiniaceae;g__Yersinia;s__Yersinia_pestis
MDJ-1_gene_134	192	RML44969.1	305	70/209(33.49)	9.31e-28	hypothetical protein ALQ95_200096 [Pseudomonas	55398	d__Bacteria;k__norank;p__Proteobacteria;c__Gammaproteobacteria;o__Pseudomonadales;f__Pseudomonadaceae;g__Pseudomonas;s__Pseudomonas_syringae_group_genomosp._3
MDJ-1_gene_136	330	CAQ54517.1	331	234/332(70.48)	6.44e-139	putative RDD domain transmembrane protein [Wolbachia endosymbiont of Culex quinquefasciatus Pel]	570417	d__Bacteria;k__norank;p__Proteobacteria;c__Alphaproteobacteria;o__Rickettsiales;f__Anaplasmataceae;g__Wolbachia;s__Wolbachia_endosymbiont_of_Culex_quinquefasciatus
MDJ-1_gene_148	417	YP_002321389.1	420	115/366(31.42)	4.61e-53	semaphorin-like protein [Oryctes rhinoceros nudivirus]	92521	d__Viruses;k__norank;p__norank;c__norank;o__norank;f__Nudiviridae;g__Alphanudivirus;s__Oryctes_rhinoceros_nudivirus
MDJ-1_gene_150	139	PVZ99478.1	628	51/119(42.86)	6.65e-24	hypothetical protein BB558_004496 [Smittium	133377	d__Eukaryota;k__Fungi;p__Zoopagomycota;c__Harpellomycetes;o__Harpellales;f__Legeriomycetaceae;g__Smittium;s__Smittium_angustum
MDJ-1_gene_151	200	OCA06716.1	282	187/195(95.90)	1.10e-131	pseudouridine synthase, RluA family protein [Wolbachia endosymbiont of Trichogramma	125593	d__Bacteria;k__norank;p__Proteobacteria;c__Alphaproteobacteria;o__Rickettsiales;f__Anaplasmataceae;g__Wolbachia;s__Wolbachia_endosymbiont_of_Trichogramma_pretiosum
MDJ-1_gene_152	115	PBD15475.1	116	110/115(95.65)	3.32e-62	diacylglycerol kinase [Wolbachia endosymbiont of Drosophila subpulchrella]	2033655	d__Bacteria;k__norank;p__Proteobacteria;c__Alphaproteobacteria;o__Rickettsiales;f__Anaplasmataceae;g__Wolbachia;s__Wolbachia_endosymbiont_of_Drosophila_subpulchrella
MDJ-1_gene_161	103	RJQ46050.1	103	65/100(65.00)	9.95e-40	30S ribosomal protein S10 [Gammaproteobacteria	1913989	d__Bacteria;k__norank;p__Proteobacteria;c__Gammaproteobacteria;o__norank;f__norank;g__norank;s__Gammaproteobacteria_bacterium
MDJ-1_gene_162	493	BBA85267.1	700	316/489(64.62)	3.96e-213	elongation factor G [endosymbiont of Sipalinus gigas]	1972134	d__Bacteria;k__norank;p__Proteobacteria;c__Gammaproteobacteria;o__norank;f__norank;g__Candidatus_Nardonella;s__endosymbiont_of_Sipalinus_gigas
MDJ-1_gene_163	49	WP_129169998.1	699	30/49(61.22)	2.32e-11	elongation factor G [Candidatus Marinimicrobia bacterium]	None	Unclassified
MDJ-1_gene_169	76	WP_052692921.1	60	51/60(85.00)	3.37e-32	hypothetical protein [Rickettsia bellii]	None	Unclassified
MDJ-1_gene_170	205	EER20755.1	190	167/194(86.08)	3.28e-104	conserved hypothetical protein (plasmid) [Rickettsia endosymbiont of Ixodes	444612	d__Bacteria;k__norank;p__Proteobacteria;c__Alphaproteobacteria;o__Rickettsiales;f__Rickettsiaceae;g__Rickettsia;s__Rickettsia_endosymbiont_of_Ixodes_scapularis
MDJ-1_gene_174	490	TDB41637.1	478	288/460(62.61)	2.97e-207	group II intron reverse transcriptase/maturase [Photorhabdus sp. MEX20-17]	2100166	d__Bacteria;k__norank;p__Proteobacteria;c__Gammaproteobacteria;o__Enterobacterales;f__Morganellaceae;g__Photorhabdus;s__Photorhabdus_khanii
MDJ-1_gene_176	592	WP_114517780.1	713	451/592(76.18)	1.70e-271	phage tail tape measure protein, partial [Wolbachia endosymbiont of Cylisticus convexus]	118728	d__Bacteria;k__norank;p__Proteobacteria;c__Alphaproteobacteria;o__Rickettsiales;f__Anaplasmataceae;g__Wolbachia;s__Wolbachia_endosymbiont_of_Cylisticus_convexus
MDJ-1_gene_179	428	EAL58619.1	406	298/428(69.63)	2.56e-210	conserved hypothetical protein [Wolbachia endosymbiont of Drosophila ananassae]	307502	d__Bacteria;k__norank;p__Proteobacteria;c__Alphaproteobacteria;o__Rickettsiales;f__Anaplasmataceae;g__Wolbachia;s__Wolbachia_endosymbiont_of_Drosophila_ananassae
MDJ-1_gene_190	100	WP_041077565.1	234	96/100(96.00)	2.49e-64	tRNA (guanosine(37)-N1)-methyltransferase TrmD [Rickettsia asembonensis]	1068590	d__Bacteria;k__norank;p__Proteobacteria;c__Alphaproteobacteria;o__Rickettsiales;f__Rickettsiaceae;g__Rickettsia;s__Rickettsia_asembonensis
MDJ-1_gene_191	445	KJV90348.1	445	353/445(79.33)	7.18e-259	hypothetical protein RBEAN4_1351 [Rickettsia bellii	1359193	d__Bacteria;k__norank;p__Proteobacteria;c__Alphaproteobacteria;o__Rickettsiales;f__Rickettsiaceae;g__Rickettsia;s__Rickettsia_bellii
MDJ-1_gene_192	59	ABV84452.1	246	41/44(93.18)	1.64e-17	tRNA (guanine-n1)-methyltransferase [Rickettsia massiliae MTU5]	416276	d__Bacteria;k__norank;p__Proteobacteria;c__Alphaproteobacteria;o__Rickettsiales;f__Rickettsiaceae;g__Rickettsia;s__Rickettsia_massiliae

MDJ-1_gene_197	133	XP_018294527.1	281	61/132(46.21)	3.79e-36	hypothetical protein PHYBLDRAFT_36080 [Phycomyces blakesleeanus	763407	d__Eukaryota;k__Fungi;p__Mucoromycota;c__Mucoromycetes;o__Mucorales;f__Phycomycetaceae;g__Phycomyces;s__Phycomyces_blakesleeanus
MDJ-1_gene_203	109	ONI57837.1	109	107/109(98.17)	1.64e-68	helix-turn-helix family protein [Wolbachia pipientis wVitA]	1379786	d__Bacteria;k__norank;p__Proteobacteria;c__Alphaproteobacteria;o__Rickettsiales;f__Anaplasmataceae;g__Wolbachia;s__Wolbachia_pipientis
MDJ-1_gene_204	133	AGJ99405.1	136	129/130(99.23)	7.56e-86	Putative transcriptional regulator [Wolbachia endosymbiont of Drosophila simulans wNo]	1236908	d__Bacteria;k__norank;p__Proteobacteria;c__Alphaproteobacteria;o__Rickettsiales;f__Anaplasmataceae;g__Wolbachia;s__Wolbachia_endosymbiont_of_Drosophila_simulans
MDJ-1_gene_207	313	CAQ54180.1	308	306/308(99.35)	8.28e-211	Frusctose-1,6-bisphosphatase, class II [Wolbachia endosymbiont of Culex quinquefasciatus Pel]	570417	d__Bacteria;k__norank;p__Proteobacteria;c__Alphaproteobacteria;o__Rickettsiales;f__Anaplasmataceae;g__Wolbachia;s__Wolbachia_endosymbiont_of_Culex_quinquefasciatus
MDJ-1_gene_213	206	CQD09011.1	206	205/206(99.51)	1.54e-147	Putative Lipoprotein [Wolbachia endosymbiont wPip_Mol of Culex molestus]	1402163	d__Bacteria;k__norank;p__Proteobacteria;c__Alphaproteobacteria;o__Rickettsiales;f__Anaplasmataceae;g__Wolbachia;s__Wolbachia_endosymbiont_of_Culex_molestus
MDJ-1_gene_215	99	PBQ29197.1	99	97/99(97.98)	6.27e-57	integration host factor subunit alpha [Wolbachia pipientis wAus]	2049495	d__Bacteria;k__norank;p__Proteobacteria;c__Alphaproteobacteria;o__Rickettsiales;f__Anaplasmataceae;g__Wolbachia;s__Wolbachia_pipientis
MDJ-1_gene_218	262	WP_010403563.1	262	254/262(96.95)	2.07e-182	TatD family deoxyribonuclease [Wolbachia endosymbiont of Nasonia vitripennis]	2640676	d__Bacteria;k__norank;p__Proteobacteria;c__Alphaproteobacteria;o__Rickettsiales;f__Anaplasmataceae;g__Wolbachia;s__norank
MDJ-1_gene_222	45	WP_017443570.1	392	39/45(86.67)	7.98e-18	hypothetical protein [Rickettsia gravesii]	354585	d__Bacteria;k__norank;p__Proteobacteria;c__Alphaproteobacteria;o__Rickettsiales;f__Rickettsiaceae;g__Rickettsia;s__Rickettsia_gravesii
MDJ-1_gene_223	71	WP_068042359.1	382	50/67(74.63)	6.60e-22	hypothetical protein [Rickettsia endosymbiont of Bemisia tabaci]	337479	d__Bacteria;k__norank;p__Proteobacteria;c__Alphaproteobacteria;o__Rickettsiales;f__Rickettsiaceae;g__Rickettsia;s__Rickettsia_endosymbiont_of_Bemisia_tabaci
MDJ-1_gene_224	184	WP_017443569.1	184	139/184(75.54)	1.26e-85	hypothetical protein [Rickettsia gravesii]	354585	d__Bacteria;k__norank;p__Proteobacteria;c__Alphaproteobacteria;o__Rickettsiales;f__Rickettsiaceae;g__Rickettsia;s__Rickettsia_gravesii
MDJ-1_gene_230	181	WP_010402757.1	184	173/184(94.02)	3.73e-107	MULTISPECIES: 50S ribosomal protein L9 [Wolbachia]	180837	d__Bacteria;k__norank;p__Proteobacteria;c__Alphaproteobacteria;o__Rickettsiales;f__Anaplasmataceae;g__Wolbachia;s__Wolbachia_endosymbiont_of_Nasonia_vitripennis
MDJ-1_gene_233	111	OMH81055.1	556	39/110(35.45)	2.68e-15	EH domain-containing protein 1 [Zancudomyces culisetae]	1213189	d__Eukaryota;k__Fungi;p__Zoopagomycota;c__Harpellomycetes;o__Harpellales;f__Legeriomycetaceae;g__Zancudomyces;s__Zancudomyces_culisetae
MDJ-1_gene_237	171	EIJ34101.1	736	56/142(39.44)	1.19e-21	Animal heme peroxidase [Thiothrix nivea DSM 5205]	870187	d__Bacteria;k__norank;p__Proteobacteria;c__Gammaproteobacteria;o__Thiotrichales;f__Thiotrichaceae;g__Thiothrix;s__Thiothrix_nivea
MDJ-1_gene_241	151	WP_010404199.1	151	149/151(98.68)	4.95e-99	outer membrane lipid asymmetry maintenance protein MlaD [Wolbachia endosymbiont of Nasonia vitripennis]	180837	d__Bacteria;k__norank;p__Proteobacteria;c__Alphaproteobacteria;o__Rickettsiales;f__Anaplasmataceae;g__Wolbachia;s__Wolbachia_endosymbiont_of_Nasonia_vitripennis
MDJ-1_gene_243	45	RDD34554.1	91	22/25(88.00)	5.67e-07	Transposase [Wolbachia endosymbiont of Cylisticus convexus]	118728	d__Bacteria;k__norank;p__Proteobacteria;c__Alphaproteobacteria;o__Rickettsiales;f__Anaplasmataceae;g__Wolbachia;s__Wolbachia_endosymbiont_of_Cylisticus_convexus
MDJ-1_gene_247	440	PBD15602.1	437	358/433(82.68)	4.53e-268	group II intron reverse transcriptase/maturase [Wolbachia endosymbiont of Drosophila subpulchrella]	2033655	d__Bacteria;k__norank;p__Proteobacteria;c__Alphaproteobacteria;o__Rickettsiales;f__Anaplasmataceae;g__Wolbachia;s__Wolbachia_endosymbiont_of_Drosophila_subpulchrella
MDJ-1_gene_250	184	AGJ98584.1	185	142/173(82.08)	1.45e-75	hypothetical protein wNo_01430 [Wolbachia endosymbiont of Drosophila simulans wNo]	1236908	d__Bacteria;k__norank;p__Proteobacteria;c__Alphaproteobacteria;o__Rickettsiales;f__Anaplasmataceae;g__Wolbachia;s__Wolbachia_endosymbiont_of_Drosophila_simulans
MDJ-1_gene_253	339	WP_064085736.1	335	259/339(76.40)	5.79e-169	hypothetical protein [Wolbachia endosymbiont of Dactylopius coccus]	None	Unclassified

MDJ-1_gene_255	172	WP_108784434.1	172	168/172(97.67)	1.07e-110	translation initiation factor IF-3 [Wolbachia endosymbiont of Bemisia tabaci]	215173	d__Bacteria;k__norank;p__Proteobacteria;c__Alphaproteobacteria;o__Rickettsiales;f__Anaplasmataceae;g__Wolbachia;s__Wolbachia_endosymbiont_of_Bemisia_tabaci
MDJ-1_gene_257	66	RYE13645.1	39	29/36(80.56)	1.58e-11	conjugal transfer protein TrbI, partial [Sphingobacteriaceae	2021370	d__Bacteria;k__norank;p__Bacteroidetes;c__Sphingobacteriia;o__Sphingobacteriales;f__Sphingobacteriaceae;g__norank;s__Sphingobacteriaceae_bacterium
MDJ-1_gene_269	196	WP_053217360.1	281	36/94(38.30)	4.68e-07	ion transporter [Virgibacillus senegalensis]	1499679	d__Bacteria;k__norank;p__Firmicutes;c__Bacilli;o__Bacillales;f__Bacillaceae;g__Virgibacillus;s__Virgibacillus_senegalensis
MDJ-1_gene_271	47	WP_029237693.1	47	46/47(97.87)	1.80e-24	hypothetical protein [Wolbachia endosymbiont of Nasonia vitripennis]	2640676	d__Bacteria;k__norank;p__Proteobacteria;c__Alphaproteobacteria;o__Rickettsiales;f__Anaplasmataceae;g__Wolbachia;s__norank
MDJ-1_gene_272	50	WP_029237694.1	50	49/50(98.00)	2.94e-31	hypothetical protein [Wolbachia endosymbiont of Nasonia vitripennis]	2640676	d__Bacteria;k__norank;p__Proteobacteria;c__Alphaproteobacteria;o__Rickettsiales;f__Anaplasmataceae;g__Wolbachia;s__norank
MDJ-1_gene_275	97	OAJ45083.1	1837	29/38(76.32)	1.71e-10	hypothetical protein BDEG_28248 [Batrachochytrium dendrobatidis JEL423]	403673	d__Eukaryota;k__Fungi;p__Chytridiomycota;c__Chytridiomycetes;o__Rhizophydiales;f__norank;g__Batrachochytrium;s__Batrachochytrium_dendrobatidis
MDJ-1_gene_278	173	PBQ27261.1	173	166/173(95.95)	1.64e-110	cytochrome B [Wolbachia pipientis wAus]	2049495	d__Bacteria;k__norank;p__Proteobacteria;c__Alphaproteobacteria;o__Rickettsiales;f__Anaplasmataceae;g__Wolbachia;s__Wolbachia_pipientis
MDJ-1_gene_282	338	WP_129827251.1	338	329/338(97.34)	1.41e-234	extracellular solute-binding protein [Wolbachia pipientis]	955	d__Bacteria;k__norank;p__Proteobacteria;c__Alphaproteobacteria;o__Rickettsiales;f__Anaplasmataceae;g__Wolbachia;s__Wolbachia_pipientis
MDJ-1_gene_287	94	PBQ28089.1	94	94/94(100.00)	9.86e-63	30S ribosomal protein S19 [Wolbachia pipientis wAus]	2049495	d__Bacteria;k__norank;p__Proteobacteria;c__Alphaproteobacteria;o__Rickettsiales;f__Anaplasmataceae;g__Wolbachia;s__Wolbachia_pipientis
MDJ-1_gene_290	376	WP_096097267.1	518	345/376(91.76)	4.35e-241	recombinase family protein [Wolbachia pipientis]	955	d__Bacteria;k__norank;p__Proteobacteria;c__Alphaproteobacteria;o__Rickettsiales;f__Anaplasmataceae;g__Wolbachia;s__Wolbachia_pipientis
MDJ-1_gene_302	172	WP_019236534.1	527	77/78(98.72)	2.59e-40	ankyrin repeat domain-containing protein, partial [Wolbachia	955	d__Bacteria;k__norank;p__Proteobacteria;c__Alphaproteobacteria;o__Rickettsiales;f__Anaplasmataceae;g__Wolbachia;s__Wolbachia_pipientis
MDJ-1_gene_308	139	WP_108784119.1	139	128/139(92.09)	7.53e-86	nucleoside-diphosphate kinase [Wolbachia endosymbiont of Bemisia tabaci]	215173	d__Bacteria;k__norank;p__Proteobacteria;c__Alphaproteobacteria;o__Rickettsiales;f__Anaplasmataceae;g__Wolbachia;s__Wolbachia_endosymbiont_of_Bemisia_tabaci
MDJ-1_gene_309	243	AGJ98838.1	269	191/274(69.71)	8.26e-119	hypothetical protein wNo_04110 [Wolbachia endosymbiont of Drosophila simulans wNo]	1236908	d__Bacteria;k__norank;p__Proteobacteria;c__Alphaproteobacteria;o__Rickettsiales;f__Anaplasmataceae;g__Wolbachia;s__Wolbachia_endosymbiont_of_Drosophila_simulans
MDJ-1_gene_316	215	WP_032139750.1	215	202/215(93.95)	5.70e-143	ParA family protein [Rickettsia tamurae]	334545	d__Bacteria;k__norank;p__Proteobacteria;c__Alphaproteobacteria;o__Rickettsiales;f__Rickettsiaceae;g__Rickettsia;s__Rickettsia_tamurae
MDJ-1_gene_317	97	ARD88240.1	97	93/97(95.88)	1.68e-60	hypothetical protein A3305_07730 (plasmid)	33989	d__Bacteria;k__norank;p__Proteobacteria;c__Alphaproteobacteria;o__Rickettsiales;f__Rickettsiaceae;g__Rickettsia;s__Rickettsia_amblyommatis
MDJ-1_gene_318	68	ARD88239.1	67	63/67(94.03)	7.39e-35	hypothetical protein A3305_07725 (plasmid)	33989	d__Bacteria;k__norank;p__Proteobacteria;c__Alphaproteobacteria;o__Rickettsiales;f__Rickettsiaceae;g__Rickettsia;s__Rickettsia_amblyommatis
MDJ-1_gene_329	87	RUA06468.1	1672	38/86(44.19)	4.23e-13	hypothetical protein DSY43_01915	1913989	d__Bacteria;k__norank;p__Proteobacteria;c__Gammaproteobacteria;o__norank;f__norank;g__norank;s__Gammaproteobacteria_bacterium
MDJ-1_gene_333	239	OUU19531.1	1556	88/200(44.00)	1.56e-43	hypothetical protein CBC13_12255 [Planctomycetia	1986839	d__Bacteria;k__norank;p__Planctomycetes;c__Planctomycetia;o__norank;f__norank;g__norank;s__Planctomycetia_bacterium_TMED53
MDJ-1_gene_334	129	RYE27181.1	1800	46/123(37.40)	8.72e-16	DUF1759 domain-containing protein [Sphingobacteriaceae	2021370	d__Bacteria;k__norank;p__Bacteroidetes;c__Sphingobacteriia;o__Sphingobacteriales;f__Sphingobacteriaceae;g__norank;s__Sphingobacteriaceae_bacterium
MDJ-1_gene_342	105	PKK80305.1	2047	47/104(45.19)	1.25e-20	hypothetical protein RhiirC2_145426 [Rhizophagus	588596	d__Eukaryota;k__Fungi;p__Mucoromycota;c__Glomeromycetes;o__Glomerales;f__Glomeraceae;g__Rhizophagus;s__Rhizophagus_irregularis
MDJ-1_gene_352	98	RYE05570.1	82	32/81(39.51)	8.03e-10	CopG family transcriptional regulator [Rickettsiaceae	2023360	d__Bacteria;k__norank;p__Proteobacteria;c__Alphaproteobacteria;o__Rickettsiales;f__Rickettsiaceae;g__norank;s__Rickettsiaceae_bacterium
MDJ-1_gene_353	162	KDO02109.1	156	54/157(34.39)	2.49e-24	hypothetical protein REISMN_08630 (plasmid)	1462938	d__Bacteria;k__norank;p__Proteobacteria;c__Alphaproteobacteria;o__Rickettsiales;f__Rickettsiaceae;g__Rickettsia;s__Rickettsia_buchneri

MDJ-1_gene_368	64	PWS22913.1	124	30/55(54.55)	7.91e-16	hypothetical protein DKP78_15895, partial	1352	d__Bacteria;k__norank;p__Firmicutes;c__Bacilli;o__Lactobacillales;f__Enterococcaceae;g__Enterococcus;s__Enterococcus_faecium
MDJ-1_gene_369	134	PWS22913.1	124	27/52(51.92)	1.09e-06	hypothetical protein DKP78_15895, partial	1352	d__Bacteria;k__norank;p__Firmicutes;c__Bacilli;o__Lactobacillales;f__Enterococcaceae;g__Enterococcus;s__Enterococcus_faecium
MDJ-1_gene_371	192	AIA94545.1	62	50/62(80.65)	5.26e-32	trehalase, partial [uncultured bacterium]	77133	d__Bacteria;k__norank;p__norank;c__norank;o__norank;f__norank;g__norank;s__uncultured_bacterium
MDJ-1_gene_377	240	PCG11258.1	240	237/240(98.75)	4.69e-168	phosphoribosylaminoimidazolesuccinocarboxamide synthase [Wolbachia pipientis]	None	Unclassified
MDJ-1_gene_393	227	WP_064086014.1	227	222/227(97.80)	1.51e-153	phosphatidylserine decarboxylase [Wolbachia endosymbiont of Dactylopius coccus]	None	Unclassified
MDJ-1_gene_394	256	WP_063631018.1	256	255/256(99.61)	6.64e-176	CDP-diacylglycerol--serine O-phosphatidyltransferase [Wolbachia endosymbiont of Laodelphax striatella]	2640676	d__Bacteria;k__norank;p__Proteobacteria;c__Alphaproteobacteria;o__Rickettsiales;f__Anaplasmataceae;g__Wolbachia;s__norank
MDJ-1_gene_404	223	OSY19256.1	647	110/221(49.77)	1.34e-66	hypothetical protein B0D75_01370 [Entomoplasmatales bacterium	None	Unclassified
MDJ-1_gene_406	87	WP_084741746.1	71	69/71(97.18)	3.11e-44	MULTISPECIES: hypothetical protein [Wolbachia]	None	Unclassified
MDJ-1_gene_412	295	WP_010401351.1	295	293/295(99.32)	9.92e-202	protoheme IX farnesyltransferase [Wolbachia endosymbiont of Nasonia vitripennis]	2640676	d__Bacteria;k__norank;p__Proteobacteria;c__Alphaproteobacteria;o__Rickettsiales;f__Anaplasmataceae;g__Wolbachia;s__norank
MDJ-1_gene_416	86	WP_092403603.1	203	35/81(43.21)	8.11e-14	reverse transcriptase family protein [bacterium 2013Ark19i]	None	Unclassified
MDJ-1_gene_425	356	AGJ98525.1	339	332/339(97.94)	9.43e-238	Transposase [Wolbachia endosymbiont of Drosophila simulans wNo]	1236908	d__Bacteria;k__norank;p__Proteobacteria;c__Alphaproteobacteria;o__Rickettsiales;f__Anaplasmataceae;g__Wolbachia;s__Wolbachia_endosymbiont_of_Drosophila_simulans
MDJ-1_gene_429	74	PCG16568.1	75	65/72(90.28)	1.97e-33	hypothetical protein CO538_05905 [Wolbachia	None	Unclassified
MDJ-1_gene_430	46	CCE77201.1	46	44/46(95.65)	2.24e-20	hypothetical protein WALBB_1590005 [Wolbachia pipientis wAlbB]	1116230	d__Bacteria;k__norank;p__Proteobacteria;c__Alphaproteobacteria;o__Rickettsiales;f__Anaplasmataceae;g__Wolbachia;s__Wolbachia_pipientis
MDJ-1_gene_435	212	WP_106480545.1	121	37/105(35.24)	3.39e-10	MULTISPECIES: fibronectin type III domain-containing protein [Butyricimonas]	574697	d__Bacteria;k__norank;p__Bacteroidetes;c__Bacteroidia;o__Bacteroidales;f__Odoribacteraceae;g__Butyricimonas;s__norank
MDJ-1_gene_440	108	WP_064660563.1	407	39/89(43.82)	2.51e-11	hypothetical protein [Anaplasma phagocytophilum]	None	Unclassified
MDJ-1_gene_441	84	WP_039120656.1	1331	34/84(40.48)	1.84e-20	hypothetical protein [Flavobacterium sp. JRM]	None	Unclassified
MDJ-1_gene_443	160	RLJ22758.1	810	72/145(49.66)	1.93e-40	hypothetical protein DJ031_00290, partial [bacterium endosymbiont of Escarpia	1221601	d__Bacteria;k__norank;p__norank;c__norank;o__norank;f__norank;g__norank;s__bacterium_endosymbiont_of_Escarpia_laminata
MDJ-1_gene_447	182	AGJ98729.1	182	181/182(99.45)	3.86e-127	Putative methyltransferase [Wolbachia endosymbiont of Drosophila simulans wNo]	1236908	d__Bacteria;k__norank;p__Proteobacteria;c__Alphaproteobacteria;o__Rickettsiales;f__Anaplasmataceae;g__Wolbachia;s__Wolbachia_endosymbiont_of_Drosophila_simulans
MDJ-1_gene_448	105	WP_068652429.1	121	100/105(95.24)	5.61e-57	hypothetical protein [Wolbachia endosymbiont of Trichogramma pretiosum]	125593	d__Bacteria;k__norank;p__Proteobacteria;c__Alphaproteobacteria;o__Rickettsiales;f__Anaplasmataceae;g__Wolbachia;s__Wolbachia_endosymbiont_of_Trichogramma_pretiosum

MDJ-1_gene_450	256	WP_127087886.1	238	87/244(35.66)	8.64e-34	type IV secretion system protein VirB5 [Pectobacterium	1905730	d__Bacteria;k__norank;p__Proteobacteria;c__Gammaproteobacteria;o__Enterobacterales;f__Pectobacteriaceae;g__Pectobacterium;s__Pectobacterium_parmentieri
MDJ-1_gene_459	70	PBQ29185.1	101	45/45(100.00)	3.28e-22	hypothetical protein BTO27_01025 [Wolbachia	2049495	d__Bacteria;k__norank;p__Proteobacteria;c__Alphaproteobacteria;o__Rickettsiales;f__Anaplasmataceae;g__Wolbachia;s__Wolbachia_pipientis
MDJ-1_gene_467	176	RKJ14628.1	193	70/148(47.30)	1.33e-41	RNA-directed DNA polymerase, partial [Ruminococcus sp.	2320089	d__Bacteria;k__norank;p__Firmicutes;c__Clostridia;o__Clostridiales;f__Ruminococcaceae;g__Ruminococcus;s__Ruminococcus_sp._1xD21-23
MDJ-1_gene_474	165	OJH31409.1	162	158/165(95.76)	5.25e-107	RNA polymerase sigma factor [Wolbachia endosymbiont of Armadillidium vulgare]	77039	d__Bacteria;k__norank;p__Proteobacteria;c__Alphaproteobacteria;o__Rickettsiales;f__Anaplasmataceae;g__Wolbachia;s__Wolbachia_endosymbiont_of_Armadillidium_vulgare
MDJ-1_gene_477	394	WP_108784438.1	392	336/394(85.28)	1.36e-209	hypothetical protein [Wolbachia endosymbiont of Bemisia tabaci]	215173	d__Bacteria;k__norank;p__Proteobacteria;c__Alphaproteobacteria;o__Rickettsiales;f__Anaplasmataceae;g__Wolbachia;s__Wolbachia_endosymbiont_of_Bemisia_tabaci
MDJ-1_gene_484	101	AGJ99125.1	101	91/101(90.10)	5.51e-61	hypothetical protein wNo_07270 [Wolbachia endosymbiont of Drosophila simulans wNo]	1236908	d__Bacteria;k__norank;p__Proteobacteria;c__Alphaproteobacteria;o__Rickettsiales;f__Anaplasmataceae;g__Wolbachia;s__Wolbachia_endosymbiont_of_Drosophila_simulans
MDJ-1_gene_485	162	WP_019236396.1	162	156/162(96.30)	6.81e-100	tail protein [Wolbachia pipientis]	955	d__Bacteria;k__norank;p__Proteobacteria;c__Alphaproteobacteria;o__Rickettsiales;f__Anaplasmataceae;g__Wolbachia;s__Wolbachia_pipientis
MDJ-1_gene_486	111	WP_065095515.1	160	96/99(96.97)	1.59e-59	hypothetical protein [Wolbachia endosymbiont of Nomada leucophthalma]	1854758	d__Bacteria;k__norank;p__Proteobacteria;c__Alphaproteobacteria;o__Rickettsiales;f__Anaplasmataceae;g__Wolbachia;s__Wolbachia_endosymbiont_of_Nomada_leucophthalma
MDJ-1_gene_509	224	PBQ26861.1	224	216/224(96.43)	2.29e-142	ABC transporter ATP-binding protein [Wolbachia pipientis	2049495	d__Bacteria;k__norank;p__Proteobacteria;c__Alphaproteobacteria;o__Rickettsiales;f__Anaplasmataceae;g__Wolbachia;s__Wolbachia_pipientis
MDJ-1_gene_513	165	RKP14564.1	565	44/118(37.29)	1.87e-15	pyridoxal phosphate-dependent transferase [Piptocephalis cylindrospora]	1907219	d__Eukaryota;k__Fungi;p__Zoopagomycota;c__Zoopagomycetes;o__Zoopagales;f__Piptocephalidaceae;g__Piptocephalis;s__Piptocephalis_cylindrospora
MDJ-1_gene_514	69	SUY63870.1	98	34/57(59.65)	1.09e-12	3-methyladenine DNA glycosylase [Clostridium	1509	d__Bacteria;k__norank;p__Firmicutes;c__Clostridia;o__Clostridiales;f__Clostridiaceae;g__Clostridium;s__Clostridium_sporogenes
MDJ-1_gene_519	175	WP_063630701.1	186	150/176(85.23)	5.41e-92	hypothetical protein [Wolbachia endosymbiont of Laodelphax striatella]	368602	d__Bacteria;k__norank;p__Proteobacteria;c__Alphaproteobacteria;o__Rickettsiales;f__Anaplasmataceae;g__Wolbachia;s__Wolbachia_endosymbiont_of_Laodelphax_striatellus
MDJ-1_gene_521	92	WP_083200205.1	92	81/92(88.04)	3.79e-48	MULTISPECIES: hypothetical protein [unclassified Rickettsia]	114295	d__Bacteria;k__norank;p__Proteobacteria;c__Alphaproteobacteria;o__Rickettsiales;f__Rickettsiaceae;g__Rickettsia;s__norank
MDJ-1_gene_522	144	WP_068042252.1	144	131/144(90.97)	5.23e-93	MULTISPECIES: hypothetical protein [unclassified Rickettsia]	114295	d__Bacteria;k__norank;p__Proteobacteria;c__Alphaproteobacteria;o__Rickettsiales;f__Rickettsiaceae;g__Rickettsia;s__norank
MDJ-1_gene_527	104	PBQ29257.1	104	104/104(100.00)	2.58e-65	30S ribosomal protein S16 [Wolbachia pipientis wAus]	2049495	d__Bacteria;k__norank;p__Proteobacteria;c__Alphaproteobacteria;o__Rickettsiales;f__Anaplasmataceae;g__Wolbachia;s__Wolbachia_pipientis
MDJ-1_gene_533	209	OJH31193.1	211	159/212(75.00)	2.91e-106	hypothetical protein Wxf_00574 [Wolbachia endosymbiont of Armadillidium vulgare]	77039	d__Bacteria;k__norank;p__Proteobacteria;c__Alphaproteobacteria;o__Rickettsiales;f__Anaplasmataceae;g__Wolbachia;s__Wolbachia_endosymbiont_of_Armadillidium_vulgare
MDJ-1_gene_542	90	WP_041079171.1	660	64/89(71.91)	3.80e-30	hypothetical protein [Rickettsia asembonensis]	1068590	d__Bacteria;k__norank;p__Proteobacteria;c__Alphaproteobacteria;o__Rickettsiales;f__Rickettsiaceae;g__Rickettsia;s__Rickettsia_asembonensis
MDJ-1_gene_545	264	WP_010401420.1	264	260/264(98.48)	6.18e-185	4-hydroxy-tetrahydrodipicolinate reductase [Wolbachia endosymbiont of Nasonia	None	Unclassified
MDJ-1_gene_550	236	WP_064085252.1	236	232/236(98.31)	2.33e-166	hypothetical protein [Wolbachia endosymbiont of Dactylopius coccus]	None	Unclassified
MDJ-1_gene_555	197	WP_010403811.1	197	195/197(98.98)	7.36e-137	dTMP kinase [Wolbachia endosymbiont of Nasonia vitripennis]	180837	d__Bacteria;k__norank;p__Proteobacteria;c__Alphaproteobacteria;o__Rickettsiales;f__Anaplasmataceae;g__Wolbachia;s__Wolbachia_endosymbiont_of_Nasonia_vitripennis

MDJ-1_gene_562	170	CCE77441.1	57	41/50(82.00)	9.03e-19	hypothetical protein (ankyrin repeat domain) [Wolbachia pipientis wAlbB]	1116230	d__Bacteria;k__norank;p__Proteobacteria;c__Alphaproteobacteria;o__Rickettsiales;f__Anaplasmataceae;g__Wolbachia;s__Wolbachia_pipientis
MDJ-1_gene_566	162	PBQ25023.1	293	123/135(91.11)	8.04e-74	hypothetical protein BTO27_05620 [Wolbachia	2049495	d__Bacteria;k__norank;p__Proteobacteria;c__Alphaproteobacteria;o__Rickettsiales;f__Anaplasmataceae;g__Wolbachia;s__Wolbachia_pipientis
MDJ-1_gene_567	119	PBQ28191.1	119	115/119(96.64)	3.42e-68	hypothetical protein BTO27_02075 [Wolbachia	2049495	d__Bacteria;k__norank;p__Proteobacteria;c__Alphaproteobacteria;o__Rickettsiales;f__Anaplasmataceae;g__Wolbachia;s__Wolbachia_pipientis
MDJ-1_gene_569	65	EAL58933.1	34	28/30(93.33)	1.70e-12	hypothetical protein WwAna1347 [Wolbachia endosymbiont of Drosophila ananassae]	307502	d__Bacteria;k__norank;p__Proteobacteria;c__Alphaproteobacteria;o__Rickettsiales;f__Anaplasmataceae;g__Wolbachia;s__Wolbachia_endosymbiont_of_Drosophila_ananassae
MDJ-1_gene_579	181	PBQ28100.1	181	180/181(99.45)	2.94e-126	50S ribosomal protein L6 [Wolbachia pipientis wAus]	2049495	d__Bacteria;k__norank;p__Proteobacteria;c__Alphaproteobacteria;o__Rickettsiales;f__Anaplasmataceae;g__Wolbachia;s__Wolbachia_pipientis
MDJ-1_gene_580	123	PBQ28101.1	123	122/123(99.19)	9.30e-77	50S ribosomal protein L18 [Wolbachia pipientis wAus]	2049495	d__Bacteria;k__norank;p__Proteobacteria;c__Alphaproteobacteria;o__Rickettsiales;f__Anaplasmataceae;g__Wolbachia;s__Wolbachia_pipientis
MDJ-1_gene_583	203	WP_039833846.1	203	194/201(96.52)	1.48e-139	SURF1 family protein [Wolbachia endosymbiont of Nasonia vitripennis]	180837	d__Bacteria;k__norank;p__Proteobacteria;c__Alphaproteobacteria;o__Rickettsiales;f__Anaplasmataceae;g__Wolbachia;s__Wolbachia_endosymbiont_of_Nasonia_vitripennis
MDJ-1_gene_587	230	B3CMC0.1	355	222/228(97.37)	4.26e-147	RecName: Full=Protein RecA; AltName: Full=Recombinase A	570417	d__Bacteria;k__norank;p__Proteobacteria;c__Alphaproteobacteria;o__Rickettsiales;f__Anaplasmataceae;g__Wolbachia;s__Wolbachia_endosymbiont_of_Culex_quinquefasciatus
MDJ-1_gene_590	416	POG50308.1	410	376/407(92.38)	4.88e-273	IS256 family transposase [Wolbachia sp. wMel_AMD]	1912095	d__Bacteria;k__norank;p__Proteobacteria;c__Alphaproteobacteria;o__Rickettsiales;f__Anaplasmataceae;g__Wolbachia;s__Wolbachia_sp._wMel_AMD
MDJ-1_gene_601	79	OCA05934.1	79	77/79(97.47)	7.41e-49	hypothetical protein wTpre_252 [Wolbachia endosymbiont of Trichogramma pretiosum]	125593	d__Bacteria;k__norank;p__Proteobacteria;c__Alphaproteobacteria;o__Rickettsiales;f__Anaplasmataceae;g__Wolbachia;s__Wolbachia_endosymbiont_of_Trichogramma_pretiosum
MDJ-1_gene_604	169	WP_114517339.1	169	165/169(97.63)	1.60e-115	sigma-70 family RNA polymerase sigma factor [Wolbachia endosymbiont of Cylisticus convexus]	118728	d__Bacteria;k__norank;p__Proteobacteria;c__Alphaproteobacteria;o__Rickettsiales;f__Anaplasmataceae;g__Wolbachia;s__Wolbachia_endosymbiont_of_Cylisticus_convexus
MDJ-1_gene_607	112	ESK97688.1	622	46/91(50.55)	9.86e-20	domain-containing histone demethylation protein 1 [Moniliophthora roreri MCA	1381753	d__Eukaryota;k__Fungi;p__Basidiomycota;c__Agaricomycetes;o__Agaricales;f__Marasmiaceae;g__Moniliophthora;s__Moniliophthora_roreri
MDJ-1_gene_617	141	RKP24770.1	351	68/131(51.91)	2.76e-41	putative phenylalanine hydroxylase [Syncephalis	1712513	d__Eukaryota;k__Fungi;p__Zoopagomycota;c__Zoopagomycetes;o__Zoopagales;f__Piptocephalidaceae;g__Syncephalis;s__Syncephalis_pseudoplumigaleata
MDJ-1_gene_619	166	AGJ98713.1	163	116/156(74.36)	3.99e-61	Putative membrane protein [Wolbachia endosymbiont of Drosophila simulans wNo]	1236908	d__Bacteria;k__norank;p__Proteobacteria;c__Alphaproteobacteria;o__Rickettsiales;f__Anaplasmataceae;g__Wolbachia;s__Wolbachia_endosymbiont_of_Drosophila_simulans
MDJ-1_gene_621	118	PGH37161.1	162	53/110(48.18)	1.39e-26	hypothetical protein CRN43_22805 [Candidatus	2044729	d__Bacteria;k__norank;p__Bacteroidetes;c__Cytophagia;o__Cytophagales;f__Cytophagaceae;g__Candidatus_Nephrothrix;s__Candidatus_Nephrothrix_sp._EaCA
MDJ-1_gene_629	58	OSY19256.1	647	36/51(70.59)	2.62e-14	hypothetical protein B0D75_01370 [Entomoplasmatales bacterium	None	Unclassified
MDJ-1_gene_636	90	XP_016609030.1	605	34/71(47.89)	1.25e-10	hypothetical protein SPPG_04087 [Spizellomyces punctatus DAOM BR117]	645134	d__Eukaryota;k__Fungi;p__Chytridiomycota;c__Chytridiomycetes;o__Spizellomycetales;f__Spizellomycetaceae;g__Spizellomyces;s__Spizellomyces_punctatus
MDJ-1_gene_640	441	AVJ51765.1	885	374/441(84.81)	3.23e-279	structural protein containing EPS-depolymerase domain [Pantoea phage vB_PagS_Vid5]	2099652	d__Viruses;k__norank;p__Uroviricota;c__Caudoviricetes;o__Caudovirales;f__Siphoviridae;g__Vidquintavirus;s__Pantoea_virus_Vid5

MDJ-1_gene_648	299	WP_064085537.1	289	267/298(89.60)	2.46e-181	Rpn family recombination-promoting nuclease/putative transposase [Wolbachia endosymbiont of Dactylopius	None	Unclassified
MDJ-1_gene_654	240	RKP07829.1	398	76/223(34.08)	6.26e-32	hypothetical protein THASP1DRAFT_16510, partial [Thamnocephalis sphaerospora]	78915	d__Eukaryota;k__Fungi;p__Zoopagomycota;c__Zoopagomycetes;o__Zoopagales;f__Sigmoideomycetaceae;g__Thamnocephalis;s__Thamnocephalis_sphaerospora
MDJ-1_gene_672	414	BBA85188.1	462	286/414(69.08)	2.46e-187	replicative DNA helicase [endosymbiont of Sipalinus gigas]	1972134	d__Bacteria;k__norank;p__Proteobacteria;c__Gammaproteobacteria;o__norank;f__norank;g__Candidatus_Nardonella;s__endosymbiont_of_Sipalinus_gigas
MDJ-1_gene_680	181	AAB00457.1	304	58/152(38.16)	6.29e-22	beta-hydroxylase [Streptomyces verticillus]	29309	d__Bacteria;k__norank;p__Actinobacteria;c__Actinobacteria;o__Streptomycetales;f__Streptomycetaceae;g__Streptomyces;s__Streptomyces_verticillus
MDJ-1_gene_687	246	WP_063630556.1	246	242/246(98.37)	9.45e-171	DsbA family protein [Wolbachia endosymbiont of Laodelphax striatella]	368602	d__Bacteria;k__norank;p__Proteobacteria;c__Alphaproteobacteria;o__Rickettsiales;f__Anaplasmataceae;g__Wolbachia;s__Wolbachia_endosymbiont_of_Laodelphax_striatellus
MDJ-1_gene_694	226	WP_127464142.1	226	222/226(98.23)	1.07e-157	thiamine diphosphokinase [Wolbachia endosymbiont of Bemisia tabaci]	215173	d__Bacteria;k__norank;p__Proteobacteria;c__Alphaproteobacteria;o__Rickettsiales;f__Anaplasmataceae;g__Wolbachia;s__Wolbachia_endosymbiont_of_Bemisia_tabaci
MDJ-1_gene_708	64	RKU40650.1	493	30/65(46.15)	1.67e-08	hypothetical protein DL546_000537 [Coniochaeta	177199	d__Eukaryota;k__Fungi;p__Ascomycota;c__Sordariomycetes;o__Coniochaetales;f__Coniochaetaceae;g__Coniochaeta;s__Coniochaeta_pulveracea
MDJ-1_gene_712	127	WP_010402262.1	124	122/127(96.06)	5.84e-79	hypothetical protein [Wolbachia endosymbiont of Nasonia vitripennis]	180837	d__Bacteria;k__norank;p__Proteobacteria;c__Alphaproteobacteria;o__Rickettsiales;f__Anaplasmataceae;g__Wolbachia;s__Wolbachia_endosymbiont_of_Nasonia_vitripennis
MDJ-1_gene_719	78	ORX89024.1	171	37/73(50.68)	4.45e-23	OV-16 antigen, variant 2 [Basidiobolus meristosporus CBS 931.73]	1314790	d__Eukaryota;k__Fungi;p__Zoopagomycota;c__Basidiobolomycetes;o__Basidiobolales;f__Basidiobolaceae;g__Basidiobolus;s__Basidiobolus_meristosporus
MDJ-1_gene_735	349	BBA84810.1	346	274/348(78.74)	2.83e-186	rod shape-determining protein MreB [endosymbiont of Pachyrhynchus infernalis]	1971488	d__Bacteria;k__norank;p__Proteobacteria;c__Gammaproteobacteria;o__norank;f__norank;g__Candidatus_Nardonella;s__endosymbiont_of_Pachyrhynchus_infernalis
MDJ-1_gene_745	47	RYE13645.1	39	29/36(80.56)	3.59e-12	conjugal transfer protein TrbI, partial [Sphingobacteriaceae	2021370	d__Bacteria;k__norank;p__Bacteroidetes;c__Sphingobacteriia;o__Sphingobacteriales;f__Sphingobacteriaceae;g__norank;s__Sphingobacteriaceae_bacterium
MDJ-1_gene_746	352	RYE13646.1	338	191/336(56.85)	5.50e-133	P-type DNA transfer ATPase VirB11, partial [Sphingobacteriaceae bacterium]	2021370	d__Bacteria;k__norank;p__Bacteroidetes;c__Sphingobacteriia;o__Sphingobacteriales;f__Sphingobacteriaceae;g__norank;s__Sphingobacteriaceae_bacterium
MDJ-1_gene_747	92	WP_118714888.1	132	30/52(57.69)	1.47e-09	fibronectin type III domain-containing protein [Odoribacter sp. AF15-53]	2292236	d__Bacteria;k__norank;p__Bacteroidetes;c__Bacteroidia;o__Bacteroidales;f__Odoribacteraceae;g__Odoribacter;s__Odoribacter_sp._AF15-53
MDJ-1_gene_751	90	XP_018276748.1	557	40/74(54.05)	1.87e-16	putative ER-associated protein catabolism-related protein [Cutaneotrichosporon	879819	d__Eukaryota;k__Fungi;p__Basidiomycota;c__Tremellomycetes;o__Trichosporonales;f__Trichosporonaceae;g__Cutaneotrichosporon;s__Cutaneotrichosporon_oleaginosum
MDJ-1_gene_755	389	WP_010422352.1	344	162/344(47.09)	6.41e-100	IS4 family transposase [Rickettsia helvetica]	35789	d__Bacteria;k__norank;p__Proteobacteria;c__Alphaproteobacteria;o__Rickettsiales;f__Rickettsiaceae;g__Rickettsia;s__Rickettsia_helvetica
MDJ-1_gene_773	260	WP_064125329.1	260	251/260(96.54)	5.84e-172	30S ribosomal protein S6 [Wolbachia endosymbiont of Dactylopius coccus]	955	d__Bacteria;k__norank;p__Proteobacteria;c__Alphaproteobacteria;o__Rickettsiales;f__Anaplasmataceae;g__Wolbachia;s__Wolbachia_pipientis
MDJ-1_gene_789	64	CCE77694.1	64	64/64(100.00)	4.20e-37	Phosphatidylglycerophosphatase A (fragment) [Wolbachia pipientis wAlbB]	1116230	d__Bacteria;k__norank;p__Proteobacteria;c__Alphaproteobacteria;o__Rickettsiales;f__Anaplasmataceae;g__Wolbachia;s__Wolbachia_pipientis
MDJ-1_gene_793	83	BAH22243.1	83	81/83(97.59)	4.70e-47	hypothetical protein [Wolbachia endosymbiont of Cadra cautella]	190193	d__Bacteria;k__norank;p__Proteobacteria;c__Alphaproteobacteria;o__Rickettsiales;f__Anaplasmataceae;g__Wolbachia;s__Wolbachia_endosymbiont_of_Cadra_cautella

MDJ-1_gene_794	166	AGJ99393.1	166	162/166(97.59)	1.95e-110	contractile tail tube protein [Wolbachia endosymbiont of Drosophila simulans wNo]	1236908	d__Bacteria;k__norank;p__Proteobacteria;c__Alphaproteobacteria;o__Rickettsiales;f__Anaplasmataceae;g__Wolbachia;s__Wolbachia_endosymbiont_of_Drosophila_simulans
MDJ-1_gene_813	191	WP_029237657.1	191	180/191(94.24)	8.46e-115	glycerol-3-phosphate acyltransferase [Wolbachia endosymbiont of Nasonia	2640676	d__Bacteria;k__norank;p__Proteobacteria;c__Alphaproteobacteria;o__Rickettsiales;f__Anaplasmataceae;g__Wolbachia;s__norank
MDJ-1_gene_815	203	WP_010401580.1	376	202/203(99.51)	7.36e-129	cation:dicarboxylase symporter family transporter [Wolbachia endosymbiont of Nasonia vitripennis]	180837	d__Bacteria;k__norank;p__Proteobacteria;c__Alphaproteobacteria;o__Rickettsiales;f__Anaplasmataceae;g__Wolbachia;s__Wolbachia_endosymbiont_of_Nasonia_vitripennis
MDJ-1_gene_821	281	ETW98760.1	656	129/282(45.74)	1.75e-71	hypothetical protein ETSY2_42270 [Candidatus	1429439	d__Bacteria;k__norank;p__Candidatus_Tectomicrobia;c__norank;o__norank;f__norank;g__Candidatus_Entotheonella;s__Candidatus_Entotheonella_gemina
MDJ-1_gene_827	160	WP_065094330.1	165	131/160(81.88)	1.06e-78	MULTISPECIES: phage tail protein [Wolbachia]	2640676	d__Bacteria;k__norank;p__Proteobacteria;c__Alphaproteobacteria;o__Rickettsiales;f__Anaplasmataceae;g__Wolbachia;s__norank
MDJ-1_gene_829	397	XP_016610584.1	507	151/415(36.39)	1.16e-70	protoporphyrinogen oxidase [Spizellomyces punctatus DAOM BR117]	645134	d__Eukaryota;k__Fungi;p__Chytridiomycota;c__Chytridiomycetes;o__Spizellomycetales;f__Spizellomycetaceae;g__Spizellomyces;s__Spizellomyces_punctatus
MDJ-1_gene_832	222	WP_019078568.1	614	152/222(68.47)	3.45e-76	MULTISPECIES: AAA family ATPase [Wolbachia]	2640676	d__Bacteria;k__norank;p__Proteobacteria;c__Alphaproteobacteria;o__Rickettsiales;f__Anaplasmataceae;g__Wolbachia;s__norank
MDJ-1_gene_841	132	AZU37357.1	134	132/132(100.00)	6.28e-91	Co2+/Mg2+ efflux protein ApaG [Wolbachia endosymbiont of Bemisia tabaci]	215173	d__Bacteria;k__norank;p__Proteobacteria;c__Alphaproteobacteria;o__Rickettsiales;f__Anaplasmataceae;g__Wolbachia;s__Wolbachia_endosymbiont_of_Bemisia_tabaci
MDJ-1_gene_850	168	CCE77906.1	438	153/168(91.07)	1.29e-101	conserved hypothetical protein [Wolbachia pipientis wAlbB]	1116230	d__Bacteria;k__norank;p__Proteobacteria;c__Alphaproteobacteria;o__Rickettsiales;f__Anaplasmataceae;g__Wolbachia;s__Wolbachia_pipientis
MDJ-1_gene_858	150	WP_010406829.1	146	142/144(98.61)	2.92e-92	MULTISPECIES: 50S ribosomal protein L11 [Wolbachia]	953	d__Bacteria;k__norank;p__Proteobacteria;c__Alphaproteobacteria;o__Rickettsiales;f__Anaplasmataceae;g__Wolbachia;s__norank
MDJ-1_gene_875	173	PCG11693.1	173	173/173(100.00)	4.91e-112	Na+/H+ antiporter subunit E [Wolbachia pipientis]	None	Unclassified
MDJ-1_gene_876	138	PBQ29240.1	138	138/138(100.00)	2.74e-92	hypothetical protein BTO27_00615 [Wolbachia	2049495	d__Bacteria;k__norank;p__Proteobacteria;c__Alphaproteobacteria;o__Rickettsiales;f__Anaplasmataceae;g__Wolbachia;s__Wolbachia_pipientis
MDJ-1_gene_904	163	ONI57208.1	163	150/163(92.02)	3.25e-103	hypothetical protein N499_1126 [Wolbachia pipientis wVitA]	1379786	d__Bacteria;k__norank;p__Proteobacteria;c__Alphaproteobacteria;o__Rickettsiales;f__Anaplasmataceae;g__Wolbachia;s__Wolbachia_pipientis
MDJ-1_gene_905	70	OJH31496.1	66	52/66(78.79)	5.31e-33	hypothetical protein Wxf_00887 [Wolbachia endosymbiont of Armadillidium vulgare]	77039	d__Bacteria;k__norank;p__Proteobacteria;c__Alphaproteobacteria;o__Rickettsiales;f__Anaplasmataceae;g__Wolbachia;s__Wolbachia_endosymbiont_of_Armadillidium_vulgare
MDJ-1_gene_921	132	SKA13374.1	261	49/125(39.20)	6.86e-12	Methyltransferase domain-containing protein [Legionella maceachernii]	466	d__Bacteria;k__norank;p__Proteobacteria;c__Gammaproteobacteria;o__Legionellales;f__Legionellaceae;g__Tatlockia;s__Legionella_maceachernii
MDJ-1_gene_922	111	XP_027620157.1	824	39/95(41.05)	4.32e-16	hypothetical protein SCP_1502520 [Sparassis crispa]	139825	d__Eukaryota;k__Fungi;p__Basidiomycota;c__Agaricomycetes;o__Polyporales;f__Sparassidaceae;g__Sparassis;s__Sparassis_crispa
MDJ-1_gene_924	97	GAM83190.1	812	36/86(41.86)	7.89e-10	hypothetical protein ANO11243_011760 [fungal sp. No.11243]	1603295	d__Eukaryota;k__Fungi;p__norank;c__norank;o__norank;f__norank;g__norank;s__fungal_sp._No.11243
MDJ-1_gene_935	73	WP_127937584.1	1420	33/71(46.48)	3.40e-08	G-D-S-L family lipolytic protein [Nonomuraea polychroma]	46176	d__Bacteria;k__norank;p__Actinobacteria;c__Actinobacteria;o__Streptosporangiales;f__Streptosporangiaceae;g__Nonomuraea;s__Nonomuraea_polychroma
MDJ-1_gene_943	73	OOQ55351.1	97	20/37(54.05)	4.93e-07	hypothetical protein AFM16_04955 [Streptomyces	1890	d__Bacteria;k__norank;p__Actinobacteria;c__Actinobacteria;o__Streptomycetales;f__Streptomycetaceae;g__Streptomyces;s__Streptomyces_antibioticus
MDJ-1_gene_949	363	WP_077190443.1	363	320/363(88.15)	1.77e-236	phage tail protein, partial [Wolbachia pipientis]	955	d__Bacteria;k__norank;p__Proteobacteria;c__Alphaproteobacteria;o__Rickettsiales;f__Anaplasmataceae;g__Wolbachia;s__Wolbachia_pipientis

MDJ-1_gene_954	90	WP_010403478.1	90	89/90(98.89)	6.35e-55	30S ribosomal protein S15 [Wolbachia endosymbiont of Nasonia vitripennis]	180837	d__Bacteria;k__norank;p__Proteobacteria;c__Alphaproteobacteria;o__Rickettsiales;f__Anaplasmataceae;g__Wolbachia;s__Wolbachia_endosymbiont_of_Nasonia_vitripennis
MDJ-1_gene_983	119	PCG11878.1	119	119/119(100.00)	1.57e-73	50S ribosomal protein L14 [Wolbachia pipientis]	None	Unclassified
MDJ-1_gene_984	105	PBQ28096.1	105	103/105(98.10)	2.29e-64	50S ribosomal protein L24 [Wolbachia pipientis wAus]	2049495	d__Bacteria;k__norank;p__Proteobacteria;c__Alphaproteobacteria;o__Rickettsiales;f__Anaplasmataceae;g__Wolbachia;s__Wolbachia_pipientis
MDJ-1_gene_1021	105	PCG11740.1	159	104/105(99.05)	1.83e-70	disulfide bond formation protein B [Wolbachia pipientis]	None	Unclassified
MDJ-1_gene_1043	99	WP_068652145.1	99	97/99(97.98)	4.39e-66	oxidoreductase [Wolbachia endosymbiont of Trichogramma pretiosum]	125593	d__Bacteria;k__norank;p__Proteobacteria;c__Alphaproteobacteria;o__Rickettsiales;f__Anaplasmataceae;g__Wolbachia;s__Wolbachia_endosymbiont_of_Trichogramma_pretiosum
MDJ-1_gene_1054	171	WP_108783927.1	326	152/156(97.44)	2.65e-99	hypothetical protein [Wolbachia endosymbiont of Bemisia tabaci]	215173	d__Bacteria;k__norank;p__Proteobacteria;c__Alphaproteobacteria;o__Rickettsiales;f__Anaplasmataceae;g__Wolbachia;s__Wolbachia_endosymbiont_of_Bemisia_tabaci
MDJ-1_gene_1055	110	WP_029237629.1	321	106/108(98.15)	2.80e-70	hypothetical protein [Wolbachia endosymbiont of Nasonia vitripennis]	180837	d__Bacteria;k__norank;p__Proteobacteria;c__Alphaproteobacteria;o__Rickettsiales;f__Anaplasmataceae;g__Wolbachia;s__Wolbachia_endosymbiont_of_Nasonia_vitripennis
MDJ-1_gene_1076	153	GBB90963.1	602	53/142(37.32)	1.05e-18	hypothetical protein RclHR1_18020001 [Rhizophagus clarus]	94130	d__Eukaryota;k__Fungi;p__Mucoromycota;c__Glomeromycetes;o__Glomerales;f__Glomeraceae;g__Rhizophagus;s__Rhizophagus_clarus
MDJ-1_gene_1080	133	OSY19256.1	647	54/129(41.86)	1.97e-16	hypothetical protein B0D75_01370 [Entomoplasmatales bacterium	None	Unclassified
MDJ-1_gene_1087	90	ARC83301.1	425	49/77(63.64)	1.56e-25	serine--tRNA ligase [Clostridium argentinense]	29341	d__Bacteria;k__norank;p__Firmicutes;c__Clostridia;o__Clostridiales;f__Clostridiaceae;g__Clostridium;s__Clostridium_argentinense
MDJ-1_gene_1097	87	ETE43231.1	289	61/82(74.39)	4.67e-35	hypothetical protein M574_21520, partial [Salmonella enterica subsp. enterica serovar	1347789	d__Bacteria;k__norank;p__Proteobacteria;c__Gammaproteobacteria;o__Enterobacterales;f__Enterobacteriaceae;g__Salmonella;s__Salmonella_enterica
MDJ-1_gene_1147	112	WP_041063190.1	421	74/112(66.07)	5.94e-39	UDP-N-acetylglucosamine 1-carboxyvinyltransferase [Candidatus Tachikawaea	1410383	d__Bacteria;k__norank;p__Proteobacteria;c__Gammaproteobacteria;o__Enterobacterales;f__Enterobacteriaceae;g__Candidatus_Tachikawaea;s__Candidatus_Tachikawaea_gelatinosa
MDJ-1_gene_1154	101	BBA84926.1	816	77/98(78.57)	4.44e-47	leucine--tRNA ligase [endosymbiont of Pachyrhynchus	1971488	d__Bacteria;k__norank;p__Proteobacteria;c__Gammaproteobacteria;o__norank;f__norank;g__Candidatus_Nardonella;s__endosymbiont_of_Pachyrhynchus_infernalis
MDJ-1_gene_1167	202	WP_096617525.1	295	105/202(51.98)	6.86e-50	hypothetical protein [Wolbachia pipientis]	955	d__Bacteria;k__norank;p__Proteobacteria;c__Alphaproteobacteria;o__Rickettsiales;f__Anaplasmataceae;g__Wolbachia;s__Wolbachia_pipientis
MDJ-1_gene_1182	75	WP_090028788.1	255	30/52(57.69)	8.40e-07	ion transporter [Lutimaribacter saemankumensis]	490829	d__Bacteria;k__norank;p__Proteobacteria;c__Alphaproteobacteria;o__Rhodobacterales;f__Rhodobacteraceae;g__Lutimaribacter;s__Lutimaribacter_saemankumensis
MDJ-1_gene_1191	157	PBQ29246.1	175	157/157(100.00)	7.23e-108	nuclease [Wolbachia pipientis wAus]	2049495	d__Bacteria;k__norank;p__Proteobacteria;c__Alphaproteobacteria;o__Rickettsiales;f__Anaplasmataceae;g__Wolbachia;s__Wolbachia_pipientis
MDJ-1_gene_1196	155	WP_064085976.1	155	155/155(100.00)	6.45e-106	Holliday junction resolvase RuvX [Wolbachia endosymbiont of Dactylopius coccus]	None	Unclassified
MDJ-1_gene_1215	240	OUB07123.1	301	83/243(34.16)	8.27e-39	hypothetical protein BK709_13615 [Bacillus thuringiensis serovar	180886	d__Bacteria;k__norank;p__Firmicutes;c__Bacilli;o__Bacillales;f__Bacillaceae;g__Bacillus;s__Bacillus_thuringiensis
MDJ-1_gene_1225	125	RYG68038.1	307	39/126(30.95)	5.17e-09	hypothetical protein EON64_06080 [archaeon]	1906665	d__Archaea;k__norank;p__norank;c__norank;o__norank;f__norank;g__norank;s__archaeon
MDJ-1_gene_1232	333	WP_109990923.1	357	293/333(87.99)	3.41e-227	hypothetical protein [Lactococcus lactis]	1358	d__Bacteria;k__norank;p__Firmicutes;c__Bacilli;o__Lactobacillales;f__Streptococcaceae;g__Lactococcus;s__Lactococcus_lactis

MDJ-1_gene_1237	44	BBA84631.1	514	34/44(77.27)	1.03e-14	putative lipid II flippase MurJ [endosymbiont of Euscepes postfasciatus]	650377	d__Bacteria;k__norank;p__Proteobacteria;c__Gammaproteobacteria;o__norank;f__norank;g__norank;s__endosymbiont_of_Euscepes_postfasciatus
MDJ-1_gene_1238	120	BBA84631.1	514	83/119(69.75)	7.07e-45	putative lipid II flippase MurJ [endosymbiont of Euscepes postfasciatus]	650377	d__Bacteria;k__norank;p__Proteobacteria;c__Gammaproteobacteria;o__norank;f__norank;g__norank;s__endosymbiont_of_Euscepes_postfasciatus
MDJ-1_gene_1245	89	OJH30281.1	187	41/81(50.62)	1.72e-14	hypothetical protein Wxf_03236 [Armadillidium vulgare]	77039	d__Bacteria;k__norank;p__Proteobacteria;c__Alphaproteobacteria;o__Rickettsiales;f__Anaplasmataceae;g__Wolbachia;s__Wolbachia_endosymbiont_of_Armadillidium_vulgare
MDJ-1_gene_1253	278	WP_114517457.1	490	248/263(94.30)	1.12e-143	ankyrin repeat domain-containing protein [Wolbachia endosymbiont of Cylisticus convexus]	118728	d__Bacteria;k__norank;p__Proteobacteria;c__Alphaproteobacteria;o__Rickettsiales;f__Anaplasmataceae;g__Wolbachia;s__Wolbachia_endosymbiont_of_Cylisticus_convexus
MDJ-1_gene_1266	243	WP_109990909.1	451	217/243(89.30)	8.19e-159	PBSX family phage terminase large subunit [Lactococcus lactis]	1358	d__Bacteria;k__norank;p__Firmicutes;c__Bacilli;o__Lactobacillales;f__Streptococcaceae;g__Lactococcus;s__Lactococcus_lactis
MDJ-1_gene_1267	87	WP_082779242.1	182	69/87(79.31)	4.71e-45	hypothetical protein [Lactococcus sp. DD01]	1776443	d__Bacteria;k__norank;p__Firmicutes;c__Bacilli;o__Lactobacillales;f__Streptococcaceae;g__Lactococcus;s__Lactococcus_sp._DD01
MDJ-1_gene_1271	91	BBA85065.1	539	57/83(68.67)	1.15e-28	glutamine--tRNA ligase [endosymbiont of Rhynchophorus ferrugineus]	1972133	d__Bacteria;k__norank;p__Proteobacteria;c__Gammaproteobacteria;o__norank;f__norank;g__Candidatus_Nardonella;s__Candidatus_Nardonella_dryophthoridicola
MDJ-1_gene_1278	114	WP_110883498.1	325	30/66(45.45)	1.74e-07	SDR family NAD(P)-dependent oxidoreductase, partial [Gammaproteobacteria bacterium 2W06]	None	Unclassified
MDJ-1_gene_1280	121	WP_038197974.1	307	111/121(91.74)	4.07e-65	helix-turn-helix domain-containing protein [Wolbachia	955	d__Bacteria;k__norank;p__Proteobacteria;c__Alphaproteobacteria;o__Rickettsiales;f__Anaplasmataceae;g__Wolbachia;s__Wolbachia_pipientis
MDJ-1_gene_1286	93	WP_010403548.1	93	93/93(100.00)	1.47e-59	MULTISPECIES: hypothetical protein [Wolbachia]	180837	d__Bacteria;k__norank;p__Proteobacteria;c__Alphaproteobacteria;o__Rickettsiales;f__Anaplasmataceae;g__Wolbachia;s__Wolbachia_endosymbiont_of_Nasonia_vitripennis
MDJ-1_gene_1287	122	ONI55738.1	122	116/122(95.08)	5.62e-82	HIT domain protein [Wolbachia pipientis wUni]	1379787	d__Bacteria;k__norank;p__Proteobacteria;c__Alphaproteobacteria;o__Rickettsiales;f__Anaplasmataceae;g__Wolbachia;s__Wolbachia_pipientis
MDJ-1_gene_1292	63	AGC67887.1	508	29/60(48.33)	9.94e-13	para-nitrobenzyl esterase PnbA [Thermoclostridium stercorarium subsp. stercorarium DSM 8532]	1121335	d__Bacteria;k__norank;p__Firmicutes;c__Clostridia;o__Clostridiales;f__Hungateiclostridiaceae;g__Thermoclostridium;s__Thermoclostridium_stercorarium
MDJ-1_gene_1293	164	WP_032139675.1	258	42/68(61.76)	5.60e-18	hypothetical protein [Rickettsia tamurae]	334545	d__Bacteria;k__norank;p__Proteobacteria;c__Alphaproteobacteria;o__Rickettsiales;f__Rickettsiaceae;g__Rickettsia;s__Rickettsia_tamurae
PB1_gene_2603	69	SJZ30921.1	69	69/69(100.00)	3.28e-43	cold shock protein E (CspE) [Pantoea agglomerans]	470933	d__Bacteria;k__norank;p__Proteobacteria;c__Gammaproteobacteria;o__Enterobacterales;f__Erwiniaceae;g__Pantoea;s__Pantoea_eucalypti
PB1_gene_2606	169	SVK54143.1	234	87/168(51.79)	5.33e-40	Uncharacterised protein [Acinetobacter baumannii]	470	d__Bacteria;k__norank;p__Proteobacteria;c__Gammaproteobacteria;o__Pseudomonadales;f__Moraxellaceae;g__Acinetobacter;s__Acinetobacter_baumannii
PB1_gene_2609	72	ORM77593.1	224	44/52(84.62)	1.36e-24	hypothetical protein HA42_17590 [Pantoea deleyi]	470932	d__Bacteria;k__norank;p__Proteobacteria;c__Gammaproteobacteria;o__Enterobacterales;f__Erwiniaceae;g__Pantoea;s__Pantoea_deleyi
PB1_gene_2612	174	SJZ66327.1	174	173/174(99.43)	2.35e-124	Isopentenyldiphosphate isomerase [Pantoea agglomerans]	470933	d__Bacteria;k__norank;p__Proteobacteria;c__Gammaproteobacteria;o__Enterobacterales;f__Erwiniaceae;g__Pantoea;s__Pantoea_eucalypti
PB1_gene_2614	249	ORM85482.1	249	240/249(96.39)	1.69e-170	xylose isomerase [Pantoea deleyi]	470932	d__Bacteria;k__norank;p__Proteobacteria;c__Gammaproteobacteria;o__Enterobacterales;f__Erwiniaceae;g__Pantoea;s__Pantoea_deleyi
PB1_gene_2615	315	WP_111141068.1	321	310/315(98.41)	4.43e-221	sugar kinase [Pantoea sp. ARC607]	2027922	d__Bacteria;k__norank;p__Proteobacteria;c__Gammaproteobacteria;o__Enterobacterales;f__Erwiniaceae;g__Pantoea;s__Pantoea_sp._ARC607
PB1_gene_2618	142	WP_063881016.1	142	132/142(92.96)	2.51e-90	hypothetical protein [Pantoea sp. OXWO6B1]	None	Unclassified
PB1_gene_2620	172	ORM80384.1	170	163/169(96.45)	1.67e-118	double-stranded uracil-DNA glycosylase [Pantoea deleyi]	470932	d__Bacteria;k__norank;p__Proteobacteria;c__Gammaproteobacteria;o__Enterobacterales;f__Erwiniaceae;g__Pantoea;s__Pantoea_deleyi
PB1_gene_2625	103	ORM85057.1	113	99/103(96.12)	7.21e-60	hypothetical protein HA42_03280 [Pantoea deleyi]	470932	d__Bacteria;k__norank;p__Proteobacteria;c__Gammaproteobacteria;o__Enterobacterales;f__Erwiniaceae;g__Pantoea;s__Pantoea_deleyi

PB1_gene_2626	127	ORM85058.1	127	126/127(99.21)	3.98e-64	hypothetical protein HA42_03285 [Pantoea deleyi]	470932	d__Bacteria;k__norank;p__Proteobacteria;c__Gammaproteobacteria;o__Enterobacterales;f__Erwiniaceae;g__Pantoea;s__Pantoea_deleyi
PB1_gene_2630	521	OQV42781.1	521	513/521(98.46)	0	TyrR family transcriptional regulator [Pantoea vagans]	470934	d__Bacteria;k__norank;p__Proteobacteria;c__Gammaproteobacteria;o__Enterobacterales;f__Erwiniaceae;g__Pantoea;s__Pantoea_vagans
PB1_gene_2632	281	ORM82917.1	281	278/281(98.93)	6.96e-195	ABC transporter permease [Pantoea deleyi]	470932	d__Bacteria;k__norank;p__Proteobacteria;c__Gammaproteobacteria;o__Enterobacterales;f__Erwiniaceae;g__Pantoea;s__Pantoea_deleyi
PB1_gene_2633	266	ORM82916.1	261	259/261(99.23)	3.42e-175	ABC transporter permease [Pantoea deleyi]	470932	d__Bacteria;k__norank;p__Proteobacteria;c__Gammaproteobacteria;o__Enterobacterales;f__Erwiniaceae;g__Pantoea;s__Pantoea_deleyi
PB1_gene_2636	232	WP_088900990.1	232	176/232(75.86)	1.71e-118	molecular chaperone [Pantoea sp. AMG 501]	2008894	d__Bacteria;k__norank;p__Proteobacteria;c__Gammaproteobacteria;o__Enterobacterales;f__Erwiniaceae;g__Pantoea;s__Pantoea_sp._AMG_501
PB1_gene_2637	190	OWY74743.1	188	172/188(91.49)	2.71e-94	type 1 fimbrial protein [Pantoea sp. AMG 501]	2008894	d__Bacteria;k__norank;p__Proteobacteria;c__Gammaproteobacteria;o__Enterobacterales;f__Erwiniaceae;g__Pantoea;s__Pantoea_sp._AMG_501
PB1_gene_2640	420	ORM80809.1	420	401/420(95.48)	1.28e-308	hypothetical protein HA42_12850 [Pantoea deleyi]	470932	d__Bacteria;k__norank;p__Proteobacteria;c__Gammaproteobacteria;o__Enterobacterales;f__Erwiniaceae;g__Pantoea;s__Pantoea_deleyi
PB1_gene_2643	167	ORM80727.1	167	167/167(100.00)	2.19e-112	DNA starvation/stationary phase protection protein Dps [Pantoea deleyi]	470932	d__Bacteria;k__norank;p__Proteobacteria;c__Gammaproteobacteria;o__Enterobacterales;f__Erwiniaceae;g__Pantoea;s__Pantoea_deleyi
PB1_gene_2648	300	ORM77870.1	299	295/300(98.33)	3.36e-209	lipid kinase YegS [Pantoea deleyi]	470932	d__Bacteria;k__norank;p__Proteobacteria;c__Gammaproteobacteria;o__Enterobacterales;f__Erwiniaceae;g__Pantoea;s__Pantoea_deleyi
PB1_gene_2652	101	ORM81063.1	79	73/79(92.41)	1.84e-48	hypothetical protein HA42_12020 [Pantoea deleyi]	470932	d__Bacteria;k__norank;p__Proteobacteria;c__Gammaproteobacteria;o__Enterobacterales;f__Erwiniaceae;g__Pantoea;s__Pantoea_deleyi
PB1_gene_2653	131	ORM81038.1	133	127/131(96.95)	4.43e-86	secY/secA suppressor protein [Pantoea deleyi]	470932	d__Bacteria;k__norank;p__Proteobacteria;c__Gammaproteobacteria;o__Enterobacterales;f__Erwiniaceae;g__Pantoea;s__Pantoea_deleyi
PB1_gene_2654	88	ORM81064.1	88	85/88(96.59)	8.41e-59	hypothetical protein HA42_12030 [Pantoea deleyi]	470932	d__Bacteria;k__norank;p__Proteobacteria;c__Gammaproteobacteria;o__Enterobacterales;f__Erwiniaceae;g__Pantoea;s__Pantoea_deleyi
PB1_gene_2656	118	EIB98869.1	118	105/118(88.98)	1.35e-69	HicB family protein [Pantoea sp. Sc1]	593105	d__Bacteria;k__norank;p__Proteobacteria;c__Gammaproteobacteria;o__Enterobacterales;f__Erwiniaceae;g__Pantoea;s__Pantoea_sp._Sc1
PB1_gene_2658	631	ORM80607.1	631	628/631(99.52)	0	chemotaxis protein [Pantoea deleyi]	470932	d__Bacteria;k__norank;p__Proteobacteria;c__Gammaproteobacteria;o__Enterobacterales;f__Erwiniaceae;g__Pantoea;s__Pantoea_deleyi
PB1_gene_2660	243	WP_111138873.1	232	228/232(98.28)	1.08e-156	tRNA (adenosine(37)-N6)-threonylcarbamoyltransferase complex dimerization subunit type 1 TsaB [Pantoea sp.	2027922	d__Bacteria;k__norank;p__Proteobacteria;c__Gammaproteobacteria;o__Enterobacterales;f__Erwiniaceae;g__Pantoea;s__Pantoea_sp._ARC607
PB1_gene_2662	429	ORM78628.1	429	424/429(98.83)	3.62e-283	C4-dicarboxylate transporter DctA [Pantoea deleyi]	470932	d__Bacteria;k__norank;p__Proteobacteria;c__Gammaproteobacteria;o__Enterobacterales;f__Erwiniaceae;g__Pantoea;s__Pantoea_deleyi
PB1_gene_2665	285	WP_083070284.1	281	277/281(98.58)	3.33e-194	BtpA/SgcQ family protein [Pantoea vagans]	53335	d__Bacteria;k__norank;p__Proteobacteria;c__Gammaproteobacteria;o__Enterobacterales;f__Erwiniaceae;g__Pantoea;s__norank
PB1_gene_2667	286	ORM80777.1	286	282/286(98.60)	2.35e-200	formate transporter FocA [Pantoea deleyi]	470932	d__Bacteria;k__norank;p__Proteobacteria;c__Gammaproteobacteria;o__Enterobacterales;f__Erwiniaceae;g__Pantoea;s__Pantoea_deleyi
PB1_gene_2669	370	WP_111139471.1	370	348/370(94.05)	1.18e-249	oxidoreductase [Pantoea sp. ARC607]	2027922	d__Bacteria;k__norank;p__Proteobacteria;c__Gammaproteobacteria;o__Enterobacterales;f__Erwiniaceae;g__Pantoea;s__Pantoea_sp._ARC607
PB1_gene_2670	82	ORM80561.1	82	82/82(100.00)	6.70e-51	30S ribosomal protein S16 [Pantoea deleyi]	470932	d__Bacteria;k__norank;p__Proteobacteria;c__Gammaproteobacteria;o__Enterobacterales;f__Erwiniaceae;g__Pantoea;s__Pantoea_deleyi
PB1_gene_2671	182	ORM80560.1	182	182/182(100.00)	4.35e-133	ribosome maturation factor RimM [Pantoea deleyi]	470932	d__Bacteria;k__norank;p__Proteobacteria;c__Gammaproteobacteria;o__Enterobacterales;f__Erwiniaceae;g__Pantoea;s__Pantoea_deleyi
PB1_gene_2672	254	ORM80559.1	254	254/254(100.00)	5.52e-183	tRNA (guanosine(37)-N1)-methyltransferase TrmD [Pantoea deleyi]	470932	d__Bacteria;k__norank;p__Proteobacteria;c__Gammaproteobacteria;o__Enterobacterales;f__Erwiniaceae;g__Pantoea;s__Pantoea_deleyi
PB1_gene_2673	115	SJZ95973.1	115	115/115(100.00)	3.92e-72	LSU ribosomal protein L19P [Pantoea agglomerans]	470933	d__Bacteria;k__norank;p__Proteobacteria;c__Gammaproteobacteria;o__Enterobacterales;f__Erwiniaceae;g__Pantoea;s__Pantoea_eucalypti

PB1_gene_2676	195	WP_050491308.1	195	136/195(69.74)	3.12e-89	type 1 fimbrial protein [Pantoea agglomerans]	None	Unclassified
PB1_gene_2677	445	ORM81496.1	445	416/445(93.48)	1.34e-309	cytochrome C [Pantoea deleyi]	470932	d__Bacteria;k__norank;p__Proteobacteria;c__Gammaproteobacteria;o__Enterobacterales;f__Erwiniaceae;g__Pantoea;s__Pantoea_deleyi
PB1_gene_2680	113	OQV42947.1	113	113/113(100.00)	3.12e-77	AraC family transcriptional regulator [Pantoea vagans]	470934	d__Bacteria;k__norank;p__Proteobacteria;c__Gammaproteobacteria;o__Enterobacterales;f__Erwiniaceae;g__Pantoea;s__Pantoea_vagans
PB1_gene_2681	384	SJZ46224.1	384	341/384(88.80)	4.43e-236	RND family efflux transporter, MFP subunit [Pantoea	470933	d__Bacteria;k__norank;p__Proteobacteria;c__Gammaproteobacteria;o__Enterobacterales;f__Erwiniaceae;g__Pantoea;s__Pantoea_eucalypti
PB1_gene_2684	235	WP_111139487.1	235	234/235(99.57)	5.33e-176	murein tripeptide amidase MpaA [Pantoea sp. ARC607]	2027922	d__Bacteria;k__norank;p__Proteobacteria;c__Gammaproteobacteria;o__Enterobacterales;f__Erwiniaceae;g__Pantoea;s__Pantoea_sp._ARC607
PB1_gene_2687	131	WP_111141033.1	131	130/131(99.24)	1.56e-90	hypothetical protein [Pantoea sp. ARC607]	2027922	d__Bacteria;k__norank;p__Proteobacteria;c__Gammaproteobacteria;o__Enterobacterales;f__Erwiniaceae;g__Pantoea;s__Pantoea_sp._ARC607
PB1_gene_2688	147	ORM80846.1	147	147/147(100.00)	1.18e-104	ferric iron uptake transcriptional regulator [Pantoea deleyi]	470932	d__Bacteria;k__norank;p__Proteobacteria;c__Gammaproteobacteria;o__Enterobacterales;f__Erwiniaceae;g__Pantoea;s__Pantoea_deleyi
PB1_gene_2691	197	WP_128084137.1	198	185/197(93.91)	2.28e-138	DUF1349 domain-containing protein [Pantoea deleyi]	None	Unclassified
PB1_gene_2692	78	SJZ30802.1	78	78/78(100.00)	1.44e-50	hypothetical protein SAMN03097723_0241 [Pantoea agglomerans]	470933	d__Bacteria;k__norank;p__Proteobacteria;c__Gammaproteobacteria;o__Enterobacterales;f__Erwiniaceae;g__Pantoea;s__Pantoea_eucalypti
PB1_gene_2695	80	ORM86116.1	80	77/80(96.25)	8.98e-46	hypothetical protein HA42_01375 [Pantoea deleyi]	470932	d__Bacteria;k__norank;p__Proteobacteria;c__Gammaproteobacteria;o__Enterobacterales;f__Erwiniaceae;g__Pantoea;s__Pantoea_deleyi
PB1_gene_2696	61	ORM86115.1	61	57/61(93.44)	1.47e-29	hypothetical protein HA42_01370 [Pantoea deleyi]	470932	d__Bacteria;k__norank;p__Proteobacteria;c__Gammaproteobacteria;o__Enterobacterales;f__Erwiniaceae;g__Pantoea;s__Pantoea_deleyi
PB1_gene_2697	98	ORM86114.1	98	97/98(98.98)	1.32e-62	hypothetical protein HA42_01365 [Pantoea deleyi]	470932	d__Bacteria;k__norank;p__Proteobacteria;c__Gammaproteobacteria;o__Enterobacterales;f__Erwiniaceae;g__Pantoea;s__Pantoea_deleyi
PB1_gene_2700	327	ORM80740.1	327	326/327(99.69)	5.13e-236	sugar ABC transporter substrate-binding protein [Pantoea deleyi]	470932	d__Bacteria;k__norank;p__Proteobacteria;c__Gammaproteobacteria;o__Enterobacterales;f__Erwiniaceae;g__Pantoea;s__Pantoea_deleyi
PB1_gene_2701	246	ORM80754.1	244	242/244(99.18)	1.16e-175	phosphoadenosine phosphosulfate reductase [Pantoea deleyi]	470932	d__Bacteria;k__norank;p__Proteobacteria;c__Gammaproteobacteria;o__Enterobacterales;f__Erwiniaceae;g__Pantoea;s__Pantoea_deleyi
PB1_gene_2704	182	WP_089561989.1	314	177/182(97.25)	4.35e-115	MULTISPECIES: hypothetical protein [Pantoea]	53335	d__Bacteria;k__norank;p__Proteobacteria;c__Gammaproteobacteria;o__Enterobacterales;f__Erwiniaceae;g__Pantoea;s__norank
PB1_gene_2707	219	ORM86825.1	218	214/218(98.17)	6.67e-146	hypothetical protein HA42_00150 [Pantoea deleyi]	470932	d__Bacteria;k__norank;p__Proteobacteria;c__Gammaproteobacteria;o__Enterobacterales;f__Erwiniaceae;g__Pantoea;s__Pantoea_deleyi
PB1_gene_2710	172	ORM80948.1	168	167/168(99.40)	1.28e-117	anti-RNA polymerase sigma 70 factor [Pantoea deleyi]	470932	d__Bacteria;k__norank;p__Proteobacteria;c__Gammaproteobacteria;o__Enterobacterales;f__Erwiniaceae;g__Pantoea;s__Pantoea_deleyi
PB1_gene_2715	279	WP_111140300.1	279	273/278(98.20)	6.72e-176	metal ABC transporter permease [Pantoea sp. ARC607]	2027922	d__Bacteria;k__norank;p__Proteobacteria;c__Gammaproteobacteria;o__Enterobacterales;f__Erwiniaceae;g__Pantoea;s__Pantoea_sp._ARC607
PB1_gene_2716	292	WP_111140301.1	292	284/292(97.26)	1.85e-200	metal ABC transporter substrate-binding protein [Pantoea sp. ARC607]	2027922	d__Bacteria;k__norank;p__Proteobacteria;c__Gammaproteobacteria;o__Enterobacterales;f__Erwiniaceae;g__Pantoea;s__Pantoea_sp._ARC607
PB1_gene_2718	383	OQV40317.1	383	381/383(99.48)	8.31e-268	methionine adenosyltransferase [Pantoea vagans]	470934	d__Bacteria;k__norank;p__Proteobacteria;c__Gammaproteobacteria;o__Enterobacterales;f__Erwiniaceae;g__Pantoea;s__Pantoea_vagans
PB1_gene_2721	149	OWS73455.1	270	134/144(93.06)	4.32e-89	IS4 family transposase, partial [Pantoea sp. VS1]	2003658	d__Bacteria;k__norank;p__Proteobacteria;c__Gammaproteobacteria;o__Enterobacterales;f__Erwiniaceae;g__Pantoea;s__Pantoea_sp._VS1
PB1_gene_2722	105	WP_094106191.1	105	103/105(98.10)	4.10e-67	addiction module antidote protein, HigA family [Pantoea	472694	d__Bacteria;k__norank;p__Proteobacteria;c__Gammaproteobacteria;o__Enterobacterales;f__Erwiniaceae;g__Pantoea;s__Pantoea_brenneri
PB1_gene_2723	93	WP_094106190.1	93	91/93(97.85)	1.07e-61	Killer protein [Pantoea brenneri]	472694	d__Bacteria;k__norank;p__Proteobacteria;c__Gammaproteobacteria;o__Enterobacterales;f__Erwiniaceae;g__Pantoea;s__Pantoea_brenneri
PB1_gene_2724	83	WP_107320597.1	268	35/48(72.92)	2.33e-16	hypothetical protein [Pantoea vagans]	None	Unclassified

PB1_gene_2726	158	OWS73455.1	270	60/71(84.51)	1.46e-28	IS4 family transposase, partial [Pantoea sp. VS1]	2003658	d__Bacteria;k__norank;p__Proteobacteria;c__Gammaproteobacteria;o__Enterobacterales;f__Erwiniaceae;g__Pantoea;s__Pantoea_sp._VS1
PB1_gene_2728	90	ORM76858.1	90	90/90(100.00)	6.07e-62	oxidative damage protection protein [Pantoea deleyi]	470932	d__Bacteria;k__norank;p__Proteobacteria;c__Gammaproteobacteria;o__Enterobacterales;f__Erwiniaceae;g__Pantoea;s__Pantoea_deleyi
PB1_gene_2729	359	ORM76857.1	359	356/359(99.16)	2.64e-261	murein transglycosylase C [Pantoea deleyi]	470932	d__Bacteria;k__norank;p__Proteobacteria;c__Gammaproteobacteria;o__Enterobacterales;f__Erwiniaceae;g__Pantoea;s__Pantoea_deleyi
PB1_gene_2730	125	WP_039659957.1	87	64/80(80.00)	8.88e-34	hypothetical protein [Pantoea sp. MBLJ3]	None	Unclassified
PB1_gene_2734	289	WP_111141500.1	299	286/289(98.96)	5.00e-199	ATP phosphoribosyltransferase [Pantoea sp. ARC607]	2027922	d__Bacteria;k__norank;p__Proteobacteria;c__Gammaproteobacteria;o__Enterobacterales;f__Erwiniaceae;g__Pantoea;s__Pantoea_sp._ARC607
PB1_gene_2737	476	WP_031590309.1	495	465/476(97.69)	0	guanosine-5'-triphosphate,3'-diphosphate diphosphatase [Pantoea agglomerans]	549	d__Bacteria;k__norank;p__Proteobacteria;c__Gammaproteobacteria;o__Enterobacterales;f__Erwiniaceae;g__Pantoea;s__Pantoea_agglomerans
PB1_gene_2739	387	WP_064124695.1	387	355/387(91.73)	3.40e-269	phage tail protein [Wolbachia endosymbiont of Dactylopius coccus]	None	Unclassified
PB1_gene_2748	265	WP_031590413.1	265	223/265(84.15)	7.69e-159	ImpE family T6SS protein Cts1E [Pantoea agglomerans]	549	d__Bacteria;k__norank;p__Proteobacteria;c__Gammaproteobacteria;o__Enterobacterales;f__Erwiniaceae;g__Pantoea;s__Pantoea_agglomerans
PB1_gene_2749	167	WP_031590412.1	159	132/159(83.02)	7.10e-89	lysozyme [Pantoea agglomerans]	549	d__Bacteria;k__norank;p__Proteobacteria;c__Gammaproteobacteria;o__Enterobacterales;f__Erwiniaceae;g__Pantoea;s__Pantoea_agglomerans
PB1_gene_2751	343	ORM83051.1	343	338/343(98.54)	1.46e-234	D-methionine ABC transporter, ATP-binding protein [Pantoea	470932	d__Bacteria;k__norank;p__Proteobacteria;c__Gammaproteobacteria;o__Enterobacterales;f__Erwiniaceae;g__Pantoea;s__Pantoea_deleyi
PB1_gene_2752	217	WP_111140604.1	217	217/217(100.00)	3.76e-117	methionine ABC transporter permease MetI [Pantoea sp. ARC607]	2027922	d__Bacteria;k__norank;p__Proteobacteria;c__Gammaproteobacteria;o__Enterobacterales;f__Erwiniaceae;g__Pantoea;s__Pantoea_sp._ARC607
PB1_gene_2755	140	ORM78938.1	140	129/140(92.14)	3.73e-91	C-lysozyme inhibitor [Pantoea deleyi]	470932	d__Bacteria;k__norank;p__Proteobacteria;c__Gammaproteobacteria;o__Enterobacterales;f__Erwiniaceae;g__Pantoea;s__Pantoea_deleyi
PB1_gene_2757	446	ORM81638.1	446	444/446(99.55)	0	glycerol-3-phosphate transporter [Pantoea deleyi]	470932	d__Bacteria;k__norank;p__Proteobacteria;c__Gammaproteobacteria;o__Enterobacterales;f__Erwiniaceae;g__Pantoea;s__Pantoea_deleyi
PB1_gene_2762	110	EAL59809.1	283	98/110(89.09)	3.09e-56	hypothetical protein WwSim0310 [Wolbachia endosymbiont of Drosophila simulans]	77038	d__Bacteria;k__norank;p__Proteobacteria;c__Alphaproteobacteria;o__Rickettsiales;f__Anaplasmataceae;g__Wolbachia;s__Wolbachia_endosymbiont_of_Drosophila_simulans
PB1_gene_2768	372	PXW21517.1	372	367/372(98.66)	2.39e-250	cell elongation-specific peptidoglycan biosynthesis regulator RodA [Pantoea sp. JKS000250]	1938795	d__Bacteria;k__norank;p__Proteobacteria;c__Gammaproteobacteria;o__Enterobacterales;f__Erwiniaceae;g__Pantoea;s__Pantoea_sp._JKS000250
PB1_gene_2770	424	ORM81159.1	424	420/424(99.06)	8.68e-309	tyrosine--tRNA ligase [Pantoea deleyi]	470932	d__Bacteria;k__norank;p__Proteobacteria;c__Gammaproteobacteria;o__Enterobacterales;f__Erwiniaceae;g__Pantoea;s__Pantoea_deleyi
PB1_gene_2779	504	ORM84533.1	504	504/504(100.00)	0	glycerol kinase [Pantoea deleyi]	470932	d__Bacteria;k__norank;p__Proteobacteria;c__Gammaproteobacteria;o__Enterobacterales;f__Erwiniaceae;g__Pantoea;s__Pantoea_deleyi
PB1_gene_2781	370	WP_111142277.1	370	362/370(97.84)	2.37e-265	porin OmpC [Pantoea sp. ARC607]	2027922	d__Bacteria;k__norank;p__Proteobacteria;c__Gammaproteobacteria;o__Enterobacterales;f__Erwiniaceae;g__Pantoea;s__Pantoea_sp._ARC607
PB1_gene_2782	132	ORM80418.1	133	131/131(100.00)	6.56e-95	sigma factor-binding protein Crl [Pantoea deleyi]	470932	d__Bacteria;k__norank;p__Proteobacteria;c__Gammaproteobacteria;o__Enterobacterales;f__Erwiniaceae;g__Pantoea;s__Pantoea_deleyi
PB1_gene_2784	47	SKA18952.1	43	43/43(100.00)	1.87e-21	hypothetical protein SAMN03097723_3486 [Pantoea agglomerans]	470933	d__Bacteria;k__norank;p__Proteobacteria;c__Gammaproteobacteria;o__Enterobacterales;f__Erwiniaceae;g__Pantoea;s__Pantoea_eucalypti
PB1_gene_2785	59	ORM85080.1	59	58/59(98.31)	5.80e-35	DUF2158 domain-containing protein [Pantoea deleyi]	470932	d__Bacteria;k__norank;p__Proteobacteria;c__Gammaproteobacteria;o__Enterobacterales;f__Erwiniaceae;g__Pantoea;s__Pantoea_deleyi

PB1_gene_2786	406	WP_111141186.1	406	405/406(99.75)	2.65e-303	glycosyl transferase [Pantoea sp. ARC607]	2027922	d__Bacteria;k__norank;p__Proteobacteria;c__Gammaproteobacteria;o__Enterobacterales;f__Erwiniaceae;g__Pantoea;s__Pantoea_sp._ARC607
PB1_gene_2789	161	WP_111140160.1	162	139/162(85.80)	1.32e-99	diguanylate cyclase [Pantoea sp. ARC607]	2027922	d__Bacteria;k__norank;p__Proteobacteria;c__Gammaproteobacteria;o__Enterobacterales;f__Erwiniaceae;g__Pantoea;s__Pantoea_sp._ARC607
PB1_gene_2790	116	ORM83593.1	116	111/116(95.69)	1.93e-68	hypothetical protein HA42_05970 [Pantoea deleyi]	470932	d__Bacteria;k__norank;p__Proteobacteria;c__Gammaproteobacteria;o__Enterobacterales;f__Erwiniaceae;g__Pantoea;s__Pantoea_deleyi
PB1_gene_2792	226	WP_111141483.1	214	210/214(98.13)	6.85e-149	aldolase [Pantoea sp. ARC607]	2027922	d__Bacteria;k__norank;p__Proteobacteria;c__Gammaproteobacteria;o__Enterobacterales;f__Erwiniaceae;g__Pantoea;s__Pantoea_sp._ARC607
PB1_gene_2793	261	ORM84476.1	261	254/261(97.32)	9.19e-190	hydroxypyruvate isomerase [Pantoea deleyi]	470932	d__Bacteria;k__norank;p__Proteobacteria;c__Gammaproteobacteria;o__Enterobacterales;f__Erwiniaceae;g__Pantoea;s__Pantoea_deleyi
PB1_gene_2799	161	WP_031590428.1	161	151/161(93.79)	2.91e-105	Hcp1 family type VI secretion system effector [Pantoea	549	d__Bacteria;k__norank;p__Proteobacteria;c__Gammaproteobacteria;o__Enterobacterales;f__Erwiniaceae;g__Pantoea;s__Pantoea_agglomerans
PB1_gene_2801	254	WP_111139047.1	254	253/254(99.61)	2.82e-176	SDR family oxidoreductase [Pantoea sp. ARC607]	2027922	d__Bacteria;k__norank;p__Proteobacteria;c__Gammaproteobacteria;o__Enterobacterales;f__Erwiniaceae;g__Pantoea;s__Pantoea_sp._ARC607
PB1_gene_2803	535	WP_098052929.1	535	519/535(97.01)	0	ABC transporter substrate-binding protein [Pantoea	None	Unclassified
PB1_gene_2810	509	WP_111140559.1	509	498/509(97.84)	0	efflux transporter outer membrane subunit [Pantoea sp. ARC607]	2027922	d__Bacteria;k__norank;p__Proteobacteria;c__Gammaproteobacteria;o__Enterobacterales;f__Erwiniaceae;g__Pantoea;s__Pantoea_sp._ARC607
PB1_gene_2812	376	ORM81450.1	376	375/376(99.73)	4.06e-260	dTDP-4-amino-4,6-dideoxygalactose transaminase	470932	d__Bacteria;k__norank;p__Proteobacteria;c__Gammaproteobacteria;o__Enterobacterales;f__Erwiniaceae;g__Pantoea;s__Pantoea_deleyi
PB1_gene_2813	235	ORM81451.1	235	229/235(97.45)	7.75e-160	TDP-D-fucosamine acetyltransferase [Pantoea deleyi]	470932	d__Bacteria;k__norank;p__Proteobacteria;c__Gammaproteobacteria;o__Enterobacterales;f__Erwiniaceae;g__Pantoea;s__Pantoea_deleyi
PB1_gene_2819	175	WP_111140986.1	175	154/173(89.02)	1.24e-106	DUF2892 domain-containing protein [Pantoea sp. ARC607]	2027922	d__Bacteria;k__norank;p__Proteobacteria;c__Gammaproteobacteria;o__Enterobacterales;f__Erwiniaceae;g__Pantoea;s__Pantoea_sp._ARC607
PB1_gene_2820	142	CCJ84733.1	142	126/142(88.73)	6.90e-88	Arsenate reductase [Cronobacter dublinensis 582]	1208661	d__Bacteria;k__norank;p__Proteobacteria;c__Gammaproteobacteria;o__Enterobacterales;f__Enterobacteriaceae;g__Cronobacter;s__Cronobacter_dublinensis
PB1_gene_2824	433	ORM77161.1	431	429/431(99.54)	1.70e-278	guanine permease [Pantoea deleyi]	470932	d__Bacteria;k__norank;p__Proteobacteria;c__Gammaproteobacteria;o__Enterobacterales;f__Erwiniaceae;g__Pantoea;s__Pantoea_deleyi
PB1_gene_2827	203	ORM85649.1	203	200/202(99.01)	3.77e-142	bifunctional phosphoribosyl-AMP cyclohydrolase/phosphoribosyl-ATP diphosphatase [Pantoea	470932	d__Bacteria;k__norank;p__Proteobacteria;c__Gammaproteobacteria;o__Enterobacterales;f__Erwiniaceae;g__Pantoea;s__Pantoea_deleyi
PB1_gene_2828	258	ORM85650.1	258	256/258(99.22)	2.25e-184	imidazole glycerol phosphate synthase subunit HisF [Pantoea deleyi]	470932	d__Bacteria;k__norank;p__Proteobacteria;c__Gammaproteobacteria;o__Enterobacterales;f__Erwiniaceae;g__Pantoea;s__Pantoea_deleyi
PB1_gene_2832	191	WP_111138615.1	191	183/191(95.81)	3.79e-127	cytochrome b [Pantoea sp. ARC607]	2027922	d__Bacteria;k__norank;p__Proteobacteria;c__Gammaproteobacteria;o__Enterobacterales;f__Erwiniaceae;g__Pantoea;s__Pantoea_sp._ARC607
PB1_gene_2834	505	WP_026743546.1	496	266/486(54.73)	3.44e-152	hypothetical protein [Lonsdalea quercina]	71657	d__Bacteria;k__norank;p__Proteobacteria;c__Gammaproteobacteria;o__Enterobacterales;f__Pectobacteriaceae;g__Lonsdalea;s__Lonsdalea_quercina
PB1_gene_2837	350	WP_111139847.1	811	348/350(99.43)	7.69e-232	mechanosensitive ion channel family protein [Pantoea sp.	2027922	d__Bacteria;k__norank;p__Proteobacteria;c__Gammaproteobacteria;o__Enterobacterales;f__Erwiniaceae;g__Pantoea;s__Pantoea_sp._ARC607
PB1_gene_2839	678	WP_123346400.1	692	403/692(58.24)	9.01e-257	MULTISPECIES: ATP-binding protein [Enterobacter]	2608935	d__Bacteria;k__norank;p__Proteobacteria;c__Gammaproteobacteria;o__Enterobacterales;f__Enterobacteriaceae;g__Enterobacter;s__norank
PB1_gene_2844	104	SUB15949.1	367	98/104(94.23)	3.19e-61	Polyol:NADP oxidoreductase [Pantoea agglomerans]	549	d__Bacteria;k__norank;p__Proteobacteria;c__Gammaproteobacteria;o__Enterobacterales;f__Erwiniaceae;g__Pantoea;s__Pantoea_agglomerans
PB1_gene_2845	427	EIB98223.1	427	415/427(97.19)	1.91e-293	MFS family transporter [Pantoea sp. Sc1]	593105	d__Bacteria;k__norank;p__Proteobacteria;c__Gammaproteobacteria;o__Enterobacterales;f__Erwiniaceae;g__Pantoea;s__Pantoea_sp._Sc1
PB1_gene_2847	622	ORM83138.1	622	615/622(98.87)	0	peptidylprolyl isomerase [Pantoea deleyi]	470932	d__Bacteria;k__norank;p__Proteobacteria;c__Gammaproteobacteria;o__Enterobacterales;f__Erwiniaceae;g__Pantoea;s__Pantoea_deleyi
PB1_gene_2849	124	SJZ90721.1	124	109/124(87.90)	1.54e-71	Helix-turn-helix [Pantoea agglomerans]	470933	d__Bacteria;k__norank;p__Proteobacteria;c__Gammaproteobacteria;o__Enterobacterales;f__Erwiniaceae;g__Pantoea;s__Pantoea_eucalypti

PB1_gene_2850	91	WP_046101027.1	91	87/91(95.60)	4.99e-57	hypothetical protein [Pantoea anthophila]	53335	d__Bacteria;k__norank;p__Proteobacteria;c__Gammaproteobacteria;o__Enterobacterales;f__Erwiniaceae;g__Pantoea;s__norank
PB1_gene_2851	135	ORM85446.1	135	134/135(99.26)	2.88e-88	TIGR00156 family protein [Pantoea deleyi]	470932	d__Bacteria;k__norank;p__Proteobacteria;c__Gammaproteobacteria;o__Enterobacterales;f__Erwiniaceae;g__Pantoea;s__Pantoea_deleyi
PB1_gene_2852	139	ORM85445.1	139	139/139(100.00)	2.96e-92	D-ribose pyranase [Pantoea deleyi]	470932	d__Bacteria;k__norank;p__Proteobacteria;c__Gammaproteobacteria;o__Enterobacterales;f__Erwiniaceae;g__Pantoea;s__Pantoea_deleyi
PB1_gene_2856	275	WP_111140771.1	275	253/275(92.00)	1.97e-182	protein of avirulence locus ImpE [Pantoea sp. ARC607]	2027922	d__Bacteria;k__norank;p__Proteobacteria;c__Gammaproteobacteria;o__Enterobacterales;f__Erwiniaceae;g__Pantoea;s__Pantoea_sp._ARC607
PB1_gene_2857	324	ORM77613.1	322	314/324(96.91)	4.34e-226	hypothetical protein HA42_17575 [Pantoea deleyi]	470932	d__Bacteria;k__norank;p__Proteobacteria;c__Gammaproteobacteria;o__Enterobacterales;f__Erwiniaceae;g__Pantoea;s__Pantoea_deleyi
PB1_gene_2859	218	KDA96086.1	218	217/218(99.54)	6.21e-153	Clp protease proteolytic subunit ClpP [Pantoea agglomerans	1408177	d__Bacteria;k__norank;p__Proteobacteria;c__Gammaproteobacteria;o__Enterobacterales;f__Erwiniaceae;g__Pantoea;s__Pantoea_agglomerans
PB1_gene_2861	466	ORM80362.1	466	466/466(100.00)	0	NAD(P)(+) transhydrogenase [Pantoea deleyi]	470932	d__Bacteria;k__norank;p__Proteobacteria;c__Gammaproteobacteria;o__Enterobacterales;f__Erwiniaceae;g__Pantoea;s__Pantoea_deleyi
PB1_gene_2863	316	WP_098050431.1	316	280/314(89.17)	3.16e-204	glycosyltransferase [Pantoea agglomerans]	None	Unclassified
PB1_gene_2867	448	WP_111138865.1	454	447/448(99.78)	0	L-serine ammonia-lyase [Pantoea sp. ARC607]	2027922	d__Bacteria;k__norank;p__Proteobacteria;c__Gammaproteobacteria;o__Enterobacterales;f__Erwiniaceae;g__Pantoea;s__Pantoea_sp._ARC607
PB1_gene_2869	363	WP_111139394.1	363	360/363(99.17)	1.41e-258	3-isopropylmalate dehydrogenase [Pantoea sp. ARC607]	2027922	d__Bacteria;k__norank;p__Proteobacteria;c__Gammaproteobacteria;o__Enterobacterales;f__Erwiniaceae;g__Pantoea;s__Pantoea_sp._ARC607
PB1_gene_2871	391	ORM86090.1	389	387/389(99.49)	2.75e-281	lipopolysaccharide assembly protein LapB [Pantoea deleyi]	470932	d__Bacteria;k__norank;p__Proteobacteria;c__Gammaproteobacteria;o__Enterobacterales;f__Erwiniaceae;g__Pantoea;s__Pantoea_deleyi
PB1_gene_2873	232	WP_111140083.1	232	228/232(98.28)	6.47e-171	L-ribulose-5-phosphate 4-epimerase [Pantoea sp. ARC607]	2027922	d__Bacteria;k__norank;p__Proteobacteria;c__Gammaproteobacteria;o__Enterobacterales;f__Erwiniaceae;g__Pantoea;s__Pantoea_sp._ARC607
PB1_gene_2876	511	WP_111138885.1	511	509/511(99.61)	0	SpoVR family protein [Pantoea sp. ARC607]	2027922	d__Bacteria;k__norank;p__Proteobacteria;c__Gammaproteobacteria;o__Enterobacterales;f__Erwiniaceae;g__Pantoea;s__Pantoea_sp._ARC607
PB1_gene_2878	395	ORM78516.1	395	392/395(99.24)	8.92e-285	alpha-hydroxy-acid oxidizing enzyme [Pantoea deleyi]	470932	d__Bacteria;k__norank;p__Proteobacteria;c__Gammaproteobacteria;o__Enterobacterales;f__Erwiniaceae;g__Pantoea;s__Pantoea_deleyi
PB1_gene_2879	142	ORM78517.1	142	141/142(99.30)	3.43e-97	OsmC family peroxiredoxin [Pantoea deleyi]	470932	d__Bacteria;k__norank;p__Proteobacteria;c__Gammaproteobacteria;o__Enterobacterales;f__Erwiniaceae;g__Pantoea;s__Pantoea_deleyi
PB1_gene_2880	34	KDA96315.1	34	29/33(87.88)	7.60e-12	hypothetical protein T296_01255 [Pantoea agglomerans Eh318]	1408177	d__Bacteria;k__norank;p__Proteobacteria;c__Gammaproteobacteria;o__Enterobacterales;f__Erwiniaceae;g__Pantoea;s__Pantoea_agglomerans
PB1_gene_2882	387	WP_111141752.1	387	381/387(98.45)	4.23e-247	MFS transporter [Pantoea sp. ARC607]	2027922	d__Bacteria;k__norank;p__Proteobacteria;c__Gammaproteobacteria;o__Enterobacterales;f__Erwiniaceae;g__Pantoea;s__Pantoea_sp._ARC607
PB1_gene_2884	126	ORM84916.1	126	125/126(99.21)	3.75e-82	holo-ACP synthase [Pantoea deleyi]	470932	d__Bacteria;k__norank;p__Proteobacteria;c__Gammaproteobacteria;o__Enterobacterales;f__Erwiniaceae;g__Pantoea;s__Pantoea_deleyi
PB1_gene_2885	69	ORM84917.1	82	67/69(97.10)	3.47e-45	ferredoxin [Pantoea deleyi]	470932	d__Bacteria;k__norank;p__Proteobacteria;c__Gammaproteobacteria;o__Enterobacterales;f__Erwiniaceae;g__Pantoea;s__Pantoea_deleyi
PB1_gene_2886	279	ORM84918.1	279	279/279(100.00)	8.00e-184	RpiR family transcriptional regulator [Pantoea deleyi]	470932	d__Bacteria;k__norank;p__Proteobacteria;c__Gammaproteobacteria;o__Enterobacterales;f__Erwiniaceae;g__Pantoea;s__Pantoea_deleyi
PB1_gene_2888	191	WP_111141621.1	240	187/191(97.91)	1.36e-129	nicotinamide riboside transporter PnuC [Pantoea sp. ARC607]	2027922	d__Bacteria;k__norank;p__Proteobacteria;c__Gammaproteobacteria;o__Enterobacterales;f__Erwiniaceae;g__Pantoea;s__Pantoea_sp._ARC607
PB1_gene_2889	353	WP_111141619.1	353	351/353(99.43)	1.40e-253	quinolinate synthase NadA [Pantoea sp. ARC607]	2027922	d__Bacteria;k__norank;p__Proteobacteria;c__Gammaproteobacteria;o__Enterobacterales;f__Erwiniaceae;g__Pantoea;s__Pantoea_sp._ARC607
PB1_gene_2901	284	ORM83272.1	273	266/272(97.79)	2.78e-193	arylesterase [Pantoea deleyi]	470932	d__Bacteria;k__norank;p__Proteobacteria;c__Gammaproteobacteria;o__Enterobacterales;f__Erwiniaceae;g__Pantoea;s__Pantoea_deleyi
PB1_gene_2903	539	ORM80868.1	539	536/539(99.44)	0	phosphoenolpyruvate carboxykinase (ATP) [Pantoea	470932	d__Bacteria;k__norank;p__Proteobacteria;c__Gammaproteobacteria;o__Enterobacterales;f__Erwiniaceae;g__Pantoea;s__Pantoea_deleyi
PB1_gene_2906	141	ORM85828.1	141	127/141(90.07)	3.58e-94	DUF4186 domain-containing protein [Pantoea deleyi]	470932	d__Bacteria;k__norank;p__Proteobacteria;c__Gammaproteobacteria;o__Enterobacterales;f__Erwiniaceae;g__Pantoea;s__Pantoea_deleyi

PB1_gene_2907	90	ORM85829.1	90	87/90(96.67)	1.62e-57	cupin [Pantoea deleyi]	470932	d__Bacteria;k__norank;p__Proteobacteria;c__Gammaproteobacteria;o__Enterobacterales;f__Erwiniaceae;g__Pantoea;s__Pantoea_deleyi
PB1_gene_2908	87	WP_111139039.1	80	68/80(85.00)	8.14e-44	hypothetical protein [Pantoea sp. ARC607]	2027922	d__Bacteria;k__norank;p__Proteobacteria;c__Gammaproteobacteria;o__Enterobacterales;f__Erwiniaceae;g__Pantoea;s__Pantoea_sp._ARC607
PB1_gene_2910	431	ORM85645.1	432	420/430(97.67)	3.09e-299	hypothetical protein HA42_02255 [Pantoea deleyi]	470932	d__Bacteria;k__norank;p__Proteobacteria;c__Gammaproteobacteria;o__Enterobacterales;f__Erwiniaceae;g__Pantoea;s__Pantoea_deleyi
PB1_gene_2917	112	SJZ30320.1	112	112/112(100.00)	3.53e-69	nitrogen regulatory protein P-II family [Pantoea agglomerans]	470933	d__Bacteria;k__norank;p__Proteobacteria;c__Gammaproteobacteria;o__Enterobacterales;f__Erwiniaceae;g__Pantoea;s__Pantoea_eucalypti
PB1_gene_2921	315	ORM82820.1	321	302/315(95.87)	2.57e-204	flagellar hook-filament junction protein FlgL [Pantoea deleyi]	470932	d__Bacteria;k__norank;p__Proteobacteria;c__Gammaproteobacteria;o__Enterobacterales;f__Erwiniaceae;g__Pantoea;s__Pantoea_deleyi
PB1_gene_2924	150	WP_119803837.1	149	130/148(87.84)	3.47e-87	phage tail protein [Serratia sp. S40]	2338073	d__Bacteria;k__norank;p__Proteobacteria;c__Gammaproteobacteria;o__Enterobacterales;f__Yersiniaceae;g__Serratia;s__Serratia_sp._S40
PB1_gene_2925	414	WP_123346421.1	408	270/414(65.22)	3.24e-184	MULTISPECIES: phage tail sheath family protein	2608935	d__Bacteria;k__norank;p__Proteobacteria;c__Gammaproteobacteria;o__Enterobacterales;f__Enterobacteriaceae;g__Enterobacter;s__norank
PB1_gene_2928	440	WP_111138802.1	440	439/440(99.77)	9.54e-316	chromosome partition protein MukF [Pantoea sp. ARC607]	2027922	d__Bacteria;k__norank;p__Proteobacteria;c__Gammaproteobacteria;o__Enterobacterales;f__Erwiniaceae;g__Pantoea;s__Pantoea_sp._ARC607
PB1_gene_2930	315	WP_111139846.1	315	314/315(99.68)	2.10e-213	D-alanyl-D-alanine endopeptidase [Pantoea sp. ARC607]	2027922	d__Bacteria;k__norank;p__Proteobacteria;c__Gammaproteobacteria;o__Enterobacterales;f__Erwiniaceae;g__Pantoea;s__Pantoea_sp._ARC607
PB1_gene_2931	69	WP_111139847.1	811	69/69(100.00)	2.94e-34	mechanosensitive ion channel family protein [Pantoea sp.	2027922	d__Bacteria;k__norank;p__Proteobacteria;c__Gammaproteobacteria;o__Enterobacterales;f__Erwiniaceae;g__Pantoea;s__Pantoea_sp._ARC607
PB1_gene_2933	260	ORM82815.1	260	260/260(100.00)	1.70e-180	flagellar basal-body rod protein FlgG [Pantoea deleyi]	470932	d__Bacteria;k__norank;p__Proteobacteria;c__Gammaproteobacteria;o__Enterobacterales;f__Erwiniaceae;g__Pantoea;s__Pantoea_deleyi
PB1_gene_2934	236	PAW36956.1	236	230/236(97.46)	5.65e-158	flagellar basal body L-ring protein FlgH [Pantoea vagans]	470934	d__Bacteria;k__norank;p__Proteobacteria;c__Gammaproteobacteria;o__Enterobacterales;f__Erwiniaceae;g__Pantoea;s__Pantoea_vagans
PB1_gene_2937	263	WP_111138854.1	263	260/263(98.86)	2.14e-180	DNA-binding transcriptional regulator KdgR [Pantoea sp. ARC607]	2027922	d__Bacteria;k__norank;p__Proteobacteria;c__Gammaproteobacteria;o__Enterobacterales;f__Erwiniaceae;g__Pantoea;s__Pantoea_sp._ARC607
PB1_gene_2939	220	ORM85479.1	220	219/220(99.55)	3.55e-144	OmpA family lipoprotein [Pantoea deleyi]	470932	d__Bacteria;k__norank;p__Proteobacteria;c__Gammaproteobacteria;o__Enterobacterales;f__Erwiniaceae;g__Pantoea;s__Pantoea_deleyi
PB1_gene_2940	188	WP_111141076.1	188	178/188(94.68)	3.41e-133	DNA-3-methyladenine glycosylase I [Pantoea sp.	2027922	d__Bacteria;k__norank;p__Proteobacteria;c__Gammaproteobacteria;o__Enterobacterales;f__Erwiniaceae;g__Pantoea;s__Pantoea_sp._ARC607
PB1_gene_2947	256	ORM80479.1	256	252/256(98.44)	3.06e-181	2-dehydro-3-deoxyglucarate aldolase [Pantoea deleyi]	470932	d__Bacteria;k__norank;p__Proteobacteria;c__Gammaproteobacteria;o__Enterobacterales;f__Erwiniaceae;g__Pantoea;s__Pantoea_deleyi
PB1_gene_2950	266	ORM82066.1	266	263/266(98.87)	2.31e-181	bifunctional hydroxymethylpyrimidine kinase/phosphomethylpyrimidine kinase [Pantoea deleyi]	470932	d__Bacteria;k__norank;p__Proteobacteria;c__Gammaproteobacteria;o__Enterobacterales;f__Erwiniaceae;g__Pantoea;s__Pantoea_deleyi
PB1_gene_2951	236	WP_128085253.1	265	230/236(97.46)	4.05e-151	hydroxyethylthiazole kinase [Pantoea deleyi]	470932	d__Bacteria;k__norank;p__Proteobacteria;c__Gammaproteobacteria;o__Enterobacterales;f__Erwiniaceae;g__Pantoea;s__Pantoea_deleyi
PB1_gene_2953	453	ORM80562.1	453	452/453(99.78)	5.52e-313	signal recognition particle protein [Pantoea deleyi]	470932	d__Bacteria;k__norank;p__Proteobacteria;c__Gammaproteobacteria;o__Enterobacterales;f__Erwiniaceae;g__Pantoea;s__Pantoea_deleyi
PB1_gene_2954	196	WP_111140068.1	196	189/196(96.43)	1.48e-131	cob(I)yrinic acid a,c-diamide adenosyltransferase [Pantoea sp. ARC607]	2027922	d__Bacteria;k__norank;p__Proteobacteria;c__Gammaproteobacteria;o__Enterobacterales;f__Erwiniaceae;g__Pantoea;s__Pantoea_sp._ARC607
PB1_gene_2955	296	ORM86099.1	296	293/296(98.99)	1.27e-209	23S rRNA pseudouridylate synthase [Pantoea deleyi]	470932	d__Bacteria;k__norank;p__Proteobacteria;c__Gammaproteobacteria;o__Enterobacterales;f__Erwiniaceae;g__Pantoea;s__Pantoea_deleyi
PB1_gene_2957	151	ORM81554.1	152	148/151(98.01)	1.41e-96	cytochrome C [Pantoea deleyi]	470932	d__Bacteria;k__norank;p__Proteobacteria;c__Gammaproteobacteria;o__Enterobacterales;f__Erwiniaceae;g__Pantoea;s__Pantoea_deleyi
PB1_gene_2963	154	AWP34177.1	154	153/154(99.35)	1.78e-107	aspartate carbamoyltransferase regulatory subunit [Pantoea	470934	d__Bacteria;k__norank;p__Proteobacteria;c__Gammaproteobacteria;o__Enterobacterales;f__Erwiniaceae;g__Pantoea;s__Pantoea_vagans

PB1_gene_2964	129	ORM86008.1	128	126/128(98.44)	8.80e-80	reactive intermediate/imine deaminase [Pantoea deleyi]	470932	d__Bacteria;k__norank;p__Proteobacteria;c__Gammaproteobacteria;o__Enterobacterales;f__Erwiniaceae;g__Pantoea;s__Pantoea_deleyi
PB1_gene_2967	286	ADO10522.1	286	275/286(96.15)	1.07e-196	Putative ABC transporter, ATP-binding protein [Pantoea vagans C9-1]	712898	d__Bacteria;k__norank;p__Proteobacteria;c__Gammaproteobacteria;o__Enterobacterales;f__Erwiniaceae;g__Pantoea;s__Pantoea_vagans
PB1_gene_2968	294	WP_111139926.1	285	284/285(99.65)	7.24e-190	ABC transporter permease [Pantoea sp. ARC607]	53335	d__Bacteria;k__norank;p__Proteobacteria;c__Gammaproteobacteria;o__Enterobacterales;f__Erwiniaceae;g__Pantoea;s__norank
PB1_gene_2970	324	ORM84686.1	324	304/324(93.83)	4.18e-229	hydrolase [Pantoea deleyi]	470932	d__Bacteria;k__norank;p__Proteobacteria;c__Gammaproteobacteria;o__Enterobacterales;f__Erwiniaceae;g__Pantoea;s__Pantoea_deleyi
PB1_gene_2972	71	ORM83126.1	71	70/71(98.59)	1.54e-42	transcriptional regulator [Pantoea deleyi]	470932	d__Bacteria;k__norank;p__Proteobacteria;c__Gammaproteobacteria;o__Enterobacterales;f__Erwiniaceae;g__Pantoea;s__Pantoea_deleyi
PB1_gene_2973	130	ORM83125.1	125	125/125(100.00)	6.83e-88	Hha toxicity attenuator [Pantoea deleyi]	470932	d__Bacteria;k__norank;p__Proteobacteria;c__Gammaproteobacteria;o__Enterobacterales;f__Erwiniaceae;g__Pantoea;s__Pantoea_deleyi
PB1_gene_2974	117	WP_111140298.1	117	114/117(97.44)	1.85e-80	hypothetical protein [Pantoea sp. ARC607]	2027922	d__Bacteria;k__norank;p__Proteobacteria;c__Gammaproteobacteria;o__Enterobacterales;f__Erwiniaceae;g__Pantoea;s__Pantoea_sp._ARC607
PB1_gene_2977	213	WP_111138726.1	213	211/213(99.06)	1.12e-157	DNA oxidative demethylase AlkB [Pantoea sp. ARC607]	53335	d__Bacteria;k__norank;p__Proteobacteria;c__Gammaproteobacteria;o__Enterobacterales;f__Erwiniaceae;g__Pantoea;s__norank
PB1_gene_2982	77	WP_111142270.1	77	76/77(98.70)	1.78e-46	hypothetical protein [Pantoea sp. ARC607]	2027922	d__Bacteria;k__norank;p__Proteobacteria;c__Gammaproteobacteria;o__Enterobacterales;f__Erwiniaceae;g__Pantoea;s__Pantoea_sp._ARC607
PB1_gene_2987	506	ORM81632.1	499	498/499(99.80)	9.65e-307	recombinase RmuC [Pantoea deleyi]	470932	d__Bacteria;k__norank;p__Proteobacteria;c__Gammaproteobacteria;o__Enterobacterales;f__Erwiniaceae;g__Pantoea;s__Pantoea_deleyi
PB1_gene_2989	126	ORM77976.1	126	126/126(100.00)	1.12e-83	aspartate 1-decarboxylase [Pantoea deleyi]	470932	d__Bacteria;k__norank;p__Proteobacteria;c__Gammaproteobacteria;o__Enterobacterales;f__Erwiniaceae;g__Pantoea;s__Pantoea_deleyi
PB1_gene_2990	284	ORM77977.1	284	279/284(98.24)	1.52e-195	pantoate--beta-alanine ligase [Pantoea deleyi]	470932	d__Bacteria;k__norank;p__Proteobacteria;c__Gammaproteobacteria;o__Enterobacterales;f__Erwiniaceae;g__Pantoea;s__Pantoea_deleyi
PB1_gene_2994	212	ASQ15713.1	212	166/212(78.30)	2.71e-119	hypothetical protein BJM06_a00067 (plasmid)	550	d__Bacteria;k__norank;p__Proteobacteria;c__Gammaproteobacteria;o__Enterobacterales;f__Enterobacteriaceae;g__Enterobacter;s__Enterobacter_cloacae
PB1_gene_2997	300	ORM84514.1	300	291/300(97.00)	4.18e-201	hypothetical protein HA42_04195 [Pantoea deleyi]	470932	d__Bacteria;k__norank;p__Proteobacteria;c__Gammaproteobacteria;o__Enterobacterales;f__Erwiniaceae;g__Pantoea;s__Pantoea_deleyi
PB1_gene_2999	159	ORM85082.1	159	155/159(97.48)	4.87e-112	very short patch repair endonuclease [Pantoea deleyi]	470932	d__Bacteria;k__norank;p__Proteobacteria;c__Gammaproteobacteria;o__Enterobacterales;f__Erwiniaceae;g__Pantoea;s__Pantoea_deleyi
PB1_gene_3000	91	SKA18920.1	91	88/91(96.70)	1.05e-58	hypothetical protein SAMN03097723_3484 [Pantoea agglomerans]	470933	d__Bacteria;k__norank;p__Proteobacteria;c__Gammaproteobacteria;o__Enterobacterales;f__Erwiniaceae;g__Pantoea;s__Pantoea_eucalypti
PB1_gene_3001	303	ORM85081.1	303	296/303(97.69)	1.55e-193	hypothetical protein HA42_03420 [Pantoea deleyi]	470932	d__Bacteria;k__norank;p__Proteobacteria;c__Gammaproteobacteria;o__Enterobacterales;f__Erwiniaceae;g__Pantoea;s__Pantoea_deleyi
PB1_gene_3002	308	ORM83146.1	308	308/308(100.00)	6.87e-220	cytochrome o ubiquinol oxidase subunit II [Pantoea deleyi]	470932	d__Bacteria;k__norank;p__Proteobacteria;c__Gammaproteobacteria;o__Enterobacterales;f__Erwiniaceae;g__Pantoea;s__Pantoea_deleyi
PB1_gene_3005	244	ORM85097.1	243	243/243(100.00)	1.49e-159	flagellar biosynthetic protein FliP [Pantoea deleyi]	470932	d__Bacteria;k__norank;p__Proteobacteria;c__Gammaproteobacteria;o__Enterobacterales;f__Erwiniaceae;g__Pantoea;s__Pantoea_deleyi
PB1_gene_3006	132	ORM85071.1	132	130/132(98.48)	1.14e-79	flagellar biosynthetic protein FliO [Pantoea deleyi]	470932	d__Bacteria;k__norank;p__Proteobacteria;c__Gammaproteobacteria;o__Enterobacterales;f__Erwiniaceae;g__Pantoea;s__Pantoea_deleyi
PB1_gene_3007	135	SKA19127.1	135	135/135(100.00)	7.94e-86	flagellar motor switch protein FliN/FliY [Pantoea agglomerans]	470933	d__Bacteria;k__norank;p__Proteobacteria;c__Gammaproteobacteria;o__Enterobacterales;f__Erwiniaceae;g__Pantoea;s__Pantoea_eucalypti
PB1_gene_3009	276	WP_114517341.1	270	255/268(95.15)	1.78e-184	AAA family ATPase [Wolbachia endosymbiont of Cylisticus convexus]	118728	d__Bacteria;k__norank;p__Proteobacteria;c__Alphaproteobacteria;o__Rickettsiales;f__Anaplasmataceae;g__Wolbachia;s__Wolbachia_endosymbiont_of_Cylisticus_convexus
PB1_gene_3010	167	OJH31406.1	167	153/167(91.62)	3.76e-104	hypothetical protein Wxf_00797 [Wolbachia endosymbiont of Armadillidium vulgare]	77039	d__Bacteria;k__norank;p__Proteobacteria;c__Alphaproteobacteria;o__Rickettsiales;f__Anaplasmataceae;g__Wolbachia;s__Wolbachia_endosymbiont_of_Armadillidium_vulgare

PB1_gene_3019	194	WP_111141953.1	194	189/194(97.42)	2.27e-125	response regulator transcription factor [Pantoea sp. ARC607]	2027922	d__Bacteria;k__norank;p__Proteobacteria;c__Gammaproteobacteria;o__Enterobacterales;f__Erwiniaceae;g__Pantoea;s__Pantoea_sp._ARC607
PB1_gene_3021	93	ORM81533.1	93	90/93(96.77)	1.78e-60	hypothetical protein HA42_10305 [Pantoea deleyi]	470932	d__Bacteria;k__norank;p__Proteobacteria;c__Gammaproteobacteria;o__Enterobacterales;f__Erwiniaceae;g__Pantoea;s__Pantoea_deleyi
PB1_gene_3022	291	ORM81534.1	291	283/291(97.25)	3.40e-216	gluconolactonase [Pantoea deleyi]	470932	d__Bacteria;k__norank;p__Proteobacteria;c__Gammaproteobacteria;o__Enterobacterales;f__Erwiniaceae;g__Pantoea;s__Pantoea_deleyi
PB1_gene_3027	239	WP_111138768.1	239	236/239(98.74)	3.83e-162	outer membrane permeability protein SanA [Pantoea sp.	2027922	d__Bacteria;k__norank;p__Proteobacteria;c__Gammaproteobacteria;o__Enterobacterales;f__Erwiniaceae;g__Pantoea;s__Pantoea_sp._ARC607
PB1_gene_3030	367	WP_111140433.1	367	361/367(98.37)	2.55e-263	tRNA (uridine(54)-C5)-methyltransferase TrmA [Pantoea sp. ARC607]	2027922	d__Bacteria;k__norank;p__Proteobacteria;c__Gammaproteobacteria;o__Enterobacterales;f__Erwiniaceae;g__Pantoea;s__Pantoea_sp._ARC607
PB1_gene_3034	499	WP_111140487.1	499	491/499(98.40)	0	L-arabinose isomerase [Pantoea sp. ARC607]	2027922	d__Bacteria;k__norank;p__Proteobacteria;c__Gammaproteobacteria;o__Enterobacterales;f__Erwiniaceae;g__Pantoea;s__Pantoea_sp._ARC607
PB1_gene_3037	81	WP_111139528.1	81	79/81(97.53)	2.28e-44	hypothetical protein [Pantoea sp. ARC607]	2027922	d__Bacteria;k__norank;p__Proteobacteria;c__Gammaproteobacteria;o__Enterobacterales;f__Erwiniaceae;g__Pantoea;s__Pantoea_sp._ARC607
PB1_gene_3038	153	WP_046103196.1	204	147/153(96.08)	1.11e-96	response regulator transcription factor [Pantoea anthophila]	470931	d__Bacteria;k__norank;p__Proteobacteria;c__Gammaproteobacteria;o__Enterobacterales;f__Erwiniaceae;g__Pantoea;s__Pantoea_anthophila
PB1_gene_3040	307	ORM84451.1	307	307/307(100.00)	2.98e-209	rhizopine-binding protein [Pantoea deleyi]	470932	d__Bacteria;k__norank;p__Proteobacteria;c__Gammaproteobacteria;o__Enterobacterales;f__Erwiniaceae;g__Pantoea;s__Pantoea_deleyi
PB1_gene_3041	522	WP_111139928.1	522	517/522(99.04)	0	ABC transporter substrate-binding protein [Pantoea sp.	2027922	d__Bacteria;k__norank;p__Proteobacteria;c__Gammaproteobacteria;o__Enterobacterales;f__Erwiniaceae;g__Pantoea;s__Pantoea_sp._ARC607
PB1_gene_3044	315	ORM75629.1	315	314/315(99.68)	2.83e-227	type I pantothenate kinase [Pantoea deleyi]	470932	d__Bacteria;k__norank;p__Proteobacteria;c__Gammaproteobacteria;o__Enterobacterales;f__Erwiniaceae;g__Pantoea;s__Pantoea_deleyi
PB1_gene_3047	335	WP_111139562.1	311	301/311(96.78)	2.85e-213	transporter substrate-binding domain-containing protein [Pantoea sp. ARC607]	2027922	d__Bacteria;k__norank;p__Proteobacteria;c__Gammaproteobacteria;o__Enterobacterales;f__Erwiniaceae;g__Pantoea;s__Pantoea_sp._ARC607
PB1_gene_3048	89	ORM76423.1	179	63/83(75.90)	2.58e-32	transcriptional regulator [Pantoea deleyi]	470932	d__Bacteria;k__norank;p__Proteobacteria;c__Gammaproteobacteria;o__Enterobacterales;f__Erwiniaceae;g__Pantoea;s__Pantoea_deleyi
PB1_gene_3050	78	WP_111139049.1	77	74/77(96.10)	1.16e-49	hypothetical protein [Pantoea sp. ARC607]	None	Unclassified
PB1_gene_3053	208	PCG11194.1	205	196/205(95.61)	2.67e-146	lipoate-protein ligase B [Wolbachia pipientis]	None	Unclassified
PB1_gene_3059	438	WP_090963068.1	438	437/438(99.77)	8.15e-296	gluconate transporter [Pantoea sp. OV426]	1881036	d__Bacteria;k__norank;p__Proteobacteria;c__Gammaproteobacteria;o__Enterobacterales;f__Erwiniaceae;g__Pantoea;s__Pantoea_sp._OV426
PB1_gene_3061	308	KEY41158.1	308	290/308(94.16)	2.64e-208	hypothetical protein FB99_34460 [Pantoea agglomerans]	549	d__Bacteria;k__norank;p__Proteobacteria;c__Gammaproteobacteria;o__Enterobacterales;f__Erwiniaceae;g__Pantoea;s__Pantoea_agglomerans
PB1_gene_3064	144	WP_081142544.1	122	109/122(89.34)	5.41e-79	DUF2778 domain-containing protein [Pantoea latae]	1964541	d__Bacteria;k__norank;p__Proteobacteria;c__Gammaproteobacteria;o__Enterobacterales;f__Erwiniaceae;g__Pantoea;s__Pantoea_latae
PB1_gene_3065	144	WP_120455513.1	144	106/144(73.61)	1.34e-70	Shiga toxin A subunit [Erwiniaceae bacterium IIIF1SW-	2364647	d__Bacteria;k__norank;p__Proteobacteria;c__Gammaproteobacteria;o__Enterobacterales;f__Erwiniaceae;g__Kalamiella;s__Kalamiella_piersonii
PB1_gene_3066	105	KYR35448.1	125	77/101(76.24)	2.78e-51	integrase [Escherichia coli]	562	d__Bacteria;k__norank;p__Proteobacteria;c__Gammaproteobacteria;o__Enterobacterales;f__Enterobacteriaceae;g__Escherichia;s__Escherichia_coli
PB1_gene_3069	396	ORM86105.1	396	394/396(99.49)	9.00e-290	tryptophan synthase subunit beta [Pantoea deleyi]	470932	d__Bacteria;k__norank;p__Proteobacteria;c__Gammaproteobacteria;o__Enterobacterales;f__Erwiniaceae;g__Pantoea;s__Pantoea_deleyi
PB1_gene_3075	171	OJH31718.1	166	131/165(79.39)	3.93e-84	hypothetical protein Wxf_01114 [Wolbachia endosymbiont of Armadillidium vulgare]	77039	d__Bacteria;k__norank;p__Proteobacteria;c__Alphaproteobacteria;o__Rickettsiales;f__Anaplasmataceae;g__Wolbachia;s__Wolbachia_endosymbiont_of_Armadillidium_vulgare
PB1_gene_3078	219	ORM84924.1	219	218/219(99.54)	1.58e-151	hypothetical protein HA42_03755 [Pantoea deleyi]	470932	d__Bacteria;k__norank;p__Proteobacteria;c__Gammaproteobacteria;o__Enterobacterales;f__Erwiniaceae;g__Pantoea;s__Pantoea_deleyi
PB1_gene_3086	292	ORM81643.1	292	277/292(94.86)	4.61e-210	phospholipase [Pantoea deleyi]	470932	d__Bacteria;k__norank;p__Proteobacteria;c__Gammaproteobacteria;o__Enterobacterales;f__Erwiniaceae;g__Pantoea;s__Pantoea_deleyi

PB1_gene_3091	54	WP_128085964.1	54	50/54(92.59)	7.94e-22	DUF2559 family protein [Pantoea deleyi]	470932	d__Bacteria;k__norank;p__Proteobacteria;c__Gammaproteobacteria;o__Enterobacterales;f__Erwiniaceae;g__Pantoea;s__Pantoea_deleyi
PB1_gene_3092	203	ORM80543.1	200	197/200(98.50)	3.27e-149	cell filamentation protein Fic [Pantoea deleyi]	470932	d__Bacteria;k__norank;p__Proteobacteria;c__Gammaproteobacteria;o__Enterobacterales;f__Erwiniaceae;g__Pantoea;s__Pantoea_deleyi
PB1_gene_3093	298	WP_111138472.1	298	293/298(98.32)	1.96e-214	LysR family transcriptional regulator [Pantoea sp. ARC607]	2027922	d__Bacteria;k__norank;p__Proteobacteria;c__Gammaproteobacteria;o__Enterobacterales;f__Erwiniaceae;g__Pantoea;s__Pantoea_sp._ARC607
PB1_gene_3095	225	SKA03853.1	224	196/225(87.11)	1.92e-132	hypothetical protein SAMN03097723_2904 [Pantoea agglomerans]	470933	d__Bacteria;k__norank;p__Proteobacteria;c__Gammaproteobacteria;o__Enterobacterales;f__Erwiniaceae;g__Pantoea;s__Pantoea_eucalypti
PB1_gene_3096	254	KEY44697.1	254	246/253(97.23)	3.53e-170	FklB [Pantoea agglomerans]	549	d__Bacteria;k__norank;p__Proteobacteria;c__Gammaproteobacteria;o__Enterobacterales;f__Erwiniaceae;g__Pantoea;s__Pantoea_agglomerans
PB1_gene_3099	375	ORM78495.1	375	373/375(99.47)	9.77e-272	succinyl-diaminopimelate desuccinylase [Pantoea deleyi]	470932	d__Bacteria;k__norank;p__Proteobacteria;c__Gammaproteobacteria;o__Enterobacterales;f__Erwiniaceae;g__Pantoea;s__Pantoea_deleyi
PB1_gene_3100	62	ORM78494.1	62	62/62(100.00)	7.64e-39	hypothetical protein HA42_16595 [Pantoea deleyi]	470932	d__Bacteria;k__norank;p__Proteobacteria;c__Gammaproteobacteria;o__Enterobacterales;f__Erwiniaceae;g__Pantoea;s__Pantoea_deleyi
PB1_gene_3103	150	ORM79877.1	150	147/150(98.00)	8.58e-104	molybdenum cofactor biosynthesis protein MoaE	470932	d__Bacteria;k__norank;p__Proteobacteria;c__Gammaproteobacteria;o__Enterobacterales;f__Erwiniaceae;g__Pantoea;s__Pantoea_deleyi
PB1_gene_3104	81	ORM79878.1	81	78/81(96.30)	4.95e-48	molybdopterin synthase sulfur carrier subunit [Pantoea deleyi]	470932	d__Bacteria;k__norank;p__Proteobacteria;c__Gammaproteobacteria;o__Enterobacterales;f__Erwiniaceae;g__Pantoea;s__Pantoea_deleyi
PB1_gene_3116	301	WP_050680557.1	301	291/301(96.68)	1.72e-205	tyrosine recombinase XerC [Pantoea vagans]	53335	d__Bacteria;k__norank;p__Proteobacteria;c__Gammaproteobacteria;o__Enterobacterales;f__Erwiniaceae;g__Pantoea;s__norank
PB1_gene_3117	234	ORM81442.1	234	233/234(99.57)	1.11e-163	hypothetical protein HA42_10585 [Pantoea deleyi]	470932	d__Bacteria;k__norank;p__Proteobacteria;c__Gammaproteobacteria;o__Enterobacterales;f__Erwiniaceae;g__Pantoea;s__Pantoea_deleyi
PB1_gene_3118	311	WP_111139818.1	383	302/311(97.11)	9.36e-214	8-amino-7-oxononanoate synthase [Pantoea sp. ARC607]	2027922	d__Bacteria;k__norank;p__Proteobacteria;c__Gammaproteobacteria;o__Enterobacterales;f__Erwiniaceae;g__Pantoea;s__Pantoea_sp._ARC607
PB1_gene_3120	176	ORM82476.1	176	176/176(100.00)	9.94e-127	inorganic pyrophosphatase [Pantoea deleyi]	470932	d__Bacteria;k__norank;p__Proteobacteria;c__Gammaproteobacteria;o__Enterobacterales;f__Erwiniaceae;g__Pantoea;s__Pantoea_deleyi
PB1_gene_3121	149	ORM82475.1	149	147/149(98.66)	1.21e-89	hypothetical protein HA42_08230 [Pantoea deleyi]	470932	d__Bacteria;k__norank;p__Proteobacteria;c__Gammaproteobacteria;o__Enterobacterales;f__Erwiniaceae;g__Pantoea;s__Pantoea_deleyi
PB1_gene_3122	156	ORM82494.1	136	130/136(95.59)	4.54e-84	hypothetical protein HA42_08225 [Pantoea deleyi]	470932	d__Bacteria;k__norank;p__Proteobacteria;c__Gammaproteobacteria;o__Enterobacterales;f__Erwiniaceae;g__Pantoea;s__Pantoea_deleyi
PB1_gene_3128	61	PAW37544.1	60	59/60(98.33)	1.08e-33	DUF4089 domain-containing protein [Pantoea vagans]	470934	d__Bacteria;k__norank;p__Proteobacteria;c__Gammaproteobacteria;o__Enterobacterales;f__Erwiniaceae;g__Pantoea;s__Pantoea_vagans
PB1_gene_3135	150	WP_111141177.1	150	148/150(98.67)	6.01e-95	universal stress protein [Pantoea sp. ARC607]	2027922	d__Bacteria;k__norank;p__Proteobacteria;c__Gammaproteobacteria;o__Enterobacterales;f__Erwiniaceae;g__Pantoea;s__Pantoea_sp._ARC607
PB1_gene_3141	165	ORM83866.1	164	163/164(99.39)	3.27e-115	hypothetical protein HA42_05600 [Pantoea deleyi]	470932	d__Bacteria;k__norank;p__Proteobacteria;c__Gammaproteobacteria;o__Enterobacterales;f__Erwiniaceae;g__Pantoea;s__Pantoea_deleyi
PB1_gene_3142	226	WP_111140470.1	219	207/218(94.95)	9.84e-141	sugar phosphatase [Pantoea sp. ARC607]	2027922	d__Bacteria;k__norank;p__Proteobacteria;c__Gammaproteobacteria;o__Enterobacterales;f__Erwiniaceae;g__Pantoea;s__Pantoea_sp._ARC607
PB1_gene_3145	224	RMS54710.1	357	79/222(35.59)	4.19e-29	hypothetical protein ALP66_05720 [Pseudomonas amygdali pv. photiniae]	251724	d__Bacteria;k__norank;p__Proteobacteria;c__Gammaproteobacteria;o__Pseudomonadales;f__Pseudomonadaceae;g__Pseudomonas;s__Pseudomonas_amygdali
PB1_gene_3146	162	SSL82024.1	348	69/156(44.23)	3.13e-25	Uncharacterised protein [Klebsiella pneumoniae]	573	d__Bacteria;k__norank;p__Proteobacteria;c__Gammaproteobacteria;o__Enterobacterales;f__Enterobacteriaceae;g__Klebsiella;s__Klebsiella_pneumoniae
PB1_gene_3151	312	WP_111141548.1	312	310/312(99.36)	4.27e-216	transcriptional regulator [Pantoea sp. ARC607]	2027922	d__Bacteria;k__norank;p__Proteobacteria;c__Gammaproteobacteria;o__Enterobacterales;f__Erwiniaceae;g__Pantoea;s__Pantoea_sp._ARC607
PB1_gene_3154	376	WP_111141222.1	376	365/376(97.07)	4.12e-269	23S rRNA (uracil(747)-C(5))-methyltransferase RlmC [Pantoea sp. ARC607]	2027922	d__Bacteria;k__norank;p__Proteobacteria;c__Gammaproteobacteria;o__Enterobacterales;f__Erwiniaceae;g__Pantoea;s__Pantoea_sp._ARC607
PB1_gene_3166	371	ORM77700.1	371	368/371(99.19)	3.69e-274	ABC transporter substrate-binding protein [Pantoea deleyi]	470932	d__Bacteria;k__norank;p__Proteobacteria;c__Gammaproteobacteria;o__Enterobacterales;f__Erwiniaceae;g__Pantoea;s__Pantoea_deleyi

PB1_gene_3185	139	ORM83160.1	139	138/139(99.28)	1.78e-93	N utilization substance protein B [Pantoea deleyi]	470932	d__Bacteria;k__norank;p__Proteobacteria;c__Gammaproteobacteria;o__Enterobacterales;f__Erwiniaceae;g__Pantoea;s__Pantoea_deleyi
PB1_gene_3186	325	ORM83159.1	325	319/325(98.15)	5.13e-235	thiamine-phosphate kinase [Pantoea deleyi]	470932	d__Bacteria;k__norank;p__Proteobacteria;c__Gammaproteobacteria;o__Enterobacterales;f__Erwiniaceae;g__Pantoea;s__Pantoea_deleyi
PB1_gene_3189	343	WP_033733735.1	343	306/343(89.21)	7.60e-221	hemin-degrading factor [Pantoea vagans]	470934	d__Bacteria;k__norank;p__Proteobacteria;c__Gammaproteobacteria;o__Enterobacterales;f__Erwiniaceae;g__Pantoea;s__Pantoea_vagans
PB1_gene_3193	243	WP_086905239.1	243	240/243(98.77)	1.24e-169	DNA polymerase III subunit epsilon [Pantoea agglomerans]	549	d__Bacteria;k__norank;p__Proteobacteria;c__Gammaproteobacteria;o__Enterobacterales;f__Erwiniaceae;g__Pantoea;s__Pantoea_agglomerans
PB1_gene_3194	155	ORM78251.1	155	155/155(100.00)	1.58e-115	ribonuclease HI [Pantoea deleyi]	470932	d__Bacteria;k__norank;p__Proteobacteria;c__Gammaproteobacteria;o__Enterobacterales;f__Erwiniaceae;g__Pantoea;s__Pantoea_deleyi
PB1_gene_3200	316	ORM75412.1	316	308/316(97.47)	4.28e-218	LysR family transcriptional regulator [Pantoea deleyi]	470932	d__Bacteria;k__norank;p__Proteobacteria;c__Gammaproteobacteria;o__Enterobacterales;f__Erwiniaceae;g__Pantoea;s__Pantoea_deleyi
PB1_gene_3203	91	ORM72637.1	91	91/91(100.00)	2.78e-54	hypothetical protein HA42_22240 [Pantoea deleyi]	470932	d__Bacteria;k__norank;p__Proteobacteria;c__Gammaproteobacteria;o__Enterobacterales;f__Erwiniaceae;g__Pantoea;s__Pantoea_deleyi
PB1_gene_3204	331	ORM72638.1	367	304/368(82.61)	2.90e-207	nickel/cobalt efflux protein RcnA [Pantoea deleyi]	470932	d__Bacteria;k__norank;p__Proteobacteria;c__Gammaproteobacteria;o__Enterobacterales;f__Erwiniaceae;g__Pantoea;s__Pantoea_deleyi
PB1_gene_3210	131	ORM82490.1	131	131/131(100.00)	2.92e-86	30S ribosomal protein S6 [Pantoea deleyi]	470932	d__Bacteria;k__norank;p__Proteobacteria;c__Gammaproteobacteria;o__Enterobacterales;f__Erwiniaceae;g__Pantoea;s__Pantoea_deleyi
PB1_gene_3212	75	OMK92923.1	75	75/75(100.00)	7.64e-47	30S ribosomal protein S18 [Yersinia pestis subsp. microtus bv. Caucasica]	385966	d__Bacteria;k__norank;p__Proteobacteria;c__Gammaproteobacteria;o__Enterobacterales;f__Yersiniaceae;g__Yersinia;s__Yersinia_pestis
PB1_gene_3216	148	ORM79280.1	148	148/148(100.00)	1.74e-111	hypothetical protein HA42_15690 [Pantoea deleyi]	470932	d__Bacteria;k__norank;p__Proteobacteria;c__Gammaproteobacteria;o__Enterobacterales;f__Erwiniaceae;g__Pantoea;s__Pantoea_deleyi
PB1_gene_3218	159	ELP24266.1	159	140/159(88.05)	1.05e-97	putative acetyltransferase [Pantoea agglomerans 299R]	1261128	d__Bacteria;k__norank;p__Proteobacteria;c__Gammaproteobacteria;o__Enterobacterales;f__Erwiniaceae;g__Pantoea;s__Pantoea_agglomerans
PB1_gene_3219	285	OQV39204.1	285	285/285(100.00)	3.60e-199	RNA polymerase factor sigma-32 [Pantoea vagans]	470934	d__Bacteria;k__norank;p__Proteobacteria;c__Gammaproteobacteria;o__Enterobacterales;f__Erwiniaceae;g__Pantoea;s__Pantoea_vagans
PB1_gene_3221	288	SSK77812.1	93	40/92(43.48)	2.36e-07	Uncharacterised protein [Klebsiella pneumoniae]	573	d__Bacteria;k__norank;p__Proteobacteria;c__Gammaproteobacteria;o__Enterobacterales;f__Enterobacteriaceae;g__Klebsiella;s__Klebsiella_pneumoniae
PB1_gene_3222	562	WP_111142022.1	672	540/562(96.09)	0	NADPH-dependent 2,4-dienoyl-CoA reductase [Pantoea sp.	2027922	d__Bacteria;k__norank;p__Proteobacteria;c__Gammaproteobacteria;o__Enterobacterales;f__Erwiniaceae;g__Pantoea;s__Pantoea_sp._ARC607
PB1_gene_3224	242	ORM81339.1	242	235/242(97.11)	6.19e-174	GMP synthase [Pantoea deleyi]	470932	d__Bacteria;k__norank;p__Proteobacteria;c__Gammaproteobacteria;o__Enterobacterales;f__Erwiniaceae;g__Pantoea;s__Pantoea_deleyi
PB1_gene_3228	80	ORM83156.1	80	80/80(100.00)	7.39e-45	exodeoxyribonuclease VII small subunit [Pantoea deleyi]	470932	d__Bacteria;k__norank;p__Proteobacteria;c__Gammaproteobacteria;o__Enterobacterales;f__Erwiniaceae;g__Pantoea;s__Pantoea_deleyi
PB1_gene_3231	297	ORM81089.1	297	288/297(96.97)	7.70e-216	hypothetical protein HA42_11915 [Pantoea deleyi]	470932	d__Bacteria;k__norank;p__Proteobacteria;c__Gammaproteobacteria;o__Enterobacterales;f__Erwiniaceae;g__Pantoea;s__Pantoea_deleyi
PB1_gene_3232	284	KDA95344.1	295	255/284(89.79)	1.96e-180	membrane protein [Pantoea agglomerans Eh318]	1408177	d__Bacteria;k__norank;p__Proteobacteria;c__Gammaproteobacteria;o__Enterobacterales;f__Erwiniaceae;g__Pantoea;s__Pantoea_agglomerans
PB1_gene_3239	129	ORM75565.1	128	127/128(99.22)	3.32e-84	hypothetical protein HA42_20370 [Pantoea deleyi]	470932	d__Bacteria;k__norank;p__Proteobacteria;c__Gammaproteobacteria;o__Enterobacterales;f__Erwiniaceae;g__Pantoea;s__Pantoea_deleyi
PB1_gene_3240	302	ORM86130.1	302	301/302(99.67)	3.40e-217	UTP--glucose-1-phosphate uridylyltransferase [Pantoea	470932	d__Bacteria;k__norank;p__Proteobacteria;c__Gammaproteobacteria;o__Enterobacterales;f__Erwiniaceae;g__Pantoea;s__Pantoea_deleyi
PB1_gene_3243	85	SJZ68565.1	85	85/85(100.00)	6.84e-50	phosphocarrier protein HPr [Pantoea agglomerans]	470933	d__Bacteria;k__norank;p__Proteobacteria;c__Gammaproteobacteria;o__Enterobacterales;f__Erwiniaceae;g__Pantoea;s__Pantoea_eucalypti
PB1_gene_3247	363	WP_111138570.1	363	362/363(99.72)	6.52e-264	pyrimidine utilization protein A [Pantoea sp. ARC607]	2027922	d__Bacteria;k__norank;p__Proteobacteria;c__Gammaproteobacteria;o__Enterobacterales;f__Erwiniaceae;g__Pantoea;s__Pantoea_sp._ARC607
PB1_gene_3258	218	ORM81160.1	218	215/218(98.62)	9.24e-164	pyridoxamine 5'-phosphate oxidase [Pantoea deleyi]	470932	d__Bacteria;k__norank;p__Proteobacteria;c__Gammaproteobacteria;o__Enterobacterales;f__Erwiniaceae;g__Pantoea;s__Pantoea_deleyi
PB1_gene_3259	142	WP_111141146.1	104	101/104(97.12)	2.17e-67	hypothetical protein [Pantoea sp. ARC607]	2027922	d__Bacteria;k__norank;p__Proteobacteria;c__Gammaproteobacteria;o__Enterobacterales;f__Erwiniaceae;g__Pantoea;s__Pantoea_sp._ARC607

PB1_gene_3260	67	PAW36678.1	67	66/67(98.51)	7.61e-39	hypothetical protein CIL06_13565 [Pantoea vagans]	470934	d__Bacteria;k__norank;p__Proteobacteria;c__Gammaproteobacteria;o__Enterobacterales;f__Erwiniaceae;g__Pantoea;s__Pantoea_vagans
PB1_gene_3265	243	ORM75675.1	243	238/243(97.94)	1.10e-163	3-oxoacyl-ACP reductase [Pantoea deleyi]	470932	d__Bacteria;k__norank;p__Proteobacteria;c__Gammaproteobacteria;o__Enterobacterales;f__Erwiniaceae;g__Pantoea;s__Pantoea_deleyi
PB1_gene_3267	150	ORM86575.1	149	142/148(95.95)	2.03e-104	GNAT family N-acetyltransferase [Pantoea deleyi]	470932	d__Bacteria;k__norank;p__Proteobacteria;c__Gammaproteobacteria;o__Enterobacterales;f__Erwiniaceae;g__Pantoea;s__Pantoea_deleyi
PB1_gene_3268	346	WP_111141760.1	346	343/346(99.13)	3.39e-246	NADP(H)-dependent aldo-keto reductase [Pantoea sp. ARC607]	2027922	d__Bacteria;k__norank;p__Proteobacteria;c__Gammaproteobacteria;o__Enterobacterales;f__Erwiniaceae;g__Pantoea;s__Pantoea_sp._ARC607
PB1_gene_3270	227	WP_111140172.1	227	222/227(97.80)	1.25e-136	VIT family protein [Pantoea sp. ARC607]	2027922	d__Bacteria;k__norank;p__Proteobacteria;c__Gammaproteobacteria;o__Enterobacterales;f__Erwiniaceae;g__Pantoea;s__Pantoea_sp._ARC607
PB1_gene_3272	221	ORM75523.1	221	221/221(100.00)	2.13e-125	phage shock protein PspA [Pantoea deleyi]	470932	d__Bacteria;k__norank;p__Proteobacteria;c__Gammaproteobacteria;o__Enterobacterales;f__Erwiniaceae;g__Pantoea;s__Pantoea_deleyi
PB1_gene_3273	75	ORM75522.1	75	72/75(96.00)	1.01e-42	phage shock protein B [Pantoea deleyi]	470932	d__Bacteria;k__norank;p__Proteobacteria;c__Gammaproteobacteria;o__Enterobacterales;f__Erwiniaceae;g__Pantoea;s__Pantoea_deleyi
PB1_gene_3274	117	ORM75524.1	116	115/116(99.14)	7.02e-69	DNA-binding transcriptional activator PspC [Pantoea deleyi]	470932	d__Bacteria;k__norank;p__Proteobacteria;c__Gammaproteobacteria;o__Enterobacterales;f__Erwiniaceae;g__Pantoea;s__Pantoea_deleyi
PB1_gene_3276	498	ORM83145.1	496	491/496(98.99)	0	muropeptide transporter AmpG [Pantoea deleyi]	470932	d__Bacteria;k__norank;p__Proteobacteria;c__Gammaproteobacteria;o__Enterobacterales;f__Erwiniaceae;g__Pantoea;s__Pantoea_deleyi
PB1_gene_3284	76	WP_111140901.1	76	75/76(98.68)	4.68e-55	hypothetical protein [Pantoea sp. ARC607]	2027922	d__Bacteria;k__norank;p__Proteobacteria;c__Gammaproteobacteria;o__Enterobacterales;f__Erwiniaceae;g__Pantoea;s__Pantoea_sp._ARC607
PB1_gene_3285	216	OQV38510.1	216	215/216(99.54)	1.58e-145	CPBP family intramembrane metalloprotease [Pantoea vagans]	470934	d__Bacteria;k__norank;p__Proteobacteria;c__Gammaproteobacteria;o__Enterobacterales;f__Erwiniaceae;g__Pantoea;s__Pantoea_vagans
PB1_gene_3288	131	WP_111140250.1	130	129/130(99.23)	4.16e-79	siroheme synthase [Pantoea sp. ARC607]	2027922	d__Bacteria;k__norank;p__Proteobacteria;c__Gammaproteobacteria;o__Enterobacterales;f__Erwiniaceae;g__Pantoea;s__Pantoea_sp._ARC607
PB1_gene_3289	284	WP_111140251.1	284	269/284(94.72)	1.40e-191	peptide chain release factor N(5)-glutamine methyltransferase [Pantoea sp. ARC607]	2027922	d__Bacteria;k__norank;p__Proteobacteria;c__Gammaproteobacteria;o__Enterobacterales;f__Erwiniaceae;g__Pantoea;s__Pantoea_sp._ARC607
PB1_gene_3291	563	PBQ27256.1	522	453/506(89.53)	5.83e-303	hypothetical protein BTO27_03640, partial	2049495	d__Bacteria;k__norank;p__Proteobacteria;c__Alphaproteobacteria;o__Rickettsiales;f__Anaplasmataceae;g__Wolbachia;s__Wolbachia_pipientis
PB1_gene_3293	157	PQL26978.1	157	156/157(99.36)	1.35e-108	transcription elongation factor GreB [Pantoea ananatis]	553	d__Bacteria;k__norank;p__Proteobacteria;c__Gammaproteobacteria;o__Enterobacterales;f__Erwiniaceae;g__Pantoea;s__Pantoea_ananatis
PB1_gene_3297	262	ORM82564.1	262	253/262(96.56)	5.45e-178	trehalose-phosphatase [Pantoea deleyi]	470932	d__Bacteria;k__norank;p__Proteobacteria;c__Gammaproteobacteria;o__Enterobacterales;f__Erwiniaceae;g__Pantoea;s__Pantoea_deleyi
PB1_gene_3299	189	ORM80911.1	189	189/189(100.00)	8.60e-123	polyketide synthase [Pantoea deleyi]	470932	d__Bacteria;k__norank;p__Proteobacteria;c__Gammaproteobacteria;o__Enterobacterales;f__Erwiniaceae;g__Pantoea;s__Pantoea_deleyi
PB1_gene_3300	171	ORM80910.1	171	166/170(97.65)	4.73e-109	fimbrial protein [Pantoea deleyi]	470932	d__Bacteria;k__norank;p__Proteobacteria;c__Gammaproteobacteria;o__Enterobacterales;f__Erwiniaceae;g__Pantoea;s__Pantoea_deleyi
PB1_gene_3308	355	ORM83253.1	355	348/355(98.03)	2.02e-263	NAD-dependent dehydratase [Pantoea deleyi]	470932	d__Bacteria;k__norank;p__Proteobacteria;c__Gammaproteobacteria;o__Enterobacterales;f__Erwiniaceae;g__Pantoea;s__Pantoea_deleyi
PB1_gene_3311	132	ORM81506.1	344	131/132(99.24)	8.06e-89	LacI family transcriptional regulator [Pantoea deleyi]	470932	d__Bacteria;k__norank;p__Proteobacteria;c__Gammaproteobacteria;o__Enterobacterales;f__Erwiniaceae;g__Pantoea;s__Pantoea_deleyi
PB1_gene_3312	176	ORM81506.1	344	157/161(97.52)	6.57e-103	LacI family transcriptional regulator [Pantoea deleyi]	470932	d__Bacteria;k__norank;p__Proteobacteria;c__Gammaproteobacteria;o__Enterobacterales;f__Erwiniaceae;g__Pantoea;s__Pantoea_deleyi
PB1_gene_3314	274	ORM81552.1	310	273/274(99.64)	3.13e-203	ribosomal protein L3 N(5)-glutamine methyltransferase	470932	d__Bacteria;k__norank;p__Proteobacteria;c__Gammaproteobacteria;o__Enterobacterales;f__Erwiniaceae;g__Pantoea;s__Pantoea_deleyi
PB1_gene_3316	148	WP_111141907.1	148	145/148(97.97)	7.09e-102	flavodoxin [Pantoea sp. ARC607]	2027922	d__Bacteria;k__norank;p__Proteobacteria;c__Gammaproteobacteria;o__Enterobacterales;f__Erwiniaceae;g__Pantoea;s__Pantoea_sp._ARC607
PB1_gene_3318	226	ORM78493.1	226	221/226(97.79)	4.53e-159	esterase [Pantoea deleyi]	470932	d__Bacteria;k__norank;p__Proteobacteria;c__Gammaproteobacteria;o__Enterobacterales;f__Erwiniaceae;g__Pantoea;s__Pantoea_deleyi

PB1_gene_3319	351	WP_111139336.1	662	320/351(91.17)	3.91e-212	tRNA(Met) cytidine acetyltransferase [Pantoea sp. ARC607]	2027922	d__Bacteria;k__norank;p__Proteobacteria;c__Gammaproteobacteria;o__Enterobacterales;f__Erwiniaceae;g__Pantoea;s__Pantoea_sp._ARC607
PB1_gene_3320	493	WP_125289999.1	459	381/465(81.94)	2.14e-262	purine permease [Erwinia sp. ARC607]	None	Unclassified
PB1_gene_3323	147	ORM75483.1	147	144/147(97.96)	4.81e-104	cellulose synthase [Pantoea deleyi]	470932	d__Bacteria;k__norank;p__Proteobacteria;c__Gammaproteobacteria;o__Enterobacterales;f__Erwiniaceae;g__Pantoea;s__Pantoea_deleyi
PB1_gene_3326	424	ORM78402.1	424	421/424(99.29)	1.29e-310	glutamate dehydrogenase [Pantoea deleyi]	470932	d__Bacteria;k__norank;p__Proteobacteria;c__Gammaproteobacteria;o__Enterobacterales;f__Erwiniaceae;g__Pantoea;s__Pantoea_deleyi
PB1_gene_3331	423	CAQ54658.1	423	419/423(99.05)	3.00e-304	phosphoribosylamine--glycine ligase [Wolbachia endosymbiont of Culex quinquefasciatus Pel]	570417	d__Bacteria;k__norank;p__Proteobacteria;c__Alphaproteobacteria;o__Rickettsiales;f__Anaplasmataceae;g__Wolbachia;s__Wolbachia_endosymbiont_of_Culex_quinquefasciatus
PB1_gene_3333	176	KMV36493.1	169	62/152(40.79)	2.18e-29	fimbrial protein [Franconibacter pulveris]	435910	d__Bacteria;k__norank;p__Proteobacteria;c__Gammaproteobacteria;o__Enterobacterales;f__Enterobacteriaceae;g__Franconibacter;s__Franconibacter_pulveris
PB1_gene_3335	252	VEA61591.1	254	141/248(56.85)	1.95e-92	Chaperone protein papD precursor [Serratia plymuthica]	82996	d__Bacteria;k__norank;p__Proteobacteria;c__Gammaproteobacteria;o__Enterobacterales;f__Yersiniaceae;g__Serratia;s__Serratia_plymuthica
PB1_gene_3337	402	ORM80494.1	402	387/402(96.27)	1.27e-283	cysteine sulfinate desulfinase [Pantoea deleyi]	470932	d__Bacteria;k__norank;p__Proteobacteria;c__Gammaproteobacteria;o__Enterobacterales;f__Erwiniaceae;g__Pantoea;s__Pantoea_deleyi
PB1_gene_3341	67	KXN45458.1	99	50/67(74.63)	9.58e-23	hypothetical protein AUS42_27445 [Escherichia coli]	562	d__Bacteria;k__norank;p__Proteobacteria;c__Gammaproteobacteria;o__Enterobacterales;f__Enterobacteriaceae;g__Escherichia;s__Escherichia_coli
PB1_gene_3344	97	ORM81770.1	96	95/96(98.96)	1.34e-59	lipoprotein bor [Pantoea deleyi]	470932	d__Bacteria;k__norank;p__Proteobacteria;c__Gammaproteobacteria;o__Enterobacterales;f__Erwiniaceae;g__Pantoea;s__Pantoea_deleyi
PB1_gene_3345	79	ORM81166.1	79	77/79(97.47)	6.10e-48	two-component-system connector protein YcgZ [Pantoea deleyi]	470932	d__Bacteria;k__norank;p__Proteobacteria;c__Gammaproteobacteria;o__Enterobacterales;f__Erwiniaceae;g__Pantoea;s__Pantoea_deleyi
PB1_gene_3346	212	ORM81167.1	212	206/212(97.17)	2.93e-139	transcriptional regulator [Pantoea deleyi]	470932	d__Bacteria;k__norank;p__Proteobacteria;c__Gammaproteobacteria;o__Enterobacterales;f__Erwiniaceae;g__Pantoea;s__Pantoea_deleyi
PB1_gene_3352	116	KDA93306.1	95	87/94(92.55)	2.15e-54	DNA polymerase III subunit theta [Pantoea agglomerans Eh318]	1408177	d__Bacteria;k__norank;p__Proteobacteria;c__Gammaproteobacteria;o__Enterobacterales;f__Erwiniaceae;g__Pantoea;s__Pantoea_agglomerans
PB1_gene_3358	363	SJZ41026.1	363	362/363(99.72)	6.99e-259	hypothetical protein SAMN03097723_0834 [Pantoea agglomerans]	470933	d__Bacteria;k__norank;p__Proteobacteria;c__Gammaproteobacteria;o__Enterobacterales;f__Erwiniaceae;g__Pantoea;s__Pantoea_eucalypti
PB1_gene_3363	128	ORM82565.1	117	116/117(99.15)	6.10e-73	flagellar transcriptional activator FlhD [Pantoea deleyi]	470932	d__Bacteria;k__norank;p__Proteobacteria;c__Gammaproteobacteria;o__Enterobacterales;f__Erwiniaceae;g__Pantoea;s__Pantoea_deleyi
PB1_gene_3364	192	ORM82566.1	192	192/192(100.00)	4.29e-138	transcriptional regulator FlhC [Pantoea deleyi]	470932	d__Bacteria;k__norank;p__Proteobacteria;c__Gammaproteobacteria;o__Enterobacterales;f__Erwiniaceae;g__Pantoea;s__Pantoea_deleyi
PB1_gene_3375	403	ORM76740.1	403	388/403(96.28)	3.60e-279	diguanylate phosphodiesterase [Pantoea deleyi]	470932	d__Bacteria;k__norank;p__Proteobacteria;c__Gammaproteobacteria;o__Enterobacterales;f__Erwiniaceae;g__Pantoea;s__Pantoea_deleyi
PB1_gene_3376	77	ORM76739.1	77	77/77(100.00)	8.81e-47	hypothetical protein HA42_18750 [Pantoea deleyi]	470932	d__Bacteria;k__norank;p__Proteobacteria;c__Gammaproteobacteria;o__Enterobacterales;f__Erwiniaceae;g__Pantoea;s__Pantoea_deleyi
PB1_gene_3377	201	OQV38911.1	201	199/201(99.00)	6.80e-144	3-isopropylmalate dehydratase small subunit [Pantoea vagans]	470934	d__Bacteria;k__norank;p__Proteobacteria;c__Gammaproteobacteria;o__Enterobacterales;f__Erwiniaceae;g__Pantoea;s__Pantoea_vagans
PB1_gene_3384	459	ORM79295.1	459	447/459(97.39)	0	aminodeoxychorismate synthase, component I [Pantoea deleyi]	470932	d__Bacteria;k__norank;p__Proteobacteria;c__Gammaproteobacteria;o__Enterobacterales;f__Erwiniaceae;g__Pantoea;s__Pantoea_deleyi
PB1_gene_3387	356	WP_090966414.1	356	336/355(94.65)	1.24e-253	dTDP-glucose 4,6-dehydratase [Pantoea sp. OV426]	1881036	d__Bacteria;k__norank;p__Proteobacteria;c__Gammaproteobacteria;o__Enterobacterales;f__Erwiniaceae;g__Pantoea;s__Pantoea_sp._OV426
PB1_gene_3390	261	ORM83624.1	259	246/258(95.35)	5.27e-159	iron export ABC transporter permease subunit FetB [Pantoea deleyi]	470932	d__Bacteria;k__norank;p__Proteobacteria;c__Gammaproteobacteria;o__Enterobacterales;f__Erwiniaceae;g__Pantoea;s__Pantoea_deleyi
PB1_gene_3391	103	ORM83597.1	103	99/103(96.12)	2.21e-70	hypothetical protein HA42_06000 [Pantoea deleyi]	470932	d__Bacteria;k__norank;p__Proteobacteria;c__Gammaproteobacteria;o__Enterobacterales;f__Erwiniaceae;g__Pantoea;s__Pantoea_deleyi
PB1_gene_3393	235	ORM82920.1	235	228/235(97.02)	1.63e-161	MFS transporter [Pantoea deleyi]	470932	d__Bacteria;k__norank;p__Proteobacteria;c__Gammaproteobacteria;o__Enterobacterales;f__Erwiniaceae;g__Pantoea;s__Pantoea_deleyi

PB1_gene_3394	194	SST11316.1	264	79/151(52.32)	1.26e-46	Uncharacterised protein [Acinetobacter baumannii]	470	d__Bacteria;k__norank;p__Proteobacteria;c__Gammaproteobacteria;o__Pseudomonadales;f__Moraxellaceae;g__Acinetobacter;s__Acinetobacter_baumannii
PB1_gene_3396	188	ORM80689.1	188	185/188(98.40)	8.04e-132	DNA endonuclease [Pantoea deleyi]	470932	d__Bacteria;k__norank;p__Proteobacteria;c__Gammaproteobacteria;o__Enterobacterales;f__Erwiniaceae;g__Pantoea;s__Pantoea_deleyi
PB1_gene_3397	75	ORM80690.1	75	62/75(82.67)	1.19e-32	hypothetical protein HA42_13455 [Pantoea deleyi]	470932	d__Bacteria;k__norank;p__Proteobacteria;c__Gammaproteobacteria;o__Enterobacterales;f__Erwiniaceae;g__Pantoea;s__Pantoea_deleyi
PB1_gene_3399	258	ORM77140.1	258	257/258(99.61)	3.06e-182	transcriptional regulator ExuR [Pantoea deleyi]	470932	d__Bacteria;k__norank;p__Proteobacteria;c__Gammaproteobacteria;o__Enterobacterales;f__Erwiniaceae;g__Pantoea;s__Pantoea_deleyi
PB1_gene_3401	378	ORM80457.1	378	372/377(98.67)	1.61e-267	type VI secretion system, core protein [Pantoea deleyi]	470932	d__Bacteria;k__norank;p__Proteobacteria;c__Gammaproteobacteria;o__Enterobacterales;f__Erwiniaceae;g__Pantoea;s__Pantoea_deleyi
PB1_gene_3403	131	ORM78405.1	131	126/131(96.18)	1.34e-82	hypothetical protein HA42_16685 [Pantoea deleyi]	470932	d__Bacteria;k__norank;p__Proteobacteria;c__Gammaproteobacteria;o__Enterobacterales;f__Erwiniaceae;g__Pantoea;s__Pantoea_deleyi
PB1_gene_3404	157	WP_111138414.1	157	152/157(96.82)	1.71e-111	nuclear transport factor 2 family protein [Pantoea sp. ARC607]	2027922	d__Bacteria;k__norank;p__Proteobacteria;c__Gammaproteobacteria;o__Enterobacterales;f__Erwiniaceae;g__Pantoea;s__Pantoea_sp._ARC607
PB1_gene_3405	144	ORM78407.1	144	143/144(99.31)	2.05e-99	riboflavin synthase subunit beta [Pantoea deleyi]	470932	d__Bacteria;k__norank;p__Proteobacteria;c__Gammaproteobacteria;o__Enterobacterales;f__Erwiniaceae;g__Pantoea;s__Pantoea_deleyi
PB1_gene_3409	98	WP_079942172.1	102	54/101(53.47)	3.61e-29	hypothetical protein [Salmonella enterica]	28901	d__Bacteria;k__norank;p__Proteobacteria;c__Gammaproteobacteria;o__Enterobacterales;f__Enterobacteriaceae;g__Salmonella;s__Salmonella_enterica
PB1_gene_3411	318	ORM77354.1	318	317/318(99.69)	1.28e-220	sugar ABC transporter substrate-binding protein [Pantoea deleyi]	470932	d__Bacteria;k__norank;p__Proteobacteria;c__Gammaproteobacteria;o__Enterobacterales;f__Erwiniaceae;g__Pantoea;s__Pantoea_deleyi
PB1_gene_3414	43	WP_111139813.1	272	27/31(87.10)	2.58e-08	pyridoxal phosphatase [Pantoea sp. ARC607]	2027922	d__Bacteria;k__norank;p__Proteobacteria;c__Gammaproteobacteria;o__Enterobacterales;f__Erwiniaceae;g__Pantoea;s__Pantoea_sp._ARC607
PB1_gene_3415	210	WP_061062242.1	210	186/210(88.57)	1.56e-133	hypothetical protein [Pantoea vagans]	470934	d__Bacteria;k__norank;p__Proteobacteria;c__Gammaproteobacteria;o__Enterobacterales;f__Erwiniaceae;g__Pantoea;s__Pantoea_vagans
PB1_gene_3417	220	ORM79507.1	270	209/220(95.00)	2.43e-149	inositol monophosphatase [Pantoea deleyi]	470932	d__Bacteria;k__norank;p__Proteobacteria;c__Gammaproteobacteria;o__Enterobacterales;f__Erwiniaceae;g__Pantoea;s__Pantoea_deleyi
PB1_gene_3418	193	ORM79508.1	193	186/193(96.37)	1.23e-133	ACP phosphodiesterase [Pantoea deleyi]	470932	d__Bacteria;k__norank;p__Proteobacteria;c__Gammaproteobacteria;o__Enterobacterales;f__Erwiniaceae;g__Pantoea;s__Pantoea_deleyi
PB1_gene_3422	139	WP_125364749.1	149	135/139(97.12)	8.39e-92	type VI secretion system baseplate subunit TssE	550	d__Bacteria;k__norank;p__Proteobacteria;c__Gammaproteobacteria;o__Enterobacterales;f__Enterobacteriaceae;g__Enterobacter;s__Enterobacter_cloacae
PB1_gene_3423	156	WP_126534691.1	156	147/156(94.23)	1.51e-100	hypothetical protein [Enterobacter sp. WCHEn090040]	2497436	d__Bacteria;k__norank;p__Proteobacteria;c__Gammaproteobacteria;o__Enterobacterales;f__Enterobacteriaceae;g__Enterobacter;s__Enterobacter_sp._WCHEn090040
PB1_gene_3425	289	ORM85855.1	289	286/289(98.96)	2.65e-203	transcriptional regulator [Pantoea deleyi]	470932	d__Bacteria;k__norank;p__Proteobacteria;c__Gammaproteobacteria;o__Enterobacterales;f__Erwiniaceae;g__Pantoea;s__Pantoea_deleyi
PB1_gene_3427	261	KEY42230.1	253	230/253(90.91)	2.05e-166	queuosine biosynthesis protein QueC [Pantoea agglomerans]	549	d__Bacteria;k__norank;p__Proteobacteria;c__Gammaproteobacteria;o__Enterobacterales;f__Erwiniaceae;g__Pantoea;s__Pantoea_agglomerans
PB1_gene_3428	134	ORM83162.1	132	130/131(99.24)	3.28e-84	thioesterase [Pantoea deleyi]	470932	d__Bacteria;k__norank;p__Proteobacteria;c__Gammaproteobacteria;o__Enterobacterales;f__Erwiniaceae;g__Pantoea;s__Pantoea_deleyi
PB1_gene_3434	577	WP_111139514.1	1320	489/582(84.02)	0	BapA prefix-like domain-containing protein [Pantoea sp.	2027922	d__Bacteria;k__norank;p__Proteobacteria;c__Gammaproteobacteria;o__Enterobacterales;f__Erwiniaceae;g__Pantoea;s__Pantoea_sp._ARC607
PB1_gene_3439	189	KDA95604.1	222	174/189(92.06)	4.46e-128	type IV secretion protein Rhs [Pantoea agglomerans Eh318]	1408177	d__Bacteria;k__norank;p__Proteobacteria;c__Gammaproteobacteria;o__Enterobacterales;f__Erwiniaceae;g__Pantoea;s__Pantoea_agglomerans
PB1_gene_3440	51	WP_041474165.1	89	34/51(66.67)	9.16e-10	type I addiction module toxin, SymE family [Erwinia pyrifoliae]	79967	d__Bacteria;k__norank;p__Proteobacteria;c__Gammaproteobacteria;o__Enterobacterales;f__Erwiniaceae;g__Erwinia;s__Erwinia_pyrifoliae
PB1_gene_3444	87	WP_049090784.1	74	27/55(49.09)	8.41e-08	hypothetical protein [Klebsiella oxytoca]	571	d__Bacteria;k__norank;p__Proteobacteria;c__Gammaproteobacteria;o__Enterobacterales;f__Enterobacteriaceae;g__Klebsiella;s__Klebsiella_oxytoca
PB1_gene_3446	109	WP_126690644.1	109	73/103(70.87)	4.27e-46	hypothetical protein [Pantoea sp. YU22]	None	Unclassified
PB1_gene_3447	128	KFC12705.1	113	51/111(45.95)	5.85e-26	phage protein [Trabulsiella guamensis ATCC 49490]	1005994	d__Bacteria;k__norank;p__Proteobacteria;c__Gammaproteobacteria;o__Enterobacterales;f__Enterobacteriaceae;g__Trabulsiella;s__Trabulsiella_guamensis

PB1_gene_3448	414	WP_094106333.1	516	402/414(97.10)	1.63e-278	chromosome partition protein MukB, partial [Pantoea brenneri]	472694	d__Bacteria;k__norank;p__Proteobacteria;c__Gammaproteobacteria;o__Enterobacterales;f__Erwiniaceae;g__Pantoea;s__Pantoea_brenneri
PB1_gene_3451	173	WP_111138493.1	237	162/172(94.19)	7.44e-117	N-acetyltransferase [Pantoea sp. ARC607]	2027922	d__Bacteria;k__norank;p__Proteobacteria;c__Gammaproteobacteria;o__Enterobacterales;f__Erwiniaceae;g__Pantoea;s__Pantoea_sp._ARC607
PB1_gene_3452	189	WP_128086358.1	266	42/50(84.00)	9.25e-11	hypothetical protein [Pantoea deleyi]	470932	d__Bacteria;k__norank;p__Proteobacteria;c__Gammaproteobacteria;o__Enterobacterales;f__Erwiniaceae;g__Pantoea;s__Pantoea_deleyi
PB1_gene_3456	253	WP_111139098.1	253	247/253(97.63)	3.41e-172	2-dehydro-3-deoxy-D-gluconate 5-dehydrogenase KduD [Pantoea sp. ARC607]	2027922	d__Bacteria;k__norank;p__Proteobacteria;c__Gammaproteobacteria;o__Enterobacterales;f__Erwiniaceae;g__Pantoea;s__Pantoea_sp._ARC607
PB1_gene_3460	463	WP_111139506.1	463	457/463(98.70)	4.21e-313	amino acid permease [Pantoea sp. ARC607]	2027922	d__Bacteria;k__norank;p__Proteobacteria;c__Gammaproteobacteria;o__Enterobacterales;f__Erwiniaceae;g__Pantoea;s__Pantoea_sp._ARC607
PB1_gene_3465	161	ORM85068.1	161	159/161(98.76)	6.12e-107	flagellar basal body-associated protein FliL [Pantoea deleyi]	470932	d__Bacteria;k__norank;p__Proteobacteria;c__Gammaproteobacteria;o__Enterobacterales;f__Erwiniaceae;g__Pantoea;s__Pantoea_deleyi
PB1_gene_3476	114	ORM75314.1	114	114/114(100.00)	1.43e-69	hypothetical protein HA42_21080 [Pantoea deleyi]	470932	d__Bacteria;k__norank;p__Proteobacteria;c__Gammaproteobacteria;o__Enterobacterales;f__Erwiniaceae;g__Pantoea;s__Pantoea_deleyi
PB1_gene_3479	320	ORM83264.1	297	292/297(98.32)	8.92e-196	hypothetical protein HA42_06680 [Pantoea deleyi]	470932	d__Bacteria;k__norank;p__Proteobacteria;c__Gammaproteobacteria;o__Enterobacterales;f__Erwiniaceae;g__Pantoea;s__Pantoea_deleyi
PB1_gene_3481	126	ORM85090.1	126	123/126(97.62)	5.12e-80	ribosomal protein P2 [Pantoea deleyi]	470932	d__Bacteria;k__norank;p__Proteobacteria;c__Gammaproteobacteria;o__Enterobacterales;f__Erwiniaceae;g__Pantoea;s__Pantoea_deleyi
PB1_gene_3482	86	ORM85089.1	86	80/86(93.02)	1.18e-46	hypothetical protein HA42_03470 [Pantoea deleyi]	470932	d__Bacteria;k__norank;p__Proteobacteria;c__Gammaproteobacteria;o__Enterobacterales;f__Erwiniaceae;g__Pantoea;s__Pantoea_deleyi
PB1_gene_3483	83	WP_111141196.1	84	82/84(97.62)	1.23e-49	LysM peptidoglycan-binding domain-containing protein [Pantoea sp. ARC607]	2027922	d__Bacteria;k__norank;p__Proteobacteria;c__Gammaproteobacteria;o__Enterobacterales;f__Erwiniaceae;g__Pantoea;s__Pantoea_sp._ARC607
PB1_gene_3484	54	AAX65913.1	104	27/27(100.00)	1.69e-08	hypothetical protein SCH_2007 [Salmonella enterica subsp. enterica serovar Choleraesuis str.	321314	d__Bacteria;k__norank;p__Proteobacteria;c__Gammaproteobacteria;o__Enterobacterales;f__Enterobacteriaceae;g__Salmonella;s__Salmonella_enterica
PB1_gene_3485	124	OON39288.1	124	124/124(100.00)	6.78e-84	30S ribosomal protein S12 [Izhakiella australiensis]	1926881	d__Bacteria;k__norank;p__Proteobacteria;c__Gammaproteobacteria;o__Enterobacterales;f__Erwiniaceae;g__Izhakiella;s__Izhakiella_australiensis
PB1_gene_3486	95	ORM81811.1	95	91/95(95.79)	2.72e-56	sulfurtransferase TusB [Pantoea deleyi]	470932	d__Bacteria;k__norank;p__Proteobacteria;c__Gammaproteobacteria;o__Enterobacterales;f__Erwiniaceae;g__Pantoea;s__Pantoea_deleyi
PB1_gene_3487	119	WP_111139693.1	119	114/119(95.80)	7.78e-74	sulfurtransferase complex subunit TusC [Pantoea sp. ARC607]	2027922	d__Bacteria;k__norank;p__Proteobacteria;c__Gammaproteobacteria;o__Enterobacterales;f__Erwiniaceae;g__Pantoea;s__Pantoea_sp._ARC607
PB1_gene_3488	128	WP_111139694.1	128	124/128(96.88)	5.29e-83	sulfurtransferase complex subunit TusD [Pantoea sp. ARC607]	2027922	d__Bacteria;k__norank;p__Proteobacteria;c__Gammaproteobacteria;o__Enterobacterales;f__Erwiniaceae;g__Pantoea;s__Pantoea_sp._ARC607
PB1_gene_3493	206	ORM72643.1	206	199/206(96.60)	1.11e-131	protein phosphatase CheZ [Pantoea deleyi]	470932	d__Bacteria;k__norank;p__Proteobacteria;c__Gammaproteobacteria;o__Enterobacterales;f__Erwiniaceae;g__Pantoea;s__Pantoea_deleyi
PB1_gene_3497	303	ORM75500.1	303	302/303(99.67)	3.94e-212	recombination-associated protein RdgC [Pantoea deleyi]	470932	d__Bacteria;k__norank;p__Proteobacteria;c__Gammaproteobacteria;o__Enterobacterales;f__Erwiniaceae;g__Pantoea;s__Pantoea_deleyi
PB1_gene_3500	567	WP_120457444.1	3283	460/570(80.70)	1.17e-256	filamentous hemagglutinin N-terminal domain-containing protein [Erwiniaceae bacterium	2364647	d__Bacteria;k__norank;p__Proteobacteria;c__Gammaproteobacteria;o__Enterobacterales;f__Erwiniaceae;g__Kalamiella;s__Kalamiella_piersonii
PB1_gene_3504	353	ORM86308.1	353	352/353(99.72)	2.78e-244	outer membrane-stress sensor serine endopeptidase DegS [Pantoea deleyi]	470932	d__Bacteria;k__norank;p__Proteobacteria;c__Gammaproteobacteria;o__Enterobacterales;f__Erwiniaceae;g__Pantoea;s__Pantoea_deleyi
PB1_gene_3505	271	ORM86329.1	271	271/271(100.00)	2.83e-189	ABC transporter ATP-binding protein [Pantoea deleyi]	470932	d__Bacteria;k__norank;p__Proteobacteria;c__Gammaproteobacteria;o__Enterobacterales;f__Erwiniaceae;g__Pantoea;s__Pantoea_deleyi
PB1_gene_3513	457	WP_111138451.1	549	453/457(99.12)	0	malate dehydrogenase (quinone) [Pantoea sp. ARC607]	2027922	d__Bacteria;k__norank;p__Proteobacteria;c__Gammaproteobacteria;o__Enterobacterales;f__Erwiniaceae;g__Pantoea;s__Pantoea_sp._ARC607
PB1_gene_3519	358	ORM85653.1	358	355/358(99.16)	1.36e-258	histidinol-phosphate transaminase [Pantoea deleyi]	470932	d__Bacteria;k__norank;p__Proteobacteria;c__Gammaproteobacteria;o__Enterobacterales;f__Erwiniaceae;g__Pantoea;s__Pantoea_deleyi

PB1_gene_3522	275	ORM76691.1	275	270/275(98.18)	6.40e-188	transcriptional regulator IclR [Pantoea deleyi]	470932	d__Bacteria;k__norank;p__Proteobacteria;c__Gammaproteobacteria;o__Enterobacterales;f__Erwiniaceae;g__Pantoea;s__Pantoea_deleyi
PB1_gene_3526	220	RYE05569.1	215	137/213(64.32)	1.04e-98	chromosome partitioning protein ParA [Rickettsiaceae bacterium]	2023360	d__Bacteria;k__norank;p__Proteobacteria;c__Alphaproteobacteria;o__Rickettsiales;f__Rickettsiaceae;g__norank;s__Rickettsiaceae_bacterium
PB1_gene_3529	261	ADO09423.1	278	236/261(90.42)	1.20e-164	CBS domain-containing membrane protein [Pantoea	712898	d__Bacteria;k__norank;p__Proteobacteria;c__Gammaproteobacteria;o__Enterobacterales;f__Erwiniaceae;g__Pantoea;s__Pantoea_vagans
PB1_gene_3532	44	ORM81753.1	300	41/44(93.18)	4.66e-19	serine dehydratase [Pantoea deleyi]	470932	d__Bacteria;k__norank;p__Proteobacteria;c__Gammaproteobacteria;o__Enterobacterales;f__Erwiniaceae;g__Pantoea;s__Pantoea_deleyi
PB1_gene_3534	90	SKA25667.1	90	90/90(100.00)	4.31e-53	bacterial nucleoid protein HU alpha subunit [Pantoea	470933	d__Bacteria;k__norank;p__Proteobacteria;c__Gammaproteobacteria;o__Enterobacterales;f__Erwiniaceae;g__Pantoea;s__Pantoea_eucalypti
PB1_gene_3535	196	ORM80952.1	196	196/196(100.00)	4.11e-138	hypothetical protein HA42_12445 [Pantoea deleyi]	470932	d__Bacteria;k__norank;p__Proteobacteria;c__Gammaproteobacteria;o__Enterobacterales;f__Erwiniaceae;g__Pantoea;s__Pantoea_deleyi
PB1_gene_3547	427	PQL29315.1	427	419/427(98.13)	9.83e-316	long-chain fatty acid transporter FadL [Pantoea ananatis]	553	d__Bacteria;k__norank;p__Proteobacteria;c__Gammaproteobacteria;o__Enterobacterales;f__Erwiniaceae;g__Pantoea;s__Pantoea_ananatis
PB1_gene_3549	281	ORM81976.1	281	279/281(99.29)	7.79e-192	putrescine ABC transporter permease PotI [Pantoea deleyi]	470932	d__Bacteria;k__norank;p__Proteobacteria;c__Gammaproteobacteria;o__Enterobacterales;f__Erwiniaceae;g__Pantoea;s__Pantoea_deleyi
PB1_gene_3554	351	ORM80425.1	351	349/351(99.43)	1.98e-252	DNA polymerase IV [Pantoea deleyi]	470932	d__Bacteria;k__norank;p__Proteobacteria;c__Gammaproteobacteria;o__Enterobacterales;f__Erwiniaceae;g__Pantoea;s__Pantoea_deleyi
PB1_gene_3555	157	ORM81274.1	157	155/157(98.73)	2.24e-107	hypothetical protein HA42_11450 [Pantoea deleyi]	470932	d__Bacteria;k__norank;p__Proteobacteria;c__Gammaproteobacteria;o__Enterobacterales;f__Erwiniaceae;g__Pantoea;s__Pantoea_deleyi
PB1_gene_3556	181	ORM81275.1	181	176/181(97.24)	9.62e-107	hypothetical protein HA42_11455 [Pantoea deleyi]	470932	d__Bacteria;k__norank;p__Proteobacteria;c__Gammaproteobacteria;o__Enterobacterales;f__Erwiniaceae;g__Pantoea;s__Pantoea_deleyi
PB1_gene_3559	314	ORM82568.1	314	312/314(99.36)	7.39e-218	flagellar motor protein MotB [Pantoea deleyi]	470932	d__Bacteria;k__norank;p__Proteobacteria;c__Gammaproteobacteria;o__Enterobacterales;f__Erwiniaceae;g__Pantoea;s__Pantoea_deleyi
PB1_gene_3562	249	ORM80799.1	249	237/249(95.18)	3.82e-167	vitamin B12 ABC transporter ATP-binding protein BtuD [Pantoea deleyi]	470932	d__Bacteria;k__norank;p__Proteobacteria;c__Gammaproteobacteria;o__Enterobacterales;f__Erwiniaceae;g__Pantoea;s__Pantoea_deleyi
PB1_gene_3563	152	ORM80798.1	152	152/152(100.00)	1.86e-108	endopeptidase [Pantoea deleyi]	470932	d__Bacteria;k__norank;p__Proteobacteria;c__Gammaproteobacteria;o__Enterobacterales;f__Erwiniaceae;g__Pantoea;s__Pantoea_deleyi
PB1_gene_3567	234	ORM84002.1	234	233/234(99.57)	7.18e-160	tRNA/rRNA methyltransferase [Pantoea deleyi]	470932	d__Bacteria;k__norank;p__Proteobacteria;c__Gammaproteobacteria;o__Enterobacterales;f__Erwiniaceae;g__Pantoea;s__Pantoea_deleyi
PB1_gene_3568	59	SUB18516.1	58	36/58(62.07)	1.09e-12	Uncharacterised protein [Pantoea agglomerans]	549	d__Bacteria;k__norank;p__Proteobacteria;c__Gammaproteobacteria;o__Enterobacterales;f__Erwiniaceae;g__Pantoea;s__Pantoea_agglomerans
PB1_gene_3574	232	SUB08536.1	232	231/232(99.57)	4.52e-162	LIV-I protein F [Pantoea agglomerans]	549	d__Bacteria;k__norank;p__Proteobacteria;c__Gammaproteobacteria;o__Enterobacterales;f__Erwiniaceae;g__Pantoea;s__Pantoea_agglomerans
PB1_gene_3577	281	ORM80401.1	281	280/281(99.64)	6.13e-189	glycerol-3-phosphate transporter [Pantoea deleyi]	470932	d__Bacteria;k__norank;p__Proteobacteria;c__Gammaproteobacteria;o__Enterobacterales;f__Erwiniaceae;g__Pantoea;s__Pantoea_deleyi
PB1_gene_3580	348	ORM80744.1	348	345/348(99.14)	1.57e-252	oxidoreductase [Pantoea deleyi]	470932	d__Bacteria;k__norank;p__Proteobacteria;c__Gammaproteobacteria;o__Enterobacterales;f__Erwiniaceae;g__Pantoea;s__Pantoea_deleyi
PB1_gene_3590	161	ERM11188.1	161	160/161(99.38)	5.67e-112	2-C-methyl-D-erythritol 2,4-cyclodiphosphate synthase [Pantoea agglomerans Tx10]	1332072	d__Bacteria;k__norank;p__Proteobacteria;c__Gammaproteobacteria;o__Enterobacterales;f__Erwiniaceae;g__Pantoea;s__Pantoea_agglomerans
PB1_gene_3591	238	ORM80762.1	238	236/238(99.16)	2.72e-166	2-C-methyl-D-erythritol 4-phosphate cytidylyltransferase	470932	d__Bacteria;k__norank;p__Proteobacteria;c__Gammaproteobacteria;o__Enterobacterales;f__Erwiniaceae;g__Pantoea;s__Pantoea_deleyi
PB1_gene_3594	103	ORM84112.1	125	96/103(93.20)	5.63e-55	enamine deaminase RidA [Pantoea deleyi]	470932	d__Bacteria;k__norank;p__Proteobacteria;c__Gammaproteobacteria;o__Enterobacterales;f__Erwiniaceae;g__Pantoea;s__Pantoea_deleyi
PB1_gene_3595	175	ORM84111.1	175	160/175(91.43)	5.09e-115	DUF4865 domain-containing protein [Pantoea deleyi]	470932	d__Bacteria;k__norank;p__Proteobacteria;c__Gammaproteobacteria;o__Enterobacterales;f__Erwiniaceae;g__Pantoea;s__Pantoea_deleyi
PB1_gene_3597	106	ORM84109.1	106	101/106(95.28)	4.41e-67	carboxymuconolactone decarboxylase [Pantoea deleyi]	470932	d__Bacteria;k__norank;p__Proteobacteria;c__Gammaproteobacteria;o__Enterobacterales;f__Erwiniaceae;g__Pantoea;s__Pantoea_deleyi

PB1_gene_3599	330	ORM82749.1	328	318/328(96.95)	5.00e-244	stress response serine/threonine protein kinase YihE [Pantoea	470932	d__Bacteria;k__norank;p__Proteobacteria;c__Gammaproteobacteria;o__Enterobacterales;f__Erwiniaceae;g__Pantoea;s__Pantoea_deleyi
PB1_gene_3605	80	ORM83386.1	80	80/80(100.00)	7.31e-54	hypothetical protein HA42_06530 [Pantoea deleyi]	470932	d__Bacteria;k__norank;p__Proteobacteria;c__Gammaproteobacteria;o__Enterobacterales;f__Erwiniaceae;g__Pantoea;s__Pantoea_deleyi
PB1_gene_3608	109	SJZ30201.1	109	109/109(100.00)	2.07e-71	cytochrome o ubiquinol oxidase operon protein cyoD [Pantoea agglomerans]	470933	d__Bacteria;k__norank;p__Proteobacteria;c__Gammaproteobacteria;o__Enterobacterales;f__Erwiniaceae;g__Pantoea;s__Pantoea_eucalypti
PB1_gene_3609	204	SJZ30207.1	204	202/204(99.02)	1.48e-144	cytochrome bo3 quinol oxidase subunit 3 [Pantoea agglomerans]	470933	d__Bacteria;k__norank;p__Proteobacteria;c__Gammaproteobacteria;o__Enterobacterales;f__Erwiniaceae;g__Pantoea;s__Pantoea_eucalypti
PB1_gene_3611	261	ORM80773.1	447	258/260(99.23)	6.28e-179	recombination factor protein RarA [Pantoea deleyi]	470932	d__Bacteria;k__norank;p__Proteobacteria;c__Gammaproteobacteria;o__Enterobacterales;f__Erwiniaceae;g__Pantoea;s__Pantoea_deleyi
PB1_gene_3612	203	ORM55825.1	203	203/203(100.00)	1.13e-143	outer-membrane lipoprotein carrier protein LolA [Pantoea	472694	d__Bacteria;k__norank;p__Proteobacteria;c__Gammaproteobacteria;o__Enterobacterales;f__Erwiniaceae;g__Pantoea;s__Pantoea_brenneri
PB1_gene_3613	87	ORM83372.1	87	85/87(97.70)	1.36e-50	hypothetical protein HA42_06425 [Pantoea deleyi]	470932	d__Bacteria;k__norank;p__Proteobacteria;c__Gammaproteobacteria;o__Enterobacterales;f__Erwiniaceae;g__Pantoea;s__Pantoea_deleyi
PB1_gene_3614	87	WP_111139665.1	87	86/87(98.85)	2.75e-50	peroxide/acid stress response protein YhcN [Pantoea sp.	2027922	d__Bacteria;k__norank;p__Proteobacteria;c__Gammaproteobacteria;o__Enterobacterales;f__Erwiniaceae;g__Pantoea;s__Pantoea_sp._ARC607
PB1_gene_3615	94	ERM10970.1	94	92/94(97.87)	1.64e-61	hypothetical protein L584_01095 [Pantoea agglomerans Tx10]	1332072	d__Bacteria;k__norank;p__Proteobacteria;c__Gammaproteobacteria;o__Enterobacterales;f__Erwiniaceae;g__Pantoea;s__Pantoea_agglomerans
PB1_gene_3618	333	WP_111141623.1	329	311/333(93.39)	1.10e-207	CDF family zinc transporter ZitB [Pantoea sp. ARC607]	2027922	d__Bacteria;k__norank;p__Proteobacteria;c__Gammaproteobacteria;o__Enterobacterales;f__Erwiniaceae;g__Pantoea;s__Pantoea_sp._ARC607
PB1_gene_3621	330	WP_086906400.1	335	288/330(87.27)	6.85e-198	type IV pilus twitching motility protein PilT [Pantoea	549	d__Bacteria;k__norank;p__Proteobacteria;c__Gammaproteobacteria;o__Enterobacterales;f__Erwiniaceae;g__Pantoea;s__Pantoea_agglomerans
PB1_gene_3625	102	WP_111142073.1	102	98/102(96.08)	3.29e-67	DUF1496 domain-containing protein [Pantoea sp. ARC607]	2027922	d__Bacteria;k__norank;p__Proteobacteria;c__Gammaproteobacteria;o__Enterobacterales;f__Erwiniaceae;g__Pantoea;s__Pantoea_sp._ARC607
PB1_gene_3630	92	SKA19100.1	89	89/89(100.00)	8.49e-49	flagellar biosynthetic protein FliQ [Pantoea agglomerans]	470933	d__Bacteria;k__norank;p__Proteobacteria;c__Gammaproteobacteria;o__Enterobacterales;f__Erwiniaceae;g__Pantoea;s__Pantoea_eucalypti
PB1_gene_3631	261	WP_111142210.1	261	258/260(99.23)	3.65e-171	flagellar type III secretion system protein FliR [Pantoea sp.	2027922	d__Bacteria;k__norank;p__Proteobacteria;c__Gammaproteobacteria;o__Enterobacterales;f__Erwiniaceae;g__Pantoea;s__Pantoea_sp._ARC607
PB1_gene_3634	135	WP_111139963.1	135	132/135(97.78)	3.23e-85	flagellar export chaperone FliS [Pantoea sp. ARC607]	2027922	d__Bacteria;k__norank;p__Proteobacteria;c__Gammaproteobacteria;o__Enterobacterales;f__Erwiniaceae;g__Pantoea;s__Pantoea_sp._ARC607
PB1_gene_3635	121	ORM85050.1	121	117/121(96.69)	5.22e-73	flagellar biosynthesis protein FliT [Pantoea deleyi]	470932	d__Bacteria;k__norank;p__Proteobacteria;c__Gammaproteobacteria;o__Enterobacterales;f__Erwiniaceae;g__Pantoea;s__Pantoea_deleyi
PB1_gene_3638	171	ORM79880.1	171	168/171(98.25)	1.86e-115	molybdenum cofactor biosynthesis protein B [Pantoea	470932	d__Bacteria;k__norank;p__Proteobacteria;c__Gammaproteobacteria;o__Enterobacterales;f__Erwiniaceae;g__Pantoea;s__Pantoea_deleyi
PB1_gene_3647	130	ORM76847.1	130	126/130(96.92)	1.91e-86	hypothetical protein HA42_18625 [Pantoea deleyi]	470932	d__Bacteria;k__norank;p__Proteobacteria;c__Gammaproteobacteria;o__Enterobacterales;f__Erwiniaceae;g__Pantoea;s__Pantoea_deleyi
PB1_gene_3652	224	WP_111140245.1	246	217/224(96.88)	3.51e-158	gamma-glutamylcyclotransferase [Pantoea sp. ARC607]	2027922	d__Bacteria;k__norank;p__Proteobacteria;c__Gammaproteobacteria;o__Enterobacterales;f__Erwiniaceae;g__Pantoea;s__Pantoea_sp._ARC607
PB1_gene_3658	393	ORM82064.1	393	375/393(95.42)	1.77e-274	XylR family transcriptional regulator [Pantoea deleyi]	470932	d__Bacteria;k__norank;p__Proteobacteria;c__Gammaproteobacteria;o__Enterobacterales;f__Erwiniaceae;g__Pantoea;s__Pantoea_deleyi
PB1_gene_3661	369	WP_111139856.1	369	362/369(98.10)	1.40e-277	suppressor of fused domain protein [Pantoea sp. ARC607]	2027922	d__Bacteria;k__norank;p__Proteobacteria;c__Gammaproteobacteria;o__Enterobacterales;f__Erwiniaceae;g__Pantoea;s__Pantoea_sp._ARC607
PB1_gene_3663	208	CNU97221.1	235	126/208(60.58)	1.26e-84	Uncharacterised protein [Salmonella enterica subsp. enterica serovar	58097	d__Bacteria;k__norank;p__Proteobacteria;c__Gammaproteobacteria;o__Enterobacterales;f__Enterobacteriaceae;g__Salmonella;s__Salmonella_enterica
PB1_gene_3664	192	SJZ98835.1	192	191/192(99.48)	1.96e-134	hypothetical protein SAMN03097723_2679 [Pantoea agglomerans]	470933	d__Bacteria;k__norank;p__Proteobacteria;c__Gammaproteobacteria;o__Enterobacterales;f__Erwiniaceae;g__Pantoea;s__Pantoea_eucalypti
PB1_gene_3667	286	WP_084934050.1	288	245/284(86.27)	5.50e-179	MurR/RpiR family transcriptional regulator [Pantoea rwandensis]	1076550	d__Bacteria;k__norank;p__Proteobacteria;c__Gammaproteobacteria;o__Enterobacterales;f__Erwiniaceae;g__Pantoea;s__Pantoea_rwandensis

PB1_gene_3670	67	ORM81374.1	67	67/67(100.00)	1.65e-42	hypothetical protein HA42_10910 [Pantoea deleyi]	470932	d__Bacteria;k__norank;p__Proteobacteria;c__Gammaproteobacteria;o__Enterobacterales;f__Erwiniaceae;g__Pantoea;s__Pantoea_deleyi
PB1_gene_3672	155	WP_031590311.1	155	95/152(62.50)	8.56e-70	DUF943 family protein [Pantoea agglomerans]	549	d__Bacteria;k__norank;p__Proteobacteria;c__Gammaproteobacteria;o__Enterobacterales;f__Erwiniaceae;g__Pantoea;s__Pantoea_agglomerans
PB1_gene_3673	265	WP_031591891.1	281	216/264(81.82)	8.66e-162	DUF3289 family protein [Pantoea agglomerans]	549	d__Bacteria;k__norank;p__Proteobacteria;c__Gammaproteobacteria;o__Enterobacterales;f__Erwiniaceae;g__Pantoea;s__Pantoea_agglomerans
PB1_gene_3674	529	CAQ55150.1	550	525/529(99.24)	0	ribosomal protein S1 [Wolbachia endosymbiont of Culex quinquefasciatus Pel]	570417	d__Bacteria;k__norank;p__Proteobacteria;c__Alphaproteobacteria;o__Rickettsiales;f__Anaplasmataceae;g__Wolbachia;s__Wolbachia_endosymbiont_of_Culex_quinquefasciatus
PB1_gene_3676	515	WP_111141103.1	515	507/515(98.45)	0	threonine ammonia-lyase, biosynthetic [Pantoea sp.	2027922	d__Bacteria;k__norank;p__Proteobacteria;c__Gammaproteobacteria;o__Enterobacterales;f__Erwiniaceae;g__Pantoea;s__Pantoea_sp._ARC607
PB1_gene_3678	179	WP_098050381.1	179	178/179(99.44)	3.05e-118	septation protein A [Pantoea agglomerans]	None	Unclassified
PB1_gene_3684	368	WP_101854147.1	772	313/360(86.94)	1.66e-219	DNA primase [Yersiniaceae bacterium 2016Iso3]	2060066	d__Bacteria;k__norank;p__Proteobacteria;c__Gammaproteobacteria;o__Enterobacterales;f__Yersiniaceae;g__Chimaeribacter;s__Chimaeribacter_arupi
PB1_gene_3686	150	EZI33774.1	149	95/149(63.76)	4.10e-56	Tail completion protein S (GpS) [Pantoea agglomerans]	549	d__Bacteria;k__norank;p__Proteobacteria;c__Gammaproteobacteria;o__Enterobacterales;f__Erwiniaceae;g__Pantoea;s__Pantoea_agglomerans
PB1_gene_3689	240	WP_111138575.1	240	234/240(97.50)	3.86e-167	transcriptional regulator SdiA [Pantoea sp. ARC607]	2027922	d__Bacteria;k__norank;p__Proteobacteria;c__Gammaproteobacteria;o__Enterobacterales;f__Erwiniaceae;g__Pantoea;s__Pantoea_sp._ARC607
PB1_gene_3691	162	ADO08492.1	180	158/162(97.53)	3.42e-113	guanine-hypoxanthine phosphoribosyltransferase [Pantoea vagans C9-1]	712898	d__Bacteria;k__norank;p__Proteobacteria;c__Gammaproteobacteria;o__Enterobacterales;f__Erwiniaceae;g__Pantoea;s__Pantoea_vagans
PB1_gene_3692	298	SUB18217.1	60	39/60(65.00)	1.31e-14	Uncharacterised protein [Pantoea agglomerans]	549	d__Bacteria;k__norank;p__Proteobacteria;c__Gammaproteobacteria;o__Enterobacterales;f__Erwiniaceae;g__Pantoea;s__Pantoea_agglomerans
PB1_gene_3700	406	ORM81493.1	279	242/264(91.67)	6.60e-167	hypothetical protein HA42_10500 [Pantoea deleyi]	470932	d__Bacteria;k__norank;p__Proteobacteria;c__Gammaproteobacteria;o__Enterobacterales;f__Erwiniaceae;g__Pantoea;s__Pantoea_deleyi
PB1_gene_3702	455	ORM83619.1	457	449/451(99.56)	8.45e-314	PTS ascorbate transporter subunit IIC [Pantoea deleyi]	470932	d__Bacteria;k__norank;p__Proteobacteria;c__Gammaproteobacteria;o__Enterobacterales;f__Erwiniaceae;g__Pantoea;s__Pantoea_deleyi
PB1_gene_3704	362	ORM77684.1	362	362/362(100.00)	5.54e-260	3-dehydroquinate synthase [Pantoea deleyi]	470932	d__Bacteria;k__norank;p__Proteobacteria;c__Gammaproteobacteria;o__Enterobacterales;f__Erwiniaceae;g__Pantoea;s__Pantoea_deleyi
PB1_gene_3716	218	ORM81988.1	218	215/218(98.62)	1.72e-159	hydrolase [Pantoea deleyi]	470932	d__Bacteria;k__norank;p__Proteobacteria;c__Gammaproteobacteria;o__Enterobacterales;f__Erwiniaceae;g__Pantoea;s__Pantoea_deleyi
PB1_gene_3719	176	ORM85215.1	162	154/162(95.06)	1.72e-108	heat-shock protein [Pantoea deleyi]	470932	d__Bacteria;k__norank;p__Proteobacteria;c__Gammaproteobacteria;o__Enterobacterales;f__Erwiniaceae;g__Pantoea;s__Pantoea_deleyi
PB1_gene_3720	70	ORM85216.1	79	69/70(98.57)	1.26e-40	transcriptional regulator [Pantoea deleyi]	470932	d__Bacteria;k__norank;p__Proteobacteria;c__Gammaproteobacteria;o__Enterobacterales;f__Erwiniaceae;g__Pantoea;s__Pantoea_deleyi
PB1_gene_3721	74	ORM85217.1	74	72/74(97.30)	4.83e-45	hypothetical protein HA42_02985 [Pantoea deleyi]	470932	d__Bacteria;k__norank;p__Proteobacteria;c__Gammaproteobacteria;o__Enterobacterales;f__Erwiniaceae;g__Pantoea;s__Pantoea_deleyi
PB1_gene_3724	175	WP_111139136.1	208	171/175(97.71)	3.32e-123	DUF1007 family protein [Pantoea sp. ARC607]	2027922	d__Bacteria;k__norank;p__Proteobacteria;c__Gammaproteobacteria;o__Enterobacterales;f__Erwiniaceae;g__Pantoea;s__Pantoea_sp._ARC607
PB1_gene_3725	332	WP_111139137.1	332	296/332(89.16)	5.21e-195	nickel/cobalt transporter [Pantoea sp. ARC607]	2027922	d__Bacteria;k__norank;p__Proteobacteria;c__Gammaproteobacteria;o__Enterobacterales;f__Erwiniaceae;g__Pantoea;s__Pantoea_sp._ARC607
PB1_gene_3739	429	WP_085016766.1	429	429/429(100.00)	1.34e-310	MULTISPECIES: phosphopyruvate hydratase	91347	d__Bacteria;k__norank;p__Proteobacteria;c__Gammaproteobacteria;o__Enterobacterales;f__norank;g__norank;s__norank
PB1_gene_3745	419	ORM86335.1	419	417/419(99.52)	2.63e-296	UDP-N-acetylglucosamine 1-carboxyvinyltransferase [Pantoea deleyi]	470932	d__Bacteria;k__norank;p__Proteobacteria;c__Gammaproteobacteria;o__Enterobacterales;f__Erwiniaceae;g__Pantoea;s__Pantoea_deleyi
PB1_gene_3746	177	WP_111141576.1	177	175/177(98.87)	7.55e-127	peptide-methionine (S)-S-oxide reductase [Pantoea sp. ARC607]	2027922	d__Bacteria;k__norank;p__Proteobacteria;c__Gammaproteobacteria;o__Enterobacterales;f__Erwiniaceae;g__Pantoea;s__Pantoea_sp._ARC607
PB1_gene_3747	188	ORM82560.1	188	186/188(98.94)	8.71e-136	flavin reductase [Pantoea deleyi]	470932	d__Bacteria;k__norank;p__Proteobacteria;c__Gammaproteobacteria;o__Enterobacterales;f__Erwiniaceae;g__Pantoea;s__Pantoea_deleyi

PB1_gene_3751	53	WP_110331865.1	263	51/53(96.23)	1.64e-22	glucose 1-dehydrogenase [Pantoea sp. JKS000250]	1938795	d__Bacteria;k__norank;p__Proteobacteria;c__Gammaproteobacteria;o__Enterobacterales;f__Erwiniaceae;g__Pantoea;s__Pantoea_sp._JKS000250
PB1_gene_3753	85	WP_111142009.1	85	84/85(98.82)	6.84e-50	hypothetical protein [Pantoea sp. ARC607]	2027922	d__Bacteria;k__norank;p__Proteobacteria;c__Gammaproteobacteria;o__Enterobacterales;f__Erwiniaceae;g__Pantoea;s__Pantoea_sp._ARC607
PB1_gene_3755	326	ORM75909.1	326	321/325(98.77)	5.95e-230	4-hydroxythreonine-4-phosphate dehydrogenase PdxA [Pantoea deleyi]	470932	d__Bacteria;k__norank;p__Proteobacteria;c__Gammaproteobacteria;o__Enterobacterales;f__Erwiniaceae;g__Pantoea;s__Pantoea_deleyi
PB1_gene_3757	49	EFM21820.1	49	33/45(73.33)	2.46e-14	hypothetical protein PanABDRAFT_0348 [Pantoea sp. aB]	517433	d__Bacteria;k__norank;p__Proteobacteria;c__Gammaproteobacteria;o__Enterobacterales;f__Erwiniaceae;g__Pantoea;s__Pantoea_sp._aB
PB1_gene_3760	131	PAW35329.1	131	128/131(97.71)	8.70e-88	YraN family protein [Pantoea vagans]	470934	d__Bacteria;k__norank;p__Proteobacteria;c__Gammaproteobacteria;o__Enterobacterales;f__Erwiniaceae;g__Pantoea;s__Pantoea_vagans
PB1_gene_3761	195	ORM82457.1	195	194/195(99.49)	2.77e-131	phosphoheptose isomerase [Pantoea deleyi]	470932	d__Bacteria;k__norank;p__Proteobacteria;c__Gammaproteobacteria;o__Enterobacterales;f__Erwiniaceae;g__Pantoea;s__Pantoea_deleyi
PB1_gene_3768	305	ORM79855.1	305	295/305(96.72)	1.43e-215	LysR family transcriptional regulator [Pantoea deleyi]	470932	d__Bacteria;k__norank;p__Proteobacteria;c__Gammaproteobacteria;o__Enterobacterales;f__Erwiniaceae;g__Pantoea;s__Pantoea_deleyi
PB1_gene_3772	71	AVV38290.1	118	66/71(92.96)	2.44e-39	hypothetical protein C9381_14275 [Pantoea vagans]	470934	d__Bacteria;k__norank;p__Proteobacteria;c__Gammaproteobacteria;o__Enterobacterales;f__Erwiniaceae;g__Pantoea;s__Pantoea_vagans
PB1_gene_3773	72	WP_061062761.1	72	63/72(87.50)	5.35e-39	type I toxin-antitoxin system SymE family toxin [Pantoea	470934	d__Bacteria;k__norank;p__Proteobacteria;c__Gammaproteobacteria;o__Enterobacterales;f__Erwiniaceae;g__Pantoea;s__Pantoea_vagans
PB1_gene_3774	97	WP_064690486.1	92	82/92(89.13)	7.42e-45	hypothetical protein [Pantoea agglomerans]	549	d__Bacteria;k__norank;p__Proteobacteria;c__Gammaproteobacteria;o__Enterobacterales;f__Erwiniaceae;g__Pantoea;s__Pantoea_agglomerans
PB1_gene_3775	101	WP_064690442.1	99	87/98(88.78)	8.60e-60	type II toxin-antitoxin system RelE/ParE family toxin [Pantoea agglomerans]	549	d__Bacteria;k__norank;p__Proteobacteria;c__Gammaproteobacteria;o__Enterobacterales;f__Erwiniaceae;g__Pantoea;s__Pantoea_agglomerans
PB1_gene_3778	418	ORM79506.1	418	394/418(94.26)	5.86e-288	3-phytase [Pantoea deleyi]	470932	d__Bacteria;k__norank;p__Proteobacteria;c__Gammaproteobacteria;o__Enterobacterales;f__Erwiniaceae;g__Pantoea;s__Pantoea_deleyi
PB1_gene_3788	103	ORM81045.1	90	90/90(100.00)	5.39e-55	hypothetical protein HA42_12065 [Pantoea deleyi]	470932	d__Bacteria;k__norank;p__Proteobacteria;c__Gammaproteobacteria;o__Enterobacterales;f__Erwiniaceae;g__Pantoea;s__Pantoea_deleyi
PB1_gene_3792	166	SJZ68591.1	166	166/166(100.00)	7.95e-110	PTS system D-glucose-specific IIA component, Glc family [Pantoea agglomerans]	470933	d__Bacteria;k__norank;p__Proteobacteria;c__Gammaproteobacteria;o__Enterobacterales;f__Erwiniaceae;g__Pantoea;s__Pantoea_eucalypti
PB1_gene_3797	136	WP_052259209.1	120	117/120(97.50)	1.89e-81	hypothetical protein [Pantoea agglomerans]	549	d__Bacteria;k__norank;p__Proteobacteria;c__Gammaproteobacteria;o__Enterobacterales;f__Erwiniaceae;g__Pantoea;s__Pantoea_agglomerans
PB1_gene_3802	197	WP_111139673.1	197	194/197(98.48)	3.22e-131	septum formation inhibitor Maf [Pantoea sp. ARC607]	2027922	d__Bacteria;k__norank;p__Proteobacteria;c__Gammaproteobacteria;o__Enterobacterales;f__Erwiniaceae;g__Pantoea;s__Pantoea_sp._ARC607
PB1_gene_3819	217	SJZ33362.1	217	217/217(100.00)	1.44e-137	modulator of FtsH protease [Pantoea agglomerans]	470933	d__Bacteria;k__norank;p__Proteobacteria;c__Gammaproteobacteria;o__Enterobacterales;f__Erwiniaceae;g__Pantoea;s__Pantoea_eucalypti
PB1_gene_3821	97	WP_111141983.1	91	89/91(97.80)	2.71e-58	DUF1040 family protein [Pantoea sp. ARC607]	2027922	d__Bacteria;k__norank;p__Proteobacteria;c__Gammaproteobacteria;o__Enterobacterales;f__Erwiniaceae;g__Pantoea;s__Pantoea_sp._ARC607
PB1_gene_3822	188	WP_111141982.1	188	180/188(95.74)	6.35e-129	molybdenum cofactor guanylyltransferase MobA [Pantoea sp. ARC607]	2027922	d__Bacteria;k__norank;p__Proteobacteria;c__Gammaproteobacteria;o__Enterobacterales;f__Erwiniaceae;g__Pantoea;s__Pantoea_sp._ARC607
PB1_gene_3823	168	SUB04921.1	168	138/166(83.13)	4.86e-96	Molybdopterin-guanine dinucleotide biosynthesis protein B [Pantoea agglomerans]	549	d__Bacteria;k__norank;p__Proteobacteria;c__Gammaproteobacteria;o__Enterobacterales;f__Erwiniaceae;g__Pantoea;s__Pantoea_agglomerans
PB1_gene_3824	78	ORM81417.1	78	74/78(94.87)	3.06e-43	hypothetical protein HA42_10815 [Pantoea deleyi]	470932	d__Bacteria;k__norank;p__Proteobacteria;c__Gammaproteobacteria;o__Enterobacterales;f__Erwiniaceae;g__Pantoea;s__Pantoea_deleyi
PB1_gene_3825	180	ORM81418.1	180	177/180(98.33)	8.76e-123	porin [Pantoea deleyi]	470932	d__Bacteria;k__norank;p__Proteobacteria;c__Gammaproteobacteria;o__Enterobacterales;f__Erwiniaceae;g__Pantoea;s__Pantoea_deleyi

PB1_gene_3826	100	ORM81419.1	100	96/100(96.00)	3.61e-52	hypothetical protein HA42_10825 [Pantoea deleyi]	470932	d__Bacteria;k__norank;p__Proteobacteria;c__Gammaproteobacteria;o__Enterobacterales;f__Erwiniaceae;g__Pantoea;s__Pantoea_deleyi
PB1_gene_3828	459	WP_111141718.1	459	434/459(94.55)	0	acyl-CoA transferase [Pantoea sp. ARC607]	2027922	d__Bacteria;k__norank;p__Proteobacteria;c__Gammaproteobacteria;o__Enterobacterales;f__Erwiniaceae;g__Pantoea;s__Pantoea_sp._ARC607
PB1_gene_3830	299	ORM81645.1	299	293/299(97.99)	9.51e-202	chloramphenical resistance permease RarD [Pantoea deleyi]	470932	d__Bacteria;k__norank;p__Proteobacteria;c__Gammaproteobacteria;o__Enterobacterales;f__Erwiniaceae;g__Pantoea;s__Pantoea_deleyi
PB1_gene_3832	111	SWG71156.1	256	103/111(92.79)	6.21e-68	IS2 transposase [Klebsiella pneumoniae]	573	d__Bacteria;k__norank;p__Proteobacteria;c__Gammaproteobacteria;o__Enterobacterales;f__Enterobacteriaceae;g__Klebsiella;s__Klebsiella_pneumoniae
PB1_gene_3833	132	EHD23251.1	153	120/132(90.91)	3.80e-78	transposase IS3/IS911 family protein [Brenneria sp. EniD312]	598467	d__Bacteria;k__norank;p__Proteobacteria;c__Gammaproteobacteria;o__Enterobacterales;f__Pectobacteriaceae;g__Brenneria;s__Brenneria_sp._EniD312
PB1_gene_3840	457	ORM82737.1	457	451/457(98.69)	1.98e-308	xanthine permease XanP [Pantoea deleyi]	470932	d__Bacteria;k__norank;p__Proteobacteria;c__Gammaproteobacteria;o__Enterobacterales;f__Erwiniaceae;g__Pantoea;s__Pantoea_deleyi
PB1_gene_3842	263	WP_111138790.1	263	261/263(99.24)	9.09e-182	aliphatic sulfonate ABC transporter permease SsuC [Pantoea sp. ARC607]	2027922	d__Bacteria;k__norank;p__Proteobacteria;c__Gammaproteobacteria;o__Enterobacterales;f__Erwiniaceae;g__Pantoea;s__Pantoea_sp._ARC607
PB1_gene_3847	385	ORM76163.1	385	374/385(97.14)	3.03e-271	monooxygenase [Pantoea deleyi]	470932	d__Bacteria;k__norank;p__Proteobacteria;c__Gammaproteobacteria;o__Enterobacterales;f__Erwiniaceae;g__Pantoea;s__Pantoea_deleyi
PB1_gene_3852	137	WP_111208828.1	139	96/131(73.28)	2.51e-63	hypothetical protein [Pantoea sp. ARC270]	2630326	d__Bacteria;k__norank;p__Proteobacteria;c__Gammaproteobacteria;o__Enterobacterales;f__Erwiniaceae;g__Pantoea;s__norank
PB1_gene_3864	235	WP_111139179.1	235	232/235(98.72)	1.02e-164	two-component system response regulator BaeR [Pantoea sp. ARC607]	2027922	d__Bacteria;k__norank;p__Proteobacteria;c__Gammaproteobacteria;o__Enterobacterales;f__Erwiniaceae;g__Pantoea;s__Pantoea_sp._ARC607
PB1_gene_3866	417	ORM84927.1	417	415/416(99.76)	1.54e-303	serine hydroxymethyltransferase [Pantoea deleyi]	470932	d__Bacteria;k__norank;p__Proteobacteria;c__Gammaproteobacteria;o__Enterobacterales;f__Erwiniaceae;g__Pantoea;s__Pantoea_deleyi
PB1_gene_3869	159	ORM78818.1	159	155/159(97.48)	4.17e-113	hypothetical protein HA42_16295 [Pantoea deleyi]	470932	d__Bacteria;k__norank;p__Proteobacteria;c__Gammaproteobacteria;o__Enterobacterales;f__Erwiniaceae;g__Pantoea;s__Pantoea_deleyi
PB1_gene_3872	211	ORM86332.1	211	211/211(100.00)	4.37e-145	phospholipid-binding protein MlaC [Pantoea deleyi]	470932	d__Bacteria;k__norank;p__Proteobacteria;c__Gammaproteobacteria;o__Enterobacterales;f__Erwiniaceae;g__Pantoea;s__Pantoea_deleyi
PB1_gene_3873	181	ORM86331.1	181	162/162(100.00)	4.09e-108	outer membrane lipid asymmetry maintenance protein MlaD [Pantoea deleyi]	470932	d__Bacteria;k__norank;p__Proteobacteria;c__Gammaproteobacteria;o__Enterobacterales;f__Erwiniaceae;g__Pantoea;s__Pantoea_deleyi
PB1_gene_3876	304	WP_090960845.1	304	284/304(93.42)	5.22e-197	LysR family transcriptional regulator [Pantoea sp. OV426]	1881036	d__Bacteria;k__norank;p__Proteobacteria;c__Gammaproteobacteria;o__Enterobacterales;f__Erwiniaceae;g__Pantoea;s__Pantoea_sp._OV426
PB1_gene_3878	225	WP_111141194.1	225	225/225(100.00)	3.61e-169	hypothetical protein [Pantoea sp. ARC607]	2027922	d__Bacteria;k__norank;p__Proteobacteria;c__Gammaproteobacteria;o__Enterobacterales;f__Erwiniaceae;g__Pantoea;s__Pantoea_sp._ARC607
PB1_gene_3880	184	ORM78067.1	463	183/184(99.46)	6.82e-117	nitrate/nitrite transporter [Pantoea deleyi]	470932	d__Bacteria;k__norank;p__Proteobacteria;c__Gammaproteobacteria;o__Enterobacterales;f__Erwiniaceae;g__Pantoea;s__Pantoea_deleyi
PB1_gene_3884	86	ORM83046.1	86	86/86(100.00)	1.42e-56	Rho-binding antiterminator [Pantoea deleyi]	470932	d__Bacteria;k__norank;p__Proteobacteria;c__Gammaproteobacteria;o__Enterobacterales;f__Erwiniaceae;g__Pantoea;s__Pantoea_deleyi
PB1_gene_3885	102	WP_111140596.1	104	98/102(96.08)	1.81e-60	cytochrome c [Pantoea sp. ARC607]	2027922	d__Bacteria;k__norank;p__Proteobacteria;c__Gammaproteobacteria;o__Enterobacterales;f__Erwiniaceae;g__Pantoea;s__Pantoea_sp._ARC607
PB1_gene_3892	109	ORM81837.1	108	102/108(94.44)	9.93e-72	nitrite reductase small subunit [Pantoea deleyi]	470932	d__Bacteria;k__norank;p__Proteobacteria;c__Gammaproteobacteria;o__Enterobacterales;f__Erwiniaceae;g__Pantoea;s__Pantoea_deleyi
PB1_gene_3896	254	ORM80849.1	254	244/254(96.06)	2.70e-174	acyl-CoA esterase [Pantoea deleyi]	470932	d__Bacteria;k__norank;p__Proteobacteria;c__Gammaproteobacteria;o__Enterobacterales;f__Erwiniaceae;g__Pantoea;s__Pantoea_deleyi
PB1_gene_3897	91	SJZ31213.1	91	90/91(98.90)	5.41e-52	Ribbon-helix-helix protein, copG family [Pantoea agglomerans]	470933	d__Bacteria;k__norank;p__Proteobacteria;c__Gammaproteobacteria;o__Enterobacterales;f__Erwiniaceae;g__Pantoea;s__Pantoea_eucalypti
PB1_gene_3900	203	WP_111139769.1	201	198/201(98.51)	7.32e-135	SCP2 domain-containing protein [Pantoea sp. ARC607]	2027922	d__Bacteria;k__norank;p__Proteobacteria;c__Gammaproteobacteria;o__Enterobacterales;f__Erwiniaceae;g__Pantoea;s__Pantoea_sp._ARC607
PB1_gene_3909	61	WP_081469648.1	72	50/61(81.97)	3.40e-30	hypothetical protein [Cronobacter turicensis]	413502	d__Bacteria;k__norank;p__Proteobacteria;c__Gammaproteobacteria;o__Enterobacterales;f__Enterobacteriaceae;g__Cronobacter;s__Cronobacter_turicensis

PB1_gene_3914	215	WP_111141005.1	215	205/215(95.35)	5.70e-143	pyroglutamyl-peptidase I [Pantoea sp. ARC607]	2027922	d__Bacteria;k__norank;p__Proteobacteria;c__Gammaproteobacteria;o__Enterobacterales;f__Erwiniaceae;g__Pantoea;s__Pantoea_sp._ARC607
PB1_gene_3919	310	WP_111139668.1	310	305/309(98.71)	5.11e-207	p-hydroxybenzoic acid efflux pump subunit AaeA [Pantoea sp.	2027922	d__Bacteria;k__norank;p__Proteobacteria;c__Gammaproteobacteria;o__Enterobacterales;f__Erwiniaceae;g__Pantoea;s__Pantoea_sp._ARC607
PB1_gene_3923	258	WP_080694244.1	258	233/258(90.31)	5.99e-164	MULTISPECIES: heme ABC transporter ATP-binding protein [Pantoea]	53335	d__Bacteria;k__norank;p__Proteobacteria;c__Gammaproteobacteria;o__Enterobacterales;f__Erwiniaceae;g__Pantoea;s__norank
PB1_gene_3933	333	ORM80423.1	333	331/333(99.40)	5.33e-216	sugar ABC transporter permease [Pantoea deleyi]	470932	d__Bacteria;k__norank;p__Proteobacteria;c__Gammaproteobacteria;o__Enterobacterales;f__Erwiniaceae;g__Pantoea;s__Pantoea_deleyi
PB1_gene_3940	68	WP_111139468.1	68	65/68(95.59)	9.91e-40	hypothetical protein [Pantoea sp. ARC607]	2027922	d__Bacteria;k__norank;p__Proteobacteria;c__Gammaproteobacteria;o__Enterobacterales;f__Erwiniaceae;g__Pantoea;s__Pantoea_sp._ARC607
PB1_gene_3941	158	ORM86077.1	158	129/158(81.65)	5.96e-83	hypothetical protein HA42_01155 [Pantoea deleyi]	470932	d__Bacteria;k__norank;p__Proteobacteria;c__Gammaproteobacteria;o__Enterobacterales;f__Erwiniaceae;g__Pantoea;s__Pantoea_deleyi
PB1_gene_3948	126	ORM77287.1	126	122/126(96.83)	1.21e-78	transcriptional regulator [Pantoea deleyi]	470932	d__Bacteria;k__norank;p__Proteobacteria;c__Gammaproteobacteria;o__Enterobacterales;f__Erwiniaceae;g__Pantoea;s__Pantoea_deleyi
PB1_gene_3950	115	ORM86831.1	115	111/115(96.52)	1.37e-72	hypothetical protein HA42_00180 [Pantoea deleyi]	470932	d__Bacteria;k__norank;p__Proteobacteria;c__Gammaproteobacteria;o__Enterobacterales;f__Erwiniaceae;g__Pantoea;s__Pantoea_deleyi
PB1_gene_3952	311	WP_086905719.1	311	308/311(99.04)	1.54e-213	ABC transporter substrate-binding protein [Pantoea	53335	d__Bacteria;k__norank;p__Proteobacteria;c__Gammaproteobacteria;o__Enterobacterales;f__Erwiniaceae;g__Pantoea;s__norank
PB1_gene_3953	132	SUB17157.1	206	132/132(100.00)	4.66e-86	3-oxoacyl-[acyl-carrier-protein] reductase FabG [Pantoea agglomerans]	549	d__Bacteria;k__norank;p__Proteobacteria;c__Gammaproteobacteria;o__Enterobacterales;f__Erwiniaceae;g__Pantoea;s__Pantoea_agglomerans
PB1_gene_3956	152	ORM83850.1	152	144/152(94.74)	1.66e-102	GNAT family N-acetyltransferase [Pantoea deleyi]	470932	d__Bacteria;k__norank;p__Proteobacteria;c__Gammaproteobacteria;o__Enterobacterales;f__Erwiniaceae;g__Pantoea;s__Pantoea_deleyi
PB1_gene_3958	360	SUB14859.1	425	321/359(89.42)	3.35e-188	Ribose and galactose chemoreceptor protein [Pantoea	549	d__Bacteria;k__norank;p__Proteobacteria;c__Gammaproteobacteria;o__Enterobacterales;f__Erwiniaceae;g__Pantoea;s__Pantoea_agglomerans
PB1_gene_3959	108	WP_111139221.1	108	106/108(98.15)	7.26e-67	biofilm peroxide resistance protein BsmA [Pantoea sp.	2027922	d__Bacteria;k__norank;p__Proteobacteria;c__Gammaproteobacteria;o__Enterobacterales;f__Erwiniaceae;g__Pantoea;s__Pantoea_sp._ARC607
PB1_gene_3961	86	ORM83848.1	86	86/86(100.00)	7.28e-59	2Fe-2S ferredoxin [Pantoea deleyi]	470932	d__Bacteria;k__norank;p__Proteobacteria;c__Gammaproteobacteria;o__Enterobacterales;f__Erwiniaceae;g__Pantoea;s__Pantoea_deleyi
PB1_gene_3965	61	AGJ99991.1	909	36/46(78.26)	5.58e-14	Ankyrin repeat domain protein [Wolbachia endosymbiont of Drosophila simulans wHa]	1236909	d__Bacteria;k__norank;p__Proteobacteria;c__Alphaproteobacteria;o__Rickettsiales;f__Anaplasmataceae;g__Wolbachia;s__Wolbachia_endosymbiont_of_Drosophila_simulans
PB1_gene_3966	143	ATZ93011.1	143	88/143(61.54)	4.23e-62	DUF29 domain-containing protein [Dickeya fangzhongdai]	1778540	d__Bacteria;k__norank;p__Proteobacteria;c__Gammaproteobacteria;o__Enterobacterales;f__Pectobacteriaceae;g__Dickeya;s__Dickeya_fangzhongdai
PB1_gene_3967	104	SPY66662.1	104	54/105(51.43)	7.11e-24	Uncharacterized HTH-type transcriptional regulator YddM [Providencia stuartii]	588	d__Bacteria;k__norank;p__Proteobacteria;c__Gammaproteobacteria;o__Enterobacterales;f__Morganellaceae;g__Providencia;s__Providencia_stuartii
PB1_gene_3970	156	SEL23886.1	156	108/156(69.23)	1.01e-73	hypothetical protein SAMN04487787_10871 [Kosakonia sacchari]	1158459	d__Bacteria;k__norank;p__Proteobacteria;c__Gammaproteobacteria;o__Enterobacterales;f__Enterobacteriaceae;g__Kosakonia;s__Kosakonia_sacchari
PB1_gene_3971	192	WP_085336005.1	217	146/190(76.84)	2.12e-105	hypothetical protein [Salmonella enterica]	28901	d__Bacteria;k__norank;p__Proteobacteria;c__Gammaproteobacteria;o__Enterobacterales;f__Enterobacteriaceae;g__Salmonella;s__Salmonella_enterica
PB1_gene_3976	63	ORM75502.1	63	62/63(98.41)	9.27e-36	hypothetical protein HA42_20520 [Pantoea deleyi]	470932	d__Bacteria;k__norank;p__Proteobacteria;c__Gammaproteobacteria;o__Enterobacterales;f__Erwiniaceae;g__Pantoea;s__Pantoea_deleyi
PB1_gene_3977	174	ORM75503.1	174	171/174(98.28)	1.16e-115	shikimate kinase II [Pantoea deleyi]	470932	d__Bacteria;k__norank;p__Proteobacteria;c__Gammaproteobacteria;o__Enterobacterales;f__Erwiniaceae;g__Pantoea;s__Pantoea_deleyi
PB1_gene_3983	149	WP_111138292.1	171	140/149(93.96)	1.09e-90	sigma-70 family RNA polymerase sigma factor [Pantoea sp.	2027922	d__Bacteria;k__norank;p__Proteobacteria;c__Gammaproteobacteria;o__Enterobacterales;f__Erwiniaceae;g__Pantoea;s__Pantoea_sp._ARC607
PB1_gene_3986	260	ORM86024.1	260	258/260(99.23)	1.23e-173	ABC transporter ATP-binding protein [Pantoea deleyi]	470932	d__Bacteria;k__norank;p__Proteobacteria;c__Gammaproteobacteria;o__Enterobacterales;f__Erwiniaceae;g__Pantoea;s__Pantoea_deleyi

PB1_gene_3991	350	WP_111138697.1	336	306/336(91.07)	1.03e-209	phosphatase [Pantoea sp. ARC607]	2027922	d__Bacteria;k__norank;p__Proteobacteria;c__Gammaproteobacteria;o__Enterobacterales;f__Erwiniaceae;g__Pantoea;s__Pantoea_sp._ARC607
PB1_gene_3995	362	WP_111138753.1	351	338/351(96.30)	5.61e-223	YeiH family putative sulfate export transporter [Pantoea sp.	2027922	d__Bacteria;k__norank;p__Proteobacteria;c__Gammaproteobacteria;o__Enterobacterales;f__Erwiniaceae;g__Pantoea;s__Pantoea_sp._ARC607
PB1_gene_3997	138	ORM83275.1	138	129/134(96.27)	2.35e-93	aldehyde-activating protein [Pantoea deleyi]	470932	d__Bacteria;k__norank;p__Proteobacteria;c__Gammaproteobacteria;o__Enterobacterales;f__Erwiniaceae;g__Pantoea;s__Pantoea_deleyi
PB1_gene_4001	129	ORM82469.1	128	120/128(93.75)	1.11e-73	cytochrome B562 [Pantoea deleyi]	470932	d__Bacteria;k__norank;p__Proteobacteria;c__Gammaproteobacteria;o__Enterobacterales;f__Erwiniaceae;g__Pantoea;s__Pantoea_deleyi
PB1_gene_4005	111	ORM78935.1	115	111/111(100.00)	7.54e-69	hypothetical protein HA42_16080 [Pantoea deleyi]	470932	d__Bacteria;k__norank;p__Proteobacteria;c__Gammaproteobacteria;o__Enterobacterales;f__Erwiniaceae;g__Pantoea;s__Pantoea_deleyi
PB1_gene_4006	155	ORM78934.1	155	154/155(99.35)	1.58e-106	lysozyme [Pantoea deleyi]	470932	d__Bacteria;k__norank;p__Proteobacteria;c__Gammaproteobacteria;o__Enterobacterales;f__Erwiniaceae;g__Pantoea;s__Pantoea_deleyi
PB1_gene_4008	65	ORM75440.1	65	63/65(96.92)	7.76e-38	hypothetical protein HA42_20695 [Pantoea deleyi]	470932	d__Bacteria;k__norank;p__Proteobacteria;c__Gammaproteobacteria;o__Enterobacterales;f__Erwiniaceae;g__Pantoea;s__Pantoea_deleyi
PB1_gene_4014	155	ORM77013.1	155	153/155(98.71)	4.91e-110	thioredoxin-dependent thiol peroxidase [Pantoea deleyi]	470932	d__Bacteria;k__norank;p__Proteobacteria;c__Gammaproteobacteria;o__Enterobacterales;f__Erwiniaceae;g__Pantoea;s__Pantoea_deleyi
PB1_gene_4023	75	ELP22924.1	44	36/41(87.80)	4.00e-17	hypothetical protein F385_4028 [Pantoea agglomerans 299R]	1261128	d__Bacteria;k__norank;p__Proteobacteria;c__Gammaproteobacteria;o__Enterobacterales;f__Erwiniaceae;g__Pantoea;s__Pantoea_agglomerans
PB1_gene_4026	237	WP_111139334.1	237	234/237(98.73)	5.07e-166	phosphoribosylaminoimidazolesuccinocarboxamide synthase [Pantoea sp. ARC607]	2027922	d__Bacteria;k__norank;p__Proteobacteria;c__Gammaproteobacteria;o__Enterobacterales;f__Erwiniaceae;g__Pantoea;s__Pantoea_sp._ARC607
PB1_gene_4031	181	ORM77793.1	181	180/181(99.45)	3.72e-129	porin [Pantoea deleyi]	470932	d__Bacteria;k__norank;p__Proteobacteria;c__Gammaproteobacteria;o__Enterobacterales;f__Erwiniaceae;g__Pantoea;s__Pantoea_deleyi
PB1_gene_4034	107	OYN57106.1	107	102/107(95.33)	1.31e-64	transcriptional regulator [Pectobacterium carotovorum]	554	d__Bacteria;k__norank;p__Proteobacteria;c__Gammaproteobacteria;o__Enterobacterales;f__Pectobacteriaceae;g__Pectobacterium;s__Pectobacterium_carotovorum
PB1_gene_4035	119	WP_100684799.1	119	114/119(95.80)	9.83e-77	MULTISPECIES: type II toxin-antitoxin system RelE/ParE family toxin [Raoultella]	160674	d__Bacteria;k__norank;p__Proteobacteria;c__Gammaproteobacteria;o__Enterobacterales;f__Enterobacteriaceae;g__Raoultella;s__norank
PB1_gene_4038	201	ORM80760.1	201	200/201(99.50)	9.28e-142	adenylyl-sulfate kinase [Pantoea deleyi]	470932	d__Bacteria;k__norank;p__Proteobacteria;c__Gammaproteobacteria;o__Enterobacterales;f__Erwiniaceae;g__Pantoea;s__Pantoea_deleyi
PB1_gene_4039	106	ORM80761.1	106	105/106(99.06)	3.11e-67	hypothetical protein HA42_13170 [Pantoea deleyi]	470932	d__Bacteria;k__norank;p__Proteobacteria;c__Gammaproteobacteria;o__Enterobacterales;f__Erwiniaceae;g__Pantoea;s__Pantoea_deleyi
PB1_gene_4041	68	SKA03709.1	68	68/68(100.00)	2.95e-41	Protein of unknown function [Pantoea agglomerans]	470933	d__Bacteria;k__norank;p__Proteobacteria;c__Gammaproteobacteria;o__Enterobacterales;f__Erwiniaceae;g__Pantoea;s__Pantoea_eucalypti
PB1_gene_4042	185	ORM82482.1	185	181/185(97.84)	4.47e-123	hypothetical protein HA42_08265 [Pantoea deleyi]	470932	d__Bacteria;k__norank;p__Proteobacteria;c__Gammaproteobacteria;o__Enterobacterales;f__Erwiniaceae;g__Pantoea;s__Pantoea_deleyi
PB1_gene_4045	358	ORM77451.1	358	358/358(100.00)	2.53e-238	iron-siderophore ABC transporter permease [Pantoea deleyi]	470932	d__Bacteria;k__norank;p__Proteobacteria;c__Gammaproteobacteria;o__Enterobacterales;f__Erwiniaceae;g__Pantoea;s__Pantoea_deleyi
PB1_gene_4050	125	CRH28589.1	124	105/123(85.37)	1.79e-68	Ribosomal silencing factor RsfS {ECO:0000255|HAMAP-Rule:MF_01477} [Pantoea	553	d__Bacteria;k__norank;p__Proteobacteria;c__Gammaproteobacteria;o__Enterobacterales;f__Erwiniaceae;g__Pantoea;s__Pantoea_ananatis
PB1_gene_4051	156	ORM79045.1	156	154/155(99.35)	2.16e-109	23S rRNA (pseudouridine(1915)-N(3))-methyltransferase RlmH [Pantoea deleyi]	470932	d__Bacteria;k__norank;p__Proteobacteria;c__Gammaproteobacteria;o__Enterobacterales;f__Erwiniaceae;g__Pantoea;s__Pantoea_deleyi
PB1_gene_4054	437	EWG64811.1	449	435/437(99.54)	3.74e-315	hypothetical protein P348_04613 [Enterobacter sp. DC3]	1395579	d__Bacteria;k__norank;p__Proteobacteria;c__Gammaproteobacteria;o__Enterobacterales;f__Enterobacteriaceae;g__Enterobacter;s__Enterobacter_sp._DC3
PB1_gene_4063	92	ORM78933.1	92	92/92(100.00)	4.76e-60	hypothetical protein HA42_16070 [Pantoea deleyi]	470932	d__Bacteria;k__norank;p__Proteobacteria;c__Gammaproteobacteria;o__Enterobacterales;f__Erwiniaceae;g__Pantoea;s__Pantoea_deleyi
PB1_gene_4070	312	WP_111139662.1	312	307/312(98.40)	2.08e-207	malate dehydrogenase [Pantoea sp. ARC607]	2027922	d__Bacteria;k__norank;p__Proteobacteria;c__Gammaproteobacteria;o__Enterobacterales;f__Erwiniaceae;g__Pantoea;s__Pantoea_sp._ARC607

PB1_gene_4074	76	WP_111140353.1	79	71/76(93.42)	1.97e-41	hypothetical protein [Pantoea sp. ARC607]	2027922	d__Bacteria;k__norank;p__Proteobacteria;c__Gammaproteobacteria;o__Enterobacterales;f__Erwiniaceae;g__Pantoea;s__Pantoea_sp._ARC607
PB1_gene_4086	174	ORM83384.1	153	153/153(100.00)	1.76e-94	acetyl-CoA carboxylase, biotin carboxyl carrier protein [Pantoea deleyi]	470932	d__Bacteria;k__norank;p__Proteobacteria;c__Gammaproteobacteria;o__Enterobacterales;f__Erwiniaceae;g__Pantoea;s__Pantoea_deleyi
PB1_gene_4087	350	RAH29237.1	350	331/350(94.57)	8.01e-247	patatin family protein [Pantoea agglomerans]	549	d__Bacteria;k__norank;p__Proteobacteria;c__Gammaproteobacteria;o__Enterobacterales;f__Erwiniaceae;g__Pantoea;s__Pantoea_agglomerans
PB1_gene_4091	286	ORM82369.1	286	283/286(98.95)	2.07e-194	spermidine/putrescine ABC transporter permease PotB [Pantoea deleyi]	470932	d__Bacteria;k__norank;p__Proteobacteria;c__Gammaproteobacteria;o__Enterobacterales;f__Erwiniaceae;g__Pantoea;s__Pantoea_deleyi
PB1_gene_4093	121	STV47190.1	154	74/117(63.25)	7.49e-43	Uncharacterised protein [Klebsiella pneumoniae subsp.	574	d__Bacteria;k__norank;p__Proteobacteria;c__Gammaproteobacteria;o__Enterobacterales;f__Enterobacteriaceae;g__Klebsiella;s__Klebsiella_pneumoniae
PB1_gene_4102	156	WP_111140005.1	156	156/156(100.00)	1.19e-97	6,7-dimethyl-8-ribityllumazine synthase [Pantoea sp. ARC607]	53335	d__Bacteria;k__norank;p__Proteobacteria;c__Gammaproteobacteria;o__Enterobacterales;f__Erwiniaceae;g__Pantoea;s__norank
PB1_gene_4110	119	SKA09814.1	119	118/119(99.16)	7.19e-79	ribonuclease P protein component [Pantoea agglomerans]	470933	d__Bacteria;k__norank;p__Proteobacteria;c__Gammaproteobacteria;o__Enterobacterales;f__Erwiniaceae;g__Pantoea;s__Pantoea_eucalypti
PB1_gene_4112	52	WP_111208649.1	67	26/28(92.86)	3.09e-07	hypothetical protein [Pantoea sp. ARC270]	None	Unclassified
PB1_gene_4113	188	ORM80568.1	188	185/188(98.40)	8.66e-127	fructose-1-phosphate/6-phosphogluconate phosphatase [Pantoea deleyi]	470932	d__Bacteria;k__norank;p__Proteobacteria;c__Gammaproteobacteria;o__Enterobacterales;f__Erwiniaceae;g__Pantoea;s__Pantoea_deleyi
PB1_gene_4116	132	WP_111138965.1	130	116/129(89.92)	1.11e-81	DUF4440 domain-containing protein [Pantoea sp. ARC607]	2027922	d__Bacteria;k__norank;p__Proteobacteria;c__Gammaproteobacteria;o__Enterobacterales;f__Erwiniaceae;g__Pantoea;s__Pantoea_sp._ARC607
PB1_gene_4120	355	ORM80950.1	355	355/355(100.00)	7.33e-266	uroporphyrinogen decarboxylase [Pantoea deleyi]	470932	d__Bacteria;k__norank;p__Proteobacteria;c__Gammaproteobacteria;o__Enterobacterales;f__Erwiniaceae;g__Pantoea;s__Pantoea_deleyi
PB1_gene_4127	69	SJZ53640.1	69	69/69(100.00)	3.28e-43	cold-shock DNA-binding protein family [Pantoea agglomerans]	470933	d__Bacteria;k__norank;p__Proteobacteria;c__Gammaproteobacteria;o__Enterobacterales;f__Erwiniaceae;g__Pantoea;s__Pantoea_eucalypti
PB1_gene_4137	67	WP_111138868.1	64	63/64(98.44)	6.31e-33	YoaH family protein [Pantoea sp. ARC607]	2027922	d__Bacteria;k__norank;p__Proteobacteria;c__Gammaproteobacteria;o__Enterobacterales;f__Erwiniaceae;g__Pantoea;s__Pantoea_sp._ARC607
PB1_gene_4139	119	WP_111138870.1	121	116/119(97.48)	5.41e-79	hypothetical protein [Pantoea sp. ARC607]	2027922	d__Bacteria;k__norank;p__Proteobacteria;c__Gammaproteobacteria;o__Enterobacterales;f__Erwiniaceae;g__Pantoea;s__Pantoea_sp._ARC607
PB1_gene_4141	255	ORM85660.1	255	244/255(95.69)	8.47e-183	gamma-glutamyl-gamma-aminobutyrate hydrolase [Pantoea deleyi]	470932	d__Bacteria;k__norank;p__Proteobacteria;c__Gammaproteobacteria;o__Enterobacterales;f__Erwiniaceae;g__Pantoea;s__Pantoea_deleyi
PB1_gene_4143	204	ORM86813.1	204	201/204(98.53)	1.59e-139	Holliday junction DNA helicase RuvA [Pantoea deleyi]	470932	d__Bacteria;k__norank;p__Proteobacteria;c__Gammaproteobacteria;o__Enterobacterales;f__Erwiniaceae;g__Pantoea;s__Pantoea_deleyi
PB1_gene_4144	175	ORM86812.1	175	175/175(100.00)	4.36e-116	crossover junction endodeoxyribonuclease RuvC [Pantoea deleyi]	470932	d__Bacteria;k__norank;p__Proteobacteria;c__Gammaproteobacteria;o__Enterobacterales;f__Erwiniaceae;g__Pantoea;s__Pantoea_deleyi
PB1_gene_4148	275	WP_099350309.1	394	219/269(81.41)	9.87e-146	hypothetical protein [Erwinia amylovora]	552	d__Bacteria;k__norank;p__Proteobacteria;c__Gammaproteobacteria;o__Enterobacterales;f__Erwiniaceae;g__Erwinia;s__Erwinia_amylovora
PB1_gene_4149	157	WP_094111772.1	2334	143/154(92.86)	3.31e-80	filamentous hemagglutinin N-terminal domain-containing protein, partial [Pantoea eucrina]	472693	d__Bacteria;k__norank;p__Proteobacteria;c__Gammaproteobacteria;o__Enterobacterales;f__Erwiniaceae;g__Pantoea;s__Pantoea_eucrina
PB1_gene_4151	182	WP_033731858.1	182	180/182(98.90)	2.50e-123	replication initiation negative regulator SeqA [Pantoea vagans]	470934	d__Bacteria;k__norank;p__Proteobacteria;c__Gammaproteobacteria;o__Enterobacterales;f__Erwiniaceae;g__Pantoea;s__Pantoea_vagans
PB1_gene_4167	77	WP_111141722.1	82	57/67(85.07)	1.11e-32	hypothetical protein [Pantoea sp. ARC607]	2027922	d__Bacteria;k__norank;p__Proteobacteria;c__Gammaproteobacteria;o__Enterobacterales;f__Erwiniaceae;g__Pantoea;s__Pantoea_sp._ARC607
PB1_gene_4168	62	WP_074400013.1	62	52/62(83.87)	1.34e-30	hypothetical protein [Pantoea agglomerans]	None	Unclassified

PB1_gene_4172	176	ORM77281.1	176	170/176(96.59)	4.85e-109	transcriptional regulator [Pantoea deleyi]	470932	d__Bacteria;k__norank;p__Proteobacteria;c__Gammaproteobacteria;o__Enterobacterales;f__Erwiniaceae;g__Pantoea;s__Pantoea_deleyi
PB1_gene_4173	98	ORM77280.1	98	97/98(98.98)	7.31e-69	selenoprotein W-related protein [Pantoea deleyi]	470932	d__Bacteria;k__norank;p__Proteobacteria;c__Gammaproteobacteria;o__Enterobacterales;f__Erwiniaceae;g__Pantoea;s__Pantoea_deleyi
PB1_gene_4177	357	ORM75855.1	357	352/357(98.60)	1.35e-253	porin OmpC [Pantoea deleyi]	470932	d__Bacteria;k__norank;p__Proteobacteria;c__Gammaproteobacteria;o__Enterobacterales;f__Erwiniaceae;g__Pantoea;s__Pantoea_deleyi
PB1_gene_4186	127	WP_128081731.1	346	109/134(81.34)	7.58e-68	hypothetical protein [Wolbachia endosymbiont of Dactylopius coccus]	None	Unclassified
PB1_gene_4187	259	ORM81126.1	238	238/238(100.00)	5.74e-178	succinate dehydrogenase iron-sulfur subunit [Pantoea deleyi]	470932	d__Bacteria;k__norank;p__Proteobacteria;c__Gammaproteobacteria;o__Enterobacterales;f__Erwiniaceae;g__Pantoea;s__Pantoea_deleyi
PB1_gene_4191	127	ORM84905.1	127	126/127(99.21)	8.82e-86	autonomous glycyl radical cofactor GrcA [Pantoea deleyi]	470932	d__Bacteria;k__norank;p__Proteobacteria;c__Gammaproteobacteria;o__Enterobacterales;f__Erwiniaceae;g__Pantoea;s__Pantoea_deleyi
PB1_gene_4196	234	ORM82459.1	234	227/234(97.01)	8.38e-159	hypothetical protein HA42_08140 [Pantoea deleyi]	470932	d__Bacteria;k__norank;p__Proteobacteria;c__Gammaproteobacteria;o__Enterobacterales;f__Erwiniaceae;g__Pantoea;s__Pantoea_deleyi
PB1_gene_4206	244	SUB15380.1	257	240/244(98.36)	5.08e-168	P-protein [Pantoea agglomerans]	549	d__Bacteria;k__norank;p__Proteobacteria;c__Gammaproteobacteria;o__Enterobacterales;f__Erwiniaceae;g__Pantoea;s__Pantoea_agglomerans
PB1_gene_4207	198	AAA24868.1	336	195/198(98.48)	6.29e-136	prephenate dehydrogenase, partial [Pantoea agglomerans]	549	d__Bacteria;k__norank;p__Proteobacteria;c__Gammaproteobacteria;o__Enterobacterales;f__Erwiniaceae;g__Pantoea;s__Pantoea_agglomerans
PB1_gene_4209	457	WP_107025452.1	612	98/299(32.78)	4.18e-17	hypothetical protein [Pseudomonas sp. BBP2017]	2109731	d__Bacteria;k__norank;p__Proteobacteria;c__Gammaproteobacteria;o__Pseudomonadales;f__Pseudomonadaceae;g__Pseudomonas;s__Pseudomonas_sp._BBP2017
PB1_gene_4212	432	ORM79170.1	432	430/432(99.54)	3.06e-313	adenylosuccinate synthase [Pantoea deleyi]	470932	d__Bacteria;k__norank;p__Proteobacteria;c__Gammaproteobacteria;o__Enterobacterales;f__Erwiniaceae;g__Pantoea;s__Pantoea_deleyi
PB1_gene_4220	265	PQL28734.1	264	197/209(94.26)	4.60e-111	cell division protein CpoB [Pantoea ananatis]	553	d__Bacteria;k__norank;p__Proteobacteria;c__Gammaproteobacteria;o__Enterobacterales;f__Erwiniaceae;g__Pantoea;s__Pantoea_ananatis
PB1_gene_4223	220	ORM80475.1	220	218/220(99.09)	1.57e-147	hypothetical protein HA42_14430 [Pantoea deleyi]	470932	d__Bacteria;k__norank;p__Proteobacteria;c__Gammaproteobacteria;o__Enterobacterales;f__Erwiniaceae;g__Pantoea;s__Pantoea_deleyi
PB1_gene_4226	252	ORM76055.1	252	250/252(99.21)	1.07e-179	DeoR/GlpR family transcriptional regulator [Pantoea deleyi]	470932	d__Bacteria;k__norank;p__Proteobacteria;c__Gammaproteobacteria;o__Enterobacterales;f__Erwiniaceae;g__Pantoea;s__Pantoea_deleyi
PB1_gene_4228	184	ORM84724.1	183	182/183(99.45)	5.06e-126	NAD(P)H-dependent FMN reductase [Pantoea deleyi]	470932	d__Bacteria;k__norank;p__Proteobacteria;c__Gammaproteobacteria;o__Enterobacterales;f__Erwiniaceae;g__Pantoea;s__Pantoea_deleyi
PB1_gene_4230	262	ORM81579.1	262	257/262(98.09)	1.11e-186	MltA-interacting MipA family protein [Pantoea deleyi]	470932	d__Bacteria;k__norank;p__Proteobacteria;c__Gammaproteobacteria;o__Enterobacterales;f__Erwiniaceae;g__Pantoea;s__Pantoea_deleyi
PB1_gene_4241	78	AVT58164.1	78	74/78(94.87)	1.84e-44	Post-segregation antitoxin CcdA [Pectobacterium carotovorum]	2488639	d__Bacteria;k__norank;p__Proteobacteria;c__Gammaproteobacteria;o__Enterobacterales;f__Pectobacteriaceae;g__Pectobacterium;s__Pectobacterium_versatile
PB1_gene_4242	70	KHN92618.1	70	63/70(90.00)	6.85e-41	hypothetical protein KKH3_26450 [Pectobacterium carotovorum subsp. actinidiae]	1507808	d__Bacteria;k__norank;p__Proteobacteria;c__Gammaproteobacteria;o__Enterobacterales;f__Pectobacteriaceae;g__Pectobacterium;s__Pectobacterium_actinidiae
PB1_gene_4244	315	CSY96252.1	272	105/190(55.26)	1.71e-59	Uncharacterised protein [Shigella sonnei]	624	d__Bacteria;k__norank;p__Proteobacteria;c__Gammaproteobacteria;o__Enterobacterales;f__Enterobacteriaceae;g__Shigella;s__Shigella_sonnei
PB1_gene_4247	156	ORM83595.1	161	147/155(94.84)	3.94e-104	hypothetical protein HA42_05985 [Pantoea deleyi]	470932	d__Bacteria;k__norank;p__Proteobacteria;c__Gammaproteobacteria;o__Enterobacterales;f__Erwiniaceae;g__Pantoea;s__Pantoea_deleyi
PB1_gene_4254	450	WP_122459656.1	742	290/447(64.88)	1.32e-144	hemagglutinin [Pantoea agglomerans]	549	d__Bacteria;k__norank;p__Proteobacteria;c__Gammaproteobacteria;o__Enterobacterales;f__Erwiniaceae;g__Pantoea;s__Pantoea_agglomerans
PB1_gene_4257	85	KDB19172.1	84	48/49(97.96)	2.13e-28	hypothetical protein wGmm_0972 [Wolbachia endosymbiont of Glossina morsitans morsitans]	1150948	d__Bacteria;k__norank;p__Proteobacteria;c__Alphaproteobacteria;o__Rickettsiales;f__Anaplasmataceae;g__Wolbachia;s__Wolbachia_endosymbiont_of_Glossina_morsitans_morsitans
PB1_gene_4262	202	KEY44262.1	202	196/201(97.51)	8.75e-127	heat shock protein GrpE [Pantoea agglomerans]	549	d__Bacteria;k__norank;p__Proteobacteria;c__Gammaproteobacteria;o__Enterobacterales;f__Erwiniaceae;g__Pantoea;s__Pantoea_agglomerans
PB1_gene_4268	101	OOL08822.1	62	40/41(97.56)	9.37e-16	hypothetical protein BXQ27_34455, partial	548	d__Bacteria;k__norank;p__Proteobacteria;c__Gammaproteobacteria;o__Enterobacterales;f__Enterobacteriaceae;g__Klebsiella;s__Klebsiella_aerogenes

PB1_gene_4278	92	OLU10845.1	92	92/92(100.00)	1.66e-60	30S ribosomal protein S19 [Klebsiella pneumoniae]	573	d__Bacteria;k__norank;p__Proteobacteria;c__Gammaproteobacteria;o__Enterobacterales;f__Enterobacteriaceae;g__Klebsiella;s__Klebsiella_pneumoniae
PB1_gene_4284	277	ORM75382.1	277	276/277(99.64)	5.48e-190	biotin-independent malonate decarboxylase subunit beta [Pantoea deleyi]	470932	d__Bacteria;k__norank;p__Proteobacteria;c__Gammaproteobacteria;o__Enterobacterales;f__Erwiniaceae;g__Pantoea;s__Pantoea_deleyi
PB1_gene_4285	99	WP_111141794.1	99	95/99(95.96)	5.79e-62	malonate decarboxylase acyl carrier protein [Pantoea sp.	2027922	d__Bacteria;k__norank;p__Proteobacteria;c__Gammaproteobacteria;o__Enterobacterales;f__Erwiniaceae;g__Pantoea;s__Pantoea_sp._ARC607
PB1_gene_4287	343	WP_111138481.1	343	336/343(97.96)	5.19e-244	16S rRNA (guanine(1207)-N(2))-methyltransferase RsmC [Pantoea sp. ARC607]	2027922	d__Bacteria;k__norank;p__Proteobacteria;c__Gammaproteobacteria;o__Enterobacterales;f__Erwiniaceae;g__Pantoea;s__Pantoea_sp._ARC607
PB1_gene_4291	222	WP_111141224.1	222	210/222(94.59)	1.11e-132	arginine ABC transporter permease ArtM [Pantoea sp.	2027922	d__Bacteria;k__norank;p__Proteobacteria;c__Gammaproteobacteria;o__Enterobacterales;f__Erwiniaceae;g__Pantoea;s__Pantoea_sp._ARC607
PB1_gene_4293	145	ORM83862.1	145	145/145(100.00)	2.49e-96	NADH-quinone oxidoreductase subunit A [Pantoea deleyi]	470932	d__Bacteria;k__norank;p__Proteobacteria;c__Gammaproteobacteria;o__Enterobacterales;f__Erwiniaceae;g__Pantoea;s__Pantoea_deleyi
PB1_gene_4298	65	ORM84677.1	65	65/65(100.00)	1.39e-40	hypothetical protein HA42_03800 [Pantoea deleyi]	470932	d__Bacteria;k__norank;p__Proteobacteria;c__Gammaproteobacteria;o__Enterobacterales;f__Erwiniaceae;g__Pantoea;s__Pantoea_deleyi
PB1_gene_4301	122	RLM02045.1	122	119/122(97.54)	3.01e-77	transposase [Gibbsiella quercinecans]	929813	d__Bacteria;k__norank;p__Proteobacteria;c__Gammaproteobacteria;o__Enterobacterales;f__Yersiniaceae;g__Gibbsiella;s__Gibbsiella_quercinecans
PB1_gene_4302	197	KJM47320.1	292	187/197(94.92)	6.16e-135	transposase [[Enterobacter] aerogenes]	548	d__Bacteria;k__norank;p__Proteobacteria;c__Gammaproteobacteria;o__Enterobacterales;f__Enterobacteriaceae;g__Klebsiella;s__Klebsiella_aerogenes
PB1_gene_4306	297	ORM86567.1	297	296/297(99.66)	2.89e-211	site-specific tyrosine recombinase XerD [Pantoea deleyi]	470932	d__Bacteria;k__norank;p__Proteobacteria;c__Gammaproteobacteria;o__Enterobacterales;f__Erwiniaceae;g__Pantoea;s__Pantoea_deleyi
PB1_gene_4308	56	WP_111142007.1	83	38/38(100.00)	3.26e-15	hypothetical protein [Pantoea sp. ARC607]	2027922	d__Bacteria;k__norank;p__Proteobacteria;c__Gammaproteobacteria;o__Enterobacterales;f__Erwiniaceae;g__Pantoea;s__Pantoea_sp._ARC607
PB1_gene_4309	139	ORM81169.1	139	137/139(98.56)	5.11e-93	DNA polymerase V subunit UmuD [Pantoea deleyi]	470932	d__Bacteria;k__norank;p__Proteobacteria;c__Gammaproteobacteria;o__Enterobacterales;f__Erwiniaceae;g__Pantoea;s__Pantoea_deleyi
PB1_gene_4312	182	ORM79697.1	182	179/181(98.90)	1.51e-124	phosphatidylethanolamine-binding protein [Pantoea deleyi]	470932	d__Bacteria;k__norank;p__Proteobacteria;c__Gammaproteobacteria;o__Enterobacterales;f__Erwiniaceae;g__Pantoea;s__Pantoea_deleyi
PB1_gene_4323	232	ORM83021.1	232	228/232(98.28)	1.89e-154	5'-methylthioadenosine/S-adenosylhomocysteine nucleosidase [Pantoea deleyi]	470932	d__Bacteria;k__norank;p__Proteobacteria;c__Gammaproteobacteria;o__Enterobacterales;f__Erwiniaceae;g__Pantoea;s__Pantoea_deleyi
PB1_gene_4328	93	ABV11453.1	181	48/84(57.14)	9.60e-29	hypothetical protein CKO_00290 [Citrobacter koseri ATCC BAA-	290338	d__Bacteria;k__norank;p__Proteobacteria;c__Gammaproteobacteria;o__Enterobacterales;f__Enterobacteriaceae;g__Citrobacter;s__Citrobacter_koseri
PB1_gene_4339	290	ORM82573.1	290	289/290(99.66)	1.13e-209	chemotaxis protein-glutamate O-methyltransferase [Pantoea	470932	d__Bacteria;k__norank;p__Proteobacteria;c__Gammaproteobacteria;o__Enterobacterales;f__Erwiniaceae;g__Pantoea;s__Pantoea_deleyi
PB1_gene_4341	223	ORM84153.1	223	221/223(99.10)	1.59e-162	hypothetical protein HA42_05205 [Pantoea deleyi]	470932	d__Bacteria;k__norank;p__Proteobacteria;c__Gammaproteobacteria;o__Enterobacterales;f__Erwiniaceae;g__Pantoea;s__Pantoea_deleyi
PB1_gene_4342	104	WP_033735285.1	104	94/104(90.38)	1.97e-60	helix-turn-helix domain-containing protein [Pantoea	470934	d__Bacteria;k__norank;p__Proteobacteria;c__Gammaproteobacteria;o__Enterobacterales;f__Erwiniaceae;g__Pantoea;s__Pantoea_vagans
PB1_gene_4346	395	CVA82460.1	229	116/226(51.33)	9.17e-53	Uncharacterised protein [Serratia marcescens]	615	d__Bacteria;k__norank;p__Proteobacteria;c__Gammaproteobacteria;o__Enterobacterales;f__Yersiniaceae;g__Serratia;s__Serratia_marcescens
PB1_gene_4347	346	WP_041079171.1	660	176/340(51.76)	7.16e-88	hypothetical protein [Rickettsia asembonensis]	1068590	d__Bacteria;k__norank;p__Proteobacteria;c__Alphaproteobacteria;o__Rickettsiales;f__Rickettsiaceae;g__Rickettsia;s__Rickettsia_asembonensis
PB1_gene_4349	323	WP_111139590.1	323	321/323(99.38)	6.87e-223	octaprenyl diphosphate synthase [Pantoea sp. ARC607]	2027922	d__Bacteria;k__norank;p__Proteobacteria;c__Gammaproteobacteria;o__Enterobacterales;f__Erwiniaceae;g__Pantoea;s__Pantoea_sp._ARC607
PB1_gene_4352	309	ORM81116.1	309	302/309(97.73)	8.42e-226	hypothetical protein HA42_11765 [Pantoea deleyi]	470932	d__Bacteria;k__norank;p__Proteobacteria;c__Gammaproteobacteria;o__Enterobacterales;f__Erwiniaceae;g__Pantoea;s__Pantoea_deleyi
PB1_gene_4363	149	WP_123346431.1	149	135/149(90.60)	2.65e-93	MULTISPECIES: phage tail protein [Enterobacter]	2608935	d__Bacteria;k__norank;p__Proteobacteria;c__Gammaproteobacteria;o__Enterobacterales;f__Enterobacteriaceae;g__Enterobacter;s__norank
PB1_gene_4366	125	ORM85310.1	125	125/125(100.00)	1.86e-86	hypothetical protein HA42_02925 [Pantoea deleyi]	470932	d__Bacteria;k__norank;p__Proteobacteria;c__Gammaproteobacteria;o__Enterobacterales;f__Erwiniaceae;g__Pantoea;s__Pantoea_deleyi

PB1_gene_4370	266	ORM80842.1	266	266/266(100.00)	1.79e-194	glucosamine-6-phosphate deaminase [Pantoea deleyi]	470932	d__Bacteria;k__norank;p__Proteobacteria;c__Gammaproteobacteria;o__Enterobacterales;f__Erwiniaceae;g__Pantoea;s__Pantoea_deleyi
PB1_gene_4383	147	WP_111138530.1	340	147/147(100.00)	7.33e-98	LacI family DNA-binding transcriptional regulator [Pantoea sp. ARC607]	2027922	d__Bacteria;k__norank;p__Proteobacteria;c__Gammaproteobacteria;o__Enterobacterales;f__Erwiniaceae;g__Pantoea;s__Pantoea_sp._ARC607
PB1_gene_4385	370	ORM78831.1	576	296/303(97.69)	9.73e-193	Cl- channel voltage-gated family protein [Pantoea deleyi]	470932	d__Bacteria;k__norank;p__Proteobacteria;c__Gammaproteobacteria;o__Enterobacterales;f__Erwiniaceae;g__Pantoea;s__Pantoea_deleyi
PB1_gene_4388	80	ORM82136.1	80	79/80(98.75)	6.55e-48	hypothetical protein HA42_09035 [Pantoea deleyi]	470932	d__Bacteria;k__norank;p__Proteobacteria;c__Gammaproteobacteria;o__Enterobacterales;f__Erwiniaceae;g__Pantoea;s__Pantoea_deleyi
PB1_gene_4391	108	CQR76626.1	107	45/87(51.72)	2.51e-13	hypothetical protein BN1385_01477 [Enterobacter	158836	d__Bacteria;k__norank;p__Proteobacteria;c__Gammaproteobacteria;o__Enterobacterales;f__Enterobacteriaceae;g__Enterobacter;s__Enterobacter_hormaechei
PB1_gene_4398	202	WP_111138599.1	202	199/202(98.51)	9.23e-138	outer membrane lipoprotein carrier protein LolA [Pantoea sp.	2027922	d__Bacteria;k__norank;p__Proteobacteria;c__Gammaproteobacteria;o__Enterobacterales;f__Erwiniaceae;g__Pantoea;s__Pantoea_sp._ARC607
PB1_gene_4399	137	ORM75293.1	137	136/137(99.27)	2.27e-95	4-hydroxybenzoyl-CoA thioesterase [Pantoea deleyi]	470932	d__Bacteria;k__norank;p__Proteobacteria;c__Gammaproteobacteria;o__Enterobacterales;f__Erwiniaceae;g__Pantoea;s__Pantoea_deleyi
PB1_gene_4402	223	ORM79885.1	223	212/223(95.07)	4.90e-157	dethiobiotin synthase [Pantoea deleyi]	470932	d__Bacteria;k__norank;p__Proteobacteria;c__Gammaproteobacteria;o__Enterobacterales;f__Erwiniaceae;g__Pantoea;s__Pantoea_deleyi
PB1_gene_4404	82	PBQ25407.1	129	55/55(100.00)	3.40e-28	transposase [Wolbachia pipientis wAus]	2049495	d__Bacteria;k__norank;p__Proteobacteria;c__Alphaproteobacteria;o__Rickettsiales;f__Anaplasmataceae;g__Wolbachia;s__Wolbachia_pipientis
PB1_gene_4410	206	ORM86050.1	343	195/206(94.66)	4.29e-140	hypothetical protein HA42_01655 [Pantoea deleyi]	470932	d__Bacteria;k__norank;p__Proteobacteria;c__Gammaproteobacteria;o__Enterobacterales;f__Erwiniaceae;g__Pantoea;s__Pantoea_deleyi
PB1_gene_4427	59	WP_123346423.1	438	33/84(39.29)	7.89e-07	MULTISPECIES: phage tail sheath family protein	2608935	d__Bacteria;k__norank;p__Proteobacteria;c__Gammaproteobacteria;o__Enterobacterales;f__Enterobacteriaceae;g__Enterobacter;s__norank
PB1_gene_4430	308	WP_085016768.1	302	301/302(99.67)	1.89e-211	MULTISPECIES: inorganic pyrophosphatase	91347	d__Bacteria;k__norank;p__Proteobacteria;c__Gammaproteobacteria;o__Enterobacterales;f__norank;g__norank;s__norank
PB1_gene_4433	162	ORM81209.1	162	160/162(98.77)	1.97e-108	cold-shock protein [Pantoea deleyi]	470932	d__Bacteria;k__norank;p__Proteobacteria;c__Gammaproteobacteria;o__Enterobacterales;f__Erwiniaceae;g__Pantoea;s__Pantoea_deleyi
PB1_gene_4441	131	ORM82731.1	131	127/131(96.95)	6.62e-92	polyketide cyclase [Pantoea deleyi]	470932	d__Bacteria;k__norank;p__Proteobacteria;c__Gammaproteobacteria;o__Enterobacterales;f__Erwiniaceae;g__Pantoea;s__Pantoea_deleyi
PB1_gene_4443	225	KDA95512.1	225	218/225(96.89)	8.25e-141	hypothetical protein T296_04530 [Pantoea agglomerans Eh318]	1408177	d__Bacteria;k__norank;p__Proteobacteria;c__Gammaproteobacteria;o__Enterobacterales;f__Erwiniaceae;g__Pantoea;s__Pantoea_agglomerans
PB1_gene_4451	261	PEI05484.1	451	255/259(98.46)	1.22e-165	GntP family transporter [Pantoea agglomerans]	549	d__Bacteria;k__norank;p__Proteobacteria;c__Gammaproteobacteria;o__Enterobacterales;f__Erwiniaceae;g__Pantoea;s__Pantoea_agglomerans
PB1_gene_4457	147	ORM76104.1	145	142/145(97.93)	1.76e-92	PTS fructose transporter subunit IIA [Pantoea deleyi]	470932	d__Bacteria;k__norank;p__Proteobacteria;c__Gammaproteobacteria;o__Enterobacterales;f__Erwiniaceae;g__Pantoea;s__Pantoea_deleyi
PB1_gene_4458	89	ORM76105.1	89	83/89(93.26)	1.84e-49	hypothetical protein HA42_19490 [Pantoea deleyi]	470932	d__Bacteria;k__norank;p__Proteobacteria;c__Gammaproteobacteria;o__Enterobacterales;f__Erwiniaceae;g__Pantoea;s__Pantoea_deleyi
PB1_gene_4463	100	ORM81055.1	100	99/100(99.00)	1.36e-56	anti-sigma-28 factor FlgM [Pantoea deleyi]	470932	d__Bacteria;k__norank;p__Proteobacteria;c__Gammaproteobacteria;o__Enterobacterales;f__Erwiniaceae;g__Pantoea;s__Pantoea_deleyi
PB1_gene_4469	279	WP_111139702.1	279	273/279(97.85)	7.05e-203	taurine dioxygenase [Pantoea sp. ARC607]	2027922	d__Bacteria;k__norank;p__Proteobacteria;c__Gammaproteobacteria;o__Enterobacterales;f__Erwiniaceae;g__Pantoea;s__Pantoea_sp._ARC607
PB1_gene_4475	172	ORM84714.1	172	172/172(100.00)	1.22e-125	3-hydroxyacyl-[acyl-carrier-protein] dehydratase FabA	470932	d__Bacteria;k__norank;p__Proteobacteria;c__Gammaproteobacteria;o__Enterobacterales;f__Erwiniaceae;g__Pantoea;s__Pantoea_deleyi
PB1_gene_4479	134	ORM81058.1	134	134/134(100.00)	1.81e-86	flagellar basal body rod protein FlgC [Pantoea deleyi]	470932	d__Bacteria;k__norank;p__Proteobacteria;c__Gammaproteobacteria;o__Enterobacterales;f__Erwiniaceae;g__Pantoea;s__Pantoea_deleyi
PB1_gene_4480	157	ADD76631.1	157	144/157(91.72)	1.44e-94	FlgB [Pantoea ananatis LMG 20103]	706191	d__Bacteria;k__norank;p__Proteobacteria;c__Gammaproteobacteria;o__Enterobacterales;f__Erwiniaceae;g__Pantoea;s__Pantoea_ananatis
PB1_gene_4483	253	WP_126528128.1	254	194/251(77.29)	2.98e-148	N-acetylmuramoyl-L-alanine amidase [Serratia plymuthica]	82996	d__Bacteria;k__norank;p__Proteobacteria;c__Gammaproteobacteria;o__Enterobacterales;f__Yersiniaceae;g__Serratia;s__Serratia_plymuthica
PB1_gene_4489	229	WP_123346417.1	229	175/226(77.43)	6.41e-124	MULTISPECIES: hypothetical protein [Enterobacter]	2608935	d__Bacteria;k__norank;p__Proteobacteria;c__Gammaproteobacteria;o__Enterobacterales;f__Enterobacteriaceae;g__Enterobacter;s__norank

PB1_gene_4494	150	ORM84192.1	150	128/150(85.33)	2.98e-95	hypothetical protein HA38_15440 [Pantoea allii]	574096	d__Bacteria;k__norank;p__Proteobacteria;c__Gammaproteobacteria;o__Enterobacterales;f__Erwiniaceae;g__Pantoea;s__Pantoea_allii
PB1_gene_4506	225	ORM79039.1	225	224/225(99.56)	6.32e-145	amino acid ABC transporter permease [Pantoea deleyi]	470932	d__Bacteria;k__norank;p__Proteobacteria;c__Gammaproteobacteria;o__Enterobacterales;f__Erwiniaceae;g__Pantoea;s__Pantoea_deleyi
PB1_gene_4511	131	ORM80811.1	130	128/130(98.46)	2.15e-90	transcriptional regulator [Pantoea deleyi]	470932	d__Bacteria;k__norank;p__Proteobacteria;c__Gammaproteobacteria;o__Enterobacterales;f__Erwiniaceae;g__Pantoea;s__Pantoea_deleyi
PB1_gene_4512	160	EHM53212.1	224	50/125(40.00)	7.34e-18	hypothetical protein HMPREF9080_01895 [Cardiobacterium valvarum	797473	d__Bacteria;k__norank;p__Proteobacteria;c__Gammaproteobacteria;o__Cardiobacteriales;f__Cardiobacteriaceae;g__Cardiobacterium;s__Cardiobacterium_valvarum
PB1_gene_4519	184	ORM81593.1	182	168/182(92.31)	1.12e-106	fimbrial protein [Pantoea deleyi]	470932	d__Bacteria;k__norank;p__Proteobacteria;c__Gammaproteobacteria;o__Enterobacterales;f__Erwiniaceae;g__Pantoea;s__Pantoea_deleyi
PB1_gene_4528	196	ADD78617.1	195	170/196(86.73)	1.16e-121	YgjP [Pantoea ananatis LMG 20103]	706191	d__Bacteria;k__norank;p__Proteobacteria;c__Gammaproteobacteria;o__Enterobacterales;f__Erwiniaceae;g__Pantoea;s__Pantoea_ananatis
PB1_gene_4532	254	ORM81546.1	254	254/254(100.00)	6.24e-189	phospholipid-binding lipoprotein MlaA [Pantoea deleyi]	470932	d__Bacteria;k__norank;p__Proteobacteria;c__Gammaproteobacteria;o__Enterobacterales;f__Erwiniaceae;g__Pantoea;s__Pantoea_deleyi
PB1_gene_4534	326	WP_128085322.1	325	321/325(98.77)	4.83e-222	taurine ABC transporter substrate-binding protein	None	Unclassified
PB1_gene_4538	190	ORM83128.1	185	185/185(100.00)	1.76e-119	hypothetical protein HA42_06785 [Pantoea deleyi]	470932	d__Bacteria;k__norank;p__Proteobacteria;c__Gammaproteobacteria;o__Enterobacterales;f__Erwiniaceae;g__Pantoea;s__Pantoea_deleyi
PB1_gene_4544	228	AUX74599.1	193	179/192(93.23)	1.58e-120	DNA invertase (plasmid) [Erwinia pyrifoliae]	79967	d__Bacteria;k__norank;p__Proteobacteria;c__Gammaproteobacteria;o__Enterobacterales;f__Erwiniaceae;g__Erwinia;s__Erwinia_pyrifoliae
PB1_gene_4545	83	WP_089562408.1	272	80/83(96.39)	3.94e-46	MULTISPECIES: DUF4225 domain-containing protein	53335	d__Bacteria;k__norank;p__Proteobacteria;c__Gammaproteobacteria;o__Enterobacterales;f__Erwiniaceae;g__Pantoea;s__norank
PB1_gene_4548	210	ORM84465.1	210	209/210(99.52)	1.65e-144	TetR family transcriptional regulator [Pantoea deleyi]	470932	d__Bacteria;k__norank;p__Proteobacteria;c__Gammaproteobacteria;o__Enterobacterales;f__Erwiniaceae;g__Pantoea;s__Pantoea_deleyi
PB1_gene_4553	229	EJK92299.1	203	79/151(52.32)	6.57e-45	hypothetical protein UUU_09050 [Klebsiella pneumoniae subsp. pneumoniae DSM 30104]	1162296	d__Bacteria;k__norank;p__Proteobacteria;c__Gammaproteobacteria;o__Enterobacterales;f__Enterobacteriaceae;g__Klebsiella;s__Klebsiella_pneumoniae
PB1_gene_4560	174	PBQ30189.1	174	173/174(99.43)	1.34e-105	cation:proton antiporter [Wolbachia pipientis wAus]	2049495	d__Bacteria;k__norank;p__Proteobacteria;c__Alphaproteobacteria;o__Rickettsiales;f__Anaplasmataceae;g__Wolbachia;s__Wolbachia_pipientis
PB1_gene_4566	356	WP_111141680.1	356	356/356(100.00)	1.19e-251	porin OmpA [Pantoea sp. ARC607]	2027922	d__Bacteria;k__norank;p__Proteobacteria;c__Gammaproteobacteria;o__Enterobacterales;f__Erwiniaceae;g__Pantoea;s__Pantoea_sp._ARC607
PB1_gene_4568	147	ORM77355.1	147	145/147(98.64)	7.97e-94	hypothetical protein HA42_17915 [Pantoea deleyi]	470932	d__Bacteria;k__norank;p__Proteobacteria;c__Gammaproteobacteria;o__Enterobacterales;f__Erwiniaceae;g__Pantoea;s__Pantoea_deleyi
PB1_gene_4569	74	EFM19264.1	74	72/74(97.30)	5.01e-47	Hemolysin expression modulating family protein [Pantoea sp. aB]	517433	d__Bacteria;k__norank;p__Proteobacteria;c__Gammaproteobacteria;o__Enterobacterales;f__Erwiniaceae;g__Pantoea;s__Pantoea_sp._aB
PB1_gene_4573	292	WP_111139808.1	290	276/290(95.17)	1.09e-187	transcriptional regulator HexR [Pantoea sp. ARC607]	2027922	d__Bacteria;k__norank;p__Proteobacteria;c__Gammaproteobacteria;o__Enterobacterales;f__Erwiniaceae;g__Pantoea;s__Pantoea_sp._ARC607
PB1_gene_4575	90	WP_111141148.1	90	85/90(94.44)	1.06e-53	hypothetical protein [Pantoea sp. ARC607]	2027922	d__Bacteria;k__norank;p__Proteobacteria;c__Gammaproteobacteria;o__Enterobacterales;f__Erwiniaceae;g__Pantoea;s__Pantoea_sp._ARC607
PB1_gene_4576	76	SJZ45140.1	76	76/76(100.00)	3.92e-45	protein of unknown function [Pantoea agglomerans]	470933	d__Bacteria;k__norank;p__Proteobacteria;c__Gammaproteobacteria;o__Enterobacterales;f__Erwiniaceae;g__Pantoea;s__Pantoea_eucalypti
PB1_gene_4580	390	WP_049012351.1	420	384/390(98.46)	1.32e-284	site-specific integrase [Enterobacter kobei]	208224	d__Bacteria;k__norank;p__Proteobacteria;c__Gammaproteobacteria;o__Enterobacterales;f__Enterobacteriaceae;g__Enterobacter;s__Enterobacter_kobei
PB1_gene_4586	337	ARF48495.1	337	326/337(96.74)	1.42e-238	3-dehydro-L-gulonate 2-dehydrogenase [Pantoea stewartii subsp. stewartii DC283]	660596	d__Bacteria;k__norank;p__Proteobacteria;c__Gammaproteobacteria;o__Enterobacterales;f__Erwiniaceae;g__Pantoea;s__Pantoea_stewartii
PB1_gene_4592	71	WP_123220337.1	146	67/71(94.37)	8.48e-41	DUF87 domain-containing protein, partial [Salmonella	28901	d__Bacteria;k__norank;p__Proteobacteria;c__Gammaproteobacteria;o__Enterobacterales;f__Enterobacteriaceae;g__Salmonella;s__Salmonella_enterica
PB1_gene_4595	207	WP_086905640.1	218	140/207(67.63)	5.40e-103	hypothetical protein [Pantoea agglomerans]	549	d__Bacteria;k__norank;p__Proteobacteria;c__Gammaproteobacteria;o__Enterobacterales;f__Erwiniaceae;g__Pantoea;s__Pantoea_agglomerans

PB1_gene_4603	72	ORM80515.1	72	71/71(100.00)	3.58e-46	hypothetical protein HA42_14235 [Pantoea deleyi]	470932	d__Bacteria;k__norank;p__Proteobacteria;c__Gammaproteobacteria;o__Enterobacterales;f__Erwiniaceae;g__Pantoea;s__Pantoea_deleyi
PB1_gene_4610	338	WP_111139355.1	338	331/338(97.93)	3.77e-239	sulfate ABC transporter substrate-binding protein [Pantoea sp. ARC607]	2027922	d__Bacteria;k__norank;p__Proteobacteria;c__Gammaproteobacteria;o__Enterobacterales;f__Erwiniaceae;g__Pantoea;s__Pantoea_sp._ARC607
PB1_gene_4621	87	WP_111138602.1	384	80/87(91.95)	6.34e-46	beta-ketoacyl-[acyl-carrier-protein] synthase family protein [Pantoea sp. ARC607]	2027922	d__Bacteria;k__norank;p__Proteobacteria;c__Gammaproteobacteria;o__Enterobacterales;f__Erwiniaceae;g__Pantoea;s__Pantoea_sp._ARC607
PB1_gene_4622	190	ORM74226.1	190	184/190(96.84)	3.37e-125	hypothetical protein HA42_21600 [Pantoea deleyi]	470932	d__Bacteria;k__norank;p__Proteobacteria;c__Gammaproteobacteria;o__Enterobacterales;f__Erwiniaceae;g__Pantoea;s__Pantoea_deleyi
PB1_gene_4623	122	AVV37391.1	768	116/121(95.87)	7.81e-60	hypothetical protein C9381_09415 [Pantoea vagans]	470934	d__Bacteria;k__norank;p__Proteobacteria;c__Gammaproteobacteria;o__Enterobacterales;f__Erwiniaceae;g__Pantoea;s__Pantoea_vagans
PB1_gene_4639	248	PZL87202.1	248	245/248(98.79)	8.79e-177	transpeptidase [Pantoea sp. ARC270]	2027923	d__Bacteria;k__norank;p__Proteobacteria;c__Gammaproteobacteria;o__Enterobacterales;f__Erwiniaceae;g__Pantoea;s__Pantoea_sp._ARC270
PB1_gene_4649	100	ORM84684.1	231	89/96(92.71)	1.85e-63	extensin [Pantoea deleyi]	470932	d__Bacteria;k__norank;p__Proteobacteria;c__Gammaproteobacteria;o__Enterobacterales;f__Erwiniaceae;g__Pantoea;s__Pantoea_deleyi
PB1_gene_4652	213	WP_090392808.1	539	207/213(97.18)	2.47e-145	type VI secretion system protein TssA [Enterobacter sp. NFIX58]	1566251	d__Bacteria;k__norank;p__Proteobacteria;c__Gammaproteobacteria;o__Enterobacterales;f__Enterobacteriaceae;g__Enterobacter;s__Enterobacter_sp._NFIX58
PB1_gene_4657	43	WP_120455517.1	163	22/42(52.38)	3.56e-07	type VI secretion system tube protein Hcp [Erwiniaceae bacterium IIIF1SW-P2]	2364647	d__Bacteria;k__norank;p__Proteobacteria;c__Gammaproteobacteria;o__Enterobacterales;f__Erwiniaceae;g__Kalamiella;s__Kalamiella_piersonii
PB1_gene_4658	89	WP_046101148.1	97	70/78(89.74)	1.21e-42	hypothetical protein [Pantoea anthophila]	470931	d__Bacteria;k__norank;p__Proteobacteria;c__Gammaproteobacteria;o__Enterobacterales;f__Erwiniaceae;g__Pantoea;s__Pantoea_anthophila
PB1_gene_4661	244	ORM76533.1	244	244/244(100.00)	2.44e-181	helix-turn-helix-type transcriptional regulator [Pantoea	470932	d__Bacteria;k__norank;p__Proteobacteria;c__Gammaproteobacteria;o__Enterobacterales;f__Erwiniaceae;g__Pantoea;s__Pantoea_deleyi
PB1_gene_4669	229	ORM82299.1	229	220/229(96.07)	1.17e-142	hypothetical protein HA42_08710 [Pantoea deleyi]	470932	d__Bacteria;k__norank;p__Proteobacteria;c__Gammaproteobacteria;o__Enterobacterales;f__Erwiniaceae;g__Pantoea;s__Pantoea_deleyi
PB1_gene_4672	306	ORM80517.1	306	302/306(98.69)	2.17e-206	LysR family transcriptional regulator [Pantoea deleyi]	470932	d__Bacteria;k__norank;p__Proteobacteria;c__Gammaproteobacteria;o__Enterobacterales;f__Erwiniaceae;g__Pantoea;s__Pantoea_deleyi
PB1_gene_4676	219	ORM80725.1	219	219/219(100.00)	8.65e-140	glutamine ABC transporter permease GlnP [Pantoea deleyi]	470932	d__Bacteria;k__norank;p__Proteobacteria;c__Gammaproteobacteria;o__Enterobacterales;f__Erwiniaceae;g__Pantoea;s__Pantoea_deleyi
PB1_gene_4683	112	SJZ71349.1	112	112/112(100.00)	7.70e-73	nitrogen regulatory protein P-II family [Pantoea agglomerans]	470933	d__Bacteria;k__norank;p__Proteobacteria;c__Gammaproteobacteria;o__Enterobacterales;f__Erwiniaceae;g__Pantoea;s__Pantoea_eucalypti
PB1_gene_4691	97	ORM81134.1	97	93/97(95.88)	2.04e-61	cyd operon protein YbgE [Pantoea deleyi]	470932	d__Bacteria;k__norank;p__Proteobacteria;c__Gammaproteobacteria;o__Enterobacterales;f__Erwiniaceae;g__Pantoea;s__Pantoea_deleyi
PB1_gene_4692	134	ORM81135.1	134	132/134(98.51)	7.37e-86	tol-pal system-associated acyl-CoA thioesterase [Pantoea deleyi]	470932	d__Bacteria;k__norank;p__Proteobacteria;c__Gammaproteobacteria;o__Enterobacterales;f__Erwiniaceae;g__Pantoea;s__Pantoea_deleyi
PB1_gene_4697	139	WP_111139911.1	139	139/139(100.00)	6.98e-100	thioredoxin TrxC [Pantoea sp. ARC607]	2027922	d__Bacteria;k__norank;p__Proteobacteria;c__Gammaproteobacteria;o__Enterobacterales;f__Erwiniaceae;g__Pantoea;s__Pantoea_sp._ARC607
PB1_gene_4703	82	WP_111140505.1	82	81/82(98.78)	2.25e-49	type II toxin-antitoxin system antitoxin HipB [Pantoea sp.	2027922	d__Bacteria;k__norank;p__Proteobacteria;c__Gammaproteobacteria;o__Enterobacterales;f__Erwiniaceae;g__Pantoea;s__Pantoea_sp._ARC607
PB1_gene_4714	140	SQK72294.1	144	102/139(73.38)	3.33e-63	Uncharacterised protein [Tatumella ptyseos]	82987	d__Bacteria;k__norank;p__Proteobacteria;c__Gammaproteobacteria;o__Enterobacterales;f__Erwiniaceae;g__Tatumella;s__Tatumella_ptyseos
PB1_gene_4715	51	WP_107318792.1	180	34/44(77.27)	4.61e-15	hypothetical protein, partial [Klebsiella pneumoniae]	573	d__Bacteria;k__norank;p__Proteobacteria;c__Gammaproteobacteria;o__Enterobacterales;f__Enterobacteriaceae;g__Klebsiella;s__Klebsiella_pneumoniae
PB1_gene_4719	106	SFO51531.1	106	94/106(88.68)	9.65e-62	hypothetical protein SAMN05428971_4306 [Pantoea sp. OV426]	1881036	d__Bacteria;k__norank;p__Proteobacteria;c__Gammaproteobacteria;o__Enterobacterales;f__Erwiniaceae;g__Pantoea;s__Pantoea_sp._OV426
PB1_gene_4722	171	ORM84690.1	171	164/170(96.47)	1.08e-114	pyrimidine utilization flavin reductase protein F [Pantoea	470932	d__Bacteria;k__norank;p__Proteobacteria;c__Gammaproteobacteria;o__Enterobacterales;f__Erwiniaceae;g__Pantoea;s__Pantoea_deleyi

PB1_gene_4725	252	ORM78256.1	251	244/251(97.21)	1.78e-180	hydroxyacylglutathione hydrolase [Pantoea deleyi]	470932	d__Bacteria;k__norank;p__Proteobacteria;c__Gammaproteobacteria;o__Enterobacterales;f__Erwiniaceae;g__Pantoea;s__Pantoea_deleyi
PB1_gene_4751	201	WP_089562464.1	449	191/201(95.02)	1.15e-114	MULTISPECIES: flagellin FliC [Pantoea]	53335	d__Bacteria;k__norank;p__Proteobacteria;c__Gammaproteobacteria;o__Enterobacterales;f__Erwiniaceae;g__Pantoea;s__norank
PB1_gene_4752	56	EIB98452.1	471	53/56(94.64)	1.22e-21	flagellar capping protein [Pantoea sp. Sc1]	593105	d__Bacteria;k__norank;p__Proteobacteria;c__Gammaproteobacteria;o__Enterobacterales;f__Erwiniaceae;g__Pantoea;s__Pantoea_sp._Sc1
PB1_gene_4753	314	SBH69708.1	440	121/269(44.98)	9.84e-63	Uncharacterised protein [Klebsiella pneumoniae]	1463165	d__Bacteria;k__norank;p__Proteobacteria;c__Gammaproteobacteria;o__Enterobacterales;f__Enterobacteriaceae;g__Klebsiella;s__Klebsiella_quasipneumoniae
PB1_gene_4762	192	ORM77357.1	192	192/192(100.00)	5.58e-125	electron transport complex subunit RsxB [Pantoea deleyi]	470932	d__Bacteria;k__norank;p__Proteobacteria;c__Gammaproteobacteria;o__Enterobacterales;f__Erwiniaceae;g__Pantoea;s__Pantoea_deleyi
PB1_gene_4767	132	KTR98997.1	132	74/132(56.06)	6.16e-45	hypothetical protein NS375_11770 [Pantoea dispersa]	59814	d__Bacteria;k__norank;p__Proteobacteria;c__Gammaproteobacteria;o__Enterobacterales;f__Erwiniaceae;g__Pantoea;s__Pantoea_dispersa
PB1_gene_4779	111	SJZ29942.1	111	108/111(97.30)	3.68e-75	5-hydroxyisourate hydrolase [Pantoea agglomerans]	470933	d__Bacteria;k__norank;p__Proteobacteria;c__Gammaproteobacteria;o__Enterobacterales;f__Erwiniaceae;g__Pantoea;s__Pantoea_eucalypti
PB1_gene_4793	160	ORM81550.1	160	157/160(98.12)	2.92e-109	phosphohistidine phosphatase SixA [Pantoea deleyi]	470932	d__Bacteria;k__norank;p__Proteobacteria;c__Gammaproteobacteria;o__Enterobacterales;f__Erwiniaceae;g__Pantoea;s__Pantoea_deleyi
PB1_gene_4797	178	ORM85227.1	178	173/178(97.19)	8.36e-111	chorismate mutase [Pantoea deleyi]	470932	d__Bacteria;k__norank;p__Proteobacteria;c__Gammaproteobacteria;o__Enterobacterales;f__Erwiniaceae;g__Pantoea;s__Pantoea_deleyi
PB1_gene_4803	187	ORM82059.1	187	187/187(100.00)	1.34e-134	peroxiredoxin [Pantoea deleyi]	470932	d__Bacteria;k__norank;p__Proteobacteria;c__Gammaproteobacteria;o__Enterobacterales;f__Erwiniaceae;g__Pantoea;s__Pantoea_deleyi
PB1_gene_4813	184	ORM76865.1	184	181/184(98.37)	4.45e-118	hypothetical protein HA42_18595 [Pantoea deleyi]	470932	d__Bacteria;k__norank;p__Proteobacteria;c__Gammaproteobacteria;o__Enterobacterales;f__Erwiniaceae;g__Pantoea;s__Pantoea_deleyi
PB1_gene_4817	222	ORM79528.1	222	212/222(95.50)	4.01e-151	2-deoxyglucose-6-phosphatase [Pantoea deleyi]	470932	d__Bacteria;k__norank;p__Proteobacteria;c__Gammaproteobacteria;o__Enterobacterales;f__Erwiniaceae;g__Pantoea;s__Pantoea_deleyi
PB1_gene_4820	206	ORM86101.1	206	203/206(98.54)	1.26e-146	threonylcarbamoyl-AMP synthase [Pantoea deleyi]	470932	d__Bacteria;k__norank;p__Proteobacteria;c__Gammaproteobacteria;o__Enterobacterales;f__Erwiniaceae;g__Pantoea;s__Pantoea_deleyi
PB1_gene_4825	145	WP_067436139.1	145	125/144(86.81)	1.81e-89	hypothetical protein [Erwinia gerundensis]	1619313	d__Bacteria;k__norank;p__Proteobacteria;c__Gammaproteobacteria;o__Enterobacterales;f__Erwiniaceae;g__Erwinia;s__Erwinia_gerundensis
PB1_gene_4843	269	ORM81634.1	277	266/269(98.88)	5.25e-195	carboxymethylenebutenolidase [Pantoea deleyi]	470932	d__Bacteria;k__norank;p__Proteobacteria;c__Gammaproteobacteria;o__Enterobacterales;f__Erwiniaceae;g__Pantoea;s__Pantoea_deleyi
PB1_gene_4850	91	ORM79282.1	91	91/91(100.00)	2.56e-59	hypothetical protein HA42_15700 [Pantoea deleyi]	470932	d__Bacteria;k__norank;p__Proteobacteria;c__Gammaproteobacteria;o__Enterobacterales;f__Erwiniaceae;g__Pantoea;s__Pantoea_deleyi
PB1_gene_4854	280	SAI94527.1	421	54/121(44.63)	7.40e-12	Uncharacterised protein [Enterobacter cloacae]	550	d__Bacteria;k__norank;p__Proteobacteria;c__Gammaproteobacteria;o__Enterobacterales;f__Enterobacteriaceae;g__Enterobacter;s__Enterobacter_cloacae
PB1_gene_4856	97	ORM84123.1	97	95/97(97.94)	2.69e-57	hypothetical protein HA42_05025 [Pantoea deleyi]	470932	d__Bacteria;k__norank;p__Proteobacteria;c__Gammaproteobacteria;o__Enterobacterales;f__Erwiniaceae;g__Pantoea;s__Pantoea_deleyi
PB1_gene_4857	81	ORM84124.1	81	80/81(98.77)	2.87e-47	two-component-system connector protein YcgZ [Pantoea deleyi]	470932	d__Bacteria;k__norank;p__Proteobacteria;c__Gammaproteobacteria;o__Enterobacterales;f__Erwiniaceae;g__Pantoea;s__Pantoea_deleyi
PB1_gene_4869	98	OQV39488.1	98	98/98(100.00)	1.05e-59	transcriptional regulator [Pantoea vagans]	470934	d__Bacteria;k__norank;p__Proteobacteria;c__Gammaproteobacteria;o__Enterobacterales;f__Erwiniaceae;g__Pantoea;s__Pantoea_vagans
PB1_gene_4870	195	WP_111141305.1	195	191/194(98.45)	5.36e-129	HD domain-containing protein [Pantoea sp. ARC607]	2027922	d__Bacteria;k__norank;p__Proteobacteria;c__Gammaproteobacteria;o__Enterobacterales;f__Erwiniaceae;g__Pantoea;s__Pantoea_sp._ARC607
PB1_gene_4872	320	ORM83257.1	320	307/320(95.94)	1.50e-220	thiamine biosynthesis protein ApbE [Pantoea deleyi]	470932	d__Bacteria;k__norank;p__Proteobacteria;c__Gammaproteobacteria;o__Enterobacterales;f__Erwiniaceae;g__Pantoea;s__Pantoea_deleyi
PB1_gene_4875	347	OMP53686.1	347	347/347(100.00)	2.37e-242	rod shape-determining protein [Serratia marcescens]	615	d__Bacteria;k__norank;p__Proteobacteria;c__Gammaproteobacteria;o__Enterobacterales;f__Yersiniaceae;g__Serratia;s__Serratia_marcescens
PB1_gene_4878	203	EJT84489.1	144	61/111(54.95)	4.01e-31	hypothetical protein PPS11_21259 [Pseudomonas	1218169	d__Bacteria;k__norank;p__Proteobacteria;c__Gammaproteobacteria;o__Pseudomonadales;f__Pseudomonadaceae;g__Pseudomonas;s__Pseudomonas_putida
PB1_gene_4885	186	ORM86800.1	186	179/186(96.24)	1.34e-129	metalloprotein [Pantoea deleyi]	470932	d__Bacteria;k__norank;p__Proteobacteria;c__Gammaproteobacteria;o__Enterobacterales;f__Erwiniaceae;g__Pantoea;s__Pantoea_deleyi

PB1_gene_4901	137	ORM81200.1	137	134/136(98.53)	3.76e-94	ribonucleotide reductase assembly protein NrdI [Pantoea	470932	d__Bacteria;k__norank;p__Proteobacteria;c__Gammaproteobacteria;o__Enterobacterales;f__Erwiniaceae;g__Pantoea;s__Pantoea_deleyi
PB1_gene_4902	80	ORM81201.1	80	75/80(93.75)	2.77e-49	NrdH-redoxin [Pantoea deleyi]	470932	d__Bacteria;k__norank;p__Proteobacteria;c__Gammaproteobacteria;o__Enterobacterales;f__Erwiniaceae;g__Pantoea;s__Pantoea_deleyi
PB1_gene_4905	165	ORM78372.1	165	165/165(100.00)	8.97e-111	Fe-S cluster assembly transcriptional regulator IscR	470932	d__Bacteria;k__norank;p__Proteobacteria;c__Gammaproteobacteria;o__Enterobacterales;f__Erwiniaceae;g__Pantoea;s__Pantoea_deleyi
PB1_gene_4913	282	OXH80879.1	282	278/282(98.58)	2.77e-206	carbon-phosphorus lyase complex subunit PhnJ [Pantoea	549	d__Bacteria;k__norank;p__Proteobacteria;c__Gammaproteobacteria;o__Enterobacterales;f__Erwiniaceae;g__Pantoea;s__Pantoea_agglomerans
PB1_gene_4925	327	WP_111140739.1	327	319/327(97.55)	4.34e-228	oxidoreductase [Pantoea sp. ARC607]	2027922	d__Bacteria;k__norank;p__Proteobacteria;c__Gammaproteobacteria;o__Enterobacterales;f__Erwiniaceae;g__Pantoea;s__Pantoea_sp._ARC607
PB1_gene_4929	180	WP_111140575.1	180	164/180(91.11)	1.63e-118	HutD family protein [Pantoea sp. ARC607]	2027922	d__Bacteria;k__norank;p__Proteobacteria;c__Gammaproteobacteria;o__Enterobacterales;f__Erwiniaceae;g__Pantoea;s__Pantoea_sp._ARC607
PB1_gene_4941	94	WP_036011915.1	306	31/96(32.29)	1.47e-06	3-hydroxyacyl-CoA dehydrogenase [Bradyrhizobium	108015	d__Bacteria;k__norank;p__Proteobacteria;c__Alphaproteobacteria;o__Rhizobiales;f__Bradyrhizobiaceae;g__Bradyrhizobium;s__Bradyrhizobium_yuanmingense
PB1_gene_4944	214	WP_039833213.1	302	213/214(99.53)	1.63e-146	acetylglutamate kinase [Wolbachia endosymbiont of Nasonia vitripennis]	180837	d__Bacteria;k__norank;p__Proteobacteria;c__Alphaproteobacteria;o__Rickettsiales;f__Anaplasmataceae;g__Wolbachia;s__Wolbachia_endosymbiont_of_Nasonia_vitripennis
PB1_gene_4950	107	ORM82383.1	112	98/107(91.59)	1.27e-63	hypothetical protein HA42_08450 [Pantoea deleyi]	470932	d__Bacteria;k__norank;p__Proteobacteria;c__Gammaproteobacteria;o__Enterobacterales;f__Erwiniaceae;g__Pantoea;s__Pantoea_deleyi
PB1_gene_4953	246	WP_111139544.1	246	229/246(93.09)	1.65e-155	ABC transporter ATP-binding protein [Pantoea sp. ARC607]	2027922	d__Bacteria;k__norank;p__Proteobacteria;c__Gammaproteobacteria;o__Enterobacterales;f__Erwiniaceae;g__Pantoea;s__Pantoea_sp._ARC607
PB1_gene_4968	165	ORM84705.1	152	150/152(98.68)	5.06e-105	methylglyoxal synthase [Pantoea deleyi]	470932	d__Bacteria;k__norank;p__Proteobacteria;c__Gammaproteobacteria;o__Enterobacterales;f__Erwiniaceae;g__Pantoea;s__Pantoea_deleyi
PB1_gene_4973	138	ORM86109.1	138	136/138(98.55)	2.74e-101	ferredoxin [Pantoea deleyi]	470932	d__Bacteria;k__norank;p__Proteobacteria;c__Gammaproteobacteria;o__Enterobacterales;f__Erwiniaceae;g__Pantoea;s__Pantoea_deleyi
PB1_gene_4980	50	OCA06005.1	41	34/41(82.93)	3.08e-14	hypothetical protein wTpre_326 [Wolbachia endosymbiont of Trichogramma pretiosum]	125593	d__Bacteria;k__norank;p__Proteobacteria;c__Alphaproteobacteria;o__Rickettsiales;f__Anaplasmataceae;g__Wolbachia;s__Wolbachia_endosymbiont_of_Trichogramma_pretiosum
PB1_gene_4982	160	WP_111139032.1	160	160/160(100.00)	4.32e-111	zinc ribbon-containing protein [Pantoea sp. ARC607]	53335	d__Bacteria;k__norank;p__Proteobacteria;c__Gammaproteobacteria;o__Enterobacterales;f__Erwiniaceae;g__Pantoea;s__norank
PB1_gene_4985	111	ORM80553.1	111	111/111(100.00)	3.41e-71	ribosomal subunit interface protein [Pantoea deleyi]	470932	d__Bacteria;k__norank;p__Proteobacteria;c__Gammaproteobacteria;o__Enterobacterales;f__Erwiniaceae;g__Pantoea;s__Pantoea_deleyi
PB1_gene_4987	299	WP_019236681.1	390	297/299(99.33)	1.35e-216	NADH-quinone oxidoreductase subunit D [Wolbachia pipientis]	955	d__Bacteria;k__norank;p__Proteobacteria;c__Alphaproteobacteria;o__Rickettsiales;f__Anaplasmataceae;g__Wolbachia;s__Wolbachia_pipientis
PB1_gene_4988	91	ORM83726.1	91	89/91(97.80)	2.47e-57	hypothetical protein HA42_05730 [Pantoea deleyi]	470932	d__Bacteria;k__norank;p__Proteobacteria;c__Gammaproteobacteria;o__Enterobacterales;f__Erwiniaceae;g__Pantoea;s__Pantoea_deleyi
PB1_gene_4989	55	ELP25362.1	51	40/50(80.00)	1.76e-18	hypothetical protein F385_1595 [Pantoea agglomerans 299R]	1261128	d__Bacteria;k__norank;p__Proteobacteria;c__Gammaproteobacteria;o__Enterobacterales;f__Erwiniaceae;g__Pantoea;s__Pantoea_agglomerans
PB1_gene_4996	218	WP_081273830.1	217	196/217(90.32)	6.24e-130	MULTISPECIES: hypothetical protein [Pantoea]	53335	d__Bacteria;k__norank;p__Proteobacteria;c__Gammaproteobacteria;o__Enterobacterales;f__Erwiniaceae;g__Pantoea;s__norank
PB1_gene_4999	161	ORM83882.1	161	161/161(100.00)	1.23e-106	colicin V production protein [Pantoea deleyi]	470932	d__Bacteria;k__norank;p__Proteobacteria;c__Gammaproteobacteria;o__Enterobacterales;f__Erwiniaceae;g__Pantoea;s__Pantoea_deleyi
PB1_gene_5006	148	WP_111141389.1	148	146/148(98.65)	4.61e-98	phosphonate C-P lyase system protein PhnG [Pantoea sp.	2027922	d__Bacteria;k__norank;p__Proteobacteria;c__Gammaproteobacteria;o__Enterobacterales;f__Erwiniaceae;g__Pantoea;s__Pantoea_sp._ARC607
PB1_gene_5012	64	ORM85075.1	64	64/64(100.00)	1.10e-41	hypothetical protein HA42_03390 [Pantoea deleyi]	470932	d__Bacteria;k__norank;p__Proteobacteria;c__Gammaproteobacteria;o__Enterobacterales;f__Erwiniaceae;g__Pantoea;s__Pantoea_deleyi
PB1_gene_5013	52	ORM51191.1	52	52/52(100.00)	5.42e-28	proteolipid membrane potential modulator [Pantoea conspicua]	472705	d__Bacteria;k__norank;p__Proteobacteria;c__Gammaproteobacteria;o__Enterobacterales;f__Erwiniaceae;g__Pantoea;s__Pantoea_conspicua
PB1_gene_5025	62	ORM75456.1	62	62/62(100.00)	2.91e-34	YnbE family lipoprotein [Pantoea deleyi]	470932	d__Bacteria;k__norank;p__Proteobacteria;c__Gammaproteobacteria;o__Enterobacterales;f__Erwiniaceae;g__Pantoea;s__Pantoea_deleyi

PB1_gene_5026	114	ORM75455.1	114	113/114(99.12)	2.03e-69	amine metabolic protein ydbL [Pantoea deleyi]	470932	d__Bacteria;k__norank;p__Proteobacteria;c__Gammaproteobacteria;o__Enterobacterales;f__Erwiniaceae;g__Pantoea;s__Pantoea_deleyi
PB1_gene_5028	84	ORM75563.1	84	84/84(100.00)	5.44e-51	acyl carrier protein [Pantoea deleyi]	470932	d__Bacteria;k__norank;p__Proteobacteria;c__Gammaproteobacteria;o__Enterobacterales;f__Erwiniaceae;g__Pantoea;s__Pantoea_deleyi
PB1_gene_5029	85	WP_111138592.1	85	85/85(100.00)	3.96e-49	acyl carrier protein [Pantoea sp. ARC607]	2027922	d__Bacteria;k__norank;p__Proteobacteria;c__Gammaproteobacteria;o__Enterobacterales;f__Erwiniaceae;g__Pantoea;s__Pantoea_sp._ARC607
PB1_gene_5032	204	PBQ28085.1	240	200/204(98.04)	1.80e-142	50S ribosomal protein L3 [Wolbachia pipientis wAus]	2049495	d__Bacteria;k__norank;p__Proteobacteria;c__Alphaproteobacteria;o__Rickettsiales;f__Anaplasmataceae;g__Wolbachia;s__Wolbachia_pipientis
PB1_gene_5033	112	ONI56352.1	112	102/110(92.73)	1.82e-62	ribosomal protein S10 [Wolbachia pipientis wUni]	1379787	d__Bacteria;k__norank;p__Proteobacteria;c__Alphaproteobacteria;o__Rickettsiales;f__Anaplasmataceae;g__Wolbachia;s__Wolbachia_pipientis
PB1_gene_5036	70	KPA07663.1	70	60/70(85.71)	8.11e-31	putative membrane protein [Pantoea agglomerans]	549	d__Bacteria;k__norank;p__Proteobacteria;c__Gammaproteobacteria;o__Enterobacterales;f__Erwiniaceae;g__Pantoea;s__Pantoea_agglomerans
PB1_gene_5037	222	KPA07662.1	219	204/219(93.15)	5.89e-141	hypothetical protein PAP10c_0975 [Pantoea	549	d__Bacteria;k__norank;p__Proteobacteria;c__Gammaproteobacteria;o__Enterobacterales;f__Erwiniaceae;g__Pantoea;s__Pantoea_agglomerans
PB1_gene_5046	102	SXF49311.1	213	33/102(32.35)	8.19e-08	Uncharacterised protein [Klebsiella variicola]	244366	d__Bacteria;k__norank;p__Proteobacteria;c__Gammaproteobacteria;o__Enterobacterales;f__Enterobacteriaceae;g__Klebsiella;s__Klebsiella_variicola
PB1_gene_5053	172	WP_090963822.1	172	150/172(87.21)	3.03e-101	type 1 fimbrial protein [Pantoea sp. OV426]	1881036	d__Bacteria;k__norank;p__Proteobacteria;c__Gammaproteobacteria;o__Enterobacterales;f__Erwiniaceae;g__Pantoea;s__Pantoea_sp._OV426
PB1_gene_5056	88	WP_111139519.1	88	61/82(74.39)	8.77e-34	hypothetical protein [Pantoea sp. ARC607]	None	Unclassified
PB1_gene_5062	151	WP_111138480.1	136	134/136(98.53)	3.07e-92	DNA polymerase III subunit psi [Pantoea sp. ARC607]	2027922	d__Bacteria;k__norank;p__Proteobacteria;c__Gammaproteobacteria;o__Enterobacterales;f__Erwiniaceae;g__Pantoea;s__Pantoea_sp._ARC607
PB1_gene_5063	146	ORM80540.1	146	142/146(97.26)	7.69e-96	ribosomal-protein-alanine N-acetyltransferase [Pantoea deleyi]	470932	d__Bacteria;k__norank;p__Proteobacteria;c__Gammaproteobacteria;o__Enterobacterales;f__Erwiniaceae;g__Pantoea;s__Pantoea_deleyi
PB1_gene_5073	106	CGW45829.1	102	36/89(40.45)	3.92e-16	Uncharacterised protein [Salmonella enterica subsp. enterica serovar Typhi]	90370	d__Bacteria;k__norank;p__Proteobacteria;c__Gammaproteobacteria;o__Enterobacterales;f__Enterobacteriaceae;g__Salmonella;s__Salmonella_enterica
PB1_gene_5076	216	WP_096097255.1	394	211/216(97.69)	1.37e-141	hypothetical protein [Wolbachia pipientis]	955	d__Bacteria;k__norank;p__Proteobacteria;c__Alphaproteobacteria;o__Rickettsiales;f__Anaplasmataceae;g__Wolbachia;s__Wolbachia_pipientis
PB1_gene_5080	209	ORM78497.1	209	206/209(98.56)	2.24e-137	DNA-binding response regulator [Pantoea deleyi]	470932	d__Bacteria;k__norank;p__Proteobacteria;c__Gammaproteobacteria;o__Enterobacterales;f__Erwiniaceae;g__Pantoea;s__Pantoea_deleyi
PB1_gene_5086	112	WP_128085513.1	139	53/84(63.10)	8.89e-24	DUF1090 family protein [Pantoea deleyi]	470932	d__Bacteria;k__norank;p__Proteobacteria;c__Gammaproteobacteria;o__Enterobacterales;f__Erwiniaceae;g__Pantoea;s__Pantoea_deleyi
PB1_gene_5087	128	WP_096073981.1	400	98/128(76.56)	4.89e-55	AAA family ATPase [Pantoea sp. FDAARGOS_194]	None	Unclassified
PB1_gene_5101	152	WP_128083999.1	154	152/152(100.00)	2.52e-102	ribosome maturation factor RimP [Pantoea deleyi]	None	Unclassified
PB1_gene_5116	40	WP_123803255.1	450	39/40(97.50)	2.25e-18	DUF87 domain-containing protein [Pantoea sp. RIT388]	1869313	d__Bacteria;k__norank;p__Proteobacteria;c__Gammaproteobacteria;o__Enterobacterales;f__Erwiniaceae;g__Pantoea;s__Pantoea_sp._RIT388
PB1_gene_5118	68	WP_039659145.1	68	57/67(85.07)	1.00e-30	hypothetical protein [Pantoea sp. MBLJ3]	53335	d__Bacteria;k__norank;p__Proteobacteria;c__Gammaproteobacteria;o__Enterobacterales;f__Erwiniaceae;g__Pantoea;s__norank
PB1_gene_5128	81	RLT62200.1	53	49/53(92.45)	3.09e-26	hypothetical protein WANA13_0161 [Wolbachia endosymbiont of Drosophila	307502	d__Bacteria;k__norank;p__Proteobacteria;c__Alphaproteobacteria;o__Rickettsiales;f__Anaplasmataceae;g__Wolbachia;s__Wolbachia_endosymbiont_of_Drosophila_ananassae
PB1_gene_5132	192	ORM83263.1	191	185/191(96.86)	1.23e-130	TetR family transcriptional regulator [Pantoea deleyi]	470932	d__Bacteria;k__norank;p__Proteobacteria;c__Gammaproteobacteria;o__Enterobacterales;f__Erwiniaceae;g__Pantoea;s__Pantoea_deleyi
PB1_gene_5141	167	WP_090958504.1	157	137/157(87.26)	2.42e-91	hypothetical protein [Pantoea sp. OV426]	1881036	d__Bacteria;k__norank;p__Proteobacteria;c__Gammaproteobacteria;o__Enterobacterales;f__Erwiniaceae;g__Pantoea;s__Pantoea_sp._OV426
PB1_gene_5165	96	KGQ20443.1	117	35/85(41.18)	4.22e-13	hypothetical protein LF41_980 [Lysobacter dokdonensis DS-58]	1300345	d__Bacteria;k__norank;p__Proteobacteria;c__Gammaproteobacteria;o__Xanthomonadales;f__Xanthomonadaceae;g__Lysobacter;s__Lysobacter_dokdonensis

PB1_gene_5174	309	SSM19634.1	101	52/98(53.06)	7.45e-21	Uncharacterised protein [Klebsiella pneumoniae]	573	d__Bacteria;k__norank;p__Proteobacteria;c__Gammaproteobacteria;o__Enterobacterales;f__Enterobacteriaceae;g__Klebsiella;s__Klebsiella_pneumoniae
PB1_gene_5177	149	WP_101700226.1	149	138/149(92.62)	1.95e-95	type VI secretion system baseplate subunit TssE	1981983	d__Bacteria;k__norank;p__Proteobacteria;c__Gammaproteobacteria;o__Enterobacterales;f__Enterobacteriaceae;g__Citrobacter;s__Citrobacter_sp._L55
PB1_gene_5183	293	ORM82125.1	293	293/293(100.00)	2.11e-211	transcriptional regulator IlvY [Pantoea deleyi]	470932	d__Bacteria;k__norank;p__Proteobacteria;c__Gammaproteobacteria;o__Enterobacterales;f__Erwiniaceae;g__Pantoea;s__Pantoea_deleyi
PB1_gene_5186	137	WP_111139259.1	136	135/136(99.26)	3.93e-89	DoxX family protein [Pantoea sp. ARC607]	2027922	d__Bacteria;k__norank;p__Proteobacteria;c__Gammaproteobacteria;o__Enterobacterales;f__Erwiniaceae;g__Pantoea;s__Pantoea_sp._ARC607
PB1_gene_5187	94	ORM77145.1	94	92/94(97.87)	3.87e-60	hypothetical protein HA42_18225 [Pantoea deleyi]	470932	d__Bacteria;k__norank;p__Proteobacteria;c__Gammaproteobacteria;o__Enterobacterales;f__Erwiniaceae;g__Pantoea;s__Pantoea_deleyi
PB1_gene_5191	187	ORM82840.1	199	185/187(98.93)	1.41e-100	penicillin-binding protein activator LpoB [Pantoea deleyi]	470932	d__Bacteria;k__norank;p__Proteobacteria;c__Gammaproteobacteria;o__Enterobacterales;f__Erwiniaceae;g__Pantoea;s__Pantoea_deleyi
PB1_gene_5200	274	WP_111141410.1	274	270/274(98.54)	2.27e-201	penicillin-insensitive murein endopeptidase [Pantoea sp. ARC607]	2027922	d__Bacteria;k__norank;p__Proteobacteria;c__Gammaproteobacteria;o__Enterobacterales;f__Erwiniaceae;g__Pantoea;s__Pantoea_sp._ARC607
PB1_gene_5206	335	ORM80534.1	333	308/310(99.35)	1.33e-221	NADPH:quinone reductase [Pantoea deleyi]	470932	d__Bacteria;k__norank;p__Proteobacteria;c__Gammaproteobacteria;o__Enterobacterales;f__Erwiniaceae;g__Pantoea;s__Pantoea_deleyi
PB1_gene_5208	259	ORM80472.1	259	253/259(97.68)	5.96e-185	AraC family transcriptional regulator [Pantoea deleyi]	470932	d__Bacteria;k__norank;p__Proteobacteria;c__Gammaproteobacteria;o__Enterobacterales;f__Erwiniaceae;g__Pantoea;s__Pantoea_deleyi
PB1_gene_5225	83	WP_078804583.1	336	80/83(96.39)	4.44e-44	Zn-dependent oxidoreductase [Pantoea agglomerans]	470933	d__Bacteria;k__norank;p__Proteobacteria;c__Gammaproteobacteria;o__Enterobacterales;f__Erwiniaceae;g__Pantoea;s__Pantoea_eucalypti
PB1_gene_5234	164	ORM77141.1	224	164/164(100.00)	4.95e-112	hypothetical protein HA42_18200 [Pantoea deleyi]	470932	d__Bacteria;k__norank;p__Proteobacteria;c__Gammaproteobacteria;o__Enterobacterales;f__Erwiniaceae;g__Pantoea;s__Pantoea_deleyi
PB1_gene_5242	331	WP_111140628.1	383	321/331(96.98)	3.14e-231	iron ABC transporter substrate-binding protein [Pantoea sp. ARC607]	2027922	d__Bacteria;k__norank;p__Proteobacteria;c__Gammaproteobacteria;o__Enterobacterales;f__Erwiniaceae;g__Pantoea;s__Pantoea_sp._ARC607
PB1_gene_5247	118	WP_064085664.1	149	112/149(75.17)	8.11e-43	ankyrin repeat domain-containing protein [Wolbachia endosymbiont of Dactylopius coccus]	None	Unclassified
PB1_gene_5269	156	AAA24582.1	176	154/156(98.72)	2.61e-110	RNA polymerase beta subunit (rpoC) (gtg start codon) (EC 2.7.7.6), partial [Escherichia coli]	562	d__Bacteria;k__norank;p__Proteobacteria;c__Gammaproteobacteria;o__Enterobacterales;f__Enterobacteriaceae;g__Escherichia;s__Escherichia_coli
PB1_gene_5281	328	WP_111140327.1	836	316/328(96.34)	1.65e-207	copper-exporting P-type ATPase CopA [Pantoea sp. ARC607]	2027922	d__Bacteria;k__norank;p__Proteobacteria;c__Gammaproteobacteria;o__Enterobacterales;f__Erwiniaceae;g__Pantoea;s__Pantoea_sp._ARC607
PB1_gene_5297	111	SJZ73797.1	111	111/111(100.00)	3.15e-76	Protein of unknown function [Pantoea agglomerans]	470933	d__Bacteria;k__norank;p__Proteobacteria;c__Gammaproteobacteria;o__Enterobacterales;f__Erwiniaceae;g__Pantoea;s__Pantoea_eucalypti
PB1_gene_5298	105	ORM81202.1	105	105/105(100.00)	4.80e-66	hypothetical protein HA42_11500 [Pantoea deleyi]	470932	d__Bacteria;k__norank;p__Proteobacteria;c__Gammaproteobacteria;o__Enterobacterales;f__Erwiniaceae;g__Pantoea;s__Pantoea_deleyi
PB1_gene_5300	146	WP_111142204.1	146	134/146(91.78)	3.01e-93	DUF1795 domain-containing protein [Pantoea sp. ARC607]	2027922	d__Bacteria;k__norank;p__Proteobacteria;c__Gammaproteobacteria;o__Enterobacterales;f__Erwiniaceae;g__Pantoea;s__Pantoea_sp._ARC607
PB1_gene_5305	110	OIC88333.1	98	57/94(60.64)	7.96e-26	hypothetical protein A7M48_20540 [Acinetobacter	470	d__Bacteria;k__norank;p__Proteobacteria;c__Gammaproteobacteria;o__Pseudomonadales;f__Moraxellaceae;g__Acinetobacter;s__Acinetobacter_baumannii
PB1_gene_5310	100	KEA50682.1	311	70/95(73.68)	2.45e-41	hypothetical protein DT73_21755 [Mangrovibacter sp. MFB070]	1224318	d__Bacteria;k__norank;p__Proteobacteria;c__Gammaproteobacteria;o__Enterobacterales;f__Enterobacteriaceae;g__Mangrovibacter;s__Mangrovibacter_sp._MFB070
PB1_gene_5311	71	KEA50683.1	71	51/70(72.86)	1.23e-30	hypothetical protein DT73_21760 [Mangrovibacter sp. MFB070]	1224318	d__Bacteria;k__norank;p__Proteobacteria;c__Gammaproteobacteria;o__Enterobacterales;f__Enterobacteriaceae;g__Mangrovibacter;s__Mangrovibacter_sp._MFB070
PB1_gene_5318	121	ORM78387.1	130	32/40(80.00)	1.52e-14	hypothetical protein HA42_16745 [Pantoea deleyi]	470932	d__Bacteria;k__norank;p__Proteobacteria;c__Gammaproteobacteria;o__Enterobacterales;f__Erwiniaceae;g__Pantoea;s__Pantoea_deleyi
PB1_gene_5324	74	WP_031378135.1	74	61/74(82.43)	6.94e-36	hypothetical protein [Pantoea sp. 3.5.1]	1522060	d__Bacteria;k__norank;p__Proteobacteria;c__Gammaproteobacteria;o__Enterobacterales;f__Erwiniaceae;g__Pantoea;s__Pantoea_sp._3.5.1

PB1_gene_5327	139	AXL94553.1	174	63/100(63.00)	1.79e-35	adenylate kinase, partial [Escherichia coli]	562	d__Bacteria;k__norank;p__Proteobacteria;c__Gammaproteobacteria;o__Enterobacterales;f__Enterobacteriaceae;g__Escherichia;s__Escherichia_coli
PB1_gene_5342	57	WP_111138394.1	313	57/57(100.00)	1.39e-29	ABC transporter substrate-binding protein [Pantoea sp.	2027922	d__Bacteria;k__norank;p__Proteobacteria;c__Gammaproteobacteria;o__Enterobacterales;f__Erwiniaceae;g__Pantoea;s__Pantoea_sp._ARC607
PB1_gene_5343	172	WP_111138395.1	172	167/172(97.09)	6.25e-119	GNAT family N-acetyltransferase [Pantoea sp. ARC607]	2027922	d__Bacteria;k__norank;p__Proteobacteria;c__Gammaproteobacteria;o__Enterobacterales;f__Erwiniaceae;g__Pantoea;s__Pantoea_sp._ARC607
PB1_gene_5346	85	ORM81909.1	85	84/85(98.82)	3.14e-46	hypothetical protein HA42_09500 [Pantoea deleyi]	470932	d__Bacteria;k__norank;p__Proteobacteria;c__Gammaproteobacteria;o__Enterobacterales;f__Erwiniaceae;g__Pantoea;s__Pantoea_deleyi
PB1_gene_5347	86	ORM81908.1	85	82/85(96.47)	6.13e-42	hypothetical protein HA42_09495 [Pantoea deleyi]	470932	d__Bacteria;k__norank;p__Proteobacteria;c__Gammaproteobacteria;o__Enterobacterales;f__Erwiniaceae;g__Pantoea;s__Pantoea_deleyi
PB1_gene_5352	68	WP_075211886.1	75	54/64(84.38)	1.04e-31	hypothetical protein [Klebsiella pneumoniae]	None	Unclassified
PB1_gene_5353	96	TCZ24204.1	96	72/96(75.00)	5.21e-41	hypothetical protein EYB39_17615 [Pantoea	549	d__Bacteria;k__norank;p__Proteobacteria;c__Gammaproteobacteria;o__Enterobacterales;f__Erwiniaceae;g__Pantoea;s__Pantoea_agglomerans
PB1_gene_5370	118	WP_053092177.1	373	118/118(100.00)	2.45e-77	translesion error-prone DNA polymerase V subunit UmuC [Pantoea vagans]	None	Unclassified
PB1_gene_5371	86	ORM90765.1	86	85/86(98.84)	7.63e-52	DNA polymerase III subunit theta [Pantoea septica]	472695	d__Bacteria;k__norank;p__Proteobacteria;c__Gammaproteobacteria;o__Enterobacterales;f__Erwiniaceae;g__Pantoea;s__Pantoea_septica
PB1_gene_5377	306	WP_111139995.1	306	302/306(98.69)	1.19e-219	2-dehydropantoate 2-reductase [Pantoea sp. ARC607]	2027922	d__Bacteria;k__norank;p__Proteobacteria;c__Gammaproteobacteria;o__Enterobacterales;f__Erwiniaceae;g__Pantoea;s__Pantoea_sp._ARC607
PB1_gene_5388	254	ORM85214.1	254	243/254(95.67)	3.58e-179	MerR family transcriptional regulator [Pantoea deleyi]	470932	d__Bacteria;k__norank;p__Proteobacteria;c__Gammaproteobacteria;o__Enterobacterales;f__Erwiniaceae;g__Pantoea;s__Pantoea_deleyi
PB1_gene_5389	85	ORM81278.1	85	77/85(90.59)	2.04e-51	hypothetical protein HA42_11470 [Pantoea deleyi]	470932	d__Bacteria;k__norank;p__Proteobacteria;c__Gammaproteobacteria;o__Enterobacterales;f__Erwiniaceae;g__Pantoea;s__Pantoea_deleyi
PB1_gene_5390	184	ORM81279.1	184	177/184(96.20)	3.27e-120	gamma carbonic anhydrase family protein [Pantoea deleyi]	470932	d__Bacteria;k__norank;p__Proteobacteria;c__Gammaproteobacteria;o__Enterobacterales;f__Erwiniaceae;g__Pantoea;s__Pantoea_deleyi
PB1_gene_5394	283	WP_114599321.1	355	246/283(86.93)	1.35e-185	arginine kinase [Microbacterium arborescens]	33883	d__Bacteria;k__norank;p__Actinobacteria;c__Actinobacteria;o__Micrococcales;f__Microbacteriaceae;g__Microbacterium;s__Microbacterium_arborescens
PB1_gene_5398	88	WP_095706365.1	88	84/87(96.55)	7.56e-44	envelope stress response protein PspG [Pantoea vagans]	470934	d__Bacteria;k__norank;p__Proteobacteria;c__Gammaproteobacteria;o__Enterobacterales;f__Erwiniaceae;g__Pantoea;s__Pantoea_vagans
PB1_gene_5408	56	PXW15544.1	56	40/56(71.43)	5.45e-23	hypothetical protein BY447_4055 [Pantoea sp. JKS000250]	1938795	d__Bacteria;k__norank;p__Proteobacteria;c__Gammaproteobacteria;o__Enterobacterales;f__Erwiniaceae;g__Pantoea;s__Pantoea_sp._JKS000250
PB1_gene_5410	260	AGJ98862.1	485	222/260(85.38)	1.16e-144	Ankyrin repeat domain protein [Wolbachia endosymbiont of Drosophila simulans wNo]	1236908	d__Bacteria;k__norank;p__Proteobacteria;c__Alphaproteobacteria;o__Rickettsiales;f__Anaplasmataceae;g__Wolbachia;s__Wolbachia_endosymbiont_of_Drosophila_simulans
PB1_gene_5414	268	BAK13746.1	723	262/268(97.76)	7.20e-183	5-methylcytosine-specific restriction enzyme subunit McrB [Pantoea ananatis AJ13355]	932677	d__Bacteria;k__norank;p__Proteobacteria;c__Gammaproteobacteria;o__Enterobacterales;f__Erwiniaceae;g__Pantoea;s__Pantoea_ananatis
PB1_gene_5415	39	WP_054688213.1	404	35/39(89.74)	9.28e-14	restriction endonuclease [Pantoea stewartii]	66269	d__Bacteria;k__norank;p__Proteobacteria;c__Gammaproteobacteria;o__Enterobacterales;f__Erwiniaceae;g__Pantoea;s__Pantoea_stewartii
PB1_gene_5419	98	ORM99034.1	98	94/98(95.92)	1.42e-66	type IV conjugative transfer system protein TraL [Pantoea	472695	d__Bacteria;k__norank;p__Proteobacteria;c__Gammaproteobacteria;o__Enterobacterales;f__Erwiniaceae;g__Pantoea;s__Pantoea_septica
PB1_gene_5420	190	WP_039390119.1	190	183/190(96.32)	3.14e-130	hypothetical protein [Pantoea agglomerans]	549	d__Bacteria;k__norank;p__Proteobacteria;c__Gammaproteobacteria;o__Enterobacterales;f__Erwiniaceae;g__Pantoea;s__Pantoea_agglomerans
PB1_gene_5436	299	ORM81405.1	367	284/299(94.98)	2.23e-198	ABC transporter [Pantoea deleyi]	470932	d__Bacteria;k__norank;p__Proteobacteria;c__Gammaproteobacteria;o__Enterobacterales;f__Erwiniaceae;g__Pantoea;s__Pantoea_deleyi
PB1_gene_5437	279	ORM76961.1	279	276/279(98.92)	3.86e-191	RpiR family transcriptional regulator [Pantoea deleyi]	470932	d__Bacteria;k__norank;p__Proteobacteria;c__Gammaproteobacteria;o__Enterobacterales;f__Erwiniaceae;g__Pantoea;s__Pantoea_deleyi

PB1_gene_5446	83	CDQ16061.1	368	31/60(51.67)	3.74e-06	hypothetical protein KQQSB11_380072 [Klebsiella quasipneumoniae subsp. quasipneumoniae]	1667327	d__Bacteria;k__norank;p__Proteobacteria;c__Gammaproteobacteria;o__Enterobacterales;f__Enterobacteriaceae;g__Klebsiella;s__Klebsiella_quasipneumoniae
PB1_gene_5454	70	RID23772.1	72	69/70(98.57)	2.71e-45	DNA helicase, partial [Escherichia coli]	562	d__Bacteria;k__norank;p__Proteobacteria;c__Gammaproteobacteria;o__Enterobacterales;f__Enterobacteriaceae;g__Escherichia;s__Escherichia_coli
PB1_gene_5455	159	ORM82935.1	159	159/159(100.00)	1.69e-103	AsnC family transcriptional regulator [Pantoea deleyi]	470932	d__Bacteria;k__norank;p__Proteobacteria;c__Gammaproteobacteria;o__Enterobacterales;f__Erwiniaceae;g__Pantoea;s__Pantoea_deleyi
PB1_gene_5462	81	ORM82928.1	70	69/70(98.57)	5.78e-45	hypothetical protein HA42_07315 [Pantoea deleyi]	470932	d__Bacteria;k__norank;p__Proteobacteria;c__Gammaproteobacteria;o__Enterobacterales;f__Erwiniaceae;g__Pantoea;s__Pantoea_deleyi
PB1_gene_5463	173	ORM82929.1	173	169/173(97.69)	1.07e-115	transcriptional regulator Zur [Pantoea deleyi]	470932	d__Bacteria;k__norank;p__Proteobacteria;c__Gammaproteobacteria;o__Enterobacterales;f__Erwiniaceae;g__Pantoea;s__Pantoea_deleyi
PB1_gene_5466	104	KPA06335.1	107	94/104(90.38)	1.78e-59	hypothetical protein PAP10c_2437 [Pantoea	549	d__Bacteria;k__norank;p__Proteobacteria;c__Gammaproteobacteria;o__Enterobacterales;f__Erwiniaceae;g__Pantoea;s__Pantoea_agglomerans
PB1_gene_5467	43	RAW82612.1	132	27/35(77.14)	4.24e-07	phage tail protein [Photorhabdus sp. S5P8-50]	2029687	d__Bacteria;k__norank;p__Proteobacteria;c__Gammaproteobacteria;o__Enterobacterales;f__Morganellaceae;g__Photorhabdus;s__Photorhabdus_sp._S5P8-50
PB1_gene_5471	68	ORM81499.1	68	66/68(97.06)	1.06e-43	XRE family transcriptional regulator [Pantoea deleyi]	470932	d__Bacteria;k__norank;p__Proteobacteria;c__Gammaproteobacteria;o__Enterobacterales;f__Erwiniaceae;g__Pantoea;s__Pantoea_deleyi
PB1_gene_5474	207	ORM83606.1	190	186/190(97.89)	1.44e-144	phospholipid:lipid A palmitoyltransferase [Pantoea	470932	d__Bacteria;k__norank;p__Proteobacteria;c__Gammaproteobacteria;o__Enterobacterales;f__Erwiniaceae;g__Pantoea;s__Pantoea_deleyi
PB1_gene_5479	270	WP_111139371.1	270	263/269(97.77)	7.73e-182	4-hydroxy-tetrahydrodipicolinate reductase [Pantoea sp. ARC607]	2027922	d__Bacteria;k__norank;p__Proteobacteria;c__Gammaproteobacteria;o__Enterobacterales;f__Erwiniaceae;g__Pantoea;s__Pantoea_sp._ARC607
PB1_gene_5502	258	WP_111140242.1	258	253/258(98.06)	3.88e-185	serine/threonine-protein phosphatase [Pantoea sp.	2027922	d__Bacteria;k__norank;p__Proteobacteria;c__Gammaproteobacteria;o__Enterobacterales;f__Erwiniaceae;g__Pantoea;s__Pantoea_sp._ARC607
PB1_gene_5508	153	ORM81502.1	153	137/153(89.54)	5.52e-97	blasticidin S-acetyltransferase [Pantoea deleyi]	470932	d__Bacteria;k__norank;p__Proteobacteria;c__Gammaproteobacteria;o__Enterobacterales;f__Erwiniaceae;g__Pantoea;s__Pantoea_deleyi
PB1_gene_5520	123	SQT09779.1	141	28/70(40.00)	3.08e-06	Uncharacterised protein [Escherichia coli]	562	d__Bacteria;k__norank;p__Proteobacteria;c__Gammaproteobacteria;o__Enterobacterales;f__Enterobacteriaceae;g__Escherichia;s__Escherichia_coli
PB1_gene_5535	185	WP_105635585.1	185	137/185(74.05)	8.38e-103	hypothetical protein [Cronobacter dublinensis]	413497	d__Bacteria;k__norank;p__Proteobacteria;c__Gammaproteobacteria;o__Enterobacterales;f__Enterobacteriaceae;g__Cronobacter;s__Cronobacter_dublinensis
PB1_gene_5546	222	WP_061068149.1	433	208/222(93.69)	1.52e-139	integrase [Citrobacter sp. FDAARGOS_156]	1702170	d__Bacteria;k__norank;p__Proteobacteria;c__Gammaproteobacteria;o__Enterobacterales;f__Enterobacteriaceae;g__Citrobacter;s__Citrobacter_sp._FDAARGOS_156
PB1_gene_5555	170	SUI47542.1	274	166/170(97.65)	2.62e-99	virulence factor Mce family protein [Serratia marcescens]	615	d__Bacteria;k__norank;p__Proteobacteria;c__Gammaproteobacteria;o__Enterobacterales;f__Yersiniaceae;g__Serratia;s__Serratia_marcescens
PB1_gene_5563	290	ORY26091.1	284	136/291(46.74)	3.16e-70	Golgi phospho protein 3 [Neocallimastix californiae]	1754190	d__Eukaryota;k__Fungi;p__Chytridiomycota;c__Neocallimastigomycetes;o__Neocallimastigales;f__Neocallimastigaceae;g__Neocallimastix;s__Neocallimastix_californiae
PB1_gene_5571	139	SQI28983.1	128	80/128(62.50)	1.67e-50	Uncharacterised protein [Serratia plymuthica]	82996	d__Bacteria;k__norank;p__Proteobacteria;c__Gammaproteobacteria;o__Enterobacterales;f__Yersiniaceae;g__Serratia;s__Serratia_plymuthica
PB1_gene_5580	298	ORM75520.1	465	288/291(98.97)	1.03e-210	hypothetical protein HA42_20455 [Pantoea deleyi]	470932	d__Bacteria;k__norank;p__Proteobacteria;c__Gammaproteobacteria;o__Enterobacterales;f__Erwiniaceae;g__Pantoea;s__Pantoea_deleyi
PB1_gene_5582	114	EIB96875.1	344	96/115(83.48)	4.83e-56	capsid portal protein [Pantoea sp. Sc1]	593105	d__Bacteria;k__norank;p__Proteobacteria;c__Gammaproteobacteria;o__Enterobacterales;f__Erwiniaceae;g__Pantoea;s__Pantoea_sp._Sc1
PB1_gene_5583	93	WP_090965619.1	123	71/93(76.34)	7.38e-41	hypothetical protein [Pantoea sp. OV426]	1881036	d__Bacteria;k__norank;p__Proteobacteria;c__Gammaproteobacteria;o__Enterobacterales;f__Erwiniaceae;g__Pantoea;s__Pantoea_sp._OV426
PB1_gene_5601	252	ORM86021.1	252	247/252(98.02)	2.54e-187	beta-galactosidase [Pantoea deleyi]	470932	d__Bacteria;k__norank;p__Proteobacteria;c__Gammaproteobacteria;o__Enterobacterales;f__Erwiniaceae;g__Pantoea;s__Pantoea_deleyi
PB1_gene_5619	74	ORM80815.1	74	72/74(97.30)	5.66e-44	hypothetical protein HA42_12880 [Pantoea deleyi]	470932	d__Bacteria;k__norank;p__Proteobacteria;c__Gammaproteobacteria;o__Enterobacterales;f__Erwiniaceae;g__Pantoea;s__Pantoea_deleyi
PB1_gene_5621	101	ORM77143.1	101	101/101(100.00)	1.11e-60	hypothetical protein HA42_18215 [Pantoea deleyi]	470932	d__Bacteria;k__norank;p__Proteobacteria;c__Gammaproteobacteria;o__Enterobacterales;f__Erwiniaceae;g__Pantoea;s__Pantoea_deleyi

PB1_gene_5626	70	ORM83266.1	70	70/70(100.00)	3.64e-45	cold-shock protein [Pantoea deleyi]	470932	d__Bacteria;k__norank;p__Proteobacteria;c__Gammaproteobacteria;o__Enterobacterales;f__Erwiniaceae;g__Pantoea;s__Pantoea_deleyi
PB1_gene_5631	211	ORM85074.1	211	211/211(100.00)	1.86e-146	helix-turn-helix transcriptional regulator [Pantoea deleyi]	470932	d__Bacteria;k__norank;p__Proteobacteria;c__Gammaproteobacteria;o__Enterobacterales;f__Erwiniaceae;g__Pantoea;s__Pantoea_deleyi
PB1_gene_5636	205	ORM82654.1	501	129/129(100.00)	1.01e-82	methylmalonate-semialdehyde dehydrogenase (acylating) [Pantoea deleyi]	470932	d__Bacteria;k__norank;p__Proteobacteria;c__Gammaproteobacteria;o__Enterobacterales;f__Erwiniaceae;g__Pantoea;s__Pantoea_deleyi
PB1_gene_5659	209	ORM81097.1	209	205/209(98.09)	1.80e-152	hypothetical protein HA42_11955 [Pantoea deleyi]	470932	d__Bacteria;k__norank;p__Proteobacteria;c__Gammaproteobacteria;o__Enterobacterales;f__Erwiniaceae;g__Pantoea;s__Pantoea_deleyi
PB1_gene_5670	86	AUH00637.1	86	81/86(94.19)	3.38e-46	type II toxin-antitoxin system ParD family antitoxin [Serratia sp. ATCC 39006]	104623	d__Bacteria;k__norank;p__Proteobacteria;c__Gammaproteobacteria;o__Enterobacterales;f__Yersiniaceae;g__Serratia;s__Serratia_sp._ATCC_39006
PB1_gene_5671	107	WP_024964879.1	109	100/107(93.46)	3.43e-65	type II toxin-antitoxin system RelE/ParE family toxin [Pantoea sp. IMH]	1267600	d__Bacteria;k__norank;p__Proteobacteria;c__Gammaproteobacteria;o__Enterobacterales;f__Erwiniaceae;g__Pantoea;s__Pantoea_sp._IMH
PB1_gene_5682	96	GBE70466.1	413	40/75(53.33)	2.77e-10	hypothetical protein EKINANG_21160 [Enterobacter sp. KINAN-G]	1535203	d__Bacteria;k__norank;p__Proteobacteria;c__Gammaproteobacteria;o__Enterobacterales;f__Enterobacteriaceae;g__Enterobacter;s__Enterobacter_sp._KINAN-G
PB1_gene_5724	123	ORM81968.1	106	106/106(100.00)	7.29e-66	hypothetical protein HA42_09235 [Pantoea deleyi]	470932	d__Bacteria;k__norank;p__Proteobacteria;c__Gammaproteobacteria;o__Enterobacterales;f__Erwiniaceae;g__Pantoea;s__Pantoea_deleyi
PB1_gene_5725	283	ORM81207.1	350	282/283(99.65)	1.80e-198	peptide ABC transporter permease [Pantoea deleyi]	470932	d__Bacteria;k__norank;p__Proteobacteria;c__Gammaproteobacteria;o__Enterobacterales;f__Erwiniaceae;g__Pantoea;s__Pantoea_deleyi
PB1_gene_5727	56	CRH33078.1	49	36/43(83.72)	2.32e-14	hypothetical protein BN1183_AP_00530 [Pantoea ananatis]	553	d__Bacteria;k__norank;p__Proteobacteria;c__Gammaproteobacteria;o__Enterobacterales;f__Erwiniaceae;g__Pantoea;s__Pantoea_ananatis
PB1_gene_5737	75	ORM75839.1	75	72/75(96.00)	7.37e-45	hypothetical protein HA42_19890 [Pantoea deleyi]	470932	d__Bacteria;k__norank;p__Proteobacteria;c__Gammaproteobacteria;o__Enterobacterales;f__Erwiniaceae;g__Pantoea;s__Pantoea_deleyi
PB1_gene_5760	65	WP_111208559.1	65	55/65(84.62)	1.59e-28	hypothetical protein [Pantoea sp. ARC270]	None	Unclassified
PB1_gene_5770	101	KEY44581.1	44	40/44(90.91)	9.22e-18	hypothetical protein FB99_30330 [Pantoea agglomerans]	549	d__Bacteria;k__norank;p__Proteobacteria;c__Gammaproteobacteria;o__Enterobacterales;f__Erwiniaceae;g__Pantoea;s__Pantoea_agglomerans
PB1_gene_5776	262	ONI55937.1	635	257/261(98.47)	1.47e-180	NADPH-dependent glutamate synthase beta chain domain protein [Wolbachia pipientis	1379787	d__Bacteria;k__norank;p__Proteobacteria;c__Alphaproteobacteria;o__Rickettsiales;f__Anaplasmataceae;g__Wolbachia;s__Wolbachia_pipientis
PB1_gene_5788	131	WP_033761757.1	131	117/131(89.31)	1.06e-79	head-tail adaptor protein [Pantoea agglomerans]	None	Unclassified
PB1_gene_5792	269	AEW75009.1	208	100/193(51.81)	2.29e-54	hypothetical protein EcWSU1_03581 [Enterobacter	299767	d__Bacteria;k__norank;p__Proteobacteria;c__Gammaproteobacteria;o__Enterobacterales;f__Enterobacteriaceae;g__Enterobacter;s__Enterobacter_ludwigii
PB1_gene_5793	109	ORM84010.1	414	92/96(95.83)	5.09e-57	allophanate hydrolase, partial [Pantoea deleyi]	470932	d__Bacteria;k__norank;p__Proteobacteria;c__Gammaproteobacteria;o__Enterobacterales;f__Erwiniaceae;g__Pantoea;s__Pantoea_deleyi
PB1_gene_5804	277	WP_065095520.1	324	276/277(99.64)	4.87e-202	major capsid protein, partial [Wolbachia endosymbiont of Nomada leucophthalma]	1854758	d__Bacteria;k__norank;p__Proteobacteria;c__Alphaproteobacteria;o__Rickettsiales;f__Anaplasmataceae;g__Wolbachia;s__Wolbachia_endosymbiont_of_Nomada_leucophthalma
PB1_gene_5810	150	AGJ99273.1	141	141/141(100.00)	6.80e-99	Cytidine and deoxycytidylate deaminase family protein [Wolbachia endosymbiont of Drosophila simulans wNo]	1236908	d__Bacteria;k__norank;p__Proteobacteria;c__Alphaproteobacteria;o__Rickettsiales;f__Anaplasmataceae;g__Wolbachia;s__Wolbachia_endosymbiont_of_Drosophila_simulans
PB1_gene_5811	105	WP_048786827.1	2720	79/90(87.78)	1.71e-10	filamentous hemagglutinin N-terminal domain-containing protein, partial [Pantoea vagans]	470934	d__Bacteria;k__norank;p__Proteobacteria;c__Gammaproteobacteria;o__Enterobacterales;f__Erwiniaceae;g__Pantoea;s__Pantoea_vagans

PB1_gene_5817	170	ORM60121.1	404	144/161(89.44)	2.43e-97	phage portal protein [Pantoea rodasii]	1076549	d__Bacteria;k__norank;p__Proteobacteria;c__Gammaproteobacteria;o__Enterobacterales;f__Erwiniaceae;g__Pantoea;s__Pantoea_rodasii
PB1_gene_5818	74	PVY82787.1	186	63/74(85.14)	5.55e-36	hypothetical protein C7427_109187 [Pantoea	553	d__Bacteria;k__norank;p__Proteobacteria;c__Gammaproteobacteria;o__Enterobacterales;f__Erwiniaceae;g__Pantoea;s__Pantoea_ananatis
PB1_gene_5834	274	KPA08757.1	544	254/274(92.70)	2.78e-175	Urea amidolyase [Pantoea agglomerans]	549	d__Bacteria;k__norank;p__Proteobacteria;c__Gammaproteobacteria;o__Enterobacterales;f__Erwiniaceae;g__Pantoea;s__Pantoea_agglomerans
PB1_gene_5845	59	WP_111140075.1	59	57/59(96.61)	5.80e-35	hypothetical protein [Pantoea sp. ARC607]	None	Unclassified
PB1_gene_5846	52	WP_111140074.1	52	52/52(100.00)	1.95e-30	MULTISPECIES: YmiA family putative membrane protein	53335	d__Bacteria;k__norank;p__Proteobacteria;c__Gammaproteobacteria;o__Enterobacterales;f__Erwiniaceae;g__Pantoea;s__norank
PB1_gene_5847	186	ORM83052.1	186	182/186(97.85)	1.98e-131	D-glycero-beta-D-manno-heptose-1,7-bisphosphate 7-phosphatase [Pantoea deleyi]	470932	d__Bacteria;k__norank;p__Proteobacteria;c__Gammaproteobacteria;o__Enterobacterales;f__Erwiniaceae;g__Pantoea;s__Pantoea_deleyi
PB1_gene_5850	178	WP_111140859.1	178	178/178(100.00)	1.07e-122	hypoxanthine phosphoribosyltransferase [Pantoea sp. ARC607]	2027922	d__Bacteria;k__norank;p__Proteobacteria;c__Gammaproteobacteria;o__Enterobacterales;f__Erwiniaceae;g__Pantoea;s__Pantoea_sp._ARC607
PB1_gene_5852	218	WP_130100471.1	336	124/231(53.68)	2.41e-67	phage tail protein [Siccibacter turicensis]	357233	d__Bacteria;k__norank;p__Proteobacteria;c__Gammaproteobacteria;o__Enterobacterales;f__Enterobacteriaceae;g__Siccibacter;s__Siccibacter_turicensis
PB1_gene_5854	86	WP_061062828.1	86	80/86(93.02)	2.47e-48	hypothetical protein [Pantoea vagans]	470934	d__Bacteria;k__norank;p__Proteobacteria;c__Gammaproteobacteria;o__Enterobacterales;f__Erwiniaceae;g__Pantoea;s__Pantoea_vagans
PB1_gene_5855	82	WP_111140691.1	68	33/54(61.11)	2.00e-06	hypothetical protein [Pantoea sp. ARC607]	None	Unclassified
PB1_gene_5865	235	WP_064085243.1	235	235/235(100.00)	4.01e-162	uroporphyrinogen-III synthase [Wolbachia endosymbiont of Dactylopius coccus]	None	Unclassified
PB1_gene_5870	71	TCZ24161.1	132	36/71(50.70)	4.14e-14	hypothetical protein EYB39_17340 [Pantoea	549	d__Bacteria;k__norank;p__Proteobacteria;c__Gammaproteobacteria;o__Enterobacterales;f__Erwiniaceae;g__Pantoea;s__Pantoea_agglomerans
PB1_gene_5885	125	WP_080496780.1	165	120/124(96.77)	1.97e-83	hypothetical protein [Yersinia enterocolitica]	None	Unclassified
PB1_gene_5886	240	WP_111141459.1	243	240/240(100.00)	4.48e-169	outer membrane protein assembly factor BamD [Pantoea sp.	2027922	d__Bacteria;k__norank;p__Proteobacteria;c__Gammaproteobacteria;o__Enterobacterales;f__Erwiniaceae;g__Pantoea;s__Pantoea_sp._ARC607
PB1_gene_5894	270	ELP25069.1	569	263/270(97.41)	3.39e-188	Phage terminase large subunit [Pantoea agglomerans 299R]	1261128	d__Bacteria;k__norank;p__Proteobacteria;c__Gammaproteobacteria;o__Enterobacterales;f__Erwiniaceae;g__Pantoea;s__Pantoea_agglomerans
PB1_gene_5901	107	ORM53240.1	107	107/107(100.00)	7.86e-75	dsDNA-mimic protein [Pantoea conspicua]	472705	d__Bacteria;k__norank;p__Proteobacteria;c__Gammaproteobacteria;o__Enterobacterales;f__Erwiniaceae;g__Pantoea;s__Pantoea_conspicua
PB1_gene_5904	98	WP_128085453.1	98	96/98(97.96)	7.94e-64	DUF406 family protein [Pantoea deleyi]	470932	d__Bacteria;k__norank;p__Proteobacteria;c__Gammaproteobacteria;o__Enterobacterales;f__Erwiniaceae;g__Pantoea;s__Pantoea_deleyi
PB1_gene_5908	52	SCM55026.1	273	38/51(74.51)	3.23e-19	hypothetical protein BN1044_04539 [Hafnia alvei]	569	d__Bacteria;k__norank;p__Proteobacteria;c__Gammaproteobacteria;o__Enterobacterales;f__Hafniaceae;g__Hafnia;s__Hafnia_alvei
PB1_gene_5909	142	RXO32787.1	138	114/125(91.20)	4.21e-79	IS1 family transposase, partial [Salmonella enterica]	28901	d__Bacteria;k__norank;p__Proteobacteria;c__Gammaproteobacteria;o__Enterobacterales;f__Enterobacteriaceae;g__Salmonella;s__Salmonella_enterica
PB1_gene_5916	77	ORM85077.1	77	73/77(94.81)	2.01e-43	hypothetical protein HA42_03400 [Pantoea deleyi]	470932	d__Bacteria;k__norank;p__Proteobacteria;c__Gammaproteobacteria;o__Enterobacterales;f__Erwiniaceae;g__Pantoea;s__Pantoea_deleyi
PB1_gene_5921	62	ORM81371.1	64	60/62(96.77)	1.30e-35	hypothetical protein HA42_10885 [Pantoea deleyi]	470932	d__Bacteria;k__norank;p__Proteobacteria;c__Gammaproteobacteria;o__Enterobacterales;f__Erwiniaceae;g__Pantoea;s__Pantoea_deleyi
PB1_gene_5945	161	SQA98068.1	241	152/161(94.41)	2.29e-104	protein disaggregation chaperone [Cedecea neteri]	158822	d__Bacteria;k__norank;p__Proteobacteria;c__Gammaproteobacteria;o__Enterobacterales;f__Enterobacteriaceae;g__Cedecea;s__Cedecea_neteri
PB1_gene_5946	48	POE17328.1	88	47/48(97.92)	1.45e-28	hypothetical protein BV918_13145 [Pectobacterium carotovorum subsp. odoriferum]	78398	d__Bacteria;k__norank;p__Proteobacteria;c__Gammaproteobacteria;o__Enterobacterales;f__Pectobacteriaceae;g__Pectobacterium;s__Pectobacterium_odoriferum

PB1_gene_5950	130	WP_111139801.1	130	121/130(93.08)	2.06e-81	flagellar protein FlhE [Pantoea sp. ARC607]	2027922	d__Bacteria;k__norank;p__Proteobacteria;c__Gammaproteobacteria;o__Enterobacterales;f__Erwiniaceae;g__Pantoea;s__Pantoea_sp._ARC607
PB1_gene_5953	95	ORM75146.1	95	80/95(84.21)	1.74e-34	hypothetical protein HA42_21220 [Pantoea deleyi]	470932	d__Bacteria;k__norank;p__Proteobacteria;c__Gammaproteobacteria;o__Enterobacterales;f__Erwiniaceae;g__Pantoea;s__Pantoea_deleyi
PB1_gene_5967	88	AUX73165.1	336	53/84(63.10)	3.35e-26	hypothetical protein CPI84_12130 [Erwinia	79967	d__Bacteria;k__norank;p__Proteobacteria;c__Gammaproteobacteria;o__Enterobacterales;f__Erwiniaceae;g__Erwinia;s__Erwinia_pyrifoliae
PB1_gene_5975	149	KEY41786.1	149	138/149(92.62)	8.18e-88	RNase E inhibitor protein [Pantoea agglomerans]	549	d__Bacteria;k__norank;p__Proteobacteria;c__Gammaproteobacteria;o__Enterobacterales;f__Erwiniaceae;g__Pantoea;s__Pantoea_agglomerans
PB1_gene_5982	251	SRN47158.1	110	41/100(41.00)	9.19e-13	Uncharacterised protein [Shigella flexneri]	623	d__Bacteria;k__norank;p__Proteobacteria;c__Gammaproteobacteria;o__Enterobacterales;f__Enterobacteriaceae;g__Shigella;s__Shigella_flexneri
PB1_gene_5989	85	ORM84678.1	85	72/85(84.71)	7.14e-43	hypothetical protein HA42_03805 [Pantoea deleyi]	470932	d__Bacteria;k__norank;p__Proteobacteria;c__Gammaproteobacteria;o__Enterobacterales;f__Erwiniaceae;g__Pantoea;s__Pantoea_deleyi
PB1_gene_6017	89	TCZ25656.1	180	81/89(91.01)	4.45e-55	DUF3304 domain-containing protein [Pantoea agglomerans]	549	d__Bacteria;k__norank;p__Proteobacteria;c__Gammaproteobacteria;o__Enterobacterales;f__Erwiniaceae;g__Pantoea;s__Pantoea_agglomerans
PB1_gene_6038	155	SLR08046.1	148	69/125(55.20)	9.79e-44	Uncharacterised protein [Klebsiella pneumoniae]	573	d__Bacteria;k__norank;p__Proteobacteria;c__Gammaproteobacteria;o__Enterobacterales;f__Enterobacteriaceae;g__Klebsiella;s__Klebsiella_pneumoniae
PB1_gene_6042	90	WP_111138856.1	90	86/90(95.56)	9.42e-48	hypothetical protein [Pantoea sp. ARC607]	2027922	d__Bacteria;k__norank;p__Proteobacteria;c__Gammaproteobacteria;o__Enterobacterales;f__Erwiniaceae;g__Pantoea;s__Pantoea_sp._ARC607
PB1_gene_6061	186	QAV47661.1	185	183/185(98.92)	7.17e-127	type-F conjugative transfer system pilin assembly thiol-disulfide isomerase TrbB (plasmid) [Pantoea agglomerans]	549	d__Bacteria;k__norank;p__Proteobacteria;c__Gammaproteobacteria;o__Enterobacterales;f__Erwiniaceae;g__Pantoea;s__Pantoea_agglomerans
PB1_gene_6086	79	WP_010248714.1	192	57/61(93.44)	4.90e-32	phage baseplate assembly protein V [Pantoea agglomerans]	549	d__Bacteria;k__norank;p__Proteobacteria;c__Gammaproteobacteria;o__Enterobacterales;f__Erwiniaceae;g__Pantoea;s__Pantoea_agglomerans
PB1_gene_6088	111	WP_111139081.1	793	111/111(100.00)	1.20e-66	phosphoenolpyruvate synthase [Pantoea sp. ARC607]	2027922	d__Bacteria;k__norank;p__Proteobacteria;c__Gammaproteobacteria;o__Enterobacterales;f__Erwiniaceae;g__Pantoea;s__Pantoea_sp._ARC607
PB1_gene_6093	90	VEC84129.1	171	39/91(42.86)	9.46e-06	Uncharacterised protein [Klebsiella aerogenes]	54291	d__Bacteria;k__norank;p__Proteobacteria;c__Gammaproteobacteria;o__Enterobacterales;f__Enterobacteriaceae;g__Raoultella;s__Raoultella_ornithinolytica
PB1_gene_6100	246	ADD78831.1	231	227/230(98.70)	3.26e-163	Rpe [Pantoea ananatis LMG 20103]	706191	d__Bacteria;k__norank;p__Proteobacteria;c__Gammaproteobacteria;o__Enterobacterales;f__Erwiniaceae;g__Pantoea;s__Pantoea_ananatis
PB1_gene_6104	74	ORM81171.1	74	72/72(100.00)	4.17e-37	hypothetical protein HA42_11725 [Pantoea deleyi]	470932	d__Bacteria;k__norank;p__Proteobacteria;c__Gammaproteobacteria;o__Enterobacterales;f__Erwiniaceae;g__Pantoea;s__Pantoea_deleyi
PB1_gene_6122	170	WP_123346406.1	435	67/136(49.26)	8.84e-28	MULTISPECIES: hypothetical protein [Enterobacter]	2608935	d__Bacteria;k__norank;p__Proteobacteria;c__Gammaproteobacteria;o__Enterobacterales;f__Enterobacteriaceae;g__Enterobacter;s__norank
PB1_gene_6123	75	WP_123346408.1	968	63/75(84.00)	2.38e-35	MULTISPECIES: hypothetical protein [Enterobacter]	2608935	d__Bacteria;k__norank;p__Proteobacteria;c__Gammaproteobacteria;o__Enterobacterales;f__Enterobacteriaceae;g__Enterobacter;s__norank
PB1_gene_6144	174	PZL87017.1	163	162/163(99.39)	8.77e-104	YajQ family cyclic di-GMP-binding protein [Pantoea sp.	2027923	d__Bacteria;k__norank;p__Proteobacteria;c__Gammaproteobacteria;o__Enterobacterales;f__Erwiniaceae;g__Pantoea;s__Pantoea_sp._ARC270
PB1_gene_6147	93	PXW21412.1	93	93/93(100.00)	8.20e-57	DNA-binding protein HU-beta [Pantoea sp. JKS000250]	1938795	d__Bacteria;k__norank;p__Proteobacteria;c__Gammaproteobacteria;o__Enterobacterales;f__Erwiniaceae;g__Pantoea;s__Pantoea_sp._JKS000250
PB1_gene_6189	121	WP_126690658.1	213	53/91(58.24)	4.51e-25	helix-turn-helix domain-containing protein [Pantoea sp.	None	Unclassified
PB1_gene_6199	116	WP_082457448.1	169	68/115(59.13)	6.84e-43	DUF2938 family protein [Serratia sp. Leaf50]	None	Unclassified
PB1_gene_6216	67	SKA04674.1	67	67/67(100.00)	3.11e-38	Protein of unknown function [Pantoea agglomerans]	470933	d__Bacteria;k__norank;p__Proteobacteria;c__Gammaproteobacteria;o__Enterobacterales;f__Erwiniaceae;g__Pantoea;s__Pantoea_eucalypti
PB1_gene_6218	93	ORM79531.1	97	85/93(91.40)	1.22e-52	hypothetical protein HA42_15320 [Pantoea deleyi]	470932	d__Bacteria;k__norank;p__Proteobacteria;c__Gammaproteobacteria;o__Enterobacterales;f__Erwiniaceae;g__Pantoea;s__Pantoea_deleyi
PB1_gene_6243	144	PCG11985.1	195	143/144(99.31)	7.34e-93	CvpA family protein [Wolbachia pipientis]	None	Unclassified

PB1_gene_6250	64	WP_081439699.1	61	58/61(95.08)	3.43e-40	MULTISPECIES: hypothetical protein [Wolbachia]	2640676	d__Bacteria;k__norank;p__Proteobacteria;c__Alphaproteobacteria;o__Rickettsiales;f__Anaplasmataceae;g__Wolbachia;s__norank
PB1_gene_6277	127	WP_111139861.1	315	117/127(92.13)	3.12e-73	ABC transporter ATP-binding protein [Pantoea sp. ARC607]	2027922	d__Bacteria;k__norank;p__Proteobacteria;c__Gammaproteobacteria;o__Enterobacterales;f__Erwiniaceae;g__Pantoea;s__Pantoea_sp._ARC607
PB1_gene_6282	95	KHE00173.1	105	43/69(62.32)	1.31e-19	hypothetical protein NL54_17085 [Pantoea stewartii]	66269	d__Bacteria;k__norank;p__Proteobacteria;c__Gammaproteobacteria;o__Enterobacterales;f__Erwiniaceae;g__Pantoea;s__Pantoea_stewartii
PB1_gene_6288	80	WP_064365660.1	227	43/73(58.90)	5.45e-21	EAL domain-containing protein [Pantoea ananatis]	553	d__Bacteria;k__norank;p__Proteobacteria;c__Gammaproteobacteria;o__Enterobacterales;f__Erwiniaceae;g__Pantoea;s__Pantoea_ananatis
PB1_gene_6299	174	WP_031590432.1	175	170/174(97.70)	8.11e-114	type VI secretion system contractile sheath small subunit [Pantoea agglomerans]	549	d__Bacteria;k__norank;p__Proteobacteria;c__Gammaproteobacteria;o__Enterobacterales;f__Erwiniaceae;g__Pantoea;s__Pantoea_agglomerans
PB1_gene_6305	241	WP_025119481.1	1244	165/242(68.18)	9.57e-100	MULTISPECIES: methyltransferase domain-	2647522	d__Bacteria;k__norank;p__Proteobacteria;c__Gammaproteobacteria;o__Enterobacterales;f__Yersiniaceae;g__Serratia;s__norank
PB1_gene_6311	43	WP_052271401.1	107	34/41(82.93)	7.41e-17	phage repressor protein [Pantoea sp. CFSAN033090]	1690502	d__Bacteria;k__norank;p__Proteobacteria;c__Gammaproteobacteria;o__Enterobacterales;f__Erwiniaceae;g__Pantoea;s__Pantoea_sp._CFSAN033090
PB1_gene_6312	97	WP_069729876.1	79	47/64(73.44)	5.72e-26	DNA polymerase V [Pantoea brenneri]	472694	d__Bacteria;k__norank;p__Proteobacteria;c__Gammaproteobacteria;o__Enterobacterales;f__Erwiniaceae;g__Pantoea;s__Pantoea_brenneri
PB1_gene_6322	130	ORM82467.1	120	118/118(100.00)	1.37e-77	hypothetical protein HA42_08185 [Pantoea deleyi]	470932	d__Bacteria;k__norank;p__Proteobacteria;c__Gammaproteobacteria;o__Enterobacterales;f__Erwiniaceae;g__Pantoea;s__Pantoea_deleyi
PB1_gene_6324	87	OWA99223.1	172	56/84(66.67)	8.47e-34	hypothetical protein BV380_28965 [Klebsiella	573	d__Bacteria;k__norank;p__Proteobacteria;c__Gammaproteobacteria;o__Enterobacterales;f__Enterobacteriaceae;g__Klebsiella;s__Klebsiella_pneumoniae
PB1_gene_6325	125	WP_094964475.1	94	66/93(70.97)	8.40e-45	hypothetical protein [Salmonella enterica]	28901	d__Bacteria;k__norank;p__Proteobacteria;c__Gammaproteobacteria;o__Enterobacterales;f__Enterobacteriaceae;g__Salmonella;s__Salmonella_enterica
PB1_gene_6327	171	AZU37094.1	168	167/168(99.40)	6.56e-113	pantetheine-phosphate adenylyltransferase [Wolbachia endosymbiont of Bemisia tabaci]	215173	d__Bacteria;k__norank;p__Proteobacteria;c__Alphaproteobacteria;o__Rickettsiales;f__Anaplasmataceae;g__Wolbachia;s__Wolbachia_endosymbiont_of_Bemisia_tabaci
PB1_gene_6347	215	ORM80922.1	204	202/204(99.02)	9.16e-126	molecular chaperone OsmY [Pantoea deleyi]	470932	d__Bacteria;k__norank;p__Proteobacteria;c__Gammaproteobacteria;o__Enterobacterales;f__Erwiniaceae;g__Pantoea;s__Pantoea_deleyi
PB1_gene_6349	45	PCG11713.1	92	45/45(100.00)	1.26e-22	hypothetical protein CO539_01545 [Wolbachia	None	Unclassified
PB1_gene_6354	189	ORM83880.1	189	188/189(99.47)	1.33e-126	3-octaprenyl-4-hydroxybenzoate carboxy-lyase [Pantoea deleyi]	470932	d__Bacteria;k__norank;p__Proteobacteria;c__Gammaproteobacteria;o__Enterobacterales;f__Erwiniaceae;g__Pantoea;s__Pantoea_deleyi
PB1_gene_6371	236	SSN10832.1	249	80/173(46.24)	8.70e-41	Uncharacterised protein [Klebsiella pneumoniae]	573	d__Bacteria;k__norank;p__Proteobacteria;c__Gammaproteobacteria;o__Enterobacterales;f__Enterobacteriaceae;g__Klebsiella;s__Klebsiella_pneumoniae
PB1_gene_6375	54	ERP05348.1	68	51/54(94.44)	3.21e-30	chromosomal replication initiator protein dnaA [Enterobacter sp. MGH 14]	1329823	d__Bacteria;k__norank;p__Proteobacteria;c__Gammaproteobacteria;o__Enterobacterales;f__Enterobacteriaceae;g__Enterobacter;s__Enterobacter_sp._MGH_14
PB1_gene_6376	163	WP_123803203.1	419	154/154(100.00)	7.27e-103	translesion error-prone DNA polymerase V subunit UmuC [Pantoea sp. RIT388]	1869313	d__Bacteria;k__norank;p__Proteobacteria;c__Gammaproteobacteria;o__Enterobacterales;f__Erwiniaceae;g__Pantoea;s__Pantoea_sp._RIT388
PB1_gene_6377	61	WP_069729394.1	125	60/61(98.36)	8.92e-36	phage repressor protein [Pantoea brenneri]	53335	d__Bacteria;k__norank;p__Proteobacteria;c__Gammaproteobacteria;o__Enterobacterales;f__Erwiniaceae;g__Pantoea;s__norank
PB1_gene_6386	196	ORM76109.1	194	190/193(98.45)	1.01e-133	polyisoprenoid-binding protein [Pantoea deleyi]	470932	d__Bacteria;k__norank;p__Proteobacteria;c__Gammaproteobacteria;o__Enterobacterales;f__Erwiniaceae;g__Pantoea;s__Pantoea_deleyi
PB1_gene_6411	90	ORM83620.1	90	90/90(100.00)	7.43e-54	PTS mannitol transporter subunit IIB [Pantoea deleyi]	470932	d__Bacteria;k__norank;p__Proteobacteria;c__Gammaproteobacteria;o__Enterobacterales;f__Erwiniaceae;g__Pantoea;s__Pantoea_deleyi
PB1_gene_6423	151	ORM85066.1	147	147/147(100.00)	1.44e-97	flagellar biosynthesis chaperone FliJ [Pantoea deleyi]	470932	d__Bacteria;k__norank;p__Proteobacteria;c__Gammaproteobacteria;o__Enterobacterales;f__Erwiniaceae;g__Pantoea;s__Pantoea_deleyi
PB1_gene_6466	145	WP_107320583.1	145	130/145(89.66)	1.18e-85	hypothetical protein [Pantoea vagans]	470934	d__Bacteria;k__norank;p__Proteobacteria;c__Gammaproteobacteria;o__Enterobacterales;f__Erwiniaceae;g__Pantoea;s__Pantoea_vagans

PB1_gene_6471	146	ETW98760.1	656	68/146(46.58)	1.11e-31	hypothetical protein ETSY2_42270 [Candidatus	1429439	d__Bacteria;k__norank;p__Candidatus_Tectomicrobia;c__norank;o__norank;f__norank;g__Candidatus_Entotheonella;s__Candidatus_Entotheonella_gemina
PB1_gene_6483	178	ORM80557.1	178	173/178(97.19)	1.16e-126	ribosomal-protein-serine acetyltransferase [Pantoea deleyi]	470932	d__Bacteria;k__norank;p__Proteobacteria;c__Gammaproteobacteria;o__Enterobacterales;f__Erwiniaceae;g__Pantoea;s__Pantoea_deleyi
PB1_gene_6488	61	OXH79011.1	132	49/60(81.67)	2.78e-24	hypothetical protein CBI57_05640 [Pantoea	549	d__Bacteria;k__norank;p__Proteobacteria;c__Gammaproteobacteria;o__Enterobacterales;f__Erwiniaceae;g__Pantoea;s__Pantoea_agglomerans
PB1_gene_6493	199	KEY42616.1	209	180/198(90.91)	1.87e-131	hypothetical protein FB99_10680 [Pantoea agglomerans]	549	d__Bacteria;k__norank;p__Proteobacteria;c__Gammaproteobacteria;o__Enterobacterales;f__Erwiniaceae;g__Pantoea;s__Pantoea_agglomerans
PB1_gene_6519	134	WP_010403494.1	396	134/134(100.00)	1.59e-85	phosphoglycerate kinase [Wolbachia endosymbiont of Nasonia vitripennis]	None	Unclassified
PB1_gene_6526	65	ORM74511.1	65	62/65(95.38)	6.17e-35	thiamine biosynthesis protein ThiS [Pantoea deleyi]	470932	d__Bacteria;k__norank;p__Proteobacteria;c__Gammaproteobacteria;o__Enterobacterales;f__Erwiniaceae;g__Pantoea;s__Pantoea_deleyi
PB1_gene_6543	196	ORM85652.1	196	196/196(100.00)	3.13e-142	imidazole glycerol phosphate synthase, glutamine amidotransferase subunit [Pantoea deleyi]	470932	d__Bacteria;k__norank;p__Proteobacteria;c__Gammaproteobacteria;o__Enterobacterales;f__Erwiniaceae;g__Pantoea;s__Pantoea_deleyi
PB1_gene_6559	226	WP_081115544.1	526	187/221(84.62)	2.39e-110	hypothetical protein [Pantoea agglomerans]	None	Unclassified
PB1_gene_6562	134	ORM86585.1	134	124/133(93.23)	3.25e-89	hypothetical protein HA42_00710 [Pantoea deleyi]	470932	d__Bacteria;k__norank;p__Proteobacteria;c__Gammaproteobacteria;o__Enterobacterales;f__Erwiniaceae;g__Pantoea;s__Pantoea_deleyi
PB1_gene_6575	209	WP_045140818.1	666	201/209(96.17)	9.57e-131	DNA sulfur modification protein DndD [Pantoea agglomerans]	549	d__Bacteria;k__norank;p__Proteobacteria;c__Gammaproteobacteria;o__Enterobacterales;f__Erwiniaceae;g__Pantoea;s__Pantoea_agglomerans
PB1_gene_6581	141	ORM75770.1	141	137/141(97.16)	1.64e-90	murein hydrolase regulator LrgA [Pantoea deleyi]	470932	d__Bacteria;k__norank;p__Proteobacteria;c__Gammaproteobacteria;o__Enterobacterales;f__Erwiniaceae;g__Pantoea;s__Pantoea_deleyi
PB1_gene_6600	194	ORM79327.1	315	191/194(98.45)	5.48e-131	ABC transporter [Pantoea deleyi]	470932	d__Bacteria;k__norank;p__Proteobacteria;c__Gammaproteobacteria;o__Enterobacterales;f__Erwiniaceae;g__Pantoea;s__Pantoea_deleyi
PB1_gene_6603	159	OQV43489.1	182	159/159(100.00)	9.60e-106	hypothetical protein BZ160_05060 [Pantoea vagans]	470934	d__Bacteria;k__norank;p__Proteobacteria;c__Gammaproteobacteria;o__Enterobacterales;f__Erwiniaceae;g__Pantoea;s__Pantoea_vagans
PB1_gene_6606	177	WP_107320573.1	165	163/165(98.79)	3.07e-112	conjugal transfer protein TraH [Pantoea vagans]	470934	d__Bacteria;k__norank;p__Proteobacteria;c__Gammaproteobacteria;o__Enterobacterales;f__Erwiniaceae;g__Pantoea;s__Pantoea_vagans
PB1_gene_6608	60	WP_098050859.1	121	57/60(95.00)	1.98e-31	hypothetical protein [Pantoea agglomerans]	None	Unclassified
PB1_gene_6609	49	WP_098050859.1	121	43/49(87.76)	1.10e-20	hypothetical protein [Pantoea agglomerans]	None	Unclassified
PB1_gene_6623	42	RZK07260.1	341	40/42(95.24)	1.31e-18	translesion error-prone DNA polymerase V subunit UmuC [Pantoea sp.]	69393	d__Bacteria;k__norank;p__Proteobacteria;c__Gammaproteobacteria;o__Enterobacterales;f__Erwiniaceae;g__Pantoea;s__Pantoea_sp.
PB1_gene_6633	108	PZL98852.1	95	55/65(84.62)	2.15e-29	hypothetical protein CKF43_00810 [Pantoea sp.	2027922	d__Bacteria;k__norank;p__Proteobacteria;c__Gammaproteobacteria;o__Enterobacterales;f__Erwiniaceae;g__Pantoea;s__Pantoea_sp._ARC607
PB1_gene_6645	122	SUB14910.1	284	122/122(100.00)	3.39e-79	HTH-type transcriptional activator AllS [Pantoea	549	d__Bacteria;k__norank;p__Proteobacteria;c__Gammaproteobacteria;o__Enterobacterales;f__Erwiniaceae;g__Pantoea;s__Pantoea_agglomerans
PB1_gene_6667	101	APV13804.1	99	66/84(78.57)	5.48e-38	microcin [Klebsiella pneumoniae]	573	d__Bacteria;k__norank;p__Proteobacteria;c__Gammaproteobacteria;o__Enterobacterales;f__Enterobacteriaceae;g__Klebsiella;s__Klebsiella_pneumoniae
PB1_gene_6685	105	RAH26284.1	121	103/105(98.10)	8.35e-68	hypothetical protein DOT37_23985 [Pantoea	549	d__Bacteria;k__norank;p__Proteobacteria;c__Gammaproteobacteria;o__Enterobacterales;f__Erwiniaceae;g__Pantoea;s__Pantoea_agglomerans
PB1_gene_6691	151	ELP25058.1	353	142/151(94.04)	1.23e-95	Prophage tail protein [Pantoea agglomerans 299R]	1261128	d__Bacteria;k__norank;p__Proteobacteria;c__Gammaproteobacteria;o__Enterobacterales;f__Erwiniaceae;g__Pantoea;s__Pantoea_agglomerans
PB1_gene_6711	122	WP_126690660.1	216	72/122(59.02)	2.56e-39	ParA family protein [Pantoea sp. YU22]	None	Unclassified

PB1_gene_6718	114	ERM08937.1	114	112/114(98.25)	3.11e-73	membrane protein [Pantoea agglomerans Tx10]	1332072	d__Bacteria;k__norank;p__Proteobacteria;c__Gammaproteobacteria;o__Enterobacterales;f__Erwiniaceae;g__Pantoea;s__Pantoea_agglomerans
PB1_gene_6723	217	WP_048786827.1	2720	142/192(73.96)	1.35e-56	filamentous hemagglutinin N-terminal domain-containing protein, partial [Pantoea vagans]	470934	d__Bacteria;k__norank;p__Proteobacteria;c__Gammaproteobacteria;o__Enterobacterales;f__Erwiniaceae;g__Pantoea;s__Pantoea_vagans
PB1_gene_6724	81	SJZ73476.1	81	77/81(95.06)	4.95e-48	Protein of unknown function [Pantoea agglomerans]	470933	d__Bacteria;k__norank;p__Proteobacteria;c__Gammaproteobacteria;o__Enterobacterales;f__Erwiniaceae;g__Pantoea;s__Pantoea_eucalypti
PB1_gene_6728	200	CCK07774.1	274	66/135(48.89)	1.03e-25	hypothetical protein BN128_1746 [Cronobacter sakazakii 696]	1208664	d__Bacteria;k__norank;p__Proteobacteria;c__Gammaproteobacteria;o__Enterobacterales;f__Enterobacteriaceae;g__Cronobacter;s__Cronobacter_sakazakii
PB1_gene_6734	44	WP_083068304.1	354	30/36(83.33)	1.21e-11	hypothetical protein [Pantoea vagans]	53335	d__Bacteria;k__norank;p__Proteobacteria;c__Gammaproteobacteria;o__Enterobacterales;f__Erwiniaceae;g__Pantoea;s__norank
PB1_gene_6770	192	WP_069788038.1	546	90/179(50.28)	3.42e-51	acyltransferase [Pseudomonas salomonii]	191391	d__Bacteria;k__norank;p__Proteobacteria;c__Gammaproteobacteria;o__Pseudomonadales;f__Pseudomonadaceae;g__Pseudomonas;s__Pseudomonas_salomonii
PB1_gene_6783	74	WP_091003960.1	74	48/71(67.61)	2.83e-26	MULTISPECIES: hypothetical protein [Pantoea]	2630326	d__Bacteria;k__norank;p__Proteobacteria;c__Gammaproteobacteria;o__Enterobacterales;f__Erwiniaceae;g__Pantoea;s__norank
PB1_gene_6856	132	SSN11031.1	85	68/85(80.00)	4.42e-40	Uncharacterised protein [Klebsiella pneumoniae]	573	d__Bacteria;k__norank;p__Proteobacteria;c__Gammaproteobacteria;o__Enterobacterales;f__Enterobacteriaceae;g__Klebsiella;s__Klebsiella_pneumoniae
PB1_gene_6866	170	WP_096011481.1	169	158/169(93.49)	3.93e-114	N-acetyltransferase [Pantoea allii]	574096	d__Bacteria;k__norank;p__Proteobacteria;c__Gammaproteobacteria;o__Enterobacterales;f__Erwiniaceae;g__Pantoea;s__Pantoea_allii
PB1_gene_6895	183	WP_107320607.1	247	182/183(99.45)	2.08e-128	conjugal transfer protein [Pantoea vagans]	None	Unclassified
PB1_gene_6900	207	ORM79065.1	363	193/207(93.24)	2.87e-124	mechanosensitive ion channel protein MscS [Pantoea deleyi]	470932	d__Bacteria;k__norank;p__Proteobacteria;c__Gammaproteobacteria;o__Enterobacterales;f__Erwiniaceae;g__Pantoea;s__Pantoea_deleyi
PB1_gene_6913	77	AIR84876.1	77	67/77(87.01)	1.93e-41	damage-inducible protein DinI [Pantoea rwandensis]	1076550	d__Bacteria;k__norank;p__Proteobacteria;c__Gammaproteobacteria;o__Enterobacterales;f__Erwiniaceae;g__Pantoea;s__Pantoea_rwandensis
PB1_gene_6944	66	OWZ24955.1	54	43/54(79.63)	8.90e-20	hypothetical protein CCY16_00416 [Wolbachia endosymbiont of Wuchereria	96496	d__Bacteria;k__norank;p__Proteobacteria;c__Alphaproteobacteria;o__Rickettsiales;f__Anaplasmataceae;g__Wolbachia;s__Wolbachia_endosymbiont_of_Wuchereria_bancrofti
PB1_gene_6963	168	PBD16227.1	241	161/168(95.83)	2.62e-105	ATP synthase subunit A [Wolbachia endosymbiont of Drosophila subpulchrella]	2033655	d__Bacteria;k__norank;p__Proteobacteria;c__Alphaproteobacteria;o__Rickettsiales;f__Anaplasmataceae;g__Wolbachia;s__Wolbachia_endosymbiont_of_Drosophila_subpulchrella
PB1_gene_6973	145	ORM76736.1	145	145/145(100.00)	2.91e-95	transcriptional regulator SlyA [Pantoea deleyi]	470932	d__Bacteria;k__norank;p__Proteobacteria;c__Gammaproteobacteria;o__Enterobacterales;f__Erwiniaceae;g__Pantoea;s__Pantoea_deleyi
PB1_gene_6978	184	CNI80496.1	162	76/151(50.33)	1.44e-21	Uncharacterised protein [Yersinia pekkanenii]	1288385	d__Bacteria;k__norank;p__Proteobacteria;c__Gammaproteobacteria;o__Enterobacterales;f__Yersiniaceae;g__Yersinia;s__Yersinia_pekkanenii
PB1_gene_6979	133	ORM74227.1	298	129/133(96.99)	1.15e-83	beta-ketoacyl-[acyl-carrier-protein] synthase II, partial	470932	d__Bacteria;k__norank;p__Proteobacteria;c__Gammaproteobacteria;o__Enterobacterales;f__Erwiniaceae;g__Pantoea;s__Pantoea_deleyi
PB1_gene_6991	66	WP_128086569.1	66	51/66(77.27)	6.69e-26	hypothetical protein [Pantoea deleyi]	470932	d__Bacteria;k__norank;p__Proteobacteria;c__Gammaproteobacteria;o__Enterobacterales;f__Erwiniaceae;g__Pantoea;s__Pantoea_deleyi
PB1_gene_7011	85	RAR74666.1	369	46/64(71.88)	9.90e-18	integrase [Pantoea ananatis]	553	d__Bacteria;k__norank;p__Proteobacteria;c__Gammaproteobacteria;o__Enterobacterales;f__Erwiniaceae;g__Pantoea;s__Pantoea_ananatis
PB1_gene_7013	177	VDZ81076.1	97	26/28(92.86)	1.46e-08	lipoate-protein ligase A [Salmonella bongori]	54736	d__Bacteria;k__norank;p__Proteobacteria;c__Gammaproteobacteria;o__Enterobacterales;f__Enterobacteriaceae;g__Salmonella;s__Salmonella_bongori
PB1_gene_7017	202	WP_090966254.1	483	199/202(98.51)	1.64e-132	NAD-dependent succinate-semialdehyde dehydrogenase [Pantoea sp. OV426]	1881036	d__Bacteria;k__norank;p__Proteobacteria;c__Gammaproteobacteria;o__Enterobacterales;f__Erwiniaceae;g__Pantoea;s__Pantoea_sp._OV426
PB1_gene_7025	116	WP_111139518.1	116	110/116(94.83)	8.48e-63	anti-adapter protein IraP [Pantoea sp. ARC607]	2027922	d__Bacteria;k__norank;p__Proteobacteria;c__Gammaproteobacteria;o__Enterobacterales;f__Erwiniaceae;g__Pantoea;s__Pantoea_sp._ARC607
PB1_gene_7037	200	WP_111139514.1	1320	161/200(80.50)	1.00e-85	BapA prefix-like domain-containing protein [Pantoea sp.	2027922	d__Bacteria;k__norank;p__Proteobacteria;c__Gammaproteobacteria;o__Enterobacterales;f__Erwiniaceae;g__Pantoea;s__Pantoea_sp._ARC607

PB1_gene_7053	103	ORM83127.1	103	97/102(95.10)	2.40e-65	methyltransferase [Pantoea deleyi]	470932	d__Bacteria;k__norank;p__Proteobacteria;c__Gammaproteobacteria;o__Enterobacterales;f__Erwiniaceae;g__Pantoea;s__Pantoea_deleyi
PB1_gene_7054	90	PCG11096.1	355	90/90(100.00)	9.62e-53	rod shape-determining protein [Wolbachia pipientis]	None	Unclassified
PB1_gene_7057	83	WP_094106241.1	78	78/78(100.00)	1.94e-45	MULTISPECIES: replication regulatory protein RepA	53335	d__Bacteria;k__norank;p__Proteobacteria;c__Gammaproteobacteria;o__Enterobacterales;f__Erwiniaceae;g__Pantoea;s__norank
PB1_gene_7062	69	WP_111141498.1	275	68/69(98.55)	3.92e-42	SDR family oxidoreductase [Pantoea sp. ARC607]	2027922	d__Bacteria;k__norank;p__Proteobacteria;c__Gammaproteobacteria;o__Enterobacterales;f__Erwiniaceae;g__Pantoea;s__Pantoea_sp._ARC607
PB2_gene_8	382	ORM83039.1	382	378/382(98.95)	2.92e-272	lipid-A-disaccharide synthase [Pantoea deleyi]	470932	d__Bacteria;k__norank;p__Proteobacteria;c__Gammaproteobacteria;o__Enterobacterales;f__Erwiniaceae;g__Pantoea;s__Pantoea_deleyi
PB2_gene_10	1144	WP_111140591.1	1160	1140/1144(99.65)	0	DNA polymerase III subunit alpha [Pantoea sp. ARC607]	2027922	d__Bacteria;k__norank;p__Proteobacteria;c__Gammaproteobacteria;o__Enterobacterales;f__Erwiniaceae;g__Pantoea;s__Pantoea_sp._ARC607
PB2_gene_12	727	WP_111140593.1	716	708/716(98.88)	0	lysine decarboxylase LdcC [Pantoea sp. ARC607]	2027922	d__Bacteria;k__norank;p__Proteobacteria;c__Gammaproteobacteria;o__Enterobacterales;f__Erwiniaceae;g__Pantoea;s__Pantoea_sp._ARC607
PB2_gene_14	439	ORM83044.1	439	420/439(95.67)	8.41e-310	tRNA lysidine(34) synthetase TilS [Pantoea deleyi]	470932	d__Bacteria;k__norank;p__Proteobacteria;c__Gammaproteobacteria;o__Enterobacterales;f__Erwiniaceae;g__Pantoea;s__Pantoea_deleyi
PB2_gene_17	136	WP_111140599.1	136	135/136(99.26)	1.94e-82	aminoacyl-tRNA hydrolase [Pantoea sp. ARC607]	2027922	d__Bacteria;k__norank;p__Proteobacteria;c__Gammaproteobacteria;o__Enterobacterales;f__Erwiniaceae;g__Pantoea;s__Pantoea_sp._ARC607
PB2_gene_20	305	WP_111139669.1	305	305/305(100.00)	1.23e-216	HTH-type transcriptional activator AaeR [Pantoea sp.	2027922	d__Bacteria;k__norank;p__Proteobacteria;c__Gammaproteobacteria;o__Enterobacterales;f__Erwiniaceae;g__Pantoea;s__Pantoea_sp._ARC607
PB2_gene_23	489	ORM83378.1	489	487/489(99.59)	0	ribonuclease G [Pantoea deleyi]	470932	d__Bacteria;k__norank;p__Proteobacteria;c__Gammaproteobacteria;o__Enterobacterales;f__Erwiniaceae;g__Pantoea;s__Pantoea_deleyi
PB2_gene_25	162	ORM83379.1	162	161/162(99.38)	9.37e-107	rod shape-determining protein MreD [Pantoea deleyi]	470932	d__Bacteria;k__norank;p__Proteobacteria;c__Gammaproteobacteria;o__Enterobacterales;f__Erwiniaceae;g__Pantoea;s__Pantoea_deleyi
PB2_gene_26	343	WP_111139675.1	341	339/343(98.83)	5.79e-220	rod shape-determining protein MreC [Pantoea sp. ARC607]	2027922	d__Bacteria;k__norank;p__Proteobacteria;c__Gammaproteobacteria;o__Enterobacterales;f__Erwiniaceae;g__Pantoea;s__Pantoea_sp._ARC607
PB2_gene_43	502	ORM85444.1	502	498/502(99.20)	0	ribose ABC transporter ATP-binding protein RbsA [Pantoea	470932	d__Bacteria;k__norank;p__Proteobacteria;c__Gammaproteobacteria;o__Enterobacterales;f__Erwiniaceae;g__Pantoea;s__Pantoea_deleyi
PB2_gene_44	323	ORM85443.1	323	323/323(100.00)	7.21e-209	ribose ABC transporter permease [Pantoea deleyi]	470932	d__Bacteria;k__norank;p__Proteobacteria;c__Gammaproteobacteria;o__Enterobacterales;f__Erwiniaceae;g__Pantoea;s__Pantoea_deleyi
PB2_gene_48	459	ORM85441.1	466	457/459(99.56)	3.16e-310	MFS transporter [Pantoea deleyi]	470932	d__Bacteria;k__norank;p__Proteobacteria;c__Gammaproteobacteria;o__Enterobacterales;f__Erwiniaceae;g__Pantoea;s__Pantoea_deleyi
PB2_gene_49	65	WP_095706374.1	106	30/37(81.08)	1.98e-13	hypothetical protein [Pantoea vagans]	None	Unclassified
PB2_gene_51	188	WP_111139305.1	188	180/188(95.74)	5.78e-116	potassium-transporting ATPase subunit KdpC [Pantoea sp.	2027922	d__Bacteria;k__norank;p__Proteobacteria;c__Gammaproteobacteria;o__Enterobacterales;f__Erwiniaceae;g__Pantoea;s__Pantoea_sp._ARC607
PB2_gene_58	374	WP_111139312.1	374	366/374(97.86)	1.52e-279	N-acetyltransferase [Pantoea sp. ARC607]	2027922	d__Bacteria;k__norank;p__Proteobacteria;c__Gammaproteobacteria;o__Enterobacterales;f__Erwiniaceae;g__Pantoea;s__Pantoea_sp._ARC607
PB2_gene_61	201	AVV35947.1	201	188/201(93.53)	1.12e-133	phospholipase [Pantoea vagans]	470934	d__Bacteria;k__norank;p__Proteobacteria;c__Gammaproteobacteria;o__Enterobacterales;f__Erwiniaceae;g__Pantoea;s__Pantoea_vagans
PB2_gene_67	393	WP_111141257.1	380	361/380(95.00)	7.02e-255	alanine racemase [Pantoea sp. ARC607]	2027922	d__Bacteria;k__norank;p__Proteobacteria;c__Gammaproteobacteria;o__Enterobacterales;f__Erwiniaceae;g__Pantoea;s__Pantoea_sp._ARC607
PB2_gene_69	54	WP_071987793.1	86	24/39(61.54)	8.49e-06	hypothetical protein [Pantoea sp. Sc1]	None	Unclassified
PB2_gene_70	215	ORM82754.1	214	213/214(99.53)	9.12e-142	PST family transport protein [Pantoea deleyi]	470932	d__Bacteria;k__norank;p__Proteobacteria;c__Gammaproteobacteria;o__Enterobacterales;f__Erwiniaceae;g__Pantoea;s__Pantoea_deleyi
PB2_gene_73	224	SKA24458.1	224	220/224(98.21)	1.40e-152	guanylate kinase [Pantoea agglomerans]	470933	d__Bacteria;k__norank;p__Proteobacteria;c__Gammaproteobacteria;o__Enterobacterales;f__Erwiniaceae;g__Pantoea;s__Pantoea_eucalypti
PB2_gene_76	232	ORM82735.1	232	232/232(100.00)	4.71e-164	tRNA (guanosine(18)-2'-O)-methyltransferase TrmH [Pantoea deleyi]	470932	d__Bacteria;k__norank;p__Proteobacteria;c__Gammaproteobacteria;o__Enterobacterales;f__Erwiniaceae;g__Pantoea;s__Pantoea_deleyi

PB2_gene_77	692	ORM82736.1	692	691/692(99.86)	0	ATP-dependent DNA helicase RecG [Pantoea deleyi]	470932	d__Bacteria;k__norank;p__Proteobacteria;c__Gammaproteobacteria;o__Enterobacterales;f__Erwiniaceae;g__Pantoea;s__Pantoea_deleyi
PB2_gene_79	559	ORM82738.1	559	530/559(94.81)	0	hypothetical protein HA42_07670 [Pantoea deleyi]	470932	d__Bacteria;k__norank;p__Proteobacteria;c__Gammaproteobacteria;o__Enterobacterales;f__Erwiniaceae;g__Pantoea;s__Pantoea_deleyi
PB2_gene_82	425	ORM72641.1	425	402/425(94.59)	5.97e-280	hypothetical protein HA42_22260 [Pantoea deleyi]	470932	d__Bacteria;k__norank;p__Proteobacteria;c__Gammaproteobacteria;o__Enterobacterales;f__Erwiniaceae;g__Pantoea;s__Pantoea_deleyi
PB2_gene_85	414	ORM72645.1	414	404/414(97.58)	4.48e-290	tryptophan permease [Pantoea deleyi]	470932	d__Bacteria;k__norank;p__Proteobacteria;c__Gammaproteobacteria;o__Enterobacterales;f__Erwiniaceae;g__Pantoea;s__Pantoea_deleyi
PB2_gene_87	503	WP_111138966.1	459	422/459(91.94)	4.85e-287	MFS transporter [Pantoea sp. ARC607]	2027922	d__Bacteria;k__norank;p__Proteobacteria;c__Gammaproteobacteria;o__Enterobacterales;f__Erwiniaceae;g__Pantoea;s__Pantoea_sp._ARC607
PB2_gene_88	300	WP_111142283.1	301	283/300(94.33)	5.06e-200	LysR family transcriptional regulator, partial [Pantoea sp.	2027922	d__Bacteria;k__norank;p__Proteobacteria;c__Gammaproteobacteria;o__Enterobacterales;f__Erwiniaceae;g__Pantoea;s__Pantoea_sp._ARC607
PB2_gene_89	158	ORM72648.1	158	151/158(95.57)	2.51e-109	glutathione peroxidase [Pantoea deleyi]	470932	d__Bacteria;k__norank;p__Proteobacteria;c__Gammaproteobacteria;o__Enterobacterales;f__Erwiniaceae;g__Pantoea;s__Pantoea_deleyi
PB2_gene_90	266	ORM72649.1	266	258/266(96.99)	3.15e-179	sugar dehydrogenase [Pantoea deleyi]	470932	d__Bacteria;k__norank;p__Proteobacteria;c__Gammaproteobacteria;o__Enterobacterales;f__Erwiniaceae;g__Pantoea;s__Pantoea_deleyi
PB2_gene_91	223	ORM72650.1	238	201/223(90.13)	1.18e-147	DNA-binding protein [Pantoea deleyi]	470932	d__Bacteria;k__norank;p__Proteobacteria;c__Gammaproteobacteria;o__Enterobacterales;f__Erwiniaceae;g__Pantoea;s__Pantoea_deleyi
PB2_gene_92	120	ORM72665.1	120	108/120(90.00)	7.45e-77	glyoxalase [Pantoea deleyi]	470932	d__Bacteria;k__norank;p__Proteobacteria;c__Gammaproteobacteria;o__Enterobacterales;f__Erwiniaceae;g__Pantoea;s__Pantoea_deleyi
PB2_gene_93	446	ORM72652.1	446	444/446(99.55)	1.32e-314	MFS transporter permease [Pantoea deleyi]	470932	d__Bacteria;k__norank;p__Proteobacteria;c__Gammaproteobacteria;o__Enterobacterales;f__Erwiniaceae;g__Pantoea;s__Pantoea_deleyi
PB2_gene_97	493	WP_111141615.1	491	481/490(98.16)	0	DNA-3-methyladenine glycosylase 2 family protein	2027922	d__Bacteria;k__norank;p__Proteobacteria;c__Gammaproteobacteria;o__Enterobacterales;f__Erwiniaceae;g__Pantoea;s__Pantoea_sp._ARC607
PB2_gene_98	76	KGD85290.1	72	32/46(69.57)	2.85e-10	hypothetical protein HA47_03565, partial [Pantoea stewartii subsp. indologenes]	66270	d__Bacteria;k__norank;p__Proteobacteria;c__Gammaproteobacteria;o__Enterobacterales;f__Erwiniaceae;g__Pantoea;s__Pantoea_stewartii
PB2_gene_99	471	ORM77231.1	471	468/471(99.36)	0	glutamate--tRNA ligase [Pantoea deleyi]	470932	d__Bacteria;k__norank;p__Proteobacteria;c__Gammaproteobacteria;o__Enterobacterales;f__Erwiniaceae;g__Pantoea;s__Pantoea_deleyi
PB2_gene_102	396	ORM77228.1	396	396/396(100.00)	4.42e-265	nucleoside permease [Pantoea deleyi]	470932	d__Bacteria;k__norank;p__Proteobacteria;c__Gammaproteobacteria;o__Enterobacterales;f__Erwiniaceae;g__Pantoea;s__Pantoea_deleyi
PB2_gene_103	413	WP_111141165.1	413	407/413(98.55)	8.38e-274	divalent metal cation transporter [Pantoea sp. ARC607]	2027922	d__Bacteria;k__norank;p__Proteobacteria;c__Gammaproteobacteria;o__Enterobacterales;f__Erwiniaceae;g__Pantoea;s__Pantoea_sp._ARC607
PB2_gene_106	550	WP_111141168.1	550	523/550(95.09)	0	indolepyruvate decarboxylase [Pantoea sp. ARC607]	2027922	d__Bacteria;k__norank;p__Proteobacteria;c__Gammaproteobacteria;o__Enterobacterales;f__Erwiniaceae;g__Pantoea;s__Pantoea_sp._ARC607
PB2_gene_111	409	ORM84513.1	409	397/409(97.07)	1.61e-285	glycosyltransferase [Pantoea deleyi]	470932	d__Bacteria;k__norank;p__Proteobacteria;c__Gammaproteobacteria;o__Enterobacterales;f__Erwiniaceae;g__Pantoea;s__Pantoea_deleyi
PB2_gene_114	399	ORM84548.1	398	389/398(97.74)	6.24e-280	glycine C-acetyltransferase [Pantoea deleyi]	470932	d__Bacteria;k__norank;p__Proteobacteria;c__Gammaproteobacteria;o__Enterobacterales;f__Erwiniaceae;g__Pantoea;s__Pantoea_deleyi
PB2_gene_118	358	ORM84508.1	358	352/358(98.32)	3.57e-254	glycosyltransferase [Pantoea deleyi]	470932	d__Bacteria;k__norank;p__Proteobacteria;c__Gammaproteobacteria;o__Enterobacterales;f__Erwiniaceae;g__Pantoea;s__Pantoea_deleyi
PB2_gene_120	348	ORM84506.1	348	339/348(97.41)	7.15e-249	glycosyltransferase [Pantoea deleyi]	470932	d__Bacteria;k__norank;p__Proteobacteria;c__Gammaproteobacteria;o__Enterobacterales;f__Erwiniaceae;g__Pantoea;s__Pantoea_deleyi
PB2_gene_121	358	ORM84505.1	358	353/358(98.60)	4.41e-264	putative lipopolysaccharide heptosyltransferase III [Pantoea deleyi]	470932	d__Bacteria;k__norank;p__Proteobacteria;c__Gammaproteobacteria;o__Enterobacterales;f__Erwiniaceae;g__Pantoea;s__Pantoea_deleyi
PB2_gene_134	257	ORM72620.1	257	250/257(97.28)	1.81e-169	hypothetical protein HA42_22140 [Pantoea deleyi]	470932	d__Bacteria;k__norank;p__Proteobacteria;c__Gammaproteobacteria;o__Enterobacterales;f__Erwiniaceae;g__Pantoea;s__Pantoea_deleyi
PB2_gene_136	442	WP_111138973.1	411	378/411(91.97)	1.71e-280	FAD-binding oxidoreductase [Pantoea sp. ARC607]	2027922	d__Bacteria;k__norank;p__Proteobacteria;c__Gammaproteobacteria;o__Enterobacterales;f__Erwiniaceae;g__Pantoea;s__Pantoea_sp._ARC607

PB2_gene_137	546	ORM72619.1	546	523/546(95.79)	0	phosphoethanolamine transferase EptA [Pantoea deleyi]	470932	d__Bacteria;k__norank;p__Proteobacteria;c__Gammaproteobacteria;o__Enterobacterales;f__Erwiniaceae;g__Pantoea;s__Pantoea_deleyi
PB2_gene_146	225	ORM72615.1	228	222/225(98.67)	2.75e-142	ABC transporter permease [Pantoea deleyi]	470932	d__Bacteria;k__norank;p__Proteobacteria;c__Gammaproteobacteria;o__Enterobacterales;f__Erwiniaceae;g__Pantoea;s__Pantoea_deleyi
PB2_gene_148	286	ORM72661.1	281	277/281(98.58)	9.14e-190	ABC transporter substrate-binding protein [Pantoea deleyi]	470932	d__Bacteria;k__norank;p__Proteobacteria;c__Gammaproteobacteria;o__Enterobacterales;f__Erwiniaceae;g__Pantoea;s__Pantoea_deleyi
PB2_gene_150	277	ORM80503.1	277	277/277(100.00)	1.29e-188	sulfate ABC transporter permease subunit CysT [Pantoea deleyi]	470932	d__Bacteria;k__norank;p__Proteobacteria;c__Gammaproteobacteria;o__Enterobacterales;f__Erwiniaceae;g__Pantoea;s__Pantoea_deleyi
PB2_gene_152	225	ORM80501.1	205	199/205(97.07)	1.25e-144	RpoE-regulated lipoprotein [Pantoea deleyi]	470932	d__Bacteria;k__norank;p__Proteobacteria;c__Gammaproteobacteria;o__Enterobacterales;f__Erwiniaceae;g__Pantoea;s__Pantoea_deleyi
PB2_gene_153	155	WP_111139353.1	155	150/155(96.77)	4.36e-104	DUF2919 domain-containing protein [Pantoea sp. ARC607]	2027922	d__Bacteria;k__norank;p__Proteobacteria;c__Gammaproteobacteria;o__Enterobacterales;f__Erwiniaceae;g__Pantoea;s__Pantoea_sp._ARC607
PB2_gene_154	141	ORM76909.1	141	141/141(100.00)	8.44e-102	GNAT family acetyltransferase [Pantoea deleyi]	470932	d__Bacteria;k__norank;p__Proteobacteria;c__Gammaproteobacteria;o__Enterobacterales;f__Erwiniaceae;g__Pantoea;s__Pantoea_deleyi
PB2_gene_158	304	WP_111139350.1	304	300/303(99.01)	1.11e-232	oxygen-dependent coproporphyrinogen oxidase [Pantoea sp. ARC607]	2027922	d__Bacteria;k__norank;p__Proteobacteria;c__Gammaproteobacteria;o__Enterobacterales;f__Erwiniaceae;g__Pantoea;s__Pantoea_sp._ARC607
PB2_gene_161	387	ORM76914.1	387	387/387(100.00)	1.94e-284	D-alanyl-D-alanine-carboxypeptidase/endopeptidase AmpH [Pantoea deleyi]	470932	d__Bacteria;k__norank;p__Proteobacteria;c__Gammaproteobacteria;o__Enterobacterales;f__Erwiniaceae;g__Pantoea;s__Pantoea_deleyi
PB2_gene_162	357	WP_111139346.1	357	349/357(97.76)	1.31e-260	DUF1176 domain-containing protein [Pantoea sp. ARC607]	2027922	d__Bacteria;k__norank;p__Proteobacteria;c__Gammaproteobacteria;o__Enterobacterales;f__Erwiniaceae;g__Pantoea;s__Pantoea_sp._ARC607
PB2_gene_179	723	BAH22244.1	723	713/723(98.62)	0	phage tail tape measure protein [Wolbachia endosymbiont of Cadra cautella]	190193	d__Bacteria;k__norank;p__Proteobacteria;c__Alphaproteobacteria;o__Rickettsiales;f__Anaplasmataceae;g__Wolbachia;s__Wolbachia_endosymbiont_of_Cadra_cautella
PB2_gene_181	431	KTS71821.1	431	408/431(94.66)	9.53e-296	2-ketogluconate transporter [Pantoea stewartii]	66269	d__Bacteria;k__norank;p__Proteobacteria;c__Gammaproteobacteria;o__Enterobacterales;f__Erwiniaceae;g__Pantoea;s__Pantoea_stewartii
PB2_gene_182	155	ARF48496.1	155	140/155(90.32)	5.72e-100	hypothetical protein DSJ_03415 [Pantoea stewartii subsp. stewartii DC283]	660596	d__Bacteria;k__norank;p__Proteobacteria;c__Gammaproteobacteria;o__Enterobacterales;f__Erwiniaceae;g__Pantoea;s__Pantoea_stewartii
PB2_gene_188	274	ARF48490.1	270	254/270(94.07)	2.91e-178	transcriptional regulator [Pantoea stewartii subsp. stewartii DC283]	660596	d__Bacteria;k__norank;p__Proteobacteria;c__Gammaproteobacteria;o__Enterobacterales;f__Erwiniaceae;g__Pantoea;s__Pantoea_stewartii
PB2_gene_189	148	WP_031590445.1	163	133/148(89.86)	1.23e-94	DUF1795 domain-containing protein [Pantoea agglomerans]	549	d__Bacteria;k__norank;p__Proteobacteria;c__Gammaproteobacteria;o__Enterobacterales;f__Erwiniaceae;g__Pantoea;s__Pantoea_agglomerans
PB2_gene_190	605	WP_031590444.1	605	448/604(74.17)	0	OmpA family protein [Pantoea agglomerans]	549	d__Bacteria;k__norank;p__Proteobacteria;c__Gammaproteobacteria;o__Enterobacterales;f__Erwiniaceae;g__Pantoea;s__Pantoea_agglomerans
PB2_gene_195	378	ORM80477.1	377	366/377(97.08)	1.55e-267	4-phosphoerythronate dehydrogenase [Pantoea deleyi]	470932	d__Bacteria;k__norank;p__Proteobacteria;c__Gammaproteobacteria;o__Enterobacterales;f__Erwiniaceae;g__Pantoea;s__Pantoea_deleyi
PB2_gene_197	267	ORM80474.1	267	267/267(100.00)	4.24e-198	tRNA pseudouridine(38-40) synthase TruA [Pantoea deleyi]	470932	d__Bacteria;k__norank;p__Proteobacteria;c__Gammaproteobacteria;o__Enterobacterales;f__Erwiniaceae;g__Pantoea;s__Pantoea_deleyi
PB2_gene_199	304	WP_046102281.1	304	299/304(98.36)	3.00e-212	acetyl-CoA carboxylase, carboxyltransferase subunit beta [Pantoea anthophila]	53335	d__Bacteria;k__norank;p__Proteobacteria;c__Gammaproteobacteria;o__Enterobacterales;f__Erwiniaceae;g__Pantoea;s__norank
PB2_gene_200	422	WP_111140453.1	422	410/422(97.16)	5.40e-286	bifunctional tetrahydrofolate synthase/dihydrofolate synthase [Pantoea sp. ARC607]	2027922	d__Bacteria;k__norank;p__Proteobacteria;c__Gammaproteobacteria;o__Enterobacterales;f__Erwiniaceae;g__Pantoea;s__Pantoea_sp._ARC607
PB2_gene_201	272	WP_111140454.1	262	249/271(91.88)	7.74e-123	cell division protein DedD [Pantoea sp. ARC607]	2027922	d__Bacteria;k__norank;p__Proteobacteria;c__Gammaproteobacteria;o__Enterobacterales;f__Erwiniaceae;g__Pantoea;s__Pantoea_sp._ARC607
PB2_gene_204	505	ORM83881.1	505	505/505(100.00)	0	amidophosphoribosyltransferase [Pantoea deleyi]	470932	d__Bacteria;k__norank;p__Proteobacteria;c__Gammaproteobacteria;o__Enterobacterales;f__Erwiniaceae;g__Pantoea;s__Pantoea_deleyi

PB2_gene_208	356	WP_111140011.1	356	354/356(99.44)	3.06e-254	tRNA preQ1(34) S-adenosylmethionine ribosyltransferase-isomerase QueA [Pantoea sp. ARC607]	2027922	d__Bacteria;k__norank;p__Proteobacteria;c__Gammaproteobacteria;o__Enterobacterales;f__Erwiniaceae;g__Pantoea;s__Pantoea_sp._ARC607
PB2_gene_209	382	ORM79510.1	382	381/382(99.74)	1.73e-289	tRNA guanosine(34) transglycosylase Tgt [Pantoea deleyi]	470932	d__Bacteria;k__norank;p__Proteobacteria;c__Gammaproteobacteria;o__Enterobacterales;f__Erwiniaceae;g__Pantoea;s__Pantoea_deleyi
PB2_gene_215	117	OQV38556.1	149	117/117(100.00)	2.45e-76	transcriptional regulator NrdR [Pantoea vagans]	470934	d__Bacteria;k__norank;p__Proteobacteria;c__Gammaproteobacteria;o__Enterobacterales;f__Erwiniaceae;g__Pantoea;s__Pantoea_vagans
PB2_gene_216	366	WP_111140006.1	366	359/366(98.09)	5.33e-260	bifunctional diaminohydroxyphosphoribosylaminopyrimidine deaminase/5-amino-6-(5-phosphoribosylamino)uracil reductase RibD [Pantoea sp.	2027922	d__Bacteria;k__norank;p__Proteobacteria;c__Gammaproteobacteria;o__Enterobacterales;f__Erwiniaceae;g__Pantoea;s__Pantoea_sp._ARC607
PB2_gene_220	159	WP_111140002.1	159	156/159(98.11)	1.34e-118	phosphatidylglycerophosphatase A [Pantoea sp. ARC607]	2027922	d__Bacteria;k__norank;p__Proteobacteria;c__Gammaproteobacteria;o__Enterobacterales;f__Erwiniaceae;g__Pantoea;s__Pantoea_sp._ARC607
PB2_gene_223	143	SJZ70816.1	143	143/143(100.00)	2.60e-97	nucleoside diphosphate kinase [Pantoea agglomerans]	470933	d__Bacteria;k__norank;p__Proteobacteria;c__Gammaproteobacteria;o__Enterobacterales;f__Erwiniaceae;g__Pantoea;s__Pantoea_eucalypti
PB2_gene_224	389	ORM75432.1	389	389/389(100.00)	3.61e-281	bifunctional tRNA (adenosine(37)-C2)-methyltransferase TrmG/ribosomal RNA large subunit methyltransferase RlmN	470932	d__Bacteria;k__norank;p__Proteobacteria;c__Gammaproteobacteria;o__Enterobacterales;f__Erwiniaceae;g__Pantoea;s__Pantoea_deleyi
PB2_gene_239	326	SUB05579.1	326	306/325(94.15)	4.19e-230	Prolyl oligopeptidase family [Pantoea agglomerans]	549	d__Bacteria;k__norank;p__Proteobacteria;c__Gammaproteobacteria;o__Enterobacterales;f__Erwiniaceae;g__Pantoea;s__Pantoea_agglomerans
PB2_gene_241	448	WP_111139300.1	448	442/447(98.88)	1.07e-312	sodium:proton antiporter [Pantoea sp. ARC607]	2027922	d__Bacteria;k__norank;p__Proteobacteria;c__Gammaproteobacteria;o__Enterobacterales;f__Erwiniaceae;g__Pantoea;s__Pantoea_sp._ARC607
PB2_gene_242	400	WP_111139301.1	400	390/400(97.50)	2.39e-255	MFS transporter [Pantoea sp. ARC607]	2027922	d__Bacteria;k__norank;p__Proteobacteria;c__Gammaproteobacteria;o__Enterobacterales;f__Erwiniaceae;g__Pantoea;s__Pantoea_sp._ARC607
PB2_gene_244	165	ORM75411.1	165	165/165(100.00)	2.47e-108	hypothetical protein HA42_20755 [Pantoea deleyi]	470932	d__Bacteria;k__norank;p__Proteobacteria;c__Gammaproteobacteria;o__Enterobacterales;f__Erwiniaceae;g__Pantoea;s__Pantoea_deleyi
PB2_gene_250	412	ORM81344.1	412	409/412(99.27)	2.94e-294	hypothetical protein HA42_11120 [Pantoea deleyi]	470932	d__Bacteria;k__norank;p__Proteobacteria;c__Gammaproteobacteria;o__Enterobacterales;f__Erwiniaceae;g__Pantoea;s__Pantoea_deleyi
PB2_gene_251	1208	ORM81345.1	1209	1185/1209(98.01)	0	type VI secretion system protein ImpL [Pantoea deleyi]	470932	d__Bacteria;k__norank;p__Proteobacteria;c__Gammaproteobacteria;o__Enterobacterales;f__Erwiniaceae;g__Pantoea;s__Pantoea_deleyi
PB2_gene_254	181	ORM77607.1	177	176/177(99.44)	1.59e-120	type VI secretion system-associated protein [Pantoea	470932	d__Bacteria;k__norank;p__Proteobacteria;c__Gammaproteobacteria;o__Enterobacterales;f__Erwiniaceae;g__Pantoea;s__Pantoea_deleyi
PB2_gene_255	499	WP_111140778.1	499	499/499(100.00)	0	type VI secretion system contractile sheath large subunit [Pantoea sp. ARC607]	2027922	d__Bacteria;k__norank;p__Proteobacteria;c__Gammaproteobacteria;o__Enterobacterales;f__Erwiniaceae;g__Pantoea;s__Pantoea_sp._ARC607
PB2_gene_260	136	ORM82921.1	136	135/136(99.26)	2.55e-87	phosphate-starvation-inducible protein PsiE [Pantoea deleyi]	470932	d__Bacteria;k__norank;p__Proteobacteria;c__Gammaproteobacteria;o__Enterobacterales;f__Erwiniaceae;g__Pantoea;s__Pantoea_deleyi
PB2_gene_262	353	WP_031591814.1	353	348/353(98.58)	3.84e-251	extracellular solute-binding protein [Pantoea agglomerans]	549	d__Bacteria;k__norank;p__Proteobacteria;c__Gammaproteobacteria;o__Enterobacterales;f__Erwiniaceae;g__Pantoea;s__Pantoea_agglomerans
PB2_gene_263	270	WP_111141939.1	270	268/270(99.26)	1.22e-203	alkaline phosphatase family protein [Pantoea sp. ARC607]	2027922	d__Bacteria;k__norank;p__Proteobacteria;c__Gammaproteobacteria;o__Enterobacterales;f__Erwiniaceae;g__Pantoea;s__Pantoea_sp._ARC607
PB2_gene_266	330	ORM82915.1	330	325/330(98.48)	9.07e-227	spermidine/putrescine ABC transporter ATP-binding protein [Pantoea deleyi]	470932	d__Bacteria;k__norank;p__Proteobacteria;c__Gammaproteobacteria;o__Enterobacterales;f__Erwiniaceae;g__Pantoea;s__Pantoea_deleyi

PB2_gene_269	265	ORM82913.1	230	228/230(99.13)	1.28e-151	aquaporin Z [Pantoea deleyi]	470932	d__Bacteria;k__norank;p__Proteobacteria;c__Gammaproteobacteria;o__Enterobacterales;f__Erwiniaceae;g__Pantoea;s__Pantoea_deleyi
PB2_gene_282	327	WP_098051694.1	327	325/327(99.39)	7.82e-231	arabinose-5-phosphate isomerase KdsD [Pantoea agglomerans]	None	Unclassified
PB2_gene_283	331	ORM86328.1	326	324/326(99.39)	7.99e-218	calcium/sodium antiporter [Pantoea deleyi]	470932	d__Bacteria;k__norank;p__Proteobacteria;c__Gammaproteobacteria;o__Enterobacterales;f__Erwiniaceae;g__Pantoea;s__Pantoea_deleyi
PB2_gene_285	260	ORM86330.1	260	258/260(99.23)	5.01e-173	ABC transporter permease [Pantoea deleyi]	470932	d__Bacteria;k__norank;p__Proteobacteria;c__Gammaproteobacteria;o__Enterobacterales;f__Erwiniaceae;g__Pantoea;s__Pantoea_deleyi
PB2_gene_291	335	ORM85069.1	335	334/335(99.70)	1.04e-239	flagellar motor switch protein FliM [Pantoea deleyi]	470932	d__Bacteria;k__norank;p__Proteobacteria;c__Gammaproteobacteria;o__Enterobacterales;f__Erwiniaceae;g__Pantoea;s__Pantoea_deleyi
PB2_gene_293	421	ORM85067.1	421	394/421(93.59)	1.26e-256	flagellar hook-length control protein [Pantoea deleyi]	470932	d__Bacteria;k__norank;p__Proteobacteria;c__Gammaproteobacteria;o__Enterobacterales;f__Erwiniaceae;g__Pantoea;s__Pantoea_deleyi
PB2_gene_295	454	WP_111139990.1	454	453/454(99.78)	0	flagellum-specific ATP synthase FliI [Pantoea sp. ARC607]	2027922	d__Bacteria;k__norank;p__Proteobacteria;c__Gammaproteobacteria;o__Enterobacterales;f__Erwiniaceae;g__Pantoea;s__Pantoea_sp._ARC607
PB2_gene_298	574	WP_010670852.1	574	544/574(94.77)	0	flagellar basal body M-ring protein FliF [Pantoea	549	d__Bacteria;k__norank;p__Proteobacteria;c__Gammaproteobacteria;o__Enterobacterales;f__Erwiniaceae;g__Pantoea;s__Pantoea_agglomerans
PB2_gene_305	163	ORM77682.1	162	149/162(91.98)	1.74e-100	hypothetical protein HA42_17475 [Pantoea deleyi]	470932	d__Bacteria;k__norank;p__Proteobacteria;c__Gammaproteobacteria;o__Enterobacterales;f__Erwiniaceae;g__Pantoea;s__Pantoea_deleyi
PB2_gene_306	181	WP_111139731.1	181	147/178(82.58)	4.54e-89	fimbrial assembly protein [Pantoea sp. ARC607]	2027922	d__Bacteria;k__norank;p__Proteobacteria;c__Gammaproteobacteria;o__Enterobacterales;f__Erwiniaceae;g__Pantoea;s__Pantoea_sp._ARC607
PB2_gene_307	279	KDA94456.1	280	236/279(84.59)	1.60e-165	pilus assembly protein HofM [Pantoea agglomerans Eh318]	1408177	d__Bacteria;k__norank;p__Proteobacteria;c__Gammaproteobacteria;o__Enterobacterales;f__Erwiniaceae;g__Pantoea;s__Pantoea_agglomerans
PB2_gene_308	849	ORM80876.1	849	842/848(99.29)	0	carboxypeptidase/penicillin-binding protein 1A [Pantoea	470932	d__Bacteria;k__norank;p__Proteobacteria;c__Gammaproteobacteria;o__Enterobacterales;f__Erwiniaceae;g__Pantoea;s__Pantoea_deleyi
PB2_gene_312	432	ORM80873.1	432	427/432(98.84)	2.22e-290	allantoin permease [Pantoea deleyi]	470932	d__Bacteria;k__norank;p__Proteobacteria;c__Gammaproteobacteria;o__Enterobacterales;f__Erwiniaceae;g__Pantoea;s__Pantoea_deleyi
PB2_gene_314	711	ORM80872.1	711	700/711(98.45)	0	Intracellular growth attenuator protein igaA [Pantoea deleyi]	470932	d__Bacteria;k__norank;p__Proteobacteria;c__Gammaproteobacteria;o__Enterobacterales;f__Erwiniaceae;g__Pantoea;s__Pantoea_deleyi
PB2_gene_317	178	ORM76741.1	178	173/178(97.19)	1.35e-125	phosphinothricin acetyltransferase [Pantoea deleyi]	470932	d__Bacteria;k__norank;p__Proteobacteria;c__Gammaproteobacteria;o__Enterobacterales;f__Erwiniaceae;g__Pantoea;s__Pantoea_deleyi
PB2_gene_322	691	ORM76420.1	691	686/691(99.28)	0	glycogen debranching enzyme GlgX [Pantoea deleyi]	470932	d__Bacteria;k__norank;p__Proteobacteria;c__Gammaproteobacteria;o__Enterobacterales;f__Erwiniaceae;g__Pantoea;s__Pantoea_deleyi
PB2_gene_326	88	ORM75753.1	96	84/88(95.45)	5.79e-54	hypothetical protein HA42_19985 [Pantoea deleyi]	470932	d__Bacteria;k__norank;p__Proteobacteria;c__Gammaproteobacteria;o__Enterobacterales;f__Erwiniaceae;g__Pantoea;s__Pantoea_deleyi
PB2_gene_329	304	WP_065095474.1	303	278/304(91.45)	4.04e-187	MULTISPECIES: XRE family transcriptional regulator	2640676	d__Bacteria;k__norank;p__Proteobacteria;c__Alphaproteobacteria;o__Rickettsiales;f__Anaplasmataceae;g__Wolbachia;s__norank
PB2_gene_333	321	WP_114517424.1	321	312/321(97.20)	5.04e-224	phage tail protein [Wolbachia endosymbiont of Cylisticus convexus]	118728	d__Bacteria;k__norank;p__Proteobacteria;c__Alphaproteobacteria;o__Rickettsiales;f__Anaplasmataceae;g__Wolbachia;s__Wolbachia_endosymbiont_of_Cylisticus_convexus
PB2_gene_341	473	BAH22237.1	473	453/473(95.77)	4.36e-288	hypothetical protein [Wolbachia endosymbiont of Cadra cautella]	190193	d__Bacteria;k__norank;p__Proteobacteria;c__Alphaproteobacteria;o__Rickettsiales;f__Anaplasmataceae;g__Wolbachia;s__Wolbachia_endosymbiont_of_Cadra_cautella
PB2_gene_344	70	PXW21864.1	47	45/47(95.74)	1.54e-20	hypothetical protein BY447_3594 [Pantoea sp. JKS000250]	1938795	d__Bacteria;k__norank;p__Proteobacteria;c__Gammaproteobacteria;o__Enterobacterales;f__Erwiniaceae;g__Pantoea;s__Pantoea_sp._JKS000250
PB2_gene_345	61	ORM75674.1	63	57/61(93.44)	4.07e-34	hypothetical protein HA42_20125 [Pantoea deleyi]	470932	d__Bacteria;k__norank;p__Proteobacteria;c__Gammaproteobacteria;o__Enterobacterales;f__Erwiniaceae;g__Pantoea;s__Pantoea_deleyi
PB2_gene_346	387	ORM75673.1	387	375/387(96.90)	4.97e-287	beta-glucosidase [Pantoea deleyi]	470932	d__Bacteria;k__norank;p__Proteobacteria;c__Gammaproteobacteria;o__Enterobacterales;f__Erwiniaceae;g__Pantoea;s__Pantoea_deleyi
PB2_gene_347	486	WP_111138607.1	486	473/486(97.33)	0	phospholipase D family protein [Pantoea sp. ARC607]	2027922	d__Bacteria;k__norank;p__Proteobacteria;c__Gammaproteobacteria;o__Enterobacterales;f__Erwiniaceae;g__Pantoea;s__Pantoea_sp._ARC607
PB2_gene_349	521	PXW21869.1	521	506/521(97.12)	0	glucans biosynthesis protein [Pantoea sp. JKS000250]	1938795	d__Bacteria;k__norank;p__Proteobacteria;c__Gammaproteobacteria;o__Enterobacterales;f__Erwiniaceae;g__Pantoea;s__Pantoea_sp._JKS000250

PB2_gene_350	849	ORM81037.1	849	842/849(99.18)	0	glucan biosynthesis glucosyltransferase H [Pantoea deleyi]	470932	d__Bacteria;k__norank;p__Proteobacteria;c__Gammaproteobacteria;o__Enterobacterales;f__Erwiniaceae;g__Pantoea;s__Pantoea_deleyi
PB2_gene_364	397	ORM84531.1	397	394/397(99.24)	4.18e-275	multidrug transporter EmrD [Pantoea deleyi]	470932	d__Bacteria;k__norank;p__Proteobacteria;c__Gammaproteobacteria;o__Enterobacterales;f__Erwiniaceae;g__Pantoea;s__Pantoea_deleyi
PB2_gene_365	336	ORM84532.1	336	333/336(99.11)	6.98e-234	fructose-bisphosphatase, class II [Pantoea deleyi]	470932	d__Bacteria;k__norank;p__Proteobacteria;c__Gammaproteobacteria;o__Enterobacterales;f__Erwiniaceae;g__Pantoea;s__Pantoea_deleyi
PB2_gene_369	1075	WP_111139373.1	1075	1071/1075(99.63)	0	carbamoyl-phosphate synthase large subunit [Pantoea sp.	2027922	d__Bacteria;k__norank;p__Proteobacteria;c__Gammaproteobacteria;o__Enterobacterales;f__Erwiniaceae;g__Pantoea;s__Pantoea_sp._ARC607
PB2_gene_370	391	KGD78765.1	391	381/391(97.44)	2.09e-281	carbamoyl phosphate synthase small subunit [Pantoea vagans]	470934	d__Bacteria;k__norank;p__Proteobacteria;c__Gammaproteobacteria;o__Enterobacterales;f__Erwiniaceae;g__Pantoea;s__Pantoea_vagans
PB2_gene_372	317	WP_111139370.1	317	317/317(100.00)	4.43e-216	4-hydroxy-3-methylbut-2-enyl diphosphate reductase [Pantoea sp. ARC607]	2027922	d__Bacteria;k__norank;p__Proteobacteria;c__Gammaproteobacteria;o__Enterobacterales;f__Erwiniaceae;g__Pantoea;s__Pantoea_sp._ARC607
PB2_gene_378	573	WP_090963832.1	566	505/573(88.13)	0	protein-disulfide reductase DsbD [Pantoea sp. OV426]	1881036	d__Bacteria;k__norank;p__Proteobacteria;c__Gammaproteobacteria;o__Enterobacterales;f__Erwiniaceae;g__Pantoea;s__Pantoea_sp._OV426
PB2_gene_380	433	WP_111142143.1	433	425/433(98.15)	2.61e-278	anaerobic C4-dicarboxylate transporter [Pantoea sp. ARC607]	2027922	d__Bacteria;k__norank;p__Proteobacteria;c__Gammaproteobacteria;o__Enterobacterales;f__Erwiniaceae;g__Pantoea;s__Pantoea_sp._ARC607
PB2_gene_384	549	ORM86300.1	549	536/537(99.81)	0	chaperonin GroL [Pantoea deleyi]	470932	d__Bacteria;k__norank;p__Proteobacteria;c__Gammaproteobacteria;o__Enterobacterales;f__Erwiniaceae;g__Pantoea;s__Pantoea_deleyi
PB2_gene_385	115	ORM86302.1	115	115/115(100.00)	4.07e-74	hypothetical protein HA42_00775 [Pantoea deleyi]	470932	d__Bacteria;k__norank;p__Proteobacteria;c__Gammaproteobacteria;o__Enterobacterales;f__Erwiniaceae;g__Pantoea;s__Pantoea_deleyi
PB2_gene_403	192	VEC01732.1	192	137/192(71.35)	2.72e-100	Uncharacterised protein [Cedecea lapagei]	158823	d__Bacteria;k__norank;p__Proteobacteria;c__Gammaproteobacteria;o__Enterobacterales;f__Enterobacteriaceae;g__Cedecea;s__Cedecea_lapagei
PB2_gene_404	95	ORM81635.1	756	43/45(95.56)	9.77e-20	5-methyltetrahydropteroyltriglutamate--homocysteine S-methyltransferase [Pantoea	470932	d__Bacteria;k__norank;p__Proteobacteria;c__Gammaproteobacteria;o__Enterobacterales;f__Erwiniaceae;g__Pantoea;s__Pantoea_deleyi
PB2_gene_405	47	SUB04729.1	252	41/47(87.23)	2.13e-17	5-methyltetrahydropteroyltriglutamate--homocysteine methyltransferase [Pantoea	549	d__Bacteria;k__norank;p__Proteobacteria;c__Gammaproteobacteria;o__Enterobacterales;f__Erwiniaceae;g__Pantoea;s__Pantoea_agglomerans
PB2_gene_406	308	WP_111139753.1	308	305/308(99.03)	1.39e-219	HTH-type transcriptional regulator MetR [Pantoea sp.	2027922	d__Bacteria;k__norank;p__Proteobacteria;c__Gammaproteobacteria;o__Enterobacterales;f__Erwiniaceae;g__Pantoea;s__Pantoea_sp._ARC607
PB2_gene_408	312	PXW20685.1	312	299/312(95.83)	3.51e-215	carboxylate/amino acid/amine transporter [Pantoea sp.	1938795	d__Bacteria;k__norank;p__Proteobacteria;c__Gammaproteobacteria;o__Enterobacterales;f__Erwiniaceae;g__Pantoea;s__Pantoea_sp._JKS000250
PB2_gene_410	266	WP_111139757.1	266	262/265(98.87)	2.01e-191	sugar/pyridoxal phosphate phosphatase YigL [Pantoea sp. ARC607]	2027922	d__Bacteria;k__norank;p__Proteobacteria;c__Gammaproteobacteria;o__Enterobacterales;f__Erwiniaceae;g__Pantoea;s__Pantoea_sp._ARC607
PB2_gene_415	246	WP_125355431.1	237	159/234(67.95)	7.85e-108	ATP-binding cassette domain-containing protein [Enterobacteriaceae bacterium CCUG 66741]	1851514	d__Bacteria;k__norank;p__Proteobacteria;c__Gammaproteobacteria;o__Enterobacterales;f__Enterobacteriaceae;g__Scandinavium;s__Scandinavium_goeteborgense
PB2_gene_419	146	WP_111140648.1	136	123/136(90.44)	5.24e-85	DUF937 domain-containing protein [Pantoea sp. ARC607]	2027922	d__Bacteria;k__norank;p__Proteobacteria;c__Gammaproteobacteria;o__Enterobacterales;f__Erwiniaceae;g__Pantoea;s__Pantoea_sp._ARC607
PB2_gene_421	658	ORM86539.1	658	658/658(100.00)	0	arginine decarboxylase [Pantoea deleyi]	470932	d__Bacteria;k__norank;p__Proteobacteria;c__Gammaproteobacteria;o__Enterobacterales;f__Erwiniaceae;g__Pantoea;s__Pantoea_deleyi
PB2_gene_429	156	ORM85456.1	156	155/155(100.00)	2.33e-79	F0F1 ATP synthase subunit B [Pantoea deleyi]	470932	d__Bacteria;k__norank;p__Proteobacteria;c__Gammaproteobacteria;o__Enterobacterales;f__Erwiniaceae;g__Pantoea;s__Pantoea_deleyi

PB2_gene_440	548	ORM82121.1	548	541/548(98.72)	0	acetolactate synthase 2 catalytic subunit [Pantoea deleyi]	470932	d__Bacteria;k__norank;p__Proteobacteria;c__Gammaproteobacteria;o__Enterobacterales;f__Erwiniaceae;g__Pantoea;s__Pantoea_deleyi
PB2_gene_441	85	SKA13068.1	85	85/85(100.00)	9.72e-50	acetolactate synthase, small subunit [Pantoea agglomerans]	470933	d__Bacteria;k__norank;p__Proteobacteria;c__Gammaproteobacteria;o__Enterobacterales;f__Erwiniaceae;g__Pantoea;s__Pantoea_eucalypti
PB2_gene_442	309	ORM82123.1	309	309/309(100.00)	1.45e-226	branched chain amino acid aminotransferase [Pantoea deleyi]	470932	d__Bacteria;k__norank;p__Proteobacteria;c__Gammaproteobacteria;o__Enterobacterales;f__Erwiniaceae;g__Pantoea;s__Pantoea_deleyi
PB2_gene_443	616	ORM82124.1	616	614/616(99.68)	0	dihydroxy-acid dehydratase [Pantoea deleyi]	470932	d__Bacteria;k__norank;p__Proteobacteria;c__Gammaproteobacteria;o__Enterobacterales;f__Erwiniaceae;g__Pantoea;s__Pantoea_deleyi
PB2_gene_450	93	ORM81503.1	263	54/69(78.26)	9.18e-25	inositol monophosphatase [Pantoea deleyi]	470932	d__Bacteria;k__norank;p__Proteobacteria;c__Gammaproteobacteria;o__Enterobacterales;f__Erwiniaceae;g__Pantoea;s__Pantoea_deleyi
PB2_gene_453	324	WP_111140512.1	297	294/297(98.99)	1.04e-211	SDR family oxidoreductase [Pantoea sp. ARC607]	53335	d__Bacteria;k__norank;p__Proteobacteria;c__Gammaproteobacteria;o__Enterobacterales;f__Erwiniaceae;g__Pantoea;s__norank
PB2_gene_454	159	ORM76167.1	155	143/155(92.26)	5.76e-101	hypothetical protein HA42_19435, partial [Pantoea	470932	d__Bacteria;k__norank;p__Proteobacteria;c__Gammaproteobacteria;o__Enterobacterales;f__Erwiniaceae;g__Pantoea;s__Pantoea_deleyi
PB2_gene_457	81	WP_111140515.1	81	77/81(95.06)	9.64e-46	hypothetical protein [Pantoea sp. ARC607]	2027922	d__Bacteria;k__norank;p__Proteobacteria;c__Gammaproteobacteria;o__Enterobacterales;f__Erwiniaceae;g__Pantoea;s__Pantoea_sp._ARC607
PB2_gene_458	393	ORM76164.1	393	386/393(98.22)	3.47e-281	glycosyl transferase [Pantoea deleyi]	470932	d__Bacteria;k__norank;p__Proteobacteria;c__Gammaproteobacteria;o__Enterobacterales;f__Erwiniaceae;g__Pantoea;s__Pantoea_deleyi
PB2_gene_461	344	WP_111140518.1	681	328/344(95.35)	2.11e-243	peptidyl-dipeptidase Dcp [Pantoea sp. ARC607]	2027922	d__Bacteria;k__norank;p__Proteobacteria;c__Gammaproteobacteria;o__Enterobacterales;f__Erwiniaceae;g__Pantoea;s__Pantoea_sp._ARC607
PB2_gene_464	485	WP_111141248.1	485	473/485(97.53)	0	PLP-dependent aminotransferase family protein [Pantoea sp.	2027922	d__Bacteria;k__norank;p__Proteobacteria;c__Gammaproteobacteria;o__Enterobacterales;f__Erwiniaceae;g__Pantoea;s__Pantoea_sp._ARC607
PB2_gene_465	211	WP_111141247.1	211	201/211(95.26)	1.29e-137	thiamine phosphate synthase [Pantoea sp. ARC607]	2027922	d__Bacteria;k__norank;p__Proteobacteria;c__Gammaproteobacteria;o__Enterobacterales;f__Erwiniaceae;g__Pantoea;s__Pantoea_sp._ARC607
PB2_gene_466	635	ORM80947.1	640	622/635(97.95)	0	phosphomethylpyrimidine synthase ThiC [Pantoea deleyi]	470932	d__Bacteria;k__norank;p__Proteobacteria;c__Gammaproteobacteria;o__Enterobacterales;f__Erwiniaceae;g__Pantoea;s__Pantoea_deleyi
PB2_gene_468	256	ORM80949.1	256	255/256(99.61)	5.56e-193	NADH pyrophosphatase [Pantoea deleyi]	470932	d__Bacteria;k__norank;p__Proteobacteria;c__Gammaproteobacteria;o__Enterobacterales;f__Erwiniaceae;g__Pantoea;s__Pantoea_deleyi
PB2_gene_472	231	ADD76855.1	232	196/225(87.11)	5.59e-149	YdjM [Pantoea ananatis LMG 20103]	706191	d__Bacteria;k__norank;p__Proteobacteria;c__Gammaproteobacteria;o__Enterobacterales;f__Erwiniaceae;g__Pantoea;s__Pantoea_ananatis
PB2_gene_474	278	ORM79527.1	278	262/278(94.24)	1.16e-196	5-dehydro-4-deoxy-D-glucuronate isomerase [Pantoea	470932	d__Bacteria;k__norank;p__Proteobacteria;c__Gammaproteobacteria;o__Enterobacterales;f__Erwiniaceae;g__Pantoea;s__Pantoea_deleyi
PB2_gene_476	178	ORM79529.1	178	177/178(99.44)	4.01e-118	hypothetical protein HA42_15310 [Pantoea deleyi]	470932	d__Bacteria;k__norank;p__Proteobacteria;c__Gammaproteobacteria;o__Enterobacterales;f__Erwiniaceae;g__Pantoea;s__Pantoea_deleyi
PB2_gene_477	297	ORM79530.1	297	294/297(98.99)	1.15e-226	hypothetical protein HA42_15315 [Pantoea deleyi]	470932	d__Bacteria;k__norank;p__Proteobacteria;c__Gammaproteobacteria;o__Enterobacterales;f__Erwiniaceae;g__Pantoea;s__Pantoea_deleyi
PB2_gene_482	70	KDA95683.1	70	68/70(97.14)	2.79e-40	50S ribosomal protein L35 [Pantoea agglomerans Eh318]	1408177	d__Bacteria;k__norank;p__Proteobacteria;c__Gammaproteobacteria;o__Enterobacterales;f__Erwiniaceae;g__Pantoea;s__Pantoea_agglomerans
PB2_gene_485	379	ORM82836.1	379	369/379(97.36)	5.52e-280	glycosyl hydrolase family 88 [Pantoea deleyi]	470932	d__Bacteria;k__norank;p__Proteobacteria;c__Gammaproteobacteria;o__Enterobacterales;f__Erwiniaceae;g__Pantoea;s__Pantoea_deleyi
PB2_gene_486	478	WP_111138654.1	478	475/478(99.37)	0	PTS glucose transporter subunit IIBC [Pantoea sp. ARC607]	2027922	d__Bacteria;k__norank;p__Proteobacteria;c__Gammaproteobacteria;o__Enterobacterales;f__Erwiniaceae;g__Pantoea;s__Pantoea_sp._ARC607
PB2_gene_488	329	ORM82833.1	329	322/329(97.87)	4.59e-240	DNA polymerase III subunit delta' [Pantoea deleyi]	470932	d__Bacteria;k__norank;p__Proteobacteria;c__Gammaproteobacteria;o__Enterobacterales;f__Erwiniaceae;g__Pantoea;s__Pantoea_deleyi
PB2_gene_494	244	ORM82827.1	244	243/244(99.59)	8.94e-159	3-oxoacyl-ACP reductase [Pantoea deleyi]	470932	d__Bacteria;k__norank;p__Proteobacteria;c__Gammaproteobacteria;o__Enterobacterales;f__Erwiniaceae;g__Pantoea;s__Pantoea_deleyi
PB2_gene_496	712	ORM76262.1	714	703/712(98.74)	0	ATP-binding protein [Pantoea deleyi]	470932	d__Bacteria;k__norank;p__Proteobacteria;c__Gammaproteobacteria;o__Enterobacterales;f__Erwiniaceae;g__Pantoea;s__Pantoea_deleyi
PB2_gene_498	370	WP_111138723.1	371	366/371(98.65)	9.35e-270	porin OmpC [Pantoea sp. ARC607]	2027922	d__Bacteria;k__norank;p__Proteobacteria;c__Gammaproteobacteria;o__Enterobacterales;f__Erwiniaceae;g__Pantoea;s__Pantoea_sp._ARC607

PB2_gene_501	349	WP_111138725.1	349	343/349(98.28)	3.67e-247	FAD:protein FMN transferase ApbE [Pantoea sp. ARC607]	2027922	d__Bacteria;k__norank;p__Proteobacteria;c__Gammaproteobacteria;o__Enterobacterales;f__Erwiniaceae;g__Pantoea;s__Pantoea_sp._ARC607
PB2_gene_503	550	ORM81372.1	550	543/550(98.73)	0	multidrug ABC transporter permease/ATP-binding protein [Pantoea deleyi]	470932	d__Bacteria;k__norank;p__Proteobacteria;c__Gammaproteobacteria;o__Enterobacterales;f__Erwiniaceae;g__Pantoea;s__Pantoea_deleyi
PB2_gene_508	325	WP_111142162.1	325	325/325(100.00)	7.28e-235	elongation factor P--(R)-beta-lysine ligase [Pantoea sp.	2027922	d__Bacteria;k__norank;p__Proteobacteria;c__Gammaproteobacteria;o__Enterobacterales;f__Erwiniaceae;g__Pantoea;s__Pantoea_sp._ARC607
PB2_gene_510	457	ORM86309.1	457	456/457(99.78)	7.55e-313	serine endoprotease DegQ [Pantoea deleyi]	470932	d__Bacteria;k__norank;p__Proteobacteria;c__Gammaproteobacteria;o__Enterobacterales;f__Erwiniaceae;g__Pantoea;s__Pantoea_deleyi
PB2_gene_518	86	ORM81906.1	86	82/86(95.35)	2.97e-58	hypothetical protein HA42_09485 [Pantoea deleyi]	470932	d__Bacteria;k__norank;p__Proteobacteria;c__Gammaproteobacteria;o__Enterobacterales;f__Erwiniaceae;g__Pantoea;s__Pantoea_deleyi
PB2_gene_519	394	WP_111140535.1	394	392/394(99.49)	4.71e-259	sugar transporter [Pantoea sp. ARC607]	2027922	d__Bacteria;k__norank;p__Proteobacteria;c__Gammaproteobacteria;o__Enterobacterales;f__Erwiniaceae;g__Pantoea;s__Pantoea_sp._ARC607
PB2_gene_522	216	ORM81910.1	216	207/216(95.83)	1.58e-145	TetR family transcriptional regulator [Pantoea deleyi]	470932	d__Bacteria;k__norank;p__Proteobacteria;c__Gammaproteobacteria;o__Enterobacterales;f__Erwiniaceae;g__Pantoea;s__Pantoea_deleyi
PB2_gene_526	490	ORM81914.1	490	478/490(97.55)	0	rhamnulokinase [Pantoea deleyi]	470932	d__Bacteria;k__norank;p__Proteobacteria;c__Gammaproteobacteria;o__Enterobacterales;f__Erwiniaceae;g__Pantoea;s__Pantoea_deleyi
PB2_gene_530	191	WP_111140397.1	249	185/191(96.86)	3.37e-132	glycerophosphodiester phosphodiesterase [Pantoea sp. ARC607]	2027922	d__Bacteria;k__norank;p__Proteobacteria;c__Gammaproteobacteria;o__Enterobacterales;f__Erwiniaceae;g__Pantoea;s__Pantoea_sp._ARC607
PB2_gene_531	357	WP_111140398.1	357	352/357(98.60)	1.50e-250	sn-glycerol-3-phosphate import ATP-binding protein UgpC [Pantoea sp. ARC607]	2027922	d__Bacteria;k__norank;p__Proteobacteria;c__Gammaproteobacteria;o__Enterobacterales;f__Erwiniaceae;g__Pantoea;s__Pantoea_sp._ARC607
PB2_gene_533	295	ORM80402.1	295	292/295(98.98)	7.81e-199	glycerol-3-phosphate transporter permease [Pantoea deleyi]	470932	d__Bacteria;k__norank;p__Proteobacteria;c__Gammaproteobacteria;o__Enterobacterales;f__Erwiniaceae;g__Pantoea;s__Pantoea_deleyi
PB2_gene_534	438	WP_111140401.1	438	428/438(97.72)	0	sn-glycerol-3-phosphate ABC transporter substrate-binding protein UgpB [Pantoea sp.	2027922	d__Bacteria;k__norank;p__Proteobacteria;c__Gammaproteobacteria;o__Enterobacterales;f__Erwiniaceae;g__Pantoea;s__Pantoea_sp._ARC607
PB2_gene_543	282	ORM82131.1	282	278/282(98.58)	2.74e-197	phenazine biosynthesis protein PhzF family [Pantoea deleyi]	470932	d__Bacteria;k__norank;p__Proteobacteria;c__Gammaproteobacteria;o__Enterobacterales;f__Erwiniaceae;g__Pantoea;s__Pantoea_deleyi
PB2_gene_544	252	WP_033784264.1	252	235/252(93.25)	4.81e-167	SDR family oxidoreductase [Pantoea sp. 9140]	1500896	d__Bacteria;k__norank;p__Proteobacteria;c__Gammaproteobacteria;o__Enterobacterales;f__Erwiniaceae;g__Pantoea;s__Pantoea_sp._9140
PB2_gene_545	299	WP_111141113.1	299	296/299(99.00)	3.66e-215	LysR family transcriptional regulator [Pantoea sp. ARC607]	2027922	d__Bacteria;k__norank;p__Proteobacteria;c__Gammaproteobacteria;o__Enterobacterales;f__Erwiniaceae;g__Pantoea;s__Pantoea_sp._ARC607
PB2_gene_546	338	ORM82128.1	338	337/338(99.70)	9.11e-231	inner membrane protein YhjD [Pantoea deleyi]	470932	d__Bacteria;k__norank;p__Proteobacteria;c__Gammaproteobacteria;o__Enterobacterales;f__Erwiniaceae;g__Pantoea;s__Pantoea_deleyi
PB2_gene_547	443	ORM82127.1	443	441/443(99.55)	9.66e-302	transporter [Pantoea deleyi]	470932	d__Bacteria;k__norank;p__Proteobacteria;c__Gammaproteobacteria;o__Enterobacterales;f__Erwiniaceae;g__Pantoea;s__Pantoea_deleyi
PB2_gene_548	479	AVV39184.1	491	479/479(100.00)	0	ketol-acid reductoisomerase [Pantoea vagans]	470934	d__Bacteria;k__norank;p__Proteobacteria;c__Gammaproteobacteria;o__Enterobacterales;f__Erwiniaceae;g__Pantoea;s__Pantoea_vagans
PB2_gene_554	516	ORM79688.1	508	497/508(97.83)	0	maltose ABC transporter permease MalF [Pantoea deleyi]	470932	d__Bacteria;k__norank;p__Proteobacteria;c__Gammaproteobacteria;o__Enterobacterales;f__Erwiniaceae;g__Pantoea;s__Pantoea_deleyi
PB2_gene_559	900	WP_111140076.1	893	879/893(98.43)	0	aconitate hydratase AcnA [Pantoea sp. ARC607]	2027922	d__Bacteria;k__norank;p__Proteobacteria;c__Gammaproteobacteria;o__Enterobacterales;f__Erwiniaceae;g__Pantoea;s__Pantoea_sp._ARC607
PB2_gene_562	127	ORM86091.1	103	100/102(98.04)	6.90e-60	hypothetical protein HA42_01230 [Pantoea deleyi]	470932	d__Bacteria;k__norank;p__Proteobacteria;c__Gammaproteobacteria;o__Enterobacterales;f__Erwiniaceae;g__Pantoea;s__Pantoea_deleyi
PB2_gene_564	239	ORM86089.1	239	238/239(99.58)	2.07e-166	orotidine 5'-phosphate decarboxylase [Pantoea deleyi]	470932	d__Bacteria;k__norank;p__Proteobacteria;c__Gammaproteobacteria;o__Enterobacterales;f__Erwiniaceae;g__Pantoea;s__Pantoea_deleyi
PB2_gene_565	107	ORM86088.1	107	101/107(94.39)	6.50e-65	translation initiation factor Sui1 [Pantoea deleyi]	470932	d__Bacteria;k__norank;p__Proteobacteria;c__Gammaproteobacteria;o__Enterobacterales;f__Erwiniaceae;g__Pantoea;s__Pantoea_deleyi

PB2_gene_568	288	ORM86085.1	288	288/288(100.00)	3.24e-208	enoyl-CoA hydratase [Pantoea deleyi]	470932	d__Bacteria;k__norank;p__Proteobacteria;c__Gammaproteobacteria;o__Enterobacterales;f__Erwiniaceae;g__Pantoea;s__Pantoea_deleyi
PB2_gene_570	156	ORM81809.1	156	156/156(100.00)	1.11e-102	30S ribosomal protein S7 [Pantoea deleyi]	470932	d__Bacteria;k__norank;p__Proteobacteria;c__Gammaproteobacteria;o__Enterobacterales;f__Erwiniaceae;g__Pantoea;s__Pantoea_deleyi
PB2_gene_575	240	ORM53846.1	240	240/240(100.00)	6.66e-168	hypothetical protein HA41_07450 [Pantoea	472705	d__Bacteria;k__norank;p__Proteobacteria;c__Gammaproteobacteria;o__Enterobacterales;f__Erwiniaceae;g__Pantoea;s__Pantoea_conspicua
PB2_gene_580	601	ORM81818.1	601	596/601(99.17)	0	glutathione-regulated potassium-efflux system protein KefB [Pantoea deleyi]	470932	d__Bacteria;k__norank;p__Proteobacteria;c__Gammaproteobacteria;o__Enterobacterales;f__Erwiniaceae;g__Pantoea;s__Pantoea_deleyi
PB2_gene_587	457	ORM80359.1	457	452/457(98.91)	0	argininosuccinate lyase [Pantoea deleyi]	470932	d__Bacteria;k__norank;p__Proteobacteria;c__Gammaproteobacteria;o__Enterobacterales;f__Erwiniaceae;g__Pantoea;s__Pantoea_deleyi
PB2_gene_589	305	ORM80361.1	305	304/305(99.67)	6.09e-217	DNA-binding transcriptional regulator OxyR [Pantoea deleyi]	470932	d__Bacteria;k__norank;p__Proteobacteria;c__Gammaproteobacteria;o__Enterobacterales;f__Erwiniaceae;g__Pantoea;s__Pantoea_deleyi
PB2_gene_591	215	WP_111140431.1	215	215/215(100.00)	2.71e-141	HTH-type transcriptional repressor FabR [Pantoea sp.	53335	d__Bacteria;k__norank;p__Proteobacteria;c__Gammaproteobacteria;o__Enterobacterales;f__Erwiniaceae;g__Pantoea;s__norank
PB2_gene_592	122	KDA96509.1	123	119/122(97.54)	1.95e-80	membrane protein [Pantoea agglomerans Eh318]	1408177	d__Bacteria;k__norank;p__Proteobacteria;c__Gammaproteobacteria;o__Enterobacterales;f__Erwiniaceae;g__Pantoea;s__Pantoea_agglomerans
PB2_gene_596	266	WP_111141790.1	266	264/266(99.25)	6.63e-181	biotin-independent malonate decarboxylase subunit gamma [Pantoea sp. ARC607]	2027922	d__Bacteria;k__norank;p__Proteobacteria;c__Gammaproteobacteria;o__Enterobacterales;f__Erwiniaceae;g__Pantoea;s__Pantoea_sp._ARC607
PB2_gene_597	319	ORM75380.1	319	315/319(98.75)	1.77e-207	transporter [Pantoea deleyi]	470932	d__Bacteria;k__norank;p__Proteobacteria;c__Gammaproteobacteria;o__Enterobacterales;f__Erwiniaceae;g__Pantoea;s__Pantoea_deleyi
PB2_gene_605	492	WP_111139618.1	492	483/492(98.17)	0	phytoene desaturase [Pantoea sp. ARC607]	2027922	d__Bacteria;k__norank;p__Proteobacteria;c__Gammaproteobacteria;o__Enterobacterales;f__Erwiniaceae;g__Pantoea;s__Pantoea_sp._ARC607
PB2_gene_607	431	WP_111139620.1	431	406/431(94.20)	2.92e-290	glycosyltransferase [Pantoea sp. ARC607]	2027922	d__Bacteria;k__norank;p__Proteobacteria;c__Gammaproteobacteria;o__Enterobacterales;f__Erwiniaceae;g__Pantoea;s__Pantoea_sp._ARC607
PB2_gene_611	225	ORM76798.1	225	218/225(96.89)	1.91e-155	hypothetical protein HA42_18675 [Pantoea deleyi]	470932	d__Bacteria;k__norank;p__Proteobacteria;c__Gammaproteobacteria;o__Enterobacterales;f__Erwiniaceae;g__Pantoea;s__Pantoea_deleyi
PB2_gene_625	1041	WP_111140896.1	1043	988/1038(95.18)	0	beta-galactosidase [Pantoea sp. ARC607]	2027922	d__Bacteria;k__norank;p__Proteobacteria;c__Gammaproteobacteria;o__Enterobacterales;f__Erwiniaceae;g__Pantoea;s__Pantoea_sp._ARC607
PB2_gene_630	691	ORM81831.1	691	683/690(98.99)	0	hypothetical protein HA42_09690 [Pantoea deleyi]	470932	d__Bacteria;k__norank;p__Proteobacteria;c__Gammaproteobacteria;o__Enterobacterales;f__Erwiniaceae;g__Pantoea;s__Pantoea_deleyi
PB2_gene_635	190	ORM81834.1	190	187/190(98.42)	4.11e-126	peptidylprolyl isomerase A [Pantoea deleyi]	470932	d__Bacteria;k__norank;p__Proteobacteria;c__Gammaproteobacteria;o__Enterobacterales;f__Erwiniaceae;g__Pantoea;s__Pantoea_deleyi
PB2_gene_639	239	ORM81316.1	239	238/239(99.58)	1.35e-171	fatty acid metabolism regulator [Pantoea deleyi]	470932	d__Bacteria;k__norank;p__Proteobacteria;c__Gammaproteobacteria;o__Enterobacterales;f__Erwiniaceae;g__Pantoea;s__Pantoea_deleyi
PB2_gene_644	239	WP_111138879.1	239	237/239(99.16)	1.87e-153	septum site-determining protein MinC [Pantoea sp. ARC607]	2027922	d__Bacteria;k__norank;p__Proteobacteria;c__Gammaproteobacteria;o__Enterobacterales;f__Erwiniaceae;g__Pantoea;s__Pantoea_sp._ARC607
PB2_gene_649	568	WP_111138875.1	568	559/568(98.42)	0	long-chain-fatty-acid--CoA ligase FadD [Pantoea sp. ARC607]	None	Unclassified
PB2_gene_650	198	ORM79288.1	198	197/198(99.49)	6.57e-145	hypothetical protein HA42_15730 [Pantoea deleyi]	470932	d__Bacteria;k__norank;p__Proteobacteria;c__Gammaproteobacteria;o__Enterobacterales;f__Erwiniaceae;g__Pantoea;s__Pantoea_deleyi
PB2_gene_652	373	WP_111140697.1	373	369/373(98.93)	4.83e-271	Gfo/Idh/MocA family oxidoreductase [Pantoea sp. ARC607]	2027922	d__Bacteria;k__norank;p__Proteobacteria;c__Gammaproteobacteria;o__Enterobacterales;f__Erwiniaceae;g__Pantoea;s__Pantoea_sp._ARC607
PB2_gene_654	460	WP_124890098.1	460	444/460(96.52)	0	FAD-binding protein [Pantoea agglomerans]	549	d__Bacteria;k__norank;p__Proteobacteria;c__Gammaproteobacteria;o__Enterobacterales;f__Erwiniaceae;g__Pantoea;s__Pantoea_agglomerans
PB2_gene_656	179	WP_111140693.1	179	177/179(98.88)	3.14e-111	DUF2058 domain-containing protein [Pantoea sp. ARC607]	2027922	d__Bacteria;k__norank;p__Proteobacteria;c__Gammaproteobacteria;o__Enterobacterales;f__Erwiniaceae;g__Pantoea;s__Pantoea_sp._ARC607
PB2_gene_657	243	ORM84463.1	232	215/232(92.67)	2.95e-154	RNA pseudouridine synthase [Pantoea deleyi]	470932	d__Bacteria;k__norank;p__Proteobacteria;c__Gammaproteobacteria;o__Enterobacterales;f__Erwiniaceae;g__Pantoea;s__Pantoea_deleyi

PB2_gene_659	640	ORM84461.1	641	568/641(88.61)	0	methyl-accepting chemotaxis protein [Pantoea deleyi]	470932	d__Bacteria;k__norank;p__Proteobacteria;c__Gammaproteobacteria;o__Enterobacterales;f__Erwiniaceae;g__Pantoea;s__Pantoea_deleyi
PB2_gene_663	526	ERM08663.1	526	501/526(95.25)	0	ABC transporter [Pantoea agglomerans Tx10]	1332072	d__Bacteria;k__norank;p__Proteobacteria;c__Gammaproteobacteria;o__Enterobacterales;f__Erwiniaceae;g__Pantoea;s__Pantoea_agglomerans
PB2_gene_664	485	ORM80421.1	485	479/485(98.76)	0	cytosol nonspecific dipeptidase [Pantoea deleyi]	470932	d__Bacteria;k__norank;p__Proteobacteria;c__Gammaproteobacteria;o__Enterobacterales;f__Erwiniaceae;g__Pantoea;s__Pantoea_deleyi
PB2_gene_666	415	ORM80419.1	415	411/415(99.04)	1.05e-309	fermentation/respiration switch protein [Pantoea deleyi]	470932	d__Bacteria;k__norank;p__Proteobacteria;c__Gammaproteobacteria;o__Enterobacterales;f__Erwiniaceae;g__Pantoea;s__Pantoea_deleyi
PB2_gene_669	340	ELP23429.1	340	335/340(98.53)	2.01e-235	Glutamate 5-kinase [Pantoea agglomerans 299R]	1261128	d__Bacteria;k__norank;p__Proteobacteria;c__Gammaproteobacteria;o__Enterobacterales;f__Erwiniaceae;g__Pantoea;s__Pantoea_agglomerans
PB2_gene_671	220	ORM77534.1	199	194/199(97.49)	5.36e-141	GNAT family N-acetyltransferase [Pantoea deleyi]	470932	d__Bacteria;k__norank;p__Proteobacteria;c__Gammaproteobacteria;o__Enterobacterales;f__Erwiniaceae;g__Pantoea;s__Pantoea_deleyi
PB2_gene_672	363	WP_111141331.1	363	354/363(97.52)	1.24e-252	ABC transporter ATP-binding protein [Pantoea sp. ARC607]	2027922	d__Bacteria;k__norank;p__Proteobacteria;c__Gammaproteobacteria;o__Enterobacterales;f__Erwiniaceae;g__Pantoea;s__Pantoea_sp._ARC607
PB2_gene_682	955	WP_010402443.1	968	890/960(92.71)	0	TrbL/VirB6 plasmid conjugal transfer family protein [Wolbachia endosymbiont of Nasonia vitripennis]	180837	d__Bacteria;k__norank;p__Proteobacteria;c__Alphaproteobacteria;o__Rickettsiales;f__Anaplasmataceae;g__Wolbachia;s__Wolbachia_endosymbiont_of_Nasonia_vitripennis
PB2_gene_690	356	ORM81314.1	356	350/356(98.31)	1.35e-257	alanine racemase [Pantoea deleyi]	470932	d__Bacteria;k__norank;p__Proteobacteria;c__Gammaproteobacteria;o__Enterobacterales;f__Erwiniaceae;g__Pantoea;s__Pantoea_deleyi
PB2_gene_691	397	ORM81313.1	397	392/397(98.74)	1.74e-267	Bcr/CflA family drug resistance efflux transporter [Pantoea	470932	d__Bacteria;k__norank;p__Proteobacteria;c__Gammaproteobacteria;o__Enterobacterales;f__Erwiniaceae;g__Pantoea;s__Pantoea_deleyi
PB2_gene_694	603	ADO09746.1	619	589/603(97.68)	0	Putative K+:H+ antiporter khaP2 [Pantoea vagans C9-1]	712898	d__Bacteria;k__norank;p__Proteobacteria;c__Gammaproteobacteria;o__Enterobacterales;f__Erwiniaceae;g__Pantoea;s__Pantoea_vagans
PB2_gene_700	290	ORM83398.1	321	290/290(100.00)	5.67e-206	tRNA dihydrouridine synthase DusB [Pantoea deleyi]	470932	d__Bacteria;k__norank;p__Proteobacteria;c__Gammaproteobacteria;o__Enterobacterales;f__Erwiniaceae;g__Pantoea;s__Pantoea_deleyi
PB2_gene_702	293	ORM83388.1	293	291/293(99.32)	6.58e-215	ribosomal protein L11 methyltransferase [Pantoea	470932	d__Bacteria;k__norank;p__Proteobacteria;c__Gammaproteobacteria;o__Enterobacterales;f__Erwiniaceae;g__Pantoea;s__Pantoea_deleyi
PB2_gene_703	481	ORM83387.1	481	481/481(100.00)	0	sodium/panthothenate symporter [Pantoea deleyi]	470932	d__Bacteria;k__norank;p__Proteobacteria;c__Gammaproteobacteria;o__Enterobacterales;f__Erwiniaceae;g__Pantoea;s__Pantoea_deleyi
PB2_gene_706	300	ORM78255.1	300	289/300(96.33)	8.81e-203	LysR family transcriptional regulator [Pantoea deleyi]	470932	d__Bacteria;k__norank;p__Proteobacteria;c__Gammaproteobacteria;o__Enterobacterales;f__Erwiniaceae;g__Pantoea;s__Pantoea_deleyi
PB2_gene_708	458	ORM78253.1	458	457/458(99.78)	0	murein transglycosylase D [Pantoea deleyi]	470932	d__Bacteria;k__norank;p__Proteobacteria;c__Gammaproteobacteria;o__Enterobacterales;f__Erwiniaceae;g__Pantoea;s__Pantoea_deleyi
PB2_gene_710	245	ORM78252.1	245	244/245(99.59)	1.78e-179	hypothetical protein HA42_16915 [Pantoea deleyi]	470932	d__Bacteria;k__norank;p__Proteobacteria;c__Gammaproteobacteria;o__Enterobacterales;f__Erwiniaceae;g__Pantoea;s__Pantoea_deleyi
PB2_gene_723	644	WP_111141308.1	644	640/644(99.38)	0	PTS mannitol transporter subunit IICBA [Pantoea sp. ARC607]	2027922	d__Bacteria;k__norank;p__Proteobacteria;c__Gammaproteobacteria;o__Enterobacterales;f__Erwiniaceae;g__Pantoea;s__Pantoea_sp._ARC607
PB2_gene_724	231	ORM80632.1	239	230/231(99.57)	1.36e-153	hypothetical protein HA42_13725 [Pantoea deleyi]	470932	d__Bacteria;k__norank;p__Proteobacteria;c__Gammaproteobacteria;o__Enterobacterales;f__Erwiniaceae;g__Pantoea;s__Pantoea_deleyi
PB2_gene_731	346	ORM80763.1	353	330/353(93.48)	1.69e-234	tRNA pseudouridine(13) synthase TruD [Pantoea deleyi]	470932	d__Bacteria;k__norank;p__Proteobacteria;c__Gammaproteobacteria;o__Enterobacterales;f__Erwiniaceae;g__Pantoea;s__Pantoea_deleyi
PB2_gene_734	114	ADD78216.1	114	108/114(94.74)	1.73e-70	FtsB [Pantoea ananatis LMG 20103]	706191	d__Bacteria;k__norank;p__Proteobacteria;c__Gammaproteobacteria;o__Enterobacterales;f__Erwiniaceae;g__Pantoea;s__Pantoea_ananatis
PB2_gene_737	475	WP_111141639.1	475	472/475(99.37)	0	sulfate adenylyltransferase subunit CysN [Pantoea sp.	2027922	d__Bacteria;k__norank;p__Proteobacteria;c__Gammaproteobacteria;o__Enterobacterales;f__Erwiniaceae;g__Pantoea;s__Pantoea_sp._ARC607
PB2_gene_741	560	ORM86843.1	560	558/560(99.64)	0	choline dehydrogenase [Pantoea deleyi]	470932	d__Bacteria;k__norank;p__Proteobacteria;c__Gammaproteobacteria;o__Enterobacterales;f__Erwiniaceae;g__Pantoea;s__Pantoea_deleyi
PB2_gene_742	490	WP_111138841.1	490	485/490(98.98)	0	betaine-aldehyde dehydrogenase [Pantoea sp. ARC607]	2027922	d__Bacteria;k__norank;p__Proteobacteria;c__Gammaproteobacteria;o__Enterobacterales;f__Erwiniaceae;g__Pantoea;s__Pantoea_sp._ARC607

PB2_gene_743	192	ORM86845.1	197	190/192(98.96)	5.09e-129	transcriptional regulator BetI [Pantoea deleyi]	470932	d__Bacteria;k__norank;p__Proteobacteria;c__Gammaproteobacteria;o__Enterobacterales;f__Erwiniaceae;g__Pantoea;s__Pantoea_deleyi
PB2_gene_744	676	ORM86846.1	676	674/676(99.70)	0	high-affinity choline transporter BetT [Pantoea deleyi]	470932	d__Bacteria;k__norank;p__Proteobacteria;c__Gammaproteobacteria;o__Enterobacterales;f__Erwiniaceae;g__Pantoea;s__Pantoea_deleyi
PB2_gene_748	576	WP_090966535.1	576	537/576(93.23)	0	ShlB/FhaC/HecB family hemolysin secretion/activation protein [Pantoea sp. OV426]	1881036	d__Bacteria;k__norank;p__Proteobacteria;c__Gammaproteobacteria;o__Enterobacterales;f__Erwiniaceae;g__Pantoea;s__Pantoea_sp._OV426
PB2_gene_749	689	ORM80434.1	689	684/689(99.27)	0	glycine--tRNA ligase subunit beta [Pantoea deleyi]	470932	d__Bacteria;k__norank;p__Proteobacteria;c__Gammaproteobacteria;o__Enterobacterales;f__Erwiniaceae;g__Pantoea;s__Pantoea_deleyi
PB2_gene_761	219	ORM80974.1	219	217/219(99.09)	1.16e-153	DNA-binding response regulator [Pantoea deleyi]	470932	d__Bacteria;k__norank;p__Proteobacteria;c__Gammaproteobacteria;o__Enterobacterales;f__Erwiniaceae;g__Pantoea;s__Pantoea_deleyi
PB2_gene_762	451	ORM80975.1	451	444/451(98.45)	0	two-component sensor histidine kinase [Pantoea deleyi]	470932	d__Bacteria;k__norank;p__Proteobacteria;c__Gammaproteobacteria;o__Enterobacterales;f__Erwiniaceae;g__Pantoea;s__Pantoea_deleyi
PB2_gene_766	437	WP_098051751.1	437	415/437(94.97)	6.05e-302	amidohydrolase [Pantoea agglomerans]	None	Unclassified
PB2_gene_772	413	PXW19464.1	424	409/413(99.03)	1.47e-304	alanine-synthesizing transaminase [Pantoea sp. JKS000250]	1938795	d__Bacteria;k__norank;p__Proteobacteria;c__Gammaproteobacteria;o__Enterobacterales;f__Erwiniaceae;g__Pantoea;s__Pantoea_sp._JKS000250
PB2_gene_773	199	ORM83865.1	199	198/199(99.50)	2.46e-136	5'-deoxynucleotidase [Pantoea deleyi]	470932	d__Bacteria;k__norank;p__Proteobacteria;c__Gammaproteobacteria;o__Enterobacterales;f__Erwiniaceae;g__Pantoea;s__Pantoea_deleyi
PB2_gene_776	138	ORM83867.1	150	135/138(97.83)	3.82e-97	hypothetical protein HA42_05605 [Pantoea deleyi]	470932	d__Bacteria;k__norank;p__Proteobacteria;c__Gammaproteobacteria;o__Enterobacterales;f__Erwiniaceae;g__Pantoea;s__Pantoea_deleyi
PB2_gene_786	416	WP_111139459.1	416	413/416(99.28)	1.83e-290	lipid III flippase WzxE [Pantoea sp. ARC607]	2027922	d__Bacteria;k__norank;p__Proteobacteria;c__Gammaproteobacteria;o__Enterobacterales;f__Erwiniaceae;g__Pantoea;s__Pantoea_sp._ARC607
PB2_gene_790	419	WP_111139456.1	419	411/419(98.09)	9.99e-301	UDP-N-acetyl-D-mannosamine dehydrogenase [Pantoea sp. ARC607]	2027922	d__Bacteria;k__norank;p__Proteobacteria;c__Gammaproteobacteria;o__Enterobacterales;f__Erwiniaceae;g__Pantoea;s__Pantoea_sp._ARC607
PB2_gene_796	497	ORM78627.1	497	492/497(98.99)	0	peptidase [Pantoea deleyi]	470932	d__Bacteria;k__norank;p__Proteobacteria;c__Gammaproteobacteria;o__Enterobacterales;f__Erwiniaceae;g__Pantoea;s__Pantoea_deleyi
PB2_gene_798	657	WP_111139443.1	668	652/657(99.24)	0	biofilm formation regulator HmsP [Pantoea sp. ARC607]	2027922	d__Bacteria;k__norank;p__Proteobacteria;c__Gammaproteobacteria;o__Enterobacterales;f__Erwiniaceae;g__Pantoea;s__Pantoea_sp._ARC607
PB2_gene_801	251	WP_111138634.1	251	251/251(100.00)	3.82e-168	flagellar basal body rod protein FlgF [Pantoea sp. ARC607]	53335	d__Bacteria;k__norank;p__Proteobacteria;c__Gammaproteobacteria;o__Enterobacterales;f__Erwiniaceae;g__Pantoea;s__norank
PB2_gene_804	369	ORM82817.1	369	367/369(99.46)	6.48e-242	flagellar biosynthesis protein FlgA [Pantoea deleyi]	470932	d__Bacteria;k__norank;p__Proteobacteria;c__Gammaproteobacteria;o__Enterobacterales;f__Erwiniaceae;g__Pantoea;s__Pantoea_deleyi
PB2_gene_806	541	ORM82819.1	549	532/541(98.34)	0	flagellar hook-associated protein FlgK [Pantoea deleyi]	470932	d__Bacteria;k__norank;p__Proteobacteria;c__Gammaproteobacteria;o__Enterobacterales;f__Erwiniaceae;g__Pantoea;s__Pantoea_deleyi
PB2_gene_811	134	WP_111139343.1	90	86/90(95.56)	4.91e-50	DUF1471 domain-containing protein [Pantoea sp. ARC607]	2027922	d__Bacteria;k__norank;p__Proteobacteria;c__Gammaproteobacteria;o__Enterobacterales;f__Erwiniaceae;g__Pantoea;s__Pantoea_sp._ARC607
PB2_gene_812	562	ORM78498.1	562	546/561(97.33)	0	two-component system sensor histidine kinase NarQ [Pantoea deleyi]	470932	d__Bacteria;k__norank;p__Proteobacteria;c__Gammaproteobacteria;o__Enterobacterales;f__Erwiniaceae;g__Pantoea;s__Pantoea_deleyi
PB2_gene_814	1038	ORM78496.1	1038	1033/1038(99.52)	0	aminoglycoside/multidrug transporter subunit AcrD [Pantoea deleyi]	470932	d__Bacteria;k__norank;p__Proteobacteria;c__Gammaproteobacteria;o__Enterobacterales;f__Erwiniaceae;g__Pantoea;s__Pantoea_deleyi
PB2_gene_815	144	ELP26584.1	144	119/144(82.64)	4.27e-83	a glutathione-dependent thiol reductase [Pantoea agglomerans 299R]	1261128	d__Bacteria;k__norank;p__Proteobacteria;c__Gammaproteobacteria;o__Enterobacterales;f__Erwiniaceae;g__Pantoea;s__Pantoea_agglomerans
PB2_gene_823	142	CCE77815.1	144	142/142(100.00)	3.82e-99	conserved hypothetical protein [Wolbachia pipientis wAlbB]	1116230	d__Bacteria;k__norank;p__Proteobacteria;c__Alphaproteobacteria;o__Rickettsiales;f__Anaplasmataceae;g__Wolbachia;s__Wolbachia_pipientis
PB2_gene_831	291	WP_111138828.1	291	266/289(92.04)	5.76e-183	copper homeostasis membrane protein CopD [Pantoea sp.	2027922	d__Bacteria;k__norank;p__Proteobacteria;c__Gammaproteobacteria;o__Enterobacterales;f__Erwiniaceae;g__Pantoea;s__Pantoea_sp._ARC607

PB2_gene_832	125	ORM86829.1	125	115/125(92.00)	6.73e-71	hypothetical protein HA42_00170 [Pantoea deleyi]	470932	d__Bacteria;k__norank;p__Proteobacteria;c__Gammaproteobacteria;o__Enterobacterales;f__Erwiniaceae;g__Pantoea;s__Pantoea_deleyi
PB2_gene_834	223	ORM86827.1	222	220/222(99.10)	2.85e-158	exodeoxyribonuclease X [Pantoea deleyi]	470932	d__Bacteria;k__norank;p__Proteobacteria;c__Gammaproteobacteria;o__Enterobacterales;f__Erwiniaceae;g__Pantoea;s__Pantoea_deleyi
PB2_gene_835	687	ORM86826.1	687	673/687(97.96)	0	oligopeptidase B [Pantoea deleyi]	470932	d__Bacteria;k__norank;p__Proteobacteria;c__Gammaproteobacteria;o__Enterobacterales;f__Erwiniaceae;g__Pantoea;s__Pantoea_deleyi
PB2_gene_840	748	ORM84467.1	739	728/739(98.51)	0	catalase/peroxidase HPI [Pantoea deleyi]	470932	d__Bacteria;k__norank;p__Proteobacteria;c__Gammaproteobacteria;o__Enterobacterales;f__Erwiniaceae;g__Pantoea;s__Pantoea_deleyi
PB2_gene_844	484	SJZ61302.1	483	466/483(96.48)	0	Uncharacterized conserved protein, circularly permuted ATPgrasp superfamily [Pantoea	470933	d__Bacteria;k__norank;p__Proteobacteria;c__Gammaproteobacteria;o__Enterobacterales;f__Erwiniaceae;g__Pantoea;s__Pantoea_eucalypti
PB2_gene_855	231	WP_111141831.1	249	218/231(94.37)	2.15e-166	MipA/OmpV family protein [Pantoea sp. ARC607]	2027922	d__Bacteria;k__norank;p__Proteobacteria;c__Gammaproteobacteria;o__Enterobacterales;f__Erwiniaceae;g__Pantoea;s__Pantoea_sp._ARC607
PB2_gene_860	93	ORM81304.1	93	92/93(98.92)	3.08e-61	hypothetical protein HA42_11175 [Pantoea deleyi]	470932	d__Bacteria;k__norank;p__Proteobacteria;c__Gammaproteobacteria;o__Enterobacterales;f__Erwiniaceae;g__Pantoea;s__Pantoea_deleyi
PB2_gene_864	169	ORM85307.1	169	167/169(98.82)	3.24e-124	phenolic acid decarboxylase [Pantoea deleyi]	470932	d__Bacteria;k__norank;p__Proteobacteria;c__Gammaproteobacteria;o__Enterobacterales;f__Erwiniaceae;g__Pantoea;s__Pantoea_deleyi
PB2_gene_865	299	ORM85306.1	301	289/293(98.63)	3.20e-205	LysR family transcriptional regulator [Pantoea deleyi]	470932	d__Bacteria;k__norank;p__Proteobacteria;c__Gammaproteobacteria;o__Enterobacterales;f__Erwiniaceae;g__Pantoea;s__Pantoea_deleyi
PB2_gene_868	213	WP_095706191.1	211	174/212(82.08)	4.04e-121	hypothetical protein [Pantoea vagans]	470934	d__Bacteria;k__norank;p__Proteobacteria;c__Gammaproteobacteria;o__Enterobacterales;f__Erwiniaceae;g__Pantoea;s__Pantoea_vagans
PB2_gene_870	475	ERM08454.1	475	474/475(99.79)	0	dihydrolipoamide dehydrogenase [Pantoea agglomerans Tx10]	1332072	d__Bacteria;k__norank;p__Proteobacteria;c__Gammaproteobacteria;o__Enterobacterales;f__Erwiniaceae;g__Pantoea;s__Pantoea_agglomerans
PB2_gene_874	327	SKA14749.1	327	326/327(99.69)	1.89e-222	cell division transport system permease protein [Pantoea agglomerans]	470933	d__Bacteria;k__norank;p__Proteobacteria;c__Gammaproteobacteria;o__Enterobacterales;f__Erwiniaceae;g__Pantoea;s__Pantoea_eucalypti
PB2_gene_875	222	ORM80411.1	222	222/222(100.00)	1.85e-156	cell division ATP-binding protein FtsE [Pantoea deleyi]	470932	d__Bacteria;k__norank;p__Proteobacteria;c__Gammaproteobacteria;o__Enterobacterales;f__Erwiniaceae;g__Pantoea;s__Pantoea_deleyi
PB2_gene_876	509	WP_111140409.1	537	489/537(91.06)	2.46e-310	signal recognition particle-docking protein FtsY [Pantoea sp.	2027922	d__Bacteria;k__norank;p__Proteobacteria;c__Gammaproteobacteria;o__Enterobacterales;f__Erwiniaceae;g__Pantoea;s__Pantoea_sp._ARC607
PB2_gene_883	60	WP_111141895.1	761	57/60(95.00)	2.29e-25	zinc/cadmium/mercury/lead-transporting ATPase [Pantoea sp. ARC607]	2027922	d__Bacteria;k__norank;p__Proteobacteria;c__Gammaproteobacteria;o__Enterobacterales;f__Erwiniaceae;g__Pantoea;s__Pantoea_sp._ARC607
PB2_gene_885	492	ORM85282.1	491	488/491(99.39)	0	UDP-N-acetylmuramate--L-alanine ligase [Pantoea deleyi]	470932	d__Bacteria;k__norank;p__Proteobacteria;c__Gammaproteobacteria;o__Enterobacterales;f__Erwiniaceae;g__Pantoea;s__Pantoea_deleyi
PB2_gene_890	305	ORM85286.1	305	305/305(100.00)	9.02e-219	UDP-3-O-[3-hydroxymyristoyl] N-acetylglucosamine deacetylase [Pantoea deleyi]	470932	d__Bacteria;k__norank;p__Proteobacteria;c__Gammaproteobacteria;o__Enterobacterales;f__Erwiniaceae;g__Pantoea;s__Pantoea_deleyi
PB2_gene_896	275	PVY82187.1	275	254/275(92.36)	3.64e-178	nucleotide-binding universal stress UspA family protein [Pantoea ananatis]	553	d__Bacteria;k__norank;p__Proteobacteria;c__Gammaproteobacteria;o__Enterobacterales;f__Erwiniaceae;g__Pantoea;s__Pantoea_ananatis
PB2_gene_897	492	WP_109653980.1	492	482/492(97.97)	0	MULTISPECIES: SulP family inorganic anion transporter [Pantoea]	549	d__Bacteria;k__norank;p__Proteobacteria;c__Gammaproteobacteria;o__Enterobacterales;f__Erwiniaceae;g__Pantoea;s__Pantoea_agglomerans
PB2_gene_899	427	WP_024553948.1	427	409/426(96.01)	2.09e-281	arsenic transporter [Franconibacter helveticus]	357240	d__Bacteria;k__norank;p__Proteobacteria;c__Gammaproteobacteria;o__Enterobacterales;f__Enterobacteriaceae;g__Franconibacter;s__Franconibacter_helveticus
PB2_gene_903	208	WP_090963827.1	194	166/194(85.57)	1.29e-98	type 1 fimbrial protein [Pantoea sp. OV426]	1881036	d__Bacteria;k__norank;p__Proteobacteria;c__Gammaproteobacteria;o__Enterobacterales;f__Erwiniaceae;g__Pantoea;s__Pantoea_sp._OV426
PB2_gene_905	862	WP_128084579.1	862	759/862(88.05)	0	fimbrial biogenesis outer membrane usher protein [Pantoea	470932	d__Bacteria;k__norank;p__Proteobacteria;c__Gammaproteobacteria;o__Enterobacterales;f__Erwiniaceae;g__Pantoea;s__Pantoea_deleyi

PB2_gene_907	337	WP_069025665.1	338	269/337(79.82)	1.54e-185	fimbrial protein [Pantoea agglomerans]	549	d__Bacteria;k__norank;p__Proteobacteria;c__Gammaproteobacteria;o__Enterobacterales;f__Erwiniaceae;g__Pantoea;s__Pantoea_agglomerans
PB2_gene_908	287	ORM84331.1	287	283/287(98.61)	1.96e-213	glutathione-dependent disulfide-bond oxidoreductase [Pantoea deleyi]	470932	d__Bacteria;k__norank;p__Proteobacteria;c__Gammaproteobacteria;o__Enterobacterales;f__Erwiniaceae;g__Pantoea;s__Pantoea_deleyi
PB2_gene_909	97	ORM84332.1	97	96/97(98.97)	2.48e-62	hypothetical protein HA42_04925 [Pantoea deleyi]	470932	d__Bacteria;k__norank;p__Proteobacteria;c__Gammaproteobacteria;o__Enterobacterales;f__Erwiniaceae;g__Pantoea;s__Pantoea_deleyi
PB2_gene_910	364	ORM84333.1	364	336/364(92.31)	1.41e-238	hypothetical protein HA42_04930 [Pantoea deleyi]	470932	d__Bacteria;k__norank;p__Proteobacteria;c__Gammaproteobacteria;o__Enterobacterales;f__Erwiniaceae;g__Pantoea;s__Pantoea_deleyi
PB2_gene_915	313	ORM81461.1	313	310/313(99.04)	1.83e-222	hydroxymethylbilane synthase [Pantoea deleyi]	470932	d__Bacteria;k__norank;p__Proteobacteria;c__Gammaproteobacteria;o__Enterobacterales;f__Erwiniaceae;g__Pantoea;s__Pantoea_deleyi
PB2_gene_916	242	WP_111142000.1	242	241/242(99.59)	5.09e-173	uroporphyrinogen-III synthase [Pantoea sp. ARC607]	2027922	d__Bacteria;k__norank;p__Proteobacteria;c__Gammaproteobacteria;o__Enterobacterales;f__Erwiniaceae;g__Pantoea;s__Pantoea_sp._ARC607
PB2_gene_917	377	WP_111141999.1	377	376/377(99.73)	9.46e-255	uroporphyrinogen-III C-methyltransferase [Pantoea sp. ARC607]	2027922	d__Bacteria;k__norank;p__Proteobacteria;c__Gammaproteobacteria;o__Enterobacterales;f__Erwiniaceae;g__Pantoea;s__Pantoea_sp._ARC607
PB2_gene_918	398	ORM81446.1	398	395/397(99.50)	1.16e-277	protoheme IX biogenesis protein HemY [Pantoea deleyi]	470932	d__Bacteria;k__norank;p__Proteobacteria;c__Gammaproteobacteria;o__Enterobacterales;f__Erwiniaceae;g__Pantoea;s__Pantoea_deleyi
PB2_gene_920	1076	EXU76823.1	1072	845/1071(78.90)	0	aldehyde oxidase [Erwinia mallotivora]	69222	d__Bacteria;k__norank;p__Proteobacteria;c__Gammaproteobacteria;o__Enterobacterales;f__Erwiniaceae;g__Erwinia;s__Erwinia_mallotivora
PB2_gene_921	645	EXU76822.1	645	454/642(70.72)	0	(Fe-S)-binding protein [Erwinia mallotivora]	69222	d__Bacteria;k__norank;p__Proteobacteria;c__Gammaproteobacteria;o__Enterobacterales;f__Erwiniaceae;g__Erwinia;s__Erwinia_mallotivora
PB2_gene_922	176	WP_125289954.1	175	102/176(57.95)	2.85e-59	4Fe-4S dicluster domain-containing protein [Erwinia sp.	2022746	d__Bacteria;k__norank;p__Proteobacteria;c__Gammaproteobacteria;o__Enterobacterales;f__Erwiniaceae;g__Erwinia;s__Erwinia_sp._198
PB2_gene_923	716	EXU76820.1	716	606/716(84.64)	0	formate dehydrogenase [Erwinia mallotivora]	69222	d__Bacteria;k__norank;p__Proteobacteria;c__Gammaproteobacteria;o__Enterobacterales;f__Erwiniaceae;g__Erwinia;s__Erwinia_mallotivora
PB2_gene_926	1124	WP_077064459.1	1121	1003/1108(90.52)	0	type VI secretion protein VasK [Enterobacter chengduensis]	2494701	d__Bacteria;k__norank;p__Proteobacteria;c__Gammaproteobacteria;o__Enterobacterales;f__Enterobacteriaceae;g__Enterobacter;s__Enterobacter_chengduensis
PB2_gene_927	539	WP_115396274.1	539	516/539(95.73)	0	type VI secretion system protein TssA [Salmonella enterica]	28901	d__Bacteria;k__norank;p__Proteobacteria;c__Gammaproteobacteria;o__Enterobacterales;f__Enterobacteriaceae;g__Salmonella;s__Salmonella_enterica
PB2_gene_928	589	WP_101700232.1	589	576/589(97.79)	0	type VI secretion system baseplate subunit TssF	1981983	d__Bacteria;k__norank;p__Proteobacteria;c__Gammaproteobacteria;o__Enterobacterales;f__Enterobacteriaceae;g__Citrobacter;s__Citrobacter_sp._L55
PB2_gene_931	295	WP_111139082.1	275	275/275(100.00)	1.82e-190	kinase/pyrophosphorylase [Pantoea sp. ARC607]	2027922	d__Bacteria;k__norank;p__Proteobacteria;c__Gammaproteobacteria;o__Enterobacterales;f__Erwiniaceae;g__Pantoea;s__Pantoea_sp._ARC607
PB2_gene_934	248	ORM80797.1	248	239/248(96.37)	1.18e-165	diguanylate phosphodiesterase [Pantoea deleyi]	470932	d__Bacteria;k__norank;p__Proteobacteria;c__Gammaproteobacteria;o__Enterobacterales;f__Erwiniaceae;g__Pantoea;s__Pantoea_deleyi
PB2_gene_937	181	WP_111139088.1	181	175/181(96.69)	1.77e-127	glutathione peroxidase [Pantoea sp. ARC607]	2027922	d__Bacteria;k__norank;p__Proteobacteria;c__Gammaproteobacteria;o__Enterobacterales;f__Erwiniaceae;g__Pantoea;s__Pantoea_sp._ARC607
PB2_gene_941	681	WP_124890080.1	681	642/681(94.27)	0	hydantoinase/oxoprolinase family protein [Pantoea agglomerans]	549	d__Bacteria;k__norank;p__Proteobacteria;c__Gammaproteobacteria;o__Enterobacterales;f__Erwiniaceae;g__Pantoea;s__Pantoea_agglomerans
PB2_gene_942	654	WP_095706967.1	652	613/648(94.60)	0	hydantoinase B/oxoprolinase family protein [Pantoea vagans]	470934	d__Bacteria;k__norank;p__Proteobacteria;c__Gammaproteobacteria;o__Enterobacterales;f__Erwiniaceae;g__Pantoea;s__Pantoea_vagans
PB2_gene_943	332	QAV45094.1	189	151/189(79.89)	3.45e-105	hypothetical protein D1629_10895 [Pantoea	549	d__Bacteria;k__norank;p__Proteobacteria;c__Gammaproteobacteria;o__Enterobacterales;f__Erwiniaceae;g__Pantoea;s__Pantoea_agglomerans
PB2_gene_944	478	WP_111141380.1	478	455/478(95.19)	0	amidase [Pantoea sp. ARC607]	2027922	d__Bacteria;k__norank;p__Proteobacteria;c__Gammaproteobacteria;o__Enterobacterales;f__Erwiniaceae;g__Pantoea;s__Pantoea_sp._ARC607
PB2_gene_948	399	ORM85293.1	399	363/399(90.98)	1.59e-248	type IV pilin biogenesis protein [Pantoea deleyi]	470932	d__Bacteria;k__norank;p__Proteobacteria;c__Gammaproteobacteria;o__Enterobacterales;f__Erwiniaceae;g__Pantoea;s__Pantoea_deleyi
PB2_gene_950	202	WP_111139418.1	202	195/202(96.53)	3.05e-127	dephospho-CoA kinase [Pantoea sp. ARC607]	2027922	d__Bacteria;k__norank;p__Proteobacteria;c__Gammaproteobacteria;o__Enterobacterales;f__Erwiniaceae;g__Pantoea;s__Pantoea_sp._ARC607

PB2_gene_954	900	WP_111139414.1	900	895/900(99.44)	0	preprotein translocase subunit SecA [Pantoea sp. ARC607]	2027922	d__Bacteria;k__norank;p__Proteobacteria;c__Gammaproteobacteria;o__Enterobacterales;f__Erwiniaceae;g__Pantoea;s__Pantoea_sp._ARC607
PB2_gene_956	237	ORM82063.1	237	226/237(95.36)	2.15e-167	hypothetical protein HA42_09170 [Pantoea deleyi]	470932	d__Bacteria;k__norank;p__Proteobacteria;c__Gammaproteobacteria;o__Enterobacterales;f__Erwiniaceae;g__Pantoea;s__Pantoea_deleyi
PB2_gene_960	228	ORM82067.1	228	221/228(96.93)	9.03e-151	GntR family transcriptional regulator [Pantoea deleyi]	470932	d__Bacteria;k__norank;p__Proteobacteria;c__Gammaproteobacteria;o__Enterobacterales;f__Erwiniaceae;g__Pantoea;s__Pantoea_deleyi
PB2_gene_961	409	ORM82068.1	501	382/407(93.86)	7.65e-279	fructuronate reductase [Pantoea deleyi]	470932	d__Bacteria;k__norank;p__Proteobacteria;c__Gammaproteobacteria;o__Enterobacterales;f__Erwiniaceae;g__Pantoea;s__Pantoea_deleyi
PB2_gene_962	2243	WP_082861179.1	2839	2221/2242(99.06)	0	DNA-directed RNA polymerase subunit beta/beta' [Wolbachia endosymbiont of Dactylopius coccus]	None	Unclassified
PB2_gene_969	351	WP_111138420.1	351	346/351(98.58)	4.11e-245	lipid kinase [Pantoea sp. ARC607]	2027922	d__Bacteria;k__norank;p__Proteobacteria;c__Gammaproteobacteria;o__Enterobacterales;f__Erwiniaceae;g__Pantoea;s__Pantoea_sp._ARC607
PB2_gene_970	349	ORM76228.1	349	335/349(95.99)	1.43e-244	hypothetical protein HA42_19360 [Pantoea deleyi]	470932	d__Bacteria;k__norank;p__Proteobacteria;c__Gammaproteobacteria;o__Enterobacterales;f__Erwiniaceae;g__Pantoea;s__Pantoea_deleyi
PB2_gene_971	232	WP_111138418.1	236	214/230(93.04)	2.98e-152	molybdenum ABC transporter substrate-binding protein [Pantoea sp. ARC607]	2027922	d__Bacteria;k__norank;p__Proteobacteria;c__Gammaproteobacteria;o__Enterobacterales;f__Erwiniaceae;g__Pantoea;s__Pantoea_sp._ARC607
PB2_gene_980	166	WP_111141053.1	166	162/166(97.59)	3.78e-108	YbaK/prolyl-tRNA synthetase associated domain-containing protein [Pantoea sp. ARC607]	2027922	d__Bacteria;k__norank;p__Proteobacteria;c__Gammaproteobacteria;o__Enterobacterales;f__Erwiniaceae;g__Pantoea;s__Pantoea_sp._ARC607
PB2_gene_981	266	WP_111141055.1	266	258/266(96.99)	8.17e-191	hypothetical protein [Pantoea sp. ARC607]	2027922	d__Bacteria;k__norank;p__Proteobacteria;c__Gammaproteobacteria;o__Enterobacterales;f__Erwiniaceae;g__Pantoea;s__Pantoea_sp._ARC607
PB2_gene_984	467	ORM85487.1	488	446/467(95.50)	0	hypothetical protein HA42_02650 [Pantoea deleyi]	470932	d__Bacteria;k__norank;p__Proteobacteria;c__Gammaproteobacteria;o__Enterobacterales;f__Erwiniaceae;g__Pantoea;s__Pantoea_deleyi
PB2_gene_987	488	WP_064085246.1	488	487/488(99.80)	0	Asp-tRNA(Asn)/Glu-tRNA(Gln) amidotransferase subunit GatA [Wolbachia endosymbiont of Dactylopius coccus]	None	Unclassified
PB2_gene_991	471	ORM80757.1	471	464/471(98.51)	0	uroporphyrinogen-III C-methyltransferase [Pantoea	470932	d__Bacteria;k__norank;p__Proteobacteria;c__Gammaproteobacteria;o__Enterobacterales;f__Erwiniaceae;g__Pantoea;s__Pantoea_deleyi
PB2_gene_992	325	ORM80756.1	337	315/325(96.92)	1.55e-232	aminopeptidase [Pantoea deleyi]	470932	d__Bacteria;k__norank;p__Proteobacteria;c__Gammaproteobacteria;o__Enterobacterales;f__Erwiniaceae;g__Pantoea;s__Pantoea_deleyi
PB2_gene_994	573	ORM80753.1	573	569/573(99.30)	0	sulfite reductase subunit beta [Pantoea deleyi]	470932	d__Bacteria;k__norank;p__Proteobacteria;c__Gammaproteobacteria;o__Enterobacterales;f__Erwiniaceae;g__Pantoea;s__Pantoea_deleyi
PB2_gene_998	659	ORM78173.1	659	653/659(99.09)	0	bifunctional UDP-glucuronic acid oxidase/UDP-4-amino-4-deoxy-L-arabinose formyltransferase [Pantoea deleyi]	470932	d__Bacteria;k__norank;p__Proteobacteria;c__Gammaproteobacteria;o__Enterobacterales;f__Erwiniaceae;g__Pantoea;s__Pantoea_deleyi
PB2_gene_1001	203	ORM78181.1	193	185/191(96.86)	3.15e-125	hypothetical protein HA42_16965 [Pantoea deleyi]	470932	d__Bacteria;k__norank;p__Proteobacteria;c__Gammaproteobacteria;o__Enterobacterales;f__Erwiniaceae;g__Pantoea;s__Pantoea_deleyi
PB2_gene_1002	259	ORM78176.1	259	241/259(93.05)	2.02e-167	hypothetical protein HA42_16970 [Pantoea deleyi]	470932	d__Bacteria;k__norank;p__Proteobacteria;c__Gammaproteobacteria;o__Enterobacterales;f__Erwiniaceae;g__Pantoea;s__Pantoea_deleyi
PB2_gene_1005	299	ORM82199.1	299	290/298(97.32)	2.05e-196	LysR family transcriptional regulator [Pantoea deleyi]	470932	d__Bacteria;k__norank;p__Proteobacteria;c__Gammaproteobacteria;o__Enterobacterales;f__Erwiniaceae;g__Pantoea;s__Pantoea_deleyi
PB2_gene_1006	631	ORM82200.1	631	628/631(99.52)	0	DNA topoisomerase IV subunit B [Pantoea deleyi]	470932	d__Bacteria;k__norank;p__Proteobacteria;c__Gammaproteobacteria;o__Enterobacterales;f__Erwiniaceae;g__Pantoea;s__Pantoea_deleyi
PB2_gene_1009	141	WP_111139895.1	141	141/141(100.00)	5.71e-100	DUF1249 family protein [Pantoea sp. ARC607]	2027922	d__Bacteria;k__norank;p__Proteobacteria;c__Gammaproteobacteria;o__Enterobacterales;f__Erwiniaceae;g__Pantoea;s__Pantoea_sp._ARC607

PB2_gene_1010	212	ORM82204.1	211	211/211(100.00)	1.17e-147	ADP-ribose diphosphatase [Pantoea deleyi]	470932	d__Bacteria;k__norank;p__Proteobacteria;c__Gammaproteobacteria;o__Enterobacterales;f__Erwiniaceae;g__Pantoea;s__Pantoea_deleyi
PB2_gene_1014	287	SKA17738.1	283	278/283(98.23)	6.18e-191	peptide/nickel transport system permease protein [Pantoea agglomerans]	470933	d__Bacteria;k__norank;p__Proteobacteria;c__Gammaproteobacteria;o__Enterobacterales;f__Erwiniaceae;g__Pantoea;s__Pantoea_eucalypti
PB2_gene_1015	318	OQV41666.1	318	312/318(98.11)	1.35e-206	ABC transporter permease [Pantoea vagans]	470934	d__Bacteria;k__norank;p__Proteobacteria;c__Gammaproteobacteria;o__Enterobacterales;f__Erwiniaceae;g__Pantoea;s__Pantoea_vagans
PB2_gene_1017	425	WP_039659901.1	425	404/425(95.06)	4.58e-300	FAD-binding oxidoreductase [Pantoea sp. MBLJ3]	53335	d__Bacteria;k__norank;p__Proteobacteria;c__Gammaproteobacteria;o__Enterobacterales;f__Erwiniaceae;g__Pantoea;s__norank
PB2_gene_1021	173	SKA15836.1	173	172/173(99.42)	6.22e-115	shikimate kinase [Pantoea agglomerans]	470933	d__Bacteria;k__norank;p__Proteobacteria;c__Gammaproteobacteria;o__Enterobacterales;f__Erwiniaceae;g__Pantoea;s__Pantoea_eucalypti
PB2_gene_1024	301	WP_111139733.1	271	268/271(98.89)	4.09e-199	adenine-specific DNA-methyltransferase [Pantoea sp. ARC607]	2027922	d__Bacteria;k__norank;p__Proteobacteria;c__Gammaproteobacteria;o__Enterobacterales;f__Erwiniaceae;g__Pantoea;s__Pantoea_sp._ARC607
PB2_gene_1026	225	WP_111139723.1	225	224/225(99.56)	4.90e-158	phosphoglycolate phosphatase [Pantoea sp. ARC607]	2027922	d__Bacteria;k__norank;p__Proteobacteria;c__Gammaproteobacteria;o__Enterobacterales;f__Erwiniaceae;g__Pantoea;s__Pantoea_sp._ARC607
PB2_gene_1027	333	ORM77689.1	333	332/333(99.70)	2.19e-240	tryptophan--tRNA ligase [Pantoea deleyi]	470932	d__Bacteria;k__norank;p__Proteobacteria;c__Gammaproteobacteria;o__Enterobacterales;f__Erwiniaceae;g__Pantoea;s__Pantoea_deleyi
PB2_gene_1060	337	ORM86564.1	328	323/328(98.48)	7.65e-234	tRNA-modifying protein YgfZ [Pantoea deleyi]	470932	d__Bacteria;k__norank;p__Proteobacteria;c__Gammaproteobacteria;o__Enterobacterales;f__Erwiniaceae;g__Pantoea;s__Pantoea_deleyi
PB2_gene_1074	109	ORM81649.1	84	83/84(98.81)	2.42e-47	preprotein translocase subunit TatA [Pantoea deleyi]	470932	d__Bacteria;k__norank;p__Proteobacteria;c__Gammaproteobacteria;o__Enterobacterales;f__Erwiniaceae;g__Pantoea;s__Pantoea_deleyi
PB2_gene_1075	174	ORM83592.1	174	171/174(98.28)	2.01e-125	cytochrome b [Pantoea deleyi]	470932	d__Bacteria;k__norank;p__Proteobacteria;c__Gammaproteobacteria;o__Enterobacterales;f__Erwiniaceae;g__Pantoea;s__Pantoea_deleyi
PB2_gene_1078	219	ORM83594.1	219	213/219(97.26)	2.13e-140	hypothetical protein HA42_05980 [Pantoea deleyi]	470932	d__Bacteria;k__norank;p__Proteobacteria;c__Gammaproteobacteria;o__Enterobacterales;f__Erwiniaceae;g__Pantoea;s__Pantoea_deleyi
PB2_gene_1080	222	ORM83596.1	222	208/222(93.69)	1.61e-141	iron ABC transporter ATP-binding protein FetA [Pantoea	470932	d__Bacteria;k__norank;p__Proteobacteria;c__Gammaproteobacteria;o__Enterobacterales;f__Erwiniaceae;g__Pantoea;s__Pantoea_deleyi
PB2_gene_1083	76	ORM83599.1	76	75/76(98.68)	1.23e-14	hypothetical protein HA42_06010 [Pantoea deleyi]	470932	d__Bacteria;k__norank;p__Proteobacteria;c__Gammaproteobacteria;o__Enterobacterales;f__Erwiniaceae;g__Pantoea;s__Pantoea_deleyi
PB2_gene_1088	308	WP_090966673.1	269	217/269(80.67)	3.62e-153	DUF1460 domain-containing protein [Pantoea sp. OV426]	1881036	d__Bacteria;k__norank;p__Proteobacteria;c__Gammaproteobacteria;o__Enterobacterales;f__Erwiniaceae;g__Pantoea;s__Pantoea_sp._OV426
PB2_gene_1089	207	PQL26783.1	207	206/207(99.52)	3.09e-143	threonine export protein RhtC [Pantoea ananatis]	553	d__Bacteria;k__norank;p__Proteobacteria;c__Gammaproteobacteria;o__Enterobacterales;f__Erwiniaceae;g__Pantoea;s__Pantoea_ananatis
PB2_gene_1090	608	ORM81642.1	608	604/608(99.34)	0	ATP-dependent DNA helicase RecQ [Pantoea deleyi]	470932	d__Bacteria;k__norank;p__Proteobacteria;c__Gammaproteobacteria;o__Enterobacterales;f__Erwiniaceae;g__Pantoea;s__Pantoea_deleyi
PB2_gene_1092	156	ORM81644.1	156	156/156(100.00)	1.35e-103	hypothetical protein HA42_10120 [Pantoea deleyi]	470932	d__Bacteria;k__norank;p__Proteobacteria;c__Gammaproteobacteria;o__Enterobacterales;f__Erwiniaceae;g__Pantoea;s__Pantoea_deleyi
PB2_gene_1102	332	ORM75276.1	332	317/332(95.48)	1.91e-229	aldo/keto reductase [Pantoea deleyi]	470932	d__Bacteria;k__norank;p__Proteobacteria;c__Gammaproteobacteria;o__Enterobacterales;f__Erwiniaceae;g__Pantoea;s__Pantoea_deleyi
PB2_gene_1106	381	WP_111139796.1	381	362/381(95.01)	1.75e-268	putative C-S lyase [Pantoea sp. ARC607]	2027922	d__Bacteria;k__norank;p__Proteobacteria;c__Gammaproteobacteria;o__Enterobacterales;f__Erwiniaceae;g__Pantoea;s__Pantoea_sp._ARC607
PB2_gene_1112	423	PXV72741.1	416	330/415(79.52)	7.37e-246	glycine/D-amino acid oxidase-like deaminating enzyme [Pantoea sp. PNA 03-3]	2135460	d__Bacteria;k__norank;p__Proteobacteria;c__Gammaproteobacteria;o__Enterobacterales;f__Erwiniaceae;g__Pantoea;s__Pantoea_sp._PNA_03-3
PB2_gene_1118	249	ORM80726.1	248	247/248(99.60)	1.98e-171	glutamine ABC transporter substrate-binding protein GlnH [Pantoea deleyi]	470932	d__Bacteria;k__norank;p__Proteobacteria;c__Gammaproteobacteria;o__Enterobacterales;f__Erwiniaceae;g__Pantoea;s__Pantoea_deleyi
PB2_gene_1120	240	WP_111140569.1	240	237/240(98.75)	3.17e-166	glutamine ABC transporter ATP-binding protein GlnQ [Pantoea sp. ARC607]	2027922	d__Bacteria;k__norank;p__Proteobacteria;c__Gammaproteobacteria;o__Enterobacterales;f__Erwiniaceae;g__Pantoea;s__Pantoea_sp._ARC607

PB2_gene_1121	310	ORM80723.1	319	300/310(96.77)	1.31e-218	23S rRNA (adenine(1618)-N(6))-methyltransferase [Pantoea	470932	d__Bacteria;k__norank;p__Proteobacteria;c__Gammaproteobacteria;o__Enterobacterales;f__Erwiniaceae;g__Pantoea;s__Pantoea_deleyi
PB2_gene_1122	210	ORM80722.1	210	205/210(97.62)	3.47e-146	Asp/Glu/hydantoin racemase [Pantoea deleyi]	470932	d__Bacteria;k__norank;p__Proteobacteria;c__Gammaproteobacteria;o__Enterobacterales;f__Erwiniaceae;g__Pantoea;s__Pantoea_deleyi
PB2_gene_1129	544	ERM08264.1	488	480/488(98.36)	0	D-alanyl-D-alanine carboxypeptidase [Pantoea	1332072	d__Bacteria;k__norank;p__Proteobacteria;c__Gammaproteobacteria;o__Enterobacterales;f__Erwiniaceae;g__Pantoea;s__Pantoea_agglomerans
PB2_gene_1130	390	ORM86338.1	390	389/390(99.74)	2.32e-273	GTPase ObgE [Pantoea deleyi]	470932	d__Bacteria;k__norank;p__Proteobacteria;c__Gammaproteobacteria;o__Enterobacterales;f__Erwiniaceae;g__Pantoea;s__Pantoea_deleyi
PB2_gene_1131	85	ORM86337.1	85	85/85(100.00)	5.18e-54	50S ribosomal protein L27 [Pantoea deleyi]	470932	d__Bacteria;k__norank;p__Proteobacteria;c__Gammaproteobacteria;o__Enterobacterales;f__Erwiniaceae;g__Pantoea;s__Pantoea_deleyi
PB2_gene_1132	103	SJZ88050.1	103	103/103(100.00)	4.14e-66	LSU ribosomal protein L21P [Pantoea agglomerans]	470933	d__Bacteria;k__norank;p__Proteobacteria;c__Gammaproteobacteria;o__Enterobacterales;f__Erwiniaceae;g__Pantoea;s__Pantoea_eucalypti
PB2_gene_1135	548	WP_111140102.1	552	546/548(99.64)	0	glucan biosynthesis protein D [Pantoea sp. ARC607]	2027922	d__Bacteria;k__norank;p__Proteobacteria;c__Gammaproteobacteria;o__Enterobacterales;f__Erwiniaceae;g__Pantoea;s__Pantoea_sp._ARC607
PB2_gene_1137	393	WP_109650850.1	398	372/393(94.66)	4.41e-267	nicotinamide mononucleotide deamidase-related protein YfaY [Pantoea allii]	549	d__Bacteria;k__norank;p__Proteobacteria;c__Gammaproteobacteria;o__Enterobacterales;f__Erwiniaceae;g__Pantoea;s__Pantoea_agglomerans
PB2_gene_1139	376	ORM83847.1	376	375/376(99.73)	1.91e-274	ribonucleotide-diphosphate reductase subunit beta [Pantoea deleyi]	470932	d__Bacteria;k__norank;p__Proteobacteria;c__Gammaproteobacteria;o__Enterobacterales;f__Erwiniaceae;g__Pantoea;s__Pantoea_deleyi
PB2_gene_1140	761	ORM83846.1	761	758/761(99.61)	0	ribonucleoside-diphosphate reductase subunit alpha [Pantoea deleyi]	470932	d__Bacteria;k__norank;p__Proteobacteria;c__Gammaproteobacteria;o__Enterobacterales;f__Erwiniaceae;g__Pantoea;s__Pantoea_deleyi
PB2_gene_1142	428	WP_111140352.1	435	424/428(99.07)	3.83e-293	MFS transporter [Pantoea sp. ARC607]	2027922	d__Bacteria;k__norank;p__Proteobacteria;c__Gammaproteobacteria;o__Enterobacterales;f__Erwiniaceae;g__Pantoea;s__Pantoea_sp._ARC607
PB2_gene_1145	82	EIB98834.1	149	45/56(80.36)	1.29e-24	hypothetical protein S7A_09985 [Pantoea sp. Sc1]	593105	d__Bacteria;k__norank;p__Proteobacteria;c__Gammaproteobacteria;o__Enterobacterales;f__Erwiniaceae;g__Pantoea;s__Pantoea_sp._Sc1
PB2_gene_1146	207	WP_111140348.1	207	193/207(93.24)	1.65e-138	glucokinase [Pantoea sp. ARC607]	2027922	d__Bacteria;k__norank;p__Proteobacteria;c__Gammaproteobacteria;o__Enterobacterales;f__Erwiniaceae;g__Pantoea;s__Pantoea_sp._ARC607
PB2_gene_1149	280	ORM76106.1	280	278/280(99.29)	1.41e-198	iron ABC transporter substrate-binding protein [Pantoea deleyi]	470932	d__Bacteria;k__norank;p__Proteobacteria;c__Gammaproteobacteria;o__Enterobacterales;f__Erwiniaceae;g__Pantoea;s__Pantoea_deleyi
PB2_gene_1152	561	WP_111140578.1	561	555/561(98.93)	0	urocanate hydratase [Pantoea sp. ARC607]	2027922	d__Bacteria;k__norank;p__Proteobacteria;c__Gammaproteobacteria;o__Enterobacterales;f__Erwiniaceae;g__Pantoea;s__Pantoea_sp._ARC607
PB2_gene_1154	321	ORM76529.1	321	316/321(98.44)	3.97e-221	DNA-binding protein YbiB [Pantoea deleyi]	470932	d__Bacteria;k__norank;p__Proteobacteria;c__Gammaproteobacteria;o__Enterobacterales;f__Erwiniaceae;g__Pantoea;s__Pantoea_deleyi
PB2_gene_1155	722	ORM76530.1	722	713/722(98.75)	0	ATP-dependent DNA helicase DinG [Pantoea deleyi]	470932	d__Bacteria;k__norank;p__Proteobacteria;c__Gammaproteobacteria;o__Enterobacterales;f__Erwiniaceae;g__Pantoea;s__Pantoea_deleyi
PB2_gene_1157	477	WP_111139165.1	477	470/477(98.53)	0	magnesium transporter [Pantoea sp. ARC607]	2027922	d__Bacteria;k__norank;p__Proteobacteria;c__Gammaproteobacteria;o__Enterobacterales;f__Erwiniaceae;g__Pantoea;s__Pantoea_sp._ARC607
PB2_gene_1161	686	ORM81018.1	686	683/686(99.56)	0	RNA degradosome polyphosphate kinase [Pantoea	470932	d__Bacteria;k__norank;p__Proteobacteria;c__Gammaproteobacteria;o__Enterobacterales;f__Erwiniaceae;g__Pantoea;s__Pantoea_deleyi
PB2_gene_1167	358	SJZ86354.1	358	354/358(98.88)	4.78e-243	lipopolysaccharide export system permease protein [Pantoea agglomerans]	470933	d__Bacteria;k__norank;p__Proteobacteria;c__Gammaproteobacteria;o__Enterobacterales;f__Erwiniaceae;g__Pantoea;s__Pantoea_eucalypti
PB2_gene_1168	369	ORM86020.1	371	350/368(95.11)	2.43e-258	peptidase M20 [Pantoea deleyi]	470932	d__Bacteria;k__norank;p__Proteobacteria;c__Gammaproteobacteria;o__Enterobacterales;f__Erwiniaceae;g__Pantoea;s__Pantoea_deleyi
PB2_gene_1170	359	ORM86022.1	359	350/359(97.49)	2.64e-261	aryldialkylphosphatase [Pantoea deleyi]	470932	d__Bacteria;k__norank;p__Proteobacteria;c__Gammaproteobacteria;o__Enterobacterales;f__Erwiniaceae;g__Pantoea;s__Pantoea_deleyi
PB2_gene_1171	326	WP_111138532.1	336	324/326(99.39)	1.06e-214	ABC transporter permease [Pantoea sp. ARC607]	2027922	d__Bacteria;k__norank;p__Proteobacteria;c__Gammaproteobacteria;o__Enterobacterales;f__Erwiniaceae;g__Pantoea;s__Pantoea_sp._ARC607

PB2_gene_1174	151	ORM82058.1	151	145/150(96.67)	2.36e-106	GNAT family N-acetyltransferase [Pantoea deleyi]	470932	d__Bacteria;k__norank;p__Proteobacteria;c__Gammaproteobacteria;o__Enterobacterales;f__Erwiniaceae;g__Pantoea;s__Pantoea_deleyi
PB2_gene_1175	370	WP_111138774.1	370	370/370(100.00)	2.03e-266	iron-sulfur cluster carrier protein ApbC [Pantoea sp. ARC607]	53335	d__Bacteria;k__norank;p__Proteobacteria;c__Gammaproteobacteria;o__Enterobacterales;f__Erwiniaceae;g__Pantoea;s__norank
PB2_gene_1176	677	WP_111138773.1	677	659/677(97.34)	0	methionine--tRNA ligase [Pantoea sp. ARC607]	2027922	d__Bacteria;k__norank;p__Proteobacteria;c__Gammaproteobacteria;o__Enterobacterales;f__Erwiniaceae;g__Pantoea;s__Pantoea_sp._ARC607
PB2_gene_1181	388	PBQ28175.1	388	385/388(99.23)	1.42e-282	methionine adenosyltransferase [Wolbachia pipientis wAus]	2049495	d__Bacteria;k__norank;p__Proteobacteria;c__Alphaproteobacteria;o__Rickettsiales;f__Anaplasmataceae;g__Wolbachia;s__Wolbachia_pipientis
PB2_gene_1194	273	ORM84520.1	273	273/273(100.00)	7.26e-193	serine O-acetyltransferase [Pantoea deleyi]	470932	d__Bacteria;k__norank;p__Proteobacteria;c__Gammaproteobacteria;o__Enterobacterales;f__Erwiniaceae;g__Pantoea;s__Pantoea_deleyi
PB2_gene_1207	392	SKA10930.1	394	389/394(98.73)	3.18e-245	xylose ABC transporter membrane protein [Pantoea	470933	d__Bacteria;k__norank;p__Proteobacteria;c__Gammaproteobacteria;o__Enterobacterales;f__Erwiniaceae;g__Pantoea;s__Pantoea_eucalypti
PB2_gene_1208	404	ORM85473.1	392	387/390(99.23)	8.06e-285	XylR family transcriptional regulator [Pantoea deleyi]	470932	d__Bacteria;k__norank;p__Proteobacteria;c__Gammaproteobacteria;o__Enterobacterales;f__Erwiniaceae;g__Pantoea;s__Pantoea_deleyi
PB2_gene_1209	267	WP_034827539.1	288	62/287(21.60)	8.04e-07	hypothetical protein [Enterobacter cancerogenus]	69218	d__Bacteria;k__norank;p__Proteobacteria;c__Gammaproteobacteria;o__Enterobacterales;f__Enterobacteriaceae;g__Enterobacter;s__Enterobacter_cancerogenus
PB2_gene_1214	424	PBD15897.1	424	408/424(96.23)	1.09e-286	insulinase family protein [Wolbachia endosymbiont of Drosophila subpulchrella]	2033655	d__Bacteria;k__norank;p__Proteobacteria;c__Alphaproteobacteria;o__Rickettsiales;f__Anaplasmataceae;g__Wolbachia;s__Wolbachia_endosymbiont_of_Drosophila_subpulchrella
PB2_gene_1215	552	POG51885.1	456	329/475(69.26)	2.44e-214	hypothetical protein BJU59_03170, partial	1932745	d__Bacteria;k__norank;p__Proteobacteria;c__Alphaproteobacteria;o__Rickettsiales;f__Anaplasmataceae;g__Wolbachia;s__Wolbachia_sp._wRi_2
PB2_gene_1222	140	PAW36569.1	140	138/140(98.57)	5.51e-93	5-hydroxyisourate hydrolase [Pantoea vagans]	470934	d__Bacteria;k__norank;p__Proteobacteria;c__Gammaproteobacteria;o__Enterobacterales;f__Erwiniaceae;g__Pantoea;s__Pantoea_vagans
PB2_gene_1225	534	WP_111140499.1	534	528/534(98.88)	0	GMC family oxidoreductase [Pantoea sp. ARC607]	2027922	d__Bacteria;k__norank;p__Proteobacteria;c__Gammaproteobacteria;o__Enterobacterales;f__Erwiniaceae;g__Pantoea;s__Pantoea_sp._ARC607
PB2_gene_1227	551	WP_064085779.1	558	529/533(99.25)	8.48e-264	hypothetical protein [Wolbachia endosymbiont of Dactylopius coccus]	None	Unclassified
PB2_gene_1234	420	WP_071242811.1	420	388/420(92.38)	1.27e-283	lipopolysaccharide heptosyltransferase family protein [Pantoea vagans]	470934	d__Bacteria;k__norank;p__Proteobacteria;c__Gammaproteobacteria;o__Enterobacterales;f__Erwiniaceae;g__Pantoea;s__Pantoea_vagans
PB2_gene_1237	213	ORM77221.1	213	209/213(98.12)	1.93e-149	formate dehydrogenase cytochrome b556 subunit	470932	d__Bacteria;k__norank;p__Proteobacteria;c__Gammaproteobacteria;o__Enterobacterales;f__Erwiniaceae;g__Pantoea;s__Pantoea_deleyi
PB2_gene_1241	243	ORM76964.1	243	242/243(99.59)	9.80e-167	amino acid ABC transporter ATP-binding protein [Pantoea	470932	d__Bacteria;k__norank;p__Proteobacteria;c__Gammaproteobacteria;o__Enterobacterales;f__Erwiniaceae;g__Pantoea;s__Pantoea_deleyi
PB2_gene_1242	219	WP_098051407.1	219	217/219(99.09)	5.51e-152	amino acid ABC transporter permease [Pantoea agglomerans]	2608017	d__Bacteria;k__norank;p__Proteobacteria;c__Gammaproteobacteria;o__Enterobacterales;f__Erwiniaceae;g__Pantoea;s__Pantoea_sp._Bo_3
PB2_gene_1243	222	ORM76963.1	222	219/222(98.65)	3.30e-150	polar amino acid ABC transporter permease [Pantoea deleyi]	470932	d__Bacteria;k__norank;p__Proteobacteria;c__Gammaproteobacteria;o__Enterobacterales;f__Erwiniaceae;g__Pantoea;s__Pantoea_deleyi
PB2_gene_1244	261	ORM76962.1	261	259/261(99.23)	1.19e-176	amino acid ABC transporter substrate-binding protein [Pantoea deleyi]	470932	d__Bacteria;k__norank;p__Proteobacteria;c__Gammaproteobacteria;o__Enterobacterales;f__Erwiniaceae;g__Pantoea;s__Pantoea_deleyi
PB2_gene_1246	528	WP_111140129.1	528	511/528(96.78)	0	gamma-glutamyltransferase family protein [Pantoea sp.	2027922	d__Bacteria;k__norank;p__Proteobacteria;c__Gammaproteobacteria;o__Enterobacterales;f__Erwiniaceae;g__Pantoea;s__Pantoea_sp._ARC607
PB2_gene_1252	393	WP_111139912.1	388	383/388(98.71)	3.30e-255	tRNA/rRNA methyltransferase [Pantoea sp. ARC607]	2027922	d__Bacteria;k__norank;p__Proteobacteria;c__Gammaproteobacteria;o__Enterobacterales;f__Erwiniaceae;g__Pantoea;s__Pantoea_sp._ARC607
PB2_gene_1254	397	ORM75814.1	390	355/361(98.34)	9.56e-245	multidrug export protein EmrA [Pantoea deleyi]	470932	d__Bacteria;k__norank;p__Proteobacteria;c__Gammaproteobacteria;o__Enterobacterales;f__Erwiniaceae;g__Pantoea;s__Pantoea_deleyi

PB2_gene_1257	328	WP_064085336.1	362	327/328(99.70)	1.93e-210	phosphate ABC transporter permease subunit PstC [Wolbachia endosymbiont of Dactylopius coccus]	None	Unclassified
PB2_gene_1265	382	WP_111140644.1	387	381/382(99.74)	5.36e-267	phosphoglycerate kinase [Pantoea sp. ARC607]	2027922	d__Bacteria;k__norank;p__Proteobacteria;c__Gammaproteobacteria;o__Enterobacterales;f__Erwiniaceae;g__Pantoea;s__Pantoea_sp._ARC607
PB2_gene_1266	359	ORM86546.1	359	359/359(100.00)	4.08e-265	class II fructose-bisphosphate aldolase [Pantoea deleyi]	470932	d__Bacteria;k__norank;p__Proteobacteria;c__Gammaproteobacteria;o__Enterobacterales;f__Erwiniaceae;g__Pantoea;s__Pantoea_deleyi
PB2_gene_1273	156	WP_111142260.1	191	43/60(71.67)	1.80e-17	acetate uptake transporter [Pantoea sp. ARC607]	2027922	d__Bacteria;k__norank;p__Proteobacteria;c__Gammaproteobacteria;o__Enterobacterales;f__Erwiniaceae;g__Pantoea;s__Pantoea_sp._ARC607
PB2_gene_1274	436	ORM83995.1	436	419/436(96.10)	3.60e-282	MFS transporter [Pantoea deleyi]	470932	d__Bacteria;k__norank;p__Proteobacteria;c__Gammaproteobacteria;o__Enterobacterales;f__Erwiniaceae;g__Pantoea;s__Pantoea_deleyi
PB2_gene_1281	309	WP_111138428.1	308	294/308(95.45)	3.49e-211	LysR family transcriptional regulator [Pantoea sp. ARC607]	2027922	d__Bacteria;k__norank;p__Proteobacteria;c__Gammaproteobacteria;o__Enterobacterales;f__Erwiniaceae;g__Pantoea;s__Pantoea_sp._ARC607
PB2_gene_1287	305	ORM80531.1	305	295/305(96.72)	6.71e-205	ribokinase [Pantoea deleyi]	470932	d__Bacteria;k__norank;p__Proteobacteria;c__Gammaproteobacteria;o__Enterobacterales;f__Erwiniaceae;g__Pantoea;s__Pantoea_deleyi
PB2_gene_1294	354	ORM80914.1	354	349/354(98.59)	9.78e-250	cytochrome-c peroxidase [Pantoea deleyi]	470932	d__Bacteria;k__norank;p__Proteobacteria;c__Gammaproteobacteria;o__Enterobacterales;f__Erwiniaceae;g__Pantoea;s__Pantoea_deleyi
PB2_gene_1295	436	WP_111141818.1	436	434/436(99.54)	0	MFS transporter [Pantoea sp. ARC607]	2027922	d__Bacteria;k__norank;p__Proteobacteria;c__Gammaproteobacteria;o__Enterobacterales;f__Erwiniaceae;g__Pantoea;s__Pantoea_sp._ARC607
PB2_gene_1300	261	ORM81091.1	261	257/261(98.47)	8.47e-186	tRNA uridine 5-oxyacetic acid(34) methyltransferase CmoM [Pantoea deleyi]	470932	d__Bacteria;k__norank;p__Proteobacteria;c__Gammaproteobacteria;o__Enterobacterales;f__Erwiniaceae;g__Pantoea;s__Pantoea_deleyi
PB2_gene_1310	237	ORM86568.1	237	236/237(99.58)	9.52e-171	bifunctional protein-disulfide isomerase/oxidoreductase DsbC [Pantoea deleyi]	470932	d__Bacteria;k__norank;p__Proteobacteria;c__Gammaproteobacteria;o__Enterobacterales;f__Erwiniaceae;g__Pantoea;s__Pantoea_deleyi
PB2_gene_1311	578	ORM86569.1	578	575/578(99.48)	0	single-stranded-DNA-specific exonuclease RecJ [Pantoea	470932	d__Bacteria;k__norank;p__Proteobacteria;c__Gammaproteobacteria;o__Enterobacterales;f__Erwiniaceae;g__Pantoea;s__Pantoea_deleyi
PB2_gene_1326	215	ORM86100.1	215	212/215(98.60)	3.33e-151	hypothetical protein HA42_01285 [Pantoea deleyi]	470932	d__Bacteria;k__norank;p__Proteobacteria;c__Gammaproteobacteria;o__Enterobacterales;f__Erwiniaceae;g__Pantoea;s__Pantoea_deleyi
PB2_gene_1328	305	WP_111140064.1	294	281/294(95.58)	1.33e-206	PHP domain-containing protein [Pantoea sp. ARC607]	2027922	d__Bacteria;k__norank;p__Proteobacteria;c__Gammaproteobacteria;o__Enterobacterales;f__Erwiniaceae;g__Pantoea;s__Pantoea_sp._ARC607
PB2_gene_1333	175	WP_113654913.1	175	160/175(91.43)	2.44e-122	toxin-activating lysine-acyltransferase [Pantoea sp.	2259618	d__Bacteria;k__norank;p__Proteobacteria;c__Gammaproteobacteria;o__Enterobacterales;f__Erwiniaceae;g__Pantoea;s__Pantoea_sp._3_1284
PB2_gene_1334	76	OCA55060.1	69	32/56(57.14)	3.24e-11	hypothetical protein Phpb_01867 [Photorhabdus namnaonensis]	1851568	d__Bacteria;k__norank;p__Proteobacteria;c__Gammaproteobacteria;o__Enterobacterales;f__Morganellaceae;g__Photorhabdus;s__Photorhabdus_namnaonensis
PB2_gene_1335	191	SAQ00603.1	649	82/165(49.70)	5.49e-43	Uncharacterised protein [Klebsiella oxytoca]	575	d__Bacteria;k__norank;p__Proteobacteria;c__Gammaproteobacteria;o__Enterobacterales;f__Enterobacteriaceae;g__Raoultella;s__Raoultella_planticola
PB2_gene_1339	393	SAQ00592.1	499	136/305(44.59)	1.19e-70	Uncharacterised protein [Klebsiella oxytoca]	575	d__Bacteria;k__norank;p__Proteobacteria;c__Gammaproteobacteria;o__Enterobacterales;f__Enterobacteriaceae;g__Raoultella;s__Raoultella_planticola
PB2_gene_1341	331	CZY10323.1	842	128/321(39.88)	3.57e-53	Uncharacterised protein [Enterobacter cloacae]	208224	d__Bacteria;k__norank;p__Proteobacteria;c__Gammaproteobacteria;o__Enterobacterales;f__Enterobacteriaceae;g__Enterobacter;s__Enterobacter_kobei
PB2_gene_1343	1017	WP_111139080.1	1017	988/1017(97.15)	0	FAD-binding oxidoreductase [Pantoea sp. ARC607]	2027922	d__Bacteria;k__norank;p__Proteobacteria;c__Gammaproteobacteria;o__Enterobacterales;f__Erwiniaceae;g__Pantoea;s__Pantoea_sp._ARC607
PB2_gene_1346	497	ORM80791.1	497	497/497(100.00)	0	Fe-S cluster assembly protein SufB [Pantoea deleyi]	470932	d__Bacteria;k__norank;p__Proteobacteria;c__Gammaproteobacteria;o__Enterobacterales;f__Erwiniaceae;g__Pantoea;s__Pantoea_deleyi
PB2_gene_1347	248	ORM80790.1	248	247/248(99.60)	1.40e-173	Fe-S cluster assembly ATPase SufC [Pantoea deleyi]	470932	d__Bacteria;k__norank;p__Proteobacteria;c__Gammaproteobacteria;o__Enterobacterales;f__Erwiniaceae;g__Pantoea;s__Pantoea_deleyi
PB2_gene_1353	209	ORM82748.1	209	206/209(98.56)	6.22e-144	protein disulfide oxidoreductase DsbA [Pantoea deleyi]	470932	d__Bacteria;k__norank;p__Proteobacteria;c__Gammaproteobacteria;o__Enterobacterales;f__Erwiniaceae;g__Pantoea;s__Pantoea_deleyi

PB2_gene_1356	522	ORM80734.1	522	522/522(100.00)	0	EmrB/QacA family drug resistance transporter [Pantoea	470932	d__Bacteria;k__norank;p__Proteobacteria;c__Gammaproteobacteria;o__Enterobacterales;f__Erwiniaceae;g__Pantoea;s__Pantoea_deleyi
PB2_gene_1357	384	ORM80733.1	381	356/381(93.44)	5.74e-161	multidrug export protein EmrA [Pantoea deleyi]	470932	d__Bacteria;k__norank;p__Proteobacteria;c__Gammaproteobacteria;o__Enterobacterales;f__Erwiniaceae;g__Pantoea;s__Pantoea_deleyi
PB2_gene_1361	170	WP_111140564.1	170	170/170(100.00)	7.62e-119	outer membrane protein OmpX [Pantoea sp. ARC607]	2027922	d__Bacteria;k__norank;p__Proteobacteria;c__Gammaproteobacteria;o__Enterobacterales;f__Erwiniaceae;g__Pantoea;s__Pantoea_sp._ARC607
PB2_gene_1363	453	WP_041689518.1	453	418/453(92.27)	3.78e-288	2-hydroxycarboxylate transporter family protein [Enterobacter sp.	2608935	d__Bacteria;k__norank;p__Proteobacteria;c__Gammaproteobacteria;o__Enterobacterales;f__Enterobacteriaceae;g__Enterobacter;s__norank
PB2_gene_1365	346	WP_089561848.1	341	267/341(78.30)	9.84e-192	MULTISPECIES: [citrate (pro-3S)-lyase] ligase [Pantoea]	53335	d__Bacteria;k__norank;p__Proteobacteria;c__Gammaproteobacteria;o__Enterobacterales;f__Erwiniaceae;g__Pantoea;s__norank
PB2_gene_1373	758	ORM76078.1	758	754/758(99.47)	0	ATP-dependent Clp protease ATP-binding subunit ClpA [Pantoea deleyi]	470932	d__Bacteria;k__norank;p__Proteobacteria;c__Gammaproteobacteria;o__Enterobacterales;f__Erwiniaceae;g__Pantoea;s__Pantoea_deleyi
PB2_gene_1377	434	WP_111142127.1	434	431/434(99.31)	1.43e-303	MFS transporter [Pantoea sp. ARC607]	2027922	d__Bacteria;k__norank;p__Proteobacteria;c__Gammaproteobacteria;o__Enterobacterales;f__Erwiniaceae;g__Pantoea;s__Pantoea_sp._ARC607
PB2_gene_1378	471	WP_111142128.1	471	462/471(98.09)	0	glucuronate isomerase [Pantoea sp. ARC607]	2027922	d__Bacteria;k__norank;p__Proteobacteria;c__Gammaproteobacteria;o__Enterobacterales;f__Erwiniaceae;g__Pantoea;s__Pantoea_sp._ARC607
PB2_gene_1380	496	ORM78834.1	496	484/496(97.58)	0	altronate hydrolase [Pantoea deleyi]	470932	d__Bacteria;k__norank;p__Proteobacteria;c__Gammaproteobacteria;o__Enterobacterales;f__Erwiniaceae;g__Pantoea;s__Pantoea_deleyi
PB2_gene_1384	345	WP_010405337.1	345	343/345(99.42)	2.03e-242	phosphate acyltransferase PlsX [Wolbachia endosymbiont of Nasonia vitripennis]	2640676	d__Bacteria;k__norank;p__Proteobacteria;c__Alphaproteobacteria;o__Rickettsiales;f__Anaplasmataceae;g__Wolbachia;s__norank
PB2_gene_1386	233	AZU37648.1	233	231/233(99.14)	3.69e-157	ABC transporter ATP-binding protein [Wolbachia endosymbiont of Bemisia tabaci]	215173	d__Bacteria;k__norank;p__Proteobacteria;c__Alphaproteobacteria;o__Rickettsiales;f__Anaplasmataceae;g__Wolbachia;s__Wolbachia_endosymbiont_of_Bemisia_tabaci
PB2_gene_1391	841	WP_088901008.1	838	672/836(80.38)	0	fimbrial biogenesis outer membrane usher protein [Pantoea	2008894	d__Bacteria;k__norank;p__Proteobacteria;c__Gammaproteobacteria;o__Enterobacterales;f__Erwiniaceae;g__Pantoea;s__Pantoea_sp._AMG_501
PB2_gene_1394	213	WP_064365660.1	227	104/200(52.00)	1.64e-72	EAL domain-containing protein [Pantoea ananatis]	553	d__Bacteria;k__norank;p__Proteobacteria;c__Gammaproteobacteria;o__Enterobacterales;f__Erwiniaceae;g__Pantoea;s__Pantoea_ananatis
PB2_gene_1397	301	ORM85245.1	301	293/301(97.34)	4.92e-205	transcriptional regulator [Pantoea deleyi]	470932	d__Bacteria;k__norank;p__Proteobacteria;c__Gammaproteobacteria;o__Enterobacterales;f__Erwiniaceae;g__Pantoea;s__Pantoea_deleyi
PB2_gene_1398	260	ORM85244.1	260	257/260(98.85)	3.46e-189	alpha-acetolactate decarboxylase [Pantoea deleyi]	470932	d__Bacteria;k__norank;p__Proteobacteria;c__Gammaproteobacteria;o__Enterobacterales;f__Erwiniaceae;g__Pantoea;s__Pantoea_deleyi
PB2_gene_1399	561	WP_111138261.1	559	544/559(97.32)	0	acetolactate synthase AlsS [Pantoea sp. ARC607]	2027922	d__Bacteria;k__norank;p__Proteobacteria;c__Gammaproteobacteria;o__Enterobacterales;f__Erwiniaceae;g__Pantoea;s__Pantoea_sp._ARC607
PB2_gene_1401	483	ORM85242.1	483	470/483(97.31)	0	flagellin [Pantoea deleyi]	470932	d__Bacteria;k__norank;p__Proteobacteria;c__Gammaproteobacteria;o__Enterobacterales;f__Erwiniaceae;g__Pantoea;s__Pantoea_deleyi
PB2_gene_1405	623	WP_111140756.1	623	606/623(97.27)	0	penicillin-binding protein 2 [Pantoea sp. ARC607]	2027922	d__Bacteria;k__norank;p__Proteobacteria;c__Gammaproteobacteria;o__Enterobacterales;f__Erwiniaceae;g__Pantoea;s__Pantoea_sp._ARC607
PB2_gene_1409	404	WP_111142043.1	422	390/404(96.53)	2.56e-277	membrane integrity-associated transporter subunit PqiA [Pantoea sp. ARC607]	2027922	d__Bacteria;k__norank;p__Proteobacteria;c__Gammaproteobacteria;o__Enterobacterales;f__Erwiniaceae;g__Pantoea;s__Pantoea_sp._ARC607
PB2_gene_1411	189	ORM84715.1	189	185/188(98.40)	3.26e-127	hypothetical protein HA42_04030 [Pantoea deleyi]	470932	d__Bacteria;k__norank;p__Proteobacteria;c__Gammaproteobacteria;o__Enterobacterales;f__Erwiniaceae;g__Pantoea;s__Pantoea_deleyi
PB2_gene_1413	1263	WP_111138968.1	1510	1138/1265(89.96)	0	ATP-dependent helicase [Pantoea sp. ARC607]	2027922	d__Bacteria;k__norank;p__Proteobacteria;c__Gammaproteobacteria;o__Enterobacterales;f__Erwiniaceae;g__Pantoea;s__Pantoea_sp._ARC607
PB2_gene_1415	483	WP_111138922.1	483	478/483(98.96)	0	sugar porter family MFS transporter [Pantoea sp. ARC607]	2027922	d__Bacteria;k__norank;p__Proteobacteria;c__Gammaproteobacteria;o__Enterobacterales;f__Erwiniaceae;g__Pantoea;s__Pantoea_sp._ARC607
PB2_gene_1416	346	ORM78918.1	327	323/327(98.78)	1.54e-226	L-Ala-D/L-Glu epimerase [Pantoea deleyi]	470932	d__Bacteria;k__norank;p__Proteobacteria;c__Gammaproteobacteria;o__Enterobacterales;f__Erwiniaceae;g__Pantoea;s__Pantoea_deleyi

PB2_gene_1418	539	WP_111139488.1	539	530/539(98.33)	0	peptide ABC transporter substrate-binding protein [Pantoea sp. ARC607]	2027922	d__Bacteria;k__norank;p__Proteobacteria;c__Gammaproteobacteria;o__Enterobacterales;f__Erwiniaceae;g__Pantoea;s__Pantoea_sp._ARC607
PB2_gene_1421	311	ORM78921.1	311	309/311(99.36)	5.93e-227	tRNA 2-thiocytidine(32) synthetase TtcA [Pantoea deleyi]	470932	d__Bacteria;k__norank;p__Proteobacteria;c__Gammaproteobacteria;o__Enterobacterales;f__Erwiniaceae;g__Pantoea;s__Pantoea_deleyi
PB2_gene_1424	320	ORM86862.1	322	297/321(92.52)	1.45e-207	PTS mannose transporter subunit IIAB [Pantoea deleyi]	470932	d__Bacteria;k__norank;p__Proteobacteria;c__Gammaproteobacteria;o__Enterobacterales;f__Erwiniaceae;g__Pantoea;s__Pantoea_deleyi
PB2_gene_1427	520	WP_111138864.1	520	491/520(94.42)	0	EAL domain-containing protein [Pantoea sp. ARC607]	2027922	d__Bacteria;k__norank;p__Proteobacteria;c__Gammaproteobacteria;o__Enterobacterales;f__Erwiniaceae;g__Pantoea;s__Pantoea_sp._ARC607
PB2_gene_1436	252	WP_111142139.1	252	244/252(96.83)	7.75e-173	thiazole synthase [Pantoea sp. ARC607]	2027922	d__Bacteria;k__norank;p__Proteobacteria;c__Gammaproteobacteria;o__Enterobacterales;f__Erwiniaceae;g__Pantoea;s__Pantoea_sp._ARC607
PB2_gene_1438	315	WP_111142137.1	312	300/312(96.15)	5.03e-218	FAD-dependent oxidoreductase [Pantoea sp. ARC607]	2027922	d__Bacteria;k__norank;p__Proteobacteria;c__Gammaproteobacteria;o__Enterobacterales;f__Erwiniaceae;g__Pantoea;s__Pantoea_sp._ARC607
PB2_gene_1441	104	ORM75444.1	104	103/104(99.04)	9.35e-68	transcriptional regulator [Pantoea deleyi]	470932	d__Bacteria;k__norank;p__Proteobacteria;c__Gammaproteobacteria;o__Enterobacterales;f__Erwiniaceae;g__Pantoea;s__Pantoea_deleyi
PB2_gene_1442	372	ORM75445.1	372	358/372(96.24)	2.77e-265	alkene reductase [Pantoea deleyi]	470932	d__Bacteria;k__norank;p__Proteobacteria;c__Gammaproteobacteria;o__Enterobacterales;f__Erwiniaceae;g__Pantoea;s__Pantoea_deleyi
PB2_gene_1460	226	PAW34299.1	217	211/217(97.24)	2.58e-156	bifunctional tRNA pseudouridine(32) synthase/23S rRNA pseudouridine(746) synthase RluA [Pantoea vagans]	470934	d__Bacteria;k__norank;p__Proteobacteria;c__Gammaproteobacteria;o__Enterobacterales;f__Erwiniaceae;g__Pantoea;s__Pantoea_vagans
PB2_gene_1461	434	SUB18469.1	474	425/434(97.93)	1.37e-304	RNA polymerase-associated protein rapA [Pantoea	549	d__Bacteria;k__norank;p__Proteobacteria;c__Gammaproteobacteria;o__Enterobacterales;f__Erwiniaceae;g__Pantoea;s__Pantoea_agglomerans
PB2_gene_1463	433	ORM84474.1	388	386/388(99.48)	5.00e-281	serine-type D-Ala-D-Ala carboxypeptidase [Pantoea	470932	d__Bacteria;k__norank;p__Proteobacteria;c__Gammaproteobacteria;o__Enterobacterales;f__Erwiniaceae;g__Pantoea;s__Pantoea_deleyi
PB2_gene_1464	352	WP_111142096.1	352	346/352(98.30)	1.27e-244	FUSC family protein [Pantoea sp. ARC607]	2027922	d__Bacteria;k__norank;p__Proteobacteria;c__Gammaproteobacteria;o__Enterobacterales;f__Erwiniaceae;g__Pantoea;s__Pantoea_sp._ARC607
PB2_gene_1465	108	SJZ61627.1	108	108/108(100.00)	2.44e-65	hypothetical protein SAMN03097723_1501 [Pantoea agglomerans]	470933	d__Bacteria;k__norank;p__Proteobacteria;c__Gammaproteobacteria;o__Enterobacterales;f__Erwiniaceae;g__Pantoea;s__Pantoea_eucalypti
PB2_gene_1466	385	ORM84471.1	353	343/353(97.17)	1.77e-237	lac repressor [Pantoea deleyi]	470932	d__Bacteria;k__norank;p__Proteobacteria;c__Gammaproteobacteria;o__Enterobacterales;f__Erwiniaceae;g__Pantoea;s__Pantoea_deleyi
PB2_gene_1469	420	ORM84894.1	420	417/420(99.29)	7.29e-299	oxalate/formate MFS antiporter [Pantoea deleyi]	470932	d__Bacteria;k__norank;p__Proteobacteria;c__Gammaproteobacteria;o__Enterobacterales;f__Erwiniaceae;g__Pantoea;s__Pantoea_deleyi
PB2_gene_1471	240	STQ79075.1	240	229/240(95.42)	8.59e-155	Uncharacterised protein [Hafnia alvei]	569	d__Bacteria;k__norank;p__Proteobacteria;c__Gammaproteobacteria;o__Enterobacterales;f__Hafniaceae;g__Hafnia;s__Hafnia_alvei
PB2_gene_1474	160	ORM84897.1	160	160/160(100.00)	4.32e-111	SsrA-binding protein [Pantoea deleyi]	470932	d__Bacteria;k__norank;p__Proteobacteria;c__Gammaproteobacteria;o__Enterobacterales;f__Erwiniaceae;g__Pantoea;s__Pantoea_deleyi
PB2_gene_1475	484	ORM85623.1	606	472/484(97.52)	0	outer membrane assembly protein AsmA [Pantoea deleyi]	470932	d__Bacteria;k__norank;p__Proteobacteria;c__Gammaproteobacteria;o__Enterobacterales;f__Erwiniaceae;g__Pantoea;s__Pantoea_deleyi
PB2_gene_1478	204	ORM85621.1	204	200/204(98.04)	4.57e-139	hypothetical protein HA42_02110 [Pantoea deleyi]	470932	d__Bacteria;k__norank;p__Proteobacteria;c__Gammaproteobacteria;o__Enterobacterales;f__Erwiniaceae;g__Pantoea;s__Pantoea_deleyi
PB2_gene_1488	180	WP_010402210.1	180	178/180(98.89)	8.80e-132	hypothetical protein [Wolbachia endosymbiont of Nasonia vitripennis]	180837	d__Bacteria;k__norank;p__Proteobacteria;c__Alphaproteobacteria;o__Rickettsiales;f__Anaplasmataceae;g__Wolbachia;s__Wolbachia_endosymbiont_of_Nasonia_vitripennis
PB2_gene_1495	303	WP_095706530.1	303	286/303(94.39)	3.14e-218	alpha/beta fold hydrolase [Pantoea vagans]	470934	d__Bacteria;k__norank;p__Proteobacteria;c__Gammaproteobacteria;o__Enterobacterales;f__Erwiniaceae;g__Pantoea;s__Pantoea_vagans
PB2_gene_1496	463	ORM75699.1	463	456/463(98.49)	0	hypothetical protein HA42_20065 [Pantoea deleyi]	470932	d__Bacteria;k__norank;p__Proteobacteria;c__Gammaproteobacteria;o__Enterobacterales;f__Erwiniaceae;g__Pantoea;s__Pantoea_deleyi
PB2_gene_1497	450	WP_111142123.1	450	445/450(98.89)	0	MFS transporter [Pantoea sp. ARC607]	2027922	d__Bacteria;k__norank;p__Proteobacteria;c__Gammaproteobacteria;o__Enterobacterales;f__Erwiniaceae;g__Pantoea;s__Pantoea_sp._ARC607

PB2_gene_1502	582	ORM76692.1	582	570/582(97.94)	0	bifunctional isocitrate dehydrogenase kinase/phosphatase [Pantoea	470932	d__Bacteria;k__norank;p__Proteobacteria;c__Gammaproteobacteria;o__Enterobacterales;f__Erwiniaceae;g__Pantoea;s__Pantoea_deleyi
PB2_gene_1506	103	ORM85482.1	249	97/103(94.17)	6.38e-64	xylose isomerase [Pantoea deleyi]	470932	d__Bacteria;k__norank;p__Proteobacteria;c__Gammaproteobacteria;o__Enterobacterales;f__Erwiniaceae;g__Pantoea;s__Pantoea_deleyi
PB2_gene_1508	426	WP_111141066.1	426	420/426(98.59)	1.96e-306	MFS transporter [Pantoea sp. ARC607]	2027922	d__Bacteria;k__norank;p__Proteobacteria;c__Gammaproteobacteria;o__Enterobacterales;f__Erwiniaceae;g__Pantoea;s__Pantoea_sp._ARC607
PB2_gene_1525	429	WP_111138526.1	429	420/428(98.13)	8.89e-300	DUF445 domain-containing protein [Pantoea sp. ARC607]	2027922	d__Bacteria;k__norank;p__Proteobacteria;c__Gammaproteobacteria;o__Enterobacterales;f__Erwiniaceae;g__Pantoea;s__Pantoea_sp._ARC607
PB2_gene_1532	443	ORM80480.1	443	436/443(98.42)	0	glucarate dehydratase [Pantoea deleyi]	470932	d__Bacteria;k__norank;p__Proteobacteria;c__Gammaproteobacteria;o__Enterobacterales;f__Erwiniaceae;g__Pantoea;s__Pantoea_deleyi
PB2_gene_1537	960	WP_096616468.1	956	893/963(92.73)	0	recombinase RecB [Wolbachia pipientis]	955	d__Bacteria;k__norank;p__Proteobacteria;c__Alphaproteobacteria;o__Rickettsiales;f__Anaplasmataceae;g__Wolbachia;s__Wolbachia_pipientis
PB2_gene_1539	284	ORM84534.1	284	281/284(98.94)	8.19e-200	aquaporin [Pantoea deleyi]	470932	d__Bacteria;k__norank;p__Proteobacteria;c__Gammaproteobacteria;o__Enterobacterales;f__Erwiniaceae;g__Pantoea;s__Pantoea_deleyi
PB2_gene_1542	445	ORM84537.1	445	444/445(99.78)	8.12e-311	HslU--HslV peptidase ATPase subunit [Pantoea deleyi]	470932	d__Bacteria;k__norank;p__Proteobacteria;c__Gammaproteobacteria;o__Enterobacterales;f__Erwiniaceae;g__Pantoea;s__Pantoea_deleyi
PB2_gene_1543	163	WP_111140416.1	176	163/163(100.00)	4.06e-109	MULTISPECIES: ATP-dependent protease subunit HslV	53335	d__Bacteria;k__norank;p__Proteobacteria;c__Gammaproteobacteria;o__Enterobacterales;f__Erwiniaceae;g__Pantoea;s__norank
PB2_gene_1545	362	WP_111208710.1	351	339/350(96.86)	5.04e-242	DNA-binding transcriptional regulator CytR [Pantoea sp.	2027923	d__Bacteria;k__norank;p__Proteobacteria;c__Gammaproteobacteria;o__Enterobacterales;f__Erwiniaceae;g__Pantoea;s__Pantoea_sp._ARC270
PB2_gene_1548	168	ORM85845.1	168	165/168(98.21)	1.26e-107	5-(carboxyamino)imidazole ribonucleotide mutase [Pantoea deleyi]	470932	d__Bacteria;k__norank;p__Proteobacteria;c__Gammaproteobacteria;o__Enterobacterales;f__Erwiniaceae;g__Pantoea;s__Pantoea_deleyi
PB2_gene_1549	354	WP_111138984.1	354	346/354(97.74)	6.70e-257	5-(carboxyamino)imidazole ribonucleotide synthase [Pantoea sp. ARC607]	2027922	d__Bacteria;k__norank;p__Proteobacteria;c__Gammaproteobacteria;o__Enterobacterales;f__Erwiniaceae;g__Pantoea;s__Pantoea_sp._ARC607
PB2_gene_1550	805	WP_111138983.1	805	786/805(97.64)	0	ABC transporter permease [Pantoea sp. ARC607]	2027922	d__Bacteria;k__norank;p__Proteobacteria;c__Gammaproteobacteria;o__Enterobacterales;f__Erwiniaceae;g__Pantoea;s__Pantoea_sp._ARC607
PB2_gene_1555	787	ORM82300.1	787	762/787(96.82)	0	hypothetical protein HA42_08720 [Pantoea deleyi]	470932	d__Bacteria;k__norank;p__Proteobacteria;c__Gammaproteobacteria;o__Enterobacterales;f__Erwiniaceae;g__Pantoea;s__Pantoea_deleyi
PB2_gene_1566	218	WP_111139901.1	218	218/218(100.00)	2.17e-153	3,4-dihydroxy-2-butanone-4-phosphate synthase [Pantoea sp. ARC607]	2027922	d__Bacteria;k__norank;p__Proteobacteria;c__Gammaproteobacteria;o__Enterobacterales;f__Erwiniaceae;g__Pantoea;s__Pantoea_sp._ARC607
PB2_gene_1567	129	WP_107320111.1	129	109/129(84.50)	1.08e-62	accessory factor UbiK family protein [Pantoea vagans]	470934	d__Bacteria;k__norank;p__Proteobacteria;c__Gammaproteobacteria;o__Enterobacterales;f__Erwiniaceae;g__Pantoea;s__Pantoea_vagans
PB2_gene_1568	474	WP_111139903.1	474	471/474(99.37)	0	bifunctional D-glycero-beta-D-manno-heptose-7-phosphate kinase/D-glycero-beta-D-manno-heptose 1-phosphate adenylyltransferase HldE [Pantoea sp. ARC607]	2027922	d__Bacteria;k__norank;p__Proteobacteria;c__Gammaproteobacteria;o__Enterobacterales;f__Erwiniaceae;g__Pantoea;s__Pantoea_sp._ARC607
PB2_gene_1577	203	PCG11334.1	195	194/195(99.49)	1.14e-132	threonylcarbamoyl-AMP synthase [Wolbachia pipientis]	None	Unclassified
PB2_gene_1583	799	WP_111140863.1	801	771/799(96.50)	0	maltodextrin phosphorylase [Pantoea sp. ARC607]	2027922	d__Bacteria;k__norank;p__Proteobacteria;c__Gammaproteobacteria;o__Enterobacterales;f__Erwiniaceae;g__Pantoea;s__Pantoea_sp._ARC607
PB2_gene_1585	273	WP_111140883.1	273	268/273(98.17)	8.21e-199	rhomboid family intramembrane serine protease GlpG [Pantoea sp. ARC607]	2027922	d__Bacteria;k__norank;p__Proteobacteria;c__Gammaproteobacteria;o__Enterobacterales;f__Erwiniaceae;g__Pantoea;s__Pantoea_sp._ARC607
PB2_gene_1586	108	ORM76053.1	108	105/108(97.22)	4.54e-70	thiosulfate sulfurtransferase [Pantoea deleyi]	470932	d__Bacteria;k__norank;p__Proteobacteria;c__Gammaproteobacteria;o__Enterobacterales;f__Erwiniaceae;g__Pantoea;s__Pantoea_deleyi

PB2_gene_1587	61	WP_128086738.1	61	59/61(96.72)	2.72e-34	hypothetical protein [Pantoea	None	Unclassified
PB2_gene_1588	501	ORM76052.1	501	499/501(99.60)	0	glycerol-3-phosphate dehydrogenase [Pantoea deleyi]	470932	d__Bacteria;k__norank;p__Proteobacteria;c__Gammaproteobacteria;o__Enterobacterales;f__Erwiniaceae;g__Pantoea;s__Pantoea_deleyi
PB2_gene_1589	560	ORM80843.1	675	556/560(99.29)	0	PTS N-acetyl glucosamine transporter subunit IIABC [Pantoea deleyi]	470932	d__Bacteria;k__norank;p__Proteobacteria;c__Gammaproteobacteria;o__Enterobacterales;f__Erwiniaceae;g__Pantoea;s__Pantoea_deleyi
PB2_gene_1591	382	ORM80841.1	382	379/382(99.21)	2.92e-272	N-acetylglucosamine-6-phosphate deacetylase [Pantoea deleyi]	470932	d__Bacteria;k__norank;p__Proteobacteria;c__Gammaproteobacteria;o__Enterobacterales;f__Erwiniaceae;g__Pantoea;s__Pantoea_deleyi
PB2_gene_1595	320	ORM81977.1	320	320/320(100.00)	8.42e-227	putrescine ABC transporter permease PotH [Pantoea deleyi]	470932	d__Bacteria;k__norank;p__Proteobacteria;c__Gammaproteobacteria;o__Enterobacterales;f__Erwiniaceae;g__Pantoea;s__Pantoea_deleyi
PB2_gene_1597	155	ORM81975.1	155	152/155(98.06)	2.83e-100	hypothetical protein HA42_09275 [Pantoea deleyi]	470932	d__Bacteria;k__norank;p__Proteobacteria;c__Gammaproteobacteria;o__Enterobacterales;f__Erwiniaceae;g__Pantoea;s__Pantoea_deleyi
PB2_gene_1599	243	ORM81973.1	243	239/243(98.35)	1.02e-168	arginine ABC transporter substrate-binding protein	470932	d__Bacteria;k__norank;p__Proteobacteria;c__Gammaproteobacteria;o__Enterobacterales;f__Erwiniaceae;g__Pantoea;s__Pantoea_deleyi
PB2_gene_1601	328	WP_111139483.1	353	321/328(97.87)	8.02e-226	TIGR01620 family protein [Pantoea sp. ARC607]	2027922	d__Bacteria;k__norank;p__Proteobacteria;c__Gammaproteobacteria;o__Enterobacterales;f__Erwiniaceae;g__Pantoea;s__Pantoea_sp._ARC607
PB2_gene_1602	472	WP_083067955.1	472	396/471(84.08)	5.47e-286	hypothetical protein [Pantoea vagans]	470934	d__Bacteria;k__norank;p__Proteobacteria;c__Gammaproteobacteria;o__Enterobacterales;f__Erwiniaceae;g__Pantoea;s__Pantoea_vagans
PB2_gene_1604	170	KDA93129.1	158	125/155(80.65)	2.36e-83	polysaccharide synthesis protein GtrA [Pantoea agglomerans	1408177	d__Bacteria;k__norank;p__Proteobacteria;c__Gammaproteobacteria;o__Enterobacterales;f__Erwiniaceae;g__Pantoea;s__Pantoea_agglomerans
PB2_gene_1606	177	ORM78917.1	167	167/167(100.00)	3.30e-112	lipid hydroperoxide peroxidase [Pantoea deleyi]	470932	d__Bacteria;k__norank;p__Proteobacteria;c__Gammaproteobacteria;o__Enterobacterales;f__Erwiniaceae;g__Pantoea;s__Pantoea_deleyi
PB2_gene_1608	233	WP_111139387.1	233	230/233(98.71)	6.38e-158	thiamine ABC transporter ATP-binding protein ThiQ [Pantoea sp. ARC607]	2027922	d__Bacteria;k__norank;p__Proteobacteria;c__Gammaproteobacteria;o__Enterobacterales;f__Erwiniaceae;g__Pantoea;s__Pantoea_sp._ARC607
PB2_gene_1609	528	WP_111139388.1	536	510/528(96.59)	0	thiamine/thiamine pyrophosphate ABC transporter permease ThiP [Pantoea sp. ARC607]	2027922	d__Bacteria;k__norank;p__Proteobacteria;c__Gammaproteobacteria;o__Enterobacterales;f__Erwiniaceae;g__Pantoea;s__Pantoea_sp._ARC607
PB2_gene_1611	555	ORM85265.1	555	549/555(98.92)	0	transcriptional regulator SgrR [Pantoea deleyi]	470932	d__Bacteria;k__norank;p__Proteobacteria;c__Gammaproteobacteria;o__Enterobacterales;f__Erwiniaceae;g__Pantoea;s__Pantoea_deleyi
PB2_gene_1614	1843	ORM86316.1	1843	1830/1843(99.29)	0	glutamate synthase large subunit [Pantoea deleyi]	470932	d__Bacteria;k__norank;p__Proteobacteria;c__Gammaproteobacteria;o__Enterobacterales;f__Erwiniaceae;g__Pantoea;s__Pantoea_deleyi
PB2_gene_1617	891	WP_033763528.1	887	788/887(88.84)	0	type VI secretion system ATPase TssH [Pantoea agglomerans]	549	d__Bacteria;k__norank;p__Proteobacteria;c__Gammaproteobacteria;o__Enterobacterales;f__Erwiniaceae;g__Pantoea;s__Pantoea_agglomerans
PB2_gene_1632	662	WP_111141578.1	665	637/663(96.08)	0	chemotaxis protein CheA [Pantoea sp. ARC607]	2027922	d__Bacteria;k__norank;p__Proteobacteria;c__Gammaproteobacteria;o__Enterobacterales;f__Erwiniaceae;g__Pantoea;s__Pantoea_sp._ARC607
PB2_gene_1634	282	ORM82567.1	295	282/282(100.00)	1.93e-191	flagellar motor stator protein MotA [Pantoea deleyi]	470932	d__Bacteria;k__norank;p__Proteobacteria;c__Gammaproteobacteria;o__Enterobacterales;f__Erwiniaceae;g__Pantoea;s__Pantoea_deleyi
PB2_gene_1638	488	WP_111139159.1	488	487/488(99.80)	0	IMP dehydrogenase [Pantoea sp. ARC607]	2027922	d__Bacteria;k__norank;p__Proteobacteria;c__Gammaproteobacteria;o__Enterobacterales;f__Erwiniaceae;g__Pantoea;s__Pantoea_sp._ARC607
PB2_gene_1641	110	WP_111139157.1	110	104/110(94.55)	1.06e-69	hypothetical protein [Pantoea sp. ARC607]	2027922	d__Bacteria;k__norank;p__Proteobacteria;c__Gammaproteobacteria;o__Enterobacterales;f__Erwiniaceae;g__Pantoea;s__Pantoea_sp._ARC607
PB2_gene_1644	369	ORM84719.1	369	354/369(95.93)	6.28e-265	hypothetical protein HA42_04055 [Pantoea deleyi]	470932	d__Bacteria;k__norank;p__Proteobacteria;c__Gammaproteobacteria;o__Enterobacterales;f__Erwiniaceae;g__Pantoea;s__Pantoea_deleyi
PB2_gene_1646	336	WP_111138784.1	336	331/336(98.51)	9.25e-239	quinone-dependent dihydroorotate dehydrogenase [Pantoea sp. ARC607]	2027922	d__Bacteria;k__norank;p__Proteobacteria;c__Gammaproteobacteria;o__Enterobacterales;f__Erwiniaceae;g__Pantoea;s__Pantoea_sp._ARC607
PB2_gene_1651	361	WP_111140736.1	361	354/361(98.06)	2.79e-273	A/G-specific adenine glycosylase [Pantoea sp. ARC607]	2027922	d__Bacteria;k__norank;p__Proteobacteria;c__Gammaproteobacteria;o__Enterobacterales;f__Erwiniaceae;g__Pantoea;s__Pantoea_sp._ARC607
PB2_gene_1655	379	ORM76863.1	379	375/379(98.94)	2.44e-283	YggW family oxidoreductase [Pantoea deleyi]	470932	d__Bacteria;k__norank;p__Proteobacteria;c__Gammaproteobacteria;o__Enterobacterales;f__Erwiniaceae;g__Pantoea;s__Pantoea_deleyi

PB2_gene_1658	495	WP_111139581.1	495	493/495(99.60)	0	transcription termination/antitermination protein NusA [Pantoea sp.	2027922	d__Bacteria;k__norank;p__Proteobacteria;c__Gammaproteobacteria;o__Enterobacterales;f__Erwiniaceae;g__Pantoea;s__Pantoea_sp._ARC607
PB2_gene_1665	246	ORM80776.1	246	245/246(99.59)	5.56e-188	pyruvate formate lyase-activating protein [Pantoea deleyi]	470932	d__Bacteria;k__norank;p__Proteobacteria;c__Gammaproteobacteria;o__Enterobacterales;f__Erwiniaceae;g__Pantoea;s__Pantoea_deleyi
PB2_gene_1666	760	WP_111138816.1	760	756/760(99.47)	0	formate C-acetyltransferase [Pantoea sp. ARC607]	2027922	d__Bacteria;k__norank;p__Proteobacteria;c__Gammaproteobacteria;o__Enterobacterales;f__Erwiniaceae;g__Pantoea;s__Pantoea_sp._ARC607
PB2_gene_1672	155	EAL60022.1	290	136/150(90.67)	1.78e-94	biotin-acetyl-CoA-carboxylase ligase [Wolbachia endosymbiont of Drosophila simulans]	77038	d__Bacteria;k__norank;p__Proteobacteria;c__Alphaproteobacteria;o__Rickettsiales;f__Anaplasmataceae;g__Wolbachia;s__Wolbachia_endosymbiont_of_Drosophila_simulans
PB2_gene_1682	332	EEB55842.1	332	317/332(95.48)	1.06e-226	murC-like protein [Wolbachia endosymbiont of Culex quinquefasciatus JHB]	569881	d__Bacteria;k__norank;p__Proteobacteria;c__Alphaproteobacteria;o__Rickettsiales;f__Anaplasmataceae;g__Wolbachia;s__Wolbachia_endosymbiont_of_Culex_quinquefasciatus
PB2_gene_1692	370	ORM83605.1	366	354/366(96.72)	1.72e-257	integrase [Pantoea deleyi]	470932	d__Bacteria;k__norank;p__Proteobacteria;c__Gammaproteobacteria;o__Enterobacterales;f__Erwiniaceae;g__Pantoea;s__Pantoea_deleyi
PB2_gene_1693	856	WP_087769488.1	889	807/856(94.28)	0	type VI secretion system ATPase TssH [Yersinia kristensenii]	28152	d__Bacteria;k__norank;p__Proteobacteria;c__Gammaproteobacteria;o__Enterobacterales;f__Yersiniaceae;g__Yersinia;s__Yersinia_kristensenii
PB2_gene_1699	202	WP_031590410.1	202	158/202(78.22)	3.41e-108	helix-turn-helix transcriptional regulator [Pantoea agglomerans]	549	d__Bacteria;k__norank;p__Proteobacteria;c__Gammaproteobacteria;o__Enterobacterales;f__Erwiniaceae;g__Pantoea;s__Pantoea_agglomerans
PB2_gene_1703	494	ORM84450.1	494	493/494(99.80)	0	D-xylose ABC transporter ATP-binding protein [Pantoea deleyi]	470932	d__Bacteria;k__norank;p__Proteobacteria;c__Gammaproteobacteria;o__Enterobacterales;f__Erwiniaceae;g__Pantoea;s__Pantoea_deleyi
PB2_gene_1704	350	SJZ60225.1	344	344/344(100.00)	2.30e-224	inositol transport system permease protein [Pantoea	470933	d__Bacteria;k__norank;p__Proteobacteria;c__Gammaproteobacteria;o__Enterobacterales;f__Erwiniaceae;g__Pantoea;s__Pantoea_eucalypti
PB2_gene_1724	163	WP_111141678.1	163	162/163(99.39)	2.69e-111	cell division inhibitor SulA [Pantoea sp. ARC607]	2027922	d__Bacteria;k__norank;p__Proteobacteria;c__Gammaproteobacteria;o__Enterobacterales;f__Erwiniaceae;g__Pantoea;s__Pantoea_sp._ARC607
PB2_gene_1742	221	WP_111140218.1	221	210/221(95.02)	1.92e-153	glutathione S-transferase [Pantoea sp. ARC607]	2027922	d__Bacteria;k__norank;p__Proteobacteria;c__Gammaproteobacteria;o__Enterobacterales;f__Erwiniaceae;g__Pantoea;s__Pantoea_sp._ARC607
PB2_gene_1743	449	WP_111140219.1	449	448/449(99.78)	1.95e-288	NCS2 family permease [Pantoea sp. ARC607]	2027922	d__Bacteria;k__norank;p__Proteobacteria;c__Gammaproteobacteria;o__Enterobacterales;f__Erwiniaceae;g__Pantoea;s__Pantoea_sp._ARC607
PB2_gene_1744	95	BAO21142.1	563	44/85(51.76)	3.57e-12	putative cation/proton antiporter [Serratia marcescens]	615	d__Bacteria;k__norank;p__Proteobacteria;c__Gammaproteobacteria;o__Enterobacterales;f__Yersiniaceae;g__Serratia;s__Serratia_marcescens
PB2_gene_1751	610	WP_111140827.1	613	587/610(96.23)	0	exodeoxyribonuclease V subunit alpha [Pantoea sp. ARC607]	2027922	d__Bacteria;k__norank;p__Proteobacteria;c__Gammaproteobacteria;o__Enterobacterales;f__Erwiniaceae;g__Pantoea;s__Pantoea_sp._ARC607
PB2_gene_1754	142	ORM81096.1	182	142/142(100.00)	2.09e-100	hypothetical protein HA42_11950 [Pantoea deleyi]	470932	d__Bacteria;k__norank;p__Proteobacteria;c__Gammaproteobacteria;o__Enterobacterales;f__Erwiniaceae;g__Pantoea;s__Pantoea_deleyi
PB2_gene_1756	396	ORM81098.1	396	394/396(99.49)	1.17e-285	aromatic amino acid aminotransferase [Pantoea deleyi]	470932	d__Bacteria;k__norank;p__Proteobacteria;c__Gammaproteobacteria;o__Enterobacterales;f__Erwiniaceae;g__Pantoea;s__Pantoea_deleyi
PB2_gene_1757	368	SUB17546.1	368	345/368(93.75)	1.11e-253	Porin OmpF [Pantoea agglomerans]	549	d__Bacteria;k__norank;p__Proteobacteria;c__Gammaproteobacteria;o__Enterobacterales;f__Erwiniaceae;g__Pantoea;s__Pantoea_agglomerans
PB2_gene_1761	1133	ORM85045.1	1133	1024/1133(90.38)	0	O-antigen biosynthesis protein [Pantoea deleyi]	470932	d__Bacteria;k__norank;p__Proteobacteria;c__Gammaproteobacteria;o__Enterobacterales;f__Erwiniaceae;g__Pantoea;s__Pantoea_deleyi
PB2_gene_1763	305	EIB96967.1	305	303/305(99.34)	2.12e-217	DNA-binding transcriptional activator GcvA [Pantoea sp. Sc1]	593105	d__Bacteria;k__norank;p__Proteobacteria;c__Gammaproteobacteria;o__Enterobacterales;f__Erwiniaceae;g__Pantoea;s__Pantoea_sp._Sc1
PB2_gene_1767	444	ORM80488.1	454	438/444(98.65)	0	LOG family protein [Pantoea deleyi]	470932	d__Bacteria;k__norank;p__Proteobacteria;c__Gammaproteobacteria;o__Enterobacterales;f__Erwiniaceae;g__Pantoea;s__Pantoea_deleyi
PB2_gene_1770	660	WP_111140968.1	660	658/660(99.70)	0	cytochrome o ubiquinol oxidase subunit I [Pantoea sp. ARC607]	2027922	d__Bacteria;k__norank;p__Proteobacteria;c__Gammaproteobacteria;o__Enterobacterales;f__Erwiniaceae;g__Pantoea;s__Pantoea_sp._ARC607
PB2_gene_1773	295	ORM83150.1	295	295/295(100.00)	3.87e-199	protoheme IX farnesyltransferase [Pantoea deleyi]	470932	d__Bacteria;k__norank;p__Proteobacteria;c__Gammaproteobacteria;o__Enterobacterales;f__Erwiniaceae;g__Pantoea;s__Pantoea_deleyi
PB2_gene_1779	290	OQV39898.1	290	290/290(100.00)	2.45e-204	hypothetical protein BZ160_16105 [Pantoea vagans]	470934	d__Bacteria;k__norank;p__Proteobacteria;c__Gammaproteobacteria;o__Enterobacterales;f__Erwiniaceae;g__Pantoea;s__Pantoea_vagans

PB2_gene_1780	443	ORM77535.1	443	437/443(98.65)	0	ABC transporter substrate-binding protein [Pantoea deleyi]	470932	d__Bacteria;k__norank;p__Proteobacteria;c__Gammaproteobacteria;o__Enterobacterales;f__Erwiniaceae;g__Pantoea;s__Pantoea_deleyi
PB2_gene_1790	166	ORM86850.1	166	164/166(98.80)	4.27e-114	Free methionine-R-sulfoxide reductase [Pantoea deleyi]	470932	d__Bacteria;k__norank;p__Proteobacteria;c__Gammaproteobacteria;o__Enterobacterales;f__Erwiniaceae;g__Pantoea;s__Pantoea_deleyi
PB2_gene_1792	567	WP_111140486.1	567	558/567(98.41)	0	ribulokinase [Pantoea sp. ARC607]	2027922	d__Bacteria;k__norank;p__Proteobacteria;c__Gammaproteobacteria;o__Enterobacterales;f__Erwiniaceae;g__Pantoea;s__Pantoea_sp._ARC607
PB2_gene_1794	506	WP_111140484.1	506	502/506(99.21)	0	L-arabinose ABC transporter ATP-binding protein AraG [Pantoea sp. ARC607]	2027922	d__Bacteria;k__norank;p__Proteobacteria;c__Gammaproteobacteria;o__Enterobacterales;f__Erwiniaceae;g__Pantoea;s__Pantoea_sp._ARC607
PB2_gene_1798	39	EIB98472.1	39	31/39(79.49)	3.48e-10	hypothetical protein S7A_08160 [Pantoea sp. Sc1]	593105	d__Bacteria;k__norank;p__Proteobacteria;c__Gammaproteobacteria;o__Enterobacterales;f__Erwiniaceae;g__Pantoea;s__Pantoea_sp._Sc1
PB2_gene_1799	352	ORM82561.1	352	346/352(98.30)	3.91e-239	two-component system sensor histidine kinase BasS [Pantoea deleyi]	470932	d__Bacteria;k__norank;p__Proteobacteria;c__Gammaproteobacteria;o__Enterobacterales;f__Erwiniaceae;g__Pantoea;s__Pantoea_deleyi
PB2_gene_1801	542	ORM82563.1	542	524/542(96.68)	0	phosphoethanolamine transferase EptA [Pantoea deleyi]	470932	d__Bacteria;k__norank;p__Proteobacteria;c__Gammaproteobacteria;o__Enterobacterales;f__Erwiniaceae;g__Pantoea;s__Pantoea_deleyi
PB2_gene_1805	609	ORM85462.1	609	607/609(99.67)	0	glutamine--fructose-6-phosphate aminotransferase [Pantoea deleyi]	470932	d__Bacteria;k__norank;p__Proteobacteria;c__Gammaproteobacteria;o__Enterobacterales;f__Erwiniaceae;g__Pantoea;s__Pantoea_deleyi
PB2_gene_1806	456	WP_083070455.1	456	442/456(96.93)	9.09e-309	bifunctional UDP-N-acetylglucosamine diphosphorylase/glucosamine-1-phosphate N-acetyltransferase GlmU [Pantoea vagans]	470934	d__Bacteria;k__norank;p__Proteobacteria;c__Gammaproteobacteria;o__Enterobacterales;f__Erwiniaceae;g__Pantoea;s__Pantoea_vagans
PB2_gene_1807	137	ELP26146.1	137	137/137(100.00)	8.21e-89	ATP synthase epsilon chain [Pantoea agglomerans 299R]	1261128	d__Bacteria;k__norank;p__Proteobacteria;c__Gammaproteobacteria;o__Enterobacterales;f__Erwiniaceae;g__Pantoea;s__Pantoea_agglomerans
PB2_gene_1812	137	ORM80697.1	137	136/137(99.27)	1.26e-92	hypothetical protein HA42_13490 [Pantoea deleyi]	470932	d__Bacteria;k__norank;p__Proteobacteria;c__Gammaproteobacteria;o__Enterobacterales;f__Erwiniaceae;g__Pantoea;s__Pantoea_deleyi
PB2_gene_1815	399	ORM75470.1	399	391/399(97.99)	4.18e-276	exonuclease subunit SbcD [Pantoea deleyi]	470932	d__Bacteria;k__norank;p__Proteobacteria;c__Gammaproteobacteria;o__Enterobacterales;f__Erwiniaceae;g__Pantoea;s__Pantoea_deleyi
PB2_gene_1816	229	SJZ30085.1	229	229/229(100.00)	4.54e-165	two-component system, OmpR family, phosphate regulon response regulator PhoB [Pantoea agglomerans]	470933	d__Bacteria;k__norank;p__Proteobacteria;c__Gammaproteobacteria;o__Enterobacterales;f__Erwiniaceae;g__Pantoea;s__Pantoea_eucalypti
PB2_gene_1817	437	ORM75468.1	437	434/437(99.31)	0	two-component system sensor histidine kinase PhoR [Pantoea deleyi]	470932	d__Bacteria;k__norank;p__Proteobacteria;c__Gammaproteobacteria;o__Enterobacterales;f__Erwiniaceae;g__Pantoea;s__Pantoea_deleyi
PB2_gene_1818	317	ADO08551.1	317	291/317(91.80)	2.93e-205	Phosphate-binding protein pstS precursor (PBP) [Pantoea vagans C9-1]	712898	d__Bacteria;k__norank;p__Proteobacteria;c__Gammaproteobacteria;o__Enterobacterales;f__Erwiniaceae;g__Pantoea;s__Pantoea_vagans
PB2_gene_1820	593	ORM85235.1	593	577/593(97.30)	0	histidine kinase [Pantoea deleyi]	470932	d__Bacteria;k__norank;p__Proteobacteria;c__Gammaproteobacteria;o__Enterobacterales;f__Erwiniaceae;g__Pantoea;s__Pantoea_deleyi
PB2_gene_1821	526	WP_111138364.1	526	474/526(90.11)	0	GGDEF domain-containing protein [Pantoea sp. ARC607]	2027922	d__Bacteria;k__norank;p__Proteobacteria;c__Gammaproteobacteria;o__Enterobacterales;f__Erwiniaceae;g__Pantoea;s__Pantoea_sp._ARC607
PB2_gene_1831	322	ORM86806.1	322	319/322(99.07)	7.62e-240	tRNA 5-methoxyuridine(34)/uridine 5-oxyacetic acid(34) synthase	470932	d__Bacteria;k__norank;p__Proteobacteria;c__Gammaproteobacteria;o__Enterobacterales;f__Erwiniaceae;g__Pantoea;s__Pantoea_deleyi
PB2_gene_1836	246	SAJ34659.1	188	103/178(57.87)	3.84e-63	Uncharacterised protein [Enterobacter cloacae]	550	d__Bacteria;k__norank;p__Proteobacteria;c__Gammaproteobacteria;o__Enterobacterales;f__Enterobacteriaceae;g__Enterobacter;s__Enterobacter_cloacae
PB2_gene_1843	348	ORM80465.1	348	342/348(98.28)	1.02e-232	flagella biosynthesis regulator [Pantoea deleyi]	470932	d__Bacteria;k__norank;p__Proteobacteria;c__Gammaproteobacteria;o__Enterobacterales;f__Erwiniaceae;g__Pantoea;s__Pantoea_deleyi

PB2_gene_1846	305	ORM80743.1	305	305/305(100.00)	4.71e-230	DNA-binding transcriptional regulator AraC [Pantoea deleyi]	470932	d__Bacteria;k__norank;p__Proteobacteria;c__Gammaproteobacteria;o__Enterobacterales;f__Erwiniaceae;g__Pantoea;s__Pantoea_deleyi
PB2_gene_1849	373	WP_108701196.1	352	176/213(82.63)	1.83e-125	hypothetical protein [Phytobacter sp. SCO41]	1756993	d__Bacteria;k__norank;p__Proteobacteria;c__Gammaproteobacteria;o__Enterobacterales;f__Enterobacteriaceae;g__Phytobacter;s__Phytobacter_sp._SCO41
PB2_gene_1853	563	WP_082861194.1	559	524/536(97.76)	0	recombinase family protein [Wolbachia endosymbiont of Dactylopius coccus]	None	Unclassified
PB2_gene_1854	398	WP_065095175.1	398	375/398(94.22)	9.93e-279	MULTISPECIES: hypothetical protein [Wolbachia]	2640676	d__Bacteria;k__norank;p__Proteobacteria;c__Alphaproteobacteria;o__Rickettsiales;f__Anaplasmataceae;g__Wolbachia;s__norank
PB2_gene_1864	328	WP_010404031.1	328	327/328(99.70)	2.64e-234	MULTISPECIES: tyrosine recombinase XerD [Wolbachia]	180837	d__Bacteria;k__norank;p__Proteobacteria;c__Alphaproteobacteria;o__Rickettsiales;f__Anaplasmataceae;g__Wolbachia;s__Wolbachia_endosymbiont_of_Nasonia_vitripennis
PB2_gene_1866	211	WP_017531897.1	211	206/211(97.63)	2.83e-141	MULTISPECIES: chromosomal DNA replication initiator DnaA [Wolbachia]	2640676	d__Bacteria;k__norank;p__Proteobacteria;c__Alphaproteobacteria;o__Rickettsiales;f__Anaplasmataceae;g__Wolbachia;s__norank
PB2_gene_1872	820	WP_111141367.1	820	808/820(98.54)	0	bifunctional aspartate kinase/homoserine dehydrogenase I [Pantoea sp. ARC607]	2027922	d__Bacteria;k__norank;p__Proteobacteria;c__Gammaproteobacteria;o__Enterobacterales;f__Erwiniaceae;g__Pantoea;s__Pantoea_sp._ARC607
PB2_gene_1875	218	ORM83254.1	218	207/218(94.95)	7.69e-138	NAD-dependent dehydratase [Pantoea deleyi]	470932	d__Bacteria;k__norank;p__Proteobacteria;c__Gammaproteobacteria;o__Enterobacterales;f__Erwiniaceae;g__Pantoea;s__Pantoea_deleyi
PB2_gene_1877	300	ORM83252.1	300	290/300(96.67)	4.76e-207	LysR family transcriptional regulator [Pantoea deleyi]	470932	d__Bacteria;k__norank;p__Proteobacteria;c__Gammaproteobacteria;o__Enterobacterales;f__Erwiniaceae;g__Pantoea;s__Pantoea_deleyi
PB2_gene_1899	249	WP_111142292.1	249	245/249(98.39)	3.31e-177	tRNA1(Val) (adenine(37)-N6)-methyltransferase [Pantoea sp. ARC607]	2027922	d__Bacteria;k__norank;p__Proteobacteria;c__Gammaproteobacteria;o__Enterobacterales;f__Erwiniaceae;g__Pantoea;s__Pantoea_sp._ARC607
PB2_gene_1901	963	WP_111140829.1	963	935/963(97.09)	0	pitrilysin [Pantoea sp. ARC607]	2027922	d__Bacteria;k__norank;p__Proteobacteria;c__Gammaproteobacteria;o__Enterobacterales;f__Erwiniaceae;g__Pantoea;s__Pantoea_sp._ARC607
PB2_gene_1907	172	ORM80534.1	333	172/172(100.00)	1.01e-117	NADPH:quinone reductase [Pantoea deleyi]	470932	d__Bacteria;k__norank;p__Proteobacteria;c__Gammaproteobacteria;o__Enterobacterales;f__Erwiniaceae;g__Pantoea;s__Pantoea_deleyi
PB2_gene_1908	413	ORM80535.1	413	412/413(99.76)	1.49e-308	trifunctional nicotinamide-nucleotide adenylyltransferase/ribosylnicotinamide kinase/transcriptional	470932	d__Bacteria;k__norank;p__Proteobacteria;c__Gammaproteobacteria;o__Enterobacterales;f__Erwiniaceae;g__Pantoea;s__Pantoea_deleyi
PB2_gene_1909	460	ORM80536.1	460	459/460(99.78)	0	DNA repair protein RadA [Pantoea deleyi]	470932	d__Bacteria;k__norank;p__Proteobacteria;c__Gammaproteobacteria;o__Enterobacterales;f__Erwiniaceae;g__Pantoea;s__Pantoea_deleyi
PB2_gene_1914	325	ORM80551.1	325	324/325(99.69)	6.98e-233	23S rRNA pseudouridine(1911/1915/1917) synthase [Pantoea deleyi]	470932	d__Bacteria;k__norank;p__Proteobacteria;c__Gammaproteobacteria;o__Enterobacterales;f__Erwiniaceae;g__Pantoea;s__Pantoea_deleyi
PB2_gene_1915	242	WP_111141461.1	242	224/242(92.56)	1.29e-166	polyphenol oxidase [Pantoea sp. ARC607]	2027922	d__Bacteria;k__norank;p__Proteobacteria;c__Gammaproteobacteria;o__Enterobacterales;f__Erwiniaceae;g__Pantoea;s__Pantoea_sp._ARC607
PB2_gene_1916	857	ORM80550.1	857	854/857(99.65)	0	ATP-dependent chaperone ClpB [Pantoea deleyi]	470932	d__Bacteria;k__norank;p__Proteobacteria;c__Gammaproteobacteria;o__Enterobacterales;f__Erwiniaceae;g__Pantoea;s__Pantoea_deleyi
PB2_gene_1922	270	WP_111138715.1	260	237/260(91.15)	5.27e-173	thiamine kinase [Pantoea sp. ARC607]	2027922	d__Bacteria;k__norank;p__Proteobacteria;c__Gammaproteobacteria;o__Enterobacterales;f__Erwiniaceae;g__Pantoea;s__Pantoea_sp._ARC607
PB2_gene_1924	122	ORM82839.1	122	121/122(99.18)	8.98e-79	hypothetical protein HA42_07525 [Pantoea deleyi]	470932	d__Bacteria;k__norank;p__Proteobacteria;c__Gammaproteobacteria;o__Enterobacterales;f__Erwiniaceae;g__Pantoea;s__Pantoea_deleyi
PB2_gene_1925	116	WP_111138657.1	116	116/116(100.00)	6.73e-78	purine nucleoside phosphoramidase [Pantoea sp.	2027922	d__Bacteria;k__norank;p__Proteobacteria;c__Gammaproteobacteria;o__Enterobacterales;f__Erwiniaceae;g__Pantoea;s__Pantoea_sp._ARC607
PB2_gene_1942	409	WP_111138932.1	409	399/409(97.56)	2.32e-294	GTP-binding protein [Pantoea sp. ARC607]	2027922	d__Bacteria;k__norank;p__Proteobacteria;c__Gammaproteobacteria;o__Enterobacterales;f__Erwiniaceae;g__Pantoea;s__Pantoea_sp._ARC607
PB2_gene_1950	831	WP_111139218.1	831	828/831(99.64)	0	ribonuclease R [Pantoea sp. ARC607]	2027922	d__Bacteria;k__norank;p__Proteobacteria;c__Gammaproteobacteria;o__Enterobacterales;f__Erwiniaceae;g__Pantoea;s__Pantoea_sp._ARC607

PB2_gene_1960	347	AGJ99190.1	347	343/347(98.85)	7.72e-248	Phenylalanyl-tRNA synthetase alpha chain [Wolbachia endosymbiont of Drosophila	1236908	d__Bacteria;k__norank;p__Proteobacteria;c__Alphaproteobacteria;o__Rickettsiales;f__Anaplasmataceae;g__Wolbachia;s__Wolbachia_endosymbiont_of_Drosophila_simulans
PB2_gene_1961	278	SUB15777.1	616	277/278(99.64)	2.62e-215	NADH-quinone oxidoreductase subunit G [Pantoea agglomerans]	549	d__Bacteria;k__norank;p__Proteobacteria;c__Gammaproteobacteria;o__Enterobacterales;f__Erwiniaceae;g__Pantoea;s__Pantoea_agglomerans
PB2_gene_1965	225	ORM54845.1	225	225/225(100.00)	3.47e-167	NADH dehydrogenase [Pantoea conspicua]	472705	d__Bacteria;k__norank;p__Proteobacteria;c__Gammaproteobacteria;o__Enterobacterales;f__Erwiniaceae;g__Pantoea;s__Pantoea_conspicua
PB2_gene_1973	440	ORM85469.1	440	435/440(98.86)	0	xylose isomerase [Pantoea deleyi]	470932	d__Bacteria;k__norank;p__Proteobacteria;c__Gammaproteobacteria;o__Enterobacterales;f__Erwiniaceae;g__Pantoea;s__Pantoea_deleyi
PB2_gene_1978	234	ORM86535.1	234	227/234(97.01)	4.06e-175	deoxyribonuclease I [Pantoea deleyi]	470932	d__Bacteria;k__norank;p__Proteobacteria;c__Gammaproteobacteria;o__Enterobacterales;f__Erwiniaceae;g__Pantoea;s__Pantoea_deleyi
PB2_gene_1980	314	ORM86533.1	314	313/314(99.68)	2.61e-227	glutathione synthase [Pantoea deleyi]	470932	d__Bacteria;k__norank;p__Proteobacteria;c__Gammaproteobacteria;o__Enterobacterales;f__Erwiniaceae;g__Pantoea;s__Pantoea_deleyi
PB2_gene_1981	187	ORM86532.1	187	187/187(100.00)	3.69e-132	hypothetical protein HA42_00400 [Pantoea deleyi]	470932	d__Bacteria;k__norank;p__Proteobacteria;c__Gammaproteobacteria;o__Enterobacterales;f__Erwiniaceae;g__Pantoea;s__Pantoea_deleyi
PB2_gene_1982	139	ORM86531.1	139	139/139(100.00)	5.31e-95	Holliday junction DNA helicase RuvA [Pantoea deleyi]	470932	d__Bacteria;k__norank;p__Proteobacteria;c__Gammaproteobacteria;o__Enterobacterales;f__Erwiniaceae;g__Pantoea;s__Pantoea_deleyi
PB2_gene_1984	384	ORM75941.1	400	380/384(98.96)	2.51e-287	glycosyl hydrolase [Pantoea deleyi]	470932	d__Bacteria;k__norank;p__Proteobacteria;c__Gammaproteobacteria;o__Enterobacterales;f__Erwiniaceae;g__Pantoea;s__Pantoea_deleyi
PB2_gene_1986	298	ORM75942.1	294	288/294(97.96)	3.93e-204	dihydrodipicolinate synthase family protein [Pantoea deleyi]	470932	d__Bacteria;k__norank;p__Proteobacteria;c__Gammaproteobacteria;o__Enterobacterales;f__Erwiniaceae;g__Pantoea;s__Pantoea_deleyi
PB2_gene_1993	269	WP_111140249.1	269	267/269(99.26)	4.20e-190	tetratricopeptide repeat-containing protein [Pantoea sp.	2027922	d__Bacteria;k__norank;p__Proteobacteria;c__Gammaproteobacteria;o__Enterobacterales;f__Erwiniaceae;g__Pantoea;s__Pantoea_sp._ARC607
PB2_gene_1998	334	ORM81338.1	294	292/293(99.66)	2.81e-195	multidrug DMT transporter permease [Pantoea deleyi]	470932	d__Bacteria;k__norank;p__Proteobacteria;c__Gammaproteobacteria;o__Enterobacterales;f__Erwiniaceae;g__Pantoea;s__Pantoea_deleyi
PB2_gene_2000	188	ORM81337.1	188	180/188(95.74)	1.50e-127	molecular chaperone [Pantoea deleyi]	470932	d__Bacteria;k__norank;p__Proteobacteria;c__Gammaproteobacteria;o__Enterobacterales;f__Erwiniaceae;g__Pantoea;s__Pantoea_deleyi
PB2_gene_2001	245	ORM81334.1	245	242/245(98.78)	1.78e-179	phosphatase [Pantoea deleyi]	470932	d__Bacteria;k__norank;p__Proteobacteria;c__Gammaproteobacteria;o__Enterobacterales;f__Erwiniaceae;g__Pantoea;s__Pantoea_deleyi
PB2_gene_2008	440	WP_111140195.1	440	432/440(98.18)	3.32e-296	MATE family efflux transporter DinF [Pantoea sp. ARC607]	2027922	d__Bacteria;k__norank;p__Proteobacteria;c__Gammaproteobacteria;o__Enterobacterales;f__Erwiniaceae;g__Pantoea;s__Pantoea_sp._ARC607
PB2_gene_2011	324	WP_111139333.1	343	316/324(97.53)	4.51e-224	outer membrane protein assembly factor BamC [Pantoea sp.	2027922	d__Bacteria;k__norank;p__Proteobacteria;c__Gammaproteobacteria;o__Enterobacterales;f__Erwiniaceae;g__Pantoea;s__Pantoea_sp._ARC607
PB2_gene_2012	280	WP_111139332.1	292	279/280(99.64)	1.81e-197	4-hydroxy-tetrahydrodipicolinate synthase [Pantoea sp. ARC607]	2027922	d__Bacteria;k__norank;p__Proteobacteria;c__Gammaproteobacteria;o__Enterobacterales;f__Erwiniaceae;g__Pantoea;s__Pantoea_sp._ARC607
PB2_gene_2013	231	ORM77014.1	231	227/231(98.27)	2.23e-157	glycine cleavage system transcriptional repressor [Pantoea deleyi]	470932	d__Bacteria;k__norank;p__Proteobacteria;c__Gammaproteobacteria;o__Enterobacterales;f__Erwiniaceae;g__Pantoea;s__Pantoea_deleyi
PB2_gene_2017	375	WP_111138402.1	378	357/373(95.71)	3.76e-263	Fic family protein [Pantoea sp. ARC607]	2027922	d__Bacteria;k__norank;p__Proteobacteria;c__Gammaproteobacteria;o__Enterobacterales;f__Erwiniaceae;g__Pantoea;s__Pantoea_sp._ARC607
PB2_gene_2021	285	WP_111138980.1	285	284/285(99.65)	1.84e-192	co-chaperone YbbN [Pantoea sp. ARC607]	2027922	d__Bacteria;k__norank;p__Proteobacteria;c__Gammaproteobacteria;o__Enterobacterales;f__Erwiniaceae;g__Pantoea;s__Pantoea_sp._ARC607
PB2_gene_2022	304	ORM85851.1	304	304/304(100.00)	6.88e-163	paraslipin [Pantoea deleyi]	470932	d__Bacteria;k__norank;p__Proteobacteria;c__Gammaproteobacteria;o__Enterobacterales;f__Erwiniaceae;g__Pantoea;s__Pantoea_deleyi
PB2_gene_2029	262	ORM82556.1	262	262/262(100.00)	3.57e-183	hypothetical protein HA42_07980 [Pantoea deleyi]	470932	d__Bacteria;k__norank;p__Proteobacteria;c__Gammaproteobacteria;o__Enterobacterales;f__Erwiniaceae;g__Pantoea;s__Pantoea_deleyi
PB2_gene_2030	425	WP_111141949.1	425	418/425(98.35)	1.98e-310	deferrochelatase/peroxidase EfeB [Pantoea sp. ARC607]	2027922	d__Bacteria;k__norank;p__Proteobacteria;c__Gammaproteobacteria;o__Enterobacterales;f__Erwiniaceae;g__Pantoea;s__Pantoea_sp._ARC607
PB2_gene_2031	368	ORM82553.1	371	357/368(97.01)	2.19e-247	iron uptake system protein EfeO [Pantoea deleyi]	470932	d__Bacteria;k__norank;p__Proteobacteria;c__Gammaproteobacteria;o__Enterobacterales;f__Erwiniaceae;g__Pantoea;s__Pantoea_deleyi

PB2_gene_2035	396	WP_111139838.1	396	384/396(96.97)	1.61e-267	MFS transporter [Pantoea sp. ARC607]	2027922	d__Bacteria;k__norank;p__Proteobacteria;c__Gammaproteobacteria;o__Enterobacterales;f__Erwiniaceae;g__Pantoea;s__Pantoea_sp._ARC607
PB2_gene_2043	233	WP_111139543.1	233	216/233(92.70)	3.16e-158	thiaminase II [Pantoea sp. ARC607]	2027922	d__Bacteria;k__norank;p__Proteobacteria;c__Gammaproteobacteria;o__Enterobacterales;f__Erwiniaceae;g__Pantoea;s__Pantoea_sp._ARC607
PB2_gene_2055	360	ORM85278.1	360	360/360(100.00)	1.12e-258	phospho-N-acetylmuramoyl-pentapeptide-transferase [Pantoea deleyi]	470932	d__Bacteria;k__norank;p__Proteobacteria;c__Gammaproteobacteria;o__Enterobacterales;f__Erwiniaceae;g__Pantoea;s__Pantoea_deleyi
PB2_gene_2056	438	ORM85279.1	438	433/438(98.86)	3.45e-313	UDP-N-acetylmuramoyl-L-alanine--D-glutamate ligase	470932	d__Bacteria;k__norank;p__Proteobacteria;c__Gammaproteobacteria;o__Enterobacterales;f__Erwiniaceae;g__Pantoea;s__Pantoea_deleyi
PB2_gene_2060	400	ORM77077.1	400	385/400(96.25)	2.61e-259	Bcr/CflA family drug resistance efflux transporter [Pantoea	470932	d__Bacteria;k__norank;p__Proteobacteria;c__Gammaproteobacteria;o__Enterobacterales;f__Erwiniaceae;g__Pantoea;s__Pantoea_deleyi
PB2_gene_2079	285	WP_111140750.1	285	277/285(97.19)	5.76e-180	SDR family oxidoreductase [Pantoea sp. ARC607]	53335	d__Bacteria;k__norank;p__Proteobacteria;c__Gammaproteobacteria;o__Enterobacterales;f__Erwiniaceae;g__Pantoea;s__norank
PB2_gene_2080	519	ORM79150.1	552	519/519(100.00)	0	alpha,alpha-trehalase [Pantoea deleyi]	470932	d__Bacteria;k__norank;p__Proteobacteria;c__Gammaproteobacteria;o__Enterobacterales;f__Erwiniaceae;g__Pantoea;s__Pantoea_deleyi
PB2_gene_2083	455	ORM80867.1	455	453/454(99.78)	0	two-component system sensor histidine kinase EnvZ [Pantoea deleyi]	470932	d__Bacteria;k__norank;p__Proteobacteria;c__Gammaproteobacteria;o__Enterobacterales;f__Erwiniaceae;g__Pantoea;s__Pantoea_deleyi
PB2_gene_2085	82	ORM80865.1	82	28/28(100.00)	1.38e-06	hypothetical protein HA42_12610 [Pantoea deleyi]	470932	d__Bacteria;k__norank;p__Proteobacteria;c__Gammaproteobacteria;o__Enterobacterales;f__Erwiniaceae;g__Pantoea;s__Pantoea_deleyi
PB2_gene_2090	213	WP_111140463.1	213	210/213(98.59)	7.86e-149	glutathione transferase [Pantoea sp. ARC607]	2027922	d__Bacteria;k__norank;p__Proteobacteria;c__Gammaproteobacteria;o__Enterobacterales;f__Erwiniaceae;g__Pantoea;s__Pantoea_sp._ARC607
PB2_gene_2091	299	ORM83871.1	298	292/296(98.65)	1.75e-215	TIGR01777 family protein [Pantoea deleyi]	470932	d__Bacteria;k__norank;p__Proteobacteria;c__Gammaproteobacteria;o__Enterobacterales;f__Erwiniaceae;g__Pantoea;s__Pantoea_deleyi
PB2_gene_2098	196	ADO10355.1	194	175/193(90.67)	3.38e-132	Uncharacterized protein ykkB [Pantoea vagans C9-1]	712898	d__Bacteria;k__norank;p__Proteobacteria;c__Gammaproteobacteria;o__Enterobacterales;f__Erwiniaceae;g__Pantoea;s__Pantoea_vagans
PB2_gene_2099	410	ORM77371.1	410	407/410(99.27)	1.04e-302	alanine transaminase [Pantoea deleyi]	470932	d__Bacteria;k__norank;p__Proteobacteria;c__Gammaproteobacteria;o__Enterobacterales;f__Erwiniaceae;g__Pantoea;s__Pantoea_deleyi
PB2_gene_2107	81	ORM81046.1	81	81/81(100.00)	4.39e-51	DNA damage-inducible protein I [Pantoea deleyi]	470932	d__Bacteria;k__norank;p__Proteobacteria;c__Gammaproteobacteria;o__Enterobacterales;f__Erwiniaceae;g__Pantoea;s__Pantoea_deleyi
PB2_gene_2112	176	ORM80847.1	176	176/176(100.00)	7.28e-129	flavodoxin [Pantoea deleyi]	470932	d__Bacteria;k__norank;p__Proteobacteria;c__Gammaproteobacteria;o__Enterobacterales;f__Erwiniaceae;g__Pantoea;s__Pantoea_deleyi
PB2_gene_2125	125	ORM79486.1	113	106/113(93.81)	4.20e-67	arsenate reductase (glutaredoxin) [Pantoea deleyi]	470932	d__Bacteria;k__norank;p__Proteobacteria;c__Gammaproteobacteria;o__Enterobacterales;f__Erwiniaceae;g__Pantoea;s__Pantoea_deleyi
PB2_gene_2126	249	SUB06377.1	270	246/249(98.80)	6.38e-180	DnaA-homolog protein hda [Pantoea agglomerans]	549	d__Bacteria;k__norank;p__Proteobacteria;c__Gammaproteobacteria;o__Enterobacterales;f__Erwiniaceae;g__Pantoea;s__Pantoea_agglomerans
PB2_gene_2128	243	WP_111139256.1	677	237/244(97.13)	1.83e-157	penicillin-binding protein activator [Pantoea sp. ARC607]	2027922	d__Bacteria;k__norank;p__Proteobacteria;c__Gammaproteobacteria;o__Enterobacterales;f__Erwiniaceae;g__Pantoea;s__Pantoea_sp._ARC607
PB2_gene_2131	190	WP_111139253.1	190	188/190(98.95)	7.31e-120	divisome-associated lipoprotein YraP [Pantoea sp. ARC607]	2027922	d__Bacteria;k__norank;p__Proteobacteria;c__Gammaproteobacteria;o__Enterobacterales;f__Erwiniaceae;g__Pantoea;s__Pantoea_sp._ARC607
PB2_gene_2136	238	ORM84003.1	238	238/238(100.00)	2.16e-172	two-component system response regulator ArcA [Pantoea deleyi]	470932	d__Bacteria;k__norank;p__Proteobacteria;c__Gammaproteobacteria;o__Enterobacterales;f__Erwiniaceae;g__Pantoea;s__Pantoea_deleyi
PB2_gene_2144	240	ORM78249.1	240	229/240(95.42)	1.39e-160	GntR family transcriptional regulator [Pantoea deleyi]	470932	d__Bacteria;k__norank;p__Proteobacteria;c__Gammaproteobacteria;o__Enterobacterales;f__Erwiniaceae;g__Pantoea;s__Pantoea_deleyi
PB2_gene_2145	243	WP_111140124.1	243	242/243(99.59)	4.66e-165	allantoin racemase [Pantoea sp. ARC607]	2027922	d__Bacteria;k__norank;p__Proteobacteria;c__Gammaproteobacteria;o__Enterobacterales;f__Erwiniaceae;g__Pantoea;s__Pantoea_sp._ARC607
PB2_gene_2150	878	WP_082721017.1	886	594/876(67.81)	0	fimbrial protein FimD [Burkholderia sp. ABCPW 14]	1637860	d__Bacteria;k__norank;p__Proteobacteria;c__Betaproteobacteria;o__Burkholderiales;f__Burkholderiaceae;g__Burkholderia;s__Burkholderia_sp._ABCPW_14
PB2_gene_2154	192	ORM59752.1	192	191/192(99.48)	6.31e-131	phosphoheptose isomerase [Pantoea brenneri]	472694	d__Bacteria;k__norank;p__Proteobacteria;c__Gammaproteobacteria;o__Enterobacterales;f__Erwiniaceae;g__Pantoea;s__Pantoea_brenneri

PB2_gene_2155	816	ORM80428.1	816	807/816(98.90)	0	acyl-CoA dehydrogenase [Pantoea deleyi]	470932	d__Bacteria;k__norank;p__Proteobacteria;c__Gammaproteobacteria;o__Enterobacterales;f__Erwiniaceae;g__Pantoea;s__Pantoea_deleyi
PB2_gene_2169	535	WP_111142230.1	535	530/535(99.07)	0	glutamate--cysteine ligase [Pantoea sp. ARC607]	2027922	d__Bacteria;k__norank;p__Proteobacteria;c__Gammaproteobacteria;o__Enterobacterales;f__Erwiniaceae;g__Pantoea;s__Pantoea_sp._ARC607
PB2_gene_2170	142	WP_111142231.1	142	140/142(98.59)	1.11e-93	DedA family protein [Pantoea sp. ARC607]	2027922	d__Bacteria;k__norank;p__Proteobacteria;c__Gammaproteobacteria;o__Enterobacterales;f__Erwiniaceae;g__Pantoea;s__Pantoea_sp._ARC607
PB2_gene_2178	309	PZL90030.1	309	309/309(100.00)	5.85e-217	RepB family plasmid replication initiator protein [Pantoea sp. ARC270]	2027923	d__Bacteria;k__norank;p__Proteobacteria;c__Gammaproteobacteria;o__Enterobacterales;f__Erwiniaceae;g__Pantoea;s__Pantoea_sp._ARC270
PB2_gene_2179	404	ERM09683.1	404	398/404(98.51)	7.34e-284	chromosome partitioning protein ParA [Pantoea agglomerans	1332072	d__Bacteria;k__norank;p__Proteobacteria;c__Gammaproteobacteria;o__Enterobacterales;f__Erwiniaceae;g__Pantoea;s__Pantoea_agglomerans
PB2_gene_2180	323	ORM72605.1	323	320/323(99.07)	2.68e-220	chromosome partitioning protein ParB [Pantoea deleyi]	470932	d__Bacteria;k__norank;p__Proteobacteria;c__Gammaproteobacteria;o__Enterobacterales;f__Erwiniaceae;g__Pantoea;s__Pantoea_deleyi
PB2_gene_2182	224	ORM81059.1	224	220/224(98.21)	1.13e-142	flagellar basal body rod modification protein [Pantoea	470932	d__Bacteria;k__norank;p__Proteobacteria;c__Gammaproteobacteria;o__Enterobacterales;f__Erwiniaceae;g__Pantoea;s__Pantoea_deleyi
PB2_gene_2185	219	WP_111138629.1	219	214/217(98.62)	3.57e-148	flagellar basal body P-ring formation protein FlgA [Pantoea	2027922	d__Bacteria;k__norank;p__Proteobacteria;c__Gammaproteobacteria;o__Enterobacterales;f__Erwiniaceae;g__Pantoea;s__Pantoea_sp._ARC607
PB2_gene_2187	143	ORM81054.1	143	141/143(98.60)	2.31e-91	flagellar biosynthesis protein FlgN [Pantoea deleyi]	470932	d__Bacteria;k__norank;p__Proteobacteria;c__Gammaproteobacteria;o__Enterobacterales;f__Erwiniaceae;g__Pantoea;s__Pantoea_deleyi
PB2_gene_2192	430	ORM81456.1	430	426/430(99.07)	9.78e-309	ATP-dependent RNA helicase RhlB [Pantoea deleyi]	470932	d__Bacteria;k__norank;p__Proteobacteria;c__Gammaproteobacteria;o__Enterobacterales;f__Erwiniaceae;g__Pantoea;s__Pantoea_deleyi
PB2_gene_2201	428	ORM83130.1	428	426/428(99.53)	2.87e-300	ammonium transporter [Pantoea deleyi]	470932	d__Bacteria;k__norank;p__Proteobacteria;c__Gammaproteobacteria;o__Enterobacterales;f__Erwiniaceae;g__Pantoea;s__Pantoea_deleyi
PB2_gene_2202	287	ORM83129.1	287	287/287(100.00)	2.36e-205	acyl-CoA thioesterase II [Pantoea deleyi]	470932	d__Bacteria;k__norank;p__Proteobacteria;c__Gammaproteobacteria;o__Enterobacterales;f__Erwiniaceae;g__Pantoea;s__Pantoea_deleyi
PB2_gene_2208	575	WP_111142242.1	575	573/575(99.65)	0	phosphoenolpyruvate-protein phosphotransferase PtsI [Pantoea sp. ARC607]	2027922	d__Bacteria;k__norank;p__Proteobacteria;c__Gammaproteobacteria;o__Enterobacterales;f__Erwiniaceae;g__Pantoea;s__Pantoea_sp._ARC607
PB2_gene_2210	438	ORM80508.1	446	420/438(95.89)	3.09e-302	two-component sensor histidine kinase [Pantoea deleyi]	470932	d__Bacteria;k__norank;p__Proteobacteria;c__Gammaproteobacteria;o__Enterobacterales;f__Erwiniaceae;g__Pantoea;s__Pantoea_deleyi
PB2_gene_2230	299	ORM81964.1	299	292/299(97.66)	2.40e-195	hypothetical protein HA42_09215 [Pantoea deleyi]	470932	d__Bacteria;k__norank;p__Proteobacteria;c__Gammaproteobacteria;o__Enterobacterales;f__Erwiniaceae;g__Pantoea;s__Pantoea_deleyi
PB2_gene_2231	583	ORM81965.1	573	572/573(99.83)	0	pyruvate dehydrogenase [Pantoea deleyi]	470932	d__Bacteria;k__norank;p__Proteobacteria;c__Gammaproteobacteria;o__Enterobacterales;f__Erwiniaceae;g__Pantoea;s__Pantoea_deleyi
PB2_gene_2232	319	ORM81966.1	334	311/319(97.49)	1.52e-223	low-specificity L-threonine aldolase [Pantoea deleyi]	470932	d__Bacteria;k__norank;p__Proteobacteria;c__Gammaproteobacteria;o__Enterobacterales;f__Erwiniaceae;g__Pantoea;s__Pantoea_deleyi
PB2_gene_2234	412	ORM85827.1	412	412/412(100.00)	5.18e-256	AI-2E family transporter [Pantoea deleyi]	470932	d__Bacteria;k__norank;p__Proteobacteria;c__Gammaproteobacteria;o__Enterobacterales;f__Erwiniaceae;g__Pantoea;s__Pantoea_deleyi
PB2_gene_2235	108	ORM85826.1	108	103/108(95.37)	2.34e-63	hypothetical protein HA42_01855 [Pantoea deleyi]	470932	d__Bacteria;k__norank;p__Proteobacteria;c__Gammaproteobacteria;o__Enterobacterales;f__Erwiniaceae;g__Pantoea;s__Pantoea_deleyi
PB2_gene_2237	428	ORM85821.1	428	423/428(98.83)	1.98e-291	HAAAP family serine/threonine permease [Pantoea deleyi]	470932	d__Bacteria;k__norank;p__Proteobacteria;c__Gammaproteobacteria;o__Enterobacterales;f__Erwiniaceae;g__Pantoea;s__Pantoea_deleyi
PB2_gene_2242	242	ORM84915.1	242	242/242(100.00)	2.95e-172	DNA repair protein RecO [Pantoea deleyi]	470932	d__Bacteria;k__norank;p__Proteobacteria;c__Gammaproteobacteria;o__Enterobacterales;f__Erwiniaceae;g__Pantoea;s__Pantoea_deleyi
PB2_gene_2243	245	KDA92575.1	245	242/245(98.78)	4.16e-169	pyridoxine 5'-phosphate synthase [Pantoea agglomerans Eh318]	1408177	d__Bacteria;k__norank;p__Proteobacteria;c__Gammaproteobacteria;o__Enterobacterales;f__Erwiniaceae;g__Pantoea;s__Pantoea_agglomerans
PB2_gene_2248	213	WP_111138683.1	213	209/213(98.12)	7.46e-138	high frequency lysogenization protein HflD [Pantoea sp.	2027922	d__Bacteria;k__norank;p__Proteobacteria;c__Gammaproteobacteria;o__Enterobacterales;f__Erwiniaceae;g__Pantoea;s__Pantoea_sp._ARC607
PB2_gene_2249	456	ORM82375.1	456	455/456(99.78)	0	adenylosuccinate lyase [Pantoea deleyi]	470932	d__Bacteria;k__norank;p__Proteobacteria;c__Gammaproteobacteria;o__Enterobacterales;f__Erwiniaceae;g__Pantoea;s__Pantoea_deleyi

PB2_gene_2250	222	ORM82374.1	222	222/222(100.00)	4.36e-155	two-component system response regulator PhoP [Pantoea deleyi]	470932	d__Bacteria;k__norank;p__Proteobacteria;c__Gammaproteobacteria;o__Enterobacterales;f__Erwiniaceae;g__Pantoea;s__Pantoea_deleyi
PB2_gene_2279	371	WP_064085479.1	371	367/371(98.92)	4.55e-259	RIP metalloprotease RseP [Wolbachia endosymbiont of Dactylopius coccus]	None	Unclassified
PB2_gene_2280	787	WP_064085494.1	777	767/777(98.71)	0	outer membrane protein assembly factor BamA [Wolbachia endosymbiont of Dactylopius coccus]	None	Unclassified
PB2_gene_2289	321	KDA92559.1	321	318/321(99.07)	1.08e-234	inorganic polyphosphate/ATP-NAD kinase [Pantoea	1408177	d__Bacteria;k__norank;p__Proteobacteria;c__Gammaproteobacteria;o__Enterobacterales;f__Erwiniaceae;g__Pantoea;s__Pantoea_agglomerans
PB2_gene_2294	339	ORM80624.1	339	339/339(100.00)	6.47e-236	dipeptide ABC transporter permease DppB [Pantoea deleyi]	470932	d__Bacteria;k__norank;p__Proteobacteria;c__Gammaproteobacteria;o__Enterobacterales;f__Erwiniaceae;g__Pantoea;s__Pantoea_deleyi
PB2_gene_2299	234	SJZ80532.1	234	225/234(96.15)	6.41e-163	sugar fermentation stimulation protein A [Pantoea agglomerans]	470933	d__Bacteria;k__norank;p__Proteobacteria;c__Gammaproteobacteria;o__Enterobacterales;f__Erwiniaceae;g__Pantoea;s__Pantoea_eucalypti
PB2_gene_2315	214	WP_111139028.1	214	212/214(99.07)	1.08e-160	nicotinate-nucleotide adenylyltransferase [Pantoea sp. ARC607]	53335	d__Bacteria;k__norank;p__Proteobacteria;c__Gammaproteobacteria;o__Enterobacterales;f__Erwiniaceae;g__Pantoea;s__norank
PB2_gene_2316	343	WP_111139029.1	343	341/343(99.42)	7.07e-242	DNA polymerase III subunit delta [Pantoea sp. ARC607]	2027922	d__Bacteria;k__norank;p__Proteobacteria;c__Gammaproteobacteria;o__Enterobacterales;f__Erwiniaceae;g__Pantoea;s__Pantoea_sp._ARC607
PB2_gene_2317	189	ORM79042.1	199	183/189(96.83)	1.74e-122	LPS assembly lipoprotein LptE [Pantoea deleyi]	470932	d__Bacteria;k__norank;p__Proteobacteria;c__Gammaproteobacteria;o__Enterobacterales;f__Erwiniaceae;g__Pantoea;s__Pantoea_deleyi
PB2_gene_2324	138	KDA96013.1	702	121/129(93.80)	7.40e-82	hypothetical protein T296_05015 [Pantoea agglomerans Eh318]	1408177	d__Bacteria;k__norank;p__Proteobacteria;c__Gammaproteobacteria;o__Enterobacterales;f__Erwiniaceae;g__Pantoea;s__Pantoea_agglomerans
PB2_gene_2333	236	ORM86578.1	236	235/236(99.58)	2.88e-151	hypothetical protein HA42_00670 [Pantoea deleyi]	470932	d__Bacteria;k__norank;p__Proteobacteria;c__Gammaproteobacteria;o__Enterobacterales;f__Erwiniaceae;g__Pantoea;s__Pantoea_deleyi
PB2_gene_2335	336	PAW34285.1	336	332/335(99.10)	1.95e-240	catabolite repressor/activator [Pantoea vagans]	470934	d__Bacteria;k__norank;p__Proteobacteria;c__Gammaproteobacteria;o__Enterobacterales;f__Erwiniaceae;g__Pantoea;s__Pantoea_vagans
PB2_gene_2336	163	ORM85271.1	163	163/163(100.00)	1.18e-105	acetolactate synthase small subunit [Pantoea deleyi]	470932	d__Bacteria;k__norank;p__Proteobacteria;c__Gammaproteobacteria;o__Enterobacterales;f__Erwiniaceae;g__Pantoea;s__Pantoea_deleyi
PB2_gene_2337	574	WP_111139397.1	574	569/574(99.13)	0	acetolactate synthase 3 large subunit [Pantoea sp. ARC607]	2027922	d__Bacteria;k__norank;p__Proteobacteria;c__Gammaproteobacteria;o__Enterobacterales;f__Erwiniaceae;g__Pantoea;s__Pantoea_sp._ARC607
PB2_gene_2340	528	ORM80921.1	528	520/528(98.48)	0	peptide chain release factor 3 [Pantoea deleyi]	470932	d__Bacteria;k__norank;p__Proteobacteria;c__Gammaproteobacteria;o__Enterobacterales;f__Erwiniaceae;g__Pantoea;s__Pantoea_deleyi
PB2_gene_2342	53	SKA00820.1	53	53/53(100.00)	3.81e-24	Uncharacterized membrane protein YtjA, UPF0391 family [Pantoea agglomerans]	470933	d__Bacteria;k__norank;p__Proteobacteria;c__Gammaproteobacteria;o__Enterobacterales;f__Erwiniaceae;g__Pantoea;s__Pantoea_eucalypti
PB2_gene_2351	906	WP_109651142.1	900	879/900(97.67)	0	HTH-type transcriptional regulator MalT [Pantoea allii]	None	Unclassified
PB2_gene_2354	403	ORM81545.1	403	397/403(98.51)	6.23e-280	c-type cytochrome biogenesis protein CcmI [Pantoea deleyi]	470932	d__Bacteria;k__norank;p__Proteobacteria;c__Gammaproteobacteria;o__Enterobacterales;f__Erwiniaceae;g__Pantoea;s__Pantoea_deleyi
PB2_gene_2356	185	WP_111141421.1	185	179/184(97.28)	1.06e-130	DsbE family thiol:disulfide interchange protein [Pantoea sp. ARC607]	2027922	d__Bacteria;k__norank;p__Proteobacteria;c__Gammaproteobacteria;o__Enterobacterales;f__Erwiniaceae;g__Pantoea;s__Pantoea_sp._ARC607
PB2_gene_2357	652	ORM81543.1	652	640/652(98.16)	0	c-type cytochrome biogenesis protein CcmF [Pantoea deleyi]	470932	d__Bacteria;k__norank;p__Proteobacteria;c__Gammaproteobacteria;o__Enterobacterales;f__Erwiniaceae;g__Pantoea;s__Pantoea_deleyi
PB2_gene_2360	1516	WP_063631092.1	1489	497/1186(41.91)	9.31e-250	hypothetical protein [Wolbachia endosymbiont of Laodelphax striatella]	368602	d__Bacteria;k__norank;p__Proteobacteria;c__Alphaproteobacteria;o__Rickettsiales;f__Anaplasmataceae;g__Wolbachia;s__Wolbachia_endosymbiont_of_Laodelphax_striatellus
PB2_gene_2367	379	WP_111139194.1	379	371/377(98.41)	3.67e-269	polysaccharide export protein [Pantoea sp. ARC607]	2027922	d__Bacteria;k__norank;p__Proteobacteria;c__Gammaproteobacteria;o__Enterobacterales;f__Erwiniaceae;g__Pantoea;s__Pantoea_sp._ARC607

PB2_gene_2374	228	ORM85477.1	228	226/228(99.12)	9.13e-160	autotransporter [Pantoea deleyi]	470932	d__Bacteria;k__norank;p__Proteobacteria;c__Gammaproteobacteria;o__Enterobacterales;f__Erwiniaceae;g__Pantoea;s__Pantoea_deleyi
PB2_gene_2380	96	ORM80385.1	80	57/80(71.25)	1.39e-26	hypothetical protein HA42_14830 [Pantoea deleyi]	470932	d__Bacteria;k__norank;p__Proteobacteria;c__Gammaproteobacteria;o__Enterobacterales;f__Erwiniaceae;g__Pantoea;s__Pantoea_deleyi
PB2_gene_2382	613	ORM80383.1	613	611/613(99.67)	0	RNA polymerase sigma factor RpoD [Pantoea deleyi]	470932	d__Bacteria;k__norank;p__Proteobacteria;c__Gammaproteobacteria;o__Enterobacterales;f__Erwiniaceae;g__Pantoea;s__Pantoea_deleyi
PB2_gene_2391	155	WP_111138912.1	129	112/129(86.82)	1.97e-74	tautomerase family protein [Pantoea sp. ARC607]	2027922	d__Bacteria;k__norank;p__Proteobacteria;c__Gammaproteobacteria;o__Enterobacterales;f__Erwiniaceae;g__Pantoea;s__Pantoea_sp._ARC607
PB2_gene_2394	514	ORM78065.1	514	506/514(98.44)	0	nitrate reductase subunit beta [Pantoea deleyi]	470932	d__Bacteria;k__norank;p__Proteobacteria;c__Gammaproteobacteria;o__Enterobacterales;f__Erwiniaceae;g__Pantoea;s__Pantoea_deleyi
PB2_gene_2404	250	ORM85038.1	250	248/250(99.20)	5.02e-168	L-cystine ABC transporter ATP-binding protein YecC [Pantoea deleyi]	470932	d__Bacteria;k__norank;p__Proteobacteria;c__Gammaproteobacteria;o__Enterobacterales;f__Erwiniaceae;g__Pantoea;s__Pantoea_deleyi
PB2_gene_2405	222	WP_111139953.1	222	222/222(100.00)	7.45e-147	cystine ABC transporter permease [Pantoea sp. ARC607]	2027922	d__Bacteria;k__norank;p__Proteobacteria;c__Gammaproteobacteria;o__Enterobacterales;f__Erwiniaceae;g__Pantoea;s__Pantoea_sp._ARC607
PB2_gene_2406	266	WP_111139954.1	266	263/266(98.87)	1.20e-183	cystine ABC transporter substrate-binding protein [Pantoea sp. ARC607]	2027922	d__Bacteria;k__norank;p__Proteobacteria;c__Gammaproteobacteria;o__Enterobacterales;f__Erwiniaceae;g__Pantoea;s__Pantoea_sp._ARC607
PB2_gene_2407	328	WP_111139955.1	328	319/328(97.26)	1.57e-226	D-cysteine desulfhydrase [Pantoea sp. ARC607]	2027922	d__Bacteria;k__norank;p__Proteobacteria;c__Gammaproteobacteria;o__Enterobacterales;f__Erwiniaceae;g__Pantoea;s__Pantoea_sp._ARC607
PB2_gene_2413	396	WP_125289959.1	396	340/395(86.08)	2.28e-251	knotted carbamoyltransferase YgeW [Erwinia sp. 198]	2022746	d__Bacteria;k__norank;p__Proteobacteria;c__Gammaproteobacteria;o__Enterobacterales;f__Erwiniaceae;g__Erwinia;s__Erwinia_sp._198
PB2_gene_2414	603	WP_125289958.1	594	423/600(70.50)	2.91e-296	AAA family ATPase [Erwinia sp. 198]	2022746	d__Bacteria;k__norank;p__Proteobacteria;c__Gammaproteobacteria;o__Enterobacterales;f__Erwiniaceae;g__Erwinia;s__Erwinia_sp._198
PB2_gene_2415	194	WP_125289998.1	183	118/178(66.29)	9.92e-83	CTP--molybdopterin cytidylyltransferase [Erwinia sp.	2022746	d__Bacteria;k__norank;p__Proteobacteria;c__Gammaproteobacteria;o__Enterobacterales;f__Erwiniaceae;g__Erwinia;s__Erwinia_sp._198
PB2_gene_2419	302	ORM83991.1	302	297/302(98.34)	1.27e-212	transcriptional activator NhaR [Pantoea deleyi]	470932	d__Bacteria;k__norank;p__Proteobacteria;c__Gammaproteobacteria;o__Enterobacterales;f__Erwiniaceae;g__Pantoea;s__Pantoea_deleyi
PB2_gene_2423	428	WP_111138812.1	428	423/428(98.83)	1.97e-307	3-phosphoshikimate 1-carboxyvinyltransferase [Pantoea sp. ARC607]	2027922	d__Bacteria;k__norank;p__Proteobacteria;c__Gammaproteobacteria;o__Enterobacterales;f__Erwiniaceae;g__Pantoea;s__Pantoea_sp._ARC607
PB2_gene_2424	361	ORM80779.1	361	355/361(98.34)	7.59e-262	phosphoserine transaminase [Pantoea deleyi]	470932	d__Bacteria;k__norank;p__Proteobacteria;c__Gammaproteobacteria;o__Enterobacterales;f__Erwiniaceae;g__Pantoea;s__Pantoea_deleyi
PB2_gene_2429	109	PXW18719.1	109	107/108(99.07)	1.20e-70	small multidrug resistance family-3 protein [Pantoea sp.	1938795	d__Bacteria;k__norank;p__Proteobacteria;c__Gammaproteobacteria;o__Enterobacterales;f__Erwiniaceae;g__Pantoea;s__Pantoea_sp._JKS000250
PB2_gene_2430	266	ERM06985.1	266	254/266(95.49)	4.17e-184	malonic semialdehyde reductase [Pantoea agglomerans Tx10]	1332072	d__Bacteria;k__norank;p__Proteobacteria;c__Gammaproteobacteria;o__Enterobacterales;f__Erwiniaceae;g__Pantoea;s__Pantoea_agglomerans
PB2_gene_2431	454	ORM81894.1	442	427/442(96.61)	0	guanine deaminase [Pantoea deleyi]	470932	d__Bacteria;k__norank;p__Proteobacteria;c__Gammaproteobacteria;o__Enterobacterales;f__Erwiniaceae;g__Pantoea;s__Pantoea_deleyi
PB2_gene_2432	483	ORM81893.1	480	465/480(96.88)	0	xanthine dehydrogenase small subunit [Pantoea deleyi]	470932	d__Bacteria;k__norank;p__Proteobacteria;c__Gammaproteobacteria;o__Enterobacterales;f__Erwiniaceae;g__Pantoea;s__Pantoea_deleyi
PB2_gene_2435	303	WP_111138407.1	302	300/302(99.34)	1.88e-212	sugar ABC transporter permease [Pantoea sp. ARC607]	2027922	d__Bacteria;k__norank;p__Proteobacteria;c__Gammaproteobacteria;o__Enterobacterales;f__Erwiniaceae;g__Pantoea;s__Pantoea_sp._ARC607
PB2_gene_2436	279	ORM78400.1	279	277/279(99.28)	2.72e-191	sugar ABC transporter permease [Pantoea deleyi]	470932	d__Bacteria;k__norank;p__Proteobacteria;c__Gammaproteobacteria;o__Enterobacterales;f__Erwiniaceae;g__Pantoea;s__Pantoea_deleyi
PB2_gene_2437	315	WP_111138405.1	315	308/315(97.78)	1.20e-228	carbohydrate kinase family protein [Pantoea sp. ARC607]	2027922	d__Bacteria;k__norank;p__Proteobacteria;c__Gammaproteobacteria;o__Enterobacterales;f__Erwiniaceae;g__Pantoea;s__Pantoea_sp._ARC607
PB2_gene_2448	164	SJZ32588.1	164	164/164(100.00)	2.04e-111	transcriptional regulator, AsnC family [Pantoea agglomerans]	470933	d__Bacteria;k__norank;p__Proteobacteria;c__Gammaproteobacteria;o__Enterobacterales;f__Erwiniaceae;g__Pantoea;s__Pantoea_eucalypti
PB2_gene_2449	1225	WP_111138821.1	1222	1108/1235(89.72)	0	cell division protein FtsK [Pantoea sp. ARC607]	2027922	d__Bacteria;k__norank;p__Proteobacteria;c__Gammaproteobacteria;o__Enterobacterales;f__Erwiniaceae;g__Pantoea;s__Pantoea_sp._ARC607

PB2_gene_2450	215	WP_073970639.1	337	213/215(99.07)	1.30e-142	ribose-phosphate pyrophosphokinase [Pantoea agglomerans]	None	Unclassified
PB2_gene_2451	285	ORM83723.1	285	280/285(98.25)	2.17e-200	4-(cytidine 5'-diphospho)-2-C-methyl-D-erythritol kinase [Pantoea deleyi]	470932	d__Bacteria;k__norank;p__Proteobacteria;c__Gammaproteobacteria;o__Enterobacterales;f__Erwiniaceae;g__Pantoea;s__Pantoea_deleyi
PB2_gene_2458	291	ORM84161.1	290	287/290(98.97)	8.41e-194	hypothetical protein HA42_05050 [Pantoea deleyi]	470932	d__Bacteria;k__norank;p__Proteobacteria;c__Gammaproteobacteria;o__Enterobacterales;f__Erwiniaceae;g__Pantoea;s__Pantoea_deleyi
PB2_gene_2469	376	ORM81330.1	376	372/376(98.94)	2.06e-253	mechanosensitive ion channel protein MscS [Pantoea deleyi]	470932	d__Bacteria;k__norank;p__Proteobacteria;c__Gammaproteobacteria;o__Enterobacterales;f__Erwiniaceae;g__Pantoea;s__Pantoea_deleyi
PB2_gene_2481	97	ORM85301.1	81	67/81(82.72)	3.16e-21	hypothetical protein HA42_02875 [Pantoea deleyi]	470932	d__Bacteria;k__norank;p__Proteobacteria;c__Gammaproteobacteria;o__Enterobacterales;f__Erwiniaceae;g__Pantoea;s__Pantoea_deleyi
PB2_gene_2490	302	ORM82191.1	302	299/302(99.01)	1.68e-217	AraC family transcriptional regulator [Pantoea deleyi]	470932	d__Bacteria;k__norank;p__Proteobacteria;c__Gammaproteobacteria;o__Enterobacterales;f__Erwiniaceae;g__Pantoea;s__Pantoea_deleyi
PB2_gene_2493	809	WP_033763527.1	745	649/693(93.65)	0	type VI secretion system tip protein VgrG [Pantoea	549	d__Bacteria;k__norank;p__Proteobacteria;c__Gammaproteobacteria;o__Enterobacterales;f__Erwiniaceae;g__Pantoea;s__Pantoea_agglomerans
PB2_gene_2494	235	WP_031590421.1	229	164/227(72.25)	1.91e-122	hypothetical protein [Pantoea agglomerans]	549	d__Bacteria;k__norank;p__Proteobacteria;c__Gammaproteobacteria;o__Enterobacterales;f__Erwiniaceae;g__Pantoea;s__Pantoea_agglomerans
PB2_gene_2503	264	ORM76324.1	264	264/264(100.00)	7.59e-195	Sir2 family NAD-dependent protein deacetylase [Pantoea	470932	d__Bacteria;k__norank;p__Proteobacteria;c__Gammaproteobacteria;o__Enterobacterales;f__Erwiniaceae;g__Pantoea;s__Pantoea_deleyi
PB2_gene_2504	123	ORM76326.1	113	109/113(96.46)	1.84e-65	DNA damage-inducible SOS regulon protein [Pantoea deleyi]	470932	d__Bacteria;k__norank;p__Proteobacteria;c__Gammaproteobacteria;o__Enterobacterales;f__Erwiniaceae;g__Pantoea;s__Pantoea_deleyi
PB2_gene_2508	296	WP_111140855.1	287	278/287(96.86)	1.24e-207	polyamine aminopropyltransferase [Pantoea	2027922	d__Bacteria;k__norank;p__Proteobacteria;c__Gammaproteobacteria;o__Enterobacterales;f__Erwiniaceae;g__Pantoea;s__Pantoea_sp._ARC607
PB2_gene_2509	269	ORM75907.1	269	266/269(98.88)	2.55e-200	S-adenosylmethionine decarboxylase [Pantoea deleyi]	470932	d__Bacteria;k__norank;p__Proteobacteria;c__Gammaproteobacteria;o__Enterobacterales;f__Erwiniaceae;g__Pantoea;s__Pantoea_deleyi
PB2_gene_2510	254	ORM75908.1	254	253/254(99.61)	2.70e-174	DeoR family transcriptional regulator [Pantoea deleyi]	470932	d__Bacteria;k__norank;p__Proteobacteria;c__Gammaproteobacteria;o__Enterobacterales;f__Erwiniaceae;g__Pantoea;s__Pantoea_deleyi
PB2_gene_2518	47	OJH30452.1	388	46/46(100.00)	9.38e-24	Phage tail protein (Tail_P2_I) [Armadillidium vulgare]	77039	d__Bacteria;k__norank;p__Proteobacteria;c__Alphaproteobacteria;o__Rickettsiales;f__Anaplasmataceae;g__Wolbachia;s__Wolbachia_endosymbiont_of_Armadillidium_vulgare
PB2_gene_2521	345	WP_108784508.1	345	342/345(99.13)	2.21e-246	DUF1016 domain-containing protein [Wolbachia endosymbiont of Bemisia tabaci]	215173	d__Bacteria;k__norank;p__Proteobacteria;c__Alphaproteobacteria;o__Rickettsiales;f__Anaplasmataceae;g__Wolbachia;s__Wolbachia_endosymbiont_of_Bemisia_tabaci
PB2_gene_2522	84	WP_064085560.1	84	49/49(100.00)	5.02e-29	hypothetical protein [Wolbachia endosymbiont of Dactylopius coccus]	None	Unclassified
PB2_gene_2529	561	ORM80862.1	561	551/561(98.22)	0	sugar phosphorylase [Pantoea deleyi]	470932	d__Bacteria;k__norank;p__Proteobacteria;c__Gammaproteobacteria;o__Enterobacterales;f__Erwiniaceae;g__Pantoea;s__Pantoea_deleyi
PB2_gene_2537	94	SAJ21625.1	102	41/47(87.23)	2.05e-15	Uncharacterised protein [Enterobacter cloacae]	550	d__Bacteria;k__norank;p__Proteobacteria;c__Gammaproteobacteria;o__Enterobacterales;f__Enterobacteriaceae;g__Enterobacter;s__Enterobacter_cloacae
PB2_gene_2540	129	ORM84697.1	563	129/129(100.00)	3.20e-80	bifunctional glucose-1-phosphatase/inositol phosphatase [Pantoea deleyi]	470932	d__Bacteria;k__norank;p__Proteobacteria;c__Gammaproteobacteria;o__Enterobacterales;f__Erwiniaceae;g__Pantoea;s__Pantoea_deleyi
PB2_gene_2542	108	ORM84699.1	108	105/108(97.22)	2.34e-72	sulfurtransferase TusE [Pantoea deleyi]	470932	d__Bacteria;k__norank;p__Proteobacteria;c__Gammaproteobacteria;o__Enterobacterales;f__Erwiniaceae;g__Pantoea;s__Pantoea_deleyi
PB2_gene_2551	303	ORM81899.1	303	300/303(99.01)	3.78e-210	glycine/betaine ABC transporter substrate-binding protein [Pantoea deleyi]	470932	d__Bacteria;k__norank;p__Proteobacteria;c__Gammaproteobacteria;o__Enterobacterales;f__Erwiniaceae;g__Pantoea;s__Pantoea_deleyi
PB2_gene_2556	523	AZI50847.1	518	444/516(86.05)	1.92e-299	bifunctional diguanylate cyclase/phosphodiesterase [Pantoea agglomerans]	549	d__Bacteria;k__norank;p__Proteobacteria;c__Gammaproteobacteria;o__Enterobacterales;f__Erwiniaceae;g__Pantoea;s__Pantoea_agglomerans

PB2_gene_2564	85	ELP24922.1	70	62/67(92.54)	5.23e-31	hypothetical protein F385_2007 [Pantoea agglomerans 299R]	1261128	d__Bacteria;k__norank;p__Proteobacteria;c__Gammaproteobacteria;o__Enterobacterales;f__Erwiniaceae;g__Pantoea;s__Pantoea_agglomerans
PB2_gene_2566	306	WP_111139629.1	321	304/306(99.35)	5.46e-215	glutaminase B [Pantoea sp. ARC607]	2027922	d__Bacteria;k__norank;p__Proteobacteria;c__Gammaproteobacteria;o__Enterobacterales;f__Erwiniaceae;g__Pantoea;s__Pantoea_sp._ARC607
PB2_gene_2567	293	WP_111139628.1	286	248/279(88.89)	2.51e-174	Rpn family recombination-promoting nuclease/putative transposase [Pantoea sp.	2027922	d__Bacteria;k__norank;p__Proteobacteria;c__Gammaproteobacteria;o__Enterobacterales;f__Erwiniaceae;g__Pantoea;s__Pantoea_sp._ARC607
PB2_gene_2570	692	WP_111140310.1	692	670/692(96.82)	0	DNA polymerase III subunit gamma/tau [Pantoea sp. ARC607]	2027922	d__Bacteria;k__norank;p__Proteobacteria;c__Gammaproteobacteria;o__Enterobacterales;f__Erwiniaceae;g__Pantoea;s__Pantoea_sp._ARC607
PB2_gene_2575	219	ORM79314.1	219	216/219(98.63)	3.32e-153	riboflavin synthase subunit alpha [Pantoea deleyi]	470932	d__Bacteria;k__norank;p__Proteobacteria;c__Gammaproteobacteria;o__Enterobacterales;f__Erwiniaceae;g__Pantoea;s__Pantoea_deleyi
PB2_gene_2576	457	ORM79315.1	457	454/456(99.56)	0	MATE family efflux transporter [Pantoea deleyi]	470932	d__Bacteria;k__norank;p__Proteobacteria;c__Gammaproteobacteria;o__Enterobacterales;f__Erwiniaceae;g__Pantoea;s__Pantoea_deleyi
PB2_gene_2578	267	WP_111139514.1	1320	164/272(60.29)	8.57e-80	BapA prefix-like domain-containing protein [Pantoea sp.	2027922	d__Bacteria;k__norank;p__Proteobacteria;c__Gammaproteobacteria;o__Enterobacterales;f__Erwiniaceae;g__Pantoea;s__Pantoea_sp._ARC607
PB2_gene_2582	232	CCK16010.1	342	69/154(44.81)	7.61e-30	hypothetical protein BN136_2020 [Cronobacter universalis NCTC	1074000	d__Bacteria;k__norank;p__Proteobacteria;c__Gammaproteobacteria;o__Enterobacterales;f__Enterobacteriaceae;g__Cronobacter;s__Cronobacter_universalis
PB2_gene_2586	49	RDT64030.1	52	47/49(95.92)	6.33e-24	hypothetical protein DXF97_31255, partial	573	d__Bacteria;k__norank;p__Proteobacteria;c__Gammaproteobacteria;o__Enterobacterales;f__Enterobacteriaceae;g__Klebsiella;s__Klebsiella_pneumoniae
PB2_gene_2587	698	ORM81549.1	698	674/698(96.56)	0	multifunctional fatty acid oxidation complex subunit alpha [Pantoea deleyi]	470932	d__Bacteria;k__norank;p__Proteobacteria;c__Gammaproteobacteria;o__Enterobacterales;f__Erwiniaceae;g__Pantoea;s__Pantoea_deleyi
PB2_gene_2589	182	ORM81551.1	182	182/182(100.00)	3.57e-132	hypothetical protein HA42_10435 [Pantoea deleyi]	470932	d__Bacteria;k__norank;p__Proteobacteria;c__Gammaproteobacteria;o__Enterobacterales;f__Erwiniaceae;g__Pantoea;s__Pantoea_deleyi
PB2_gene_2597	816	WP_010404951.1	816	813/816(99.63)	0	type I DNA topoisomerase [Wolbachia endosymbiont of Nasonia vitripennis]	2640676	d__Bacteria;k__norank;p__Proteobacteria;c__Alphaproteobacteria;o__Rickettsiales;f__Anaplasmataceae;g__Wolbachia;s__norank
PB2_gene_2600	468	ORM77874.1	468	464/468(99.15)	0	multidrug transporter subunit MdtD [Pantoea deleyi]	470932	d__Bacteria;k__norank;p__Proteobacteria;c__Gammaproteobacteria;o__Enterobacterales;f__Erwiniaceae;g__Pantoea;s__Pantoea_deleyi
PB2_gene_2604	237	OQV41631.1	237	229/237(96.62)	1.56e-160	phosphonate C-P lyase system protein PhnL [Pantoea vagans]	470934	d__Bacteria;k__norank;p__Proteobacteria;c__Gammaproteobacteria;o__Enterobacterales;f__Erwiniaceae;g__Pantoea;s__Pantoea_vagans
PB2_gene_2605	253	WP_111141393.1	253	247/253(97.63)	1.14e-170	phosphonate C-P lyase system protein PhnK [Pantoea sp.	2027922	d__Bacteria;k__norank;p__Proteobacteria;c__Gammaproteobacteria;o__Enterobacterales;f__Erwiniaceae;g__Pantoea;s__Pantoea_sp._ARC607
PB2_gene_2607	358	WP_111141391.1	358	343/358(95.81)	2.47e-245	carbon-phosphorus lyase complex subunit PhnI [Pantoea sp.	2027922	d__Bacteria;k__norank;p__Proteobacteria;c__Gammaproteobacteria;o__Enterobacterales;f__Erwiniaceae;g__Pantoea;s__Pantoea_sp._ARC607
PB2_gene_2610	577	WP_068651372.1	577	558/577(96.71)	0	DNA primase [Wolbachia endosymbiont of Trichogramma pretiosum]	125593	d__Bacteria;k__norank;p__Proteobacteria;c__Alphaproteobacteria;o__Rickettsiales;f__Anaplasmataceae;g__Wolbachia;s__Wolbachia_endosymbiont_of_Trichogramma_pretiosum
PB2_gene_2613	105	SFN15833.1	105	100/105(95.24)	2.67e-63	YfcL protein [Pantoea sp. OV426]	1881036	d__Bacteria;k__norank;p__Proteobacteria;c__Gammaproteobacteria;o__Enterobacterales;f__Erwiniaceae;g__Pantoea;s__Pantoea_sp._OV426
PB2_gene_2614	181	ORM74996.1	181	179/181(98.90)	7.52e-138	elongation factor P hydroxylase [Pantoea deleyi]	470932	d__Bacteria;k__norank;p__Proteobacteria;c__Gammaproteobacteria;o__Enterobacterales;f__Erwiniaceae;g__Pantoea;s__Pantoea_deleyi
PB2_gene_2615	267	WP_111141409.1	267	262/267(98.13)	2.20e-175	hypothetical protein [Pantoea sp. ARC607]	2027922	d__Bacteria;k__norank;p__Proteobacteria;c__Gammaproteobacteria;o__Enterobacterales;f__Erwiniaceae;g__Pantoea;s__Pantoea_sp._ARC607
PB2_gene_2617	364	WP_033783750.1	361	358/361(99.17)	1.73e-261	MULTISPECIES: chorismate synthase [Pantoea]	53335	d__Bacteria;k__norank;p__Proteobacteria;c__Gammaproteobacteria;o__Enterobacterales;f__Erwiniaceae;g__Pantoea;s__norank
PB2_gene_2631	250	OQV42808.1	250	249/250(99.60)	1.20e-175	transcriptional regulator FNR [Pantoea vagans]	470934	d__Bacteria;k__norank;p__Proteobacteria;c__Gammaproteobacteria;o__Enterobacterales;f__Erwiniaceae;g__Pantoea;s__Pantoea_vagans
PB2_gene_2634	306	WP_111141298.1	306	306/306(100.00)	1.33e-216	LysR family transcriptional regulator [Pantoea sp. ARC607]	2027922	d__Bacteria;k__norank;p__Proteobacteria;c__Gammaproteobacteria;o__Enterobacterales;f__Erwiniaceae;g__Pantoea;s__Pantoea_sp._ARC607

PB2_gene_2635	444	ORM80687.1	444	439/444(98.87)	1.07e-310	4-hydroxybenzoate transporter [Pantoea deleyi]	470932	d__Bacteria;k__norank;p__Proteobacteria;c__Gammaproteobacteria;o__Enterobacterales;f__Erwiniaceae;g__Pantoea;s__Pantoea_deleyi
PB2_gene_2637	390	WP_063630777.1	388	304/390(77.95)	5.38e-179	hypothetical protein [Wolbachia endosymbiont of Laodelphax striatella]	368602	d__Bacteria;k__norank;p__Proteobacteria;c__Alphaproteobacteria;o__Rickettsiales;f__Anaplasmataceae;g__Wolbachia;s__Wolbachia_endosymbiont_of_Laodelphax_striatellus
PB2_gene_2644	386	PCG11211.1	386	364/386(94.30)	2.47e-266	porin [Wolbachia pipientis]	None	Unclassified
PB2_gene_2651	423	ERM07711.1	423	383/423(90.54)	3.09e-281	alcohol dehydrogenase [Pantoea agglomerans Tx10]	1332072	d__Bacteria;k__norank;p__Proteobacteria;c__Gammaproteobacteria;o__Enterobacterales;f__Erwiniaceae;g__Pantoea;s__Pantoea_agglomerans
PB2_gene_2670	324	ORM85867.1	321	317/321(98.75)	4.06e-233	magnesium and cobalt transport protein CorA [Pantoea deleyi]	470932	d__Bacteria;k__norank;p__Proteobacteria;c__Gammaproteobacteria;o__Enterobacterales;f__Erwiniaceae;g__Pantoea;s__Pantoea_deleyi
PB2_gene_2671	339	ORM85833.1	339	330/339(97.35)	4.82e-247	hypothetical protein HA42_01900 [Pantoea deleyi]	470932	d__Bacteria;k__norank;p__Proteobacteria;c__Gammaproteobacteria;o__Enterobacterales;f__Erwiniaceae;g__Pantoea;s__Pantoea_deleyi
PB2_gene_2674	991	WP_108784365.1	1043	464/1053(44.06)	4.41e-147	collagen-like protein [Wolbachia endosymbiont of Bemisia tabaci]	215173	d__Bacteria;k__norank;p__Proteobacteria;c__Alphaproteobacteria;o__Rickettsiales;f__Anaplasmataceae;g__Wolbachia;s__Wolbachia_endosymbiont_of_Bemisia_tabaci
PB2_gene_2677	301	WP_107319856.1	3822	283/301(94.02)	1.15e-157	filamentous hemagglutinin N-terminal domain-containing protein [Pantoea vagans]	470934	d__Bacteria;k__norank;p__Proteobacteria;c__Gammaproteobacteria;o__Enterobacterales;f__Erwiniaceae;g__Pantoea;s__Pantoea_vagans
PB2_gene_2686	112	WP_109650904.1	112	99/111(89.19)	7.40e-62	hypothetical protein [Pantoea	None	Unclassified
PB2_gene_2687	90	WP_109650906.1	90	79/90(87.78)	6.12e-53	hypothetical protein [Pantoea	None	Unclassified
PB2_gene_2688	118	ORM87437.1	314	114/118(96.61)	2.41e-72	hypothetical protein HA38_04360 [Pantoea allii]	574096	d__Bacteria;k__norank;p__Proteobacteria;c__Gammaproteobacteria;o__Enterobacterales;f__Erwiniaceae;g__Pantoea;s__Pantoea_allii
PB2_gene_2690	148	WP_111138731.1	128	114/128(89.06)	1.01e-74	hypothetical protein [Pantoea sp. ARC607]	None	Unclassified
PB2_gene_2694	843	WP_109728766.1	843	783/843(92.88)	0	type VI secretion system tip protein VgrG [Edwardsiella	636	d__Bacteria;k__norank;p__Proteobacteria;c__Gammaproteobacteria;o__Enterobacterales;f__Hafniaceae;g__Edwardsiella;s__Edwardsiella_tarda
PB2_gene_2697	337	ORM81174.1	335	324/335(96.72)	1.67e-241	UV damage endonuclease UvsE [Pantoea deleyi]	470932	d__Bacteria;k__norank;p__Proteobacteria;c__Gammaproteobacteria;o__Enterobacterales;f__Erwiniaceae;g__Pantoea;s__Pantoea_deleyi
PB2_gene_2702	83	WP_111142007.1	83	83/83(100.00)	4.33e-52	hypothetical protein [Pantoea sp. ARC607]	2027922	d__Bacteria;k__norank;p__Proteobacteria;c__Gammaproteobacteria;o__Enterobacterales;f__Erwiniaceae;g__Pantoea;s__Pantoea_sp._ARC607
PB2_gene_2706	263	ORM77612.1	263	259/263(98.48)	3.08e-189	serine/threonine phosphatase [Pantoea deleyi]	470932	d__Bacteria;k__norank;p__Proteobacteria;c__Gammaproteobacteria;o__Enterobacterales;f__Erwiniaceae;g__Pantoea;s__Pantoea_deleyi
PB2_gene_2707	624	WP_111140774.1	619	579/624(92.79)	0	type VI secretion system-associated FHA domain protein TagH [Pantoea sp. ARC607]	2027922	d__Bacteria;k__norank;p__Proteobacteria;c__Gammaproteobacteria;o__Enterobacterales;f__Erwiniaceae;g__Pantoea;s__Pantoea_sp._ARC607
PB2_gene_2708	137	WP_086905639.1	137	73/137(53.28)	7.79e-41	hypothetical protein [Pantoea agglomerans]	549	d__Bacteria;k__norank;p__Proteobacteria;c__Gammaproteobacteria;o__Enterobacterales;f__Erwiniaceae;g__Pantoea;s__Pantoea_agglomerans
PB2_gene_2714	178	WP_031590427.1	178	118/175(67.43)	5.47e-84	type VI secretion system lipoprotein TssJ [Pantoea	549	d__Bacteria;k__norank;p__Proteobacteria;c__Gammaproteobacteria;o__Enterobacterales;f__Erwiniaceae;g__Pantoea;s__Pantoea_agglomerans
PB2_gene_2716	499	WP_031590429.1	499	488/498(97.99)	0	type VI secretion system contractile sheath large subunit [Pantoea agglomerans]	549	d__Bacteria;k__norank;p__Proteobacteria;c__Gammaproteobacteria;o__Enterobacterales;f__Erwiniaceae;g__Pantoea;s__Pantoea_agglomerans
PB2_gene_2721	308	ORM82391.1	308	298/308(96.75)	7.37e-215	muramoyltetrapeptide carboxypeptidase [Pantoea	470932	d__Bacteria;k__norank;p__Proteobacteria;c__Gammaproteobacteria;o__Enterobacterales;f__Erwiniaceae;g__Pantoea;s__Pantoea_deleyi
PB2_gene_2724	549	ORM76355.1	531	522/531(98.31)	0	peptide ABC transporter substrate-binding protein	470932	d__Bacteria;k__norank;p__Proteobacteria;c__Gammaproteobacteria;o__Enterobacterales;f__Erwiniaceae;g__Pantoea;s__Pantoea_deleyi
PB2_gene_2726	258	ORM76353.1	258	250/258(96.90)	2.70e-176	ABC transporter substrate-binding protein [Pantoea deleyi]	470932	d__Bacteria;k__norank;p__Proteobacteria;c__Gammaproteobacteria;o__Enterobacterales;f__Erwiniaceae;g__Pantoea;s__Pantoea_deleyi
PB2_gene_2728	591	ORM79850.1	591	572/591(96.79)	0	histidine kinase [Pantoea deleyi]	470932	d__Bacteria;k__norank;p__Proteobacteria;c__Gammaproteobacteria;o__Enterobacterales;f__Erwiniaceae;g__Pantoea;s__Pantoea_deleyi

PB2_gene_2733	300	WP_111139823.1	300	294/299(98.33)	1.25e-202	uridine diphosphate-N-acetylglucosamine-binding protein YvcK [Pantoea sp.	2027922	d__Bacteria;k__norank;p__Proteobacteria;c__Gammaproteobacteria;o__Enterobacterales;f__Erwiniaceae;g__Pantoea;s__Pantoea_sp._ARC607
PB2_gene_2748	294	WP_111140839.1	293	288/293(98.29)	1.97e-214	prolipoprotein diacylglyceryl transferase [Pantoea sp. ARC607]	2027922	d__Bacteria;k__norank;p__Proteobacteria;c__Gammaproteobacteria;o__Enterobacterales;f__Erwiniaceae;g__Pantoea;s__Pantoea_sp._ARC607
PB2_gene_2749	264	ORM86582.1	264	263/264(99.62)	1.04e-201	thymidylate synthase [Pantoea deleyi]	470932	d__Bacteria;k__norank;p__Proteobacteria;c__Gammaproteobacteria;o__Enterobacterales;f__Erwiniaceae;g__Pantoea;s__Pantoea_deleyi
PB2_gene_2750	155	ORM86583.1	155	140/155(90.32)	1.29e-96	prepilin-type N-terminal cleavage/methylation domain-containing protein [Pantoea	470932	d__Bacteria;k__norank;p__Proteobacteria;c__Gammaproteobacteria;o__Enterobacterales;f__Erwiniaceae;g__Pantoea;s__Pantoea_deleyi
PB2_gene_2751	175	WP_111140832.1	177	165/174(94.83)	3.84e-115	prepilin peptidase-dependent protein [Pantoea sp. ARC607]	2027922	d__Bacteria;k__norank;p__Proteobacteria;c__Gammaproteobacteria;o__Enterobacterales;f__Erwiniaceae;g__Pantoea;s__Pantoea_sp._ARC607
PB2_gene_2770	350	EFM19496.1	350	340/350(97.14)	2.99e-242	37kDa nucleoid-associated protein [Pantoea sp. aB]	517433	d__Bacteria;k__norank;p__Proteobacteria;c__Gammaproteobacteria;o__Enterobacterales;f__Erwiniaceae;g__Pantoea;s__Pantoea_sp._aB
PB2_gene_2774	67	ORM85811.1	67	67/67(100.00)	6.05e-36	Sec-independent protein translocase TatE [Pantoea deleyi]	470932	d__Bacteria;k__norank;p__Proteobacteria;c__Gammaproteobacteria;o__Enterobacterales;f__Erwiniaceae;g__Pantoea;s__Pantoea_deleyi
PB2_gene_2775	264	ORM85812.1	264	254/264(96.21)	1.82e-177	hydrolase [Pantoea deleyi]	470932	d__Bacteria;k__norank;p__Proteobacteria;c__Gammaproteobacteria;o__Enterobacterales;f__Erwiniaceae;g__Pantoea;s__Pantoea_deleyi
PB2_gene_2776	140	ORM85813.1	140	138/140(98.57)	1.35e-93	GNAT family N-acetyltransferase [Pantoea deleyi]	470932	d__Bacteria;k__norank;p__Proteobacteria;c__Gammaproteobacteria;o__Enterobacterales;f__Erwiniaceae;g__Pantoea;s__Pantoea_deleyi
PB2_gene_2780	140	ORM83163.1	119	106/119(89.08)	1.16e-60	hypothetical protein HA42_06845 [Pantoea deleyi]	470932	d__Bacteria;k__norank;p__Proteobacteria;c__Gammaproteobacteria;o__Enterobacterales;f__Erwiniaceae;g__Pantoea;s__Pantoea_deleyi
PB2_gene_2784	344	ORM83134.1	344	343/344(99.71)	2.25e-259	cysteine synthase [Pantoea deleyi]	470932	d__Bacteria;k__norank;p__Proteobacteria;c__Gammaproteobacteria;o__Enterobacterales;f__Erwiniaceae;g__Pantoea;s__Pantoea_deleyi
PB2_gene_2789	598	WP_064085737.1	598	595/598(99.50)	0	elongation factor 4 [Wolbachia endosymbiont of Dactylopius coccus]	None	Unclassified
PB2_gene_2797	332	WP_111138391.1	332	324/332(97.59)	9.89e-232	MsnO8 family LLM class oxidoreductase [Pantoea sp. ARC607]	2027922	d__Bacteria;k__norank;p__Proteobacteria;c__Gammaproteobacteria;o__Enterobacterales;f__Erwiniaceae;g__Pantoea;s__Pantoea_sp._ARC607
PB2_gene_2799	249	WP_111138390.1	244	231/244(94.67)	2.21e-158	GntR family transcriptional regulator [Pantoea sp. ARC607]	53335	d__Bacteria;k__norank;p__Proteobacteria;c__Gammaproteobacteria;o__Enterobacterales;f__Erwiniaceae;g__Pantoea;s__norank
PB2_gene_2800	149	ORM83621.1	149	145/149(97.32)	5.81e-97	PTS mannitol transporter subunit IIA [Pantoea deleyi]	470932	d__Bacteria;k__norank;p__Proteobacteria;c__Gammaproteobacteria;o__Enterobacterales;f__Erwiniaceae;g__Pantoea;s__Pantoea_deleyi
PB2_gene_2803	188	WP_111140981.1	188	174/188(92.55)	3.25e-122	nucleotidyltransferase family protein [Pantoea sp. ARC607]	2027922	d__Bacteria;k__norank;p__Proteobacteria;c__Gammaproteobacteria;o__Enterobacterales;f__Erwiniaceae;g__Pantoea;s__Pantoea_sp._ARC607
PB2_gene_2805	379	ORM84146.1	378	358/379(94.46)	9.13e-256	hypothetical protein HA42_05165 [Pantoea deleyi]	470932	d__Bacteria;k__norank;p__Proteobacteria;c__Gammaproteobacteria;o__Enterobacterales;f__Erwiniaceae;g__Pantoea;s__Pantoea_deleyi
PB2_gene_2808	714	WP_111141806.1	714	679/714(95.10)	0	bifunctional diguanylate cyclase/phosphodiesterase [Pantoea sp. ARC607]	2027922	d__Bacteria;k__norank;p__Proteobacteria;c__Gammaproteobacteria;o__Enterobacterales;f__Erwiniaceae;g__Pantoea;s__Pantoea_sp._ARC607
PB2_gene_2813	408	PBD15876.1	408	388/408(95.10)	1.17e-282	hypothetical protein CLD06_03675 [Wolbachia endosymbiont of Drosophila	2033655	d__Bacteria;k__norank;p__Proteobacteria;c__Alphaproteobacteria;o__Rickettsiales;f__Anaplasmataceae;g__Wolbachia;s__Wolbachia_endosymbiont_of_Drosophila_subpulchrella
PB2_gene_2816	599	PCG11591.1	599	592/599(98.83)	0	succinate dehydrogenase flavoprotein subunit [Wolbachia pipientis]	None	Unclassified
PB2_gene_2824	271	AGJ99259.1	349	261/271(96.31)	5.73e-187	M23 peptidase domain protein [Wolbachia endosymbiont of Drosophila simulans wNo]	1236908	d__Bacteria;k__norank;p__Proteobacteria;c__Alphaproteobacteria;o__Rickettsiales;f__Anaplasmataceae;g__Wolbachia;s__Wolbachia_endosymbiont_of_Drosophila_simulans

PB2_gene_2827	440	ORM82480.1	440	439/440(99.77)	2.29e-303	hypothetical protein HA42_08255 [Pantoea deleyi]	470932	d__Bacteria;k__norank;p__Proteobacteria;c__Gammaproteobacteria;o__Enterobacterales;f__Erwiniaceae;g__Pantoea;s__Pantoea_deleyi
PB2_gene_2831	454	WP_111140026.1	454	447/454(98.46)	2.09e-313	ethanolamine permease [Pantoea sp. ARC607]	2027922	d__Bacteria;k__norank;p__Proteobacteria;c__Gammaproteobacteria;o__Enterobacterales;f__Erwiniaceae;g__Pantoea;s__Pantoea_sp._ARC607
PB2_gene_2832	303	ORM75499.1	296	288/296(97.30)	8.35e-210	fructokinase, partial [Pantoea deleyi]	470932	d__Bacteria;k__norank;p__Proteobacteria;c__Gammaproteobacteria;o__Enterobacterales;f__Erwiniaceae;g__Pantoea;s__Pantoea_deleyi
PB2_gene_2841	438	EEB55502.1	440	417/435(95.86)	2.41e-293	peptidase, M16 family [Wolbachia endosymbiont of Culex quinquefasciatus JHB]	569881	d__Bacteria;k__norank;p__Proteobacteria;c__Alphaproteobacteria;o__Rickettsiales;f__Anaplasmataceae;g__Wolbachia;s__Wolbachia_endosymbiont_of_Culex_quinquefasciatus
PB2_gene_2849	323	ORM85221.1	323	311/323(96.28)	5.08e-209	chromosome partitioning protein ParB [Pantoea deleyi]	470932	d__Bacteria;k__norank;p__Proteobacteria;c__Gammaproteobacteria;o__Enterobacterales;f__Erwiniaceae;g__Pantoea;s__Pantoea_deleyi
PB2_gene_2853	406	WP_111138793.1	401	391/401(97.51)	5.08e-293	nicotinate phosphoribosyltransferase [Pantoea sp. ARC607]	2027922	d__Bacteria;k__norank;p__Proteobacteria;c__Gammaproteobacteria;o__Enterobacterales;f__Erwiniaceae;g__Pantoea;s__Pantoea_sp._ARC607
PB2_gene_2858	456	WP_111138589.1	460	438/455(96.26)	4.20e-299	efflux transporter outer membrane subunit [Pantoea sp. ARC607]	2027922	d__Bacteria;k__norank;p__Proteobacteria;c__Gammaproteobacteria;o__Enterobacterales;f__Erwiniaceae;g__Pantoea;s__Pantoea_sp._ARC607
PB2_gene_2859	364	WP_111138588.1	364	354/364(97.25)	1.29e-234	HlyD family secretion protein [Pantoea sp. ARC607]	2027922	d__Bacteria;k__norank;p__Proteobacteria;c__Gammaproteobacteria;o__Enterobacterales;f__Erwiniaceae;g__Pantoea;s__Pantoea_sp._ARC607
PB2_gene_2862	347	ORM82050.1	331	331/331(100.00)	1.82e-233	methyl-galactoside ABC transporter substrate-binding protein [Pantoea deleyi]	470932	d__Bacteria;k__norank;p__Proteobacteria;c__Gammaproteobacteria;o__Enterobacterales;f__Erwiniaceae;g__Pantoea;s__Pantoea_deleyi
PB2_gene_2875	371	ORM82370.1	371	362/371(97.57)	2.11e-264	putrescine/spermidine ABC transporter ATP-binding protein [Pantoea deleyi]	470932	d__Bacteria;k__norank;p__Proteobacteria;c__Gammaproteobacteria;o__Enterobacterales;f__Erwiniaceae;g__Pantoea;s__Pantoea_deleyi
PB2_gene_2877	257	ORM82368.1	257	253/257(98.44)	6.76e-165	spermidine/putrescine ABC transporter permease PotC [Pantoea deleyi]	470932	d__Bacteria;k__norank;p__Proteobacteria;c__Gammaproteobacteria;o__Enterobacterales;f__Erwiniaceae;g__Pantoea;s__Pantoea_deleyi
PB2_gene_2880	693	ORM81217.1	693	684/693(98.70)	0	ribonucleotide-diphosphate reductase subunit alpha [Pantoea deleyi]	470932	d__Bacteria;k__norank;p__Proteobacteria;c__Gammaproteobacteria;o__Enterobacterales;f__Erwiniaceae;g__Pantoea;s__Pantoea_deleyi
PB2_gene_2884	951	ORM86016.1	951	942/951(99.05)	0	valine--tRNA ligase [Pantoea deleyi]	470932	d__Bacteria;k__norank;p__Proteobacteria;c__Gammaproteobacteria;o__Enterobacterales;f__Erwiniaceae;g__Pantoea;s__Pantoea_deleyi
PB2_gene_2885	152	WP_111138539.1	152	152/152(100.00)	1.08e-107	DNA polymerase III subunit chi [Pantoea sp. ARC607]	2027922	d__Bacteria;k__norank;p__Proteobacteria;c__Gammaproteobacteria;o__Enterobacterales;f__Erwiniaceae;g__Pantoea;s__Pantoea_sp._ARC607
PB2_gene_2887	212	WP_128081746.1	336	152/207(73.43)	2.86e-95	hypothetical protein [Wolbachia endosymbiont of Dactylopius coccus]	None	Unclassified
PB2_gene_2896	641	ORM85208.1	641	615/640(96.09)	0	chemotaxis protein [Pantoea deleyi]	470932	d__Bacteria;k__norank;p__Proteobacteria;c__Gammaproteobacteria;o__Enterobacterales;f__Erwiniaceae;g__Pantoea;s__Pantoea_deleyi
PB2_gene_2904	811	ORM84546.1	811	807/811(99.51)	0	bifunctional aspartate kinase/homoserine dehydrogenase II [Pantoea deleyi]	470932	d__Bacteria;k__norank;p__Proteobacteria;c__Gammaproteobacteria;o__Enterobacterales;f__Erwiniaceae;g__Pantoea;s__Pantoea_deleyi
PB2_gene_2910	375	WP_111138761.1	375	363/375(96.80)	1.53e-259	YbfB/YjiJ family MFS transporter [Pantoea sp. ARC607]	2027922	d__Bacteria;k__norank;p__Proteobacteria;c__Gammaproteobacteria;o__Enterobacterales;f__Erwiniaceae;g__Pantoea;s__Pantoea_sp._ARC607
PB2_gene_2912	1305	ORM82550.1	1315	1291/1305(98.93)	0	trifunctional transcriptional regulator/proline dehydrogenase/L-glutamate gamma-semialdehyde	470932	d__Bacteria;k__norank;p__Proteobacteria;c__Gammaproteobacteria;o__Enterobacterales;f__Erwiniaceae;g__Pantoea;s__Pantoea_deleyi

PB2_gene_2914	423	ORM83140.1	423	423/423(100.00)	4.26e-304	ATP-dependent protease ATP-binding subunit ClpX [Pantoea deleyi]	470932	d__Bacteria;k__norank;p__Proteobacteria;c__Gammaproteobacteria;o__Enterobacterales;f__Erwiniaceae;g__Pantoea;s__Pantoea_deleyi
PB2_gene_2920	469	ORM81203.1	470	468/468(100.00)	0	amino acid permease [Pantoea deleyi]	470932	d__Bacteria;k__norank;p__Proteobacteria;c__Gammaproteobacteria;o__Enterobacterales;f__Erwiniaceae;g__Pantoea;s__Pantoea_deleyi
PB2_gene_2925	599	WP_064085538.1	602	538/602(89.37)	0	DNA mismatch repair endonuclease MutL [Wolbachia endosymbiont of Dactylopius	None	Unclassified
PB2_gene_2930	306	WP_110411001.1	289	250/288(86.81)	1.45e-184	transporter substrate-binding domain-containing protein [Pantoea vagans]	None	Unclassified
PB2_gene_2931	757	WP_095706663.1	757	744/757(98.28)	0	DNA topoisomerase IV subunit A [Pantoea vagans]	470934	d__Bacteria;k__norank;p__Proteobacteria;c__Gammaproteobacteria;o__Enterobacterales;f__Erwiniaceae;g__Pantoea;s__Pantoea_vagans
PB2_gene_2933	555	ORM80838.1	555	551/555(99.28)	0	asparagine synthase B [Pantoea deleyi]	470932	d__Bacteria;k__norank;p__Proteobacteria;c__Gammaproteobacteria;o__Enterobacterales;f__Erwiniaceae;g__Pantoea;s__Pantoea_deleyi
PB2_gene_2934	72	CRH33026.1	89	49/53(92.45)	1.14e-25	hypothetical protein BN1183_AP_00010 [Pantoea ananatis]	553	d__Bacteria;k__norank;p__Proteobacteria;c__Gammaproteobacteria;o__Enterobacterales;f__Erwiniaceae;g__Pantoea;s__Pantoea_ananatis
PB2_gene_2948	942	WP_111140208.1	942	939/942(99.68)	0	excinuclease ABC subunit UvrA [Pantoea sp. ARC607]	2027922	d__Bacteria;k__norank;p__Proteobacteria;c__Gammaproteobacteria;o__Enterobacterales;f__Erwiniaceae;g__Pantoea;s__Pantoea_sp._ARC607
PB2_gene_2951	222	ORM86032.1	222	221/222(99.55)	2.05e-144	stress protection protein MarC [Pantoea deleyi]	470932	d__Bacteria;k__norank;p__Proteobacteria;c__Gammaproteobacteria;o__Enterobacterales;f__Erwiniaceae;g__Pantoea;s__Pantoea_deleyi
PB2_gene_2956	696	ORM82908.1	696	675/696(96.98)	0	hypothetical protein HA42_07205 [Pantoea deleyi]	470932	d__Bacteria;k__norank;p__Proteobacteria;c__Gammaproteobacteria;o__Enterobacterales;f__Erwiniaceae;g__Pantoea;s__Pantoea_deleyi
PB2_gene_2959	298	WP_119803819.1	306	188/306(61.44)	3.09e-125	DUF4255 domain-containing protein [Serratia sp. S40]	None	Unclassified
PB2_gene_2965	364	WP_062844679.1	365	263/364(72.25)	3.53e-193	NADH:flavin oxidoreductase [Psychrobacter alimentarius]	497	d__Bacteria;k__norank;p__Proteobacteria;c__Gammaproteobacteria;o__Pseudomonadales;f__Moraxellaceae;g__Psychrobacter;s__norank
PB2_gene_2972	428	ORM80585.1	428	419/428(97.90)	1.63e-290	chemotaxis protein [Pantoea deleyi]	470932	d__Bacteria;k__norank;p__Proteobacteria;c__Gammaproteobacteria;o__Enterobacterales;f__Erwiniaceae;g__Pantoea;s__Pantoea_deleyi
PB2_gene_2976	250	ORM79859.1	246	241/246(97.97)	1.07e-168	NADPH-dependent oxidoreductase [Pantoea deleyi]	470932	d__Bacteria;k__norank;p__Proteobacteria;c__Gammaproteobacteria;o__Enterobacterales;f__Erwiniaceae;g__Pantoea;s__Pantoea_deleyi
PB2_gene_2979	380	WP_111140681.1	380	334/380(87.89)	2.74e-244	hypothetical protein [Pantoea sp. ARC607]	2027922	d__Bacteria;k__norank;p__Proteobacteria;c__Gammaproteobacteria;o__Enterobacterales;f__Erwiniaceae;g__Pantoea;s__Pantoea_sp._ARC607
PB2_gene_2980	326	WP_111140680.1	326	289/326(88.65)	1.06e-207	hypothetical protein [Pantoea sp. ARC607]	2027922	d__Bacteria;k__norank;p__Proteobacteria;c__Gammaproteobacteria;o__Enterobacterales;f__Erwiniaceae;g__Pantoea;s__Pantoea_sp._ARC607
PB2_gene_2987	189	WP_105686070.1	189	162/187(86.63)	5.12e-115	hypothetical protein [Cronobacter dublinensis]	413497	d__Bacteria;k__norank;p__Proteobacteria;c__Gammaproteobacteria;o__Enterobacterales;f__Enterobacteriaceae;g__Cronobacter;s__Cronobacter_dublinensis
PB2_gene_2991	425	ORM81548.1	436	421/425(99.06)	9.05e-296	3-ketoacyl-CoA thiolase [Pantoea deleyi]	470932	d__Bacteria;k__norank;p__Proteobacteria;c__Gammaproteobacteria;o__Enterobacterales;f__Erwiniaceae;g__Pantoea;s__Pantoea_deleyi
PB2_gene_2996	167	AGJ99140.1	167	167/167(100.00)	2.78e-124	NADH-quinone oxidoreductase subunit B [Wolbachia endosymbiont of Drosophila	1236908	d__Bacteria;k__norank;p__Proteobacteria;c__Alphaproteobacteria;o__Rickettsiales;f__Anaplasmataceae;g__Wolbachia;s__Wolbachia_endosymbiont_of_Drosophila_simulans
PB2_gene_3006	1050	ORM80752.1	1050	1035/1050(98.57)	0	multidrug efflux RND transporter permease subunit [Pantoea deleyi]	470932	d__Bacteria;k__norank;p__Proteobacteria;c__Gammaproteobacteria;o__Enterobacterales;f__Erwiniaceae;g__Pantoea;s__Pantoea_deleyi
PB2_gene_3014	88	ORM83019.1	83	78/83(93.98)	9.26e-44	hypothetical protein HA42_07000 [Pantoea deleyi]	470932	d__Bacteria;k__norank;p__Proteobacteria;c__Gammaproteobacteria;o__Enterobacterales;f__Erwiniaceae;g__Pantoea;s__Pantoea_deleyi
PB2_gene_3021	412	WP_111138383.1	409	383/404(94.80)	3.66e-269	MFS transporter [Pantoea sp. ARC607]	2027922	d__Bacteria;k__norank;p__Proteobacteria;c__Gammaproteobacteria;o__Enterobacterales;f__Erwiniaceae;g__Pantoea;s__Pantoea_sp._ARC607
PB2_gene_3027	488	ORM79695.1	488	488/488(100.00)	0	MFS transporter [Pantoea deleyi]	470932	d__Bacteria;k__norank;p__Proteobacteria;c__Gammaproteobacteria;o__Enterobacterales;f__Erwiniaceae;g__Pantoea;s__Pantoea_deleyi

PB2_gene_3028	236	ORM81207.1	350	235/236(99.58)	1.00e-164	peptide ABC transporter permease [Pantoea deleyi]	470932	d__Bacteria;k__norank;p__Proteobacteria;c__Gammaproteobacteria;o__Enterobacterales;f__Erwiniaceae;g__Pantoea;s__Pantoea_deleyi
PB2_gene_3033	52	SKA25161.1	100	52/52(100.00)	1.57e-24	LSU ribosomal protein L23P [Pantoea agglomerans]	470933	d__Bacteria;k__norank;p__Proteobacteria;c__Gammaproteobacteria;o__Enterobacterales;f__Erwiniaceae;g__Pantoea;s__Pantoea_eucalypti
PB2_gene_3042	293	WP_111138914.1	293	286/293(97.61)	1.03e-202	LysR family transcriptional regulator [Pantoea sp. ARC607]	2027922	d__Bacteria;k__norank;p__Proteobacteria;c__Gammaproteobacteria;o__Enterobacterales;f__Erwiniaceae;g__Pantoea;s__Pantoea_sp._ARC607
PB2_gene_3043	181	ORM76844.1	181	176/181(97.24)	7.46e-120	TetR family transcriptional regulator [Pantoea deleyi]	470932	d__Bacteria;k__norank;p__Proteobacteria;c__Gammaproteobacteria;o__Enterobacterales;f__Erwiniaceae;g__Pantoea;s__Pantoea_deleyi
PB2_gene_3045	205	ORM76846.1	205	196/205(95.61)	6.72e-137	flavin-nucleotide-binding protein [Pantoea deleyi]	470932	d__Bacteria;k__norank;p__Proteobacteria;c__Gammaproteobacteria;o__Enterobacterales;f__Erwiniaceae;g__Pantoea;s__Pantoea_deleyi
PB2_gene_3049	720	ORM81646.1	720	718/720(99.72)	0	DNA helicase II [Pantoea deleyi]	470932	d__Bacteria;k__norank;p__Proteobacteria;c__Gammaproteobacteria;o__Enterobacterales;f__Erwiniaceae;g__Pantoea;s__Pantoea_deleyi
PB2_gene_3055	230	WP_111138302.1	231	218/231(94.37)	3.65e-149	haloacid dehalogenase type II [Pantoea sp. ARC607]	2027922	d__Bacteria;k__norank;p__Proteobacteria;c__Gammaproteobacteria;o__Enterobacterales;f__Erwiniaceae;g__Pantoea;s__Pantoea_sp._ARC607
PB2_gene_3056	334	WP_111139214.1	334	334/334(100.00)	2.05e-225	protease modulator HflC [Pantoea sp. ARC607]	2027922	d__Bacteria;k__norank;p__Proteobacteria;c__Gammaproteobacteria;o__Enterobacterales;f__Erwiniaceae;g__Pantoea;s__Pantoea_sp._ARC607
PB2_gene_3057	412	ORM79173.1	412	412/412(100.00)	2.83e-276	HflK protein [Pantoea deleyi]	470932	d__Bacteria;k__norank;p__Proteobacteria;c__Gammaproteobacteria;o__Enterobacterales;f__Erwiniaceae;g__Pantoea;s__Pantoea_deleyi
PB2_gene_3062	520	WP_128084751.1	520	513/520(98.65)	2.38e-274	resolvase [Pantoea deleyi]	470932	d__Bacteria;k__norank;p__Proteobacteria;c__Gammaproteobacteria;o__Enterobacterales;f__Erwiniaceae;g__Pantoea;s__Pantoea_deleyi
PB2_gene_3065	269	WP_111138858.1	269	255/269(94.80)	1.29e-184	23S rRNA (guanine(745)-N(1))-methyltransferase [Pantoea sp. ARC607]	2027922	d__Bacteria;k__norank;p__Proteobacteria;c__Gammaproteobacteria;o__Enterobacterales;f__Erwiniaceae;g__Pantoea;s__Pantoea_sp._ARC607
PB2_gene_3067	336	WP_111138857.1	336	313/336(93.15)	6.07e-244	MBL fold metallo-hydrolase [Pantoea sp. ARC607]	2027922	d__Bacteria;k__norank;p__Proteobacteria;c__Gammaproteobacteria;o__Enterobacterales;f__Erwiniaceae;g__Pantoea;s__Pantoea_sp._ARC607
PB2_gene_3071	95	Q9ZHG0.1	95	55/95(57.89)	3.05e-28	RecName: Full=Microcin E492 immunity protein	573	d__Bacteria;k__norank;p__Proteobacteria;c__Gammaproteobacteria;o__Enterobacterales;f__Enterobacteriaceae;g__Klebsiella;s__Klebsiella_pneumoniae
PB2_gene_3072	212	WP_111140355.1	212	208/212(98.11)	2.97e-148	hypothetical protein [Pantoea sp. ARC607]	2027922	d__Bacteria;k__norank;p__Proteobacteria;c__Gammaproteobacteria;o__Enterobacterales;f__Erwiniaceae;g__Pantoea;s__Pantoea_sp._ARC607
PB2_gene_3074	139	WP_105099190.1	135	132/135(97.78)	1.22e-81	hypothetical protein [Pantoea ananatis]	549	d__Bacteria;k__norank;p__Proteobacteria;c__Gammaproteobacteria;o__Enterobacterales;f__Erwiniaceae;g__Pantoea;s__Pantoea_agglomerans
PB2_gene_3077	926	WP_111138469.1	926	909/926(98.16)	0	flavocytochrome c [Pantoea sp. ARC607]	2027922	d__Bacteria;k__norank;p__Proteobacteria;c__Gammaproteobacteria;o__Enterobacterales;f__Erwiniaceae;g__Pantoea;s__Pantoea_sp._ARC607
PB2_gene_3092	280	ORM78368.1	280	273/280(97.50)	1.47e-200	3-mercaptopyruvate sulfurtransferase [Pantoea deleyi]	470932	d__Bacteria;k__norank;p__Proteobacteria;c__Gammaproteobacteria;o__Enterobacterales;f__Erwiniaceae;g__Pantoea;s__Pantoea_deleyi
PB2_gene_3102	86	ORM84675.1	86	84/86(97.67)	8.93e-51	hypothetical protein HA42_03790 [Pantoea deleyi]	470932	d__Bacteria;k__norank;p__Proteobacteria;c__Gammaproteobacteria;o__Enterobacterales;f__Erwiniaceae;g__Pantoea;s__Pantoea_deleyi
PB2_gene_3103	501	ORM84676.1	501	495/501(98.80)	0	transporter [Pantoea deleyi]	470932	d__Bacteria;k__norank;p__Proteobacteria;c__Gammaproteobacteria;o__Enterobacterales;f__Erwiniaceae;g__Pantoea;s__Pantoea_deleyi
PB2_gene_3112	1051	ORM77993.1	1051	1033/1051(98.29)	0	aminoglycoside/multidrug transporter permease [Pantoea deleyi]	470932	d__Bacteria;k__norank;p__Proteobacteria;c__Gammaproteobacteria;o__Enterobacterales;f__Erwiniaceae;g__Pantoea;s__Pantoea_deleyi
PB2_gene_3114	341	ORM79311.1	341	340/341(99.71)	1.14e-246	transcriptional repressor PurR [Pantoea deleyi]	470932	d__Bacteria;k__norank;p__Proteobacteria;c__Gammaproteobacteria;o__Enterobacterales;f__Erwiniaceae;g__Pantoea;s__Pantoea_deleyi
PB2_gene_3115	301	WP_111139068.1	301	300/301(99.67)	5.05e-223	LysR family transcriptional regulator [Pantoea sp. ARC607]	2027922	d__Bacteria;k__norank;p__Proteobacteria;c__Gammaproteobacteria;o__Enterobacterales;f__Erwiniaceae;g__Pantoea;s__Pantoea_sp._ARC607
PB2_gene_3119	410	WP_111139776.1	410	401/410(97.80)	3.75e-280	MFS transporter [Pantoea sp. ARC607]	2027922	d__Bacteria;k__norank;p__Proteobacteria;c__Gammaproteobacteria;o__Enterobacterales;f__Erwiniaceae;g__Pantoea;s__Pantoea_sp._ARC607
PB2_gene_3126	275	WP_019236610.1	317	270/274(98.54)	1.84e-197	hypothetical protein [Wolbachia pipientis]	955	d__Bacteria;k__norank;p__Proteobacteria;c__Alphaproteobacteria;o__Rickettsiales;f__Anaplasmataceae;g__Wolbachia;s__Wolbachia_pipientis

PB2_gene_3132	257	ORM85087.1	279	244/257(94.94)	1.43e-178	DgsA anti-repressor MtfA [Pantoea deleyi]	470932	d__Bacteria;k__norank;p__Proteobacteria;c__Gammaproteobacteria;o__Enterobacterales;f__Erwiniaceae;g__Pantoea;s__Pantoea_deleyi
PB2_gene_3137	129	WP_111138410.1	129	98/102(96.08)	3.85e-56	DUF1090 domain-containing protein [Pantoea sp. ARC607]	2027922	d__Bacteria;k__norank;p__Proteobacteria;c__Gammaproteobacteria;o__Enterobacterales;f__Erwiniaceae;g__Pantoea;s__Pantoea_sp._ARC607
PB2_gene_3138	256	ORM78404.1	256	235/256(91.80)	7.96e-168	transcriptional regulator [Pantoea deleyi]	470932	d__Bacteria;k__norank;p__Proteobacteria;c__Gammaproteobacteria;o__Enterobacterales;f__Erwiniaceae;g__Pantoea;s__Pantoea_deleyi
PB2_gene_3147	133	ORM80735.1	133	133/133(100.00)	1.61e-84	DNA-binding protein [Pantoea deleyi]	470932	d__Bacteria;k__norank;p__Proteobacteria;c__Gammaproteobacteria;o__Enterobacterales;f__Erwiniaceae;g__Pantoea;s__Pantoea_deleyi
PB2_gene_3148	522	ORM80736.1	530	518/522(99.23)	0	ABC-F family ATPase [Pantoea deleyi]	470932	d__Bacteria;k__norank;p__Proteobacteria;c__Gammaproteobacteria;o__Enterobacterales;f__Erwiniaceae;g__Pantoea;s__Pantoea_deleyi
PB2_gene_3156	115	ORM86117.1	105	96/105(91.43)	4.36e-67	hypothetical protein HA42_01380 [Pantoea deleyi]	470932	d__Bacteria;k__norank;p__Proteobacteria;c__Gammaproteobacteria;o__Enterobacterales;f__Erwiniaceae;g__Pantoea;s__Pantoea_deleyi
PB2_gene_3160	233	ORM86113.1	248	221/233(94.85)	4.74e-77	TonB system transport protein TonB [Pantoea deleyi]	470932	d__Bacteria;k__norank;p__Proteobacteria;c__Gammaproteobacteria;o__Enterobacterales;f__Erwiniaceae;g__Pantoea;s__Pantoea_deleyi
PB2_gene_3165	422	ORM74872.1	422	408/421(96.91)	2.10e-283	hypothetical protein HA42_21315 [Pantoea deleyi]	470932	d__Bacteria;k__norank;p__Proteobacteria;c__Gammaproteobacteria;o__Enterobacterales;f__Erwiniaceae;g__Pantoea;s__Pantoea_deleyi
PB2_gene_3166	473	WP_111141287.1	473	455/473(96.19)	0	M20 peptidase family dipeptidase [Pantoea sp. ARC607]	2027922	d__Bacteria;k__norank;p__Proteobacteria;c__Gammaproteobacteria;o__Enterobacterales;f__Erwiniaceae;g__Pantoea;s__Pantoea_sp._ARC607
PB2_gene_3178	190	ORM81276.1	190	188/190(98.95)	2.39e-134	L-threonylcarbamoyladenylate synthase type 1 TsaC [Pantoea deleyi]	470932	d__Bacteria;k__norank;p__Proteobacteria;c__Gammaproteobacteria;o__Enterobacterales;f__Erwiniaceae;g__Pantoea;s__Pantoea_deleyi
PB2_gene_3179	272	ORM81277.1	272	256/272(94.12)	2.31e-184	shikimate dehydrogenase [Pantoea deleyi]	470932	d__Bacteria;k__norank;p__Proteobacteria;c__Gammaproteobacteria;o__Enterobacterales;f__Erwiniaceae;g__Pantoea;s__Pantoea_deleyi
PB2_gene_3184	845	ORM81836.1	846	823/845(97.40)	0	nitrite reductase large subunit [Pantoea deleyi]	470932	d__Bacteria;k__norank;p__Proteobacteria;c__Gammaproteobacteria;o__Enterobacterales;f__Erwiniaceae;g__Pantoea;s__Pantoea_deleyi
PB2_gene_3187	857	WP_010402784.1	857	838/857(97.78)	0	alanine--tRNA ligase [Wolbachia endosymbiont of Nasonia vitripennis]	180837	d__Bacteria;k__norank;p__Proteobacteria;c__Alphaproteobacteria;o__Rickettsiales;f__Anaplasmataceae;g__Wolbachia;s__Wolbachia_endosymbiont_of_Nasonia_vitripennis
PB2_gene_3201	300	WP_111141216.1	300	293/300(97.67)	7.88e-206	30S ribosomal protein S6--L-glutamate ligase [Pantoea sp. ARC607]	2027922	d__Bacteria;k__norank;p__Proteobacteria;c__Gammaproteobacteria;o__Enterobacterales;f__Erwiniaceae;g__Pantoea;s__Pantoea_sp._ARC607
PB2_gene_3214	80	SUE06631.1	375	32/42(76.19)	6.34e-09	Purine ribonucleoside efflux pump nepI [Pantoea agglomerans]	549	d__Bacteria;k__norank;p__Proteobacteria;c__Gammaproteobacteria;o__Enterobacterales;f__Erwiniaceae;g__Pantoea;s__Pantoea_agglomerans
PB2_gene_3221	129	ORM78388.1	129	127/129(98.45)	4.34e-87	tautomerase [Pantoea deleyi]	470932	d__Bacteria;k__norank;p__Proteobacteria;c__Gammaproteobacteria;o__Enterobacterales;f__Erwiniaceae;g__Pantoea;s__Pantoea_deleyi
PB2_gene_3225	546	WP_115764766.1	546	534/546(97.80)	0	oligopeptide ABC transporter substrate-binding protein OppA [Pantoea agglomerans]	549	d__Bacteria;k__norank;p__Proteobacteria;c__Gammaproteobacteria;o__Enterobacterales;f__Erwiniaceae;g__Pantoea;s__Pantoea_agglomerans
PB2_gene_3232	133	OJH30898.1	294	129/133(96.99)	1.01e-83	Succinyl-CoA ligase [ADP-forming] subunit alpha [Wolbachia endosymbiont of Armadillidium vulgare]	77039	d__Bacteria;k__norank;p__Proteobacteria;c__Alphaproteobacteria;o__Rickettsiales;f__Anaplasmataceae;g__Wolbachia;s__Wolbachia_endosymbiont_of_Armadillidium_vulgare
PB2_gene_3233	321	ORM76581.1	321	317/320(99.06)	1.14e-220	antimicrobial peptide ABC transporter permease SapB [Pantoea deleyi]	470932	d__Bacteria;k__norank;p__Proteobacteria;c__Gammaproteobacteria;o__Enterobacterales;f__Erwiniaceae;g__Pantoea;s__Pantoea_deleyi
PB2_gene_3234	541	ORM76582.1	540	536/539(99.44)	0	peptide ABC transporter substrate-binding protein SapA [Pantoea deleyi]	470932	d__Bacteria;k__norank;p__Proteobacteria;c__Gammaproteobacteria;o__Enterobacterales;f__Erwiniaceae;g__Pantoea;s__Pantoea_deleyi
PB2_gene_3237	84	STV04709.1	215	51/74(68.92)	1.01e-23	prophage integrase [Klebsiella pneumoniae]	573	d__Bacteria;k__norank;p__Proteobacteria;c__Gammaproteobacteria;o__Enterobacterales;f__Enterobacteriaceae;g__Klebsiella;s__Klebsiella_pneumoniae

PB2_gene_3251	282	WP_111142203.1	281	227/281(80.78)	2.08e-162	hypothetical protein [Pantoea sp. ARC607]	2027922	d__Bacteria;k__norank;p__Proteobacteria;c__Gammaproteobacteria;o__Enterobacterales;f__Erwiniaceae;g__Pantoea;s__Pantoea_sp._ARC607
PB2_gene_3252	145	ORM77299.1	154	117/144(81.25)	1.99e-79	hypothetical protein HA42_17950 [Pantoea deleyi]	470932	d__Bacteria;k__norank;p__Proteobacteria;c__Gammaproteobacteria;o__Enterobacterales;f__Erwiniaceae;g__Pantoea;s__Pantoea_deleyi
PB2_gene_3254	502	ORM83256.1	502	502/502(100.00)	0	anion permease [Pantoea deleyi]	470932	d__Bacteria;k__norank;p__Proteobacteria;c__Gammaproteobacteria;o__Enterobacterales;f__Erwiniaceae;g__Pantoea;s__Pantoea_deleyi
PB2_gene_3255	548	ORM83255.1	548	544/548(99.27)	0	fumarate hydratase [Pantoea deleyi]	470932	d__Bacteria;k__norank;p__Proteobacteria;c__Gammaproteobacteria;o__Enterobacterales;f__Erwiniaceae;g__Pantoea;s__Pantoea_deleyi
PB2_gene_3258	633	WP_010405007.1	633	616/633(97.31)	0	molecular chaperone HscA [Wolbachia endosymbiont of Nasonia vitripennis]	180837	d__Bacteria;k__norank;p__Proteobacteria;c__Alphaproteobacteria;o__Rickettsiales;f__Anaplasmataceae;g__Wolbachia;s__Wolbachia_endosymbiont_of_Nasonia_vitripennis
PB2_gene_3262	336	WP_111142089.1	336	333/336(99.11)	3.55e-227	bile acid:sodium symporter [Pantoea sp. ARC607]	2027922	d__Bacteria;k__norank;p__Proteobacteria;c__Gammaproteobacteria;o__Enterobacterales;f__Erwiniaceae;g__Pantoea;s__Pantoea_sp._ARC607
PB2_gene_3272	68	WP_081276221.1	103	41/52(78.85)	3.51e-19	hypothetical protein [Pantoea agglomerans]	None	Unclassified
PB2_gene_3276	109	WP_078804792.1	109	103/109(94.50)	4.35e-64	MULTISPECIES: endoribonuclease SymE	53335	d__Bacteria;k__norank;p__Proteobacteria;c__Gammaproteobacteria;o__Enterobacterales;f__Erwiniaceae;g__Pantoea;s__norank
PB2_gene_3280	282	ORM86133.1	282	281/282(99.65)	2.18e-203	formyltetrahydrofolate deformylase [Pantoea deleyi]	470932	d__Bacteria;k__norank;p__Proteobacteria;c__Gammaproteobacteria;o__Enterobacterales;f__Erwiniaceae;g__Pantoea;s__Pantoea_deleyi
PB2_gene_3281	118	WP_111140031.1	151	118/118(100.00)	1.47e-89	MULTISPECIES: YchJ family protein [Pantoea]	53335	d__Bacteria;k__norank;p__Proteobacteria;c__Gammaproteobacteria;o__Enterobacterales;f__Erwiniaceae;g__Pantoea;s__norank
PB2_gene_3283	340	ORM86131.1	338	337/338(99.70)	4.38e-234	two-component system response regulator RssB [Pantoea deleyi]	470932	d__Bacteria;k__norank;p__Proteobacteria;c__Gammaproteobacteria;o__Enterobacterales;f__Erwiniaceae;g__Pantoea;s__Pantoea_deleyi
PB2_gene_3289	501	ORM84887.1	501	496/501(99.00)	0	lipase [Pantoea deleyi]	470932	d__Bacteria;k__norank;p__Proteobacteria;c__Gammaproteobacteria;o__Enterobacterales;f__Erwiniaceae;g__Pantoea;s__Pantoea_deleyi
PB2_gene_3290	329	ORM84886.1	329	328/329(99.70)	5.80e-218	sugar ABC transporter permease [Pantoea deleyi]	470932	d__Bacteria;k__norank;p__Proteobacteria;c__Gammaproteobacteria;o__Enterobacterales;f__Erwiniaceae;g__Pantoea;s__Pantoea_deleyi
PB2_gene_3297	433	ORM77790.1	433	433/433(100.00)	2.40e-290	glutamate/aspartate:proton symporter GltP [Pantoea deleyi]	470932	d__Bacteria;k__norank;p__Proteobacteria;c__Gammaproteobacteria;o__Enterobacterales;f__Erwiniaceae;g__Pantoea;s__Pantoea_deleyi
PB2_gene_3303	178	OQV39188.1	178	174/178(97.75)	7.22e-121	gluconokinase [Pantoea vagans]	470934	d__Bacteria;k__norank;p__Proteobacteria;c__Gammaproteobacteria;o__Enterobacterales;f__Erwiniaceae;g__Pantoea;s__Pantoea_vagans
PB2_gene_3304	446	WP_111140391.1	446	445/446(99.78)	1.09e-304	gluconate transporter [Pantoea sp. ARC607]	53335	d__Bacteria;k__norank;p__Proteobacteria;c__Gammaproteobacteria;o__Enterobacterales;f__Erwiniaceae;g__Pantoea;s__norank
PB2_gene_3307	323	WP_111140370.1	323	321/323(99.38)	3.13e-203	ABC transporter permease [Pantoea sp. ARC607]	53335	d__Bacteria;k__norank;p__Proteobacteria;c__Gammaproteobacteria;o__Enterobacterales;f__Erwiniaceae;g__Pantoea;s__norank
PB2_gene_3308	285	ORM81486.1	285	284/285(99.65)	6.14e-191	RpiR family transcriptional regulator [Pantoea deleyi]	470932	d__Bacteria;k__norank;p__Proteobacteria;c__Gammaproteobacteria;o__Enterobacterales;f__Erwiniaceae;g__Pantoea;s__Pantoea_deleyi
PB2_gene_3310	415	WP_111138810.1	751	373/415(89.88)	2.29e-257	DNA internalization-related competence protein ComEC/Rec2 [Pantoea sp. ARC607]	2027922	d__Bacteria;k__norank;p__Proteobacteria;c__Gammaproteobacteria;o__Enterobacterales;f__Erwiniaceae;g__Pantoea;s__Pantoea_sp._ARC607
PB2_gene_3311	94	SJZ32721.1	94	94/94(100.00)	8.42e-64	integration host factor subunit beta [Pantoea agglomerans]	470933	d__Bacteria;k__norank;p__Proteobacteria;c__Gammaproteobacteria;o__Enterobacterales;f__Erwiniaceae;g__Pantoea;s__Pantoea_eucalypti
PB2_gene_3314	870	WP_111140423.1	883	860/870(98.85)	0	phosphoenolpyruvate carboxylase [Pantoea sp. ARC607]	2027922	d__Bacteria;k__norank;p__Proteobacteria;c__Gammaproteobacteria;o__Enterobacterales;f__Erwiniaceae;g__Pantoea;s__Pantoea_sp._ARC607
PB2_gene_3316	506	WP_128084828.1	506	490/506(96.84)	0	bifunctional ADP-dependent NAD(P)H-hydrate dehydratase/NAD(P)H-hydrate epimerase [Pantoea deleyi]	470932	d__Bacteria;k__norank;p__Proteobacteria;c__Gammaproteobacteria;o__Enterobacterales;f__Erwiniaceae;g__Pantoea;s__Pantoea_deleyi
PB2_gene_3325	779	ORM76317.1	775	748/775(96.52)	0	CbbBc protein [Pantoea deleyi]	470932	d__Bacteria;k__norank;p__Proteobacteria;c__Gammaproteobacteria;o__Enterobacterales;f__Erwiniaceae;g__Pantoea;s__Pantoea_deleyi

PB2_gene_3330	144	SKA25342.1	144	144/144(100.00)	4.83e-89	LSU ribosomal protein L15P [Pantoea agglomerans]	470933	d__Bacteria;k__norank;p__Proteobacteria;c__Gammaproteobacteria;o__Enterobacterales;f__Erwiniaceae;g__Pantoea;s__Pantoea_eucalypti
PB2_gene_3336	271	ORM83050.1	271	270/271(99.63)	1.15e-188	methionine ABC transporter substrate-binding protein MetQ [Pantoea deleyi]	470932	d__Bacteria;k__norank;p__Proteobacteria;c__Gammaproteobacteria;o__Enterobacterales;f__Erwiniaceae;g__Pantoea;s__Pantoea_deleyi
PB2_gene_3338	458	WP_019236618.1	458	447/458(97.60)	0	malonyl-CoA decarboxylase [Wolbachia pipientis]	953	d__Bacteria;k__norank;p__Proteobacteria;c__Alphaproteobacteria;o__Rickettsiales;f__Anaplasmataceae;g__Wolbachia;s__norank
PB2_gene_3342	341	WP_063630919.1	341	339/341(99.41)	7.56e-236	FtsH protease activity modulator HflK [Wolbachia endosymbiont of Laodelphax striatella]	368602	d__Bacteria;k__norank;p__Proteobacteria;c__Alphaproteobacteria;o__Rickettsiales;f__Anaplasmataceae;g__Wolbachia;s__Wolbachia_endosymbiont_of_Laodelphax_striatellus
PB2_gene_3348	286	WP_090965439.1	286	247/286(86.36)	1.26e-179	alpha/beta fold hydrolase [Pantoea sp. OV426]	1881036	d__Bacteria;k__norank;p__Proteobacteria;c__Gammaproteobacteria;o__Enterobacterales;f__Erwiniaceae;g__Pantoea;s__Pantoea_sp._OV426
PB2_gene_3354	373	ORM76738.1	373	366/373(98.12)	5.64e-270	anhydro-N-acetylmuramic acid kinase [Pantoea deleyi]	470932	d__Bacteria;k__norank;p__Proteobacteria;c__Gammaproteobacteria;o__Enterobacterales;f__Erwiniaceae;g__Pantoea;s__Pantoea_deleyi
PB2_gene_3355	155	ORM76737.1	155	153/155(98.71)	1.43e-84	hypothetical protein HA42_18740 [Pantoea deleyi]	470932	d__Bacteria;k__norank;p__Proteobacteria;c__Gammaproteobacteria;o__Enterobacterales;f__Erwiniaceae;g__Pantoea;s__Pantoea_deleyi
PB2_gene_3356	305	ORM84119.1	306	294/305(96.39)	3.76e-209	LysR family transcriptional regulator [Pantoea deleyi]	470932	d__Bacteria;k__norank;p__Proteobacteria;c__Gammaproteobacteria;o__Enterobacterales;f__Erwiniaceae;g__Pantoea;s__Pantoea_deleyi
PB2_gene_3361	607	WP_063631182.1	689	534/606(88.12)	0	heme lyase CcmF/NrfE family subunit [Wolbachia endosymbiont of Laodelphax	368602	d__Bacteria;k__norank;p__Proteobacteria;c__Alphaproteobacteria;o__Rickettsiales;f__Anaplasmataceae;g__Wolbachia;s__Wolbachia_endosymbiont_of_Laodelphax_striatellus
PB2_gene_3364	367	SUB06460.1	330	293/328(89.33)	2.43e-204	putative DMT superfamily transporter inner membrane protein [Pantoea agglomerans]	549	d__Bacteria;k__norank;p__Proteobacteria;c__Gammaproteobacteria;o__Enterobacterales;f__Erwiniaceae;g__Pantoea;s__Pantoea_agglomerans
PB2_gene_3366	409	ORM81553.1	408	402/408(98.53)	9.89e-250	hypothetical protein HA42_10315 [Pantoea deleyi]	470932	d__Bacteria;k__norank;p__Proteobacteria;c__Gammaproteobacteria;o__Enterobacterales;f__Erwiniaceae;g__Pantoea;s__Pantoea_deleyi
PB2_gene_3373	558	ORM82572.1	558	520/558(93.19)	7.84e-311	methyl-accepting chemotaxis protein II [Pantoea deleyi]	470932	d__Bacteria;k__norank;p__Proteobacteria;c__Gammaproteobacteria;o__Enterobacterales;f__Erwiniaceae;g__Pantoea;s__Pantoea_deleyi
PB2_gene_3381	150	WP_111139881.1	428	132/138(95.65)	3.89e-71	TRAP transporter large permease [Pantoea sp. ARC607]	2027922	d__Bacteria;k__norank;p__Proteobacteria;c__Gammaproteobacteria;o__Enterobacterales;f__Erwiniaceae;g__Pantoea;s__Pantoea_sp._ARC607
PB2_gene_3382	240	ORM82185.1	246	235/240(97.92)	2.51e-153	tonB-system energizer ExbB [Pantoea deleyi]	470932	d__Bacteria;k__norank;p__Proteobacteria;c__Gammaproteobacteria;o__Enterobacterales;f__Erwiniaceae;g__Pantoea;s__Pantoea_deleyi
PB2_gene_3383	140	ORM82184.1	140	137/140(97.86)	8.44e-88	TonB system transport protein ExbD [Pantoea deleyi]	470932	d__Bacteria;k__norank;p__Proteobacteria;c__Gammaproteobacteria;o__Enterobacterales;f__Erwiniaceae;g__Pantoea;s__Pantoea_deleyi
PB2_gene_3385	264	ORM83020.1	264	256/264(96.97)	2.15e-185	vitamin B12 ABC transporter substrate-binding protein BtuF [Pantoea deleyi]	470932	d__Bacteria;k__norank;p__Proteobacteria;c__Gammaproteobacteria;o__Enterobacterales;f__Erwiniaceae;g__Pantoea;s__Pantoea_deleyi
PB2_gene_3387	496	ORM83022.1	496	494/496(99.60)	0	dGTPase [Pantoea deleyi]	470932	d__Bacteria;k__norank;p__Proteobacteria;c__Gammaproteobacteria;o__Enterobacterales;f__Erwiniaceae;g__Pantoea;s__Pantoea_deleyi
PB2_gene_3390	468	WP_111139393.1	468	463/468(98.93)	0	3-isopropylmalate dehydratase large subunit [Pantoea sp.	2027922	d__Bacteria;k__norank;p__Proteobacteria;c__Gammaproteobacteria;o__Enterobacterales;f__Erwiniaceae;g__Pantoea;s__Pantoea_sp._ARC607
PB2_gene_3393	1130	WP_083070393.1	1227	1063/1130(94.07)	0	methionine synthase [Pantoea vagans]	470934	d__Bacteria;k__norank;p__Proteobacteria;c__Gammaproteobacteria;o__Enterobacterales;f__Erwiniaceae;g__Pantoea;s__Pantoea_vagans
PB2_gene_3397	524	WP_111139237.1	519	489/519(94.22)	1.39e-269	methyl-accepting chemotaxis protein [Pantoea sp. ARC607]	2027922	d__Bacteria;k__norank;p__Proteobacteria;c__Gammaproteobacteria;o__Enterobacterales;f__Erwiniaceae;g__Pantoea;s__Pantoea_sp._ARC607
PB2_gene_3402	638	ORM79290.1	638	634/638(99.37)	0	ATP-dependent helicase [Pantoea deleyi]	470932	d__Bacteria;k__norank;p__Proteobacteria;c__Gammaproteobacteria;o__Enterobacterales;f__Erwiniaceae;g__Pantoea;s__Pantoea_deleyi
PB2_gene_3403	245	WP_111138871.1	245	227/245(92.65)	5.67e-167	carbonic anhydrase family protein [Pantoea sp. ARC607]	2027922	d__Bacteria;k__norank;p__Proteobacteria;c__Gammaproteobacteria;o__Enterobacterales;f__Erwiniaceae;g__Pantoea;s__Pantoea_sp._ARC607
PB2_gene_3408	77	ORM81163.1	77	77/77(100.00)	4.05e-43	hypothetical protein HA42_11680 [Pantoea deleyi]	470932	d__Bacteria;k__norank;p__Proteobacteria;c__Gammaproteobacteria;o__Enterobacterales;f__Erwiniaceae;g__Pantoea;s__Pantoea_deleyi

PB2_gene_3413	263	WP_111141514.1	263	258/263(98.10)	1.76e-179	urea ABC transporter ATP-binding protein UrtD [Pantoea sp.	2027922	d__Bacteria;k__norank;p__Proteobacteria;c__Gammaproteobacteria;o__Enterobacterales;f__Erwiniaceae;g__Pantoea;s__Pantoea_sp._ARC607
PB2_gene_3415	337	WP_111141510.1	337	331/337(98.22)	3.29e-253	DUF4432 family protein [Pantoea sp. ARC607]	2027922	d__Bacteria;k__norank;p__Proteobacteria;c__Gammaproteobacteria;o__Enterobacterales;f__Erwiniaceae;g__Pantoea;s__Pantoea_sp._ARC607
PB2_gene_3418	326	ORM77156.1	326	324/326(99.39)	1.72e-213	ribose ABC transporter permease [Pantoea deleyi]	470932	d__Bacteria;k__norank;p__Proteobacteria;c__Gammaproteobacteria;o__Enterobacterales;f__Erwiniaceae;g__Pantoea;s__Pantoea_deleyi
PB2_gene_3422	446	ORM82470.1	446	441/446(98.88)	6.59e-315	metalloprotease PmbA [Pantoea deleyi]	470932	d__Bacteria;k__norank;p__Proteobacteria;c__Gammaproteobacteria;o__Enterobacterales;f__Erwiniaceae;g__Pantoea;s__Pantoea_deleyi
PB2_gene_3424	129	ORM82468.1	111	107/111(96.40)	8.00e-68	glycine dehydrogenase [Pantoea deleyi]	470932	d__Bacteria;k__norank;p__Proteobacteria;c__Gammaproteobacteria;o__Enterobacterales;f__Erwiniaceae;g__Pantoea;s__Pantoea_deleyi
PB2_gene_3427	262	WP_111139472.1	262	262/262(100.00)	2.15e-184	enoyl-ACP reductase FabI [Pantoea sp. ARC607]	53335	d__Bacteria;k__norank;p__Proteobacteria;c__Gammaproteobacteria;o__Enterobacterales;f__Erwiniaceae;g__Pantoea;s__norank
PB2_gene_3428	267	ORM76578.1	267	267/267(100.00)	2.23e-184	peptide ABC transporter ATP-binding protein [Pantoea deleyi]	470932	d__Bacteria;k__norank;p__Proteobacteria;c__Gammaproteobacteria;o__Enterobacterales;f__Erwiniaceae;g__Pantoea;s__Pantoea_deleyi
PB2_gene_3434	330	ORM78925.1	330	325/330(98.48)	5.11e-233	lactate dehydrogenase [Pantoea deleyi]	470932	d__Bacteria;k__norank;p__Proteobacteria;c__Gammaproteobacteria;o__Enterobacterales;f__Erwiniaceae;g__Pantoea;s__Pantoea_deleyi
PB2_gene_3438	289	WP_111139852.1	291	283/287(98.61)	5.14e-206	DUF2236 domain-containing protein [Pantoea sp. ARC607]	2027922	d__Bacteria;k__norank;p__Proteobacteria;c__Gammaproteobacteria;o__Enterobacterales;f__Erwiniaceae;g__Pantoea;s__Pantoea_sp._ARC607
PB2_gene_3442	287	WP_108784143.1	287	285/287(99.30)	2.20e-210	lipoyl synthase [Wolbachia endosymbiont of Bemisia tabaci]	215173	d__Bacteria;k__norank;p__Proteobacteria;c__Alphaproteobacteria;o__Rickettsiales;f__Anaplasmataceae;g__Wolbachia;s__Wolbachia_endosymbiont_of_Bemisia_tabaci
PB2_gene_3446	198	ORM83154.1	198	194/198(97.98)	3.63e-133	oxidative-stress-resistance chaperone [Pantoea deleyi]	470932	d__Bacteria;k__norank;p__Proteobacteria;c__Gammaproteobacteria;o__Enterobacterales;f__Erwiniaceae;g__Pantoea;s__Pantoea_deleyi
PB2_gene_3456	525	ORM78940.1	525	507/525(96.57)	4.00e-308	chemotaxis protein [Pantoea deleyi]	470932	d__Bacteria;k__norank;p__Proteobacteria;c__Gammaproteobacteria;o__Enterobacterales;f__Erwiniaceae;g__Pantoea;s__Pantoea_deleyi
PB2_gene_3458	442	WP_125289965.1	441	345/441(78.23)	4.50e-258	putative aminohydrolase SsnA [Erwinia sp. 198]	2022746	d__Bacteria;k__norank;p__Proteobacteria;c__Gammaproteobacteria;o__Enterobacterales;f__Erwiniaceae;g__Erwinia;s__Erwinia_sp._198
PB2_gene_3467	355	WP_111142174.1	355	352/355(99.15)	1.12e-260	bifunctional histidinol-phosphatase/imidazoleglycerol-phosphate dehydratase HisB [Pantoea sp. ARC607]	2027922	d__Bacteria;k__norank;p__Proteobacteria;c__Gammaproteobacteria;o__Enterobacterales;f__Erwiniaceae;g__Pantoea;s__Pantoea_sp._ARC607
PB2_gene_3469	245	ORM85651.1	245	242/245(98.78)	7.19e-170	1-(5-phosphoribosyl)-5-[(5-phosphoribosylamino)methylideneamino]imidazole-4-carboxamide isomerase [Pantoea deleyi]	470932	d__Bacteria;k__norank;p__Proteobacteria;c__Gammaproteobacteria;o__Enterobacterales;f__Erwiniaceae;g__Pantoea;s__Pantoea_deleyi
PB2_gene_3471	237	ORM72656.1	236	234/236(99.15)	6.95e-166	IclR family transcriptional regulator [Pantoea deleyi]	470932	d__Bacteria;k__norank;p__Proteobacteria;c__Gammaproteobacteria;o__Enterobacterales;f__Erwiniaceae;g__Pantoea;s__Pantoea_deleyi
PB2_gene_3472	326	ORM72657.1	326	322/326(98.77)	6.47e-234	hypothetical protein HA42_22360 [Pantoea deleyi]	470932	d__Bacteria;k__norank;p__Proteobacteria;c__Gammaproteobacteria;o__Enterobacterales;f__Erwiniaceae;g__Pantoea;s__Pantoea_deleyi
PB2_gene_3479	80	KDA93256.1	92	53/76(69.74)	2.18e-28	hypothetical protein T296_16355 [Pantoea agglomerans Eh318]	1408177	d__Bacteria;k__norank;p__Proteobacteria;c__Gammaproteobacteria;o__Enterobacterales;f__Erwiniaceae;g__Pantoea;s__Pantoea_agglomerans
PB2_gene_3484	328	ORM82641.1	328	323/328(98.48)	4.02e-229	aldo/keto reductase [Pantoea deleyi]	470932	d__Bacteria;k__norank;p__Proteobacteria;c__Gammaproteobacteria;o__Enterobacterales;f__Erwiniaceae;g__Pantoea;s__Pantoea_deleyi
PB2_gene_3485	372	ORM82664.1	379	359/372(96.51)	3.61e-265	hypothetical protein HA42_07800 [Pantoea deleyi]	470932	d__Bacteria;k__norank;p__Proteobacteria;c__Gammaproteobacteria;o__Enterobacterales;f__Erwiniaceae;g__Pantoea;s__Pantoea_deleyi
PB2_gene_3488	464	WP_064085935.1	464	462/464(99.57)	0	cysteine--tRNA ligase [Wolbachia endosymbiont of Dactylopius coccus]	None	Unclassified
PB2_gene_3489	234	WP_010401348.1	924	233/234(99.57)	5.25e-151	DUF3971 domain-containing protein [Wolbachia endosymbiont of Nasonia vitripennis]	180837	d__Bacteria;k__norank;p__Proteobacteria;c__Alphaproteobacteria;o__Rickettsiales;f__Anaplasmataceae;g__Wolbachia;s__Wolbachia_endosymbiont_of_Nasonia_vitripennis

PB2_gene_3494	336	ORM82052.1	336	336/336(100.00)	7.16e-227	galactoside ABC transporter permease MglC [Pantoea deleyi]	470932	d__Bacteria;k__norank;p__Proteobacteria;c__Gammaproteobacteria;o__Enterobacterales;f__Erwiniaceae;g__Pantoea;s__Pantoea_deleyi
PB2_gene_3500	319	ORM84448.1	319	318/319(99.69)	3.17e-226	transcriptional regulator Cbl [Pantoea deleyi]	470932	d__Bacteria;k__norank;p__Proteobacteria;c__Gammaproteobacteria;o__Enterobacterales;f__Erwiniaceae;g__Pantoea;s__Pantoea_deleyi
PB2_gene_3501	485	SUB06755.1	485	448/485(92.37)	0	Succinate-semialdehyde dehydrogenase [NADP(+)] GabD [Pantoea agglomerans]	549	d__Bacteria;k__norank;p__Proteobacteria;c__Gammaproteobacteria;o__Enterobacterales;f__Erwiniaceae;g__Pantoea;s__Pantoea_agglomerans
PB2_gene_3502	56	WP_062757935.1	81	35/51(68.63)	2.18e-15	hypothetical protein [Pantoea agglomerans]	None	Unclassified
PB2_gene_3516	233	WP_111139818.1	383	225/233(96.57)	9.48e-156	8-amino-7-oxononanoate synthase [Pantoea sp. ARC607]	2027922	d__Bacteria;k__norank;p__Proteobacteria;c__Gammaproteobacteria;o__Enterobacterales;f__Erwiniaceae;g__Pantoea;s__Pantoea_sp._ARC607
PB2_gene_3517	251	WP_111139819.1	251	243/251(96.81)	8.74e-174	malonyl-ACP O-methyltransferase BioC [Pantoea	2027922	d__Bacteria;k__norank;p__Proteobacteria;c__Gammaproteobacteria;o__Enterobacterales;f__Erwiniaceae;g__Pantoea;s__Pantoea_sp._ARC607
PB2_gene_3519	240	ORM79884.1	240	240/240(100.00)	1.46e-171	ABC transporter ATP-binding protein [Pantoea deleyi]	470932	d__Bacteria;k__norank;p__Proteobacteria;c__Gammaproteobacteria;o__Enterobacterales;f__Erwiniaceae;g__Pantoea;s__Pantoea_deleyi
PB2_gene_3521	68	EFM19271.1	70	24/31(77.42)	3.11e-07	hypothetical protein PanABDRAFT_2866 [Pantoea sp. aB]	517433	d__Bacteria;k__norank;p__Proteobacteria;c__Gammaproteobacteria;o__Enterobacterales;f__Erwiniaceae;g__Pantoea;s__Pantoea_sp._aB
PB2_gene_3522	888	WP_111140096.1	890	869/870(99.89)	0	DNA topoisomerase (ATP-hydrolyzing) subunit A [Pantoea sp. ARC607]	2027922	d__Bacteria;k__norank;p__Proteobacteria;c__Gammaproteobacteria;o__Enterobacterales;f__Erwiniaceae;g__Pantoea;s__Pantoea_sp._ARC607
PB2_gene_3533	1083	WP_017531741.1	1178	1050/1083(96.95)	0	helicase SNF2 [Wolbachia endosymbiont of Diaphorina citri]	116598	d__Bacteria;k__norank;p__Proteobacteria;c__Alphaproteobacteria;o__Rickettsiales;f__Anaplasmataceae;g__Wolbachia;s__Wolbachia_endosymbiont_of_Diaphorina_citri
PB2_gene_3540	247	ORM77160.1	247	242/247(97.98)	8.74e-172	DNA-binding response regulator [Pantoea deleyi]	470932	d__Bacteria;k__norank;p__Proteobacteria;c__Gammaproteobacteria;o__Enterobacterales;f__Erwiniaceae;g__Pantoea;s__Pantoea_deleyi
PB2_gene_3543	255	ORM77223.1	274	251/255(98.43)	3.29e-180	sufurtransferase FdhD [Pantoea deleyi]	470932	d__Bacteria;k__norank;p__Proteobacteria;c__Gammaproteobacteria;o__Enterobacterales;f__Erwiniaceae;g__Pantoea;s__Pantoea_deleyi
PB2_gene_3546	203	ORM81176.1	201	193/201(96.02)	6.53e-138	glutathione transferase GstA [Pantoea deleyi]	470932	d__Bacteria;k__norank;p__Proteobacteria;c__Gammaproteobacteria;o__Enterobacterales;f__Erwiniaceae;g__Pantoea;s__Pantoea_deleyi
PB2_gene_3547	493	ORM81157.1	493	488/493(98.99)	0	dipeptide/tripeptide permease A [Pantoea deleyi]	470932	d__Bacteria;k__norank;p__Proteobacteria;c__Gammaproteobacteria;o__Enterobacterales;f__Erwiniaceae;g__Pantoea;s__Pantoea_deleyi
PB2_gene_3556	170	CCK10395.1	331	65/126(51.59)	3.31e-30	hypothetical protein BN126_539 [Cronobacter sakazakii 680]	1208592	d__Bacteria;k__norank;p__Proteobacteria;c__Gammaproteobacteria;o__Enterobacterales;f__Enterobacteriaceae;g__Cronobacter;s__Cronobacter_sakazakii
PB2_gene_3558	405	ORM81903.1	405	402/405(99.26)	2.48e-287	transcriptional regulator [Pantoea deleyi]	470932	d__Bacteria;k__norank;p__Proteobacteria;c__Gammaproteobacteria;o__Enterobacterales;f__Erwiniaceae;g__Pantoea;s__Pantoea_deleyi
PB2_gene_3559	297	ORM81904.1	297	289/297(97.31)	2.73e-200	LysR family transcriptional regulator [Pantoea deleyi]	470932	d__Bacteria;k__norank;p__Proteobacteria;c__Gammaproteobacteria;o__Enterobacterales;f__Erwiniaceae;g__Pantoea;s__Pantoea_deleyi
PB2_gene_3561	269	ORM78817.1	269	265/269(98.51)	1.11e-185	sugar-phosphatase [Pantoea deleyi]	470932	d__Bacteria;k__norank;p__Proteobacteria;c__Gammaproteobacteria;o__Enterobacterales;f__Erwiniaceae;g__Pantoea;s__Pantoea_deleyi
PB2_gene_3567	313	WP_094110179.1	306	228/303(75.25)	1.81e-170	hypothetical protein [Lonsdalea iberica]	1082703	d__Bacteria;k__norank;p__Proteobacteria;c__Gammaproteobacteria;o__Enterobacterales;f__Pectobacteriaceae;g__Lonsdalea;s__Lonsdalea_iberica
PB2_gene_3575	386	ORM78371.1	386	380/386(98.45)	3.14e-269	IscS subfamily cysteine desulfurase [Pantoea deleyi]	470932	d__Bacteria;k__norank;p__Proteobacteria;c__Gammaproteobacteria;o__Enterobacterales;f__Erwiniaceae;g__Pantoea;s__Pantoea_deleyi
PB2_gene_3578	144	WP_111140443.1	143	140/143(97.90)	2.23e-96	tripartite tricarboxylate transporter TctB family protein [Pantoea sp. ARC607]	2027922	d__Bacteria;k__norank;p__Proteobacteria;c__Gammaproteobacteria;o__Enterobacterales;f__Erwiniaceae;g__Pantoea;s__Pantoea_sp._ARC607
PB2_gene_3579	330	ORM80468.1	326	320/326(98.16)	1.79e-232	tricarboxylic transporter [Pantoea deleyi]	470932	d__Bacteria;k__norank;p__Proteobacteria;c__Gammaproteobacteria;o__Enterobacterales;f__Erwiniaceae;g__Pantoea;s__Pantoea_deleyi
PB2_gene_3583	405	ORM81382.1	398	394/398(98.99)	2.60e-269	Bcr/CflA family drug resistance efflux transporter [Pantoea	470932	d__Bacteria;k__norank;p__Proteobacteria;c__Gammaproteobacteria;o__Enterobacterales;f__Erwiniaceae;g__Pantoea;s__Pantoea_deleyi

PB2_gene_3587	506	WP_111140490.1	506	503/506(99.41)	0	sugar ABC transporter ATP-binding protein [Pantoea sp.	2027922	d__Bacteria;k__norank;p__Proteobacteria;c__Gammaproteobacteria;o__Enterobacterales;f__Erwiniaceae;g__Pantoea;s__Pantoea_sp._ARC607
PB2_gene_3591	221	ADO11643.1	221	218/221(98.64)	2.93e-148	Uncharacterized protein Pvag_3524 [Pantoea vagans C9-	712898	d__Bacteria;k__norank;p__Proteobacteria;c__Gammaproteobacteria;o__Enterobacterales;f__Erwiniaceae;g__Pantoea;s__Pantoea_vagans
PB2_gene_3592	397	WP_111140227.1	397	390/397(98.24)	2.11e-284	aspartate/tyrosine/aromatic aminotransferase [Pantoea sp. ARC607]	2027922	d__Bacteria;k__norank;p__Proteobacteria;c__Gammaproteobacteria;o__Enterobacterales;f__Erwiniaceae;g__Pantoea;s__Pantoea_sp._ARC607
PB2_gene_3597	275	ORM81822.1	275	268/275(97.45)	2.99e-177	taurine transporter subunit [Pantoea deleyi]	470932	d__Bacteria;k__norank;p__Proteobacteria;c__Gammaproteobacteria;o__Enterobacterales;f__Erwiniaceae;g__Pantoea;s__Pantoea_deleyi
PB2_gene_3612	778	YP_026154.1	2366	218/492(44.31)	9.98e-122	Afp18 (plasmid) [Serratia entomophila]	None	Unclassified
PB2_gene_3622	715	ORM86006.1	715	701/714(98.18)	0	bifunctional 2-acylglycerophosphoethanolamine acyltransferase/acyl-ACP synthetase [Pantoea deleyi]	470932	d__Bacteria;k__norank;p__Proteobacteria;c__Gammaproteobacteria;o__Enterobacterales;f__Erwiniaceae;g__Pantoea;s__Pantoea_deleyi
PB2_gene_3628	239	WP_111139439.1	239	225/239(94.14)	5.92e-166	cellulose synthase operon protein YhjQ [Pantoea sp. ARC607]	2027922	d__Bacteria;k__norank;p__Proteobacteria;c__Gammaproteobacteria;o__Enterobacterales;f__Erwiniaceae;g__Pantoea;s__Pantoea_sp._ARC607
PB2_gene_3629	65	ORM75439.1	65	65/65(100.00)	2.70e-38	hypothetical protein HA42_20685 [Pantoea deleyi]	470932	d__Bacteria;k__norank;p__Proteobacteria;c__Gammaproteobacteria;o__Enterobacterales;f__Erwiniaceae;g__Pantoea;s__Pantoea_deleyi
PB2_gene_3630	511	WP_111139437.1	512	469/510(91.96)	0	cellulose biosynthesis protein BcsE [Pantoea sp. ARC607]	2027922	d__Bacteria;k__norank;p__Proteobacteria;c__Gammaproteobacteria;o__Enterobacterales;f__Erwiniaceae;g__Pantoea;s__Pantoea_sp._ARC607
PB2_gene_3639	85	SJZ88142.1	85	85/85(100.00)	7.37e-54	Acid stress-induced BolA-like protein IbaG/YrbA, predicted regulator of iron metabolism [Pantoea agglomerans]	470933	d__Bacteria;k__norank;p__Proteobacteria;c__Gammaproteobacteria;o__Enterobacterales;f__Erwiniaceae;g__Pantoea;s__Pantoea_eucalypti
PB2_gene_3640	101	ORM86333.1	101	101/101(100.00)	6.96e-64	anti-sigma B factor antagonist [Pantoea deleyi]	470932	d__Bacteria;k__norank;p__Proteobacteria;c__Gammaproteobacteria;o__Enterobacterales;f__Erwiniaceae;g__Pantoea;s__Pantoea_deleyi
PB2_gene_3648	124	KPA06336.1	151	117/124(94.35)	2.56e-78	hypothetical protein PAP10c_2438 [Pantoea	549	d__Bacteria;k__norank;p__Proteobacteria;c__Gammaproteobacteria;o__Enterobacterales;f__Erwiniaceae;g__Pantoea;s__Pantoea_agglomerans
PB2_gene_3650	93	ADO08073.1	94	86/93(92.47)	8.45e-57	Baseplate assembly protein V (plasmid) [Pantoea vagans C9-1]	712898	d__Bacteria;k__norank;p__Proteobacteria;c__Gammaproteobacteria;o__Enterobacterales;f__Erwiniaceae;g__Pantoea;s__Pantoea_vagans
PB2_gene_3651	224	WP_041691885.1	198	172/198(86.87)	1.47e-116	hypothetical protein [Erwinia billingiae]	None	Unclassified
PB2_gene_3655	340	WP_111139279.1	340	328/340(96.47)	5.36e-240	alpha/beta hydrolase [Pantoea sp. ARC607]	2027922	d__Bacteria;k__norank;p__Proteobacteria;c__Gammaproteobacteria;o__Enterobacterales;f__Erwiniaceae;g__Pantoea;s__Pantoea_sp._ARC607
PB2_gene_3664	562	ORM81985.1	562	557/561(99.29)	0	transporter [Pantoea deleyi]	470932	d__Bacteria;k__norank;p__Proteobacteria;c__Gammaproteobacteria;o__Enterobacterales;f__Erwiniaceae;g__Pantoea;s__Pantoea_deleyi
PB2_gene_3668	253	WP_111139750.1	253	251/253(99.21)	3.05e-175	uridine phosphorylase [Pantoea sp. ARC607]	2027922	d__Bacteria;k__norank;p__Proteobacteria;c__Gammaproteobacteria;o__Enterobacterales;f__Erwiniaceae;g__Pantoea;s__Pantoea_sp._ARC607
PB2_gene_3671	317	ORM82825.1	317	315/317(99.37)	7.15e-222	3-oxoacyl-ACP synthase [Pantoea deleyi]	470932	d__Bacteria;k__norank;p__Proteobacteria;c__Gammaproteobacteria;o__Enterobacterales;f__Erwiniaceae;g__Pantoea;s__Pantoea_deleyi
PB2_gene_3672	343	WP_111138645.1	346	337/343(98.25)	1.34e-233	phosphate acyltransferase PlsX [Pantoea sp. ARC607]	2027922	d__Bacteria;k__norank;p__Proteobacteria;c__Gammaproteobacteria;o__Enterobacterales;f__Erwiniaceae;g__Pantoea;s__Pantoea_sp._ARC607
PB2_gene_3675	247	ORM82483.1	247	245/247(99.19)	5.32e-182	3'(2'),5'-bisphosphate nucleotidase CysQ [Pantoea deleyi]	470932	d__Bacteria;k__norank;p__Proteobacteria;c__Gammaproteobacteria;o__Enterobacterales;f__Erwiniaceae;g__Pantoea;s__Pantoea_deleyi
PB2_gene_3680	389	WP_111138464.1	389	381/389(97.94)	4.04e-278	ROK family transcriptional regulator [Pantoea sp. ARC607]	2027922	d__Bacteria;k__norank;p__Proteobacteria;c__Gammaproteobacteria;o__Enterobacterales;f__Erwiniaceae;g__Pantoea;s__Pantoea_sp._ARC607
PB2_gene_3682	257	WP_096097191.1	516	255/257(99.22)	2.93e-185	cytochrome c oxidase subunit I [Wolbachia pipientis]	955	d__Bacteria;k__norank;p__Proteobacteria;c__Alphaproteobacteria;o__Rickettsiales;f__Anaplasmataceae;g__Wolbachia;s__Wolbachia_pipientis

PB2_gene_3691	47	ERM09466.1	3473	46/47(97.87)	5.48e-19	filamentous hemagglutinin [Pantoea agglomerans Tx10]	1332072	d__Bacteria;k__norank;p__Proteobacteria;c__Gammaproteobacteria;o__Enterobacterales;f__Erwiniaceae;g__Pantoea;s__Pantoea_agglomerans
PB2_gene_3694	548	WP_089561897.1	545	473/539(87.76)	0	MULTISPECIES: sensor histidine kinase [Pantoea]	53335	d__Bacteria;k__norank;p__Proteobacteria;c__Gammaproteobacteria;o__Enterobacterales;f__Erwiniaceae;g__Pantoea;s__norank
PB2_gene_3712	359	ORM80556.1	359	357/359(99.44)	7.58e-261	3-deoxy-7-phosphoheptulonate synthase [Pantoea deleyi]	470932	d__Bacteria;k__norank;p__Proteobacteria;c__Gammaproteobacteria;o__Enterobacterales;f__Erwiniaceae;g__Pantoea;s__Pantoea_deleyi
PB2_gene_3717	175	ORM81139.1	175	175/175(100.00)	1.30e-92	peptidoglycan-associated lipoprotein [Pantoea deleyi]	470932	d__Bacteria;k__norank;p__Proteobacteria;c__Gammaproteobacteria;o__Enterobacterales;f__Erwiniaceae;g__Pantoea;s__Pantoea_deleyi
PB2_gene_3724	496	WP_111208838.1	500	455/500(91.00)	1.68e-315	serine/threonine protein kinase [Pantoea sp. ARC270]	2027923	d__Bacteria;k__norank;p__Proteobacteria;c__Gammaproteobacteria;o__Enterobacterales;f__Erwiniaceae;g__Pantoea;s__Pantoea_sp._ARC270
PB2_gene_3749	404	WP_111140684.1	403	335/403(83.13)	1.25e-238	acyltransferase [Pantoea sp. ARC607]	2027922	d__Bacteria;k__norank;p__Proteobacteria;c__Gammaproteobacteria;o__Enterobacterales;f__Erwiniaceae;g__Pantoea;s__Pantoea_sp._ARC607
PB2_gene_3752	349	ORM82744.1	349	348/349(99.71)	5.22e-247	two-component system sensor histidine kinase NtrB [Pantoea deleyi]	470932	d__Bacteria;k__norank;p__Proteobacteria;c__Gammaproteobacteria;o__Enterobacterales;f__Erwiniaceae;g__Pantoea;s__Pantoea_deleyi
PB2_gene_3765	311	ORM86010.1	311	307/311(98.71)	3.03e-220	aspartate carbamoyltransferase [Pantoea deleyi]	470932	d__Bacteria;k__norank;p__Proteobacteria;c__Gammaproteobacteria;o__Enterobacterales;f__Erwiniaceae;g__Pantoea;s__Pantoea_deleyi
PB2_gene_3778	382	OQV43480.1	382	380/382(99.48)	2.72e-277	alkanesulfonate monooxygenase, FMNH(2)-dependent [Pantoea vagans]	470934	d__Bacteria;k__norank;p__Proteobacteria;c__Gammaproteobacteria;o__Enterobacterales;f__Erwiniaceae;g__Pantoea;s__Pantoea_vagans
PB2_gene_3782	530	WP_119803833.1	543	290/533(54.41)	2.31e-191	hypothetical protein [Serratia sp. S40]	None	Unclassified
PB2_gene_3784	55	ROP62297.1	55	38/55(69.09)	3.22e-17	hypothetical protein EDF81_0782 [Enterobacter sp. BIGb0383]	2485118	d__Bacteria;k__norank;p__Proteobacteria;c__Gammaproteobacteria;o__Enterobacterales;f__Enterobacteriaceae;g__Enterobacter;s__Enterobacter_sp._BIGb0383
PB2_gene_3788	169	WP_111142187.1	507	167/169(98.82)	9.78e-112	apolipoprotein N-acyltransferase [Pantoea sp. ARC607]	2027922	d__Bacteria;k__norank;p__Proteobacteria;c__Gammaproteobacteria;o__Enterobacterales;f__Erwiniaceae;g__Pantoea;s__Pantoea_sp._ARC607
PB2_gene_3796	607	ORM82743.1	607	605/606(99.83)	0	GTP-binding protein TypA [Pantoea deleyi]	470932	d__Bacteria;k__norank;p__Proteobacteria;c__Gammaproteobacteria;o__Enterobacterales;f__Erwiniaceae;g__Pantoea;s__Pantoea_deleyi
PB2_gene_3800	520	WP_111138386.1	520	505/520(97.12)	0	pyridoxal-dependent decarboxylase [Pantoea sp.	2027922	d__Bacteria;k__norank;p__Proteobacteria;c__Gammaproteobacteria;o__Enterobacterales;f__Erwiniaceae;g__Pantoea;s__Pantoea_sp._ARC607
PB2_gene_3807	303	ORM77788.1	303	297/303(98.02)	1.51e-200	N-acetylmuramic acid 6-phosphate etherase [Pantoea	470932	d__Bacteria;k__norank;p__Proteobacteria;c__Gammaproteobacteria;o__Enterobacterales;f__Erwiniaceae;g__Pantoea;s__Pantoea_deleyi
PB2_gene_3812	151	WP_110330190.1	466	139/148(93.92)	1.41e-88	hypothetical protein [Pantoea sp. JKS000250]	1938795	d__Bacteria;k__norank;p__Proteobacteria;c__Gammaproteobacteria;o__Enterobacterales;f__Erwiniaceae;g__Pantoea;s__Pantoea_sp._JKS000250
PB2_gene_3833	460	ORM77871.1	460	451/460(98.04)	0	U32 family peptidase [Pantoea deleyi]	470932	d__Bacteria;k__norank;p__Proteobacteria;c__Gammaproteobacteria;o__Enterobacterales;f__Erwiniaceae;g__Pantoea;s__Pantoea_deleyi
PB2_gene_3834	205	CNU35085.1	182	53/136(38.97)	1.11e-21	Uncharacterised protein [Salmonella enterica subsp. enterica serovar	58097	d__Bacteria;k__norank;p__Proteobacteria;c__Gammaproteobacteria;o__Enterobacterales;f__Enterobacteriaceae;g__Salmonella;s__Salmonella_enterica
PB2_gene_3840	464	ORM84685.1	464	463/464(99.78)	0	D-serine/D-alanine/glycine transporter [Pantoea deleyi]	470932	d__Bacteria;k__norank;p__Proteobacteria;c__Gammaproteobacteria;o__Enterobacterales;f__Erwiniaceae;g__Pantoea;s__Pantoea_deleyi
PB2_gene_3846	210	ORM81156.1	210	207/210(98.57)	1.26e-148	endonuclease III [Pantoea deleyi]	470932	d__Bacteria;k__norank;p__Proteobacteria;c__Gammaproteobacteria;o__Enterobacterales;f__Erwiniaceae;g__Pantoea;s__Pantoea_deleyi
PB2_gene_3848	234	WP_111141137.1	230	204/226(90.27)	6.56e-146	hypothetical protein [Pantoea sp. ARC607]	2027922	d__Bacteria;k__norank;p__Proteobacteria;c__Gammaproteobacteria;o__Enterobacterales;f__Erwiniaceae;g__Pantoea;s__Pantoea_sp._ARC607
PB2_gene_3851	66	WP_090962986.1	66	66/66(100.00)	1.27e-41	MULTISPECIES: DUF2517 family protein [Pantoea]	53335	d__Bacteria;k__norank;p__Proteobacteria;c__Gammaproteobacteria;o__Enterobacterales;f__Erwiniaceae;g__Pantoea;s__norank
PB2_gene_3852	224	SJZ31226.1	224	218/223(97.76)	5.93e-154	two-component system, OmpR family, KDP operon response regulator KdpE [Pantoea agglomerans]	470933	d__Bacteria;k__norank;p__Proteobacteria;c__Gammaproteobacteria;o__Enterobacterales;f__Erwiniaceae;g__Pantoea;s__Pantoea_eucalypti

PB2_gene_3857	626	WP_111139376.1	627	613/627(97.77)	0	PhoX family phosphatase [Pantoea sp. ARC607]	2027922	d__Bacteria;k__norank;p__Proteobacteria;c__Gammaproteobacteria;o__Enterobacterales;f__Erwiniaceae;g__Pantoea;s__Pantoea_sp._ARC607
PB2_gene_3863	286	OXH80886.1	286	279/285(97.89)	1.98e-192	phosphonate ABC transporter, permease protein PhnE [Pantoea agglomerans]	549	d__Bacteria;k__norank;p__Proteobacteria;c__Gammaproteobacteria;o__Enterobacterales;f__Erwiniaceae;g__Pantoea;s__Pantoea_agglomerans
PB2_gene_3874	570	ORM85098.1	568	549/568(96.65)	0	chemotaxis protein [Pantoea deleyi]	470932	d__Bacteria;k__norank;p__Proteobacteria;c__Gammaproteobacteria;o__Enterobacterales;f__Erwiniaceae;g__Pantoea;s__Pantoea_deleyi
PB2_gene_3892	408	WP_010402133.1	408	406/408(99.51)	9.37e-289	MULTISPECIES: tRNA (N(6)-L-threonylcarbamoyladenosine(37)-C(2))-methylthiotransferase MtaB [Wolbachia]	2640676	d__Bacteria;k__norank;p__Proteobacteria;c__Alphaproteobacteria;o__Rickettsiales;f__Anaplasmataceae;g__Wolbachia;s__norank
PB2_gene_3895	363	ORM80609.1	303	297/303(98.02)	1.97e-207	osmoprotectant uptake system substrate-binding protein [Pantoea deleyi]	470932	d__Bacteria;k__norank;p__Proteobacteria;c__Gammaproteobacteria;o__Enterobacterales;f__Erwiniaceae;g__Pantoea;s__Pantoea_deleyi
PB2_gene_3897	461	WP_111141522.1	1209	450/461(97.61)	2.91e-288	urea carboxylase [Pantoea sp. ARC607]	2027922	d__Bacteria;k__norank;p__Proteobacteria;c__Gammaproteobacteria;o__Enterobacterales;f__Erwiniaceae;g__Pantoea;s__Pantoea_sp._ARC607
PB2_gene_3898	231	ORM75476.1	231	228/231(98.70)	4.49e-157	GntR family transcriptional regulator [Pantoea deleyi]	470932	d__Bacteria;k__norank;p__Proteobacteria;c__Gammaproteobacteria;o__Enterobacterales;f__Erwiniaceae;g__Pantoea;s__Pantoea_deleyi
PB2_gene_3905	328	ORM76684.1	328	321/328(97.87)	1.15e-228	hydrolase [Pantoea deleyi]	470932	d__Bacteria;k__norank;p__Proteobacteria;c__Gammaproteobacteria;o__Enterobacterales;f__Erwiniaceae;g__Pantoea;s__Pantoea_deleyi
PB2_gene_3908	469	WP_111140244.1	475	456/475(96.00)	0	serine/threonine protein kinase [Pantoea sp. ARC607]	2027922	d__Bacteria;k__norank;p__Proteobacteria;c__Gammaproteobacteria;o__Enterobacterales;f__Erwiniaceae;g__Pantoea;s__Pantoea_sp._ARC607
PB2_gene_3909	317	ORM80459.1	317	309/317(97.48)	4.02e-228	hypothetical protein HA42_14515 [Pantoea deleyi]	470932	d__Bacteria;k__norank;p__Proteobacteria;c__Gammaproteobacteria;o__Enterobacterales;f__Erwiniaceae;g__Pantoea;s__Pantoea_deleyi
PB2_gene_3912	384	WP_099257935.1	384	343/384(89.32)	4.76e-263	DUF2169 domain-containing protein [Pantoea agglomerans]	549	d__Bacteria;k__norank;p__Proteobacteria;c__Gammaproteobacteria;o__Enterobacterales;f__Erwiniaceae;g__Pantoea;s__Pantoea_agglomerans
PB2_gene_3927	582	ORM80784.1	582	581/582(99.83)	0	lipid ABC transporter permease/ATP-binding protein [Pantoea deleyi]	470932	d__Bacteria;k__norank;p__Proteobacteria;c__Gammaproteobacteria;o__Enterobacterales;f__Erwiniaceae;g__Pantoea;s__Pantoea_deleyi
PB2_gene_3934	458	ERM09464.1	440	431/440(97.95)	7.28e-291	adenine permease [Pantoea agglomerans Tx10]	1332072	d__Bacteria;k__norank;p__Proteobacteria;c__Gammaproteobacteria;o__Enterobacterales;f__Erwiniaceae;g__Pantoea;s__Pantoea_agglomerans
PB2_gene_3942	77	SUB19039.1	411	76/77(98.70)	5.24e-46	Probable ubiquinone biosynthesis protein UbiB [Pantoea	549	d__Bacteria;k__norank;p__Proteobacteria;c__Gammaproteobacteria;o__Enterobacterales;f__Erwiniaceae;g__Pantoea;s__Pantoea_agglomerans
PB2_gene_3944	260	KDA94880.1	260	259/260(99.62)	3.73e-184	ubiquinone/menaquinone biosynthesis methyltransferase [Pantoea agglomerans Eh318]	1408177	d__Bacteria;k__norank;p__Proteobacteria;c__Gammaproteobacteria;o__Enterobacterales;f__Erwiniaceae;g__Pantoea;s__Pantoea_agglomerans
PB2_gene_3946	929	KKB02732.1	952	434/946(45.88)	1.42e-225	hypothetical protein TN98_21080, partial [Pantoea	470931	d__Bacteria;k__norank;p__Proteobacteria;c__Gammaproteobacteria;o__Enterobacterales;f__Erwiniaceae;g__Pantoea;s__Pantoea_anthophila
PB2_gene_3950	223	ORM80918.1	223	216/223(96.86)	1.56e-144	hypothetical protein HA42_12535 [Pantoea deleyi]	470932	d__Bacteria;k__norank;p__Proteobacteria;c__Gammaproteobacteria;o__Enterobacterales;f__Erwiniaceae;g__Pantoea;s__Pantoea_deleyi
PB2_gene_3957	195	ORM82823.1	195	189/195(96.92)	1.95e-131	septum formation inhibitor Maf [Pantoea deleyi]	470932	d__Bacteria;k__norank;p__Proteobacteria;c__Gammaproteobacteria;o__Enterobacterales;f__Erwiniaceae;g__Pantoea;s__Pantoea_deleyi
PB2_gene_3958	318	ORM82822.1	318	317/318(99.69)	7.73e-222	23S rRNA pseudouridine(955/2504/2580) synthase [Pantoea deleyi]	470932	d__Bacteria;k__norank;p__Proteobacteria;c__Gammaproteobacteria;o__Enterobacterales;f__Erwiniaceae;g__Pantoea;s__Pantoea_deleyi
PB2_gene_3962	544	WP_095101017.1	542	402/542(74.17)	2.27e-300	carbohydrate porin [Enterobacter sp. 10-1]	543	d__Bacteria;k__norank;p__Proteobacteria;c__Gammaproteobacteria;o__Enterobacterales;f__Enterobacteriaceae;g__norank;s__norank

PB2_gene_3967	485	WP_063631106.1	470	456/470(97.02)	0	UDP-N-acetylmuramoyl-L-alanyl-D-glutamate--2,6-diaminopimelate ligase [Wolbachia endosymbiont of	2640676	d__Bacteria;k__norank;p__Proteobacteria;c__Alphaproteobacteria;o__Rickettsiales;f__Anaplasmataceae;g__Wolbachia;s__norank
PB2_gene_3970	221	WP_111140299.1	222	214/222(96.40)	3.55e-147	ABC transporter ATP-binding protein [Pantoea sp. ARC607]	2027922	d__Bacteria;k__norank;p__Proteobacteria;c__Gammaproteobacteria;o__Enterobacterales;f__Erwiniaceae;g__Pantoea;s__Pantoea_sp._ARC607
PB2_gene_3975	874	WP_111140072.1	874	868/874(99.31)	0	type I DNA topoisomerase [Pantoea sp. ARC607]	2027922	d__Bacteria;k__norank;p__Proteobacteria;c__Gammaproteobacteria;o__Enterobacterales;f__Erwiniaceae;g__Pantoea;s__Pantoea_sp._ARC607
PB2_gene_3981	692	ORM85046.1	1173	667/692(96.39)	0	O-antigen biosynthesis protein [Pantoea deleyi]	470932	d__Bacteria;k__norank;p__Proteobacteria;c__Gammaproteobacteria;o__Enterobacterales;f__Erwiniaceae;g__Pantoea;s__Pantoea_deleyi
PB2_gene_3982	180	WP_111139961.1	401	176/180(97.78)	9.03e-79	FliC/FljB family flagellin [Pantoea sp. ARC607]	2027922	d__Bacteria;k__norank;p__Proteobacteria;c__Gammaproteobacteria;o__Enterobacterales;f__Erwiniaceae;g__Pantoea;s__Pantoea_sp._ARC607
PB2_gene_3991	258	WP_094120579.1	258	255/258(98.84)	4.00e-178	DUF4311 domain-containing protein [Pantoea conspicua]	472705	d__Bacteria;k__norank;p__Proteobacteria;c__Gammaproteobacteria;o__Enterobacterales;f__Erwiniaceae;g__Pantoea;s__Pantoea_conspicua
PB2_gene_3993	295	CDL59978.1	140	55/119(46.22)	3.23e-26	hypothetical protein [Klebsiella pneumoniae IS39]	1432561	d__Bacteria;k__norank;p__Proteobacteria;c__Gammaproteobacteria;o__Enterobacterales;f__Enterobacteriaceae;g__Klebsiella;s__Klebsiella_pneumoniae
PB2_gene_3997	69	WP_038227351.1	60	55/60(91.67)	2.95e-31	MULTISPECIES: hypothetical protein [Wolbachia]	2640676	d__Bacteria;k__norank;p__Proteobacteria;c__Alphaproteobacteria;o__Rickettsiales;f__Anaplasmataceae;g__Wolbachia;s__norank
PB2_gene_4000	434	ORM85654.1	434	428/434(98.62)	1.26e-297	histidinol dehydrogenase [Pantoea deleyi]	470932	d__Bacteria;k__norank;p__Proteobacteria;c__Gammaproteobacteria;o__Enterobacterales;f__Erwiniaceae;g__Pantoea;s__Pantoea_deleyi
PB2_gene_4011	697	WP_111139802.1	697	694/697(99.57)	0	flagellar biosynthesis protein FlhA [Pantoea sp. ARC607]	2027922	d__Bacteria;k__norank;p__Proteobacteria;c__Gammaproteobacteria;o__Enterobacterales;f__Erwiniaceae;g__Pantoea;s__Pantoea_sp._ARC607
PB2_gene_4014	219	PZL87095.1	219	217/219(99.09)	2.51e-148	two-component system response regulator UvrY [Pantoea sp. ARC270]	2027923	d__Bacteria;k__norank;p__Proteobacteria;c__Gammaproteobacteria;o__Enterobacterales;f__Erwiniaceae;g__Pantoea;s__Pantoea_sp._ARC270
PB2_gene_4020	652	WP_111139432.1	1267	635/652(97.39)	0	tetratricopeptide repeat protein [Pantoea sp. ARC607]	2027922	d__Bacteria;k__norank;p__Proteobacteria;c__Gammaproteobacteria;o__Enterobacterales;f__Erwiniaceae;g__Pantoea;s__Pantoea_sp._ARC607
PB2_gene_4023	373	ORM82463.1	373	359/373(96.25)	8.09e-254	L-seryl-tRNA selenium transferase [Pantoea deleyi]	470932	d__Bacteria;k__norank;p__Proteobacteria;c__Gammaproteobacteria;o__Enterobacterales;f__Erwiniaceae;g__Pantoea;s__Pantoea_deleyi
PB2_gene_4026	569	WP_111138597.1	569	551/569(96.84)	0	glycosyltransferase family 2 protein [Pantoea sp. ARC607]	2027922	d__Bacteria;k__norank;p__Proteobacteria;c__Gammaproteobacteria;o__Enterobacterales;f__Erwiniaceae;g__Pantoea;s__Pantoea_sp._ARC607
PB2_gene_4038	359	WP_111138693.1	356	333/356(93.54)	9.72e-245	lipopolysaccharide heptosyltransferase family protein [Pantoea sp. ARC607]	2027922	d__Bacteria;k__norank;p__Proteobacteria;c__Gammaproteobacteria;o__Enterobacterales;f__Erwiniaceae;g__Pantoea;s__Pantoea_sp._ARC607
PB2_gene_4044	392	WP_111142081.1	392	380/392(96.94)	1.71e-276	2-octaprenyl-6-methoxyphenyl hydroxylase [Pantoea sp.	2027922	d__Bacteria;k__norank;p__Proteobacteria;c__Gammaproteobacteria;o__Enterobacterales;f__Erwiniaceae;g__Pantoea;s__Pantoea_sp._ARC607
PB2_gene_4052	420	KPA07377.1	420	410/420(97.62)	1.21e-297	D-alanyl-D-alanine carboxypeptidase DacC [Pantoea	549	d__Bacteria;k__norank;p__Proteobacteria;c__Gammaproteobacteria;o__Enterobacterales;f__Erwiniaceae;g__Pantoea;s__Pantoea_agglomerans
PB2_gene_4064	387	CAQ54210.1	387	384/387(99.22)	1.08e-281	phage major capsid protein, HK97 family [Wolbachia endosymbiont of Culex	570417	d__Bacteria;k__norank;p__Proteobacteria;c__Alphaproteobacteria;o__Rickettsiales;f__Anaplasmataceae;g__Wolbachia;s__Wolbachia_endosymbiont_of_Culex_quinquefasciatus
PB2_gene_4069	342	WP_083067999.1	458	320/342(93.57)	9.03e-215	purine permease [Pantoea vagans]	470934	d__Bacteria;k__norank;p__Proteobacteria;c__Gammaproteobacteria;o__Enterobacterales;f__Erwiniaceae;g__Pantoea;s__Pantoea_vagans
PB2_gene_4076	548	ORM80439.1	548	548/548(100.00)	0	membrane protein insertase YidC [Pantoea deleyi]	470932	d__Bacteria;k__norank;p__Proteobacteria;c__Gammaproteobacteria;o__Enterobacterales;f__Erwiniaceae;g__Pantoea;s__Pantoea_deleyi
PB2_gene_4080	456	ORM86854.1	456	447/456(98.03)	5.61e-303	multidrug MFS transporter [Pantoea deleyi]	470932	d__Bacteria;k__norank;p__Proteobacteria;c__Gammaproteobacteria;o__Enterobacterales;f__Erwiniaceae;g__Pantoea;s__Pantoea_deleyi
PB2_gene_4084	529	ORM83259.1	529	523/529(98.87)	0	two-component system sensor histidine kinase DcuS [Pantoea deleyi]	470932	d__Bacteria;k__norank;p__Proteobacteria;c__Gammaproteobacteria;o__Enterobacterales;f__Erwiniaceae;g__Pantoea;s__Pantoea_deleyi

PB2_gene_4085	239	ORM83258.1	239	236/239(98.74)	1.35e-171	two-component system response regulator DcuR [Pantoea deleyi]	470932	d__Bacteria;k__norank;p__Proteobacteria;c__Gammaproteobacteria;o__Enterobacterales;f__Erwiniaceae;g__Pantoea;s__Pantoea_deleyi
PB2_gene_4093	286	ORM76574.1	286	278/285(97.54)	8.79e-196	6-phosphogluconate dehydrogenase [Pantoea deleyi]	470932	d__Bacteria;k__norank;p__Proteobacteria;c__Gammaproteobacteria;o__Enterobacterales;f__Erwiniaceae;g__Pantoea;s__Pantoea_deleyi
PB2_gene_4099	530	WP_111141798.1	551	526/530(99.25)	0	malonate decarboxylase subunit alpha [Pantoea sp. ARC607]	2027922	d__Bacteria;k__norank;p__Proteobacteria;c__Gammaproteobacteria;o__Enterobacterales;f__Erwiniaceae;g__Pantoea;s__Pantoea_sp._ARC607
PB2_gene_4101	241	ORM79040.1	241	241/241(100.00)	2.93e-167	arginine transporter ATP-binding subunit [Pantoea deleyi]	470932	d__Bacteria;k__norank;p__Proteobacteria;c__Gammaproteobacteria;o__Enterobacterales;f__Erwiniaceae;g__Pantoea;s__Pantoea_deleyi
PB2_gene_4103	253	ORM79038.1	246	245/246(99.59)	7.89e-174	amino acid ABC transporter permease [Pantoea deleyi]	470932	d__Bacteria;k__norank;p__Proteobacteria;c__Gammaproteobacteria;o__Enterobacterales;f__Erwiniaceae;g__Pantoea;s__Pantoea_deleyi
PB2_gene_4114	243	ORM78932.1	243	241/243(99.18)	6.42e-172	chromophore lyase [Pantoea deleyi]	470932	d__Bacteria;k__norank;p__Proteobacteria;c__Gammaproteobacteria;o__Enterobacterales;f__Erwiniaceae;g__Pantoea;s__Pantoea_deleyi
PB2_gene_4134	150	ORM78717.1	120	113/120(94.17)	1.18e-65	homeobox protein YbgS [Pantoea deleyi]	470932	d__Bacteria;k__norank;p__Proteobacteria;c__Gammaproteobacteria;o__Enterobacterales;f__Erwiniaceae;g__Pantoea;s__Pantoea_deleyi
PB2_gene_4145	499	OJH30323.1	499	450/497(90.54)	0	DNA-invertase hin [Armadillidium vulgare]	77039	d__Bacteria;k__norank;p__Proteobacteria;c__Alphaproteobacteria;o__Rickettsiales;f__Anaplasmataceae;g__Wolbachia;s__Wolbachia_endosymbiont_of_Armadillidium_vulgare
PB2_gene_4150	446	ORM86129.1	446	444/446(99.55)	0	UDP-glucose 6-dehydrogenase [Pantoea deleyi]	470932	d__Bacteria;k__norank;p__Proteobacteria;c__Gammaproteobacteria;o__Enterobacterales;f__Erwiniaceae;g__Pantoea;s__Pantoea_deleyi
PB2_gene_4155	51	WP_065095400.1	96	38/56(67.86)	1.50e-14	MULTISPECIES: type II toxin-antitoxin system RelE/ParE family toxin [Wolbachia]	2640676	d__Bacteria;k__norank;p__Proteobacteria;c__Alphaproteobacteria;o__Rickettsiales;f__Anaplasmataceae;g__Wolbachia;s__norank
PB2_gene_4158	264	WP_111140285.1	264	260/264(98.48)	2.70e-179	3-methyl-2-oxobutanoate hydroxymethyltransferase [Pantoea sp. ARC607]	2027922	d__Bacteria;k__norank;p__Proteobacteria;c__Gammaproteobacteria;o__Enterobacterales;f__Erwiniaceae;g__Pantoea;s__Pantoea_sp._ARC607
PB2_gene_4159	161	ORM77979.1	161	157/161(97.52)	9.04e-109	2-amino-4-hydroxy-6-hydroxymethyldihydropteridine diphosphokinase [Pantoea deleyi]	470932	d__Bacteria;k__norank;p__Proteobacteria;c__Gammaproteobacteria;o__Enterobacterales;f__Erwiniaceae;g__Pantoea;s__Pantoea_deleyi
PB2_gene_4162	361	WP_111141692.1	361	359/361(99.45)	6.24e-261	DNA replication/repair protein RecF [Pantoea sp. ARC607]	2027922	d__Bacteria;k__norank;p__Proteobacteria;c__Gammaproteobacteria;o__Enterobacterales;f__Erwiniaceae;g__Pantoea;s__Pantoea_sp._ARC607
PB2_gene_4164	136	BBH85244.1	270	93/136(68.38)	1.23e-60	DNA gyrase subunit B, partial [Erwinia rhapontici]	55212	d__Bacteria;k__norank;p__Proteobacteria;c__Gammaproteobacteria;o__Enterobacterales;f__Erwiniaceae;g__Erwinia;s__Erwinia_rhapontici
PB2_gene_4165	384	ORM85048.1	471	378/384(98.44)	5.67e-237	flagellar filament capping protein FliD [Pantoea deleyi]	470932	d__Bacteria;k__norank;p__Proteobacteria;c__Gammaproteobacteria;o__Enterobacterales;f__Erwiniaceae;g__Pantoea;s__Pantoea_deleyi
PB2_gene_4172	256	ORM82208.1	261	249/256(97.27)	1.70e-186	4,5-DOPA dioxygenase extradiol [Pantoea deleyi]	470932	d__Bacteria;k__norank;p__Proteobacteria;c__Gammaproteobacteria;o__Enterobacterales;f__Erwiniaceae;g__Pantoea;s__Pantoea_deleyi
PB2_gene_4175	184	WP_050861775.1	429	176/184(95.65)	1.08e-105	VRR-NUC domain-containing protein [Enterobacter kobei]	208224	d__Bacteria;k__norank;p__Proteobacteria;c__Gammaproteobacteria;o__Enterobacterales;f__Enterobacteriaceae;g__Enterobacter;s__Enterobacter_kobei
PB2_gene_4176	409	WP_126534693.1	404	388/402(96.52)	1.41e-296	DUF3396 domain-containing protein [Enterobacter sp.	2497436	d__Bacteria;k__norank;p__Proteobacteria;c__Gammaproteobacteria;o__Enterobacterales;f__Enterobacteriaceae;g__Enterobacter;s__Enterobacter_sp._WCHEn090040
PB2_gene_4183	531	WP_128085431.1	878	466/497(93.76)	0	hypothetical protein [Pantoea deleyi]	470932	d__Bacteria;k__norank;p__Proteobacteria;c__Gammaproteobacteria;o__Enterobacterales;f__Erwiniaceae;g__Pantoea;s__Pantoea_deleyi
PB2_gene_4187	584	WP_031590365.1	584	558/584(95.55)	0	amylovoran biosynthesis protein AmsF [Pantoea agglomerans]	53335	d__Bacteria;k__norank;p__Proteobacteria;c__Gammaproteobacteria;o__Enterobacterales;f__Erwiniaceae;g__Pantoea;s__norank
PB2_gene_4188	71	KKD34242.1	69	66/69(95.65)	2.51e-43	hypothetical protein EP46_10325 [Pantoea sp. 3.5.1]	1522060	d__Bacteria;k__norank;p__Proteobacteria;c__Gammaproteobacteria;o__Enterobacterales;f__Erwiniaceae;g__Pantoea;s__Pantoea_sp._3.5.1
PB2_gene_4205	384	ORM77130.1	384	379/384(98.70)	3.08e-259	MFS transporter [Pantoea deleyi]	470932	d__Bacteria;k__norank;p__Proteobacteria;c__Gammaproteobacteria;o__Enterobacterales;f__Erwiniaceae;g__Pantoea;s__Pantoea_deleyi
PB2_gene_4213	407	WP_111138955.1	407	403/407(99.02)	1.88e-308	cupin domain-containing protein [Pantoea sp. ARC607]	2027922	d__Bacteria;k__norank;p__Proteobacteria;c__Gammaproteobacteria;o__Enterobacterales;f__Erwiniaceae;g__Pantoea;s__Pantoea_sp._ARC607
PB2_gene_4226	111	WP_111140501.1	111	107/111(96.40)	9.38e-78	gamma-glutamylcyclotransferase [Pantoea sp. ARC607]	2027922	d__Bacteria;k__norank;p__Proteobacteria;c__Gammaproteobacteria;o__Enterobacterales;f__Erwiniaceae;g__Pantoea;s__Pantoea_sp._ARC607

PB2_gene_4227	110	PVI50923.1	110	100/110(90.91)	4.17e-67	DUF1493 domain-containing protein [Salmonella enterica subsp. enterica serovar	913079	d__Bacteria;k__norank;p__Proteobacteria;c__Gammaproteobacteria;o__Enterobacterales;f__Enterobacteriaceae;g__Salmonella;s__Salmonella_enterica
PB2_gene_4231	770	WP_111139874.1	814	739/762(96.98)	0	fimbrial biogenesis outer membrane usher protein [Pantoea	2027922	d__Bacteria;k__norank;p__Proteobacteria;c__Gammaproteobacteria;o__Enterobacterales;f__Erwiniaceae;g__Pantoea;s__Pantoea_sp._ARC607
PB2_gene_4252	234	ORM80909.1	232	228/232(98.28)	8.23e-145	protein CsuC [Pantoea deleyi]	470932	d__Bacteria;k__norank;p__Proteobacteria;c__Gammaproteobacteria;o__Enterobacterales;f__Erwiniaceae;g__Pantoea;s__Pantoea_deleyi
PB2_gene_4255	187	ORM76532.1	187	178/187(95.19)	1.89e-125	DNA-binding protein [Pantoea deleyi]	470932	d__Bacteria;k__norank;p__Proteobacteria;c__Gammaproteobacteria;o__Enterobacterales;f__Erwiniaceae;g__Pantoea;s__Pantoea_deleyi
PB2_gene_4258	459	WP_061061212.1	471	457/459(99.56)	0	chromosomal replication initiator protein DnaA [Pantoea vagans]	470934	d__Bacteria;k__norank;p__Proteobacteria;c__Gammaproteobacteria;o__Enterobacterales;f__Erwiniaceae;g__Pantoea;s__Pantoea_vagans
PB2_gene_4262	307	ORM75789.1	307	300/307(97.72)	3.88e-205	amino acid ABC transporter permease [Pantoea deleyi]	470932	d__Bacteria;k__norank;p__Proteobacteria;c__Gammaproteobacteria;o__Enterobacterales;f__Erwiniaceae;g__Pantoea;s__Pantoea_deleyi
PB2_gene_4263	65	BAN96978.1	135	62/65(95.38)	8.63e-37	ABC transporter ATP-binding protein [Plautia stali symbiont]	891974	d__Bacteria;k__norank;p__Proteobacteria;c__Gammaproteobacteria;o__Enterobacterales;f__Enterobacteriaceae;g__norank;s__Plautia_stali_symbiont
PB2_gene_4269	455	WP_111139849.1	465	406/455(89.23)	2.71e-315	outer membrane porin, OprD family [Pantoea sp. ARC607]	2027922	d__Bacteria;k__norank;p__Proteobacteria;c__Gammaproteobacteria;o__Enterobacterales;f__Erwiniaceae;g__Pantoea;s__Pantoea_sp._ARC607
PB2_gene_4279	671	WP_019236953.1	672	667/672(99.26)	0	primase [Wolbachia pipientis]	955	d__Bacteria;k__norank;p__Proteobacteria;c__Alphaproteobacteria;o__Rickettsiales;f__Anaplasmataceae;g__Wolbachia;s__Wolbachia_pipientis
PB2_gene_4288	46	CCF09261.1	263	40/46(86.96)	1.16e-18	hypothetical protein PANA5342_1868 [Pantoea ananatis LMG 5342]	1123863	d__Bacteria;k__norank;p__Proteobacteria;c__Gammaproteobacteria;o__Enterobacterales;f__Erwiniaceae;g__Pantoea;s__Pantoea_ananatis
PB2_gene_4292	222	PMT94608.1	222	126/210(60.00)	5.96e-86	hypothetical protein AFB28_20625 [Klebsiella sp.	2071634	d__Bacteria;k__norank;p__Proteobacteria;c__Gammaproteobacteria;o__Enterobacterales;f__Enterobacteriaceae;g__Klebsiella;s__Klebsiella_sp._Kd70_TUC-EEAOC
PB2_gene_4295	214	WP_111140316.1	214	213/214(99.53)	9.91e-148	adenylate kinase [Pantoea sp. ARC607]	2027922	d__Bacteria;k__norank;p__Proteobacteria;c__Gammaproteobacteria;o__Enterobacterales;f__Erwiniaceae;g__Pantoea;s__Pantoea_sp._ARC607
PB2_gene_4299	289	ORM85861.1	289	283/289(97.92)	9.63e-206	LysR family transcriptional regulator [Pantoea deleyi]	470932	d__Bacteria;k__norank;p__Proteobacteria;c__Gammaproteobacteria;o__Enterobacterales;f__Erwiniaceae;g__Pantoea;s__Pantoea_deleyi
PB2_gene_4306	150	WP_123346431.1	149	129/147(87.76)	7.30e-89	MULTISPECIES: phage tail protein [Enterobacter]	2608935	d__Bacteria;k__norank;p__Proteobacteria;c__Gammaproteobacteria;o__Enterobacterales;f__Enterobacteriaceae;g__Enterobacter;s__norank
PB2_gene_4307	189	ORM85238.1	189	175/189(92.59)	1.55e-125	pyridoxamine 5'-phosphate oxidase [Pantoea deleyi]	470932	d__Bacteria;k__norank;p__Proteobacteria;c__Gammaproteobacteria;o__Enterobacterales;f__Erwiniaceae;g__Pantoea;s__Pantoea_deleyi
PB2_gene_4310	164	WP_111141437.1	164	159/164(96.95)	6.31e-106	nicotinamide-nucleotide amidase [Pantoea sp. ARC607]	2027922	d__Bacteria;k__norank;p__Proteobacteria;c__Gammaproteobacteria;o__Enterobacterales;f__Erwiniaceae;g__Pantoea;s__Pantoea_sp._ARC607
PB2_gene_4316	545	WP_111139384.1	968	542/545(99.45)	0	RNA polymerase-associated protein RapA [Pantoea sp.	2027922	d__Bacteria;k__norank;p__Proteobacteria;c__Gammaproteobacteria;o__Enterobacterales;f__Erwiniaceae;g__Pantoea;s__Pantoea_sp._ARC607
PB2_gene_4324	332	WP_061063322.1	332	302/332(90.96)	3.84e-197	iron ABC transporter permease [Pantoea vagans]	470934	d__Bacteria;k__norank;p__Proteobacteria;c__Gammaproteobacteria;o__Enterobacterales;f__Erwiniaceae;g__Pantoea;s__Pantoea_vagans
PB2_gene_4353	194	WP_105591718.1	194	156/194(80.41)	5.25e-115	D-alanyl-D-alanine dipeptidase [Pantoea coffeiphila]	1465635	d__Bacteria;k__norank;p__Proteobacteria;c__Gammaproteobacteria;o__Enterobacterales;f__Erwiniaceae;g__Pantoea;s__Pantoea_coffeiphila
PB2_gene_4356	185	ORM82386.1	185	182/185(98.38)	1.20e-136	hypothetical protein HA42_08470 [Pantoea deleyi]	470932	d__Bacteria;k__norank;p__Proteobacteria;c__Gammaproteobacteria;o__Enterobacterales;f__Erwiniaceae;g__Pantoea;s__Pantoea_deleyi
PB2_gene_4376	158	WP_111138603.1	158	151/158(95.57)	9.84e-107	3-hydroxy-fatty acyl-ACP dehydratase [Pantoea sp.	2027922	d__Bacteria;k__norank;p__Proteobacteria;c__Gammaproteobacteria;o__Enterobacterales;f__Erwiniaceae;g__Pantoea;s__Pantoea_sp._ARC607
PB2_gene_4381	474	WP_056772425.1	995	459/474(96.84)	0	Tn3 family transposase [Serratia sp. Leaf51]	1736224	d__Bacteria;k__norank;p__Proteobacteria;c__Gammaproteobacteria;o__Enterobacterales;f__Yersiniaceae;g__Serratia;s__Serratia_sp._Leaf51
PB2_gene_4385	43	RAR87258.1	37	23/33(69.70)	7.55e-06	hypothetical protein OD26_1282 [Rahnella sp. NFIX50]	1566293	d__Bacteria;k__norank;p__Proteobacteria;c__Gammaproteobacteria;o__Enterobacterales;f__Yersiniaceae;g__Rahnella;s__Rahnella_sp._NFIX50
PB2_gene_4400	133	ORM85481.1	133	127/133(95.49)	1.61e-84	hypothetical protein HA42_02615 [Pantoea deleyi]	470932	d__Bacteria;k__norank;p__Proteobacteria;c__Gammaproteobacteria;o__Enterobacterales;f__Erwiniaceae;g__Pantoea;s__Pantoea_deleyi

PB2_gene_4401	376	ORM85480.1	341	338/341(99.12)	2.88e-240	LacI family transcriptional regulator [Pantoea deleyi]	470932	d__Bacteria;k__norank;p__Proteobacteria;c__Gammaproteobacteria;o__Enterobacterales;f__Erwiniaceae;g__Pantoea;s__Pantoea_deleyi
PB2_gene_4411	238	ORM80512.1	255	234/238(98.32)	6.40e-169	sulfate transporter CysZ [Pantoea deleyi]	470932	d__Bacteria;k__norank;p__Proteobacteria;c__Gammaproteobacteria;o__Enterobacterales;f__Erwiniaceae;g__Pantoea;s__Pantoea_deleyi
PB2_gene_4412	323	ORM80511.1	323	322/323(99.69)	1.29e-227	cysteine synthase A [Pantoea deleyi]	470932	d__Bacteria;k__norank;p__Proteobacteria;c__Gammaproteobacteria;o__Enterobacterales;f__Erwiniaceae;g__Pantoea;s__Pantoea_deleyi
PB2_gene_4424	170	ONI57228.1	170	161/170(94.71)	5.50e-103	hypothetical protein N499_1146 [Wolbachia pipientis wVitA]	1379786	d__Bacteria;k__norank;p__Proteobacteria;c__Alphaproteobacteria;o__Rickettsiales;f__Anaplasmataceae;g__Wolbachia;s__Wolbachia_pipientis
PB2_gene_4426	213	ORM84704.1	213	209/213(98.12)	4.50e-139	hypothetical protein HA42_03975 [Pantoea deleyi]	470932	d__Bacteria;k__norank;p__Proteobacteria;c__Gammaproteobacteria;o__Enterobacterales;f__Erwiniaceae;g__Pantoea;s__Pantoea_deleyi
PB2_gene_4437	223	PBQ28056.1	225	203/225(90.22)	6.40e-133	aankyrin [Wolbachia pipientis wAus]	2049495	d__Bacteria;k__norank;p__Proteobacteria;c__Alphaproteobacteria;o__Rickettsiales;f__Anaplasmataceae;g__Wolbachia;s__Wolbachia_pipientis
PB2_gene_4448	419	ORM85309.1	839	418/419(99.76)	2.95e-299	bifunctional aconitate hydratase 2/2-methylisocitrate dehydratase [Pantoea deleyi]	470932	d__Bacteria;k__norank;p__Proteobacteria;c__Gammaproteobacteria;o__Enterobacterales;f__Erwiniaceae;g__Pantoea;s__Pantoea_deleyi
PB2_gene_4450	172	WP_083070546.1	227	165/172(95.93)	1.64e-106	SDR family oxidoreductase [Pantoea vagans]	None	Unclassified
PB2_gene_4454	340	ORM81384.1	340	333/340(97.94)	3.78e-240	microcin ABC transporter permease [Pantoea deleyi]	470932	d__Bacteria;k__norank;p__Proteobacteria;c__Gammaproteobacteria;o__Enterobacterales;f__Erwiniaceae;g__Pantoea;s__Pantoea_deleyi
PB2_gene_4456	270	WP_081121039.1	224	84/162(51.85)	9.72e-33	hypothetical protein [Rickettsia amblyommatis]	33989	d__Bacteria;k__norank;p__Proteobacteria;c__Alphaproteobacteria;o__Rickettsiales;f__Rickettsiaceae;g__Rickettsia;s__Rickettsia_amblyommatis
PB2_gene_4457	168	YP_001967400.1	168	136/168(80.95)	9.00e-92	hypothetical protein RM_p20 (plasmid) [Rickettsia monacensis]	None	Unclassified
PB2_gene_4467	374	ORM84158.1	471	308/311(99.04)	3.05e-201	GABA permease [Pantoea deleyi]	470932	d__Bacteria;k__norank;p__Proteobacteria;c__Gammaproteobacteria;o__Enterobacterales;f__Erwiniaceae;g__Pantoea;s__Pantoea_deleyi
PB2_gene_4469	146	WP_111139561.1	139	132/139(94.96)	1.74e-95	VOC family protein [Pantoea sp. ARC607]	2027922	d__Bacteria;k__norank;p__Proteobacteria;c__Gammaproteobacteria;o__Enterobacterales;f__Erwiniaceae;g__Pantoea;s__Pantoea_sp._ARC607
PB2_gene_4470	402	ORM82934.1	468	402/402(100.00)	5.64e-278	replicative DNA helicase [Pantoea deleyi]	470932	d__Bacteria;k__norank;p__Proteobacteria;c__Gammaproteobacteria;o__Enterobacterales;f__Erwiniaceae;g__Pantoea;s__Pantoea_deleyi
PB2_gene_4471	327	ORM82933.1	327	320/327(97.86)	3.05e-228	quinone oxidoreductase [Pantoea deleyi]	470932	d__Bacteria;k__norank;p__Proteobacteria;c__Gammaproteobacteria;o__Enterobacterales;f__Erwiniaceae;g__Pantoea;s__Pantoea_deleyi
PB2_gene_4472	166	EWG75092.1	166	165/166(99.40)	3.11e-107	hypothetical protein P349_02461 [Enterobacter sp. DC4]	1395580	d__Bacteria;k__norank;p__Proteobacteria;c__Gammaproteobacteria;o__Enterobacterales;f__Enterobacteriaceae;g__Enterobacter;s__Enterobacter_sp._DC4
PB2_gene_4479	231	WP_111140223.1	231	227/231(98.27)	1.04e-146	LrgB family protein [Pantoea sp. ARC607]	2027922	d__Bacteria;k__norank;p__Proteobacteria;c__Gammaproteobacteria;o__Enterobacterales;f__Erwiniaceae;g__Pantoea;s__Pantoea_sp._ARC607
PB2_gene_4481	300	ORM75771.1	300	298/300(99.33)	2.79e-215	transcriptional regulator [Pantoea deleyi]	470932	d__Bacteria;k__norank;p__Proteobacteria;c__Gammaproteobacteria;o__Enterobacterales;f__Erwiniaceae;g__Pantoea;s__Pantoea_deleyi
PB2_gene_4484	510	ORM80449.1	510	508/510(99.61)	0	sugar ABC transporter ATP-binding protein [Pantoea deleyi]	470932	d__Bacteria;k__norank;p__Proteobacteria;c__Gammaproteobacteria;o__Enterobacterales;f__Erwiniaceae;g__Pantoea;s__Pantoea_deleyi
PB2_gene_4495	68	SSN11032.1	174	40/65(61.54)	2.05e-21	Uncharacterised protein [Klebsiella pneumoniae]	573	d__Bacteria;k__norank;p__Proteobacteria;c__Gammaproteobacteria;o__Enterobacterales;f__Enterobacteriaceae;g__Klebsiella;s__Klebsiella_pneumoniae
PB2_gene_4513	285	WP_111139057.1	285	281/285(98.60)	2.67e-185	HlyD family secretion protein [Pantoea sp. ARC607]	2027922	d__Bacteria;k__norank;p__Proteobacteria;c__Gammaproteobacteria;o__Enterobacterales;f__Erwiniaceae;g__Pantoea;s__Pantoea_sp._ARC607
PB2_gene_4518	179	WP_111140209.1	179	179/179(100.00)	2.40e-76	single-stranded DNA-binding protein SSB1 [Pantoea sp.	2027922	d__Bacteria;k__norank;p__Proteobacteria;c__Gammaproteobacteria;o__Enterobacterales;f__Erwiniaceae;g__Pantoea;s__Pantoea_sp._ARC607
PB2_gene_4520	235	ORM76866.1	235	230/235(97.87)	1.01e-155	YggS family pyridoxal phosphate enzyme [Pantoea deleyi]	470932	d__Bacteria;k__norank;p__Proteobacteria;c__Gammaproteobacteria;o__Enterobacterales;f__Erwiniaceae;g__Pantoea;s__Pantoea_deleyi
PB2_gene_4524	69	ORM85213.1	69	68/69(98.55)	5.65e-44	hypothetical protein HA42_02965 [Pantoea deleyi]	470932	d__Bacteria;k__norank;p__Proteobacteria;c__Gammaproteobacteria;o__Enterobacterales;f__Erwiniaceae;g__Pantoea;s__Pantoea_deleyi

PB2_gene_4536	696	WP_063630525.1	708	685/696(98.42)	0	toprim domain-containing protein [Wolbachia endosymbiont of Laodelphax striatella]	368602	d__Bacteria;k__norank;p__Proteobacteria;c__Alphaproteobacteria;o__Rickettsiales;f__Anaplasmataceae;g__Wolbachia;s__Wolbachia_endosymbiont_of_Laodelphax_striatellus
PB2_gene_4539	272	SJZ52189.1	272	258/272(94.85)	1.79e-197	exodeoxyribonuclease-3 [Pantoea agglomerans]	470933	d__Bacteria;k__norank;p__Proteobacteria;c__Gammaproteobacteria;o__Enterobacterales;f__Erwiniaceae;g__Pantoea;s__Pantoea_eucalypti
PB2_gene_4541	262	ORM81121.1	262	257/262(98.09)	9.15e-186	endonuclease VIII [Pantoea deleyi]	470932	d__Bacteria;k__norank;p__Proteobacteria;c__Gammaproteobacteria;o__Enterobacterales;f__Erwiniaceae;g__Pantoea;s__Pantoea_deleyi
PB2_gene_4551	187	CRR93654.1	235	80/175(45.71)	2.92e-39	hypothetical protein PAERUG_P61_London_9_VIM_2_11_13_03904 [Pseudomonas aeruginosa]	287	d__Bacteria;k__norank;p__Proteobacteria;c__Gammaproteobacteria;o__Pseudomonadales;f__Pseudomonadaceae;g__Pseudomonas;s__Pseudomonas_aeruginosa
PB2_gene_4552	101	CNV02031.1	48	27/46(58.70)	6.77e-06	Uncharacterised protein [Salmonella enterica subsp. enterica serovar	58097	d__Bacteria;k__norank;p__Proteobacteria;c__Gammaproteobacteria;o__Enterobacterales;f__Enterobacteriaceae;g__Salmonella;s__Salmonella_enterica
PB2_gene_4555	96	CDK62749.1	170	61/86(70.93)	1.94e-24	hypothetical protein [Klebsiella pneumoniae IS10]	1411646	d__Bacteria;k__norank;p__Proteobacteria;c__Gammaproteobacteria;o__Enterobacterales;f__Enterobacteriaceae;g__Klebsiella;s__Klebsiella_pneumoniae
PB2_gene_4559	340	ORM80621.1	340	339/340(99.71)	1.05e-246	dipeptide ABC transporter ATP-binding protein [Pantoea deleyi]	470932	d__Bacteria;k__norank;p__Proteobacteria;c__Gammaproteobacteria;o__Enterobacterales;f__Erwiniaceae;g__Pantoea;s__Pantoea_deleyi
PB2_gene_4569	391	WP_010406676.1	391	385/391(98.47)	3.65e-266	1-deoxy-D-xylulose-5-phosphate reductoisomerase [Wolbachia endosymbiont of Nasonia vitripennis]	180837	d__Bacteria;k__norank;p__Proteobacteria;c__Alphaproteobacteria;o__Rickettsiales;f__Anaplasmataceae;g__Wolbachia;s__Wolbachia_endosymbiont_of_Nasonia_vitripennis
PB2_gene_4572	346	WP_111139321.1	346	344/346(99.42)	3.16e-251	phosphoribosylformylglycinamidine cyclo-ligase [Pantoea sp.	2027922	d__Bacteria;k__norank;p__Proteobacteria;c__Gammaproteobacteria;o__Enterobacterales;f__Erwiniaceae;g__Pantoea;s__Pantoea_sp._ARC607
PB2_gene_4573	208	ORM76695.1	208	206/208(99.04)	2.25e-141	uracil phosphoribosyltransferase [Pantoea eucrina]	472693	d__Bacteria;k__norank;p__Proteobacteria;c__Gammaproteobacteria;o__Enterobacterales;f__Erwiniaceae;g__Pantoea;s__Pantoea_eucrina
PB2_gene_4579	125	WP_117006317.1	256	85/114(74.56)	1.17e-49	hypothetical protein [Klebsiella pneumoniae]	573	d__Bacteria;k__norank;p__Proteobacteria;c__Gammaproteobacteria;o__Enterobacterales;f__Enterobacteriaceae;g__Klebsiella;s__Klebsiella_pneumoniae
PB2_gene_4580	65	WP_117006317.1	256	46/63(73.02)	3.08e-23	hypothetical protein [Klebsiella pneumoniae]	573	d__Bacteria;k__norank;p__Proteobacteria;c__Gammaproteobacteria;o__Enterobacterales;f__Enterobacteriaceae;g__Klebsiella;s__Klebsiella_pneumoniae
PB2_gene_4588	604	ORM75366.1	604	591/604(97.85)	0	glucoamylase [Pantoea deleyi]	470932	d__Bacteria;k__norank;p__Proteobacteria;c__Gammaproteobacteria;o__Enterobacterales;f__Erwiniaceae;g__Pantoea;s__Pantoea_deleyi
PB2_gene_4598	296	ORM76135.1	352	293/296(98.99)	4.09e-208	molybdenum ABC transporter ATP-binding protein [Pantoea	470932	d__Bacteria;k__norank;p__Proteobacteria;c__Gammaproteobacteria;o__Enterobacterales;f__Erwiniaceae;g__Pantoea;s__Pantoea_deleyi
PB2_gene_4612	260	ORM78710.1	260	256/260(98.46)	8.30e-172	molybdenum-dependent transcriptional regulator [Pantoea deleyi]	470932	d__Bacteria;k__norank;p__Proteobacteria;c__Gammaproteobacteria;o__Enterobacterales;f__Erwiniaceae;g__Pantoea;s__Pantoea_deleyi
PB2_gene_4643	402	ORM83365.1	379	370/379(97.63)	1.98e-279	tRNA epoxyqueuosine(34) reductase QueG [Pantoea deleyi]	470932	d__Bacteria;k__norank;p__Proteobacteria;c__Gammaproteobacteria;o__Enterobacterales;f__Erwiniaceae;g__Pantoea;s__Pantoea_deleyi
PB2_gene_4647	428	PXW21515.1	420	417/420(99.29)	6.61e-304	D-alanyl-D-alanine carboxypeptidase (penicillin-binding protein 5/6) [Pantoea sp.	1938795	d__Bacteria;k__norank;p__Proteobacteria;c__Gammaproteobacteria;o__Enterobacterales;f__Erwiniaceae;g__Pantoea;s__Pantoea_sp._JKS000250
PB2_gene_4649	191	WP_111142258.1	191	182/191(95.29)	2.64e-118	manganese efflux pump MntP [Pantoea sp. ARC607]	2027922	d__Bacteria;k__norank;p__Proteobacteria;c__Gammaproteobacteria;o__Enterobacterales;f__Erwiniaceae;g__Pantoea;s__Pantoea_sp._ARC607
PB2_gene_4654	195	SJZ41035.1	195	192/195(98.46)	2.68e-138	peptidyl-tRNA hydrolase, PTH1 family [Pantoea agglomerans]	470933	d__Bacteria;k__norank;p__Proteobacteria;c__Gammaproteobacteria;o__Enterobacterales;f__Erwiniaceae;g__Pantoea;s__Pantoea_eucalypti
PB2_gene_4655	207	SSW86476.1	336	106/169(62.72)	1.46e-65	Uncharacterised protein [Klebsiella pneumoniae]	573	d__Bacteria;k__norank;p__Proteobacteria;c__Gammaproteobacteria;o__Enterobacterales;f__Enterobacteriaceae;g__Klebsiella;s__Klebsiella_pneumoniae
PB2_gene_4658	206	ORM81771.1	206	201/206(97.57)	6.13e-129	hypothetical protein HA42_09855 [Pantoea deleyi]	470932	d__Bacteria;k__norank;p__Proteobacteria;c__Gammaproteobacteria;o__Enterobacterales;f__Erwiniaceae;g__Pantoea;s__Pantoea_deleyi

PB2_gene_4661	398	WP_111140475.1	394	371/389(95.37)	1.88e-250	MFS transporter [Pantoea sp. ARC607]	2027922	d__Bacteria;k__norank;p__Proteobacteria;c__Gammaproteobacteria;o__Enterobacterales;f__Erwiniaceae;g__Pantoea;s__Pantoea_sp._ARC607
PB2_gene_4663	263	SUB18872.1	410	257/263(97.72)	5.56e-174	Aminobenzoyl-glutamate transport protein [Pantoea	549	d__Bacteria;k__norank;p__Proteobacteria;c__Gammaproteobacteria;o__Enterobacterales;f__Erwiniaceae;g__Pantoea;s__Pantoea_agglomerans
PB2_gene_4671	330	ORM80513.1	330	322/329(97.87)	1.34e-196	cell division protein ZipA [Pantoea deleyi]	470932	d__Bacteria;k__norank;p__Proteobacteria;c__Gammaproteobacteria;o__Enterobacterales;f__Erwiniaceae;g__Pantoea;s__Pantoea_deleyi
PB2_gene_4685	442	WP_111140607.1	442	438/442(99.10)	1.06e-309	ATP-dependent RNA helicase SrmB [Pantoea sp. ARC607]	2027922	d__Bacteria;k__norank;p__Proteobacteria;c__Gammaproteobacteria;o__Enterobacterales;f__Erwiniaceae;g__Pantoea;s__Pantoea_sp._ARC607
PB2_gene_4687	338	ORM81119.1	337	327/338(96.75)	1.10e-224	hypothetical protein HA42_11780 [Pantoea deleyi]	470932	d__Bacteria;k__norank;p__Proteobacteria;c__Gammaproteobacteria;o__Enterobacterales;f__Erwiniaceae;g__Pantoea;s__Pantoea_deleyi
PB2_gene_4701	249	WP_111138806.1	249	247/249(99.20)	4.33e-173	3-deoxy-manno-octulosonate cytidylyltransferase [Pantoea sp. ARC607]	2027922	d__Bacteria;k__norank;p__Proteobacteria;c__Gammaproteobacteria;o__Enterobacterales;f__Erwiniaceae;g__Pantoea;s__Pantoea_sp._ARC607
PB2_gene_4708	452	ORM85662.1	452	449/452(99.34)	1.11e-314	putrescine/spermidine ABC transporter [Pantoea deleyi]	470932	d__Bacteria;k__norank;p__Proteobacteria;c__Gammaproteobacteria;o__Enterobacterales;f__Erwiniaceae;g__Pantoea;s__Pantoea_deleyi
PB2_gene_4716	489	WP_111141201.1	484	480/484(99.17)	0	AMP nucleosidase [Pantoea sp. ARC607]	2027922	d__Bacteria;k__norank;p__Proteobacteria;c__Gammaproteobacteria;o__Enterobacterales;f__Erwiniaceae;g__Pantoea;s__Pantoea_sp._ARC607
PB2_gene_4719	129	WP_031591437.1	129	113/129(87.60)	5.67e-74	hypothetical protein [Pantoea agglomerans]	549	d__Bacteria;k__norank;p__Proteobacteria;c__Gammaproteobacteria;o__Enterobacterales;f__Erwiniaceae;g__Pantoea;s__Pantoea_agglomerans
PB2_gene_4721	126	WP_082668175.1	120	95/110(86.36)	5.05e-63	MULTISPECIES: phage holin, lambda family [Pantoea]	53335	d__Bacteria;k__norank;p__Proteobacteria;c__Gammaproteobacteria;o__Enterobacterales;f__Erwiniaceae;g__Pantoea;s__norank
PB2_gene_4729	240	SQI40124.1	224	174/223(78.03)	4.57e-121	Uncharacterised protein [Serratia plymuthica]	82996	d__Bacteria;k__norank;p__Proteobacteria;c__Gammaproteobacteria;o__Enterobacterales;f__Yersiniaceae;g__Serratia;s__Serratia_plymuthica
PB2_gene_4730	61	WP_063881017.1	62	60/62(96.77)	3.38e-35	hypothetical protein [Pantoea sp. OXWO6B1]	2630326	d__Bacteria;k__norank;p__Proteobacteria;c__Gammaproteobacteria;o__Enterobacterales;f__Erwiniaceae;g__Pantoea;s__norank
PB2_gene_4736	501	ORM82654.1	501	497/501(99.20)	0	methylmalonate-semialdehyde dehydrogenase (acylating) [Pantoea deleyi]	470932	d__Bacteria;k__norank;p__Proteobacteria;c__Gammaproteobacteria;o__Enterobacterales;f__Erwiniaceae;g__Pantoea;s__Pantoea_deleyi
PB2_gene_4738	273	ORM79065.1	363	271/273(99.27)	1.63e-183	mechanosensitive ion channel protein MscS [Pantoea deleyi]	470932	d__Bacteria;k__norank;p__Proteobacteria;c__Gammaproteobacteria;o__Enterobacterales;f__Erwiniaceae;g__Pantoea;s__Pantoea_deleyi
PB2_gene_4751	407	PAW34229.1	243	235/243(96.71)	3.54e-172	carbonate dehydratase [Pantoea vagans]	470934	d__Bacteria;k__norank;p__Proteobacteria;c__Gammaproteobacteria;o__Enterobacterales;f__Erwiniaceae;g__Pantoea;s__Pantoea_vagans
PB2_gene_4754	163	WP_128084401.1	163	160/163(98.16)	2.80e-113	tRNA adenosine(34) deaminase TadA [Pantoea deleyi]	470932	d__Bacteria;k__norank;p__Proteobacteria;c__Gammaproteobacteria;o__Enterobacterales;f__Erwiniaceae;g__Pantoea;s__Pantoea_deleyi
PB2_gene_4755	238	SUB03908.1	238	222/238(93.28)	3.41e-160	HAD hydrolase, family IF [Pantoea agglomerans]	549	d__Bacteria;k__norank;p__Proteobacteria;c__Gammaproteobacteria;o__Enterobacterales;f__Erwiniaceae;g__Pantoea;s__Pantoea_agglomerans
PB2_gene_4758	475	ORM84475.1	475	460/475(96.84)	0	exodeoxyribonuclease I [Pantoea deleyi]	470932	d__Bacteria;k__norank;p__Proteobacteria;c__Gammaproteobacteria;o__Enterobacterales;f__Erwiniaceae;g__Pantoea;s__Pantoea_deleyi
PB2_gene_4761	89	BAN94793.1	61	29/50(58.00)	6.15e-06	hypothetical protein E05_00270 [Plautia stali symbiont]	891974	d__Bacteria;k__norank;p__Proteobacteria;c__Gammaproteobacteria;o__Enterobacterales;f__Enterobacteriaceae;g__norank;s__Plautia_stali_symbiont
PB2_gene_4765	74	ORM80493.1	74	74/74(100.00)	5.66e-44	hypothetical protein HA42_14340 [Pantoea deleyi]	470932	d__Bacteria;k__norank;p__Proteobacteria;c__Gammaproteobacteria;o__Enterobacterales;f__Erwiniaceae;g__Pantoea;s__Pantoea_deleyi
PB2_gene_4770	637	ORM82658.1	637	621/637(97.49)	0	5-dehydro-2-deoxygluconokinase [Pantoea deleyi]	470932	d__Bacteria;k__norank;p__Proteobacteria;c__Gammaproteobacteria;o__Enterobacterales;f__Erwiniaceae;g__Pantoea;s__Pantoea_deleyi
PB2_gene_4772	265	WP_111140230.1	265	255/265(96.23)	5.48e-184	SDR family oxidoreductase [Pantoea sp. ARC607]	2027922	d__Bacteria;k__norank;p__Proteobacteria;c__Gammaproteobacteria;o__Enterobacterales;f__Erwiniaceae;g__Pantoea;s__Pantoea_sp._ARC607
PB2_gene_4775	200	WP_063630476.1	207	95/161(59.01)	3.34e-54	hypothetical protein [Wolbachia endosymbiont of Laodelphax striatella]	368602	d__Bacteria;k__norank;p__Proteobacteria;c__Alphaproteobacteria;o__Rickettsiales;f__Anaplasmataceae;g__Wolbachia;s__Wolbachia_endosymbiont_of_Laodelphax_striatellus
PB2_gene_4783	343	ORM76130.1	343	343/343(100.00)	7.17e-251	biotin synthase [Pantoea deleyi]	470932	d__Bacteria;k__norank;p__Proteobacteria;c__Gammaproteobacteria;o__Enterobacterales;f__Erwiniaceae;g__Pantoea;s__Pantoea_deleyi

PB2_gene_4786	418	ORM85632.1	420	418/418(100.00)	5.15e-303	colanic acid biosynthesis pyruvyl transferase WcaK [Pantoea deleyi]	470932	d__Bacteria;k__norank;p__Proteobacteria;c__Gammaproteobacteria;o__Enterobacterales;f__Erwiniaceae;g__Pantoea;s__Pantoea_deleyi
PB2_gene_4787	404	ORM81086.1	404	385/403(95.53)	6.57e-287	hypothetical protein HA42_11900 [Pantoea deleyi]	470932	d__Bacteria;k__norank;p__Proteobacteria;c__Gammaproteobacteria;o__Enterobacterales;f__Erwiniaceae;g__Pantoea;s__Pantoea_deleyi
PB2_gene_4789	674	WP_019236320.1	698	645/674(95.70)	0	hypothetical protein [Wolbachia pipientis]	955	d__Bacteria;k__norank;p__Proteobacteria;c__Alphaproteobacteria;o__Rickettsiales;f__Anaplasmataceae;g__Wolbachia;s__Wolbachia_pipientis
PB2_gene_4798	155	WP_031591460.1	155	137/155(88.39)	3.56e-94	GNAT family N-acetyltransferase [Pantoea agglomerans]	549	d__Bacteria;k__norank;p__Proteobacteria;c__Gammaproteobacteria;o__Enterobacterales;f__Erwiniaceae;g__Pantoea;s__Pantoea_agglomerans
PB2_gene_4799	328	WP_111140488.1	328	324/328(98.78)	8.90e-217	sugar ABC transporter permease YjfF [Pantoea sp. ARC607]	2027922	d__Bacteria;k__norank;p__Proteobacteria;c__Gammaproteobacteria;o__Enterobacterales;f__Erwiniaceae;g__Pantoea;s__Pantoea_sp._ARC607
PB2_gene_4831	66	WP_111141124.1	794	50/68(73.53)	5.22e-22	phosphoketolase family protein [Pantoea sp. ARC607]	2027922	d__Bacteria;k__norank;p__Proteobacteria;c__Gammaproteobacteria;o__Enterobacterales;f__Erwiniaceae;g__Pantoea;s__Pantoea_sp._ARC607
PB2_gene_4833	522	ORM82060.1	522	518/522(99.23)	0	alkyl hydroperoxide reductase subunit F [Pantoea deleyi]	470932	d__Bacteria;k__norank;p__Proteobacteria;c__Gammaproteobacteria;o__Enterobacterales;f__Erwiniaceae;g__Pantoea;s__Pantoea_deleyi
PB2_gene_4836	488	WP_111142056.1	492	467/483(96.69)	0	succinylglutamate-semialdehyde dehydrogenase [Pantoea sp. ARC607]	2027922	d__Bacteria;k__norank;p__Proteobacteria;c__Gammaproteobacteria;o__Enterobacterales;f__Erwiniaceae;g__Pantoea;s__Pantoea_sp._ARC607
PB2_gene_4841	173	WP_010403494.1	396	172/173(99.42)	1.59e-114	phosphoglycerate kinase [Wolbachia endosymbiont of Nasonia vitripennis]	None	Unclassified
PB2_gene_4843	419	ORM83265.1	398	391/398(98.24)	7.21e-266	MFS transporter [Pantoea deleyi]	470932	d__Bacteria;k__norank;p__Proteobacteria;c__Gammaproteobacteria;o__Enterobacterales;f__Erwiniaceae;g__Pantoea;s__Pantoea_deleyi
PB2_gene_4844	262	ORM76576.1	694	254/262(96.95)	6.86e-180	TonB-dependent receptor [Pantoea deleyi]	470932	d__Bacteria;k__norank;p__Proteobacteria;c__Gammaproteobacteria;o__Enterobacterales;f__Erwiniaceae;g__Pantoea;s__Pantoea_deleyi
PB2_gene_4845	186	ORM76575.1	186	180/186(96.77)	2.39e-123	hydrolase [Pantoea deleyi]	470932	d__Bacteria;k__norank;p__Proteobacteria;c__Gammaproteobacteria;o__Enterobacterales;f__Erwiniaceae;g__Pantoea;s__Pantoea_deleyi
PB2_gene_4846	141	WP_111142149.1	141	131/141(92.91)	2.51e-85	MarR family transcriptional regulator [Pantoea sp. ARC607]	2027922	d__Bacteria;k__norank;p__Proteobacteria;c__Gammaproteobacteria;o__Enterobacterales;f__Erwiniaceae;g__Pantoea;s__Pantoea_sp._ARC607
PB2_gene_4853	529	ORM80956.1	529	523/529(98.87)	0	bifunctional phosphoribosylaminoimidazolecarboxamide formyltransferase/IMP cyclohydrolase [Pantoea deleyi]	470932	d__Bacteria;k__norank;p__Proteobacteria;c__Gammaproteobacteria;o__Enterobacterales;f__Erwiniaceae;g__Pantoea;s__Pantoea_deleyi
PB2_gene_4856	620	ORM83139.1	784	619/620(99.84)	0	endopeptidase La [Pantoea deleyi]	470932	d__Bacteria;k__norank;p__Proteobacteria;c__Gammaproteobacteria;o__Enterobacterales;f__Erwiniaceae;g__Pantoea;s__Pantoea_deleyi
PB2_gene_4862	357	ORM75304.1	458	356/356(100.00)	4.46e-212	PTS fructose transporter subunit EIIBC, partial [Pantoea deleyi]	470932	d__Bacteria;k__norank;p__Proteobacteria;c__Gammaproteobacteria;o__Enterobacterales;f__Erwiniaceae;g__Pantoea;s__Pantoea_deleyi
PB2_gene_4871	334	ORM86814.1	334	333/334(99.70)	5.12e-235	Holliday junction DNA helicase RuvB [Pantoea deleyi]	470932	d__Bacteria;k__norank;p__Proteobacteria;c__Gammaproteobacteria;o__Enterobacterales;f__Erwiniaceae;g__Pantoea;s__Pantoea_deleyi
PB2_gene_4874	174	WP_111138866.1	187	170/174(97.70)	7.45e-115	CoA pyrophosphatase [Pantoea sp. ARC607]	2027922	d__Bacteria;k__norank;p__Proteobacteria;c__Gammaproteobacteria;o__Enterobacterales;f__Erwiniaceae;g__Pantoea;s__Pantoea_sp._ARC607
PB2_gene_4886	197	ORM75345.1	191	189/191(98.95)	5.94e-137	hypothetical protein HA42_20975 [Pantoea deleyi]	470932	d__Bacteria;k__norank;p__Proteobacteria;c__Gammaproteobacteria;o__Enterobacterales;f__Erwiniaceae;g__Pantoea;s__Pantoea_deleyi
PB2_gene_4911	142	WP_010406729.1	142	142/142(100.00)	4.01e-96	hypothetical protein [Wolbachia endosymbiont of Nasonia vitripennis]	2640676	d__Bacteria;k__norank;p__Proteobacteria;c__Alphaproteobacteria;o__Rickettsiales;f__Anaplasmataceae;g__Wolbachia;s__norank
PB2_gene_4915	105	AGJ98891.1	102	94/101(93.07)	9.53e-61	hypothetical protein wNo_04680 [Wolbachia endosymbiont of Drosophila simulans wNo]	1236908	d__Bacteria;k__norank;p__Proteobacteria;c__Alphaproteobacteria;o__Rickettsiales;f__Anaplasmataceae;g__Wolbachia;s__Wolbachia_endosymbiont_of_Drosophila_simulans
PB2_gene_4922	305	ORM85665.1	305	301/305(98.69)	1.75e-216	amylovoran biosynthesis protein AmsB [Pantoea deleyi]	470932	d__Bacteria;k__norank;p__Proteobacteria;c__Gammaproteobacteria;o__Enterobacterales;f__Erwiniaceae;g__Pantoea;s__Pantoea_deleyi

PB2_gene_4924	386	ORM75995.1	386	373/386(96.63)	1.07e-276	methionine aminotransferase [Pantoea deleyi]	470932	d__Bacteria;k__norank;p__Proteobacteria;c__Gammaproteobacteria;o__Enterobacterales;f__Erwiniaceae;g__Pantoea;s__Pantoea_deleyi
PB2_gene_4933	362	ORM80505.1	362	360/362(99.45)	1.36e-260	sulfate/thiosulfate transporter subunit [Pantoea deleyi]	470932	d__Bacteria;k__norank;p__Proteobacteria;c__Gammaproteobacteria;o__Enterobacterales;f__Erwiniaceae;g__Pantoea;s__Pantoea_deleyi
PB2_gene_4937	441	CCJ86249.1	434	142/371(38.27)	3.49e-62	hypothetical protein BN133_2626 [Cronobacter dublinensis 582]	1208661	d__Bacteria;k__norank;p__Proteobacteria;c__Gammaproteobacteria;o__Enterobacterales;f__Enterobacteriaceae;g__Cronobacter;s__Cronobacter_dublinensis
PB2_gene_4938	121	CPR48682.1	197	41/97(42.27)	2.52e-13	Uncharacterised protein [Salmonella enterica subsp. enterica serovar	58097	d__Bacteria;k__norank;p__Proteobacteria;c__Gammaproteobacteria;o__Enterobacterales;f__Enterobacteriaceae;g__Salmonella;s__Salmonella_enterica
PB2_gene_4940	362	WP_112894861.1	368	126/350(36.00)	6.51e-52	lipase family protein [Photorhabdus bodei]	2029681	d__Bacteria;k__norank;p__Proteobacteria;c__Gammaproteobacteria;o__Enterobacterales;f__Morganellaceae;g__Photorhabdus;s__Photorhabdus_bodei
PB2_gene_4948	47	ORM86577.1	72	47/47(100.00)	5.82e-25	DUF903 domain-containing protein [Pantoea deleyi]	470932	d__Bacteria;k__norank;p__Proteobacteria;c__Gammaproteobacteria;o__Enterobacterales;f__Erwiniaceae;g__Pantoea;s__Pantoea_deleyi
PB2_gene_4949	130	WP_024562395.1	130	106/130(81.54)	5.45e-77	MULTISPECIES: DUF943 family protein	543	d__Bacteria;k__norank;p__Proteobacteria;c__Gammaproteobacteria;o__Enterobacterales;f__Enterobacteriaceae;g__norank;s__norank
PB2_gene_4950	67	ANI85322.1	289	59/66(89.39)	1.29e-36	hypothetical protein AWR26_18185 [Kosakonia	497725	d__Bacteria;k__norank;p__Proteobacteria;c__Gammaproteobacteria;o__Enterobacterales;f__Enterobacteriaceae;g__Kosakonia;s__Kosakonia_oryzae
PB2_gene_4958	161	OXH79440.1	161	156/161(96.89)	9.74e-104	cyclic pyranopterin monophosphate synthase MoaC [Pantoea agglomerans]	549	d__Bacteria;k__norank;p__Proteobacteria;c__Gammaproteobacteria;o__Enterobacterales;f__Erwiniaceae;g__Pantoea;s__Pantoea_agglomerans
PB2_gene_4973	334	ORM85226.1	334	332/334(99.40)	1.26e-235	transcriptional regulator [Pantoea deleyi]	470932	d__Bacteria;k__norank;p__Proteobacteria;c__Gammaproteobacteria;o__Enterobacterales;f__Erwiniaceae;g__Pantoea;s__Pantoea_deleyi
PB2_gene_4974	252	WP_127464204.1	252	250/252(99.21)	1.23e-169	twin-arginine translocase subunit TatC [Wolbachia endosymbiont of Bemisia tabaci]	215173	d__Bacteria;k__norank;p__Proteobacteria;c__Alphaproteobacteria;o__Rickettsiales;f__Anaplasmataceae;g__Wolbachia;s__Wolbachia_endosymbiont_of_Bemisia_tabaci
PB2_gene_4978	437	WP_123802027.1	431	404/431(93.74)	4.13e-303	dTDP-6-deoxy-D-glucose-3,5-epimerase [Pantoea sp. RIT388]	1869313	d__Bacteria;k__norank;p__Proteobacteria;c__Gammaproteobacteria;o__Enterobacterales;f__Erwiniaceae;g__Pantoea;s__Pantoea_sp._RIT388
PB2_gene_4983	245	EWG64812.1	245	237/245(96.73)	3.99e-167	hypothetical protein P348_04614 [Enterobacter sp. DC3]	1395579	d__Bacteria;k__norank;p__Proteobacteria;c__Gammaproteobacteria;o__Enterobacterales;f__Enterobacteriaceae;g__Enterobacter;s__Enterobacter_sp._DC3
PB2_gene_4999	201	ONI58176.1	336	186/195(95.38)	2.35e-127	phage terminase large subunit family protein [Wolbachia	1379786	d__Bacteria;k__norank;p__Proteobacteria;c__Alphaproteobacteria;o__Rickettsiales;f__Anaplasmataceae;g__Wolbachia;s__Wolbachia_pipientis
PB2_gene_5005	470	WP_025119482.1	471	356/470(75.74)	3.53e-258	MULTISPECIES: ABC transporter ATP-binding protein	2647522	d__Bacteria;k__norank;p__Proteobacteria;c__Gammaproteobacteria;o__Enterobacterales;f__Yersiniaceae;g__Serratia;s__norank
PB2_gene_5006	129	WP_049216938.1	244	102/129(79.07)	5.43e-69	ABC transporter permease, partial [Serratia marcescens]	615	d__Bacteria;k__norank;p__Proteobacteria;c__Gammaproteobacteria;o__Enterobacterales;f__Yersiniaceae;g__Serratia;s__Serratia_marcescens
PB2_gene_5018	101	ORM80816.1	101	96/101(95.05)	3.45e-55	hypothetical protein HA42_12885 [Pantoea deleyi]	470932	d__Bacteria;k__norank;p__Proteobacteria;c__Gammaproteobacteria;o__Enterobacterales;f__Erwiniaceae;g__Pantoea;s__Pantoea_deleyi
PB2_gene_5020	80	ORM80814.1	80	71/80(88.75)	2.04e-42	hypothetical protein HA42_12875 [Pantoea deleyi]	470932	d__Bacteria;k__norank;p__Proteobacteria;c__Gammaproteobacteria;o__Enterobacterales;f__Erwiniaceae;g__Pantoea;s__Pantoea_deleyi
PB2_gene_5027	483	WP_128086277.1	562	475/483(98.34)	6.81e-291	Cl- channel voltage-gated family protein [Pantoea deleyi]	470932	d__Bacteria;k__norank;p__Proteobacteria;c__Gammaproteobacteria;o__Enterobacterales;f__Erwiniaceae;g__Pantoea;s__Pantoea_deleyi
PB2_gene_5034	255	RYA69880.1	216	69/171(40.35)	8.43e-35	hypothetical protein DD592_26625, partial [Enterobacter cloacae complex	2511993	d__Bacteria;k__norank;p__Proteobacteria;c__Gammaproteobacteria;o__Enterobacterales;f__Enterobacteriaceae;g__Enterobacter;s__Enterobacter_cloacae_complex_sp._2DZ2F20B
PB2_gene_5035	211	WP_129490032.1	160	49/133(36.84)	2.44e-23	hypothetical protein [Klebsiella pneumoniae]	573	d__Bacteria;k__norank;p__Proteobacteria;c__Gammaproteobacteria;o__Enterobacterales;f__Enterobacteriaceae;g__Klebsiella;s__Klebsiella_pneumoniae
PB2_gene_5037	179	WP_061901530.1	1501	170/179(94.97)	1.16e-112	type IV secretion protein Rhs [Pantoea agglomerans]	549	d__Bacteria;k__norank;p__Proteobacteria;c__Gammaproteobacteria;o__Enterobacterales;f__Erwiniaceae;g__Pantoea;s__Pantoea_agglomerans
PB2_gene_5054	77	ORM81541.1	77	74/75(98.67)	8.89e-38	heme exporter protein CcmD [Pantoea deleyi]	470932	d__Bacteria;k__norank;p__Proteobacteria;c__Gammaproteobacteria;o__Enterobacterales;f__Erwiniaceae;g__Pantoea;s__Pantoea_deleyi

PB2_gene_5056	149	AVV38625.1	163	147/149(98.66)	4.68e-106	type VI secretion system tube protein Hcp [Pantoea vagans]	470934	d__Bacteria;k__norank;p__Proteobacteria;c__Gammaproteobacteria;o__Enterobacterales;f__Erwiniaceae;g__Pantoea;s__Pantoea_vagans
PB2_gene_5068	124	SBV63087.1	57	34/40(85.00)	7.36e-15	conserved hypothetical protein [uncultured Citrobacter sp.]	200446	d__Bacteria;k__norank;p__Proteobacteria;c__Gammaproteobacteria;o__Enterobacterales;f__Enterobacteriaceae;g__Citrobacter;s__uncultured_Citrobacter_sp.
PB2_gene_5069	275	ORM86050.1	343	257/275(93.45)	2.21e-186	hypothetical protein HA42_01655 [Pantoea deleyi]	470932	d__Bacteria;k__norank;p__Proteobacteria;c__Gammaproteobacteria;o__Enterobacterales;f__Erwiniaceae;g__Pantoea;s__Pantoea_deleyi
PB2_gene_5074	230	ORM81136.1	228	226/227(99.56)	8.39e-152	protein TolQ [Pantoea deleyi]	470932	d__Bacteria;k__norank;p__Proteobacteria;c__Gammaproteobacteria;o__Enterobacterales;f__Erwiniaceae;g__Pantoea;s__Pantoea_deleyi
PB2_gene_5078	287	WP_026092648.1	498	286/287(99.65)	3.06e-184	MULTISPECIES: murein biosynthesis integral membrane protein MurJ [Wolbachia]	116598	d__Bacteria;k__norank;p__Proteobacteria;c__Alphaproteobacteria;o__Rickettsiales;f__Anaplasmataceae;g__Wolbachia;s__Wolbachia_endosymbiont_of_Diaphorina_citri
PB2_gene_5097	122	RYE27129.1	1122	41/123(33.33)	2.68e-09	hypothetical protein EOP45_02210	2021370	d__Bacteria;k__norank;p__Bacteroidetes;c__Sphingobacteriia;o__Sphingobacteriales;f__Sphingobacteriaceae;g__norank;s__Sphingobacteriaceae_bacterium
PB2_gene_5100	367	WP_123346426.1	362	259/367(70.57)	2.50e-179	MULTISPECIES: phage tail sheath family protein	2608935	d__Bacteria;k__norank;p__Proteobacteria;c__Gammaproteobacteria;o__Enterobacterales;f__Enterobacteriaceae;g__Enterobacter;s__norank
PB2_gene_5114	226	WP_052264841.1	445	224/226(99.12)	2.06e-148	cytochrome c oxidase subunit 1, partial [Wolbachia endosymbiont of Operophtera brumata]	1620420	d__Bacteria;k__norank;p__Proteobacteria;c__Alphaproteobacteria;o__Rickettsiales;f__Anaplasmataceae;g__Wolbachia;s__Wolbachia_endosymbiont_of_Operophtera_brumata
PB2_gene_5117	206	ORM77024.1	180	170/180(94.44)	8.15e-128	thioredoxin [Pantoea deleyi]	470932	d__Bacteria;k__norank;p__Proteobacteria;c__Gammaproteobacteria;o__Enterobacterales;f__Erwiniaceae;g__Pantoea;s__Pantoea_deleyi
PB2_gene_5119	252	WP_063877214.1	253	183/249(73.49)	4.07e-127	molecular chaperone [Pantoea sp. OXWO6B1]	1835724	d__Bacteria;k__norank;p__Proteobacteria;c__Gammaproteobacteria;o__Enterobacterales;f__Erwiniaceae;g__Pantoea;s__Pantoea_sp._OXWO6B1
PB2_gene_5124	292	CAQ54582.1	292	287/292(98.29)	5.31e-200	porphobilinogen deaminase [Wolbachia endosymbiont of Culex quinquefasciatus Pel]	570417	d__Bacteria;k__norank;p__Proteobacteria;c__Alphaproteobacteria;o__Rickettsiales;f__Anaplasmataceae;g__Wolbachia;s__Wolbachia_endosymbiont_of_Culex_quinquefasciatus
PB2_gene_5126	360	WP_084690491.1	753	261/276(94.57)	3.38e-184	autotransporter outer membrane beta-barrel domain-containing protein [Pantoea anthophila]	None	Unclassified
PB2_gene_5134	555	AGJ99495.1	577	100/104(96.15)	5.63e-53	Ankyrin repeat domain protein [Wolbachia endosymbiont of Drosophila simulans wNo]	1236908	d__Bacteria;k__norank;p__Proteobacteria;c__Alphaproteobacteria;o__Rickettsiales;f__Anaplasmataceae;g__Wolbachia;s__Wolbachia_endosymbiont_of_Drosophila_simulans
PB2_gene_5143	249	ORM80808.1	249	241/249(96.79)	1.05e-164	DeoR family transcriptional regulator [Pantoea deleyi]	470932	d__Bacteria;k__norank;p__Proteobacteria;c__Gammaproteobacteria;o__Enterobacterales;f__Erwiniaceae;g__Pantoea;s__Pantoea_deleyi
PB2_gene_5147	182	ORM83366.1	182	180/182(98.90)	1.99e-129	oligoribonuclease [Pantoea deleyi]	470932	d__Bacteria;k__norank;p__Proteobacteria;c__Gammaproteobacteria;o__Enterobacterales;f__Erwiniaceae;g__Pantoea;s__Pantoea_deleyi
PB2_gene_5153	155	WP_111140327.1	836	152/155(98.06)	3.32e-86	copper-exporting P-type ATPase CopA [Pantoea sp. ARC607]	2027922	d__Bacteria;k__norank;p__Proteobacteria;c__Gammaproteobacteria;o__Enterobacterales;f__Erwiniaceae;g__Pantoea;s__Pantoea_sp._ARC607
PB2_gene_5154	267	WP_111140324.1	267	261/267(97.75)	1.53e-191	conjugal transfer protein TraB [Pantoea sp. ARC607]	2027922	d__Bacteria;k__norank;p__Proteobacteria;c__Gammaproteobacteria;o__Enterobacterales;f__Erwiniaceae;g__Pantoea;s__Pantoea_sp._ARC607
PB2_gene_5171	59	SJZ98362.1	306	59/59(100.00)	3.63e-33	DNA-binding transcriptional regulator, LysR family [Pantoea agglomerans]	470933	d__Bacteria;k__norank;p__Proteobacteria;c__Gammaproteobacteria;o__Enterobacterales;f__Erwiniaceae;g__Pantoea;s__Pantoea_eucalypti
PB2_gene_5184	158	WP_111142169.1	158	150/158(94.94)	2.14e-101	YbaK/prolyl-tRNA synthetase associated domain-containing protein [Pantoea sp. ARC607]	2027922	d__Bacteria;k__norank;p__Proteobacteria;c__Gammaproteobacteria;o__Enterobacterales;f__Erwiniaceae;g__Pantoea;s__Pantoea_sp._ARC607
PB2_gene_5188	245	WP_111141011.1	245	238/244(97.54)	4.66e-166	5-oxoprolinase subunit PxpA [Pantoea sp. ARC607]	2027922	d__Bacteria;k__norank;p__Proteobacteria;c__Gammaproteobacteria;o__Enterobacterales;f__Erwiniaceae;g__Pantoea;s__Pantoea_sp._ARC607
PB2_gene_5198	403	POG52112.1	534	384/404(95.05)	3.55e-272	endopeptidase La, partial [Wolbachia sp. wRi_2]	1932745	d__Bacteria;k__norank;p__Proteobacteria;c__Alphaproteobacteria;o__Rickettsiales;f__Anaplasmataceae;g__Wolbachia;s__Wolbachia_sp._wRi_2
PB2_gene_5202	155	WP_067709249.1	155	125/154(81.17)	8.39e-93	DUF943 family protein [Erwinia sp. ErVv1]	1603299	d__Bacteria;k__norank;p__Proteobacteria;c__Gammaproteobacteria;o__Enterobacterales;f__Erwiniaceae;g__Erwinia;s__Erwinia_sp._ErVv1

PB2_gene_5203	265	WP_090133171.1	279	221/260(85.00)	1.40e-162	DUF3289 family protein [Kosakonia oryziphila]	1005667	d__Bacteria;k__norank;p__Proteobacteria;c__Gammaproteobacteria;o__Enterobacterales;f__Enterobacteriaceae;g__Kosakonia;s__Kosakonia_oryziphila
PB2_gene_5230	111	WP_054305123.1	111	88/111(79.28)	1.69e-62	hypothetical protein [Serratia rubidaea]	61652	d__Bacteria;k__norank;p__Proteobacteria;c__Gammaproteobacteria;o__Enterobacterales;f__Yersiniaceae;g__Serratia;s__Serratia_rubidaea
PB2_gene_5231	462	WP_084089836.1	527	359/469(76.55)	1.78e-239	hemagglutination activity domain protein [Erwinia oleae]	796334	d__Bacteria;k__norank;p__Proteobacteria;c__Gammaproteobacteria;o__Enterobacterales;f__Erwiniaceae;g__Erwinia;s__Erwinia_oleae
PB2_gene_5232	437	WP_111138393.1	437	422/437(96.57)	0	LLM class flavin-dependent oxidoreductase [Pantoea sp. ARC607]	2027922	d__Bacteria;k__norank;p__Proteobacteria;c__Gammaproteobacteria;o__Enterobacterales;f__Erwiniaceae;g__Pantoea;s__Pantoea_sp._ARC607
PB2_gene_5239	308	WP_052270629.1	308	285/308(92.53)	1.22e-213	glyoxylate/hydroxypyruvate reductase A [Pantoea sp. CFSAN033090]	1690502	d__Bacteria;k__norank;p__Proteobacteria;c__Gammaproteobacteria;o__Enterobacterales;f__Erwiniaceae;g__Pantoea;s__Pantoea_sp._CFSAN033090
PB2_gene_5243	56	CCE77334.1	42	42/42(100.00)	6.17e-18	hypothetical protein WALBB_280001 [Wolbachia pipientis wAlbB]	1116230	d__Bacteria;k__norank;p__Proteobacteria;c__Alphaproteobacteria;o__Rickettsiales;f__Anaplasmataceae;g__Wolbachia;s__Wolbachia_pipientis
PB2_gene_5250	460	ORM78942.1	460	457/460(99.35)	0	ATP-dependent RNA helicase DbpA [Pantoea deleyi]	470932	d__Bacteria;k__norank;p__Proteobacteria;c__Gammaproteobacteria;o__Enterobacterales;f__Erwiniaceae;g__Pantoea;s__Pantoea_deleyi
PB2_gene_5259	451	WP_111140574.1	451	433/451(96.01)	9.31e-316	formimidoylglutamate deiminase [Pantoea sp. ARC607]	2027922	d__Bacteria;k__norank;p__Proteobacteria;c__Gammaproteobacteria;o__Enterobacterales;f__Erwiniaceae;g__Pantoea;s__Pantoea_sp._ARC607
PB2_gene_5260	138	SWV26764.1	142	124/138(89.86)	6.57e-85	transposase [Klebsiella pneumoniae]	573	d__Bacteria;k__norank;p__Proteobacteria;c__Gammaproteobacteria;o__Enterobacterales;f__Enterobacteriaceae;g__Klebsiella;s__Klebsiella_pneumoniae
PB2_gene_5261	160	SCA39194.1	321	116/134(86.57)	1.53e-79	transposase [Klebsiella pneumoniae]	1463165	d__Bacteria;k__norank;p__Proteobacteria;c__Gammaproteobacteria;o__Enterobacterales;f__Enterobacteriaceae;g__Klebsiella;s__Klebsiella_quasipneumoniae
PB2_gene_5262	70	WP_079893924.1	1002	65/70(92.86)	1.60e-36	Tn3 family transposase [Salmonella enterica]	28901	d__Bacteria;k__norank;p__Proteobacteria;c__Gammaproteobacteria;o__Enterobacterales;f__Enterobacteriaceae;g__Salmonella;s__Salmonella_enterica
PB2_gene_5269	437	WP_111138450.1	437	425/437(97.25)	4.17e-293	MFS transporter [Pantoea sp. ARC607]	2027922	d__Bacteria;k__norank;p__Proteobacteria;c__Gammaproteobacteria;o__Enterobacterales;f__Erwiniaceae;g__Pantoea;s__Pantoea_sp._ARC607
PB2_gene_5279	575	WP_111139514.1	1320	529/575(92.00)	0	BapA prefix-like domain-containing protein [Pantoea sp.	2027922	d__Bacteria;k__norank;p__Proteobacteria;c__Gammaproteobacteria;o__Enterobacterales;f__Erwiniaceae;g__Pantoea;s__Pantoea_sp._ARC607
PB2_gene_5287	456	PCG11072.1	627	440/449(98.00)	6.70e-316	DUF3857 domain-containing protein [Wolbachia pipientis]	None	Unclassified
PB2_gene_5288	572	WP_010405317.1	883	559/562(99.47)	0	preprotein translocase subunit SecA [Wolbachia endosymbiont of Nasonia vitripennis]	None	Unclassified
PB2_gene_5291	506	ORM80746.1	499	492/499(98.60)	0	hypothetical protein HA42_13270 [Pantoea deleyi]	470932	d__Bacteria;k__norank;p__Proteobacteria;c__Gammaproteobacteria;o__Enterobacterales;f__Erwiniaceae;g__Pantoea;s__Pantoea_deleyi
PB2_gene_5294	345	WP_111142215.1	345	331/345(95.94)	3.01e-244	UDP-N-acetylmuramate dehydrogenase [Pantoea sp. ARC607]	2027922	d__Bacteria;k__norank;p__Proteobacteria;c__Gammaproteobacteria;o__Enterobacterales;f__Erwiniaceae;g__Pantoea;s__Pantoea_sp._ARC607
PB2_gene_5313	124	KKB02732.1	952	68/120(56.67)	2.25e-24	hypothetical protein TN98_21080, partial [Pantoea	470931	d__Bacteria;k__norank;p__Proteobacteria;c__Gammaproteobacteria;o__Enterobacterales;f__Erwiniaceae;g__Pantoea;s__Pantoea_anthophila
PB2_gene_5315	207	WP_046103198.1	207	204/207(98.55)	2.34e-138	response regulator [Pantoea anthophila]	470931	d__Bacteria;k__norank;p__Proteobacteria;c__Gammaproteobacteria;o__Enterobacterales;f__Erwiniaceae;g__Pantoea;s__Pantoea_anthophila
PB2_gene_5317	445	ORM76523.1	445	427/445(95.96)	0	porin [Pantoea deleyi]	470932	d__Bacteria;k__norank;p__Proteobacteria;c__Gammaproteobacteria;o__Enterobacterales;f__Erwiniaceae;g__Pantoea;s__Pantoea_deleyi
PB2_gene_5325	370	ORM81043.1	370	365/370(98.65)	3.41e-274	N-methyltryptophan oxidase [Pantoea deleyi]	470932	d__Bacteria;k__norank;p__Proteobacteria;c__Gammaproteobacteria;o__Enterobacterales;f__Erwiniaceae;g__Pantoea;s__Pantoea_deleyi
PB2_gene_5326	111	ORM81044.1	83	82/83(98.80)	3.93e-51	transcriptional regulator [Pantoea deleyi]	470932	d__Bacteria;k__norank;p__Proteobacteria;c__Gammaproteobacteria;o__Enterobacterales;f__Erwiniaceae;g__Pantoea;s__Pantoea_deleyi

PB2_gene_5335	210	EJK89546.1	284	98/199(49.25)	1.19e-50	hypothetical protein UUU_41690 [Klebsiella pneumoniae subsp. pneumoniae DSM 30104]	1162296	d__Bacteria;k__norank;p__Proteobacteria;c__Gammaproteobacteria;o__Enterobacterales;f__Enterobacteriaceae;g__Klebsiella;s__Klebsiella_pneumoniae
PB2_gene_5344	164	ADO08474.1	164	146/164(89.02)	1.83e-89	Uricase (Urate oxidase) [Pantoea vagans C9-1]	712898	d__Bacteria;k__norank;p__Proteobacteria;c__Gammaproteobacteria;o__Enterobacterales;f__Erwiniaceae;g__Pantoea;s__Pantoea_vagans
PB2_gene_5347	98	WP_111139223.1	249	92/98(93.88)	6.07e-58	esterase [Pantoea sp. ARC607]	2027922	d__Bacteria;k__norank;p__Proteobacteria;c__Gammaproteobacteria;o__Enterobacterales;f__Erwiniaceae;g__Pantoea;s__Pantoea_sp._ARC607
PB2_gene_5348	68	ORM82491.1	68	67/68(98.53)	1.20e-40	hypothetical protein HA42_08310 [Pantoea deleyi]	470932	d__Bacteria;k__norank;p__Proteobacteria;c__Gammaproteobacteria;o__Enterobacterales;f__Erwiniaceae;g__Pantoea;s__Pantoea_deleyi
PB2_gene_5358	478	WP_111141568.1	478	472/478(98.74)	0	alpha,alpha-trehalose-phosphate synthase [Pantoea sp. ARC607]	2027922	d__Bacteria;k__norank;p__Proteobacteria;c__Gammaproteobacteria;o__Enterobacterales;f__Erwiniaceae;g__Pantoea;s__Pantoea_sp._ARC607
PB2_gene_5361	255	PCG11005.1	329	254/255(99.61)	1.72e-179	ribonucleotide-diphosphate reductase subunit beta [Wolbachia pipientis]	None	Unclassified
PB2_gene_5369	431	WP_019236337.1	507	422/431(97.91)	6.95e-303	hypothetical protein [Wolbachia pipientis]	955	d__Bacteria;k__norank;p__Proteobacteria;c__Alphaproteobacteria;o__Rickettsiales;f__Anaplasmataceae;g__Wolbachia;s__Wolbachia_pipientis
PB2_gene_5370	414	OJH31405.1	404	284/411(69.10)	1.06e-193	DNA repair protein RadA [Wolbachia endosymbiont of Armadillidium vulgare]	77039	d__Bacteria;k__norank;p__Proteobacteria;c__Alphaproteobacteria;o__Rickettsiales;f__Anaplasmataceae;g__Wolbachia;s__Wolbachia_endosymbiont_of_Armadillidium_vulgare
PB2_gene_5393	137	CCF11341.1	137	135/137(98.54)	2.54e-101	glyoxalase/bleomycin resistance protein/dioxygenase [Pantoea ananatis LMG 5342]	1123863	d__Bacteria;k__norank;p__Proteobacteria;c__Gammaproteobacteria;o__Enterobacterales;f__Erwiniaceae;g__Pantoea;s__Pantoea_ananatis
PB2_gene_5407	43	ELP25057.1	180	33/42(78.57)	3.62e-14	Prophage baseplate assembly protein V [Pantoea agglomerans 299R]	1261128	d__Bacteria;k__norank;p__Proteobacteria;c__Gammaproteobacteria;o__Enterobacterales;f__Erwiniaceae;g__Pantoea;s__Pantoea_agglomerans
PB2_gene_5408	138	WP_033761751.1	138	126/138(91.30)	1.78e-88	phage GP46 family protein [Pantoea agglomerans]	None	Unclassified
PB2_gene_5415	199	AZU38019.1	199	198/199(99.50)	3.94e-142	alkyl hydroperoxide reductase [Wolbachia endosymbiont of Bemisia tabaci]	215173	d__Bacteria;k__norank;p__Proteobacteria;c__Alphaproteobacteria;o__Rickettsiales;f__Anaplasmataceae;g__Wolbachia;s__Wolbachia_endosymbiont_of_Bemisia_tabaci
PB2_gene_5420	97	WP_061063408.1	143	57/66(86.36)	1.13e-28	phage tail protein [Pantoea vagans]	470934	d__Bacteria;k__norank;p__Proteobacteria;c__Gammaproteobacteria;o__Enterobacterales;f__Erwiniaceae;g__Pantoea;s__Pantoea_vagans
PB2_gene_5425	110	ORM54938.1	110	110/110(100.00)	2.51e-68	50S ribosomal protein L22 [Pantoea brenneri]	472694	d__Bacteria;k__norank;p__Proteobacteria;c__Gammaproteobacteria;o__Enterobacterales;f__Erwiniaceae;g__Pantoea;s__Pantoea_brenneri
PB2_gene_5428	469	WP_111142195.1	435	352/420(83.81)	1.73e-242	MFS transporter, partial [Pantoea sp. ARC607]	2027922	d__Bacteria;k__norank;p__Proteobacteria;c__Gammaproteobacteria;o__Enterobacterales;f__Erwiniaceae;g__Pantoea;s__Pantoea_sp._ARC607
PB2_gene_5437	245	PBQ29276.1	245	243/244(99.59)	6.97e-177	glutamine synthetase [Wolbachia pipientis wAus]	2049495	d__Bacteria;k__norank;p__Proteobacteria;c__Alphaproteobacteria;o__Rickettsiales;f__Anaplasmataceae;g__Wolbachia;s__Wolbachia_pipientis
PB2_gene_5440	186	SAI94527.1	421	89/186(47.85)	2.18e-33	Uncharacterised protein [Enterobacter cloacae]	550	d__Bacteria;k__norank;p__Proteobacteria;c__Gammaproteobacteria;o__Enterobacterales;f__Enterobacteriaceae;g__Enterobacter;s__Enterobacter_cloacae
PB2_gene_5447	251	ORM75931.1	251	251/251(100.00)	4.03e-179	histidine utilization repressor [Pantoea deleyi]	470932	d__Bacteria;k__norank;p__Proteobacteria;c__Gammaproteobacteria;o__Enterobacterales;f__Erwiniaceae;g__Pantoea;s__Pantoea_deleyi
PB2_gene_5463	216	WP_019236555.1	299	212/216(98.15)	6.24e-137	DMT family transporter [Wolbachia pipientis]	955	d__Bacteria;k__norank;p__Proteobacteria;c__Alphaproteobacteria;o__Rickettsiales;f__Anaplasmataceae;g__Wolbachia;s__Wolbachia_pipientis
PB2_gene_5468	312	WP_111139109.1	407	310/312(99.36)	1.08e-225	cysteine desulfurase SufS [Pantoea sp. ARC607]	2027922	d__Bacteria;k__norank;p__Proteobacteria;c__Gammaproteobacteria;o__Enterobacterales;f__Erwiniaceae;g__Pantoea;s__Pantoea_sp._ARC607
PB2_gene_5469	138	OQV43048.1	138	136/138(98.55)	3.47e-95	cysteine desufuration protein SufE [Pantoea vagans]	470934	d__Bacteria;k__norank;p__Proteobacteria;c__Gammaproteobacteria;o__Enterobacterales;f__Erwiniaceae;g__Pantoea;s__Pantoea_vagans
PB2_gene_5481	85	WP_081998143.1	560	28/35(80.00)	1.35e-07	DUF4942 domain-containing protein [Pantoea rodasii]	1076549	d__Bacteria;k__norank;p__Proteobacteria;c__Gammaproteobacteria;o__Enterobacterales;f__Erwiniaceae;g__Pantoea;s__Pantoea_rodasii

PB2_gene_5486	119	WP_111138754.1	124	113/119(94.96)	2.16e-72	DUF1232 domain-containing protein [Pantoea sp. ARC607]	2027922	d__Bacteria;k__norank;p__Proteobacteria;c__Gammaproteobacteria;o__Enterobacterales;f__Erwiniaceae;g__Pantoea;s__Pantoea_sp._ARC607
PB2_gene_5496	350	ORM78716.1	350	349/350(99.71)	1.06e-251	3-deoxy-7-phosphoheptulonate synthase [Pantoea deleyi]	470932	d__Bacteria;k__norank;p__Proteobacteria;c__Gammaproteobacteria;o__Enterobacterales;f__Erwiniaceae;g__Pantoea;s__Pantoea_deleyi
PB2_gene_5498	247	ORM86811.1	247	247/247(100.00)	1.76e-171	hypothetical protein HA42_00075 [Pantoea deleyi]	470932	d__Bacteria;k__norank;p__Proteobacteria;c__Gammaproteobacteria;o__Enterobacterales;f__Erwiniaceae;g__Pantoea;s__Pantoea_deleyi
PB2_gene_5502	239	WP_111138398.1	302	231/239(96.65)	1.86e-161	ABC transporter substrate-binding protein [Pantoea sp.	2027922	d__Bacteria;k__norank;p__Proteobacteria;c__Gammaproteobacteria;o__Enterobacterales;f__Erwiniaceae;g__Pantoea;s__Pantoea_sp._ARC607
PB2_gene_5503	260	ORM80366.1	283	259/260(99.62)	1.18e-181	glutamate racemase [Pantoea deleyi]	470932	d__Bacteria;k__norank;p__Proteobacteria;c__Gammaproteobacteria;o__Enterobacterales;f__Erwiniaceae;g__Pantoea;s__Pantoea_deleyi
PB2_gene_5511	108	ADI78556.1	108	93/108(86.11)	1.71e-56	hypothetical protein Pvag_pPag30487 (plasmid) [Pantoea vagans C9-1]	712898	d__Bacteria;k__norank;p__Proteobacteria;c__Gammaproteobacteria;o__Enterobacterales;f__Erwiniaceae;g__Pantoea;s__Pantoea_vagans
PB2_gene_5516	517	WP_099975922.1	648	210/522(40.23)	1.60e-107	hypothetical protein [Burkholderia thailandensis]	32008	d__Bacteria;k__norank;p__Proteobacteria;c__Betaproteobacteria;o__Burkholderiales;f__Burkholderiaceae;g__Burkholderia;s__norank
PB2_gene_5519	105	ORM86573.1	306	101/105(96.19)	1.41e-60	LysR family transcriptional regulator [Pantoea deleyi]	470932	d__Bacteria;k__norank;p__Proteobacteria;c__Gammaproteobacteria;o__Enterobacterales;f__Erwiniaceae;g__Pantoea;s__Pantoea_deleyi
PB2_gene_5528	160	PBQ26332.1	160	160/160(100.00)	4.85e-108	single-stranded DNA-binding protein [Wolbachia pipientis	2049495	d__Bacteria;k__norank;p__Proteobacteria;c__Alphaproteobacteria;o__Rickettsiales;f__Anaplasmataceae;g__Wolbachia;s__Wolbachia_pipientis
PB2_gene_5531	282	ORM75489.1	225	219/225(97.33)	1.21e-150	hypothetical protein HA42_20545 [Pantoea deleyi]	470932	d__Bacteria;k__norank;p__Proteobacteria;c__Gammaproteobacteria;o__Enterobacterales;f__Erwiniaceae;g__Pantoea;s__Pantoea_deleyi
PB2_gene_5557	259	WP_111140760.1	259	254/259(98.07)	2.50e-177	deoxyribose-phosphate aldolase [Pantoea sp. ARC607]	2027922	d__Bacteria;k__norank;p__Proteobacteria;c__Gammaproteobacteria;o__Enterobacterales;f__Erwiniaceae;g__Pantoea;s__Pantoea_sp._ARC607
PB2_gene_5559	87	ETW98900.1	920	35/83(42.17)	9.40e-12	hypothetical protein ETSY2_42015 [Candidatus	1429439	d__Bacteria;k__norank;p__Candidatus_Tectomicrobia;c__norank;o__norank;f__norank;g__Candidatus_Entotheonella;s__Candidatus_Entotheonella_gemina
PB2_gene_5576	426	WP_111142026.1	788	418/426(98.12)	3.60e-297	SrfC [Pantoea sp. ARC607]	2027922	d__Bacteria;k__norank;p__Proteobacteria;c__Gammaproteobacteria;o__Enterobacterales;f__Erwiniaceae;g__Pantoea;s__Pantoea_sp._ARC607
PB2_gene_5580	328	ORM78832.1	328	312/328(95.12)	2.04e-222	oxidoreductase [Pantoea deleyi]	470932	d__Bacteria;k__norank;p__Proteobacteria;c__Gammaproteobacteria;o__Enterobacterales;f__Erwiniaceae;g__Pantoea;s__Pantoea_deleyi
PB2_gene_5590	77	GAN10912.1	754	23/59(38.98)	1.67e-06	pap 25a associated domain family protein [Mucor ambiguus]	91626	d__Eukaryota;k__Fungi;p__Mucoromycota;c__Mucoromycetes;o__Mucorales;f__Mucoraceae;g__Mucor;s__Mucor_ambiguus
PB2_gene_5593	221	ORM85809.1	219	207/219(94.52)	2.43e-151	octanoyltransferase [Pantoea deleyi]	470932	d__Bacteria;k__norank;p__Proteobacteria;c__Gammaproteobacteria;o__Enterobacterales;f__Erwiniaceae;g__Pantoea;s__Pantoea_deleyi
PB2_gene_5595	185	ORM85659.1	185	181/185(97.84)	2.21e-123	transcriptional regulator [Pantoea deleyi]	470932	d__Bacteria;k__norank;p__Proteobacteria;c__Gammaproteobacteria;o__Enterobacterales;f__Erwiniaceae;g__Pantoea;s__Pantoea_deleyi
PB2_gene_5599	92	ORM75966.1	92	92/92(100.00)	4.76e-60	pyrroloquinoline quinone biosynthesis protein PqqD [Pantoea deleyi]	470932	d__Bacteria;k__norank;p__Proteobacteria;c__Gammaproteobacteria;o__Enterobacterales;f__Erwiniaceae;g__Pantoea;s__Pantoea_deleyi
PB2_gene_5609	191	ORM86359.1	173	173/173(100.00)	4.02e-118	protease [Pantoea deleyi]	470932	d__Bacteria;k__norank;p__Proteobacteria;c__Gammaproteobacteria;o__Enterobacterales;f__Erwiniaceae;g__Pantoea;s__Pantoea_deleyi
PB2_gene_5610	141	ORM86358.1	142	130/140(92.86)	1.06e-93	hypothetical protein HA42_01125 [Pantoea deleyi]	470932	d__Bacteria;k__norank;p__Proteobacteria;c__Gammaproteobacteria;o__Enterobacterales;f__Erwiniaceae;g__Pantoea;s__Pantoea_deleyi
PB2_gene_5625	270	ORM80453.1	270	269/270(99.63)	1.34e-182	DeoR family transcriptional regulator [Pantoea deleyi]	470932	d__Bacteria;k__norank;p__Proteobacteria;c__Gammaproteobacteria;o__Enterobacterales;f__Erwiniaceae;g__Pantoea;s__Pantoea_deleyi
PB2_gene_5626	89	QAV47603.1	334	87/89(97.75)	4.11e-58	conjugal transfer protein TraU (plasmid) [Pantoea agglomerans]	549	d__Bacteria;k__norank;p__Proteobacteria;c__Gammaproteobacteria;o__Enterobacterales;f__Erwiniaceae;g__Pantoea;s__Pantoea_agglomerans
PB2_gene_5627	268	QAV47604.1	268	264/268(98.51)	6.35e-180	DsbA family protein (plasmid) [Pantoea agglomerans]	549	d__Bacteria;k__norank;p__Proteobacteria;c__Gammaproteobacteria;o__Enterobacterales;f__Erwiniaceae;g__Pantoea;s__Pantoea_agglomerans
PB2_gene_5628	130	KIC86372.1	199	129/130(99.23)	7.16e-88	hypothetical protein RN49_13930 [Pantoea agglomerans]	549	d__Bacteria;k__norank;p__Proteobacteria;c__Gammaproteobacteria;o__Enterobacterales;f__Erwiniaceae;g__Pantoea;s__Pantoea_agglomerans

PB2_gene_5641	71	WP_079967714.1	71	62/71(87.32)	8.04e-27	mobilization protein [Salmonella enterica]	1236	d__Bacteria;k__norank;p__Proteobacteria;c__Gammaproteobacteria;o__norank;f__norank;g__norank;s__norank
PB2_gene_5642	161	SQC88734.1	161	154/161(95.65)	3.93e-78	MbeB-like, N-term conserved region [Klebsiella pneumoniae]	573	d__Bacteria;k__norank;p__Proteobacteria;c__Gammaproteobacteria;o__Enterobacterales;f__Enterobacteriaceae;g__Klebsiella;s__Klebsiella_pneumoniae
PB2_gene_5643	384	EEB56179.1	444	355/384(92.45)	1.28e-238	sensor histidine kinase [Wolbachia endosymbiont of Culex quinquefasciatus JHB]	569881	d__Bacteria;k__norank;p__Proteobacteria;c__Alphaproteobacteria;o__Rickettsiales;f__Anaplasmataceae;g__Wolbachia;s__Wolbachia_endosymbiont_of_Culex_quinquefasciatus
PB2_gene_5649	59	WP_111138541.1	169	58/59(98.31)	3.43e-34	GNAT family N-acetyltransferase [Pantoea sp. ARC607]	2027922	d__Bacteria;k__norank;p__Proteobacteria;c__Gammaproteobacteria;o__Enterobacterales;f__Erwiniaceae;g__Pantoea;s__Pantoea_sp._ARC607
PB2_gene_5650	253	WP_111138542.1	253	246/253(97.23)	3.31e-179	tRNA-(ms[2]io[6]A)-hydroxylase [Pantoea sp. ARC607]	2027922	d__Bacteria;k__norank;p__Proteobacteria;c__Gammaproteobacteria;o__Enterobacterales;f__Erwiniaceae;g__Pantoea;s__Pantoea_sp._ARC607
PB2_gene_5664	183	WP_111141435.1	174	171/174(98.28)	4.99e-117	regulatory protein RecX [Pantoea sp. ARC607]	2027922	d__Bacteria;k__norank;p__Proteobacteria;c__Gammaproteobacteria;o__Enterobacterales;f__Erwiniaceae;g__Pantoea;s__Pantoea_sp._ARC607
PB2_gene_5679	370	SUB08583.1	505	346/370(93.51)	1.66e-238	Probable licABCH operon regulator [Pantoea agglomerans]	549	d__Bacteria;k__norank;p__Proteobacteria;c__Gammaproteobacteria;o__Enterobacterales;f__Erwiniaceae;g__Pantoea;s__Pantoea_agglomerans
PB2_gene_5688	180	WP_024906931.1	180	140/180(77.78)	1.26e-90	TetR/AcrR family transcriptional regulator [Enterobacter asburiae]	61645	d__Bacteria;k__norank;p__Proteobacteria;c__Gammaproteobacteria;o__Enterobacterales;f__Enterobacteriaceae;g__Enterobacter;s__Enterobacter_asburiae
PB2_gene_5689	372	PZL97515.1	372	369/372(99.19)	2.29e-248	hypothetical protein CKF43_04445 [Pantoea sp.	2027922	d__Bacteria;k__norank;p__Proteobacteria;c__Gammaproteobacteria;o__Enterobacterales;f__Erwiniaceae;g__Pantoea;s__Pantoea_sp._ARC607
PB2_gene_5693	220	ORM80954.1	220	206/220(93.64)	7.78e-148	hypothetical protein HA42_12455 [Pantoea deleyi]	470932	d__Bacteria;k__norank;p__Proteobacteria;c__Gammaproteobacteria;o__Enterobacterales;f__Erwiniaceae;g__Pantoea;s__Pantoea_deleyi
PB2_gene_5695	365	ORM79066.1	725	349/365(95.62)	4.21e-240	diguanylate cyclase [Pantoea deleyi]	470932	d__Bacteria;k__norank;p__Proteobacteria;c__Gammaproteobacteria;o__Enterobacterales;f__Erwiniaceae;g__Pantoea;s__Pantoea_deleyi
PB2_gene_5696	216	ORM77995.1	212	210/212(99.06)	7.04e-148	DNA-binding transcriptional repressor AcrR [Pantoea deleyi]	470932	d__Bacteria;k__norank;p__Proteobacteria;c__Gammaproteobacteria;o__Enterobacterales;f__Erwiniaceae;g__Pantoea;s__Pantoea_deleyi
PB2_gene_5710	59	KKB04914.1	241	55/63(87.30)	6.56e-27	hypothetical protein TN98_09405 [Pantoea anthophila]	470931	d__Bacteria;k__norank;p__Proteobacteria;c__Gammaproteobacteria;o__Enterobacterales;f__Erwiniaceae;g__Pantoea;s__Pantoea_anthophila
PB2_gene_5711	129	WP_099258003.1	129	122/129(94.57)	2.71e-90	hypothetical protein [Pantoea agglomerans]	549	d__Bacteria;k__norank;p__Proteobacteria;c__Gammaproteobacteria;o__Enterobacterales;f__Erwiniaceae;g__Pantoea;s__Pantoea_agglomerans
PB2_gene_5712	218	WP_033732359.1	1338	202/215(93.95)	2.30e-139	type IV secretion protein Rhs [Pantoea vagans]	470934	d__Bacteria;k__norank;p__Proteobacteria;c__Gammaproteobacteria;o__Enterobacterales;f__Erwiniaceae;g__Pantoea;s__Pantoea_vagans
PB2_gene_5716	243	WP_046102035.1	232	167/231(72.29)	4.59e-112	ankyrin repeat domain-containing protein [Pantoea anthophila]	53335	d__Bacteria;k__norank;p__Proteobacteria;c__Gammaproteobacteria;o__Enterobacterales;f__Erwiniaceae;g__Pantoea;s__norank
PB2_gene_5719	89	VEB50968.1	322	88/89(98.88)	1.97e-53	Type IV secretory pathway, VirB4 components [Salmonella enterica subsp. enterica]	59201	d__Bacteria;k__norank;p__Proteobacteria;c__Gammaproteobacteria;o__Enterobacterales;f__Enterobacteriaceae;g__Salmonella;s__Salmonella_enterica
PB2_gene_5724	414	ORM79873.1	414	402/414(97.10)	7.04e-303	cardiolipin synthase B [Pantoea deleyi]	470932	d__Bacteria;k__norank;p__Proteobacteria;c__Gammaproteobacteria;o__Enterobacterales;f__Erwiniaceae;g__Pantoea;s__Pantoea_deleyi
PB2_gene_5726	115	SUB09005.1	101	79/99(79.80)	9.32e-50	HSP20-like domain of uncharacterised function (DUF1813) [Pantoea	549	d__Bacteria;k__norank;p__Proteobacteria;c__Gammaproteobacteria;o__Enterobacterales;f__Erwiniaceae;g__Pantoea;s__Pantoea_agglomerans
PB2_gene_5727	74	WP_010671630.1	74	71/74(95.95)	1.68e-45	type I toxin-antitoxin system SymE family toxin [Pantoea	549	d__Bacteria;k__norank;p__Proteobacteria;c__Gammaproteobacteria;o__Enterobacterales;f__Erwiniaceae;g__Pantoea;s__Pantoea_agglomerans
PB2_gene_5728	93	CVG49806.1	264	41/70(58.57)	2.60e-09	Uncharacterised protein [Serratia marcescens]	615	d__Bacteria;k__norank;p__Proteobacteria;c__Gammaproteobacteria;o__Enterobacterales;f__Yersiniaceae;g__Serratia;s__Serratia_marcescens
PB2_gene_5733	452	EIP84389.1	1540	211/446(47.31)	3.86e-98	hypothetical protein A33K_18956 [Burkholderia thailandensis MSMB43]	441157	d__Bacteria;k__norank;p__Proteobacteria;c__Betaproteobacteria;o__Burkholderiales;f__Burkholderiaceae;g__Burkholderia;s__Burkholderia_thailandensis
PB2_gene_5744	92	ADD77487.1	116	41/83(49.40)	9.03e-19	Hypothetical Protein PANA_2320 [Pantoea ananatis	706191	d__Bacteria;k__norank;p__Proteobacteria;c__Gammaproteobacteria;o__Enterobacterales;f__Erwiniaceae;g__Pantoea;s__Pantoea_ananatis

PB2_gene_5775	79	ONI58028.1	855	78/79(98.73)	4.07e-41	NADPH-dependent glutamate synthase beta chain [Wolbachia pipientis wVitA]	1379786	d__Bacteria;k__norank;p__Proteobacteria;c__Alphaproteobacteria;o__Rickettsiales;f__Anaplasmataceae;g__Wolbachia;s__Wolbachia_pipientis
PB2_gene_5779	460	PXF62377.1	965	113/448(25.22)	3.76e-30	hypothetical protein DL796_11835, partial [Kangiella	796379	d__Bacteria;k__norank;p__Proteobacteria;c__Gammaproteobacteria;o__Oceanospirillales;f__Kangiellaceae;g__Kangiella;s__Kangiella_spongicola
PB2_gene_5789	176	BAK13732.1	332	140/152(92.11)	2.87e-84	D-ribose-binding periplasmic protein precursor RbsB [Pantoea ananatis AJ13355]	932677	d__Bacteria;k__norank;p__Proteobacteria;c__Gammaproteobacteria;o__Enterobacterales;f__Erwiniaceae;g__Pantoea;s__Pantoea_ananatis
PB2_gene_5790	190	VED51702.1	158	93/153(60.78)	5.27e-57	Uncharacterised protein [Klebsiella aerogenes]	577	d__Bacteria;k__norank;p__Proteobacteria;c__Gammaproteobacteria;o__Enterobacterales;f__Enterobacteriaceae;g__Raoultella;s__Raoultella_terrigena
PB2_gene_5791	438	WP_127464074.1	627	376/438(85.84)	2.98e-251	hypothetical protein [Wolbachia endosymbiont of Bemisia tabaci]	215173	d__Bacteria;k__norank;p__Proteobacteria;c__Alphaproteobacteria;o__Rickettsiales;f__Anaplasmataceae;g__Wolbachia;s__Wolbachia_endosymbiont_of_Bemisia_tabaci
PB2_gene_5793	270	WP_039390089.1	720	227/245(92.65)	1.07e-158	type IV conjugative transfer system coupling protein TraD [Pantoea agglomerans]	549	d__Bacteria;k__norank;p__Proteobacteria;c__Gammaproteobacteria;o__Enterobacterales;f__Erwiniaceae;g__Pantoea;s__Pantoea_agglomerans
PB2_gene_5797	344	ORM85631.1	344	338/344(98.26)	1.63e-252	amylovoran biosynthesis protein AmsD [Pantoea deleyi]	470932	d__Bacteria;k__norank;p__Proteobacteria;c__Gammaproteobacteria;o__Enterobacterales;f__Erwiniaceae;g__Pantoea;s__Pantoea_deleyi
PB2_gene_5802	237	ORM79062.1	237	236/237(99.58)	4.53e-169	phosphonate metabolism transcriptional regulator PhnF [Pantoea deleyi]	470932	d__Bacteria;k__norank;p__Proteobacteria;c__Gammaproteobacteria;o__Enterobacterales;f__Erwiniaceae;g__Pantoea;s__Pantoea_deleyi
PB2_gene_5809	154	ELP25064.1	172	146/154(94.81)	4.36e-103	hypothetical protein F385_1935 [Pantoea agglomerans 299R]	1261128	d__Bacteria;k__norank;p__Proteobacteria;c__Gammaproteobacteria;o__Enterobacterales;f__Erwiniaceae;g__Pantoea;s__Pantoea_agglomerans
PB2_gene_5811	109	WP_033761760.1	105	94/105(89.52)	9.04e-63	phage gp6-like head-tail connector protein [Pantoea	None	Unclassified
PB2_gene_5813	88	ORM81917.1	88	88/88(100.00)	2.33e-56	hypothetical protein HA42_09550 [Pantoea deleyi]	470932	d__Bacteria;k__norank;p__Proteobacteria;c__Gammaproteobacteria;o__Enterobacterales;f__Erwiniaceae;g__Pantoea;s__Pantoea_deleyi
PB2_gene_5818	316	WP_111139666.1	483	307/315(97.46)	5.22e-213	NAD-dependent succinate-semialdehyde dehydrogenase [Pantoea sp. ARC607]	2027922	d__Bacteria;k__norank;p__Proteobacteria;c__Gammaproteobacteria;o__Enterobacterales;f__Erwiniaceae;g__Pantoea;s__Pantoea_sp._ARC607
PB2_gene_5823	270	WP_105088670.1	464	259/269(96.28)	2.84e-179	His-Xaa-Ser system radical SAM maturase HxsB [Pantoea ananatis]	553	d__Bacteria;k__norank;p__Proteobacteria;c__Gammaproteobacteria;o__Enterobacterales;f__Erwiniaceae;g__Pantoea;s__Pantoea_ananatis
PB2_gene_5832	158	ORM78385.1	168	151/158(95.57)	6.01e-92	hypothetical protein HA42_16730 [Pantoea deleyi]	470932	d__Bacteria;k__norank;p__Proteobacteria;c__Gammaproteobacteria;o__Enterobacterales;f__Erwiniaceae;g__Pantoea;s__Pantoea_deleyi
PB2_gene_5835	47	PCG11639.1	262	47/47(100.00)	4.46e-23	succinate dehydrogenase iron-sulfur subunit [Wolbachia	None	Unclassified
PB2_gene_5839	355	WP_111140151.1	355	348/355(98.03)	1.18e-246	sugar ABC transporter substrate-binding protein [Pantoea sp. ARC607]	2027922	d__Bacteria;k__norank;p__Proteobacteria;c__Gammaproteobacteria;o__Enterobacterales;f__Erwiniaceae;g__Pantoea;s__Pantoea_sp._ARC607
PB2_gene_5851	341	WP_064384551.1	341	307/341(90.03)	4.89e-232	hypothetical protein [Klebsiella oxytoca]	575	d__Bacteria;k__norank;p__Proteobacteria;c__Gammaproteobacteria;o__Enterobacterales;f__Enterobacteriaceae;g__Raoultella;s__Raoultella_planticola
PB2_gene_5860	345	WP_083495750.1	352	336/345(97.39)	8.82e-241	peptide chain release factor 2 [Wolbachia endosymbiont of Trichogramma pretiosum]	125593	d__Bacteria;k__norank;p__Proteobacteria;c__Alphaproteobacteria;o__Rickettsiales;f__Anaplasmataceae;g__Wolbachia;s__Wolbachia_endosymbiont_of_Trichogramma_pretiosum
PB2_gene_5863	336	ADO10187.1	336	316/336(94.05)	2.00e-233	putative starvation sensing zinc-binding dehydrogenase [Pantoea vagans C9-1]	712898	d__Bacteria;k__norank;p__Proteobacteria;c__Gammaproteobacteria;o__Enterobacterales;f__Erwiniaceae;g__Pantoea;s__Pantoea_vagans
PB2_gene_5882	237	ORM77787.1	484	234/236(99.15)	1.55e-162	PTS N-acetylmuramic acid transporter subunits IIBC [Pantoea deleyi]	470932	d__Bacteria;k__norank;p__Proteobacteria;c__Gammaproteobacteria;o__Enterobacterales;f__Erwiniaceae;g__Pantoea;s__Pantoea_deleyi

PB2_gene_5896	244	ORM86110.1	244	240/244(98.36)	1.89e-144	hypothetical protein HA42_01345 [Pantoea deleyi]	470932	d__Bacteria;k__norank;p__Proteobacteria;c__Gammaproteobacteria;o__Enterobacterales;f__Erwiniaceae;g__Pantoea;s__Pantoea_deleyi
PB2_gene_5912	324	ORM86094.1	324	323/324(99.69)	1.52e-231	transcriptional regulator CysB [Pantoea deleyi]	470932	d__Bacteria;k__norank;p__Proteobacteria;c__Gammaproteobacteria;o__Enterobacterales;f__Erwiniaceae;g__Pantoea;s__Pantoea_deleyi
PB2_gene_5935	67	ORM75965.1	251	67/67(100.00)	3.52e-40	pyrroloquinoline quinone biosynthesis protein PqqC [Pantoea deleyi]	470932	d__Bacteria;k__norank;p__Proteobacteria;c__Gammaproteobacteria;o__Enterobacterales;f__Erwiniaceae;g__Pantoea;s__Pantoea_deleyi
PB2_gene_5936	305	ORM75964.1	305	301/305(98.69)	4.03e-231	pyrroloquinoline quinone biosynthesis protein PqqB [Pantoea deleyi]	470932	d__Bacteria;k__norank;p__Proteobacteria;c__Gammaproteobacteria;o__Enterobacterales;f__Erwiniaceae;g__Pantoea;s__Pantoea_deleyi
PB2_gene_5940	403	ORM82551.1	494	400/403(99.26)	1.27e-276	sodium/proline symporter [Pantoea deleyi]	470932	d__Bacteria;k__norank;p__Proteobacteria;c__Gammaproteobacteria;o__Enterobacterales;f__Erwiniaceae;g__Pantoea;s__Pantoea_deleyi
PB2_gene_5941	362	WP_111139432.1	1267	354/362(97.79)	1.56e-230	tetratricopeptide repeat protein [Pantoea sp. ARC607]	2027922	d__Bacteria;k__norank;p__Proteobacteria;c__Gammaproteobacteria;o__Enterobacterales;f__Erwiniaceae;g__Pantoea;s__Pantoea_sp._ARC607
PB2_gene_5954	288	WP_111139335.1	288	285/288(98.96)	1.95e-209	neutral zinc metallopeptidase [Pantoea sp. ARC607]	2027922	d__Bacteria;k__norank;p__Proteobacteria;c__Gammaproteobacteria;o__Enterobacterales;f__Erwiniaceae;g__Pantoea;s__Pantoea_sp._ARC607
PB2_gene_5964	309	WP_111139647.1	313	299/313(95.53)	3.86e-191	maltose operon protein MalM [Pantoea sp. ARC607]	2027922	d__Bacteria;k__norank;p__Proteobacteria;c__Gammaproteobacteria;o__Enterobacterales;f__Erwiniaceae;g__Pantoea;s__Pantoea_sp._ARC607
PB2_gene_5967	225	ORM81206.1	225	222/225(98.67)	9.88e-158	peptide ABC transporter ATP-binding protein [Pantoea deleyi]	470932	d__Bacteria;k__norank;p__Proteobacteria;c__Gammaproteobacteria;o__Enterobacterales;f__Erwiniaceae;g__Pantoea;s__Pantoea_deleyi
PB2_gene_5976	178	WP_111141531.1	178	175/178(98.31)	5.70e-118	ribose 1,5-bisphosphokinase [Pantoea sp. ARC607]	53335	d__Bacteria;k__norank;p__Proteobacteria;c__Gammaproteobacteria;o__Enterobacterales;f__Erwiniaceae;g__Pantoea;s__norank
PB2_gene_5978	263	WP_062749657.1	238	134/160(83.75)	2.69e-84	hypothetical protein [Erwinia persicina]	None	Unclassified
PB2_gene_5981	35	AZU37880.1	75	35/35(100.00)	5.50e-18	hypothetical protein BBB02_05375 [Wolbachia endosymbiont of Bemisia tabaci]	215173	d__Bacteria;k__norank;p__Proteobacteria;c__Alphaproteobacteria;o__Rickettsiales;f__Anaplasmataceae;g__Wolbachia;s__Wolbachia_endosymbiont_of_Bemisia_tabaci
PB2_gene_5998	117	ORM82477.1	117	117/117(100.00)	2.83e-84	gamma-glutamylcyclotransferase [Pantoea deleyi]	470932	d__Bacteria;k__norank;p__Proteobacteria;c__Gammaproteobacteria;o__Enterobacterales;f__Erwiniaceae;g__Pantoea;s__Pantoea_deleyi
PB2_gene_6000	135	PCG11625.1	133	132/133(99.25)	7.38e-77	50S ribosomal protein L7/L12 [Wolbachia pipientis]	None	Unclassified
PB2_gene_6002	79	WP_083429512.1	563	76/79(96.20)	3.73e-45	MULTISPECIES: ShlB/FhaC/HecB family hemolysin secretion/activation protein [Pantoea]	53335	d__Bacteria;k__norank;p__Proteobacteria;c__Gammaproteobacteria;o__Enterobacterales;f__Erwiniaceae;g__Pantoea;s__norank
PB2_gene_6008	374	WP_085016770.1	386	373/374(99.73)	1.12e-268	MULTISPECIES: C4-dicarboxylate ABC transporter	91347	d__Bacteria;k__norank;p__Proteobacteria;c__Gammaproteobacteria;o__Enterobacterales;f__norank;g__norank;s__norank
PB2_gene_6014	83	ORM84693.1	57	56/57(98.25)	4.71e-13	stress-induced protein [Pantoea deleyi]	470932	d__Bacteria;k__norank;p__Proteobacteria;c__Gammaproteobacteria;o__Enterobacterales;f__Erwiniaceae;g__Pantoea;s__Pantoea_deleyi
PB2_gene_6034	319	ORM83280.1	303	294/303(97.03)	3.35e-199	hypothetical protein HA42_06715 [Pantoea deleyi]	470932	d__Bacteria;k__norank;p__Proteobacteria;c__Gammaproteobacteria;o__Enterobacterales;f__Erwiniaceae;g__Pantoea;s__Pantoea_deleyi
PB2_gene_6045	107	WP_073981012.1	107	87/107(81.31)	2.86e-59	DUF1493 family protein [Erwinia sp. ErVv1]	1603299	d__Bacteria;k__norank;p__Proteobacteria;c__Gammaproteobacteria;o__Enterobacterales;f__Erwiniaceae;g__Erwinia;s__Erwinia_sp._ErVv1
PB2_gene_6046	146	KKF34305.1	146	118/146(80.82)	1.09e-86	hypothetical protein SY86_25430 [Erwinia tracheiphila]	65700	d__Bacteria;k__norank;p__Proteobacteria;c__Gammaproteobacteria;o__Enterobacterales;f__Erwiniaceae;g__Erwinia;s__Erwinia_tracheiphila
PB2_gene_6060	397	WP_111139870.1	689	369/390(94.62)	6.49e-247	bifunctional diguanylate cyclase/phosphodiesterase, partial [Pantoea sp. ARC607]	2027922	d__Bacteria;k__norank;p__Proteobacteria;c__Gammaproteobacteria;o__Enterobacterales;f__Erwiniaceae;g__Pantoea;s__Pantoea_sp._ARC607
PB2_gene_6073	328	WP_085016762.1	328	327/328(99.70)	2.46e-239	MULTISPECIES: integrase [Enterobacterales]	91347	d__Bacteria;k__norank;p__Proteobacteria;c__Gammaproteobacteria;o__Enterobacterales;f__norank;g__norank;s__norank

PB2_gene_6076	375	WP_094112323.1	420	294/349(84.24)	1.38e-173	hypothetical protein, partial [Pantoea eucrina]	None	Unclassified
PB2_gene_6078	233	EJL89406.1	233	189/233(81.12)	1.69e-137	arsenical resistance protein ArsH [Pantoea sp. GM01]	1144320	d__Bacteria;k__norank;p__Proteobacteria;c__Gammaproteobacteria;o__Enterobacterales;f__Erwiniaceae;g__Pantoea;s__Pantoea_sp._GM01
PB2_gene_6088	352	WP_124752618.1	718	60/247(24.29)	7.19e-06	hypothetical protein [Tannerella forsythia]	28112	d__Bacteria;k__norank;p__Bacteroidetes;c__Bacteroidia;o__Bacteroidales;f__Tannerellaceae;g__Tannerella;s__Tannerella_forsythia
PB2_gene_6092	125	WP_126690650.1	126	74/124(59.68)	2.36e-46	hypothetical protein [Pantoea sp. YU22]	None	Unclassified
PB2_gene_6096	390	WP_111140110.1	907	381/390(97.69)	2.52e-254	NADH-quinone oxidoreductase subunit NuoG [Pantoea sp.	2027922	d__Bacteria;k__norank;p__Proteobacteria;c__Gammaproteobacteria;o__Enterobacterales;f__Erwiniaceae;g__Pantoea;s__Pantoea_sp._ARC607
PB2_gene_6114	274	WP_095707018.1	274	267/274(97.45)	3.15e-183	FTR1 family protein [Pantoea vagans]	470934	d__Bacteria;k__norank;p__Proteobacteria;c__Gammaproteobacteria;o__Enterobacterales;f__Erwiniaceae;g__Pantoea;s__Pantoea_vagans
PB2_gene_6116	103	SUB15577.1	337	98/103(95.15)	1.04e-52	Uncharacterised protein [Pantoea agglomerans]	549	d__Bacteria;k__norank;p__Proteobacteria;c__Gammaproteobacteria;o__Enterobacterales;f__Erwiniaceae;g__Pantoea;s__Pantoea_agglomerans
PB2_gene_6130	299	WP_089527748.1	299	287/298(96.31)	2.54e-206	DUF4868 domain-containing protein [Pantoea ananatis]	553	d__Bacteria;k__norank;p__Proteobacteria;c__Gammaproteobacteria;o__Enterobacterales;f__Erwiniaceae;g__Pantoea;s__Pantoea_ananatis
PB2_gene_6137	166	WP_111142269.1	289	136/142(95.77)	1.69e-90	AraC family transcriptional regulator [Pantoea sp. ARC607]	2027922	d__Bacteria;k__norank;p__Proteobacteria;c__Gammaproteobacteria;o__Enterobacterales;f__Erwiniaceae;g__Pantoea;s__Pantoea_sp._ARC607
PB2_gene_6145	43	WP_130342508.1	541	25/42(59.52)	4.41e-08	carboxylesterase family protein [Herbihabitans rhizosphaerae]	1872711	d__Bacteria;k__norank;p__Actinobacteria;c__Actinobacteria;o__Pseudonocardiales;f__Pseudonocardiaceae;g__Herbihabitans;s__Herbihabitans_rhizosphaerae
PB2_gene_6146	323	VEA88348.1	487	315/323(97.52)	6.53e-226	DNA phosphorothioation-dependent restriction protein DptH [Salmonella enterica subsp. enterica serovar Goldcoast]	260678	d__Bacteria;k__norank;p__Proteobacteria;c__Gammaproteobacteria;o__Enterobacterales;f__Enterobacteriaceae;g__Salmonella;s__Salmonella_enterica
PB2_gene_6159	134	WP_046103196.1	204	126/134(94.03)	5.26e-83	response regulator transcription factor [Pantoea anthophila]	470931	d__Bacteria;k__norank;p__Proteobacteria;c__Gammaproteobacteria;o__Enterobacterales;f__Erwiniaceae;g__Pantoea;s__Pantoea_anthophila
PB2_gene_6166	251	ORM86816.1	251	250/251(99.60)	6.41e-176	zinc ABC transporter ATP-binding protein ZnuC [Pantoea	470932	d__Bacteria;k__norank;p__Proteobacteria;c__Gammaproteobacteria;o__Enterobacterales;f__Erwiniaceae;g__Pantoea;s__Pantoea_deleyi
PB2_gene_6201	52	WP_111140858.1	427	51/52(98.08)	4.56e-27	cystathionine gamma-synthase family protein [Pantoea sp.	2027922	d__Bacteria;k__norank;p__Proteobacteria;c__Gammaproteobacteria;o__Enterobacterales;f__Erwiniaceae;g__Pantoea;s__Pantoea_sp._ARC607
PB2_gene_6212	44	RIG55012.1	121	31/33(93.94)	2.72e-12	transposase, partial [Shigella flexneri]	623	d__Bacteria;k__norank;p__Proteobacteria;c__Gammaproteobacteria;o__Enterobacterales;f__Enterobacteriaceae;g__Shigella;s__Shigella_flexneri
PB2_gene_6216	58	ELP25062.1	700	51/58(87.93)	2.09e-25	Bacteriophage tail sheath protein [Pantoea agglomerans 299R]	1261128	d__Bacteria;k__norank;p__Proteobacteria;c__Gammaproteobacteria;o__Enterobacterales;f__Erwiniaceae;g__Pantoea;s__Pantoea_agglomerans
PB2_gene_6227	126	WP_086905642.1	124	98/122(80.33)	6.25e-67	hypothetical protein [Pantoea agglomerans]	549	d__Bacteria;k__norank;p__Proteobacteria;c__Gammaproteobacteria;o__Enterobacterales;f__Erwiniaceae;g__Pantoea;s__Pantoea_agglomerans
PB2_gene_6234	133	PJF19495.1	250	100/132(75.76)	2.70e-69	tRNA-dihydrouridine(47) synthase [NAD(P)(+)] [Paramicrosporidium	1246581	d__Eukaryota;k__Fungi;p__Cryptomycota;c__norank;o__norank;f__norank;g__Paramicrosporidium;s__Paramicrosporidium_saccamoebae
PB2_gene_6235	272	WP_111138523.1	242	236/242(97.52)	9.49e-170	gluconate 2-dehydrogenase subunit 3 family protein [Pantoea sp. ARC607]	2027922	d__Bacteria;k__norank;p__Proteobacteria;c__Gammaproteobacteria;o__Enterobacterales;f__Erwiniaceae;g__Pantoea;s__Pantoea_sp._ARC607
PB2_gene_6236	101	KXN69648.1	323	36/91(39.56)	4.11e-12	kinase-like protein [Conidiobolus coronatus NRRL 28638]	796925	d__Eukaryota;k__Fungi;p__Zoopagomycota;c__Entomophthoromycetes;o__Entomophthorales;f__Ancylistaceae;g__Conidiobolus;s__Conidiobolus_coronatus
PB2_gene_6245	179	ORM78178.1	178	175/178(98.31)	5.98e-127	hypothetical protein HA42_16980 [Pantoea deleyi]	470932	d__Bacteria;k__norank;p__Proteobacteria;c__Gammaproteobacteria;o__Enterobacterales;f__Erwiniaceae;g__Pantoea;s__Pantoea_deleyi
PB2_gene_6249	109	WP_090960067.1	471	92/109(84.40)	3.33e-44	flagellar filament capping protein FliD [Pantoea sp. OV426]	1881036	d__Bacteria;k__norank;p__Proteobacteria;c__Gammaproteobacteria;o__Enterobacterales;f__Erwiniaceae;g__Pantoea;s__Pantoea_sp._OV426
PB2_gene_6256	76	WP_105875183.1	80	46/71(64.79)	4.23e-25	hypothetical protein [Cronobacter malonaticus]	413503	d__Bacteria;k__norank;p__Proteobacteria;c__Gammaproteobacteria;o__Enterobacterales;f__Enterobacteriaceae;g__Cronobacter;s__Cronobacter_malonaticus

PB2_gene_6257	180	WP_106377164.1	185	107/179(59.78)	1.74e-68	lysozyme [Escherichia coli]	562	d__Bacteria;k__norank;p__Proteobacteria;c__Gammaproteobacteria;o__Enterobacterales;f__Enterobacteriaceae;g__Escherichia;s__Escherichia_coli
PB2_gene_6266	159	BAK10401.1	240	91/118(77.12)	2.64e-57	hypothetical protein PAJ_0321 [Pantoea ananatis AJ13355]	932677	d__Bacteria;k__norank;p__Proteobacteria;c__Gammaproteobacteria;o__Enterobacterales;f__Erwiniaceae;g__Pantoea;s__Pantoea_ananatis
PB2_gene_6296	220	WP_107320560.1	1016	220/220(100.00)	2.15e-146	conjugal transfer protein [Pantoea vagans]	470934	d__Bacteria;k__norank;p__Proteobacteria;c__Gammaproteobacteria;o__Enterobacterales;f__Erwiniaceae;g__Pantoea;s__Pantoea_vagans
PB2_gene_6317	95	PXX94221.1	205	28/78(35.90)	7.96e-09	hypothetical protein DF185_23070, partial	2184082	d__Bacteria;k__norank;p__Bacteroidetes;c__Bacteroidia;o__Marinilabiliales;f__Marinifilaceae;g__Marinifilum;s__Marinifilum_breve
PB2_gene_6323	80	ORM84682.1	203	80/80(100.00)	1.45e-53	autoinducer synthase [Pantoea deleyi]	470932	d__Bacteria;k__norank;p__Proteobacteria;c__Gammaproteobacteria;o__Enterobacterales;f__Erwiniaceae;g__Pantoea;s__Pantoea_deleyi
PB2_gene_6331	131	OUG39088.1	910	125/131(95.42)	3.51e-80	hypothetical protein AZ037_000236 [Klebsiella	1134687	d__Bacteria;k__norank;p__Proteobacteria;c__Gammaproteobacteria;o__Enterobacterales;f__Enterobacteriaceae;g__Klebsiella;s__Klebsiella_michiganensis
PB2_gene_6332	189	RMC71019.1	264	187/189(98.94)	1.51e-131	class I SAM-dependent DNA methyltransferase, partial [Klebsiella michiganensis]	1134687	d__Bacteria;k__norank;p__Proteobacteria;c__Gammaproteobacteria;o__Enterobacterales;f__Enterobacteriaceae;g__Klebsiella;s__Klebsiella_michiganensis
PB2_gene_6334	352	ORM81421.1	520	343/351(97.72)	4.61e-244	cellulose synthase [Pantoea deleyi]	470932	d__Bacteria;k__norank;p__Proteobacteria;c__Gammaproteobacteria;o__Enterobacterales;f__Erwiniaceae;g__Pantoea;s__Pantoea_deleyi
PB2_gene_6337	99	WP_046102566.1	99	95/99(95.96)	9.99e-63	DUF1493 family protein [Pantoea anthophila]	470931	d__Bacteria;k__norank;p__Proteobacteria;c__Gammaproteobacteria;o__Enterobacterales;f__Erwiniaceae;g__Pantoea;s__Pantoea_anthophila
PB2_gene_6341	91	WP_107320568.1	116	91/91(100.00)	5.41e-57	conjugal transfer protein [Pantoea vagans]	470934	d__Bacteria;k__norank;p__Proteobacteria;c__Gammaproteobacteria;o__Enterobacterales;f__Erwiniaceae;g__Pantoea;s__Pantoea_vagans
PB2_gene_6342	230	WP_107320567.1	230	229/230(99.57)	1.84e-160	conjugal transfer protein [Pantoea vagans]	470934	d__Bacteria;k__norank;p__Proteobacteria;c__Gammaproteobacteria;o__Enterobacterales;f__Erwiniaceae;g__Pantoea;s__Pantoea_vagans
PB2_gene_6346	118	ORM75564.1	187	114/117(97.44)	4.08e-72	DNA gyrase subunit B [Pantoea deleyi]	470932	d__Bacteria;k__norank;p__Proteobacteria;c__Gammaproteobacteria;o__Enterobacterales;f__Erwiniaceae;g__Pantoea;s__Pantoea_deleyi
PB2_gene_6354	275	WP_096072313.1	890	263/275(95.64)	1.85e-178	DUF927 domain-containing protein [Pantoea sp.	53335	d__Bacteria;k__norank;p__Proteobacteria;c__Gammaproteobacteria;o__Enterobacterales;f__Erwiniaceae;g__Pantoea;s__norank
PB2_gene_6355	48	WP_096072314.1	125	47/48(97.92)	1.14e-25	hypothetical protein [Pantoea sp. FDAARGOS_194]	53335	d__Bacteria;k__norank;p__Proteobacteria;c__Gammaproteobacteria;o__Enterobacterales;f__Erwiniaceae;g__Pantoea;s__norank
PB2_gene_6356	151	SUB15287.1	412	151/151(100.00)	2.19e-99	D-galactarate dehydratase [Pantoea agglomerans]	549	d__Bacteria;k__norank;p__Proteobacteria;c__Gammaproteobacteria;o__Enterobacterales;f__Erwiniaceae;g__Pantoea;s__Pantoea_agglomerans
PB2_gene_6359	134	WP_077776491.1	99	40/82(48.78)	2.99e-14	hypothetical protein [Pseudescherichia vulneris]	None	Unclassified
PB2_gene_6373	123	EEB55687.1	132	113/116(97.41)	2.08e-75	SdbB protein (putative substrate of the Dot/Icm system) [Wolbachia endosymbiont of Culex quinquefasciatus JHB]	569881	d__Bacteria;k__norank;p__Proteobacteria;c__Alphaproteobacteria;o__Rickettsiales;f__Anaplasmataceae;g__Wolbachia;s__Wolbachia_endosymbiont_of_Culex_quinquefasciatus
PB2_gene_6376	278	WP_114517845.1	495	257/272(94.49)	1.10e-183	recombinase family protein [Wolbachia endosymbiont of Cylisticus convexus]	118728	d__Bacteria;k__norank;p__Proteobacteria;c__Alphaproteobacteria;o__Rickettsiales;f__Anaplasmataceae;g__Wolbachia;s__Wolbachia_endosymbiont_of_Cylisticus_convexus
PB2_gene_6386	345	ORM80955.1	426	342/345(99.13)	6.73e-247	phosphoribosylamine--glycine ligase [Pantoea deleyi]	470932	d__Bacteria;k__norank;p__Proteobacteria;c__Gammaproteobacteria;o__Enterobacterales;f__Erwiniaceae;g__Pantoea;s__Pantoea_deleyi
PB2_gene_6387	272	PCG11619.1	375	244/272(89.71)	9.39e-160	hypothetical protein CO539_02230 [Wolbachia	None	Unclassified
PB2_gene_6392	293	WP_064124695.1	387	242/264(91.67)	1.23e-175	phage tail protein [Wolbachia endosymbiont of Dactylopius coccus]	None	Unclassified
PB2_gene_6400	171	ETW96096.1	240	47/150(31.33)	1.63e-11	hypothetical protein ETSY2_47035 [Candidatus	1429439	d__Bacteria;k__norank;p__Candidatus_Tectomicrobia;c__norank;o__norank;f__norank;g__Candidatus_Entotheonella;s__Candidatus_Entotheonella_gemina

PB2_gene_6410	65	EFM17734.1	146	51/61(83.61)	1.19e-24	hypothetical protein PanABDRAFT_4245 [Pantoea sp. aB]	517433	d__Bacteria;k__norank;p__Proteobacteria;c__Gammaproteobacteria;o__Enterobacterales;f__Erwiniaceae;g__Pantoea;s__Pantoea_sp._aB
PB2_gene_6420	88	WP_111140045.1	333	88/88(100.00)	4.35e-57	ABC transporter ATP-binding protein [Pantoea sp. ARC607]	2027922	d__Bacteria;k__norank;p__Proteobacteria;c__Gammaproteobacteria;o__Enterobacterales;f__Erwiniaceae;g__Pantoea;s__Pantoea_sp._ARC607
PB2_gene_6423	152	EWG64830.1	408	151/152(99.34)	9.38e-103	hypothetical protein P348_04632 [Enterobacter sp. DC3]	1395579	d__Bacteria;k__norank;p__Proteobacteria;c__Gammaproteobacteria;o__Enterobacterales;f__Enterobacteriaceae;g__Enterobacter;s__Enterobacter_sp._DC3
PB2_gene_6424	184	EWG64829.1	427	178/184(96.74)	1.35e-108	VRR-NUC domain protein [Enterobacter sp. DC3]	1395579	d__Bacteria;k__norank;p__Proteobacteria;c__Gammaproteobacteria;o__Enterobacterales;f__Enterobacteriaceae;g__Enterobacter;s__Enterobacter_sp._DC3
PB2_gene_6428	118	OZJ06877.1	2877	42/100(42.00)	2.96e-13	hypothetical protein BZG36_00029 [Bifiguratus	1938954	d__Eukaryota;k__Fungi;p__Mucoromycota;c__Endogonomycetes;o__Endogonales;f__norank;g__Bifiguratus;s__Bifiguratus_adelaidae
PB2_gene_6437	209	PCG11406.1	211	171/212(80.66)	5.21e-116	hypothetical protein CO539_03425 [Wolbachia	None	Unclassified
PB2_gene_6444	337	WP_095092412.1	751	115/328(35.06)	4.79e-36	autotransporter outer membrane beta-barrel domain-containing protein [Pseudomonas sp. Irchel 3A5]	2008911	d__Bacteria;k__norank;p__Proteobacteria;c__Gammaproteobacteria;o__Pseudomonadales;f__Pseudomonadaceae;g__Pseudomonas;s__Pseudomonas_sp._Irchel_3A5
PB2_gene_6448	128	SRN30406.1	57	23/48(47.92)	2.24e-06	Uncharacterised protein [Shigella flexneri]	623	d__Bacteria;k__norank;p__Proteobacteria;c__Gammaproteobacteria;o__Enterobacterales;f__Enterobacteriaceae;g__Shigella;s__Shigella_flexneri
PB2_gene_6460	134	WP_129335249.1	124	28/70(40.00)	1.84e-08	hypothetical protein [Enterobacter cloacae complex sp. 2DZ2F20B]	2511993	d__Bacteria;k__norank;p__Proteobacteria;c__Gammaproteobacteria;o__Enterobacterales;f__Enterobacteriaceae;g__Enterobacter;s__Enterobacter_cloacae_complex_sp._2DZ2F20B
PB2_gene_6467	163	WP_099458612.1	284	126/134(94.03)	7.09e-83	ankyrin repeat domain-containing protein [Wolbachia pipientis]	955	d__Bacteria;k__norank;p__Proteobacteria;c__Alphaproteobacteria;o__Rickettsiales;f__Anaplasmataceae;g__Wolbachia;s__Wolbachia_pipientis
PB2_gene_6470	133	WP_084690452.1	448	131/133(98.50)	6.78e-85	filamentous hemagglutinin [Pantoea anthophila]	470931	d__Bacteria;k__norank;p__Proteobacteria;c__Gammaproteobacteria;o__Enterobacterales;f__Erwiniaceae;g__Pantoea;s__Pantoea_anthophila
PB2_gene_6471	114	WP_084690451.1	114	101/114(88.60)	1.22e-70	zinc ABC transporter substrate-binding protein [Pantoea	None	Unclassified
PB2_gene_6478	254	ADO08422.1	257	250/254(98.43)	1.82e-175	30S ribosomal protein S2 [Pantoea vagans C9-1]	712898	d__Bacteria;k__norank;p__Proteobacteria;c__Gammaproteobacteria;o__Enterobacterales;f__Erwiniaceae;g__Pantoea;s__Pantoea_vagans
PB2_gene_6483	112	ADO08173.1	112	100/112(89.29)	8.00e-75	putative cytoplasmic protein (plasmid) [Pantoea vagans C9-1]	712898	d__Bacteria;k__norank;p__Proteobacteria;c__Gammaproteobacteria;o__Enterobacterales;f__Erwiniaceae;g__Pantoea;s__Pantoea_vagans
PB2_gene_6491	217	ORM85835.1	193	184/193(95.34)	1.61e-133	D-alanyl-D-alanine dipeptidase [Pantoea deleyi]	470932	d__Bacteria;k__norank;p__Proteobacteria;c__Gammaproteobacteria;o__Enterobacterales;f__Erwiniaceae;g__Pantoea;s__Pantoea_deleyi
PB2_gene_6494	77	PLN97445.1	115	46/77(59.74)	7.16e-26	phage portal protein, partial [Klebsiella pneumoniae]	573	d__Bacteria;k__norank;p__Proteobacteria;c__Gammaproteobacteria;o__Enterobacterales;f__Enterobacteriaceae;g__Klebsiella;s__Klebsiella_pneumoniae
PB2_gene_6499	141	WP_085016767.1	141	141/141(100.00)	8.44e-93	MULTISPECIES: universal stress protein [Enterobacterales]	91347	d__Bacteria;k__norank;p__Proteobacteria;c__Gammaproteobacteria;o__Enterobacterales;f__norank;g__norank;s__norank
PB2_gene_6501	197	ORM86552.1	197	191/197(96.95)	8.95e-138	5-formyltetrahydrofolate cyclo-ligase [Pantoea deleyi]	470932	d__Bacteria;k__norank;p__Proteobacteria;c__Gammaproteobacteria;o__Enterobacterales;f__Erwiniaceae;g__Pantoea;s__Pantoea_deleyi
PB2_gene_6504	121	AYP23583.1	72	45/49(91.84)	6.43e-18	hypothetical protein D0A61_11805 [Pantoea	549	d__Bacteria;k__norank;p__Proteobacteria;c__Gammaproteobacteria;o__Enterobacterales;f__Erwiniaceae;g__Pantoea;s__Pantoea_agglomerans
PB2_gene_6505	160	ORM77609.1	160	160/160(100.00)	7.18e-110	hypothetical protein HA42_17550 [Pantoea deleyi]	470932	d__Bacteria;k__norank;p__Proteobacteria;c__Gammaproteobacteria;o__Enterobacterales;f__Erwiniaceae;g__Pantoea;s__Pantoea_deleyi
PB2_gene_6517	156	WP_126690654.1	155	65/156(41.67)	4.85e-28	hypothetical protein [Pantoea sp. YU22]	None	Unclassified
PB2_gene_6528	283	ORM82655.1	275	270/275(98.18)	4.83e-201	5-deoxy-glucuronate isomerase [Pantoea deleyi]	470932	d__Bacteria;k__norank;p__Proteobacteria;c__Gammaproteobacteria;o__Enterobacterales;f__Erwiniaceae;g__Pantoea;s__Pantoea_deleyi
PB2_gene_6529	254	WP_064085834.1	355	252/254(99.21)	1.42e-170	rod shape-determining protein [Wolbachia endosymbiont of Dactylopius coccus]	None	Unclassified

PB2_gene_6535	205	ORM80448.1	355	203/205(99.02)	4.14e-131	sugar ABC transporter permease [Pantoea deleyi]	470932	d__Bacteria;k__norank;p__Proteobacteria;c__Gammaproteobacteria;o__Enterobacterales;f__Erwiniaceae;g__Pantoea;s__Pantoea_deleyi
PB2_gene_6550	182	KKW50957.1	180	158/180(87.78)	1.51e-119	hypothetical protein XB02_09020 [Pantoea ananatis]	553	d__Bacteria;k__norank;p__Proteobacteria;c__Gammaproteobacteria;o__Enterobacterales;f__Erwiniaceae;g__Pantoea;s__Pantoea_ananatis
PB2_gene_6565	104	ORM83371.1	87	87/87(100.00)	1.93e-50	hypothetical protein HA42_06420 [Pantoea deleyi]	470932	d__Bacteria;k__norank;p__Proteobacteria;c__Gammaproteobacteria;o__Enterobacterales;f__Erwiniaceae;g__Pantoea;s__Pantoea_deleyi
PB2_gene_6570	228	AIE08475.1	228	215/228(94.30)	1.50e-149	putative inner membrane protein [Salmonella enterica subsp. enterica serovar Typhimurium]	90371	d__Bacteria;k__norank;p__Proteobacteria;c__Gammaproteobacteria;o__Enterobacterales;f__Enterobacteriaceae;g__Salmonella;s__Salmonella_enterica
PB2_gene_6573	318	WP_045140817.1	544	317/318(99.69)	3.72e-235	DNA phosphorothioation system sulfurtransferase DndC [Pantoea agglomerans]	549	d__Bacteria;k__norank;p__Proteobacteria;c__Gammaproteobacteria;o__Enterobacterales;f__Erwiniaceae;g__Pantoea;s__Pantoea_agglomerans
PB2_gene_6575	78	SSK75623.1	208	45/78(57.69)	1.05e-19	Uncharacterised protein [Klebsiella pneumoniae]	573	d__Bacteria;k__norank;p__Proteobacteria;c__Gammaproteobacteria;o__Enterobacterales;f__Enterobacteriaceae;g__Klebsiella;s__Klebsiella_pneumoniae
PB2_gene_6587	132	AYP23558.1	120	101/119(84.87)	2.54e-69	hypothetical protein D0A61_11670 [Pantoea	549	d__Bacteria;k__norank;p__Proteobacteria;c__Gammaproteobacteria;o__Enterobacterales;f__Erwiniaceae;g__Pantoea;s__Pantoea_agglomerans
PB2_gene_6588	91	WP_094122281.1	319	59/84(70.24)	5.62e-26	AraC family transcriptional regulator [Pantoea conspicua]	472705	d__Bacteria;k__norank;p__Proteobacteria;c__Gammaproteobacteria;o__Enterobacterales;f__Erwiniaceae;g__Pantoea;s__Pantoea_conspicua
PB2_gene_6594	144	WP_128086394.1	144	135/144(93.75)	1.75e-91	PACE efflux transporter [Pantoea deleyi]	470932	d__Bacteria;k__norank;p__Proteobacteria;c__Gammaproteobacteria;o__Enterobacterales;f__Erwiniaceae;g__Pantoea;s__Pantoea_deleyi
PB2_gene_6604	67	WP_094119387.1	97	64/66(96.97)	2.89e-35	helix-turn-helix domain-containing protein [Pantoea	472705	d__Bacteria;k__norank;p__Proteobacteria;c__Gammaproteobacteria;o__Enterobacterales;f__Erwiniaceae;g__Pantoea;s__Pantoea_conspicua
PB2_gene_6606	120	WP_050491385.1	180	117/118(99.15)	5.37e-83	hypothetical protein [Pantoea agglomerans]	549	d__Bacteria;k__norank;p__Proteobacteria;c__Gammaproteobacteria;o__Enterobacterales;f__Erwiniaceae;g__Pantoea;s__Pantoea_agglomerans
PB2_gene_6607	309	BBA85312.1	354	208/301(69.10)	3.45e-148	chorismate synthase [endosymbiont of Sipalinus gigas]	1972134	d__Bacteria;k__norank;p__Proteobacteria;c__Gammaproteobacteria;o__norank;f__norank;g__Candidatus_Nardonella;s__endosymbiont_of_Sipalinus_gigas
PB2_gene_6614	279	ERM10511.1	290	279/279(100.00)	1.57e-204	hypothetical protein L584_11990 [Pantoea agglomerans Tx10]	1332072	d__Bacteria;k__norank;p__Proteobacteria;c__Gammaproteobacteria;o__Enterobacterales;f__Erwiniaceae;g__Pantoea;s__Pantoea_agglomerans
PB2_gene_6616	62	EAL59603.1	156	34/38(89.47)	5.55e-13	hypothetical protein WwSim0512 [Wolbachia endosymbiont of Drosophila simulans]	77038	d__Bacteria;k__norank;p__Proteobacteria;c__Alphaproteobacteria;o__Rickettsiales;f__Anaplasmataceae;g__Wolbachia;s__Wolbachia_endosymbiont_of_Drosophila_simulans
PB2_gene_6623	125	WP_107320594.1	179	124/125(99.20)	1.06e-81	DUF905 domain-containing protein [Pantoea vagans]	470934	d__Bacteria;k__norank;p__Proteobacteria;c__Gammaproteobacteria;o__Enterobacterales;f__Erwiniaceae;g__Pantoea;s__Pantoea_vagans
PB2_gene_6639	46	WP_109650270.1	73	24/30(80.00)	5.86e-09	hypothetical protein [Pantoea allii]	549	d__Bacteria;k__norank;p__Proteobacteria;c__Gammaproteobacteria;o__Enterobacterales;f__Erwiniaceae;g__Pantoea;s__Pantoea_agglomerans
PB2_gene_6645	303	WP_111142026.1	788	289/303(95.38)	1.37e-195	SrfC [Pantoea sp. ARC607]	2027922	d__Bacteria;k__norank;p__Proteobacteria;c__Gammaproteobacteria;o__Enterobacterales;f__Erwiniaceae;g__Pantoea;s__Pantoea_sp._ARC607
PB2_gene_6650	225	ORX95332.1	201	50/203(24.63)	7.06e-18	TATA-binding related factor [Basidiobolus meristosporus CBS 931.73]	1314790	d__Eukaryota;k__Fungi;p__Zoopagomycota;c__Basidiobolomycetes;o__Basidiobolales;f__Basidiobolaceae;g__Basidiobolus;s__Basidiobolus_meristosporus
PB2_gene_6661	125	ORX94506.1	629	30/72(41.67)	3.36e-08	WD40 repeat-like protein [Basidiobolus meristosporus CBS 931.73]	1314790	d__Eukaryota;k__Fungi;p__Zoopagomycota;c__Basidiobolomycetes;o__Basidiobolales;f__Basidiobolaceae;g__Basidiobolus;s__Basidiobolus_meristosporus
PB2_gene_6676	301	WP_024082726.1	544	274/301(91.03)	6.34e-167	PilN family type IVB pilus formation outer membrane protein [Erwinia amylovora]	552	d__Bacteria;k__norank;p__Proteobacteria;c__Gammaproteobacteria;o__Enterobacterales;f__Erwiniaceae;g__Erwinia;s__Erwinia_amylovora
PB2_gene_6688	255	ORM83132.1	591	249/255(97.65)	1.77e-164	multidrug ABC transporter permease/ATP-binding protein [Pantoea deleyi]	470932	d__Bacteria;k__norank;p__Proteobacteria;c__Gammaproteobacteria;o__Enterobacterales;f__Erwiniaceae;g__Pantoea;s__Pantoea_deleyi
PB2_gene_6690	255	WP_111140379.1	338	232/255(90.98)	6.31e-168	membrane dipeptidase [Pantoea sp. ARC607]	2027922	d__Bacteria;k__norank;p__Proteobacteria;c__Gammaproteobacteria;o__Enterobacterales;f__Erwiniaceae;g__Pantoea;s__Pantoea_sp._ARC607

PB2_gene_6694	234	ORM80456.1	232	230/232(99.14)	3.36e-178	type VI secretion-associated protein [Pantoea deleyi]	470932	d__Bacteria;k__norank;p__Proteobacteria;c__Gammaproteobacteria;o__Enterobacterales;f__Erwiniaceae;g__Pantoea;s__Pantoea_deleyi
PB2_gene_6700	137	EFM18129.1	137	133/137(97.08)	1.47e-82	histone family protein nucleoid-structuring protein H-NS [Pantoea sp. aB]	517433	d__Bacteria;k__norank;p__Proteobacteria;c__Gammaproteobacteria;o__Enterobacterales;f__Erwiniaceae;g__Pantoea;s__Pantoea_sp._aB
PB2_gene_6702	255	PBQ28066.1	264	228/255(89.41)	3.00e-160	hypothetical protein BTO27_02910, partial	2049495	d__Bacteria;k__norank;p__Proteobacteria;c__Alphaproteobacteria;o__Rickettsiales;f__Anaplasmataceae;g__Wolbachia;s__Wolbachia_pipientis
PB2_gene_6709	156	ORM52195.1	156	156/156(100.00)	1.58e-102	arginine repressor [Pantoea conspicua]	472705	d__Bacteria;k__norank;p__Proteobacteria;c__Gammaproteobacteria;o__Enterobacterales;f__Erwiniaceae;g__Pantoea;s__Pantoea_conspicua
PB2_gene_6711	223	ORM80773.1	447	221/223(99.10)	7.39e-158	recombination factor protein RarA [Pantoea deleyi]	470932	d__Bacteria;k__norank;p__Proteobacteria;c__Gammaproteobacteria;o__Enterobacterales;f__Erwiniaceae;g__Pantoea;s__Pantoea_deleyi
PB2_gene_6714	84	WP_071966126.1	117	82/84(97.62)	3.85e-53	hypothetical protein [Enterobacter kobei]	354276	d__Bacteria;k__norank;p__Proteobacteria;c__Gammaproteobacteria;o__Enterobacterales;f__Enterobacteriaceae;g__Enterobacter;s__norank
PB2_gene_6715	295	WP_108784026.1	384	67/128(52.34)	8.50e-32	hypothetical protein [Wolbachia endosymbiont of Bemisia tabaci]	215173	d__Bacteria;k__norank;p__Proteobacteria;c__Alphaproteobacteria;o__Rickettsiales;f__Anaplasmataceae;g__Wolbachia;s__Wolbachia_endosymbiont_of_Bemisia_tabaci
PB2_gene_6719	199	ORM84694.1	199	199/199(100.00)	3.24e-141	NAD(P)H:quinone oxidoreductase, type IV [Pantoea	470932	d__Bacteria;k__norank;p__Proteobacteria;c__Gammaproteobacteria;o__Enterobacterales;f__Erwiniaceae;g__Pantoea;s__Pantoea_deleyi
PB2_gene_6720	115	ORX61646.1	252	34/97(35.05)	9.54e-09	SH3-domain-containing protein [Hesseltinella vesiculosa]	101127	d__Eukaryota;k__Fungi;p__Mucoromycota;c__Mucoromycetes;o__Mucorales;f__Cunninghamellaceae;g__Hesseltinella;s__Hesseltinella_vesiculosa
PB2_gene_6728	137	ORM81422.1	136	126/136(92.65)	4.07e-82	DNA-binding protein [Pantoea deleyi]	470932	d__Bacteria;k__norank;p__Proteobacteria;c__Gammaproteobacteria;o__Enterobacterales;f__Erwiniaceae;g__Pantoea;s__Pantoea_deleyi
PB2_gene_6730	241	WP_039390117.1	241	233/241(96.68)	1.05e-160	hypothetical protein [Pantoea agglomerans]	549	d__Bacteria;k__norank;p__Proteobacteria;c__Gammaproteobacteria;o__Enterobacterales;f__Erwiniaceae;g__Pantoea;s__Pantoea_agglomerans
PB2_gene_6732	151	STI57768.1	190	111/151(73.51)	1.61e-62	putative phage tail tape measure protein [Escherichia coli]	562	d__Bacteria;k__norank;p__Proteobacteria;c__Gammaproteobacteria;o__Enterobacterales;f__Enterobacteriaceae;g__Escherichia;s__Escherichia_coli
PB2_gene_6733	98	WP_101762973.1	100	92/98(93.88)	7.84e-51	phage tail assembly protein [Pantoea sp. 596]	92488	d__Bacteria;k__norank;p__Proteobacteria;c__Gammaproteobacteria;o__Enterobacterales;f__Erwiniaceae;g__Pantoea;s__Pantoea_endophytica
PB2_gene_6743	153	WP_025119481.1	1244	88/153(57.52)	2.61e-59	MULTISPECIES: methyltransferase domain-	2647522	d__Bacteria;k__norank;p__Proteobacteria;c__Gammaproteobacteria;o__Enterobacterales;f__Yersiniaceae;g__Serratia;s__norank
PB2_gene_6744	286	WP_123253064.1	345	140/286(48.95)	1.33e-90	Abi family protein [Dickeya sp. FVG1-MFV-O17]	1577887	d__Bacteria;k__norank;p__Proteobacteria;c__Gammaproteobacteria;o__Enterobacterales;f__Pectobacteriaceae;g__Dickeya;s__Dickeya_undicola
PB2_gene_6746	97	RUM31266.1	596	36/72(50.00)	1.82e-16	hypothetical protein DSY42_03095 [Aquifex sp.]	313370	d__Bacteria;k__norank;p__Aquificae;c__Aquificae;o__Aquificales;f__Aquificaceae;g__Aquifex;s__Aquifex_sp.
PB2_gene_6750	96	WP_052259210.1	125	90/96(93.75)	1.27e-65	hypothetical protein [Pantoea agglomerans]	549	d__Bacteria;k__norank;p__Proteobacteria;c__Gammaproteobacteria;o__Enterobacterales;f__Erwiniaceae;g__Pantoea;s__Pantoea_agglomerans
PB2_gene_6753	237	ORM84688.1	237	236/237(99.58)	1.16e-171	pyrimidine utilization protein B [Pantoea deleyi]	470932	d__Bacteria;k__norank;p__Proteobacteria;c__Gammaproteobacteria;o__Enterobacterales;f__Erwiniaceae;g__Pantoea;s__Pantoea_deleyi
PB2_gene_6762	235	STR54941.1	249	188/235(80.00)	1.84e-143	Uncharacterised protein [Klebsiella oxytoca]	571	d__Bacteria;k__norank;p__Proteobacteria;c__Gammaproteobacteria;o__Enterobacterales;f__Enterobacteriaceae;g__Klebsiella;s__Klebsiella_oxytoca
PB2_gene_6769	119	ORM78615.1	119	118/119(99.16)	2.40e-86	6-carboxytetrahydropterin synthase QueD [Pantoea deleyi]	470932	d__Bacteria;k__norank;p__Proteobacteria;c__Gammaproteobacteria;o__Enterobacterales;f__Erwiniaceae;g__Pantoea;s__Pantoea_deleyi
PB2_gene_6779	112	WP_115759230.1	112	81/110(73.64)	5.16e-53	hypothetical protein [Pantoea agglomerans]	549	d__Bacteria;k__norank;p__Proteobacteria;c__Gammaproteobacteria;o__Enterobacterales;f__Erwiniaceae;g__Pantoea;s__Pantoea_agglomerans
PB2_gene_6787	104	RKP06577.1	276	35/95(36.84)	9.86e-07	hypothetical protein THASP1DRAFT_31605 [Thamnocephalis sphaerospora]	78915	d__Eukaryota;k__Fungi;p__Zoopagomycota;c__Zoopagomycetes;o__Zoopagales;f__Sigmoideomycetaceae;g__Thamnocephalis;s__Thamnocephalis_sphaerospora
PB2_gene_6817	253	WP_107320559.1	407	252/253(99.60)	1.92e-166	conjugal transfer protein [Pantoea vagans]	470934	d__Bacteria;k__norank;p__Proteobacteria;c__Gammaproteobacteria;o__Enterobacterales;f__Erwiniaceae;g__Pantoea;s__Pantoea_vagans

PB2_gene_6831	237	WP_085016765.1	235	234/235(99.57)	1.26e-170	MULTISPECIES: 2,3-bisphosphoglycerate-dependent phosphoglycerate mutase [Enterobacterales]	91347	d__Bacteria;k__norank;p__Proteobacteria;c__Gammaproteobacteria;o__Enterobacterales;f__norank;g__norank;s__norank
PB2_gene_6838	281	TBU20600.1	612	55/194(28.35)	1.96e-10	transposase IS4 [Hamiltosporidium	1176355	d__Eukaryota;k__Fungi;p__Microsporidia;c__norank;o__norank;f__Dubosqiidae;g__Hamiltosporidium;s__Hamiltosporidium_tvaerminnensis
PB2_gene_6839	84	WP_052259255.1	146	79/84(94.05)	1.45e-47	type-F conjugative transfer system pilin assembly protein TrbC [Pantoea agglomerans]	549	d__Bacteria;k__norank;p__Proteobacteria;c__Gammaproteobacteria;o__Enterobacterales;f__Erwiniaceae;g__Pantoea;s__Pantoea_agglomerans
PB2_gene_6843	123	WP_107320552.1	711	122/123(99.19)	3.97e-69	conjugal transfer protein TraY [Pantoea vagans]	470934	d__Bacteria;k__norank;p__Proteobacteria;c__Gammaproteobacteria;o__Enterobacterales;f__Erwiniaceae;g__Pantoea;s__Pantoea_vagans
PB2_gene_6850	107	WP_033761754.1	107	92/107(85.98)	9.21e-56	phage tail assembly protein [Pantoea agglomerans]	None	Unclassified
PB2_gene_6856	208	AIX51577.1	293	111/208(53.37)	1.68e-78	hypothetical protein PSNIH1_15775 [Pantoea sp.	1484158	d__Bacteria;k__norank;p__Proteobacteria;c__Gammaproteobacteria;o__Enterobacterales;f__Erwiniaceae;g__Pantoea;s__Pantoea_sp._PSNIH1
PB2_gene_6859	183	ORM79310.1	265	180/183(98.36)	2.75e-128	endopeptidase [Pantoea deleyi]	470932	d__Bacteria;k__norank;p__Proteobacteria;c__Gammaproteobacteria;o__Enterobacterales;f__Erwiniaceae;g__Pantoea;s__Pantoea_deleyi
PB2_gene_6863	235	WP_111138318.1	338	233/235(99.15)	3.12e-165	NAD(P)-dependent alcohol dehydrogenase [Pantoea sp. ARC607]	2027922	d__Bacteria;k__norank;p__Proteobacteria;c__Gammaproteobacteria;o__Enterobacterales;f__Erwiniaceae;g__Pantoea;s__Pantoea_sp._ARC607
PB2_gene_6872	175	WP_128088245.1	175	172/175(98.29)	7.54e-117	hypothetical protein [Pantoea brenneri]	472694	d__Bacteria;k__norank;p__Proteobacteria;c__Gammaproteobacteria;o__Enterobacterales;f__Erwiniaceae;g__Pantoea;s__Pantoea_brenneri
PB2_gene_6874	57	GAM65518.1	377	40/54(74.07)	9.37e-21	hypothetical protein JCM19232_5018 [Vibrio	1481914	d__Bacteria;k__norank;p__Proteobacteria;c__Gammaproteobacteria;o__Vibrionales;f__Vibrionaceae;g__Vibrio;s__Vibrio_ishigakensis
PB2_gene_6878	83	WP_128085513.1	139	35/51(68.63)	2.47e-12	DUF1090 family protein [Pantoea deleyi]	470932	d__Bacteria;k__norank;p__Proteobacteria;c__Gammaproteobacteria;o__Enterobacterales;f__Erwiniaceae;g__Pantoea;s__Pantoea_deleyi
PB2_gene_6883	176	WP_052674376.1	305	164/175(93.71)	3.07e-123	hypothetical protein [Enterobacter roggenkampii]	1812935	d__Bacteria;k__norank;p__Proteobacteria;c__Gammaproteobacteria;o__Enterobacterales;f__Enterobacteriaceae;g__Enterobacter;s__Enterobacter_roggenkampii
PB2_gene_6902	229	WP_111140056.1	223	219/223(98.21)	1.26e-156	outer membrane protein OmpW [Pantoea sp. ARC607]	2027922	d__Bacteria;k__norank;p__Proteobacteria;c__Gammaproteobacteria;o__Enterobacterales;f__Erwiniaceae;g__Pantoea;s__Pantoea_sp._ARC607
PB2_gene_6904	127	WP_034915517.1	117	117/117(100.00)	6.33e-77	MULTISPECIES: metalloregulator ArsR/SmtB family transcription factor	1903409	d__Bacteria;k__norank;p__Proteobacteria;c__Gammaproteobacteria;o__Enterobacterales;f__Erwiniaceae;g__norank;s__norank
PB2_gene_6907	108	WP_083070435.1	107	88/107(82.24)	6.75e-56	hypothetical protein [Pantoea vagans]	470934	d__Bacteria;k__norank;p__Proteobacteria;c__Gammaproteobacteria;o__Enterobacterales;f__Erwiniaceae;g__Pantoea;s__Pantoea_vagans
PB2_gene_6925	133	BBA85164.1	533	65/133(48.87)	3.84e-27	30S ribosomal protein S1 [endosymbiont of Sipalinus gigas]	1972134	d__Bacteria;k__norank;p__Proteobacteria;c__Gammaproteobacteria;o__norank;f__norank;g__Candidatus_Nardonella;s__endosymbiont_of_Sipalinus_gigas
PB2_gene_6928	70	WP_080107294.1	60	34/41(82.93)	7.87e-16	hypothetical protein [Salmonella enterica]	None	Unclassified
PB2_gene_6945	195	WP_122983673.1	563	166/195(85.13)	1.08e-101	hypothetical protein [Pantoea brenneri]	472694	d__Bacteria;k__norank;p__Proteobacteria;c__Gammaproteobacteria;o__Enterobacterales;f__Erwiniaceae;g__Pantoea;s__Pantoea_brenneri
PB2_gene_6950	186	YP_002321389.1	420	69/150(46.00)	1.06e-34	semaphorin-like protein [Oryctes rhinoceros nudivirus]	92521	d__Viruses;k__norank;p__norank;c__norank;o__norank;f__Nudiviridae;g__Alphanudivirus;s__Oryctes_rhinoceros_nudivirus
PB2_gene_6955	266	WP_022625116.1	595	254/266(95.49)	3.50e-183	hypothetical protein [Pantoea agglomerans]	549	d__Bacteria;k__norank;p__Proteobacteria;c__Gammaproteobacteria;o__Enterobacterales;f__Erwiniaceae;g__Pantoea;s__Pantoea_agglomerans
PB2_gene_6961	69	WP_072059564.1	455	61/66(92.42)	4.49e-32	ATP-binding cassette domain-containing protein [Enterobacter kobei]	208224	d__Bacteria;k__norank;p__Proteobacteria;c__Gammaproteobacteria;o__Enterobacterales;f__Enterobacteriaceae;g__Enterobacter;s__Enterobacter_kobei
PB2_gene_6967	237	RZK26366.1	325	40/119(33.61)	3.80e-12	hypothetical protein EOO43_03045, partial	239	d__Bacteria;k__norank;p__Bacteroidetes;c__Flavobacteriia;o__Flavobacteriales;f__Flavobacteriaceae;g__Flavobacterium;s__Flavobacterium_sp.

PB2_gene_7001	64	WP_039390112.1	183	62/64(96.88)	6.23e-39	hypothetical protein [Pantoea agglomerans]	549	d__Bacteria;k__norank;p__Proteobacteria;c__Gammaproteobacteria;o__Enterobacterales;f__Erwiniaceae;g__Pantoea;s__Pantoea_agglomerans
PB2_gene_7011	247	RYE27181.1	1800	111/243(45.68)	4.87e-62	DUF1759 domain-containing protein [Sphingobacteriaceae	2021370	d__Bacteria;k__norank;p__Bacteroidetes;c__Sphingobacteriia;o__Sphingobacteriales;f__Sphingobacteriaceae;g__norank;s__Sphingobacteriaceae_bacterium
PB2_gene_7017	110	WP_128144798.1	141	93/109(85.32)	3.27e-66	nucleoside triphosphatase NudI [Serratia rubidaea]	61652	d__Bacteria;k__norank;p__Proteobacteria;c__Gammaproteobacteria;o__Enterobacterales;f__Yersiniaceae;g__Serratia;s__Serratia_rubidaea
PB2_gene_7018	75	WP_113877706.1	288	59/66(89.39)	1.10e-34	radical SAM protein [Rahnella aquatilis]	34038	d__Bacteria;k__norank;p__Proteobacteria;c__Gammaproteobacteria;o__Enterobacterales;f__Yersiniaceae;g__Rahnella;s__Rahnella_aquatilis
PB2_gene_7019	113	WP_125289903.1	106	75/106(70.75)	9.62e-48	type II toxin-antitoxin system RelE/ParE family toxin [Erwinia sp. 198]	2022746	d__Bacteria;k__norank;p__Proteobacteria;c__Gammaproteobacteria;o__Enterobacterales;f__Erwiniaceae;g__Erwinia;s__Erwinia_sp._198
PB2_gene_7020	80	WP_034888359.1	92	66/80(82.50)	1.62e-39	helix-turn-helix domain-containing protein [Erwinia	371042	d__Bacteria;k__norank;p__Proteobacteria;c__Gammaproteobacteria;o__Enterobacterales;f__Erwiniaceae;g__Erwinia;s__Erwinia_typographi
PB2_gene_7029	222	ELP25233.1	201	184/197(93.40)	2.68e-126	DJ-1, YajL, PfpI superfamily protein [Pantoea agglomerans	1261128	d__Bacteria;k__norank;p__Proteobacteria;c__Gammaproteobacteria;o__Enterobacterales;f__Erwiniaceae;g__Pantoea;s__Pantoea_agglomerans
PB2_gene_7035	79	WP_107320587.1	68	67/68(98.53)	2.23e-39	TraY domain-containing protein [Pantoea vagans]	470934	d__Bacteria;k__norank;p__Proteobacteria;c__Gammaproteobacteria;o__Enterobacterales;f__Erwiniaceae;g__Pantoea;s__Pantoea_vagans
PB2_gene_7037	172	KNA26163.1	274	171/172(99.42)	2.06e-129	transposase (plasmid) [Pantoea ananatis]	553	d__Bacteria;k__norank;p__Proteobacteria;c__Gammaproteobacteria;o__Enterobacterales;f__Erwiniaceae;g__Pantoea;s__Pantoea_ananatis
PB2_gene_7039	128	WP_126690660.1	216	73/128(57.03)	2.16e-42	ParA family protein [Pantoea sp. YU22]	None	Unclassified
PB2_gene_7044	216	ORM86050.1	343	198/216(91.67)	4.28e-131	hypothetical protein HA42_01655 [Pantoea deleyi]	470932	d__Bacteria;k__norank;p__Proteobacteria;c__Gammaproteobacteria;o__Enterobacterales;f__Erwiniaceae;g__Pantoea;s__Pantoea_deleyi
PB2_gene_7045	169	PME06503.1	461	66/172(38.37)	1.11e-26	hypothetical protein A8A06_07420 [Escherichia coli]	562	d__Bacteria;k__norank;p__Proteobacteria;c__Gammaproteobacteria;o__Enterobacterales;f__Enterobacteriaceae;g__Escherichia;s__Escherichia_coli
PB2_gene_7056	142	ADO10851.1	142	132/141(93.62)	3.68e-83	Protein lysB [Pantoea vagans C9-1]	712898	d__Bacteria;k__norank;p__Proteobacteria;c__Gammaproteobacteria;o__Enterobacterales;f__Erwiniaceae;g__Pantoea;s__Pantoea_vagans
PB2_gene_7063	225	WP_062748911.1	208	144/208(69.23)	6.65e-104	DNA methyltransferase [Erwinia persicina]	55211	d__Bacteria;k__norank;p__Proteobacteria;c__Gammaproteobacteria;o__Enterobacterales;f__Erwiniaceae;g__Erwinia;s__Erwinia_persicina
PB2_gene_7064	84	WP_107320523.1	84	80/84(95.24)	3.56e-47	hypothetical protein [Pantoea vagans]	470934	d__Bacteria;k__norank;p__Proteobacteria;c__Gammaproteobacteria;o__Enterobacterales;f__Erwiniaceae;g__Pantoea;s__Pantoea_vagans
PB2_gene_7065	152	WP_058973330.1	169	149/152(98.03)	1.79e-103	lysozyme [Type-D symbiont of Plautia stali]	1560356	d__Bacteria;k__norank;p__Proteobacteria;c__Gammaproteobacteria;o__Enterobacterales;f__Enterobacteriaceae;g__norank;s__Type-D_symbiont_of_Plautia_stali
PB2_gene_7066	70	WP_058973331.1	70	68/70(97.14)	2.39e-41	hypothetical protein [Type-D symbiont of Plautia stali]	1560356	d__Bacteria;k__norank;p__Proteobacteria;c__Gammaproteobacteria;o__Enterobacterales;f__Enterobacteriaceae;g__norank;s__Type-D_symbiont_of_Plautia_stali
PB2_gene_7074	163	BBA85183.1	806	109/162(67.28)	1.64e-62	protein translocase subunit SecA [endosymbiont of Sipalinus gigas]	1972134	d__Bacteria;k__norank;p__Proteobacteria;c__Gammaproteobacteria;o__norank;f__norank;g__Candidatus_Nardonella;s__endosymbiont_of_Sipalinus_gigas
PB2_gene_7076	166	WP_052259305.1	167	165/166(99.40)	1.43e-119	hypothetical protein [Pantoea agglomerans]	549	d__Bacteria;k__norank;p__Proteobacteria;c__Gammaproteobacteria;o__Enterobacterales;f__Erwiniaceae;g__Pantoea;s__Pantoea_agglomerans
PB2_gene_7080	49	WP_096617983.1	386	41/51(80.39)	1.82e-18	phage tail protein [Wolbachia pipientis]	955	d__Bacteria;k__norank;p__Proteobacteria;c__Alphaproteobacteria;o__Rickettsiales;f__Anaplasmataceae;g__Wolbachia;s__Wolbachia_pipientis
PB2_gene_7086	98	WP_126690661.1	81	44/97(45.36)	5.97e-12	hypothetical protein [Pantoea sp. YU22]	None	Unclassified
PB2_gene_7087	84	WP_111535205.1	84	59/84(70.24)	7.20e-38	hypothetical protein [Pantoea agglomerans]	549	d__Bacteria;k__norank;p__Proteobacteria;c__Gammaproteobacteria;o__Enterobacterales;f__Erwiniaceae;g__Pantoea;s__Pantoea_agglomerans
PB2_gene_7100	231	EEB56449.1	374	229/231(99.13)	9.99e-167	mannose-1-phosphate guanylyltransferase [Wolbachia endosymbiont of Culex quinquefasciatus JHB]	569881	d__Bacteria;k__norank;p__Proteobacteria;c__Alphaproteobacteria;o__Rickettsiales;f__Anaplasmataceae;g__Wolbachia;s__Wolbachia_endosymbiont_of_Culex_quinquefasciatus
PB2_gene_7122	56	PLL88853.1	220	36/54(66.67)	2.57e-17	catalase, partial [Klebsiella michiganensis]	1134687	d__Bacteria;k__norank;p__Proteobacteria;c__Gammaproteobacteria;o__Enterobacterales;f__Enterobacteriaceae;g__Klebsiella;s__Klebsiella_michiganensis

PB2_gene_7124	66	WP_033792410.1	125	60/65(92.31)	2.22e-35	peptidase [Pantoea septica]	472695	d__Bacteria;k__norank;p__Proteobacteria;c__Gammaproteobacteria;o__Enterobacterales;f__Erwiniaceae;g__Pantoea;s__Pantoea_septica
PB2_gene_7131	183	WP_038213650.1	332	115/180(63.89)	3.53e-82	DNA cytosine methyltransferase [Xenorhabdus bovienii]	40576	d__Bacteria;k__norank;p__Proteobacteria;c__Gammaproteobacteria;o__Enterobacterales;f__Morganellaceae;g__Xenorhabdus;s__Xenorhabdus_bovienii
PB2_gene_7137	248	TCZ21607.1	310	246/248(99.19)	1.25e-177	replication initiation protein [Pantoea agglomerans]	549	d__Bacteria;k__norank;p__Proteobacteria;c__Gammaproteobacteria;o__Enterobacterales;f__Erwiniaceae;g__Pantoea;s__Pantoea_agglomerans
PB2_gene_7150	247	OGH78632.1	1034	71/185(38.38)	9.89e-18	hypothetical protein A2224_02055, partial [Candidatus Magasanikbacteria bacterium	1798695	d__Bacteria;k__norank;p__Candidatus_Magasanikbacteria;c__norank;o__norank;f__norank;g__norank;s__Candidatus_Magasanikbacteria_bacterium_RIFOXYA2_FULL_40_20
PB2_gene_7156	85	RUA06787.1	914	25/54(46.30)	9.01e-07	hypothetical protein DSY43_01230	1913989	d__Bacteria;k__norank;p__Proteobacteria;c__Gammaproteobacteria;o__norank;f__norank;g__norank;s__Gammaproteobacteria_bacterium
PB2_gene_7189	243	WP_107320552.1	711	243/243(100.00)	3.91e-159	conjugal transfer protein TraY [Pantoea vagans]	470934	d__Bacteria;k__norank;p__Proteobacteria;c__Gammaproteobacteria;o__Enterobacterales;f__Erwiniaceae;g__Pantoea;s__Pantoea_vagans
PB2_gene_7193	125	WP_109706548.1	138	115/125(92.00)	2.10e-79	hypothetical protein [Pantoea ananatis]	553	d__Bacteria;k__norank;p__Proteobacteria;c__Gammaproteobacteria;o__Enterobacterales;f__Erwiniaceae;g__Pantoea;s__Pantoea_ananatis
PB2_gene_7197	46	WP_094106205.1	542	46/46(100.00)	8.69e-21	PilN family type IVB pilus formation outer membrane protein [Pantoea brenneri]	472694	d__Bacteria;k__norank;p__Proteobacteria;c__Gammaproteobacteria;o__Enterobacterales;f__Erwiniaceae;g__Pantoea;s__Pantoea_brenneri
PB2_gene_7204	242	WP_039387120.1	438	233/242(96.28)	2.68e-168	DUF1173 family protein [Pantoea agglomerans]	549	d__Bacteria;k__norank;p__Proteobacteria;c__Gammaproteobacteria;o__Enterobacterales;f__Erwiniaceae;g__Pantoea;s__Pantoea_agglomerans
PB2_gene_7208	111	ERM11014.1	225	94/111(84.68)	1.52e-59	hypothetical protein L584_00580 [Pantoea agglomerans Tx10]	1332072	d__Bacteria;k__norank;p__Proteobacteria;c__Gammaproteobacteria;o__Enterobacterales;f__Erwiniaceae;g__Pantoea;s__Pantoea_agglomerans
PB2_gene_7218	217	EWG64833.1	485	184/208(88.46)	4.22e-125	hypothetical protein P348_04635 [Enterobacter sp. DC3]	1395579	d__Bacteria;k__norank;p__Proteobacteria;c__Gammaproteobacteria;o__Enterobacterales;f__Enterobacteriaceae;g__Enterobacter;s__Enterobacter_sp._DC3
PB2_gene_7244	163	RYE27129.1	1122	44/117(37.61)	3.08e-16	hypothetical protein EOP45_02210	2021370	d__Bacteria;k__norank;p__Bacteroidetes;c__Sphingobacteriia;o__Sphingobacteriales;f__Sphingobacteriaceae;g__norank;s__Sphingobacteriaceae_bacterium
PB2_gene_7253	238	EUL32534.1	520	216/238(90.76)	3.68e-150	hypothetical protein P851_04014 [Klebsiella aerogenes UCI 48]	1400145	d__Bacteria;k__norank;p__Proteobacteria;c__Gammaproteobacteria;o__Enterobacterales;f__Enterobacteriaceae;g__Klebsiella;s__Klebsiella_aerogenes
PB2_gene_7258	193	PAW37531.1	204	190/193(98.45)	3.98e-137	methylthioribulose 1-phosphate dehydratase [Pantoea vagans]	470934	d__Bacteria;k__norank;p__Proteobacteria;c__Gammaproteobacteria;o__Enterobacterales;f__Erwiniaceae;g__Pantoea;s__Pantoea_vagans
PB2_gene_7267	136	WP_104093808.1	240	109/136(80.15)	3.73e-63	hypothetical protein [Pantoea sp. ICBG 985]	2071683	d__Bacteria;k__norank;p__Proteobacteria;c__Gammaproteobacteria;o__Enterobacterales;f__Erwiniaceae;g__Pantoea;s__Pantoea_sp._ICBG_985
PB2_gene_7276	53	SJZ36069.1	699	50/53(94.34)	4.90e-21	iron complex outermembrane recepter protein [Pantoea	470933	d__Bacteria;k__norank;p__Proteobacteria;c__Gammaproteobacteria;o__Enterobacterales;f__Erwiniaceae;g__Pantoea;s__Pantoea_eucalypti
PB2_gene_7287	74	ORM84681.1	74	74/74(100.00)	6.62e-43	hypothetical protein HA42_03840 [Pantoea deleyi]	470932	d__Bacteria;k__norank;p__Proteobacteria;c__Gammaproteobacteria;o__Enterobacterales;f__Erwiniaceae;g__Pantoea;s__Pantoea_deleyi
PB2_gene_7297	82	PAW34390.1	82	79/82(96.34)	2.17e-47	ribbon-helix-helix protein, CopG family [Pantoea vagans]	470934	d__Bacteria;k__norank;p__Proteobacteria;c__Gammaproteobacteria;o__Enterobacterales;f__Erwiniaceae;g__Pantoea;s__Pantoea_vagans
PB2_gene_7304	163	WP_111138991.1	167	162/163(99.39)	4.22e-109	DUF1198 family protein [Pantoea sp. ARC607]	2027922	d__Bacteria;k__norank;p__Proteobacteria;c__Gammaproteobacteria;o__Enterobacterales;f__Erwiniaceae;g__Pantoea;s__Pantoea_sp._ARC607
PB2_gene_7314	50	XP_018210550.1	244	39/52(75.00)	9.35e-15	hypothetical protein OGAPODRAFT_16488, partial [Ogataea polymorpha]	460523	d__Eukaryota;k__Fungi;p__Ascomycota;c__Saccharomycetes;o__Saccharomycetales;f__Pichiaceae;g__Ogataea;s__Ogataea_polymorpha
PB2_gene_7319	231	VEF71424.1	1225	228/231(98.70)	6.56e-147	Type I restriction-modification system methyltransferase subunit [Klebsiella oxytoca]	571	d__Bacteria;k__norank;p__Proteobacteria;c__Gammaproteobacteria;o__Enterobacterales;f__Enterobacteriaceae;g__Klebsiella;s__Klebsiella_oxytoca
PB2_gene_7320	79	XP_018284744.1	273	41/61(67.21)	2.13e-16	glycosyltransferase family 8 protein [Phycomyces blakesleeanus NRRL 1555(-)]	763407	d__Eukaryota;k__Fungi;p__Mucoromycota;c__Mucoromycetes;o__Mucorales;f__Phycomycetaceae;g__Phycomyces;s__Phycomyces_blakesleeanus

PB2_gene_7330	117	NP_758759.1	126	106/115(92.17)	1.13e-67	hypothetical protein (plasmid) [Erwinia pyrifoliae Ep1/96]	None	Unclassified
PB2_gene_7331	59	CAY76405.1	175	53/58(91.38)	2.14e-26	putative membrane protein (plasmid) [Erwinia pyrifoliae	644651	d__Bacteria;k__norank;p__Proteobacteria;c__Gammaproteobacteria;o__Enterobacterales;f__Erwiniaceae;g__Erwinia;s__Erwinia_pyrifoliae
PB2_gene_7338	84	ORM86041.1	544	84/84(100.00)	1.53e-49	hypothetical protein HA42_01695 [Pantoea deleyi]	470932	d__Bacteria;k__norank;p__Proteobacteria;c__Gammaproteobacteria;o__Enterobacterales;f__Erwiniaceae;g__Pantoea;s__Pantoea_deleyi
PB2_gene_7339	147	QAV43378.1	361	144/147(97.96)	1.65e-96	DNA sulfur modification protein DndB [Pantoea agglomerans]	549	d__Bacteria;k__norank;p__Proteobacteria;c__Gammaproteobacteria;o__Enterobacterales;f__Erwiniaceae;g__Pantoea;s__Pantoea_agglomerans
PB2_gene_7345	200	WP_039392441.1	243	199/200(99.50)	1.15e-140	hypothetical protein [Pantoea agglomerans]	549	d__Bacteria;k__norank;p__Proteobacteria;c__Gammaproteobacteria;o__Enterobacterales;f__Erwiniaceae;g__Pantoea;s__Pantoea_agglomerans
PB2_gene_7346	208	ORM81594.1	196	195/196(99.49)	1.64e-138	transcriptional regulator [Pantoea deleyi]	470932	d__Bacteria;k__norank;p__Proteobacteria;c__Gammaproteobacteria;o__Enterobacterales;f__Erwiniaceae;g__Pantoea;s__Pantoea_deleyi
PB2_gene_7371	229	WP_128086922.1	623	226/229(98.69)	2.77e-158	type VI secretion system baseplate subunit TssF [Pantoea	470932	d__Bacteria;k__norank;p__Proteobacteria;c__Gammaproteobacteria;o__Enterobacterales;f__Erwiniaceae;g__Pantoea;s__Pantoea_deleyi
PB2_gene_7381	228	ORM76848.1	397	219/317(69.09)	1.11e-120	MFS transporter [Pantoea deleyi]	470932	d__Bacteria;k__norank;p__Proteobacteria;c__Gammaproteobacteria;o__Enterobacterales;f__Erwiniaceae;g__Pantoea;s__Pantoea_deleyi
PB2_gene_7396	173	ELP25067.1	207	162/173(93.64)	8.33e-114	Phage head maturation protease [Pantoea agglomerans 299R]	1261128	d__Bacteria;k__norank;p__Proteobacteria;c__Gammaproteobacteria;o__Enterobacterales;f__Erwiniaceae;g__Pantoea;s__Pantoea_agglomerans
PB2_gene_7397	55	ELP25068.1	412	51/55(92.73)	1.67e-24	Phage portal protein [Pantoea agglomerans 299R]	1261128	d__Bacteria;k__norank;p__Proteobacteria;c__Gammaproteobacteria;o__Enterobacterales;f__Erwiniaceae;g__Pantoea;s__Pantoea_agglomerans
PB2_gene_7398	179	WP_107320564.1	179	178/179(99.44)	3.86e-121	conjugal transfer protein [Pantoea vagans]	470934	d__Bacteria;k__norank;p__Proteobacteria;c__Gammaproteobacteria;o__Enterobacterales;f__Erwiniaceae;g__Pantoea;s__Pantoea_vagans
PB2_gene_7400	216	AVE16789.1	233	216/216(100.00)	1.07e-156	2,3-bisphosphoglycerate-dependent phosphoglycerate mutase (plasmid) [Pantoea	470934	d__Bacteria;k__norank;p__Proteobacteria;c__Gammaproteobacteria;o__Enterobacterales;f__Erwiniaceae;g__Pantoea;s__Pantoea_vagans
PB2_gene_7405	161	WP_069788038.1	546	76/145(52.41)	3.13e-38	acyltransferase [Pseudomonas salomonii]	191391	d__Bacteria;k__norank;p__Proteobacteria;c__Gammaproteobacteria;o__Pseudomonadales;f__Pseudomonadaceae;g__Pseudomonas;s__Pseudomonas_salomonii
PB2_gene_7410	87	WP_107320532.1	250	86/87(98.85)	6.92e-54	site-specific integrase [Pantoea vagans]	470934	d__Bacteria;k__norank;p__Proteobacteria;c__Gammaproteobacteria;o__Enterobacterales;f__Erwiniaceae;g__Pantoea;s__Pantoea_vagans
PB2_gene_7428	223	AVV38612.1	274	190/223(85.20)	1.62e-135	DUF4123 domain-containing protein [Pantoea vagans]	470934	d__Bacteria;k__norank;p__Proteobacteria;c__Gammaproteobacteria;o__Enterobacterales;f__Erwiniaceae;g__Pantoea;s__Pantoea_vagans
PB2_gene_7442	223	WP_081115544.1	526	198/223(88.79)	3.59e-114	hypothetical protein [Pantoea agglomerans]	None	Unclassified
PB2_gene_7449	222	WP_107320560.1	1016	220/222(99.10)	8.75e-150	conjugal transfer protein [Pantoea vagans]	470934	d__Bacteria;k__norank;p__Proteobacteria;c__Gammaproteobacteria;o__Enterobacterales;f__Erwiniaceae;g__Pantoea;s__Pantoea_vagans
PB2_gene_7454	221	WP_064085588.1	637	215/221(97.29)	7.12e-144	DNA helicase II [Wolbachia endosymbiont of Dactylopius coccus]	None	Unclassified
PB2_gene_7473	149	WP_046101754.1	121	106/121(87.60)	2.40e-74	hypothetical protein [Pantoea anthophila]	53335	d__Bacteria;k__norank;p__Proteobacteria;c__Gammaproteobacteria;o__Enterobacterales;f__Erwiniaceae;g__Pantoea;s__norank
PB2_gene_7478	167	WP_034888825.1	345	164/167(98.20)	1.91e-114	integrase [Erwinia typographi]	371042	d__Bacteria;k__norank;p__Proteobacteria;c__Gammaproteobacteria;o__Enterobacterales;f__Erwiniaceae;g__Erwinia;s__Erwinia_typographi
PB2_gene_7479	50	WP_034888823.1	193	48/49(97.96)	2.36e-23	phage repressor protein CI [Erwinia typographi]	371042	d__Bacteria;k__norank;p__Proteobacteria;c__Gammaproteobacteria;o__Enterobacterales;f__Erwiniaceae;g__Erwinia;s__Erwinia_typographi
PB2_gene_7492	57	XP_016605096.1	415	25/53(47.17)	3.19e-11	hypothetical protein SPPG_07453 [Spizellomyces punctatus DAOM BR117]	645134	d__Eukaryota;k__Fungi;p__Chytridiomycota;c__Chytridiomycetes;o__Spizellomycetales;f__Spizellomycetaceae;g__Spizellomyces;s__Spizellomyces_punctatus
PB2_gene_7524	214	WP_094106214.1	458	185/196(94.39)	1.12e-125	shufflon system plasmid conjugative transfer pilus tip adhesin PilV [Pantoea brenneri]	472694	d__Bacteria;k__norank;p__Proteobacteria;c__Gammaproteobacteria;o__Enterobacterales;f__Erwiniaceae;g__Pantoea;s__Pantoea_brenneri

PB2_gene_7533	99	PGH37161.1	162	34/64(53.12)	4.18e-14	hypothetical protein CRN43_22805 [Candidatus	2044729	d__Bacteria;k__norank;p__Bacteroidetes;c__Cytophagia;o__Cytophagales;f__Cytophagaceae;g__Candidatus_Nephrothrix;s__Candidatus_Nephrothrix_sp._EaCA
PB2_gene_7557	159	WP_077188252.1	355	152/154(98.70)	2.41e-88	ankyrin repeat domain-containing protein, partial [Wolbachia	955	d__Bacteria;k__norank;p__Proteobacteria;c__Alphaproteobacteria;o__Rickettsiales;f__Anaplasmataceae;g__Wolbachia;s__Wolbachia_pipientis
PB2_gene_7560	212	WP_111138968.1	1510	203/212(95.75)	8.47e-121	ATP-dependent helicase [Pantoea sp. ARC607]	2027922	d__Bacteria;k__norank;p__Proteobacteria;c__Gammaproteobacteria;o__Enterobacterales;f__Erwiniaceae;g__Pantoea;s__Pantoea_sp._ARC607
PB2_gene_7565	174	PBQ28060.1	158	153/158(96.84)	1.26e-72	hypothetical protein BTO27_02880 [Wolbachia	2049495	d__Bacteria;k__norank;p__Proteobacteria;c__Alphaproteobacteria;o__Rickettsiales;f__Anaplasmataceae;g__Wolbachia;s__Wolbachia_pipientis
PB2_gene_7587	124	AAO88956.1	175	104/124(83.87)	4.29e-73	dTDP-6-deoxy-D-glucose-3,5-epimerase [Vibrio cholerae]	666	d__Bacteria;k__norank;p__Proteobacteria;c__Gammaproteobacteria;o__Vibrionales;f__Vibrionaceae;g__Vibrio;s__Vibrio_cholerae
PB2_gene_7588	83	WP_124890119.1	293	80/83(96.39)	3.33e-49	glucose-1-phosphate thymidylyltransferase [Pantoea agglomerans]	549	d__Bacteria;k__norank;p__Proteobacteria;c__Gammaproteobacteria;o__Enterobacterales;f__Erwiniaceae;g__Pantoea;s__Pantoea_agglomerans
PB2_gene_7589	181	WP_064704803.1	375	67/126(53.17)	6.42e-33	hypothetical protein [Pantoea agglomerans]	549	d__Bacteria;k__norank;p__Proteobacteria;c__Gammaproteobacteria;o__Enterobacterales;f__Erwiniaceae;g__Pantoea;s__Pantoea_agglomerans
PB2_gene_7598	106	CDL22603.1	205	81/104(77.88)	3.77e-47	antirestriction protein [Klebsiella pneumoniae IS53]	1432554	d__Bacteria;k__norank;p__Proteobacteria;c__Gammaproteobacteria;o__Enterobacterales;f__Enterobacteriaceae;g__Klebsiella;s__Klebsiella_pneumoniae
PB2_gene_7599	52	ELP24037.1	322	52/52(100.00)	5.17e-29	PAAR repeat-containing protein [Pantoea agglomerans 299R]	1261128	d__Bacteria;k__norank;p__Proteobacteria;c__Gammaproteobacteria;o__Enterobacterales;f__Erwiniaceae;g__Pantoea;s__Pantoea_agglomerans
PB2_gene_7600	85	WP_105100181.1	85	80/85(94.12)	5.85e-51	hypothetical protein [Pantoea ananatis]	2750734	d__Bacteria;k__norank;p__Proteobacteria;c__Gammaproteobacteria;o__Enterobacterales;f__Erwiniaceae;g__Pantoea;s__Pantoea_sp._WMus005
PB2_gene_7609	50	WP_111141248.1	485	45/50(90.00)	4.52e-20	PLP-dependent aminotransferase family protein [Pantoea sp.	2027922	d__Bacteria;k__norank;p__Proteobacteria;c__Gammaproteobacteria;o__Enterobacterales;f__Erwiniaceae;g__Pantoea;s__Pantoea_sp._ARC607
PB2_gene_7618	127	WP_128086483.1	228	109/123(88.62)	7.68e-65	BapA prefix-like domain-containing protein, partial	470932	d__Bacteria;k__norank;p__Proteobacteria;c__Gammaproteobacteria;o__Enterobacterales;f__Erwiniaceae;g__Pantoea;s__Pantoea_deleyi
PB2_gene_7628	137	ELP25052.1	175	90/136(66.18)	9.83e-57	putative prophage protein [Pantoea agglomerans 299R]	1261128	d__Bacteria;k__norank;p__Proteobacteria;c__Gammaproteobacteria;o__Enterobacterales;f__Erwiniaceae;g__Pantoea;s__Pantoea_agglomerans
PB2_gene_7629	65	WP_064704804.1	250	43/65(66.15)	8.37e-22	glycosyltransferase family 25 protein [Pantoea agglomerans]	549	d__Bacteria;k__norank;p__Proteobacteria;c__Gammaproteobacteria;o__Enterobacterales;f__Erwiniaceae;g__Pantoea;s__Pantoea_agglomerans
PB2_gene_7633	171	WP_111535232.1	192	171/171(100.00)	3.10e-121	hypothetical protein [Pantoea agglomerans]	53335	d__Bacteria;k__norank;p__Proteobacteria;c__Gammaproteobacteria;o__Enterobacterales;f__Erwiniaceae;g__Pantoea;s__norank
PB2_gene_7652	104	CCO26058.1	229	53/102(51.96)	5.46e-33	JmjC domain-containing histone demethylation protein 1D/E/F [Rhizoctonia solani AG-1 IB]	1108050	d__Eukaryota;k__Fungi;p__Basidiomycota;c__Agaricomycetes;o__Cantharellales;f__Ceratobasidiaceae;g__Rhizoctonia;s__Rhizoctonia_solani
PB2_gene_7661	203	RRY99649.1	327	108/200(54.00)	2.65e-68	ATPase, partial [Klebsiella pneumoniae]	573	d__Bacteria;k__norank;p__Proteobacteria;c__Gammaproteobacteria;o__Enterobacterales;f__Enterobacteriaceae;g__Klebsiella;s__Klebsiella_pneumoniae
PB2_gene_7662	92	WP_123220264.1	332	50/88(56.82)	1.34e-26	DNA cytosine methyltransferase [Salmonella enterica]	28901	d__Bacteria;k__norank;p__Proteobacteria;c__Gammaproteobacteria;o__Enterobacterales;f__Enterobacteriaceae;g__Salmonella;s__Salmonella_enterica
PB2_gene_7667	60	ORZ14038.1	430	19/33(57.58)	2.88e-06	pyridoxal-dependent decarboxylase [Absidia repens]	90262	d__Eukaryota;k__Fungi;p__Mucoromycota;c__Mucoromycetes;o__Mucorales;f__Cunninghamellaceae;g__Absidia;s__Absidia_repens
PB2_gene_7681	87	ELP25057.1	180	84/87(96.55)	1.83e-51	Prophage baseplate assembly protein V [Pantoea agglomerans 299R]	1261128	d__Bacteria;k__norank;p__Proteobacteria;c__Gammaproteobacteria;o__Enterobacterales;f__Erwiniaceae;g__Pantoea;s__Pantoea_agglomerans
PB2_gene_7693	84	TCZ21606.1	84	84/84(100.00)	1.29e-49	replication protein RepA [Pantoea agglomerans]	549	d__Bacteria;k__norank;p__Proteobacteria;c__Gammaproteobacteria;o__Enterobacterales;f__Erwiniaceae;g__Pantoea;s__Pantoea_agglomerans
PB2_gene_7694	51	WP_039390287.1	178	50/51(98.04)	1.55e-29	phospholipase D family protein [Pantoea agglomerans]	549	d__Bacteria;k__norank;p__Proteobacteria;c__Gammaproteobacteria;o__Enterobacterales;f__Erwiniaceae;g__Pantoea;s__Pantoea_agglomerans
PB2_gene_7715	182	WP_111141457.1	387	180/182(98.90)	4.67e-124	bifunctional chorismate mutase/prephenate dehydratase [Pantoea sp. ARC607]	2027922	d__Bacteria;k__norank;p__Proteobacteria;c__Gammaproteobacteria;o__Enterobacterales;f__Erwiniaceae;g__Pantoea;s__Pantoea_sp._ARC607

PB3_gene_2	419	ORM72621.1	419	400/419(95.47)	1.86e-280	diguanylate cyclase [Pantoea deleyi]	470932	d__Bacteria;k__norank;p__Proteobacteria;c__Gammaproteobacteria;o__Enterobacterales;f__Erwiniaceae;g__Pantoea;s__Pantoea_deleyi
PB3_gene_13	166	WP_052953251.1	172	147/166(88.55)	2.72e-116	hypothetical protein [Enterobacter cloacae]	550	d__Bacteria;k__norank;p__Proteobacteria;c__Gammaproteobacteria;o__Enterobacterales;f__Enterobacteriaceae;g__Enterobacter;s__Enterobacter_cloacae
PB3_gene_15	374	WP_104189451.1	383	320/374(85.56)	1.09e-217	MFS transporter [Pantoea sp. ICBG 828]	2630326	d__Bacteria;k__norank;p__Proteobacteria;c__Gammaproteobacteria;o__Enterobacterales;f__Erwiniaceae;g__Pantoea;s__norank
PB3_gene_17	403	WP_104189455.1	403	344/403(85.36)	3.30e-236	tyrosine permease [Pantoea sp. ICBG 828]	2630326	d__Bacteria;k__norank;p__Proteobacteria;c__Gammaproteobacteria;o__Enterobacterales;f__Erwiniaceae;g__Pantoea;s__norank
PB3_gene_71	312	WP_111139627.1	312	294/312(94.23)	1.20e-206	D-2-hydroxyacid dehydrogenase [Pantoea sp. ARC607]	2027922	d__Bacteria;k__norank;p__Proteobacteria;c__Gammaproteobacteria;o__Enterobacterales;f__Erwiniaceae;g__Pantoea;s__Pantoea_sp._ARC607
PB3_gene_85	320	ORM85464.1	320	320/320(100.00)	2.04e-218	phosphate ABC transporter permease subunit PstC [Pantoea deleyi]	470932	d__Bacteria;k__norank;p__Proteobacteria;c__Gammaproteobacteria;o__Enterobacterales;f__Erwiniaceae;g__Pantoea;s__Pantoea_deleyi
PB3_gene_102	712	ORM85450.1	712	704/712(98.88)	0	SAM-dependent methyltransferase [Pantoea	470932	d__Bacteria;k__norank;p__Proteobacteria;c__Gammaproteobacteria;o__Enterobacterales;f__Erwiniaceae;g__Pantoea;s__Pantoea_deleyi
PB3_gene_128	454	WP_111139721.1	454	432/454(95.15)	4.21e-313	uroporphyrinogen-III C-methyltransferase [Pantoea sp. ARC607]	2027922	d__Bacteria;k__norank;p__Proteobacteria;c__Gammaproteobacteria;o__Enterobacterales;f__Erwiniaceae;g__Pantoea;s__Pantoea_sp._ARC607
PB3_gene_133	355	ORM77685.1	347	320/347(92.22)	6.75e-175	cell division protein DamX [Pantoea deleyi]	470932	d__Bacteria;k__norank;p__Proteobacteria;c__Gammaproteobacteria;o__Enterobacterales;f__Erwiniaceae;g__Pantoea;s__Pantoea_deleyi
PB3_gene_136	415	WP_128086470.1	423	387/415(93.25)	1.94e-259	DNA uptake porin HofQ [Pantoea deleyi]	470932	d__Bacteria;k__norank;p__Proteobacteria;c__Gammaproteobacteria;o__Enterobacterales;f__Erwiniaceae;g__Pantoea;s__Pantoea_deleyi
PB3_gene_155	775	ORM80863.1	775	772/775(99.61)	0	RNA-binding transcriptional accessory protein [Pantoea	470932	d__Bacteria;k__norank;p__Proteobacteria;c__Gammaproteobacteria;o__Enterobacterales;f__Erwiniaceae;g__Pantoea;s__Pantoea_deleyi
PB3_gene_161	693	WP_111140864.1	693	672/691(97.25)	0	4-alpha-glucanotransferase [Pantoea sp. ARC607]	2027922	d__Bacteria;k__norank;p__Proteobacteria;c__Gammaproteobacteria;o__Enterobacterales;f__Erwiniaceae;g__Pantoea;s__Pantoea_sp._ARC607
PB3_gene_174	420	WP_111139907.1	410	399/409(97.56)	2.54e-292	multifunctional CCA addition/repair protein [Pantoea	2027922	d__Bacteria;k__norank;p__Proteobacteria;c__Gammaproteobacteria;o__Enterobacterales;f__Erwiniaceae;g__Pantoea;s__Pantoea_sp._ARC607
PB3_gene_175	206	ORM82213.1	206	205/206(99.51)	8.21e-143	hypothetical protein HA42_08920 [Pantoea deleyi]	470932	d__Bacteria;k__norank;p__Proteobacteria;c__Gammaproteobacteria;o__Enterobacterales;f__Erwiniaceae;g__Pantoea;s__Pantoea_deleyi
PB3_gene_202	99	STU59524.1	131	46/83(55.42)	1.33e-20	Uncharacterised protein [Klebsiella pneumoniae subsp.	574	d__Bacteria;k__norank;p__Proteobacteria;c__Gammaproteobacteria;o__Enterobacterales;f__Enterobacteriaceae;g__Klebsiella;s__Klebsiella_pneumoniae
PB3_gene_204	78	ORM86107.1	104	78/78(100.00)	3.30e-43	hypothetical protein HA42_01330 [Pantoea deleyi]	470932	d__Bacteria;k__norank;p__Proteobacteria;c__Gammaproteobacteria;o__Enterobacterales;f__Erwiniaceae;g__Pantoea;s__Pantoea_deleyi
PB3_gene_205	267	ORM86106.1	267	266/267(99.63)	2.83e-187	tryptophan synthase subunit alpha [Pantoea deleyi]	470932	d__Bacteria;k__norank;p__Proteobacteria;c__Gammaproteobacteria;o__Enterobacterales;f__Erwiniaceae;g__Pantoea;s__Pantoea_deleyi
PB3_gene_207	453	ORM86104.1	453	445/453(98.23)	9.27e-316	bifunctional indole-3-glycerol phosphate synthase/phosphoribosylanthranilate isomerase [Pantoea deleyi]	470932	d__Bacteria;k__norank;p__Proteobacteria;c__Gammaproteobacteria;o__Enterobacterales;f__Erwiniaceae;g__Pantoea;s__Pantoea_deleyi
PB3_gene_216	280	ORM86097.1	253	248/253(98.02)	5.46e-176	YciK family oxidoreductase [Pantoea deleyi]	470932	d__Bacteria;k__norank;p__Proteobacteria;c__Gammaproteobacteria;o__Enterobacterales;f__Erwiniaceae;g__Pantoea;s__Pantoea_deleyi
PB3_gene_235	689	ORM86082.1	689	685/689(99.42)	0	carbon starvation protein A [Pantoea deleyi]	470932	d__Bacteria;k__norank;p__Proteobacteria;c__Gammaproteobacteria;o__Enterobacterales;f__Erwiniaceae;g__Pantoea;s__Pantoea_deleyi
PB3_gene_236	665	ORM86135.1	644	636/644(98.76)	0	exoribonuclease II [Pantoea deleyi]	470932	d__Bacteria;k__norank;p__Proteobacteria;c__Gammaproteobacteria;o__Enterobacterales;f__Erwiniaceae;g__Pantoea;s__Pantoea_deleyi
PB3_gene_237	767	ORM86081.1	767	760/767(99.09)	0	CbbBc protein [Pantoea deleyi]	470932	d__Bacteria;k__norank;p__Proteobacteria;c__Gammaproteobacteria;o__Enterobacterales;f__Erwiniaceae;g__Pantoea;s__Pantoea_deleyi
PB3_gene_240	83	WP_031590313.1	152	68/82(82.93)	1.95e-46	DUF943 family protein [Pantoea agglomerans]	549	d__Bacteria;k__norank;p__Proteobacteria;c__Gammaproteobacteria;o__Enterobacterales;f__Erwiniaceae;g__Pantoea;s__Pantoea_agglomerans

PB3_gene_241	103	OWD65922.1	103	98/103(95.15)	1.51e-59	transposase [Escherichia coli]	562	d__Bacteria;k__norank;p__Proteobacteria;c__Gammaproteobacteria;o__Enterobacterales;f__Enterobacteriaceae;g__Escherichia;s__Escherichia_coli
PB3_gene_261	145	ORM82740.1	145	142/145(97.93)	7.14e-96	D-tyrosyl-tRNA(Tyr) deacylase [Pantoea deleyi]	470932	d__Bacteria;k__norank;p__Proteobacteria;c__Gammaproteobacteria;o__Enterobacterales;f__Erwiniaceae;g__Pantoea;s__Pantoea_deleyi
PB3_gene_267	469	ORM82745.1	469	469/469(100.00)	0	nitrogen regulation protein NR(I) [Pantoea deleyi]	470932	d__Bacteria;k__norank;p__Proteobacteria;c__Gammaproteobacteria;o__Enterobacterales;f__Erwiniaceae;g__Pantoea;s__Pantoea_deleyi
PB3_gene_268	457	WP_111141989.1	457	434/457(94.97)	0	oxygen-independent coproporphyrinogen III oxidase [Pantoea sp. ARC607]	2027922	d__Bacteria;k__norank;p__Proteobacteria;c__Gammaproteobacteria;o__Enterobacterales;f__Erwiniaceae;g__Pantoea;s__Pantoea_sp._ARC607
PB3_gene_277	424	ORM77702.1	433	421/424(99.29)	1.94e-315	L-sorbosone dehydrogenase [Pantoea deleyi]	470932	d__Bacteria;k__norank;p__Proteobacteria;c__Gammaproteobacteria;o__Enterobacterales;f__Erwiniaceae;g__Pantoea;s__Pantoea_deleyi
PB3_gene_281	440	WP_111140364.1	440	434/440(98.64)	1.24e-307	MFS transporter [Pantoea sp. ARC607]	2027922	d__Bacteria;k__norank;p__Proteobacteria;c__Gammaproteobacteria;o__Enterobacterales;f__Erwiniaceae;g__Pantoea;s__Pantoea_sp._ARC607
PB3_gene_282	337	ORM76789.1	325	313/325(96.31)	4.95e-227	glycosyltransferase, family 8, partial [Pantoea deleyi]	470932	d__Bacteria;k__norank;p__Proteobacteria;c__Gammaproteobacteria;o__Enterobacterales;f__Erwiniaceae;g__Pantoea;s__Pantoea_deleyi
PB3_gene_287	500	ORM76785.1	500	496/500(99.20)	0	ABC transporter [Pantoea deleyi]	470932	d__Bacteria;k__norank;p__Proteobacteria;c__Gammaproteobacteria;o__Enterobacterales;f__Erwiniaceae;g__Pantoea;s__Pantoea_deleyi
PB3_gene_290	286	WP_111140372.1	286	267/286(93.36)	4.19e-178	ribokinase [Pantoea sp. ARC607]	2027922	d__Bacteria;k__norank;p__Proteobacteria;c__Gammaproteobacteria;o__Enterobacterales;f__Erwiniaceae;g__Pantoea;s__Pantoea_sp._ARC607
PB3_gene_302	681	ORM81498.1	681	663/680(97.50)	0	fusaric acid resistance protein [Pantoea deleyi]	470932	d__Bacteria;k__norank;p__Proteobacteria;c__Gammaproteobacteria;o__Enterobacterales;f__Erwiniaceae;g__Pantoea;s__Pantoea_deleyi
PB3_gene_304	437	ORM81500.1	437	423/437(96.80)	9.37e-306	serine/threonine protein kinase [Pantoea deleyi]	470932	d__Bacteria;k__norank;p__Proteobacteria;c__Gammaproteobacteria;o__Enterobacterales;f__Erwiniaceae;g__Pantoea;s__Pantoea_deleyi
PB3_gene_318	317	ORM80695.1	317	311/317(98.11)	2.20e-216	hypothetical protein HA42_13480 [Pantoea deleyi]	470932	d__Bacteria;k__norank;p__Proteobacteria;c__Gammaproteobacteria;o__Enterobacterales;f__Erwiniaceae;g__Pantoea;s__Pantoea_deleyi
PB3_gene_324	166	KOA68159.1	88	87/89(97.75)	4.55e-19	acid-shock protein, partial [Pantoea sp. CFSAN033090]	1690502	d__Bacteria;k__norank;p__Proteobacteria;c__Gammaproteobacteria;o__Enterobacterales;f__Erwiniaceae;g__Pantoea;s__Pantoea_sp._CFSAN033090
PB3_gene_325	269	ORM80701.1	269	269/269(100.00)	1.17e-196	serine protease [Pantoea deleyi]	470932	d__Bacteria;k__norank;p__Proteobacteria;c__Gammaproteobacteria;o__Enterobacterales;f__Erwiniaceae;g__Pantoea;s__Pantoea_deleyi
PB3_gene_326	1300	WP_111139499.1	1300	1268/1300(97.54)	0	ATP-dependent RNA helicase HrpA [Pantoea sp. ARC607]	2027922	d__Bacteria;k__norank;p__Proteobacteria;c__Gammaproteobacteria;o__Enterobacterales;f__Erwiniaceae;g__Pantoea;s__Pantoea_sp._ARC607
PB3_gene_330	876	WP_111139495.1	876	839/876(95.78)	0	YdbH family protein [Pantoea sp. ARC607]	2027922	d__Bacteria;k__norank;p__Proteobacteria;c__Gammaproteobacteria;o__Enterobacterales;f__Erwiniaceae;g__Pantoea;s__Pantoea_sp._ARC607
PB3_gene_355	491	WP_111138709.1	495	474/491(96.54)	0	glycerol kinase [Pantoea sp. ARC607]	2027922	d__Bacteria;k__norank;p__Proteobacteria;c__Gammaproteobacteria;o__Enterobacterales;f__Erwiniaceae;g__Pantoea;s__Pantoea_sp._ARC607
PB3_gene_368	82	ORM82385.1	82	82/82(100.00)	1.53e-47	hypothetical protein HA42_08460 [Pantoea deleyi]	470932	d__Bacteria;k__norank;p__Proteobacteria;c__Gammaproteobacteria;o__Enterobacterales;f__Erwiniaceae;g__Pantoea;s__Pantoea_deleyi
PB3_gene_370	56	SJZ35612.1	56	52/56(92.86)	9.74e-26	hypothetical protein SAMN03097723_0723 [Pantoea agglomerans]	470933	d__Bacteria;k__norank;p__Proteobacteria;c__Gammaproteobacteria;o__Enterobacterales;f__Erwiniaceae;g__Pantoea;s__Pantoea_eucalypti
PB3_gene_371	353	ORM82384.1	353	338/352(96.02)	6.10e-248	glycosyl transferase family 9 [Pantoea deleyi]	470932	d__Bacteria;k__norank;p__Proteobacteria;c__Gammaproteobacteria;o__Enterobacterales;f__Erwiniaceae;g__Pantoea;s__Pantoea_deleyi
PB3_gene_377	370	WP_111138684.1	370	367/370(99.19)	2.92e-275	tRNA 2-thiouridine(34) synthase MnmA [Pantoea sp. ARC607]	2027922	d__Bacteria;k__norank;p__Proteobacteria;c__Gammaproteobacteria;o__Enterobacterales;f__Erwiniaceae;g__Pantoea;s__Pantoea_sp._ARC607
PB3_gene_382	374	WP_111138679.1	374	369/374(98.66)	9.94e-285	cupin domain-containing protein [Pantoea sp. ARC607]	2027922	d__Bacteria;k__norank;p__Proteobacteria;c__Gammaproteobacteria;o__Enterobacterales;f__Erwiniaceae;g__Pantoea;s__Pantoea_sp._ARC607
PB3_gene_383	409	ORM82371.1	409	404/409(98.78)	1.25e-298	peptidase T [Pantoea deleyi]	470932	d__Bacteria;k__norank;p__Proteobacteria;c__Gammaproteobacteria;o__Enterobacterales;f__Erwiniaceae;g__Pantoea;s__Pantoea_deleyi
PB3_gene_387	347	WP_111138675.1	347	337/347(97.12)	3.28e-249	spermidine/putrescine ABC transporter substrate-binding protein PotD [Pantoea sp.	2027922	d__Bacteria;k__norank;p__Proteobacteria;c__Gammaproteobacteria;o__Enterobacterales;f__Erwiniaceae;g__Pantoea;s__Pantoea_sp._ARC607

PB3_gene_388	277	ORM82366.1	277	274/277(98.92)	4.56e-198	NAD-dependent protein deacylase [Pantoea deleyi]	470932	d__Bacteria;k__norank;p__Proteobacteria;c__Gammaproteobacteria;o__Enterobacterales;f__Erwiniaceae;g__Pantoea;s__Pantoea_deleyi
PB3_gene_392	312	WP_111141337.1	312	304/312(97.44)	1.17e-213	LysR family transcriptional regulator [Pantoea sp. ARC607]	2027922	d__Bacteria;k__norank;p__Proteobacteria;c__Gammaproteobacteria;o__Enterobacterales;f__Erwiniaceae;g__Pantoea;s__Pantoea_sp._ARC607
PB3_gene_411	342	WP_111142264.1	342	339/342(99.12)	2.48e-246	EF-P beta-lysylation protein EpmB [Pantoea sp. ARC607]	2027922	d__Bacteria;k__norank;p__Proteobacteria;c__Gammaproteobacteria;o__Enterobacterales;f__Erwiniaceae;g__Pantoea;s__Pantoea_sp._ARC607
PB3_gene_425	585	ORM86315.1	585	585/585(100.00)	0	sulfite reductase subunit alpha [Pantoea deleyi]	470932	d__Bacteria;k__norank;p__Proteobacteria;c__Gammaproteobacteria;o__Enterobacterales;f__Erwiniaceae;g__Pantoea;s__Pantoea_deleyi
PB3_gene_445	392	WP_111138931.1	392	371/392(94.64)	1.44e-268	FAD-binding oxidoreductase [Pantoea sp. ARC607]	2027922	d__Bacteria;k__norank;p__Proteobacteria;c__Gammaproteobacteria;o__Enterobacterales;f__Erwiniaceae;g__Pantoea;s__Pantoea_sp._ARC607
PB3_gene_467	897	ORM86347.1	897	897/897(100.00)	0	translation initiation factor IF-2 [Pantoea deleyi]	470932	d__Bacteria;k__norank;p__Proteobacteria;c__Gammaproteobacteria;o__Enterobacterales;f__Erwiniaceae;g__Pantoea;s__Pantoea_deleyi
PB3_gene_521	285	ORM83032.1	285	283/284(99.65)	2.84e-196	phosphatidate cytidylyltransferase [Pantoea deleyi]	470932	d__Bacteria;k__norank;p__Proteobacteria;c__Gammaproteobacteria;o__Enterobacterales;f__Erwiniaceae;g__Pantoea;s__Pantoea_deleyi
PB3_gene_526	283	ORM83028.1	283	283/283(100.00)	1.06e-190	translation elongation factor Ts [Pantoea deleyi]	470932	d__Bacteria;k__norank;p__Proteobacteria;c__Gammaproteobacteria;o__Enterobacterales;f__Erwiniaceae;g__Pantoea;s__Pantoea_deleyi
PB3_gene_528	38	SKA07703.1	159	38/38(100.00)	1.45e-19	Uncharacterized conserved protein YehS, DUF1456 family [Pantoea agglomerans]	470933	d__Bacteria;k__norank;p__Proteobacteria;c__Gammaproteobacteria;o__Enterobacterales;f__Erwiniaceae;g__Pantoea;s__Pantoea_eucalypti
PB3_gene_537	281	WP_108452539.1	286	178/269(66.17)	7.22e-122	hypothetical protein [Klebsiella pneumoniae]	573	d__Bacteria;k__norank;p__Proteobacteria;c__Gammaproteobacteria;o__Enterobacterales;f__Enterobacteriaceae;g__Klebsiella;s__Klebsiella_pneumoniae
PB3_gene_543	184	WP_112879344.1	198	65/174(37.36)	3.47e-28	MULTISPECIES: type 1 fimbrial protein [Photorhabdus]	2620880	d__Bacteria;k__norank;p__Proteobacteria;c__Gammaproteobacteria;o__Enterobacterales;f__Morganellaceae;g__Photorhabdus;s__norank
PB3_gene_544	198	WP_063877213.1	194	93/167(55.69)	4.15e-59	type 1 fimbrial protein [Pantoea sp. OXWO6B1]	1835724	d__Bacteria;k__norank;p__Proteobacteria;c__Gammaproteobacteria;o__Enterobacterales;f__Erwiniaceae;g__Pantoea;s__Pantoea_sp._OXWO6B1
PB3_gene_547	187	ETX44501.1	180	82/175(46.86)	6.78e-47	hypothetical protein P805_01838 [Serratia marcescens BIDMC 44]	1400186	d__Bacteria;k__norank;p__Proteobacteria;c__Gammaproteobacteria;o__Enterobacterales;f__Yersiniaceae;g__Serratia;s__Serratia_marcescens
PB3_gene_557	350	WP_111139766.1	350	341/350(97.43)	6.06e-226	AbrB family transcriptional regulator [Pantoea sp. ARC607]	2027922	d__Bacteria;k__norank;p__Proteobacteria;c__Gammaproteobacteria;o__Enterobacterales;f__Erwiniaceae;g__Pantoea;s__Pantoea_sp._ARC607
PB3_gene_561	129	WP_111139768.1	238	125/129(96.90)	6.58e-83	5-amino-6-(5-phospho-D-ribitylamino)uracil phosphatase YigB [Pantoea sp. ARC607]	2027922	d__Bacteria;k__norank;p__Proteobacteria;c__Gammaproteobacteria;o__Enterobacterales;f__Erwiniaceae;g__Pantoea;s__Pantoea_sp._ARC607
PB3_gene_564	268	ORM81772.1	268	262/268(97.76)	1.90e-181	short chain dehydrogenase [Pantoea deleyi]	470932	d__Bacteria;k__norank;p__Proteobacteria;c__Gammaproteobacteria;o__Enterobacterales;f__Erwiniaceae;g__Pantoea;s__Pantoea_deleyi
PB3_gene_583	1031	ORM83602.1	1031	999/1030(96.99)	0	multidrug efflux RND transporter permease subunit [Pantoea deleyi]	470932	d__Bacteria;k__norank;p__Proteobacteria;c__Gammaproteobacteria;o__Enterobacterales;f__Erwiniaceae;g__Pantoea;s__Pantoea_deleyi
PB3_gene_585	296	ORM83604.1	296	279/296(94.26)	6.29e-210	hypothetical protein HA42_06035 [Pantoea deleyi]	470932	d__Bacteria;k__norank;p__Proteobacteria;c__Gammaproteobacteria;o__Enterobacterales;f__Erwiniaceae;g__Pantoea;s__Pantoea_deleyi
PB3_gene_587	374	ORM83625.1	369	352/369(95.39)	1.31e-256	NAD(P)H-binding protein [Pantoea deleyi]	470932	d__Bacteria;k__norank;p__Proteobacteria;c__Gammaproteobacteria;o__Enterobacterales;f__Erwiniaceae;g__Pantoea;s__Pantoea_deleyi
PB3_gene_592	386	ORM83610.1	379	366/379(96.57)	1.05e-263	hydrolase [Pantoea deleyi]	470932	d__Bacteria;k__norank;p__Proteobacteria;c__Gammaproteobacteria;o__Enterobacterales;f__Erwiniaceae;g__Pantoea;s__Pantoea_deleyi
PB3_gene_600	238	WP_111141225.1	238	237/238(99.58)	1.33e-157	arginine ABC transporter permease ArtQ [Pantoea sp.	2027922	d__Bacteria;k__norank;p__Proteobacteria;c__Gammaproteobacteria;o__Enterobacterales;f__Erwiniaceae;g__Pantoea;s__Pantoea_sp._ARC607
PB3_gene_605	277	ORM81967.1	274	265/274(96.72)	1.08e-193	N-acetylmuramoyl-L-alanine amidase [Pantoea deleyi]	470932	d__Bacteria;k__norank;p__Proteobacteria;c__Gammaproteobacteria;o__Enterobacterales;f__Erwiniaceae;g__Pantoea;s__Pantoea_deleyi
PB3_gene_609	545	WP_111141234.1	545	527/545(96.70)	0	ATP-dependent endonuclease [Pantoea sp. ARC607]	2027922	d__Bacteria;k__norank;p__Proteobacteria;c__Gammaproteobacteria;o__Enterobacterales;f__Erwiniaceae;g__Pantoea;s__Pantoea_sp._ARC607
PB3_gene_617	322	ORM75878.1	322	321/322(99.69)	1.52e-230	thioredoxin-disulfide reductase [Pantoea deleyi]	470932	d__Bacteria;k__norank;p__Proteobacteria;c__Gammaproteobacteria;o__Enterobacterales;f__Erwiniaceae;g__Pantoea;s__Pantoea_deleyi

PB3_gene_627	378	WP_111139631.1	382	341/378(90.21)	1.06e-241	lactaldehyde reductase [Pantoea sp. ARC607]	2027922	d__Bacteria;k__norank;p__Proteobacteria;c__Gammaproteobacteria;o__Enterobacterales;f__Erwiniaceae;g__Pantoea;s__Pantoea_sp._ARC607
PB3_gene_639	431	ORM79685.1	433	429/433(99.08)	0	maltoporin [Pantoea deleyi]	470932	d__Bacteria;k__norank;p__Proteobacteria;c__Gammaproteobacteria;o__Enterobacterales;f__Erwiniaceae;g__Pantoea;s__Pantoea_deleyi
PB3_gene_644	800	ORM83249.1	800	776/800(97.00)	0	maltodextrin phosphorylase [Pantoea deleyi]	470932	d__Bacteria;k__norank;p__Proteobacteria;c__Gammaproteobacteria;o__Enterobacterales;f__Erwiniaceae;g__Pantoea;s__Pantoea_deleyi
PB3_gene_655	100	EFM17731.1	420	89/100(89.00)	1.40e-54	DNA-directed DNA polymerase [Pantoea sp. aB]	517433	d__Bacteria;k__norank;p__Proteobacteria;c__Gammaproteobacteria;o__Enterobacterales;f__Erwiniaceae;g__Pantoea;s__Pantoea_sp._aB
PB3_gene_665	404	WP_123802123.1	404	357/403(88.59)	3.52e-243	MFS transporter [Pantoea sp. RIT388]	1869313	d__Bacteria;k__norank;p__Proteobacteria;c__Gammaproteobacteria;o__Enterobacterales;f__Erwiniaceae;g__Pantoea;s__Pantoea_sp._RIT388
PB3_gene_674	103	WP_110412079.1	103	92/103(89.32)	2.92e-57	transcriptional regulator [Pantoea vagans]	470934	d__Bacteria;k__norank;p__Proteobacteria;c__Gammaproteobacteria;o__Enterobacterales;f__Erwiniaceae;g__Pantoea;s__Pantoea_vagans
PB3_gene_684	450	ORM82912.1	450	447/450(99.33)	1.15e-308	lysine-sensitive aspartokinase 3 [Pantoea deleyi]	470932	d__Bacteria;k__norank;p__Proteobacteria;c__Gammaproteobacteria;o__Enterobacterales;f__Erwiniaceae;g__Pantoea;s__Pantoea_deleyi
PB3_gene_687	551	WP_111141931.1	541	533/540(98.70)	0	Na/Pi cotransporter family protein [Pantoea sp. ARC607]	2027922	d__Bacteria;k__norank;p__Proteobacteria;c__Gammaproteobacteria;o__Enterobacterales;f__Erwiniaceae;g__Pantoea;s__Pantoea_sp._ARC607
PB3_gene_697	807	ORM82924.1	807	804/807(99.63)	0	glycerol-3-phosphate 1-O-acyltransferase [Pantoea deleyi]	470932	d__Bacteria;k__norank;p__Proteobacteria;c__Gammaproteobacteria;o__Enterobacterales;f__Erwiniaceae;g__Pantoea;s__Pantoea_deleyi
PB3_gene_714	478	WP_111141285.1	478	451/478(94.35)	0	aldehyde dehydrogenase [Pantoea sp. ARC607]	2027922	d__Bacteria;k__norank;p__Proteobacteria;c__Gammaproteobacteria;o__Enterobacterales;f__Erwiniaceae;g__Pantoea;s__Pantoea_sp._ARC607
PB3_gene_721	388	ORM76352.1	388	379/388(97.68)	5.93e-275	methionine aminotransferase [Pantoea deleyi]	470932	d__Bacteria;k__norank;p__Proteobacteria;c__Gammaproteobacteria;o__Enterobacterales;f__Erwiniaceae;g__Pantoea;s__Pantoea_deleyi
PB3_gene_724	516	WP_111141295.1	506	476/506(94.07)	0	carboxylesterase/lipase family protein [Pantoea sp. ARC607]	2027922	d__Bacteria;k__norank;p__Proteobacteria;c__Gammaproteobacteria;o__Enterobacterales;f__Erwiniaceae;g__Pantoea;s__Pantoea_sp._ARC607
PB3_gene_731	318	ORM80692.1	318	317/318(99.69)	6.71e-232	universal stress protein UspE [Pantoea deleyi]	470932	d__Bacteria;k__norank;p__Proteobacteria;c__Gammaproteobacteria;o__Enterobacterales;f__Erwiniaceae;g__Pantoea;s__Pantoea_deleyi
PB3_gene_732	462	ORM80693.1	462	458/462(99.13)	0	NAD(P) transhydrogenase subunit beta [Pantoea deleyi]	470932	d__Bacteria;k__norank;p__Proteobacteria;c__Gammaproteobacteria;o__Enterobacterales;f__Erwiniaceae;g__Pantoea;s__Pantoea_deleyi
PB3_gene_734	938	WP_111139368.1	938	930/938(99.15)	0	isoleucine--tRNA ligase [Pantoea sp. ARC607]	2027922	d__Bacteria;k__norank;p__Proteobacteria;c__Gammaproteobacteria;o__Enterobacterales;f__Erwiniaceae;g__Pantoea;s__Pantoea_sp._ARC607
PB3_gene_741	153	WP_111142260.1	191	50/87(57.47)	5.89e-18	acetate uptake transporter [Pantoea sp. ARC607]	2027922	d__Bacteria;k__norank;p__Proteobacteria;c__Gammaproteobacteria;o__Enterobacterales;f__Erwiniaceae;g__Pantoea;s__Pantoea_sp._ARC607
PB3_gene_745	257	ORM83998.1	257	256/257(99.61)	1.57e-179	hypothetical protein HA42_05405 [Pantoea deleyi]	470932	d__Bacteria;k__norank;p__Proteobacteria;c__Gammaproteobacteria;o__Enterobacterales;f__Erwiniaceae;g__Pantoea;s__Pantoea_deleyi
PB3_gene_768	495	ORM85276.1	495	487/495(98.38)	0	UDP-N-acetylmuramoyl-L-alanyl-D-glutamate--2,6-diaminopimelate ligase [Pantoea	470932	d__Bacteria;k__norank;p__Proteobacteria;c__Gammaproteobacteria;o__Enterobacterales;f__Erwiniaceae;g__Pantoea;s__Pantoea_deleyi
PB3_gene_776	523	WP_111139395.1	520	519/520(99.81)	0	2-isopropylmalate synthase [Pantoea sp. ARC607]	2027922	d__Bacteria;k__norank;p__Proteobacteria;c__Gammaproteobacteria;o__Enterobacterales;f__Erwiniaceae;g__Pantoea;s__Pantoea_sp._ARC607
PB3_gene_780	65	WP_122983618.1	92	52/66(78.79)	9.65e-28	hypothetical protein [Pantoea brenneri]	None	Unclassified
PB3_gene_794	545	ORM81629.1	545	543/545(99.63)	0	ubiquinone biosynthesis regulatory protein kinase UbiB	470932	d__Bacteria;k__norank;p__Proteobacteria;c__Gammaproteobacteria;o__Enterobacterales;f__Erwiniaceae;g__Pantoea;s__Pantoea_deleyi
PB3_gene_798	424	WP_111139749.1	422	399/422(94.55)	1.43e-302	tetratricopeptide repeat protein [Pantoea sp. ARC607]	2027922	d__Bacteria;k__norank;p__Proteobacteria;c__Gammaproteobacteria;o__Enterobacterales;f__Erwiniaceae;g__Pantoea;s__Pantoea_sp._ARC607
PB3_gene_801	92	VEC01730.1	93	56/91(61.54)	2.06e-34	Uncharacterised protein [Cedecea lapagei]	158823	d__Bacteria;k__norank;p__Proteobacteria;c__Gammaproteobacteria;o__Enterobacterales;f__Enterobacteriaceae;g__Cedecea;s__Cedecea_lapagei
PB3_gene_822	269	ORM84500.1	269	268/269(99.63)	2.35e-196	DNA-formamidopyrimidine glycosylase [Pantoea deleyi]	470932	d__Bacteria;k__norank;p__Proteobacteria;c__Gammaproteobacteria;o__Enterobacterales;f__Erwiniaceae;g__Pantoea;s__Pantoea_deleyi

PB3_gene_826	406	WP_111141476.1	406	395/406(97.29)	4.17e-275	bifunctional phosphopantothenoylcysteine decarboxylase/phosphopantothenate--cysteine ligase CoaBC [Pantoea sp. ARC607]	2027922	d__Bacteria;k__norank;p__Proteobacteria;c__Gammaproteobacteria;o__Enterobacterales;f__Erwiniaceae;g__Pantoea;s__Pantoea_sp._ARC607
PB3_gene_831	394	ORM82753.1	394	393/394(99.75)	1.11e-257	nucleoside permease [Pantoea deleyi]	470932	d__Bacteria;k__norank;p__Proteobacteria;c__Gammaproteobacteria;o__Enterobacterales;f__Erwiniaceae;g__Pantoea;s__Pantoea_deleyi
PB3_gene_834	588	ORM81125.1	588	587/588(99.83)	0	succinate dehydrogenase flavoprotein subunit [Pantoea	470932	d__Bacteria;k__norank;p__Proteobacteria;c__Gammaproteobacteria;o__Enterobacterales;f__Erwiniaceae;g__Pantoea;s__Pantoea_deleyi
PB3_gene_837	431	ORM81122.1	427	427/427(100.00)	0	citrate (Si)-synthase [Pantoea deleyi]	470932	d__Bacteria;k__norank;p__Proteobacteria;c__Gammaproteobacteria;o__Enterobacterales;f__Erwiniaceae;g__Pantoea;s__Pantoea_deleyi
PB3_gene_841	242	WP_090962971.1	242	238/242(98.35)	3.26e-160	DUF969 domain-containing protein [Pantoea sp. OV426]	53335	d__Bacteria;k__norank;p__Proteobacteria;c__Gammaproteobacteria;o__Enterobacterales;f__Erwiniaceae;g__Pantoea;s__norank
PB3_gene_844	210	WP_111141015.1	218	200/210(95.24)	5.18e-143	5-oxoprolinase subunit PxpB [Pantoea sp. ARC607]	2027922	d__Bacteria;k__norank;p__Proteobacteria;c__Gammaproteobacteria;o__Enterobacterales;f__Erwiniaceae;g__Pantoea;s__Pantoea_sp._ARC607
PB3_gene_850	546	ORM80851.1	546	545/546(99.82)	0	phosphoglucomutase, alpha-D-glucose phosphate-specific [Pantoea deleyi]	470932	d__Bacteria;k__norank;p__Proteobacteria;c__Gammaproteobacteria;o__Enterobacterales;f__Erwiniaceae;g__Pantoea;s__Pantoea_deleyi
PB3_gene_857	555	ORM80844.1	555	549/555(98.92)	0	glutamine--tRNA ligase [Pantoea deleyi]	470932	d__Bacteria;k__norank;p__Proteobacteria;c__Gammaproteobacteria;o__Enterobacterales;f__Erwiniaceae;g__Pantoea;s__Pantoea_deleyi
PB3_gene_858	445	ORM80843.1	675	441/445(99.10)	3.09e-302	PTS N-acetyl glucosamine transporter subunit IIABC [Pantoea deleyi]	470932	d__Bacteria;k__norank;p__Proteobacteria;c__Gammaproteobacteria;o__Enterobacterales;f__Erwiniaceae;g__Pantoea;s__Pantoea_deleyi
PB3_gene_860	383	ORM82577.1	383	382/383(99.74)	6.37e-272	flagellar biosynthesis protein FlhB [Pantoea deleyi]	470932	d__Bacteria;k__norank;p__Proteobacteria;c__Gammaproteobacteria;o__Enterobacterales;f__Erwiniaceae;g__Pantoea;s__Pantoea_deleyi
PB3_gene_863	350	ORM82574.1	349	348/349(99.71)	3.51e-243	chemotaxis response regulator protein-glutamate methylesterase [Pantoea deleyi]	470932	d__Bacteria;k__norank;p__Proteobacteria;c__Gammaproteobacteria;o__Enterobacterales;f__Erwiniaceae;g__Pantoea;s__Pantoea_deleyi
PB3_gene_883	1261	WP_031590422.1	1276	1075/1263(85.11)	0	type VI secretion system membrane subunit TssM [Pantoea agglomerans]	549	d__Bacteria;k__norank;p__Proteobacteria;c__Gammaproteobacteria;o__Enterobacterales;f__Erwiniaceae;g__Pantoea;s__Pantoea_agglomerans
PB3_gene_891	875	WP_080707889.1	875	733/875(83.77)	0	fimbrial biogenesis outer membrane usher protein [Pantoea	549	d__Bacteria;k__norank;p__Proteobacteria;c__Gammaproteobacteria;o__Enterobacterales;f__Erwiniaceae;g__Pantoea;s__Pantoea_agglomerans
PB3_gene_892	254	WP_031590435.1	256	211/253(83.40)	1.00e-149	pilus assembly protein [Pantoea agglomerans]	549	d__Bacteria;k__norank;p__Proteobacteria;c__Gammaproteobacteria;o__Enterobacterales;f__Erwiniaceae;g__Pantoea;s__Pantoea_agglomerans
PB3_gene_895	626	WP_031590439.1	626	584/626(93.29)	0	type VI secretion system baseplate subunit TssF [Pantoea	549	d__Bacteria;k__norank;p__Proteobacteria;c__Gammaproteobacteria;o__Enterobacterales;f__Erwiniaceae;g__Pantoea;s__Pantoea_agglomerans
PB3_gene_902	515	WP_031594320.1	527	383/510(75.10)	1.06e-235	HAMP domain-containing protein [Pantoea agglomerans]	549	d__Bacteria;k__norank;p__Proteobacteria;c__Gammaproteobacteria;o__Enterobacterales;f__Erwiniaceae;g__Pantoea;s__Pantoea_agglomerans
PB3_gene_907	520	WP_039330159.1	520	448/520(86.15)	0	ABC transporter substrate-binding protein [Pantoea rodasii]	1076549	d__Bacteria;k__norank;p__Proteobacteria;c__Gammaproteobacteria;o__Enterobacterales;f__Erwiniaceae;g__Pantoea;s__Pantoea_rodasii
PB3_gene_936	418	ORM75752.1	418	412/418(98.56)	1.44e-288	hypothetical protein HA42_19980 [Pantoea deleyi]	470932	d__Bacteria;k__norank;p__Proteobacteria;c__Gammaproteobacteria;o__Enterobacterales;f__Erwiniaceae;g__Pantoea;s__Pantoea_deleyi
PB3_gene_937	552	WP_111142015.1	552	531/552(96.20)	2.80e-275	HAMP domain-containing protein [Pantoea sp. ARC607]	2027922	d__Bacteria;k__norank;p__Proteobacteria;c__Gammaproteobacteria;o__Enterobacterales;f__Erwiniaceae;g__Pantoea;s__Pantoea_sp._ARC607
PB3_gene_938	754	ORM81175.1	754	748/754(99.20)	0	catalase HPII [Pantoea deleyi]	470932	d__Bacteria;k__norank;p__Proteobacteria;c__Gammaproteobacteria;o__Enterobacterales;f__Erwiniaceae;g__Pantoea;s__Pantoea_deleyi
PB3_gene_939	216	WP_128085629.1	216	211/216(97.69)	6.01e-159	metal-binding protein ZinT [Pantoea deleyi]	470932	d__Bacteria;k__norank;p__Proteobacteria;c__Gammaproteobacteria;o__Enterobacterales;f__Erwiniaceae;g__Pantoea;s__Pantoea_deleyi
PB3_gene_953	316	ORM76466.1	316	315/316(99.68)	5.34e-237	hypothetical protein HA42_19075 [Pantoea deleyi]	470932	d__Bacteria;k__norank;p__Proteobacteria;c__Gammaproteobacteria;o__Enterobacterales;f__Erwiniaceae;g__Pantoea;s__Pantoea_deleyi

PB3_gene_961	107	ORM82938.1	107	107/107(100.00)	4.38e-72	hypothetical protein HA42_07370 [Pantoea deleyi]	470932	d__Bacteria;k__norank;p__Proteobacteria;c__Gammaproteobacteria;o__Enterobacterales;f__Erwiniaceae;g__Pantoea;s__Pantoea_deleyi
PB3_gene_962	140	ORM82937.1	140	139/140(99.29)	1.78e-98	hypothetical protein HA42_07365 [Pantoea deleyi]	470932	d__Bacteria;k__norank;p__Proteobacteria;c__Gammaproteobacteria;o__Enterobacterales;f__Erwiniaceae;g__Pantoea;s__Pantoea_deleyi
PB3_gene_976	377	SUB06766.1	377	358/377(94.96)	7.08e-266	Uncharacterized hydrolase YxeP [Pantoea agglomerans]	549	d__Bacteria;k__norank;p__Proteobacteria;c__Gammaproteobacteria;o__Enterobacterales;f__Erwiniaceae;g__Pantoea;s__Pantoea_agglomerans
PB3_gene_980	554	WP_095706961.1	554	535/554(96.57)	0	ABC transporter ATP-binding protein [Pantoea vagans]	470934	d__Bacteria;k__norank;p__Proteobacteria;c__Gammaproteobacteria;o__Enterobacterales;f__Erwiniaceae;g__Pantoea;s__Pantoea_vagans
PB3_gene_987	423	SUB08532.1	423	417/423(98.58)	3.81e-307	Aliphatic amidase expression-regulating protein [Pantoea agglomerans]	549	d__Bacteria;k__norank;p__Proteobacteria;c__Gammaproteobacteria;o__Enterobacterales;f__Erwiniaceae;g__Pantoea;s__Pantoea_agglomerans
PB3_gene_988	530	WP_111141518.1	521	505/521(96.93)	0	urea ABC transporter permease subunit UrtB [Pantoea sp.	2027922	d__Bacteria;k__norank;p__Proteobacteria;c__Gammaproteobacteria;o__Enterobacterales;f__Erwiniaceae;g__Pantoea;s__Pantoea_sp._ARC607
PB3_gene_989	357	ORM80526.1	357	353/357(98.88)	9.72e-247	urea ABC transporter permease subunit UrtC [Pantoea deleyi]	470932	d__Bacteria;k__norank;p__Proteobacteria;c__Gammaproteobacteria;o__Enterobacterales;f__Erwiniaceae;g__Pantoea;s__Pantoea_deleyi
PB3_gene_993	441	ORM80530.1	441	431/441(97.73)	8.27e-302	L-fucose:H+ symporter permease [Pantoea deleyi]	470932	d__Bacteria;k__norank;p__Proteobacteria;c__Gammaproteobacteria;o__Enterobacterales;f__Erwiniaceae;g__Pantoea;s__Pantoea_deleyi
PB3_gene_1005	226	ORM80539.1	226	221/226(97.79)	4.04e-162	noncanonical pyrimidine nucleotidase, YjjG family [Pantoea deleyi]	470932	d__Bacteria;k__norank;p__Proteobacteria;c__Gammaproteobacteria;o__Enterobacterales;f__Erwiniaceae;g__Pantoea;s__Pantoea_deleyi
PB3_gene_1008	349	ORM85230.1	317	307/317(96.85)	1.49e-220	hydroxyacid dehydrogenase [Pantoea deleyi]	470932	d__Bacteria;k__norank;p__Proteobacteria;c__Gammaproteobacteria;o__Enterobacterales;f__Erwiniaceae;g__Pantoea;s__Pantoea_deleyi
PB3_gene_1012	478	PZL99061.1	478	440/478(92.05)	8.68e-286	transcription factor [Pantoea sp. ARC607]	2027922	d__Bacteria;k__norank;p__Proteobacteria;c__Gammaproteobacteria;o__Enterobacterales;f__Erwiniaceae;g__Pantoea;s__Pantoea_sp._ARC607
PB3_gene_1020	355	SQI43369.1	354	211/355(59.44)	5.88e-154	Phage tail sheath protein [Serratia plymuthica]	82996	d__Bacteria;k__norank;p__Proteobacteria;c__Gammaproteobacteria;o__Enterobacterales;f__Yersiniaceae;g__Serratia;s__Serratia_plymuthica
PB3_gene_1022	692	WP_123346400.1	692	455/692(65.75)	0	MULTISPECIES: ATP-binding protein [Enterobacter]	2608935	d__Bacteria;k__norank;p__Proteobacteria;c__Gammaproteobacteria;o__Enterobacterales;f__Enterobacteriaceae;g__Enterobacter;s__norank
PB3_gene_1031	465	WP_084276350.1	732	354/465(76.13)	2.28e-271	integrase [Erwinia mallotivora]	None	Unclassified
PB3_gene_1032	267	WP_089517791.1	409	238/266(89.47)	5.43e-175	integrase [Pantoea ananatis]	None	Unclassified
PB3_gene_1033	615	ASN17717.1	597	558/597(93.47)	0	hypothetical protein B7764_00980 [Pantoea ananatis]	553	d__Bacteria;k__norank;p__Proteobacteria;c__Gammaproteobacteria;o__Enterobacterales;f__Erwiniaceae;g__Pantoea;s__Pantoea_ananatis
PB3_gene_1050	408	WP_111140029.1	355	350/355(98.59)	1.90e-259	AraC family transcriptional regulator [Pantoea sp. ARC607]	2027922	d__Bacteria;k__norank;p__Proteobacteria;c__Gammaproteobacteria;o__Enterobacterales;f__Erwiniaceae;g__Pantoea;s__Pantoea_sp._ARC607
PB3_gene_1053	119	ORM75490.1	119	115/119(96.64)	2.93e-78	DUF4810 domain-containing protein [Pantoea deleyi]	470932	d__Bacteria;k__norank;p__Proteobacteria;c__Gammaproteobacteria;o__Enterobacterales;f__Erwiniaceae;g__Pantoea;s__Pantoea_deleyi
PB3_gene_1055	1224	WP_111140021.1	1224	1088/1224(88.89)	0	exonuclease subunit SbcC [Pantoea sp. ARC607]	2027922	d__Bacteria;k__norank;p__Proteobacteria;c__Gammaproteobacteria;o__Enterobacterales;f__Erwiniaceae;g__Pantoea;s__Pantoea_sp._ARC607
PB3_gene_1062	450	ORM79505.1	450	446/450(99.11)	0	proline-specific permease ProY [Pantoea deleyi]	470932	d__Bacteria;k__norank;p__Proteobacteria;c__Gammaproteobacteria;o__Enterobacterales;f__Erwiniaceae;g__Pantoea;s__Pantoea_deleyi
PB3_gene_1064	168	ORM79507.1	270	159/168(94.64)	8.60e-113	inositol monophosphatase [Pantoea deleyi]	470932	d__Bacteria;k__norank;p__Proteobacteria;c__Gammaproteobacteria;o__Enterobacterales;f__Erwiniaceae;g__Pantoea;s__Pantoea_deleyi
PB3_gene_1073	449	QAV43923.1	449	437/448(97.54)	8.90e-101	cell envelope integrity protein TolA [Pantoea agglomerans]	549	d__Bacteria;k__norank;p__Proteobacteria;c__Gammaproteobacteria;o__Enterobacterales;f__Erwiniaceae;g__Pantoea;s__Pantoea_agglomerans
PB3_gene_1084	935	WP_111141774.1	935	931/935(99.57)	0	2-oxoglutarate dehydrogenase E1 component [Pantoea sp.	2027922	d__Bacteria;k__norank;p__Proteobacteria;c__Gammaproteobacteria;o__Enterobacterales;f__Erwiniaceae;g__Pantoea;s__Pantoea_sp._ARC607
PB3_gene_1096	331	ORM80745.1	331	331/331(100.00)	2.10e-237	adenosine deaminase [Pantoea deleyi]	470932	d__Bacteria;k__norank;p__Proteobacteria;c__Gammaproteobacteria;o__Enterobacterales;f__Erwiniaceae;g__Pantoea;s__Pantoea_deleyi
PB3_gene_1102	164	PAW36638.1	164	163/164(99.39)	3.39e-110	transcriptional regulator [Pantoea vagans]	470934	d__Bacteria;k__norank;p__Proteobacteria;c__Gammaproteobacteria;o__Enterobacterales;f__Erwiniaceae;g__Pantoea;s__Pantoea_vagans

PB3_gene_1103	265	ORM79161.1	265	254/265(95.85)	4.51e-183	metal-dependent hydrolase [Pantoea deleyi]	470932	d__Bacteria;k__norank;p__Proteobacteria;c__Gammaproteobacteria;o__Enterobacterales;f__Erwiniaceae;g__Pantoea;s__Pantoea_deleyi
PB3_gene_1112	786	ORM80916.1	786	769/786(97.84)	0	alpha-glucosidase [Pantoea deleyi]	470932	d__Bacteria;k__norank;p__Proteobacteria;c__Gammaproteobacteria;o__Enterobacterales;f__Erwiniaceae;g__Pantoea;s__Pantoea_deleyi
PB3_gene_1115	949	WP_111141814.1	949	920/949(96.94)	0	bifunctional diguanylate cyclase/phosphodiesterase [Pantoea sp. ARC607]	2027922	d__Bacteria;k__norank;p__Proteobacteria;c__Gammaproteobacteria;o__Enterobacterales;f__Erwiniaceae;g__Pantoea;s__Pantoea_sp._ARC607
PB3_gene_1117	160	ORM80913.1	160	160/160(100.00)	1.34e-114	hypothetical protein HA42_12500 [Pantoea deleyi]	470932	d__Bacteria;k__norank;p__Proteobacteria;c__Gammaproteobacteria;o__Enterobacterales;f__Erwiniaceae;g__Pantoea;s__Pantoea_deleyi
PB3_gene_1118	460	ORM80912.1	467	454/460(98.70)	0	GntR family transcriptional regulator [Pantoea deleyi]	470932	d__Bacteria;k__norank;p__Proteobacteria;c__Gammaproteobacteria;o__Enterobacterales;f__Erwiniaceae;g__Pantoea;s__Pantoea_deleyi
PB3_gene_1127	479	WP_111138844.1	471	435/478(91.00)	5.97e-287	MFS transporter [Pantoea sp. ARC607]	2027922	d__Bacteria;k__norank;p__Proteobacteria;c__Gammaproteobacteria;o__Enterobacterales;f__Erwiniaceae;g__Pantoea;s__Pantoea_sp._ARC607
PB3_gene_1128	460	ORM86848.1	477	441/460(95.87)	0	16S rRNA (cytosine(1407)-C(5))-methyltransferase RsmF [Pantoea deleyi]	470932	d__Bacteria;k__norank;p__Proteobacteria;c__Gammaproteobacteria;o__Enterobacterales;f__Erwiniaceae;g__Pantoea;s__Pantoea_deleyi
PB3_gene_1135	446	WP_111139201.1	446	442/446(99.10)	1.39e-307	lipopolysaccharide biosynthesis protein [Pantoea sp. ARC607]	2027922	d__Bacteria;k__norank;p__Proteobacteria;c__Gammaproteobacteria;o__Enterobacterales;f__Erwiniaceae;g__Pantoea;s__Pantoea_sp._ARC607
PB3_gene_1136	407	SJZ62686.1	407	401/407(98.53)	3.53e-288	colanic acid/amylovoran biosynthesis glycosyltransferase [Pantoea agglomerans]	470933	d__Bacteria;k__norank;p__Proteobacteria;c__Gammaproteobacteria;o__Enterobacterales;f__Erwiniaceae;g__Pantoea;s__Pantoea_eucalypti
PB3_gene_1138	738	WP_111139198.1	738	717/738(97.15)	0	amylovoran biosynthesis protein AmsF [Pantoea sp. ARC607]	2027922	d__Bacteria;k__norank;p__Proteobacteria;c__Gammaproteobacteria;o__Enterobacterales;f__Erwiniaceae;g__Pantoea;s__Pantoea_sp._ARC607
PB3_gene_1140	250	OQV41750.1	250	249/250(99.60)	3.33e-182	hypothetical protein BZ160_08140 [Pantoea vagans]	470934	d__Bacteria;k__norank;p__Proteobacteria;c__Gammaproteobacteria;o__Enterobacterales;f__Erwiniaceae;g__Pantoea;s__Pantoea_vagans
PB3_gene_1142	378	WP_111139196.1	378	377/378(99.74)	5.87e-270	EpsG family protein [Pantoea sp. ARC607]	2027922	d__Bacteria;k__norank;p__Proteobacteria;c__Gammaproteobacteria;o__Enterobacterales;f__Erwiniaceae;g__Pantoea;s__Pantoea_sp._ARC607
PB3_gene_1143	727	ORM85628.1	727	721/727(99.17)	0	tyrosine-protein kinase [Pantoea deleyi]	470932	d__Bacteria;k__norank;p__Proteobacteria;c__Gammaproteobacteria;o__Enterobacterales;f__Erwiniaceae;g__Pantoea;s__Pantoea_deleyi
PB3_gene_1144	144	ORM85627.1	144	142/144(98.61)	1.90e-95	protein tyrosine phosphatase [Pantoea deleyi]	470932	d__Bacteria;k__norank;p__Proteobacteria;c__Gammaproteobacteria;o__Enterobacterales;f__Erwiniaceae;g__Pantoea;s__Pantoea_deleyi
PB3_gene_1147	523	ORM85624.1	523	518/523(99.04)	0	hypothetical protein HA42_02130 [Pantoea deleyi]	470932	d__Bacteria;k__norank;p__Proteobacteria;c__Gammaproteobacteria;o__Enterobacterales;f__Erwiniaceae;g__Pantoea;s__Pantoea_deleyi
PB3_gene_1148	457	ORM85623.1	606	437/457(95.62)	1.64e-302	outer membrane assembly protein AsmA [Pantoea deleyi]	470932	d__Bacteria;k__norank;p__Proteobacteria;c__Gammaproteobacteria;o__Enterobacterales;f__Erwiniaceae;g__Pantoea;s__Pantoea_deleyi
PB3_gene_1159	403	PXW20400.1	403	400/403(99.26)	4.92e-302	acetylornithine deacetylase [Pantoea sp. JKS000250]	1938795	d__Bacteria;k__norank;p__Proteobacteria;c__Gammaproteobacteria;o__Enterobacterales;f__Erwiniaceae;g__Pantoea;s__Pantoea_sp._JKS000250
PB3_gene_1173	259	WP_111140743.1	259	243/259(93.82)	1.84e-179	siderophore-interacting protein [Pantoea sp. ARC607]	2027922	d__Bacteria;k__norank;p__Proteobacteria;c__Gammaproteobacteria;o__Enterobacterales;f__Erwiniaceae;g__Pantoea;s__Pantoea_sp._ARC607
PB3_gene_1176	790	WP_061063325.1	790	689/790(87.22)	0	TonB-dependent receptor [Pantoea vagans]	470934	d__Bacteria;k__norank;p__Proteobacteria;c__Gammaproteobacteria;o__Enterobacterales;f__Erwiniaceae;g__Pantoea;s__Pantoea_vagans
PB3_gene_1178	273	WP_122889632.1	273	251/273(91.94)	7.66e-163	hemin ABC transporter substrate-binding protein [[Curtobacterium] plantarum]	53335	d__Bacteria;k__norank;p__Proteobacteria;c__Gammaproteobacteria;o__Enterobacterales;f__Erwiniaceae;g__Pantoea;s__norank
PB3_gene_1183	717	WP_111140738.1	717	700/717(97.63)	0	ornithine decarboxylase [Pantoea sp. ARC607]	2027922	d__Bacteria;k__norank;p__Proteobacteria;c__Gammaproteobacteria;o__Enterobacterales;f__Erwiniaceae;g__Pantoea;s__Pantoea_sp._ARC607
PB3_gene_1191	439	ORM81588.1	439	411/439(93.62)	1.22e-286	anibiotic ABC transporter [Pantoea deleyi]	470932	d__Bacteria;k__norank;p__Proteobacteria;c__Gammaproteobacteria;o__Enterobacterales;f__Erwiniaceae;g__Pantoea;s__Pantoea_deleyi
PB3_gene_1193	245	ORM81590.1	245	219/245(89.39)	6.38e-148	fimbrial chaperone protein [Pantoea deleyi]	470932	d__Bacteria;k__norank;p__Proteobacteria;c__Gammaproteobacteria;o__Enterobacterales;f__Erwiniaceae;g__Pantoea;s__Pantoea_deleyi

PB3_gene_1194	447	WP_111138281.1	452	392/448(87.50)	5.97e-274	fimbrial protein [Pantoea sp. ARC607]	None	Unclassified
PB3_gene_1195	861	ORM81591.1	861	809/861(93.96)	0	fimbrial assembly protein [Pantoea deleyi]	470932	d__Bacteria;k__norank;p__Proteobacteria;c__Gammaproteobacteria;o__Enterobacterales;f__Erwiniaceae;g__Pantoea;s__Pantoea_deleyi
PB3_gene_1196	279	ORM81592.1	245	216/245(88.16)	3.14e-150	fimbrial chaperone protein StbB [Pantoea deleyi]	470932	d__Bacteria;k__norank;p__Proteobacteria;c__Gammaproteobacteria;o__Enterobacterales;f__Erwiniaceae;g__Pantoea;s__Pantoea_deleyi
PB3_gene_1204	335	ORM75484.1	335	330/335(98.51)	3.28e-252	endoglucanase [Pantoea deleyi]	470932	d__Bacteria;k__norank;p__Proteobacteria;c__Gammaproteobacteria;o__Enterobacterales;f__Erwiniaceae;g__Pantoea;s__Pantoea_deleyi
PB3_gene_1205	553	WP_111139435.1	553	536/553(96.93)	0	cellulose biosynthesis protein BcsG [Pantoea sp. ARC607]	2027922	d__Bacteria;k__norank;p__Proteobacteria;c__Gammaproteobacteria;o__Enterobacterales;f__Erwiniaceae;g__Pantoea;s__Pantoea_sp._ARC607
PB3_gene_1210	860	WP_111139440.1	860	839/860(97.56)	0	UDP-forming cellulose synthase catalytic subunit [Pantoea sp. ARC607]	2027922	d__Bacteria;k__norank;p__Proteobacteria;c__Gammaproteobacteria;o__Enterobacterales;f__Erwiniaceae;g__Pantoea;s__Pantoea_sp._ARC607
PB3_gene_1212	1157	WP_111139442.1	1155	1096/1155(94.89)	0	cellulose biosynthesis protein BcsC [Pantoea sp. ARC607]	2027922	d__Bacteria;k__norank;p__Proteobacteria;c__Gammaproteobacteria;o__Enterobacterales;f__Erwiniaceae;g__Pantoea;s__Pantoea_sp._ARC607
PB3_gene_1217	344	ORM77370.1	342	337/342(98.54)	4.71e-185	efflux transporter periplasmic adaptor subunit [Pantoea deleyi]	470932	d__Bacteria;k__norank;p__Proteobacteria;c__Gammaproteobacteria;o__Enterobacterales;f__Erwiniaceae;g__Pantoea;s__Pantoea_deleyi
PB3_gene_1233	1125	ORM86587.1	1125	1102/1125(97.96)	0	exodeoxyribonuclease V subunit gamma [Pantoea deleyi]	470932	d__Bacteria;k__norank;p__Proteobacteria;c__Gammaproteobacteria;o__Enterobacterales;f__Erwiniaceae;g__Pantoea;s__Pantoea_deleyi
PB3_gene_1247	418	WP_111141756.1	418	411/417(98.56)	1.31e-300	diaminopimelate decarboxylase [Pantoea sp. ARC607]	2027922	d__Bacteria;k__norank;p__Proteobacteria;c__Gammaproteobacteria;o__Enterobacterales;f__Erwiniaceae;g__Pantoea;s__Pantoea_sp._ARC607
PB3_gene_1248	130	SAY68478.1	336	55/128(42.97)	7.29e-21	hypothetical protein KQS06HV_100342 [Klebsiella quasipneumoniae subsp. similipneumoniae]	1463164	d__Bacteria;k__norank;p__Proteobacteria;c__Gammaproteobacteria;o__Enterobacterales;f__Enterobacteriaceae;g__Klebsiella;s__Klebsiella_quasipneumoniae
PB3_gene_1275	426	ORM77971.1	427	412/424(97.17)	1.21e-298	methionine gamma-lyase [Pantoea deleyi]	470932	d__Bacteria;k__norank;p__Proteobacteria;c__Gammaproteobacteria;o__Enterobacterales;f__Erwiniaceae;g__Pantoea;s__Pantoea_deleyi
PB3_gene_1280	352	ORM77975.1	337	333/337(98.81)	6.12e-237	acyltransferase [Pantoea deleyi]	470932	d__Bacteria;k__norank;p__Proteobacteria;c__Gammaproteobacteria;o__Enterobacterales;f__Erwiniaceae;g__Pantoea;s__Pantoea_deleyi
PB3_gene_1285	443	WP_111140283.1	479	440/443(99.32)	0	polynucleotide adenylyltransferase PcnB	2027922	d__Bacteria;k__norank;p__Proteobacteria;c__Gammaproteobacteria;o__Enterobacterales;f__Erwiniaceae;g__Pantoea;s__Pantoea_sp._ARC607
PB3_gene_1290	827	WP_111140278.1	827	799/827(96.61)	0	ATP-dependent helicase HrpB [Pantoea sp. ARC607]	2027922	d__Bacteria;k__norank;p__Proteobacteria;c__Gammaproteobacteria;o__Enterobacterales;f__Erwiniaceae;g__Pantoea;s__Pantoea_sp._ARC607
PB3_gene_1310	433	WP_111140717.1	433	414/433(95.61)	5.29e-213	murein hydrolase activator EnvC [Pantoea sp. ARC607]	2027922	d__Bacteria;k__norank;p__Proteobacteria;c__Gammaproteobacteria;o__Enterobacterales;f__Erwiniaceae;g__Pantoea;s__Pantoea_sp._ARC607
PB3_gene_1315	153	WP_034917751.1	153	124/151(82.12)	1.57e-87	(2Fe-2S)-binding protein [Erwinia sp. 9145]	1500895	d__Bacteria;k__norank;p__Proteobacteria;c__Gammaproteobacteria;o__Enterobacterales;f__Erwiniaceae;g__Erwinia;s__Erwinia_sp._9145
PB3_gene_1321	461	WP_125289962.1	462	375/458(81.88)	1.40e-285	dihydropyrimidinase [Erwinia sp. 198]	2022746	d__Bacteria;k__norank;p__Proteobacteria;c__Gammaproteobacteria;o__Enterobacterales;f__Erwiniaceae;g__Erwinia;s__Erwinia_sp._198
PB3_gene_1335	660	WP_111140384.1	660	653/660(98.94)	0	glycogen debranching protein GlgX [Pantoea sp. ARC607]	2027922	d__Bacteria;k__norank;p__Proteobacteria;c__Gammaproteobacteria;o__Enterobacterales;f__Erwiniaceae;g__Pantoea;s__Pantoea_sp._ARC607
PB3_gene_1342	193	ORM77356.1	193	193/193(100.00)	1.17e-122	electron transport complex subunit RsxA [Pantoea deleyi]	470932	d__Bacteria;k__norank;p__Proteobacteria;c__Gammaproteobacteria;o__Enterobacterales;f__Erwiniaceae;g__Pantoea;s__Pantoea_deleyi
PB3_gene_1344	796	WP_107319637.1	765	720/796(90.45)	0	electron transport complex subunit RsxC [Pantoea vagans]	470934	d__Bacteria;k__norank;p__Proteobacteria;c__Gammaproteobacteria;o__Enterobacterales;f__Erwiniaceae;g__Pantoea;s__Pantoea_vagans
PB3_gene_1347	234	ORM81155.1	234	234/234(100.00)	2.37e-149	electron transport complex subunit RsxE [Pantoea deleyi]	470932	d__Bacteria;k__norank;p__Proteobacteria;c__Gammaproteobacteria;o__Enterobacterales;f__Erwiniaceae;g__Pantoea;s__Pantoea_deleyi
PB3_gene_1353	286	ADO09417.1	286	279/286(97.55)	2.98e-203	pyridoxal kinase 2 / pyridoxine kinase [Pantoea vagans C9-1]	712898	d__Bacteria;k__norank;p__Proteobacteria;c__Gammaproteobacteria;o__Enterobacterales;f__Erwiniaceae;g__Pantoea;s__Pantoea_vagans
PB3_gene_1364	885	WP_111142176.1	885	871/885(98.42)	0	GNAT family N-acetyltransferase [Pantoea sp. ARC607]	2027922	d__Bacteria;k__norank;p__Proteobacteria;c__Gammaproteobacteria;o__Enterobacterales;f__Erwiniaceae;g__Pantoea;s__Pantoea_sp._ARC607

PB3_gene_1365	225	ORM76624.1	225	220/225(97.78)	1.15e-156	DTW domain-containing protein [Pantoea deleyi]	470932	d__Bacteria;k__norank;p__Proteobacteria;c__Gammaproteobacteria;o__Enterobacterales;f__Erwiniaceae;g__Pantoea;s__Pantoea_deleyi
PB3_gene_1376	723	WP_111138265.1	1013	664/723(91.84)	0	efflux RND transporter permease subunit [Pantoea sp. ARC607]	2027922	d__Bacteria;k__norank;p__Proteobacteria;c__Gammaproteobacteria;o__Enterobacterales;f__Erwiniaceae;g__Pantoea;s__Pantoea_sp._ARC607
PB3_gene_1384	845	ORM85239.1	836	809/833(97.12)	0	excinuclease ABC subunit A [Pantoea deleyi]	470932	d__Bacteria;k__norank;p__Proteobacteria;c__Gammaproteobacteria;o__Enterobacterales;f__Erwiniaceae;g__Pantoea;s__Pantoea_deleyi
PB3_gene_1385	1411	WP_123350196.1	701	358/760(47.11)	1.58e-221	MULTISPECIES: hypothetical protein [Enterobacter]	2608935	d__Bacteria;k__norank;p__Proteobacteria;c__Gammaproteobacteria;o__Enterobacterales;f__Enterobacteriaceae;g__Enterobacter;s__norank
PB3_gene_1394	533	ORM82837.1	533	521/532(97.93)	0	MFS transporter [Pantoea deleyi]	470932	d__Bacteria;k__norank;p__Proteobacteria;c__Gammaproteobacteria;o__Enterobacterales;f__Erwiniaceae;g__Pantoea;s__Pantoea_deleyi
PB3_gene_1405	67	OQV43305.1	310	66/67(98.51)	9.99e-34	malonyl CoA-acyl carrier protein transacylase [Pantoea vagans]	470934	d__Bacteria;k__norank;p__Proteobacteria;c__Gammaproteobacteria;o__Enterobacterales;f__Erwiniaceae;g__Pantoea;s__Pantoea_vagans
PB3_gene_1416	709	ORM83869.1	709	704/709(99.29)	0	phosphate acetyltransferase [Pantoea deleyi]	470932	d__Bacteria;k__norank;p__Proteobacteria;c__Gammaproteobacteria;o__Enterobacterales;f__Erwiniaceae;g__Pantoea;s__Pantoea_deleyi
PB3_gene_1425	5681	WP_095706861.1	6003	4513/5680(79.45)	0	BapA prefix-like domain-containing protein [Pantoea	470934	d__Bacteria;k__norank;p__Proteobacteria;c__Gammaproteobacteria;o__Enterobacterales;f__Erwiniaceae;g__Pantoea;s__Pantoea_vagans
PB3_gene_1427	328	WP_111142121.1	342	317/328(96.65)	3.75e-226	zinc-binding alcohol dehydrogenase family protein	2027922	d__Bacteria;k__norank;p__Proteobacteria;c__Gammaproteobacteria;o__Enterobacterales;f__Erwiniaceae;g__Pantoea;s__Pantoea_sp._ARC607
PB3_gene_1446	498	ORM78500.1	498	495/498(99.40)	0	amino acid permease [Pantoea deleyi]	470932	d__Bacteria;k__norank;p__Proteobacteria;c__Gammaproteobacteria;o__Enterobacterales;f__Erwiniaceae;g__Pantoea;s__Pantoea_deleyi
PB3_gene_1462	664	ORM86543.1	664	656/664(98.80)	0	transketolase [Pantoea deleyi]	470932	d__Bacteria;k__norank;p__Proteobacteria;c__Gammaproteobacteria;o__Enterobacterales;f__Erwiniaceae;g__Pantoea;s__Pantoea_deleyi
PB3_gene_1475	285	ORM83980.1	285	280/285(98.25)	1.68e-213	bis(5'-nucleosyl)-tetraphosphatase (symmetrical) [Pantoea deleyi]	470932	d__Bacteria;k__norank;p__Proteobacteria;c__Gammaproteobacteria;o__Enterobacterales;f__Erwiniaceae;g__Pantoea;s__Pantoea_deleyi
PB3_gene_1485	659	WP_111140292.1	659	629/658(95.59)	0	Fe(3+)-hydroxamate ABC transporter permease FhuB [Pantoea sp. ARC607]	2027922	d__Bacteria;k__norank;p__Proteobacteria;c__Gammaproteobacteria;o__Enterobacterales;f__Erwiniaceae;g__Pantoea;s__Pantoea_sp._ARC607
PB3_gene_1492	477	ORM83023.1	477	456/477(95.60)	1.93e-300	serine endoprotease [Pantoea deleyi]	470932	d__Bacteria;k__norank;p__Proteobacteria;c__Gammaproteobacteria;o__Enterobacterales;f__Erwiniaceae;g__Pantoea;s__Pantoea_deleyi
PB3_gene_1493	385	ORM83024.1	385	379/385(98.44)	3.79e-265	carbohydrate diacid regulon transcriptional regulator CdaR [Pantoea deleyi]	470932	d__Bacteria;k__norank;p__Proteobacteria;c__Gammaproteobacteria;o__Enterobacterales;f__Erwiniaceae;g__Pantoea;s__Pantoea_deleyi
PB3_gene_1496	892	WP_111140265.1	884	868/884(98.19)	0	bifunctional uridylyltransferase/uridylyl-removing protein GlnD [Pantoea sp. ARC607]	2027922	d__Bacteria;k__norank;p__Proteobacteria;c__Gammaproteobacteria;o__Enterobacterales;f__Erwiniaceae;g__Pantoea;s__Pantoea_sp._ARC607
PB3_gene_1497	263	SJZ80004.1	264	261/263(99.24)	6.71e-191	methionyl aminopeptidase [Pantoea agglomerans]	470933	d__Bacteria;k__norank;p__Proteobacteria;c__Gammaproteobacteria;o__Enterobacterales;f__Erwiniaceae;g__Pantoea;s__Pantoea_eucalypti
PB3_gene_1515	221	RAH33956.1	219	209/219(95.43)	1.47e-152	fumarylacetoacetate hydrolase family protein [Pantoea	549	d__Bacteria;k__norank;p__Proteobacteria;c__Gammaproteobacteria;o__Enterobacterales;f__Erwiniaceae;g__Pantoea;s__Pantoea_agglomerans
PB3_gene_1517	187	WP_111138890.1	176	170/176(96.59)	1.50e-124	disulfide bond formation protein DsbB [Pantoea sp. ARC607]	2027922	d__Bacteria;k__norank;p__Proteobacteria;c__Gammaproteobacteria;o__Enterobacterales;f__Erwiniaceae;g__Pantoea;s__Pantoea_sp._ARC607
PB3_gene_1528	650	ORM83375.1	650	647/650(99.54)	0	p-hydroxybenzoic acid efflux pump subunit AaeB [Pantoea	470932	d__Bacteria;k__norank;p__Proteobacteria;c__Gammaproteobacteria;o__Enterobacterales;f__Erwiniaceae;g__Pantoea;s__Pantoea_deleyi
PB3_gene_1529	127	SAL70451.1	152	68/124(54.84)	2.26e-28	hypothetical protein AWB65_06919 [Caballeronia	326474	d__Bacteria;k__norank;p__Proteobacteria;c__Betaproteobacteria;o__Burkholderiales;f__Burkholderiaceae;g__Caballeronia;s__Caballeronia_humi
PB3_gene_1536	657	ORM84891.1	651	613/652(94.02)	0	catecholate siderophore receptor CirA [Pantoea deleyi]	470932	d__Bacteria;k__norank;p__Proteobacteria;c__Gammaproteobacteria;o__Enterobacterales;f__Erwiniaceae;g__Pantoea;s__Pantoea_deleyi

PB3_gene_1537	381	WP_111140628.1	383	362/383(94.52)	3.57e-257	iron ABC transporter substrate-binding protein [Pantoea sp. ARC607]	2027922	d__Bacteria;k__norank;p__Proteobacteria;c__Gammaproteobacteria;o__Enterobacterales;f__Erwiniaceae;g__Pantoea;s__Pantoea_sp._ARC607
PB3_gene_1538	276	PZL86319.1	276	271/276(98.19)	9.15e-193	DeoR/GlpR transcriptional regulator [Pantoea sp. ARC270]	2027923	d__Bacteria;k__norank;p__Proteobacteria;c__Gammaproteobacteria;o__Enterobacterales;f__Erwiniaceae;g__Pantoea;s__Pantoea_sp._ARC270
PB3_gene_1539	327	WP_111140630.1	327	323/327(98.78)	3.46e-234	autoinducer 2 ABC transporter substrate-binding protein [Pantoea sp. ARC607]	2027922	d__Bacteria;k__norank;p__Proteobacteria;c__Gammaproteobacteria;o__Enterobacterales;f__Erwiniaceae;g__Pantoea;s__Pantoea_sp._ARC607
PB3_gene_1551	365	ORM85820.1	365	358/365(98.08)	4.23e-261	ABC transporter substrate-binding protein [Pantoea deleyi]	470932	d__Bacteria;k__norank;p__Proteobacteria;c__Gammaproteobacteria;o__Enterobacterales;f__Erwiniaceae;g__Pantoea;s__Pantoea_deleyi
PB3_gene_1563	315	ORM85810.1	321	315/315(100.00)	1.40e-233	lipoyl synthase [Pantoea deleyi]	470932	d__Bacteria;k__norank;p__Proteobacteria;c__Gammaproteobacteria;o__Enterobacterales;f__Erwiniaceae;g__Pantoea;s__Pantoea_deleyi
PB3_gene_1578	248	ORM78254.1	256	248/248(100.00)	4.28e-179	hypothetical protein HA42_16930 [Pantoea deleyi]	470932	d__Bacteria;k__norank;p__Proteobacteria;c__Gammaproteobacteria;o__Enterobacterales;f__Erwiniaceae;g__Pantoea;s__Pantoea_deleyi
PB3_gene_1580	65	ORM85294.1	461	63/65(96.92)	1.47e-33	type II secretion system protein GspE [Pantoea deleyi]	470932	d__Bacteria;k__norank;p__Proteobacteria;c__Gammaproteobacteria;o__Enterobacterales;f__Erwiniaceae;g__Pantoea;s__Pantoea_deleyi
PB3_gene_1584	284	WP_111139425.1	284	283/284(99.65)	6.73e-199	beta-lactamase regulator AmpE [Pantoea sp. ARC607]	2027922	d__Bacteria;k__norank;p__Proteobacteria;c__Gammaproteobacteria;o__Enterobacterales;f__Erwiniaceae;g__Pantoea;s__Pantoea_sp._ARC607
PB3_gene_1591	888	ORM85304.1	888	887/888(99.89)	0	pyruvate dehydrogenase (acetyl-transferring), homodimeric type [Pantoea deleyi]	470932	d__Bacteria;k__norank;p__Proteobacteria;c__Gammaproteobacteria;o__Enterobacterales;f__Erwiniaceae;g__Pantoea;s__Pantoea_deleyi
PB3_gene_1592	635	ORM85305.1	635	604/635(95.12)	0	pyruvate dehydrogenase complex dihydrolipoyllysine-residue acetyltransferase [Pantoea deleyi]	470932	d__Bacteria;k__norank;p__Proteobacteria;c__Gammaproteobacteria;o__Enterobacterales;f__Erwiniaceae;g__Pantoea;s__Pantoea_deleyi
PB3_gene_1600	140	WP_061492465.1	140	103/140(73.57)	7.53e-66	hypothetical protein [Enterobacter sp. FY-07]	None	Unclassified
PB3_gene_1603	966	WP_123346408.1	968	627/973(64.44)	0	MULTISPECIES: hypothetical protein [Enterobacter]	2608935	d__Bacteria;k__norank;p__Proteobacteria;c__Gammaproteobacteria;o__Enterobacterales;f__Enterobacteriaceae;g__Enterobacter;s__norank
PB3_gene_1604	334	EYU13881.1	425	162/425(38.12)	2.13e-74	phage tail collar family protein [Photorhabdus luminescens BA1]	1393736	d__Bacteria;k__norank;p__Proteobacteria;c__Gammaproteobacteria;o__Enterobacterales;f__Morganellaceae;g__Photorhabdus;s__Photorhabdus_luminescens
PB3_gene_1605	467	RKS61139.1	494	205/502(40.84)	1.93e-113	hypothetical protein C8E17_0249 [Serratia plymuthica]	82996	d__Bacteria;k__norank;p__Proteobacteria;c__Gammaproteobacteria;o__Enterobacterales;f__Yersiniaceae;g__Serratia;s__Serratia_plymuthica
PB3_gene_1610	159	WP_111140323.1	159	156/159(98.11)	1.19e-103	Cys-tRNA(Pro)/Cys-tRNA(Cys) deacylase YbaK [Pantoea sp. ARC607]	2027922	d__Bacteria;k__norank;p__Proteobacteria;c__Gammaproteobacteria;o__Enterobacterales;f__Erwiniaceae;g__Pantoea;s__Pantoea_sp._ARC607
PB3_gene_1612	143	WP_111140321.1	144	142/143(99.30)	3.27e-98	multidrug/biocide efflux PACE transporter [Pantoea sp. ARC607]	2027922	d__Bacteria;k__norank;p__Proteobacteria;c__Gammaproteobacteria;o__Enterobacterales;f__Erwiniaceae;g__Pantoea;s__Pantoea_sp._ARC607
PB3_gene_1613	574	WP_111140320.1	588	557/574(97.04)	0	bifunctional UDP-sugar hydrolase/5'-nucleotidase [Pantoea sp. ARC607]	2027922	d__Bacteria;k__norank;p__Proteobacteria;c__Gammaproteobacteria;o__Enterobacterales;f__Erwiniaceae;g__Pantoea;s__Pantoea_sp._ARC607
PB3_gene_1618	291	WP_111140315.1	291	266/290(91.72)	1.21e-184	hypothetical protein [Pantoea sp. ARC607]	2027922	d__Bacteria;k__norank;p__Proteobacteria;c__Gammaproteobacteria;o__Enterobacterales;f__Erwiniaceae;g__Pantoea;s__Pantoea_sp._ARC607
PB3_gene_1631	263	WP_033733218.1	263	258/263(98.10)	8.12e-185	ABC transporter substrate-binding protein [Pantoea vagans]	470934	d__Bacteria;k__norank;p__Proteobacteria;c__Gammaproteobacteria;o__Enterobacterales;f__Erwiniaceae;g__Pantoea;s__Pantoea_vagans
PB3_gene_1633	469	WP_111141976.1	469	464/469(98.93)	0	NADP-dependent phosphogluconate dehydrogenase [Pantoea sp. ARC607]	2027922	d__Bacteria;k__norank;p__Proteobacteria;c__Gammaproteobacteria;o__Enterobacterales;f__Erwiniaceae;g__Pantoea;s__Pantoea_sp._ARC607
PB3_gene_1634	445	WP_131164458.1	444	255/442(57.69)	1.21e-186	glycosyltransferase [Serratia marcescens]	615	d__Bacteria;k__norank;p__Proteobacteria;c__Gammaproteobacteria;o__Enterobacterales;f__Yersiniaceae;g__Serratia;s__Serratia_marcescens

PB3_gene_1635	311	TBU70085.1	312	237/308(76.95)	1.45e-177	NAD-dependent epimerase/dehydratase family protein [Serratia marcescens]	615	d__Bacteria;k__norank;p__Proteobacteria;c__Gammaproteobacteria;o__Enterobacterales;f__Yersiniaceae;g__Serratia;s__Serratia_marcescens
PB3_gene_1636	443	TBU70086.1	452	343/443(77.43)	5.50e-255	glycosyltransferase [Serratia marcescens]	615	d__Bacteria;k__norank;p__Proteobacteria;c__Gammaproteobacteria;o__Enterobacterales;f__Yersiniaceae;g__Serratia;s__Serratia_marcescens
PB3_gene_1637	241	WP_103283985.1	474	177/235(75.32)	9.49e-121	MULTISPECIES: glycosyltransferase [Citrobacter	None	Unclassified
PB3_gene_1638	111	RNA75064.1	111	111/111(100.00)	6.35e-76	elongation factor Tu, partial [[Curtobacterium] plantarum]	221276	d__Bacteria;k__norank;p__Proteobacteria;c__Gammaproteobacteria;o__Enterobacterales;f__Erwiniaceae;g__Pantoea;s__[Curtobacterium]_plantarum
PB3_gene_1639	704	ORM53360.1	704	702/704(99.72)	0	translation elongation factor G [Pantoea brenneri]	472694	d__Bacteria;k__norank;p__Proteobacteria;c__Gammaproteobacteria;o__Enterobacterales;f__Erwiniaceae;g__Pantoea;s__Pantoea_brenneri
PB3_gene_1652	634	WP_111139701.1	634	630/634(99.37)	0	ABC transporter ATP-binding protein [Pantoea sp. ARC607]	2027922	d__Bacteria;k__norank;p__Proteobacteria;c__Gammaproteobacteria;o__Enterobacterales;f__Erwiniaceae;g__Pantoea;s__Pantoea_sp._ARC607
PB3_gene_1655	238	ORM81823.1	255	236/238(99.16)	1.19e-164	taurine transporter ATP-binding subunit [Pantoea deleyi]	470932	d__Bacteria;k__norank;p__Proteobacteria;c__Gammaproteobacteria;o__Enterobacterales;f__Erwiniaceae;g__Pantoea;s__Pantoea_deleyi
PB3_gene_1662	335	ORM86011.1	335	330/335(98.51)	6.02e-239	ornithine carbamoyltransferase [Pantoea deleyi]	470932	d__Bacteria;k__norank;p__Proteobacteria;c__Gammaproteobacteria;o__Enterobacterales;f__Erwiniaceae;g__Pantoea;s__Pantoea_deleyi
PB3_gene_1666	396	ORM86007.1	396	385/396(97.22)	2.38e-253	lysophospholipid transporter LplT [Pantoea deleyi]	470932	d__Bacteria;k__norank;p__Proteobacteria;c__Gammaproteobacteria;o__Enterobacterales;f__Erwiniaceae;g__Pantoea;s__Pantoea_deleyi
PB3_gene_1668	592	ORM77858.1	579	578/579(99.83)	0	peptidoglycan synthase [Pantoea deleyi]	470932	d__Bacteria;k__norank;p__Proteobacteria;c__Gammaproteobacteria;o__Enterobacterales;f__Erwiniaceae;g__Pantoea;s__Pantoea_deleyi
PB3_gene_1677	775	WP_111139147.1	775	737/775(95.10)	0	peptidoglycan glycosyltransferase PbpC [Pantoea sp. ARC607]	2027922	d__Bacteria;k__norank;p__Proteobacteria;c__Gammaproteobacteria;o__Enterobacterales;f__Erwiniaceae;g__Pantoea;s__Pantoea_sp._ARC607
PB3_gene_1678	1240	WP_111139146.1	1631	1179/1240(95.08)	0	alpha-2-macroglobulin family protein [Pantoea sp. ARC607]	2027922	d__Bacteria;k__norank;p__Proteobacteria;c__Gammaproteobacteria;o__Enterobacterales;f__Erwiniaceae;g__Pantoea;s__Pantoea_sp._ARC607
PB3_gene_1691	482	ORM83155.1	482	478/482(99.17)	0	tRNA 4-thiouridine(8) synthase ThiI [Pantoea deleyi]	470932	d__Bacteria;k__norank;p__Proteobacteria;c__Gammaproteobacteria;o__Enterobacterales;f__Erwiniaceae;g__Pantoea;s__Pantoea_deleyi
PB3_gene_1695	154	ORM83151.1	454	153/154(99.35)	1.76e-98	hypothetical protein HA42_06920 [Pantoea deleyi]	470932	d__Bacteria;k__norank;p__Proteobacteria;c__Gammaproteobacteria;o__Enterobacterales;f__Erwiniaceae;g__Pantoea;s__Pantoea_deleyi
PB3_gene_1708	349	ORM81040.1	349	347/349(99.43)	4.04e-260	hypothetical protein HA42_12040 [Pantoea deleyi]	470932	d__Bacteria;k__norank;p__Proteobacteria;c__Gammaproteobacteria;o__Enterobacterales;f__Erwiniaceae;g__Pantoea;s__Pantoea_deleyi
PB3_gene_1709	367	PXW21874.1	328	316/328(96.34)	2.28e-236	KDO2-lipid IV(A) lauroyltransferase [Pantoea sp.	1938795	d__Bacteria;k__norank;p__Proteobacteria;c__Gammaproteobacteria;o__Enterobacterales;f__Erwiniaceae;g__Pantoea;s__Pantoea_sp._JKS000250
PB3_gene_1717	680	ORM82635.1	680	678/680(99.71)	0	oligopeptidase A [Pantoea deleyi]	470932	d__Bacteria;k__norank;p__Proteobacteria;c__Gammaproteobacteria;o__Enterobacterales;f__Erwiniaceae;g__Pantoea;s__Pantoea_deleyi
PB3_gene_1731	338	ORM80807.1	338	333/338(98.52)	1.65e-233	transcriptional regulator [Pantoea deleyi]	470932	d__Bacteria;k__norank;p__Proteobacteria;c__Gammaproteobacteria;o__Enterobacterales;f__Erwiniaceae;g__Pantoea;s__Pantoea_deleyi
PB3_gene_1734	292	ORM80810.1	292	286/292(97.95)	4.42e-208	sugar-phosphatase [Pantoea deleyi]	470932	d__Bacteria;k__norank;p__Proteobacteria;c__Gammaproteobacteria;o__Enterobacterales;f__Erwiniaceae;g__Pantoea;s__Pantoea_deleyi
PB3_gene_1736	349	ORM80812.1	349	338/349(96.85)	5.67e-251	hydroxyacid dehydrogenase [Pantoea deleyi]	470932	d__Bacteria;k__norank;p__Proteobacteria;c__Gammaproteobacteria;o__Enterobacterales;f__Erwiniaceae;g__Pantoea;s__Pantoea_deleyi
PB3_gene_1737	228	ORM80813.1	228	216/228(94.74)	5.60e-145	hypothetical protein HA42_12870 [Pantoea deleyi]	470932	d__Bacteria;k__norank;p__Proteobacteria;c__Gammaproteobacteria;o__Enterobacterales;f__Erwiniaceae;g__Pantoea;s__Pantoea_deleyi
PB3_gene_1741	370	ORM80817.1	374	351/370(94.86)	4.91e-243	diguanylate cyclase AdrA [Pantoea deleyi]	470932	d__Bacteria;k__norank;p__Proteobacteria;c__Gammaproteobacteria;o__Enterobacterales;f__Erwiniaceae;g__Pantoea;s__Pantoea_deleyi
PB3_gene_1742	261	ORM80818.1	261	260/261(99.62)	4.55e-190	type I methionyl aminopeptidase [Pantoea deleyi]	470932	d__Bacteria;k__norank;p__Proteobacteria;c__Gammaproteobacteria;o__Enterobacterales;f__Erwiniaceae;g__Pantoea;s__Pantoea_deleyi
PB3_gene_1760	1061	WP_111141250.1	1407	1059/1061(99.81)	0	DNA-directed RNA polymerase subunit beta' [Pantoea sp. ARC607]	2027922	d__Bacteria;k__norank;p__Proteobacteria;c__Gammaproteobacteria;o__Enterobacterales;f__Erwiniaceae;g__Pantoea;s__Pantoea_sp._ARC607

PB3_gene_1768	222	ORM80951.1	222	220/222(99.10)	4.22e-162	endonuclease V [Pantoea deleyi]	470932	d__Bacteria;k__norank;p__Proteobacteria;c__Gammaproteobacteria;o__Enterobacterales;f__Erwiniaceae;g__Pantoea;s__Pantoea_deleyi
PB3_gene_1772	271	WP_111141239.1	426	266/271(98.15)	7.80e-194	phosphoribosylamine--glycine ligase [Pantoea sp. ARC607]	2027922	d__Bacteria;k__norank;p__Proteobacteria;c__Gammaproteobacteria;o__Enterobacterales;f__Erwiniaceae;g__Pantoea;s__Pantoea_sp._ARC607
PB3_gene_1784	211	WP_111139566.1	211	206/211(97.63)	3.90e-148	NAD-dependent epimerase/dehydratase family protein [Pantoea sp. ARC607]	2027922	d__Bacteria;k__norank;p__Proteobacteria;c__Gammaproteobacteria;o__Enterobacterales;f__Erwiniaceae;g__Pantoea;s__Pantoea_sp._ARC607
PB3_gene_1788	770	PXW20944.1	770	741/770(96.23)	0	biotin/methionine sulfoxide reductase [Pantoea sp.	1938795	d__Bacteria;k__norank;p__Proteobacteria;c__Gammaproteobacteria;o__Enterobacterales;f__Erwiniaceae;g__Pantoea;s__Pantoea_sp._JKS000250
PB3_gene_1791	882	WP_111139848.1	883	799/883(90.49)	0	beta-N-acetylhexosaminidase [Pantoea sp. ARC607]	2027922	d__Bacteria;k__norank;p__Proteobacteria;c__Gammaproteobacteria;o__Enterobacterales;f__Erwiniaceae;g__Pantoea;s__Pantoea_sp._ARC607
PB3_gene_1794	751	ORM80602.1	765	745/751(99.20)	0	beta-glucosidase [Pantoea deleyi]	470932	d__Bacteria;k__norank;p__Proteobacteria;c__Gammaproteobacteria;o__Enterobacterales;f__Erwiniaceae;g__Pantoea;s__Pantoea_deleyi
PB3_gene_1800	234	WP_111142058.1	231	226/231(97.84)	2.76e-170	succinylglutamate desuccinylase, partial [Pantoea sp. ARC607]	2027922	d__Bacteria;k__norank;p__Proteobacteria;c__Gammaproteobacteria;o__Enterobacterales;f__Erwiniaceae;g__Pantoea;s__Pantoea_sp._ARC607
PB3_gene_1812	76	WP_111139136.1	208	76/76(100.00)	5.21e-44	DUF1007 family protein [Pantoea sp. ARC607]	2027922	d__Bacteria;k__norank;p__Proteobacteria;c__Gammaproteobacteria;o__Enterobacterales;f__Erwiniaceae;g__Pantoea;s__Pantoea_sp._ARC607
PB3_gene_1816	407	ORM84933.1	394	373/394(94.67)	2.76e-275	nitric oxide dioxygenase [Pantoea deleyi]	470932	d__Bacteria;k__norank;p__Proteobacteria;c__Gammaproteobacteria;o__Enterobacterales;f__Erwiniaceae;g__Pantoea;s__Pantoea_deleyi
PB3_gene_1818	444	ORM84925.1	444	443/444(99.77)	5.52e-313	two-component system response regulator GlrR [Pantoea deleyi]	470932	d__Bacteria;k__norank;p__Proteobacteria;c__Gammaproteobacteria;o__Enterobacterales;f__Erwiniaceae;g__Pantoea;s__Pantoea_deleyi
PB3_gene_1820	475	ORM84923.1	475	466/475(98.11)	0	two-component sensor histidine kinase [Pantoea deleyi]	470932	d__Bacteria;k__norank;p__Proteobacteria;c__Gammaproteobacteria;o__Enterobacterales;f__Erwiniaceae;g__Pantoea;s__Pantoea_deleyi
PB3_gene_1821	1183	WP_111139127.1	1295	1173/1183(99.15)	0	phosphoribosylformylglycinamidine synthase [Pantoea sp.	2027922	d__Bacteria;k__norank;p__Proteobacteria;c__Gammaproteobacteria;o__Enterobacterales;f__Erwiniaceae;g__Pantoea;s__Pantoea_sp._ARC607
PB3_gene_1829	281	WP_111139793.1	287	263/281(93.59)	1.09e-188	hypothetical protein [Pantoea sp. ARC607]	2027922	d__Bacteria;k__norank;p__Proteobacteria;c__Gammaproteobacteria;o__Enterobacterales;f__Erwiniaceae;g__Pantoea;s__Pantoea_sp._ARC607
PB3_gene_1834	284	WP_111139809.1	273	267/273(97.80)	2.93e-204	DUF72 domain-containing protein [Pantoea sp. ARC607]	2027922	d__Bacteria;k__norank;p__Proteobacteria;c__Gammaproteobacteria;o__Enterobacterales;f__Erwiniaceae;g__Pantoea;s__Pantoea_sp._ARC607
PB3_gene_1845	761	WP_111141895.1	761	705/761(92.64)	0	zinc/cadmium/mercury/lead-transporting ATPase [Pantoea sp. ARC607]	2027922	d__Bacteria;k__norank;p__Proteobacteria;c__Gammaproteobacteria;o__Enterobacterales;f__Erwiniaceae;g__Pantoea;s__Pantoea_sp._ARC607
PB3_gene_1850	360	WP_111141887.1	336	333/336(99.11)	2.18e-241	Gfo/Idh/MocA family oxidoreductase [Pantoea sp. ARC607]	2027922	d__Bacteria;k__norank;p__Proteobacteria;c__Gammaproteobacteria;o__Enterobacterales;f__Erwiniaceae;g__Pantoea;s__Pantoea_sp._ARC607
PB3_gene_1869	215	ORM76848.1	397	177/275(64.36)	2.30e-91	MFS transporter [Pantoea deleyi]	470932	d__Bacteria;k__norank;p__Proteobacteria;c__Gammaproteobacteria;o__Enterobacterales;f__Erwiniaceae;g__Pantoea;s__Pantoea_deleyi
PB3_gene_1872	646	WP_111138403.1	646	583/597(97.65)	0	methyl-accepting chemotaxis protein [Pantoea sp. ARC607]	2027922	d__Bacteria;k__norank;p__Proteobacteria;c__Gammaproteobacteria;o__Enterobacterales;f__Erwiniaceae;g__Pantoea;s__Pantoea_sp._ARC607
PB3_gene_1885	587	ORM80778.1	587	581/587(98.98)	0	ribosomal protein S12 methylthiotransferase accessory factor YcaO [Pantoea deleyi]	470932	d__Bacteria;k__norank;p__Proteobacteria;c__Gammaproteobacteria;o__Enterobacterales;f__Erwiniaceae;g__Pantoea;s__Pantoea_deleyi
PB3_gene_1897	867	WP_111140899.1	867	817/865(94.45)	0	EAL domain-containing protein [Pantoea sp. ARC607]	2027922	d__Bacteria;k__norank;p__Proteobacteria;c__Gammaproteobacteria;o__Enterobacterales;f__Erwiniaceae;g__Pantoea;s__Pantoea_sp._ARC607
PB3_gene_1903	256	ORM78515.1	256	253/256(98.83)	2.22e-174	transcriptional regulator LldR [Pantoea deleyi]	470932	d__Bacteria;k__norank;p__Proteobacteria;c__Gammaproteobacteria;o__Enterobacterales;f__Erwiniaceae;g__Pantoea;s__Pantoea_deleyi
PB3_gene_1905	389	WP_111140906.1	389	374/389(96.14)	4.34e-257	MFS transporter [Pantoea sp. ARC607]	2027922	d__Bacteria;k__norank;p__Proteobacteria;c__Gammaproteobacteria;o__Enterobacterales;f__Erwiniaceae;g__Pantoea;s__Pantoea_sp._ARC607
PB3_gene_1909	502	WP_111141905.1	519	493/502(98.21)	0	galactarate dehydratase [Pantoea sp. ARC607]	2027922	d__Bacteria;k__norank;p__Proteobacteria;c__Gammaproteobacteria;o__Enterobacterales;f__Erwiniaceae;g__Pantoea;s__Pantoea_sp._ARC607

PB3_gene_1914	294	ORM80478.1	294	291/294(98.98)	2.63e-201	2-hydroxy-3-oxopropionate reductase [Pantoea deleyi]	470932	d__Bacteria;k__norank;p__Proteobacteria;c__Gammaproteobacteria;o__Enterobacterales;f__Erwiniaceae;g__Pantoea;s__Pantoea_deleyi
PB3_gene_1916	909	ORM78608.1	909	899/909(98.90)	0	two-component sensor histidine kinase BarA [Pantoea deleyi]	470932	d__Bacteria;k__norank;p__Proteobacteria;c__Gammaproteobacteria;o__Enterobacterales;f__Erwiniaceae;g__Pantoea;s__Pantoea_deleyi
PB3_gene_1931	398	ORM77994.1	398	393/398(98.74)	4.62e-268	efflux transporter periplasmic adaptor subunit [Pantoea deleyi]	470932	d__Bacteria;k__norank;p__Proteobacteria;c__Gammaproteobacteria;o__Enterobacterales;f__Erwiniaceae;g__Pantoea;s__Pantoea_deleyi
PB3_gene_1944	286	WP_111141796.1	286	273/286(95.45)	4.10e-185	triphosphoribosyl-dephospho-CoA synthase [Pantoea sp.	2027922	d__Bacteria;k__norank;p__Proteobacteria;c__Gammaproteobacteria;o__Enterobacterales;f__Erwiniaceae;g__Pantoea;s__Pantoea_sp._ARC607
PB3_gene_1959	470	ORM80785.1	470	469/470(99.79)	0	pyruvate kinase [Pantoea deleyi]	470932	d__Bacteria;k__norank;p__Proteobacteria;c__Gammaproteobacteria;o__Enterobacterales;f__Erwiniaceae;g__Pantoea;s__Pantoea_deleyi
PB3_gene_1971	238	ORM81373.1	238	220/238(92.44)	1.14e-158	4-phosphopantetheinyl transferase [Pantoea deleyi]	470932	d__Bacteria;k__norank;p__Proteobacteria;c__Gammaproteobacteria;o__Enterobacterales;f__Erwiniaceae;g__Pantoea;s__Pantoea_deleyi
PB3_gene_1981	473	ORM82303.1	473	463/473(97.89)	0	diguanylate cyclase [Pantoea deleyi]	470932	d__Bacteria;k__norank;p__Proteobacteria;c__Gammaproteobacteria;o__Enterobacterales;f__Erwiniaceae;g__Pantoea;s__Pantoea_deleyi
PB3_gene_1995	290	WP_111140565.1	304	285/290(98.28)	1.02e-188	threonine/homoserine exporter RhtA [Pantoea sp. ARC607]	2027922	d__Bacteria;k__norank;p__Proteobacteria;c__Gammaproteobacteria;o__Enterobacterales;f__Erwiniaceae;g__Pantoea;s__Pantoea_sp._ARC607
PB3_gene_2014	265	ORM75561.1	266	257/266(96.62)	4.48e-181	acyl-phosphate glycerol 3-phosphate acyltransferase [Pantoea deleyi]	470932	d__Bacteria;k__norank;p__Proteobacteria;c__Gammaproteobacteria;o__Enterobacterales;f__Erwiniaceae;g__Pantoea;s__Pantoea_deleyi
PB3_gene_2015	243	ORM75560.1	243	238/243(97.94)	2.62e-171	beta-ketoacyl synthase [Pantoea deleyi]	470932	d__Bacteria;k__norank;p__Proteobacteria;c__Gammaproteobacteria;o__Enterobacterales;f__Erwiniaceae;g__Pantoea;s__Pantoea_deleyi
PB3_gene_2019	561	AVV37377.1	561	549/561(97.86)	0	MFS transporter [Pantoea vagans]	470934	d__Bacteria;k__norank;p__Proteobacteria;c__Gammaproteobacteria;o__Enterobacterales;f__Erwiniaceae;g__Pantoea;s__Pantoea_vagans
PB3_gene_2026	82	AVV37538.1	67	31/49(63.27)	5.17e-09	hypothetical protein C9381_10200 [Pantoea vagans]	470934	d__Bacteria;k__norank;p__Proteobacteria;c__Gammaproteobacteria;o__Enterobacterales;f__Erwiniaceae;g__Pantoea;s__Pantoea_vagans
PB3_gene_2041	516	ORM75932.1	516	505/516(97.87)	0	histidine ammonia-lyase [Pantoea deleyi]	470932	d__Bacteria;k__norank;p__Proteobacteria;c__Gammaproteobacteria;o__Enterobacterales;f__Erwiniaceae;g__Pantoea;s__Pantoea_deleyi
PB3_gene_2046	172	WP_111139865.1	172	156/171(91.23)	6.12e-101	GNAT family N-acetyltransferase [Pantoea sp. ARC607]	2027922	d__Bacteria;k__norank;p__Proteobacteria;c__Gammaproteobacteria;o__Enterobacterales;f__Erwiniaceae;g__Pantoea;s__Pantoea_sp._ARC607
PB3_gene_2047	184	ORM76532.1	187	179/184(97.28)	1.67e-125	DNA-binding protein [Pantoea deleyi]	470932	d__Bacteria;k__norank;p__Proteobacteria;c__Gammaproteobacteria;o__Enterobacterales;f__Erwiniaceae;g__Pantoea;s__Pantoea_deleyi
PB3_gene_2048	423	ORM83269.1	419	381/417(91.37)	4.57e-266	MFS transporter [Pantoea deleyi]	470932	d__Bacteria;k__norank;p__Proteobacteria;c__Gammaproteobacteria;o__Enterobacterales;f__Erwiniaceae;g__Pantoea;s__Pantoea_deleyi
PB3_gene_2056	442	WP_094110181.1	442	322/442(72.85)	5.79e-231	hypothetical protein [Lonsdalea iberica]	1082703	d__Bacteria;k__norank;p__Proteobacteria;c__Gammaproteobacteria;o__Enterobacterales;f__Pectobacteriaceae;g__Lonsdalea;s__Lonsdalea_iberica
PB3_gene_2062	319	WP_111140554.1	319	312/319(97.81)	2.11e-215	isoaspartyl peptidase/L-asparaginase [Pantoea sp.	2027922	d__Bacteria;k__norank;p__Proteobacteria;c__Gammaproteobacteria;o__Enterobacterales;f__Erwiniaceae;g__Pantoea;s__Pantoea_sp._ARC607
PB3_gene_2063	618	ORM81994.1	618	612/618(99.03)	0	glutathione ABC transporter ATP-binding protein GsiA [Pantoea deleyi]	470932	d__Bacteria;k__norank;p__Proteobacteria;c__Gammaproteobacteria;o__Enterobacterales;f__Erwiniaceae;g__Pantoea;s__Pantoea_deleyi
PB3_gene_2068	187	WP_128085241.1	357	179/187(95.72)	2.30e-121	MFS transporter [Pantoea deleyi]	None	Unclassified
PB3_gene_2101	418	ORM81060.1	418	408/418(97.61)	2.14e-274	flagellar hook protein FlgE [Pantoea deleyi]	470932	d__Bacteria;k__norank;p__Proteobacteria;c__Gammaproteobacteria;o__Enterobacterales;f__Erwiniaceae;g__Pantoea;s__Pantoea_deleyi
PB3_gene_2122	87	PWV83852.1	87	86/87(98.85)	1.17e-51	putative transposase [Pantoea ananatis]	553	d__Bacteria;k__norank;p__Proteobacteria;c__Gammaproteobacteria;o__Enterobacterales;f__Erwiniaceae;g__Pantoea;s__Pantoea_ananatis
PB3_gene_2123	274	ARX41893.1	274	262/274(95.62)	1.26e-198	transposase (plasmid) [Klebsiella pneumoniae]	573	d__Bacteria;k__norank;p__Proteobacteria;c__Gammaproteobacteria;o__Enterobacterales;f__Enterobacteriaceae;g__Klebsiella;s__Klebsiella_pneumoniae
PB3_gene_2131	258	WP_110331944.1	258	240/255(94.12)	5.93e-180	CDP-diacylglycerol diphosphatase [Pantoea sp.	1938795	d__Bacteria;k__norank;p__Proteobacteria;c__Gammaproteobacteria;o__Enterobacterales;f__Erwiniaceae;g__Pantoea;s__Pantoea_sp._JKS000250
PB3_gene_2138	417	WP_033784488.1	504	391/417(93.76)	1.77e-273	transcription antiterminator [Pantoea sp. 9140]	1500896	d__Bacteria;k__norank;p__Proteobacteria;c__Gammaproteobacteria;o__Enterobacterales;f__Erwiniaceae;g__Pantoea;s__Pantoea_sp._9140

PB3_gene_2149	731	ORM84541.1	731	723/731(98.91)	0	primosomal protein N' [Pantoea deleyi]	470932	d__Bacteria;k__norank;p__Proteobacteria;c__Gammaproteobacteria;o__Enterobacterales;f__Erwiniaceae;g__Pantoea;s__Pantoea_deleyi
PB3_gene_2151	215	WP_111140520.1	788	207/215(96.28)	1.88e-140	xanthine dehydrogenase molybdopterin binding subunit [Pantoea sp. ARC607]	2027922	d__Bacteria;k__norank;p__Proteobacteria;c__Gammaproteobacteria;o__Enterobacterales;f__Erwiniaceae;g__Pantoea;s__Pantoea_sp._ARC607
PB3_gene_2166	777	ORM86317.1	777	767/776(98.84)	0	aerobic respiration two-component sensor histidine kinase ArcB [Pantoea deleyi]	470932	d__Bacteria;k__norank;p__Proteobacteria;c__Gammaproteobacteria;o__Enterobacterales;f__Erwiniaceae;g__Pantoea;s__Pantoea_deleyi
PB3_gene_2188	1040	ORM76522.1	1040	1026/1040(98.65)	0	multidrug transporter subunit MdtB [Pantoea deleyi]	470932	d__Bacteria;k__norank;p__Proteobacteria;c__Gammaproteobacteria;o__Enterobacterales;f__Erwiniaceae;g__Pantoea;s__Pantoea_deleyi
PB3_gene_2191	462	ORM77873.1	462	457/462(98.92)	0	two-component system sensor histidine kinase BaeA [Pantoea deleyi]	470932	d__Bacteria;k__norank;p__Proteobacteria;c__Gammaproteobacteria;o__Enterobacterales;f__Erwiniaceae;g__Pantoea;s__Pantoea_deleyi
PB3_gene_2193	210	CSI40465.1	229	105/172(61.05)	7.85e-63	Uncharacterised protein [Shigella sonnei]	624	d__Bacteria;k__norank;p__Proteobacteria;c__Gammaproteobacteria;o__Enterobacterales;f__Enterobacteriaceae;g__Shigella;s__Shigella_sonnei
PB3_gene_2194	909	ORM86125.1	895	890/895(99.44)	0	bifunctional acetaldehyde-CoA/alcohol dehydrogenase [Pantoea deleyi]	470932	d__Bacteria;k__norank;p__Proteobacteria;c__Gammaproteobacteria;o__Enterobacterales;f__Erwiniaceae;g__Pantoea;s__Pantoea_deleyi
PB3_gene_2195	210	ORM86126.1	210	208/209(99.52)	4.40e-149	thymidine kinase [Pantoea deleyi]	470932	d__Bacteria;k__norank;p__Proteobacteria;c__Gammaproteobacteria;o__Enterobacterales;f__Erwiniaceae;g__Pantoea;s__Pantoea_deleyi
PB3_gene_2196	136	ORM86127.1	136	135/136(99.26)	7.32e-87	transcriptional regulator [Pantoea deleyi]	470932	d__Bacteria;k__norank;p__Proteobacteria;c__Gammaproteobacteria;o__Enterobacterales;f__Erwiniaceae;g__Pantoea;s__Pantoea_deleyi
PB3_gene_2197	335	ORM86128.1	335	330/335(98.51)	5.38e-242	protein CapI [Pantoea deleyi]	470932	d__Bacteria;k__norank;p__Proteobacteria;c__Gammaproteobacteria;o__Enterobacterales;f__Erwiniaceae;g__Pantoea;s__Pantoea_deleyi
PB3_gene_2207	363	WP_111138742.1	363	355/362(98.07)	1.33e-247	microcin C ABC transporter permease YejB [Pantoea sp.	2027922	d__Bacteria;k__norank;p__Proteobacteria;c__Gammaproteobacteria;o__Enterobacterales;f__Erwiniaceae;g__Pantoea;s__Pantoea_sp._ARC607
PB3_gene_2208	602	ORM81386.1	602	589/602(97.84)	0	antibiotic ABC transporter substrate-binding protein	470932	d__Bacteria;k__norank;p__Proteobacteria;c__Gammaproteobacteria;o__Enterobacterales;f__Erwiniaceae;g__Pantoea;s__Pantoea_deleyi
PB3_gene_2223	328	WP_111138808.1	328	311/328(94.82)	3.85e-227	tetraacyldisaccharide 4'-kinase [Pantoea sp. ARC607]	2027922	d__Bacteria;k__norank;p__Proteobacteria;c__Gammaproteobacteria;o__Enterobacterales;f__Erwiniaceae;g__Pantoea;s__Pantoea_sp._ARC607
PB3_gene_2225	441	WP_111138810.1	751	402/441(91.16)	6.12e-291	DNA internalization-related competence protein ComEC/Rec2 [Pantoea sp. ARC607]	2027922	d__Bacteria;k__norank;p__Proteobacteria;c__Gammaproteobacteria;o__Enterobacterales;f__Erwiniaceae;g__Pantoea;s__Pantoea_sp._ARC607
PB3_gene_2235	740	ORM84708.1	734	728/734(99.18)	0	TIGR01666 family membrane protein [Pantoea deleyi]	470932	d__Bacteria;k__norank;p__Proteobacteria;c__Gammaproteobacteria;o__Enterobacterales;f__Erwiniaceae;g__Pantoea;s__Pantoea_deleyi
PB3_gene_2240	310	ORM78625.1	310	299/310(96.45)	1.12e-210	ketodeoxygluconokinase [Pantoea deleyi]	470932	d__Bacteria;k__norank;p__Proteobacteria;c__Gammaproteobacteria;o__Enterobacterales;f__Erwiniaceae;g__Pantoea;s__Pantoea_deleyi
PB3_gene_2253	696	WP_111139317.1	697	668/697(95.84)	0	ABC transporter permease subunit [Pantoea sp. ARC607]	2027922	d__Bacteria;k__norank;p__Proteobacteria;c__Gammaproteobacteria;o__Enterobacterales;f__Erwiniaceae;g__Pantoea;s__Pantoea_sp._ARC607
PB3_gene_2262	860	WP_111139031.1	860	854/860(99.30)	0	leucine--tRNA ligase [Pantoea sp. ARC607]	2027922	d__Bacteria;k__norank;p__Proteobacteria;c__Gammaproteobacteria;o__Enterobacterales;f__Erwiniaceae;g__Pantoea;s__Pantoea_sp._ARC607
PB3_gene_2269	249	ORM80437.1	249	242/249(97.19)	1.84e-174	phosphopantetheinyl transferase [Pantoea deleyi]	470932	d__Bacteria;k__norank;p__Proteobacteria;c__Gammaproteobacteria;o__Enterobacterales;f__Erwiniaceae;g__Pantoea;s__Pantoea_deleyi
PB3_gene_2270	236	WP_111141704.1	196	193/196(98.47)	2.93e-137	malonic semialdehyde reductase [Pantoea sp. ARC607]	2027922	d__Bacteria;k__norank;p__Proteobacteria;c__Gammaproteobacteria;o__Enterobacterales;f__Erwiniaceae;g__Pantoea;s__Pantoea_sp._ARC607
PB3_gene_2271	454	ORM80438.1	454	451/454(99.34)	0	tRNA uridine-5-carboxymethylaminomethyl(34) synthesis GTPase MnmE [Pantoea deleyi]	470932	d__Bacteria;k__norank;p__Proteobacteria;c__Gammaproteobacteria;o__Enterobacterales;f__Erwiniaceae;g__Pantoea;s__Pantoea_deleyi
PB3_gene_2275	366	ORM78820.1	366	365/366(99.73)	1.46e-257	DNA polymerase III subunit beta [Pantoea deleyi]	470932	d__Bacteria;k__norank;p__Proteobacteria;c__Gammaproteobacteria;o__Enterobacterales;f__Erwiniaceae;g__Pantoea;s__Pantoea_deleyi

PB3_gene_2290	400	ORM83132.1	591	397/400(99.25)	2.39e-269	multidrug ABC transporter permease/ATP-binding protein [Pantoea deleyi]	470932	d__Bacteria;k__norank;p__Proteobacteria;c__Gammaproteobacteria;o__Enterobacterales;f__Erwiniaceae;g__Pantoea;s__Pantoea_deleyi
PB3_gene_2291	589	WP_111140954.1	589	581/589(98.64)	0	SmdA family multidrug ABC transporter permease/ATP-binding protein [Pantoea sp.	2027922	d__Bacteria;k__norank;p__Proteobacteria;c__Gammaproteobacteria;o__Enterobacterales;f__Erwiniaceae;g__Pantoea;s__Pantoea_sp._ARC607
PB3_gene_2329	46	WP_111138425.1	1201	43/46(93.48)	3.41e-18	urea carboxylase [Pantoea sp. ARC607]	2027922	d__Bacteria;k__norank;p__Proteobacteria;c__Gammaproteobacteria;o__Enterobacterales;f__Erwiniaceae;g__Pantoea;s__Pantoea_sp._ARC607
PB3_gene_2331	400	ORM79858.1	400	394/400(98.50)	4.43e-267	MFS transporter [Pantoea deleyi]	470932	d__Bacteria;k__norank;p__Proteobacteria;c__Gammaproteobacteria;o__Enterobacterales;f__Erwiniaceae;g__Pantoea;s__Pantoea_deleyi
PB3_gene_2334	468	WP_111139557.1	468	462/468(98.72)	0	PLP-dependent aminotransferase family protein [Pantoea sp.	2027922	d__Bacteria;k__norank;p__Proteobacteria;c__Gammaproteobacteria;o__Enterobacterales;f__Erwiniaceae;g__Pantoea;s__Pantoea_sp._ARC607
PB3_gene_2340	340	WP_111139275.1	534	281/300(93.67)	1.97e-198	EAL domain-containing protein [Pantoea sp. ARC607]	2027922	d__Bacteria;k__norank;p__Proteobacteria;c__Gammaproteobacteria;o__Enterobacterales;f__Erwiniaceae;g__Pantoea;s__Pantoea_sp._ARC607
PB3_gene_2341	394	ORM82650.1	394	390/394(98.98)	5.19e-288	mannonate dehydratase [Pantoea deleyi]	470932	d__Bacteria;k__norank;p__Proteobacteria;c__Gammaproteobacteria;o__Enterobacterales;f__Erwiniaceae;g__Pantoea;s__Pantoea_deleyi
PB3_gene_2345	271	ORM82653.1	271	253/271(93.36)	8.57e-175	inosose isomerase [Pantoea deleyi]	470932	d__Bacteria;k__norank;p__Proteobacteria;c__Gammaproteobacteria;o__Enterobacterales;f__Erwiniaceae;g__Pantoea;s__Pantoea_deleyi
PB3_gene_2366	331	WP_111139474.1	331	331/331(100.00)	1.49e-246	peptide ABC transporter ATP-binding protein SapD [Pantoea sp. ARC607]	2027922	d__Bacteria;k__norank;p__Proteobacteria;c__Gammaproteobacteria;o__Enterobacterales;f__Erwiniaceae;g__Pantoea;s__Pantoea_sp._ARC607
PB3_gene_2372	338	WP_111139479.1	333	328/332(98.80)	4.76e-234	phage shock protein operon transcriptional activator [Pantoea sp. ARC607]	2027922	d__Bacteria;k__norank;p__Proteobacteria;c__Gammaproteobacteria;o__Enterobacterales;f__Erwiniaceae;g__Pantoea;s__Pantoea_sp._ARC607
PB3_gene_2376	533	WP_111139839.1	533	519/533(97.37)	0	inorganic phosphate transporter [Pantoea sp. ARC607]	2027922	d__Bacteria;k__norank;p__Proteobacteria;c__Gammaproteobacteria;o__Enterobacterales;f__Erwiniaceae;g__Pantoea;s__Pantoea_sp._ARC607
PB3_gene_2377	372	WP_111139842.1	372	366/372(98.39)	1.09e-246	L,D-transpeptidase [Pantoea sp. ARC607]	2027922	d__Bacteria;k__norank;p__Proteobacteria;c__Gammaproteobacteria;o__Enterobacterales;f__Erwiniaceae;g__Pantoea;s__Pantoea_sp._ARC607
PB3_gene_2380	195	WP_111139845.1	195	193/195(98.97)	2.20e-137	YIP1 family protein [Pantoea sp. ARC607]	2027922	d__Bacteria;k__norank;p__Proteobacteria;c__Gammaproteobacteria;o__Enterobacterales;f__Erwiniaceae;g__Pantoea;s__Pantoea_sp._ARC607
PB3_gene_2383	591	ORM78819.1	802	589/591(99.66)	0	DNA gyrase subunit B [Pantoea deleyi]	470932	d__Bacteria;k__norank;p__Proteobacteria;c__Gammaproteobacteria;o__Enterobacterales;f__Erwiniaceae;g__Pantoea;s__Pantoea_deleyi
PB3_gene_2386	419	WP_111141089.1	419	407/419(97.14)	1.90e-314	DUF3748 domain-containing protein [Pantoea sp. ARC607]	2027922	d__Bacteria;k__norank;p__Proteobacteria;c__Gammaproteobacteria;o__Enterobacterales;f__Erwiniaceae;g__Pantoea;s__Pantoea_sp._ARC607
PB3_gene_2395	839	WP_111142181.1	853	832/839(99.17)	0	DNA mismatch repair protein MutS [Pantoea sp. ARC607]	2027922	d__Bacteria;k__norank;p__Proteobacteria;c__Gammaproteobacteria;o__Enterobacterales;f__Erwiniaceae;g__Pantoea;s__Pantoea_sp._ARC607
PB3_gene_2408	249	WP_111140397.1	249	244/249(97.99)	6.96e-179	glycerophosphodiester phosphodiesterase [Pantoea sp. ARC607]	2027922	d__Bacteria;k__norank;p__Proteobacteria;c__Gammaproteobacteria;o__Enterobacterales;f__Erwiniaceae;g__Pantoea;s__Pantoea_sp._ARC607
PB3_gene_2419	74	ORM79310.1	265	74/74(100.00)	1.10e-35	endopeptidase [Pantoea deleyi]	470932	d__Bacteria;k__norank;p__Proteobacteria;c__Gammaproteobacteria;o__Enterobacterales;f__Erwiniaceae;g__Pantoea;s__Pantoea_deleyi
PB3_gene_2421	110	PAW36719.1	110	110/110(100.00)	1.15e-73	glutaredoxin [Pantoea vagans]	470934	d__Bacteria;k__norank;p__Proteobacteria;c__Gammaproteobacteria;o__Enterobacterales;f__Erwiniaceae;g__Pantoea;s__Pantoea_vagans
PB3_gene_2426	106	KHS47424.1	80	31/66(46.97)	5.78e-06	hypothetical protein RN38_09045 [Hafnia paralvei]	546367	d__Bacteria;k__norank;p__Proteobacteria;c__Gammaproteobacteria;o__Enterobacterales;f__Hafniaceae;g__Hafnia;s__Hafnia_paralvei
PB3_gene_2429	171	ORM79303.1	155	151/155(97.42)	4.84e-103	hypothetical protein HA42_15605 [Pantoea deleyi]	470932	d__Bacteria;k__norank;p__Proteobacteria;c__Gammaproteobacteria;o__Enterobacterales;f__Erwiniaceae;g__Pantoea;s__Pantoea_deleyi
PB3_gene_2430	666	WP_111139058.1	666	655/666(98.35)	0	FUSC family protein [Pantoea sp. ARC607]	2027922	d__Bacteria;k__norank;p__Proteobacteria;c__Gammaproteobacteria;o__Enterobacterales;f__Erwiniaceae;g__Pantoea;s__Pantoea_sp._ARC607
PB3_gene_2443	360	ORM83720.1	360	358/360(99.44)	4.00e-252	peptide chain release factor 1 [Pantoea deleyi]	470932	d__Bacteria;k__norank;p__Proteobacteria;c__Gammaproteobacteria;o__Enterobacterales;f__Erwiniaceae;g__Pantoea;s__Pantoea_deleyi

PB3_gene_2449	247	ORM86560.1	247	239/247(96.76)	6.79e-160	NAD(P)-dependent oxidoreductase [Pantoea deleyi]	470932	d__Bacteria;k__norank;p__Proteobacteria;c__Gammaproteobacteria;o__Enterobacterales;f__Erwiniaceae;g__Pantoea;s__Pantoea_deleyi
PB3_gene_2452	365	ORM86557.1	365	360/365(98.63)	4.93e-260	glycine cleavage system protein T [Pantoea deleyi]	470932	d__Bacteria;k__norank;p__Proteobacteria;c__Gammaproteobacteria;o__Enterobacterales;f__Erwiniaceae;g__Pantoea;s__Pantoea_deleyi
PB3_gene_2453	400	ORM86556.1	400	393/400(98.25)	2.90e-288	FAD-dependent 2-octaprenylphenol hydroxylase	470932	d__Bacteria;k__norank;p__Proteobacteria;c__Gammaproteobacteria;o__Enterobacterales;f__Erwiniaceae;g__Pantoea;s__Pantoea_deleyi
PB3_gene_2455	440	WP_128083928.1	440	431/440(97.95)	0	Xaa-Pro aminopeptidase [Pantoea deleyi]	None	Unclassified
PB3_gene_2457	512	ORM84697.1	563	386/388(99.48)	1.72e-280	bifunctional glucose-1-phosphatase/inositol phosphatase [Pantoea deleyi]	470932	d__Bacteria;k__norank;p__Proteobacteria;c__Gammaproteobacteria;o__Enterobacterales;f__Erwiniaceae;g__Pantoea;s__Pantoea_deleyi
PB3_gene_2462	304	ORM84692.1	304	299/304(98.36)	1.70e-202	hypothetical protein HA42_03905 [Pantoea deleyi]	470932	d__Bacteria;k__norank;p__Proteobacteria;c__Gammaproteobacteria;o__Enterobacterales;f__Erwiniaceae;g__Pantoea;s__Pantoea_deleyi
PB3_gene_2463	442	ORM84691.1	442	434/442(98.19)	1.30e-294	pyrimidine utilization transport protein G [Pantoea deleyi]	470932	d__Bacteria;k__norank;p__Proteobacteria;c__Gammaproteobacteria;o__Enterobacterales;f__Erwiniaceae;g__Pantoea;s__Pantoea_deleyi
PB3_gene_2464	171	WP_111138566.1	171	167/171(97.66)	1.12e-116	pyrimidine utilization flavin reductase protein F [Pantoea sp. ARC607]	2027922	d__Bacteria;k__norank;p__Proteobacteria;c__Gammaproteobacteria;o__Enterobacterales;f__Erwiniaceae;g__Pantoea;s__Pantoea_sp._ARC607
PB3_gene_2465	261	ORM84689.1	269	252/261(96.55)	3.35e-178	pyrimidine utilization protein D [Pantoea deleyi]	470932	d__Bacteria;k__norank;p__Proteobacteria;c__Gammaproteobacteria;o__Enterobacterales;f__Erwiniaceae;g__Pantoea;s__Pantoea_deleyi
PB3_gene_2466	131	WP_111138568.1	131	128/131(97.71)	1.02e-86	pyrimidine utilization protein C [Pantoea sp. ARC607]	2027922	d__Bacteria;k__norank;p__Proteobacteria;c__Gammaproteobacteria;o__Enterobacterales;f__Erwiniaceae;g__Pantoea;s__Pantoea_sp._ARC607
PB3_gene_2476	1016	EZI35766.1	1020	890/1016(87.60)	0	Virulence sensor protein bvgS [Pantoea agglomerans]	549	d__Bacteria;k__norank;p__Proteobacteria;c__Gammaproteobacteria;o__Enterobacterales;f__Erwiniaceae;g__Pantoea;s__Pantoea_agglomerans
PB3_gene_2478	100	WP_111141621.1	240	100/100(100.00)	1.88e-60	nicotinamide riboside transporter PnuC [Pantoea sp. ARC607]	2027922	d__Bacteria;k__norank;p__Proteobacteria;c__Gammaproteobacteria;o__Enterobacterales;f__Erwiniaceae;g__Pantoea;s__Pantoea_sp._ARC607
PB3_gene_2498	255	PAW37524.1	255	255/255(100.00)	5.57e-197	class II glutamine amidotransferase [Pantoea	470934	d__Bacteria;k__norank;p__Proteobacteria;c__Gammaproteobacteria;o__Enterobacterales;f__Erwiniaceae;g__Pantoea;s__Pantoea_vagans
PB3_gene_2509	120	ORM82467.1	120	119/119(100.00)	2.22e-78	hypothetical protein HA42_08185 [Pantoea deleyi]	470932	d__Bacteria;k__norank;p__Proteobacteria;c__Gammaproteobacteria;o__Enterobacterales;f__Erwiniaceae;g__Pantoea;s__Pantoea_deleyi
PB3_gene_2516	636	ORM82461.1	636	621/636(97.64)	0	transcription antiterminator BglG [Pantoea deleyi]	470932	d__Bacteria;k__norank;p__Proteobacteria;c__Gammaproteobacteria;o__Enterobacterales;f__Erwiniaceae;g__Pantoea;s__Pantoea_deleyi
PB3_gene_2517	299	OQV40490.1	299	299/299(100.00)	4.59e-209	LysR family transcriptional regulator [Pantoea vagans]	470934	d__Bacteria;k__norank;p__Proteobacteria;c__Gammaproteobacteria;o__Enterobacterales;f__Erwiniaceae;g__Pantoea;s__Pantoea_vagans
PB3_gene_2524	704	ORM84718.1	704	696/704(98.86)	0	23S rRNA (guanine(2445)-N(2))/(guanine(2069)-N(7))-methyltransferase [Pantoea	470932	d__Bacteria;k__norank;p__Proteobacteria;c__Gammaproteobacteria;o__Enterobacterales;f__Erwiniaceae;g__Pantoea;s__Pantoea_deleyi
PB3_gene_2531	219	WP_128295738.1	215	120/211(56.87)	9.17e-71	hypothetical protein [Citrobacter freundii]	546	d__Bacteria;k__norank;p__Proteobacteria;c__Gammaproteobacteria;o__Enterobacterales;f__Enterobacteriaceae;g__Citrobacter;s__Citrobacter_freundii
PB3_gene_2545	227	ORM77157.1	243	218/227(96.04)	4.91e-165	D-lyxose/D-mannose family sugar isomerase [Pantoea deleyi]	470932	d__Bacteria;k__norank;p__Proteobacteria;c__Gammaproteobacteria;o__Enterobacterales;f__Erwiniaceae;g__Pantoea;s__Pantoea_deleyi
PB3_gene_2548	496	ORM77155.1	504	490/495(98.99)	0	D-xylose ABC transporter ATP-binding protein [Pantoea deleyi]	470932	d__Bacteria;k__norank;p__Proteobacteria;c__Gammaproteobacteria;o__Enterobacterales;f__Erwiniaceae;g__Pantoea;s__Pantoea_deleyi
PB3_gene_2573	462	ORM79852.1	462	455/462(98.48)	5.29e-311	chloride channel protein [Pantoea deleyi]	470932	d__Bacteria;k__norank;p__Proteobacteria;c__Gammaproteobacteria;o__Enterobacterales;f__Erwiniaceae;g__Pantoea;s__Pantoea_deleyi
PB3_gene_2577	253	WP_111139545.1	253	237/253(93.68)	8.45e-157	ABC transporter permease [Pantoea sp. ARC607]	2027922	d__Bacteria;k__norank;p__Proteobacteria;c__Gammaproteobacteria;o__Enterobacterales;f__Erwiniaceae;g__Pantoea;s__Pantoea_sp._ARC607
PB3_gene_2579	1137	WP_111138425.1	1201	1075/1135(94.71)	0	urea carboxylase [Pantoea sp. ARC607]	2027922	d__Bacteria;k__norank;p__Proteobacteria;c__Gammaproteobacteria;o__Enterobacterales;f__Erwiniaceae;g__Pantoea;s__Pantoea_sp._ARC607
PB3_gene_2581	572	TCZ24199.1	572	461/572(80.59)	0	ABC transporter permease subunit [Pantoea agglomerans]	549	d__Bacteria;k__norank;p__Proteobacteria;c__Gammaproteobacteria;o__Enterobacterales;f__Erwiniaceae;g__Pantoea;s__Pantoea_agglomerans

PB3_gene_2588	555	WP_111139654.1	556	540/555(97.30)	0	N-acetylmuramoyl-L-alanine amidase AmiB [Pantoea sp.	2027922	d__Bacteria;k__norank;p__Proteobacteria;c__Gammaproteobacteria;o__Enterobacterales;f__Erwiniaceae;g__Pantoea;s__Pantoea_sp._ARC607
PB3_gene_2590	316	ORM79176.1	316	313/316(99.05)	3.42e-224	tRNA (adenosine(37)-N6)-dimethylallyltransferase MiaA [Pantoea deleyi]	470932	d__Bacteria;k__norank;p__Proteobacteria;c__Gammaproteobacteria;o__Enterobacterales;f__Erwiniaceae;g__Pantoea;s__Pantoea_deleyi
PB3_gene_2602	320	WP_111138788.1	317	315/317(99.37)	1.40e-222	sulfonate ABC transporter substrate-binding protein [Pantoea sp. ARC607]	2027922	d__Bacteria;k__norank;p__Proteobacteria;c__Gammaproteobacteria;o__Enterobacterales;f__Erwiniaceae;g__Pantoea;s__Pantoea_sp._ARC607
PB3_gene_2605	262	ORM81102.1	262	258/262(98.47)	3.02e-175	aliphatic sulfonates ABC transporter ATP-binding protein [Pantoea deleyi]	470932	d__Bacteria;k__norank;p__Proteobacteria;c__Gammaproteobacteria;o__Enterobacterales;f__Erwiniaceae;g__Pantoea;s__Pantoea_deleyi
PB3_gene_2606	871	ORM81101.1	871	860/871(98.74)	0	aminopeptidase N [Pantoea deleyi]	470932	d__Bacteria;k__norank;p__Proteobacteria;c__Gammaproteobacteria;o__Enterobacterales;f__Erwiniaceae;g__Pantoea;s__Pantoea_deleyi
PB3_gene_2617	2461	KDA94142.1	2911	2102/2461(85.41)	0	filamentous hemagglutinin, partial [Pantoea agglomerans Eh318]	1408177	d__Bacteria;k__norank;p__Proteobacteria;c__Gammaproteobacteria;o__Enterobacterales;f__Erwiniaceae;g__Pantoea;s__Pantoea_agglomerans
PB3_gene_2631	1342	ORM80943.1	1342	1338/1342(99.70)	0	DNA-directed RNA polymerase subunit beta [Pantoea deleyi]	470932	d__Bacteria;k__norank;p__Proteobacteria;c__Gammaproteobacteria;o__Enterobacterales;f__Erwiniaceae;g__Pantoea;s__Pantoea_deleyi
PB3_gene_2633	213	CCK17023.1	457	163/213(76.53)	3.72e-103	hypothetical protein BN136_3033 [Cronobacter universalis NCTC	1074000	d__Bacteria;k__norank;p__Proteobacteria;c__Gammaproteobacteria;o__Enterobacterales;f__Enterobacteriaceae;g__Cronobacter;s__Cronobacter_universalis
PB3_gene_2642	367	WP_128085585.1	371	342/371(92.18)	1.22e-246	DNA-protecting protein DprA [Pantoea deleyi]	470932	d__Bacteria;k__norank;p__Proteobacteria;c__Gammaproteobacteria;o__Enterobacterales;f__Erwiniaceae;g__Pantoea;s__Pantoea_deleyi
PB3_gene_2649	282	KDA94905.1	282	277/282(98.23)	3.37e-207	diaminopimelate epimerase [Pantoea agglomerans Eh318]	1408177	d__Bacteria;k__norank;p__Proteobacteria;c__Gammaproteobacteria;o__Enterobacterales;f__Erwiniaceae;g__Pantoea;s__Pantoea_agglomerans
PB3_gene_2659	672	ORM80514.1	672	666/672(99.11)	0	DNA ligase (NAD(+)) LigA [Pantoea deleyi]	470932	d__Bacteria;k__norank;p__Proteobacteria;c__Gammaproteobacteria;o__Enterobacterales;f__Erwiniaceae;g__Pantoea;s__Pantoea_deleyi
PB3_gene_2664	562	WP_095101021.1	567	481/561(85.74)	0	DUF4976 domain-containing protein [Enterobacter sp. 10-1]	543	d__Bacteria;k__norank;p__Proteobacteria;c__Gammaproteobacteria;o__Enterobacterales;f__Enterobacteriaceae;g__norank;s__norank
PB3_gene_2677	360	ORM81463.1	349	347/349(99.43)	5.97e-249	polysaccharide chain length modulation protein [Pantoea	470932	d__Bacteria;k__norank;p__Proteobacteria;c__Gammaproteobacteria;o__Enterobacterales;f__Erwiniaceae;g__Pantoea;s__Pantoea_deleyi
PB3_gene_2678	359	WP_111139455.1	359	358/359(99.72)	8.00e-247	UDP-N-acetylglucosamine--undecaprenyl-phosphate N-acetylglucosaminephosphotransferase [Pantoea sp. ARC607]	2027922	d__Bacteria;k__norank;p__Proteobacteria;c__Gammaproteobacteria;o__Enterobacterales;f__Erwiniaceae;g__Pantoea;s__Pantoea_sp._ARC607
PB3_gene_2684	36	WP_033791697.1	283	26/35(74.29)	2.07e-09	DUF3289 family protein [Pantoea septica]	472695	d__Bacteria;k__norank;p__Proteobacteria;c__Gammaproteobacteria;o__Enterobacterales;f__Erwiniaceae;g__Pantoea;s__Pantoea_septica
PB3_gene_2697	1357	ORM81094.1	1495	1347/1357(99.26)	0	cell division protein MukB [Pantoea deleyi]	470932	d__Bacteria;k__norank;p__Proteobacteria;c__Gammaproteobacteria;o__Enterobacterales;f__Erwiniaceae;g__Pantoea;s__Pantoea_deleyi
PB3_gene_2698	603	ORM81095.1	603	598/603(99.17)	0	L,D-transpeptidase [Pantoea deleyi]	470932	d__Bacteria;k__norank;p__Proteobacteria;c__Gammaproteobacteria;o__Enterobacterales;f__Erwiniaceae;g__Pantoea;s__Pantoea_deleyi
PB3_gene_2714	668	WP_109650868.1	668	588/668(88.02)	0	bifunctional tRNA (5-methylaminomethyl-2-thiouridine)(34)-methyltransferase MnmD/FAD-dependent 5-carboxymethylaminomethyl-2-thiouridine(34) oxidoreductase	None	Unclassified
PB3_gene_2734	1179	ORM86588.1	1179	1149/1179(97.46)	0	exodeoxyribonuclease V subunit beta [Pantoea deleyi]	470932	d__Bacteria;k__norank;p__Proteobacteria;c__Gammaproteobacteria;o__Enterobacterales;f__Erwiniaceae;g__Pantoea;s__Pantoea_deleyi
PB3_gene_2747	76	SKA04147.1	76	73/76(96.05)	2.66e-43	hypothetical protein SAMN03097723_2918 [Pantoea agglomerans]	470933	d__Bacteria;k__norank;p__Proteobacteria;c__Gammaproteobacteria;o__Enterobacterales;f__Erwiniaceae;g__Pantoea;s__Pantoea_eucalypti

PB3_gene_2753	280	ORM77452.1	280	277/280(98.93)	8.61e-209	S-formylglutathione hydrolase [Pantoea deleyi]	470932	d__Bacteria;k__norank;p__Proteobacteria;c__Gammaproteobacteria;o__Enterobacterales;f__Erwiniaceae;g__Pantoea;s__Pantoea_deleyi
PB3_gene_2754	262	ORM77454.1	262	259/262(98.85)	9.82e-181	iron ABC transporter ATP-binding protein [Pantoea deleyi]	470932	d__Bacteria;k__norank;p__Proteobacteria;c__Gammaproteobacteria;o__Enterobacterales;f__Erwiniaceae;g__Pantoea;s__Pantoea_deleyi
PB3_gene_2757	288	ORM77449.1	288	287/288(99.65)	5.53e-200	LysR family transcriptional regulator [Pantoea deleyi]	470932	d__Bacteria;k__norank;p__Proteobacteria;c__Gammaproteobacteria;o__Enterobacterales;f__Erwiniaceae;g__Pantoea;s__Pantoea_deleyi
PB3_gene_2758	47	ORM77448.1	124	44/47(93.62)	2.20e-20	hypothetical protein HA42_17800 [Pantoea deleyi]	470932	d__Bacteria;k__norank;p__Proteobacteria;c__Gammaproteobacteria;o__Enterobacterales;f__Erwiniaceae;g__Pantoea;s__Pantoea_deleyi
PB3_gene_2760	1110	WP_111139188.1	1110	1043/1110(93.96)	0	diguanylate cyclase [Pantoea sp. ARC607]	2027922	d__Bacteria;k__norank;p__Proteobacteria;c__Gammaproteobacteria;o__Enterobacterales;f__Erwiniaceae;g__Pantoea;s__Pantoea_sp._ARC607
PB3_gene_2780	1109	ORM83369.1	1109	1095/1109(98.74)	0	miniconductance mechanosensitive channel MscM	470932	d__Bacteria;k__norank;p__Proteobacteria;c__Gammaproteobacteria;o__Enterobacterales;f__Erwiniaceae;g__Pantoea;s__Pantoea_deleyi
PB3_gene_2782	349	ORM83367.1	349	344/349(98.57)	6.09e-246	ribosome biogenesis GTPase RsgA [Pantoea deleyi]	470932	d__Bacteria;k__norank;p__Proteobacteria;c__Gammaproteobacteria;o__Enterobacterales;f__Erwiniaceae;g__Pantoea;s__Pantoea_deleyi
PB3_gene_2787	266	ORM80563.1	266	265/266(99.62)	7.73e-180	hypothetical protein HA42_14005 [Pantoea deleyi]	470932	d__Bacteria;k__norank;p__Proteobacteria;c__Gammaproteobacteria;o__Enterobacterales;f__Erwiniaceae;g__Pantoea;s__Pantoea_deleyi
PB3_gene_2795	430	WP_111138385.1	430	417/430(96.98)	0	alcaligin biosynthesis protein [Pantoea sp. ARC607]	2027922	d__Bacteria;k__norank;p__Proteobacteria;c__Gammaproteobacteria;o__Enterobacterales;f__Erwiniaceae;g__Pantoea;s__Pantoea_sp._ARC607
PB3_gene_2813	112	WP_099257875.1	112	97/112(86.61)	4.13e-68	hypothetical protein [Pantoea agglomerans]	549	d__Bacteria;k__norank;p__Proteobacteria;c__Gammaproteobacteria;o__Enterobacterales;f__Erwiniaceae;g__Pantoea;s__Pantoea_agglomerans
PB3_gene_2824	502	WP_031590408.1	497	478/497(96.18)	0	NCS1 family nucleobase:cation symporter-1 [Pantoea	549	d__Bacteria;k__norank;p__Proteobacteria;c__Gammaproteobacteria;o__Enterobacterales;f__Erwiniaceae;g__Pantoea;s__Pantoea_agglomerans
PB3_gene_2829	361	WP_031590414.1	279	203/279(72.76)	3.37e-146	TagK domain-containing protein [Pantoea agglomerans]	549	d__Bacteria;k__norank;p__Proteobacteria;c__Gammaproteobacteria;o__Enterobacterales;f__Erwiniaceae;g__Pantoea;s__Pantoea_agglomerans
PB3_gene_2837	1242	WP_090961983.1	1454	1149/1242(92.51)	0	type IV secretion protein Rhs [Pantoea sp. OV426]	1881036	d__Bacteria;k__norank;p__Proteobacteria;c__Gammaproteobacteria;o__Enterobacterales;f__Erwiniaceae;g__Pantoea;s__Pantoea_sp._OV426
PB3_gene_2841	259	WP_083068291.1	259	215/259(83.01)	8.21e-151	MULTISPECIES: hypothetical protein [Pantoea]	53335	d__Bacteria;k__norank;p__Proteobacteria;c__Gammaproteobacteria;o__Enterobacterales;f__Erwiniaceae;g__Pantoea;s__norank
PB3_gene_2842	239	WP_111142272.1	249	197/239(82.43)	3.57e-142	hypothetical protein [Pantoea sp. ARC607]	2027922	d__Bacteria;k__norank;p__Proteobacteria;c__Gammaproteobacteria;o__Enterobacterales;f__Erwiniaceae;g__Pantoea;s__Pantoea_sp._ARC607
PB3_gene_2848	254	WP_089561851.1	267	196/254(77.17)	1.37e-138	MULTISPECIES: triphosphoribosyl-dephospho-CoA synthase CitG [Pantoea]	53335	d__Bacteria;k__norank;p__Proteobacteria;c__Gammaproteobacteria;o__Enterobacterales;f__Erwiniaceae;g__Pantoea;s__norank
PB3_gene_2862	293	ORM85228.1	303	277/293(94.54)	1.98e-207	hypothetical protein HA42_03045 [Pantoea deleyi]	470932	d__Bacteria;k__norank;p__Proteobacteria;c__Gammaproteobacteria;o__Enterobacterales;f__Erwiniaceae;g__Pantoea;s__Pantoea_deleyi
PB3_gene_2868	308	ORM85222.1	308	290/308(94.16)	1.59e-209	aminoimidazole riboside kinase [Pantoea deleyi]	470932	d__Bacteria;k__norank;p__Proteobacteria;c__Gammaproteobacteria;o__Enterobacterales;f__Erwiniaceae;g__Pantoea;s__Pantoea_deleyi
PB3_gene_2871	392	ORM86824.1	392	387/392(98.72)	1.23e-269	phosphoribosylglycinamide formyltransferase 2 [Pantoea	470932	d__Bacteria;k__norank;p__Proteobacteria;c__Gammaproteobacteria;o__Enterobacterales;f__Erwiniaceae;g__Pantoea;s__Pantoea_deleyi
PB3_gene_2913	465	WP_111140127.1	465	442/465(95.05)	3.61e-315	AtzE family amidohydrolase [Pantoea sp. ARC607]	2027922	d__Bacteria;k__norank;p__Proteobacteria;c__Gammaproteobacteria;o__Enterobacterales;f__Erwiniaceae;g__Pantoea;s__Pantoea_sp._ARC607
PB3_gene_2922	240	ORM85043.1	240	238/240(99.17)	9.75e-161	RNA polymerase sigma factor FliA [Pantoea deleyi]	470932	d__Bacteria;k__norank;p__Proteobacteria;c__Gammaproteobacteria;o__Enterobacterales;f__Erwiniaceae;g__Pantoea;s__Pantoea_deleyi
PB3_gene_2923	134	ORM85042.1	167	134/134(100.00)	6.78e-96	flagellar regulatory protein FliZ [Pantoea deleyi]	470932	d__Bacteria;k__norank;p__Proteobacteria;c__Gammaproteobacteria;o__Enterobacterales;f__Erwiniaceae;g__Pantoea;s__Pantoea_deleyi
PB3_gene_2930	638	WP_111142074.1	638	629/638(98.59)	0	DNA topoisomerase III [Pantoea sp. ARC607]	2027922	d__Bacteria;k__norank;p__Proteobacteria;c__Gammaproteobacteria;o__Enterobacterales;f__Erwiniaceae;g__Pantoea;s__Pantoea_sp._ARC607
PB3_gene_2937	44	PBQ25063.1	268	30/31(96.77)	3.87e-11	hypothetical protein BTO27_05540 [Wolbachia	2049495	d__Bacteria;k__norank;p__Proteobacteria;c__Alphaproteobacteria;o__Rickettsiales;f__Anaplasmataceae;g__Wolbachia;s__Wolbachia_pipientis
PB3_gene_2948	382	ORM76200.1	382	381/382(99.74)	3.61e-282	murein transglycosylase A [Pantoea deleyi]	470932	d__Bacteria;k__norank;p__Proteobacteria;c__Gammaproteobacteria;o__Enterobacterales;f__Erwiniaceae;g__Pantoea;s__Pantoea_deleyi

PB3_gene_2949	270	WP_111140823.1	270	263/270(97.41)	5.07e-187	tRNA cyclic N6-threonylcarbamoyladenosine(37) synthase TcdA [Pantoea sp. ARC607]	2027922	d__Bacteria;k__norank;p__Proteobacteria;c__Gammaproteobacteria;o__Enterobacterales;f__Erwiniaceae;g__Pantoea;s__Pantoea_sp._ARC607
PB3_gene_2950	151	WP_111140822.1	151	144/151(95.36)	2.64e-94	cysteine desulfurase sulfur acceptor subunit CsdE [Pantoea	2027922	d__Bacteria;k__norank;p__Proteobacteria;c__Gammaproteobacteria;o__Enterobacterales;f__Erwiniaceae;g__Pantoea;s__Pantoea_sp._ARC607
PB3_gene_2956	265	ORM80489.1	251	243/251(96.81)	5.00e-179	flap endonuclease Xni [Pantoea deleyi]	470932	d__Bacteria;k__norank;p__Proteobacteria;c__Gammaproteobacteria;o__Enterobacterales;f__Erwiniaceae;g__Pantoea;s__Pantoea_deleyi
PB3_gene_2957	281	WP_111142269.1	289	270/279(96.77)	5.04e-199	AraC family transcriptional regulator [Pantoea sp. ARC607]	2027922	d__Bacteria;k__norank;p__Proteobacteria;c__Gammaproteobacteria;o__Enterobacterales;f__Erwiniaceae;g__Pantoea;s__Pantoea_sp._ARC607
PB3_gene_2963	268	ORM81532.1	268	262/268(97.76)	2.89e-176	hypothetical protein HA42_10300 [Pantoea deleyi]	470932	d__Bacteria;k__norank;p__Proteobacteria;c__Gammaproteobacteria;o__Enterobacterales;f__Erwiniaceae;g__Pantoea;s__Pantoea_deleyi
PB3_gene_2965	261	WP_111141176.1	261	255/261(97.70)	2.57e-171	IclR family transcriptional regulator [Pantoea sp. ARC607]	2027922	d__Bacteria;k__norank;p__Proteobacteria;c__Gammaproteobacteria;o__Enterobacterales;f__Erwiniaceae;g__Pantoea;s__Pantoea_sp._ARC607
PB3_gene_2966	2320	ARF52071.1	1856	509/1371(37.13)	6.44e-253	hypothetical protein DSJ_22655 (plasmid) [Pantoea stewartii subsp. stewartii DC283]	660596	d__Bacteria;k__norank;p__Proteobacteria;c__Gammaproteobacteria;o__Enterobacterales;f__Erwiniaceae;g__Pantoea;s__Pantoea_stewartii
PB3_gene_2986	253	ORM79872.1	253	247/253(97.63)	6.74e-188	hypothetical protein HA42_15045 [Pantoea deleyi]	470932	d__Bacteria;k__norank;p__Proteobacteria;c__Gammaproteobacteria;o__Enterobacterales;f__Erwiniaceae;g__Pantoea;s__Pantoea_deleyi
PB3_gene_2988	322	ORM79874.1	321	319/321(99.38)	5.39e-217	hypothetical protein HA42_15055 [Pantoea deleyi]	470932	d__Bacteria;k__norank;p__Proteobacteria;c__Gammaproteobacteria;o__Enterobacterales;f__Erwiniaceae;g__Pantoea;s__Pantoea_deleyi
PB3_gene_2991	261	ORM79876.1	261	253/261(96.93)	7.25e-187	hypothetical protein HA42_15070 [Pantoea deleyi]	470932	d__Bacteria;k__norank;p__Proteobacteria;c__Gammaproteobacteria;o__Enterobacterales;f__Erwiniaceae;g__Pantoea;s__Pantoea_deleyi
PB3_gene_3008	485	ORM83851.1	485	483/485(99.59)	0	NADH-quinone oxidoreductase subunit N [Pantoea deleyi]	470932	d__Bacteria;k__norank;p__Proteobacteria;c__Gammaproteobacteria;o__Enterobacterales;f__Erwiniaceae;g__Pantoea;s__Pantoea_deleyi
PB3_gene_3012	204	ADO10728.1	204	196/204(96.08)	3.31e-132	HAM1 protein [Pantoea vagans C9-1]	712898	d__Bacteria;k__norank;p__Proteobacteria;c__Gammaproteobacteria;o__Enterobacterales;f__Erwiniaceae;g__Pantoea;s__Pantoea_vagans
PB3_gene_3020	485	ORM84920.1	485	479/484(98.97)	0	lytic transglycosylase F [Pantoea deleyi]	470932	d__Bacteria;k__norank;p__Proteobacteria;c__Gammaproteobacteria;o__Enterobacterales;f__Erwiniaceae;g__Pantoea;s__Pantoea_deleyi
PB3_gene_3037	348	WP_111142051.1	784	274/285(96.14)	1.93e-184	methionine synthase, partial [Pantoea sp. ARC607]	2027922	d__Bacteria;k__norank;p__Proteobacteria;c__Gammaproteobacteria;o__Enterobacterales;f__Erwiniaceae;g__Pantoea;s__Pantoea_sp._ARC607
PB3_gene_3044	589	ORM86031.1	589	584/589(99.15)	0	GMC family oxidoreductase [Pantoea deleyi]	470932	d__Bacteria;k__norank;p__Proteobacteria;c__Gammaproteobacteria;o__Enterobacterales;f__Erwiniaceae;g__Pantoea;s__Pantoea_deleyi
PB3_gene_3047	211	WP_111138519.1	211	207/211(98.10)	3.34e-149	hypothetical protein [Pantoea sp. ARC607]	2027922	d__Bacteria;k__norank;p__Proteobacteria;c__Gammaproteobacteria;o__Enterobacterales;f__Erwiniaceae;g__Pantoea;s__Pantoea_sp._ARC607
PB3_gene_3048	169	WP_111138518.1	169	165/169(97.63)	3.24e-90	DUF2501 domain-containing protein [Pantoea sp. ARC607]	2027922	d__Bacteria;k__norank;p__Proteobacteria;c__Gammaproteobacteria;o__Enterobacterales;f__Erwiniaceae;g__Pantoea;s__Pantoea_sp._ARC607
PB3_gene_3052	298	SUE07216.1	433	274/298(91.95)	1.63e-190	Multidrug-efflux transporter MexB [Pantoea agglomerans]	549	d__Bacteria;k__norank;p__Proteobacteria;c__Gammaproteobacteria;o__Enterobacterales;f__Erwiniaceae;g__Pantoea;s__Pantoea_agglomerans
PB3_gene_3064	241	WP_111140097.1	241	241/241(100.00)	6.22e-178	bifunctional 2-polyprenyl-6-hydroxyphenol methylase/3-demethylubiquinol 3-O-methyltransferase UbiG [Pantoea sp. ARC607]	2027922	d__Bacteria;k__norank;p__Proteobacteria;c__Gammaproteobacteria;o__Enterobacterales;f__Erwiniaceae;g__Pantoea;s__Pantoea_sp._ARC607
PB3_gene_3070	449	ORM83385.1	449	448/449(99.78)	0	acetyl-CoA carboxylase biotin carboxylase subunit [Pantoea	470932	d__Bacteria;k__norank;p__Proteobacteria;c__Gammaproteobacteria;o__Enterobacterales;f__Erwiniaceae;g__Pantoea;s__Pantoea_deleyi
PB3_gene_3072	172	ELP22930.1	172	164/172(95.35)	6.73e-114	3-dehydroquinate dehydratase II [Pantoea agglomerans 299R]	1261128	d__Bacteria;k__norank;p__Proteobacteria;c__Gammaproteobacteria;o__Enterobacterales;f__Erwiniaceae;g__Pantoea;s__Pantoea_agglomerans
PB3_gene_3080	303	ADO11848.1	328	272/300(90.67)	6.41e-192	putative ribokinase [Pantoea vagans C9-1]	712898	d__Bacteria;k__norank;p__Proteobacteria;c__Gammaproteobacteria;o__Enterobacterales;f__Erwiniaceae;g__Pantoea;s__Pantoea_vagans

PB3_gene_3081	181	WP_039339103.1	316	176/181(97.24)	9.49e-117	MULTISPECIES: sugar ABC transporter substrate-binding protein [Pantoea]	53335	d__Bacteria;k__norank;p__Proteobacteria;c__Gammaproteobacteria;o__Enterobacterales;f__Erwiniaceae;g__Pantoea;s__norank
PB3_gene_3100	706	ORM80455.1	706	696/706(98.58)	0	type VI secretion system protein ImpL [Pantoea deleyi]	470932	d__Bacteria;k__norank;p__Proteobacteria;c__Gammaproteobacteria;o__Enterobacterales;f__Erwiniaceae;g__Pantoea;s__Pantoea_deleyi
PB3_gene_3105	244	WP_033732358.1	217	181/215(84.19)	6.82e-127	ankyrin repeat domain-containing protein [Pantoea vagans]	470934	d__Bacteria;k__norank;p__Proteobacteria;c__Gammaproteobacteria;o__Enterobacterales;f__Erwiniaceae;g__Pantoea;s__Pantoea_vagans
PB3_gene_3106	1315	EFM17528.1	1345	1096/1217(90.06)	0	YD repeat protein [Pantoea sp. aB]	517433	d__Bacteria;k__norank;p__Proteobacteria;c__Gammaproteobacteria;o__Enterobacterales;f__Erwiniaceae;g__Pantoea;s__Pantoea_sp._aB
PB3_gene_3111	316	WP_098051585.1	319	230/316(72.78)	1.47e-159	hypothetical protein [Pantoea agglomerans]	None	Unclassified
PB3_gene_3112	225	ORM78384.1	222	218/222(98.20)	1.17e-162	hypothetical protein HA42_16720 [Pantoea deleyi]	470932	d__Bacteria;k__norank;p__Proteobacteria;c__Gammaproteobacteria;o__Enterobacterales;f__Erwiniaceae;g__Pantoea;s__Pantoea_deleyi
PB3_gene_3115	464	ORM78389.1	482	415/441(94.10)	9.75e-301	DNA-binding protein [Pantoea deleyi]	470932	d__Bacteria;k__norank;p__Proteobacteria;c__Gammaproteobacteria;o__Enterobacterales;f__Erwiniaceae;g__Pantoea;s__Pantoea_deleyi
PB3_gene_3118	81	ORM81414.1	74	66/74(89.19)	1.42e-39	iron transporter [Pantoea deleyi]	470932	d__Bacteria;k__norank;p__Proteobacteria;c__Gammaproteobacteria;o__Enterobacterales;f__Erwiniaceae;g__Pantoea;s__Pantoea_deleyi
PB3_gene_3128	469	ORM80980.1	469	457/469(97.44)	0	MFS transporter [Pantoea deleyi]	470932	d__Bacteria;k__norank;p__Proteobacteria;c__Gammaproteobacteria;o__Enterobacterales;f__Erwiniaceae;g__Pantoea;s__Pantoea_deleyi
PB3_gene_3137	180	WP_111139858.1	180	178/180(98.89)	7.24e-131	isopentenyl-diphosphate Delta-isomerase [Pantoea sp. ARC607]	2027922	d__Bacteria;k__norank;p__Proteobacteria;c__Gammaproteobacteria;o__Enterobacterales;f__Erwiniaceae;g__Pantoea;s__Pantoea_sp._ARC607
PB3_gene_3139	494	ORM80610.1	490	484/490(98.78)	0	MFS transporter [Pantoea deleyi]	470932	d__Bacteria;k__norank;p__Proteobacteria;c__Gammaproteobacteria;o__Enterobacterales;f__Erwiniaceae;g__Pantoea;s__Pantoea_deleyi
PB3_gene_3141	391	WP_111139860.1	391	374/389(96.14)	3.88e-245	ABC transporter permease [Pantoea sp. ARC607]	2027922	d__Bacteria;k__norank;p__Proteobacteria;c__Gammaproteobacteria;o__Enterobacterales;f__Erwiniaceae;g__Pantoea;s__Pantoea_sp._ARC607
PB3_gene_3143	508	ORM81341.1	506	504/506(99.60)	0	aldehyde dehydrogenase [Pantoea deleyi]	470932	d__Bacteria;k__norank;p__Proteobacteria;c__Gammaproteobacteria;o__Enterobacterales;f__Erwiniaceae;g__Pantoea;s__Pantoea_deleyi
PB3_gene_3144	187	ORM81340.1	187	180/187(96.26)	8.35e-129	methyltransferase [Pantoea deleyi]	470932	d__Bacteria;k__norank;p__Proteobacteria;c__Gammaproteobacteria;o__Enterobacterales;f__Erwiniaceae;g__Pantoea;s__Pantoea_deleyi
PB3_gene_3154	355	AGJ98927.1	367	350/355(98.59)	7.39e-237	Rod shape-determining protein RodA [Wolbachia endosymbiont of Drosophila simulans wNo]	1236908	d__Bacteria;k__norank;p__Proteobacteria;c__Alphaproteobacteria;o__Rickettsiales;f__Anaplasmataceae;g__Wolbachia;s__Wolbachia_endosymbiont_of_Drosophila_simulans
PB3_gene_3166	395	ORM85219.1	395	386/395(97.72)	5.92e-274	diguanylate phosphodiesterase [Pantoea deleyi]	470932	d__Bacteria;k__norank;p__Proteobacteria;c__Gammaproteobacteria;o__Enterobacterales;f__Erwiniaceae;g__Pantoea;s__Pantoea_deleyi
PB3_gene_3171	378	ORM81989.1	378	366/378(96.83)	4.31e-272	oxidoreductase [Pantoea deleyi]	470932	d__Bacteria;k__norank;p__Proteobacteria;c__Gammaproteobacteria;o__Enterobacterales;f__Erwiniaceae;g__Pantoea;s__Pantoea_deleyi
PB3_gene_3174	201	ORM81986.1	201	197/201(98.01)	1.01e-145	undecaprenyl-diphosphate phosphatase [Pantoea deleyi]	470932	d__Bacteria;k__norank;p__Proteobacteria;c__Gammaproteobacteria;o__Enterobacterales;f__Erwiniaceae;g__Pantoea;s__Pantoea_deleyi
PB3_gene_3177	634	ORM79046.1	634	631/634(99.53)	0	penicillin-binding protein 2 [Pantoea deleyi]	470932	d__Bacteria;k__norank;p__Proteobacteria;c__Gammaproteobacteria;o__Enterobacterales;f__Erwiniaceae;g__Pantoea;s__Pantoea_deleyi
PB3_gene_3179	364	WP_111139024.1	362	358/364(98.35)	7.22e-236	endolytic peptidoglycan transglycosylase RlpA [Pantoea sp. ARC607]	2027922	d__Bacteria;k__norank;p__Proteobacteria;c__Gammaproteobacteria;o__Enterobacterales;f__Erwiniaceae;g__Pantoea;s__Pantoea_sp._ARC607
PB3_gene_3190	188	ORM80588.1	187	186/187(99.47)	2.68e-123	alkyl hydroperoxide reductase [Pantoea deleyi]	470932	d__Bacteria;k__norank;p__Proteobacteria;c__Gammaproteobacteria;o__Enterobacterales;f__Erwiniaceae;g__Pantoea;s__Pantoea_deleyi
PB3_gene_3194	201	WP_111141750.1	208	198/201(98.51)	8.40e-142	serine protease [Pantoea sp. ARC607]	2027922	d__Bacteria;k__norank;p__Proteobacteria;c__Gammaproteobacteria;o__Enterobacterales;f__Erwiniaceae;g__Pantoea;s__Pantoea_sp._ARC607
PB3_gene_3211	415	WP_111138408.1	415	403/415(97.11)	1.19e-290	extracellular solute-binding protein [Pantoea sp. ARC607]	2027922	d__Bacteria;k__norank;p__Proteobacteria;c__Gammaproteobacteria;o__Enterobacterales;f__Erwiniaceae;g__Pantoea;s__Pantoea_sp._ARC607
PB3_gene_3214	46	ELP23126.1	51	36/40(90.00)	2.46e-09	hypothetical protein F385_3974 [Pantoea agglomerans 299R]	1261128	d__Bacteria;k__norank;p__Proteobacteria;c__Gammaproteobacteria;o__Enterobacterales;f__Erwiniaceae;g__Pantoea;s__Pantoea_agglomerans

PB3_gene_3220	942	WP_098051890.1	974	795/946(84.04)	0	cellulose biosynthesis cyclic di-GMP-binding regulatory protein BcsB [Pantoea agglomerans]	None	Unclassified
PB3_gene_3223	195	WP_111142068.1	202	166/203(81.77)	6.44e-102	cellulose biosynthesis protein BcsO [Pantoea sp. ARC607]	2027922	d__Bacteria;k__norank;p__Proteobacteria;c__Gammaproteobacteria;o__Enterobacterales;f__Erwiniaceae;g__Pantoea;s__Pantoea_sp._ARC607
PB3_gene_3226	289	WP_111139710.1	289	288/289(99.65)	3.00e-209	phosphoribulokinase [Pantoea sp. ARC607]	2027922	d__Bacteria;k__norank;p__Proteobacteria;c__Gammaproteobacteria;o__Enterobacterales;f__Erwiniaceae;g__Pantoea;s__Pantoea_sp._ARC607
PB3_gene_3233	784	ADO08374.1	886	771/784(98.34)	0	aconitate hydratase [Pantoea vagans C9-1]	712898	d__Bacteria;k__norank;p__Proteobacteria;c__Gammaproteobacteria;o__Enterobacterales;f__Erwiniaceae;g__Pantoea;s__Pantoea_vagans
PB3_gene_3244	129	AAA24853.1	301	128/129(99.22)	1.91e-79	functionally truncted p-protein [Pantoea agglomerans]	549	d__Bacteria;k__norank;p__Proteobacteria;c__Gammaproteobacteria;o__Enterobacterales;f__Erwiniaceae;g__Pantoea;s__Pantoea_agglomerans
PB3_gene_3253	593	WP_111141625.1	601	580/593(97.81)	0	NADPH-dependent assimilatory sulfite reductase flavoprotein subunit [Pantoea sp. ARC607]	2027922	d__Bacteria;k__norank;p__Proteobacteria;c__Gammaproteobacteria;o__Enterobacterales;f__Erwiniaceae;g__Pantoea;s__Pantoea_sp._ARC607
PB3_gene_3276	646	ORM82484.1	646	634/646(98.14)	0	2',3'-cyclic-nucleotide 2'-phosphodiesterase [Pantoea	470932	d__Bacteria;k__norank;p__Proteobacteria;c__Gammaproteobacteria;o__Enterobacterales;f__Erwiniaceae;g__Pantoea;s__Pantoea_deleyi
PB3_gene_3279	149	ORM82487.1	149	149/149(100.00)	4.59e-94	50S ribosomal protein L9 [Pantoea deleyi]	470932	d__Bacteria;k__norank;p__Proteobacteria;c__Gammaproteobacteria;o__Enterobacterales;f__Erwiniaceae;g__Pantoea;s__Pantoea_deleyi
PB3_gene_3284	447	WP_111139336.1	662	406/445(91.24)	7.62e-291	tRNA(Met) cytidine acetyltransferase [Pantoea sp. ARC607]	2027922	d__Bacteria;k__norank;p__Proteobacteria;c__Gammaproteobacteria;o__Enterobacterales;f__Erwiniaceae;g__Pantoea;s__Pantoea_sp._ARC607
PB3_gene_3291	215	SUB15811.1	238	189/219(86.30)	8.92e-104	Uncharacterised protein [Pantoea agglomerans]	549	d__Bacteria;k__norank;p__Proteobacteria;c__Gammaproteobacteria;o__Enterobacterales;f__Erwiniaceae;g__Pantoea;s__Pantoea_agglomerans
PB3_gene_3294	885	ORM77129.1	885	877/885(99.10)	0	phosphotransferase RcsD [Pantoea deleyi]	470932	d__Bacteria;k__norank;p__Proteobacteria;c__Gammaproteobacteria;o__Enterobacterales;f__Erwiniaceae;g__Pantoea;s__Pantoea_deleyi
PB3_gene_3298	462	WP_111139282.1	462	455/462(98.48)	0	sugar porter family MFS transporter [Pantoea sp. ARC607]	2027922	d__Bacteria;k__norank;p__Proteobacteria;c__Gammaproteobacteria;o__Enterobacterales;f__Erwiniaceae;g__Pantoea;s__Pantoea_sp._ARC607
PB3_gene_3310	73	ORM86025.1	340	70/73(95.89)	3.44e-40	cytochrome-c peroxidase [Pantoea deleyi]	470932	d__Bacteria;k__norank;p__Proteobacteria;c__Gammaproteobacteria;o__Enterobacterales;f__Erwiniaceae;g__Pantoea;s__Pantoea_deleyi
PB3_gene_3319	647	AGJ99189.1	653	578/650(88.92)	0	hypothetical protein wNo_07950 [Wolbachia endosymbiont of Drosophila simulans wNo]	1236908	d__Bacteria;k__norank;p__Proteobacteria;c__Alphaproteobacteria;o__Rickettsiales;f__Anaplasmataceae;g__Wolbachia;s__Wolbachia_endosymbiont_of_Drosophila_simulans
PB3_gene_3333	490	BAH22302.1	479	416/489(85.07)	4.64e-254	hypothetical protein [Wolbachia endosymbiont of Cadra cautella]	190193	d__Bacteria;k__norank;p__Proteobacteria;c__Alphaproteobacteria;o__Rickettsiales;f__Anaplasmataceae;g__Wolbachia;s__Wolbachia_endosymbiont_of_Cadra_cautella
PB3_gene_3351	657	WP_111138319.1	657	634/657(96.50)	0	chemoreceptor [Pantoea sp. ARC607]	2027922	d__Bacteria;k__norank;p__Proteobacteria;c__Gammaproteobacteria;o__Enterobacterales;f__Erwiniaceae;g__Pantoea;s__Pantoea_sp._ARC607
PB3_gene_3352	115	ORM81425.1	98	25/28(89.29)	2.86e-06	hypothetical protein HA42_10860 [Pantoea deleyi]	470932	d__Bacteria;k__norank;p__Proteobacteria;c__Gammaproteobacteria;o__Enterobacterales;f__Erwiniaceae;g__Pantoea;s__Pantoea_deleyi
PB3_gene_3356	226	WP_122889988.1	226	207/226(91.59)	2.82e-153	hypothetical protein [[Curtobacterium] plantarum]	221276	d__Bacteria;k__norank;p__Proteobacteria;c__Gammaproteobacteria;o__Enterobacterales;f__Erwiniaceae;g__Pantoea;s__[Curtobacterium]_plantarum
PB3_gene_3358	102	WP_122889986.1	102	95/102(93.14)	2.83e-59	hypothetical protein [[Curtobacterium] plantarum]	221276	d__Bacteria;k__norank;p__Proteobacteria;c__Gammaproteobacteria;o__Enterobacterales;f__Erwiniaceae;g__Pantoea;s__[Curtobacterium]_plantarum
PB3_gene_3359	120	EFM17738.1	120	116/120(96.67)	4.64e-89	hypothetical protein PanABDRAFT_4249 [Pantoea sp. aB]	517433	d__Bacteria;k__norank;p__Proteobacteria;c__Gammaproteobacteria;o__Enterobacterales;f__Erwiniaceae;g__Pantoea;s__Pantoea_sp._aB
PB3_gene_3364	1147	ORM82361.1	1147	1138/1147(99.22)	0	transcription-repair coupling factor [Pantoea deleyi]	470932	d__Bacteria;k__norank;p__Proteobacteria;c__Gammaproteobacteria;o__Enterobacterales;f__Erwiniaceae;g__Pantoea;s__Pantoea_deleyi

PB3_gene_3366	1333	WP_101241016.1	2357	752/1308(57.49)	0	autotransporter outer membrane beta-barrel domain-containing protein [Enterobacterales bacterium CwR94]	None	Unclassified
PB3_gene_3369	158	WP_046103198.1	207	129/131(98.47)	2.18e-82	response regulator [Pantoea anthophila]	470931	d__Bacteria;k__norank;p__Proteobacteria;c__Gammaproteobacteria;o__Enterobacterales;f__Erwiniaceae;g__Pantoea;s__Pantoea_anthophila
PB3_gene_3373	254	ORM82909.1	247	240/247(97.17)	8.17e-172	hypothetical protein HA42_07210 [Pantoea deleyi]	470932	d__Bacteria;k__norank;p__Proteobacteria;c__Gammaproteobacteria;o__Enterobacterales;f__Erwiniaceae;g__Pantoea;s__Pantoea_deleyi
PB3_gene_3381	389	ORM85294.1	461	385/389(98.97)	4.33e-270	type II secretion system protein GspE [Pantoea deleyi]	470932	d__Bacteria;k__norank;p__Proteobacteria;c__Gammaproteobacteria;o__Enterobacterales;f__Erwiniaceae;g__Pantoea;s__Pantoea_deleyi
PB3_gene_3403	293	WP_111141315.1	258	252/258(97.67)	2.42e-163	DeoR/GlpR transcriptional regulator [Pantoea sp. ARC607]	2027922	d__Bacteria;k__norank;p__Proteobacteria;c__Gammaproteobacteria;o__Enterobacterales;f__Erwiniaceae;g__Pantoea;s__Pantoea_sp._ARC607
PB3_gene_3409	383	WP_111138392.1	383	361/383(94.26)	2.00e-259	amidohydrolase [Pantoea sp. ARC607]	2027922	d__Bacteria;k__norank;p__Proteobacteria;c__Gammaproteobacteria;o__Enterobacterales;f__Erwiniaceae;g__Pantoea;s__Pantoea_sp._ARC607
PB3_gene_3411	313	WP_111138394.1	313	300/313(95.85)	1.58e-207	ABC transporter substrate-binding protein [Pantoea sp.	2027922	d__Bacteria;k__norank;p__Proteobacteria;c__Gammaproteobacteria;o__Enterobacterales;f__Erwiniaceae;g__Pantoea;s__Pantoea_sp._ARC607
PB3_gene_3418	296	WP_061061265.1	293	283/293(96.59)	2.56e-206	glucose-1-phosphate thymidylyltransferase [Pantoea vagans]	53335	d__Bacteria;k__norank;p__Proteobacteria;c__Gammaproteobacteria;o__Enterobacterales;f__Erwiniaceae;g__Pantoea;s__norank
PB3_gene_3428	1253	ORM78066.1	1253	1243/1253(99.20)	0	nitrate reductase subunit alpha [Pantoea deleyi]	470932	d__Bacteria;k__norank;p__Proteobacteria;c__Gammaproteobacteria;o__Enterobacterales;f__Erwiniaceae;g__Pantoea;s__Pantoea_deleyi
PB3_gene_3446	87	EFM17499.1	90	84/87(96.55)	1.28e-51	type VI secretion system Vgr family protein, partial [Pantoea	517433	d__Bacteria;k__norank;p__Proteobacteria;c__Gammaproteobacteria;o__Enterobacterales;f__Erwiniaceae;g__Pantoea;s__Pantoea_sp._aB
PB3_gene_3448	869	WP_111140767.1	869	853/869(98.16)	0	type VI secretion system ATPase TssH [Pantoea sp. ARC607]	2027922	d__Bacteria;k__norank;p__Proteobacteria;c__Gammaproteobacteria;o__Enterobacterales;f__Erwiniaceae;g__Pantoea;s__Pantoea_sp._ARC607
PB3_gene_3449	348	WP_111140768.1	348	338/348(97.13)	9.46e-254	type VI secretion system baseplate subunit TssG [Pantoea	2027922	d__Bacteria;k__norank;p__Proteobacteria;c__Gammaproteobacteria;o__Enterobacterales;f__Erwiniaceae;g__Pantoea;s__Pantoea_sp._ARC607
PB3_gene_3450	112	WP_111140769.1	623	111/112(99.11)	3.28e-67	type VI secretion system baseplate subunit TssF [Pantoea	2027922	d__Bacteria;k__norank;p__Proteobacteria;c__Gammaproteobacteria;o__Enterobacterales;f__Erwiniaceae;g__Pantoea;s__Pantoea_sp._ARC607
PB3_gene_3456	161	ORM81542.1	161	152/161(94.41)	1.55e-100	cytochrome c biogenesis protein CcmE [Pantoea deleyi]	470932	d__Bacteria;k__norank;p__Proteobacteria;c__Gammaproteobacteria;o__Enterobacterales;f__Erwiniaceae;g__Pantoea;s__Pantoea_deleyi
PB3_gene_3459	504	ORM82478.1	1255	495/504(98.21)	0	translocation and assembly module TamB [Pantoea deleyi]	470932	d__Bacteria;k__norank;p__Proteobacteria;c__Gammaproteobacteria;o__Enterobacterales;f__Erwiniaceae;g__Pantoea;s__Pantoea_deleyi
PB3_gene_3460	571	ORM82479.1	579	569/571(99.65)	0	hypothetical protein HA42_08250 [Pantoea deleyi]	470932	d__Bacteria;k__norank;p__Proteobacteria;c__Gammaproteobacteria;o__Enterobacterales;f__Erwiniaceae;g__Pantoea;s__Pantoea_deleyi
PB3_gene_3461	44	PQL26544.1	105	36/43(83.72)	2.98e-16	hypothetical protein C5L22_19960 [Pantoea ananatis]	553	d__Bacteria;k__norank;p__Proteobacteria;c__Gammaproteobacteria;o__Enterobacterales;f__Erwiniaceae;g__Pantoea;s__Pantoea_ananatis
PB3_gene_3464	125	WP_111141496.1	425	124/125(99.20)	1.32e-82	FAD-binding oxidoreductase [Pantoea sp. ARC607]	2027922	d__Bacteria;k__norank;p__Proteobacteria;c__Gammaproteobacteria;o__Enterobacterales;f__Erwiniaceae;g__Pantoea;s__Pantoea_sp._ARC607
PB3_gene_3467	471	ORM85661.1	471	467/471(99.15)	0	gamma-glutamylputrescine synthetase [Pantoea deleyi]	470932	d__Bacteria;k__norank;p__Proteobacteria;c__Gammaproteobacteria;o__Enterobacterales;f__Erwiniaceae;g__Pantoea;s__Pantoea_deleyi
PB3_gene_3469	187	ORM85663.1	253	185/187(98.93)	8.27e-126	DeoR family transcriptional regulator [Pantoea deleyi]	470932	d__Bacteria;k__norank;p__Proteobacteria;c__Gammaproteobacteria;o__Enterobacterales;f__Erwiniaceae;g__Pantoea;s__Pantoea_deleyi
PB3_gene_3470	776	ORM80795.1	793	768/776(98.97)	0	phosphoenolpyruvate synthase [Pantoea deleyi]	470932	d__Bacteria;k__norank;p__Proteobacteria;c__Gammaproteobacteria;o__Enterobacterales;f__Erwiniaceae;g__Pantoea;s__Pantoea_deleyi
PB3_gene_3476	705	WP_085016763.1	706	705/705(100.00)	0	integrase [Cronobacter sakazakii]	None	Unclassified
PB3_gene_3478	126	ASN13839.1	126	125/125(100.00)	3.90e-84	fluoride ion transporter CrcB [Pantoea ananatis]	553	d__Bacteria;k__norank;p__Proteobacteria;c__Gammaproteobacteria;o__Enterobacterales;f__Erwiniaceae;g__Pantoea;s__Pantoea_ananatis
PB3_gene_3514	408	WP_111140110.1	907	399/408(97.79)	8.37e-278	NADH-quinone oxidoreductase subunit NuoG [Pantoea sp.	2027922	d__Bacteria;k__norank;p__Proteobacteria;c__Gammaproteobacteria;o__Enterobacterales;f__Erwiniaceae;g__Pantoea;s__Pantoea_sp._ARC607

PB3_gene_3520	226	ORM82826.1	310	225/226(99.56)	2.45e-153	[acyl-carrier-protein] S-malonyltransferase [Pantoea	470932	d__Bacteria;k__norank;p__Proteobacteria;c__Gammaproteobacteria;o__Enterobacterales;f__Erwiniaceae;g__Pantoea;s__Pantoea_deleyi
PB3_gene_3523	173	ORM82824.1	173	173/173(100.00)	1.59e-117	hypothetical protein HA42_07445 [Pantoea deleyi]	470932	d__Bacteria;k__norank;p__Proteobacteria;c__Gammaproteobacteria;o__Enterobacterales;f__Erwiniaceae;g__Pantoea;s__Pantoea_deleyi
PB3_gene_3531	89	WP_089414212.1	82	36/44(81.82)	3.54e-16	hypothetical protein [Pantoea agglomerans]	None	Unclassified
PB3_gene_3534	333	ORM77352.1	338	331/333(99.40)	7.81e-217	sugar ABC transporter permease [Pantoea deleyi]	470932	d__Bacteria;k__norank;p__Proteobacteria;c__Gammaproteobacteria;o__Enterobacterales;f__Erwiniaceae;g__Pantoea;s__Pantoea_deleyi
PB3_gene_3544	765	WP_111138491.1	763	736/762(96.59)	0	phosphatidylglycerol--membrane-oligosaccharide glycerophosphotransferase [Pantoea sp. ARC607]	2027922	d__Bacteria;k__norank;p__Proteobacteria;c__Gammaproteobacteria;o__Enterobacterales;f__Erwiniaceae;g__Pantoea;s__Pantoea_sp._ARC607
PB3_gene_3567	735	WP_039658728.1	735	686/735(93.33)	0	ferrichrome porin FhuA [Pantoea sp. MBLJ3]	1562889	d__Bacteria;k__norank;p__Proteobacteria;c__Gammaproteobacteria;o__Enterobacterales;f__Erwiniaceae;g__Pantoea;s__Pantoea_sp._MBLJ3
PB3_gene_3577	486	ORM86118.1	486	485/486(99.79)	0	cardiolipin synthase [Pantoea deleyi]	470932	d__Bacteria;k__norank;p__Proteobacteria;c__Gammaproteobacteria;o__Enterobacterales;f__Erwiniaceae;g__Pantoea;s__Pantoea_deleyi
PB3_gene_3583	143	ORM86112.1	140	140/140(100.00)	1.12e-95	acyl-CoA esterase [Pantoea deleyi]	470932	d__Bacteria;k__norank;p__Proteobacteria;c__Gammaproteobacteria;o__Enterobacterales;f__Erwiniaceae;g__Pantoea;s__Pantoea_deleyi
PB3_gene_3588	418	WP_111140851.1	418	395/418(94.50)	2.66e-268	four-carbon acid sugar kinase family protein [Pantoea sp.	2027922	d__Bacteria;k__norank;p__Proteobacteria;c__Gammaproteobacteria;o__Enterobacterales;f__Erwiniaceae;g__Pantoea;s__Pantoea_sp._ARC607
PB3_gene_3594	77	SAI96001.1	95	22/39(56.41)	8.77e-06	Uncharacterised protein [Enterobacter cloacae]	550	d__Bacteria;k__norank;p__Proteobacteria;c__Gammaproteobacteria;o__Enterobacterales;f__Enterobacteriaceae;g__Enterobacter;s__Enterobacter_cloacae
PB3_gene_3604	237	CQD47026.1	274	190/237(80.17)	5.48e-126	Uncharacterised protein [Yersinia enterocolitica]	630	d__Bacteria;k__norank;p__Proteobacteria;c__Gammaproteobacteria;o__Enterobacterales;f__Yersiniaceae;g__Yersinia;s__Yersinia_enterocolitica
PB3_gene_3608	476	ORM76576.1	694	455/464(98.06)	0	TonB-dependent receptor [Pantoea deleyi]	470932	d__Bacteria;k__norank;p__Proteobacteria;c__Gammaproteobacteria;o__Enterobacterales;f__Erwiniaceae;g__Pantoea;s__Pantoea_deleyi
PB3_gene_3610	338	WP_111140379.1	338	320/338(94.67)	9.26e-240	membrane dipeptidase [Pantoea sp. ARC607]	2027922	d__Bacteria;k__norank;p__Proteobacteria;c__Gammaproteobacteria;o__Enterobacterales;f__Erwiniaceae;g__Pantoea;s__Pantoea_sp._ARC607
PB3_gene_3613	122	SJZ90335.1	122	122/122(100.00)	9.71e-83	transcriptional regulator, AraC family [Pantoea agglomerans]	470933	d__Bacteria;k__norank;p__Proteobacteria;c__Gammaproteobacteria;o__Enterobacterales;f__Erwiniaceae;g__Pantoea;s__Pantoea_eucalypti
PB3_gene_3616	763	WP_111141877.1	795	753/769(97.92)	0	glucose/quinate/shikimate family membrane-bound PQQ-dependent dehydrogenase [Pantoea sp. ARC607]	2027922	d__Bacteria;k__norank;p__Proteobacteria;c__Gammaproteobacteria;o__Enterobacterales;f__Erwiniaceae;g__Pantoea;s__Pantoea_sp._ARC607
PB3_gene_3625	139	ORM85847.1	805	136/139(97.84)	1.00e-80	sugar ABC transporter permease [Pantoea deleyi]	470932	d__Bacteria;k__norank;p__Proteobacteria;c__Gammaproteobacteria;o__Enterobacterales;f__Erwiniaceae;g__Pantoea;s__Pantoea_deleyi
PB3_gene_3628	120	WP_090965356.1	141	104/120(86.67)	3.94e-72	nucleoside triphosphatase NudI [Pantoea sp. OV426]	1881036	d__Bacteria;k__norank;p__Proteobacteria;c__Gammaproteobacteria;o__Enterobacterales;f__Erwiniaceae;g__Pantoea;s__Pantoea_sp._OV426
PB3_gene_3629	404	WP_010248194.1	404	400/404(99.01)	3.95e-313	D-galactonate dehydratase family protein [Pantoea agglomerans]	549	d__Bacteria;k__norank;p__Proteobacteria;c__Gammaproteobacteria;o__Enterobacterales;f__Erwiniaceae;g__Pantoea;s__Pantoea_agglomerans
PB3_gene_3641	232	SKA25195.1	232	232/232(100.00)	2.15e-160	SSU ribosomal protein S3P [Pantoea agglomerans]	470933	d__Bacteria;k__norank;p__Proteobacteria;c__Gammaproteobacteria;o__Enterobacterales;f__Erwiniaceae;g__Pantoea;s__Pantoea_eucalypti
PB3_gene_3644	273	ORM81244.1	273	273/273(100.00)	1.07e-194	50S ribosomal protein L2 [Pantoea deleyi]	470932	d__Bacteria;k__norank;p__Proteobacteria;c__Gammaproteobacteria;o__Enterobacterales;f__Erwiniaceae;g__Pantoea;s__Pantoea_deleyi
PB3_gene_3646	334	ORM82558.1	334	322/334(96.41)	2.29e-222	transcriptional regulator [Pantoea deleyi]	470932	d__Bacteria;k__norank;p__Proteobacteria;c__Gammaproteobacteria;o__Enterobacterales;f__Erwiniaceae;g__Pantoea;s__Pantoea_deleyi
PB3_gene_3647	633	ORM82557.1	627	605/627(96.49)	0	PTS beta-glucoside transporter subunit EIIBCA [Pantoea deleyi]	470932	d__Bacteria;k__norank;p__Proteobacteria;c__Gammaproteobacteria;o__Enterobacterales;f__Erwiniaceae;g__Pantoea;s__Pantoea_deleyi
PB3_gene_3656	226	WP_111140609.1	226	224/226(99.12)	1.26e-165	uracil-DNA glycosylase [Pantoea sp. ARC607]	2027922	d__Bacteria;k__norank;p__Proteobacteria;c__Gammaproteobacteria;o__Enterobacterales;f__Erwiniaceae;g__Pantoea;s__Pantoea_sp._ARC607

PB3_gene_3659	111	OXM17775.1	120	111/111(100.00)	2.01e-74	elongation factor Tu, partial [Pantoea sp. AV62]	1990688	d__Bacteria;k__norank;p__Proteobacteria;c__Gammaproteobacteria;o__Enterobacterales;f__Erwiniaceae;g__Pantoea;s__Pantoea_sp._AV62
PB3_gene_3661	319	ORM75628.1	319	311/319(97.49)	1.44e-222	biotin--[acetyl-CoA-carboxylase] synthetase [Pantoea deleyi]	470932	d__Bacteria;k__norank;p__Proteobacteria;c__Gammaproteobacteria;o__Enterobacterales;f__Erwiniaceae;g__Pantoea;s__Pantoea_deleyi
PB3_gene_3663	210	WP_104085965.1	486	205/210(97.62)	2.29e-140	MFS transporter [Pantoea sp. ICBG 1758]	2071682	d__Bacteria;k__norank;p__Proteobacteria;c__Gammaproteobacteria;o__Enterobacterales;f__Erwiniaceae;g__Pantoea;s__Pantoea_sp._ICBG_1758
PB3_gene_3664	468	ORM79328.1	468	458/468(97.86)	0	5,10-methylene tetrahydromethanopterin reductase [Pantoea deleyi]	470932	d__Bacteria;k__norank;p__Proteobacteria;c__Gammaproteobacteria;o__Enterobacterales;f__Erwiniaceae;g__Pantoea;s__Pantoea_deleyi
PB3_gene_3665	470	WP_104085967.1	470	421/470(89.57)	1.82e-306	FAD-dependent oxidoreductase [Pantoea sp. ICBG 1758]	2071682	d__Bacteria;k__norank;p__Proteobacteria;c__Gammaproteobacteria;o__Enterobacterales;f__Erwiniaceae;g__Pantoea;s__Pantoea_sp._ICBG_1758
PB3_gene_3666	384	WP_104085968.1	384	377/384(98.18)	1.69e-272	FMNH2-dependent alkanesulfonate monooxygenase [Pantoea sp. ICBG 1758]	2071682	d__Bacteria;k__norank;p__Proteobacteria;c__Gammaproteobacteria;o__Enterobacterales;f__Erwiniaceae;g__Pantoea;s__Pantoea_sp._ICBG_1758
PB3_gene_3668	145	ORM85287.1	174	145/145(100.00)	9.96e-98	SecA regulator SecM [Pantoea deleyi]	470932	d__Bacteria;k__norank;p__Proteobacteria;c__Gammaproteobacteria;o__Enterobacterales;f__Erwiniaceae;g__Pantoea;s__Pantoea_deleyi
PB3_gene_3683	1194	WP_052264686.1	1216	1102/1194(92.29)	0	DEAD/DEAH box helicase [Wolbachia endosymbiont of Operophtera brumata]	1620420	d__Bacteria;k__norank;p__Proteobacteria;c__Alphaproteobacteria;o__Rickettsiales;f__Anaplasmataceae;g__Wolbachia;s__Wolbachia_endosymbiont_of_Operophtera_brumata
PB3_gene_3697	480	ORM86820.1	480	479/480(99.79)	0	pyruvate kinase [Pantoea deleyi]	470932	d__Bacteria;k__norank;p__Proteobacteria;c__Gammaproteobacteria;o__Enterobacterales;f__Erwiniaceae;g__Pantoea;s__Pantoea_deleyi
PB3_gene_3701	740	WP_111141522.1	1209	724/740(97.84)	0	urea carboxylase [Pantoea sp. ARC607]	2027922	d__Bacteria;k__norank;p__Proteobacteria;c__Gammaproteobacteria;o__Enterobacterales;f__Erwiniaceae;g__Pantoea;s__Pantoea_sp._ARC607
PB3_gene_3702	595	WP_111141376.1	595	577/595(96.97)	0	allophanate hydrolase [Pantoea sp. ARC607]	2027922	d__Bacteria;k__norank;p__Proteobacteria;c__Gammaproteobacteria;o__Enterobacterales;f__Erwiniaceae;g__Pantoea;s__Pantoea_sp._ARC607
PB3_gene_3714	422	RZK09001.1	422	387/422(91.71)	1.04e-283	translesion error-prone DNA polymerase V subunit UmuC [Pantoea sp.]	69393	d__Bacteria;k__norank;p__Proteobacteria;c__Gammaproteobacteria;o__Enterobacterales;f__Erwiniaceae;g__Pantoea;s__Pantoea_sp.
PB3_gene_3720	704	ABV16057.1	847	402/704(57.10)	2.60e-266	hypothetical protein CKO_05014 [Citrobacter koseri ATCC BAA-	290338	d__Bacteria;k__norank;p__Proteobacteria;c__Gammaproteobacteria;o__Enterobacterales;f__Enterobacteriaceae;g__Citrobacter;s__Citrobacter_koseri
PB3_gene_3732	544	ORM86123.1	541	525/541(97.04)	0	oligopeptide ABC transporter substrate-binding protein OppA [Pantoea deleyi]	470932	d__Bacteria;k__norank;p__Proteobacteria;c__Gammaproteobacteria;o__Enterobacterales;f__Erwiniaceae;g__Pantoea;s__Pantoea_deleyi
PB3_gene_3749	684	ORM84706.1	684	669/684(97.81)	0	DNA helicase IV [Pantoea deleyi]	470932	d__Bacteria;k__norank;p__Proteobacteria;c__Gammaproteobacteria;o__Enterobacterales;f__Erwiniaceae;g__Pantoea;s__Pantoea_deleyi
PB3_gene_3760	89	ORM76105.1	89	84/89(94.38)	7.79e-51	hypothetical protein HA42_19490 [Pantoea deleyi]	470932	d__Bacteria;k__norank;p__Proteobacteria;c__Gammaproteobacteria;o__Enterobacterales;f__Erwiniaceae;g__Pantoea;s__Pantoea_deleyi
PB3_gene_3768	707	ORM84147.1	707	689/707(97.45)	0	ligand-gated channel [Pantoea deleyi]	470932	d__Bacteria;k__norank;p__Proteobacteria;c__Gammaproteobacteria;o__Enterobacterales;f__Erwiniaceae;g__Pantoea;s__Pantoea_deleyi
PB3_gene_3769	296	ORM84163.1	296	286/296(96.62)	3.84e-195	RpiR family transcriptional regulator [Pantoea deleyi]	470932	d__Bacteria;k__norank;p__Proteobacteria;c__Gammaproteobacteria;o__Enterobacterales;f__Erwiniaceae;g__Pantoea;s__Pantoea_deleyi
PB3_gene_3776	296	ORM83151.1	454	295/296(99.66)	1.11e-197	hypothetical protein HA42_06920 [Pantoea deleyi]	470932	d__Bacteria;k__norank;p__Proteobacteria;c__Gammaproteobacteria;o__Enterobacterales;f__Erwiniaceae;g__Pantoea;s__Pantoea_deleyi
PB3_gene_3782	442	WP_123346423.1	438	310/449(69.04)	2.77e-202	MULTISPECIES: phage tail sheath family protein	2608935	d__Bacteria;k__norank;p__Proteobacteria;c__Gammaproteobacteria;o__Enterobacterales;f__Enterobacteriaceae;g__Enterobacter;s__norank
PB3_gene_3783	149	WP_123346431.1	149	134/149(89.93)	1.87e-93	MULTISPECIES: phage tail protein [Enterobacter]	2608935	d__Bacteria;k__norank;p__Proteobacteria;c__Gammaproteobacteria;o__Enterobacterales;f__Enterobacteriaceae;g__Enterobacter;s__norank
PB3_gene_3784	355	WP_123346429.1	354	239/355(67.32)	2.49e-181	MULTISPECIES: phage tail sheath family protein	2608935	d__Bacteria;k__norank;p__Proteobacteria;c__Gammaproteobacteria;o__Enterobacterales;f__Enterobacteriaceae;g__Enterobacter;s__norank

PB3_gene_3789	648	ORM82659.1	646	632/646(97.83)	0	3D-(3,5/4)-trihydroxycyclohexane-1,2-dione acylhydrolase (decyclizing)	470932	d__Bacteria;k__norank;p__Proteobacteria;c__Gammaproteobacteria;o__Enterobacterales;f__Erwiniaceae;g__Pantoea;s__Pantoea_deleyi
PB3_gene_3809	254	EFM17736.1	358	240/243(98.77)	1.01e-166	acyltransferase 3 [Pantoea sp. aB]	517433	d__Bacteria;k__norank;p__Proteobacteria;c__Gammaproteobacteria;o__Enterobacterales;f__Erwiniaceae;g__Pantoea;s__Pantoea_sp._aB
PB3_gene_3814	376	EFM17731.1	420	370/376(98.40)	1.52e-268	DNA-directed DNA polymerase [Pantoea sp. aB]	517433	d__Bacteria;k__norank;p__Proteobacteria;c__Gammaproteobacteria;o__Enterobacterales;f__Erwiniaceae;g__Pantoea;s__Pantoea_sp._aB
PB3_gene_3815	329	WP_111139942.1	320	315/320(98.44)	5.90e-218	ribokinase [Pantoea sp. ARC607]	2027922	d__Bacteria;k__norank;p__Proteobacteria;c__Gammaproteobacteria;o__Enterobacterales;f__Erwiniaceae;g__Pantoea;s__Pantoea_sp._ARC607
PB3_gene_3818	284	ORM81208.1	284	280/284(98.59)	6.45e-197	kinase [Pantoea deleyi]	470932	d__Bacteria;k__norank;p__Proteobacteria;c__Gammaproteobacteria;o__Enterobacterales;f__Erwiniaceae;g__Pantoea;s__Pantoea_deleyi
PB3_gene_3821	232	WP_111142024.1	455	218/232(93.97)	2.10e-147	hypothetical protein [Pantoea sp. ARC607]	2027922	d__Bacteria;k__norank;p__Proteobacteria;c__Gammaproteobacteria;o__Enterobacterales;f__Erwiniaceae;g__Pantoea;s__Pantoea_sp._ARC607
PB3_gene_3822	390	WP_095707233.1	390	350/389(89.97)	3.13e-239	benzoate/H(+) symporter BenE family transporter [Pantoea	470934	d__Bacteria;k__norank;p__Proteobacteria;c__Gammaproteobacteria;o__Enterobacterales;f__Erwiniaceae;g__Pantoea;s__Pantoea_vagans
PB3_gene_3823	185	WP_111142150.1	185	180/185(97.30)	8.04e-126	XRE family transcriptional regulator [Pantoea sp. ARC607]	2027922	d__Bacteria;k__norank;p__Proteobacteria;c__Gammaproteobacteria;o__Enterobacterales;f__Erwiniaceae;g__Pantoea;s__Pantoea_sp._ARC607
PB3_gene_3832	1146	ORM82821.1	1139	1058/1149(92.08)	0	ribonuclease E [Pantoea deleyi]	470932	d__Bacteria;k__norank;p__Proteobacteria;c__Gammaproteobacteria;o__Enterobacterales;f__Erwiniaceae;g__Pantoea;s__Pantoea_deleyi
PB3_gene_3836	255	WP_111140139.1	255	246/255(96.47)	1.46e-183	amidohydrolase [Pantoea sp. ARC607]	2027922	d__Bacteria;k__norank;p__Proteobacteria;c__Gammaproteobacteria;o__Enterobacterales;f__Erwiniaceae;g__Pantoea;s__Pantoea_sp._ARC607
PB3_gene_3839	417	WP_111140136.1	417	406/417(97.36)	2.93e-292	allantoate amidohydrolase [Pantoea sp. ARC607]	2027922	d__Bacteria;k__norank;p__Proteobacteria;c__Gammaproteobacteria;o__Enterobacterales;f__Erwiniaceae;g__Pantoea;s__Pantoea_sp._ARC607
PB3_gene_3848	107	WP_128080209.1	126	76/97(78.35)	2.84e-47	transposase [Wolbachia endosymbiont of Glossina morsitans morsitans]	1150948	d__Bacteria;k__norank;p__Proteobacteria;c__Alphaproteobacteria;o__Rickettsiales;f__Anaplasmataceae;g__Wolbachia;s__Wolbachia_endosymbiont_of_Glossina_morsitans_morsitans
PB3_gene_3855	341	WP_111141456.1	373	338/341(99.12)	4.51e-238	bifunctional chorismate mutase/prephenate dehydrogenase [Pantoea sp. ARC607]	2027922	d__Bacteria;k__norank;p__Proteobacteria;c__Gammaproteobacteria;o__Enterobacterales;f__Erwiniaceae;g__Pantoea;s__Pantoea_sp._ARC607
PB3_gene_3862	506	SJZ64041.1	506	506/506(100.00)	0	glucose ABC transporter ATP-binding protein /galactose ABC transporter ATP-binding protein [Pantoea agglomerans]	470933	d__Bacteria;k__norank;p__Proteobacteria;c__Gammaproteobacteria;o__Enterobacterales;f__Erwiniaceae;g__Pantoea;s__Pantoea_eucalypti
PB3_gene_3867	232	ORM81902.1	232	220/231(95.24)	5.43e-154	dethiobiotin synthase [Pantoea deleyi]	470932	d__Bacteria;k__norank;p__Proteobacteria;c__Gammaproteobacteria;o__Enterobacterales;f__Erwiniaceae;g__Pantoea;s__Pantoea_deleyi
PB3_gene_3868	458	WP_107319748.1	458	429/458(93.67)	1.35e-295	purine permease [Pantoea vagans]	470934	d__Bacteria;k__norank;p__Proteobacteria;c__Gammaproteobacteria;o__Enterobacterales;f__Erwiniaceae;g__Pantoea;s__Pantoea_vagans
PB3_gene_3870	987	ORM77084.1	987	978/987(99.09)	0	virulence factor SrfB [Pantoea deleyi]	470932	d__Bacteria;k__norank;p__Proteobacteria;c__Gammaproteobacteria;o__Enterobacterales;f__Erwiniaceae;g__Pantoea;s__Pantoea_deleyi
PB3_gene_3876	254	WP_111139144.1	254	250/254(98.43)	4.87e-177	enhanced serine sensitivity protein SseB [Pantoea sp.	2027922	d__Bacteria;k__norank;p__Proteobacteria;c__Gammaproteobacteria;o__Enterobacterales;f__Erwiniaceae;g__Pantoea;s__Pantoea_sp._ARC607
PB3_gene_3881	575	ORM81333.1	561	553/561(98.57)	0	alpha,alpha-trehalase [Pantoea deleyi]	470932	d__Bacteria;k__norank;p__Proteobacteria;c__Gammaproteobacteria;o__Enterobacterales;f__Erwiniaceae;g__Pantoea;s__Pantoea_deleyi
PB3_gene_3882	349	KEY42864.1	349	311/349(89.11)	5.03e-229	2-hydroxyacid dehydrogenase [Pantoea agglomerans]	549	d__Bacteria;k__norank;p__Proteobacteria;c__Gammaproteobacteria;o__Enterobacterales;f__Erwiniaceae;g__Pantoea;s__Pantoea_agglomerans
PB3_gene_3884	104	RXG03803.1	104	98/104(94.23)	4.13e-62	hypothetical protein DB360_01880 [Enterobacter	550	d__Bacteria;k__norank;p__Proteobacteria;c__Gammaproteobacteria;o__Enterobacterales;f__Enterobacteriaceae;g__Enterobacter;s__Enterobacter_cloacae
PB3_gene_3904	442	ORM79159.1	442	438/442(99.10)	1.97e-305	thymidine phosphorylase [Pantoea deleyi]	470932	d__Bacteria;k__norank;p__Proteobacteria;c__Gammaproteobacteria;o__Enterobacterales;f__Erwiniaceae;g__Pantoea;s__Pantoea_deleyi
PB3_gene_3907	170	ORM80464.1	147	136/147(92.52)	8.95e-97	rhodanese [Pantoea deleyi]	470932	d__Bacteria;k__norank;p__Proteobacteria;c__Gammaproteobacteria;o__Enterobacterales;f__Erwiniaceae;g__Pantoea;s__Pantoea_deleyi

PB3_gene_3909	501	WP_111140442.1	501	496/501(99.00)	0	tripartite tricarboxylate transporter permease [Pantoea sp.	2027922	d__Bacteria;k__norank;p__Proteobacteria;c__Gammaproteobacteria;o__Enterobacterales;f__Erwiniaceae;g__Pantoea;s__Pantoea_sp._ARC607
PB3_gene_3914	489	WP_095706956.1	489	473/489(96.73)	0	aldehyde dehydrogenase [Pantoea vagans]	470934	d__Bacteria;k__norank;p__Proteobacteria;c__Gammaproteobacteria;o__Enterobacterales;f__Erwiniaceae;g__Pantoea;s__Pantoea_vagans
PB3_gene_3918	621	WP_058958430.1	715	597/621(96.14)	0	type VI secretion system tip protein VgrG [Type-F symbiont of Plautia stali]	1560358	d__Bacteria;k__norank;p__Proteobacteria;c__Gammaproteobacteria;o__Enterobacterales;f__Enterobacteriaceae;g__norank;s__Type-F_symbiont_of_Plautia_stali
PB3_gene_3920	430	WP_104093715.1	430	343/430(79.77)	4.63e-252	DUF4150 domain-containing protein [Pantoea sp. ICBG 985]	2071683	d__Bacteria;k__norank;p__Proteobacteria;c__Gammaproteobacteria;o__Enterobacterales;f__Erwiniaceae;g__Pantoea;s__Pantoea_sp._ICBG_985
PB3_gene_3922	722	ORM79883.1	673	671/673(99.70)	0	excinuclease ABC subunit B [Pantoea deleyi]	470932	d__Bacteria;k__norank;p__Proteobacteria;c__Gammaproteobacteria;o__Enterobacterales;f__Erwiniaceae;g__Pantoea;s__Pantoea_deleyi
PB3_gene_3936	737	ORM81892.1	788	719/736(97.69)	0	xanthine dehydrogenase molybdopterin binding subunit [Pantoea deleyi]	470932	d__Bacteria;k__norank;p__Proteobacteria;c__Gammaproteobacteria;o__Enterobacterales;f__Erwiniaceae;g__Pantoea;s__Pantoea_deleyi
PB3_gene_3938	395	SLR23617.1	92	36/88(40.91)	6.61e-09	Uncharacterised protein [Klebsiella pneumoniae]	573	d__Bacteria;k__norank;p__Proteobacteria;c__Gammaproteobacteria;o__Enterobacterales;f__Enterobacteriaceae;g__Klebsiella;s__Klebsiella_pneumoniae
PB3_gene_3940	720	AGJ99411.1	1002	423/726(58.26)	8.03e-168	Ankyrin repeat domain protein [Wolbachia endosymbiont of Drosophila simulans wNo]	1236908	d__Bacteria;k__norank;p__Proteobacteria;c__Alphaproteobacteria;o__Rickettsiales;f__Anaplasmataceae;g__Wolbachia;s__Wolbachia_endosymbiont_of_Drosophila_simulans
PB3_gene_3950	434	ORM83142.1	434	432/434(99.54)	1.87e-299	trigger factor [Pantoea deleyi]	470932	d__Bacteria;k__norank;p__Proteobacteria;c__Gammaproteobacteria;o__Enterobacterales;f__Erwiniaceae;g__Pantoea;s__Pantoea_deleyi
PB3_gene_3952	64	PZL89586.1	61	30/44(68.18)	2.76e-10	hypothetical protein CKF42_01485, partial [Pantoea	2027923	d__Bacteria;k__norank;p__Proteobacteria;c__Gammaproteobacteria;o__Enterobacterales;f__Erwiniaceae;g__Pantoea;s__Pantoea_sp._ARC270
PB3_gene_3965	182	OJH31055.1	280	94/147(63.95)	2.43e-52	Ankyrin repeats (3 copies) [Wolbachia endosymbiont of Armadillidium vulgare]	77039	d__Bacteria;k__norank;p__Proteobacteria;c__Alphaproteobacteria;o__Rickettsiales;f__Anaplasmataceae;g__Wolbachia;s__Wolbachia_endosymbiont_of_Armadillidium_vulgare
PB3_gene_3970	255	ORM79492.1	255	254/255(99.61)	1.29e-177	phosphate ABC transporter ATP-binding protein [Pantoea deleyi]	470932	d__Bacteria;k__norank;p__Proteobacteria;c__Gammaproteobacteria;o__Enterobacterales;f__Erwiniaceae;g__Pantoea;s__Pantoea_deleyi
PB3_gene_3971	212	ORM79491.1	212	206/212(97.17)	8.16e-146	phosphoribosylglycinamide formyltransferase [Pantoea	470932	d__Bacteria;k__norank;p__Proteobacteria;c__Gammaproteobacteria;o__Enterobacterales;f__Erwiniaceae;g__Pantoea;s__Pantoea_deleyi
PB3_gene_3975	1148	WP_046103197.1	1146	1041/1148(90.68)	0	response regulator [Pantoea anthophila]	470931	d__Bacteria;k__norank;p__Proteobacteria;c__Gammaproteobacteria;o__Enterobacterales;f__Erwiniaceae;g__Pantoea;s__Pantoea_anthophila
PB3_gene_3981	135	SSL82769.1	111	53/107(49.53)	3.20e-19	Uncharacterised protein [Klebsiella pneumoniae]	573	d__Bacteria;k__norank;p__Proteobacteria;c__Gammaproteobacteria;o__Enterobacterales;f__Enterobacteriaceae;g__Klebsiella;s__Klebsiella_pneumoniae
PB3_gene_3989	132	WP_090962025.1	132	110/132(83.33)	1.60e-70	DUF1311 domain-containing protein [Pantoea sp. OV426]	1881036	d__Bacteria;k__norank;p__Proteobacteria;c__Gammaproteobacteria;o__Enterobacterales;f__Erwiniaceae;g__Pantoea;s__Pantoea_sp._OV426
PB3_gene_3990	102	RZK07954.1	133	84/102(82.35)	1.57e-51	DUF1311 domain-containing protein [Pantoea sp.]	69393	d__Bacteria;k__norank;p__Proteobacteria;c__Gammaproteobacteria;o__Enterobacterales;f__Erwiniaceae;g__Pantoea;s__Pantoea_sp.
PB3_gene_3998	216	ORM84684.1	231	202/216(93.52)	2.07e-149	extensin [Pantoea deleyi]	470932	d__Bacteria;k__norank;p__Proteobacteria;c__Gammaproteobacteria;o__Enterobacterales;f__Erwiniaceae;g__Pantoea;s__Pantoea_deleyi
PB3_gene_4001	211	WP_111138571.1	211	209/211(99.05)	3.59e-144	HTH-type transcriptional regulator RutR [Pantoea sp.	2027922	d__Bacteria;k__norank;p__Proteobacteria;c__Gammaproteobacteria;o__Enterobacterales;f__Erwiniaceae;g__Pantoea;s__Pantoea_sp._ARC607
PB3_gene_4015	272	ORM79063.1	270	213/248(85.89)	7.25e-137	hypothetical protein HA42_15940 [Pantoea deleyi]	470932	d__Bacteria;k__norank;p__Proteobacteria;c__Gammaproteobacteria;o__Enterobacterales;f__Erwiniaceae;g__Pantoea;s__Pantoea_deleyi
PB3_gene_4018	182	WP_111141384.1	363	176/182(96.70)	2.17e-119	mechanosensitive ion channel [Pantoea sp. ARC607]	2027922	d__Bacteria;k__norank;p__Proteobacteria;c__Gammaproteobacteria;o__Enterobacterales;f__Erwiniaceae;g__Pantoea;s__Pantoea_sp._ARC607
PB3_gene_4022	398	WP_017531748.1	396	378/398(94.97)	1.53e-261	MULTISPECIES: hypothetical protein [Wolbachia]	2640676	d__Bacteria;k__norank;p__Proteobacteria;c__Alphaproteobacteria;o__Rickettsiales;f__Anaplasmataceae;g__Wolbachia;s__norank
PB3_gene_4051	488	ORM83262.1	488	479/487(98.36)	0	2,4-diaminobutyrate decarboxylase [Pantoea deleyi]	470932	d__Bacteria;k__norank;p__Proteobacteria;c__Gammaproteobacteria;o__Enterobacterales;f__Erwiniaceae;g__Pantoea;s__Pantoea_deleyi

PB3_gene_4052	464	WP_111138467.1	460	458/460(99.57)	0	diaminobutyrate--2-oxoglutarate transaminase [Pantoea sp.	2027922	d__Bacteria;k__norank;p__Proteobacteria;c__Gammaproteobacteria;o__Enterobacterales;f__Erwiniaceae;g__Pantoea;s__Pantoea_sp._ARC607
PB3_gene_4053	145	ORM83263.1	191	138/145(95.17)	2.96e-96	TetR family transcriptional regulator [Pantoea deleyi]	470932	d__Bacteria;k__norank;p__Proteobacteria;c__Gammaproteobacteria;o__Enterobacterales;f__Erwiniaceae;g__Pantoea;s__Pantoea_deleyi
PB3_gene_4071	355	WP_087769494.1	359	343/355(96.62)	8.07e-255	type VI secretion system baseplate subunit TssG [Yersinia	91347	d__Bacteria;k__norank;p__Proteobacteria;c__Gammaproteobacteria;o__Enterobacterales;f__norank;g__norank;s__norank
PB3_gene_4075	34	WP_068651515.1	127	33/34(97.06)	2.18e-14	Fe-S cluster assembly scaffold IscU [Wolbachia endosymbiont of Trichogramma pretiosum]	125593	d__Bacteria;k__norank;p__Proteobacteria;c__Alphaproteobacteria;o__Rickettsiales;f__Anaplasmataceae;g__Wolbachia;s__Wolbachia_endosymbiont_of_Trichogramma_pretiosum
PB3_gene_4080	200	WP_111140965.1	192	191/192(99.48)	1.39e-127	hypothetical protein [Pantoea sp. ARC607]	2027922	d__Bacteria;k__norank;p__Proteobacteria;c__Gammaproteobacteria;o__Enterobacterales;f__Erwiniaceae;g__Pantoea;s__Pantoea_sp._ARC607
PB3_gene_4088	575	CAQ54735.1	1056	566/575(98.43)	0	isoleucyl-trna synthetase [Wolbachia endosymbiont of Culex quinquefasciatus Pel]	570417	d__Bacteria;k__norank;p__Proteobacteria;c__Alphaproteobacteria;o__Rickettsiales;f__Anaplasmataceae;g__Wolbachia;s__Wolbachia_endosymbiont_of_Culex_quinquefasciatus
PB3_gene_4089	339	ORM82478.1	1255	339/339(100.00)	3.97e-226	translocation and assembly module TamB [Pantoea deleyi]	470932	d__Bacteria;k__norank;p__Proteobacteria;c__Gammaproteobacteria;o__Enterobacterales;f__Erwiniaceae;g__Pantoea;s__Pantoea_deleyi
PB3_gene_4097	261	WP_111139781.1	261	260/261(99.62)	4.26e-170	zinc ABC transporter permease subunit ZnuB [Pantoea sp.	2027922	d__Bacteria;k__norank;p__Proteobacteria;c__Gammaproteobacteria;o__Enterobacterales;f__Erwiniaceae;g__Pantoea;s__Pantoea_sp._ARC607
PB3_gene_4099	315	ORM86817.1	315	313/315(99.37)	6.17e-206	zinc ABC transporter substrate-binding protein [Pantoea deleyi]	470932	d__Bacteria;k__norank;p__Proteobacteria;c__Gammaproteobacteria;o__Enterobacterales;f__Erwiniaceae;g__Pantoea;s__Pantoea_deleyi
PB3_gene_4102	124	ADO10578.1	474	103/108(95.37)	5.18e-63	Nitrate transporter [Pantoea vagans C9-1]	712898	d__Bacteria;k__norank;p__Proteobacteria;c__Gammaproteobacteria;o__Enterobacterales;f__Erwiniaceae;g__Pantoea;s__Pantoea_vagans
PB3_gene_4105	135	WP_124231572.1	136	88/135(65.19)	9.98e-62	hypothetical protein [Erwinia psidii]	69224	d__Bacteria;k__norank;p__Proteobacteria;c__Gammaproteobacteria;o__Enterobacterales;f__Erwiniaceae;g__Erwinia;s__Erwinia_psidii
PB3_gene_4106	173	SQI44580.1	173	147/173(84.97)	9.12e-108	Uncharacterised protein [Serratia plymuthica]	82996	d__Bacteria;k__norank;p__Proteobacteria;c__Gammaproteobacteria;o__Enterobacterales;f__Yersiniaceae;g__Serratia;s__Serratia_plymuthica
PB3_gene_4108	283	WP_128086922.1	623	278/283(98.23)	2.46e-187	type VI secretion system baseplate subunit TssF [Pantoea	470932	d__Bacteria;k__norank;p__Proteobacteria;c__Gammaproteobacteria;o__Enterobacterales;f__Erwiniaceae;g__Pantoea;s__Pantoea_deleyi
PB3_gene_4128	440	ORM82183.1	440	431/440(97.95)	4.82e-305	citrate-proton symporter [Pantoea deleyi]	470932	d__Bacteria;k__norank;p__Proteobacteria;c__Gammaproteobacteria;o__Enterobacterales;f__Erwiniaceae;g__Pantoea;s__Pantoea_deleyi
PB3_gene_4129	323	ORM82182.1	324	314/323(97.21)	9.95e-214	fimbrial protein [Pantoea deleyi]	470932	d__Bacteria;k__norank;p__Proteobacteria;c__Gammaproteobacteria;o__Enterobacterales;f__Erwiniaceae;g__Pantoea;s__Pantoea_deleyi
PB3_gene_4132	626	WP_111140434.1	626	600/626(95.85)	0	TonB-dependent vitamin B12 receptor BtuB [Pantoea sp.	2027922	d__Bacteria;k__norank;p__Proteobacteria;c__Gammaproteobacteria;o__Enterobacterales;f__Erwiniaceae;g__Pantoea;s__Pantoea_sp._ARC607
PB3_gene_4144	1115	ABX12969.1	9585	299/1077(27.76)	4.81e-63	Fibronectin type III domain protein [Nitrosopumilus	436308	d__Archaea;k__norank;p__Thaumarchaeota;c__norank;o__Nitrosopumilales;f__Nitrosopumilaceae;g__Nitrosopumilus;s__Nitrosopumilus_maritimus
PB3_gene_4147	314	WP_095101019.1	413	229/304(75.33)	3.34e-173	anaerobic sulfatase maturase [Enterobacter sp. 10-1]	543	d__Bacteria;k__norank;p__Proteobacteria;c__Gammaproteobacteria;o__Enterobacterales;f__Enterobacteriaceae;g__norank;s__norank
PB3_gene_4150	617	WP_108569679.1	2892	505/593(85.16)	4.87e-265	filamentous hemagglutinin N-terminal domain-containing protein [Pantoea sp. RIT 413]	2202162	d__Bacteria;k__norank;p__Proteobacteria;c__Gammaproteobacteria;o__Enterobacterales;f__Erwiniaceae;g__Pantoea;s__Pantoea_sp._RIT_413
PB3_gene_4151	162	WP_064690125.1	162	146/162(90.12)	1.55e-105	hypothetical protein [Pantoea agglomerans]	549	d__Bacteria;k__norank;p__Proteobacteria;c__Gammaproteobacteria;o__Enterobacterales;f__Erwiniaceae;g__Pantoea;s__Pantoea_agglomerans
PB3_gene_4155	875	ORM77523.1	875	872/875(99.66)	0	alanine--tRNA ligase [Pantoea deleyi]	470932	d__Bacteria;k__norank;p__Proteobacteria;c__Gammaproteobacteria;o__Enterobacterales;f__Erwiniaceae;g__Pantoea;s__Pantoea_deleyi
PB3_gene_4157	80	ORM86838.1	61	61/61(100.00)	2.98e-34	hypothetical protein HA42_00230 [Pantoea deleyi]	470932	d__Bacteria;k__norank;p__Proteobacteria;c__Gammaproteobacteria;o__Enterobacterales;f__Erwiniaceae;g__Pantoea;s__Pantoea_deleyi
PB3_gene_4159	340	WP_111138834.1	340	337/340(99.12)	6.53e-241	alcohol dehydrogenase AdhP [Pantoea sp. ARC607]	2027922	d__Bacteria;k__norank;p__Proteobacteria;c__Gammaproteobacteria;o__Enterobacterales;f__Erwiniaceae;g__Pantoea;s__Pantoea_sp._ARC607
PB3_gene_4161	93	PBQ26887.1	82	82/82(100.00)	1.19e-47	hypothetical protein BTO27_04090 [Wolbachia	2049495	d__Bacteria;k__norank;p__Proteobacteria;c__Alphaproteobacteria;o__Rickettsiales;f__Anaplasmataceae;g__Wolbachia;s__Wolbachia_pipientis

PB3_gene_4172	388	PCG11035.1	388	343/388(88.40)	1.21e-160	hypothetical protein CO539_05530 [Wolbachia	None	Unclassified
PB3_gene_4181	170	WP_111140454.1	262	161/170(94.71)	7.39e-65	cell division protein DedD [Pantoea sp. ARC607]	2027922	d__Bacteria;k__norank;p__Proteobacteria;c__Gammaproteobacteria;o__Enterobacterales;f__Erwiniaceae;g__Pantoea;s__Pantoea_sp._ARC607
PB3_gene_4193	522	WP_111139329.1	521	499/520(95.96)	0	GGDEF domain-containing protein [Pantoea sp. ARC607]	2027922	d__Bacteria;k__norank;p__Proteobacteria;c__Gammaproteobacteria;o__Enterobacterales;f__Erwiniaceae;g__Pantoea;s__Pantoea_sp._ARC607
PB3_gene_4197	599	WP_111139117.1	599	599/599(100.00)	0	elongation factor 4 [Pantoea sp. ARC607]	2027922	d__Bacteria;k__norank;p__Proteobacteria;c__Gammaproteobacteria;o__Enterobacterales;f__Erwiniaceae;g__Pantoea;s__Pantoea_sp._ARC607
PB3_gene_4199	103	WP_111208327.1	64	47/49(95.92)	7.12e-28	hypothetical protein [Pantoea sp. ARC270]	None	Unclassified
PB3_gene_4221	386	WP_111141484.1	420	363/386(94.04)	5.22e-258	four-carbon acid sugar kinase family protein [Pantoea sp.	2027922	d__Bacteria;k__norank;p__Proteobacteria;c__Gammaproteobacteria;o__Enterobacterales;f__Erwiniaceae;g__Pantoea;s__Pantoea_sp._ARC607
PB3_gene_4228	275	WP_111141498.1	275	270/275(98.18)	5.33e-196	SDR family oxidoreductase [Pantoea sp. ARC607]	2027922	d__Bacteria;k__norank;p__Proteobacteria;c__Gammaproteobacteria;o__Enterobacterales;f__Erwiniaceae;g__Pantoea;s__Pantoea_sp._ARC607
PB3_gene_4229	290	WP_111141496.1	425	285/290(98.28)	9.27e-207	FAD-binding oxidoreductase [Pantoea sp. ARC607]	2027922	d__Bacteria;k__norank;p__Proteobacteria;c__Gammaproteobacteria;o__Enterobacterales;f__Erwiniaceae;g__Pantoea;s__Pantoea_sp._ARC607
PB3_gene_4233	412	WP_111140637.1	412	412/412(100.00)	3.43e-293	phosphoglycerate dehydrogenase [Pantoea sp. ARC607]	53335	d__Bacteria;k__norank;p__Proteobacteria;c__Gammaproteobacteria;o__Enterobacterales;f__Erwiniaceae;g__Pantoea;s__norank
PB3_gene_4239	397	ORM83725.1	383	365/382(95.55)	1.01e-258	cellulose synthase [Pantoea deleyi]	470932	d__Bacteria;k__norank;p__Proteobacteria;c__Gammaproteobacteria;o__Enterobacterales;f__Erwiniaceae;g__Pantoea;s__Pantoea_deleyi
PB3_gene_4255	565	PBQ28276.1	565	549/565(97.17)	0	multidrug ABC transporter ATP-binding protein [Wolbachia pipientis wAus]	2049495	d__Bacteria;k__norank;p__Proteobacteria;c__Alphaproteobacteria;o__Rickettsiales;f__Anaplasmataceae;g__Wolbachia;s__Wolbachia_pipientis
PB3_gene_4260	529	ORM84148.1	657	506/529(95.65)	0	autotransporter outer membrane beta-barrel domain-containing protein [Pantoea deleyi]	470932	d__Bacteria;k__norank;p__Proteobacteria;c__Gammaproteobacteria;o__Enterobacterales;f__Erwiniaceae;g__Pantoea;s__Pantoea_deleyi
PB3_gene_4268	191	WP_111141390.1	191	186/191(97.38)	3.25e-128	phosphonate C-P lyase system protein PhnH [Pantoea sp.	2027922	d__Bacteria;k__norank;p__Proteobacteria;c__Gammaproteobacteria;o__Enterobacterales;f__Erwiniaceae;g__Pantoea;s__Pantoea_sp._ARC607
PB3_gene_4286	100	ORM86856.1	78	78/78(100.00)	4.05e-47	hypothetical protein HA42_00330 [Pantoea deleyi]	470932	d__Bacteria;k__norank;p__Proteobacteria;c__Gammaproteobacteria;o__Enterobacterales;f__Erwiniaceae;g__Pantoea;s__Pantoea_deleyi
PB3_gene_4289	490	AGJ98512.1	505	408/506(80.63)	2.84e-273	hypothetical protein wNo_00510 [Wolbachia endosymbiont of Drosophila simulans wNo]	1236908	d__Bacteria;k__norank;p__Proteobacteria;c__Alphaproteobacteria;o__Rickettsiales;f__Anaplasmataceae;g__Wolbachia;s__Wolbachia_endosymbiont_of_Drosophila_simulans
PB3_gene_4292	228	ORM82649.1	534	214/228(93.86)	5.89e-147	diguanylate phosphodiesterase [Pantoea deleyi]	470932	d__Bacteria;k__norank;p__Proteobacteria;c__Gammaproteobacteria;o__Enterobacterales;f__Erwiniaceae;g__Pantoea;s__Pantoea_deleyi
PB3_gene_4294	238	STS80503.1	167	86/155(55.48)	7.67e-51	Uncharacterised protein [Klebsiella pneumoniae]	573	d__Bacteria;k__norank;p__Proteobacteria;c__Gammaproteobacteria;o__Enterobacterales;f__Enterobacteriaceae;g__Klebsiella;s__Klebsiella_pneumoniae
PB3_gene_4315	412	WP_063630943.1	867	405/411(98.54)	3.97e-292	aconitate hydratase AcnA [Wolbachia endosymbiont of Laodelphax striatella]	368602	d__Bacteria;k__norank;p__Proteobacteria;c__Alphaproteobacteria;o__Rickettsiales;f__Anaplasmataceae;g__Wolbachia;s__Wolbachia_endosymbiont_of_Laodelphax_striatellus
PB3_gene_4317	163	ORM76135.1	352	162/163(99.39)	4.90e-108	molybdenum ABC transporter ATP-binding protein [Pantoea	470932	d__Bacteria;k__norank;p__Proteobacteria;c__Gammaproteobacteria;o__Enterobacterales;f__Erwiniaceae;g__Pantoea;s__Pantoea_deleyi
PB3_gene_4323	526	WP_114683286.1	549	492/526(93.54)	0	DNA phosphorothioation-dependent restriction protein DptF [Citrobacter freundii]	546	d__Bacteria;k__norank;p__Proteobacteria;c__Gammaproteobacteria;o__Enterobacterales;f__Enterobacteriaceae;g__Citrobacter;s__Citrobacter_freundii
PB3_gene_4324	440	WP_114263361.1	440	425/440(96.59)	3.70e-309	DNA phosphorothioation-dependent restriction protein DptG [Klebsiella pneumoniae]	573	d__Bacteria;k__norank;p__Proteobacteria;c__Gammaproteobacteria;o__Enterobacterales;f__Enterobacteriaceae;g__Klebsiella;s__Klebsiella_pneumoniae
PB3_gene_4327	96	PPY37699.1	38	30/35(85.71)	2.89e-12	lipoate--protein ligase, partial [Cronobacter sakazakii]	28141	d__Bacteria;k__norank;p__Proteobacteria;c__Gammaproteobacteria;o__Enterobacterales;f__Enterobacteriaceae;g__Cronobacter;s__Cronobacter_sakazakii

PB3_gene_4339	487	WP_117030799.1	487	464/487(95.28)	0	glycosyltransferase [Klebsiella pneumoniae]	573	d__Bacteria;k__norank;p__Proteobacteria;c__Gammaproteobacteria;o__Enterobacterales;f__Enterobacteriaceae;g__Klebsiella;s__Klebsiella_pneumoniae
PB3_gene_4340	421	WP_117030798.1	421	388/421(92.16)	7.91e-283	glycosyltransferase family 2 protein [Klebsiella pneumoniae]	573	d__Bacteria;k__norank;p__Proteobacteria;c__Gammaproteobacteria;o__Enterobacterales;f__Enterobacteriaceae;g__Klebsiella;s__Klebsiella_pneumoniae
PB3_gene_4343	488	PBQ29136.1	498	478/488(97.95)	0	leucyl aminopeptidase [Wolbachia pipientis wAus]	2049495	d__Bacteria;k__norank;p__Proteobacteria;c__Alphaproteobacteria;o__Rickettsiales;f__Anaplasmataceae;g__Wolbachia;s__Wolbachia_pipientis
PB3_gene_4346	87	ORM77083.1	87	84/87(96.55)	2.01e-52	Barstar, RNAse (barnase) inhibitor [Pantoea deleyi]	470932	d__Bacteria;k__norank;p__Proteobacteria;c__Gammaproteobacteria;o__Enterobacterales;f__Erwiniaceae;g__Pantoea;s__Pantoea_deleyi
PB3_gene_4347	144	ORM77082.1	144	143/144(99.31)	1.39e-106	hypothetical protein HA42_18290 [Pantoea deleyi]	470932	d__Bacteria;k__norank;p__Proteobacteria;c__Gammaproteobacteria;o__Enterobacterales;f__Erwiniaceae;g__Pantoea;s__Pantoea_deleyi
PB3_gene_4349	252	CDL21571.1	163	48/120(40.00)	6.35e-21	hypothetical protein [Klebsiella pneumoniae IS53]	1432554	d__Bacteria;k__norank;p__Proteobacteria;c__Gammaproteobacteria;o__Enterobacterales;f__Enterobacteriaceae;g__Klebsiella;s__Klebsiella_pneumoniae
PB3_gene_4354	262	ORM85078.1	262	255/262(97.33)	3.88e-187	mannosyl-3-phosphoglycerate phosphatase [Pantoea deleyi]	470932	d__Bacteria;k__norank;p__Proteobacteria;c__Gammaproteobacteria;o__Enterobacterales;f__Erwiniaceae;g__Pantoea;s__Pantoea_deleyi
PB3_gene_4363	131	WP_111139261.1	132	130/131(99.24)	2.11e-77	hypothetical protein [Pantoea sp. ARC607]	2027922	d__Bacteria;k__norank;p__Proteobacteria;c__Gammaproteobacteria;o__Enterobacterales;f__Erwiniaceae;g__Pantoea;s__Pantoea_sp._ARC607
PB3_gene_4365	124	ORM77142.1	124	121/124(97.58)	4.58e-73	hypothetical protein HA42_18210 [Pantoea deleyi]	470932	d__Bacteria;k__norank;p__Proteobacteria;c__Gammaproteobacteria;o__Enterobacterales;f__Erwiniaceae;g__Pantoea;s__Pantoea_deleyi
PB3_gene_4366	115	OQV40476.1	115	109/115(94.78)	3.92e-72	modulator protein [Pantoea vagans]	470934	d__Bacteria;k__norank;p__Proteobacteria;c__Gammaproteobacteria;o__Enterobacterales;f__Erwiniaceae;g__Pantoea;s__Pantoea_vagans
PB3_gene_4367	205	ORM77141.1	224	205/205(100.00)	1.44e-140	hypothetical protein HA42_18200 [Pantoea deleyi]	470932	d__Bacteria;k__norank;p__Proteobacteria;c__Gammaproteobacteria;o__Enterobacterales;f__Erwiniaceae;g__Pantoea;s__Pantoea_deleyi
PB3_gene_4379	372	WP_111142021.1	372	362/372(97.31)	1.13e-264	23S rRNA (guanine(1835)-N(2))-methyltransferase RlmG [Pantoea sp. ARC607]	2027922	d__Bacteria;k__norank;p__Proteobacteria;c__Gammaproteobacteria;o__Enterobacterales;f__Erwiniaceae;g__Pantoea;s__Pantoea_sp._ARC607
PB3_gene_4380	291	WP_111142022.1	672	271/291(93.13)	4.15e-182	NADPH-dependent 2,4-dienoyl-CoA reductase [Pantoea sp.	2027922	d__Bacteria;k__norank;p__Proteobacteria;c__Gammaproteobacteria;o__Enterobacterales;f__Erwiniaceae;g__Pantoea;s__Pantoea_sp._ARC607
PB3_gene_4404	139	WP_126534692.1	149	134/139(96.40)	4.85e-91	type VI secretion system baseplate subunit TssE [Enterobacter sp.	2497436	d__Bacteria;k__norank;p__Proteobacteria;c__Gammaproteobacteria;o__Enterobacterales;f__Enterobacteriaceae;g__Enterobacter;s__Enterobacter_sp._WCHEn090040
PB3_gene_4407	414	WP_090392781.1	414	399/414(96.38)	2.49e-248	GIY-YIG nuclease family protein [Enterobacter sp. NFIX58]	1566251	d__Bacteria;k__norank;p__Proteobacteria;c__Gammaproteobacteria;o__Enterobacterales;f__Enterobacteriaceae;g__Enterobacter;s__Enterobacter_sp._NFIX58
PB3_gene_4415	202	WP_010406904.1	202	201/202(99.50)	8.50e-134	MULTISPECIES: guanylate kinase [Wolbachia]	180837	d__Bacteria;k__norank;p__Proteobacteria;c__Alphaproteobacteria;o__Rickettsiales;f__Anaplasmataceae;g__Wolbachia;s__Wolbachia_endosymbiont_of_Nasonia_vitripennis
PB3_gene_4425	517	WP_108784109.1	488	366/455(80.44)	2.34e-249	hypothetical protein [Wolbachia endosymbiont of Bemisia tabaci]	215173	d__Bacteria;k__norank;p__Proteobacteria;c__Alphaproteobacteria;o__Rickettsiales;f__Anaplasmataceae;g__Wolbachia;s__Wolbachia_endosymbiont_of_Bemisia_tabaci
PB3_gene_4437	115	WP_123251482.1	341	59/114(51.75)	1.82e-27	hypothetical protein [Dickeya sp. FVG10-MFV-A16]	1577887	d__Bacteria;k__norank;p__Proteobacteria;c__Gammaproteobacteria;o__Enterobacterales;f__Pectobacteriaceae;g__Dickeya;s__Dickeya_undicola
PB3_gene_4438	249	WP_108315923.1	248	202/245(82.45)	9.11e-152	KilA-N domain-containing protein [Vibrio cholerae]	None	Unclassified
PB3_gene_4439	243	WP_112877836.1	246	210/243(86.42)	7.27e-156	MULTISPECIES: hypothetical protein [Photorhabdus]	2620880	d__Bacteria;k__norank;p__Proteobacteria;c__Gammaproteobacteria;o__Enterobacterales;f__Morganellaceae;g__Photorhabdus;s__norank
PB3_gene_4440	186	EYU15329.1	993	121/185(65.41)	1.57e-72	type I restriction-modification system methyltransferase subunit [Photorhabdus luminescens BA1]	1393736	d__Bacteria;k__norank;p__Proteobacteria;c__Gammaproteobacteria;o__Enterobacterales;f__Morganellaceae;g__Photorhabdus;s__Photorhabdus_luminescens
PB3_gene_4463	751	WP_019236878.1	1396	716/856(83.64)	0	collagen-like protein [Wolbachia pipientis]	955	d__Bacteria;k__norank;p__Proteobacteria;c__Alphaproteobacteria;o__Rickettsiales;f__Anaplasmataceae;g__Wolbachia;s__Wolbachia_pipientis
PB3_gene_4464	569	PCG17269.1	569	558/569(98.07)	0	membrane protein insertase YidC [Wolbachia pipientis]	None	Unclassified

PB3_gene_4477	647	WP_111139870.1	689	609/642(94.86)	0	bifunctional diguanylate cyclase/phosphodiesterase, partial [Pantoea sp. ARC607]	2027922	d__Bacteria;k__norank;p__Proteobacteria;c__Gammaproteobacteria;o__Enterobacterales;f__Erwiniaceae;g__Pantoea;s__Pantoea_sp._ARC607
PB3_gene_4484	382	ORM81905.1	415	377/382(98.69)	1.41e-262	hypothetical protein HA42_09480 [Pantoea deleyi]	470932	d__Bacteria;k__norank;p__Proteobacteria;c__Gammaproteobacteria;o__Enterobacterales;f__Erwiniaceae;g__Pantoea;s__Pantoea_deleyi
PB3_gene_4485	122	WP_084400206.1	632	34/77(44.16)	1.00e-09	acyl-ACP--UDP-N-acetylglucosamine O-acyltransferase [Terrimicrobium	None	Unclassified
PB3_gene_4489	813	WP_111139156.1	813	797/813(98.03)	0	glucose/quinate/shikimate family membrane-bound PQQ-dependent dehydrogenase [Pantoea sp. ARC607]	2027922	d__Bacteria;k__norank;p__Proteobacteria;c__Gammaproteobacteria;o__Enterobacterales;f__Erwiniaceae;g__Pantoea;s__Pantoea_sp._ARC607
PB3_gene_4506	89	OQV41205.1	114	54/62(87.10)	3.08e-30	toxin [Pantoea vagans]	470934	d__Bacteria;k__norank;p__Proteobacteria;c__Gammaproteobacteria;o__Enterobacterales;f__Erwiniaceae;g__Pantoea;s__Pantoea_vagans
PB3_gene_4509	283	ORM84154.1	283	262/283(92.58)	2.50e-189	MBL fold metallo-hydrolase [Pantoea deleyi]	470932	d__Bacteria;k__norank;p__Proteobacteria;c__Gammaproteobacteria;o__Enterobacterales;f__Erwiniaceae;g__Pantoea;s__Pantoea_deleyi
PB3_gene_4515	760	WP_126796759.1	1082	748/760(98.42)	0	DEAD/DEAH box helicase [Enterobacter asburiae]	61645	d__Bacteria;k__norank;p__Proteobacteria;c__Gammaproteobacteria;o__Enterobacterales;f__Enterobacteriaceae;g__Enterobacter;s__Enterobacter_asburiae
PB3_gene_4516	87	RCX02834.1	87	82/87(94.25)	2.75e-50	putative transposase [Kosakonia sp. AG348]	2183906	d__Bacteria;k__norank;p__Proteobacteria;c__Gammaproteobacteria;o__Enterobacterales;f__Enterobacteriaceae;g__Kosakonia;s__Kosakonia_sp._AG348
PB3_gene_4522	281	ORM75303.1	281	275/281(97.86)	1.73e-204	deoxyribonuclease IV [Pantoea deleyi]	470932	d__Bacteria;k__norank;p__Proteobacteria;c__Gammaproteobacteria;o__Enterobacterales;f__Erwiniaceae;g__Pantoea;s__Pantoea_deleyi
PB3_gene_4526	515	WP_032650196.1	515	512/515(99.42)	0	MULTISPECIES: type VI secretion system contractile sheath large subunit	2608935	d__Bacteria;k__norank;p__Proteobacteria;c__Gammaproteobacteria;o__Enterobacterales;f__Enterobacteriaceae;g__Enterobacter;s__norank
PB3_gene_4529	288	ORM77148.1	288	286/288(99.31)	3.20e-199	16S rRNA (cytidine(1402)-2'-O)-methyltransferase [Pantoea deleyi]	470932	d__Bacteria;k__norank;p__Proteobacteria;c__Gammaproteobacteria;o__Enterobacterales;f__Erwiniaceae;g__Pantoea;s__Pantoea_deleyi
PB3_gene_4530	274	GBE70466.1	413	98/254(38.58)	7.91e-29	hypothetical protein EKINANG_21160 [Enterobacter sp. KINAN-G]	1535203	d__Bacteria;k__norank;p__Proteobacteria;c__Gammaproteobacteria;o__Enterobacterales;f__Enterobacteriaceae;g__Enterobacter;s__Enterobacter_sp._KINAN-G
PB3_gene_4537	965	WP_115765125.1	1263	721/965(74.72)	0	autotransporter outer membrane beta-barrel domain-containing protein [Pantoea agglomerans]	549	d__Bacteria;k__norank;p__Proteobacteria;c__Gammaproteobacteria;o__Enterobacterales;f__Erwiniaceae;g__Pantoea;s__Pantoea_agglomerans
PB3_gene_4539	421	ORM86537.1	423	400/421(95.01)	2.50e-275	ligase [Pantoea deleyi]	470932	d__Bacteria;k__norank;p__Proteobacteria;c__Gammaproteobacteria;o__Enterobacterales;f__Erwiniaceae;g__Pantoea;s__Pantoea_deleyi
PB3_gene_4546	497	ORM78711.1	490	480/490(97.96)	0	molybdate ABC transporter ATP-binding protein ModF [Pantoea deleyi]	470932	d__Bacteria;k__norank;p__Proteobacteria;c__Gammaproteobacteria;o__Enterobacterales;f__Erwiniaceae;g__Pantoea;s__Pantoea_deleyi
PB3_gene_4555	75	EFM19271.1	70	23/31(74.19)	6.59e-06	hypothetical protein PanABDRAFT_2866 [Pantoea sp. aB]	517433	d__Bacteria;k__norank;p__Proteobacteria;c__Gammaproteobacteria;o__Enterobacterales;f__Erwiniaceae;g__Pantoea;s__Pantoea_sp._aB
PB3_gene_4561	405	PBQ29093.1	410	391/404(96.78)	3.40e-293	cystathionine beta-lyase [Wolbachia pipientis wAus]	2049495	d__Bacteria;k__norank;p__Proteobacteria;c__Alphaproteobacteria;o__Rickettsiales;f__Anaplasmataceae;g__Wolbachia;s__Wolbachia_pipientis
PB3_gene_4566	410	WP_111138605.1	410	404/409(98.78)	8.77e-295	beta-ketoacyl-ACP synthase [Pantoea sp. ARC607]	2027922	d__Bacteria;k__norank;p__Proteobacteria;c__Gammaproteobacteria;o__Enterobacterales;f__Erwiniaceae;g__Pantoea;s__Pantoea_sp._ARC607
PB3_gene_4573	194	PBQ30196.1	205	194/194(100.00)	7.98e-133	hypothetical protein BTO27_00370 [Wolbachia	2049495	d__Bacteria;k__norank;p__Proteobacteria;c__Alphaproteobacteria;o__Rickettsiales;f__Anaplasmataceae;g__Wolbachia;s__Wolbachia_pipientis
PB3_gene_4581	96	OQV38528.1	96	95/96(98.96)	2.39e-64	hypothetical protein BZ160_21080 [Pantoea vagans]	470934	d__Bacteria;k__norank;p__Proteobacteria;c__Gammaproteobacteria;o__Enterobacterales;f__Erwiniaceae;g__Pantoea;s__Pantoea_vagans

PB3_gene_4585	565	ORM82054.1	565	564/565(99.82)	0	NAD-dependent malic enzyme [Pantoea deleyi]	470932	d__Bacteria;k__norank;p__Proteobacteria;c__Gammaproteobacteria;o__Enterobacterales;f__Erwiniaceae;g__Pantoea;s__Pantoea_deleyi
PB3_gene_4586	152	ORM75588.1	204	143/152(94.08)	5.03e-111	phosphonate metabolism protein PhnP, partial [Pantoea deleyi]	470932	d__Bacteria;k__norank;p__Proteobacteria;c__Gammaproteobacteria;o__Enterobacterales;f__Erwiniaceae;g__Pantoea;s__Pantoea_deleyi
PB3_gene_4611	82	ORM75521.1	82	81/82(98.78)	1.03e-45	phage shock protein D [Pantoea deleyi]	470932	d__Bacteria;k__norank;p__Proteobacteria;c__Gammaproteobacteria;o__Enterobacterales;f__Erwiniaceae;g__Pantoea;s__Pantoea_deleyi
PB3_gene_4617	203	WP_111140233.1	203	198/203(97.54)	1.42e-137	DUF2291 domain-containing protein [Pantoea sp. ARC607]	2027922	d__Bacteria;k__norank;p__Proteobacteria;c__Gammaproteobacteria;o__Enterobacterales;f__Erwiniaceae;g__Pantoea;s__Pantoea_sp._ARC607
PB3_gene_4623	629	WP_071669448.1	629	583/629(92.69)	0	sigma-54-dependent Fis family transcriptional regulator [Pantoea sp. Ae16]	1890373	d__Bacteria;k__norank;p__Proteobacteria;c__Gammaproteobacteria;o__Enterobacterales;f__Erwiniaceae;g__Pantoea;s__Pantoea_sp._Ae16
PB3_gene_4639	54	ORM78936.1	97	54/54(100.00)	4.92e-28	hypothetical protein HA42_16085 [Pantoea deleyi]	470932	d__Bacteria;k__norank;p__Proteobacteria;c__Gammaproteobacteria;o__Enterobacterales;f__Erwiniaceae;g__Pantoea;s__Pantoea_deleyi
PB3_gene_4647	274	AFQ62607.1	292	272/274(99.27)	2.59e-184	GroEL, partial [Wolbachia endosymbiont of Bemisia tabaci]	215173	d__Bacteria;k__norank;p__Proteobacteria;c__Alphaproteobacteria;o__Rickettsiales;f__Anaplasmataceae;g__Wolbachia;s__Wolbachia_endosymbiont_of_Bemisia_tabaci
PB3_gene_4653	199	WP_085016769.1	199	198/199(99.50)	4.42e-139	MULTISPECIES: uracil-DNA glycosylase [Enterobacterales]	91347	d__Bacteria;k__norank;p__Proteobacteria;c__Gammaproteobacteria;o__Enterobacterales;f__norank;g__norank;s__norank
PB3_gene_4657	174	PBQ29182.1	158	155/158(98.10)	1.81e-104	30S ribosomal protein S7 [Wolbachia pipientis wAus]	2049495	d__Bacteria;k__norank;p__Proteobacteria;c__Alphaproteobacteria;o__Rickettsiales;f__Anaplasmataceae;g__Wolbachia;s__Wolbachia_pipientis
PB3_gene_4663	84	ORM86078.1	240	81/84(96.43)	9.71e-46	hypothetical protein HA42_01160 [Pantoea deleyi]	470932	d__Bacteria;k__norank;p__Proteobacteria;c__Gammaproteobacteria;o__Enterobacterales;f__Erwiniaceae;g__Pantoea;s__Pantoea_deleyi
PB3_gene_4677	191	PBQ27595.1	190	190/190(100.00)	1.74e-125	Holliday junction DNA helicase RuvA [Wolbachia pipientis	2049495	d__Bacteria;k__norank;p__Proteobacteria;c__Alphaproteobacteria;o__Rickettsiales;f__Anaplasmataceae;g__Wolbachia;s__Wolbachia_pipientis
PB3_gene_4698	246	WP_114517771.1	368	163/220(74.09)	7.31e-100	S49 family peptidase [Wolbachia endosymbiont of Cylisticus convexus]	118728	d__Bacteria;k__norank;p__Proteobacteria;c__Alphaproteobacteria;o__Rickettsiales;f__Anaplasmataceae;g__Wolbachia;s__Wolbachia_endosymbiont_of_Cylisticus_convexus
PB3_gene_4704	792	WP_112896062.1	993	688/792(86.87)	0	SAM-dependent methyltransferase [Photorhabdus	2029681	d__Bacteria;k__norank;p__Proteobacteria;c__Gammaproteobacteria;o__Enterobacterales;f__Morganellaceae;g__Photorhabdus;s__Photorhabdus_bodei
PB3_gene_4726	73	EFM18244.1	73	70/73(95.89)	2.95e-41	hypothetical protein PanABDRAFT_3791 [Pantoea sp. aB]	517433	d__Bacteria;k__norank;p__Proteobacteria;c__Gammaproteobacteria;o__Enterobacterales;f__Erwiniaceae;g__Pantoea;s__Pantoea_sp._aB
PB3_gene_4731	443	PCG11704.1	443	439/443(99.10)	0	insulinase family protein [Wolbachia pipientis]	None	Unclassified
PB3_gene_4735	59	WP_061901529.1	68	52/59(88.14)	1.97e-30	hypothetical protein [Pantoea agglomerans]	53335	d__Bacteria;k__norank;p__Proteobacteria;c__Gammaproteobacteria;o__Enterobacterales;f__Erwiniaceae;g__Pantoea;s__norank
PB3_gene_4745	259	WP_111141909.1	259	250/259(96.53)	2.83e-183	tRNA pseudouridine(65) synthase TruC [Pantoea sp. ARC607]	2027922	d__Bacteria;k__norank;p__Proteobacteria;c__Gammaproteobacteria;o__Enterobacterales;f__Erwiniaceae;g__Pantoea;s__Pantoea_sp._ARC607
PB3_gene_4764	470	BAH22257.1	467	428/467(91.65)	0	probable dioxygenase [Wolbachia endosymbiont of Cadra cautella]	190193	d__Bacteria;k__norank;p__Proteobacteria;c__Alphaproteobacteria;o__Rickettsiales;f__Anaplasmataceae;g__Wolbachia;s__Wolbachia_endosymbiont_of_Cadra_cautella
PB3_gene_4773	104	WP_111138468.1	103	96/103(93.20)	1.19e-63	DUF2132 domain-containing protein [Pantoea sp. ARC607]	2027922	d__Bacteria;k__norank;p__Proteobacteria;c__Gammaproteobacteria;o__Enterobacterales;f__Erwiniaceae;g__Pantoea;s__Pantoea_sp._ARC607
PB3_gene_4776	289	WP_038537028.1	303	219/284(77.11)	1.75e-140	XRE family transcriptional regulator [Wolbachia endosymbiont of Culex molestus]	329647	d__Bacteria;k__norank;p__Proteobacteria;c__Alphaproteobacteria;o__Rickettsiales;f__Anaplasmataceae;g__Wolbachia;s__Wolbachia_endosymbiont_of_Culex_molestus
PB3_gene_4780	80	SAW34212.1	80	34/80(42.50)	2.00e-10	Uncharacterised protein [Klebsiella pneumoniae]	573	d__Bacteria;k__norank;p__Proteobacteria;c__Gammaproteobacteria;o__Enterobacterales;f__Enterobacteriaceae;g__Klebsiella;s__Klebsiella_pneumoniae
PB3_gene_4781	305	WP_010404948.1	294	292/294(99.32)	3.42e-209	P44/Msp2 family outer membrane protein [Wolbachia endosymbiont of Nasonia vitripennis]	180837	d__Bacteria;k__norank;p__Proteobacteria;c__Alphaproteobacteria;o__Rickettsiales;f__Anaplasmataceae;g__Wolbachia;s__Wolbachia_endosymbiont_of_Nasonia_vitripennis

PB3_gene_4784	203	ORM75965.1	251	199/203(98.03)	1.62e-145	pyrroloquinoline quinone biosynthesis protein PqqC [Pantoea deleyi]	470932	d__Bacteria;k__norank;p__Proteobacteria;c__Gammaproteobacteria;o__Enterobacterales;f__Erwiniaceae;g__Pantoea;s__Pantoea_deleyi
PB3_gene_4793	446	WP_064085538.1	602	433/446(97.09)	2.25e-303	DNA mismatch repair endonuclease MutL [Wolbachia endosymbiont of Dactylopius	None	Unclassified
PB3_gene_4796	328	ORM79881.1	328	326/328(99.39)	4.96e-239	cyclic pyranopterin phosphate synthase [Pantoea deleyi]	470932	d__Bacteria;k__norank;p__Proteobacteria;c__Gammaproteobacteria;o__Enterobacterales;f__Erwiniaceae;g__Pantoea;s__Pantoea_deleyi
PB3_gene_4799	585	WP_111139222.1	643	545/577(94.45)	0	methyl-accepting chemotaxis protein [Pantoea sp. ARC607]	2027922	d__Bacteria;k__norank;p__Proteobacteria;c__Gammaproteobacteria;o__Enterobacterales;f__Erwiniaceae;g__Pantoea;s__Pantoea_sp._ARC607
PB3_gene_4818	58	WP_099258007.1	81	28/33(84.85)	8.23e-09	type IV secretion protein Rhs [Pantoea agglomerans]	549	d__Bacteria;k__norank;p__Proteobacteria;c__Gammaproteobacteria;o__Enterobacterales;f__Erwiniaceae;g__Pantoea;s__Pantoea_agglomerans
PB3_gene_4821	154	WP_098050858.1	154	151/154(98.05)	1.52e-99	hypothetical protein [Pantoea agglomerans]	None	Unclassified
PB3_gene_4823	516	ORM81421.1	520	506/520(97.31)	0	cellulose synthase [Pantoea deleyi]	470932	d__Bacteria;k__norank;p__Proteobacteria;c__Gammaproteobacteria;o__Enterobacterales;f__Erwiniaceae;g__Pantoea;s__Pantoea_deleyi
PB3_gene_4828	163	EIB96362.1	216	161/163(98.77)	7.41e-105	ABC transporter, permease protein [Pantoea sp. Sc1]	593105	d__Bacteria;k__norank;p__Proteobacteria;c__Gammaproteobacteria;o__Enterobacterales;f__Erwiniaceae;g__Pantoea;s__Pantoea_sp._Sc1
PB3_gene_4829	414	WP_104085964.1	405	381/405(94.07)	3.20e-274	monooxygenase [Pantoea sp. ICBG 1758]	2071682	d__Bacteria;k__norank;p__Proteobacteria;c__Gammaproteobacteria;o__Enterobacterales;f__Erwiniaceae;g__Pantoea;s__Pantoea_sp._ICBG_1758
PB3_gene_4830	231	WP_107320481.1	486	221/231(95.67)	5.65e-147	MFS transporter [Pantoea vagans]	470934	d__Bacteria;k__norank;p__Proteobacteria;c__Gammaproteobacteria;o__Enterobacterales;f__Erwiniaceae;g__Pantoea;s__Pantoea_vagans
PB3_gene_4833	99	WP_094111772.1	2334	80/88(90.91)	3.45e-42	filamentous hemagglutinin N-terminal domain-containing protein, partial [Pantoea eucrina]	472693	d__Bacteria;k__norank;p__Proteobacteria;c__Gammaproteobacteria;o__Enterobacterales;f__Erwiniaceae;g__Pantoea;s__Pantoea_eucrina
PB3_gene_4838	157	BAH22217.1	156	144/157(91.72)	5.00e-88	hypothetical protein [Wolbachia endosymbiont of Cadra cautella]	190193	d__Bacteria;k__norank;p__Proteobacteria;c__Alphaproteobacteria;o__Rickettsiales;f__Anaplasmataceae;g__Wolbachia;s__Wolbachia_endosymbiont_of_Cadra_cautella
PB3_gene_4849	128	PCG11595.1	123	121/123(98.37)	1.90e-75	hypothetical protein CO539_02275 [Wolbachia	None	Unclassified
PB3_gene_4867	105	SJZ46915.1	280	104/105(99.05)	6.67e-66	fructose-bisphosphate aldolase, class II [Pantoea agglomerans]	470933	d__Bacteria;k__norank;p__Proteobacteria;c__Gammaproteobacteria;o__Enterobacterales;f__Erwiniaceae;g__Pantoea;s__Pantoea_eucalypti
PB3_gene_4868	455	WP_111140344.1	455	454/455(99.78)	2.77e-315	PTS sugar transporter subunit IIC [Pantoea sp. ARC607]	2027922	d__Bacteria;k__norank;p__Proteobacteria;c__Gammaproteobacteria;o__Enterobacterales;f__Erwiniaceae;g__Pantoea;s__Pantoea_sp._ARC607
PB3_gene_4869	109	ORM76107.1	104	104/104(100.00)	1.84e-64	PTS lactose transporter subunit IIB [Pantoea deleyi]	470932	d__Bacteria;k__norank;p__Proteobacteria;c__Gammaproteobacteria;o__Enterobacterales;f__Erwiniaceae;g__Pantoea;s__Pantoea_deleyi
PB3_gene_4870	119	ORM76108.1	326	118/119(99.16)	1.03e-76	permease [Pantoea deleyi]	470932	d__Bacteria;k__norank;p__Proteobacteria;c__Gammaproteobacteria;o__Enterobacterales;f__Erwiniaceae;g__Pantoea;s__Pantoea_deleyi
PB3_gene_4871	392	ORM85266.1	392	388/392(98.98)	5.36e-264	sugar transporter [Pantoea deleyi]	470932	d__Bacteria;k__norank;p__Proteobacteria;c__Gammaproteobacteria;o__Enterobacterales;f__Erwiniaceae;g__Pantoea;s__Pantoea_deleyi
PB3_gene_4875	378	WP_111141529.1	378	374/378(98.94)	7.14e-271	alpha-D-ribose 1-methylphosphonate 5-triphosphate diphosphatase [Pantoea sp. ARC607]	2027922	d__Bacteria;k__norank;p__Proteobacteria;c__Gammaproteobacteria;o__Enterobacterales;f__Erwiniaceae;g__Pantoea;s__Pantoea_sp._ARC607
PB3_gene_4877	66	WP_111141556.1	256	63/66(95.45)	2.39e-41	phosphonate metabolism protein PhnP [Pantoea sp. ARC607]	2027922	d__Bacteria;k__norank;p__Proteobacteria;c__Gammaproteobacteria;o__Enterobacterales;f__Erwiniaceae;g__Pantoea;s__Pantoea_sp._ARC607
PB3_gene_4891	596	PXF62283.1	519	120/399(30.08)	1.16e-44	hypothetical protein DL796_12365, partial [Kangiella	796379	d__Bacteria;k__norank;p__Proteobacteria;c__Gammaproteobacteria;o__Oceanospirillales;f__Kangiellaceae;g__Kangiella;s__Kangiella_spongicola
PB3_gene_4895	451	WP_111138720.1	451	439/451(97.34)	2.45e-299	type I secretion system outer membrane protein [Pantoea sp. ARC607]	2027922	d__Bacteria;k__norank;p__Proteobacteria;c__Gammaproteobacteria;o__Enterobacterales;f__Erwiniaceae;g__Pantoea;s__Pantoea_sp._ARC607

PB3_gene_4900	302	WP_111139470.1	298	294/298(98.66)	1.67e-207	LysR family transcriptional regulator [Pantoea sp. ARC607]	2027922	d__Bacteria;k__norank;p__Proteobacteria;c__Gammaproteobacteria;o__Enterobacterales;f__Erwiniaceae;g__Pantoea;s__Pantoea_sp._ARC607
PB3_gene_4907	348	AGJ99822.1	344	296/340(87.06)	1.11e-203	Putative minor capsid protein C [Wolbachia endosymbiont of Drosophila simulans wHa]	1236909	d__Bacteria;k__norank;p__Proteobacteria;c__Alphaproteobacteria;o__Rickettsiales;f__Anaplasmataceae;g__Wolbachia;s__Wolbachia_endosymbiont_of_Drosophila_simulans
PB3_gene_4916	589	PCG11075.1	602	565/589(95.93)	0	aspartate--tRNA ligase [Wolbachia pipientis]	None	Unclassified
PB3_gene_4920	324	ORM77147.1	324	323/324(99.69)	7.70e-250	glutathione-dependent reductase [Pantoea deleyi]	470932	d__Bacteria;k__norank;p__Proteobacteria;c__Gammaproteobacteria;o__Enterobacterales;f__Erwiniaceae;g__Pantoea;s__Pantoea_deleyi
PB3_gene_4922	104	WP_036626855.1	108	84/102(82.35)	1.15e-53	metalloregulator ArsR/SmtB family transcription factor [Pantoea sp. AS-PWVM4]	1332069	d__Bacteria;k__norank;p__Proteobacteria;c__Gammaproteobacteria;o__Enterobacterales;f__Erwiniaceae;g__Pantoea;s__Pantoea_sp._AS-PWVM4
PB3_gene_4925	45	WP_115116444.1	1042	44/45(97.78)	6.41e-21	DEAD/DEAH box helicase [Serratia marcescens]	615	d__Bacteria;k__norank;p__Proteobacteria;c__Gammaproteobacteria;o__Enterobacterales;f__Yersiniaceae;g__Serratia;s__Serratia_marcescens
PB3_gene_4926	144	AGJ98554.1	144	137/144(95.14)	1.68e-89	hypothetical protein wNo_01070 [Wolbachia endosymbiont of Drosophila simulans wNo]	1236908	d__Bacteria;k__norank;p__Proteobacteria;c__Alphaproteobacteria;o__Rickettsiales;f__Anaplasmataceae;g__Wolbachia;s__Wolbachia_endosymbiont_of_Drosophila_simulans
PB3_gene_4933	223	SLU66725.1	223	221/223(99.10)	7.26e-159	Uncharacterised protein [Klebsiella pneumoniae]	573	d__Bacteria;k__norank;p__Proteobacteria;c__Gammaproteobacteria;o__Enterobacterales;f__Enterobacteriaceae;g__Klebsiella;s__Klebsiella_pneumoniae
PB3_gene_4934	370	SLU66714.1	370	368/370(99.46)	4.21e-259	Domain of uncharacterised function (DUF955) [Klebsiella	573	d__Bacteria;k__norank;p__Proteobacteria;c__Gammaproteobacteria;o__Enterobacterales;f__Enterobacteriaceae;g__Klebsiella;s__Klebsiella_pneumoniae
PB3_gene_4935	126	SUB14528.1	298	125/125(100.00)	1.96e-87	anaerobic glycerol-3-phosphate dehydrogenase subunit B [Pantoea agglomerans]	549	d__Bacteria;k__norank;p__Proteobacteria;c__Gammaproteobacteria;o__Enterobacterales;f__Erwiniaceae;g__Pantoea;s__Pantoea_agglomerans
PB3_gene_4936	509	ORM82640.1	509	508/509(99.80)	0	phosphate transporter PitA [Pantoea deleyi]	470932	d__Bacteria;k__norank;p__Proteobacteria;c__Gammaproteobacteria;o__Enterobacterales;f__Erwiniaceae;g__Pantoea;s__Pantoea_deleyi
PB3_gene_4938	612	WP_034915512.1	816	612/612(100.00)	0	MULTISPECIES: heavy metal translocating P-type ATPase [Erwiniaceae]	1903409	d__Bacteria;k__norank;p__Proteobacteria;c__Gammaproteobacteria;o__Enterobacterales;f__Erwiniaceae;g__norank;s__norank
PB3_gene_4945	48	WP_083495776.1	2840	48/48(100.00)	9.35e-23	DNA-directed RNA polymerase subunit beta/beta' [Wolbachia endosymbiont of Trichogramma pretiosum]	125593	d__Bacteria;k__norank;p__Proteobacteria;c__Alphaproteobacteria;o__Rickettsiales;f__Anaplasmataceae;g__Wolbachia;s__Wolbachia_endosymbiont_of_Trichogramma_pretiosum
PB3_gene_4974	640	SEO98880.1	794	560/651(86.02)	0	Predicted chitinase [Enterobacter sp. NFIX58]	1566251	d__Bacteria;k__norank;p__Proteobacteria;c__Gammaproteobacteria;o__Enterobacterales;f__Enterobacteriaceae;g__Enterobacter;s__Enterobacter_sp._NFIX58
PB3_gene_4976	76	OCA05909.1	198	71/76(93.42)	3.65e-42	orotidine 5'-phosphate decarboxylase [Wolbachia endosymbiont of Trichogramma pretiosum]	125593	d__Bacteria;k__norank;p__Proteobacteria;c__Alphaproteobacteria;o__Rickettsiales;f__Anaplasmataceae;g__Wolbachia;s__Wolbachia_endosymbiont_of_Trichogramma_pretiosum
PB3_gene_4983	61	WP_074019017.1	61	36/61(59.02)	2.78e-16	hypothetical protein [Xenorhabdus thuongxuanensis]	1873484	d__Bacteria;k__norank;p__Proteobacteria;c__Gammaproteobacteria;o__Enterobacterales;f__Morganellaceae;g__Xenorhabdus;s__Xenorhabdus_thuongxuanensis
PB3_gene_4986	444	POW54916.1	400	316/402(78.61)	1.33e-210	AAA family ATPase [Pantoea sp. PSNIH6]	2080494	d__Bacteria;k__norank;p__Proteobacteria;c__Gammaproteobacteria;o__Enterobacterales;f__Erwiniaceae;g__Pantoea;s__Pantoea_sp._PSNIH6
PB3_gene_4988	151	ORM81552.1	310	149/151(98.68)	4.72e-105	ribosomal protein L3 N(5)-glutamine methyltransferase	470932	d__Bacteria;k__norank;p__Proteobacteria;c__Gammaproteobacteria;o__Enterobacterales;f__Erwiniaceae;g__Pantoea;s__Pantoea_deleyi
PB3_gene_4994	322	PQL28093.1	337	307/322(95.34)	1.04e-227	HNH endonuclease [Pantoea ananatis]	553	d__Bacteria;k__norank;p__Proteobacteria;c__Gammaproteobacteria;o__Enterobacterales;f__Erwiniaceae;g__Pantoea;s__Pantoea_ananatis
PB3_gene_4995	108	ADO11865.1	39	25/38(65.79)	1.74e-06	hypothetical protein Pvag_3749 [Pantoea vagans C9-1]	712898	d__Bacteria;k__norank;p__Proteobacteria;c__Gammaproteobacteria;o__Enterobacterales;f__Erwiniaceae;g__Pantoea;s__Pantoea_vagans

PB3_gene_4996	153	RNA77043.1	153	139/153(90.85)	1.52e-94	hypothetical protein EBO33_10105 [[Curtobacterium]	221276	d__Bacteria;k__norank;p__Proteobacteria;c__Gammaproteobacteria;o__Enterobacterales;f__Erwiniaceae;g__Pantoea;s__[Curtobacterium]_plantarum
PB3_gene_5001	710	WP_034919207.1	707	706/707(99.86)	0	integrase [Erwinia sp. 9145]	1903409	d__Bacteria;k__norank;p__Proteobacteria;c__Gammaproteobacteria;o__Enterobacterales;f__Erwiniaceae;g__norank;s__norank
PB3_gene_5013	359	ORM85636.1	359	350/359(97.49)	2.87e-265	dTDP-glucose 4,6-dehydratase [Pantoea deleyi]	470932	d__Bacteria;k__norank;p__Proteobacteria;c__Gammaproteobacteria;o__Enterobacterales;f__Erwiniaceae;g__Pantoea;s__Pantoea_deleyi
PB3_gene_5014	142	SJZ62637.1	337	139/142(97.89)	8.16e-100	UDP-glucose 4-epimerase [Pantoea agglomerans]	470933	d__Bacteria;k__norank;p__Proteobacteria;c__Gammaproteobacteria;o__Enterobacterales;f__Erwiniaceae;g__Pantoea;s__Pantoea_eucalypti
PB3_gene_5022	592	ORM86809.1	592	586/592(98.99)	0	aspartate--tRNA ligase [Pantoea deleyi]	470932	d__Bacteria;k__norank;p__Proteobacteria;c__Gammaproteobacteria;o__Enterobacterales;f__Erwiniaceae;g__Pantoea;s__Pantoea_deleyi
PB3_gene_5032	715	ORM85046.1	1173	694/715(97.06)	0	O-antigen biosynthesis protein [Pantoea deleyi]	470932	d__Bacteria;k__norank;p__Proteobacteria;c__Gammaproteobacteria;o__Enterobacterales;f__Erwiniaceae;g__Pantoea;s__Pantoea_deleyi
PB3_gene_5042	524	VEB15166.1	881	345/526(65.59)	1.19e-251	Phage-related lysozyme (muraminidase) [Enterobacter cloacae]	158836	d__Bacteria;k__norank;p__Proteobacteria;c__Gammaproteobacteria;o__Enterobacterales;f__Enterobacteriaceae;g__Enterobacter;s__Enterobacter_hormaechei
PB3_gene_5043	118	SCM51674.1	118	107/118(90.68)	3.71e-67	hypothetical protein BN1044_01142 [Hafnia alvei]	569	d__Bacteria;k__norank;p__Proteobacteria;c__Gammaproteobacteria;o__Enterobacterales;f__Hafniaceae;g__Hafnia;s__Hafnia_alvei
PB3_gene_5096	267	WP_061061876.1	267	250/267(93.63)	1.24e-181	DUF3289 family protein [Pantoea vagans]	470934	d__Bacteria;k__norank;p__Proteobacteria;c__Gammaproteobacteria;o__Enterobacterales;f__Erwiniaceae;g__Pantoea;s__Pantoea_vagans
PB3_gene_5098	49	WP_112899915.1	206	32/38(84.21)	1.37e-14	zinc ribbon domain-containing protein [Pantoea ananatis]	553	d__Bacteria;k__norank;p__Proteobacteria;c__Gammaproteobacteria;o__Enterobacterales;f__Erwiniaceae;g__Pantoea;s__Pantoea_ananatis
PB3_gene_5109	181	KNH32064.1	231	163/179(91.06)	4.91e-109	hypothetical protein ACS76_10925 [Pantoea vagans]	470934	d__Bacteria;k__norank;p__Proteobacteria;c__Gammaproteobacteria;o__Enterobacterales;f__Erwiniaceae;g__Pantoea;s__Pantoea_vagans
PB3_gene_5111	261	KNE86510.1	135	52/125(41.60)	2.09e-24	hypothetical protein PSTG_20128, partial [Puccinia striiformis f. sp. tritici PST-78]	1165861	d__Eukaryota;k__Fungi;p__Basidiomycota;c__Pucciniomycetes;o__Pucciniales;f__Pucciniaceae;g__Puccinia;s__Puccinia_striiformis
PB3_gene_5133	311	PCG11264.1	326	308/311(99.04)	1.28e-211	phospho-N-acetylmuramoyl-pentapeptide-transferase [Wolbachia pipientis]	None	Unclassified
PB3_gene_5144	333	WP_085016771.1	332	332/332(100.00)	1.69e-142	MULTISPECIES: YncE family protein [Enterobacterales]	91347	d__Bacteria;k__norank;p__Proteobacteria;c__Gammaproteobacteria;o__Enterobacterales;f__norank;g__norank;s__norank
PB3_gene_5147	124	WP_057055954.1	124	124/124(100.00)	6.52e-82	MULTISPECIES: XRE family transcriptional regulator [Enterobacter cloacae complex]	91347	d__Bacteria;k__norank;p__Proteobacteria;c__Gammaproteobacteria;o__Enterobacterales;f__norank;g__norank;s__norank
PB3_gene_5148	67	WP_086533085.1	67	67/67(100.00)	5.36e-39	AlpA family transcriptional regulator [Enterobacter ludwigii]	299767	d__Bacteria;k__norank;p__Proteobacteria;c__Gammaproteobacteria;o__Enterobacterales;f__Enterobacteriaceae;g__Enterobacter;s__Enterobacter_ludwigii
PB3_gene_5149	97	KDF40056.1	251	63/97(64.95)	3.92e-38	hypothetical protein AE07_04193 [Enterobacter cloacae BWH 43]	1439328	d__Bacteria;k__norank;p__Proteobacteria;c__Gammaproteobacteria;o__Enterobacterales;f__Enterobacteriaceae;g__Enterobacter;s__Enterobacter_cloacae
PB3_gene_5154	310	CDR79069.1	312	274/311(88.10)	3.59e-180	transcriptional regulator,anaerobic benzoate catabolism transcriptional regulator,Predicted transcriptional regulator,transcriptional regulator, y4mF family,Helix-turn-helix [Wolbachia	225364	d__Bacteria;k__norank;p__Proteobacteria;c__Alphaproteobacteria;o__Rickettsiales;f__Anaplasmataceae;g__Wolbachia;s__Wolbachia_endosymbiont_of_Drosophila_simulans
PB3_gene_5167	660	PBQ26533.1	2386	572/664(86.14)	2.93e-78	hypothetical protein BTO27_04595, partial	2049495	d__Bacteria;k__norank;p__Proteobacteria;c__Alphaproteobacteria;o__Rickettsiales;f__Anaplasmataceae;g__Wolbachia;s__Wolbachia_pipientis
PB3_gene_5181	609	WP_070356862.1	775	582/594(97.98)	0	glycosyltransferase [Wolbachia endosymbiont of Drosophila incompta]	1633785	d__Bacteria;k__norank;p__Proteobacteria;c__Alphaproteobacteria;o__Rickettsiales;f__Anaplasmataceae;g__Wolbachia;s__Wolbachia_endosymbiont_of_Drosophila_incompta

PB3_gene_5193	111	ELP25063.1	186	97/111(87.39)	7.71e-68	hypothetical protein F385_1934 [Pantoea agglomerans 299R]	1261128	d__Bacteria;k__norank;p__Proteobacteria;c__Gammaproteobacteria;o__Enterobacterales;f__Erwiniaceae;g__Pantoea;s__Pantoea_agglomerans
PB3_gene_5201	265	PBQ25930.1	244	240/244(98.36)	5.21e-152	hypothetical protein BTO27_04970 [Wolbachia	2049495	d__Bacteria;k__norank;p__Proteobacteria;c__Alphaproteobacteria;o__Rickettsiales;f__Anaplasmataceae;g__Wolbachia;s__Wolbachia_pipientis
PB3_gene_5218	61	KIC84640.1	94	48/51(94.12)	6.50e-23	hypothetical protein RN49_23180, partial [Pantoea	549	d__Bacteria;k__norank;p__Proteobacteria;c__Gammaproteobacteria;o__Enterobacterales;f__Erwiniaceae;g__Pantoea;s__Pantoea_agglomerans
PB3_gene_5219	74	WP_010671630.1	74	65/73(89.04)	6.15e-39	type I toxin-antitoxin system SymE family toxin [Pantoea	549	d__Bacteria;k__norank;p__Proteobacteria;c__Gammaproteobacteria;o__Enterobacterales;f__Erwiniaceae;g__Pantoea;s__Pantoea_agglomerans
PB3_gene_5222	156	KYP86982.1	145	117/134(87.31)	1.61e-79	filamentous hemagglutinin [bacteria symbiont BFo2 of Frankliniella occidentalis]	1628856	d__Bacteria;k__norank;p__Proteobacteria;c__Gammaproteobacteria;o__Enterobacterales;f__norank;g__norank;s__bacteria_symbiont_BFo2_of_Frankliniella_occidentalis
PB3_gene_5224	216	PBQ29070.1	289	166/246(67.48)	8.54e-90	hypothetical protein BTO27_01590 [Wolbachia	2049495	d__Bacteria;k__norank;p__Proteobacteria;c__Alphaproteobacteria;o__Rickettsiales;f__Anaplasmataceae;g__Wolbachia;s__Wolbachia_pipientis
PB3_gene_5228	288	WP_010259049.1	288	286/288(99.31)	2.19e-206	bifunctional methylenetetrahydrofolate dehydrogenase/methenyltetrahydrofolate cyclohydrolase FolD [Pantoea agglomerans]	549	d__Bacteria;k__norank;p__Proteobacteria;c__Gammaproteobacteria;o__Enterobacterales;f__Erwiniaceae;g__Pantoea;s__Pantoea_agglomerans
PB3_gene_5245	229	ORM85046.1	1173	222/229(96.94)	3.93e-152	O-antigen biosynthesis protein [Pantoea deleyi]	470932	d__Bacteria;k__norank;p__Proteobacteria;c__Gammaproteobacteria;o__Enterobacterales;f__Erwiniaceae;g__Pantoea;s__Pantoea_deleyi
PB3_gene_5272	585	WP_010403291.1	581	573/581(98.62)	0	type I secretion system permease/ATPase [Wolbachia endosymbiont of Nasonia vitripennis]	180837	d__Bacteria;k__norank;p__Proteobacteria;c__Alphaproteobacteria;o__Rickettsiales;f__Anaplasmataceae;g__Wolbachia;s__Wolbachia_endosymbiont_of_Nasonia_vitripennis
PB3_gene_5296	110	RYA37779.1	351	57/115(49.57)	1.58e-29	hypothetical protein DD603_24190, partial [Enterobacter cloacae complex	2511984	d__Bacteria;k__norank;p__Proteobacteria;c__Gammaproteobacteria;o__Enterobacterales;f__Enterobacteriaceae;g__Enterobacter;s__Enterobacter_cloacae_complex_sp._2DZ2F2B
PB3_gene_5305	248	WP_006318270.1	250	185/249(74.30)	3.56e-140	hypothetical protein [Serratia plymuthica]	82996	d__Bacteria;k__norank;p__Proteobacteria;c__Gammaproteobacteria;o__Enterobacterales;f__Yersiniaceae;g__Serratia;s__Serratia_plymuthica
PB3_gene_5306	253	WP_126528128.1	254	192/251(76.49)	1.35e-144	N-acetylmuramoyl-L-alanine amidase [Serratia plymuthica]	82996	d__Bacteria;k__norank;p__Proteobacteria;c__Gammaproteobacteria;o__Enterobacterales;f__Yersiniaceae;g__Serratia;s__Serratia_plymuthica
PB3_gene_5307	205	WP_017531719.1	689	140/179(78.21)	1.80e-87	hypothetical protein [Wolbachia endosymbiont of Diaphorina citri]	116598	d__Bacteria;k__norank;p__Proteobacteria;c__Alphaproteobacteria;o__Rickettsiales;f__Anaplasmataceae;g__Wolbachia;s__Wolbachia_endosymbiont_of_Diaphorina_citri
PB3_gene_5316	360	WP_095706550.1	360	338/359(94.15)	3.76e-241	restriction endonuclease [Pantoea vagans]	470934	d__Bacteria;k__norank;p__Proteobacteria;c__Gammaproteobacteria;o__Enterobacterales;f__Erwiniaceae;g__Pantoea;s__Pantoea_vagans
PB3_gene_5325	133	RUM44802.1	320	52/95(54.74)	1.43e-25	hypothetical protein DSY80_04010, partial	2066021	d__Bacteria;k__norank;p__Proteobacteria;c__Deltaproteobacteria;o__Desulfobacterales;f__Desulfobulbaceae;g__Desulfocapsa;s__Desulfocapsa_sp.
PB3_gene_5327	514	WP_111141277.1	514	490/514(95.33)	2.60e-296	PAS domain-containing methyl-accepting chemotaxis protein [Pantoea sp. ARC607]	2027922	d__Bacteria;k__norank;p__Proteobacteria;c__Gammaproteobacteria;o__Enterobacterales;f__Erwiniaceae;g__Pantoea;s__Pantoea_sp._ARC607
PB3_gene_5334	453	WP_064704473.1	829	447/453(98.68)	0	type VI secretion system tip protein VgrG [Pantoea	549	d__Bacteria;k__norank;p__Proteobacteria;c__Gammaproteobacteria;o__Enterobacterales;f__Erwiniaceae;g__Pantoea;s__Pantoea_agglomerans
PB3_gene_5345	441	PCG11511.1	447	435/441(98.64)	1.13e-305	DNA repair protein RadA [Wolbachia pipientis]	None	Unclassified
PB3_gene_5362	260	AOR63641.1	284	204/217(94.01)	3.31e-147	transposase [Pectobacterium wasabiae CFBP 3304]	1175631	d__Bacteria;k__norank;p__Proteobacteria;c__Gammaproteobacteria;o__Enterobacterales;f__Pectobacteriaceae;g__Pectobacterium;s__Pectobacterium_wasabiae
PB3_gene_5373	123	SQK72293.1	128	102/123(82.93)	6.61e-78	Uncharacterised protein [Tatumella ptyseos]	82987	d__Bacteria;k__norank;p__Proteobacteria;c__Gammaproteobacteria;o__Enterobacterales;f__Erwiniaceae;g__Tatumella;s__Tatumella_ptyseos
PB3_gene_5385	244	WP_016929254.1	448	201/238(84.45)	6.42e-141	restriction endonuclease subunit S [Serratia marcescens]	615	d__Bacteria;k__norank;p__Proteobacteria;c__Gammaproteobacteria;o__Enterobacterales;f__Yersiniaceae;g__Serratia;s__Serratia_marcescens

PB3_gene_5386	338	RQN05071.1	786	318/338(94.08)	3.93e-220	SAM-dependent DNA methyltransferase [Pantoea	553	d__Bacteria;k__norank;p__Proteobacteria;c__Gammaproteobacteria;o__Enterobacterales;f__Erwiniaceae;g__Pantoea;s__Pantoea_ananatis
PB3_gene_5389	110	STN03652.1	110	91/110(82.73)	1.57e-62	Protein of uncharacterised function, DUF600 [Escherichia	562	d__Bacteria;k__norank;p__Proteobacteria;c__Gammaproteobacteria;o__Enterobacterales;f__Enterobacteriaceae;g__Escherichia;s__Escherichia_coli
PB3_gene_5391	258	WP_010407098.1	487	258/258(100.00)	2.06e-169	DegQ family serine endoprotease [Wolbachia endosymbiont of Nasonia vitripennis]	180837	d__Bacteria;k__norank;p__Proteobacteria;c__Alphaproteobacteria;o__Rickettsiales;f__Anaplasmataceae;g__Wolbachia;s__Wolbachia_endosymbiont_of_Nasonia_vitripennis
PB3_gene_5394	341	WP_111140340.1	757	296/341(86.80)	3.19e-203	pyrroloquinoline quinone biosynthesis protein PqqF [Pantoea sp. ARC607]	2027922	d__Bacteria;k__norank;p__Proteobacteria;c__Gammaproteobacteria;o__Enterobacterales;f__Erwiniaceae;g__Pantoea;s__Pantoea_sp._ARC607
PB3_gene_5396	77	WP_052259275.1	123	74/77(96.10)	2.51e-46	hypothetical protein [Pantoea agglomerans]	549	d__Bacteria;k__norank;p__Proteobacteria;c__Gammaproteobacteria;o__Enterobacterales;f__Erwiniaceae;g__Pantoea;s__Pantoea_agglomerans
PB3_gene_5397	297	WP_048780791.1	297	294/297(98.99)	2.54e-205	protein RepA [Pantoea vagans]	470934	d__Bacteria;k__norank;p__Proteobacteria;c__Gammaproteobacteria;o__Enterobacterales;f__Erwiniaceae;g__Pantoea;s__Pantoea_vagans
PB3_gene_5418	96	WP_039390306.1	132	96/96(100.00)	1.53e-63	type-F conjugative transfer system pilin assembly protein TrbC [Pantoea agglomerans]	549	d__Bacteria;k__norank;p__Proteobacteria;c__Gammaproteobacteria;o__Enterobacterales;f__Erwiniaceae;g__Pantoea;s__Pantoea_agglomerans
PB3_gene_5424	210	WP_111139973.1	210	198/209(94.74)	3.07e-140	helix-turn-helix domain-containing protein [Pantoea sp.	2027922	d__Bacteria;k__norank;p__Proteobacteria;c__Gammaproteobacteria;o__Enterobacterales;f__Erwiniaceae;g__Pantoea;s__Pantoea_sp._ARC607
PB3_gene_5438	375	WP_107320550.1	404	371/375(98.93)	2.00e-278	conjugal transfer protein [Pantoea vagans]	470934	d__Bacteria;k__norank;p__Proteobacteria;c__Gammaproteobacteria;o__Enterobacterales;f__Erwiniaceae;g__Pantoea;s__Pantoea_vagans
PB3_gene_5446	107	PZL85470.1	107	105/107(98.13)	1.42e-68	hypothetical protein CKF42_16690 [Pantoea sp.	2027923	d__Bacteria;k__norank;p__Proteobacteria;c__Gammaproteobacteria;o__Enterobacterales;f__Erwiniaceae;g__Pantoea;s__Pantoea_sp._ARC270
PB3_gene_5447	133	ORM85054.1	99	98/99(98.99)	1.18e-54	hypothetical protein HA42_03265 [Pantoea deleyi]	470932	d__Bacteria;k__norank;p__Proteobacteria;c__Gammaproteobacteria;o__Enterobacterales;f__Erwiniaceae;g__Pantoea;s__Pantoea_deleyi
PB3_gene_5459	67	WP_106481696.1	133	28/65(43.08)	1.66e-09	MULTISPECIES: fibronectin type III domain-containing protein [Butyricimonas]	574697	d__Bacteria;k__norank;p__Bacteroidetes;c__Bacteroidia;o__Bacteroidales;f__Odoribacteraceae;g__Butyricimonas;s__norank
PB3_gene_5468	361	CAX58085.1	361	326/361(90.30)	5.29e-237	conserved uncharacterized protein [Erwinia billingiae Eb661]	634500	d__Bacteria;k__norank;p__Proteobacteria;c__Gammaproteobacteria;o__Enterobacterales;f__Erwiniaceae;g__Erwinia;s__Erwinia_billingiae
PB3_gene_5475	303	WP_111534981.1	303	296/303(97.69)	4.47e-218	hypothetical protein [Pantoea agglomerans]	549	d__Bacteria;k__norank;p__Proteobacteria;c__Gammaproteobacteria;o__Enterobacterales;f__Erwiniaceae;g__Pantoea;s__Pantoea_agglomerans
PB3_gene_5478	396	EWG64829.1	427	373/396(94.19)	2.50e-274	VRR-NUC domain protein [Enterobacter sp. DC3]	1395579	d__Bacteria;k__norank;p__Proteobacteria;c__Gammaproteobacteria;o__Enterobacterales;f__Enterobacteriaceae;g__Enterobacter;s__Enterobacter_sp._DC3
PB3_gene_5483	108	ORM85663.1	253	108/108(100.00)	6.66e-66	DeoR family transcriptional regulator [Pantoea deleyi]	470932	d__Bacteria;k__norank;p__Proteobacteria;c__Gammaproteobacteria;o__Enterobacterales;f__Erwiniaceae;g__Pantoea;s__Pantoea_deleyi
PB3_gene_5485	301	ORM85664.1	301	295/301(98.01)	5.05e-198	oxidoreductase [Pantoea deleyi]	470932	d__Bacteria;k__norank;p__Proteobacteria;c__Gammaproteobacteria;o__Enterobacterales;f__Erwiniaceae;g__Pantoea;s__Pantoea_deleyi
PB3_gene_5489	155	WP_090133169.1	156	130/152(85.53)	2.32e-97	DUF943 family protein [Kosakonia oryziphila]	1005667	d__Bacteria;k__norank;p__Proteobacteria;c__Gammaproteobacteria;o__Enterobacterales;f__Enterobacteriaceae;g__Kosakonia;s__Kosakonia_oryziphila
PB3_gene_5491	214	OSY19256.1	647	33/74(44.59)	1.21e-13	hypothetical protein B0D75_01370 [Entomoplasmatales bacterium	None	Unclassified
PB3_gene_5497	72	SUB17264.1	78	60/62(96.77)	1.75e-36	Probable phosphatase YcdX [Pantoea agglomerans]	549	d__Bacteria;k__norank;p__Proteobacteria;c__Gammaproteobacteria;o__Enterobacterales;f__Erwiniaceae;g__Pantoea;s__Pantoea_agglomerans
PB3_gene_5499	83	EJK88826.1	154	45/79(56.96)	1.48e-19	hypothetical protein UUU_44960 [Klebsiella pneumoniae subsp. pneumoniae DSM 30104]	1162296	d__Bacteria;k__norank;p__Proteobacteria;c__Gammaproteobacteria;o__Enterobacterales;f__Enterobacteriaceae;g__Klebsiella;s__Klebsiella_pneumoniae
PB3_gene_5504	537	WP_105100119.1	689	508/532(95.49)	0	ParB/RepB/Spo0J family partition protein [Pantoea	None	Unclassified

PB3_gene_5505	285	WP_108784029.1	309	280/285(98.25)	3.26e-194	glutamine amidotransferase [Wolbachia endosymbiont of Bemisia tabaci]	215173	d__Bacteria;k__norank;p__Proteobacteria;c__Alphaproteobacteria;o__Rickettsiales;f__Anaplasmataceae;g__Wolbachia;s__Wolbachia_endosymbiont_of_Bemisia_tabaci
PB3_gene_5511	99	WP_081325919.1	314	94/99(94.95)	4.62e-64	hypothetical protein [Pantoea agglomerans]	None	Unclassified
PB3_gene_5525	51	CAY76402.1	59	24/30(80.00)	2.47e-07	hypothetical protein EPYR_04017 (plasmid) [Erwinia pyrifoliae DSM 12163]	644651	d__Bacteria;k__norank;p__Proteobacteria;c__Gammaproteobacteria;o__Enterobacterales;f__Erwiniaceae;g__Erwinia;s__Erwinia_pyrifoliae
PB3_gene_5532	307	WP_111139202.1	300	296/300(98.67)	9.75e-211	UTP--glucose-1-phosphate uridylyltransferase GalF [Pantoea sp. ARC607]	2027922	d__Bacteria;k__norank;p__Proteobacteria;c__Gammaproteobacteria;o__Enterobacterales;f__Erwiniaceae;g__Pantoea;s__Pantoea_sp._ARC607
PB3_gene_5538	484	WP_111138451.1	549	481/484(99.38)	0	malate dehydrogenase (quinone) [Pantoea sp. ARC607]	2027922	d__Bacteria;k__norank;p__Proteobacteria;c__Gammaproteobacteria;o__Enterobacterales;f__Erwiniaceae;g__Pantoea;s__Pantoea_sp._ARC607
PB3_gene_5546	290	CRM76140.1	274	95/272(34.93)	7.82e-26	hypothetical protein [Pseudomonas sp. 22 E 5]	1844093	d__Bacteria;k__norank;p__Proteobacteria;c__Gammaproteobacteria;o__Pseudomonadales;f__Pseudomonadaceae;g__Pseudomonas;s__Pseudomonas_sp._22_E_5
PB3_gene_5555	185	WP_111535230.1	187	183/185(98.92)	6.27e-119	DNA repair protein RadC [Pantoea agglomerans]	53335	d__Bacteria;k__norank;p__Proteobacteria;c__Gammaproteobacteria;o__Enterobacterales;f__Erwiniaceae;g__Pantoea;s__norank
PB3_gene_5563	52	WP_111138412.1	84	41/56(73.21)	1.40e-15	hypothetical protein [Pantoea sp. ARC607]	None	Unclassified
PB3_gene_5564	189	WP_069730094.1	329	188/189(99.47)	8.41e-130	site-specific integrase [Pantoea brenneri]	53335	d__Bacteria;k__norank;p__Proteobacteria;c__Gammaproteobacteria;o__Enterobacterales;f__Erwiniaceae;g__Pantoea;s__norank
PB3_gene_5565	76	KEY39986.1	76	74/76(97.37)	9.95e-48	protein umuc (plasmid) [Pantoea agglomerans]	549	d__Bacteria;k__norank;p__Proteobacteria;c__Gammaproteobacteria;o__Enterobacterales;f__Erwiniaceae;g__Pantoea;s__Pantoea_agglomerans
PB3_gene_5566	120	WP_089562423.1	125	119/120(99.17)	2.52e-76	peptidase [Pantoea sp. AV62]	53335	d__Bacteria;k__norank;p__Proteobacteria;c__Gammaproteobacteria;o__Enterobacterales;f__Erwiniaceae;g__Pantoea;s__norank
PB3_gene_5567	379	WP_111139961.1	401	374/379(98.68)	4.71e-213	FliC/FljB family flagellin [Pantoea sp. ARC607]	2027922	d__Bacteria;k__norank;p__Proteobacteria;c__Gammaproteobacteria;o__Enterobacterales;f__Erwiniaceae;g__Pantoea;s__Pantoea_sp._ARC607
PB3_gene_5569	525	WP_064085936.1	978	516/525(98.29)	0	hypothetical protein [Wolbachia endosymbiont of Dactylopius coccus]	None	Unclassified
PB3_gene_5584	38	SUE06546.1	57	36/38(94.74)	4.66e-17	Uncharacterised protein [Pantoea agglomerans]	549	d__Bacteria;k__norank;p__Proteobacteria;c__Gammaproteobacteria;o__Enterobacterales;f__Erwiniaceae;g__Pantoea;s__Pantoea_agglomerans
PB3_gene_5586	77	AAN79068.1	125	46/71(64.79)	3.18e-19	Conserved hypothetical protein [Escherichia coli CFT073]	199310	d__Bacteria;k__norank;p__Proteobacteria;c__Gammaproteobacteria;o__Enterobacterales;f__Enterobacteriaceae;g__Escherichia;s__Escherichia_coli
PB3_gene_5588	77	RDD35218.1	77	61/77(79.22)	9.23e-40	hypothetical protein Wcon_00644 [Wolbachia endosymbiont of Cylisticus convexus]	118728	d__Bacteria;k__norank;p__Proteobacteria;c__Alphaproteobacteria;o__Rickettsiales;f__Anaplasmataceae;g__Wolbachia;s__Wolbachia_endosymbiont_of_Cylisticus_convexus
PB3_gene_5591	79	KNH33692.1	94	41/78(52.56)	2.37e-11	hypothetical protein ACS76_06705 [Pantoea vagans]	470934	d__Bacteria;k__norank;p__Proteobacteria;c__Gammaproteobacteria;o__Enterobacterales;f__Erwiniaceae;g__Pantoea;s__Pantoea_vagans
PB3_gene_5599	424	WP_052264683.1	610	293/365(80.27)	1.15e-153	ankyrin repeat domain-containing protein [Wolbachia endosymbiont of Operophtera brumata]	None	Unclassified
PB3_gene_5601	130	WP_065647529.1	130	128/130(98.46)	6.64e-87	transcriptional regulator [Pantoea eucrina]	472693	d__Bacteria;k__norank;p__Proteobacteria;c__Gammaproteobacteria;o__Enterobacterales;f__Erwiniaceae;g__Pantoea;s__Pantoea_eucrina
PB3_gene_5602	99	WP_065647530.1	99	94/99(94.95)	1.37e-60	type II toxin-antitoxin system HigB family toxin [Pantoea	472693	d__Bacteria;k__norank;p__Proteobacteria;c__Gammaproteobacteria;o__Enterobacterales;f__Erwiniaceae;g__Pantoea;s__Pantoea_eucrina
PB3_gene_5603	456	WP_069729355.1	454	443/454(97.58)	0	hypothetical protein [Pantoea brenneri]	53335	d__Bacteria;k__norank;p__Proteobacteria;c__Gammaproteobacteria;o__Enterobacterales;f__Erwiniaceae;g__Pantoea;s__norank
PB3_gene_5610	243	WP_111139862.1	243	234/243(96.30)	2.18e-154	ABC transporter permease [Pantoea sp. ARC607]	2027922	d__Bacteria;k__norank;p__Proteobacteria;c__Gammaproteobacteria;o__Enterobacterales;f__Erwiniaceae;g__Pantoea;s__Pantoea_sp._ARC607

PB3_gene_5611	185	WP_111139861.1	315	175/185(94.59)	5.36e-117	ABC transporter ATP-binding protein [Pantoea sp. ARC607]	2027922	d__Bacteria;k__norank;p__Proteobacteria;c__Gammaproteobacteria;o__Enterobacterales;f__Erwiniaceae;g__Pantoea;s__Pantoea_sp._ARC607
PB3_gene_5616	166	RLJ22797.1	772	76/206(36.89)	3.75e-43	hypothetical protein DJ031_00255, partial [bacterium endosymbiont of Escarpia	1221601	d__Bacteria;k__norank;p__norank;c__norank;o__norank;f__norank;g__norank;s__bacterium_endosymbiont_of_Escarpia_laminata
PB3_gene_5617	390	WP_061901155.1	367	342/367(93.19)	2.26e-247	phage late control D family protein [Pantoea agglomerans]	549	d__Bacteria;k__norank;p__Proteobacteria;c__Gammaproteobacteria;o__Enterobacterales;f__Erwiniaceae;g__Pantoea;s__Pantoea_agglomerans
PB3_gene_5631	387	PCG11209.1	883	386/387(99.74)	9.49e-248	preprotein translocase subunit SecA [Wolbachia pipientis]	None	Unclassified
PB3_gene_5647	102	WP_111139768.1	238	98/102(96.08)	1.17e-61	5-amino-6-(5-phospho-D-ribitylamino)uracil phosphatase YigB [Pantoea sp. ARC607]	2027922	d__Bacteria;k__norank;p__Proteobacteria;c__Gammaproteobacteria;o__Enterobacterales;f__Erwiniaceae;g__Pantoea;s__Pantoea_sp._ARC607
PB3_gene_5652	108	SSM36898.1	94	30/63(47.62)	4.23e-06	Uncharacterised protein [Klebsiella pneumoniae]	573	d__Bacteria;k__norank;p__Proteobacteria;c__Gammaproteobacteria;o__Enterobacterales;f__Enterobacteriaceae;g__Klebsiella;s__Klebsiella_pneumoniae
PB3_gene_5669	352	WP_125310586.1	354	256/346(73.99)	1.27e-194	type VI secretion protein ImpA [Pantoea dispersa]	59814	d__Bacteria;k__norank;p__Proteobacteria;c__Gammaproteobacteria;o__Enterobacterales;f__Erwiniaceae;g__Pantoea;s__Pantoea_dispersa
PB3_gene_5695	83	ORM81405.1	367	78/83(93.98)	3.53e-43	ABC transporter [Pantoea deleyi]	470932	d__Bacteria;k__norank;p__Proteobacteria;c__Gammaproteobacteria;o__Enterobacterales;f__Erwiniaceae;g__Pantoea;s__Pantoea_deleyi
PB3_gene_5696	268	WP_071669459.1	268	261/268(97.39)	1.20e-184	methionine ABC transporter substrate-binding protein [Pantoea sp. Ae16]	1890373	d__Bacteria;k__norank;p__Proteobacteria;c__Gammaproteobacteria;o__Enterobacterales;f__Erwiniaceae;g__Pantoea;s__Pantoea_sp._Ae16
PB3_gene_5697	121	WP_071669460.1	452	117/120(97.50)	1.95e-78	LLM class flavin-dependent oxidoreductase [Pantoea sp.	1890373	d__Bacteria;k__norank;p__Proteobacteria;c__Gammaproteobacteria;o__Enterobacterales;f__Erwiniaceae;g__Pantoea;s__Pantoea_sp._Ae16
PB3_gene_5712	147	WP_029237639.1	132	128/132(96.97)	7.34e-89	DUF1284 domain-containing protein [Wolbachia endosymbiont of Nasonia vitripennis]	2640676	d__Bacteria;k__norank;p__Proteobacteria;c__Alphaproteobacteria;o__Rickettsiales;f__Anaplasmataceae;g__Wolbachia;s__norank
PB3_gene_5720	134	WP_111140847.1	227	121/134(90.30)	1.24e-72	SDR family oxidoreductase [Pantoea sp. ARC607]	2027922	d__Bacteria;k__norank;p__Proteobacteria;c__Gammaproteobacteria;o__Enterobacterales;f__Erwiniaceae;g__Pantoea;s__Pantoea_sp._ARC607
PB3_gene_5727	410	WP_064085594.1	410	407/410(99.27)	5.43e-289	hypothetical protein [Wolbachia endosymbiont of Dactylopius coccus]	None	Unclassified
PB3_gene_5738	292	SUB18080.1	415	292/292(100.00)	2.03e-192	Lon protease [Pantoea agglomerans]	549	d__Bacteria;k__norank;p__Proteobacteria;c__Gammaproteobacteria;o__Enterobacterales;f__Erwiniaceae;g__Pantoea;s__Pantoea_agglomerans
PB3_gene_5745	48	WP_058970014.1	141	44/48(91.67)	3.33e-23	type IV pilus biogenesis protein PilP [Type-D symbiont of Plautia	1560356	d__Bacteria;k__norank;p__Proteobacteria;c__Gammaproteobacteria;o__Enterobacterales;f__Enterobacteriaceae;g__norank;s__Type-D_symbiont_of_Plautia_stali
PB3_gene_5746	428	WP_058970013.1	451	374/428(87.38)	5.20e-272	conjugal transfer protein [Type-D symbiont of Plautia stali]	1560356	d__Bacteria;k__norank;p__Proteobacteria;c__Gammaproteobacteria;o__Enterobacterales;f__Enterobacteriaceae;g__norank;s__Type-D_symbiont_of_Plautia_stali
PB3_gene_5751	78	KGB03094.1	284	54/72(75.00)	5.57e-32	bacteriophage CI repressor helix-turn-helix domain protein [Enterobacteriaceae bacterium ATCC 29904]	1593232	d__Bacteria;k__norank;p__Proteobacteria;c__Gammaproteobacteria;o__Enterobacterales;f__Enterobacteriaceae;g__norank;s__Enterobacteriaceae_bacterium_ATCC_29904
PB3_gene_5752	75	WP_036626516.1	75	75/75(100.00)	2.19e-46	DNA-binding protein [Pantoea sp. AS-PWVM4]	None	Unclassified
PB3_gene_5753	169	WP_021184461.1	169	166/169(98.22)	3.90e-107	phage regulatory CII family protein [Pantoea sp. AS-	1332069	d__Bacteria;k__norank;p__Proteobacteria;c__Gammaproteobacteria;o__Enterobacterales;f__Erwiniaceae;g__Pantoea;s__Pantoea_sp._AS-PWVM4
PB3_gene_5754	63	WP_071822197.1	62	51/62(82.26)	6.69e-29	DUF2724 domain-containing protein [Pantoea vagans]	470934	d__Bacteria;k__norank;p__Proteobacteria;c__Gammaproteobacteria;o__Enterobacterales;f__Erwiniaceae;g__Pantoea;s__Pantoea_vagans
PB3_gene_5759	425	CQD08371.1	416	400/416(96.15)	1.02e-271	putative outer membrane protein [Wolbachia endosymbiont wPip_Mol of Culex molestus]	1402163	d__Bacteria;k__norank;p__Proteobacteria;c__Alphaproteobacteria;o__Rickettsiales;f__Anaplasmataceae;g__Wolbachia;s__Wolbachia_endosymbiont_of_Culex_molestus

PB3_gene_5761	149	ORM79065.1	363	134/149(89.93)	3.79e-81	mechanosensitive ion channel protein MscS [Pantoea deleyi]	470932	d__Bacteria;k__norank;p__Proteobacteria;c__Gammaproteobacteria;o__Enterobacterales;f__Erwiniaceae;g__Pantoea;s__Pantoea_deleyi
PB3_gene_5776	376	WP_111141278.1	484	361/376(96.01)	2.06e-254	PTS N-acetylmuramic acid transporter subunit IIBC [Pantoea sp. ARC607]	2027922	d__Bacteria;k__norank;p__Proteobacteria;c__Gammaproteobacteria;o__Enterobacterales;f__Erwiniaceae;g__Pantoea;s__Pantoea_sp._ARC607
PB3_gene_5789	205	WP_107320572.1	274	205/205(100.00)	3.42e-146	conjugal transfer protein [Pantoea vagans]	470934	d__Bacteria;k__norank;p__Proteobacteria;c__Gammaproteobacteria;o__Enterobacterales;f__Erwiniaceae;g__Pantoea;s__Pantoea_vagans
PB3_gene_5798	459	WP_039390282.1	1711	454/459(98.91)	2.97e-300	conjugative transfer relaxase/helicase TraI [Pantoea agglomerans]	549	d__Bacteria;k__norank;p__Proteobacteria;c__Gammaproteobacteria;o__Enterobacterales;f__Erwiniaceae;g__Pantoea;s__Pantoea_agglomerans
PB3_gene_5812	426	WP_107320589.1	652	417/426(97.89)	4.63e-298	mobilization protein [Pantoea vagans]	470934	d__Bacteria;k__norank;p__Proteobacteria;c__Gammaproteobacteria;o__Enterobacterales;f__Erwiniaceae;g__Pantoea;s__Pantoea_vagans
PB3_gene_5813	99	WP_033761748.1	201	74/97(76.29)	1.45e-46	DUF2313 domain-containing protein [Pantoea agglomerans]	None	Unclassified
PB3_gene_5814	354	WP_050556821.1	478	295/348(84.77)	7.70e-210	baseplate J/gp47 family protein [Pantoea agglomerans]	None	Unclassified
PB3_gene_5815	361	ORM85037.1	361	355/361(98.34)	2.76e-264	acyltransferase [Pantoea deleyi]	470932	d__Bacteria;k__norank;p__Proteobacteria;c__Gammaproteobacteria;o__Enterobacterales;f__Erwiniaceae;g__Pantoea;s__Pantoea_deleyi
PB3_gene_5828	145	WP_039390269.1	614	145/145(100.00)	3.07e-95	conjugal transfer protein TraN [Pantoea agglomerans]	549	d__Bacteria;k__norank;p__Proteobacteria;c__Gammaproteobacteria;o__Enterobacterales;f__Erwiniaceae;g__Pantoea;s__Pantoea_agglomerans
PB3_gene_5829	257	WP_122983638.1	257	252/256(98.44)	2.53e-185	type-F conjugative transfer system pilin assembly protein TraF [Pantoea brenneri]	53335	d__Bacteria;k__norank;p__Proteobacteria;c__Gammaproteobacteria;o__Enterobacterales;f__Erwiniaceae;g__Pantoea;s__norank
PB3_gene_5831	354	WP_017531719.1	689	189/355(53.24)	3.55e-99	hypothetical protein [Wolbachia endosymbiont of Diaphorina citri]	116598	d__Bacteria;k__norank;p__Proteobacteria;c__Alphaproteobacteria;o__Rickettsiales;f__Anaplasmataceae;g__Wolbachia;s__Wolbachia_endosymbiont_of_Diaphorina_citri
PB3_gene_5834	233	POW26960.1	253	177/233(75.97)	1.75e-123	ankyrin [Pectobacterium parmentieri]	1905730	d__Bacteria;k__norank;p__Proteobacteria;c__Gammaproteobacteria;o__Enterobacterales;f__Pectobacteriaceae;g__Pectobacterium;s__Pectobacterium_parmentieri
PB3_gene_5835	214	SUJ36907.1	860	209/214(97.66)	1.71e-142	ATP-dependent helicase HepA [Serratia marcescens]	615	d__Bacteria;k__norank;p__Proteobacteria;c__Gammaproteobacteria;o__Enterobacterales;f__Yersiniaceae;g__Serratia;s__Serratia_marcescens
PB3_gene_5848	407	AGJ98971.1	220	58/91(63.74)	2.87e-24	hypothetical protein wNo_05520 [Wolbachia endosymbiont of Drosophila simulans wNo]	1236908	d__Bacteria;k__norank;p__Proteobacteria;c__Alphaproteobacteria;o__Rickettsiales;f__Anaplasmataceae;g__Wolbachia;s__Wolbachia_endosymbiont_of_Drosophila_simulans
PB3_gene_5855	415	WP_010403853.1	412	409/412(99.27)	1.06e-290	MULTISPECIES: cell division protein FtsA [Wolbachia]	2640676	d__Bacteria;k__norank;p__Proteobacteria;c__Alphaproteobacteria;o__Rickettsiales;f__Anaplasmataceae;g__Wolbachia;s__norank
PB3_gene_5863	82	WP_103792530.1	167	56/75(74.67)	9.14e-31	MULTISPECIES: hypothetical protein [Leclercia]	None	Unclassified
PB3_gene_5867	116	AYP23583.1	72	61/72(84.72)	2.26e-31	hypothetical protein D0A61_11805 [Pantoea	549	d__Bacteria;k__norank;p__Proteobacteria;c__Gammaproteobacteria;o__Enterobacterales;f__Erwiniaceae;g__Pantoea;s__Pantoea_agglomerans
PB3_gene_5894	215	WP_010402332.1	215	207/214(96.73)	1.46e-145	MULTISPECIES: endonuclease III [Wolbachia]	2640676	d__Bacteria;k__norank;p__Proteobacteria;c__Alphaproteobacteria;o__Rickettsiales;f__Anaplasmataceae;g__Wolbachia;s__norank
PB3_gene_5900	210	WP_111138318.1	338	206/210(98.10)	1.10e-142	NAD(P)-dependent alcohol dehydrogenase [Pantoea sp. ARC607]	2027922	d__Bacteria;k__norank;p__Proteobacteria;c__Gammaproteobacteria;o__Enterobacterales;f__Erwiniaceae;g__Pantoea;s__Pantoea_sp._ARC607
PB3_gene_5907	196	WP_025119481.1	1244	112/190(58.95)	6.57e-63	MULTISPECIES: methyltransferase domain-	2647522	d__Bacteria;k__norank;p__Proteobacteria;c__Gammaproteobacteria;o__Enterobacterales;f__Yersiniaceae;g__Serratia;s__norank
PB3_gene_5909	396	XP_016610584.1	507	155/410(37.80)	1.23e-73	protoporphyrinogen oxidase [Spizellomyces punctatus DAOM BR117]	645134	d__Eukaryota;k__Fungi;p__Chytridiomycota;c__Chytridiomycetes;o__Spizellomycetales;f__Spizellomycetaceae;g__Spizellomyces;s__Spizellomyces_punctatus
PB3_gene_5913	81	AXC32738.1	569	70/80(87.50)	2.39e-37	hypothetical protein DSD31_12905 [Raoultella sp.	2259647	d__Bacteria;k__norank;p__Proteobacteria;c__Gammaproteobacteria;o__Enterobacterales;f__Enterobacteriaceae;g__Raoultella;s__Raoultella_sp._X13

PB3_gene_5914	78	WP_075182089.1	133	77/78(98.72)	2.26e-49	hypothetical protein [Pantoea sp. 1.19]	1925589	d__Bacteria;k__norank;p__Proteobacteria;c__Gammaproteobacteria;o__Enterobacterales;f__Erwiniaceae;g__Pantoea;s__Pantoea_sp._1.19
PB3_gene_5922	402	WP_126534700.1	386	370/383(96.61)	7.60e-272	hypothetical protein [Enterobacter sp. WCHEn090040]	2497436	d__Bacteria;k__norank;p__Proteobacteria;c__Gammaproteobacteria;o__Enterobacterales;f__Enterobacteriaceae;g__Enterobacter;s__Enterobacter_sp._WCHEn090040
PB3_gene_5927	103	ORM60119.1	390	97/103(94.17)	1.73e-53	capsid protein [Pantoea rodasii]	1076549	d__Bacteria;k__norank;p__Proteobacteria;c__Gammaproteobacteria;o__Enterobacterales;f__Erwiniaceae;g__Pantoea;s__Pantoea_rodasii
PB3_gene_5941	333	RJQ63084.1	471	120/299(40.13)	3.73e-70	aspartate aminotransferase family protein [Desulfobacteraceae bacterium]	2049433	d__Bacteria;k__norank;p__Proteobacteria;c__Deltaproteobacteria;o__Desulfobacterales;f__Desulfobacteraceae;g__norank;s__Desulfobacteraceae_bacterium
PB3_gene_5976	88	EZI33758.1	88	79/88(89.77)	3.73e-53	hypothetical protein BW31_02123 [Pantoea	549	d__Bacteria;k__norank;p__Proteobacteria;c__Gammaproteobacteria;o__Enterobacterales;f__Erwiniaceae;g__Pantoea;s__Pantoea_agglomerans
PB3_gene_5994	78	SJZ85864.1	69	59/67(88.06)	3.03e-28	hypothetical protein SAMN03097723_2260 [Pantoea agglomerans]	470933	d__Bacteria;k__norank;p__Proteobacteria;c__Gammaproteobacteria;o__Enterobacterales;f__Erwiniaceae;g__Pantoea;s__Pantoea_eucalypti
PB3_gene_5995	238	SJZ85846.1	224	191/224(85.27)	1.23e-136	Methyltransferase domain-containing protein [Pantoea agglomerans]	470933	d__Bacteria;k__norank;p__Proteobacteria;c__Gammaproteobacteria;o__Enterobacterales;f__Erwiniaceae;g__Pantoea;s__Pantoea_eucalypti
PB3_gene_6004	409	RXX91571.1	153	78/152(51.32)	4.28e-47	hypothetical protein DD587_31600, partial [Klebsiella	573	d__Bacteria;k__norank;p__Proteobacteria;c__Gammaproteobacteria;o__Enterobacterales;f__Enterobacteriaceae;g__Klebsiella;s__Klebsiella_pneumoniae
PB3_gene_6007	401	WP_063630801.1	1054	219/391(56.01)	5.98e-94	ankyrin repeat domain-containing protein [Wolbachia endosymbiont of Laodelphax striatella]	368602	d__Bacteria;k__norank;p__Proteobacteria;c__Alphaproteobacteria;o__Rickettsiales;f__Anaplasmataceae;g__Wolbachia;s__Wolbachia_endosymbiont_of_Laodelphax_striatellus
PB3_gene_6008	350	WP_107319650.1	390	312/349(89.40)	2.86e-213	benzoate/H(+) symporter BenE family transporter [Pantoea	470934	d__Bacteria;k__norank;p__Proteobacteria;c__Gammaproteobacteria;o__Enterobacterales;f__Erwiniaceae;g__Pantoea;s__Pantoea_vagans
PB3_gene_6028	404	BBA84562.1	547	335/396(84.60)	6.12e-232	60 kDa chaperonin [endosymbiont of Euscepes	650377	d__Bacteria;k__norank;p__Proteobacteria;c__Gammaproteobacteria;o__norank;f__norank;g__norank;s__endosymbiont_of_Euscepes_postfasciatus
PB3_gene_6033	400	AXC32738.1	569	372/399(93.23)	1.25e-268	hypothetical protein DSD31_12905 [Raoultella sp.	2259647	d__Bacteria;k__norank;p__Proteobacteria;c__Gammaproteobacteria;o__Enterobacterales;f__Enterobacteriaceae;g__Raoultella;s__Raoultella_sp._X13
PB3_gene_6036	84	WP_077064454.1	784	79/84(94.05)	5.11e-43	type VI secretion system tip protein VgrG [Enterobacter	2494701	d__Bacteria;k__norank;p__Proteobacteria;c__Gammaproteobacteria;o__Enterobacterales;f__Enterobacteriaceae;g__Enterobacter;s__Enterobacter_chengduensis
PB3_gene_6045	257	PCG11634.1	256	254/256(99.22)	3.06e-179	DNA-binding response regulator [Wolbachia pipientis]	None	Unclassified
PB3_gene_6046	112	ORM85229.1	69	69/69(100.00)	2.94e-46	hypothetical protein HA42_03050 [Pantoea deleyi]	470932	d__Bacteria;k__norank;p__Proteobacteria;c__Gammaproteobacteria;o__Enterobacterales;f__Erwiniaceae;g__Pantoea;s__Pantoea_deleyi
PB3_gene_6049	326	WP_053092199.1	272	197/257(76.65)	3.28e-145	hypothetical protein, partial [Pantoea vagans]	470934	d__Bacteria;k__norank;p__Proteobacteria;c__Gammaproteobacteria;o__Enterobacterales;f__Erwiniaceae;g__Pantoea;s__Pantoea_vagans
PB3_gene_6050	44	WP_125312145.1	126	39/44(88.64)	1.35e-18	hypothetical protein [Pantoea dispersa]	59814	d__Bacteria;k__norank;p__Proteobacteria;c__Gammaproteobacteria;o__Enterobacterales;f__Erwiniaceae;g__Pantoea;s__Pantoea_dispersa
PB3_gene_6072	71	WP_104460723.1	111	69/71(97.18)	1.62e-38	XRE family transcriptional regulator [Enterobacter	158836	d__Bacteria;k__norank;p__Proteobacteria;c__Gammaproteobacteria;o__Enterobacterales;f__Enterobacteriaceae;g__Enterobacter;s__Enterobacter_hormaechei
PB3_gene_6073	126	WP_104460724.1	126	122/126(96.83)	8.85e-81	hypothetical protein [Enterobacter hormaechei]	158836	d__Bacteria;k__norank;p__Proteobacteria;c__Gammaproteobacteria;o__Enterobacterales;f__Enterobacteriaceae;g__Enterobacter;s__Enterobacter_hormaechei
PB3_gene_6074	45	WP_104460730.1	136	40/45(88.89)	2.40e-21	hypothetical protein [Enterobacter hormaechei]	158836	d__Bacteria;k__norank;p__Proteobacteria;c__Gammaproteobacteria;o__Enterobacterales;f__Enterobacteriaceae;g__Enterobacter;s__Enterobacter_hormaechei
PB3_gene_6113	220	WP_126690658.1	213	105/213(49.30)	2.07e-69	helix-turn-helix domain-containing protein [Pantoea sp.	None	Unclassified
PB3_gene_6118	145	OOZ40505.1	1236	37/78(47.44)	8.34e-14	hypothetical protein BOW52_05800 [Solemya	1918949	d__Bacteria;k__norank;p__Proteobacteria;c__Gammaproteobacteria;o__norank;f__norank;g__norank;s__Solemya_elarraichensis_gill_symbiont
PB3_gene_6120	105	WP_019236487.1	272	50/87(57.47)	5.24e-23	hypothetical protein [Wolbachia pipientis]	955	d__Bacteria;k__norank;p__Proteobacteria;c__Alphaproteobacteria;o__Rickettsiales;f__Anaplasmataceae;g__Wolbachia;s__Wolbachia_pipientis

PB3_gene_6121	214	PCG11813.1	192	191/192(99.48)	8.29e-135	RlmE family RNA methyltransferase [Wolbachia	None	Unclassified
PB3_gene_6126	210	WP_077944996.1	984	95/213(44.60)	2.94e-56	hypothetical protein [Salmonella enterica]	None	Unclassified
PB3_gene_6136	376	WP_045140818.1	666	362/376(96.28)	2.26e-243	DNA sulfur modification protein DndD [Pantoea agglomerans]	549	d__Bacteria;k__norank;p__Proteobacteria;c__Gammaproteobacteria;o__Enterobacterales;f__Erwiniaceae;g__Pantoea;s__Pantoea_agglomerans
PB3_gene_6137	71	WP_062749655.1	74	62/71(87.32)	1.28e-37	hypothetical protein [Erwinia persicina]	55211	d__Bacteria;k__norank;p__Proteobacteria;c__Gammaproteobacteria;o__Enterobacterales;f__Erwiniaceae;g__Erwinia;s__Erwinia_persicina
PB3_gene_6157	131	WP_126690652.1	139	90/130(69.23)	1.59e-69	hypothetical protein [Pantoea sp. YU22]	None	Unclassified
PB3_gene_6158	63	WP_115191271.1	78	26/62(41.94)	3.28e-09	hypothetical protein [Escherichia coli]	57706	d__Bacteria;k__norank;p__Proteobacteria;c__Gammaproteobacteria;o__Enterobacterales;f__Enterobacteriaceae;g__Citrobacter;s__Citrobacter_braakii
PB3_gene_6163	147	WP_045140818.1	666	142/147(96.60)	3.47e-90	DNA sulfur modification protein DndD [Pantoea agglomerans]	549	d__Bacteria;k__norank;p__Proteobacteria;c__Gammaproteobacteria;o__Enterobacterales;f__Erwiniaceae;g__Pantoea;s__Pantoea_agglomerans
PB3_gene_6173	188	WP_107320546.1	177	177/177(100.00)	1.74e-119	phospholipase D family protein [Pantoea vagans]	470934	d__Bacteria;k__norank;p__Proteobacteria;c__Gammaproteobacteria;o__Enterobacterales;f__Erwiniaceae;g__Pantoea;s__Pantoea_vagans
PB3_gene_6179	90	WP_120458490.1	107	64/89(71.91)	1.74e-35	hypothetical protein [Erwiniaceae bacterium IIIF1SW-P2]	None	Unclassified
PB3_gene_6180	127	WP_039392066.1	107	106/107(99.07)	5.14e-74	hypothetical protein [Pantoea agglomerans]	549	d__Bacteria;k__norank;p__Proteobacteria;c__Gammaproteobacteria;o__Enterobacterales;f__Erwiniaceae;g__Pantoea;s__Pantoea_agglomerans
PB3_gene_6187	147	EXU74075.1	131	121/131(92.37)	2.26e-76	transposase [Erwinia mallotivora]	69222	d__Bacteria;k__norank;p__Proteobacteria;c__Gammaproteobacteria;o__Enterobacterales;f__Erwiniaceae;g__Erwinia;s__Erwinia_mallotivora
PB3_gene_6191	88	QAV43378.1	361	88/88(100.00)	1.63e-54	DNA sulfur modification protein DndB [Pantoea agglomerans]	549	d__Bacteria;k__norank;p__Proteobacteria;c__Gammaproteobacteria;o__Enterobacterales;f__Erwiniaceae;g__Pantoea;s__Pantoea_agglomerans
PB3_gene_6192	103	CNF29651.1	92	34/48(70.83)	3.57e-14	Uncharacterised protein [Yersinia rohdei]	29485	d__Bacteria;k__norank;p__Proteobacteria;c__Gammaproteobacteria;o__Enterobacterales;f__Yersiniaceae;g__Yersinia;s__Yersinia_rohdei
PB3_gene_6193	181	SQC29497.1	482	80/178(44.94)	2.39e-42	Uncharacterised protein [Klebsiella pneumoniae]	573	d__Bacteria;k__norank;p__Proteobacteria;c__Gammaproteobacteria;o__Enterobacterales;f__Enterobacteriaceae;g__Klebsiella;s__Klebsiella_pneumoniae
PB3_gene_6194	182	SQC29497.1	482	71/164(43.29)	4.42e-32	Uncharacterised protein [Klebsiella pneumoniae]	573	d__Bacteria;k__norank;p__Proteobacteria;c__Gammaproteobacteria;o__Enterobacterales;f__Enterobacteriaceae;g__Klebsiella;s__Klebsiella_pneumoniae
PB3_gene_6195	168	WP_089414007.1	383	148/168(88.10)	1.16e-107	UDP-galactopyranose mutase [Pantoea agglomerans]	53335	d__Bacteria;k__norank;p__Proteobacteria;c__Gammaproteobacteria;o__Enterobacterales;f__Erwiniaceae;g__Pantoea;s__norank
PB3_gene_6198	151	AEA96315.1	344	98/151(64.90)	1.01e-61	hypothetical protein MADE_1000830 [Alteromonas	1774373	d__Bacteria;k__norank;p__Proteobacteria;c__Gammaproteobacteria;o__Alteromonadales;f__Alteromonadaceae;g__Alteromonas;s__Alteromonas_mediterranea
PB3_gene_6209	92	WP_090392796.1	781	90/92(97.83)	4.42e-52	type VI secretion system tip protein VgrG [Enterobacter sp.	1566251	d__Bacteria;k__norank;p__Proteobacteria;c__Gammaproteobacteria;o__Enterobacterales;f__Enterobacteriaceae;g__Enterobacter;s__Enterobacter_sp._NFIX58
PB3_gene_6240	265	WP_111138398.1	302	249/265(93.96)	1.05e-167	ABC transporter substrate-binding protein [Pantoea sp.	2027922	d__Bacteria;k__norank;p__Proteobacteria;c__Gammaproteobacteria;o__Enterobacterales;f__Erwiniaceae;g__Pantoea;s__Pantoea_sp._ARC607
PB3_gene_6241	91	ORM75790.1	264	91/91(100.00)	8.02e-52	ectoine/hydroxyectoine ABC transporter ATP-binding protein EhuA [Pantoea deleyi]	470932	d__Bacteria;k__norank;p__Proteobacteria;c__Gammaproteobacteria;o__Enterobacterales;f__Erwiniaceae;g__Pantoea;s__Pantoea_deleyi
PB3_gene_6242	355	WP_064739302.1	429	288/355(81.13)	1.12e-195	arsenic transporter [Enterobacter ludwigii]	299767	d__Bacteria;k__norank;p__Proteobacteria;c__Gammaproteobacteria;o__Enterobacterales;f__Enterobacteriaceae;g__Enterobacter;s__Enterobacter_ludwigii
PB3_gene_6249	67	ORX92126.1	388	38/62(61.29)	3.05e-21	Oxysterol-binding protein [Basidiobolus meristosporus CBS 931.73]	1314790	d__Eukaryota;k__Fungi;p__Zoopagomycota;c__Basidiobolomycetes;o__Basidiobolales;f__Basidiobolaceae;g__Basidiobolus;s__Basidiobolus_meristosporus
PB3_gene_6255	210	WP_109654246.1	214	209/210(99.52)	1.58e-152	MULTISPECIES: DNA methylase [Pantoea]	549	d__Bacteria;k__norank;p__Proteobacteria;c__Gammaproteobacteria;o__Enterobacterales;f__Erwiniaceae;g__Pantoea;s__Pantoea_agglomerans
PB3_gene_6256	76	WP_094106184.1	76	76/76(100.00)	1.66e-46	hypothetical protein [Pantoea brenneri]	472694	d__Bacteria;k__norank;p__Proteobacteria;c__Gammaproteobacteria;o__Enterobacterales;f__Erwiniaceae;g__Pantoea;s__Pantoea_brenneri

PB3_gene_6264	51	EIB98393.1	51	46/51(90.20)	9.91e-26	hypothetical protein S7A_07765 [Pantoea sp. Sc1]	593105	d__Bacteria;k__norank;p__Proteobacteria;c__Gammaproteobacteria;o__Enterobacterales;f__Erwiniaceae;g__Pantoea;s__Pantoea_sp._Sc1
PB3_gene_6267	347	WP_050861774.1	843	337/347(97.12)	2.73e-246	type VI secretion system tip protein VgrG [Enterobacter	208224	d__Bacteria;k__norank;p__Proteobacteria;c__Gammaproteobacteria;o__Enterobacterales;f__Enterobacteriaceae;g__Enterobacter;s__Enterobacter_kobei
PB3_gene_6281	209	AVE16788.1	302	208/209(99.52)	4.44e-145	inorganic pyrophosphatase (plasmid) [Pantoea vagans]	470934	d__Bacteria;k__norank;p__Proteobacteria;c__Gammaproteobacteria;o__Enterobacterales;f__Erwiniaceae;g__Pantoea;s__Pantoea_vagans
PB3_gene_6282	68	RUT16562.1	192	68/68(100.00)	2.54e-36	uracil-DNA glycosylase [Erwinia amylovora]	552	d__Bacteria;k__norank;p__Proteobacteria;c__Gammaproteobacteria;o__Enterobacterales;f__Erwiniaceae;g__Erwinia;s__Erwinia_amylovora
PB3_gene_6290	122	KIS45922.1	136	54/66(81.82)	5.17e-30	hypothetical protein LG58_1325 [Kosakonia radicincitans YD4]	1544796	d__Bacteria;k__norank;p__Proteobacteria;c__Gammaproteobacteria;o__Enterobacterales;f__Enterobacteriaceae;g__Kosakonia;s__Kosakonia_radicincitans
PB3_gene_6291	43	PVH13208.1	83	33/36(91.67)	2.28e-16	hypothetical protein APX76_01355 [Escherichia coli]	562	d__Bacteria;k__norank;p__Proteobacteria;c__Gammaproteobacteria;o__Enterobacterales;f__Enterobacteriaceae;g__Escherichia;s__Escherichia_coli
PB3_gene_6297	193	RYX86063.1	245	54/182(29.67)	2.15e-15	hypothetical protein EON73_02690 [bacterium]	1869227	d__Bacteria;k__norank;p__norank;c__norank;o__norank;f__norank;g__norank;s__bacterium
PB3_gene_6303	62	SUX28335.1	359	59/62(95.16)	3.27e-36	Uncharacterized protein conserved in bacteria [Cedecea	158484	d__Bacteria;k__norank;p__Proteobacteria;c__Gammaproteobacteria;o__Enterobacterales;f__Enterobacteriaceae;g__Cedecea;s__Cedecea_davisae
PB3_gene_6335	172	KIK12982.1	917	55/151(36.42)	1.91e-11	hypothetical protein PISMIDRAFT_18316 [Pisolithus microcarpus 441]	765257	d__Eukaryota;k__Fungi;p__Basidiomycota;c__Agaricomycetes;o__Boletales;f__Pisolithaceae;g__Pisolithus;s__Pisolithus_microcarpus
PB3_gene_6348	134	AGJ98468.1	214	114/133(85.71)	3.62e-69	hypothetical protein wNo_00070 [Wolbachia endosymbiont of Drosophila simulans wNo]	1236908	d__Bacteria;k__norank;p__Proteobacteria;c__Alphaproteobacteria;o__Rickettsiales;f__Anaplasmataceae;g__Wolbachia;s__Wolbachia_endosymbiont_of_Drosophila_simulans
PB3_gene_6358	291	WP_045139705.1	334	281/291(96.56)	1.88e-212	hypothetical protein [Pantoea agglomerans]	549	d__Bacteria;k__norank;p__Proteobacteria;c__Gammaproteobacteria;o__Enterobacterales;f__Erwiniaceae;g__Pantoea;s__Pantoea_agglomerans
PB3_gene_6363	177	WP_128086729.1	417	177/177(100.00)	3.63e-119	PTS sugar transporter subunit IIC [Pantoea deleyi]	470932	d__Bacteria;k__norank;p__Proteobacteria;c__Gammaproteobacteria;o__Enterobacterales;f__Erwiniaceae;g__Pantoea;s__Pantoea_deleyi
PB3_gene_6375	310	ORM81100.1	466	309/310(99.68)	3.47e-224	asparagine--tRNA ligase [Pantoea deleyi]	470932	d__Bacteria;k__norank;p__Proteobacteria;c__Gammaproteobacteria;o__Enterobacterales;f__Erwiniaceae;g__Pantoea;s__Pantoea_deleyi
PB3_gene_6376	73	WP_039392458.1	187	73/73(100.00)	2.53e-39	ribosomal RNA adenine dimethylase [Pantoea	549	d__Bacteria;k__norank;p__Proteobacteria;c__Gammaproteobacteria;o__Enterobacterales;f__Erwiniaceae;g__Pantoea;s__Pantoea_agglomerans
PB3_gene_6377	219	WP_039392460.1	458	218/219(99.54)	4.96e-155	DUF3560 domain-containing protein [Pantoea agglomerans]	549	d__Bacteria;k__norank;p__Proteobacteria;c__Gammaproteobacteria;o__Enterobacterales;f__Erwiniaceae;g__Pantoea;s__Pantoea_agglomerans
PB3_gene_6393	54	WP_127464243.1	79	51/52(98.08)	1.11e-32	hypothetical protein [Wolbachia endosymbiont of Bemisia tabaci]	None	Unclassified
PB3_gene_6401	277	WP_054688209.1	287	275/277(99.28)	1.74e-193	OmpA family protein [Pantoea stewartii]	66269	d__Bacteria;k__norank;p__Proteobacteria;c__Gammaproteobacteria;o__Enterobacterales;f__Erwiniaceae;g__Pantoea;s__Pantoea_stewartii
PB3_gene_6408	310	QBK88702.1	639	109/246(44.31)	3.40e-57	DEAD/SNF2-like helicase [Mimivirus LCMiAC01]	2506608	d__Viruses;k__Bamfordvirae;p__Nucleocytoviricota;c__Megaviricetes;o__Imitervirales;f__Mimiviridae;g__Mimivirus;s__Mimivirus_LCMiAC01
PB3_gene_6416	89	WP_094106209.1	126	69/89(77.53)	4.16e-42	hypothetical protein [Pantoea brenneri]	472694	d__Bacteria;k__norank;p__Proteobacteria;c__Gammaproteobacteria;o__Enterobacterales;f__Erwiniaceae;g__Pantoea;s__Pantoea_brenneri
PB3_gene_6417	233	WP_052206227.1	507	181/234(77.35)	1.55e-119	type II secretion protein E [Pantoea rodasii]	1076549	d__Bacteria;k__norank;p__Proteobacteria;c__Gammaproteobacteria;o__Enterobacterales;f__Erwiniaceae;g__Pantoea;s__Pantoea_rodasii
PB3_gene_6426	271	WP_072279413.1	267	172/269(63.94)	6.62e-106	adhesin [Escherichia coli]	562	d__Bacteria;k__norank;p__Proteobacteria;c__Gammaproteobacteria;o__Enterobacterales;f__Enterobacteriaceae;g__Escherichia;s__Escherichia_coli
PB3_gene_6432	317	WP_080170785.1	608	266/317(83.91)	1.99e-197	hypothetical protein, partial [Salmonella enterica]	28901	d__Bacteria;k__norank;p__Proteobacteria;c__Gammaproteobacteria;o__Enterobacterales;f__Enterobacteriaceae;g__Salmonella;s__Salmonella_enterica
PB3_gene_6455	163	SAW54606.1	174	159/163(97.55)	5.32e-100	Arsenic efflux pump protein [Klebsiella pneumoniae]	573	d__Bacteria;k__norank;p__Proteobacteria;c__Gammaproteobacteria;o__Enterobacterales;f__Enterobacteriaceae;g__Klebsiella;s__Klebsiella_pneumoniae
PB3_gene_6476	137	WP_039390282.1	1711	136/137(99.27)	2.34e-72	conjugative transfer relaxase/helicase TraI [Pantoea agglomerans]	549	d__Bacteria;k__norank;p__Proteobacteria;c__Gammaproteobacteria;o__Enterobacterales;f__Erwiniaceae;g__Pantoea;s__Pantoea_agglomerans

PB3_gene_6477	170	WP_111534978.1	170	159/170(93.53)	3.32e-104	hypothetical protein [Pantoea agglomerans]	None	Unclassified
PB3_gene_6483	267	WP_123548089.1	1255	70/236(29.66)	6.37e-13	hypothetical protein [Frigoribacterium sp. PhB107]	2485172	d__Bacteria;k__norank;p__Actinobacteria;c__Actinobacteria;o__Micrococcales;f__Microbacteriaceae;g__Frigoribacterium;s__Frigoribacterium_sp._PhB107
PB3_gene_6488	139	AZU37553.1	139	137/138(99.28)	9.15e-87	cation:proton antiporter [Wolbachia endosymbiont of	215173	d__Bacteria;k__norank;p__Proteobacteria;c__Alphaproteobacteria;o__Rickettsiales;f__Anaplasmataceae;g__Wolbachia;s__Wolbachia_endosymbiont_of_Bemisia_tabaci
PB3_gene_6490	199	WP_109990914.1	300	180/183(98.36)	2.55e-120	hypothetical protein [Lactococcus lactis]	1358	d__Bacteria;k__norank;p__Firmicutes;c__Bacilli;o__Lactobacillales;f__Streptococcaceae;g__Lactococcus;s__Lactococcus_lactis
PB3_gene_6491	113	WP_109990913.1	184	106/113(93.81)	1.58e-67	hypothetical protein [Lactococcus lactis]	1358	d__Bacteria;k__norank;p__Firmicutes;c__Bacilli;o__Lactobacillales;f__Streptococcaceae;g__Lactococcus;s__Lactococcus_lactis
PB3_gene_6495	121	OSY19256.1	647	37/102(36.27)	4.97e-12	hypothetical protein B0D75_01370 [Entomoplasmatales bacterium	None	Unclassified
PB3_gene_6496	309	WP_124234899.1	474	183/315(58.10)	1.20e-117	hypothetical protein [Erwinia psidii]	69224	d__Bacteria;k__norank;p__Proteobacteria;c__Gammaproteobacteria;o__Enterobacterales;f__Erwiniaceae;g__Erwinia;s__Erwinia_psidii
PB3_gene_6507	253	OSY19256.1	647	99/201(49.25)	5.55e-59	hypothetical protein B0D75_01370 [Entomoplasmatales bacterium	None	Unclassified
PB3_gene_6508	256	WP_052259273.1	724	231/258(89.53)	3.34e-140	type IV conjugative transfer system coupling protein TraD [Pantoea agglomerans]	53335	d__Bacteria;k__norank;p__Proteobacteria;c__Gammaproteobacteria;o__Enterobacterales;f__Erwiniaceae;g__Pantoea;s__norank
PB3_gene_6522	284	QAV47603.1	334	284/284(100.00)	4.72e-211	conjugal transfer protein TraU (plasmid) [Pantoea agglomerans]	549	d__Bacteria;k__norank;p__Proteobacteria;c__Gammaproteobacteria;o__Enterobacterales;f__Erwiniaceae;g__Pantoea;s__Pantoea_agglomerans
PB3_gene_6535	74	WP_058973333.1	154	74/74(100.00)	4.08e-46	head completion/stabilization protein [Type-D symbiont of	1560356	d__Bacteria;k__norank;p__Proteobacteria;c__Gammaproteobacteria;o__Enterobacterales;f__Enterobacteriaceae;g__norank;s__Type-D_symbiont_of_Plautia_stali
PB3_gene_6536	196	WP_058973334.1	215	196/196(100.00)	2.60e-134	hypothetical protein [Type-D symbiont of Plautia stali]	1560356	d__Bacteria;k__norank;p__Proteobacteria;c__Gammaproteobacteria;o__Enterobacterales;f__Enterobacteriaceae;g__norank;s__Type-D_symbiont_of_Plautia_stali
PB3_gene_6542	303	EZI33763.1	586	302/303(99.67)	2.04e-219	Terminase ATPase subunit [Pantoea agglomerans]	549	d__Bacteria;k__norank;p__Proteobacteria;c__Gammaproteobacteria;o__Enterobacterales;f__Erwiniaceae;g__Pantoea;s__Pantoea_agglomerans
PB3_gene_6551	45	SXR73217.1	124	34/45(75.56)	7.14e-16	phage portal protein, PBSX family [Klebsiella pneumoniae]	None	Unclassified
PB3_gene_6552	246	ORX87509.1	292	98/194(50.52)	2.54e-56	hypothetical protein BCR32DRAFT_215295, partial [Anaeromyces robustus]	1754192	d__Eukaryota;k__Fungi;p__Chytridiomycota;c__Neocallimastigomycetes;o__Neocallimastigales;f__Neocallimastigaceae;g__Anaeromyces;s__Anaeromyces_robustus
PB3_gene_6559	45	AOA49568.1	43	41/43(95.35)	2.96e-22	tail chaperone protein [Wolbachia phage WO]	112596	d__Viruses;k__norank;p__Uroviricota;c__Caudoviricetes;o__Caudovirales;f__Myoviridae;g__norank;s__Wolbachia_phage_WO
PB3_gene_6578	192	SAJ31832.1	95	48/99(48.48)	2.75e-07	Uncharacterised protein [Enterobacter cloacae]	550	d__Bacteria;k__norank;p__Proteobacteria;c__Gammaproteobacteria;o__Enterobacterales;f__Enterobacteriaceae;g__Enterobacter;s__Enterobacter_cloacae
PB3_gene_6580	190	WP_111139253.1	190	188/190(98.95)	1.48e-119	divisome-associated lipoprotein YraP [Pantoea sp. ARC607]	2027922	d__Bacteria;k__norank;p__Proteobacteria;c__Gammaproteobacteria;o__Enterobacterales;f__Erwiniaceae;g__Pantoea;s__Pantoea_sp._ARC607
PB3_gene_6581	68	ORM82459.1	234	66/68(97.06)	2.14e-38	hypothetical protein HA42_08140 [Pantoea deleyi]	470932	d__Bacteria;k__norank;p__Proteobacteria;c__Gammaproteobacteria;o__Enterobacterales;f__Erwiniaceae;g__Pantoea;s__Pantoea_deleyi
PB3_gene_6587	165	ORM82492.1	249	159/165(96.36)	9.17e-107	esterase [Pantoea deleyi]	470932	d__Bacteria;k__norank;p__Proteobacteria;c__Gammaproteobacteria;o__Enterobacterales;f__Erwiniaceae;g__Pantoea;s__Pantoea_deleyi
PB3_gene_6592	110	WP_094106201.1	127	71/100(71.00)	1.64e-47	HNH endonuclease [Pantoea brenneri]	472694	d__Bacteria;k__norank;p__Proteobacteria;c__Gammaproteobacteria;o__Enterobacterales;f__Erwiniaceae;g__Pantoea;s__Pantoea_brenneri
PB3_gene_6596	201	WP_038645333.1	397	194/201(96.52)	7.21e-131	DUF4102 domain-containing protein [Pantoea rwandensis]	1076550	d__Bacteria;k__norank;p__Proteobacteria;c__Gammaproteobacteria;o__Enterobacterales;f__Erwiniaceae;g__Pantoea;s__Pantoea_rwandensis
PB3_gene_6600	266	WP_064085234.1	549	264/266(99.25)	2.34e-153	chaperonin GroEL [Wolbachia endosymbiont of Dactylopius coccus]	None	Unclassified

PB3_gene_6622	271	WP_094106224.1	259	208/259(80.31)	1.83e-134	conjugal transfer protein [Pantoea brenneri]	472694	d__Bacteria;k__norank;p__Proteobacteria;c__Gammaproteobacteria;o__Enterobacterales;f__Erwiniaceae;g__Pantoea;s__Pantoea_brenneri
PB3_gene_6633	291	WP_107320569.1	1593	289/291(99.31)	1.50e-185	DNA primase [Pantoea vagans]	470934	d__Bacteria;k__norank;p__Proteobacteria;c__Gammaproteobacteria;o__Enterobacterales;f__Erwiniaceae;g__Pantoea;s__Pantoea_vagans
PB3_gene_6635	121	WP_101700225.1	156	121/121(100.00)	8.00e-80	hypothetical protein [Citrobacter sp. L55]	1981983	d__Bacteria;k__norank;p__Proteobacteria;c__Gammaproteobacteria;o__Enterobacterales;f__Enterobacteriaceae;g__Citrobacter;s__Citrobacter_sp._L55
PB3_gene_6641	291	WP_025119481.1	1244	173/281(61.57)	2.47e-122	MULTISPECIES: methyltransferase domain-	2647522	d__Bacteria;k__norank;p__Proteobacteria;c__Gammaproteobacteria;o__Enterobacterales;f__Yersiniaceae;g__Serratia;s__norank
PB3_gene_6644	290	KEP45674.1	871	55/228(24.12)	9.60e-07	transposase family Tnp2 protein, partial [Rhizoctonia solani 123E]	1423351	d__Eukaryota;k__Fungi;p__Basidiomycota;c__Agaricomycetes;o__Cantharellales;f__Ceratobasidiaceae;g__Rhizoctonia;s__Rhizoctonia_solani
PB3_gene_6653	99	OMH84864.1	339	77/95(81.05)	4.13e-54	Casein kinase II subunit alpha [Zancudomyces culisetae]	1213189	d__Eukaryota;k__Fungi;p__Zoopagomycota;c__Harpellomycetes;o__Harpellales;f__Legeriomycetaceae;g__Zancudomyces;s__Zancudomyces_culisetae
PB3_gene_6654	64	WP_119917006.1	463	64/64(100.00)	9.50e-37	PAAR domain-containing protein [Enterobacter chuandaensis]	2497875	d__Bacteria;k__norank;p__Proteobacteria;c__Gammaproteobacteria;o__Enterobacterales;f__Enterobacteriaceae;g__Enterobacter;s__Enterobacter_chuandaensis
PB3_gene_6655	221	WP_104556465.1	236	215/221(97.29)	5.95e-156	hypothetical protein [Enterobacter sichuanensis]	2071710	d__Bacteria;k__norank;p__Proteobacteria;c__Gammaproteobacteria;o__Enterobacterales;f__Enterobacteriaceae;g__Enterobacter;s__Enterobacter_sichuanensis
PB3_gene_6668	92	WP_094106234.1	711	88/92(95.65)	3.95e-45	conjugal transfer protein TraY [Pantoea brenneri]	472694	d__Bacteria;k__norank;p__Proteobacteria;c__Gammaproteobacteria;o__Enterobacterales;f__Erwiniaceae;g__Pantoea;s__Pantoea_brenneri
PB3_gene_6670	80	WP_094106244.1	128	46/80(57.50)	5.11e-22	hypothetical protein [Pantoea brenneri]	472694	d__Bacteria;k__norank;p__Proteobacteria;c__Gammaproteobacteria;o__Enterobacterales;f__Erwiniaceae;g__Pantoea;s__Pantoea_brenneri
PB3_gene_6678	92	OJH31393.1	335	87/92(94.57)	2.83e-45	Phosphocholine transferase AnkX [Wolbachia endosymbiont of Armadillidium vulgare]	77039	d__Bacteria;k__norank;p__Proteobacteria;c__Alphaproteobacteria;o__Rickettsiales;f__Anaplasmataceae;g__Wolbachia;s__Wolbachia_endosymbiont_of_Armadillidium_vulgare
PB3_gene_6686	125	WP_071884807.1	126	79/121(65.29)	5.24e-50	MULTISPECIES: hypothetical protein [Enterobacteriaceae]	543	d__Bacteria;k__norank;p__Proteobacteria;c__Gammaproteobacteria;o__Enterobacterales;f__Enterobacteriaceae;g__norank;s__norank
PB3_gene_6687	117	ETX49356.1	346	67/118(56.78)	7.20e-34	hypothetical protein P812_00172 [Serratia marcescens BIDMC 50]	1400187	d__Bacteria;k__norank;p__Proteobacteria;c__Gammaproteobacteria;o__Enterobacterales;f__Yersiniaceae;g__Serratia;s__Serratia_marcescens
PB3_gene_6688	287	WP_107320548.1	728	287/287(100.00)	2.32e-198	conjugal transfer protein [Pantoea vagans]	470934	d__Bacteria;k__norank;p__Proteobacteria;c__Gammaproteobacteria;o__Enterobacterales;f__Erwiniaceae;g__Pantoea;s__Pantoea_vagans
PB3_gene_6698	56	KNE70186.1	525	34/45(75.56)	6.84e-15	hypothetical protein AMAG_15154 [Allomyces macrogynus ATCC 38327]	578462	d__Eukaryota;k__Fungi;p__Blastocladiomycota;c__Blastocladiomycetes;o__Blastocladiales;f__Blastocladiaceae;g__Allomyces;s__Allomyces_macrogynus
PB3_gene_6705	129	WP_089414092.1	153	74/125(59.20)	8.79e-39	hypothetical protein [Pantoea agglomerans]	549	d__Bacteria;k__norank;p__Proteobacteria;c__Gammaproteobacteria;o__Enterobacterales;f__Erwiniaceae;g__Pantoea;s__Pantoea_agglomerans
PB3_gene_6706	153	WP_053092197.1	307	98/204(48.04)	6.27e-44	hypothetical protein [Pantoea vagans]	470934	d__Bacteria;k__norank;p__Proteobacteria;c__Gammaproteobacteria;o__Enterobacterales;f__Erwiniaceae;g__Pantoea;s__Pantoea_vagans
PB3_gene_6711	284	PZL85803.1	572	277/283(97.88)	2.64e-150	PTS fructose transporter subunit IIBC [Pantoea sp. ARC270]	2027923	d__Bacteria;k__norank;p__Proteobacteria;c__Gammaproteobacteria;o__Enterobacterales;f__Erwiniaceae;g__Pantoea;s__Pantoea_sp._ARC270
PB3_gene_6713	126	WP_110412182.1	162	126/126(100.00)	1.71e-79	MULTISPECIES: DUF3577 domain-containing protein	1903409	d__Bacteria;k__norank;p__Proteobacteria;c__Gammaproteobacteria;o__Enterobacterales;f__Erwiniaceae;g__norank;s__norank
PB3_gene_6714	49	WP_064703429.1	346	49/49(100.00)	2.94e-26	DUF1738 domain-containing protein [Pantoea agglomerans]	549	d__Bacteria;k__norank;p__Proteobacteria;c__Gammaproteobacteria;o__Enterobacterales;f__Erwiniaceae;g__Pantoea;s__Pantoea_agglomerans
PB3_gene_6716	144	AGJ98554.1	144	133/144(92.36)	9.35e-87	hypothetical protein wNo_01070 [Wolbachia endosymbiont of Drosophila simulans wNo]	1236908	d__Bacteria;k__norank;p__Proteobacteria;c__Alphaproteobacteria;o__Rickettsiales;f__Anaplasmataceae;g__Wolbachia;s__Wolbachia_endosymbiont_of_Drosophila_simulans
PB3_gene_6723	48	WP_074143850.1	86	47/48(97.92)	6.80e-29	hypothetical protein, partial [Enterobacter cloacae]	None	Unclassified
PB3_gene_6729	228	CNI41522.1	251	159/227(70.04)	1.04e-106	hemagglutinin-like secreted protein [Yersinia kristensenii]	28152	d__Bacteria;k__norank;p__Proteobacteria;c__Gammaproteobacteria;o__Enterobacterales;f__Yersiniaceae;g__Yersinia;s__Yersinia_kristensenii
PB3_gene_6730	182	WP_110412176.1	191	180/182(98.90)	2.23e-119	restriction endonuclease [Pantoea vagans]	470934	d__Bacteria;k__norank;p__Proteobacteria;c__Gammaproteobacteria;o__Enterobacterales;f__Erwiniaceae;g__Pantoea;s__Pantoea_vagans

PB3_gene_6738	282	WP_108784584.1	390	273/282(96.81)	6.14e-190	hypothetical protein, partial [Wolbachia endosymbiont of Bemisia tabaci]	215173	d__Bacteria;k__norank;p__Proteobacteria;c__Alphaproteobacteria;o__Rickettsiales;f__Anaplasmataceae;g__Wolbachia;s__Wolbachia_endosymbiont_of_Bemisia_tabaci
PB3_gene_6744	255	EHT05184.1	256	221/255(86.67)	2.90e-172	hypothetical protein HMPREF9694_05509 [Klebsiella michiganensis]	1134687	d__Bacteria;k__norank;p__Proteobacteria;c__Gammaproteobacteria;o__Enterobacterales;f__Enterobacteriaceae;g__Klebsiella;s__Klebsiella_michiganensis
PB3_gene_6750	59	RTN75897.1	224	53/59(89.83)	1.25e-29	hypothetical protein EKN81_20210 [Enterobacter	61645	d__Bacteria;k__norank;p__Proteobacteria;c__Gammaproteobacteria;o__Enterobacterales;f__Enterobacteriaceae;g__Enterobacter;s__Enterobacter_asburiae
PB3_gene_6758	100	WP_115740096.1	143	65/99(65.66)	1.32e-35	tail fiber assembly protein [Escherichia coli]	562	d__Bacteria;k__norank;p__Proteobacteria;c__Gammaproteobacteria;o__Enterobacterales;f__Enterobacteriaceae;g__Escherichia;s__Escherichia_coli
PB3_gene_6759	133	ORM75140.1	133	112/132(84.85)	7.94e-76	phage tail protein [Pantoea deleyi]	470932	d__Bacteria;k__norank;p__Proteobacteria;c__Gammaproteobacteria;o__Enterobacterales;f__Erwiniaceae;g__Pantoea;s__Pantoea_deleyi
PB3_gene_6762	124	EZI33771.1	124	120/124(96.77)	5.58e-83	hypothetical protein BW31_02136 [Pantoea	549	d__Bacteria;k__norank;p__Proteobacteria;c__Gammaproteobacteria;o__Enterobacterales;f__Erwiniaceae;g__Pantoea;s__Pantoea_agglomerans
PB3_gene_6763	132	KPA06348.1	169	130/132(98.48)	1.84e-93	glycoside hydrolase [Pantoea agglomerans]	549	d__Bacteria;k__norank;p__Proteobacteria;c__Gammaproteobacteria;o__Enterobacterales;f__Erwiniaceae;g__Pantoea;s__Pantoea_agglomerans
PB3_gene_6787	278	WP_104925532.1	1629	87/142(61.27)	4.85e-44	hypothetical protein [Rahnella sp. ERMR1:05]	1805933	d__Bacteria;k__norank;p__Proteobacteria;c__Gammaproteobacteria;o__Enterobacterales;f__Yersiniaceae;g__Rahnella;s__Rahnella_sp._ERMR1:05
PB3_gene_6799	136	WP_110664056.1	224	59/69(85.51)	4.08e-33	FhaB [Serratia marcescens]	615	d__Bacteria;k__norank;p__Proteobacteria;c__Gammaproteobacteria;o__Enterobacterales;f__Yersiniaceae;g__Serratia;s__Serratia_marcescens
PB3_gene_6809	94	EWG73336.1	175	44/92(47.83)	7.49e-19	hypothetical protein P348_01961 [Enterobacter sp. DC3]	1395579	d__Bacteria;k__norank;p__Proteobacteria;c__Gammaproteobacteria;o__Enterobacterales;f__Enterobacteriaceae;g__Enterobacter;s__Enterobacter_sp._DC3
PB3_gene_6820	73	AGJ98717.1	262	73/73(100.00)	4.51e-45	Succinate dehydrogenase, iron-sulfur protein [Wolbachia endosymbiont of Drosophila simulans wNo]	1236908	d__Bacteria;k__norank;p__Proteobacteria;c__Alphaproteobacteria;o__Rickettsiales;f__Anaplasmataceae;g__Wolbachia;s__Wolbachia_endosymbiont_of_Drosophila_simulans
PB3_gene_6822	67	WP_109990915.1	427	67/67(100.00)	1.36e-40	hypothetical protein [Lactococcus lactis]	1358	d__Bacteria;k__norank;p__Firmicutes;c__Bacilli;o__Lactobacillales;f__Streptococcaceae;g__Lactococcus;s__Lactococcus_lactis
PB3_gene_6833	183	CBJ48264.1	385	164/183(89.62)	3.10e-117	conjugal transfer protein (plasmid) [Erwinia amylovora	716540	d__Bacteria;k__norank;p__Proteobacteria;c__Gammaproteobacteria;o__Enterobacterales;f__Erwiniaceae;g__Erwinia;s__Erwinia_amylovora
PB3_gene_6834	90	WP_097098617.1	191	86/90(95.56)	3.77e-54	conjugal transfer protein, partial [Enterobacteriaceae bacterium JKS000234]	None	Unclassified
PB3_gene_6835	108	WP_032742446.1	140	51/87(58.62)	3.19e-28	hypothetical protein [Klebsiella michiganensis]	1134687	d__Bacteria;k__norank;p__Proteobacteria;c__Gammaproteobacteria;o__Enterobacterales;f__Enterobacteriaceae;g__Klebsiella;s__Klebsiella_michiganensis
PB3_gene_6837	231	WP_111139205.1	296	220/231(95.24)	6.36e-154	dTDP-4-dehydrorhamnose reductase [Pantoea sp. ARC607]	2027922	d__Bacteria;k__norank;p__Proteobacteria;c__Gammaproteobacteria;o__Enterobacterales;f__Erwiniaceae;g__Pantoea;s__Pantoea_sp._ARC607
PB3_gene_6850	221	EPH95573.1	260	58/213(27.23)	1.45e-14	hypothetical protein D922_01181 [Enterococcus faecalis 06-MB-DW-09]	1260358	d__Bacteria;k__norank;p__Firmicutes;c__Bacilli;o__Lactobacillales;f__Enterococcaceae;g__Enterococcus;s__Enterococcus_faecalis
PB3_gene_6853	272	AQT25384.1	473	81/130(62.31)	9.40e-46	amidase [Staphylococcus phage St 134]	1958922	d__Viruses;k__norank;p__Uroviricota;c__Caudoviricetes;o__Caudovirales;f__Podoviridae;g__Andhravirus;s__Staphylococcus_virus_St134
PB3_gene_6854	272	WP_111140045.1	333	270/272(99.26)	8.58e-190	ABC transporter ATP-binding protein [Pantoea sp. ARC607]	2027922	d__Bacteria;k__norank;p__Proteobacteria;c__Gammaproteobacteria;o__Enterobacterales;f__Erwiniaceae;g__Pantoea;s__Pantoea_sp._ARC607
PB3_gene_6859	49	WP_115388382.1	701	47/49(95.92)	1.71e-24	type IV conjugative transfer system coupling protein TraD [Pantoea agglomerans]	549	d__Bacteria;k__norank;p__Proteobacteria;c__Gammaproteobacteria;o__Enterobacterales;f__Erwiniaceae;g__Pantoea;s__Pantoea_agglomerans
PB3_gene_6863	241	WP_095706996.1	555	211/241(87.55)	8.90e-140	ShlB/FhaC/HecB family hemolysin secretion/activation protein [Pantoea vagans]	470934	d__Bacteria;k__norank;p__Proteobacteria;c__Gammaproteobacteria;o__Enterobacterales;f__Erwiniaceae;g__Pantoea;s__Pantoea_vagans

PB3_gene_6873	53	WP_094106237.1	347	50/53(94.34)	4.97e-26	conjugal transfer protein [Pantoea brenneri]	472694	d__Bacteria;k__norank;p__Proteobacteria;c__Gammaproteobacteria;o__Enterobacterales;f__Erwiniaceae;g__Pantoea;s__Pantoea_brenneri
PB3_gene_6875	223	WP_110412190.1	459	222/223(99.55)	5.69e-153	replicative DNA helicase [Pantoea vagans]	1903409	d__Bacteria;k__norank;p__Proteobacteria;c__Gammaproteobacteria;o__Enterobacterales;f__Erwiniaceae;g__norank;s__norank
PB3_gene_6880	71	WP_107320599.1	558	71/71(100.00)	7.35e-40	DUF4942 domain-containing protein [Pantoea vagans]	470934	d__Bacteria;k__norank;p__Proteobacteria;c__Gammaproteobacteria;o__Enterobacterales;f__Erwiniaceae;g__Pantoea;s__Pantoea_vagans
PB3_gene_6881	148	WP_107320598.1	148	147/148(99.32)	4.27e-103	hypothetical protein [Pantoea vagans]	470934	d__Bacteria;k__norank;p__Proteobacteria;c__Gammaproteobacteria;o__Enterobacterales;f__Erwiniaceae;g__Pantoea;s__Pantoea_vagans
PB3_gene_6893	67	BAS69360.1	2361	29/52(55.77)	6.24e-10	hypothetical protein [Negevirus Nona 1]	1615760	d__Viruses;k__norank;p__norank;c__norank;o__norank;f__norank;g__Negevirus;s__Negevirus_Nona_1
PB3_gene_6899	43	KDA94316.1	125	38/43(88.37)	1.54e-19	hypothetical protein T296_11755 [Pantoea agglomerans Eh318]	1408177	d__Bacteria;k__norank;p__Proteobacteria;c__Gammaproteobacteria;o__Enterobacterales;f__Erwiniaceae;g__Pantoea;s__Pantoea_agglomerans
PB3_gene_6902	267	ADO10835.1	353	209/262(79.77)	8.54e-140	Uncharacterized 72.8 kDa protein in lys 3'region [Pantoea vagans C9-1]	712898	d__Bacteria;k__norank;p__Proteobacteria;c__Gammaproteobacteria;o__Enterobacterales;f__Erwiniaceae;g__Pantoea;s__Pantoea_vagans
PB3_gene_6916	227	WP_111138411.1	256	210/227(92.51)	8.86e-146	MerR family transcriptional regulator [Pantoea sp. ARC607]	2027922	d__Bacteria;k__norank;p__Proteobacteria;c__Gammaproteobacteria;o__Enterobacterales;f__Erwiniaceae;g__Pantoea;s__Pantoea_sp._ARC607
PB3_gene_6917	265	WP_105595680.1	776	143/266(53.76)	6.60e-97	autotransporter outer membrane beta-barrel domain-containing protein [Pantoea coffeiphila]	1465635	d__Bacteria;k__norank;p__Proteobacteria;c__Gammaproteobacteria;o__Enterobacterales;f__Erwiniaceae;g__Pantoea;s__Pantoea_coffeiphila
PB3_gene_6918	248	WP_111139220.1	542	238/248(95.97)	1.34e-165	isovaleryl-CoA dehydrogenase [Pantoea sp. ARC607]	2027922	d__Bacteria;k__norank;p__Proteobacteria;c__Gammaproteobacteria;o__Enterobacterales;f__Erwiniaceae;g__Pantoea;s__Pantoea_sp._ARC607
PB3_gene_6923	265	ORM79066.1	725	246/265(92.83)	2.23e-160	diguanylate cyclase [Pantoea deleyi]	470932	d__Bacteria;k__norank;p__Proteobacteria;c__Gammaproteobacteria;o__Enterobacterales;f__Erwiniaceae;g__Pantoea;s__Pantoea_deleyi
PB3_gene_6927	190	WP_107320592.1	190	190/190(100.00)	3.28e-141	hypothetical protein [Pantoea vagans]	470934	d__Bacteria;k__norank;p__Proteobacteria;c__Gammaproteobacteria;o__Enterobacterales;f__Erwiniaceae;g__Pantoea;s__Pantoea_vagans
PB3_gene_6928	71	WP_107320593.1	49	49/49(100.00)	2.28e-27	succinate dehydrogenase flavoprotein subunit [Pantoea vagans]	53335	d__Bacteria;k__norank;p__Proteobacteria;c__Gammaproteobacteria;o__Enterobacterales;f__Erwiniaceae;g__Pantoea;s__norank
PB3_gene_6949	263	WP_064690377.1	901	259/263(98.48)	2.95e-167	phage tail tape measure protein [Pantoea agglomerans]	549	d__Bacteria;k__norank;p__Proteobacteria;c__Gammaproteobacteria;o__Enterobacterales;f__Erwiniaceae;g__Pantoea;s__Pantoea_agglomerans
PB3_gene_6950	263	ORM81408.1	403	241/256(94.14)	4.33e-167	SfnB family sulfur acquisition oxidoreductase [Pantoea deleyi]	470932	d__Bacteria;k__norank;p__Proteobacteria;c__Gammaproteobacteria;o__Enterobacterales;f__Erwiniaceae;g__Pantoea;s__Pantoea_deleyi
PB3_gene_6952	263	WP_111140340.1	757	213/262(81.30)	3.92e-140	pyrroloquinoline quinone biosynthesis protein PqqF [Pantoea sp. ARC607]	2027922	d__Bacteria;k__norank;p__Proteobacteria;c__Gammaproteobacteria;o__Enterobacterales;f__Erwiniaceae;g__Pantoea;s__Pantoea_sp._ARC607
PB3_gene_6956	122	WP_019106102.1	175	120/122(98.36)	7.48e-78	host cell division inhibitor Icd-like protein [Pantoea ananatis]	553	d__Bacteria;k__norank;p__Proteobacteria;c__Gammaproteobacteria;o__Enterobacterales;f__Erwiniaceae;g__Pantoea;s__Pantoea_ananatis
PB3_gene_6957	88	WP_064703297.1	88	87/88(98.86)	2.83e-57	hypothetical protein [Pantoea agglomerans]	549	d__Bacteria;k__norank;p__Proteobacteria;c__Gammaproteobacteria;o__Enterobacterales;f__Erwiniaceae;g__Pantoea;s__Pantoea_agglomerans
PB3_gene_6959	59	SUI47952.1	193	58/59(98.31)	2.18e-32	Type I restriction-modification system methyltransferase subunit [Serratia marcescens]	615	d__Bacteria;k__norank;p__Proteobacteria;c__Gammaproteobacteria;o__Enterobacterales;f__Yersiniaceae;g__Serratia;s__Serratia_marcescens
PB3_gene_6960	65	WP_088276633.1	678	65/65(100.00)	1.12e-31	hypothetical protein [Cronobacter sakazakii]	28141	d__Bacteria;k__norank;p__Proteobacteria;c__Gammaproteobacteria;o__Enterobacterales;f__Enterobacteriaceae;g__Cronobacter;s__Cronobacter_sakazakii
PB3_gene_6962	195	AAN79424.1	430	112/195(57.44)	2.69e-56	Hypothetical protein c0951 [Escherichia coli CFT073]	199310	d__Bacteria;k__norank;p__Proteobacteria;c__Gammaproteobacteria;o__Enterobacterales;f__Enterobacteriaceae;g__Escherichia;s__Escherichia_coli
PB3_gene_6964	104	WP_098050860.1	201	103/104(99.04)	4.89e-70	hypothetical protein [Pantoea agglomerans]	None	Unclassified
PB3_gene_6965	75	WP_098050860.1	201	47/52(90.38)	4.56e-22	hypothetical protein [Pantoea agglomerans]	None	Unclassified

PB3_gene_6966	59	RNA77474.1	1305	58/59(98.31)	8.70e-31	type IV secretion protein Rhs [[Curtobacterium] plantarum]	221276	d__Bacteria;k__norank;p__Proteobacteria;c__Gammaproteobacteria;o__Enterobacterales;f__Erwiniaceae;g__Pantoea;s__[Curtobacterium]_plantarum
PB3_gene_6975	257	WP_052264816.1	357	248/257(96.50)	1.42e-169	redox-regulated ATPase YchF, partial [Wolbachia endosymbiont of Operophtera brumata]	1620420	d__Bacteria;k__norank;p__Proteobacteria;c__Alphaproteobacteria;o__Rickettsiales;f__Anaplasmataceae;g__Wolbachia;s__Wolbachia_endosymbiont_of_Operophtera_brumata
PB3_gene_6976	130	WP_010248720.1	143	126/130(96.92)	5.95e-86	phage tail protein [Pantoea agglomerans]	549	d__Bacteria;k__norank;p__Proteobacteria;c__Gammaproteobacteria;o__Enterobacterales;f__Erwiniaceae;g__Pantoea;s__Pantoea_agglomerans
PB3_gene_6977	117	SWT00827.1	95	35/91(38.46)	1.43e-06	Uncharacterised protein [Klebsiella pneumoniae]	573	d__Bacteria;k__norank;p__Proteobacteria;c__Gammaproteobacteria;o__Enterobacterales;f__Enterobacteriaceae;g__Klebsiella;s__Klebsiella_pneumoniae
PB3_gene_6985	260	WP_108784238.1	773	256/260(98.46)	2.25e-164	translation initiation factor IF-2 [Wolbachia endosymbiont of Bemisia tabaci]	215173	d__Bacteria;k__norank;p__Proteobacteria;c__Alphaproteobacteria;o__Rickettsiales;f__Anaplasmataceae;g__Wolbachia;s__Wolbachia_endosymbiont_of_Bemisia_tabaci
PB3_gene_6993	201	AKA86872.1	294	164/201(81.59)	7.42e-112	AbiTii (plasmid) [Klebsiella pneumoniae subsp. pneumoniae]	72407	d__Bacteria;k__norank;p__Proteobacteria;c__Gammaproteobacteria;o__Enterobacterales;f__Enterobacteriaceae;g__Klebsiella;s__Klebsiella_pneumoniae
PB3_gene_7001	240	ELP25058.1	353	225/240(93.75)	4.17e-161	Prophage tail protein [Pantoea agglomerans 299R]	1261128	d__Bacteria;k__norank;p__Proteobacteria;c__Gammaproteobacteria;o__Enterobacterales;f__Erwiniaceae;g__Pantoea;s__Pantoea_agglomerans
PB3_gene_7012	216	WP_090392788.1	595	199/217(91.71)	1.71e-141	hypothetical protein [Enterobacter sp. NFIX58]	1566251	d__Bacteria;k__norank;p__Proteobacteria;c__Gammaproteobacteria;o__Enterobacterales;f__Enterobacteriaceae;g__Enterobacter;s__Enterobacter_sp._NFIX58
PB3_gene_7020	145	KDA92375.1	136	134/136(98.53)	5.24e-87	Z-ring-associated protein [Pantoea agglomerans Eh318]	1408177	d__Bacteria;k__norank;p__Proteobacteria;c__Gammaproteobacteria;o__Enterobacterales;f__Erwiniaceae;g__Pantoea;s__Pantoea_agglomerans
PB3_gene_7027	73	WP_107320553.1	97	69/73(94.52)	6.43e-36	hypothetical protein [Pantoea vagans]	None	Unclassified
PB3_gene_7028	67	WP_107320554.1	67	67/67(100.00)	4.05e-43	hypothetical protein [Pantoea vagans]	470934	d__Bacteria;k__norank;p__Proteobacteria;c__Gammaproteobacteria;o__Enterobacterales;f__Erwiniaceae;g__Pantoea;s__Pantoea_vagans
PB3_gene_7029	96	WP_107320555.1	139	93/96(96.88)	6.43e-62	hypothetical protein [Pantoea vagans]	470934	d__Bacteria;k__norank;p__Proteobacteria;c__Gammaproteobacteria;o__Enterobacterales;f__Erwiniaceae;g__Pantoea;s__Pantoea_vagans
PB3_gene_7041	95	RAH29121.1	203	93/95(97.89)	3.40e-61	phage tail protein I [Pantoea agglomerans]	549	d__Bacteria;k__norank;p__Proteobacteria;c__Gammaproteobacteria;o__Enterobacterales;f__Erwiniaceae;g__Pantoea;s__Pantoea_agglomerans
PB3_gene_7042	161	WP_061902077.1	304	156/161(96.89)	7.41e-101	baseplate assembly protein [Pantoea agglomerans]	549	d__Bacteria;k__norank;p__Proteobacteria;c__Gammaproteobacteria;o__Enterobacterales;f__Erwiniaceae;g__Pantoea;s__Pantoea_agglomerans
PB3_gene_7065	251	WP_094106203.1	360	178/251(70.92)	9.08e-125	hypothetical protein [Pantoea brenneri]	472694	d__Bacteria;k__norank;p__Proteobacteria;c__Gammaproteobacteria;o__Enterobacterales;f__Erwiniaceae;g__Pantoea;s__Pantoea_brenneri
PB3_gene_7067	214	WP_024082743.1	335	183/215(85.12)	3.38e-116	conjugal transfer protein TraN [Erwinia amylovora]	552	d__Bacteria;k__norank;p__Proteobacteria;c__Gammaproteobacteria;o__Enterobacterales;f__Erwiniaceae;g__Erwinia;s__Erwinia_amylovora
PB3_gene_7070	240	KPA05702.1	270	232/240(96.67)	6.09e-162	hypothetical protein PAP10c_3108 [Pantoea	549	d__Bacteria;k__norank;p__Proteobacteria;c__Gammaproteobacteria;o__Enterobacterales;f__Erwiniaceae;g__Pantoea;s__Pantoea_agglomerans
PB3_gene_7072	128	WP_107320563.1	128	116/128(90.62)	2.06e-71	conjugal transfer protein [Pantoea vagans]	470934	d__Bacteria;k__norank;p__Proteobacteria;c__Gammaproteobacteria;o__Enterobacterales;f__Erwiniaceae;g__Pantoea;s__Pantoea_vagans
PB3_gene_7075	90	XP_026615823.1	1897	39/98(39.80)	1.66e-12	hypothetical protein CDV56_104210 [Aspergillus	41047	d__Eukaryota;k__Fungi;p__Ascomycota;c__Eurotiomycetes;o__Eurotiales;f__Aspergillaceae;g__Aspergillus;s__Aspergillus_thermomutatus
PB3_gene_7076	64	EIF46677.1	179	59/64(92.19)	5.62e-34	tata-binding protein associated factor mot1 [Brettanomyces bruxellensis AWRI1499]	1124627	d__Eukaryota;k__Fungi;p__Ascomycota;c__Saccharomycetes;o__Saccharomycetales;f__Pichiaceae;g__Brettanomyces;s__Brettanomyces_bruxellensis
PB3_gene_7079	135	WP_110412193.1	156	133/135(98.52)	3.83e-93	hypothetical protein [Pantoea vagans]	470934	d__Bacteria;k__norank;p__Proteobacteria;c__Gammaproteobacteria;o__Enterobacterales;f__Erwiniaceae;g__Pantoea;s__Pantoea_vagans
PB3_gene_7080	121	WP_110412192.1	191	119/121(98.35)	9.43e-82	XRE family transcriptional regulator [Pantoea vagans]	1903409	d__Bacteria;k__norank;p__Proteobacteria;c__Gammaproteobacteria;o__Enterobacterales;f__Erwiniaceae;g__norank;s__norank
PB3_gene_7083	160	WP_094106223.1	439	135/159(84.91)	3.05e-87	conjugal transfer protein [Pantoea brenneri]	472694	d__Bacteria;k__norank;p__Proteobacteria;c__Gammaproteobacteria;o__Enterobacterales;f__Erwiniaceae;g__Pantoea;s__Pantoea_brenneri
PB3_gene_7084	88	KHJ66385.1	228	86/88(97.73)	4.62e-55	conjugal transfer protein [Pantoea rodasii]	1076549	d__Bacteria;k__norank;p__Proteobacteria;c__Gammaproteobacteria;o__Enterobacterales;f__Erwiniaceae;g__Pantoea;s__Pantoea_rodasii

PB3_gene_7091	76	WP_050491385.1	180	74/76(97.37)	3.24e-44	hypothetical protein [Pantoea agglomerans]	549	d__Bacteria;k__norank;p__Proteobacteria;c__Gammaproteobacteria;o__Enterobacterales;f__Erwiniaceae;g__Pantoea;s__Pantoea_agglomerans
PB3_gene_7092	122	WP_120458374.1	182	107/122(87.70)	2.89e-58	single-stranded DNA-binding protein [Erwiniaceae bacterium IIIF1SW-P2]	2364647	d__Bacteria;k__norank;p__Proteobacteria;c__Gammaproteobacteria;o__Enterobacterales;f__Erwiniaceae;g__Kalamiella;s__Kalamiella_piersonii
PB3_gene_7095	73	WP_061703122.1	61	35/45(77.78)	6.40e-16	hypothetical protein [Pantoea vagans]	None	Unclassified
PB3_gene_7102	65	ORM81709.1	287	65/65(100.00)	1.78e-35	incFII family plasmid replication initiator RepA [Pantoea deleyi]	470932	d__Bacteria;k__norank;p__Proteobacteria;c__Gammaproteobacteria;o__Enterobacterales;f__Erwiniaceae;g__Pantoea;s__Pantoea_deleyi
PB3_gene_7106	166	WP_107320565.1	246	162/166(97.59)	7.83e-103	conjugal transfer protein [Pantoea vagans]	470934	d__Bacteria;k__norank;p__Proteobacteria;c__Gammaproteobacteria;o__Enterobacterales;f__Erwiniaceae;g__Pantoea;s__Pantoea_vagans
PB3_gene_7108	102	XP_025181195.1	328	54/81(66.67)	2.77e-30	tetrapyrrole biosynthesis, porphobilinogen synthase [Rhizophagus irregularis DAOM 181602=DAOM 197198]	747089	d__Eukaryota;k__Fungi;p__Mucoromycota;c__Glomeromycetes;o__Glomerales;f__Glomeraceae;g__Rhizophagus;s__Rhizophagus_irregularis
PB3_gene_7112	66	WP_129321181.1	354	30/59(50.85)	7.93e-11	DUF4817 domain-containing protein [Enterobacter cloacae complex sp. 4DZ3-17B2]	2511990	d__Bacteria;k__norank;p__Proteobacteria;c__Gammaproteobacteria;o__Enterobacterales;f__Enterobacteriaceae;g__Enterobacter;s__Enterobacter_cloacae_complex_sp._4DZ3-17B2
PB3_gene_7118	173	RLJ22762.1	1286	45/152(29.61)	1.50e-11	hypothetical protein DJ031_00280 [bacterium endosymbiont of Escarpia	1221601	d__Bacteria;k__norank;p__norank;c__norank;o__norank;f__norank;g__norank;s__bacterium_endosymbiont_of_Escarpia_laminata
PB3_gene_7120	247	WP_129992308.1	787	245/247(99.19)	6.00e-169	SAM-dependent DNA methyltransferase [Rahnella sp. CFA14(1/10)]	2511203	d__Bacteria;k__norank;p__Proteobacteria;c__Gammaproteobacteria;o__Enterobacterales;f__Yersiniaceae;g__Rahnella;s__Rahnella_sp._CFA14(1/10)
PB3_gene_7122	246	WP_107320552.1	711	245/246(99.59)	3.12e-159	conjugal transfer protein TraY [Pantoea vagans]	470934	d__Bacteria;k__norank;p__Proteobacteria;c__Gammaproteobacteria;o__Enterobacterales;f__Erwiniaceae;g__Pantoea;s__Pantoea_vagans
PB3_gene_7124	99	AGJ99495.1	577	48/56(85.71)	1.82e-18	Ankyrin repeat domain protein [Wolbachia endosymbiont of Drosophila simulans wNo]	1236908	d__Bacteria;k__norank;p__Proteobacteria;c__Alphaproteobacteria;o__Rickettsiales;f__Anaplasmataceae;g__Wolbachia;s__Wolbachia_endosymbiont_of_Drosophila_simulans
PB3_gene_7138	198	WP_126690588.1	564	185/198(93.43)	2.67e-133	ShlB/FhaC/HecB family hemolysin secretion/activation protein [Pantoea sp. YU22]	None	Unclassified
PB3_gene_7159	234	ELP25069.1	569	227/234(97.01)	8.02e-162	Phage terminase large subunit [Pantoea agglomerans 299R]	1261128	d__Bacteria;k__norank;p__Proteobacteria;c__Gammaproteobacteria;o__Enterobacterales;f__Erwiniaceae;g__Pantoea;s__Pantoea_agglomerans
PB3_gene_7167	202	WP_107320548.1	728	201/202(99.50)	4.89e-137	conjugal transfer protein [Pantoea vagans]	470934	d__Bacteria;k__norank;p__Proteobacteria;c__Gammaproteobacteria;o__Enterobacterales;f__Erwiniaceae;g__Pantoea;s__Pantoea_vagans
PB3_gene_7168	38	WP_107320549.1	376	38/38(100.00)	5.33e-18	conjugal transfer protein [Pantoea vagans]	470934	d__Bacteria;k__norank;p__Proteobacteria;c__Gammaproteobacteria;o__Enterobacterales;f__Erwiniaceae;g__Pantoea;s__Pantoea_vagans
PB3_gene_7193	239	WP_058973335.1	353	237/239(99.16)	2.08e-163	phage major capsid protein, P2 family [Type-D symbiont of	1560356	d__Bacteria;k__norank;p__Proteobacteria;c__Gammaproteobacteria;o__Enterobacterales;f__Enterobacteriaceae;g__norank;s__Type-D_symbiont_of_Plautia_stali
PB3_gene_7198	83	WP_120458490.1	107	62/82(75.61)	2.94e-32	hypothetical protein [Erwiniaceae bacterium IIIF1SW-P2]	None	Unclassified
PB3_gene_7203	199	ORM84686.1	324	177/199(88.94)	1.24e-128	hydrolase [Pantoea deleyi]	470932	d__Bacteria;k__norank;p__Proteobacteria;c__Gammaproteobacteria;o__Enterobacterales;f__Erwiniaceae;g__Pantoea;s__Pantoea_deleyi
PB3_gene_7208	97	WP_010403908.1	92	92/92(100.00)	8.02e-58	MULTISPECIES: hypothetical protein [Wolbachia]	2640676	d__Bacteria;k__norank;p__Proteobacteria;c__Alphaproteobacteria;o__Rickettsiales;f__Anaplasmataceae;g__Wolbachia;s__norank
PB3_gene_7210	185	WP_024082728.1	192	145/193(75.13)	2.45e-85	type IV pilus biogenesis protein PilP [Erwinia amylovora]	552	d__Bacteria;k__norank;p__Proteobacteria;c__Gammaproteobacteria;o__Enterobacterales;f__Erwiniaceae;g__Erwinia;s__Erwinia_amylovora
PB3_gene_7223	88	OPW99755.1	88	87/88(98.86)	1.85e-53	transposase [Citrobacter sp. A316]	1639132	d__Bacteria;k__norank;p__Proteobacteria;c__Gammaproteobacteria;o__Enterobacterales;f__Enterobacteriaceae;g__Citrobacter;s__Citrobacter_sp._A316

PB3_gene_7224	91	SAX12453.1	112	48/60(80.00)	2.19e-23	insertion element IS2 transposase InsD [Klebsiella pneumoniae]	573	d__Bacteria;k__norank;p__Proteobacteria;c__Gammaproteobacteria;o__Enterobacterales;f__Enterobacteriaceae;g__Klebsiella;s__Klebsiella_pneumoniae
PB3_gene_7229	143	WP_033761770.1	143	139/143(97.20)	1.69e-93	phage terminase small subunit P27 family [Pantoea	None	Unclassified
PB3_gene_7231	77	CEG85123.1	207	32/43(74.42)	3.36e-13	Putative Phenylalanine-4-hydroxylase [Rhizopus	58291	d__Eukaryota;k__Fungi;p__Mucoromycota;c__Mucoromycetes;o__Mucorales;f__Rhizopodaceae;g__Rhizopus;s__Rhizopus_microsporus
PB3_gene_7233	106	WP_039387083.1	106	104/106(98.11)	6.27e-67	type II toxin-antitoxin system RelE/ParE family toxin [Pantoea agglomerans]	549	d__Bacteria;k__norank;p__Proteobacteria;c__Gammaproteobacteria;o__Enterobacterales;f__Erwiniaceae;g__Pantoea;s__Pantoea_agglomerans
PB3_gene_7234	94	WP_039387086.1	94	94/94(100.00)	1.21e-54	XRE family transcriptional regulator [Pantoea agglomerans]	549	d__Bacteria;k__norank;p__Proteobacteria;c__Gammaproteobacteria;o__Enterobacterales;f__Erwiniaceae;g__Pantoea;s__Pantoea_agglomerans
PB3_gene_7243	151	KIC84724.1	153	136/146(93.15)	5.94e-96	hypothetical protein RN49_22695 [Pantoea agglomerans]	549	d__Bacteria;k__norank;p__Proteobacteria;c__Gammaproteobacteria;o__Enterobacterales;f__Erwiniaceae;g__Pantoea;s__Pantoea_agglomerans
PB3_gene_7256	161	WP_099754562.1	154	144/154(93.51)	1.73e-100	lytic transglycosylase [Erwinia sp. OLMDLW33]	None	Unclassified
PB3_gene_7260	226	WP_122983671.1	237	226/226(100.00)	2.02e-160	arsenical resistance protein ArsH [Pantoea brenneri]	1903409	d__Bacteria;k__norank;p__Proteobacteria;c__Gammaproteobacteria;o__Enterobacterales;f__Erwiniaceae;g__norank;s__norank
PB3_gene_7261	154	AYD79805.1	168	69/148(46.62)	9.18e-36	hypothetical protein OGLPLLMI_00039 [Enterobacter phage phiT5282H]	2340712	d__Viruses;k__norank;p__Uroviricota;c__Caudoviricetes;o__Caudovirales;f__Myoviridae;g__Senquatrovirus;s__Enterobacter_phage_phiT5282H
PB3_gene_7270	184	WP_094106228.1	1017	168/184(91.30)	3.98e-112	conjugal transfer protein [Pantoea brenneri]	472694	d__Bacteria;k__norank;p__Proteobacteria;c__Gammaproteobacteria;o__Enterobacterales;f__Erwiniaceae;g__Pantoea;s__Pantoea_brenneri
PB3_gene_7271	58	CBJ48274.1	204	52/58(89.66)	5.13e-30	conjugal transfer protein (plasmid) [Erwinia amylovora	716540	d__Bacteria;k__norank;p__Proteobacteria;c__Gammaproteobacteria;o__Enterobacterales;f__Erwiniaceae;g__Erwinia;s__Erwinia_amylovora
PB3_gene_7276	97	RUS15136.1	703	42/87(48.28)	3.44e-16	dual specificity protein phosphatase, partial [Endogone	2340872	d__Eukaryota;k__Fungi;p__Mucoromycota;c__Endogonomycetes;o__Endogonales;f__Endogonaceae;g__Endogone;s__Endogone_sp._FLAS-F59071
PB3_gene_7286	84	WP_105099079.1	449	75/84(89.29)	3.68e-22	flagellin FliC [Pantoea ananatis]	None	Unclassified
PB3_gene_7298	107	OAB99070.1	249	53/104(50.96)	6.37e-15	hypothetical protein UVM2_127c00390 [Escherichia	562	d__Bacteria;k__norank;p__Proteobacteria;c__Gammaproteobacteria;o__Enterobacterales;f__Enterobacteriaceae;g__Escherichia;s__Escherichia_coli
PB3_gene_7299	47	EOQ76398.1	128	24/44(54.55)	7.43e-09	hypothetical protein WEW_00006, partial	1169351	d__Bacteria;k__norank;p__Proteobacteria;c__Gammaproteobacteria;o__Enterobacterales;f__Enterobacteriaceae;g__Escherichia;s__Escherichia_coli
PB3_gene_7301	55	RBO11935.1	80	46/58(79.31)	1.19e-23	hypothetical protein DSL62_14625 [Pantoea sp.	2259618	d__Bacteria;k__norank;p__Proteobacteria;c__Gammaproteobacteria;o__Enterobacterales;f__Erwiniaceae;g__Pantoea;s__Pantoea_sp._3_1284
PB3_gene_7302	173	WP_111535158.1	194	162/173(93.64)	5.91e-111	DUF2726 domain-containing protein [Pantoea agglomerans]	549	d__Bacteria;k__norank;p__Proteobacteria;c__Gammaproteobacteria;o__Enterobacterales;f__Erwiniaceae;g__Pantoea;s__Pantoea_agglomerans
PB3_gene_7304	105	OUM57044.1	2536	61/80(76.25)	4.56e-35	hypothetical protein PIROE2DRAFT_18093 [Piromyces sp. E2]	73868	d__Eukaryota;k__Fungi;p__Chytridiomycota;c__Neocallimastigomycetes;o__Neocallimastigales;f__Neocallimastigaceae;g__Piromyces;s__Piromyces_sp._E2
PB3_gene_7309	215	WP_094106206.1	434	163/215(75.81)	1.55e-115	hypothetical protein [Pantoea brenneri]	472694	d__Bacteria;k__norank;p__Proteobacteria;c__Gammaproteobacteria;o__Enterobacterales;f__Erwiniaceae;g__Pantoea;s__Pantoea_brenneri
PB3_gene_7315	230	WP_048784474.1	413	204/230(88.70)	4.60e-141	hypothetical protein [Pantoea vagans]	470934	d__Bacteria;k__norank;p__Proteobacteria;c__Gammaproteobacteria;o__Enterobacterales;f__Erwiniaceae;g__Pantoea;s__Pantoea_vagans
PB3_gene_7319	57	POE17328.1	88	55/57(96.49)	2.93e-35	hypothetical protein BV918_13145 [Pectobacterium carotovorum subsp. odoriferum]	78398	d__Bacteria;k__norank;p__Proteobacteria;c__Gammaproteobacteria;o__Enterobacterales;f__Pectobacteriaceae;g__Pectobacterium;s__Pectobacterium_odoriferum
PB3_gene_7321	198	WP_094106165.1	442	190/198(95.96)	8.56e-132	hypothetical protein [Pantoea brenneri]	472694	d__Bacteria;k__norank;p__Proteobacteria;c__Gammaproteobacteria;o__Enterobacterales;f__Erwiniaceae;g__Pantoea;s__Pantoea_brenneri
PB3_gene_7326	186	WP_033763744.1	468	85/120(70.83)	3.74e-50	shufflon system plasmid conjugative transfer pilus tip adhesin PilV [Pantoea	549	d__Bacteria;k__norank;p__Proteobacteria;c__Gammaproteobacteria;o__Enterobacterales;f__Erwiniaceae;g__Pantoea;s__Pantoea_agglomerans

PB3_gene_7328	228	KTS14707.1	750	214/228(93.86)	1.69e-148	replication protein [Pantoea dispersa]	59814	d__Bacteria;k__norank;p__Proteobacteria;c__Gammaproteobacteria;o__Enterobacterales;f__Erwiniaceae;g__Pantoea;s__Pantoea_dispersa
PB3_gene_7331	128	CUU26262.1	200	101/128(78.91)	8.08e-66	hypothetical protein EM595_p1015 (plasmid)	1619313	d__Bacteria;k__norank;p__Proteobacteria;c__Gammaproteobacteria;o__Enterobacterales;f__Erwiniaceae;g__Erwinia;s__Erwinia_gerundensis
PB3_gene_7332	69	CUU26263.1	208	62/68(91.18)	8.93e-38	hypothetical protein EM595_p1016 (plasmid)	1619313	d__Bacteria;k__norank;p__Proteobacteria;c__Gammaproteobacteria;o__Enterobacterales;f__Erwiniaceae;g__Erwinia;s__Erwinia_gerundensis
PB3_gene_7348	192	QAV47614.1	442	157/183(85.79)	7.07e-99	hypothetical protein D1629_23695 (plasmid) [Pantoea	549	d__Bacteria;k__norank;p__Proteobacteria;c__Gammaproteobacteria;o__Enterobacterales;f__Erwiniaceae;g__Pantoea;s__Pantoea_agglomerans
PB3_gene_7349	83	WP_063630957.1	90	80/83(96.39)	1.59e-53	GIY-YIG nuclease family protein [Wolbachia endosymbiont of Laodelphax striatella]	368602	d__Bacteria;k__norank;p__Proteobacteria;c__Alphaproteobacteria;o__Rickettsiales;f__Anaplasmataceae;g__Wolbachia;s__Wolbachia_endosymbiont_of_Laodelphax_striatellus
PB3_gene_7371	104	TCZ22321.1	668	104/104(100.00)	3.55e-68	integrase [Pantoea agglomerans]	549	d__Bacteria;k__norank;p__Proteobacteria;c__Gammaproteobacteria;o__Enterobacterales;f__Erwiniaceae;g__Pantoea;s__Pantoea_agglomerans
PB3_gene_7372	87	WP_082212583.1	149	87/87(100.00)	2.18e-55	translesion error-prone DNA polymerase V autoproteolytic subunit [Erwinia sp. 9145]	1903409	d__Bacteria;k__norank;p__Proteobacteria;c__Gammaproteobacteria;o__Enterobacterales;f__Erwiniaceae;g__norank;s__norank
PB3_gene_7376	224	WP_053092177.1	373	221/224(98.66)	8.45e-152	translesion error-prone DNA polymerase V subunit UmuC [Pantoea vagans]	None	Unclassified
PB3_gene_7381	173	WP_069730093.1	424	173/173(100.00)	3.86e-119	translesion error-prone DNA polymerase V subunit UmuC [Pantoea brenneri]	472694	d__Bacteria;k__norank;p__Proteobacteria;c__Gammaproteobacteria;o__Enterobacterales;f__Erwiniaceae;g__Pantoea;s__Pantoea_brenneri
PB3_gene_7383	83	WP_107320570.1	96	82/83(98.80)	7.80e-53	conjugal transfer protein [Pantoea vagans]	470934	d__Bacteria;k__norank;p__Proteobacteria;c__Gammaproteobacteria;o__Enterobacterales;f__Erwiniaceae;g__Pantoea;s__Pantoea_vagans
PB3_gene_7384	140	WP_107320571.1	388	136/140(97.14)	3.04e-88	plasmid transfer ATPase TraJ [Pantoea vagans]	53335	d__Bacteria;k__norank;p__Proteobacteria;c__Gammaproteobacteria;o__Enterobacterales;f__Erwiniaceae;g__Pantoea;s__norank
PB3_gene_7385	223	WP_059202256.1	302	201/223(90.13)	9.55e-146	hypothetical protein [Serratia fonticola]	47917	d__Bacteria;k__norank;p__Proteobacteria;c__Gammaproteobacteria;o__Enterobacterales;f__Yersiniaceae;g__Serratia;s__Serratia_fonticola
PB3_gene_7389	151	SJZ30642.1	272	145/151(96.03)	1.51e-90	Protein of unknown function [Pantoea agglomerans]	470933	d__Bacteria;k__norank;p__Proteobacteria;c__Gammaproteobacteria;o__Enterobacterales;f__Erwiniaceae;g__Pantoea;s__Pantoea_eucalypti
PB3_gene_7409	178	KWT95166.1	494	75/188(39.89)	6.76e-25	hypothetical protein APY03_2517 [Variovorax sp.	207745	d__Bacteria;k__norank;p__Proteobacteria;c__Betaproteobacteria;o__Burkholderiales;f__Comamonadaceae;g__Variovorax;s__Variovorax_sp._WDL1
PB3_gene_7412	196	PKK75287.1	184	58/201(28.86)	2.29e-06	hypothetical protein RhiirC2_736951 [Rhizophagus	588596	d__Eukaryota;k__Fungi;p__Mucoromycota;c__Glomeromycetes;o__Glomerales;f__Glomeraceae;g__Rhizophagus;s__Rhizophagus_irregularis
PB3_gene_7418	89	WP_061063772.1	192	82/89(92.13)	6.72e-50	phage baseplate assembly protein V [Pantoea vagans]	470934	d__Bacteria;k__norank;p__Proteobacteria;c__Gammaproteobacteria;o__Enterobacterales;f__Erwiniaceae;g__Pantoea;s__Pantoea_vagans
PB3_gene_7419	117	WP_061902076.1	118	113/117(96.58)	1.15e-72	baseplate assembly protein [Pantoea agglomerans]	549	d__Bacteria;k__norank;p__Proteobacteria;c__Gammaproteobacteria;o__Enterobacterales;f__Erwiniaceae;g__Pantoea;s__Pantoea_agglomerans
PB3_gene_7437	217	WP_110412189.1	525	202/217(93.09)	9.86e-133	chromosome partitioning protein ParB [Pantoea vagans]	53335	d__Bacteria;k__norank;p__Proteobacteria;c__Gammaproteobacteria;o__Enterobacterales;f__Erwiniaceae;g__Pantoea;s__norank
PB3_gene_7443	217	WP_107320569.1	1593	212/217(97.70)	2.90e-140	DNA primase [Pantoea vagans]	470934	d__Bacteria;k__norank;p__Proteobacteria;c__Gammaproteobacteria;o__Enterobacterales;f__Erwiniaceae;g__Pantoea;s__Pantoea_vagans
PB3_gene_7446	120	RAR67405.1	176	104/120(86.67)	5.15e-64	phage integrase family protein [Pantoea ananatis]	553	d__Bacteria;k__norank;p__Proteobacteria;c__Gammaproteobacteria;o__Enterobacterales;f__Erwiniaceae;g__Pantoea;s__Pantoea_ananatis
PB3_gene_7447	124	CBJ48267.1	115	107/115(93.04)	9.75e-48	conjugal transfer protein (plasmid) [Erwinia amylovora	716540	d__Bacteria;k__norank;p__Proteobacteria;c__Gammaproteobacteria;o__Enterobacterales;f__Erwiniaceae;g__Erwinia;s__Erwinia_amylovora
PB3_gene_7451	215	WP_111140327.1	836	203/215(94.42)	3.01e-125	copper-exporting P-type ATPase CopA [Pantoea sp. ARC607]	2027922	d__Bacteria;k__norank;p__Proteobacteria;c__Gammaproteobacteria;o__Enterobacterales;f__Erwiniaceae;g__Pantoea;s__Pantoea_sp._ARC607
PB3_gene_7456	214	KDA94214.1	487	176/214(82.24)	2.45e-126	hypothetical protein T296_11980 [Pantoea agglomerans Eh318]	1408177	d__Bacteria;k__norank;p__Proteobacteria;c__Gammaproteobacteria;o__Enterobacterales;f__Erwiniaceae;g__Pantoea;s__Pantoea_agglomerans

PB3_gene_7457	99	KMU90449.1	205	42/78(53.85)	1.45e-23	tyrosine decarboxylase 1 [Coccidioides immitis H538.4]	396776	d__Eukaryota;k__Fungi;p__Ascomycota;c__Eurotiomycetes;o__Onygenales;f__norank;g__Coccidioides;s__Coccidioides_immitis
PB3_gene_7460	214	WP_104085959.1	396	207/214(96.73)	2.58e-139	MULTISPECIES: SfnB family sulfur acquisition oxidoreductase	2630326	d__Bacteria;k__norank;p__Proteobacteria;c__Gammaproteobacteria;o__Enterobacterales;f__Erwiniaceae;g__Pantoea;s__norank
PB3_gene_7463	37	WP_094119891.1	59	37/37(100.00)	9.45e-17	host cell division inhibitor Icd-like protein [Pantoea conspicua]	53335	d__Bacteria;k__norank;p__Proteobacteria;c__Gammaproteobacteria;o__Enterobacterales;f__Erwiniaceae;g__Pantoea;s__norank
PB3_gene_7464	62	WP_126690258.1	62	62/62(100.00)	1.81e-37	toxin-antitoxin system HicB family antitoxin [Pantoea sp.	53335	d__Bacteria;k__norank;p__Proteobacteria;c__Gammaproteobacteria;o__Enterobacterales;f__Erwiniaceae;g__Pantoea;s__norank
PB3_gene_7476	151	WP_010671410.1	151	149/151(98.68)	2.65e-103	hypothetical protein [Pantoea agglomerans]	549	d__Bacteria;k__norank;p__Proteobacteria;c__Gammaproteobacteria;o__Enterobacterales;f__Erwiniaceae;g__Pantoea;s__Pantoea_agglomerans
PB3_gene_7479	205	WP_104085960.1	452	195/205(95.12)	2.87e-139	LLM class flavin-dependent oxidoreductase [Pantoea sp. ICBG 1758]	2071682	d__Bacteria;k__norank;p__Proteobacteria;c__Gammaproteobacteria;o__Enterobacterales;f__Erwiniaceae;g__Pantoea;s__Pantoea_sp._ICBG_1758
PB3_gene_7486	115	SJZ30639.1	107	102/106(96.23)	1.75e-62	hypothetical protein SAMN03097723_0218 [Pantoea agglomerans]	470933	d__Bacteria;k__norank;p__Proteobacteria;c__Gammaproteobacteria;o__Enterobacterales;f__Erwiniaceae;g__Pantoea;s__Pantoea_eucalypti
PB3_gene_7487	97	SJZ30642.1	272	91/97(93.81)	1.23e-59	Protein of unknown function [Pantoea agglomerans]	470933	d__Bacteria;k__norank;p__Proteobacteria;c__Gammaproteobacteria;o__Enterobacterales;f__Erwiniaceae;g__Pantoea;s__Pantoea_eucalypti
PB3_gene_7489	212	AIR84875.1	749	204/212(96.23)	4.05e-141	replication protein [Pantoea rwandensis]	1076550	d__Bacteria;k__norank;p__Proteobacteria;c__Gammaproteobacteria;o__Enterobacterales;f__Erwiniaceae;g__Pantoea;s__Pantoea_rwandensis
PB3_gene_7490	153	WP_010671412.1	286	144/145(99.31)	1.03e-101	ParA family protein [Pantoea agglomerans]	None	Unclassified
PB3_gene_7495	211	APG79155.1	1296	101/199(50.75)	5.09e-52	RNA-dependent RNA polymerase [Hubei tetragnatha maxillosa	1923251	d__Viruses;k__norank;p__norank;c__norank;o__norank;f__norank;g__norank;s__Hubei_tetragnatha_maxillosa_virus_9
PB3_gene_7507	163	SSH62353.1	180	142/157(90.45)	2.66e-99	Uncharacterised protein [Klebsiella pneumoniae]	573	d__Bacteria;k__norank;p__Proteobacteria;c__Gammaproteobacteria;o__Enterobacterales;f__Enterobacteriaceae;g__Klebsiella;s__Klebsiella_pneumoniae
PB3_gene_7515	143	WP_107320601.1	131	124/131(94.66)	5.35e-83	DUF1380 domain-containing protein [Pantoea vagans]	470934	d__Bacteria;k__norank;p__Proteobacteria;c__Gammaproteobacteria;o__Enterobacterales;f__Erwiniaceae;g__Pantoea;s__Pantoea_vagans
PB3_gene_7524	207	WP_064085864.1	1056	204/207(98.55)	6.39e-142	isoleucine--tRNA ligase [Wolbachia endosymbiont of Dactylopius coccus]	None	Unclassified
PB3_gene_7525	207	EIE92290.1	255	71/142(50.00)	9.59e-36	hypothetical protein RO3G_17161 [Rhizopus delemar	246409	d__Eukaryota;k__Fungi;p__Mucoromycota;c__Mucoromycetes;o__Mucorales;f__Rhizopodaceae;g__Rhizopus;s__Rhizopus_delemar
PB3_gene_7537	96	RYA72209.1	130	34/91(37.36)	1.45e-10	hypothetical protein DD592_26335, partial [Enterobacter cloacae complex	2511993	d__Bacteria;k__norank;p__Proteobacteria;c__Gammaproteobacteria;o__Enterobacterales;f__Enterobacteriaceae;g__Enterobacter;s__Enterobacter_cloacae_complex_sp._2DZ2F20B
PB3_gene_7549	205	STM46889.1	218	123/197(62.44)	1.93e-74	Uncharacterised protein [Escherichia coli]	562	d__Bacteria;k__norank;p__Proteobacteria;c__Gammaproteobacteria;o__Enterobacterales;f__Enterobacteriaceae;g__Escherichia;s__Escherichia_coli
PB3_gene_7551	87	WP_095522748.1	121	86/87(98.85)	1.42e-52	transposase [Pantoea	None	Unclassified
PB3_gene_7552	102	KDA93465.1	101	97/101(96.04)	8.16e-61	hypothetical protein T296_17500 [Pantoea agglomerans Eh318]	1408177	d__Bacteria;k__norank;p__Proteobacteria;c__Gammaproteobacteria;o__Enterobacterales;f__Erwiniaceae;g__Pantoea;s__Pantoea_agglomerans
PB3_gene_7559	111	ORM77140.1	258	108/111(97.30)	9.68e-75	transcriptional regulator ExuR [Pantoea deleyi]	470932	d__Bacteria;k__norank;p__Proteobacteria;c__Gammaproteobacteria;o__Enterobacterales;f__Erwiniaceae;g__Pantoea;s__Pantoea_deleyi
PB3_gene_7575	203	WP_111139847.1	811	200/203(98.52)	2.22e-124	mechanosensitive ion channel family protein [Pantoea sp.	2027922	d__Bacteria;k__norank;p__Proteobacteria;c__Gammaproteobacteria;o__Enterobacterales;f__Erwiniaceae;g__Pantoea;s__Pantoea_sp._ARC607
PB3_gene_7581	75	WP_061902057.1	75	74/75(98.67)	1.09e-46	TraR/DksA family transcriptional regulator [Pantoea agglomerans]	549	d__Bacteria;k__norank;p__Proteobacteria;c__Gammaproteobacteria;o__Enterobacterales;f__Erwiniaceae;g__Pantoea;s__Pantoea_agglomerans
PB3_gene_7588	201	WP_111138571.1	211	199/201(99.00)	5.81e-136	HTH-type transcriptional regulator RutR [Pantoea sp.	2027922	d__Bacteria;k__norank;p__Proteobacteria;c__Gammaproteobacteria;o__Enterobacterales;f__Erwiniaceae;g__Pantoea;s__Pantoea_sp._ARC607

PB3_gene_7589	196	SUB07543.1	361	183/194(94.33)	4.74e-129	ADP-ribosyl-[dinitrogen reductase] glycohydrolase [Pantoea agglomerans]	549	d__Bacteria;k__norank;p__Proteobacteria;c__Gammaproteobacteria;o__Enterobacterales;f__Erwiniaceae;g__Pantoea;s__Pantoea_agglomerans
PB3_gene_7590	85	KNE86814.1	78	43/63(68.25)	3.17e-22	hypothetical protein PSTG_19819 [Puccinia striiformis f. sp. tritici PST-78]	1165861	d__Eukaryota;k__Fungi;p__Basidiomycota;c__Pucciniomycetes;o__Pucciniales;f__Pucciniaceae;g__Puccinia;s__Puccinia_striiformis
PB3_gene_7608	59	WP_096073463.1	245	57/59(96.61)	1.29e-30	ABC transporter ATP-binding protein [Pantoea sp. FDAARGOS_194]	53335	d__Bacteria;k__norank;p__Proteobacteria;c__Gammaproteobacteria;o__Enterobacterales;f__Erwiniaceae;g__Pantoea;s__norank
PB3_gene_7609	140	WP_086906093.1	255	132/140(94.29)	1.00e-93	MULTISPECIES: ABC transporter permease [Pantoea]	549	d__Bacteria;k__norank;p__Proteobacteria;c__Gammaproteobacteria;o__Enterobacterales;f__Erwiniaceae;g__Pantoea;s__Pantoea_agglomerans
PB3_gene_7611	155	SUB16642.1	211	152/155(98.06)	1.64e-107	Predicted ATPase [Pantoea agglomerans]	549	d__Bacteria;k__norank;p__Proteobacteria;c__Gammaproteobacteria;o__Enterobacterales;f__Erwiniaceae;g__Pantoea;s__Pantoea_agglomerans
PB3_gene_7612	45	WP_111139483.1	353	42/45(93.33)	4.46e-20	TIGR01620 family protein [Pantoea sp. ARC607]	2027922	d__Bacteria;k__norank;p__Proteobacteria;c__Gammaproteobacteria;o__Enterobacterales;f__Erwiniaceae;g__Pantoea;s__Pantoea_sp._ARC607
PB3_gene_7614	199	EWG64813.1	560	196/199(98.49)	9.32e-123	Inner membrane lipoprotein YiaD precursor [Enterobacter sp. DC3]	1395579	d__Bacteria;k__norank;p__Proteobacteria;c__Gammaproteobacteria;o__Enterobacterales;f__Enterobacteriaceae;g__Enterobacter;s__Enterobacter_sp._DC3
PB3_gene_7631	156	WP_041079171.1	660	89/126(70.63)	1.74e-45	hypothetical protein [Rickettsia asembonensis]	1068590	d__Bacteria;k__norank;p__Proteobacteria;c__Alphaproteobacteria;o__Rickettsiales;f__Rickettsiaceae;g__Rickettsia;s__Rickettsia_asembonensis
SC-1_gene_3	1972	PXF62333.1	642	224/487(46.00)	5.46e-127	hypothetical protein DL796_12065, partial [Kangiella	796379	d__Bacteria;k__norank;p__Proteobacteria;c__Gammaproteobacteria;o__Oceanospirillales;f__Kangiellaceae;g__Kangiella;s__Kangiella_spongicola
SC-1_gene_38	112	WP_073470636.1	91	33/68(48.53)	8.87e-06	hypothetical protein [Enterococcus faecium]	None	Unclassified
SC-1_gene_46	115	RYE27181.1	1800	42/111(37.84)	4.05e-14	DUF1759 domain-containing protein [Sphingobacteriaceae	2021370	d__Bacteria;k__norank;p__Bacteroidetes;c__Sphingobacteriia;o__Sphingobacteriales;f__Sphingobacteriaceae;g__norank;s__Sphingobacteriaceae_bacterium
SC-1_gene_47	442	EAL58245.1	433	165/399(41.35)	9.48e-106	SD27140p [Wolbachia endosymbiont of Drosophila	307502	d__Bacteria;k__norank;p__Proteobacteria;c__Alphaproteobacteria;o__Rickettsiales;f__Anaplasmataceae;g__Wolbachia;s__Wolbachia_endosymbiont_of_Drosophila_ananassae
SC-1_gene_80	470	AKJ07713.1	1006	104/373(27.88)	4.64e-24	Fibronectin, type III domain protein [Archangium gephyra]	48	d__Bacteria;k__norank;p__Proteobacteria;c__Deltaproteobacteria;o__Myxococcales;f__Archangiaceae;g__Archangium;s__Archangium_gephyra
SC-1_gene_113	472	RUM44712.1	232	71/213(33.33)	5.23e-28	hypothetical protein DSY80_04115, partial	2066021	d__Bacteria;k__norank;p__Proteobacteria;c__Deltaproteobacteria;o__Desulfobacterales;f__Desulfobulbaceae;g__Desulfocapsa;s__Desulfocapsa_sp.
SC-1_gene_137	79	WP_120787180.1	355	46/57(80.70)	9.70e-18	arginine kinase [Macrococcus caseolyticus]	None	Unclassified
SC-1_gene_203	389	AGJ98522.1	385	369/385(95.84)	1.97e-268	2-polyprenyl-6-methoxyphenol hydroxylase, fad-dependent oxidoreductase [Wolbachia endosymbiont of Drosophila simulans wNo]	1236908	d__Bacteria;k__norank;p__Proteobacteria;c__Alphaproteobacteria;o__Rickettsiales;f__Anaplasmataceae;g__Wolbachia;s__Wolbachia_endosymbiont_of_Drosophila_simulans
SC-1_gene_204	84	OAM06453.1	70	49/51(96.08)	1.89e-26	hypothetical protein TV41_06825 [Wolbachia endosymbiont of Dactylopius coccus]	1605993	d__Bacteria;k__norank;p__Proteobacteria;c__Alphaproteobacteria;o__Rickettsiales;f__Anaplasmataceae;g__Wolbachia;s__Wolbachia_endosymbiont_of_Dactylopius_coccus
SC-1_gene_208	57	ETE43231.1	289	45/56(80.36)	1.03e-23	hypothetical protein M574_21520, partial [Salmonella enterica subsp. enterica serovar	1347789	d__Bacteria;k__norank;p__Proteobacteria;c__Gammaproteobacteria;o__Enterobacterales;f__Enterobacteriaceae;g__Salmonella;s__Salmonella_enterica
SC-1_gene_212	119	WP_106481696.1	133	32/93(34.41)	2.71e-12	MULTISPECIES: fibronectin type III domain-containing protein [Butyricimonas]	574697	d__Bacteria;k__norank;p__Bacteroidetes;c__Bacteroidia;o__Bacteroidales;f__Odoribacteraceae;g__Butyricimonas;s__norank
SC-1_gene_216	340	WP_082473862.1	368	172/353(48.73)	4.34e-114	cytochrome P450 [Curtobacterium sp. Leaf261]	None	Unclassified
SC-1_gene_233	589	WP_118960785.1	946	201/575(34.96)	4.04e-102	peptidase M16 [Colwellia sp. RSH04]	2305464	d__Bacteria;k__norank;p__Proteobacteria;c__Gammaproteobacteria;o__Alteromonadales;f__Colwelliaceae;g__Colwellia;s__Colwellia_sp._RSH04

SC-1_gene_284	84	WP_017443570.1	392	72/84(85.71)	1.46e-40	hypothetical protein [Rickettsia gravesii]	354585	d__Bacteria;k__norank;p__Proteobacteria;c__Alphaproteobacteria;o__Rickettsiales;f__Rickettsiaceae;g__Rickettsia;s__Rickettsia_gravesii
SC-1_gene_298	112	CEQ41867.1	1971	52/101(51.49)	5.38e-22	SPOSA6832_03617 [Sporidiobolus salmonicolor]	5005	d__Eukaryota;k__Fungi;p__Basidiomycota;c__Microbotryomycetes;o__Sporidiobolales;f__Sporidiobolaceae;g__Sporidiobolus;s__Sporidiobolus_salmonicolor
SC-1_gene_299	293	ORX93031.1	1869	79/275(28.73)	7.76e-25	hypothetical protein K493DRAFT_316207 [Basidiobolus meristosporus CBS	1314790	d__Eukaryota;k__Fungi;p__Zoopagomycota;c__Basidiobolomycetes;o__Basidiobolales;f__Basidiobolaceae;g__Basidiobolus;s__Basidiobolus_meristosporus
SC-1_gene_318	224	WP_010082269.1	218	211/214(98.60)	2.24e-143	DNA repair protein RadC [Wolbachia endosymbiont of Drosophila simulans]	953	d__Bacteria;k__norank;p__Proteobacteria;c__Alphaproteobacteria;o__Rickettsiales;f__Anaplasmataceae;g__Wolbachia;s__norank
SC-1_gene_354	161	ORY03104.1	698	43/136(31.62)	9.34e-16	Clavaminate synthase-like protein [Basidiobolus meristosporus CBS 931.73]	1314790	d__Eukaryota;k__Fungi;p__Zoopagomycota;c__Basidiobolomycetes;o__Basidiobolales;f__Basidiobolaceae;g__Basidiobolus;s__Basidiobolus_meristosporus
SC-1_gene_424	62	ORX88395.1	606	33/56(58.93)	6.82e-18	FMN-linked oxidoreductase [Basidiobolus meristosporus CBS 931.73]	1314790	d__Eukaryota;k__Fungi;p__Zoopagomycota;c__Basidiobolomycetes;o__Basidiobolales;f__Basidiobolaceae;g__Basidiobolus;s__Basidiobolus_meristosporus
SC-1_gene_436	160	ORY18277.1	1220	40/71(56.34)	4.41e-17	ATP-dependent DNA helicase [Neocallimastix californiae]	1754190	d__Eukaryota;k__Fungi;p__Chytridiomycota;c__Neocallimastigomycetes;o__Neocallimastigales;f__Neocallimastigaceae;g__Neocallimastix;s__Neocallimastix_californiae
SC-1_gene_441	492	RYE22399.1	469	71/268(26.49)	1.37e-26	DUF1759 domain-containing protein [Sphingobacteriaceae	2021370	d__Bacteria;k__norank;p__Bacteroidetes;c__Sphingobacteriia;o__Sphingobacteriales;f__Sphingobacteriaceae;g__norank;s__Sphingobacteriaceae_bacterium
SC-1_gene_460	319	CAQ54569.1	303	292/318(91.82)	5.17e-187	hypothetical protein WP0461 [Wolbachia endosymbiont of Culex quinquefasciatus Pel]	570417	d__Bacteria;k__norank;p__Proteobacteria;c__Alphaproteobacteria;o__Rickettsiales;f__Anaplasmataceae;g__Wolbachia;s__Wolbachia_endosymbiont_of_Culex_quinquefasciatus
SC-1_gene_495	104	OAM05077.1	94	90/94(95.74)	4.82e-59	transposase [Wolbachia endosymbiont of Dactylopius coccus]	1605993	d__Bacteria;k__norank;p__Proteobacteria;c__Alphaproteobacteria;o__Rickettsiales;f__Anaplasmataceae;g__Wolbachia;s__Wolbachia_endosymbiont_of_Dactylopius_coccus
SC-1_gene_551	463	RLJ22759.1	244	53/180(29.44)	1.22e-18	hypothetical protein DJ031_00295 [bacterium endosymbiont of Escarpia	1221601	d__Bacteria;k__norank;p__norank;c__norank;o__norank;f__norank;g__norank;s__bacterium_endosymbiont_of_Escarpia_laminata
SC-1_gene_569	285	OAM06119.1	377	233/284(82.04)	1.16e-145	hypothetical protein TV41_02185 [Wolbachia endosymbiont of Dactylopius coccus]	1605993	d__Bacteria;k__norank;p__Proteobacteria;c__Alphaproteobacteria;o__Rickettsiales;f__Anaplasmataceae;g__Wolbachia;s__Wolbachia_endosymbiont_of_Dactylopius_coccus
SC-1_gene_575	80	WP_098050858.1	154	68/70(97.14)	3.17e-40	hypothetical protein [Pantoea agglomerans]	None	Unclassified
SC-1_gene_614	104	RUS22056.1	459	58/104(55.77)	2.77e-29	actin family [Endogone sp. FLAS-F59071]	2340872	d__Eukaryota;k__Fungi;p__Mucoromycota;c__Endogonomycetes;o__Endogonales;f__Endogonaceae;g__Endogone;s__Endogone_sp._FLAS-F59071
SC-1_gene_631	159	RYE23048.1	795	39/118(33.05)	2.81e-08	DUF4371 domain-containing protein [Sphingobacteriaceae	2021370	d__Bacteria;k__norank;p__Bacteroidetes;c__Sphingobacteriia;o__Sphingobacteriales;f__Sphingobacteriaceae;g__norank;s__Sphingobacteriaceae_bacterium
SC-1_gene_660	55	RXX91571.1	153	24/49(48.98)	1.30e-07	hypothetical protein DD587_31600, partial [Klebsiella	573	d__Bacteria;k__norank;p__Proteobacteria;c__Gammaproteobacteria;o__Enterobacterales;f__Enterobacteriaceae;g__Klebsiella;s__Klebsiella_pneumoniae
SC-1_gene_669	303	WP_110883467.1	655	116/285(40.70)	5.81e-62	beta-galactosidase, partial [Gammaproteobacteria bacterium 2W06]	None	Unclassified
SC-1_gene_683	449	XP_018218916.1	618	205/399(51.38)	4.50e-132	DBP1-like protein [Saccharomyces eubayanus]	1080349	d__Eukaryota;k__Fungi;p__Ascomycota;c__Saccharomycetes;o__Saccharomycetales;f__Saccharomycetaceae;g__Saccharomyces;s__Saccharomyces_eubayanus
SC-1_gene_700	84	WP_077250806.1	79	30/60(50.00)	6.89e-12	hypothetical protein [Escherichia coli]	None	Unclassified
SC-1_gene_730	65	ELP25060.1	631	59/65(90.77)	9.18e-22	Phage tail length tape-measure protein [Pantoea agglomerans	1261128	d__Bacteria;k__norank;p__Proteobacteria;c__Gammaproteobacteria;o__Enterobacterales;f__Erwiniaceae;g__Pantoea;s__Pantoea_agglomerans

SC-1_gene_733	118	ELP25061.1	118	107/118(90.68)	7.22e-74	Phage tail tube protein [Pantoea agglomerans 299R]	1261128	d__Bacteria;k__norank;p__Proteobacteria;c__Gammaproteobacteria;o__Enterobacterales;f__Erwiniaceae;g__Pantoea;s__Pantoea_agglomerans
SC-1_gene_734	120	ELP25062.1	700	106/119(89.08)	3.56e-65	Bacteriophage tail sheath protein [Pantoea agglomerans 299R]	1261128	d__Bacteria;k__norank;p__Proteobacteria;c__Gammaproteobacteria;o__Enterobacterales;f__Erwiniaceae;g__Pantoea;s__Pantoea_agglomerans
SC-1_gene_784	110	WP_092403542.1	870	32/105(30.48)	3.44e-07	hypothetical protein [bacterium 2013Ark19i]	None	Unclassified
SC-1_gene_801	274	WP_111138941.1	259	258/259(99.61)	6.07e-182	ATP-binding cassette domain-containing protein [Pantoea sp. ARC607]	2027922	d__Bacteria;k__norank;p__Proteobacteria;c__Gammaproteobacteria;o__Enterobacterales;f__Erwiniaceae;g__Pantoea;s__Pantoea_sp._ARC607
SC-1_gene_851	208	EXU74076.1	263	195/208(93.75)	1.13e-140	transposase [Erwinia mallotivora]	69222	d__Bacteria;k__norank;p__Proteobacteria;c__Gammaproteobacteria;o__Enterobacterales;f__Erwiniaceae;g__Erwinia;s__Erwinia_mallotivora
SC-1_gene_855	322	WP_086335643.1	439	221/320(69.06)	1.81e-152	IS4 family transposase [Enterococcus mundtii]	53346	d__Bacteria;k__norank;p__Firmicutes;c__Bacilli;o__Lactobacillales;f__Enterococcaceae;g__Enterococcus;s__Enterococcus_mundtii
SC-1_gene_912	73	CRK45103.1	119	38/66(57.58)	1.33e-18	hypothetical protein BN1723_019635, partial	100787	d__Eukaryota;k__Fungi;p__Ascomycota;c__Sordariomycetes;o__Glomerellales;f__Plectosphaerellaceae;g__Verticillium;s__Verticillium_longisporum
SC-1_gene_1005	288	RUA05885.1	740	80/286(27.97)	2.24e-20	hypothetical protein DSY43_03475, partial [Gammaproteobacteria	1913989	d__Bacteria;k__norank;p__Proteobacteria;c__Gammaproteobacteria;o__norank;f__norank;g__norank;s__Gammaproteobacteria_bacterium
SC-1_gene_1034	108	SAL95647.1	1269	44/99(44.44)	6.12e-17	hypothetical protein [Absidia glauca]	4829	d__Eukaryota;k__Fungi;p__Mucoromycota;c__Mucoromycetes;o__Mucorales;f__Cunninghamellaceae;g__Absidia;s__Absidia_glauca
SC-1_gene_1057	97	PCI26675.1	169	34/89(38.20)	1.28e-09	hypothetical protein COB52_05785 [Candidatus Kaiserbacteria bacterium]	2030810	d__Bacteria;k__norank;p__Candidatus_Kaiserbacteria;c__norank;o__norank;f__norank;g__norank;s__Candidatus_Kaiserbacteria_bacterium
SC-1_gene_1105	208	RYE22988.1	1001	79/176(44.89)	2.33e-32	hypothetical protein EOP45_07445	2021370	d__Bacteria;k__norank;p__Bacteroidetes;c__Sphingobacteriia;o__Sphingobacteriales;f__Sphingobacteriaceae;g__norank;s__Sphingobacteriaceae_bacterium
SC-1_gene_1148	125	KDA94880.1	260	100/102(98.04)	2.54e-62	ubiquinone/menaquinone biosynthesis methyltransferase [Pantoea agglomerans Eh318]	1408177	d__Bacteria;k__norank;p__Proteobacteria;c__Gammaproteobacteria;o__Enterobacterales;f__Erwiniaceae;g__Pantoea;s__Pantoea_agglomerans
SC-1_gene_1173	196	WP_041504115.1	128	36/91(39.56)	3.16e-14	fibronectin type III domain-containing protein [Sanguibacteroides justesenii]	1547597	d__Bacteria;k__norank;p__Bacteroidetes;c__Bacteroidia;o__Bacteroidales;f__Porphyromonadaceae;g__Sanguibacteroides;s__Sanguibacteroides_justesenii
SC-1_gene_1369	139	WP_123350197.1	2023	53/108(49.07)	9.57e-21	MULTISPECIES: hypothetical protein [Enterobacter]	2608935	d__Bacteria;k__norank;p__Proteobacteria;c__Gammaproteobacteria;o__Enterobacterales;f__Enterobacteriaceae;g__Enterobacter;s__norank
SC-1_gene_1370	92	WP_123346399.1	297	49/74(66.22)	3.73e-22	MULTISPECIES: DUF4255 domain-containing protein	2608935	d__Bacteria;k__norank;p__Proteobacteria;c__Gammaproteobacteria;o__Enterobacterales;f__Enterobacteriaceae;g__Enterobacter;s__norank
SC-1_gene_1377	236	WP_126690504.1	334	166/233(71.24)	1.04e-113	acyltransferase [Pantoea sp.	None	Unclassified
SC-1_gene_1380	93	OZJ04519.1	282	23/43(53.49)	4.72e-06	hypothetical protein BZG36_02240 [Bifiguratus	1938954	d__Eukaryota;k__Fungi;p__Mucoromycota;c__Endogonomycetes;o__Endogonales;f__norank;g__Bifiguratus;s__Bifiguratus_adelaidae
SC-1_gene_1539	66	CCP04044.1	501	43/66(65.15)	5.03e-17	DL-methionine transporter ATP-binding subunit [Erwinia amylovora Ea644]	1255309	d__Bacteria;k__norank;p__Proteobacteria;c__Gammaproteobacteria;o__Enterobacterales;f__Erwiniaceae;g__Erwinia;s__Erwinia_amylovora
SC-1_gene_1552	247	RYE27181.1	1800	106/243(43.62)	3.20e-59	DUF1759 domain-containing protein [Sphingobacteriaceae	2021370	d__Bacteria;k__norank;p__Bacteroidetes;c__Sphingobacteriia;o__Sphingobacteriales;f__Sphingobacteriaceae;g__norank;s__Sphingobacteriaceae_bacterium
SC-1_gene_1568	283	AGO14404.1	841	117/269(43.49)	6.37e-57	hypothetical protein CsmBAC4b19.2 [Cotesia	452649	d__Viruses;k__norank;p__norank;c__norank;o__norank;f__Polydnaviridae;g__Bracovirus;s__Cotesia_sesamiae_bracovirus
SC-1_gene_1595	108	KHO52515.1	228	39/80(48.75)	1.94e-13	2-oxoglutarate and iron-dependent oxygenase protein [archaeon GW2011_AR17]	1579375	d__Archaea;k__norank;p__norank;c__norank;o__norank;f__norank;g__norank;s__archaeon_GW2011_AR17
SC-1_gene_1602	57	WP_129335269.1	107	30/49(61.22)	5.80e-08	hypothetical protein [Enterobacter cloacae complex sp. 2DZ2F20B]	2511993	d__Bacteria;k__norank;p__Proteobacteria;c__Gammaproteobacteria;o__Enterobacterales;f__Enterobacteriaceae;g__Enterobacter;s__Enterobacter_cloacae_complex_sp._2DZ2F20B

SC-1_gene_1604	252	WP_071289991.1	553	81/248(32.66)	4.99e-35	reverse transcriptase family protein [Acinetobacter	None	Unclassified
SC-1_gene_1650	152	WP_082480182.1	179	54/94(57.45)	1.34e-34	cytochrome P450 [Aeromicrobium sp. Leaf272]	None	Unclassified
SC-1_gene_1700	131	ORM80378.1	119	118/119(99.16)	2.28e-76	dihydroneopterin aldolase [Pantoea deleyi]	470932	d__Bacteria;k__norank;p__Proteobacteria;c__Gammaproteobacteria;o__Enterobacterales;f__Erwiniaceae;g__Pantoea;s__Pantoea_deleyi
SC-1_gene_1748	223	WP_033761763.1	407	194/223(87.00)	9.10e-133	phage major capsid protein [Pantoea agglomerans]	None	Unclassified
SC-1_gene_1788	135	EAL58392.1	570	57/126(45.24)	1.15e-29	SD27140p [Wolbachia endosymbiont of Drosophila	307502	d__Bacteria;k__norank;p__Proteobacteria;c__Alphaproteobacteria;o__Rickettsiales;f__Anaplasmataceae;g__Wolbachia;s__Wolbachia_endosymbiont_of_Drosophila_ananassae
SC-1_gene_1821	195	ELP25062.1	700	144/194(74.23)	7.50e-80	Bacteriophage tail sheath protein [Pantoea agglomerans 299R]	1261128	d__Bacteria;k__norank;p__Proteobacteria;c__Gammaproteobacteria;o__Enterobacterales;f__Erwiniaceae;g__Pantoea;s__Pantoea_agglomerans
SC-1_gene_1822	51	WP_071591682.1	51	48/51(94.12)	2.07e-27	DUF2635 domain-containing protein [Pantoea agglomerans]	None	Unclassified
SC-1_gene_1844	182	WP_111138580.1	182	182/182(100.00)	4.33e-124	CDP-diacylglycerol--glycerol-3-phosphate 3-phosphatidyltransferase [Pantoea sp. ARC607]	2027922	d__Bacteria;k__norank;p__Proteobacteria;c__Gammaproteobacteria;o__Enterobacterales;f__Erwiniaceae;g__Pantoea;s__Pantoea_sp._ARC607
SC-1_gene_1846	211	WP_029637389.1	92	28/60(46.67)	3.34e-09	hypothetical protein [[Scytonema hofmanni] UTEX B 1581]	1161	d__Bacteria;k__norank;p__Cyanobacteria;c__norank;o__Nostocales;f__norank;g__norank;s__norank
SC-1_gene_1880	117	KIX85720.1	601	46/82(56.10)	6.72e-18	GTP-binding protein LepA [candidate division TM6 bacterium JCVI TM6SC1]	1306947	d__Bacteria;k__norank;p__norank;c__norank;o__norank;f__norank;g__Vermiphilus;s__Vermiphilus_pyriformis
SC-1_gene_1890	44	SUB14460.1	335	40/44(90.91)	1.35e-17	D-malate degradation protein R [Pantoea agglomerans]	549	d__Bacteria;k__norank;p__Proteobacteria;c__Gammaproteobacteria;o__Enterobacterales;f__Erwiniaceae;g__Pantoea;s__Pantoea_agglomerans
SC-1_gene_1901	210	PBQ25030.1	387	179/183(97.81)	7.21e-124	phage tail protein, partial [Wolbachia pipientis wAus]	2049495	d__Bacteria;k__norank;p__Proteobacteria;c__Alphaproteobacteria;o__Rickettsiales;f__Anaplasmataceae;g__Wolbachia;s__Wolbachia_pipientis
SC-1_gene_1935	120	ORY48676.1	798	43/78(55.13)	1.40e-24	WD40 repeat-like protein [Rhizoclosmatium globosum]	329046	d__Eukaryota;k__Fungi;p__Chytridiomycota;c__Chytridiomycetes;o__Chytridiales;f__Chytriomycetaceae;g__Rhizoclosmatium;s__Rhizoclosmatium_globosum
SC-1_gene_1936	269	AGJ87894.1	447	217/260(83.46)	2.28e-150	type I restriction-modification system, specificity (S) subunit [Raoultella ornithinolytica B6]	1286170	d__Bacteria;k__norank;p__Proteobacteria;c__Gammaproteobacteria;o__Enterobacterales;f__Enterobacteriaceae;g__Raoultella;s__Raoultella_ornithinolytica
SC-1_gene_1942	103	WP_019236392.1	263	96/103(93.20)	4.24e-55	baseplate assembly protein J [Wolbachia pipientis]	955	d__Bacteria;k__norank;p__Proteobacteria;c__Alphaproteobacteria;o__Rickettsiales;f__Anaplasmataceae;g__Wolbachia;s__Wolbachia_pipientis
SC-1_gene_1949	86	WP_080190720.1	281	46/83(55.42)	9.92e-20	hypothetical protein [Salmonella enterica]	28901	d__Bacteria;k__norank;p__Proteobacteria;c__Gammaproteobacteria;o__Enterobacterales;f__Enterobacteriaceae;g__Salmonella;s__Salmonella_enterica
SC-1_gene_1955	113	GBE57033.1	143	60/111(54.05)	9.46e-22	hypothetical protein BMS3Bbin16_01248 [archaeon BMS3Bbin16]	2005739	d__Archaea;k__norank;p__norank;c__norank;o__norank;f__norank;g__norank;s__archaeon_BMS3Bbin16
SC-1_gene_1969	68	ARE12998.1	380	68/68(100.00)	1.93e-38	Glucose-1-phosphate adenylyltransferase catalytic subunit [Lactococcus lactis subsp.	1360	d__Bacteria;k__norank;p__Firmicutes;c__Bacilli;o__Lactobacillales;f__Streptococcaceae;g__Lactococcus;s__Lactococcus_lactis
SC-1_gene_1998	254	RKP09022.1	706	91/205(44.39)	1.57e-43	Noc2p family-domain-containing protein [Thamnocephalis sphaerospora]	78915	d__Eukaryota;k__Fungi;p__Zoopagomycota;c__Zoopagomycetes;o__Zoopagales;f__Sigmoideomycetaceae;g__Thamnocephalis;s__Thamnocephalis_sphaerospora
SC-1_gene_2047	193	WP_099707593.1	273	96/191(50.26)	1.31e-61	MULTISPECIES: hypothetical protein [Erwinia]	2622719	d__Bacteria;k__norank;p__Proteobacteria;c__Gammaproteobacteria;o__Enterobacterales;f__Erwiniaceae;g__Erwinia;s__norank
SC-1_gene_2063	112	WP_117281880.1	52	33/48(68.75)	1.44e-13	hypothetical protein [Pasteurella multocida]	747	d__Bacteria;k__norank;p__Proteobacteria;c__Gammaproteobacteria;o__Pasteurellales;f__Pasteurellaceae;g__Pasteurella;s__Pasteurella_multocida
SC-1_gene_2070	49	PWA03432.1	400	25/32(78.12)	2.01e-07	hypothetical protein BB558_000402 [Smittium	133377	d__Eukaryota;k__Fungi;p__Zoopagomycota;c__Harpellomycetes;o__Harpellales;f__Legeriomycetaceae;g__Smittium;s__Smittium_angustum

SC-1_gene_2112	147	WP_126690642.1	206	73/143(51.05)	6.70e-34	glycoside hydrolase family 19 protein [Pantoea sp. YU22]	None	Unclassified
SC-1_gene_2127	65	WP_120787132.1	492	55/64(85.94)	2.04e-29	aldehyde dehydrogenase family protein [Macrococcus	None	Unclassified
SC-1_gene_2139	101	RUP49600.1	192	46/93(49.46)	7.98e-27	histidine phosphatase superfamily [Jimgerdemannia flammicorona]	994334	d__Eukaryota;k__Fungi;p__Mucoromycota;c__Endogonomycetes;o__Endogonales;f__Endogonaceae;g__Jimgerdemannia;s__Jimgerdemannia_flammicorona
SC-1_gene_2150	119	ORY53105.1	322	40/109(36.70)	1.18e-15	WD40 repeat-like protein [Rhizoclosmatium globosum]	329046	d__Eukaryota;k__Fungi;p__Chytridiomycota;c__Chytridiomycetes;o__Chytridiales;f__Chytriomycetaceae;g__Rhizoclosmatium;s__Rhizoclosmatium_globosum
SC-1_gene_2169	109	RUM29380.1	438	42/91(46.15)	2.59e-17	hypothetical protein DSY42_06665 [Aquifex sp.]	313370	d__Bacteria;k__norank;p__Aquificae;c__Aquificae;o__Aquificales;f__Aquificaceae;g__Aquifex;s__Aquifex_sp.
SC-1_gene_2220	111	RUP48613.1	307	80/107(74.77)	3.09e-42	hypothetical protein BC936DRAFT_144297 [Jimgerdemannia flammicorona]	994334	d__Eukaryota;k__Fungi;p__Mucoromycota;c__Endogonomycetes;o__Endogonales;f__Endogonaceae;g__Jimgerdemannia;s__Jimgerdemannia_flammicorona
SC-1_gene_2245	211	WP_058958481.1	1473	189/195(96.92)	1.08e-131	type IV secretion protein Rhs [Type-F symbiont of Plautia stali]	1560358	d__Bacteria;k__norank;p__Proteobacteria;c__Gammaproteobacteria;o__Enterobacterales;f__Enterobacteriaceae;g__norank;s__Type-F_symbiont_of_Plautia_stali
SC-1_gene_2272	106	ORM82466.1	99	93/99(93.94)	3.98e-55	cytoplasmic protein [Pantoea deleyi]	470932	d__Bacteria;k__norank;p__Proteobacteria;c__Gammaproteobacteria;o__Enterobacterales;f__Erwiniaceae;g__Pantoea;s__Pantoea_deleyi
SC-1_gene_2274	170	ORM76682.1	365	134/136(98.53)	1.63e-81	diguanylate cyclase [Pantoea deleyi]	470932	d__Bacteria;k__norank;p__Proteobacteria;c__Gammaproteobacteria;o__Enterobacterales;f__Erwiniaceae;g__Pantoea;s__Pantoea_deleyi
SC-1_gene_2311	172	SKA25796.1	165	165/165(100.00)	2.04e-102	LSU ribosomal protein L10P [Pantoea agglomerans]	470933	d__Bacteria;k__norank;p__Proteobacteria;c__Gammaproteobacteria;o__Enterobacterales;f__Erwiniaceae;g__Pantoea;s__Pantoea_eucalypti
SC-1_gene_2324	189	SJZ72962.1	214	171/188(90.96)	3.52e-114	hypothetical protein SAMN03097723_1939 [Pantoea agglomerans]	470933	d__Bacteria;k__norank;p__Proteobacteria;c__Gammaproteobacteria;o__Enterobacterales;f__Erwiniaceae;g__Pantoea;s__Pantoea_eucalypti
SC-1_gene_2332	59	WP_073563042.1	250	30/51(58.82)	3.88e-15	hypothetical protein [Archangium sp. Cb G35]	1920190	d__Bacteria;k__norank;p__Proteobacteria;c__Deltaproteobacteria;o__Myxococcales;f__Archangiaceae;g__Archangium;s__Archangium_sp._Cb_G35
SC-1_gene_2409	98	WP_128083964.1	85	74/82(90.24)	7.62e-46	prepilin-type cleavage/methylation domain-containing protein [Pantoea	None	Unclassified
SC-1_gene_2448	233	YP_009337091.1	396	71/237(29.96)	1.81e-21	hypothetical protein [Hubei chuvirus-like virus 3]	1922858	d__Viruses;k__Orthornavirae;p__Negarnaviricota;c__Monjiviricetes;o__Jingchuvirales;f__Chuviridae;g__Mivirus;s__Hubei_odonate_mivirus
SC-1_gene_2467	150	WP_010402967.1	408	149/150(99.33)	2.83e-101	MULTISPECIES: cytochrome b/b6 [Wolbachia]	953	d__Bacteria;k__norank;p__Proteobacteria;c__Alphaproteobacteria;o__Rickettsiales;f__Anaplasmataceae;g__Wolbachia;s__norank
SC-1_gene_2506	58	WP_033761767.1	575	58/58(100.00)	7.43e-31	terminase large subunit [Pantoea agglomerans]	None	Unclassified
SC-1_gene_2509	51	ORX52731.1	288	28/47(59.57)	2.05e-08	adenylate kinase [Hesseltinella vesiculosa]	101127	d__Eukaryota;k__Fungi;p__Mucoromycota;c__Mucoromycetes;o__Mucorales;f__Cunninghamellaceae;g__Hesseltinella;s__Hesseltinella_vesiculosa
SC-1_gene_2516	108	KNE70186.1	525	41/110(37.27)	1.13e-14	hypothetical protein AMAG_15154 [Allomyces macrogynus ATCC 38327]	578462	d__Eukaryota;k__Fungi;p__Blastocladiomycota;c__Blastocladiomycetes;o__Blastocladiales;f__Blastocladiaceae;g__Allomyces;s__Allomyces_macrogynus
SC-1_gene_2559	225	ADO10014.1	463	219/225(97.33)	1.40e-140	Putative MFS family transporter [Pantoea vagans C9-1]	712898	d__Bacteria;k__norank;p__Proteobacteria;c__Gammaproteobacteria;o__Enterobacterales;f__Erwiniaceae;g__Pantoea;s__Pantoea_vagans
SC-1_gene_2573	98	RUM29494.1	235	29/66(43.94)	8.66e-11	hypothetical protein DSY42_06410 [Aquifex sp.]	313370	d__Bacteria;k__norank;p__Aquificae;c__Aquificae;o__Aquificales;f__Aquificaceae;g__Aquifex;s__Aquifex_sp.
SC-1_gene_2635	81	KXS22186.1	246	47/75(62.67)	4.44e-24	hypothetical protein M427DRAFT_92660, partial [Gonapodya prolifera JEL478]	1344416	d__Eukaryota;k__Fungi;p__Chytridiomycota;c__Monoblepharidomycetes;o__Monoblepharidales;f__Gonapodyaceae;g__Gonapodya;s__Gonapodya_prolifera
SC-1_gene_2636	83	RMJ22449.1	771	33/59(55.93)	1.23e-12	ubiquitin carboxyl-terminal hydrolase, partial [Phialosimplex sp. HF37]	1960876	d__Eukaryota;k__Fungi;p__Ascomycota;c__Eurotiomycetes;o__Eurotiales;f__Aspergillaceae;g__Aspergillus;s__Aspergillus_sp._HF37

SC-1_gene_2726	61	WP_123346431.1	149	59/61(96.72)	4.63e-31	MULTISPECIES: phage tail protein [Enterobacter]	2608935	d__Bacteria;k__norank;p__Proteobacteria;c__Gammaproteobacteria;o__Enterobacterales;f__Enterobacteriaceae;g__Enterobacter;s__norank
SC-1_gene_2728	44	AUO01785.1	149	38/44(86.36)	3.39e-18	phage tail protein [Serratia marcescens]	615	d__Bacteria;k__norank;p__Proteobacteria;c__Gammaproteobacteria;o__Enterobacterales;f__Yersiniaceae;g__Serratia;s__Serratia_marcescens
SC-1_gene_2747	211	WP_104141951.1	866	208/211(98.58)	5.54e-141	CHAP domain-containing protein [Lactococcus lactis]	None	Unclassified
SC-1_gene_2752	109	WP_083198535.1	323	89/114(78.07)	4.72e-50	MULTISPECIES: autotransporter outer membrane beta-barrel domain-containing protein [unclassified Rickettsia]	114295	d__Bacteria;k__norank;p__Proteobacteria;c__Alphaproteobacteria;o__Rickettsiales;f__Rickettsiaceae;g__Rickettsia;s__norank
SC-1_gene_2827	52	ETE43231.1	289	32/47(68.09)	2.34e-16	hypothetical protein M574_21520, partial [Salmonella enterica subsp. enterica serovar	1347789	d__Bacteria;k__norank;p__Proteobacteria;c__Gammaproteobacteria;o__Enterobacterales;f__Enterobacteriaceae;g__Salmonella;s__Salmonella_enterica
SC-1_gene_2835	104	ORM81412.1	101	99/101(98.02)	4.74e-65	hypothetical protein HA42_10780 [Pantoea deleyi]	470932	d__Bacteria;k__norank;p__Proteobacteria;c__Gammaproteobacteria;o__Enterobacterales;f__Erwiniaceae;g__Pantoea;s__Pantoea_deleyi
SC-1_gene_2890	201	RIB19600.1	1030	43/162(26.54)	8.49e-08	hypothetical protein C2G38_1254418 [Gigaspora	44941	d__Eukaryota;k__Fungi;p__Mucoromycota;c__Glomeromycetes;o__Diversisporales;f__Gigasporaceae;g__Gigaspora;s__Gigaspora_rosea
SC-1_gene_2897	201	OMJ15008.1	1073	52/174(29.89)	4.35e-06	Retrovirus-related Pol polyprotein from transposon [Smittium	133412	d__Eukaryota;k__Fungi;p__Zoopagomycota;c__Harpellomycetes;o__Harpellales;f__Legeriomycetaceae;g__Smittium;s__Smittium_culicis
SC-2_gene_37	39	EAL59029.1	40	29/36(80.56)	2.58e-12	transposase, IS5 family, OrfA [Wolbachia endosymbiont of Drosophila ananassae]	307502	d__Bacteria;k__norank;p__Proteobacteria;c__Alphaproteobacteria;o__Rickettsiales;f__Anaplasmataceae;g__Wolbachia;s__Wolbachia_endosymbiont_of_Drosophila_ananassae
SC-2_gene_64	120	WP_019236401.1	345	103/110(93.64)	9.73e-69	phage portal protein [Wolbachia pipientis]	955	d__Bacteria;k__norank;p__Proteobacteria;c__Alphaproteobacteria;o__Rickettsiales;f__Anaplasmataceae;g__Wolbachia;s__Wolbachia_pipientis
SC-2_gene_88	397	PBQ29078.1	475	365/395(92.41)	5.73e-239	two-component sensor histidine kinase [Wolbachia pipientis	2049495	d__Bacteria;k__norank;p__Proteobacteria;c__Alphaproteobacteria;o__Rickettsiales;f__Anaplasmataceae;g__Wolbachia;s__Wolbachia_pipientis
SC-2_gene_107	371	WP_039120656.1	1331	109/257(42.41)	1.73e-49	hypothetical protein [Flavobacterium sp. JRM]	None	Unclassified
SC-2_gene_164	230	WP_108784684.1	243	216/230(93.91)	3.17e-155	protein-disulfide isomerase [Wolbachia endosymbiont of Bemisia tabaci]	None	Unclassified
SC-2_gene_170	367	WP_127463694.1	878	352/367(95.91)	5.39e-235	ankyrin repeat domain-containing protein [Wolbachia endosymbiont of Bemisia tabaci]	215173	d__Bacteria;k__norank;p__Proteobacteria;c__Alphaproteobacteria;o__Rickettsiales;f__Anaplasmataceae;g__Wolbachia;s__Wolbachia_endosymbiont_of_Bemisia_tabaci
SC-2_gene_216	739	OAQ23356.1	1034	151/650(23.23)	1.59e-25	DUF221-domain-containing protein [Mortierella elongata AG-	1314771	d__Eukaryota;k__Fungi;p__Mucoromycota;c__Mortierellomycetes;o__Mortierellales;f__Mortierellaceae;g__Linnemannia;s__Linnemannia_elongata
SC-2_gene_227	102	RIB11219.1	1468	48/100(48.00)	1.03e-17	SNF2 family N-terminal domain-containing protein [Gigaspora	44941	d__Eukaryota;k__Fungi;p__Mucoromycota;c__Glomeromycetes;o__Diversisporales;f__Gigasporaceae;g__Gigaspora;s__Gigaspora_rosea
SC-2_gene_270	79	WP_051063872.1	102	67/67(100.00)	2.77e-44	hypothetical protein [Wolbachia endosymbiont of Drosophila simulans]	None	Unclassified
SC-2_gene_277	586	RUM44709.1	197	62/193(32.12)	7.71e-15	hypothetical protein DSY80_04100 [Desulfocapsa	2066021	d__Bacteria;k__norank;p__Proteobacteria;c__Deltaproteobacteria;o__Desulfobacterales;f__Desulfobulbaceae;g__Desulfocapsa;s__Desulfocapsa_sp.
SC-2_gene_281	299	EAL58605.1	1033	103/298(34.56)	5.86e-48	Zinc knuckle domain protein [Wolbachia endosymbiont of Drosophila ananassae]	307502	d__Bacteria;k__norank;p__Proteobacteria;c__Alphaproteobacteria;o__Rickettsiales;f__Anaplasmataceae;g__Wolbachia;s__Wolbachia_endosymbiont_of_Drosophila_ananassae
SC-2_gene_309	104	WP_010082361.1	98	93/98(94.90)	8.71e-65	MULTISPECIES: hypothetical protein [Wolbachia]	77038	d__Bacteria;k__norank;p__Proteobacteria;c__Alphaproteobacteria;o__Rickettsiales;f__Anaplasmataceae;g__Wolbachia;s__Wolbachia_endosymbiont_of_Drosophila_simulans
SC-2_gene_334	92	WP_114598933.1	598	25/40(62.50)	1.45e-08	hypothetical protein [Microbacterium arborescens]	33883	d__Bacteria;k__norank;p__Actinobacteria;c__Actinobacteria;o__Micrococcales;f__Microbacteriaceae;g__Microbacterium;s__Microbacterium_arborescens

SC-2_gene_364	72	PBQ27665.1	170	72/72(100.00)	8.00e-37	hypothetical protein BTO27_03145 [Wolbachia	2049495	d__Bacteria;k__norank;p__Proteobacteria;c__Alphaproteobacteria;o__Rickettsiales;f__Anaplasmataceae;g__Wolbachia;s__Wolbachia_pipientis
SC-2_gene_375	208	PBQ26131.1	208	207/208(99.52)	7.54e-140	phosphatidate cytidylyltransferase [Wolbachia pipientis wAus]	2049495	d__Bacteria;k__norank;p__Proteobacteria;c__Alphaproteobacteria;o__Rickettsiales;f__Anaplasmataceae;g__Wolbachia;s__Wolbachia_pipientis
SC-2_gene_402	365	WP_010401258.1	363	362/363(99.72)	2.78e-245	peptide chain release factor 1 [Wolbachia endosymbiont of Nasonia vitripennis]	180837	d__Bacteria;k__norank;p__Proteobacteria;c__Alphaproteobacteria;o__Rickettsiales;f__Anaplasmataceae;g__Wolbachia;s__Wolbachia_endosymbiont_of_Nasonia_vitripennis
SC-2_gene_458	103	WP_114517332.1	304	101/103(98.06)	3.57e-65	phage portal protein, partial [Wolbachia endosymbiont of Cylisticus convexus]	118728	d__Bacteria;k__norank;p__Proteobacteria;c__Alphaproteobacteria;o__Rickettsiales;f__Anaplasmataceae;g__Wolbachia;s__Wolbachia_endosymbiont_of_Cylisticus_convexus
SC-2_gene_493	117	PBQ28110.1	117	117/117(100.00)	5.10e-78	hypothetical protein BTO27_02675 [Wolbachia	2049495	d__Bacteria;k__norank;p__Proteobacteria;c__Alphaproteobacteria;o__Rickettsiales;f__Anaplasmataceae;g__Wolbachia;s__Wolbachia_pipientis
SC-2_gene_500	589	ORY04742.1	683	205/597(34.34)	3.33e-103	Sec1-like protein [Basidiobolus meristosporus CBS 931.73]	1314790	d__Eukaryota;k__Fungi;p__Zoopagomycota;c__Basidiobolomycetes;o__Basidiobolales;f__Basidiobolaceae;g__Basidiobolus;s__Basidiobolus_meristosporus
SC-2_gene_516	284	RLJ22797.1	772	92/280(32.86)	5.03e-25	hypothetical protein DJ031_00255, partial [bacterium endosymbiont of Escarpia	1221601	d__Bacteria;k__norank;p__norank;c__norank;o__norank;f__norank;g__norank;s__bacterium_endosymbiont_of_Escarpia_laminata
SC-2_gene_569	217	CAQ54184.1	217	213/217(98.16)	4.12e-137	exopolysaccharide synthesis protein ExoD-like [Wolbachia endosymbiont of Culex	570417	d__Bacteria;k__norank;p__Proteobacteria;c__Alphaproteobacteria;o__Rickettsiales;f__Anaplasmataceae;g__Wolbachia;s__Wolbachia_endosymbiont_of_Culex_quinquefasciatus
SC-2_gene_612	111	AGJ99034.1	100	100/100(100.00)	1.56e-58	NADH-ubiquinone oxidoreductase chain 4L [Wolbachia endosymbiont of	1236908	d__Bacteria;k__norank;p__Proteobacteria;c__Alphaproteobacteria;o__Rickettsiales;f__Anaplasmataceae;g__Wolbachia;s__Wolbachia_endosymbiont_of_Drosophila_simulans
SC-2_gene_709	90	KNE68187.1	373	32/54(59.26)	2.08e-14	hypothetical protein AMAG_12868 [Allomyces macrogynus ATCC 38327]	578462	d__Eukaryota;k__Fungi;p__Blastocladiomycota;c__Blastocladiomycetes;o__Blastocladiales;f__Blastocladiaceae;g__Allomyces;s__Allomyces_macrogynus
SC-2_gene_719	456	WP_064085731.1	456	451/456(98.90)	1.62e-279	magnesium transporter [Wolbachia endosymbiont of Dactylopius coccus]	None	Unclassified
SC-2_gene_732	66	WP_068042328.1	244	48/52(92.31)	1.63e-24	MULTISPECIES: bifunctional (p)ppGpp synthetase/guanosine-3',5'-bis(diphosphate) 3'-pyrophosphohydrolase	114295	d__Bacteria;k__norank;p__Proteobacteria;c__Alphaproteobacteria;o__Rickettsiales;f__Rickettsiaceae;g__Rickettsia;s__norank
SC-2_gene_804	185	RLJ22864.1	226	49/175(28.00)	4.76e-15	hypothetical protein DJ031_00085 [bacterium endosymbiont of Escarpia	1221601	d__Bacteria;k__norank;p__norank;c__norank;o__norank;f__norank;g__norank;s__bacterium_endosymbiont_of_Escarpia_laminata
SC-2_gene_807	168	Q73GH4.1	169	160/167(95.81)	1.29e-98	RecName: Full=NADH-quinone oxidoreductase subunit I; AltName: Full=NADH dehydrogenase I subunit I; AltName: Full=NDH-1 subunit I	163164	d__Bacteria;k__norank;p__Proteobacteria;c__Alphaproteobacteria;o__Rickettsiales;f__Anaplasmataceae;g__Wolbachia;s__Wolbachia_endosymbiont_of_Drosophila_melanogaster
SC-2_gene_828	160	AGO14404.1	841	43/123(34.96)	1.53e-12	hypothetical protein CsmBAC4b19.2 [Cotesia	452649	d__Viruses;k__norank;p__norank;c__norank;o__norank;f__Polydnaviridae;g__Bracovirus;s__Cotesia_sesamiae_bracovirus
SC-2_gene_829	578	AGO14404.1	841	62/154(40.26)	2.74e-14	hypothetical protein CsmBAC4b19.2 [Cotesia	452649	d__Viruses;k__norank;p__norank;c__norank;o__norank;f__Polydnaviridae;g__Bracovirus;s__Cotesia_sesamiae_bracovirus
SC-2_gene_865	75	KXN87716.1	1396	36/79(45.57)	1.01e-13	ATP-dependent DNA helicase hus2/rqh1 [Leucoagaricus sp. SymC.cos]	1714833	d__Eukaryota;k__Fungi;p__Basidiomycota;c__Agaricomycetes;o__Agaricales;f__Agaricaceae;g__Leucoagaricus;s__Leucoagaricus_sp._SymC.cos

SC-2_gene_881	204	WP_010404216.1	915	198/206(96.12)	1.73e-124	hypothetical protein, partial [Wolbachia endosymbiont of Nasonia vitripennis]	180837	d__Bacteria;k__norank;p__Proteobacteria;c__Alphaproteobacteria;o__Rickettsiales;f__Anaplasmataceae;g__Wolbachia;s__Wolbachia_endosymbiont_of_Nasonia_vitripennis
SC-2_gene_943	457	PBQ30180.1	487	457/457(100.00)	8.62e-316	protease Do [Wolbachia pipientis wAus]	2049495	d__Bacteria;k__norank;p__Proteobacteria;c__Alphaproteobacteria;o__Rickettsiales;f__Anaplasmataceae;g__Wolbachia;s__Wolbachia_pipientis
SC-2_gene_984	274	WP_063630881.1	274	274/274(100.00)	7.67e-209	oxygen-dependent coproporphyrinogen oxidase [Wolbachia endosymbiont of Laodelphax striatella]	2640676	d__Bacteria;k__norank;p__Proteobacteria;c__Alphaproteobacteria;o__Rickettsiales;f__Anaplasmataceae;g__Wolbachia;s__norank
SC-2_gene_1027	598	AYV77951.1	687	177/458(38.65)	8.53e-95	DEAD/SNF2-like helicase [Edafosvirus sp.]	2487765	d__Viruses;k__Bamfordvirae;p__Nucleocytoviricota;c__Megaviricetes;o__Imitervirales;f__Mimiviridae;g__norank;s__Edafosvirus_sp.
SC-2_gene_1075	639	AGJ98551.1	2380	126/162(77.78)	3.19e-70	Ankyrin repeat domain protein [Wolbachia endosymbiont of Drosophila simulans wNo]	1236908	d__Bacteria;k__norank;p__Proteobacteria;c__Alphaproteobacteria;o__Rickettsiales;f__Anaplasmataceae;g__Wolbachia;s__Wolbachia_endosymbiont_of_Drosophila_simulans
SC-2_gene_1078	343	WP_063631091.1	343	338/343(98.54)	2.68e-246	DUF1016 domain-containing protein [Wolbachia endosymbiont of Laodelphax striatella]	2640676	d__Bacteria;k__norank;p__Proteobacteria;c__Alphaproteobacteria;o__Rickettsiales;f__Anaplasmataceae;g__Wolbachia;s__norank
SC-2_gene_1143	79	ONI57892.1	140	71/79(89.87)	4.90e-41	phage baseplate assembly protein V family protein [Wolbachia pipientis wVitA]	1379786	d__Bacteria;k__norank;p__Proteobacteria;c__Alphaproteobacteria;o__Rickettsiales;f__Anaplasmataceae;g__Wolbachia;s__Wolbachia_pipientis
SC-2_gene_1208	152	RUM44711.1	113	24/48(50.00)	4.59e-08	hypothetical protein DSY80_04110 [Desulfocapsa	2066021	d__Bacteria;k__norank;p__Proteobacteria;c__Deltaproteobacteria;o__Desulfobacterales;f__Desulfobulbaceae;g__Desulfocapsa;s__Desulfocapsa_sp.
SC-2_gene_1254	54	RDD33885.1	42	23/34(67.65)	6.48e-06	hypothetical protein Wcon_02086 [Wolbachia endosymbiont of Cylisticus convexus]	118728	d__Bacteria;k__norank;p__Proteobacteria;c__Alphaproteobacteria;o__Rickettsiales;f__Anaplasmataceae;g__Wolbachia;s__Wolbachia_endosymbiont_of_Cylisticus_convexus
SC-2_gene_1286	198	WP_063630634.1	391	182/198(91.92)	9.62e-122	hypothetical protein [Wolbachia endosymbiont of Laodelphax striatella]	368602	d__Bacteria;k__norank;p__Proteobacteria;c__Alphaproteobacteria;o__Rickettsiales;f__Anaplasmataceae;g__Wolbachia;s__Wolbachia_endosymbiont_of_Laodelphax_striatellus
SC-2_gene_1314	264	WP_064085973.1	259	238/259(91.89)	1.46e-159	baseplate assembly protein J, partial [Wolbachia endosymbiont of Dactylopius coccus]	None	Unclassified
SC-2_gene_1325	511	PBQ26533.1	2386	128/161(79.50)	9.77e-73	hypothetical protein BTO27_04595, partial	2049495	d__Bacteria;k__norank;p__Proteobacteria;c__Alphaproteobacteria;o__Rickettsiales;f__Anaplasmataceae;g__Wolbachia;s__Wolbachia_pipientis
SC-2_gene_1343	59	AIA93542.1	102	34/43(79.07)	1.43e-20	CAZy families GH27 protein, partial [uncultured bacterium]	77133	d__Bacteria;k__norank;p__norank;c__norank;o__norank;f__norank;g__norank;s__uncultured_bacterium
SC-2_gene_1362	200	PCG11384.1	186	184/186(98.92)	5.98e-123	nucleotide exchange factor GrpE [Wolbachia pipientis]	None	Unclassified
SC-2_gene_1374	536	WP_131019729.1	851	265/528(50.19)	1.90e-185	ATPase, partial [Obesumbacterium proteus]	82983	d__Bacteria;k__norank;p__Proteobacteria;c__Gammaproteobacteria;o__Enterobacterales;f__Hafniaceae;g__Obesumbacterium;s__Obesumbacterium_proteus
SC-2_gene_1499	145	PCG11131.1	269	145/145(100.00)	2.18e-78	metal ABC transporter permease [Wolbachia pipientis]	None	Unclassified
SC-2_gene_1641	266	RUM45879.1	275	119/266(44.74)	2.80e-73	hypothetical protein DSY80_02865 [Desulfocapsa	2066021	d__Bacteria;k__norank;p__Proteobacteria;c__Deltaproteobacteria;o__Desulfobacterales;f__Desulfobulbaceae;g__Desulfocapsa;s__Desulfocapsa_sp.
SC-2_gene_1666	233	PKK73235.1	172	35/96(36.46)	3.77e-08	hypothetical protein RhiirC2_709772, partial	588596	d__Eukaryota;k__Fungi;p__Mucoromycota;c__Glomeromycetes;o__Glomerales;f__Glomeraceae;g__Rhizophagus;s__Rhizophagus_irregularis
SC-2_gene_1669	90	WP_127785998.1	87	38/87(43.68)	1.03e-16	hypothetical protein [Citrobacter freundii]	546	d__Bacteria;k__norank;p__Proteobacteria;c__Gammaproteobacteria;o__Enterobacterales;f__Enterobacteriaceae;g__Citrobacter;s__Citrobacter_freundii
SC-2_gene_1711	267	RUM45879.1	275	114/269(42.38)	8.77e-59	hypothetical protein DSY80_02865 [Desulfocapsa	2066021	d__Bacteria;k__norank;p__Proteobacteria;c__Deltaproteobacteria;o__Desulfobacterales;f__Desulfobulbaceae;g__Desulfocapsa;s__Desulfocapsa_sp.

SC-2_gene_1729	64	WP_068042242.1	163	42/63(66.67)	3.77e-21	MULTISPECIES: hypothetical protein [unclassified Rickettsia]	114295	d__Bacteria;k__norank;p__Proteobacteria;c__Alphaproteobacteria;o__Rickettsiales;f__Rickettsiaceae;g__Rickettsia;s__norank
SC-2_gene_1740	284	WP_114598937.1	353	209/273(76.56)	3.17e-137	J domain-containing protein [Microbacterium arborescens]	33883	d__Bacteria;k__norank;p__Actinobacteria;c__Actinobacteria;o__Micrococcales;f__Microbacteriaceae;g__Microbacterium;s__Microbacterium_arborescens
SC-2_gene_1747	162	CQD11744.1	167	117/165(70.91)	2.90e-70	Ankyrin repeat domain protein [Wolbachia endosymbiont wPip_Mol of Culex molestus]	1402163	d__Bacteria;k__norank;p__Proteobacteria;c__Alphaproteobacteria;o__Rickettsiales;f__Anaplasmataceae;g__Wolbachia;s__Wolbachia_endosymbiont_of_Culex_molestus
SC-2_gene_1761	60	RHZ46530.1	112	29/59(49.15)	3.98e-10	hypothetical protein Glove_615g15 [Diversispora	1348612	d__Eukaryota;k__Fungi;p__Mucoromycota;c__Glomeromycetes;o__Diversisporales;f__Diversisporaceae;g__Diversispora;s__Diversispora_epigaea
SC-2_gene_1790	225	YP_009342457.1	380	78/212(36.79)	2.47e-24	hypothetical protein [Wuhan insect virus 15]	1923719	d__Viruses;k__norank;p__norank;c__norank;o__norank;f__norank;g__norank;s__Wuhan_insect_virus_15
SC-2_gene_1806	250	WP_010082270.1	418	232/250(92.80)	1.40e-151	hypothetical protein [Wolbachia endosymbiont of Drosophila simulans]	953	d__Bacteria;k__norank;p__Proteobacteria;c__Alphaproteobacteria;o__Rickettsiales;f__Anaplasmataceae;g__Wolbachia;s__norank
SC-2_gene_1807	166	PCG11506.1	328	166/166(100.00)	8.03e-112	P-type DNA transfer ATPase VirB11 [Wolbachia pipientis]	None	Unclassified
SC-2_gene_1817	190	XP_013239454.1	419	64/161(39.75)	1.36e-23	O-acyltransferase [Mitosporidium daphniae]	1485682	d__Eukaryota;k__Fungi;p__Microsporidia;c__norank;o__norank;f__norank;g__Mitosporidium;s__Mitosporidium_daphniae
SC-2_gene_1833	346	OEU83495.1	395	52/218(23.85)	3.53e-06	hypothetical protein BA873_07100 [Desulfobulbaceae bacterium C00003063]	1869307	d__Bacteria;k__norank;p__Proteobacteria;c__Deltaproteobacteria;o__Desulfobacterales;f__Desulfobulbaceae;g__norank;s__Desulfobulbaceae_bacterium_C00003063
SC-2_gene_1862	215	RUM28374.1	405	69/166(41.57)	4.20e-20	hypothetical protein DSY42_08515 [Aquifex sp.]	313370	d__Bacteria;k__norank;p__Aquificae;c__Aquificae;o__Aquificales;f__Aquificaceae;g__Aquifex;s__Aquifex_sp.
SC-2_gene_1888	175	WP_026092668.1	214	156/175(89.14)	1.37e-105	hypothetical protein [Wolbachia endosymbiont of Diaphorina citri]	116598	d__Bacteria;k__norank;p__Proteobacteria;c__Alphaproteobacteria;o__Rickettsiales;f__Anaplasmataceae;g__Wolbachia;s__Wolbachia_endosymbiont_of_Diaphorina_citri
SC-2_gene_1903	70	GAV99509.1	1201	30/71(42.25)	1.75e-06	coatomer subunit alpha-2 [Lentinula edodes]	5353	d__Eukaryota;k__Fungi;p__Basidiomycota;c__Agaricomycetes;o__Agaricales;f__Omphalotaceae;g__Lentinula;s__Lentinula_edodes
SC-2_gene_1918	423	WP_114517387.1	94	29/68(42.65)	1.27e-07	hypothetical protein [Wolbachia endosymbiont of Cylisticus convexus]	118728	d__Bacteria;k__norank;p__Proteobacteria;c__Alphaproteobacteria;o__Rickettsiales;f__Anaplasmataceae;g__Wolbachia;s__Wolbachia_endosymbiont_of_Cylisticus_convexus
SC-2_gene_1941	110	KXS22186.1	246	51/104(49.04)	3.78e-19	hypothetical protein M427DRAFT_92660, partial [Gonapodya prolifera JEL478]	1344416	d__Eukaryota;k__Fungi;p__Chytridiomycota;c__Monoblepharidomycetes;o__Monoblepharidales;f__Gonapodyaceae;g__Gonapodya;s__Gonapodya_prolifera
SC-2_gene_1953	180	PBD15389.1	175	170/175(97.14)	9.52e-110	biotin transporter BioY [Wolbachia endosymbiont of Drosophila subpulchrella]	2033655	d__Bacteria;k__norank;p__Proteobacteria;c__Alphaproteobacteria;o__Rickettsiales;f__Anaplasmataceae;g__Wolbachia;s__Wolbachia_endosymbiont_of_Drosophila_subpulchrella
SC-2_gene_1954	296	EXX75326.1	1066	161/279(57.71)	1.56e-99	Cop1p [Rhizophagus irregularis DAOM 197198w]	1432141	d__Eukaryota;k__Fungi;p__Mucoromycota;c__Glomeromycetes;o__Glomerales;f__Glomeraceae;g__Rhizophagus;s__Rhizophagus_irregularis
SC-2_gene_1987	301	PCG11648.1	441	291/301(96.68)	2.32e-209	hypothetical protein CO539_01835 [Wolbachia	None	Unclassified
SC-2_gene_1993	261	ADF45505.1	398	249/260(95.77)	1.92e-187	DNA methylase-like protein, partial [Wolbachia endosymbiont of Culex pipiens]	363538	d__Bacteria;k__norank;p__Proteobacteria;c__Alphaproteobacteria;o__Rickettsiales;f__Anaplasmataceae;g__Wolbachia;s__Wolbachia_endosymbiont_of_Culex_pipiens
SC-2_gene_1999	151	WP_010401349.1	145	138/145(95.17)	3.58e-102	MULTISPECIES: ribonuclease HI [Wolbachia]	2640676	d__Bacteria;k__norank;p__Proteobacteria;c__Alphaproteobacteria;o__Rickettsiales;f__Anaplasmataceae;g__Wolbachia;s__norank
SC-2_gene_2001	60	OZJ02319.1	300	28/57(49.12)	2.51e-10	hypothetical protein BZG36_04448 [Bifiguratus	1938954	d__Eukaryota;k__Fungi;p__Mucoromycota;c__Endogonomycetes;o__Endogonales;f__norank;g__Bifiguratus;s__Bifiguratus_adelaidae
SC-2_gene_2002	305	WP_108784238.1	773	301/305(98.69)	3.95e-192	translation initiation factor IF-2 [Wolbachia endosymbiont of Bemisia tabaci]	215173	d__Bacteria;k__norank;p__Proteobacteria;c__Alphaproteobacteria;o__Rickettsiales;f__Anaplasmataceae;g__Wolbachia;s__Wolbachia_endosymbiont_of_Bemisia_tabaci

SC-2_gene_2025	335	WP_026092648.1	498	333/335(99.40)	9.48e-213	MULTISPECIES: murein biosynthesis integral membrane protein MurJ [Wolbachia]	116598	d__Bacteria;k__norank;p__Proteobacteria;c__Alphaproteobacteria;o__Rickettsiales;f__Anaplasmataceae;g__Wolbachia;s__Wolbachia_endosymbiont_of_Diaphorina_citri
SC-2_gene_2102	93	OSY17481.1	124	28/64(43.75)	4.91e-06	hypothetical protein B0D75_05885, partial [Entomoplasmatales bacterium	None	Unclassified
SC-2_gene_2134	307	WP_092403542.1	870	60/218(27.52)	5.71e-11	hypothetical protein [bacterium 2013Ark19i]	None	Unclassified
SC-2_gene_2148	148	OOZ40505.1	1236	63/146(43.15)	1.18e-24	hypothetical protein BOW52_05800 [Solemya	1918949	d__Bacteria;k__norank;p__Proteobacteria;c__Gammaproteobacteria;o__norank;f__norank;g__norank;s__Solemya_elarraichensis_gill_symbiont
SC-2_gene_2224	149	WP_010082217.1	394	128/140(91.43)	1.89e-80	hypothetical protein [Wolbachia endosymbiont of Drosophila simulans]	953	d__Bacteria;k__norank;p__Proteobacteria;c__Alphaproteobacteria;o__Rickettsiales;f__Anaplasmataceae;g__Wolbachia;s__norank
SC-2_gene_2313	147	RLJ22758.1	810	47/137(34.31)	3.30e-23	hypothetical protein DJ031_00290, partial [bacterium endosymbiont of Escarpia	1221601	d__Bacteria;k__norank;p__norank;c__norank;o__norank;f__norank;g__norank;s__bacterium_endosymbiont_of_Escarpia_laminata
SC-2_gene_2314	154	RYE25774.1	627	61/148(41.22)	2.11e-33	hypothetical protein EOP45_04445	2021370	d__Bacteria;k__norank;p__Bacteroidetes;c__Sphingobacteriia;o__Sphingobacteriales;f__Sphingobacteriaceae;g__norank;s__Sphingobacteriaceae_bacterium
SC-2_gene_2327	66	WP_091038853.1	387	38/63(60.32)	3.88e-19	glycoside hydrolase family 27 protein [Paenibacillus sp. CF095]	1881033	d__Bacteria;k__norank;p__Firmicutes;c__Bacilli;o__Bacillales;f__Paenibacillaceae;g__Paenibacillus;s__Paenibacillus_sp._CF095
SC-2_gene_2353	132	OSY19256.1	647	50/136(36.76)	3.54e-16	hypothetical protein B0D75_01370 [Entomoplasmatales bacterium	None	Unclassified
SC-2_gene_2365	101	PCG11334.1	195	78/79(98.73)	3.05e-45	threonylcarbamoyl-AMP synthase [Wolbachia pipientis]	None	Unclassified
SC-2_gene_2404	147	CDR79069.1	312	131/150(87.33)	1.96e-79	transcriptional regulator,anaerobic benzoate catabolism transcriptional regulator,Predicted transcriptional regulator,transcriptional regulator, y4mF family,Helix-turn-helix [Wolbachia	225364	d__Bacteria;k__norank;p__Proteobacteria;c__Alphaproteobacteria;o__Rickettsiales;f__Anaplasmataceae;g__Wolbachia;s__Wolbachia_endosymbiont_of_Drosophila_simulans
SC-2_gene_2435	111	PBQ26882.1	107	107/107(100.00)	1.67e-68	hypothetical protein BTO27_04175 [Wolbachia	2049495	d__Bacteria;k__norank;p__Proteobacteria;c__Alphaproteobacteria;o__Rickettsiales;f__Anaplasmataceae;g__Wolbachia;s__Wolbachia_pipientis
SC-2_gene_2490	78	PYZ99289.1	379	39/58(67.24)	1.54e-17	hypothetical protein A6K26_010040, partial [Gammaproteobacteria bacterium	1838287	d__Bacteria;k__norank;p__Proteobacteria;c__Gammaproteobacteria;o__norank;f__norank;g__norank;s__Gammaproteobacteria_bacterium_2W06
SC-2_gene_2496	73	APG77802.1	3002	34/69(49.28)	6.12e-13	hypothetical protein [Hubei virga-like virus 10]	1923325	d__Viruses;k__norank;p__norank;c__norank;o__norank;f__norank;g__norank;s__Hubei_virga-like_virus_10
SC-2_gene_2542	45	WP_019236610.1	317	40/41(97.56)	1.66e-16	hypothetical protein [Wolbachia pipientis]	955	d__Bacteria;k__norank;p__Proteobacteria;c__Alphaproteobacteria;o__Rickettsiales;f__Anaplasmataceae;g__Wolbachia;s__Wolbachia_pipientis
SC-2_gene_2556	139	CQD10618.1	976	72/132(54.55)	5.80e-34	Ankyrin repeat domain protein [Wolbachia endosymbiont wPip_Mol of Culex molestus]	1402163	d__Bacteria;k__norank;p__Proteobacteria;c__Alphaproteobacteria;o__Rickettsiales;f__Anaplasmataceae;g__Wolbachia;s__Wolbachia_endosymbiont_of_Culex_molestus
SC-2_gene_2641	124	RUM45877.1	86	45/85(52.94)	2.57e-18	hypothetical protein DSY80_02855 [Desulfocapsa	2066021	d__Bacteria;k__norank;p__Proteobacteria;c__Deltaproteobacteria;o__Desulfobacterales;f__Desulfobulbaceae;g__Desulfocapsa;s__Desulfocapsa_sp.
SC-2_gene_2665	199	WP_010406668.1	515	197/199(98.99)	1.14e-136	hypothetical protein, partial [Wolbachia endosymbiont of Nasonia vitripennis]	180837	d__Bacteria;k__norank;p__Proteobacteria;c__Alphaproteobacteria;o__Rickettsiales;f__Anaplasmataceae;g__Wolbachia;s__Wolbachia_endosymbiont_of_Nasonia_vitripennis

SC-2_gene_2696	301	KIJ47554.1	407	97/299(32.44)	1.38e-26	hypothetical protein M422DRAFT_112401, partial [Sphaerobolus stellatus SS14]	990650	d__Eukaryota;k__Fungi;p__Basidiomycota;c__Agaricomycetes;o__Geastrales;f__Sphaerobolaceae;g__Sphaerobolus;s__Sphaerobolus_stellatus
SC-2_gene_2714	306	RYA69540.1	154	58/146(39.73)	1.94e-32	hypothetical protein DD592_26655, partial [Enterobacter cloacae complex	2511993	d__Bacteria;k__norank;p__Proteobacteria;c__Gammaproteobacteria;o__Enterobacterales;f__Enterobacteriaceae;g__Enterobacter;s__Enterobacter_cloacae_complex_sp._2DZ2F20B
SC-2_gene_2824	120	WP_096617525.1	295	77/165(46.67)	9.60e-28	hypothetical protein [Wolbachia pipientis]	955	d__Bacteria;k__norank;p__Proteobacteria;c__Alphaproteobacteria;o__Rickettsiales;f__Anaplasmataceae;g__Wolbachia;s__Wolbachia_pipientis
SC-2_gene_2832	311	AGJ98551.1	2380	251/312(80.45)	8.60e-152	Ankyrin repeat domain protein [Wolbachia endosymbiont of Drosophila simulans wNo]	1236908	d__Bacteria;k__norank;p__Proteobacteria;c__Alphaproteobacteria;o__Rickettsiales;f__Anaplasmataceae;g__Wolbachia;s__Wolbachia_endosymbiont_of_Drosophila_simulans
SC-2_gene_2839	87	OMH85348.1	365	37/49(75.51)	1.94e-16	tRNA-aminoacylation cofactor arc1 [Zancudomyces culisetae]	1213189	d__Eukaryota;k__Fungi;p__Zoopagomycota;c__Harpellomycetes;o__Harpellales;f__Legeriomycetaceae;g__Zancudomyces;s__Zancudomyces_culisetae
SC-2_gene_2842	70	AGJ99204.1	69	67/69(97.10)	1.11e-39	hypothetical protein wNo_08110 [Wolbachia endosymbiont of Drosophila simulans wNo]	1236908	d__Bacteria;k__norank;p__Proteobacteria;c__Alphaproteobacteria;o__Rickettsiales;f__Anaplasmataceae;g__Wolbachia;s__Wolbachia_endosymbiont_of_Drosophila_simulans
SC-2_gene_2985	83	ORX49773.1	623	37/60(61.67)	1.28e-15	GTP-binding protein lepa [Hesseltinella vesiculosa]	101127	d__Eukaryota;k__Fungi;p__Mucoromycota;c__Mucoromycetes;o__Mucorales;f__Cunninghamellaceae;g__Hesseltinella;s__Hesseltinella_vesiculosa
SC-2_gene_2996	85	AJQ52466.1	438	50/76(65.79)	2.70e-17	hypothetical protein UQ52_07485 (plasmid)	369822	d__Bacteria;k__norank;p__Proteobacteria;c__Alphaproteobacteria;o__Rickettsiales;f__Rickettsiaceae;g__Rickettsia;s__Rickettsia_raoultii
SC-2_gene_3015	230	SRN42973.1	205	34/50(68.00)	6.90e-11	Uncharacterised protein [Shigella flexneri]	623	d__Bacteria;k__norank;p__Proteobacteria;c__Gammaproteobacteria;o__Enterobacterales;f__Enterobacteriaceae;g__Shigella;s__Shigella_flexneri
SC-2_gene_3023	54	RZO49937.1	82	27/54(50.00)	2.51e-09	30S ribosomal protein S15 [Pelagibacterales bacterium]	2026776	d__Bacteria;k__norank;p__Proteobacteria;c__Alphaproteobacteria;o__Pelagibacterales;f__norank;g__norank;s__Pelagibacterales_bacterium
SC-2_gene_3029	89	WP_039120656.1	1331	24/36(66.67)	4.17e-07	hypothetical protein [Flavobacterium sp. JRM]	None	Unclassified
SC-2_gene_3062	177	WP_128081746.1	336	38/42(90.48)	2.64e-12	hypothetical protein [Wolbachia endosymbiont of Dactylopius coccus]	None	Unclassified
SC-2_gene_3073	82	PJF19955.1	1609	34/64(53.12)	4.19e-12	DNA-directed RNA polymerase subunit [Paramicrosporidium saccamoebae]	1246581	d__Eukaryota;k__Fungi;p__Cryptomycota;c__norank;o__norank;f__norank;g__Paramicrosporidium;s__Paramicrosporidium_saccamoebae
SC-2_gene_3082	60	WP_084241024.1	73	40/60(66.67)	3.46e-14	hypothetical protein [Wolbachia endosymbiont of Laodelphax striatella]	None	Unclassified
SC-2_gene_3206	209	OSY19256.1	647	88/218(40.37)	1.04e-43	hypothetical protein B0D75_01370 [Entomoplasmatales bacterium	None	Unclassified
SC-2_gene_3238	89	TBR11056.1	293	31/76(40.79)	4.03e-11	fibronectin type III domain-containing protein [Candidatus Nitrosotenuis sp.]	2507556	d__Archaea;k__norank;p__Thaumarchaeota;c__norank;o__norank;f__norank;g__Candidatus_Nitrosotenuis;s__Candidatus_Nitrosotenuis_sp.
SC-2_gene_3250	270	AAF27554.1	397	191/273(69.96)	5.59e-130	putative transposase, partial (plasmid) [Lactococcus lactis]	1358	d__Bacteria;k__norank;p__Firmicutes;c__Bacilli;o__Lactobacillales;f__Streptococcaceae;g__Lactococcus;s__Lactococcus_lactis
SC-2_gene_3294	101	XP_011389183.1	932	29/62(46.77)	1.52e-08	hypothetical protein UMAG_10300 [Ustilago maydis	237631	d__Eukaryota;k__Fungi;p__Basidiomycota;c__Ustilaginomycetes;o__Ustilaginales;f__Ustilaginaceae;g__Ustilago;s__Ustilago_maydis
SC-2_gene_3317	81	GBC40048.1	94	35/90(38.89)	9.89e-06	putative nuclease harbi1 [Rhizophagus irregularis DAOM 181602=DAOM 197198]	747089	d__Eukaryota;k__Fungi;p__Mucoromycota;c__Glomeromycetes;o__Glomerales;f__Glomeraceae;g__Rhizophagus;s__Rhizophagus_irregularis
SC-2_gene_3336	194	WP_129335235.1	136	95/131(72.52)	3.40e-68	transposase [Enterobacter cloacae complex sp. 2DZ2F20B]	None	Unclassified

SC-2_gene_3341	141	RUM44711.1	113	27/43(62.79)	2.46e-08	hypothetical protein DSY80_04110 [Desulfocapsa	2066021	d__Bacteria;k__norank;p__Proteobacteria;c__Deltaproteobacteria;o__Desulfobacterales;f__Desulfobulbaceae;g__Desulfocapsa;s__Desulfocapsa_sp.
SC-2_gene_3348	137	WP_077720394.1	483	67/103(65.05)	3.72e-41	catalase [Novibacillus thermophilus]	1471761	d__Bacteria;k__norank;p__Firmicutes;c__Bacilli;o__Bacillales;f__Thermoactinomycetaceae;g__Novibacillus;s__Novibacillus_thermophilus
SC-2_gene_3361	122	WP_080872969.1	227	79/121(65.29)	3.59e-44	30S ribosomal protein S3 [bacterium endosymbiont of	1719126	d__Bacteria;k__norank;p__norank;c__norank;o__norank;f__norank;g__norank;s__bacterium_endosymbiont_of_Pedicinus_badii
SC-2_gene_3389	72	KXN68773.1	420	33/55(60.00)	4.26e-14	phosphoglycerate kinase [Conidiobolus coronatus NRRL 28638]	796925	d__Eukaryota;k__Fungi;p__Zoopagomycota;c__Entomophthoromycetes;o__Entomophthorales;f__Ancylistaceae;g__Conidiobolus;s__Conidiobolus_coronatus
SC-2_gene_3394	260	SCV66471.1	543	91/256(35.55)	2.90e-34	Integrase core domain protein [Anaplasma phagocytophilum]	948	d__Bacteria;k__norank;p__Proteobacteria;c__Alphaproteobacteria;o__Rickettsiales;f__Anaplasmataceae;g__Anaplasma;s__Anaplasma_phagocytophilum
SC-2_gene_3409	53	WP_080600782.1	140	53/53(100.00)	4.84e-29	DNA repair protein RadC [Wolbachia pipientis]	None	Unclassified
SC-2_gene_3458	256	CRQ23390.1	227	58/173(33.53)	5.61e-07	hypothetical protein PAERUG_E5_London_17_VIM_2_12_12_02321 [Pseudomonas aeruginosa]	287	d__Bacteria;k__norank;p__Proteobacteria;c__Gammaproteobacteria;o__Pseudomonadales;f__Pseudomonadaceae;g__Pseudomonas;s__Pseudomonas_aeruginosa
SC-2_gene_3460	145	KXS12630.1	421	33/96(34.38)	6.58e-09	hypothetical protein M427DRAFT_137151 [Gonapodya prolifera JEL478]	1344416	d__Eukaryota;k__Fungi;p__Chytridiomycota;c__Monoblepharidomycetes;o__Monoblepharidales;f__Gonapodyaceae;g__Gonapodya;s__Gonapodya_prolifera
SC-2_gene_3469	67	WP_131058314.1	410	27/53(50.94)	3.82e-06	reverse transcriptase family protein, partial [Clostridioides	None	Unclassified
SC-2_gene_3477	98	OYT14912.1	1239	34/98(34.69)	2.71e-11	hypothetical protein B7C24_15830 [Bacteroidetes	1972459	d__Bacteria;k__norank;p__Bacteroidetes;c__norank;o__norank;f__norank;g__norank;s__Bacteroidetes_bacterium_4572_77
SC-2_gene_3483	83	WP_129335241.1	178	41/73(56.16)	1.83e-22	transposase [Enterobacter cloacae complex sp. 2DZ2F20B]	None	Unclassified
MDJ-1_gene_1312	254	PCG11100.1	254	247/254(97.24)	5.85e-169	NAD(P)-dependent oxidoreductase [Wolbachia	None	Unclassified
MDJ-1_gene_1329	76	RYE27181.1	1800	35/67(52.24)	2.60e-10	DUF1759 domain-containing protein [Sphingobacteriaceae	2021370	d__Bacteria;k__norank;p__Bacteroidetes;c__Sphingobacteriia;o__Sphingobacteriales;f__Sphingobacteriaceae;g__norank;s__Sphingobacteriaceae_bacterium
MDJ-1_gene_1349	304	WP_064125474.1	360	275/304(90.46)	5.20e-199	hypothetical protein [Wolbachia endosymbiont of Dactylopius coccus]	None	Unclassified
MDJ-1_gene_1352	129	PBQ25931.1	129	129/129(100.00)	2.07e-85	hypothetical protein BTO27_04975 [Wolbachia	2049495	d__Bacteria;k__norank;p__Proteobacteria;c__Alphaproteobacteria;o__Rickettsiales;f__Anaplasmataceae;g__Wolbachia;s__Wolbachia_pipientis
MDJ-1_gene_1353	156	CAQ54121.1	151	145/151(96.03)	4.79e-96	hypothetical protein WP0012 [Wolbachia endosymbiont of Culex quinquefasciatus Pel]	570417	d__Bacteria;k__norank;p__Proteobacteria;c__Alphaproteobacteria;o__Rickettsiales;f__Anaplasmataceae;g__Wolbachia;s__Wolbachia_endosymbiont_of_Culex_quinquefasciatus
MDJ-1_gene_1356	166	PBQ28113.1	164	164/164(100.00)	2.39e-106	transcription elongation factor GreA [Wolbachia pipientis wAus]	2049495	d__Bacteria;k__norank;p__Proteobacteria;c__Alphaproteobacteria;o__Rickettsiales;f__Anaplasmataceae;g__Wolbachia;s__Wolbachia_pipientis
MDJ-1_gene_1359	196	YP_001967398.1	196	180/195(92.31)	4.75e-128	ParA-like protein (plasmid) [Rickettsia monacensis]	None	Unclassified
MDJ-1_gene_1362	116	RYD06921.1	87	41/95(43.16)	2.89e-10	hypothetical protein N752_01975 [Desulfotomaculum aquiferis]	1397668	d__Bacteria;k__norank;p__Firmicutes;c__Clostridia;o__Clostridiales;f__Peptococcaceae;g__Desulfotomaculum;s__Desulfotomaculum_aquiferis
MDJ-1_gene_1369	156	AGJ99318.1	156	153/156(98.08)	1.24e-99	hypothetical protein wNo_09390 [Wolbachia endosymbiont of Drosophila simulans wNo]	1236908	d__Bacteria;k__norank;p__Proteobacteria;c__Alphaproteobacteria;o__Rickettsiales;f__Anaplasmataceae;g__Wolbachia;s__Wolbachia_endosymbiont_of_Drosophila_simulans
MDJ-1_gene_1373	204	EER20755.1	190	164/195(84.10)	2.47e-101	conserved hypothetical protein (plasmid) [Rickettsia endosymbiont of Ixodes	444612	d__Bacteria;k__norank;p__Proteobacteria;c__Alphaproteobacteria;o__Rickettsiales;f__Rickettsiaceae;g__Rickettsia;s__Rickettsia_endosymbiont_of_Ixodes_scapularis

MDJ-1_gene_1374	56	WP_052692921.1	60	27/52(51.92)	6.53e-09	hypothetical protein [Rickettsia bellii]	None	Unclassified
MDJ-1_gene_1389	107	WP_017532512.1	107	106/107(99.07)	8.85e-72	Grx4 family monothiol glutaredoxin [Wolbachia endosymbiont of Diaphorina citri]	116598	d__Bacteria;k__norank;p__Proteobacteria;c__Alphaproteobacteria;o__Rickettsiales;f__Anaplasmataceae;g__Wolbachia;s__Wolbachia_endosymbiont_of_Diaphorina_citri
MDJ-1_gene_1390	72	PCG11434.1	72	72/72(100.00)	1.71e-44	cell division protein BolA [Wolbachia pipientis]	None	Unclassified
MDJ-1_gene_1412	136	WP_127463894.1	139	101/139(72.66)	5.24e-24	collagen-like protein [Wolbachia endosymbiont of Bemisia tabaci]	215173	d__Bacteria;k__norank;p__Proteobacteria;c__Alphaproteobacteria;o__Rickettsiales;f__Anaplasmataceae;g__Wolbachia;s__Wolbachia_endosymbiont_of_Bemisia_tabaci
MDJ-1_gene_1416	75	RPH68112.1	255	31/71(43.66)	1.24e-09	carotenoid synthesis regulator CarF [Myxococcaceae bacterium]	2484252	d__Bacteria;k__norank;p__Proteobacteria;c__Deltaproteobacteria;o__Myxococcales;f__Myxococcaceae;g__norank;s__Myxococcaceae_bacterium
MDJ-1_gene_1421	76	P23092.1	390	62/75(82.67)	4.93e-37	RecName: Full=Transforming protein cbl	11793	d__Viruses;k__Pararnavirae;p__Artverviricota;c__Revtraviricetes;o__Ortervirales;f__Retroviridae;g__Gammaretrovirus;s__Murine_leukemia_virus
MDJ-1_gene_1424	55	XP_008073719.1	103	24/40(60.00)	1.39e-07	hypothetical protein VCUG_00698 [Vavraia culicis	948595	d__Eukaryota;k__Fungi;p__Microsporidia;c__norank;o__norank;f__Pleistophoridae;g__Vavraia;s__Vavraia_culicis
MDJ-1_gene_1426	207	EER20887.1	207	141/205(68.78)	2.03e-86	peptidase M50 (plasmid) [Rickettsia endosymbiont of	444612	d__Bacteria;k__norank;p__Proteobacteria;c__Alphaproteobacteria;o__Rickettsiales;f__Rickettsiaceae;g__Rickettsia;s__Rickettsia_endosymbiont_of_Ixodes_scapularis
MDJ-1_gene_1438	96	RUA04709.1	567	38/101(37.62)	5.17e-07	hypothetical protein DSY43_05900, partial [Gammaproteobacteria	1913989	d__Bacteria;k__norank;p__Proteobacteria;c__Gammaproteobacteria;o__norank;f__norank;g__norank;s__Gammaproteobacteria_bacterium
MDJ-1_gene_1441	71	WP_130477711.1	262	35/70(50.00)	1.57e-10	ATP-binding cassette domain-containing protein [Amycolatopsis sp. 8-3EHSu]	2510978	d__Bacteria;k__norank;p__Actinobacteria;c__Actinobacteria;o__Pseudonocardiales;f__Pseudonocardiaceae;g__Amycolatopsis;s__Amycolatopsis_suaedae
MDJ-1_gene_1451	85	AWA82275.1	2735	48/85(56.47)	6.34e-22	polyprotein [Beult virus]	2170589	d__Viruses;k__norank;p__norank;c__norank;o__norank;f__norank;g__norank;s__Beult_virus
MDJ-1_gene_1453	310	WP_096097157.1	1237	222/224(99.11)	4.96e-135	collagen-like protein [Wolbachia pipientis]	955	d__Bacteria;k__norank;p__Proteobacteria;c__Alphaproteobacteria;o__Rickettsiales;f__Anaplasmataceae;g__Wolbachia;s__Wolbachia_pipientis
MDJ-1_gene_1483	171	WP_114517464.1	142	101/120(84.17)	3.30e-66	hypothetical protein [Wolbachia endosymbiont of Cylisticus convexus]	118728	d__Bacteria;k__norank;p__Proteobacteria;c__Alphaproteobacteria;o__Rickettsiales;f__Anaplasmataceae;g__Wolbachia;s__Wolbachia_endosymbiont_of_Cylisticus_convexus
MDJ-1_gene_1490	108	PBQ27221.1	108	98/108(90.74)	1.78e-67	thioredoxin [Wolbachia pipientis wAus]	2049495	d__Bacteria;k__norank;p__Proteobacteria;c__Alphaproteobacteria;o__Rickettsiales;f__Anaplasmataceae;g__Wolbachia;s__Wolbachia_pipientis
MDJ-1_gene_1504	64	WP_108448738.1	167	40/64(62.50)	2.94e-21	lysozyme [Halomonas sp. BN3-1]	2082393	d__Bacteria;k__norank;p__Proteobacteria;c__Gammaproteobacteria;o__Oceanospirillales;f__Halomonadaceae;g__Halomonas;s__Halomonas_sp._BN3-1
MDJ-1_gene_1505	99	CNL53069.1	93	51/92(55.43)	6.12e-31	Uncharacterised protein [Yersinia intermedia]	631	d__Bacteria;k__norank;p__Proteobacteria;c__Gammaproteobacteria;o__Enterobacterales;f__Yersiniaceae;g__Yersinia;s__Yersinia_intermedia
MDJ-1_gene_1508	95	AGJ99217.1	95	93/95(97.89)	6.67e-57	50S ribosomal protein L23 [Wolbachia endosymbiont of Drosophila simulans wNo]	1236908	d__Bacteria;k__norank;p__Proteobacteria;c__Alphaproteobacteria;o__Rickettsiales;f__Anaplasmataceae;g__Wolbachia;s__Wolbachia_endosymbiont_of_Drosophila_simulans
MDJ-1_gene_1520	215	CQD09022.1	223	208/223(93.27)	6.98e-139	ompA-like protein [Wolbachia endosymbiont wPip_Mol of Culex molestus]	1402163	d__Bacteria;k__norank;p__Proteobacteria;c__Alphaproteobacteria;o__Rickettsiales;f__Anaplasmataceae;g__Wolbachia;s__Wolbachia_endosymbiont_of_Culex_molestus
MDJ-1_gene_1535	141	GBC14668.1	475	57/149(38.26)	2.40e-20	retrotransposable element [Rhizophagus irregularis DAOM 181602=DAOM 197198]	747089	d__Eukaryota;k__Fungi;p__Mucoromycota;c__Glomeromycetes;o__Glomerales;f__Glomeraceae;g__Rhizophagus;s__Rhizophagus_irregularis
MDJ-1_gene_1543	292	WP_096616095.1	519	287/292(98.29)	2.61e-189	cation:proton antiporter [Wolbachia pipientis]	955	d__Bacteria;k__norank;p__Proteobacteria;c__Alphaproteobacteria;o__Rickettsiales;f__Anaplasmataceae;g__Wolbachia;s__Wolbachia_pipientis
MDJ-1_gene_1546	158	ETW98372.1	393	68/158(43.04)	1.50e-32	hypothetical protein ETSY2_42930 [Candidatus	1429439	d__Bacteria;k__norank;p__Candidatus_Tectomicrobia;c__norank;o__norank;f__norank;g__Candidatus_Entotheonella;s__Candidatus_Entotheonella_gemina
MDJ-1_gene_1564	260	WP_127464084.1	260	256/260(98.46)	2.44e-189	exodeoxyribonuclease III [Wolbachia endosymbiont of	215173	d__Bacteria;k__norank;p__Proteobacteria;c__Alphaproteobacteria;o__Rickettsiales;f__Anaplasmataceae;g__Wolbachia;s__Wolbachia_endosymbiont_of_Bemisia_tabaci

MDJ-1_gene_1576	165	PCS11289.1	165	137/164(83.54)	1.02e-91	hypothetical protein RU90_GL000771 [Lactococcus	203404	d__Bacteria;k__norank;p__Firmicutes;c__Bacilli;o__Lactobacillales;f__Streptococcaceae;g__Lactococcus;s__Lactococcus_lactis
MDJ-1_gene_1578	217	WP_061412686.1	317	183/216(84.72)	5.48e-133	site-specific integrase [Lactococcus sp. DD01]	1776443	d__Bacteria;k__norank;p__Firmicutes;c__Bacilli;o__Lactobacillales;f__Streptococcaceae;g__Lactococcus;s__Lactococcus_sp._DD01
MDJ-1_gene_1579	79	EQC91185.1	135	78/79(98.73)	2.15e-50	hypothetical protein LLT3_16395, partial [Lactococcus lactis subsp.	1234873	d__Bacteria;k__norank;p__Firmicutes;c__Bacilli;o__Lactobacillales;f__Streptococcaceae;g__Lactococcus;s__Lactococcus_lactis
MDJ-1_gene_1581	243	EIE92290.1	255	90/201(44.78)	1.44e-51	hypothetical protein RO3G_17161 [Rhizopus delemar	246409	d__Eukaryota;k__Fungi;p__Mucoromycota;c__Mucoromycetes;o__Mucorales;f__Rhizopodaceae;g__Rhizopus;s__Rhizopus_delemar
MDJ-1_gene_1587	61	RYG62530.1	303	37/52(71.15)	2.09e-14	hypothetical protein EON64_17700, partial [archaeon]	1906665	d__Archaea;k__norank;p__norank;c__norank;o__norank;f__norank;g__norank;s__archaeon
MDJ-1_gene_1588	102	ORZ31702.1	625	65/98(66.33)	8.36e-31	P-loop containing nucleoside triphosphate hydrolase protein [Catenaria anguillulae PL171]	765915	d__Eukaryota;k__Fungi;p__Blastocladiomycota;c__Blastocladiomycetes;o__Blastocladiales;f__Catenariaceae;g__Catenaria;s__Catenaria_anguillulae
MDJ-1_gene_1598	44	ONI56074.1	67	37/44(84.09)	2.41e-20	transposase domain protein [Wolbachia pipientis wUni]	1379787	d__Bacteria;k__norank;p__Proteobacteria;c__Alphaproteobacteria;o__Rickettsiales;f__Anaplasmataceae;g__Wolbachia;s__Wolbachia_pipientis
MDJ-1_gene_1609	57	AGO15620.1	71	31/45(68.89)	4.13e-14	hypothetical protein K756_01780 [Glaesserella parasuis ZJ0906]	1322346	d__Bacteria;k__norank;p__Proteobacteria;c__Gammaproteobacteria;o__Pasteurellales;f__Pasteurellaceae;g__Glaesserella;s__Glaesserella_parasuis
MDJ-1_gene_1610	120	AUR81760.1	126	41/116(35.34)	5.32e-14	hypothetical protein NVP1013O_13 [Vibrio phage 1.013.O._10N.286.54.F9]	1881244	d__Viruses;k__norank;p__Uroviricota;c__Caudoviricetes;o__Caudovirales;f__Siphoviridae;g__norank;s__Vibrio_phage_1.013.O._10N.286.54.F9
MDJ-1_gene_1624	42	RLT60163.1	42	34/42(80.95)	3.71e-13	hypothetical protein WANA31_0778 [Wolbachia endosymbiont of Drosophila	307502	d__Bacteria;k__norank;p__Proteobacteria;c__Alphaproteobacteria;o__Rickettsiales;f__Anaplasmataceae;g__Wolbachia;s__Wolbachia_endosymbiont_of_Drosophila_ananassae
MDJ-1_gene_1625	261	CQD05446.1	571	203/211(96.21)	2.29e-139	collagen triple helix repeat protein [Wolbachia endosymbiont wPip_Mol of Culex molestus]	1402163	d__Bacteria;k__norank;p__Proteobacteria;c__Alphaproteobacteria;o__Rickettsiales;f__Anaplasmataceae;g__Wolbachia;s__Wolbachia_endosymbiont_of_Culex_molestus
MDJ-1_gene_1630	127	YP_008126240.1	127	55/127(43.31)	1.79e-32	hypothetical protein VPRG_00077 [Vibrio phage	754051	d__Viruses;k__norank;p__Uroviricota;c__Caudoviricetes;o__Caudovirales;f__Siphoviridae;g__Roufvirus;s__Vibrio_phage_pYD38-A
MDJ-1_gene_1634	66	RKO95007.1	90	27/60(45.00)	5.25e-11	C-terminal domain of adenylylcyclase associated protein, partial [Caulochytrium protostelioides]	1555241	d__Eukaryota;k__Fungi;p__Chytridiomycota;c__Chytridiomycetes;o__Caulochytriales;f__Caulochytriaceae;g__Caulochytrium;s__Caulochytrium_protostelioides
MDJ-1_gene_1642	106	ORY98566.1	712	33/84(39.29)	1.31e-08	Endonuclease/exonuclease/phosphatase [Syncephalastrum	13706	d__Eukaryota;k__Fungi;p__Mucoromycota;c__Mucoromycetes;o__Mucorales;f__Syncephalastraceae;g__Syncephalastrum;s__Syncephalastrum_racemosum
MDJ-1_gene_1655	43	WP_108784111.1	97	41/41(100.00)	6.28e-21	GIY-YIG nuclease family protein [Wolbachia endosymbiont of Bemisia tabaci]	215173	d__Bacteria;k__norank;p__Proteobacteria;c__Alphaproteobacteria;o__Rickettsiales;f__Anaplasmataceae;g__Wolbachia;s__Wolbachia_endosymbiont_of_Bemisia_tabaci
MDJ-1_gene_1668	168	WP_019236469.1	168	161/168(95.83)	4.09e-113	phage major tail tube protein [Wolbachia pipientis]	955	d__Bacteria;k__norank;p__Proteobacteria;c__Alphaproteobacteria;o__Rickettsiales;f__Anaplasmataceae;g__Wolbachia;s__Wolbachia_pipientis
MDJ-1_gene_1671	154	EEB56513.1	153	137/154(88.96)	9.54e-96	putative outer membrane protein [Wolbachia endosymbiont of Culex quinquefasciatus JHB]	569881	d__Bacteria;k__norank;p__Proteobacteria;c__Alphaproteobacteria;o__Rickettsiales;f__Anaplasmataceae;g__Wolbachia;s__Wolbachia_endosymbiont_of_Culex_quinquefasciatus
MDJ-1_gene_1676	147	PBQ27635.1	147	147/147(100.00)	6.84e-104	PAS domain protein [Wolbachia pipientis wAus]	2049495	d__Bacteria;k__norank;p__Proteobacteria;c__Alphaproteobacteria;o__Rickettsiales;f__Anaplasmataceae;g__Wolbachia;s__Wolbachia_pipientis
MDJ-1_gene_1682	156	WP_065094961.1	156	152/156(97.44)	1.52e-109	MULTISPECIES: Holliday junction resolvase [Wolbachia]	2640676	d__Bacteria;k__norank;p__Proteobacteria;c__Alphaproteobacteria;o__Rickettsiales;f__Anaplasmataceae;g__Wolbachia;s__norank
MDJ-1_gene_1713	122	BBA84857.1	122	71/119(59.66)	6.74e-35	50S ribosomal protein L17 [endosymbiont of Pachyrhynchus infernalis]	1971488	d__Bacteria;k__norank;p__Proteobacteria;c__Gammaproteobacteria;o__norank;f__norank;g__Candidatus_Nardonella;s__endosymbiont_of_Pachyrhynchus_infernalis

MDJ-1_gene_1723	101	RUS23965.1	228	67/89(75.28)	5.72e-37	kinase-like domain-containing protein [Jimgerdemannia flammicorona]	994334	d__Eukaryota;k__Fungi;p__Mucoromycota;c__Endogonomycetes;o__Endogonales;f__Endogonaceae;g__Jimgerdemannia;s__Jimgerdemannia_flammicorona
MDJ-1_gene_1730	139	RUM44802.1	320	50/136(36.76)	8.55e-17	hypothetical protein DSY80_04010, partial	2066021	d__Bacteria;k__norank;p__Proteobacteria;c__Deltaproteobacteria;o__Desulfobacterales;f__Desulfobulbaceae;g__Desulfocapsa;s__Desulfocapsa_sp.
MDJ-1_gene_1772	187	CAQ55039.1	187	178/187(95.19)	1.19e-128	peptidyl-tRNA hydrolase [Wolbachia endosymbiont of Culex quinquefasciatus Pel]	570417	d__Bacteria;k__norank;p__Proteobacteria;c__Alphaproteobacteria;o__Rickettsiales;f__Anaplasmataceae;g__Wolbachia;s__Wolbachia_endosymbiont_of_Culex_quinquefasciatus
MDJ-1_gene_1779	87	RKP08420.1	406	37/83(44.58)	9.36e-13	hypothetical protein THASP1DRAFT_29778 [Thamnocephalis sphaerospora]	78915	d__Eukaryota;k__Fungi;p__Zoopagomycota;c__Zoopagomycetes;o__Zoopagales;f__Sigmoideomycetaceae;g__Thamnocephalis;s__Thamnocephalis_sphaerospora
MDJ-1_gene_1788	177	WP_127464147.1	189	157/190(82.63)	2.80e-86	hypothetical protein [Wolbachia endosymbiont of Bemisia tabaci]	215173	d__Bacteria;k__norank;p__Proteobacteria;c__Alphaproteobacteria;o__Rickettsiales;f__Anaplasmataceae;g__Wolbachia;s__Wolbachia_endosymbiont_of_Bemisia_tabaci
MDJ-1_gene_1795	59	WP_041581345.1	59	57/59(96.61)	1.50e-28	hypothetical protein [Wolbachia endosymbiont of Drosophila simulans]	2640676	d__Bacteria;k__norank;p__Proteobacteria;c__Alphaproteobacteria;o__Rickettsiales;f__Anaplasmataceae;g__Wolbachia;s__norank
MDJ-1_gene_1805	59	ORX86125.1	150	35/58(60.34)	1.78e-20	histone methyltransferase, partial [Anaeromyces robustus]	1754192	d__Eukaryota;k__Fungi;p__Chytridiomycota;c__Neocallimastigomycetes;o__Neocallimastigales;f__Neocallimastigaceae;g__Anaeromyces;s__Anaeromyces_robustus
MDJ-1_gene_1811	272	EQC57300.1	524	141/261(54.02)	1.72e-77	hypothetical protein LLT6_11180 [Lactococcus lactis subsp. cremoris TIFN6]	1234876	d__Bacteria;k__norank;p__Firmicutes;c__Bacilli;o__Lactobacillales;f__Streptococcaceae;g__Lactococcus;s__Lactococcus_lactis
MDJ-1_gene_1814	142	RIZ67154.1	143	76/140(54.29)	4.97e-49	50S ribosomal protein L11 [Methylococcales bacterium]	2304002	d__Bacteria;k__norank;p__Proteobacteria;c__Gammaproteobacteria;o__Methylococcales;f__norank;g__norank;s__Methylococcales_bacterium
MDJ-1_gene_1825	171	CCA67182.1	661	78/170(45.88)	3.55e-39	probable SAC1-recessive suppressor of secretory defect [Serendipita indica DSM 11827]	1109443	d__Eukaryota;k__Fungi;p__Basidiomycota;c__Agaricomycetes;o__Sebacinales;f__Serendipitaceae;g__Serendipita;s__Serendipita_indica
MDJ-1_gene_1829	270	PKK63813.1	416	70/227(30.84)	3.57e-25	hypothetical protein RhiirC2_812061 [Rhizophagus	588596	d__Eukaryota;k__Fungi;p__Mucoromycota;c__Glomeromycetes;o__Glomerales;f__Glomeraceae;g__Rhizophagus;s__Rhizophagus_irregularis
MDJ-1_gene_1836	214	PCG11679.1	214	206/214(96.26)	1.64e-146	phospholipase [Wolbachia	None	Unclassified
MDJ-1_gene_1859	47	WP_127897150.1	114	40/47(85.11)	1.37e-19	hypothetical protein [Streptomyces sp. S10(2018)]	None	Unclassified
MDJ-1_gene_1861	206	WP_109990918.1	438	173/208(83.17)	2.47e-120	hypothetical protein [Lactococcus lactis]	1358	d__Bacteria;k__norank;p__Firmicutes;c__Bacilli;o__Lactobacillales;f__Streptococcaceae;g__Lactococcus;s__Lactococcus_lactis
MDJ-1_gene_1862	60	KXT63144.1	57	37/55(67.27)	1.84e-11	hypothetical protein LACDD01_00171 [Lactococcus sp. DD01]	1776443	d__Bacteria;k__norank;p__Firmicutes;c__Bacilli;o__Lactobacillales;f__Streptococcaceae;g__Lactococcus;s__Lactococcus_sp._DD01
MDJ-1_gene_1896	80	EPS45076.1	222	38/62(61.29)	7.68e-16	hypothetical protein H072_875 [Dactylellina haptotyla CBS	1284197	d__Eukaryota;k__Fungi;p__Ascomycota;c__Orbiliomycetes;o__Orbiliales;f__Orbiliaceae;g__Dactylellina;s__Dactylellina_haptotyla
MDJ-1_gene_1904	65	RQW07486.1	201	44/61(72.13)	8.47e-25	catalase, partial [candidate division KSB1 bacterium]	2172550	d__Bacteria;k__norank;p__norank;c__norank;o__norank;f__norank;g__norank;s__candidate_division_KSB1_bacterium
MDJ-1_gene_1946	174	AGJ99091.1	174	169/174(97.13)	4.72e-115	Cytochrome c family protein [Wolbachia endosymbiont of Drosophila simulans wNo]	1236908	d__Bacteria;k__norank;p__Proteobacteria;c__Alphaproteobacteria;o__Rickettsiales;f__Anaplasmataceae;g__Wolbachia;s__Wolbachia_endosymbiont_of_Drosophila_simulans
MDJ-1_gene_1983	152	AGJ99336.1	151	119/154(77.27)	3.62e-74	Ankyrin repeat domain protein [Wolbachia endosymbiont of Drosophila simulans wNo]	1236908	d__Bacteria;k__norank;p__Proteobacteria;c__Alphaproteobacteria;o__Rickettsiales;f__Anaplasmataceae;g__Wolbachia;s__Wolbachia_endosymbiont_of_Drosophila_simulans
MDJ-1_gene_1984	224	CAQ55484.1	771	108/227(47.58)	3.87e-46	Hypothetical protein WP1377 [Wolbachia endosymbiont of Culex quinquefasciatus Pel]	570417	d__Bacteria;k__norank;p__Proteobacteria;c__Alphaproteobacteria;o__Rickettsiales;f__Anaplasmataceae;g__Wolbachia;s__Wolbachia_endosymbiont_of_Culex_quinquefasciatus

MDJ-1_gene_1991	195	WP_109990909.1	451	179/195(91.79)	9.67e-120	PBSX family phage terminase large subunit [Lactococcus lactis]	1358	d__Bacteria;k__norank;p__Firmicutes;c__Bacilli;o__Lactobacillales;f__Streptococcaceae;g__Lactococcus;s__Lactococcus_lactis
MDJ-1_gene_1996	110	TBU01100.1	452	38/112(33.93)	8.10e-12	hypothetical protein CWI37_0807p0020 [Hamiltosporidium	1176355	d__Eukaryota;k__Fungi;p__Microsporidia;c__norank;o__norank;f__Dubosqiidae;g__Hamiltosporidium;s__Hamiltosporidium_tvaerminnensis
MDJ-1_gene_2019	142	RYE27181.1	1800	43/112(38.39)	2.45e-17	DUF1759 domain-containing protein [Sphingobacteriaceae	2021370	d__Bacteria;k__norank;p__Bacteroidetes;c__Sphingobacteriia;o__Sphingobacteriales;f__Sphingobacteriaceae;g__norank;s__Sphingobacteriaceae_bacterium
MDJ-1_gene_2031	146	ERN55802.1	146	132/146(90.41)	6.33e-95	ribonuclease h [Wolbachia pipientis wMelPop]	1317678	d__Bacteria;k__norank;p__Proteobacteria;c__Alphaproteobacteria;o__Rickettsiales;f__Anaplasmataceae;g__Wolbachia;s__Wolbachia_endosymbiont_of_Drosophila_melanogaster
MDJ-1_gene_2067	80	ORY23344.1	653	36/63(57.14)	4.78e-14	hypothetical protein BCR33DRAFT_135222 [Rhizoclosmatium globosum]	329046	d__Eukaryota;k__Fungi;p__Chytridiomycota;c__Chytridiomycetes;o__Chytridiales;f__Chytriomycetaceae;g__Rhizoclosmatium;s__Rhizoclosmatium_globosum
MDJ-1_gene_2070	163	BBA85342.1	484	126/163(77.30)	1.11e-75	RNA polymerase sigma factor RpoD [endosymbiont of Sipalinus	1972134	d__Bacteria;k__norank;p__Proteobacteria;c__Gammaproteobacteria;o__norank;f__norank;g__Candidatus_Nardonella;s__endosymbiont_of_Sipalinus_gigas
MDJ-1_gene_2083	62	PWN51876.1	2094	25/49(51.02)	2.94e-08	hypothetical protein IE53DRAFT_31699 [Violaceomyces palustris]	1673888	d__Eukaryota;k__Fungi;p__Basidiomycota;c__Ustilaginomycetes;o__Violaceomycetales;f__Violaceomycetaceae;g__Violaceomyces;s__Violaceomyces_palustris
MDJ-1_gene_2085	245	WP_108783926.1	197	190/192(98.96)	2.81e-133	ribonuclease HII [Wolbachia endosymbiont of Bemisia tabaci]	215173	d__Bacteria;k__norank;p__Proteobacteria;c__Alphaproteobacteria;o__Rickettsiales;f__Anaplasmataceae;g__Wolbachia;s__Wolbachia_endosymbiont_of_Bemisia_tabaci
MDJ-1_gene_2095	55	RAM39399.1	98	22/44(50.00)	3.09e-08	hypothetical protein DOZ52_29480, partial	158836	d__Bacteria;k__norank;p__Proteobacteria;c__Gammaproteobacteria;o__Enterobacterales;f__Enterobacteriaceae;g__Enterobacter;s__Enterobacter_hormaechei
MDJ-1_gene_2168	71	CAQ54561.1	71	63/71(88.73)	1.68e-37	hypothetical protein WP0453 [Wolbachia endosymbiont of Culex quinquefasciatus Pel]	570417	d__Bacteria;k__norank;p__Proteobacteria;c__Alphaproteobacteria;o__Rickettsiales;f__Anaplasmataceae;g__Wolbachia;s__Wolbachia_endosymbiont_of_Culex_quinquefasciatus
MDJ-1_gene_2181	94	EEF60287.1	444	29/76(38.16)	1.20e-06	proteinase inhibitor I4 serpin [Pedosphaera parvula Ellin514]	320771	d__Bacteria;k__norank;p__Verrucomicrobia;c__Verrucomicrobiae;o__Verrucomicrobiales;f__Verrucomicrobia_subdivision_3;g__Pedosphaera;s__Pedosphaera_parvula
MDJ-1_gene_2198	128	CZW33430.1	304	75/129(58.14)	2.23e-44	Uncharacterised protein [Enterobacter cloacae]	158836	d__Bacteria;k__norank;p__Proteobacteria;c__Gammaproteobacteria;o__Enterobacterales;f__Enterobacteriaceae;g__Enterobacter;s__Enterobacter_hormaechei
MDJ-1_gene_2245	67	WP_109716113.1	67	63/67(94.03)	1.43e-34	hypothetical protein [Pantoea allii]	574096	d__Bacteria;k__norank;p__Proteobacteria;c__Gammaproteobacteria;o__Enterobacterales;f__Erwiniaceae;g__Pantoea;s__Pantoea_allii
MDJ-1_gene_2273	177	WP_109990916.1	216	153/176(86.93)	1.78e-118	hypothetical protein [Lactococcus lactis]	1358	d__Bacteria;k__norank;p__Firmicutes;c__Bacilli;o__Lactobacillales;f__Streptococcaceae;g__Lactococcus;s__Lactococcus_lactis
MDJ-1_gene_2280	59	XP_016610393.1	332	37/47(78.72)	1.30e-17	hypothetical protein SPPG_02824 [Spizellomyces punctatus DAOM BR117]	645134	d__Eukaryota;k__Fungi;p__Chytridiomycota;c__Chytridiomycetes;o__Spizellomycetales;f__Spizellomycetaceae;g__Spizellomyces;s__Spizellomyces_punctatus
MDJ-1_gene_2303	176	OLS13299.1	237	85/129(65.89)	2.17e-47	ribose-5-phosphate isomerase A [Candidatus Lokiarchaeota archaeon CR_4]	1849166	d__Archaea;k__norank;p__Candidatus_Lokiarchaeota;c__norank;o__norank;f__norank;g__norank;s__Candidatus_Lokiarchaeota_archaeon_CR_4
MDJ-1_gene_2304	78	AGH25189.1	72	25/39(64.10)	4.72e-07	putative single-stranded DNA binding protein [Escherichia virus KP26]	1920999	d__Viruses;k__norank;p__Uroviricota;c__Caudoviricetes;o__Caudovirales;f__Drexlerviridae;g__Rogunavirus;s__Escherichia_virus_KP26
MDJ-1_gene_2305	116	AUE23435.1	141	54/114(47.37)	4.74e-25	hypothetical protein Ro1_00230 [Raoultella phage Ro1]	2053702	d__Viruses;k__norank;p__Uroviricota;c__Caudoviricetes;o__Caudovirales;f__Myoviridae;g__norank;s__Raoultella_phage_Ro1
MDJ-1_gene_2307	78	KNE88261.1	117	43/55(78.18)	3.38e-27	hypothetical protein PSTG_18343, partial [Puccinia striiformis f. sp. tritici PST-78]	1165861	d__Eukaryota;k__Fungi;p__Basidiomycota;c__Pucciniomycetes;o__Pucciniales;f__Pucciniaceae;g__Puccinia;s__Puccinia_striiformis
MDJ-1_gene_2325	126	ACF98009.1	341	38/87(43.68)	1.07e-11	SsnF, partial [Aspergillus nidulans]	162425	d__Eukaryota;k__Fungi;p__Ascomycota;c__Eurotiomycetes;o__Eurotiales;f__Aspergillaceae;g__Aspergillus;s__Aspergillus_nidulans
MDJ-1_gene_2326	226	AYR02305.1	470	144/223(64.57)	4.72e-108	hypothetical protein [Escherichia phage C1]	2340716	d__Viruses;k__norank;p__Uroviricota;c__Caudoviricetes;o__Caudovirales;f__Siphoviridae;g__norank;s__Escherichia_phage_C1

MDJ-1_gene_2330	225	WP_017532601.1	773	222/225(98.67)	9.28e-144	translation initiation factor IF-2 [Wolbachia endosymbiont of Diaphorina citri]	116598	d__Bacteria;k__norank;p__Proteobacteria;c__Alphaproteobacteria;o__Rickettsiales;f__Anaplasmataceae;g__Wolbachia;s__Wolbachia_endosymbiont_of_Diaphorina_citri
MDJ-1_gene_2333	155	WP_077190435.1	153	137/148(92.57)	6.24e-99	Holliday junction resolvase [Wolbachia pipientis]	955	d__Bacteria;k__norank;p__Proteobacteria;c__Alphaproteobacteria;o__Rickettsiales;f__Anaplasmataceae;g__Wolbachia;s__Wolbachia_pipientis
MDJ-1_gene_2340	75	RKP36381.1	554	28/57(49.12)	2.15e-09	sphinganine-1-phosphate aldolase [Dimargaris cristalligena]	215637	d__Eukaryota;k__Fungi;p__Zoopagomycota;c__Dimargaritomycetes;o__Dimargaritales;f__Dimargaritaceae;g__Dimargaris;s__Dimargaris_cristalligena
MDJ-1_gene_2343	139	WP_065649197.1	135	35/57(61.40)	1.38e-15	hypothetical protein [Pantoea eucrina]	None	Unclassified
MDJ-1_gene_2344	79	VAV78196.1	271	36/43(83.72)	2.90e-16	Uncharacterised protein [Enterobacter cloacae]	158836	d__Bacteria;k__norank;p__Proteobacteria;c__Gammaproteobacteria;o__Enterobacterales;f__Enterobacteriaceae;g__Enterobacter;s__Enterobacter_hormaechei
MDJ-1_gene_2349	61	KIJ47554.1	407	24/52(46.15)	1.17e-06	hypothetical protein M422DRAFT_112401, partial [Sphaerobolus stellatus SS14]	990650	d__Eukaryota;k__Fungi;p__Basidiomycota;c__Agaricomycetes;o__Geastrales;f__Sphaerobolaceae;g__Sphaerobolus;s__Sphaerobolus_stellatus
MDJ-1_gene_2359	70	WP_127110902.1	211	28/45(62.22)	9.29e-09	DUF243 domain-containing protein, partial [Pararhodobacter	2479545	d__Bacteria;k__norank;p__Proteobacteria;c__Alphaproteobacteria;o__Rhodobacterales;f__Rhodobacteraceae;g__Pararhodobacter;s__Pararhodobacter_zhoushanensis
MDJ-1_gene_2360	82	BBA85036.1	174	58/82(70.73)	1.69e-32	ATP-dependent protease subunit HslV [endosymbiont of Rhynchophorus ferrugineus]	1972133	d__Bacteria;k__norank;p__Proteobacteria;c__Gammaproteobacteria;o__norank;f__norank;g__Candidatus_Nardonella;s__Candidatus_Nardonella_dryophthoridicola
MDJ-1_gene_2361	80	BBA85322.1	176	59/79(74.68)	4.98e-29	ATP-dependent protease subunit HslV [endosymbiont of Sipalinus gigas]	1972134	d__Bacteria;k__norank;p__Proteobacteria;c__Gammaproteobacteria;o__norank;f__norank;g__Candidatus_Nardonella;s__endosymbiont_of_Sipalinus_gigas
MDJ-1_gene_2371	71	XP_016605529.1	4556	37/67(55.22)	3.57e-17	hypothetical protein SPPG_07404 [Spizellomyces punctatus DAOM BR117]	645134	d__Eukaryota;k__Fungi;p__Chytridiomycota;c__Chytridiomycetes;o__Spizellomycetales;f__Spizellomycetaceae;g__Spizellomyces;s__Spizellomyces_punctatus
MDJ-1_gene_2380	221	OCA06393.1	825	218/221(98.64)	6.43e-143	aconitate hydratase 1 [Wolbachia endosymbiont of Trichogramma pretiosum]	125593	d__Bacteria;k__norank;p__Proteobacteria;c__Alphaproteobacteria;o__Rickettsiales;f__Anaplasmataceae;g__Wolbachia;s__Wolbachia_endosymbiont_of_Trichogramma_pretiosum
MDJ-1_gene_2387	69	OJH31509.1	69	67/69(97.10)	1.97e-44	Phage Tail Protein X [Wolbachia endosymbiont of Armadillidium vulgare]	77039	d__Bacteria;k__norank;p__Proteobacteria;c__Alphaproteobacteria;o__Rickettsiales;f__Anaplasmataceae;g__Wolbachia;s__Wolbachia_endosymbiont_of_Armadillidium_vulgare
MDJ-1_gene_2390	78	ORM76735.1	78	77/77(100.00)	5.89e-50	hypothetical protein HA42_18730 [Pantoea deleyi]	470932	d__Bacteria;k__norank;p__Proteobacteria;c__Gammaproteobacteria;o__Enterobacterales;f__Erwiniaceae;g__Pantoea;s__Pantoea_deleyi
MDJ-1_gene_2418	88	WP_055069600.1	498	29/66(43.94)	7.13e-07	catalase [Clostridium sp. ND2]	1347366	d__Bacteria;k__norank;p__Firmicutes;c__Clostridia;o__Clostridiales;f__Clostridiaceae;g__Clostridium;s__Clostridium_massiliamazoniense
MDJ-1_gene_2437	45	WP_120787132.1	492	36/45(80.00)	2.45e-13	aldehyde dehydrogenase family protein [Macrococcus	None	Unclassified
MDJ-1_gene_2444	82	ORY49622.1	449	27/81(33.33)	2.48e-08	hypothetical protein BCR33DRAFT_713943 [Rhizoclosmatium globosum]	329046	d__Eukaryota;k__Fungi;p__Chytridiomycota;c__Chytridiomycetes;o__Chytridiales;f__Chytriomycetaceae;g__Rhizoclosmatium;s__Rhizoclosmatium_globosum
MDJ-1_gene_2446	111	WP_063630722.1	111	108/111(97.30)	5.67e-70	divalent-cation tolerance protein CutA [Wolbachia endosymbiont of Laodelphax striatella]	2640676	d__Bacteria;k__norank;p__Proteobacteria;c__Alphaproteobacteria;o__Rickettsiales;f__Anaplasmataceae;g__Wolbachia;s__norank
MDJ-1_gene_2484	65	PBQ29265.1	276	64/65(98.46)	1.38e-35	UDP-N-acetylmuramoylalanine--D-glutamate ligase, partial [Wolbachia pipientis wAus]	2049495	d__Bacteria;k__norank;p__Proteobacteria;c__Alphaproteobacteria;o__Rickettsiales;f__Anaplasmataceae;g__Wolbachia;s__Wolbachia_pipientis
MDJ-1_gene_2486	77	OSY19256.1	647	35/73(47.95)	5.99e-09	hypothetical protein B0D75_01370 [Entomoplasmatales bacterium	None	Unclassified

MDJ-1_gene_2520	84	WP_114598922.1	433	54/67(80.60)	9.55e-26	phosphopyruvate hydratase [Microbacterium arborescens]	33883	d__Bacteria;k__norank;p__Actinobacteria;c__Actinobacteria;o__Micrococcales;f__Microbacteriaceae;g__Microbacterium;s__Microbacterium_arborescens
MDJ-1_gene_2525	112	WP_111139583.1	112	112/112(100.00)	2.69e-64	preprotein translocase subunit SecG [Pantoea sp. ARC607]	2027922	d__Bacteria;k__norank;p__Proteobacteria;c__Gammaproteobacteria;o__Enterobacterales;f__Erwiniaceae;g__Pantoea;s__Pantoea_sp._ARC607
MDJ-1_gene_2553	76	WP_120787132.1	492	55/70(78.57)	8.79e-30	aldehyde dehydrogenase family protein [Macrococcus	None	Unclassified
MDJ-1_gene_2561	85	WP_127116310.1	188	66/83(79.52)	4.29e-39	hypothetical protein [Thalassobius sp. ZQ172]	2497945	d__Bacteria;k__norank;p__Proteobacteria;c__Alphaproteobacteria;o__Rhodobacterales;f__Rhodobacteraceae;g__Thalassobius;s__Thalassobius_sp._ZQ172
MDJ-1_gene_2564	112	OWS73455.1	270	85/92(92.39)	6.68e-48	IS4 family transposase, partial [Pantoea sp. VS1]	2003658	d__Bacteria;k__norank;p__Proteobacteria;c__Gammaproteobacteria;o__Enterobacterales;f__Erwiniaceae;g__Pantoea;s__Pantoea_sp._VS1
MDJ-1_gene_2570	80	WP_127463812.1	92	80/80(100.00)	8.33e-51	type II toxin-antitoxin system RelE/ParE family toxin [Wolbachia endosymbiont of	215173	d__Bacteria;k__norank;p__Proteobacteria;c__Alphaproteobacteria;o__Rickettsiales;f__Anaplasmataceae;g__Wolbachia;s__Wolbachia_endosymbiont_of_Bemisia_tabaci
MDJ-1_gene_2571	74	WP_127463811.1	74	74/74(100.00)	1.68e-45	hypothetical protein [Wolbachia endosymbiont of Bemisia tabaci]	215173	d__Bacteria;k__norank;p__Proteobacteria;c__Alphaproteobacteria;o__Rickettsiales;f__Anaplasmataceae;g__Wolbachia;s__Wolbachia_endosymbiont_of_Bemisia_tabaci
MDJ-1_gene_2586	128	PBQ27621.1	128	128/128(100.00)	5.72e-87	Fe-S cluster assembly protein HesB [Wolbachia pipientis wAus]	2049495	d__Bacteria;k__norank;p__Proteobacteria;c__Alphaproteobacteria;o__Rickettsiales;f__Anaplasmataceae;g__Wolbachia;s__Wolbachia_pipientis
MDJ-1_gene_2593	85	YP_001967399.1	167	74/85(87.06)	7.45e-43	hypothetical protein RM_p19 (plasmid) [Rickettsia monacensis]	None	Unclassified
MDJ-1_gene_2629	74	YP_009322261.1	328	51/74(68.92)	2.96e-32	DNA primase/helicase [Salmonella phage 64795_sal3]	1813769	d__Viruses;k__norank;p__Uroviricota;c__Caudoviricetes;o__Caudovirales;f__Siphoviridae;g__norank;s__Salmonella_phage_64795_sal3
MDJ-1_gene_2639	107	EDL62366.1	558	30/69(43.48)	1.93e-11	peroxidase [Gimesia maris DSM 8797]	344747	d__Bacteria;k__norank;p__Planctomycetes;c__Planctomycetia;o__Planctomycetales;f__Planctomycetaceae;g__Gimesia;s__Gimesia_maris
MDJ-2_gene_5	250	WP_064125253.1	250	240/250(96.00)	2.22e-171	5'/3'-nucleotidase SurE [Wolbachia endosymbiont of Dactylopius coccus]	None	Unclassified
MDJ-2_gene_6	470	WP_096616995.1	477	379/477(79.45)	1.08e-265	hypothetical protein [Wolbachia pipientis]	955	d__Bacteria;k__norank;p__Proteobacteria;c__Alphaproteobacteria;o__Rickettsiales;f__Anaplasmataceae;g__Wolbachia;s__Wolbachia_pipientis
MDJ-2_gene_7	424	CAQ54831.1	424	415/424(97.88)	1.33e-287	amino acid permease family protein [Wolbachia endosymbiont of Culex quinquefasciatus Pel]	570417	d__Bacteria;k__norank;p__Proteobacteria;c__Alphaproteobacteria;o__Rickettsiales;f__Anaplasmataceae;g__Wolbachia;s__Wolbachia_endosymbiont_of_Culex_quinquefasciatus
MDJ-2_gene_8	428	WP_064085324.1	428	424/428(99.07)	3.21e-297	amino acid permease [Wolbachia endosymbiont of Dactylopius coccus]	None	Unclassified
MDJ-2_gene_9	138	PBQ27199.1	141	138/141(97.87)	6.62e-87	hypothetical protein BTO27_04030 [Wolbachia	2049495	d__Bacteria;k__norank;p__Proteobacteria;c__Alphaproteobacteria;o__Rickettsiales;f__Anaplasmataceae;g__Wolbachia;s__Wolbachia_pipientis
MDJ-2_gene_10	340	WP_017532206.1	340	339/340(99.71)	5.14e-238	aspartate-semialdehyde dehydrogenase [Wolbachia endosymbiont of Diaphorina citri]	116598	d__Bacteria;k__norank;p__Proteobacteria;c__Alphaproteobacteria;o__Rickettsiales;f__Anaplasmataceae;g__Wolbachia;s__Wolbachia_endosymbiont_of_Diaphorina_citri
MDJ-2_gene_11	359	WP_017532205.1	358	354/358(98.88)	2.34e-257	MULTISPECIES: 5-(carboxyamino)imidazole ribonucleotide synthase	2640676	d__Bacteria;k__norank;p__Proteobacteria;c__Alphaproteobacteria;o__Rickettsiales;f__Anaplasmataceae;g__Wolbachia;s__norank
MDJ-2_gene_14	281	PCG11488.1	281	272/281(96.80)	1.53e-198	rhodanese domain-containing protein [Wolbachia pipientis]	None	Unclassified
MDJ-2_gene_15	133	WP_127463814.1	133	123/130(94.62)	8.27e-78	hypothetical protein [Wolbachia endosymbiont of Bemisia tabaci]	215173	d__Bacteria;k__norank;p__Proteobacteria;c__Alphaproteobacteria;o__Rickettsiales;f__Anaplasmataceae;g__Wolbachia;s__Wolbachia_endosymbiont_of_Bemisia_tabaci
MDJ-2_gene_17	392	OJH31039.1	392	377/392(96.17)	2.42e-276	Acetylornithine aminotransferase [Wolbachia endosymbiont of Armadillidium vulgare]	77039	d__Bacteria;k__norank;p__Proteobacteria;c__Alphaproteobacteria;o__Rickettsiales;f__Anaplasmataceae;g__Wolbachia;s__Wolbachia_endosymbiont_of_Armadillidium_vulgare
MDJ-2_gene_18	425	WP_029237684.1	425	424/425(99.76)	1.40e-294	MULTISPECIES: DUF21 domain-containing protein	180837	d__Bacteria;k__norank;p__Proteobacteria;c__Alphaproteobacteria;o__Rickettsiales;f__Anaplasmataceae;g__Wolbachia;s__Wolbachia_endosymbiont_of_Nasonia_vitripennis

MDJ-2_gene_19	85	PCG11844.1	85	81/85(95.29)	8.35e-42	DUF2312 domain-containing protein [Wolbachia pipientis]	None	Unclassified
MDJ-2_gene_20	191	WP_108784635.1	191	183/190(96.32)	5.79e-122	UbiX family flavin prenyltransferase [Wolbachia endosymbiont of Bemisia tabaci]	215173	d__Bacteria;k__norank;p__Proteobacteria;c__Alphaproteobacteria;o__Rickettsiales;f__Anaplasmataceae;g__Wolbachia;s__Wolbachia_endosymbiont_of_Bemisia_tabaci
MDJ-2_gene_21	293	WP_127463833.1	295	284/293(96.93)	2.25e-202	bifunctional methylenetetrahydrofolate dehydrogenase/methenyltetrahydrofolate cyclohydrolase FolD [Wolbachia endosymbiont of Bemisia tabaci]	215173	d__Bacteria;k__norank;p__Proteobacteria;c__Alphaproteobacteria;o__Rickettsiales;f__Anaplasmataceae;g__Wolbachia;s__Wolbachia_endosymbiont_of_Bemisia_tabaci
MDJ-2_gene_22	237	EEB56140.1	243	231/237(97.47)	9.03e-134	ompA-like protein [Wolbachia endosymbiont of Culex quinquefasciatus JHB]	569881	d__Bacteria;k__norank;p__Proteobacteria;c__Alphaproteobacteria;o__Rickettsiales;f__Anaplasmataceae;g__Wolbachia;s__Wolbachia_endosymbiont_of_Culex_quinquefasciatus
MDJ-2_gene_24	819	WP_063630807.1	819	802/819(97.92)	0	leucine--tRNA ligase [Wolbachia endosymbiont of Laodelphax striatella]	368602	d__Bacteria;k__norank;p__Proteobacteria;c__Alphaproteobacteria;o__Rickettsiales;f__Anaplasmataceae;g__Wolbachia;s__Wolbachia_endosymbiont_of_Laodelphax_striatellus
MDJ-2_gene_28	398	CAQ54845.1	398	395/398(99.25)	1.27e-265	major facilitator family transporter [Wolbachia endosymbiont of Culex	570417	d__Bacteria;k__norank;p__Proteobacteria;c__Alphaproteobacteria;o__Rickettsiales;f__Anaplasmataceae;g__Wolbachia;s__Wolbachia_endosymbiont_of_Culex_quinquefasciatus
MDJ-2_gene_29	644	PBQ27212.1	644	633/643(98.44)	0	excinuclease ABC subunit B [Wolbachia pipientis wAus]	2049495	d__Bacteria;k__norank;p__Proteobacteria;c__Alphaproteobacteria;o__Rickettsiales;f__Anaplasmataceae;g__Wolbachia;s__Wolbachia_pipientis
MDJ-2_gene_32	671	WP_096560733.1	720	399/696(57.33)	6.64e-141	hypothetical protein [Wolbachia pipientis]	953	d__Bacteria;k__norank;p__Proteobacteria;c__Alphaproteobacteria;o__Rickettsiales;f__Anaplasmataceae;g__Wolbachia;s__norank
MDJ-2_gene_33	405	WP_096560729.1	405	391/405(96.54)	1.67e-285	CCA tRNA nucleotidyltransferase [Wolbachia pipientis]	953	d__Bacteria;k__norank;p__Proteobacteria;c__Alphaproteobacteria;o__Rickettsiales;f__Anaplasmataceae;g__Wolbachia;s__norank
MDJ-2_gene_34	609	PBQ28195.1	609	608/609(99.84)	0	GTP-binding protein TypA [Wolbachia pipientis wAus]	2049495	d__Bacteria;k__norank;p__Proteobacteria;c__Alphaproteobacteria;o__Rickettsiales;f__Anaplasmataceae;g__Wolbachia;s__Wolbachia_pipientis
MDJ-2_gene_35	419	AGJ99363.1	444	405/419(96.66)	2.88e-297	Glutamyl-tRNA synthetase 2 [Wolbachia endosymbiont of Drosophila simulans wNo]	1236908	d__Bacteria;k__norank;p__Proteobacteria;c__Alphaproteobacteria;o__Rickettsiales;f__Anaplasmataceae;g__Wolbachia;s__Wolbachia_endosymbiont_of_Drosophila_simulans
MDJ-2_gene_36	339	PBQ28199.1	339	336/339(99.12)	1.52e-234	DNA polymerase III subunit delta [Wolbachia pipientis wAus]	2049495	d__Bacteria;k__norank;p__Proteobacteria;c__Alphaproteobacteria;o__Rickettsiales;f__Anaplasmataceae;g__Wolbachia;s__Wolbachia_pipientis
MDJ-2_gene_37	63	AGJ99361.1	60	60/60(100.00)	5.82e-34	hypothetical protein wNo_09850 [Wolbachia endosymbiont of Drosophila simulans wNo]	1236908	d__Bacteria;k__norank;p__Proteobacteria;c__Alphaproteobacteria;o__Rickettsiales;f__Anaplasmataceae;g__Wolbachia;s__Wolbachia_endosymbiont_of_Drosophila_simulans
MDJ-2_gene_40	115	PBQ28202.1	115	115/115(100.00)	5.34e-79	hypothetical protein BTO27_02130 [Wolbachia	2049495	d__Bacteria;k__norank;p__Proteobacteria;c__Alphaproteobacteria;o__Rickettsiales;f__Anaplasmataceae;g__Wolbachia;s__Wolbachia_pipientis
MDJ-2_gene_41	288	AGJ99358.1	288	282/288(97.92)	3.20e-199	4-diphosphocytidyl-2-C-methyl-D-erythritol kinase [Wolbachia endosymbiont of Drosophila simulans wNo]	1236908	d__Bacteria;k__norank;p__Proteobacteria;c__Alphaproteobacteria;o__Rickettsiales;f__Anaplasmataceae;g__Wolbachia;s__Wolbachia_endosymbiont_of_Drosophila_simulans
MDJ-2_gene_42	1089	WP_064085305.1	1089	1060/1089(97.34)	0	DNA helicase UvrD [Wolbachia endosymbiont of Dactylopius coccus]	None	Unclassified
MDJ-2_gene_44	842	WP_064085843.1	843	810/843(96.09)	0	type IV secretion system protein [Wolbachia endosymbiont of Dactylopius coccus]	None	Unclassified

MDJ-2_gene_45	865	WP_064085842.1	865	781/872(89.56)	0	conjugal transfer protein TraH [Wolbachia endosymbiont of Dactylopius coccus]	None	Unclassified
MDJ-2_gene_46	801	WP_010402460.1	801	799/801(99.75)	0	VirB4 family type IV secretion/conjugal transfer ATPase [Wolbachia endosymbiont of Nasonia	180837	d__Bacteria;k__norank;p__Proteobacteria;c__Alphaproteobacteria;o__Rickettsiales;f__Anaplasmataceae;g__Wolbachia;s__Wolbachia_endosymbiont_of_Nasonia_vitripennis
MDJ-2_gene_47	98	WP_108784517.1	98	98/98(100.00)	2.28e-63	type IV secretion system protein VirB3 [Wolbachia endosymbiont of Bemisia tabaci]	215173	d__Bacteria;k__norank;p__Proteobacteria;c__Alphaproteobacteria;o__Rickettsiales;f__Anaplasmataceae;g__Wolbachia;s__Wolbachia_endosymbiont_of_Bemisia_tabaci
MDJ-2_gene_48	525	WP_108784516.1	525	521/525(99.24)	0	lysine--tRNA ligase [Wolbachia endosymbiont of Bemisia tabaci]	215173	d__Bacteria;k__norank;p__Proteobacteria;c__Alphaproteobacteria;o__Rickettsiales;f__Anaplasmataceae;g__Wolbachia;s__Wolbachia_endosymbiont_of_Bemisia_tabaci
MDJ-2_gene_49	87	WP_127463908.1	226	84/86(97.67)	8.50e-50	hypothetical protein [Wolbachia endosymbiont of Bemisia tabaci]	215173	d__Bacteria;k__norank;p__Proteobacteria;c__Alphaproteobacteria;o__Rickettsiales;f__Anaplasmataceae;g__Wolbachia;s__Wolbachia_endosymbiont_of_Bemisia_tabaci
MDJ-2_gene_51	190	WP_064085908.1	190	189/190(99.47)	3.68e-138	thioredoxin family protein [Wolbachia endosymbiont of Dactylopius coccus]	None	Unclassified
MDJ-2_gene_52	572	PBQ28213.1	572	568/572(99.30)	0	potassium transporter [Wolbachia pipientis wAus]	2049495	d__Bacteria;k__norank;p__Proteobacteria;c__Alphaproteobacteria;o__Rickettsiales;f__Anaplasmataceae;g__Wolbachia;s__Wolbachia_pipientis
MDJ-2_gene_53	386	PBQ28214.1	386	372/386(96.37)	2.42e-257	aspartate kinase [Wolbachia pipientis wAus]	2049495	d__Bacteria;k__norank;p__Proteobacteria;c__Alphaproteobacteria;o__Rickettsiales;f__Anaplasmataceae;g__Wolbachia;s__Wolbachia_pipientis
MDJ-2_gene_54	359	PBQ28215.1	359	352/359(98.05)	8.13e-256	DNA replication/repair protein RecF [Wolbachia pipientis wAus]	2049495	d__Bacteria;k__norank;p__Proteobacteria;c__Alphaproteobacteria;o__Rickettsiales;f__Anaplasmataceae;g__Wolbachia;s__Wolbachia_pipientis
MDJ-2_gene_55	159	PCG11751.1	155	154/155(99.35)	9.24e-107	thioredoxin-dependent thiol peroxidase [Wolbachia pipientis]	None	Unclassified
MDJ-2_gene_56	308	PCG11743.1	308	301/308(97.73)	3.13e-216	glutathione synthase [Wolbachia pipientis]	None	Unclassified
MDJ-2_gene_57	340	B3CM87.1	340	330/340(97.06)	6.00e-237	RecName: Full=UDP-N-acetylglucosamine--N-acetylmuramyl-(pentapeptide) pyrophosphoryl-undecaprenol N-acetylglucosamine transferase; AltName: Full=Undecaprenyl-PP-MurNAc-pentapeptide-UDPGlcNAc GlcNAc transferase	570417	d__Bacteria;k__norank;p__Proteobacteria;c__Alphaproteobacteria;o__Rickettsiales;f__Anaplasmataceae;g__Wolbachia;s__Wolbachia_endosymbiont_of_Culex_quinquefasciatus
MDJ-2_gene_58	119	WP_017531872.1	119	116/119(97.48)	1.45e-69	hypothetical protein [Wolbachia endosymbiont of Diaphorina citri]	116598	d__Bacteria;k__norank;p__Proteobacteria;c__Alphaproteobacteria;o__Rickettsiales;f__Anaplasmataceae;g__Wolbachia;s__Wolbachia_endosymbiont_of_Diaphorina_citri
MDJ-2_gene_59	457	PCG11741.1	461	436/455(95.82)	0	dihydrolipoyl dehydrogenase [Wolbachia pipientis]	None	Unclassified
MDJ-2_gene_61	425	PCG11247.1	425	422/425(99.29)	3.82e-308	adenylosuccinate synthase [Wolbachia pipientis]	None	Unclassified
MDJ-2_gene_62	724	CAQ54147.1	709	555/728(76.24)	5.48e-311	hypothetical protein WP0038 [Wolbachia endosymbiont of Culex quinquefasciatus Pel]	570417	d__Bacteria;k__norank;p__Proteobacteria;c__Alphaproteobacteria;o__Rickettsiales;f__Anaplasmataceae;g__Wolbachia;s__Wolbachia_endosymbiont_of_Culex_quinquefasciatus
MDJ-2_gene_63	87	PBQ25492.1	87	87/87(100.00)	9.96e-53	translation initiation factor IF-1 [Wolbachia pipientis wAus]	2049495	d__Bacteria;k__norank;p__Proteobacteria;c__Alphaproteobacteria;o__Rickettsiales;f__Anaplasmataceae;g__Wolbachia;s__Wolbachia_pipientis
MDJ-2_gene_64	196	PBQ25491.1	196	195/196(99.49)	6.54e-135	septum formation protein Maf [Wolbachia pipientis wAus]	2049495	d__Bacteria;k__norank;p__Proteobacteria;c__Alphaproteobacteria;o__Rickettsiales;f__Anaplasmataceae;g__Wolbachia;s__Wolbachia_pipientis

MDJ-2_gene_65	754	WP_064085505.1	755	708/758(93.40)	0	hypothetical protein [Wolbachia endosymbiont of Dactylopius coccus]	None	Unclassified
MDJ-2_gene_66	445	CAQ54142.1	445	429/444(96.62)	1.07e-283	sodium/alanine symporter family protein [Wolbachia endosymbiont of Culex quinquefasciatus Pel]	570417	d__Bacteria;k__norank;p__Proteobacteria;c__Alphaproteobacteria;o__Rickettsiales;f__Anaplasmataceae;g__Wolbachia;s__Wolbachia_endosymbiont_of_Culex_quinquefasciatus
MDJ-2_gene_67	395	WP_108784649.1	299	249/297(83.84)	2.73e-175	hypothetical protein [Wolbachia endosymbiont of Bemisia tabaci]	215173	d__Bacteria;k__norank;p__Proteobacteria;c__Alphaproteobacteria;o__Rickettsiales;f__Anaplasmataceae;g__Wolbachia;s__Wolbachia_endosymbiont_of_Bemisia_tabaci
MDJ-2_gene_68	150	WP_029237594.1	167	149/150(99.33)	3.01e-110	hypothetical protein [Wolbachia endosymbiont of Nasonia vitripennis]	180837	d__Bacteria;k__norank;p__Proteobacteria;c__Alphaproteobacteria;o__Rickettsiales;f__Anaplasmataceae;g__Wolbachia;s__Wolbachia_endosymbiont_of_Nasonia_vitripennis
MDJ-2_gene_70	1029	AGJ99479.1	1028	963/1032(93.31)	0	Phosphoribosylformylglycinamidine synthase II domain protein [Wolbachia endosymbiont of Drosophila simulans wNo]	1236908	d__Bacteria;k__norank;p__Proteobacteria;c__Alphaproteobacteria;o__Rickettsiales;f__Anaplasmataceae;g__Wolbachia;s__Wolbachia_endosymbiont_of_Drosophila_simulans
MDJ-2_gene_71	372	PBQ27666.1	372	371/372(99.73)	1.84e-238	molecular chaperone DnaJ [Wolbachia pipientis wAus]	2049495	d__Bacteria;k__norank;p__Proteobacteria;c__Alphaproteobacteria;o__Rickettsiales;f__Anaplasmataceae;g__Wolbachia;s__Wolbachia_pipientis
MDJ-2_gene_72	544	WP_010401701.1	544	533/544(97.98)	0	ribonuclease J [Wolbachia endosymbiont of Nasonia vitripennis]	180837	d__Bacteria;k__norank;p__Proteobacteria;c__Alphaproteobacteria;o__Rickettsiales;f__Anaplasmataceae;g__Wolbachia;s__Wolbachia_endosymbiont_of_Nasonia_vitripennis
MDJ-2_gene_73	439	CCE77080.1	439	433/439(98.63)	5.37e-230	Periplasmic component of the Tol-Pal transport system [Wolbachia pipientis wAlbB]	1116230	d__Bacteria;k__norank;p__Proteobacteria;c__Alphaproteobacteria;o__Rickettsiales;f__Anaplasmataceae;g__Wolbachia;s__Wolbachia_pipientis
MDJ-2_gene_74	117	CAQ54334.1	117	115/117(98.29)	4.04e-75	glutamyl-tRNA(Gln) amidotransferase, C subunit [Wolbachia endosymbiont of Culex quinquefasciatus Pel]	570417	d__Bacteria;k__norank;p__Proteobacteria;c__Alphaproteobacteria;o__Rickettsiales;f__Anaplasmataceae;g__Wolbachia;s__Wolbachia_endosymbiont_of_Culex_quinquefasciatus
MDJ-2_gene_75	306	WP_064085819.1	306	294/306(96.08)	2.95e-204	ribose-phosphate diphosphokinase [Wolbachia endosymbiont of Dactylopius	None	Unclassified
MDJ-2_gene_76	360	CAQ54336.1	666	292/352(82.95)	3.29e-131	AAA ATPase family protein [Wolbachia endosymbiont of Culex quinquefasciatus Pel]	570417	d__Bacteria;k__norank;p__Proteobacteria;c__Alphaproteobacteria;o__Rickettsiales;f__Anaplasmataceae;g__Wolbachia;s__Wolbachia_endosymbiont_of_Culex_quinquefasciatus
MDJ-2_gene_77	282	WP_127463848.1	282	276/282(97.87)	3.62e-202	FAD-dependent thymidylate synthase [Wolbachia endosymbiont of Bemisia tabaci]	215173	d__Bacteria;k__norank;p__Proteobacteria;c__Alphaproteobacteria;o__Rickettsiales;f__Anaplasmataceae;g__Wolbachia;s__Wolbachia_endosymbiont_of_Bemisia_tabaci
MDJ-2_gene_78	425	WP_127463847.1	425	421/425(99.06)	6.23e-298	UDP-N-acetylglucosamine 1-carboxyvinyltransferase [Wolbachia endosymbiont of	215173	d__Bacteria;k__norank;p__Proteobacteria;c__Alphaproteobacteria;o__Rickettsiales;f__Anaplasmataceae;g__Wolbachia;s__Wolbachia_endosymbiont_of_Bemisia_tabaci
MDJ-2_gene_79	423	WP_017532239.1	423	408/423(96.45)	4.19e-295	beta-ketoacyl-[acyl-carrier-protein] synthase II [Wolbachia endosymbiont of Diaphorina citri]	116598	d__Bacteria;k__norank;p__Proteobacteria;c__Alphaproteobacteria;o__Rickettsiales;f__Anaplasmataceae;g__Wolbachia;s__Wolbachia_endosymbiont_of_Diaphorina_citri
MDJ-2_gene_80	86	WP_017532238.1	86	81/86(94.19)	9.68e-46	MULTISPECIES: acyl carrier protein [Wolbachia]	2640676	d__Bacteria;k__norank;p__Proteobacteria;c__Alphaproteobacteria;o__Rickettsiales;f__Anaplasmataceae;g__Wolbachia;s__norank
MDJ-2_gene_81	260	WP_017532237.1	260	258/260(99.23)	7.42e-175	MULTISPECIES: polyprenyl synthetase family protein [Wolbachia]	2640676	d__Bacteria;k__norank;p__Proteobacteria;c__Alphaproteobacteria;o__Rickettsiales;f__Anaplasmataceae;g__Wolbachia;s__norank
MDJ-2_gene_82	289	WP_010406447.1	290	276/292(94.52)	1.32e-185	peptidyl-prolyl cis-trans isomerase [Wolbachia endosymbiont of Nasonia	2640676	d__Bacteria;k__norank;p__Proteobacteria;c__Alphaproteobacteria;o__Rickettsiales;f__Anaplasmataceae;g__Wolbachia;s__norank

MDJ-2_gene_83	483	WP_108784123.1	483	478/483(98.96)	0	ATP-dependent protease ATPase subunit HslU [Wolbachia endosymbiont of Bemisia tabaci]	215173	d__Bacteria;k__norank;p__Proteobacteria;c__Alphaproteobacteria;o__Rickettsiales;f__Anaplasmataceae;g__Wolbachia;s__Wolbachia_endosymbiont_of_Bemisia_tabaci
MDJ-2_gene_84	184	PCG11837.1	184	181/184(98.37)	3.98e-121	ATP-dependent protease subunit HslV [Wolbachia pipientis]	None	Unclassified
MDJ-2_gene_85	386	WP_108784122.1	384	354/386(91.71)	3.72e-256	glutamate--cysteine ligase [Wolbachia endosymbiont of Bemisia tabaci]	215173	d__Bacteria;k__norank;p__Proteobacteria;c__Alphaproteobacteria;o__Rickettsiales;f__Anaplasmataceae;g__Wolbachia;s__Wolbachia_endosymbiont_of_Bemisia_tabaci
MDJ-2_gene_86	266	PBQ29208.1	266	248/266(93.23)	5.83e-175	hypothetical protein BTO27_00835 [Wolbachia	2049495	d__Bacteria;k__norank;p__Proteobacteria;c__Alphaproteobacteria;o__Rickettsiales;f__Anaplasmataceae;g__Wolbachia;s__Wolbachia_pipientis
MDJ-2_gene_87	317	PBQ29207.1	317	313/317(98.74)	4.19e-230	ferrochelatase [Wolbachia pipientis wAus]	2049495	d__Bacteria;k__norank;p__Proteobacteria;c__Alphaproteobacteria;o__Rickettsiales;f__Anaplasmataceae;g__Wolbachia;s__Wolbachia_pipientis
MDJ-2_gene_90	237	WP_064124954.1	393	222/239(92.89)	5.82e-158	phage tail protein [Wolbachia endosymbiont of Dactylopius coccus]	None	Unclassified
MDJ-2_gene_91	168	WP_019236469.1	168	165/168(98.21)	7.06e-114	phage major tail tube protein [Wolbachia pipientis]	955	d__Bacteria;k__norank;p__Proteobacteria;c__Alphaproteobacteria;o__Rickettsiales;f__Anaplasmataceae;g__Wolbachia;s__Wolbachia_pipientis
MDJ-2_gene_92	84	WP_114517669.1	84	78/84(92.86)	1.98e-44	phage tail assembly protein [Wolbachia endosymbiont of Cylisticus convexus]	118728	d__Bacteria;k__norank;p__Proteobacteria;c__Alphaproteobacteria;o__Rickettsiales;f__Anaplasmataceae;g__Wolbachia;s__Wolbachia_endosymbiont_of_Cylisticus_convexus
MDJ-2_gene_93	764	WP_019236472.1	759	652/768(84.90)	0	phage tail tape measure protein [Wolbachia pipientis]	955	d__Bacteria;k__norank;p__Proteobacteria;c__Alphaproteobacteria;o__Rickettsiales;f__Anaplasmataceae;g__Wolbachia;s__Wolbachia_pipientis
MDJ-2_gene_94	114	OJH31508.1	112	99/115(86.09)	8.03e-62	Phage P2 GpU [Wolbachia endosymbiont of Armadillidium vulgare]	77039	d__Bacteria;k__norank;p__Proteobacteria;c__Alphaproteobacteria;o__Rickettsiales;f__Anaplasmataceae;g__Wolbachia;s__Wolbachia_endosymbiont_of_Armadillidium_vulgare
MDJ-2_gene_96	314	WP_065094822.1	314	275/314(87.58)	5.01e-200	MULTISPECIES: phage tail protein [Wolbachia]	2640676	d__Bacteria;k__norank;p__Proteobacteria;c__Alphaproteobacteria;o__Rickettsiales;f__Anaplasmataceae;g__Wolbachia;s__norank
MDJ-2_gene_97	172	CAQ55444.1	172	163/172(94.77)	1.88e-95	ankyrin repeat domain protein [Wolbachia endosymbiont of Culex quinquefasciatus Pel]	570417	d__Bacteria;k__norank;p__Proteobacteria;c__Alphaproteobacteria;o__Rickettsiales;f__Anaplasmataceae;g__Wolbachia;s__Wolbachia_endosymbiont_of_Culex_quinquefasciatus
MDJ-2_gene_99	651	RDD35489.1	586	424/587(72.23)	0	Group II intron-encoded protein ltrA [Wolbachia endosymbiont of Cylisticus convexus]	118728	d__Bacteria;k__norank;p__Proteobacteria;c__Alphaproteobacteria;o__Rickettsiales;f__Anaplasmataceae;g__Wolbachia;s__Wolbachia_endosymbiont_of_Cylisticus_convexus
MDJ-2_gene_100	160	WP_019236478.1	170	115/127(90.55)	9.81e-76	phospholipase D family protein [Wolbachia pipientis]	None	Unclassified
MDJ-2_gene_101	309	WP_010082204.1	304	272/309(88.03)	1.75e-194	hypothetical protein [Wolbachia endosymbiont of Drosophila simulans]	953	d__Bacteria;k__norank;p__Proteobacteria;c__Alphaproteobacteria;o__Rickettsiales;f__Anaplasmataceae;g__Wolbachia;s__norank
MDJ-2_gene_102	110	PBQ26575.1	110	107/110(97.27)	5.26e-61	hypothetical protein BTO27_04390 [Wolbachia	2049495	d__Bacteria;k__norank;p__Proteobacteria;c__Alphaproteobacteria;o__Rickettsiales;f__Anaplasmataceae;g__Wolbachia;s__Wolbachia_pipientis
MDJ-2_gene_104	234	AGJ99055.1	234	225/234(96.15)	3.05e-161	DNA polymerase III, epsilon subunit [Wolbachia endosymbiont of Drosophila	1236908	d__Bacteria;k__norank;p__Proteobacteria;c__Alphaproteobacteria;o__Rickettsiales;f__Anaplasmataceae;g__Wolbachia;s__Wolbachia_endosymbiont_of_Drosophila_simulans
MDJ-2_gene_105	199	PBQ29121.1	199	184/199(92.46)	8.13e-126	photosynthetic protein synthase II [Wolbachia pipientis wAus]	2049495	d__Bacteria;k__norank;p__Proteobacteria;c__Alphaproteobacteria;o__Rickettsiales;f__Anaplasmataceae;g__Wolbachia;s__Wolbachia_pipientis
MDJ-2_gene_106	235	EEB56463.1	233	221/231(95.67)	2.22e-154	ribonuclease III [Wolbachia endosymbiont of Culex quinquefasciatus JHB]	569881	d__Bacteria;k__norank;p__Proteobacteria;c__Alphaproteobacteria;o__Rickettsiales;f__Anaplasmataceae;g__Wolbachia;s__Wolbachia_endosymbiont_of_Culex_quinquefasciatus

MDJ-2_gene_107	355	WP_010404352.1	355	352/355(99.15)	2.60e-250	quinone-dependent dihydroorotate dehydrogenase [Wolbachia endosymbiont of Nasonia vitripennis]	180837	d__Bacteria;k__norank;p__Proteobacteria;c__Alphaproteobacteria;o__Rickettsiales;f__Anaplasmataceae;g__Wolbachia;s__Wolbachia_endosymbiont_of_Nasonia_vitripennis
MDJ-2_gene_108	285	PCG11372.1	265	265/265(100.00)	3.06e-193	hypothetical protein CO539_03585 [Wolbachia	None	Unclassified
MDJ-2_gene_109	298	WP_108784145.1	298	294/298(98.66)	1.00e-207	class I fructose-bisphosphate aldolase [Wolbachia endosymbiont of Bemisia tabaci]	215173	d__Bacteria;k__norank;p__Proteobacteria;c__Alphaproteobacteria;o__Rickettsiales;f__Anaplasmataceae;g__Wolbachia;s__Wolbachia_endosymbiont_of_Bemisia_tabaci
MDJ-2_gene_110	328	WP_064085707.1	332	301/332(90.66)	1.18e-206	hypothetical protein [Wolbachia endosymbiont of Dactylopius coccus]	None	Unclassified
MDJ-2_gene_111	759	WP_010404321.1	759	756/759(99.60)	0	AAA family ATPase [Wolbachia endosymbiont of Nasonia vitripennis]	180837	d__Bacteria;k__norank;p__Proteobacteria;c__Alphaproteobacteria;o__Rickettsiales;f__Anaplasmataceae;g__Wolbachia;s__Wolbachia_endosymbiont_of_Nasonia_vitripennis
MDJ-2_gene_112	408	PBQ29126.1	408	406/408(99.51)	2.46e-284	DEAD/DEAH box helicase [Wolbachia pipientis wAus]	2049495	d__Bacteria;k__norank;p__Proteobacteria;c__Alphaproteobacteria;o__Rickettsiales;f__Anaplasmataceae;g__Wolbachia;s__Wolbachia_pipientis
MDJ-2_gene_113	83	PBQ29127.1	83	82/83(98.80)	4.33e-52	cold-shock protein [Wolbachia pipientis wAus]	2049495	d__Bacteria;k__norank;p__Proteobacteria;c__Alphaproteobacteria;o__Rickettsiales;f__Anaplasmataceae;g__Wolbachia;s__Wolbachia_pipientis
MDJ-2_gene_116	1150	WP_019078655.1	1151	1079/1154(93.50)	0	MULTISPECIES: hypothetical protein [Wolbachia]	2640676	d__Bacteria;k__norank;p__Proteobacteria;c__Alphaproteobacteria;o__Rickettsiales;f__Anaplasmataceae;g__Wolbachia;s__norank
MDJ-2_gene_117	292	WP_063630795.1	323	287/292(98.29)	1.17e-183	hypothetical protein [Wolbachia endosymbiont of Laodelphax striatella]	368602	d__Bacteria;k__norank;p__Proteobacteria;c__Alphaproteobacteria;o__Rickettsiales;f__Anaplasmataceae;g__Wolbachia;s__Wolbachia_endosymbiont_of_Laodelphax_striatellus
MDJ-2_gene_118	654	CAQ54874.1	654	631/654(96.48)	0	primosomal protein N [Wolbachia endosymbiont of Culex quinquefasciatus Pel]	570417	d__Bacteria;k__norank;p__Proteobacteria;c__Alphaproteobacteria;o__Rickettsiales;f__Anaplasmataceae;g__Wolbachia;s__Wolbachia_endosymbiont_of_Culex_quinquefasciatus
MDJ-2_gene_119	272	WP_127463852.1	266	188/265(70.94)	5.77e-85	ankyrin repeat domain-containing protein [Wolbachia endosymbiont of Bemisia tabaci]	215173	d__Bacteria;k__norank;p__Proteobacteria;c__Alphaproteobacteria;o__Rickettsiales;f__Anaplasmataceae;g__Wolbachia;s__Wolbachia_endosymbiont_of_Bemisia_tabaci
MDJ-2_gene_122	325	PBQ29194.1	316	306/316(96.84)	4.84e-215	tRNA dihydrouridine synthase DusB [Wolbachia pipientis	2049495	d__Bacteria;k__norank;p__Proteobacteria;c__Alphaproteobacteria;o__Rickettsiales;f__Anaplasmataceae;g__Wolbachia;s__Wolbachia_pipientis
MDJ-2_gene_123	452	WP_063630637.1	384	268/382(70.16)	8.40e-179	hypothetical protein [Wolbachia endosymbiont of Laodelphax striatella]	None	Unclassified
MDJ-2_gene_124	434	CQD08089.1	434	417/434(96.08)	7.27e-297	seryl-tRNA synthetase [Wolbachia endosymbiont wPip_Mol of Culex molestus]	1402163	d__Bacteria;k__norank;p__Proteobacteria;c__Alphaproteobacteria;o__Rickettsiales;f__Anaplasmataceae;g__Wolbachia;s__Wolbachia_endosymbiont_of_Culex_molestus
MDJ-2_gene_126	217	WP_063631066.1	325	193/207(93.24)	2.16e-108	hypothetical protein [Wolbachia endosymbiont of Laodelphax striatella]	368602	d__Bacteria;k__norank;p__Proteobacteria;c__Alphaproteobacteria;o__Rickettsiales;f__Anaplasmataceae;g__Wolbachia;s__Wolbachia_endosymbiont_of_Laodelphax_striatellus
MDJ-2_gene_127	180	PBQ28265.1	180	176/180(97.78)	2.59e-115	CDP-diacylglycerol--glycerol-3-phosphate 3-phosphatidyltransferase [Wolbachia pipientis wAus]	2049495	d__Bacteria;k__norank;p__Proteobacteria;c__Alphaproteobacteria;o__Rickettsiales;f__Anaplasmataceae;g__Wolbachia;s__Wolbachia_pipientis
MDJ-2_gene_128	440	WP_096560671.1	439	423/440(96.14)	1.05e-301	malate dehydrogenase [Wolbachia pipientis]	955	d__Bacteria;k__norank;p__Proteobacteria;c__Alphaproteobacteria;o__Rickettsiales;f__Anaplasmataceae;g__Wolbachia;s__Wolbachia_pipientis

MDJ-2_gene_131	344	WP_029237664.1	360	335/344(97.38)	2.16e-229	efflux RND transporter periplasmic adaptor subunit [Wolbachia endosymbiont of Nasonia vitripennis]	180837	d__Bacteria;k__norank;p__Proteobacteria;c__Alphaproteobacteria;o__Rickettsiales;f__Anaplasmataceae;g__Wolbachia;s__Wolbachia_endosymbiont_of_Nasonia_vitripennis
MDJ-2_gene_133	453	WP_019236931.1	456	422/454(92.95)	8.06e-309	MULTISPECIES: hypothetical protein [Wolbachia]	953	d__Bacteria;k__norank;p__Proteobacteria;c__Alphaproteobacteria;o__Rickettsiales;f__Anaplasmataceae;g__Wolbachia;s__norank
MDJ-2_gene_134	889	WP_127464133.1	889	849/889(95.50)	0	pyruvate, phosphate dikinase [Wolbachia endosymbiont of Bemisia tabaci]	None	Unclassified
MDJ-2_gene_139	397	WP_096617147.1	402	159/428(37.15)	3.54e-69	hypothetical protein [Wolbachia pipientis]	955	d__Bacteria;k__norank;p__Proteobacteria;c__Alphaproteobacteria;o__Rickettsiales;f__Anaplasmataceae;g__Wolbachia;s__Wolbachia_pipientis
MDJ-2_gene_143	96	PBQ25783.1	96	96/96(100.00)	8.33e-65	hypothetical protein BTO27_05125 [Wolbachia	2049495	d__Bacteria;k__norank;p__Proteobacteria;c__Alphaproteobacteria;o__Rickettsiales;f__Anaplasmataceae;g__Wolbachia;s__Wolbachia_pipientis
MDJ-2_gene_144	398	WP_010402830.1	398	395/398(99.25)	3.15e-291	succinyl-diaminopimelate desuccinylase [Wolbachia endosymbiont of Nasonia vitripennis]	180837	d__Bacteria;k__norank;p__Proteobacteria;c__Alphaproteobacteria;o__Rickettsiales;f__Anaplasmataceae;g__Wolbachia;s__Wolbachia_endosymbiont_of_Nasonia_vitripennis
MDJ-2_gene_145	422	WP_010402832.1	421	414/422(98.10)	4.54e-296	iron-containing alcohol dehydrogenase [Wolbachia endosymbiont of Nasonia vitripennis]	180837	d__Bacteria;k__norank;p__Proteobacteria;c__Alphaproteobacteria;o__Rickettsiales;f__Anaplasmataceae;g__Wolbachia;s__Wolbachia_endosymbiont_of_Nasonia_vitripennis
MDJ-2_gene_146	433	KDB19457.1	430	409/430(95.12)	1.11e-292	adenylosuccinate lyase [Wolbachia endosymbiont of Glossina morsitans morsitans]	1150948	d__Bacteria;k__norank;p__Proteobacteria;c__Alphaproteobacteria;o__Rickettsiales;f__Anaplasmataceae;g__Wolbachia;s__Wolbachia_endosymbiont_of_Glossina_morsitans_morsitans
MDJ-2_gene_149	732	WP_096616554.1	1109	721/732(98.50)	0	DNA polymerase III subunit alpha [Wolbachia pipientis]	955	d__Bacteria;k__norank;p__Proteobacteria;c__Alphaproteobacteria;o__Rickettsiales;f__Anaplasmataceae;g__Wolbachia;s__Wolbachia_pipientis
MDJ-2_gene_150	458	WP_010401888.1	458	455/458(99.34)	0	MULTISPECIES: PleD family two-component system response regulator [Wolbachia]	180837	d__Bacteria;k__norank;p__Proteobacteria;c__Alphaproteobacteria;o__Rickettsiales;f__Anaplasmataceae;g__Wolbachia;s__Wolbachia_endosymbiont_of_Nasonia_vitripennis
MDJ-2_gene_151	96	WP_010401887.1	96	96/96(100.00)	6.37e-60	hypothetical protein [Wolbachia endosymbiont of Nasonia vitripennis]	2640676	d__Bacteria;k__norank;p__Proteobacteria;c__Alphaproteobacteria;o__Rickettsiales;f__Anaplasmataceae;g__Wolbachia;s__norank
MDJ-2_gene_152	1556	WP_010401886.1	1556	1525/1556(98.01)	0	NAD-glutamate dehydrogenase [Wolbachia endosymbiont of Nasonia vitripennis]	180837	d__Bacteria;k__norank;p__Proteobacteria;c__Alphaproteobacteria;o__Rickettsiales;f__Anaplasmataceae;g__Wolbachia;s__Wolbachia_endosymbiont_of_Nasonia_vitripennis
MDJ-2_gene_153	853	WP_010401885.1	853	842/853(98.71)	0	ATP-dependent chaperone ClpB [Wolbachia endosymbiont of Nasonia vitripennis]	180837	d__Bacteria;k__norank;p__Proteobacteria;c__Alphaproteobacteria;o__Rickettsiales;f__Anaplasmataceae;g__Wolbachia;s__Wolbachia_endosymbiont_of_Nasonia_vitripennis
MDJ-2_gene_155	84	PCG11724.1	84	84/84(100.00)	8.02e-53	hypothetical protein CO539_01495 [Wolbachia	None	Unclassified
MDJ-2_gene_156	57	WP_083810074.1	78	57/57(100.00)	8.22e-40	hypothetical protein [Wolbachia endosymbiont of Nasonia vitripennis]	None	Unclassified
MDJ-2_gene_158	271	WP_029237631.1	271	271/271(100.00)	1.53e-193	bifunctional DNA-formamidopyrimidine glycosylase/DNA-(apurinic or apyrimidinic site) lyase [Wolbachia endosymbiont of Nasonia vitripennis]	2640676	d__Bacteria;k__norank;p__Proteobacteria;c__Alphaproteobacteria;o__Rickettsiales;f__Anaplasmataceae;g__Wolbachia;s__norank

SC-2_gene_3542	106	OUM58834.1	228	54/106(50.94)	1.60e-23	hypothetical protein PIROE2DRAFT_26121, partial [Piromyces sp. E2]	73868	d__Eukaryota;k__Fungi;p__Chytridiomycota;c__Neocallimastigomycetes;o__Neocallimastigales;f__Neocallimastigaceae;g__Piromyces;s__Piromyces_sp._E2
SC-2_gene_3592	159	RYA67263.1	108	56/108(51.85)	9.23e-32	hypothetical protein DD592_27655 [Enterobacter cloacae complex sp. 2DZ2F20B]	2511993	d__Bacteria;k__norank;p__Proteobacteria;c__Gammaproteobacteria;o__Enterobacterales;f__Enterobacteriaceae;g__Enterobacter;s__Enterobacter_cloacae_complex_sp._2DZ2F20B
SC-2_gene_3606	66	PWS22544.1	96	39/50(78.00)	5.40e-19	phosphoglycerate kinase, partial [Enterococcus faecium]	1352	d__Bacteria;k__norank;p__Firmicutes;c__Bacilli;o__Lactobacillales;f__Enterococcaceae;g__Enterococcus;s__Enterococcus_faecium
SC-2_gene_3615	90	ORX92154.1	1289	40/77(51.95)	5.65e-17	hypothetical protein K493DRAFT_226350 [Basidiobolus meristosporus CBS	1314790	d__Eukaryota;k__Fungi;p__Zoopagomycota;c__Basidiobolomycetes;o__Basidiobolales;f__Basidiobolaceae;g__Basidiobolus;s__Basidiobolus_meristosporus
SC-2_gene_3702	111	PBQ29198.1	108	108/108(100.00)	1.86e-72	MerR family transcriptional regulator [Wolbachia pipientis	2049495	d__Bacteria;k__norank;p__Proteobacteria;c__Alphaproteobacteria;o__Rickettsiales;f__Anaplasmataceae;g__Wolbachia;s__Wolbachia_pipientis
SC-2_gene_3744	111	RUM28817.1	108	30/55(54.55)	1.03e-10	hypothetical protein DSY42_07730 [Aquifex sp.]	313370	d__Bacteria;k__norank;p__Aquificae;c__Aquificae;o__Aquificales;f__Aquificaceae;g__Aquifex;s__Aquifex_sp.
SC-2_gene_3746	201	WP_064086031.1	201	199/201(99.00)	4.75e-135	DUF1013 domain-containing protein [Wolbachia endosymbiont of Dactylopius coccus]	None	Unclassified
SC-2_gene_3748	90	CSA68231.1	138	56/90(62.22)	1.05e-28	Uncharacterised protein [Vibrio cholerae]	666	d__Bacteria;k__norank;p__Proteobacteria;c__Gammaproteobacteria;o__Vibrionales;f__Vibrionaceae;g__Vibrio;s__Vibrio_cholerae
SC-2_gene_3803	62	PXF62285.1	265	27/56(48.21)	3.85e-07	hypothetical protein DL796_12355, partial [Kangiella	796379	d__Bacteria;k__norank;p__Proteobacteria;c__Gammaproteobacteria;o__Oceanospirillales;f__Kangiellaceae;g__Kangiella;s__Kangiella_spongicola
SC-2_gene_3876	149	WP_065094948.1	360	135/145(93.10)	1.14e-84	MULTISPECIES: hypothetical protein [Wolbachia]	2640676	d__Bacteria;k__norank;p__Proteobacteria;c__Alphaproteobacteria;o__Rickettsiales;f__Anaplasmataceae;g__Wolbachia;s__norank
SC-2_gene_3943	215	WP_064085588.1	637	213/215(99.07)	1.97e-144	DNA helicase II [Wolbachia endosymbiont of Dactylopius coccus]	None	Unclassified
SC-2_gene_3959	147	RYE22780.1	229	65/154(42.21)	6.91e-30	hypothetical protein EOP45_07755	2021370	d__Bacteria;k__norank;p__Bacteroidetes;c__Sphingobacteriia;o__Sphingobacteriales;f__Sphingobacteriaceae;g__norank;s__Sphingobacteriaceae_bacterium
SC-2_gene_3971	90	WP_120787132.1	492	61/81(75.31)	4.27e-33	aldehyde dehydrogenase family protein [Macrococcus	None	Unclassified
SC-2_gene_3991	133	RUP46318.1	260	33/75(44.00)	1.34e-10	pseudouridine synthase [Jimgerdemannia flammicorona]	994334	d__Eukaryota;k__Fungi;p__Mucoromycota;c__Endogonomycetes;o__Endogonales;f__Endogonaceae;g__Jimgerdemannia;s__Jimgerdemannia_flammicorona
SC-2_gene_3994	157	SWA30651.1	193	104/157(66.24)	9.08e-62	Uncharacterised protein [Klebsiella pneumoniae]	573	d__Bacteria;k__norank;p__Proteobacteria;c__Gammaproteobacteria;o__Enterobacterales;f__Enterobacteriaceae;g__Klebsiella;s__Klebsiella_pneumoniae
SC-2_gene_4012	129	EGY28405.1	198	40/96(41.67)	2.82e-10	Reverse transcriptase (RNA-dependent DNA polymerase), partial [Candidatus Regiella	1005043	d__Bacteria;k__norank;p__Proteobacteria;c__Gammaproteobacteria;o__Enterobacterales;f__Enterobacteriaceae;g__Candidatus_Regiella;s__Candidatus_Regiella_insecticola
SC-2_gene_4036	87	RUS15143.1	330	34/64(53.12)	1.34e-12	HAD-like domain-containing protein [Endogone sp. FLAS-	2340872	d__Eukaryota;k__Fungi;p__Mucoromycota;c__Endogonomycetes;o__Endogonales;f__Endogonaceae;g__Endogone;s__Endogone_sp._FLAS-F59071
SC-2_gene_4064	120	WP_129321181.1	354	39/106(36.79)	2.33e-17	DUF4817 domain-containing protein [Enterobacter cloacae complex sp. 4DZ3-17B2]	2511990	d__Bacteria;k__norank;p__Proteobacteria;c__Gammaproteobacteria;o__Enterobacterales;f__Enterobacteriaceae;g__Enterobacter;s__Enterobacter_cloacae_complex_sp._4DZ3-17B2
SC-3_gene_6	889	ORY08127.1	1260	311/864(36.00)	2.67e-130	hypothetical protein K493DRAFT_250596 [Basidiobolus meristosporus CBS	1314790	d__Eukaryota;k__Fungi;p__Zoopagomycota;c__Basidiobolomycetes;o__Basidiobolales;f__Basidiobolaceae;g__Basidiobolus;s__Basidiobolus_meristosporus
SC-3_gene_76	161	WP_019236285.1	159	152/159(95.60)	5.43e-96	hypothetical protein [Wolbachia pipientis]	955	d__Bacteria;k__norank;p__Proteobacteria;c__Alphaproteobacteria;o__Rickettsiales;f__Anaplasmataceae;g__Wolbachia;s__Wolbachia_pipientis
SC-3_gene_81	83	EEQ44064.1	335	24/54(44.44)	6.09e-08	casein kinase II [Candida albicans WO-1]	294748	d__Eukaryota;k__Fungi;p__Ascomycota;c__Saccharomycetes;o__Saccharomycetales;f__Debaryomycetaceae;g__Candida;s__Candida_albicans

SC-3_gene_89	137	ORZ30243.1	495	49/105(46.67)	5.00e-22	dual specificity protein phosphatase [Catenaria	765915	d__Eukaryota;k__Fungi;p__Blastocladiomycota;c__Blastocladiomycetes;o__Blastocladiales;f__Catenariaceae;g__Catenaria;s__Catenaria_anguillulae
SC-3_gene_99	520	WP_064085965.1	693	463/520(89.04)	0	hypothetical protein [Wolbachia endosymbiont of Dactylopius coccus]	None	Unclassified
SC-3_gene_200	633	PCI92573.1	660	120/376(31.91)	6.05e-36	hypothetical protein COB15_17455, partial	2021391	d__Bacteria;k__norank;p__Bacteroidetes;c__Flavobacteriia;o__Flavobacteriales;f__norank;g__norank;s__Flavobacteriales_bacterium
SC-3_gene_238	169	AGJ98777.1	333	102/113(90.27)	2.54e-65	Ankyrin repeat domain protein [Wolbachia endosymbiont of Drosophila simulans wNo]	1236908	d__Bacteria;k__norank;p__Proteobacteria;c__Alphaproteobacteria;o__Rickettsiales;f__Anaplasmataceae;g__Wolbachia;s__Wolbachia_endosymbiont_of_Drosophila_simulans
SC-3_gene_407	322	YP_009304996.1	781	94/317(29.65)	2.61e-20	glycoprotein precursor [Wuchang Cockroach Virus 1]	1608097	d__Viruses;k__Orthornavirae;p__Negarnaviricota;c__Ellioviricetes;o__Bunyavirales;f__Phasmaviridae;g__Orthophasmavirus;s__Wuchang_cockroach_orthophasmavirus_1
SC-3_gene_415	102	WP_118714888.1	132	35/96(36.46)	1.18e-11	fibronectin type III domain-containing protein [Odoribacter sp. AF15-53]	2292236	d__Bacteria;k__norank;p__Bacteroidetes;c__Bacteroidia;o__Bacteroidales;f__Odoribacteraceae;g__Odoribacter;s__Odoribacter_sp._AF15-53
SC-3_gene_506	459	RIB02370.1	378	74/338(21.89)	6.28e-08	Glycosyltransferase Family 49 protein, partial [Gigaspora rosea]	44941	d__Eukaryota;k__Fungi;p__Mucoromycota;c__Glomeromycetes;o__Diversisporales;f__Gigasporaceae;g__Gigaspora;s__Gigaspora_rosea
SC-3_gene_546	156	WP_099264718.1	354	43/149(28.86)	7.73e-17	type IV secretion system protein [Pseudomonas syringae]	317	d__Bacteria;k__norank;p__Proteobacteria;c__Gammaproteobacteria;o__Pseudomonadales;f__Pseudomonadaceae;g__Pseudomonas;s__Pseudomonas_syringae
SC-3_gene_608	494	AFN42309.1	742	131/363(36.09)	2.17e-51	leucine-rich protein [Cotesia sesamiae Mombasa bracovirus]	452649	d__Viruses;k__norank;p__norank;c__norank;o__norank;f__Polydnaviridae;g__Bracovirus;s__Cotesia_sesamiae_bracovirus
SC-3_gene_628	538	WP_010401348.1	924	527/538(97.96)	0	DUF3971 domain-containing protein [Wolbachia endosymbiont of Nasonia vitripennis]	180837	d__Bacteria;k__norank;p__Proteobacteria;c__Alphaproteobacteria;o__Rickettsiales;f__Anaplasmataceae;g__Wolbachia;s__Wolbachia_endosymbiont_of_Nasonia_vitripennis
SC-3_gene_661	460	WP_064125417.1	485	441/449(98.22)	0	IS4 family transposase [Wolbachia endosymbiont of Dactylopius coccus]	None	Unclassified
SC-3_gene_668	187	WP_065095187.1	304	157/174(90.23)	6.77e-104	MULTISPECIES: helix-turn-helix domain-containing protein [Wolbachia]	2640676	d__Bacteria;k__norank;p__Proteobacteria;c__Alphaproteobacteria;o__Rickettsiales;f__Anaplasmataceae;g__Wolbachia;s__norank
SC-3_gene_670	63	EAL60281.1	279	62/63(98.41)	2.39e-33	transcriptional regulator, putative [Wolbachia endosymbiont of Drosophila simulans]	77038	d__Bacteria;k__norank;p__Proteobacteria;c__Alphaproteobacteria;o__Rickettsiales;f__Anaplasmataceae;g__Wolbachia;s__Wolbachia_endosymbiont_of_Drosophila_simulans
SC-3_gene_695	261	ONI41439.1	491	82/232(35.34)	1.74e-26	hypothetical protein AN396_03550 [Epulopiscium sp. SCG-B11WGA-EpuloA1]	1710701	d__Bacteria;k__norank;p__Firmicutes;c__Clostridia;o__Clostridiales;f__Lachnospiraceae;g__Epulopiscium;s__Epulopiscium_sp._SCG-B11WGA-EpuloA1
SC-3_gene_743	46	PIE02179.1	628	26/44(59.09)	6.90e-08	peroxidase [Thiothrix nivea]	1031	d__Bacteria;k__norank;p__Proteobacteria;c__Gammaproteobacteria;o__Thiotrichales;f__Thiotrichaceae;g__Thiothrix;s__Thiothrix_nivea
SC-3_gene_758	305	WP_064085638.1	295	289/295(97.97)	7.36e-202	aspartate carbamoyltransferase catalytic subunit [Wolbachia endosymbiont of Dactylopius coccus]	None	Unclassified
SC-3_gene_793	84	ODA37401.1	348	66/76(86.84)	2.82e-34	hypothetical protein A8V34_00070 [Rickettsia sp.	1851203	d__Bacteria;k__norank;p__Proteobacteria;c__Alphaproteobacteria;o__Rickettsiales;f__Rickettsiaceae;g__Rickettsia;s__Rickettsia_sp._wq
SC-3_gene_827	478	PCG11796.1	923	383/478(80.13)	1.07e-241	hypothetical protein CO539_01055 [Wolbachia	None	Unclassified
SC-3_gene_867	466	OSY19256.1	647	221/470(47.02)	9.43e-138	hypothetical protein B0D75_01370 [Entomoplasmatales bacterium	None	Unclassified

SC-3_gene_875	65	KNE70860.1	411	34/64(53.12)	2.62e-12	hypothetical protein AMAG_14967 [Allomyces macrogynus ATCC 38327]	578462	d__Eukaryota;k__Fungi;p__Blastocladiomycota;c__Blastocladiomycetes;o__Blastocladiales;f__Blastocladiaceae;g__Allomyces;s__Allomyces_macrogynus
SC-3_gene_905	336	RXK52920.1	1956	76/304(25.00)	5.05e-14	hypothetical protein ESB00_14505 [Lacunisphaera sp.	2508720	d__Bacteria;k__norank;p__Verrucomicrobia;c__Opitutae;o__Opitutales;f__Opitutaceae;g__Oleiharenicola;s__Oleiharenicola_lentus
SC-3_gene_939	217	AGJ98616.1	627	184/192(95.83)	1.34e-121	hypothetical protein wNo_01790 [Wolbachia endosymbiont of Drosophila simulans wNo]	1236908	d__Bacteria;k__norank;p__Proteobacteria;c__Alphaproteobacteria;o__Rickettsiales;f__Anaplasmataceae;g__Wolbachia;s__Wolbachia_endosymbiont_of_Drosophila_simulans
SC-3_gene_1036	154	BAH22302.1	479	150/154(97.40)	3.33e-52	hypothetical protein [Wolbachia endosymbiont of Cadra cautella]	190193	d__Bacteria;k__norank;p__Proteobacteria;c__Alphaproteobacteria;o__Rickettsiales;f__Anaplasmataceae;g__Wolbachia;s__Wolbachia_endosymbiont_of_Cadra_cautella
SC-3_gene_1172	181	ONI57883.1	310	174/181(96.13)	1.16e-118	phage portal protein, lambda family [Wolbachia pipientis	1379786	d__Bacteria;k__norank;p__Proteobacteria;c__Alphaproteobacteria;o__Rickettsiales;f__Anaplasmataceae;g__Wolbachia;s__Wolbachia_pipientis
SC-3_gene_1189	212	YP_002321388.1	122	44/117(37.61)	2.43e-16	semaphorin-like protein [Oryctes rhinoceros nudivirus]	92521	d__Viruses;k__norank;p__norank;c__norank;o__norank;f__Nudiviridae;g__Alphanudivirus;s__Oryctes_rhinoceros_nudivirus
SC-3_gene_1228	230	RUA06787.1	914	68/239(28.45)	1.11e-15	hypothetical protein DSY43_01230	1913989	d__Bacteria;k__norank;p__Proteobacteria;c__Gammaproteobacteria;o__norank;f__norank;g__norank;s__Gammaproteobacteria_bacterium
SC-3_gene_1376	58	PYZ99289.1	379	40/58(68.97)	6.77e-18	hypothetical protein A6K26_010040, partial [Gammaproteobacteria bacterium	1838287	d__Bacteria;k__norank;p__Proteobacteria;c__Gammaproteobacteria;o__norank;f__norank;g__norank;s__Gammaproteobacteria_bacterium_2W06
SC-3_gene_1555	72	OPY05021.1	85	42/70(60.00)	2.42e-17	hypothetical protein A4E61_00470	1811701	d__Bacteria;k__norank;p__Proteobacteria;c__Deltaproteobacteria;o__Syntrophobacterales;f__Syntrophorhabdaceae;g__Syntrophorhabdus;s__Syntrophorhabdus_sp._PtaB.Bin184
SC-3_gene_1563	76	WP_114598937.1	353	48/76(63.16)	1.90e-26	J domain-containing protein [Microbacterium arborescens]	33883	d__Bacteria;k__norank;p__Actinobacteria;c__Actinobacteria;o__Micrococcales;f__Microbacteriaceae;g__Microbacterium;s__Microbacterium_arborescens
SC-3_gene_1571	234	OSY19256.1	647	81/156(51.92)	1.29e-44	hypothetical protein B0D75_01370 [Entomoplasmatales bacterium	None	Unclassified
SC-3_gene_1581	99	KIJ53482.1	404	33/99(33.33)	2.74e-12	hypothetical protein M422DRAFT_154980 [Sphaerobolus stellatus SS14]	990650	d__Eukaryota;k__Fungi;p__Basidiomycota;c__Agaricomycetes;o__Geastrales;f__Sphaerobolaceae;g__Sphaerobolus;s__Sphaerobolus_stellatus
SC-3_gene_1611	253	RUQ96634.1	195	53/148(35.81)	4.92e-22	hypothetical protein ELQ94_17130, partial [Labedella	1523160	d__Bacteria;k__norank;p__Actinobacteria;c__Actinobacteria;o__Micrococcales;f__Microbacteriaceae;g__Labedella;s__Labedella_endophytica
SC-3_gene_1619	178	ORM75813.1	176	176/176(100.00)	2.35e-121	transcriptional repressor MprA [Pantoea deleyi]	470932	d__Bacteria;k__norank;p__Proteobacteria;c__Gammaproteobacteria;o__Enterobacterales;f__Erwiniaceae;g__Pantoea;s__Pantoea_deleyi
SC-3_gene_1634	206	TBU13203.1	486	58/164(35.37)	2.17e-15	hypothetical protein CWI38_0504p0030 [Hamiltosporidium	1176355	d__Eukaryota;k__Fungi;p__Microsporidia;c__norank;o__norank;f__Dubosqiidae;g__Hamiltosporidium;s__Hamiltosporidium_tvaerminnensis
SC-3_gene_1636	55	RLJ22758.1	810	27/47(57.45)	4.50e-09	hypothetical protein DJ031_00290, partial [bacterium endosymbiont of Escarpia	1221601	d__Bacteria;k__norank;p__norank;c__norank;o__norank;f__norank;g__norank;s__bacterium_endosymbiont_of_Escarpia_laminata
SC-3_gene_1696	164	WP_126690656.1	164	61/164(37.20)	6.51e-27	hypothetical protein [Pantoea sp. YU22]	None	Unclassified
SC-3_gene_1769	213	RUA06787.1	914	56/168(33.33)	1.73e-13	hypothetical protein DSY43_01230	1913989	d__Bacteria;k__norank;p__Proteobacteria;c__Gammaproteobacteria;o__norank;f__norank;g__norank;s__Gammaproteobacteria_bacterium
SC-3_gene_1807	92	WP_041581368.1	87	74/90(82.22)	6.84e-50	hypothetical protein [Wolbachia endosymbiont of Drosophila simulans]	77038	d__Bacteria;k__norank;p__Proteobacteria;c__Alphaproteobacteria;o__Rickettsiales;f__Anaplasmataceae;g__Wolbachia;s__Wolbachia_endosymbiont_of_Drosophila_simulans
SC-3_gene_1812	163	PBQ29067.1	159	159/159(100.00)	3.73e-108	hypothetical protein BTO27_01575 [Wolbachia	2049495	d__Bacteria;k__norank;p__Proteobacteria;c__Alphaproteobacteria;o__Rickettsiales;f__Anaplasmataceae;g__Wolbachia;s__Wolbachia_pipientis

SC-3_gene_1871	98	RMZ86238.1	1436	24/41(58.54)	1.09e-06	hypothetical protein DV737_g46, partial [Chaetothyriales sp. CBS 132003]	2249419	d__Eukaryota;k__Fungi;p__Ascomycota;c__Eurotiomycetes;o__Chaetothyriales;f__norank;g__norank;s__Chaetothyriales_sp._CBS_132003
SC-3_gene_1901	74	PKY59095.1	184	27/66(40.91)	1.49e-08	hypothetical protein RhiirA4_301286, partial	588596	d__Eukaryota;k__Fungi;p__Mucoromycota;c__Glomeromycetes;o__Glomerales;f__Glomeraceae;g__Rhizophagus;s__Rhizophagus_irregularis
SC-3_gene_1941	205	CAS06612.1	275	37/85(43.53)	6.62e-09	protein tyrosine phosphatase, partial [Cotesia melanoscela	523799	d__Viruses;k__norank;p__norank;c__norank;o__norank;f__Polydnaviridae;g__Bracovirus;s__Cotesia_melanoscela_bracovirus
SC-3_gene_1945	121	WP_063630962.1	504	94/121(77.69)	1.52e-56	hypothetical protein [Wolbachia endosymbiont of Laodelphax striatella]	368602	d__Bacteria;k__norank;p__Proteobacteria;c__Alphaproteobacteria;o__Rickettsiales;f__Anaplasmataceae;g__Wolbachia;s__Wolbachia_endosymbiont_of_Laodelphax_striatellus
SC-3_gene_2058	102	RYE20179.1	510	35/87(40.23)	1.17e-11	hypothetical protein EOP45_11485	2021370	d__Bacteria;k__norank;p__Bacteroidetes;c__Sphingobacteriia;o__Sphingobacteriales;f__Sphingobacteriaceae;g__norank;s__Sphingobacteriaceae_bacterium
SC-3_gene_2136	75	KFH62861.1	369	29/50(58.00)	6.25e-11	hypothetical protein MVEG_11386 [Mortierella	1069443	d__Eukaryota;k__Fungi;p__Mucoromycota;c__Mortierellomycetes;o__Mortierellales;f__Mortierellaceae;g__Podila;s__Podila_verticillata
SC-3_gene_2208	190	XP_021884214.1	317	78/174(44.83)	1.97e-44	PIN domain-like protein [Lobosporangium transversale]	64571	d__Eukaryota;k__Fungi;p__Mucoromycota;c__Mortierellomycetes;o__Mortierellales;f__Mortierellaceae;g__Lobosporangium;s__Lobosporangium_transversale
SC-3_gene_2214	101	WP_031575742.1	469	33/75(44.00)	1.70e-12	aldehyde dehydrogenase family protein [Proteiniclasticum	398199	d__Bacteria;k__norank;p__Firmicutes;c__Clostridia;o__Clostridiales;f__Clostridiaceae;g__Proteiniclasticum;s__Proteiniclasticum_ruminis
SC-3_gene_2246	199	RUM30001.1	189	85/194(43.81)	2.11e-40	hypothetical protein DSY42_05315 [Aquifex sp.]	313370	d__Bacteria;k__norank;p__Aquificae;c__Aquificae;o__Aquificales;f__Aquificaceae;g__Aquifex;s__Aquifex_sp.
SC-3_gene_2292	248	OSY19287.1	671	59/163(36.20)	1.72e-19	hypothetical protein B0D75_01270, partial [Entomoplasmatales bacterium	None	Unclassified
SC-3_gene_2325	123	OCB90619.1	242	38/94(40.43)	6.41e-17	hypothetical protein A7U60_g2137 [Sanghuangporus	108892	d__Eukaryota;k__Fungi;p__Basidiomycota;c__Agaricomycetes;o__Hymenochaetales;f__Hymenochaetaceae;g__Sanghuangporus;s__Sanghuangporus_baumii
SC-3_gene_2352	151	EXX60179.1	425	29/92(31.52)	1.03e-10	hypothetical protein RirG_182340 [Rhizophagus irregularis DAOM 197198w]	1432141	d__Eukaryota;k__Fungi;p__Mucoromycota;c__Glomeromycetes;o__Glomerales;f__Glomeraceae;g__Rhizophagus;s__Rhizophagus_irregularis
SC-3_gene_2435	112	KIK96327.1	945	38/112(33.93)	1.46e-07	hypothetical protein PAXRUDRAFT_32465 [Paxillus rubicundulus Ve08.2h10]	930991	d__Eukaryota;k__Fungi;p__Basidiomycota;c__Agaricomycetes;o__Boletales;f__Paxillaceae;g__Paxillus;s__Paxillus_rubicundulus
SC-3_gene_2448	252	EEH12371.1	797	249/252(98.81)	2.95e-173	DNA polymerase III, alpha subunit [Wolbachia endosymbiont of Muscidifurax	77037	d__Bacteria;k__norank;p__Proteobacteria;c__Alphaproteobacteria;o__Rickettsiales;f__Anaplasmataceae;g__Wolbachia;s__Wolbachia_endosymbiont_of_Muscidifurax_uniraptor
SC-3_gene_2483	63	WP_064085590.1	488	56/59(94.92)	1.61e-25	hypothetical protein [Wolbachia endosymbiont of Dactylopius coccus]	None	Unclassified
SC-3_gene_2484	104	PBQ29141.1	413	104/104(100.00)	2.91e-66	citrate (Si)-synthase [Wolbachia pipientis wAus]	2049495	d__Bacteria;k__norank;p__Proteobacteria;c__Alphaproteobacteria;o__Rickettsiales;f__Anaplasmataceae;g__Wolbachia;s__Wolbachia_pipientis
SC-3_gene_2502	62	PGH38173.1	63	42/63(66.67)	8.38e-23	hypothetical protein CRN43_16775 [Candidatus	2044729	d__Bacteria;k__norank;p__Bacteroidetes;c__Cytophagia;o__Cytophagales;f__Cytophagaceae;g__Candidatus_Nephrothrix;s__Candidatus_Nephrothrix_sp._EaCA
SC-3_gene_2506	180	ELP25067.1	207	164/180(91.11)	1.44e-109	Phage head maturation protease [Pantoea agglomerans 299R]	1261128	d__Bacteria;k__norank;p__Proteobacteria;c__Gammaproteobacteria;o__Enterobacterales;f__Erwiniaceae;g__Pantoea;s__Pantoea_agglomerans
SC-3_gene_2579	148	QAY00891.1	469	91/148(61.49)	3.74e-61	putative portal protein [Salmonella phage SeSz-2]	2419753	d__Viruses;k__norank;p__Uroviricota;c__Caudoviricetes;o__Caudovirales;f__Siphoviridae;g__norank;s__Salmonella_phage_SeSz-2
SC-3_gene_2582	224	RUA04709.1	567	57/183(31.15)	9.51e-15	hypothetical protein DSY43_05900, partial [Gammaproteobacteria	1913989	d__Bacteria;k__norank;p__Proteobacteria;c__Gammaproteobacteria;o__norank;f__norank;g__norank;s__Gammaproteobacteria_bacterium

SC-3_gene_2598	60	PIA14317.1	195	39/58(67.24)	1.69e-18	RHO protein GDP dissociation inhibitor-containing protein [Coemansia reversa NRRL 1564]	763665	d__Eukaryota;k__Fungi;p__Zoopagomycota;c__Kickxellomycetes;o__Kickxellales;f__Kickxellaceae;g__Coemansia;s__Coemansia_reversa
SC-3_gene_2676	215	CEL61987.1	692	72/202(35.64)	5.28e-22	hypothetical protein RSOLAG1IB_04738 [Rhizoctonia solani AG-1 IB]	1108050	d__Eukaryota;k__Fungi;p__Basidiomycota;c__Agaricomycetes;o__Cantharellales;f__Ceratobasidiaceae;g__Rhizoctonia;s__Rhizoctonia_solani
SC-3_gene_2693	74	RYG70345.1	3410	32/51(62.75)	3.19e-07	hypothetical protein EON64_00415 [archaeon]	1906665	d__Archaea;k__norank;p__norank;c__norank;o__norank;f__norank;g__norank;s__archaeon
SC-3_gene_2704	47	OCA05871.1	38	26/35(74.29)	2.23e-06	hypothetical protein wTpre_190 [Wolbachia endosymbiont of Trichogramma pretiosum]	125593	d__Bacteria;k__norank;p__Proteobacteria;c__Alphaproteobacteria;o__Rickettsiales;f__Anaplasmataceae;g__Wolbachia;s__Wolbachia_endosymbiont_of_Trichogramma_pretiosum
SC-3_gene_2730	84	ORM81087.1	60	60/60(100.00)	2.13e-37	hypothetical protein HA42_11905 [Pantoea deleyi]	470932	d__Bacteria;k__norank;p__Proteobacteria;c__Gammaproteobacteria;o__Enterobacterales;f__Erwiniaceae;g__Pantoea;s__Pantoea_deleyi
SC-3_gene_2746	194	OAJ10991.1	2020	53/178(29.78)	1.91e-07	hypothetical protein A4X03_g7226, partial [Tilletia	None	Unclassified
SC-3_gene_2753	50	KDB19242.1	119	49/50(98.00)	3.48e-24	F0F1 ATP synthase subunit A [Wolbachia endosymbiont of Glossina morsitans morsitans]	1150948	d__Bacteria;k__norank;p__Proteobacteria;c__Alphaproteobacteria;o__Rickettsiales;f__Anaplasmataceae;g__Wolbachia;s__Wolbachia_endosymbiont_of_Glossina_morsitans_morsitans
SC-3_gene_2773	128	WP_106437099.1	473	31/67(46.27)	2.18e-14	cytochrome P450 [Streptomyces prunicolor]	67348	d__Bacteria;k__norank;p__Actinobacteria;c__Actinobacteria;o__Streptomycetales;f__Streptomycetaceae;g__Streptomyces;s__Streptomyces_prunicolor
SC-3_gene_2807	147	KXN73839.1	878	43/88(48.86)	5.65e-20	PLC-like phosphodiesterase [Conidiobolus coronatus NRRL 28638]	796925	d__Eukaryota;k__Fungi;p__Zoopagomycota;c__Entomophthoromycetes;o__Entomophthorales;f__Ancylistaceae;g__Conidiobolus;s__Conidiobolus_coronatus
SC-3_gene_2817	219	RZK26366.1	325	48/150(32.00)	3.65e-20	hypothetical protein EOO43_03045, partial	239	d__Bacteria;k__norank;p__Bacteroidetes;c__Flavobacteriia;o__Flavobacteriales;f__Flavobacteriaceae;g__Flavobacterium;s__Flavobacterium_sp.
SC-3_gene_2876	69	RYG97378.1	103	28/68(41.18)	1.42e-11	hypothetical protein EON65_52965, partial [archaeon]	1906665	d__Archaea;k__norank;p__norank;c__norank;o__norank;f__norank;g__norank;s__archaeon
SC-3_gene_2905	90	XP_006677549.1	766	32/78(41.03)	9.29e-11	hypothetical protein BATDEDRAFT_86893 [Batrachochytrium dendrobatidis JAM81]	684364	d__Eukaryota;k__Fungi;p__Chytridiomycota;c__Chytridiomycetes;o__Rhizophydiales;f__norank;g__Batrachochytrium;s__Batrachochytrium_dendrobatidis
SC-3_gene_2910	72	APR78210.1	847	30/70(42.86)	3.22e-08	Fucolectin-related protein [Minicystis rosea]	888845	d__Bacteria;k__norank;p__Proteobacteria;c__Deltaproteobacteria;o__Myxococcales;f__norank;g__Minicystis;s__Minicystis_rosea
SC-3_gene_2994	86	RAM39464.1	115	60/72(83.33)	4.15e-39	50S ribosomal protein L30e, partial [Enterobacter hormaechei]	158836	d__Bacteria;k__norank;p__Proteobacteria;c__Gammaproteobacteria;o__Enterobacterales;f__Enterobacteriaceae;g__Enterobacter;s__Enterobacter_hormaechei
MDJ-2_gene_159	194	PBQ30128.1	197	193/197(97.97)	2.87e-140	pyridoxamine 5'-phosphate oxidase [Wolbachia pipientis	2049495	d__Bacteria;k__norank;p__Proteobacteria;c__Alphaproteobacteria;o__Rickettsiales;f__Anaplasmataceae;g__Wolbachia;s__Wolbachia_pipientis
MDJ-2_gene_160	265	WP_108784327.1	265	257/265(96.98)	3.08e-190	phosphoribosylformylglycinamidine synthase subunit PurQ [Wolbachia endosymbiont of	215173	d__Bacteria;k__norank;p__Proteobacteria;c__Alphaproteobacteria;o__Rickettsiales;f__Anaplasmataceae;g__Wolbachia;s__Wolbachia_endosymbiont_of_Bemisia_tabaci
MDJ-2_gene_161	416	WP_017531954.1	416	406/416(97.60)	7.79e-276	DUF3333 domain-containing protein [Wolbachia endosymbiont of Diaphorina citri]	116598	d__Bacteria;k__norank;p__Proteobacteria;c__Alphaproteobacteria;o__Rickettsiales;f__Anaplasmataceae;g__Wolbachia;s__Wolbachia_endosymbiont_of_Diaphorina_citri
MDJ-2_gene_162	125	PBQ30131.1	125	125/125(100.00)	3.48e-82	50S ribosomal protein L19 [Wolbachia pipientis wAus]	2049495	d__Bacteria;k__norank;p__Proteobacteria;c__Alphaproteobacteria;o__Rickettsiales;f__Anaplasmataceae;g__Wolbachia;s__Wolbachia_pipientis
MDJ-2_gene_163	232	WP_063630865.1	232	231/232(99.57)	8.82e-169	tRNA (guanosine(37)-N1)-methyltransferase TrmD [Wolbachia endosymbiont of Laodelphax striatella]	2640676	d__Bacteria;k__norank;p__Proteobacteria;c__Alphaproteobacteria;o__Rickettsiales;f__Anaplasmataceae;g__Wolbachia;s__norank

MDJ-2_gene_165	345	PCG11259.1	345	340/345(98.55)	7.48e-254	phosphoribosylformylglycinamidine cyclo-ligase [Wolbachia	None	Unclassified
MDJ-2_gene_166	86	CCE77472.1	86	86/86(100.00)	3.23e-53	conserved hypothetical protein [Wolbachia pipientis wAlbB]	1116230	d__Bacteria;k__norank;p__Proteobacteria;c__Alphaproteobacteria;o__Rickettsiales;f__Anaplasmataceae;g__Wolbachia;s__Wolbachia_pipientis
MDJ-2_gene_167	92	PCG11261.1	92	91/92(98.91)	3.94e-50	hypothetical protein CO539_04235 [Wolbachia	None	Unclassified
MDJ-2_gene_170	1095	WP_010405587.1	1072	1042/1095(95.16)	0	carbamoyl-phosphate synthase large subunit [Wolbachia endosymbiont of Nasonia	180837	d__Bacteria;k__norank;p__Proteobacteria;c__Alphaproteobacteria;o__Rickettsiales;f__Anaplasmataceae;g__Wolbachia;s__Wolbachia_endosymbiont_of_Nasonia_vitripennis
MDJ-2_gene_172	271	CAQ55023.1	271	269/270(99.63)	5.04e-183	ribonuclease, BN family [Wolbachia endosymbiont of Culex quinquefasciatus Pel]	570417	d__Bacteria;k__norank;p__Proteobacteria;c__Alphaproteobacteria;o__Rickettsiales;f__Anaplasmataceae;g__Wolbachia;s__Wolbachia_endosymbiont_of_Culex_quinquefasciatus
MDJ-2_gene_173	117	WP_010405591.1	117	110/116(94.83)	1.07e-70	hypothetical protein [Wolbachia endosymbiont of Nasonia vitripennis]	2640676	d__Bacteria;k__norank;p__Proteobacteria;c__Alphaproteobacteria;o__Rickettsiales;f__Anaplasmataceae;g__Wolbachia;s__norank
MDJ-2_gene_174	334	WP_010404148.1	334	316/334(94.61)	5.97e-234	NAD(P)/FAD-dependent oxidoreductase [Wolbachia endosymbiont of Nasonia vitripennis]	180837	d__Bacteria;k__norank;p__Proteobacteria;c__Alphaproteobacteria;o__Rickettsiales;f__Anaplasmataceae;g__Wolbachia;s__Wolbachia_endosymbiont_of_Nasonia_vitripennis
MDJ-2_gene_176	294	AGJ98799.1	294	291/294(98.98)	9.58e-204	GTP-binding protein Era [Wolbachia endosymbiont of Drosophila simulans wNo]	1236908	d__Bacteria;k__norank;p__Proteobacteria;c__Alphaproteobacteria;o__Rickettsiales;f__Anaplasmataceae;g__Wolbachia;s__Wolbachia_endosymbiont_of_Drosophila_simulans
MDJ-2_gene_177	275	PBQ27225.1	275	270/275(98.18)	1.53e-195	3'-5' exonuclease [Wolbachia pipientis wAus]	2049495	d__Bacteria;k__norank;p__Proteobacteria;c__Alphaproteobacteria;o__Rickettsiales;f__Anaplasmataceae;g__Wolbachia;s__Wolbachia_pipientis
MDJ-2_gene_178	437	WP_127463809.1	437	428/437(97.94)	0	lipase family protein [Wolbachia endosymbiont of Bemisia tabaci]	215173	d__Bacteria;k__norank;p__Proteobacteria;c__Alphaproteobacteria;o__Rickettsiales;f__Anaplasmataceae;g__Wolbachia;s__Wolbachia_endosymbiont_of_Bemisia_tabaci
MDJ-2_gene_179	680	WP_010402866.1	680	630/680(92.65)	0	porin [Wolbachia endosymbiont of Nasonia vitripennis]	180837	d__Bacteria;k__norank;p__Proteobacteria;c__Alphaproteobacteria;o__Rickettsiales;f__Anaplasmataceae;g__Wolbachia;s__Wolbachia_endosymbiont_of_Nasonia_vitripennis
MDJ-2_gene_182	265	WP_108784358.1	262	258/265(97.36)	9.91e-184	DUF2163 domain-containing protein [Wolbachia endosymbiont of Bemisia tabaci]	215173	d__Bacteria;k__norank;p__Proteobacteria;c__Alphaproteobacteria;o__Rickettsiales;f__Anaplasmataceae;g__Wolbachia;s__Wolbachia_endosymbiont_of_Bemisia_tabaci
MDJ-2_gene_183	103	PCG16567.1	100	81/86(94.19)	3.49e-53	hypothetical protein CO538_05900 [Wolbachia	None	Unclassified
MDJ-2_gene_186	509	WP_064085553.1	509	502/509(98.62)	0	methionine--tRNA ligase [Wolbachia endosymbiont of Dactylopius coccus]	None	Unclassified
MDJ-2_gene_187	301	WP_064085554.1	297	291/301(96.68)	5.62e-210	hypothetical protein [Wolbachia endosymbiont of Dactylopius coccus]	None	Unclassified
MDJ-2_gene_189	192	WP_064085887.1	192	189/192(98.44)	3.51e-128	dephospho-CoA kinase [Wolbachia endosymbiont of Dactylopius coccus]	None	Unclassified
MDJ-2_gene_190	392	WP_029237687.1	392	390/392(99.49)	5.56e-282	ribonuclease D [Wolbachia endosymbiont of Nasonia vitripennis]	2640676	d__Bacteria;k__norank;p__Proteobacteria;c__Alphaproteobacteria;o__Rickettsiales;f__Anaplasmataceae;g__Wolbachia;s__norank
MDJ-2_gene_191	439	WP_064085885.1	439	428/439(97.49)	4.66e-307	dihydroorotase [Wolbachia endosymbiont of Dactylopius coccus]	None	Unclassified
MDJ-2_gene_192	142	WP_099458610.1	739	87/96(90.62)	2.27e-42	lipase [Wolbachia pipientis]	953	d__Bacteria;k__norank;p__Proteobacteria;c__Alphaproteobacteria;o__Rickettsiales;f__Anaplasmataceae;g__Wolbachia;s__norank

MDJ-2_gene_193	118	WP_064085658.1	342	116/118(98.31)	3.15e-31	hypothetical protein [Wolbachia endosymbiont of Dactylopius coccus]	None	Unclassified
MDJ-2_gene_194	262	PBQ28131.1	259	250/262(95.42)	7.54e-182	NAD(P)-dependent oxidoreductase [Wolbachia	2049495	d__Bacteria;k__norank;p__Proteobacteria;c__Alphaproteobacteria;o__Rickettsiales;f__Anaplasmataceae;g__Wolbachia;s__Wolbachia_pipientis
MDJ-2_gene_196	121	WP_063630698.1	121	120/121(99.17)	1.89e-75	hypothetical protein [Wolbachia endosymbiont of Laodelphax striatella]	368602	d__Bacteria;k__norank;p__Proteobacteria;c__Alphaproteobacteria;o__Rickettsiales;f__Anaplasmataceae;g__Wolbachia;s__Wolbachia_endosymbiont_of_Laodelphax_striatellus
MDJ-2_gene_197	598	WP_052264809.1	598	578/598(96.66)	0	aminopeptidase P family protein [Wolbachia endosymbiont of Operophtera brumata]	1620420	d__Bacteria;k__norank;p__Proteobacteria;c__Alphaproteobacteria;o__Rickettsiales;f__Anaplasmataceae;g__Wolbachia;s__Wolbachia_endosymbiont_of_Operophtera_brumata
MDJ-2_gene_198	682	WP_127463782.1	682	668/682(97.95)	0	tRNA uridine-5-carboxymethylaminomethyl(34) synthesis enzyme MnmG [Wolbachia endosymbiont of Bemisia tabaci]	215173	d__Bacteria;k__norank;p__Proteobacteria;c__Alphaproteobacteria;o__Rickettsiales;f__Anaplasmataceae;g__Wolbachia;s__Wolbachia_endosymbiont_of_Bemisia_tabaci
MDJ-2_gene_200	104	CCE77729.1	104	95/103(92.23)	5.85e-53	conserved hypothetical protein [Wolbachia pipientis wAlbB]	1116230	d__Bacteria;k__norank;p__Proteobacteria;c__Alphaproteobacteria;o__Rickettsiales;f__Anaplasmataceae;g__Wolbachia;s__Wolbachia_pipientis
MDJ-2_gene_203	357	WP_029237642.1	357	354/357(99.16)	2.06e-232	AI-2E family transporter [Wolbachia endosymbiont of Nasonia vitripennis]	180837	d__Bacteria;k__norank;p__Proteobacteria;c__Alphaproteobacteria;o__Rickettsiales;f__Anaplasmataceae;g__Wolbachia;s__Wolbachia_endosymbiont_of_Nasonia_vitripennis
MDJ-2_gene_204	188	WP_010404012.1	188	187/188(99.47)	1.23e-126	alpha/beta hydrolase [Wolbachia endosymbiont of Nasonia vitripennis]	180837	d__Bacteria;k__norank;p__Proteobacteria;c__Alphaproteobacteria;o__Rickettsiales;f__Anaplasmataceae;g__Wolbachia;s__Wolbachia_endosymbiont_of_Nasonia_vitripennis
MDJ-2_gene_205	48	OCA06783.1	48	33/48(68.75)	3.84e-13	hypothetical protein wTpre_1130 [Wolbachia endosymbiont of Trichogramma pretiosum]	125593	d__Bacteria;k__norank;p__Proteobacteria;c__Alphaproteobacteria;o__Rickettsiales;f__Anaplasmataceae;g__Wolbachia;s__Wolbachia_endosymbiont_of_Trichogramma_pretiosum
MDJ-2_gene_206	398	WP_029237641.1	401	397/398(99.75)	7.63e-286	2-C-methyl-D-erythritol 4-phosphate cytidylyltransferase [Wolbachia endosymbiont of Nasonia vitripennis]	2640676	d__Bacteria;k__norank;p__Proteobacteria;c__Alphaproteobacteria;o__Rickettsiales;f__Anaplasmataceae;g__Wolbachia;s__norank
MDJ-2_gene_207	288	WP_064085913.1	288	276/288(95.83)	6.63e-192	ParB/RepB/Spo0J family partition protein [Wolbachia endosymbiont of Dactylopius	None	Unclassified
MDJ-2_gene_208	280	AGJ98978.1	280	270/279(96.77)	7.41e-185	Chromosome partitioning protein,ParA family [Wolbachia endosymbiont of Drosophila simulans wNo]	1236908	d__Bacteria;k__norank;p__Proteobacteria;c__Alphaproteobacteria;o__Rickettsiales;f__Anaplasmataceae;g__Wolbachia;s__Wolbachia_endosymbiont_of_Drosophila_simulans
MDJ-2_gene_209	826	WP_063630880.1	826	823/826(99.64)	0	response regulator [Wolbachia endosymbiont of Laodelphax striatella]	368602	d__Bacteria;k__norank;p__Proteobacteria;c__Alphaproteobacteria;o__Rickettsiales;f__Anaplasmataceae;g__Wolbachia;s__Wolbachia_endosymbiont_of_Laodelphax_striatellus
MDJ-2_gene_212	408	WP_021827502.1	408	290/407(71.25)	2.97e-209	hypothetical protein [Holospora obtusa]	49893	d__Bacteria;k__norank;p__Proteobacteria;c__Alphaproteobacteria;o__Holosporales;f__Holosporaceae;g__Holospora;s__Holospora_obtusa
MDJ-2_gene_213	66	WP_110410122.1	66	47/66(71.21)	1.40e-27	hypothetical protein [Wolbachia endosymbiont of Folsomia	169402	d__Bacteria;k__norank;p__Proteobacteria;c__Alphaproteobacteria;o__Rickettsiales;f__Anaplasmataceae;g__Wolbachia;s__Wolbachia_endosymbiont_of_Folsomia_candida
MDJ-2_gene_214	509	AGJ99390.1	491	449/504(89.09)	8.59e-227	hypothetical protein wNo_10170 [Wolbachia endosymbiont of Drosophila simulans wNo]	1236908	d__Bacteria;k__norank;p__Proteobacteria;c__Alphaproteobacteria;o__Rickettsiales;f__Anaplasmataceae;g__Wolbachia;s__Wolbachia_endosymbiont_of_Drosophila_simulans
MDJ-2_gene_215	189	WP_019236467.1	186	140/183(76.50)	4.02e-93	hypothetical protein [Wolbachia pipientis]	953	d__Bacteria;k__norank;p__Proteobacteria;c__Alphaproteobacteria;o__Rickettsiales;f__Anaplasmataceae;g__Wolbachia;s__norank

MDJ-2_gene_216	397	WP_112477225.1	393	318/403(78.91)	6.65e-232	phage tail protein [Wolbachia endosymbiont of Folsomia	169402	d__Bacteria;k__norank;p__Proteobacteria;c__Alphaproteobacteria;o__Rickettsiales;f__Anaplasmataceae;g__Wolbachia;s__Wolbachia_endosymbiont_of_Folsomia_candida
MDJ-2_gene_223	1207	PBQ29075.1	1208	1125/1209(93.05)	0	hypothetical protein BTO27_01615 [Wolbachia	2049495	d__Bacteria;k__norank;p__Proteobacteria;c__Alphaproteobacteria;o__Rickettsiales;f__Anaplasmataceae;g__Wolbachia;s__Wolbachia_pipientis
MDJ-2_gene_225	253	PBQ29073.1	253	248/253(98.02)	1.39e-171	hypothetical protein BTO27_01605 [Wolbachia	2049495	d__Bacteria;k__norank;p__Proteobacteria;c__Alphaproteobacteria;o__Rickettsiales;f__Anaplasmataceae;g__Wolbachia;s__Wolbachia_pipientis
MDJ-2_gene_228	213	WP_063631052.1	213	210/213(98.59)	1.30e-147	nucleoside monophosphate kinase [Wolbachia endosymbiont of Laodelphax striatella]	368602	d__Bacteria;k__norank;p__Proteobacteria;c__Alphaproteobacteria;o__Rickettsiales;f__Anaplasmataceae;g__Wolbachia;s__Wolbachia_endosymbiont_of_Laodelphax_striatellus
MDJ-2_gene_229	442	WP_010404717.1	442	441/442(99.77)	2.45e-299	MULTISPECIES: preprotein translocase subunit SecY [Wolbachia]	180837	d__Bacteria;k__norank;p__Proteobacteria;c__Alphaproteobacteria;o__Rickettsiales;f__Anaplasmataceae;g__Wolbachia;s__Wolbachia_endosymbiont_of_Nasonia_vitripennis
MDJ-2_gene_230	156	PCG11870.1	156	156/156(100.00)	3.18e-102	50S ribosomal protein L15 [Wolbachia pipientis]	None	Unclassified
MDJ-2_gene_231	170	PBQ28102.1	170	170/170(100.00)	5.78e-114	30S ribosomal protein S5 [Wolbachia pipientis wAus]	2049495	d__Bacteria;k__norank;p__Proteobacteria;c__Alphaproteobacteria;o__Rickettsiales;f__Anaplasmataceae;g__Wolbachia;s__Wolbachia_pipientis
MDJ-2_gene_234	131	CQD06037.1	156	130/131(99.24)	7.06e-88	30S ribosomal protein S8 [Wolbachia endosymbiont wPip_Mol of Culex molestus]	1402163	d__Bacteria;k__norank;p__Proteobacteria;c__Alphaproteobacteria;o__Rickettsiales;f__Anaplasmataceae;g__Wolbachia;s__Wolbachia_endosymbiont_of_Culex_molestus
MDJ-2_gene_235	102	PBQ28098.1	102	102/102(100.00)	1.29e-64	30S ribosomal protein S14 [Wolbachia pipientis wAus]	2049495	d__Bacteria;k__norank;p__Proteobacteria;c__Alphaproteobacteria;o__Rickettsiales;f__Anaplasmataceae;g__Wolbachia;s__Wolbachia_pipientis
MDJ-2_gene_236	178	WP_010404703.1	178	178/178(100.00)	3.19e-124	50S ribosomal protein L5 [Wolbachia endosymbiont of Nasonia vitripennis]	2640676	d__Bacteria;k__norank;p__Proteobacteria;c__Alphaproteobacteria;o__Rickettsiales;f__Anaplasmataceae;g__Wolbachia;s__norank
MDJ-2_gene_239	76	PBD16021.1	78	70/76(92.11)	2.92e-45	30S ribosomal protein S17 [Wolbachia endosymbiont of Drosophila subpulchrella]	2033655	d__Bacteria;k__norank;p__Proteobacteria;c__Alphaproteobacteria;o__Rickettsiales;f__Anaplasmataceae;g__Wolbachia;s__Wolbachia_endosymbiont_of_Drosophila_subpulchrella
MDJ-2_gene_240	72	CCE76947.1	72	70/72(97.22)	6.49e-40	50S ribosomal protein L29 [Wolbachia pipientis wAlbB]	1116230	d__Bacteria;k__norank;p__Proteobacteria;c__Alphaproteobacteria;o__Rickettsiales;f__Anaplasmataceae;g__Wolbachia;s__Wolbachia_pipientis
MDJ-2_gene_241	137	PCG11880.1	137	136/137(99.27)	1.79e-92	50S ribosomal protein L16 [Wolbachia pipientis]	None	Unclassified
MDJ-2_gene_242	206	B3CN32.1	206	198/206(96.12)	2.95e-136	RecName: Full=30S ribosomal protein S3	570417	d__Bacteria;k__norank;p__Proteobacteria;c__Alphaproteobacteria;o__Rickettsiales;f__Anaplasmataceae;g__Wolbachia;s__Wolbachia_endosymbiont_of_Culex_quinquefasciatus
MDJ-2_gene_243	119	PBQ28090.1	116	114/116(98.28)	2.02e-73	50S ribosomal protein L22 [Wolbachia pipientis wAus]	2049495	d__Bacteria;k__norank;p__Proteobacteria;c__Alphaproteobacteria;o__Rickettsiales;f__Anaplasmataceae;g__Wolbachia;s__Wolbachia_pipientis
MDJ-2_gene_245	404	BAH22191.1	403	386/401(96.26)	1.89e-288	queuine tRNA-ribosyltransferase [Wolbachia endosymbiont of Cadra cautella]	190193	d__Bacteria;k__norank;p__Proteobacteria;c__Alphaproteobacteria;o__Rickettsiales;f__Anaplasmataceae;g__Wolbachia;s__Wolbachia_endosymbiont_of_Cadra_cautella
MDJ-2_gene_246	166	WP_010402402.1	166	166/166(100.00)	1.13e-118	NADH-quinone oxidoreductase subunit NuoE [Wolbachia endosymbiont of Nasonia vitripennis]	180837	d__Bacteria;k__norank;p__Proteobacteria;c__Alphaproteobacteria;o__Rickettsiales;f__Anaplasmataceae;g__Wolbachia;s__Wolbachia_endosymbiont_of_Nasonia_vitripennis
MDJ-2_gene_247	687	WP_063630586.1	695	595/692(85.98)	0	ankyrin repeat domain-containing protein [Wolbachia endosymbiont of Laodelphax striatella]	368602	d__Bacteria;k__norank;p__Proteobacteria;c__Alphaproteobacteria;o__Rickettsiales;f__Anaplasmataceae;g__Wolbachia;s__Wolbachia_endosymbiont_of_Laodelphax_striatellus
MDJ-2_gene_248	737	WP_108784210.1	742	577/743(77.66)	6.22e-290	hypothetical protein [Wolbachia endosymbiont of Bemisia tabaci]	215173	d__Bacteria;k__norank;p__Proteobacteria;c__Alphaproteobacteria;o__Rickettsiales;f__Anaplasmataceae;g__Wolbachia;s__Wolbachia_endosymbiont_of_Bemisia_tabaci
MDJ-2_gene_250	1058	WP_064085845.1	1081	996/1061(93.87)	0	conjugal transfer protein [Wolbachia endosymbiont of Dactylopius coccus]	None	Unclassified

MDJ-2_gene_251	209	WP_064085846.1	209	209/209(100.00)	1.11e-137	hypothetical protein [Wolbachia endosymbiont of Dactylopius coccus]	None	Unclassified
MDJ-2_gene_254	180	WP_108784480.1	176	131/180(72.78)	6.14e-81	hypothetical protein [Wolbachia endosymbiont of Bemisia tabaci]	215173	d__Bacteria;k__norank;p__Proteobacteria;c__Alphaproteobacteria;o__Rickettsiales;f__Anaplasmataceae;g__Wolbachia;s__Wolbachia_endosymbiont_of_Bemisia_tabaci
MDJ-2_gene_257	191	WP_017532371.1	191	191/191(100.00)	1.44e-140	MULTISPECIES: NADH-quinone oxidoreductase subunit C [Wolbachia]	2640676	d__Bacteria;k__norank;p__Proteobacteria;c__Alphaproteobacteria;o__Rickettsiales;f__Anaplasmataceae;g__Wolbachia;s__norank
MDJ-2_gene_258	314	WP_064085466.1	314	311/314(99.04)	4.10e-215	malate dehydrogenase [Wolbachia endosymbiont of Dactylopius coccus]	None	Unclassified
MDJ-2_gene_260	162	PBQ30147.1	162	160/162(98.77)	5.89e-119	RNA pyrophosphohydrolase [Wolbachia pipientis wAus]	2049495	d__Bacteria;k__norank;p__Proteobacteria;c__Alphaproteobacteria;o__Rickettsiales;f__Anaplasmataceae;g__Wolbachia;s__Wolbachia_pipientis
MDJ-2_gene_261	199	WP_064085464.1	199	199/199(100.00)	2.87e-135	hypothetical protein [Wolbachia endosymbiont of Dactylopius coccus]	None	Unclassified
MDJ-2_gene_262	105	WP_064085463.1	105	104/105(99.05)	2.47e-68	ribosome silencing factor [Wolbachia endosymbiont of Dactylopius coccus]	None	Unclassified
MDJ-2_gene_264	420	WP_064085462.1	420	411/420(97.86)	5.35e-301	tyrosine--tRNA ligase [Wolbachia endosymbiont of Dactylopius coccus]	None	Unclassified
MDJ-2_gene_265	80	OAM06169.1	81	79/80(98.75)	8.58e-42	hypothetical protein TV41_00320 [Wolbachia endosymbiont of Dactylopius coccus]	1605993	d__Bacteria;k__norank;p__Proteobacteria;c__Alphaproteobacteria;o__Rickettsiales;f__Anaplasmataceae;g__Wolbachia;s__Wolbachia_endosymbiont_of_Dactylopius_coccus
MDJ-2_gene_266	96	CCE77484.1	287	91/95(95.79)	2.89e-53	conserved hypothetical protein [Wolbachia pipientis wAlbB]	1116230	d__Bacteria;k__norank;p__Proteobacteria;c__Alphaproteobacteria;o__Rickettsiales;f__Anaplasmataceae;g__Wolbachia;s__Wolbachia_pipientis
MDJ-2_gene_268	387	WP_063631131.1	407	346/384(90.10)	7.03e-181	hypothetical protein [Wolbachia endosymbiont of Laodelphax striatella]	2640676	d__Bacteria;k__norank;p__Proteobacteria;c__Alphaproteobacteria;o__Rickettsiales;f__Anaplasmataceae;g__Wolbachia;s__norank
MDJ-2_gene_269	426	AGJ99342.1	426	423/426(99.30)	2.05e-308	4-hydroxy-3-methylbut-2-en-1-yl diphosphate synthase [Wolbachia endosymbiont of Drosophila simulans wNo]	1236908	d__Bacteria;k__norank;p__Proteobacteria;c__Alphaproteobacteria;o__Rickettsiales;f__Anaplasmataceae;g__Wolbachia;s__Wolbachia_endosymbiont_of_Drosophila_simulans
MDJ-2_gene_270	211	AGJ99341.1	212	195/212(91.98)	3.62e-126	hypothetical protein wNo_09630 [Wolbachia endosymbiont of Drosophila simulans wNo]	1236908	d__Bacteria;k__norank;p__Proteobacteria;c__Alphaproteobacteria;o__Rickettsiales;f__Anaplasmataceae;g__Wolbachia;s__Wolbachia_endosymbiont_of_Drosophila_simulans
MDJ-2_gene_271	181	WP_127464185.1	182	157/163(96.32)	1.06e-59	hypothetical protein [Wolbachia endosymbiont of Bemisia tabaci]	215173	d__Bacteria;k__norank;p__Proteobacteria;c__Alphaproteobacteria;o__Rickettsiales;f__Anaplasmataceae;g__Wolbachia;s__Wolbachia_endosymbiont_of_Bemisia_tabaci
MDJ-2_gene_272	689	PCG11086.1	703	678/689(98.40)	0	cell division protein FtsK [Wolbachia pipientis]	None	Unclassified
MDJ-2_gene_274	59	BBA84692.1	202	39/59(66.10)	7.59e-14	50S ribosomal protein L4 [endosymbiont of Euscepes postfasciatus]	650377	d__Bacteria;k__norank;p__Proteobacteria;c__Gammaproteobacteria;o__norank;f__norank;g__norank;s__endosymbiont_of_Euscepes_postfasciatus
MDJ-2_gene_275	47	BBA84693.1	208	27/44(61.36)	4.67e-10	50S ribosomal protein L3 [endosymbiont of Euscepes postfasciatus]	650377	d__Bacteria;k__norank;p__Proteobacteria;c__Gammaproteobacteria;o__norank;f__norank;g__norank;s__endosymbiont_of_Euscepes_postfasciatus
MDJ-2_gene_276	127	BBA85265.1	206	62/124(50.00)	1.51e-26	50S ribosomal protein L3 [endosymbiont of Sipalinus gigas]	1972134	d__Bacteria;k__norank;p__Proteobacteria;c__Gammaproteobacteria;o__norank;f__norank;g__Candidatus_Nardonella;s__endosymbiont_of_Sipalinus_gigas

MDJ-2_gene_280	51	RZI64264.1	56	43/51(84.31)	2.55e-22	hypothetical protein EOP13_32710, partial	306	d__Bacteria;k__norank;p__Proteobacteria;c__Gammaproteobacteria;o__Pseudomonadales;f__Pseudomonadaceae;g__Pseudomonas;s__Pseudomonas_sp.
MDJ-2_gene_282	102	WP_080872942.1	156	58/102(56.86)	2.27e-32	30S ribosomal protein S7 [bacterium endosymbiont of	1719126	d__Bacteria;k__norank;p__norank;c__norank;o__norank;f__norank;g__norank;s__bacterium_endosymbiont_of_Pedicinus_badii
MDJ-2_gene_283	123	BBA85269.1	124	105/122(86.07)	4.94e-70	30S ribosomal protein S12 [endosymbiont of Sipalinus gigas]	1972134	d__Bacteria;k__norank;p__Proteobacteria;c__Gammaproteobacteria;o__norank;f__norank;g__Candidatus_Nardonella;s__endosymbiont_of_Sipalinus_gigas
MDJ-2_gene_284	114	PIP79808.1	144	68/114(59.65)	1.44e-36	DNA-directed RNA polymerase subunit beta', partial [Gammaproteobacteria bacterium CG22_combo_CG10-13_8_21_14_all_40_8]	1974025	d__Bacteria;k__norank;p__Proteobacteria;c__Gammaproteobacteria;o__norank;f__norank;g__norank;s__Gammaproteobacteria_bacterium_CG22_combo_CG10-13_8_21_14_all_40_8
MDJ-2_gene_285	494	ANF16857.1	1403	280/475(58.95)	4.76e-178	DNA-directed RNA polymerase subunit beta' [Buchnera aphidicola (Schlechtendalia chinensis)]	118110	d__Bacteria;k__norank;p__Proteobacteria;c__Gammaproteobacteria;o__Enterobacterales;f__Erwiniaceae;g__Buchnera;s__Buchnera_aphidicola
MDJ-2_gene_286	237	WP_130617011.1	1350	138/238(57.98)	1.32e-81	DNA-directed RNA polymerase subunit beta' [endosymbiont of Sipalinus gigas]	1972134	d__Bacteria;k__norank;p__Proteobacteria;c__Gammaproteobacteria;o__norank;f__norank;g__Candidatus_Nardonella;s__endosymbiont_of_Sipalinus_gigas
MDJ-2_gene_290	400	WP_017531652.1	606	388/398(97.49)	1.36e-267	hypothetical protein [Wolbachia endosymbiont of Diaphorina citri]	116598	d__Bacteria;k__norank;p__Proteobacteria;c__Alphaproteobacteria;o__Rickettsiales;f__Anaplasmataceae;g__Wolbachia;s__Wolbachia_endosymbiont_of_Diaphorina_citri
MDJ-2_gene_291	211	WP_017531652.1	606	163/210(77.62)	1.52e-89	hypothetical protein [Wolbachia endosymbiont of Diaphorina citri]	116598	d__Bacteria;k__norank;p__Proteobacteria;c__Alphaproteobacteria;o__Rickettsiales;f__Anaplasmataceae;g__Wolbachia;s__Wolbachia_endosymbiont_of_Diaphorina_citri
MDJ-2_gene_292	245	PCG11534.1	245	244/245(99.59)	2.24e-173	ABC transporter ATP-binding protein [Wolbachia pipientis]	None	Unclassified
MDJ-2_gene_293	55	ONI57290.1	55	54/55(98.18)	2.31e-28	twin arginine-targeting protein translocase, TatA/E family protein [Wolbachia pipientis	1379786	d__Bacteria;k__norank;p__Proteobacteria;c__Alphaproteobacteria;o__Rickettsiales;f__Anaplasmataceae;g__Wolbachia;s__Wolbachia_pipientis
MDJ-2_gene_294	218	WP_096097193.1	218	217/218(99.54)	1.02e-142	exopolysaccharide biosynthesis protein [Wolbachia pipientis]	955	d__Bacteria;k__norank;p__Proteobacteria;c__Alphaproteobacteria;o__Rickettsiales;f__Anaplasmataceae;g__Wolbachia;s__Wolbachia_pipientis
MDJ-2_gene_297	118	PBQ27617.1	118	110/117(94.02)	3.31e-70	hypothetical protein BTO27_03425 [Wolbachia	2049495	d__Bacteria;k__norank;p__Proteobacteria;c__Alphaproteobacteria;o__Rickettsiales;f__Anaplasmataceae;g__Wolbachia;s__Wolbachia_pipientis
MDJ-2_gene_298	332	WP_063631208.1	332	326/332(98.19)	1.26e-234	pyruvate dehydrogenase complex E1 component subunit beta [Wolbachia endosymbiont of Laodelphax striatella]	368602	d__Bacteria;k__norank;p__Proteobacteria;c__Alphaproteobacteria;o__Rickettsiales;f__Anaplasmataceae;g__Wolbachia;s__Wolbachia_endosymbiont_of_Laodelphax_striatellus
MDJ-2_gene_299	445	WP_068651503.1	445	401/445(90.11)	5.82e-265	hypothetical protein [Wolbachia endosymbiont of Trichogramma pretiosum]	125593	d__Bacteria;k__norank;p__Proteobacteria;c__Alphaproteobacteria;o__Rickettsiales;f__Anaplasmataceae;g__Wolbachia;s__Wolbachia_endosymbiont_of_Trichogramma_pretiosum
MDJ-2_gene_300	937	WP_010405033.1	937	935/937(99.79)	0	excinuclease ABC subunit UvrA [Wolbachia endosymbiont of Nasonia vitripennis]	180837	d__Bacteria;k__norank;p__Proteobacteria;c__Alphaproteobacteria;o__Rickettsiales;f__Anaplasmataceae;g__Wolbachia;s__Wolbachia_endosymbiont_of_Nasonia_vitripennis
MDJ-2_gene_301	234	WP_017532560.1	234	231/234(98.72)	1.51e-161	ComF family protein [Wolbachia endosymbiont of Diaphorina citri]	116598	d__Bacteria;k__norank;p__Proteobacteria;c__Alphaproteobacteria;o__Rickettsiales;f__Anaplasmataceae;g__Wolbachia;s__Wolbachia_endosymbiont_of_Diaphorina_citri
MDJ-2_gene_302	430	CAQ54742.1	487	391/431(90.72)	7.28e-242	hypothetical protein WP0634 [Wolbachia endosymbiont of Culex quinquefasciatus Pel]	570417	d__Bacteria;k__norank;p__Proteobacteria;c__Alphaproteobacteria;o__Rickettsiales;f__Anaplasmataceae;g__Wolbachia;s__Wolbachia_endosymbiont_of_Culex_quinquefasciatus
MDJ-2_gene_303	121	PBQ27264.1	121	119/121(98.35)	3.02e-72	hypothetical protein BTO27_03685 [Wolbachia	2049495	d__Bacteria;k__norank;p__Proteobacteria;c__Alphaproteobacteria;o__Rickettsiales;f__Anaplasmataceae;g__Wolbachia;s__Wolbachia_pipientis
MDJ-2_gene_304	784	WP_017532162.1	784	776/784(98.98)	0	type VI secretion protein [Wolbachia endosymbiont of	116598	d__Bacteria;k__norank;p__Proteobacteria;c__Alphaproteobacteria;o__Rickettsiales;f__Anaplasmataceae;g__Wolbachia;s__Wolbachia_endosymbiont_of_Diaphorina_citri

MDJ-2_gene_305	944	PCG11227.1	942	822/949(86.62)	0	hypothetical protein CO539_04370 [Wolbachia	None	Unclassified
MDJ-2_gene_307	315	PBQ25580.1	315	311/315(98.73)	3.54e-221	thioredoxin-disulfide reductase [Wolbachia pipientis wAus]	2049495	d__Bacteria;k__norank;p__Proteobacteria;c__Alphaproteobacteria;o__Rickettsiales;f__Anaplasmataceae;g__Wolbachia;s__Wolbachia_pipientis
MDJ-2_gene_308	479	WP_108784652.1	384	297/379(78.36)	3.44e-192	ankyrin repeat domain-containing protein [Wolbachia endosymbiont of Bemisia tabaci]	215173	d__Bacteria;k__norank;p__Proteobacteria;c__Alphaproteobacteria;o__Rickettsiales;f__Anaplasmataceae;g__Wolbachia;s__Wolbachia_endosymbiont_of_Bemisia_tabaci
MDJ-2_gene_309	454	WP_108784125.1	473	451/454(99.34)	0	2-polyprenylphenol 6-hydroxylase [Wolbachia endosymbiont of Bemisia tabaci]	215173	d__Bacteria;k__norank;p__Proteobacteria;c__Alphaproteobacteria;o__Rickettsiales;f__Anaplasmataceae;g__Wolbachia;s__Wolbachia_endosymbiont_of_Bemisia_tabaci
MDJ-2_gene_310	243	WP_010401464.1	243	238/243(97.94)	5.21e-162	metal ABC transporter ATP-binding protein [Wolbachia endosymbiont of Nasonia	2640676	d__Bacteria;k__norank;p__Proteobacteria;c__Alphaproteobacteria;o__Rickettsiales;f__Anaplasmataceae;g__Wolbachia;s__norank
MDJ-2_gene_312	338	WP_064085486.1	338	334/338(98.82)	5.36e-239	uroporphyrinogen decarboxylase [Wolbachia endosymbiont of Dactylopius coccus]	None	Unclassified
MDJ-2_gene_313	52	CAQ54950.1	52	43/52(82.69)	1.02e-23	Hypothetical protein WP0842 [Wolbachia endosymbiont of Culex quinquefasciatus Pel]	570417	d__Bacteria;k__norank;p__Proteobacteria;c__Alphaproteobacteria;o__Rickettsiales;f__Anaplasmataceae;g__Wolbachia;s__Wolbachia_endosymbiont_of_Culex_quinquefasciatus
MDJ-2_gene_314	390	WP_064085485.1	397	379/390(97.18)	7.41e-274	pyridoxal phosphate-dependent aminotransferase [Wolbachia endosymbiont of Dactylopius coccus]	None	Unclassified
MDJ-2_gene_315	105	PBQ28035.1	105	104/105(99.05)	1.09e-62	QacE family quaternary ammonium compound efflux SMR transporter [Wolbachia	2049495	d__Bacteria;k__norank;p__Proteobacteria;c__Alphaproteobacteria;o__Rickettsiales;f__Anaplasmataceae;g__Wolbachia;s__Wolbachia_pipientis
MDJ-2_gene_316	169	PCG11545.1	169	164/169(97.04)	1.17e-108	hypothetical protein CO539_02565 [Wolbachia	None	Unclassified
MDJ-2_gene_318	425	PCG16978.1	425	406/425(95.53)	4.90e-295	serine hydroxymethyltransferase [Wolbachia pipientis]	None	Unclassified
MDJ-2_gene_319	62	PCG11424.1	396	62/62(100.00)	7.64e-33	phosphoglycerate kinase [Wolbachia pipientis]	None	Unclassified
MDJ-2_gene_320	453	RLT60954.1	656	402/456(88.16)	1.90e-284	wolbachia palindromic element domain protein [Wolbachia endosymbiont of Drosophila ananassae]	307502	d__Bacteria;k__norank;p__Proteobacteria;c__Alphaproteobacteria;o__Rickettsiales;f__Anaplasmataceae;g__Wolbachia;s__Wolbachia_endosymbiont_of_Drosophila_ananassae
MDJ-2_gene_321	416	WP_108784321.1	416	384/416(92.31)	1.87e-267	hypothetical protein [Wolbachia endosymbiont of Bemisia tabaci]	215173	d__Bacteria;k__norank;p__Proteobacteria;c__Alphaproteobacteria;o__Rickettsiales;f__Anaplasmataceae;g__Wolbachia;s__Wolbachia_endosymbiont_of_Bemisia_tabaci
MDJ-2_gene_322	226	PBQ29285.1	226	218/226(96.46)	9.43e-152	hypothetical protein BTO27_00475 [Wolbachia	2049495	d__Bacteria;k__norank;p__Proteobacteria;c__Alphaproteobacteria;o__Rickettsiales;f__Anaplasmataceae;g__Wolbachia;s__Wolbachia_pipientis
MDJ-2_gene_323	368	WP_096560716.1	368	364/368(98.91)	4.90e-257	D-alanyl-D-alanine carboxypeptidase [Wolbachia	955	d__Bacteria;k__norank;p__Proteobacteria;c__Alphaproteobacteria;o__Rickettsiales;f__Anaplasmataceae;g__Wolbachia;s__Wolbachia_pipientis
MDJ-2_gene_326	70	ONI56798.1	70	70/70(100.00)	4.99e-43	hypothetical protein N500_0117 [Wolbachia pipientis wUni]	1379787	d__Bacteria;k__norank;p__Proteobacteria;c__Alphaproteobacteria;o__Rickettsiales;f__Anaplasmataceae;g__Wolbachia;s__Wolbachia_pipientis
MDJ-2_gene_327	682	CQD05673.1	689	672/682(98.53)	0	NADH dehydrogenase subunit G [Wolbachia endosymbiont wPip_Mol of Culex molestus]	1402163	d__Bacteria;k__norank;p__Proteobacteria;c__Alphaproteobacteria;o__Rickettsiales;f__Anaplasmataceae;g__Wolbachia;s__Wolbachia_endosymbiont_of_Culex_molestus
MDJ-2_gene_328	149	WP_010404400.1	149	147/149(98.66)	1.41e-88	MULTISPECIES: hypothetical protein [Wolbachia]	2640676	d__Bacteria;k__norank;p__Proteobacteria;c__Alphaproteobacteria;o__Rickettsiales;f__Anaplasmataceae;g__Wolbachia;s__norank

MDJ-2_gene_329	341	WP_010404403.1	341	337/341(98.83)	1.10e-228	NADH-quinone oxidoreductase subunit NuoH [Wolbachia endosymbiont of Nasonia vitripennis]	180837	d__Bacteria;k__norank;p__Proteobacteria;c__Alphaproteobacteria;o__Rickettsiales;f__Anaplasmataceae;g__Wolbachia;s__Wolbachia_endosymbiont_of_Nasonia_vitripennis
MDJ-2_gene_330	332	WP_010404406.1	332	321/332(96.69)	1.64e-230	porphobilinogen synthase [Wolbachia endosymbiont of Nasonia vitripennis]	180837	d__Bacteria;k__norank;p__Proteobacteria;c__Alphaproteobacteria;o__Rickettsiales;f__Anaplasmataceae;g__Wolbachia;s__Wolbachia_endosymbiont_of_Nasonia_vitripennis
MDJ-2_gene_332	255	AGJ99071.1	255	252/255(98.82)	2.00e-181	RNA methyltransferase, TrmH family, group 3 [Wolbachia endosymbiont of Drosophila simulans wNo]	1236908	d__Bacteria;k__norank;p__Proteobacteria;c__Alphaproteobacteria;o__Rickettsiales;f__Anaplasmataceae;g__Wolbachia;s__Wolbachia_endosymbiont_of_Drosophila_simulans
MDJ-2_gene_333	163	PCG11394.1	163	162/163(99.39)	1.23e-116	DNA mismatch repair protein MutS [Wolbachia pipientis]	None	Unclassified
MDJ-2_gene_334	605	PCG11395.1	605	596/605(98.51)	0	excinuclease ABC subunit UvrC [Wolbachia pipientis]	None	Unclassified
MDJ-2_gene_335	702	WP_064085630.1	702	672/702(95.73)	0	glycine--tRNA ligase subunit beta [Wolbachia endosymbiont of Dactylopius coccus]	None	Unclassified
MDJ-2_gene_336	279	WP_019236899.1	279	274/279(98.21)	1.80e-205	MULTISPECIES: glycine--tRNA ligase subunit alpha [Wolbachia]	953	d__Bacteria;k__norank;p__Proteobacteria;c__Alphaproteobacteria;o__Rickettsiales;f__Anaplasmataceae;g__Wolbachia;s__norank
MDJ-2_gene_339	481	WP_010404618.1	481	475/481(98.75)	0	F0F1 ATP synthase subunit beta [Wolbachia endosymbiont of Nasonia vitripennis]	None	Unclassified
MDJ-2_gene_340	113	PCG11301.1	113	104/113(92.04)	7.38e-67	hypothetical protein CO539_03955 [Wolbachia	None	Unclassified
MDJ-2_gene_341	916	EEB56532.1	1118	725/1123(64.56)	3.78e-254	hypothetical protein C1A_714 [Wolbachia endosymbiont of Culex quinquefasciatus JHB]	569881	d__Bacteria;k__norank;p__Proteobacteria;c__Alphaproteobacteria;o__Rickettsiales;f__Anaplasmataceae;g__Wolbachia;s__Wolbachia_endosymbiont_of_Culex_quinquefasciatus
MDJ-2_gene_342	213	PBQ26117.1	214	205/213(96.24)	6.17e-144	fructose-6-phosphate aldolase [Wolbachia pipientis wAus]	2049495	d__Bacteria;k__norank;p__Proteobacteria;c__Alphaproteobacteria;o__Rickettsiales;f__Anaplasmataceae;g__Wolbachia;s__Wolbachia_pipientis
MDJ-2_gene_344	265	RDD34710.1	272	166/215(77.21)	1.41e-109	Transposase [Wolbachia endosymbiont of Cylisticus convexus]	118728	d__Bacteria;k__norank;p__Proteobacteria;c__Alphaproteobacteria;o__Rickettsiales;f__Anaplasmataceae;g__Wolbachia;s__Wolbachia_endosymbiont_of_Cylisticus_convexus
MDJ-2_gene_345	390	WP_064085656.1	390	388/390(99.49)	2.27e-264	2-oxoglutarate dehydrogenase complex dihydrolipoyllysine-residue succinyltransferase [Wolbachia endosymbiont of Dactylopius coccus]	None	Unclassified
MDJ-2_gene_346	495	CCE77373.1	557	413/498(82.93)	9.66e-146	conserved hypothetical protein [Wolbachia pipientis wAlbB]	1116230	d__Bacteria;k__norank;p__Proteobacteria;c__Alphaproteobacteria;o__Rickettsiales;f__Anaplasmataceae;g__Wolbachia;s__Wolbachia_pipientis
MDJ-2_gene_347	231	WP_063630495.1	231	229/231(99.13)	4.73e-168	MULTISPECIES: di-trans,poly-cis-decaprenylcistransferase [Wolbachia]	2640676	d__Bacteria;k__norank;p__Proteobacteria;c__Alphaproteobacteria;o__Rickettsiales;f__Anaplasmataceae;g__Wolbachia;s__norank
MDJ-2_gene_349	69	PCG11402.1	69	69/69(100.00)	2.39e-45	zinc-finger domain-containing protein [Wolbachia pipientis]	None	Unclassified
MDJ-2_gene_350	205	WP_010401448.1	205	204/205(99.51)	1.11e-135	RDD family protein [Wolbachia endosymbiont of Nasonia vitripennis]	180837	d__Bacteria;k__norank;p__Proteobacteria;c__Alphaproteobacteria;o__Rickettsiales;f__Anaplasmataceae;g__Wolbachia;s__Wolbachia_endosymbiont_of_Nasonia_vitripennis

MDJ-2_gene_351	480	WP_064085592.1	480	478/480(99.58)	0	replicative DNA helicase [Wolbachia endosymbiont of Dactylopius coccus]	None	Unclassified
MDJ-2_gene_352	498	WP_010407433.1	498	496/498(99.60)	0	cation:proton antiporter [Wolbachia endosymbiont of Nasonia vitripennis]	2640676	d__Bacteria;k__norank;p__Proteobacteria;c__Alphaproteobacteria;o__Rickettsiales;f__Anaplasmataceae;g__Wolbachia;s__norank
MDJ-2_gene_353	219	WP_039833963.1	221	216/221(97.74)	6.83e-142	conjugal transfer protein TraJ [Wolbachia endosymbiont of Nasonia vitripennis]	180837	d__Bacteria;k__norank;p__Proteobacteria;c__Alphaproteobacteria;o__Rickettsiales;f__Anaplasmataceae;g__Wolbachia;s__Wolbachia_endosymbiont_of_Nasonia_vitripennis
MDJ-2_gene_354	264	WP_127464173.1	264	254/264(96.21)	4.35e-185	hypothetical protein [Wolbachia endosymbiont of Bemisia tabaci]	215173	d__Bacteria;k__norank;p__Proteobacteria;c__Alphaproteobacteria;o__Rickettsiales;f__Anaplasmataceae;g__Wolbachia;s__Wolbachia_endosymbiont_of_Bemisia_tabaci
MDJ-2_gene_355	283	BAH22268.1	283	268/283(94.70)	4.32e-190	putative DNA polymerase III, gamma/tau subunits [Wolbachia endosymbiont of Cadra cautella]	190193	d__Bacteria;k__norank;p__Proteobacteria;c__Alphaproteobacteria;o__Rickettsiales;f__Anaplasmataceae;g__Wolbachia;s__Wolbachia_endosymbiont_of_Cadra_cautella
MDJ-2_gene_358	476	WP_019236421.1	469	442/469(94.24)	0	phage portal protein [Wolbachia pipientis]	955	d__Bacteria;k__norank;p__Proteobacteria;c__Alphaproteobacteria;o__Rickettsiales;f__Anaplasmataceae;g__Wolbachia;s__Wolbachia_pipientis
MDJ-2_gene_359	358	AGJ99822.1	344	267/354(75.42)	8.76e-178	Putative minor capsid protein C [Wolbachia endosymbiont of Drosophila simulans wHa]	1236909	d__Bacteria;k__norank;p__Proteobacteria;c__Alphaproteobacteria;o__Rickettsiales;f__Anaplasmataceae;g__Wolbachia;s__Wolbachia_endosymbiont_of_Drosophila_simulans
MDJ-2_gene_360	124	WP_114517770.1	124	107/123(86.99)	2.09e-69	head decoration protein [Wolbachia endosymbiont of Cylisticus convexus]	118728	d__Bacteria;k__norank;p__Proteobacteria;c__Alphaproteobacteria;o__Rickettsiales;f__Anaplasmataceae;g__Wolbachia;s__Wolbachia_endosymbiont_of_Cylisticus_convexus
MDJ-2_gene_361	334	OJH30460.1	334	320/333(96.10)	4.58e-238	hypothetical protein Wxf_03088 [Armadillidium vulgare]	77039	d__Bacteria;k__norank;p__Proteobacteria;c__Alphaproteobacteria;o__Rickettsiales;f__Anaplasmataceae;g__Wolbachia;s__Wolbachia_endosymbiont_of_Armadillidium_vulgare
MDJ-2_gene_362	103	WP_114517876.1	106	87/99(87.88)	2.35e-59	hypothetical protein [Wolbachia endosymbiont of Cylisticus convexus]	118728	d__Bacteria;k__norank;p__Proteobacteria;c__Alphaproteobacteria;o__Rickettsiales;f__Anaplasmataceae;g__Wolbachia;s__Wolbachia_endosymbiont_of_Cylisticus_convexus
MDJ-2_gene_364	154	RDD34057.1	175	119/153(77.78)	6.64e-85	hypothetical protein Wcon_01904 [Wolbachia endosymbiont of Cylisticus convexus]	118728	d__Bacteria;k__norank;p__Proteobacteria;c__Alphaproteobacteria;o__Rickettsiales;f__Anaplasmataceae;g__Wolbachia;s__Wolbachia_endosymbiont_of_Cylisticus_convexus
MDJ-2_gene_365	189	WP_114517904.1	188	170/186(91.40)	4.44e-102	phage baseplate assembly protein V [Wolbachia endosymbiont of Cylisticus convexus]	118728	d__Bacteria;k__norank;p__Proteobacteria;c__Alphaproteobacteria;o__Rickettsiales;f__Anaplasmataceae;g__Wolbachia;s__Wolbachia_endosymbiont_of_Cylisticus_convexus
MDJ-2_gene_368	221	WP_064085980.1	221	216/221(97.74)	4.04e-155	TenA family transcriptional regulator [Wolbachia endosymbiont of Dactylopius	None	Unclassified
MDJ-2_gene_369	219	PCG11579.1	219	215/219(98.17)	1.41e-154	TenA family transcriptional regulator [Wolbachia pipientis]	None	Unclassified
MDJ-2_gene_370	283	WP_127464095.1	275	271/275(98.55)	4.26e-195	cytochrome c oxidase subunit 3 [Wolbachia endosymbiont of Bemisia tabaci]	215173	d__Bacteria;k__norank;p__Proteobacteria;c__Alphaproteobacteria;o__Rickettsiales;f__Anaplasmataceae;g__Wolbachia;s__Wolbachia_endosymbiont_of_Bemisia_tabaci
MDJ-2_gene_371	162	WP_127464096.1	162	161/162(99.38)	4.30e-112	crossover junction endodeoxyribonuclease RuvC [Wolbachia endosymbiont of Bemisia tabaci]	215173	d__Bacteria;k__norank;p__Proteobacteria;c__Alphaproteobacteria;o__Rickettsiales;f__Anaplasmataceae;g__Wolbachia;s__Wolbachia_endosymbiont_of_Bemisia_tabaci
MDJ-2_gene_373	594	WP_064085975.1	594	590/594(99.33)	0	ribonucleoside-diphosphate reductase subunit alpha [Wolbachia endosymbiont of Dactylopius coccus]	None	Unclassified

MDJ-2_gene_374	356	WP_068651701.1	356	345/356(96.91)	2.85e-259	methyltransferase domain-containing protein [Wolbachia endosymbiont of Trichogramma	125593	d__Bacteria;k__norank;p__Proteobacteria;c__Alphaproteobacteria;o__Rickettsiales;f__Anaplasmataceae;g__Wolbachia;s__Wolbachia_endosymbiont_of_Trichogramma_pretiosum
MDJ-2_gene_375	218	WP_017531719.1	689	154/218(70.64)	1.36e-99	hypothetical protein [Wolbachia endosymbiont of Diaphorina citri]	116598	d__Bacteria;k__norank;p__Proteobacteria;c__Alphaproteobacteria;o__Rickettsiales;f__Anaplasmataceae;g__Wolbachia;s__Wolbachia_endosymbiont_of_Diaphorina_citri
MDJ-2_gene_379	207	CAQ54504.1	240	184/206(89.32)	2.89e-126	putative site-specific recombinase, resolvase family [Wolbachia endosymbiont of Culex quinquefasciatus Pel]	570417	d__Bacteria;k__norank;p__Proteobacteria;c__Alphaproteobacteria;o__Rickettsiales;f__Anaplasmataceae;g__Wolbachia;s__Wolbachia_endosymbiont_of_Culex_quinquefasciatus
MDJ-2_gene_381	561	WP_019236321.1	854	546/561(97.33)	0	valine--tRNA ligase [Wolbachia pipientis]	955	d__Bacteria;k__norank;p__Proteobacteria;c__Alphaproteobacteria;o__Rickettsiales;f__Anaplasmataceae;g__Wolbachia;s__Wolbachia_pipientis
MDJ-2_gene_382	218	WP_064085916.1	217	175/217(80.65)	3.60e-106	hypothetical protein [Wolbachia endosymbiont of Dactylopius coccus]	None	Unclassified
MDJ-2_gene_383	331	CCE77406.1	334	329/331(99.40)	2.41e-230	conserved membrane hypothetical protein [Wolbachia pipientis	1116230	d__Bacteria;k__norank;p__Proteobacteria;c__Alphaproteobacteria;o__Rickettsiales;f__Anaplasmataceae;g__Wolbachia;s__Wolbachia_pipientis
MDJ-2_gene_385	369	WP_064085945.1	369	368/369(99.73)	1.39e-252	dehydrogenase [Wolbachia endosymbiont of Dactylopius coccus]	None	Unclassified
MDJ-2_gene_386	459	WP_010401379.1	459	451/459(98.26)	0	dihydrolipoyl dehydrogenase [Wolbachia endosymbiont of Nasonia vitripennis]	2640676	d__Bacteria;k__norank;p__Proteobacteria;c__Alphaproteobacteria;o__Rickettsiales;f__Anaplasmataceae;g__Wolbachia;s__norank
MDJ-2_gene_389	181	BBA85190.1	285	92/176(52.27)	6.23e-48	DNA polymerase I [endosymbiont of Sipalinus gigas]	1972134	d__Bacteria;k__norank;p__Proteobacteria;c__Gammaproteobacteria;o__norank;f__norank;g__Candidatus_Nardonella;s__endosymbiont_of_Sipalinus_gigas
MDJ-2_gene_390	140	BBA84566.1	144	82/138(59.42)	4.92e-44	marR family transcriptional regulator [endosymbiont of Euscepes postfasciatus]	650377	d__Bacteria;k__norank;p__Proteobacteria;c__Gammaproteobacteria;o__norank;f__norank;g__norank;s__endosymbiont_of_Euscepes_postfasciatus
MDJ-2_gene_392	190	BBA84988.1	477	80/173(46.24)	1.41e-28	tRNA(Ile)-lysidine synthase [endosymbiont of Rhynchophorus ferrugineus]	1972133	d__Bacteria;k__norank;p__Proteobacteria;c__Gammaproteobacteria;o__norank;f__norank;g__Candidatus_Nardonella;s__Candidatus_Nardonella_dryophthoridicola
MDJ-2_gene_394	381	AFA41297.1	419	302/381(79.27)	5.95e-214	transcription termination factor [Wigglesworthia glossinidia endosymbiont of Glossina morsitans morsitans (Yale	1142511	d__Bacteria;k__norank;p__Proteobacteria;c__Gammaproteobacteria;o__Enterobacterales;f__Erwiniaceae;g__Wigglesworthia;s__Wigglesworthia_glossinidia
MDJ-2_gene_395	78	PPI86366.1	112	41/74(55.41)	2.91e-15	hypothetical protein CRV10_03375 [Pantoea sp.	2044610	d__Bacteria;k__norank;p__Proteobacteria;c__Gammaproteobacteria;o__Enterobacterales;f__Erwiniaceae;g__Pantoea;s__Pantoea_sp._SoEL
MDJ-2_gene_398	488	PCG11287.1	488	485/488(99.39)	0	amino acid carrier protein [Wolbachia pipientis]	None	Unclassified
MDJ-2_gene_399	444	WP_010404445.1	444	443/444(99.77)	6.45e-296	MULTISPECIES: amino acid carrier protein [Wolbachia]	2640676	d__Bacteria;k__norank;p__Proteobacteria;c__Alphaproteobacteria;o__Rickettsiales;f__Anaplasmataceae;g__Wolbachia;s__norank
MDJ-2_gene_402	126	WP_019236893.1	288	106/116(91.38)	3.81e-35	MULTISPECIES: ankyrin repeat domain-containing protein [Wolbachia]	953	d__Bacteria;k__norank;p__Proteobacteria;c__Alphaproteobacteria;o__Rickettsiales;f__Anaplasmataceae;g__Wolbachia;s__norank
MDJ-2_gene_403	262	PCG11685.1	262	249/262(95.04)	3.57e-183	hypothetical protein CO539_01730 [Wolbachia	None	Unclassified
MDJ-2_gene_404	202	PBQ26334.1	202	196/202(97.03)	3.19e-129	hypothetical protein BTO27_04655 [Wolbachia	2049495	d__Bacteria;k__norank;p__Proteobacteria;c__Alphaproteobacteria;o__Rickettsiales;f__Anaplasmataceae;g__Wolbachia;s__Wolbachia_pipientis
MDJ-2_gene_405	479	WP_039833900.1	486	462/479(96.45)	0	hypothetical protein [Wolbachia endosymbiont of Nasonia vitripennis]	None	Unclassified

MDJ-2_gene_406	308	PBQ26336.1	308	304/308(98.70)	1.33e-217	4-hydroxy-3-methylbut-2-enyl diphosphate reductase [Wolbachia pipientis wAus]	2049495	d__Bacteria;k__norank;p__Proteobacteria;c__Alphaproteobacteria;o__Rickettsiales;f__Anaplasmataceae;g__Wolbachia;s__Wolbachia_pipientis
MDJ-2_gene_408	232	WP_108784616.1	232	224/232(96.55)	1.10e-153	isoprenoid biosynthesis glyoxalase ElbB [Wolbachia endosymbiont of Bemisia tabaci]	215173	d__Bacteria;k__norank;p__Proteobacteria;c__Alphaproteobacteria;o__Rickettsiales;f__Anaplasmataceae;g__Wolbachia;s__Wolbachia_endosymbiont_of_Bemisia_tabaci
MDJ-2_gene_409	424	WP_077188444.1	345	221/330(66.97)	9.45e-122	hypothetical protein [Wolbachia pipientis]	955	d__Bacteria;k__norank;p__Proteobacteria;c__Alphaproteobacteria;o__Rickettsiales;f__Anaplasmataceae;g__Wolbachia;s__Wolbachia_pipientis
MDJ-2_gene_410	370	WP_010402764.1	1106	360/370(97.30)	2.21e-246	DNA polymerase III subunit alpha [Wolbachia endosymbiont of Nasonia vitripennis]	180837	d__Bacteria;k__norank;p__Proteobacteria;c__Alphaproteobacteria;o__Rickettsiales;f__Anaplasmataceae;g__Wolbachia;s__Wolbachia_endosymbiont_of_Nasonia_vitripennis
MDJ-2_gene_411	328	OAM05911.1	383	282/382(73.82)	1.51e-99	hypothetical protein TV41_01775 [Wolbachia endosymbiont of Dactylopius coccus]	1605993	d__Bacteria;k__norank;p__Proteobacteria;c__Alphaproteobacteria;o__Rickettsiales;f__Anaplasmataceae;g__Wolbachia;s__Wolbachia_endosymbiont_of_Dactylopius_coccus
MDJ-2_gene_412	401	WP_108784650.1	319	290/302(96.03)	8.58e-211	LD-carboxypeptidase [Wolbachia endosymbiont of Bemisia tabaci]	215173	d__Bacteria;k__norank;p__Proteobacteria;c__Alphaproteobacteria;o__Rickettsiales;f__Anaplasmataceae;g__Wolbachia;s__Wolbachia_endosymbiont_of_Bemisia_tabaci
MDJ-2_gene_414	579	RDD35374.1	702	434/659(65.86)	1.87e-222	hypothetical protein Wcon_00439 [Wolbachia endosymbiont of Cylisticus convexus]	118728	d__Bacteria;k__norank;p__Proteobacteria;c__Alphaproteobacteria;o__Rickettsiales;f__Anaplasmataceae;g__Wolbachia;s__Wolbachia_endosymbiont_of_Cylisticus_convexus
MDJ-2_gene_416	138	WP_064085877.1	193	136/137(99.27)	2.96e-90	hypothetical protein [Wolbachia endosymbiont of Dactylopius coccus]	None	Unclassified
MDJ-2_gene_417	441	AGJ98950.1	441	436/441(98.87)	6.92e-310	GTP-binding protein EngA [Wolbachia endosymbiont of Drosophila simulans wNo]	1236908	d__Bacteria;k__norank;p__Proteobacteria;c__Alphaproteobacteria;o__Rickettsiales;f__Anaplasmataceae;g__Wolbachia;s__Wolbachia_endosymbiont_of_Drosophila_simulans
MDJ-2_gene_418	274	PBQ28225.1	274	261/274(95.26)	7.73e-184	hypothetical protein BTO27_02035 [Wolbachia	2049495	d__Bacteria;k__norank;p__Proteobacteria;c__Alphaproteobacteria;o__Rickettsiales;f__Anaplasmataceae;g__Wolbachia;s__Wolbachia_pipientis
MDJ-2_gene_419	508	WP_017531865.1	508	504/508(99.21)	0	acyl-CoA carboxylase subunit beta [Wolbachia endosymbiont of Diaphorina citri]	116598	d__Bacteria;k__norank;p__Proteobacteria;c__Alphaproteobacteria;o__Rickettsiales;f__Anaplasmataceae;g__Wolbachia;s__Wolbachia_endosymbiont_of_Diaphorina_citri
MDJ-2_gene_420	342	PBQ28230.1	263	204/207(98.55)	2.15e-138	peptidase M61 [Wolbachia pipientis wAus]	2049495	d__Bacteria;k__norank;p__Proteobacteria;c__Alphaproteobacteria;o__Rickettsiales;f__Anaplasmataceae;g__Wolbachia;s__Wolbachia_pipientis
MDJ-2_gene_421	237	WP_127464074.1	627	235/237(99.16)	1.31e-154	hypothetical protein [Wolbachia endosymbiont of Bemisia tabaci]	215173	d__Bacteria;k__norank;p__Proteobacteria;c__Alphaproteobacteria;o__Rickettsiales;f__Anaplasmataceae;g__Wolbachia;s__Wolbachia_endosymbiont_of_Bemisia_tabaci
MDJ-2_gene_422	298	CAQ55222.1	298	293/298(98.32)	9.84e-199	cation efflux family protein [Wolbachia endosymbiont of Culex quinquefasciatus Pel]	570417	d__Bacteria;k__norank;p__Proteobacteria;c__Alphaproteobacteria;o__Rickettsiales;f__Anaplasmataceae;g__Wolbachia;s__Wolbachia_endosymbiont_of_Culex_quinquefasciatus
MDJ-2_gene_423	1172	WP_064086020.1	1154	1049/1169(89.73)	0	hypothetical protein [Wolbachia endosymbiont of Dactylopius coccus]	None	Unclassified
MDJ-2_gene_427	69	WP_114517867.1	69	62/69(89.86)	5.08e-38	phage tail protein [Wolbachia endosymbiont of Cylisticus convexus]	118728	d__Bacteria;k__norank;p__Proteobacteria;c__Alphaproteobacteria;o__Rickettsiales;f__Anaplasmataceae;g__Wolbachia;s__Wolbachia_endosymbiont_of_Cylisticus_convexus
MDJ-2_gene_428	115	RDD34869.1	115	109/115(94.78)	7.03e-75	Phage protein U [Wolbachia endosymbiont of Cylisticus convexus]	118728	d__Bacteria;k__norank;p__Proteobacteria;c__Alphaproteobacteria;o__Rickettsiales;f__Anaplasmataceae;g__Wolbachia;s__Wolbachia_endosymbiont_of_Cylisticus_convexus
MDJ-2_gene_429	763	WP_114517780.1	713	666/713(93.41)	0	phage tail tape measure protein, partial [Wolbachia endosymbiont of Cylisticus convexus]	118728	d__Bacteria;k__norank;p__Proteobacteria;c__Alphaproteobacteria;o__Rickettsiales;f__Anaplasmataceae;g__Wolbachia;s__Wolbachia_endosymbiont_of_Cylisticus_convexus

MDJ-2_gene_430	85	WP_096097245.1	85	82/85(96.47)	2.79e-49	phage tail assembly protein [Wolbachia pipientis]	955	d__Bacteria;k__norank;p__Proteobacteria;c__Alphaproteobacteria;o__Rickettsiales;f__Anaplasmataceae;g__Wolbachia;s__Wolbachia_pipientis
MDJ-2_gene_432	390	WP_064124954.1	393	364/393(92.62)	6.25e-265	phage tail protein [Wolbachia endosymbiont of Dactylopius coccus]	None	Unclassified
MDJ-2_gene_433	194	WP_065094986.1	186	158/186(84.95)	4.54e-105	MULTISPECIES: hypothetical protein [Wolbachia]	2640676	d__Bacteria;k__norank;p__Proteobacteria;c__Alphaproteobacteria;o__Rickettsiales;f__Anaplasmataceae;g__Wolbachia;s__norank
MDJ-2_gene_435	428	WP_010405239.1	428	421/428(98.36)	1.45e-277	sodium:proton antiporter [Wolbachia endosymbiont of Nasonia vitripennis]	180837	d__Bacteria;k__norank;p__Proteobacteria;c__Alphaproteobacteria;o__Rickettsiales;f__Anaplasmataceae;g__Wolbachia;s__Wolbachia_endosymbiont_of_Nasonia_vitripennis
MDJ-2_gene_436	778	WP_017531723.1	797	590/776(76.03)	5.45e-247	hypothetical protein [Wolbachia endosymbiont of Diaphorina citri]	116598	d__Bacteria;k__norank;p__Proteobacteria;c__Alphaproteobacteria;o__Rickettsiales;f__Anaplasmataceae;g__Wolbachia;s__Wolbachia_endosymbiont_of_Diaphorina_citri
MDJ-2_gene_437	307	PCG17463.1	307	287/307(93.49)	4.53e-204	pseudouridine synthase [Wolbachia pipientis]	None	Unclassified
MDJ-2_gene_438	420	PBD16206.1	420	376/420(89.52)	1.02e-273	MFS transporter [Wolbachia endosymbiont of Drosophila subpulchrella]	2033655	d__Bacteria;k__norank;p__Proteobacteria;c__Alphaproteobacteria;o__Rickettsiales;f__Anaplasmataceae;g__Wolbachia;s__Wolbachia_endosymbiont_of_Drosophila_subpulchrella
MDJ-2_gene_440	341	WP_063630573.1	383	314/383(81.98)	2.30e-215	hypothetical protein [Wolbachia endosymbiont of Laodelphax striatella]	368602	d__Bacteria;k__norank;p__Proteobacteria;c__Alphaproteobacteria;o__Rickettsiales;f__Anaplasmataceae;g__Wolbachia;s__Wolbachia_endosymbiont_of_Laodelphax_striatellus
MDJ-2_gene_441	261	WP_010402806.1	261	257/261(98.47)	4.90e-185	MULTISPECIES: NAD kinase [Wolbachia]	953	d__Bacteria;k__norank;p__Proteobacteria;c__Alphaproteobacteria;o__Rickettsiales;f__Anaplasmataceae;g__Wolbachia;s__norank
MDJ-2_gene_442	70	WP_068651074.1	70	70/70(100.00)	4.42e-46	50S ribosomal protein L31 [Wolbachia endosymbiont of Trichogramma pretiosum]	125593	d__Bacteria;k__norank;p__Proteobacteria;c__Alphaproteobacteria;o__Rickettsiales;f__Anaplasmataceae;g__Wolbachia;s__Wolbachia_endosymbiont_of_Trichogramma_pretiosum
MDJ-2_gene_443	333	WP_019236705.1	328	314/328(95.73)	4.14e-221	ACP S-malonyltransferase [Wolbachia pipientis]	955	d__Bacteria;k__norank;p__Proteobacteria;c__Alphaproteobacteria;o__Rickettsiales;f__Anaplasmataceae;g__Wolbachia;s__Wolbachia_pipientis
MDJ-2_gene_444	498	WP_010402794.1	498	481/498(96.59)	0	2,3-bisphosphoglycerate-independent phosphoglycerate mutase [Wolbachia endosymbiont of Nasonia vitripennis]	180837	d__Bacteria;k__norank;p__Proteobacteria;c__Alphaproteobacteria;o__Rickettsiales;f__Anaplasmataceae;g__Wolbachia;s__Wolbachia_endosymbiont_of_Nasonia_vitripennis
MDJ-2_gene_445	244	WP_019236703.1	244	240/244(98.36)	1.78e-71	hypothetical protein [Wolbachia pipientis]	955	d__Bacteria;k__norank;p__Proteobacteria;c__Alphaproteobacteria;o__Rickettsiales;f__Anaplasmataceae;g__Wolbachia;s__Wolbachia_pipientis
MDJ-2_gene_446	472	PBQ29066.1	472	470/472(99.58)	0	carboxypeptidase [Wolbachia pipientis wAus]	2049495	d__Bacteria;k__norank;p__Proteobacteria;c__Alphaproteobacteria;o__Rickettsiales;f__Anaplasmataceae;g__Wolbachia;s__Wolbachia_pipientis
MDJ-2_gene_448	430	WP_017532346.1	430	398/430(92.56)	3.29e-291	tRNA lysidine(34) synthetase TilS [Wolbachia endosymbiont of Diaphorina citri]	116598	d__Bacteria;k__norank;p__Proteobacteria;c__Alphaproteobacteria;o__Rickettsiales;f__Anaplasmataceae;g__Wolbachia;s__Wolbachia_endosymbiont_of_Diaphorina_citri
MDJ-2_gene_449	609	PBQ29069.1	609	609/609(100.00)	0	cell division protein FtsH [Wolbachia pipientis wAus]	2049495	d__Bacteria;k__norank;p__Proteobacteria;c__Alphaproteobacteria;o__Rickettsiales;f__Anaplasmataceae;g__Wolbachia;s__Wolbachia_pipientis
MDJ-2_gene_451	256	OJH30787.1	278	241/256(94.14)	7.04e-174	Peptidoglycan-N-acetylglucosamine deacetylase [Wolbachia endosymbiont of Armadillidium vulgare]	77039	d__Bacteria;k__norank;p__Proteobacteria;c__Alphaproteobacteria;o__Rickettsiales;f__Anaplasmataceae;g__Wolbachia;s__Wolbachia_endosymbiont_of_Armadillidium_vulgare
MDJ-2_gene_452	500	WP_127463733.1	500	472/500(94.40)	0	AAA family ATPase [Wolbachia endosymbiont of Bemisia tabaci]	215173	d__Bacteria;k__norank;p__Proteobacteria;c__Alphaproteobacteria;o__Rickettsiales;f__Anaplasmataceae;g__Wolbachia;s__Wolbachia_endosymbiont_of_Bemisia_tabaci
MDJ-2_gene_454	394	WP_010406864.1	394	392/394(99.49)	1.99e-276	cell division protein FtsZ [Wolbachia endosymbiont of Nasonia vitripennis]	2640676	d__Bacteria;k__norank;p__Proteobacteria;c__Alphaproteobacteria;o__Rickettsiales;f__Anaplasmataceae;g__Wolbachia;s__norank

MDJ-2_gene_455	132	AEN25597.1	132	132/132(100.00)	1.18e-90	hypothetical protein [Wolbachia endosymbiont of Nilaparvata	706983	d__Bacteria;k__norank;p__Proteobacteria;c__Alphaproteobacteria;o__Rickettsiales;f__Anaplasmataceae;g__Wolbachia;s__Wolbachia_endosymbiont_of_Nilaparvata_muiri
MDJ-2_gene_456	152	AGJ98716.1	152	152/152(100.00)	8.54e-105	Putative rRNA maturation factor [Wolbachia endosymbiont of Drosophila simulans wNo]	1236908	d__Bacteria;k__norank;p__Proteobacteria;c__Alphaproteobacteria;o__Rickettsiales;f__Anaplasmataceae;g__Wolbachia;s__Wolbachia_endosymbiont_of_Drosophila_simulans
MDJ-2_gene_458	354	PBQ28044.1	353	315/360(87.50)	2.08e-221	tRNA pseudouridine(55) synthase TruB [Wolbachia pipientis wAus]	2049495	d__Bacteria;k__norank;p__Proteobacteria;c__Alphaproteobacteria;o__Rickettsiales;f__Anaplasmataceae;g__Wolbachia;s__Wolbachia_pipientis
MDJ-2_gene_460	402	WP_096097224.1	402	370/400(92.50)	1.19e-272	bifunctional diaminohydroxyphosphoribosylaminopyrimidine deaminase/5-amino-6-(5-phosphoribosylamino)uracil reductase RibD [Wolbachia	955	d__Bacteria;k__norank;p__Proteobacteria;c__Alphaproteobacteria;o__Rickettsiales;f__Anaplasmataceae;g__Wolbachia;s__Wolbachia_pipientis
MDJ-2_gene_461	393	PBQ26859.1	393	385/393(97.96)	2.34e-279	deoxyguanosinetriphosphate triphosphohydrolase [Wolbachia pipientis wAus]	2049495	d__Bacteria;k__norank;p__Proteobacteria;c__Alphaproteobacteria;o__Rickettsiales;f__Anaplasmataceae;g__Wolbachia;s__Wolbachia_pipientis
MDJ-2_gene_462	148	WP_063630824.1	148	146/148(98.65)	7.92e-90	iron-sulfur cluster assembly accessory protein [Wolbachia endosymbiont of Laodelphax striatella]	368602	d__Bacteria;k__norank;p__Proteobacteria;c__Alphaproteobacteria;o__Rickettsiales;f__Anaplasmataceae;g__Wolbachia;s__Wolbachia_endosymbiont_of_Laodelphax_striatellus
MDJ-2_gene_466	182	AZU37732.1	182	181/182(99.45)	1.43e-113	ATP synthase F1 subunit delta [Wolbachia endosymbiont of Bemisia tabaci]	215173	d__Bacteria;k__norank;p__Proteobacteria;c__Alphaproteobacteria;o__Rickettsiales;f__Anaplasmataceae;g__Wolbachia;s__Wolbachia_endosymbiont_of_Bemisia_tabaci
MDJ-2_gene_467	513	PBQ28112.1	513	508/513(99.03)	0	F0F1 ATP synthase subunit alpha [Wolbachia pipientis wAus]	2049495	d__Bacteria;k__norank;p__Proteobacteria;c__Alphaproteobacteria;o__Rickettsiales;f__Anaplasmataceae;g__Wolbachia;s__Wolbachia_pipientis
MDJ-2_gene_469	214	PBQ28114.1	214	210/214(98.13)	2.84e-147	3,4-dihydroxy-2-butanone-4-phosphate synthase [Wolbachia pipientis wAus]	2049495	d__Bacteria;k__norank;p__Proteobacteria;c__Alphaproteobacteria;o__Rickettsiales;f__Anaplasmataceae;g__Wolbachia;s__Wolbachia_pipientis
MDJ-2_gene_470	429	WP_063631059.1	429	419/429(97.67)	4.51e-293	peptidase M48 [Wolbachia endosymbiont of Laodelphax striatella]	2640676	d__Bacteria;k__norank;p__Proteobacteria;c__Alphaproteobacteria;o__Rickettsiales;f__Anaplasmataceae;g__Wolbachia;s__norank
MDJ-2_gene_471	111	WP_108784084.1	111	106/111(95.50)	1.63e-69	hypothetical protein [Wolbachia endosymbiont of Bemisia tabaci]	215173	d__Bacteria;k__norank;p__Proteobacteria;c__Alphaproteobacteria;o__Rickettsiales;f__Anaplasmataceae;g__Wolbachia;s__Wolbachia_endosymbiont_of_Bemisia_tabaci
MDJ-2_gene_472	244	PCG11856.1	244	228/244(93.44)	1.88e-160	3-oxoacyl-[acyl-carrier-protein] reductase [Wolbachia pipientis]	None	Unclassified
MDJ-2_gene_473	224	BAC24299.1	776	170/224(75.89)	2.65e-105	lon [Wigglesworthia glossinidia endosymbiont of Glossina brevipalpis]	36870	d__Bacteria;k__norank;p__Proteobacteria;c__Gammaproteobacteria;o__Enterobacterales;f__Erwiniaceae;g__Wigglesworthia;s__Wigglesworthia_glossinidia
MDJ-2_gene_474	300	WP_130617016.1	785	152/302(50.33)	1.03e-74	endopeptidase La [endosymbiont of Sipalinus gigas]	1972134	d__Bacteria;k__norank;p__Proteobacteria;c__Gammaproteobacteria;o__norank;f__norank;g__Candidatus_Nardonella;s__endosymbiont_of_Sipalinus_gigas
MDJ-2_gene_475	405	WP_053940400.1	426	203/399(50.88)	3.97e-128	ATP-dependent Clp protease ATP-binding subunit ClpX [Buchnera aphidicola]	9	d__Bacteria;k__norank;p__Proteobacteria;c__Gammaproteobacteria;o__Enterobacterales;f__Erwiniaceae;g__Buchnera;s__Buchnera_aphidicola
MDJ-2_gene_476	185	WP_096257963.1	195	116/171(67.84)	6.32e-77	ATP-dependent Clp endopeptidase proteolytic subunit ClpP [Lebetimonas natsushimae]	1936991	d__Bacteria;k__norank;p__Proteobacteria;c__Epsilonproteobacteria;o__Nautiliales;f__Nautiliaceae;g__Lebetimonas;s__Lebetimonas_natsushimae
MDJ-2_gene_477	224	AZP36158.1	225	135/222(60.81)	3.86e-83	DNA polymerase III subunit epsilon [Candidatus Annandia adelgestsuga]	1302411	d__Bacteria;k__norank;p__Proteobacteria;c__Gammaproteobacteria;o__Enterobacterales;f__Enterobacteriaceae;g__Candidatus_Annandia;s__Candidatus_Annandia_adelgestsuga

MDJ-2_gene_478	87	BBA84629.1	159	54/87(62.07)	2.05e-24	ribonuclease H [endosymbiont of Euscepes postfasciatus]	650377	d__Bacteria;k__norank;p__Proteobacteria;c__Gammaproteobacteria;o__norank;f__norank;g__norank;s__endosymbiont_of_Euscepes_postfasciatus
MDJ-2_gene_480	115	AZP36390.1	576	63/115(54.78)	1.12e-30	Arginine--tRNA ligase [Candidatus Annandia	1302411	d__Bacteria;k__norank;p__Proteobacteria;c__Gammaproteobacteria;o__Enterobacterales;f__Enterobacteriaceae;g__Candidatus_Annandia;s__Candidatus_Annandia_adelgestsuga
MDJ-2_gene_482	626	WP_096097186.1	676	506/689(73.44)	1.14e-259	hypothetical protein [Wolbachia pipientis]	955	d__Bacteria;k__norank;p__Proteobacteria;c__Alphaproteobacteria;o__Rickettsiales;f__Anaplasmataceae;g__Wolbachia;s__Wolbachia_pipientis
MDJ-2_gene_483	441	WP_038198185.1	452	437/441(99.09)	0	phosphomannomutase/phosphoglucomutase [Wolbachia pipientis]	955	d__Bacteria;k__norank;p__Proteobacteria;c__Alphaproteobacteria;o__Rickettsiales;f__Anaplasmataceae;g__Wolbachia;s__Wolbachia_pipientis
MDJ-2_gene_485	198	WP_063631027.1	198	194/198(97.98)	2.66e-135	riboflavin synthase [Wolbachia endosymbiont of Laodelphax striatella]	368602	d__Bacteria;k__norank;p__Proteobacteria;c__Alphaproteobacteria;o__Rickettsiales;f__Anaplasmataceae;g__Wolbachia;s__Wolbachia_endosymbiont_of_Laodelphax_striatellus
MDJ-2_gene_488	46	CQD11114.1	46	43/46(93.48)	3.86e-21	Uncharacterised protein [Wolbachia endosymbiont wPip_Mol of Culex molestus]	1402163	d__Bacteria;k__norank;p__Proteobacteria;c__Alphaproteobacteria;o__Rickettsiales;f__Anaplasmataceae;g__Wolbachia;s__Wolbachia_endosymbiont_of_Culex_molestus
MDJ-2_gene_489	358	WP_010405215.1	358	335/358(93.58)	8.46e-237	DNA-protecting protein DprA [Wolbachia endosymbiont of Nasonia vitripennis]	180837	d__Bacteria;k__norank;p__Proteobacteria;c__Alphaproteobacteria;o__Rickettsiales;f__Anaplasmataceae;g__Wolbachia;s__Wolbachia_endosymbiont_of_Nasonia_vitripennis
MDJ-2_gene_490	125	WP_026092603.1	123	121/123(98.37)	5.00e-86	ferredoxin family protein [Wolbachia endosymbiont of	116598	d__Bacteria;k__norank;p__Proteobacteria;c__Alphaproteobacteria;o__Rickettsiales;f__Anaplasmataceae;g__Wolbachia;s__Wolbachia_endosymbiont_of_Diaphorina_citri
MDJ-2_gene_491	315	WP_039833309.1	317	313/315(99.37)	1.84e-228	D-alanine--D-alanine ligase [Wolbachia endosymbiont of Nasonia vitripennis]	2640676	d__Bacteria;k__norank;p__Proteobacteria;c__Alphaproteobacteria;o__Rickettsiales;f__Anaplasmataceae;g__Wolbachia;s__norank
MDJ-2_gene_492	234	AGJ99412.1	252	230/233(98.71)	6.11e-163	Cell division protein FtsQ, putative [Wolbachia endosymbiont of Drosophila	1236908	d__Bacteria;k__norank;p__Proteobacteria;c__Alphaproteobacteria;o__Rickettsiales;f__Anaplasmataceae;g__Wolbachia;s__Wolbachia_endosymbiont_of_Drosophila_simulans
MDJ-2_gene_495	659	PCG11804.1	659	629/659(95.45)	0	propionyl-CoA carboxylase [Wolbachia pipientis]	None	Unclassified
MDJ-2_gene_496	331	AGJ98749.1	331	329/331(99.40)	1.81e-222	Heme A synthase [Wolbachia endosymbiont of Drosophila simulans wNo]	1236908	d__Bacteria;k__norank;p__Proteobacteria;c__Alphaproteobacteria;o__Rickettsiales;f__Anaplasmataceae;g__Wolbachia;s__Wolbachia_endosymbiont_of_Drosophila_simulans
MDJ-2_gene_499	159	PBQ28061.1	159	158/159(99.37)	1.05e-97	hypothetical protein BTO27_02885 [Wolbachia	2049495	d__Bacteria;k__norank;p__Proteobacteria;c__Alphaproteobacteria;o__Rickettsiales;f__Anaplasmataceae;g__Wolbachia;s__Wolbachia_pipientis
MDJ-2_gene_500	75	ONI56563.1	75	75/75(100.00)	1.33e-38	ATP synthase F0, C subunit [Wolbachia pipientis wUni]	1379787	d__Bacteria;k__norank;p__Proteobacteria;c__Alphaproteobacteria;o__Rickettsiales;f__Anaplasmataceae;g__Wolbachia;s__Wolbachia_pipientis
MDJ-2_gene_501	139	RDD33651.1	143	136/138(98.55)	9.72e-92	Transposase DDE domain protein [Wolbachia endosymbiont of Cylisticus convexus]	118728	d__Bacteria;k__norank;p__Proteobacteria;c__Alphaproteobacteria;o__Rickettsiales;f__Anaplasmataceae;g__Wolbachia;s__Wolbachia_endosymbiont_of_Cylisticus_convexus
MDJ-2_gene_502	76	WP_081600821.1	115	70/76(92.11)	1.80e-44	hypothetical protein [Wolbachia endosymbiont of Drosophila simulans]	None	Unclassified
MDJ-2_gene_503	506	PCG11471.1	506	501/506(99.01)	0	bifunctional phosphoribosylaminoimidazolecarboxamide formyltransferase/IMP cyclohydrolase PurH [Wolbachia pipientis]	None	Unclassified
MDJ-2_gene_505	118	WP_063630562.1	118	118/118(100.00)	3.18e-77	MULTISPECIES: 50S ribosomal protein L20 [Wolbachia]	2640676	d__Bacteria;k__norank;p__Proteobacteria;c__Alphaproteobacteria;o__Rickettsiales;f__Anaplasmataceae;g__Wolbachia;s__norank
MDJ-2_gene_506	68	PBQ27634.1	68	68/68(100.00)	1.36e-37	50S ribosomal protein L35 [Wolbachia pipientis wAus]	2049495	d__Bacteria;k__norank;p__Proteobacteria;c__Alphaproteobacteria;o__Rickettsiales;f__Anaplasmataceae;g__Wolbachia;s__Wolbachia_pipientis

MDJ-2_gene_508	238	WP_017531796.1	253	236/237(99.58)	5.03e-161	hypothetical protein [Wolbachia endosymbiont of Diaphorina citri]	116598	d__Bacteria;k__norank;p__Proteobacteria;c__Alphaproteobacteria;o__Rickettsiales;f__Anaplasmataceae;g__Wolbachia;s__Wolbachia_endosymbiont_of_Diaphorina_citri
MDJ-2_gene_510	78	WP_041581364.1	78	69/78(88.46)	3.89e-37	hypothetical protein [Wolbachia endosymbiont of Drosophila simulans]	77038	d__Bacteria;k__norank;p__Proteobacteria;c__Alphaproteobacteria;o__Rickettsiales;f__Anaplasmataceae;g__Wolbachia;s__Wolbachia_endosymbiont_of_Drosophila_simulans
MDJ-2_gene_511	410	CAQ55212.1	410	403/410(98.29)	1.43e-284	Hypothetical protein WP1104 [Wolbachia endosymbiont of Culex quinquefasciatus Pel]	570417	d__Bacteria;k__norank;p__Proteobacteria;c__Alphaproteobacteria;o__Rickettsiales;f__Anaplasmataceae;g__Wolbachia;s__Wolbachia_endosymbiont_of_Culex_quinquefasciatus
MDJ-2_gene_512	790	WP_064085247.1	790	783/790(99.11)	0	DNA topoisomerase (ATP-hydrolyzing) subunit B [Wolbachia endosymbiont of Dactylopius coccus]	None	Unclassified
MDJ-2_gene_516	45	RDD33639.1	45	44/45(97.78)	2.10e-20	hypothetical protein Wcon_02350 [Wolbachia endosymbiont of Cylisticus convexus]	118728	d__Bacteria;k__norank;p__Proteobacteria;c__Alphaproteobacteria;o__Rickettsiales;f__Anaplasmataceae;g__Wolbachia;s__Wolbachia_endosymbiont_of_Cylisticus_convexus
MDJ-2_gene_518	123	PBQ27605.1	278	114/123(92.68)	6.01e-74	biotin--[acetyl-CoA-carboxylase] ligase [Wolbachia pipientis	2049495	d__Bacteria;k__norank;p__Proteobacteria;c__Alphaproteobacteria;o__Rickettsiales;f__Anaplasmataceae;g__Wolbachia;s__Wolbachia_pipientis
MDJ-2_gene_519	461	AGJ99099.1	461	444/461(96.31)	5.84e-280	NADH-quinone oxidoreductase subunit N [Wolbachia endosymbiont of Drosophila	1236908	d__Bacteria;k__norank;p__Proteobacteria;c__Alphaproteobacteria;o__Rickettsiales;f__Anaplasmataceae;g__Wolbachia;s__Wolbachia_endosymbiont_of_Drosophila_simulans
MDJ-2_gene_520	480	WP_029237646.1	480	476/479(99.37)	0	NADH-quinone oxidoreductase subunit M [Wolbachia endosymbiont of Nasonia vitripennis]	180837	d__Bacteria;k__norank;p__Proteobacteria;c__Alphaproteobacteria;o__Rickettsiales;f__Anaplasmataceae;g__Wolbachia;s__Wolbachia_endosymbiont_of_Nasonia_vitripennis
MDJ-2_gene_521	615	QBB83738.1	615	606/615(98.54)	0	NADH-quinone oxidoreductase subunit L [Wolbachia pipientis wAlbB]	1116230	d__Bacteria;k__norank;p__Proteobacteria;c__Alphaproteobacteria;o__Rickettsiales;f__Anaplasmataceae;g__Wolbachia;s__Wolbachia_pipientis
MDJ-2_gene_523	446	PBQ28164.1	446	443/446(99.33)	1.43e-233	hypothetical protein BTO27_02370 [Wolbachia	2049495	d__Bacteria;k__norank;p__Proteobacteria;c__Alphaproteobacteria;o__Rickettsiales;f__Anaplasmataceae;g__Wolbachia;s__Wolbachia_pipientis
MDJ-2_gene_524	139	WP_019078870.1	326	110/133(82.71)	1.15e-71	MULTISPECIES: IS481 family transposase [Wolbachia]	2640676	d__Bacteria;k__norank;p__Proteobacteria;c__Alphaproteobacteria;o__Rickettsiales;f__Anaplasmataceae;g__Wolbachia;s__norank
MDJ-2_gene_525	196	CAQ54642.1	196	195/196(99.49)	3.11e-133	hypothetical protein WP0534 [Wolbachia endosymbiont of Culex quinquefasciatus Pel]	570417	d__Bacteria;k__norank;p__Proteobacteria;c__Alphaproteobacteria;o__Rickettsiales;f__Anaplasmataceae;g__Wolbachia;s__Wolbachia_endosymbiont_of_Culex_quinquefasciatus
MDJ-2_gene_526	502	AGJ98655.1	510	500/502(99.60)	0	UDP-N-acetylmuramoylalanine--D-glutamate ligase [Wolbachia endosymbiont of Drosophila simulans wNo]	1236908	d__Bacteria;k__norank;p__Proteobacteria;c__Alphaproteobacteria;o__Rickettsiales;f__Anaplasmataceae;g__Wolbachia;s__Wolbachia_endosymbiont_of_Drosophila_simulans
MDJ-2_gene_527	236	AGJ98634.1	236	233/236(98.73)	2.53e-170	Triosephosphate isomerase [Wolbachia endosymbiont of Drosophila simulans wNo]	1236908	d__Bacteria;k__norank;p__Proteobacteria;c__Alphaproteobacteria;o__Rickettsiales;f__Anaplasmataceae;g__Wolbachia;s__Wolbachia_endosymbiont_of_Drosophila_simulans
MDJ-2_gene_528	50	PCG11391.1	264	47/47(100.00)	1.13e-21	16S rRNA (adenine(1518)-N(6)/adenine(1519)-N(6))-dimethyltransferase RsmA [Wolbachia pipientis]	None	Unclassified
MDJ-2_gene_530	691	PCG11157.1	691	689/691(99.71)	0	elongation factor G [Wolbachia pipientis]	None	Unclassified
MDJ-2_gene_532	124	PBQ29183.1	124	124/124(100.00)	9.64e-84	30S ribosomal protein S12 [Wolbachia pipientis wAus]	2049495	d__Bacteria;k__norank;p__Proteobacteria;c__Alphaproteobacteria;o__Rickettsiales;f__Anaplasmataceae;g__Wolbachia;s__Wolbachia_pipientis

MDJ-2_gene_534	283	WP_010406841.1	283	282/283(99.65)	8.94e-208	peptide chain release factor N(5)-glutamine methyltransferase [Wolbachia endosymbiont of Nasonia vitripennis]	180837	d__Bacteria;k__norank;p__Proteobacteria;c__Alphaproteobacteria;o__Rickettsiales;f__Anaplasmataceae;g__Wolbachia;s__Wolbachia_endosymbiont_of_Nasonia_vitripennis
MDJ-2_gene_535	199	WP_010406846.1	199	198/199(99.50)	3.86e-124	MULTISPECIES: BON domain-containing protein [Wolbachia]	2640676	d__Bacteria;k__norank;p__Proteobacteria;c__Alphaproteobacteria;o__Rickettsiales;f__Anaplasmataceae;g__Wolbachia;s__norank
MDJ-2_gene_537	704	WP_019236567.1	704	686/704(97.44)	0	hypothetical protein [Wolbachia pipientis]	955	d__Bacteria;k__norank;p__Proteobacteria;c__Alphaproteobacteria;o__Rickettsiales;f__Anaplasmataceae;g__Wolbachia;s__Wolbachia_pipientis
MDJ-2_gene_538	249	WP_065094352.1	249	235/249(94.38)	2.03e-162	MULTISPECIES: glycosyl transferase family 2 [Wolbachia]	2640676	d__Bacteria;k__norank;p__Proteobacteria;c__Alphaproteobacteria;o__Rickettsiales;f__Anaplasmataceae;g__Wolbachia;s__norank
MDJ-2_gene_539	312	WP_065094353.1	317	299/310(96.45)	4.11e-222	MULTISPECIES: NAD(P)-dependent oxidoreductase	2640676	d__Bacteria;k__norank;p__Proteobacteria;c__Alphaproteobacteria;o__Rickettsiales;f__Anaplasmataceae;g__Wolbachia;s__norank
MDJ-2_gene_541	235	WP_019236557.1	381	216/228(94.74)	5.49e-148	MFS transporter [Wolbachia pipientis]	955	d__Bacteria;k__norank;p__Proteobacteria;c__Alphaproteobacteria;o__Rickettsiales;f__Anaplasmataceae;g__Wolbachia;s__Wolbachia_pipientis
MDJ-2_gene_542	347	WP_108784044.1	347	340/347(97.98)	6.61e-249	low specificity L-threonine aldolase [Wolbachia endosymbiont of Bemisia tabaci]	215173	d__Bacteria;k__norank;p__Proteobacteria;c__Alphaproteobacteria;o__Rickettsiales;f__Anaplasmataceae;g__Wolbachia;s__Wolbachia_endosymbiont_of_Bemisia_tabaci
MDJ-2_gene_543	354	RLT59542.1	354	345/354(97.46)	8.03e-249	beta-eliminating lyase family protein [Wolbachia endosymbiont of Drosophila ananassae]	307502	d__Bacteria;k__norank;p__Proteobacteria;c__Alphaproteobacteria;o__Rickettsiales;f__Anaplasmataceae;g__Wolbachia;s__Wolbachia_endosymbiont_of_Drosophila_ananassae
MDJ-2_gene_544	568	WP_077188276.1	597	553/568(97.36)	0	ABC transporter ATP-binding protein [Wolbachia pipientis]	955	d__Bacteria;k__norank;p__Proteobacteria;c__Alphaproteobacteria;o__Rickettsiales;f__Anaplasmataceae;g__Wolbachia;s__Wolbachia_pipientis
MDJ-2_gene_545	335	CCE77504.1	335	326/335(97.31)	3.48e-238	conserved hypothetical protein (Phytanoyl-CoA dioxygenase domain) [Wolbachia pipientis wAlbB]	1116230	d__Bacteria;k__norank;p__Proteobacteria;c__Alphaproteobacteria;o__Rickettsiales;f__Anaplasmataceae;g__Wolbachia;s__Wolbachia_pipientis
MDJ-2_gene_547	279	WP_010403492.1	279	278/279(99.64)	4.28e-213	hypothetical protein [Wolbachia endosymbiont of Nasonia vitripennis]	180837	d__Bacteria;k__norank;p__Proteobacteria;c__Alphaproteobacteria;o__Rickettsiales;f__Anaplasmataceae;g__Wolbachia;s__Wolbachia_endosymbiont_of_Nasonia_vitripennis
MDJ-2_gene_548	488	WP_108783938.1	488	479/488(98.16)	0	N-acetylmuramoyl-L-alanine amidase [Wolbachia endosymbiont of Bemisia tabaci]	215173	d__Bacteria;k__norank;p__Proteobacteria;c__Alphaproteobacteria;o__Rickettsiales;f__Anaplasmataceae;g__Wolbachia;s__Wolbachia_endosymbiont_of_Bemisia_tabaci
MDJ-2_gene_549	226	WP_010403481.1	226	226/226(100.00)	4.17e-155	ribulose-phosphate 3-epimerase [Wolbachia endosymbiont of Nasonia vitripennis]	180837	d__Bacteria;k__norank;p__Proteobacteria;c__Alphaproteobacteria;o__Rickettsiales;f__Anaplasmataceae;g__Wolbachia;s__Wolbachia_endosymbiont_of_Nasonia_vitripennis
MDJ-2_gene_552	408	WP_063630963.1	408	376/410(91.71)	1.47e-223	hypothetical protein [Wolbachia endosymbiont of Laodelphax striatella]	368602	d__Bacteria;k__norank;p__Proteobacteria;c__Alphaproteobacteria;o__Rickettsiales;f__Anaplasmataceae;g__Wolbachia;s__Wolbachia_endosymbiont_of_Laodelphax_striatellus
MDJ-2_gene_553	329	WP_068651961.1	329	325/329(98.78)	1.30e-230	Holliday junction branch migration DNA helicase RuvB [Wolbachia endosymbiont of Trichogramma pretiosum]	125593	d__Bacteria;k__norank;p__Proteobacteria;c__Alphaproteobacteria;o__Rickettsiales;f__Anaplasmataceae;g__Wolbachia;s__Wolbachia_endosymbiont_of_Trichogramma_pretiosum
MDJ-2_gene_556	251	PCG11352.1	251	237/251(94.42)	1.39e-170	alpha/beta hydrolase [Wolbachia pipientis]	None	Unclassified
MDJ-2_gene_559	578	WP_019078674.1	578	554/578(95.85)	0	MULTISPECIES: single-stranded-DNA-specific exonuclease RecJ [Wolbachia]	2640676	d__Bacteria;k__norank;p__Proteobacteria;c__Alphaproteobacteria;o__Rickettsiales;f__Anaplasmataceae;g__Wolbachia;s__norank

MDJ-2_gene_560	440	PBQ27258.1	440	430/440(97.73)	5.73e-315	tRNA (N6-isopentenyl adenosine(37)-C2)-methylthiotransferase MiaB [Wolbachia pipientis wAus]	2049495	d__Bacteria;k__norank;p__Proteobacteria;c__Alphaproteobacteria;o__Rickettsiales;f__Anaplasmataceae;g__Wolbachia;s__Wolbachia_pipientis
MDJ-2_gene_563	1046	WP_108783915.1	1046	1042/1046(99.62)	0	bifunctional proline dehydrogenase/L-glutamate gamma-semialdehyde dehydrogenase PutA [Wolbachia endosymbiont of Bemisia tabaci]	215173	d__Bacteria;k__norank;p__Proteobacteria;c__Alphaproteobacteria;o__Rickettsiales;f__Anaplasmataceae;g__Wolbachia;s__Wolbachia_endosymbiont_of_Bemisia_tabaci
MDJ-2_gene_564	232	EEB56470.1	232	230/232(99.14)	2.17e-169	dioxygenase [Wolbachia endosymbiont of Culex quinquefasciatus JHB]	569881	d__Bacteria;k__norank;p__Proteobacteria;c__Alphaproteobacteria;o__Rickettsiales;f__Anaplasmataceae;g__Wolbachia;s__Wolbachia_endosymbiont_of_Culex_quinquefasciatus
MDJ-2_gene_566	269	CAQ54373.1	269	253/269(94.05)	1.35e-186	Putative phage related protein [Wolbachia endosymbiont of Culex quinquefasciatus Pel]	570417	d__Bacteria;k__norank;p__Proteobacteria;c__Alphaproteobacteria;o__Rickettsiales;f__Anaplasmataceae;g__Wolbachia;s__Wolbachia_endosymbiont_of_Culex_quinquefasciatus
MDJ-2_gene_568	163	ONI57208.1	163	154/163(94.48)	5.01e-107	hypothetical protein N499_1126 [Wolbachia pipientis wVitA]	1379786	d__Bacteria;k__norank;p__Proteobacteria;c__Alphaproteobacteria;o__Rickettsiales;f__Anaplasmataceae;g__Wolbachia;s__Wolbachia_pipientis
MDJ-2_gene_570	227	WP_010082176.1	227	214/227(94.27)	1.39e-149	hypothetical protein [Wolbachia endosymbiont of Drosophila simulans]	953	d__Bacteria;k__norank;p__Proteobacteria;c__Alphaproteobacteria;o__Rickettsiales;f__Anaplasmataceae;g__Wolbachia;s__norank
MDJ-2_gene_571	642	BAH22234.1	644	619/644(96.12)	0	hypothetical protein [Wolbachia endosymbiont of Cadra cautella]	190193	d__Bacteria;k__norank;p__Proteobacteria;c__Alphaproteobacteria;o__Rickettsiales;f__Anaplasmataceae;g__Wolbachia;s__Wolbachia_endosymbiont_of_Cadra_cautella
MDJ-2_gene_573	66	PBQ29110.1	66	66/66(100.00)	6.85e-37	50S ribosomal protein L33 [Wolbachia pipientis wAus]	2049495	d__Bacteria;k__norank;p__Proteobacteria;c__Alphaproteobacteria;o__Rickettsiales;f__Anaplasmataceae;g__Wolbachia;s__Wolbachia_pipientis
MDJ-2_gene_574	585	WP_064085814.1	585	579/585(98.97)	0	Rne/Rng family ribonuclease [Wolbachia endosymbiont of Dactylopius coccus]	None	Unclassified
MDJ-2_gene_575	637	PBQ29091.1	637	624/637(97.96)	0	molecular chaperone DnaK [Wolbachia pipientis wAus]	2049495	d__Bacteria;k__norank;p__Proteobacteria;c__Alphaproteobacteria;o__Rickettsiales;f__Anaplasmataceae;g__Wolbachia;s__Wolbachia_pipientis
MDJ-2_gene_576	101	CCE77254.1	101	97/101(96.04)	1.30e-59	conserved hypothetical protein (BolA-like domain) [Wolbachia pipientis wAlbB]	1116230	d__Bacteria;k__norank;p__Proteobacteria;c__Alphaproteobacteria;o__Rickettsiales;f__Anaplasmataceae;g__Wolbachia;s__Wolbachia_pipientis
MDJ-2_gene_579	304	PBQ28140.1	316	234/318(73.58)	1.11e-143	hypothetical protein BTO27_02410 [Wolbachia	2049495	d__Bacteria;k__norank;p__Proteobacteria;c__Alphaproteobacteria;o__Rickettsiales;f__Anaplasmataceae;g__Wolbachia;s__Wolbachia_pipientis
MDJ-2_gene_581	842	WP_010404431.1	842	827/842(98.22)	0	DNA polymerase I [Wolbachia endosymbiont of Nasonia vitripennis]	180837	d__Bacteria;k__norank;p__Proteobacteria;c__Alphaproteobacteria;o__Rickettsiales;f__Anaplasmataceae;g__Wolbachia;s__Wolbachia_endosymbiont_of_Nasonia_vitripennis
MDJ-2_gene_583	137	PBQ28246.1	137	137/137(100.00)	5.74e-80	preprotein translocase subunit YajC [Wolbachia pipientis wAus]	2049495	d__Bacteria;k__norank;p__Proteobacteria;c__Alphaproteobacteria;o__Rickettsiales;f__Anaplasmataceae;g__Wolbachia;s__Wolbachia_pipientis
MDJ-2_gene_584	603	WP_010407205.1	603	597/602(99.17)	0	MULTISPECIES: glutamine--fructose-6-phosphate transaminase (isomerizing)	2640676	d__Bacteria;k__norank;p__Proteobacteria;c__Alphaproteobacteria;o__Rickettsiales;f__Anaplasmataceae;g__Wolbachia;s__norank
MDJ-2_gene_585	248	PBQ28248.1	249	231/244(94.67)	1.87e-144	hypothetical protein BTO27_01910 [Wolbachia	2049495	d__Bacteria;k__norank;p__Proteobacteria;c__Alphaproteobacteria;o__Rickettsiales;f__Anaplasmataceae;g__Wolbachia;s__Wolbachia_pipientis
MDJ-2_gene_586	276	CCE77017.1	311	259/282(91.84)	3.46e-178	conserved hypothetical protein [Wolbachia pipientis wAlbB]	1116230	d__Bacteria;k__norank;p__Proteobacteria;c__Alphaproteobacteria;o__Rickettsiales;f__Anaplasmataceae;g__Wolbachia;s__Wolbachia_pipientis
MDJ-2_gene_589	245	WP_064085620.1	245	238/245(97.14)	1.92e-174	tRNA pseudouridine(38-40) synthase TruA [Wolbachia endosymbiont of Dactylopius coccus]	None	Unclassified

MDJ-2_gene_590	379	WP_010407054.1	383	366/380(96.32)	2.32e-257	DNA polymerase III subunit beta [Wolbachia endosymbiont of Nasonia vitripennis]	2640676	d__Bacteria;k__norank;p__Proteobacteria;c__Alphaproteobacteria;o__Rickettsiales;f__Anaplasmataceae;g__Wolbachia;s__norank
MDJ-2_gene_591	165	WP_108784179.1	165	163/165(98.79)	1.96e-114	hypothetical protein [Wolbachia endosymbiont of Bemisia tabaci]	215173	d__Bacteria;k__norank;p__Proteobacteria;c__Alphaproteobacteria;o__Rickettsiales;f__Anaplasmataceae;g__Wolbachia;s__Wolbachia_endosymbiont_of_Bemisia_tabaci
MDJ-2_gene_592	297	PBQ27678.1	297	292/297(98.32)	3.18e-199	RNA polymerase factor sigma-32 [Wolbachia pipientis wAus]	2049495	d__Bacteria;k__norank;p__Proteobacteria;c__Alphaproteobacteria;o__Rickettsiales;f__Anaplasmataceae;g__Wolbachia;s__Wolbachia_pipientis
MDJ-2_gene_593	225	WP_127463957.1	227	207/227(91.19)	1.53e-141	P44/Msp2 family outer membrane protein [Wolbachia endosymbiont of Bemisia tabaci]	215173	d__Bacteria;k__norank;p__Proteobacteria;c__Alphaproteobacteria;o__Rickettsiales;f__Anaplasmataceae;g__Wolbachia;s__Wolbachia_endosymbiont_of_Bemisia_tabaci
MDJ-2_gene_594	342	WP_029237556.1	342	338/342(98.83)	1.10e-249	MULTISPECIES: hypothetical protein [Wolbachia]	2640676	d__Bacteria;k__norank;p__Proteobacteria;c__Alphaproteobacteria;o__Rickettsiales;f__Anaplasmataceae;g__Wolbachia;s__norank
MDJ-2_gene_596	93	WP_010406917.1	93	93/93(100.00)	1.58e-63	MULTISPECIES: DUF2610 domain-containing protein	2640676	d__Bacteria;k__norank;p__Proteobacteria;c__Alphaproteobacteria;o__Rickettsiales;f__Anaplasmataceae;g__Wolbachia;s__norank
MDJ-2_gene_597	280	WP_010406915.1	280	279/280(99.64)	4.10e-182	2,3,4,5-tetrahydropyridine-2,6-dicarboxylate N-succinyltransferase [Wolbachia endosymbiont of Nasonia	180837	d__Bacteria;k__norank;p__Proteobacteria;c__Alphaproteobacteria;o__Rickettsiales;f__Anaplasmataceae;g__Wolbachia;s__Wolbachia_endosymbiont_of_Nasonia_vitripennis
MDJ-2_gene_598	103	WP_127463693.1	136	102/103(99.03)	5.97e-66	hypothetical protein [Wolbachia endosymbiont of Bemisia tabaci]	215173	d__Bacteria;k__norank;p__Proteobacteria;c__Alphaproteobacteria;o__Rickettsiales;f__Anaplasmataceae;g__Wolbachia;s__Wolbachia_endosymbiont_of_Bemisia_tabaci
MDJ-2_gene_602	295	WP_064085285.1	295	283/295(95.93)	4.98e-211	4-hydroxy-tetrahydrodipicolinate synthase [Wolbachia endosymbiont of Dactylopius	None	Unclassified
MDJ-2_gene_603	213	WP_063630898.1	203	166/197(84.26)	2.24e-100	hypothetical protein [Wolbachia endosymbiont of Laodelphax striatella]	368602	d__Bacteria;k__norank;p__Proteobacteria;c__Alphaproteobacteria;o__Rickettsiales;f__Anaplasmataceae;g__Wolbachia;s__Wolbachia_endosymbiont_of_Laodelphax_striatellus
MDJ-2_gene_606	178	WP_096617306.1	178	168/178(94.38)	7.78e-116	cytochrome c oxidase assembly protein [Wolbachia pipientis]	955	d__Bacteria;k__norank;p__Proteobacteria;c__Alphaproteobacteria;o__Rickettsiales;f__Anaplasmataceae;g__Wolbachia;s__Wolbachia_pipientis
MDJ-2_gene_607	237	AGJ98901.1	240	237/237(100.00)	8.50e-152	putative bax inhibitor (bi)-1 like protein [Wolbachia endosymbiont of Drosophila simulans wNo]	1236908	d__Bacteria;k__norank;p__Proteobacteria;c__Alphaproteobacteria;o__Rickettsiales;f__Anaplasmataceae;g__Wolbachia;s__Wolbachia_endosymbiont_of_Drosophila_simulans
MDJ-2_gene_609	347	WP_063630537.1	347	317/347(91.35)	1.91e-232	hypothetical protein [Wolbachia endosymbiont of Laodelphax striatella]	368602	d__Bacteria;k__norank;p__Proteobacteria;c__Alphaproteobacteria;o__Rickettsiales;f__Anaplasmataceae;g__Wolbachia;s__Wolbachia_endosymbiont_of_Laodelphax_striatellus
MDJ-2_gene_610	236	WP_127463644.1	236	231/236(97.88)	2.61e-163	ABC transporter permease [Wolbachia endosymbiont of Bemisia tabaci]	215173	d__Bacteria;k__norank;p__Proteobacteria;c__Alphaproteobacteria;o__Rickettsiales;f__Anaplasmataceae;g__Wolbachia;s__Wolbachia_endosymbiont_of_Bemisia_tabaci
MDJ-2_gene_611	272	WP_127463643.1	272	265/272(97.43)	4.66e-184	pyruvate, phosphate dikinase/phosphoenolpyruvate synthase regulator [Wolbachia endosymbiont of Bemisia tabaci]	2640676	d__Bacteria;k__norank;p__Proteobacteria;c__Alphaproteobacteria;o__Rickettsiales;f__Anaplasmataceae;g__Wolbachia;s__norank
MDJ-2_gene_612	329	RDD34666.1	436	185/241(76.76)	4.44e-142	hypothetical protein Wcon_01216 [Wolbachia endosymbiont of Cylisticus convexus]	118728	d__Bacteria;k__norank;p__Proteobacteria;c__Alphaproteobacteria;o__Rickettsiales;f__Anaplasmataceae;g__Wolbachia;s__Wolbachia_endosymbiont_of_Cylisticus_convexus
MDJ-2_gene_613	565	WP_064085642.1	568	556/568(97.89)	0	arginine--tRNA ligase [Wolbachia endosymbiont of Dactylopius coccus]	None	Unclassified
MDJ-2_gene_615	143	WP_064085636.1	143	143/143(100.00)	6.90e-93	hypothetical protein [Wolbachia endosymbiont of Dactylopius coccus]	None	Unclassified

MDJ-2_gene_616	154	PBQ28282.1	142	37/37(100.00)	1.44e-14	hypothetical protein BTO27_01800, partial	2049495	d__Bacteria;k__norank;p__Proteobacteria;c__Alphaproteobacteria;o__Rickettsiales;f__Anaplasmataceae;g__Wolbachia;s__Wolbachia_pipientis
MDJ-2_gene_617	140	BBA85016.1	943	66/146(45.21)	2.09e-26	isoleucine--tRNA ligase [endosymbiont of Rhynchophorus ferrugineus]	1972133	d__Bacteria;k__norank;p__Proteobacteria;c__Gammaproteobacteria;o__norank;f__norank;g__Candidatus_Nardonella;s__Candidatus_Nardonella_dryophthoridicola
MDJ-2_gene_618	58	BBA84905.1	936	40/58(68.97)	2.96e-15	isoleucine--tRNA ligase [endosymbiont of Pachyrhynchus infernalis]	1971488	d__Bacteria;k__norank;p__Proteobacteria;c__Gammaproteobacteria;o__norank;f__norank;g__Candidatus_Nardonella;s__endosymbiont_of_Pachyrhynchus_infernalis
MDJ-2_gene_619	216	BBA84861.1	276	107/210(50.95)	5.09e-55	mechanosensitive channel [endosymbiont of Pachyrhynchus infernalis]	1971488	d__Bacteria;k__norank;p__Proteobacteria;c__Gammaproteobacteria;o__norank;f__norank;g__Candidatus_Nardonella;s__endosymbiont_of_Pachyrhynchus_infernalis
MDJ-2_gene_620	147	BBA84560.1	143	72/146(49.32)	1.81e-38	heat shock protein Hsp20 [endosymbiont of Euscepes postfasciatus]	650377	d__Bacteria;k__norank;p__Proteobacteria;c__Gammaproteobacteria;o__norank;f__norank;g__norank;s__endosymbiont_of_Euscepes_postfasciatus
MDJ-2_gene_621	253	WP_041063354.1	276	105/245(42.86)	2.61e-51	16S rRNA (adenine(1518)-N(6)/adenine(1519)-N(6))-dimethyltransferase RsmA [Candidatus Tachikawaea gelatinosa]	1410383	d__Bacteria;k__norank;p__Proteobacteria;c__Gammaproteobacteria;o__Enterobacterales;f__Enterobacteriaceae;g__Candidatus_Tachikawaea;s__Candidatus_Tachikawaea_gelatinosa
MDJ-2_gene_622	308	PBQ27661.1	462	302/308(98.05)	8.41e-212	fumarate hydratase, class II [Wolbachia pipientis wAus]	2049495	d__Bacteria;k__norank;p__Proteobacteria;c__Alphaproteobacteria;o__Rickettsiales;f__Anaplasmataceae;g__Wolbachia;s__Wolbachia_pipientis
MDJ-2_gene_623	468	WP_010401668.1	468	462/468(98.72)	8.59e-225	hypothetical protein [Wolbachia endosymbiont of Nasonia vitripennis]	180837	d__Bacteria;k__norank;p__Proteobacteria;c__Alphaproteobacteria;o__Rickettsiales;f__Anaplasmataceae;g__Wolbachia;s__Wolbachia_endosymbiont_of_Nasonia_vitripennis
MDJ-2_gene_627	247	AGJ99283.1	247	247/247(100.00)	1.69e-169	Uridylate kinase [Wolbachia endosymbiont of Drosophila simulans wNo]	1236908	d__Bacteria;k__norank;p__Proteobacteria;c__Alphaproteobacteria;o__Rickettsiales;f__Anaplasmataceae;g__Wolbachia;s__Wolbachia_endosymbiont_of_Drosophila_simulans
MDJ-2_gene_628	185	WP_017532537.1	185	185/185(100.00)	6.09e-121	MULTISPECIES: ribosome recycling factor [Wolbachia]	2640676	d__Bacteria;k__norank;p__Proteobacteria;c__Alphaproteobacteria;o__Rickettsiales;f__Anaplasmataceae;g__Wolbachia;s__norank
MDJ-2_gene_630	272	EEB55775.1	272	271/272(99.63)	9.94e-195	phosphate ABC transporter, periplasmic phosphate-binding protein [Wolbachia endosymbiont of Culex quinquefasciatus JHB]	569881	d__Bacteria;k__norank;p__Proteobacteria;c__Alphaproteobacteria;o__Rickettsiales;f__Anaplasmataceae;g__Wolbachia;s__Wolbachia_endosymbiont_of_Culex_quinquefasciatus
MDJ-2_gene_631	276	AGJ99286.1	446	235/271(86.72)	2.31e-154	hypothetical protein wNo_08960 [Wolbachia endosymbiont of Drosophila simulans wNo]	1236908	d__Bacteria;k__norank;p__Proteobacteria;c__Alphaproteobacteria;o__Rickettsiales;f__Anaplasmataceae;g__Wolbachia;s__Wolbachia_endosymbiont_of_Drosophila_simulans
MDJ-2_gene_632	89	WP_096617064.1	89	88/89(98.88)	1.06e-57	50S ribosomal protein L27 [Wolbachia pipientis]	955	d__Bacteria;k__norank;p__Proteobacteria;c__Alphaproteobacteria;o__Rickettsiales;f__Anaplasmataceae;g__Wolbachia;s__Wolbachia_pipientis
MDJ-2_gene_633	100	AZU37690.1	100	100/100(100.00)	1.03e-60	50S ribosomal protein L21 [Wolbachia endosymbiont of Bemisia tabaci]	215173	d__Bacteria;k__norank;p__Proteobacteria;c__Alphaproteobacteria;o__Rickettsiales;f__Anaplasmataceae;g__Wolbachia;s__Wolbachia_endosymbiont_of_Bemisia_tabaci
MDJ-2_gene_635	156	PBQ28171.1	156	150/156(96.15)	4.36e-109	hypothetical protein BTO27_02180 [Wolbachia	2049495	d__Bacteria;k__norank;p__Proteobacteria;c__Alphaproteobacteria;o__Rickettsiales;f__Anaplasmataceae;g__Wolbachia;s__Wolbachia_pipientis
MDJ-2_gene_636	181	WP_063631043.1	186	170/186(91.40)	2.98e-118	hypothetical protein [Wolbachia endosymbiont of Laodelphax striatella]	368602	d__Bacteria;k__norank;p__Proteobacteria;c__Alphaproteobacteria;o__Rickettsiales;f__Anaplasmataceae;g__Wolbachia;s__Wolbachia_endosymbiont_of_Laodelphax_striatellus

MDJ-2_gene_637	411	WP_064085986.1	411	408/411(99.27)	5.87e-289	bifunctional N-acetylglucosamine-1-phosphate uridyltransferase/glucosamine-1-phosphate acetyltransferase [Wolbachia endosymbiont of Dactylopius coccus]	None	Unclassified
MDJ-2_gene_639	86	WP_064085965.1	693	85/86(98.84)	8.34e-41	hypothetical protein [Wolbachia endosymbiont of Dactylopius coccus]	None	Unclassified
MDJ-2_gene_641	699	ONI58209.1	1214	335/677(49.48)	1.05e-197	ankyrin repeat family protein [Wolbachia pipientis wVitA]	1379786	d__Bacteria;k__norank;p__Proteobacteria;c__Alphaproteobacteria;o__Rickettsiales;f__Anaplasmataceae;g__Wolbachia;s__Wolbachia_pipientis
MDJ-2_gene_651	485	WP_010404910.1	485	465/485(95.88)	0	TrkH family potassium uptake protein [Wolbachia endosymbiont of Nasonia vitripennis]	180837	d__Bacteria;k__norank;p__Proteobacteria;c__Alphaproteobacteria;o__Rickettsiales;f__Anaplasmataceae;g__Wolbachia;s__Wolbachia_endosymbiont_of_Nasonia_vitripennis
MDJ-2_gene_652	185	WP_063631088.1	185	182/185(98.38)	3.85e-133	dCTP deaminase [Wolbachia endosymbiont of Laodelphax striatella]	368602	d__Bacteria;k__norank;p__Proteobacteria;c__Alphaproteobacteria;o__Rickettsiales;f__Anaplasmataceae;g__Wolbachia;s__Wolbachia_endosymbiont_of_Laodelphax_striatellus
MDJ-2_gene_653	341	WP_108784219.1	341	331/341(97.07)	1.91e-229	hypothetical protein [Wolbachia endosymbiont of Bemisia tabaci]	215173	d__Bacteria;k__norank;p__Proteobacteria;c__Alphaproteobacteria;o__Rickettsiales;f__Anaplasmataceae;g__Wolbachia;s__Wolbachia_endosymbiont_of_Bemisia_tabaci
MDJ-2_gene_654	411	CAQ55361.1	411	410/411(99.76)	3.65e-308	Metallophosphoesterase [Wolbachia endosymbiont of Culex quinquefasciatus Pel]	570417	d__Bacteria;k__norank;p__Proteobacteria;c__Alphaproteobacteria;o__Rickettsiales;f__Anaplasmataceae;g__Wolbachia;s__Wolbachia_endosymbiont_of_Culex_quinquefasciatus
MDJ-2_gene_655	227	CAQ54546.1	227	202/227(88.99)	8.23e-142	putative phage related protein [Wolbachia endosymbiont of Culex quinquefasciatus Pel]	570417	d__Bacteria;k__norank;p__Proteobacteria;c__Alphaproteobacteria;o__Rickettsiales;f__Anaplasmataceae;g__Wolbachia;s__Wolbachia_endosymbiont_of_Culex_quinquefasciatus
MDJ-2_gene_656	641	WP_052265016.1	643	591/643(91.91)	0	DUF4815 domain-containing protein [Wolbachia endosymbiont of Operophtera brumata]	1620420	d__Bacteria;k__norank;p__Proteobacteria;c__Alphaproteobacteria;o__Rickettsiales;f__Anaplasmataceae;g__Wolbachia;s__Wolbachia_endosymbiont_of_Operophtera_brumata
MDJ-2_gene_657	394	AGJ99837.1	394	372/393(94.66)	4.81e-268	hypothetical protein wHa_03700 [Wolbachia endosymbiont of Drosophila simulans wHa]	1236909	d__Bacteria;k__norank;p__Proteobacteria;c__Alphaproteobacteria;o__Rickettsiales;f__Anaplasmataceae;g__Wolbachia;s__Wolbachia_endosymbiont_of_Drosophila_simulans
MDJ-2_gene_658	64	ONI57200.1	64	60/64(93.75)	1.71e-36	hypothetical protein N499_1118 [Wolbachia pipientis wVitA]	1379786	d__Bacteria;k__norank;p__Proteobacteria;c__Alphaproteobacteria;o__Rickettsiales;f__Anaplasmataceae;g__Wolbachia;s__Wolbachia_pipientis
MDJ-2_gene_662	588	WP_010406902.1	588	586/588(99.66)	4.96e-46	pentapeptide repeat-containing protein [Wolbachia endosymbiont of Nasonia vitripennis]	180837	d__Bacteria;k__norank;p__Proteobacteria;c__Alphaproteobacteria;o__Rickettsiales;f__Anaplasmataceae;g__Wolbachia;s__Wolbachia_endosymbiont_of_Nasonia_vitripennis
MDJ-2_gene_663	324	WP_127463697.1	324	320/324(98.77)	1.56e-192	ankyrin repeat domain-containing protein [Wolbachia endosymbiont of Bemisia tabaci]	215173	d__Bacteria;k__norank;p__Proteobacteria;c__Alphaproteobacteria;o__Rickettsiales;f__Anaplasmataceae;g__Wolbachia;s__Wolbachia_endosymbiont_of_Bemisia_tabaci
MDJ-2_gene_667	164	WP_010406678.1	164	163/164(99.39)	1.56e-115	MULTISPECIES: hypothetical protein [Wolbachia]	2640676	d__Bacteria;k__norank;p__Proteobacteria;c__Alphaproteobacteria;o__Rickettsiales;f__Anaplasmataceae;g__Wolbachia;s__norank
MDJ-2_gene_668	553	AGJ98477.1	584	303/581(52.15)	6.98e-126	hypothetical protein wNo_00160 [Wolbachia endosymbiont of Drosophila simulans wNo]	1236908	d__Bacteria;k__norank;p__Proteobacteria;c__Alphaproteobacteria;o__Rickettsiales;f__Anaplasmataceae;g__Wolbachia;s__Wolbachia_endosymbiont_of_Drosophila_simulans
MDJ-2_gene_669	419	WP_127463558.1	419	406/419(96.90)	8.21e-300	proline--tRNA ligase [Wolbachia endosymbiont of Bemisia tabaci]	215173	d__Bacteria;k__norank;p__Proteobacteria;c__Alphaproteobacteria;o__Rickettsiales;f__Anaplasmataceae;g__Wolbachia;s__Wolbachia_endosymbiont_of_Bemisia_tabaci
MDJ-2_gene_671	166	B3CLX1.1	166	166/166(100.00)	4.79e-111	RecName: Full=Ribosome maturation factor RimM	570417	d__Bacteria;k__norank;p__Proteobacteria;c__Alphaproteobacteria;o__Rickettsiales;f__Anaplasmataceae;g__Wolbachia;s__Wolbachia_endosymbiont_of_Culex_quinquefasciatus
MDJ-2_gene_672	188	B3CLX0.1	188	185/188(98.40)	1.28e-128	RecName: Full=Elongation factor P; Short=EF-P	570417	d__Bacteria;k__norank;p__Proteobacteria;c__Alphaproteobacteria;o__Rickettsiales;f__Anaplasmataceae;g__Wolbachia;s__Wolbachia_endosymbiont_of_Culex_quinquefasciatus

MDJ-2_gene_673	230	WP_127463858.1	230	214/230(93.04)	3.52e-149	inositol monophosphatase family protein [Wolbachia endosymbiont of Bemisia tabaci]	215173	d__Bacteria;k__norank;p__Proteobacteria;c__Alphaproteobacteria;o__Rickettsiales;f__Anaplasmataceae;g__Wolbachia;s__Wolbachia_endosymbiont_of_Bemisia_tabaci
MDJ-2_gene_674	406	PBQ29084.1	404	391/398(98.24)	5.14e-280	5-aminolevulinic acid synthase [Wolbachia pipientis wAus]	2049495	d__Bacteria;k__norank;p__Proteobacteria;c__Alphaproteobacteria;o__Rickettsiales;f__Anaplasmataceae;g__Wolbachia;s__Wolbachia_pipientis
MDJ-2_gene_675	192	WP_096097173.1	438	164/177(92.66)	1.64e-96	ankyrin repeat domain-containing protein [Wolbachia pipientis]	955	d__Bacteria;k__norank;p__Proteobacteria;c__Alphaproteobacteria;o__Rickettsiales;f__Anaplasmataceae;g__Wolbachia;s__Wolbachia_pipientis
MDJ-2_gene_676	358	WP_052264683.1	610	285/321(88.79)	7.43e-157	ankyrin repeat domain-containing protein [Wolbachia endosymbiont of Operophtera brumata]	None	Unclassified
MDJ-2_gene_683	337	WP_010401559.1	337	327/337(97.03)	4.88e-230	hypothetical protein [Wolbachia endosymbiont of Nasonia vitripennis]	2640676	d__Bacteria;k__norank;p__Proteobacteria;c__Alphaproteobacteria;o__Rickettsiales;f__Anaplasmataceae;g__Wolbachia;s__norank
MDJ-2_gene_684	115	PCG11410.1	115	115/115(100.00)	5.34e-79	2Fe-2S ferredoxin [Wolbachia pipientis]	None	Unclassified
MDJ-2_gene_685	593	WP_019236372.1	586	518/590(87.80)	0	hypothetical protein [Wolbachia pipientis]	955	d__Bacteria;k__norank;p__Proteobacteria;c__Alphaproteobacteria;o__Rickettsiales;f__Anaplasmataceae;g__Wolbachia;s__Wolbachia_pipientis
MDJ-2_gene_687	306	WP_017532312.1	306	277/306(90.52)	3.44e-187	hypothetical protein [Wolbachia endosymbiont of Diaphorina citri]	116598	d__Bacteria;k__norank;p__Proteobacteria;c__Alphaproteobacteria;o__Rickettsiales;f__Anaplasmataceae;g__Wolbachia;s__Wolbachia_endosymbiont_of_Diaphorina_citri
MDJ-2_gene_688	429	WP_064085350.1	432	418/429(97.44)	3.54e-309	bifunctional folylpolyglutamate synthase/dihydrofolate synthase [Wolbachia endosymbiont of Dactylopius coccus]	None	Unclassified
MDJ-2_gene_689	184	CAQ55054.1	194	170/184(92.39)	1.74e-115	hypothetical protein WP0946 [Wolbachia endosymbiont of Culex quinquefasciatus Pel]	570417	d__Bacteria;k__norank;p__Proteobacteria;c__Alphaproteobacteria;o__Rickettsiales;f__Anaplasmataceae;g__Wolbachia;s__Wolbachia_endosymbiont_of_Culex_quinquefasciatus
MDJ-2_gene_690	153	WP_127463953.1	153	152/153(99.35)	1.59e-105	type II toxin-antitoxin system RatA family toxin [Wolbachia endosymbiont of Bemisia tabaci]	215173	d__Bacteria;k__norank;p__Proteobacteria;c__Alphaproteobacteria;o__Rickettsiales;f__Anaplasmataceae;g__Wolbachia;s__Wolbachia_endosymbiont_of_Bemisia_tabaci
MDJ-2_gene_692	263	WP_010403864.1	260	259/260(99.62)	4.61e-197	type I methionyl aminopeptidase [Wolbachia endosymbiont of Nasonia vitripennis]	2640676	d__Bacteria;k__norank;p__Proteobacteria;c__Alphaproteobacteria;o__Rickettsiales;f__Anaplasmataceae;g__Wolbachia;s__norank
MDJ-2_gene_693	381	EEB56308.1	381	381/381(100.00)	1.26e-261	major facilitator family transporter [Wolbachia endosymbiont of Culex	569881	d__Bacteria;k__norank;p__Proteobacteria;c__Alphaproteobacteria;o__Rickettsiales;f__Anaplasmataceae;g__Wolbachia;s__Wolbachia_endosymbiont_of_Culex_quinquefasciatus
MDJ-2_gene_694	318	WP_114517798.1	318	297/318(93.40)	1.64e-207	16S rRNA (cytosine(1402)-N(4))-methyltransferase RsmH [Wolbachia endosymbiont of Cylisticus convexus]	118728	d__Bacteria;k__norank;p__Proteobacteria;c__Alphaproteobacteria;o__Rickettsiales;f__Anaplasmataceae;g__Wolbachia;s__Wolbachia_endosymbiont_of_Cylisticus_convexus
MDJ-2_gene_697	232	CAQ54601.1	230	225/229(98.25)	3.22e-118	membrane protein, TerC family [Wolbachia endosymbiont of Culex quinquefasciatus Pel]	570417	d__Bacteria;k__norank;p__Proteobacteria;c__Alphaproteobacteria;o__Rickettsiales;f__Anaplasmataceae;g__Wolbachia;s__Wolbachia_endosymbiont_of_Culex_quinquefasciatus
MDJ-2_gene_698	520	PBQ29231.1	520	513/520(98.65)	0	glutamine-hydrolyzing GMP synthase [Wolbachia pipientis wAus]	2049495	d__Bacteria;k__norank;p__Proteobacteria;c__Alphaproteobacteria;o__Rickettsiales;f__Anaplasmataceae;g__Wolbachia;s__Wolbachia_pipientis
MDJ-2_gene_700	70	CAQ55154.1	70	63/70(90.00)	1.14e-39	Hypothetical protein WP1046 [Wolbachia endosymbiont of Culex quinquefasciatus Pel]	570417	d__Bacteria;k__norank;p__Proteobacteria;c__Alphaproteobacteria;o__Rickettsiales;f__Anaplasmataceae;g__Wolbachia;s__Wolbachia_endosymbiont_of_Culex_quinquefasciatus
MDJ-2_gene_701	483	PBQ30175.1	465	445/483(92.13)	0	terminase [Wolbachia pipientis wAus]	2049495	d__Bacteria;k__norank;p__Proteobacteria;c__Alphaproteobacteria;o__Rickettsiales;f__Anaplasmataceae;g__Wolbachia;s__Wolbachia_pipientis

MDJ-2_gene_702	173	WP_127464069.1	173	171/173(98.84)	1.53e-124	hypothetical protein [Wolbachia endosymbiont of Bemisia tabaci]	215173	d__Bacteria;k__norank;p__Proteobacteria;c__Alphaproteobacteria;o__Rickettsiales;f__Anaplasmataceae;g__Wolbachia;s__Wolbachia_endosymbiont_of_Bemisia_tabaci
MDJ-2_gene_703	195	WP_063631013.1	214	189/195(96.92)	6.91e-134	MULTISPECIES: tRNA (guanosine(46)-N7)-methyltransferase TrmB	2640676	d__Bacteria;k__norank;p__Proteobacteria;c__Alphaproteobacteria;o__Rickettsiales;f__Anaplasmataceae;g__Wolbachia;s__norank
MDJ-2_gene_705	318	WP_064085442.1	321	312/314(99.36)	5.59e-218	UDP-N-acetylmuramate--alanine ligase [Wolbachia endosymbiont of Dactylopius coccus]	None	Unclassified
MDJ-2_gene_706	182	CQD07071.1	196	150/182(82.42)	5.23e-94	drug resistance transporter [Wolbachia endosymbiont wPip_Mol of Culex molestus]	1402163	d__Bacteria;k__norank;p__Proteobacteria;c__Alphaproteobacteria;o__Rickettsiales;f__Anaplasmataceae;g__Wolbachia;s__Wolbachia_endosymbiont_of_Culex_molestus
MDJ-2_gene_707	208	WP_064085443.1	386	187/196(95.41)	2.46e-121	MFS transporter [Wolbachia endosymbiont of Dactylopius coccus]	None	Unclassified
MDJ-2_gene_708	185	RDD35553.1	185	174/185(94.05)	3.70e-131	Bifunctional AAC/APH [Wolbachia endosymbiont of Cylisticus convexus]	118728	d__Bacteria;k__norank;p__Proteobacteria;c__Alphaproteobacteria;o__Rickettsiales;f__Anaplasmataceae;g__Wolbachia;s__Wolbachia_endosymbiont_of_Cylisticus_convexus
MDJ-2_gene_709	309	WP_064125222.1	309	309/309(100.00)	1.92e-206	permease [Wolbachia endosymbiont of Dactylopius	None	Unclassified
MDJ-2_gene_712	232	WP_017532524.1	232	230/232(99.14)	6.96e-166	bifunctional demethylmenaquinone methyltransferase/2-methoxy-6-polyprenyl-1,4-benzoquinol methylase UbiE [Wolbachia	116598	d__Bacteria;k__norank;p__Proteobacteria;c__Alphaproteobacteria;o__Rickettsiales;f__Anaplasmataceae;g__Wolbachia;s__Wolbachia_endosymbiont_of_Diaphorina_citri
MDJ-2_gene_713	398	CCE77004.1	403	377/392(96.17)	4.55e-251	putative permease of the Major facilitator superfamily [Wolbachia pipientis wAlbB]	1116230	d__Bacteria;k__norank;p__Proteobacteria;c__Alphaproteobacteria;o__Rickettsiales;f__Anaplasmataceae;g__Wolbachia;s__Wolbachia_pipientis
MDJ-2_gene_717	122	CAQ54411.1	111	108/111(97.30)	1.80e-69	baseplate assembly protein GpW [Wolbachia endosymbiont of Culex quinquefasciatus Pel]	570417	d__Bacteria;k__norank;p__Proteobacteria;c__Alphaproteobacteria;o__Rickettsiales;f__Anaplasmataceae;g__Wolbachia;s__Wolbachia_endosymbiont_of_Culex_quinquefasciatus
MDJ-2_gene_718	269	BAA89651.1	266	247/269(91.82)	1.07e-164	gp26 [Wolbachia phage WO]	112596	d__Viruses;k__norank;p__Uroviricota;c__Caudoviricetes;o__Caudovirales;f__Myoviridae;g__norank;s__Wolbachia_phage_WO
MDJ-2_gene_725	337	AGJ99335.1	337	328/337(97.33)	2.46e-239	GTPase Obg [Wolbachia endosymbiont of Drosophila simulans wNo]	1236908	d__Bacteria;k__norank;p__Proteobacteria;c__Alphaproteobacteria;o__Rickettsiales;f__Anaplasmataceae;g__Wolbachia;s__Wolbachia_endosymbiont_of_Drosophila_simulans
MDJ-2_gene_726	423	PBQ27235.1	424	400/423(94.56)	3.50e-285	phosphopyruvate hydratase [Wolbachia pipientis wAus]	2049495	d__Bacteria;k__norank;p__Proteobacteria;c__Alphaproteobacteria;o__Rickettsiales;f__Anaplasmataceae;g__Wolbachia;s__Wolbachia_pipientis
MDJ-2_gene_730	62	OVZ94212.1	61	35/62(56.45)	2.38e-15	hypothetical protein CBW53_21615 [Yersinia	29484	d__Bacteria;k__norank;p__Proteobacteria;c__Gammaproteobacteria;o__Enterobacterales;f__Yersiniaceae;g__Yersinia;s__Yersinia_frederiksenii
MDJ-2_gene_731	110	WP_069728501.1	113	27/67(40.30)	1.12e-10	hypothetical protein [Pantoea brenneri]	472694	d__Bacteria;k__norank;p__Proteobacteria;c__Gammaproteobacteria;o__Enterobacterales;f__Erwiniaceae;g__Pantoea;s__Pantoea_brenneri
MDJ-2_gene_732	65	WP_047357507.1	59	33/51(64.71)	7.34e-15	hypothetical protein [Enterobacter kobei]	208224	d__Bacteria;k__norank;p__Proteobacteria;c__Gammaproteobacteria;o__Enterobacterales;f__Enterobacteriaceae;g__Enterobacter;s__Enterobacter_kobei
MDJ-2_gene_733	70	YP_009322198.1	68	29/56(51.79)	8.77e-12	hypothetical protein [Salmonella phage 64795_sal3]	1813769	d__Viruses;k__norank;p__Uroviricota;c__Caudoviricetes;o__Caudovirales;f__Siphoviridae;g__norank;s__Salmonella_phage_64795_sal3
MDJ-2_gene_735	473	QAY00882.1	473	328/471(69.64)	1.35e-254	large terminase subunit [Salmonella phage SeSz-2]	2419753	d__Viruses;k__norank;p__Uroviricota;c__Caudoviricetes;o__Caudovirales;f__Siphoviridae;g__norank;s__Salmonella_phage_SeSz-2
MDJ-2_gene_736	148	YP_008126205.1	146	112/145(77.24)	1.84e-74	hypothetical protein VPRG_00041 [Vibrio phage	754051	d__Viruses;k__norank;p__Uroviricota;c__Caudoviricetes;o__Caudovirales;f__Siphoviridae;g__Roufvirus;s__Vibrio_phage_pYD38-A

MDJ-2_gene_740	455	WP_114517842.1	446	309/456(67.76)	1.89e-217	hypothetical protein [Wolbachia endosymbiont of Cylisticus convexus]	118728	d__Bacteria;k__norank;p__Proteobacteria;c__Alphaproteobacteria;o__Rickettsiales;f__Anaplasmataceae;g__Wolbachia;s__Wolbachia_endosymbiont_of_Cylisticus_convexus
MDJ-2_gene_741	763	WP_019236878.1	1396	614/868(70.74)	6.91e-264	collagen-like protein [Wolbachia pipientis]	955	d__Bacteria;k__norank;p__Proteobacteria;c__Alphaproteobacteria;o__Rickettsiales;f__Anaplasmataceae;g__Wolbachia;s__Wolbachia_pipientis
MDJ-2_gene_744	263	CQD06359.1	263	261/263(99.24)	1.40e-185	type IV secretion system protein VirB9 [Wolbachia endosymbiont wPip_Mol of Culex molestus]	1402163	d__Bacteria;k__norank;p__Proteobacteria;c__Alphaproteobacteria;o__Rickettsiales;f__Anaplasmataceae;g__Wolbachia;s__Wolbachia_endosymbiont_of_Culex_molestus
MDJ-2_gene_745	226	PBQ28235.1	226	226/226(100.00)	5.45e-151	type IV secretion system protein VirB8 [Wolbachia pipientis	2049495	d__Bacteria;k__norank;p__Proteobacteria;c__Alphaproteobacteria;o__Rickettsiales;f__Anaplasmataceae;g__Wolbachia;s__Wolbachia_pipientis
MDJ-2_gene_746	365	CCE77032.1	383	361/365(98.90)	3.03e-263	putative GTP cyclohydrolase II (ribA-like, riboflavine synthesis) [Wolbachia pipientis wAlbB]	1116230	d__Bacteria;k__norank;p__Proteobacteria;c__Alphaproteobacteria;o__Rickettsiales;f__Anaplasmataceae;g__Wolbachia;s__Wolbachia_pipientis
MDJ-2_gene_747	774	AGJ99404.1	3199	683/741(92.17)	8.66e-315	Ankyrin repeat protein [Wolbachia endosymbiont of Drosophila simulans wNo]	1236908	d__Bacteria;k__norank;p__Proteobacteria;c__Alphaproteobacteria;o__Rickettsiales;f__Anaplasmataceae;g__Wolbachia;s__Wolbachia_endosymbiont_of_Drosophila_simulans
MDJ-2_gene_750	314	ONI57838.1	307	292/307(95.11)	4.81e-194	hypothetical protein N499_0492 [Wolbachia pipientis wVitA]	1379786	d__Bacteria;k__norank;p__Proteobacteria;c__Alphaproteobacteria;o__Rickettsiales;f__Anaplasmataceae;g__Wolbachia;s__Wolbachia_pipientis
MDJ-2_gene_757	47	WP_063630631.1	178	45/45(100.00)	3.60e-24	ribonuclease HI [Wolbachia endosymbiont of Laodelphax striatella]	368602	d__Bacteria;k__norank;p__Proteobacteria;c__Alphaproteobacteria;o__Rickettsiales;f__Anaplasmataceae;g__Wolbachia;s__Wolbachia_endosymbiont_of_Laodelphax_striatellus
MDJ-2_gene_758	64	PBQ26883.1	64	64/64(100.00)	1.03e-37	Fe-S assembly protein IscX [Wolbachia pipientis wAus]	2049495	d__Bacteria;k__norank;p__Proteobacteria;c__Alphaproteobacteria;o__Rickettsiales;f__Anaplasmataceae;g__Wolbachia;s__Wolbachia_pipientis
MDJ-2_gene_759	204	PBQ26884.1	204	204/204(100.00)	6.76e-141	30S ribosomal protein S4 [Wolbachia pipientis wAus]	2049495	d__Bacteria;k__norank;p__Proteobacteria;c__Alphaproteobacteria;o__Rickettsiales;f__Anaplasmataceae;g__Wolbachia;s__Wolbachia_pipientis
MDJ-2_gene_760	646	CAQ54337.1	654	639/646(98.92)	0	transketolase [Wolbachia endosymbiont of Culex quinquefasciatus Pel]	570417	d__Bacteria;k__norank;p__Proteobacteria;c__Alphaproteobacteria;o__Rickettsiales;f__Anaplasmataceae;g__Wolbachia;s__Wolbachia_endosymbiont_of_Culex_quinquefasciatus
MDJ-2_gene_761	231	WP_096097221.1	578	186/219(84.93)	1.48e-105	AAA family ATPase, partial [Wolbachia pipientis]	955	d__Bacteria;k__norank;p__Proteobacteria;c__Alphaproteobacteria;o__Rickettsiales;f__Anaplasmataceae;g__Wolbachia;s__Wolbachia_pipientis
MDJ-2_gene_763	139	RDD34701.1	439	79/106(74.53)	6.29e-48	Ankyrin repeat domain protein [Wolbachia endosymbiont of Cylisticus convexus]	118728	d__Bacteria;k__norank;p__Proteobacteria;c__Alphaproteobacteria;o__Rickettsiales;f__Anaplasmataceae;g__Wolbachia;s__Wolbachia_endosymbiont_of_Cylisticus_convexus
MDJ-2_gene_765	579	OUM57805.1	900	77/277(27.80)	1.10e-06	hypothetical protein PIROE2DRAFT_21440 [Piromyces sp. E2]	73868	d__Eukaryota;k__Fungi;p__Chytridiomycota;c__Neocallimastigomycetes;o__Neocallimastigales;f__Neocallimastigaceae;g__Piromyces;s__Piromyces_sp._E2
MDJ-2_gene_767	146	WP_019078826.1	2166	81/102(79.41)	4.86e-42	tetratricopeptide repeat protein, partial [Wolbachia endosymbiont of Culex pipiens molestus]	263438	d__Bacteria;k__norank;p__Proteobacteria;c__Alphaproteobacteria;o__Rickettsiales;f__Anaplasmataceae;g__Wolbachia;s__Wolbachia_endosymbiont_of_Culex_pipiens_molestus
MDJ-2_gene_768	738	WP_127464236.1	740	643/740(86.89)	0	hypothetical protein [Wolbachia endosymbiont of Bemisia tabaci]	215173	d__Bacteria;k__norank;p__Proteobacteria;c__Alphaproteobacteria;o__Rickettsiales;f__Anaplasmataceae;g__Wolbachia;s__Wolbachia_endosymbiont_of_Bemisia_tabaci
MDJ-2_gene_769	287	WP_108784365.1	1043	229/275(83.27)	1.13e-148	collagen-like protein [Wolbachia endosymbiont of Bemisia tabaci]	215173	d__Bacteria;k__norank;p__Proteobacteria;c__Alphaproteobacteria;o__Rickettsiales;f__Anaplasmataceae;g__Wolbachia;s__Wolbachia_endosymbiont_of_Bemisia_tabaci
MDJ-2_gene_771	687	WP_065094956.1	684	652/684(95.32)	0	MULTISPECIES: hypothetical protein [Wolbachia]	2640676	d__Bacteria;k__norank;p__Proteobacteria;c__Alphaproteobacteria;o__Rickettsiales;f__Anaplasmataceae;g__Wolbachia;s__norank
MDJ-2_gene_772	78	WP_065094955.1	78	70/78(89.74)	5.09e-42	MULTISPECIES: hypothetical protein [Wolbachia]	None	Unclassified
MDJ-2_gene_773	376	BAH22208.1	369	350/369(94.85)	4.74e-255	hypothetical protein [Wolbachia endosymbiont of Cadra cautella]	190193	d__Bacteria;k__norank;p__Proteobacteria;c__Alphaproteobacteria;o__Rickettsiales;f__Anaplasmataceae;g__Wolbachia;s__Wolbachia_endosymbiont_of_Cadra_cautella

MDJ-2_gene_775	362	WP_065106864.1	363	342/361(94.74)	1.69e-252	carbamoyl-phosphate synthase small subunit [Wolbachia endosymbiont of Nomada	1854761	d__Bacteria;k__norank;p__Proteobacteria;c__Alphaproteobacteria;o__Rickettsiales;f__Anaplasmataceae;g__Wolbachia;s__Wolbachia_endosymbiont_of_Nomada_ferruginata
MDJ-2_gene_776	238	WP_010407189.1	238	234/238(98.32)	7.79e-166	SDR family NAD(P)-dependent oxidoreductase [Wolbachia endosymbiont of Nasonia vitripennis]	180837	d__Bacteria;k__norank;p__Proteobacteria;c__Alphaproteobacteria;o__Rickettsiales;f__Anaplasmataceae;g__Wolbachia;s__Wolbachia_endosymbiont_of_Nasonia_vitripennis
MDJ-2_gene_777	158	WP_010407187.1	155	151/155(97.42)	2.33e-93	hypothetical protein [Wolbachia endosymbiont of Nasonia vitripennis]	180837	d__Bacteria;k__norank;p__Proteobacteria;c__Alphaproteobacteria;o__Rickettsiales;f__Anaplasmataceae;g__Wolbachia;s__Wolbachia_endosymbiont_of_Nasonia_vitripennis
MDJ-2_gene_778	124	WP_081600821.1	115	85/111(76.58)	2.99e-58	hypothetical protein [Wolbachia endosymbiont of Drosophila simulans]	None	Unclassified
MDJ-2_gene_779	99	WP_010404973.1	162	89/90(98.89)	1.20e-58	transposase [Wolbachia endosymbiont of Nasonia vitripennis]	180837	d__Bacteria;k__norank;p__Proteobacteria;c__Alphaproteobacteria;o__Rickettsiales;f__Anaplasmataceae;g__Wolbachia;s__Wolbachia_endosymbiont_of_Nasonia_vitripennis
MDJ-2_gene_782	179	WP_010403849.1	179	173/179(96.65)	9.10e-120	peptide deformylase [Wolbachia endosymbiont of Nasonia vitripennis]	180837	d__Bacteria;k__norank;p__Proteobacteria;c__Alphaproteobacteria;o__Rickettsiales;f__Anaplasmataceae;g__Wolbachia;s__Wolbachia_endosymbiont_of_Nasonia_vitripennis
MDJ-2_gene_783	257	CAQ55030.1	257	255/257(99.22)	2.53e-185	putative DNA glycosylase [Wolbachia endosymbiont of Culex quinquefasciatus Pel]	570417	d__Bacteria;k__norank;p__Proteobacteria;c__Alphaproteobacteria;o__Rickettsiales;f__Anaplasmataceae;g__Wolbachia;s__Wolbachia_endosymbiont_of_Culex_quinquefasciatus
MDJ-2_gene_784	175	PBQ25676.1	175	174/175(99.43)	2.16e-116	demethoxyubiquinone hydroxylase family protein [Wolbachia pipientis wAus]	2049495	d__Bacteria;k__norank;p__Proteobacteria;c__Alphaproteobacteria;o__Rickettsiales;f__Anaplasmataceae;g__Wolbachia;s__Wolbachia_pipientis
MDJ-2_gene_787	406	WP_010404310.1	406	401/406(98.77)	6.75e-281	heme biosynthesis protein HemY [Wolbachia endosymbiont of Nasonia vitripennis]	2640676	d__Bacteria;k__norank;p__Proteobacteria;c__Alphaproteobacteria;o__Rickettsiales;f__Anaplasmataceae;g__Wolbachia;s__norank
MDJ-2_gene_788	290	WP_063630933.1	290	287/290(98.97)	3.52e-188	F0F1 ATP synthase subunit gamma [Wolbachia endosymbiont of Laodelphax striatella]	368602	d__Bacteria;k__norank;p__Proteobacteria;c__Alphaproteobacteria;o__Rickettsiales;f__Anaplasmataceae;g__Wolbachia;s__Wolbachia_endosymbiont_of_Laodelphax_striatellus
MDJ-2_gene_789	444	WP_108784149.1	444	440/444(99.10)	8.38e-308	TldD/PmbA family protein [Wolbachia endosymbiont of Bemisia tabaci]	215173	d__Bacteria;k__norank;p__Proteobacteria;c__Alphaproteobacteria;o__Rickettsiales;f__Anaplasmataceae;g__Wolbachia;s__Wolbachia_endosymbiont_of_Bemisia_tabaci
MDJ-2_gene_791	599	WP_064085382.1	599	595/599(99.33)	0	peptidylprolyl isomerase [Wolbachia endosymbiont of Dactylopius coccus]	None	Unclassified
MDJ-2_gene_793	328	WP_064085383.1	328	325/328(99.09)	1.46e-231	polyprenyl synthetase family protein [Wolbachia endosymbiont of Dactylopius coccus]	None	Unclassified
MDJ-2_gene_796	124	WP_106481696.1	133	38/108(35.19)	2.53e-17	MULTISPECIES: fibronectin type III domain-containing protein [Butyricimonas]	574697	d__Bacteria;k__norank;p__Bacteroidetes;c__Bacteroidia;o__Bacteroidales;f__Odoribacteraceae;g__Butyricimonas;s__norank
MDJ-2_gene_798	216	KNE87445.1	122	33/96(34.38)	9.44e-12	hypothetical protein PSTG_19170, partial [Puccinia striiformis f. sp. tritici PST-78]	1165861	d__Eukaryota;k__Fungi;p__Basidiomycota;c__Pucciniomycetes;o__Pucciniales;f__Pucciniaceae;g__Puccinia;s__Puccinia_striiformis
MDJ-2_gene_799	459	GBL21947.1	1737	132/442(29.86)	5.24e-36	aqualysin-1 [Acidimicrobiaceae bacterium]	2024894	d__Bacteria;k__norank;p__Actinobacteria;c__Acidimicrobiia;o__Acidimicrobiales;f__Acidimicrobiaceae;g__norank;s__Acidimicrobiaceae_bacterium
MDJ-2_gene_800	251	PXF62283.1	519	64/180(35.56)	2.60e-21	hypothetical protein DL796_12365, partial [Kangiella	796379	d__Bacteria;k__norank;p__Proteobacteria;c__Gammaproteobacteria;o__Oceanospirillales;f__Kangiellaceae;g__Kangiella;s__Kangiella_spongicola

MDJ-2_gene_801	53	BBA84766.1	354	37/48(77.08)	3.06e-15	UDP-N-acetylglucosamine--N-acetylmuramyl-(pentapeptide) pyrophosphoryl-undecaprenol N-acetylglucosamine transferase [endosymbiont of Pachyrhynchus infernalis]	1971488	d__Bacteria;k__norank;p__Proteobacteria;c__Gammaproteobacteria;o__norank;f__norank;g__Candidatus_Nardonella;s__endosymbiont_of_Pachyrhynchus_infernalis
MDJ-2_gene_803	200	BBA85179.1	468	108/189(57.14)	3.52e-57	UDP-N-acetylmuramate-alanine ligase [endosymbiont of Sipalinus gigas]	1972134	d__Bacteria;k__norank;p__Proteobacteria;c__Gammaproteobacteria;o__norank;f__norank;g__Candidatus_Nardonella;s__endosymbiont_of_Sipalinus_gigas
MDJ-2_gene_804	164	AEO08021.1	484	80/163(49.08)	2.41e-33	UDP-N-acetylmuramate-alanine ligase [Buchnera aphidicola str. Ua (Uroleucon ambrosiae)]	1005057	d__Bacteria;k__norank;p__Proteobacteria;c__Gammaproteobacteria;o__Enterobacterales;f__Erwiniaceae;g__Buchnera;s__Buchnera_aphidicola
MDJ-2_gene_805	350	BBA85183.1	806	197/324(60.80)	2.07e-115	protein translocase subunit SecA [endosymbiont of Sipalinus gigas]	1972134	d__Bacteria;k__norank;p__Proteobacteria;c__Gammaproteobacteria;o__norank;f__norank;g__Candidatus_Nardonella;s__endosymbiont_of_Sipalinus_gigas
MDJ-2_gene_807	139	WP_096617545.1	320	109/135(80.74)	1.02e-68	RluA family pseudouridine synthase [Wolbachia pipientis]	955	d__Bacteria;k__norank;p__Proteobacteria;c__Alphaproteobacteria;o__Rickettsiales;f__Anaplasmataceae;g__Wolbachia;s__Wolbachia_pipientis
MDJ-2_gene_808	603	WP_064085516.1	602	530/603(87.89)	0	hypothetical protein [Wolbachia endosymbiont of Dactylopius coccus]	None	Unclassified
MDJ-2_gene_809	597	PCG11597.1	674	572/600(95.33)	0	competence protein ComEC [Wolbachia pipientis]	None	Unclassified
MDJ-2_gene_810	460	PBQ29277.1	460	459/460(99.78)	0	chromosomal replication initiation protein DnaA [Wolbachia pipientis wAus]	2049495	d__Bacteria;k__norank;p__Proteobacteria;c__Alphaproteobacteria;o__Rickettsiales;f__Anaplasmataceae;g__Wolbachia;s__Wolbachia_pipientis
MDJ-2_gene_811	290	WP_010401100.1	291	289/290(99.66)	1.89e-190	MULTISPECIES: protein translocase subunit SecF	2640676	d__Bacteria;k__norank;p__Proteobacteria;c__Alphaproteobacteria;o__Rickettsiales;f__Anaplasmataceae;g__Wolbachia;s__norank
MDJ-2_gene_812	226	WP_017532608.1	226	99/249(39.76)	6.66e-41	hypothetical protein [Wolbachia endosymbiont of Diaphorina citri]	116598	d__Bacteria;k__norank;p__Proteobacteria;c__Alphaproteobacteria;o__Rickettsiales;f__Anaplasmataceae;g__Wolbachia;s__Wolbachia_endosymbiont_of_Diaphorina_citri
MDJ-2_gene_813	167	PCG11977.1	174	166/167(99.40)	2.05e-114	TIGR02281 family clan AA aspartic protease [Wolbachia	None	Unclassified
MDJ-2_gene_814	245	AGC03678.1	261	105/239(43.93)	1.18e-68	putative deoxyribonuclease ycfH [Candidatus Blochmannia chromaiodes str. 640]	1240471	d__Bacteria;k__norank;p__Proteobacteria;c__Gammaproteobacteria;o__Enterobacterales;f__Enterobacteriaceae;g__Candidatus_Blochmannia;s__Candidatus_Blochmannia_chromaiodes
MDJ-2_gene_815	115	WP_115955955.1	114	74/107(69.16)	3.65e-45	HIT domain-containing protein [Candidatus Purcelliella pentastirinorum]	472834	d__Bacteria;k__norank;p__Proteobacteria;c__Gammaproteobacteria;o__Enterobacterales;f__Enterobacteriaceae;g__Candidatus_Purcelliella;s__Candidatus_Purcelliella_pentastirinorum
MDJ-2_gene_817	153	BBA84631.1	514	94/152(61.84)	9.99e-50	putative lipid II flippase MurJ [endosymbiont of Euscepes postfasciatus]	650377	d__Bacteria;k__norank;p__Proteobacteria;c__Gammaproteobacteria;o__norank;f__norank;g__norank;s__endosymbiont_of_Euscepes_postfasciatus
MDJ-2_gene_818	66	BBA84631.1	514	37/65(56.92)	6.54e-11	putative lipid II flippase MurJ [endosymbiont of Euscepes postfasciatus]	650377	d__Bacteria;k__norank;p__Proteobacteria;c__Gammaproteobacteria;o__norank;f__norank;g__norank;s__endosymbiont_of_Euscepes_postfasciatus
MDJ-2_gene_821	212	WP_010402279.1	264	210/212(99.06)	2.92e-142	16S rRNA (adenine(1518)-N(6)/adenine(1519)-N(6))-dimethyltransferase RsmA [Wolbachia endosymbiont of Nasonia vitripennis]	2640676	d__Bacteria;k__norank;p__Proteobacteria;c__Alphaproteobacteria;o__Rickettsiales;f__Anaplasmataceae;g__Wolbachia;s__norank
MDJ-2_gene_822	495	WP_063630597.1	495	491/495(99.19)	0	IMP dehydrogenase [Wolbachia endosymbiont of Laodelphax striatella]	368602	d__Bacteria;k__norank;p__Proteobacteria;c__Alphaproteobacteria;o__Rickettsiales;f__Anaplasmataceae;g__Wolbachia;s__Wolbachia_endosymbiont_of_Laodelphax_striatellus

MDJ-2_gene_823	468	AZU37200.1	468	467/468(99.79)	0	transcription termination factor Rho [Wolbachia endosymbiont of Bemisia tabaci]	215173	d__Bacteria;k__norank;p__Proteobacteria;c__Alphaproteobacteria;o__Rickettsiales;f__Anaplasmataceae;g__Wolbachia;s__Wolbachia_endosymbiont_of_Bemisia_tabaci
MDJ-2_gene_825	444	WP_010406848.1	447	419/447(93.74)	2.16e-297	trigger factor [Wolbachia endosymbiont of Nasonia vitripennis]	180837	d__Bacteria;k__norank;p__Proteobacteria;c__Alphaproteobacteria;o__Rickettsiales;f__Anaplasmataceae;g__Wolbachia;s__Wolbachia_endosymbiont_of_Nasonia_vitripennis
MDJ-2_gene_826	213	ONI57058.1	208	203/207(98.07)	5.57e-141	ATP-dependent Clp protease, proteolytic subunit ClpP [Wolbachia pipientis wVitA]	1379786	d__Bacteria;k__norank;p__Proteobacteria;c__Alphaproteobacteria;o__Rickettsiales;f__Anaplasmataceae;g__Wolbachia;s__Wolbachia_pipientis
MDJ-2_gene_827	425	WP_010406852.1	425	414/425(97.41)	1.64e-293	MULTISPECIES: ATP-dependent Clp protease ATP-binding subunit ClpX	2640676	d__Bacteria;k__norank;p__Proteobacteria;c__Alphaproteobacteria;o__Rickettsiales;f__Anaplasmataceae;g__Wolbachia;s__norank
MDJ-2_gene_829	900	WP_010403949.1	900	888/900(98.67)	0	MULTISPECIES: DNA topoisomerase (ATP-hydrolyzing) subunit A [Wolbachia]	180837	d__Bacteria;k__norank;p__Proteobacteria;c__Alphaproteobacteria;o__Rickettsiales;f__Anaplasmataceae;g__Wolbachia;s__Wolbachia_endosymbiont_of_Nasonia_vitripennis
MDJ-2_gene_830	199	PBQ29164.1	199	198/199(99.50)	3.79e-140	ubiquinol-cytochrome c reductase iron-sulfur subunit [Wolbachia pipientis wAus]	2049495	d__Bacteria;k__norank;p__Proteobacteria;c__Alphaproteobacteria;o__Rickettsiales;f__Anaplasmataceae;g__Wolbachia;s__Wolbachia_pipientis
MDJ-2_gene_832	284	WP_010401424.1	297	252/278(90.65)	5.19e-181	ankyrin repeat domain-containing protein [Wolbachia endosymbiont of Nasonia vitripennis]	None	Unclassified
MDJ-2_gene_833	252	AZU37065.1	252	252/252(100.00)	9.54e-183	phosphate ABC transporter ATP-binding protein [Wolbachia endosymbiont of Bemisia tabaci]	215173	d__Bacteria;k__norank;p__Proteobacteria;c__Alphaproteobacteria;o__Rickettsiales;f__Anaplasmataceae;g__Wolbachia;s__Wolbachia_endosymbiont_of_Bemisia_tabaci
MDJ-2_gene_835	65	WP_127463564.1	65	64/65(98.46)	7.49e-36	hypothetical protein [Wolbachia endosymbiont of Bemisia tabaci]	215173	d__Bacteria;k__norank;p__Proteobacteria;c__Alphaproteobacteria;o__Rickettsiales;f__Anaplasmataceae;g__Wolbachia;s__Wolbachia_endosymbiont_of_Bemisia_tabaci
MDJ-2_gene_837	166	PBQ30184.1	166	166/166(100.00)	1.95e-110	5-(carboxyamino)imidazole ribonucleotide mutase [Wolbachia pipientis wAus]	2049495	d__Bacteria;k__norank;p__Proteobacteria;c__Alphaproteobacteria;o__Rickettsiales;f__Anaplasmataceae;g__Wolbachia;s__Wolbachia_pipientis
MDJ-2_gene_838	609	WP_010404258.1	609	602/609(98.85)	0	DNA mismatch repair endonuclease MutL [Wolbachia endosymbiont of Nasonia	180837	d__Bacteria;k__norank;p__Proteobacteria;c__Alphaproteobacteria;o__Rickettsiales;f__Anaplasmataceae;g__Wolbachia;s__Wolbachia_endosymbiont_of_Nasonia_vitripennis
MDJ-2_gene_840	196	WP_010402118.1	196	192/196(97.96)	2.10e-131	MULTISPECIES: orotate phosphoribosyltransferase [Wolbachia]	180837	d__Bacteria;k__norank;p__Proteobacteria;c__Alphaproteobacteria;o__Rickettsiales;f__Anaplasmataceae;g__Wolbachia;s__Wolbachia_endosymbiont_of_Nasonia_vitripennis
MDJ-2_gene_841	394	AGJ98583.1	394	390/394(98.98)	4.41e-264	Sodium/glutamate symporter family protein, putative [Wolbachia endosymbiont of Drosophila simulans wNo]	1236908	d__Bacteria;k__norank;p__Proteobacteria;c__Alphaproteobacteria;o__Rickettsiales;f__Anaplasmataceae;g__Wolbachia;s__Wolbachia_endosymbiont_of_Drosophila_simulans
MDJ-2_gene_843	294	WP_010405304.1	294	286/293(97.61)	1.48e-207	UDP-N-acetylmuramate dehydrogenase [Wolbachia endosymbiont of Nasonia vitripennis]	2640676	d__Bacteria;k__norank;p__Proteobacteria;c__Alphaproteobacteria;o__Rickettsiales;f__Anaplasmataceae;g__Wolbachia;s__norank
MDJ-2_gene_845	49	OCA06908.1	47	38/49(77.55)	1.66e-14	hypothetical protein wTpre_1261 [Wolbachia endosymbiont of Trichogramma pretiosum]	125593	d__Bacteria;k__norank;p__Proteobacteria;c__Alphaproteobacteria;o__Rickettsiales;f__Anaplasmataceae;g__Wolbachia;s__Wolbachia_endosymbiont_of_Trichogramma_pretiosum
MDJ-2_gene_846	809	WP_010402722.1	826	801/809(99.01)	0	hypothetical protein [Wolbachia endosymbiont of Nasonia vitripennis]	180837	d__Bacteria;k__norank;p__Proteobacteria;c__Alphaproteobacteria;o__Rickettsiales;f__Anaplasmataceae;g__Wolbachia;s__Wolbachia_endosymbiont_of_Nasonia_vitripennis

MDJ-2_gene_847	151	WP_064085664.1	149	145/149(97.32)	6.13e-44	ankyrin repeat domain-containing protein [Wolbachia endosymbiont of Dactylopius coccus]	None	Unclassified
MDJ-2_gene_849	197	BBA84820.1	440	103/201(51.24)	3.04e-46	alanine--tRNA ligase [endosymbiont of Pachyrhynchus	1971488	d__Bacteria;k__norank;p__Proteobacteria;c__Gammaproteobacteria;o__norank;f__norank;g__Candidatus_Nardonella;s__endosymbiont_of_Pachyrhynchus_infernalis
MDJ-2_gene_851	141	CRK85687.1	875	82/141(58.16)	3.26e-48	Alanine--tRNA ligase [Candidatus Providencia siddallii]	1715285	d__Bacteria;k__norank;p__Proteobacteria;c__Gammaproteobacteria;o__Enterobacterales;f__Morganellaceae;g__Providencia;s__Candidatus_Providencia_siddallii
MDJ-2_gene_852	291	BBA84819.1	333	248/286(86.71)	3.48e-171	protein RecA [endosymbiont of Pachyrhynchus infernalis]	1971488	d__Bacteria;k__norank;p__Proteobacteria;c__Gammaproteobacteria;o__norank;f__norank;g__Candidatus_Nardonella;s__endosymbiont_of_Pachyrhynchus_infernalis
MDJ-2_gene_853	230	BBA85281.1	379	139/231(60.17)	7.45e-85	chaperone protein DnaJ [endosymbiont of Sipalinus gigas]	1972134	d__Bacteria;k__norank;p__Proteobacteria;c__Gammaproteobacteria;o__norank;f__norank;g__Candidatus_Nardonella;s__endosymbiont_of_Sipalinus_gigas
MDJ-2_gene_855	245	AGJ98500.1	254	243/245(99.18)	2.28e-175	Cytochrome c oxidase subunit 2 [Wolbachia endosymbiont of Drosophila simulans wNo]	1236908	d__Bacteria;k__norank;p__Proteobacteria;c__Alphaproteobacteria;o__Rickettsiales;f__Anaplasmataceae;g__Wolbachia;s__Wolbachia_endosymbiont_of_Drosophila_simulans
MDJ-2_gene_856	503	WP_108784659.1	503	496/503(98.61)	0	HlyD family type I secretion periplasmic adaptor subunit [Wolbachia endosymbiont of Bemisia tabaci]	215173	d__Bacteria;k__norank;p__Proteobacteria;c__Alphaproteobacteria;o__Rickettsiales;f__Anaplasmataceae;g__Wolbachia;s__Wolbachia_endosymbiont_of_Bemisia_tabaci
MDJ-2_gene_858	178	BBA84759.1	422	111/179(62.01)	1.13e-66	serine--tRNA ligase [endosymbiont of Pachyrhynchus	1971488	d__Bacteria;k__norank;p__Proteobacteria;c__Gammaproteobacteria;o__norank;f__norank;g__Candidatus_Nardonella;s__endosymbiont_of_Pachyrhynchus_infernalis
MDJ-2_gene_860	294	BBA84758.1	429	193/293(65.87)	4.02e-114	3-phosphoshikimate 1-carboxyvinyltransferase [endosymbiont of Pachyrhynchus infernalis]	1971488	d__Bacteria;k__norank;p__Proteobacteria;c__Gammaproteobacteria;o__norank;f__norank;g__Candidatus_Nardonella;s__endosymbiont_of_Pachyrhynchus_infernalis
MDJ-2_gene_861	80	AYN24755.1	428	53/80(66.25)	5.45e-29	3-phosphoshikimate 1-carboxyvinyltransferase [Buchnera aphidicola	118109	d__Bacteria;k__norank;p__Proteobacteria;c__Gammaproteobacteria;o__Enterobacterales;f__Erwiniaceae;g__Buchnera;s__Buchnera_aphidicola
MDJ-2_gene_862	94	AZP36166.1	520	45/92(48.91)	2.62e-21	30S ribosomal protein S1 [Candidatus Annandia adelgestsuga]	1302411	d__Bacteria;k__norank;p__Proteobacteria;c__Gammaproteobacteria;o__Enterobacterales;f__Enterobacteriaceae;g__Candidatus_Annandia;s__Candidatus_Annandia_adelgestsuga
MDJ-2_gene_865	417	WP_064085745.1	417	411/417(98.56)	6.10e-310	NADH-quinone oxidoreductase subunit NuoF [Wolbachia endosymbiont of Dactylopius coccus]	None	Unclassified
MDJ-2_gene_866	493	WP_063630981.1	486	353/456(77.41)	4.25e-96	hypothetical protein [Wolbachia endosymbiont of Laodelphax striatella]	368602	d__Bacteria;k__norank;p__Proteobacteria;c__Alphaproteobacteria;o__Rickettsiales;f__Anaplasmataceae;g__Wolbachia;s__Wolbachia_endosymbiont_of_Laodelphax_striatellus
MDJ-2_gene_868	189	WP_065094608.1	305	141/165(85.45)	1.87e-92	XRE family transcriptional regulator [Wolbachia endosymbiont of Nomada	1854760	d__Bacteria;k__norank;p__Proteobacteria;c__Alphaproteobacteria;o__Rickettsiales;f__Anaplasmataceae;g__Wolbachia;s__Wolbachia_endosymbiont_of_Nomada_panzeri
MDJ-2_gene_870	218	WP_064085961.1	218	214/218(98.17)	1.29e-145	DNA repair protein RadC [Wolbachia endosymbiont of Dactylopius coccus]	None	Unclassified
MDJ-2_gene_871	397	WP_019236553.1	373	321/383(83.81)	4.88e-206	hypothetical protein [Wolbachia pipientis]	955	d__Bacteria;k__norank;p__Proteobacteria;c__Alphaproteobacteria;o__Rickettsiales;f__Anaplasmataceae;g__Wolbachia;s__Wolbachia_pipientis
MDJ-2_gene_874	517	PBQ29273.1	517	514/517(99.42)	0	transcription termination/antitermination protein NusA [Wolbachia	2049495	d__Bacteria;k__norank;p__Proteobacteria;c__Alphaproteobacteria;o__Rickettsiales;f__Anaplasmataceae;g__Wolbachia;s__Wolbachia_pipientis

MDJ-2_gene_877	326	AZU37076.1	326	326/326(100.00)	1.66e-236	pyruvate dehydrogenase (acetyl-transferring) E1 component subunit alpha [Wolbachia endosymbiont of Bemisia tabaci]	215173	d__Bacteria;k__norank;p__Proteobacteria;c__Alphaproteobacteria;o__Rickettsiales;f__Anaplasmataceae;g__Wolbachia;s__Wolbachia_endosymbiont_of_Bemisia_tabaci
MDJ-2_gene_878	476	AGJ98496.1	476	473/476(99.37)	0	Aspartyl/glutamyl-tRNA(Asn/Gln) amidotransferase subunit B [Wolbachia endosymbiont of Drosophila	1236908	d__Bacteria;k__norank;p__Proteobacteria;c__Alphaproteobacteria;o__Rickettsiales;f__Anaplasmataceae;g__Wolbachia;s__Wolbachia_endosymbiont_of_Drosophila_simulans
MDJ-2_gene_879	282	EEB55944.1	278	276/277(99.64)	1.53e-193	site-specific recombinase, phage integrase family [Wolbachia endosymbiont of Culex quinquefasciatus JHB]	569881	d__Bacteria;k__norank;p__Proteobacteria;c__Alphaproteobacteria;o__Rickettsiales;f__Anaplasmataceae;g__Wolbachia;s__Wolbachia_endosymbiont_of_Culex_quinquefasciatus
MDJ-2_gene_884	201	PBQ29251.1	257	187/201(93.03)	1.96e-124	hypothetical protein BTO27_00675 [Wolbachia	2049495	d__Bacteria;k__norank;p__Proteobacteria;c__Alphaproteobacteria;o__Rickettsiales;f__Anaplasmataceae;g__Wolbachia;s__Wolbachia_pipientis
MDJ-2_gene_885	885	AGJ98622.1	888	853/881(96.82)	0	2-oxoglutarate dehydrogenase, E1 component [Wolbachia endosymbiont of Drosophila simulans wNo]	1236908	d__Bacteria;k__norank;p__Proteobacteria;c__Alphaproteobacteria;o__Rickettsiales;f__Anaplasmataceae;g__Wolbachia;s__Wolbachia_endosymbiont_of_Drosophila_simulans
MDJ-2_gene_887	181	WP_114517423.1	181	175/181(96.69)	9.22e-105	ankyrin repeat domain-containing protein [Wolbachia endosymbiont of Cylisticus convexus]	118728	d__Bacteria;k__norank;p__Proteobacteria;c__Alphaproteobacteria;o__Rickettsiales;f__Anaplasmataceae;g__Wolbachia;s__Wolbachia_endosymbiont_of_Cylisticus_convexus
MDJ-2_gene_888	75	WP_114517602.1	75	72/75(96.00)	3.97e-40	hypothetical protein [Wolbachia endosymbiont of Cylisticus convexus]	118728	d__Bacteria;k__norank;p__Proteobacteria;c__Alphaproteobacteria;o__Rickettsiales;f__Anaplasmataceae;g__Wolbachia;s__Wolbachia_endosymbiont_of_Cylisticus_convexus
MDJ-2_gene_889	309	CAQ54563.1	307	272/301(90.37)	2.04e-196	patatin family protein [Wolbachia endosymbiont of Culex quinquefasciatus Pel]	570417	d__Bacteria;k__norank;p__Proteobacteria;c__Alphaproteobacteria;o__Rickettsiales;f__Anaplasmataceae;g__Wolbachia;s__Wolbachia_endosymbiont_of_Culex_quinquefasciatus
MDJ-2_gene_890	301	OJH31432.1	307	277/307(90.23)	5.06e-188	Antitoxin PezA [Wolbachia endosymbiont of Armadillidium vulgare]	77039	d__Bacteria;k__norank;p__Proteobacteria;c__Alphaproteobacteria;o__Rickettsiales;f__Anaplasmataceae;g__Wolbachia;s__Wolbachia_endosymbiont_of_Armadillidium_vulgare
MDJ-2_gene_891	234	OJH31433.1	304	213/234(91.03)	1.45e-141	HTH-type transcriptional regulator ImmR [Wolbachia endosymbiont of Armadillidium	77039	d__Bacteria;k__norank;p__Proteobacteria;c__Alphaproteobacteria;o__Rickettsiales;f__Anaplasmataceae;g__Wolbachia;s__Wolbachia_endosymbiont_of_Armadillidium_vulgare
MDJ-2_gene_895	310	PBQ29236.1	310	310/310(100.00)	1.11e-226	riboflavin biosynthesis protein RibF [Wolbachia pipientis wAus]	2049495	d__Bacteria;k__norank;p__Proteobacteria;c__Alphaproteobacteria;o__Rickettsiales;f__Anaplasmataceae;g__Wolbachia;s__Wolbachia_pipientis
MDJ-2_gene_896	120	PBQ29237.1	158	120/120(100.00)	1.08e-84	signal peptidase II [Wolbachia pipientis wAus]	2049495	d__Bacteria;k__norank;p__Proteobacteria;c__Alphaproteobacteria;o__Rickettsiales;f__Anaplasmataceae;g__Wolbachia;s__Wolbachia_pipientis
MDJ-2_gene_899	313	WP_114517193.1	303	281/313(89.78)	7.71e-192	Rpn family recombination-promoting nuclease/putative transposase [Wolbachia endosymbiont of Cylisticus	118728	d__Bacteria;k__norank;p__Proteobacteria;c__Alphaproteobacteria;o__Rickettsiales;f__Anaplasmataceae;g__Wolbachia;s__Wolbachia_endosymbiont_of_Cylisticus_convexus
MDJ-2_gene_900	303	CAQ54347.1	305	250/308(81.17)	1.87e-160	Putative transcriptional regulator [Wolbachia endosymbiont of Culex quinquefasciatus Pel]	570417	d__Bacteria;k__norank;p__Proteobacteria;c__Alphaproteobacteria;o__Rickettsiales;f__Anaplasmataceae;g__Wolbachia;s__Wolbachia_endosymbiont_of_Culex_quinquefasciatus
MDJ-2_gene_902	288	CAQ55062.1	288	280/288(97.22)	9.83e-194	TPR domain protein [Wolbachia endosymbiont of Culex quinquefasciatus Pel]	570417	d__Bacteria;k__norank;p__Proteobacteria;c__Alphaproteobacteria;o__Rickettsiales;f__Anaplasmataceae;g__Wolbachia;s__Wolbachia_endosymbiont_of_Culex_quinquefasciatus
MDJ-2_gene_905	195	ONI55895.1	195	186/195(95.38)	6.06e-135	nitroreductase family protein [Wolbachia pipientis wUni]	1379787	d__Bacteria;k__norank;p__Proteobacteria;c__Alphaproteobacteria;o__Rickettsiales;f__Anaplasmataceae;g__Wolbachia;s__Wolbachia_pipientis

MDJ-2_gene_906	262	RLT60422.1	262	234/262(89.31)	1.37e-171	diaminopimelate epimerase [Wolbachia endosymbiont of Drosophila ananassae]	307502	d__Bacteria;k__norank;p__Proteobacteria;c__Alphaproteobacteria;o__Rickettsiales;f__Anaplasmataceae;g__Wolbachia;s__Wolbachia_endosymbiont_of_Drosophila_ananassae
MDJ-2_gene_910	217	AGJ98695.1	217	214/217(98.62)	3.06e-148	50S ribosomal protein L1 [Wolbachia endosymbiont of Drosophila simulans wNo]	1236908	d__Bacteria;k__norank;p__Proteobacteria;c__Alphaproteobacteria;o__Rickettsiales;f__Anaplasmataceae;g__Wolbachia;s__Wolbachia_endosymbiont_of_Drosophila_simulans
MDJ-2_gene_911	126	PCG11624.1	172	125/126(99.21)	1.08e-75	50S ribosomal protein L10 [Wolbachia pipientis]	None	Unclassified
MDJ-2_gene_915	131	ARC54937.1	132	70/129(54.26)	1.57e-35	30S ribosomal protein S8 [Candidatus Riesia sp. GBBU]	1719125	d__Bacteria;k__norank;p__Proteobacteria;c__Gammaproteobacteria;o__Enterobacterales;f__Enterobacteriaceae;g__Candidatus_Riesia;s__Candidatus_Riesia_sp._GBBU
MDJ-2_gene_916	184	AZP36207.1	177	82/186(44.09)	1.23e-32	50S ribosomal protein L6 [Candidatus Annandia adelgestsuga]	1302411	d__Bacteria;k__norank;p__Proteobacteria;c__Gammaproteobacteria;o__Enterobacterales;f__Enterobacteriaceae;g__Candidatus_Annandia;s__Candidatus_Annandia_adelgestsuga
MDJ-2_gene_917	125	Q8D1Z6.1	117	42/98(42.86)	1.11e-15	RecName: Full=50S ribosomal protein L18	36870	d__Bacteria;k__norank;p__Proteobacteria;c__Gammaproteobacteria;o__Enterobacterales;f__Erwiniaceae;g__Wigglesworthia;s__Wigglesworthia_glossinidia
MDJ-2_gene_918	158	BBA84676.1	160	102/148(68.92)	3.78e-61	30S ribosomal protein S5 [endosymbiont of Euscepes postfasciatus]	650377	d__Bacteria;k__norank;p__Proteobacteria;c__Gammaproteobacteria;o__norank;f__norank;g__norank;s__endosymbiont_of_Euscepes_postfasciatus
MDJ-2_gene_919	149	BBA84675.1	145	79/150(52.67)	3.63e-34	50S ribosomal protein L15 [endosymbiont of Euscepes postfasciatus]	650377	d__Bacteria;k__norank;p__Proteobacteria;c__Gammaproteobacteria;o__norank;f__norank;g__norank;s__endosymbiont_of_Euscepes_postfasciatus
MDJ-2_gene_920	184	VFP85075.1	431	43/101(42.57)	3.32e-06	Preprotein translocase subunit SecY [Buchnera aphidicola (Cinara splendens)]	2518979	d__Bacteria;k__norank;p__Proteobacteria;c__Gammaproteobacteria;o__Enterobacterales;f__Erwiniaceae;g__Buchnera;s__Buchnera_aphidicola
MDJ-2_gene_922	836	AGJ99372.1	836	824/836(98.56)	0	DNA mismatch repair protein MutS [Wolbachia endosymbiont of Drosophila simulans wNo]	1236908	d__Bacteria;k__norank;p__Proteobacteria;c__Alphaproteobacteria;o__Rickettsiales;f__Anaplasmataceae;g__Wolbachia;s__Wolbachia_endosymbiont_of_Drosophila_simulans
MDJ-2_gene_927	50	PBQ26888.1	184	43/46(93.48)	6.72e-24	hypothetical protein BTO27_04095 [Wolbachia	2049495	d__Bacteria;k__norank;p__Proteobacteria;c__Alphaproteobacteria;o__Rickettsiales;f__Anaplasmataceae;g__Wolbachia;s__Wolbachia_pipientis
MDJ-2_gene_931	216	WP_063631240.1	216	212/216(98.15)	1.77e-103	hypothetical protein [Wolbachia endosymbiont of Laodelphax striatella]	2640676	d__Bacteria;k__norank;p__Proteobacteria;c__Alphaproteobacteria;o__Rickettsiales;f__Anaplasmataceae;g__Wolbachia;s__norank
MDJ-2_gene_932	679	WP_026092710.1	1027	661/673(98.22)	0	efflux RND transporter permease subunit [Wolbachia endosymbiont of Diaphorina citri]	None	Unclassified
MDJ-2_gene_939	121	PCG11052.1	124	93/120(77.50)	2.66e-53	hypothetical protein CO539_05455 [Wolbachia	None	Unclassified
MDJ-2_gene_940	504	WP_010405309.1	504	496/502(98.80)	0	MULTISPECIES: protein translocase subunit SecD	2640676	d__Bacteria;k__norank;p__Proteobacteria;c__Alphaproteobacteria;o__Rickettsiales;f__Anaplasmataceae;g__Wolbachia;s__norank
MDJ-2_gene_941	51	WP_010405314.1	83	34/40(85.00)	3.75e-15	transposase [Wolbachia endosymbiont of Nasonia vitripennis]	180837	d__Bacteria;k__norank;p__Proteobacteria;c__Alphaproteobacteria;o__Rickettsiales;f__Anaplasmataceae;g__Wolbachia;s__Wolbachia_endosymbiont_of_Nasonia_vitripennis
MDJ-2_gene_944	633	WP_010404278.1	632	629/632(99.53)	0	RNA polymerase sigma factor RpoD [Wolbachia endosymbiont of Nasonia vitripennis]	180837	d__Bacteria;k__norank;p__Proteobacteria;c__Alphaproteobacteria;o__Rickettsiales;f__Anaplasmataceae;g__Wolbachia;s__Wolbachia_endosymbiont_of_Nasonia_vitripennis
MDJ-2_gene_947	799	AGJ98551.1	2380	581/799(72.72)	0	Ankyrin repeat domain protein [Wolbachia endosymbiont of Drosophila simulans wNo]	1236908	d__Bacteria;k__norank;p__Proteobacteria;c__Alphaproteobacteria;o__Rickettsiales;f__Anaplasmataceae;g__Wolbachia;s__Wolbachia_endosymbiont_of_Drosophila_simulans

MDJ-2_gene_954	235	PBQ28052.1	235	233/235(99.15)	2.52e-165	pyridoxine 5'-phosphate synthase [Wolbachia pipientis wAus]	2049495	d__Bacteria;k__norank;p__Proteobacteria;c__Alphaproteobacteria;o__Rickettsiales;f__Anaplasmataceae;g__Wolbachia;s__Wolbachia_pipientis
MDJ-2_gene_955	103	WP_068652405.1	103	96/103(93.20)	9.06e-61	HU family DNA-binding protein [Wolbachia endosymbiont of Trichogramma pretiosum]	125593	d__Bacteria;k__norank;p__Proteobacteria;c__Alphaproteobacteria;o__Rickettsiales;f__Anaplasmataceae;g__Wolbachia;s__Wolbachia_endosymbiont_of_Trichogramma_pretiosum
MDJ-2_gene_956	145	WP_127463760.1	149	144/145(99.31)	1.65e-95	30S ribosomal protein S9 [Wolbachia endosymbiont of	215173	d__Bacteria;k__norank;p__Proteobacteria;c__Alphaproteobacteria;o__Rickettsiales;f__Anaplasmataceae;g__Wolbachia;s__Wolbachia_endosymbiont_of_Bemisia_tabaci
MDJ-2_gene_957	152	PBQ28049.1	152	152/152(100.00)	4.40e-107	50S ribosomal protein L13 [Wolbachia pipientis wAus]	2049495	d__Bacteria;k__norank;p__Proteobacteria;c__Alphaproteobacteria;o__Rickettsiales;f__Anaplasmataceae;g__Wolbachia;s__Wolbachia_pipientis
MDJ-2_gene_959	168	WP_127463874.1	168	162/168(96.43)	2.37e-112	inorganic diphosphatase [Wolbachia endosymbiont of	2640676	d__Bacteria;k__norank;p__Proteobacteria;c__Alphaproteobacteria;o__Rickettsiales;f__Anaplasmataceae;g__Wolbachia;s__norank
MDJ-2_gene_960	418	WP_068651847.1	418	382/418(91.39)	2.66e-268	hypothetical protein [Wolbachia endosymbiont of Trichogramma pretiosum]	125593	d__Bacteria;k__norank;p__Proteobacteria;c__Alphaproteobacteria;o__Rickettsiales;f__Anaplasmataceae;g__Wolbachia;s__Wolbachia_endosymbiont_of_Trichogramma_pretiosum
MDJ-2_gene_961	370	WP_127463872.1	370	362/370(97.84)	3.23e-263	tRNA 2-thiouridine(34) synthase MnmA [Wolbachia endosymbiont of Bemisia tabaci]	215173	d__Bacteria;k__norank;p__Proteobacteria;c__Alphaproteobacteria;o__Rickettsiales;f__Anaplasmataceae;g__Wolbachia;s__Wolbachia_endosymbiont_of_Bemisia_tabaci
MDJ-2_gene_964	648	AGJ98552.1	600	102/214(47.66)	2.85e-45	Ankyrin repeat domain protein [Wolbachia endosymbiont of Drosophila simulans wNo]	1236908	d__Bacteria;k__norank;p__Proteobacteria;c__Alphaproteobacteria;o__Rickettsiales;f__Anaplasmataceae;g__Wolbachia;s__Wolbachia_endosymbiont_of_Drosophila_simulans
MDJ-2_gene_972	457	WP_127463905.1	482	447/457(97.81)	1.10e-302	cation:proton antiporter [Wolbachia endosymbiont of	215173	d__Bacteria;k__norank;p__Proteobacteria;c__Alphaproteobacteria;o__Rickettsiales;f__Anaplasmataceae;g__Wolbachia;s__Wolbachia_endosymbiont_of_Bemisia_tabaci
MDJ-2_gene_973	101	WP_010404196.1	101	93/101(92.08)	1.77e-66	MULTISPECIES: NADH:ubiquinone	2640676	d__Bacteria;k__norank;p__Proteobacteria;c__Alphaproteobacteria;o__Rickettsiales;f__Anaplasmataceae;g__Wolbachia;s__norank
MDJ-2_gene_981	679	WP_010402142.1	679	676/679(99.56)	0	ATP-dependent DNA helicase RecG [Wolbachia endosymbiont of Nasonia vitripennis]	180837	d__Bacteria;k__norank;p__Proteobacteria;c__Alphaproteobacteria;o__Rickettsiales;f__Anaplasmataceae;g__Wolbachia;s__Wolbachia_endosymbiont_of_Nasonia_vitripennis
MDJ-2_gene_988	97	PBQ28253.1	97	95/97(97.94)	6.59e-58	hypothetical protein BTO27_01935 [Wolbachia	2049495	d__Bacteria;k__norank;p__Proteobacteria;c__Alphaproteobacteria;o__Rickettsiales;f__Anaplasmataceae;g__Wolbachia;s__Wolbachia_pipientis
MDJ-2_gene_993	400	EEB55791.1	400	396/400(99.00)	1.19e-271	major facilitator family transporter [Wolbachia endosymbiont of Culex	569881	d__Bacteria;k__norank;p__Proteobacteria;c__Alphaproteobacteria;o__Rickettsiales;f__Anaplasmataceae;g__Wolbachia;s__Wolbachia_endosymbiont_of_Culex_quinquefasciatus
MDJ-2_gene_995	451	AZU37400.1	729	288/344(83.72)	6.51e-197	hypothetical protein BBB02_02240 [Wolbachia endosymbiont of Bemisia tabaci]	215173	d__Bacteria;k__norank;p__Proteobacteria;c__Alphaproteobacteria;o__Rickettsiales;f__Anaplasmataceae;g__Wolbachia;s__Wolbachia_endosymbiont_of_Bemisia_tabaci
MDJ-2_gene_996	638	PCG11851.1	634	626/634(98.74)	0	molecular chaperone HtpG [Wolbachia pipientis]	None	Unclassified
MDJ-2_gene_998	393	WP_010406367.1	393	387/393(98.47)	1.07e-275	MULTISPECIES: hypothetical protein [Wolbachia]	2640676	d__Bacteria;k__norank;p__Proteobacteria;c__Alphaproteobacteria;o__Rickettsiales;f__Anaplasmataceae;g__Wolbachia;s__norank
MDJ-2_gene_1000	258	WP_010406361.1	258	255/258(98.84)	4.17e-180	MULTISPECIES: SDR family oxidoreductase [Wolbachia]	180837	d__Bacteria;k__norank;p__Proteobacteria;c__Alphaproteobacteria;o__Rickettsiales;f__Anaplasmataceae;g__Wolbachia;s__Wolbachia_endosymbiont_of_Nasonia_vitripennis
MDJ-2_gene_1001	57	KIM03216.1	75	30/57(52.63)	4.57e-14	30S ribosomal protein S16 [Sulfurovum sp. FS06-10]	1539064	d__Bacteria;k__norank;p__Proteobacteria;c__Epsilonproteobacteria;o__Campylobacterales;f__Sulfurovaceae;g__Sulfurovum;s__Sulfurovum_sp._FS06-10
MDJ-2_gene_1003	74	BBA84883.1	343	41/74(55.41)	7.15e-18	UDP-N-acetylenolpyruvoylglucosamine reductase [endosymbiont of Pachyrhynchus infernalis]	1971488	d__Bacteria;k__norank;p__Proteobacteria;c__Gammaproteobacteria;o__norank;f__norank;g__Candidatus_Nardonella;s__endosymbiont_of_Pachyrhynchus_infernalis

MDJ-2_gene_1004	86	BBA84883.1	343	41/80(51.25)	2.63e-12	UDP-N-acetylenolpyruvoylglucosamine reductase [endosymbiont of Pachyrhynchus infernalis]	1971488	d__Bacteria;k__norank;p__Proteobacteria;c__Gammaproteobacteria;o__norank;f__norank;g__Candidatus_Nardonella;s__endosymbiont_of_Pachyrhynchus_infernalis
MDJ-2_gene_1005	463	BBA85220.1	625	308/459(67.10)	4.10e-209	tRNA uridine 5-carboxymethylaminomethyl modification enzyme MnmG [endosymbiont of Sipalinus gigas]	1972134	d__Bacteria;k__norank;p__Proteobacteria;c__Gammaproteobacteria;o__norank;f__norank;g__Candidatus_Nardonella;s__endosymbiont_of_Sipalinus_gigas
MDJ-2_gene_1011	197	PBQ29224.1	197	194/197(98.48)	1.17e-133	recombination protein RecR [Wolbachia pipientis wAus]	2049495	d__Bacteria;k__norank;p__Proteobacteria;c__Alphaproteobacteria;o__Rickettsiales;f__Anaplasmataceae;g__Wolbachia;s__Wolbachia_pipientis
MDJ-2_gene_1012	276	PBQ29223.1	462	247/276(89.49)	3.43e-167	hypothetical protein BTO27_00910 [Wolbachia	2049495	d__Bacteria;k__norank;p__Proteobacteria;c__Alphaproteobacteria;o__Rickettsiales;f__Anaplasmataceae;g__Wolbachia;s__Wolbachia_pipientis
MDJ-2_gene_1013	183	CAQ54876.1	462	149/178(83.71)	1.06e-94	Ankyrin repeat domain protein [Wolbachia endosymbiont of Culex quinquefasciatus Pel]	570417	d__Bacteria;k__norank;p__Proteobacteria;c__Alphaproteobacteria;o__Rickettsiales;f__Anaplasmataceae;g__Wolbachia;s__Wolbachia_endosymbiont_of_Culex_quinquefasciatus
MDJ-2_gene_1014	263	PBQ29244.1	263	258/263(98.10)	1.52e-189	prolipoprotein diacylglyceryl transferase [Wolbachia pipientis wAus]	2049495	d__Bacteria;k__norank;p__Proteobacteria;c__Alphaproteobacteria;o__Rickettsiales;f__Anaplasmataceae;g__Wolbachia;s__Wolbachia_pipientis
MDJ-2_gene_1015	149	PBQ29243.1	149	149/149(100.00)	1.17e-96	SsrA-binding protein [Wolbachia pipientis wAus]	2049495	d__Bacteria;k__norank;p__Proteobacteria;c__Alphaproteobacteria;o__Rickettsiales;f__Anaplasmataceae;g__Wolbachia;s__Wolbachia_pipientis
MDJ-2_gene_1016	303	WP_017531779.1	303	281/303(92.74)	1.06e-73	ankyrin repeat domain-containing protein [Wolbachia endosymbiont of Diaphorina citri]	116598	d__Bacteria;k__norank;p__Proteobacteria;c__Alphaproteobacteria;o__Rickettsiales;f__Anaplasmataceae;g__Wolbachia;s__Wolbachia_endosymbiont_of_Diaphorina_citri
MDJ-2_gene_1020	186	CAQ55144.1	186	182/186(97.85)	6.77e-114	Outer membrane protein [Wolbachia endosymbiont of Culex quinquefasciatus Pel]	570417	d__Bacteria;k__norank;p__Proteobacteria;c__Alphaproteobacteria;o__Rickettsiales;f__Anaplasmataceae;g__Wolbachia;s__Wolbachia_endosymbiont_of_Culex_quinquefasciatus
MDJ-2_gene_1021	146	WP_064125117.1	146	144/146(98.63)	1.96e-98	3-hydroxyacyl-ACP dehydratase FabZ [Wolbachia endosymbiont of Dactylopius coccus]	None	Unclassified
MDJ-2_gene_1025	267	PCG11210.1	267	241/267(90.26)	2.02e-171	hypothetical protein CO539_04525 [Wolbachia	None	Unclassified
MDJ-2_gene_1026	276	PCG11214.1	357	254/268(94.78)	2.36e-173	hypothetical protein CO539_04520 [Wolbachia	None	Unclassified
MDJ-2_gene_1027	100	AGJ99418.1	328	76/83(91.57)	4.62e-42	hypothetical protein wNo_10470 [Wolbachia endosymbiont of Drosophila simulans wNo]	1236908	d__Bacteria;k__norank;p__Proteobacteria;c__Alphaproteobacteria;o__Rickettsiales;f__Anaplasmataceae;g__Wolbachia;s__Wolbachia_endosymbiont_of_Drosophila_simulans
MDJ-2_gene_1034	51	CQD08251.1	51	50/51(98.04)	7.21e-28	phosphatidylglycerophosphatase A [Wolbachia endosymbiont wPip_Mol of Culex molestus]	1402163	d__Bacteria;k__norank;p__Proteobacteria;c__Alphaproteobacteria;o__Rickettsiales;f__Anaplasmataceae;g__Wolbachia;s__Wolbachia_endosymbiont_of_Culex_molestus
MDJ-2_gene_1036	263	BAH22198.1	263	263/263(100.00)	1.66e-193	acetyltranferase, GNAT family [Wolbachia endosymbiont of Cadra cautella]	190193	d__Bacteria;k__norank;p__Proteobacteria;c__Alphaproteobacteria;o__Rickettsiales;f__Anaplasmataceae;g__Wolbachia;s__Wolbachia_endosymbiont_of_Cadra_cautella
MDJ-2_gene_1037	378	WP_019236658.1	378	377/378(99.74)	1.54e-265	hypothetical protein [Wolbachia pipientis]	953	d__Bacteria;k__norank;p__Proteobacteria;c__Alphaproteobacteria;o__Rickettsiales;f__Anaplasmataceae;g__Wolbachia;s__norank
MDJ-2_gene_1040	461	WP_010403833.1	461	452/461(98.05)	0	amidophosphoribosyltransferase [Wolbachia endosymbiont of Nasonia vitripennis]	180837	d__Bacteria;k__norank;p__Proteobacteria;c__Alphaproteobacteria;o__Rickettsiales;f__Anaplasmataceae;g__Wolbachia;s__Wolbachia_endosymbiont_of_Nasonia_vitripennis
MDJ-2_gene_1042	155	PBQ28054.1	151	149/149(100.00)	5.62e-106	heat-shock protein Hsp20 [Wolbachia pipientis wAus]	2049495	d__Bacteria;k__norank;p__Proteobacteria;c__Alphaproteobacteria;o__Rickettsiales;f__Anaplasmataceae;g__Wolbachia;s__Wolbachia_pipientis

MDJ-2_gene_1043	450	PBQ28055.1	450	429/450(95.33)	0	glutamate--tRNA ligase [Wolbachia pipientis wAus]	2049495	d__Bacteria;k__norank;p__Proteobacteria;c__Alphaproteobacteria;o__Rickettsiales;f__Anaplasmataceae;g__Wolbachia;s__Wolbachia_pipientis
MDJ-2_gene_1046	651	AGJ99409.1	651	646/651(99.23)	0	DNA ligase [Wolbachia endosymbiont of Drosophila simulans wNo]	1236908	d__Bacteria;k__norank;p__Proteobacteria;c__Alphaproteobacteria;o__Rickettsiales;f__Anaplasmataceae;g__Wolbachia;s__Wolbachia_endosymbiont_of_Drosophila_simulans
MDJ-2_gene_1049	82	BBA84612.1	821	36/77(46.75)	1.27e-10	valine--tRNA ligase [endosymbiont of Euscepes	650377	d__Bacteria;k__norank;p__Proteobacteria;c__Gammaproteobacteria;o__norank;f__norank;g__norank;s__endosymbiont_of_Euscepes_postfasciatus
MDJ-2_gene_1051	112	BBA84912.1	871	51/113(45.13)	7.92e-18	valine--tRNA ligase [endosymbiont of Pachyrhynchus	1971488	d__Bacteria;k__norank;p__Proteobacteria;c__Gammaproteobacteria;o__norank;f__norank;g__Candidatus_Nardonella;s__endosymbiont_of_Pachyrhynchus_infernalis
MDJ-2_gene_1052	84	BBA84612.1	821	42/81(51.85)	1.44e-13	valine--tRNA ligase [endosymbiont of Euscepes	650377	d__Bacteria;k__norank;p__Proteobacteria;c__Gammaproteobacteria;o__norank;f__norank;g__norank;s__endosymbiont_of_Euscepes_postfasciatus
MDJ-2_gene_1055	163	BAH22213.1	163	152/163(93.25)	4.29e-108	sigma-70 region 2 domain protein [Wolbachia endosymbiont of Cadra cautella]	190193	d__Bacteria;k__norank;p__Proteobacteria;c__Alphaproteobacteria;o__Rickettsiales;f__Anaplasmataceae;g__Wolbachia;s__Wolbachia_endosymbiont_of_Cadra_cautella
MDJ-2_gene_1056	239	BAH22212.1	265	234/239(97.91)	3.12e-174	hypothetical protein [Wolbachia endosymbiont of Cadra cautella]	190193	d__Bacteria;k__norank;p__Proteobacteria;c__Alphaproteobacteria;o__Rickettsiales;f__Anaplasmataceae;g__Wolbachia;s__Wolbachia_endosymbiont_of_Cadra_cautella
MDJ-2_gene_1058	316	WP_064085471.1	321	314/316(99.37)	3.63e-218	MULTISPECIES: complex I NDUFA9 subunit family protein	None	Unclassified
MDJ-2_gene_1059	318	WP_010405340.1	318	315/318(99.06)	2.03e-217	ketoacyl-ACP synthase III [Wolbachia endosymbiont of Nasonia vitripennis]	180837	d__Bacteria;k__norank;p__Proteobacteria;c__Alphaproteobacteria;o__Rickettsiales;f__Anaplasmataceae;g__Wolbachia;s__Wolbachia_endosymbiont_of_Nasonia_vitripennis
MDJ-2_gene_1062	48	PBQ29251.1	257	48/48(100.00)	4.25e-23	hypothetical protein BTO27_00675 [Wolbachia	2049495	d__Bacteria;k__norank;p__Proteobacteria;c__Alphaproteobacteria;o__Rickettsiales;f__Anaplasmataceae;g__Wolbachia;s__Wolbachia_pipientis
MDJ-2_gene_1064	197	PBQ29250.1	197	197/197(100.00)	1.18e-142	hypothetical protein BTO27_00670 [Wolbachia	2049495	d__Bacteria;k__norank;p__Proteobacteria;c__Alphaproteobacteria;o__Rickettsiales;f__Anaplasmataceae;g__Wolbachia;s__Wolbachia_pipientis
MDJ-2_gene_1065	394	PBQ29268.1	479	302/484(62.40)	2.32e-179	hypothetical protein BTO27_00665 [Wolbachia	2049495	d__Bacteria;k__norank;p__Proteobacteria;c__Alphaproteobacteria;o__Rickettsiales;f__Anaplasmataceae;g__Wolbachia;s__Wolbachia_pipientis
MDJ-2_gene_1068	159	WP_064085991.1	159	158/159(99.37)	2.06e-113	dihydrofolate reductase [Wolbachia endosymbiont of Dactylopius coccus]	None	Unclassified
MDJ-2_gene_1069	417	WP_064085992.1	417	413/417(99.04)	3.24e-305	dihydropteroate synthase [Wolbachia endosymbiont of Dactylopius coccus]	None	Unclassified
MDJ-2_gene_1070	128	WP_127463799.1	128	122/128(95.31)	3.87e-85	FolB domain-containing protein [Wolbachia endosymbiont of Bemisia tabaci]	215173	d__Bacteria;k__norank;p__Proteobacteria;c__Alphaproteobacteria;o__Rickettsiales;f__Anaplasmataceae;g__Wolbachia;s__Wolbachia_endosymbiont_of_Bemisia_tabaci
MDJ-2_gene_1071	219	WP_108784268.1	219	217/219(99.09)	4.38e-158	CADD family putative folate metabolism protein [Wolbachia endosymbiont of Bemisia tabaci]	215173	d__Bacteria;k__norank;p__Proteobacteria;c__Alphaproteobacteria;o__Rickettsiales;f__Anaplasmataceae;g__Wolbachia;s__Wolbachia_endosymbiont_of_Bemisia_tabaci
MDJ-2_gene_1078	184	CQD09626.1	184	181/184(98.37)	5.27e-135	3-methyladenine DNA glycosylase [Wolbachia endosymbiont wPip_Mol of	1402163	d__Bacteria;k__norank;p__Proteobacteria;c__Alphaproteobacteria;o__Rickettsiales;f__Anaplasmataceae;g__Wolbachia;s__Wolbachia_endosymbiont_of_Culex_molestus
MDJ-2_gene_1079	58	PBQ30140.1	329	52/54(96.30)	1.11e-21	hypothetical protein BTO27_00070 [Wolbachia	2049495	d__Bacteria;k__norank;p__Proteobacteria;c__Alphaproteobacteria;o__Rickettsiales;f__Anaplasmataceae;g__Wolbachia;s__Wolbachia_pipientis
MDJ-2_gene_1083	74	WP_108784436.1	378	45/72(62.50)	1.37e-19	magnesium transporter [Wolbachia endosymbiont of	215173	d__Bacteria;k__norank;p__Proteobacteria;c__Alphaproteobacteria;o__Rickettsiales;f__Anaplasmataceae;g__Wolbachia;s__Wolbachia_endosymbiont_of_Bemisia_tabaci
MDJ-2_gene_1084	204	WP_017532622.1	423	193/279(69.18)	3.90e-73	hypothetical protein [Wolbachia endosymbiont of Diaphorina citri]	116598	d__Bacteria;k__norank;p__Proteobacteria;c__Alphaproteobacteria;o__Rickettsiales;f__Anaplasmataceae;g__Wolbachia;s__Wolbachia_endosymbiont_of_Diaphorina_citri
MDJ-2_gene_1086	46	QBB83836.1	62	44/46(95.65)	5.26e-17	hypothetical protein DEJ70_03340 [Wolbachia	1116230	d__Bacteria;k__norank;p__Proteobacteria;c__Alphaproteobacteria;o__Rickettsiales;f__Anaplasmataceae;g__Wolbachia;s__Wolbachia_pipientis

MDJ-2_gene_1091	244	WP_064085469.1	244	241/244(98.77)	2.05e-164	hypothetical protein [Wolbachia endosymbiont of Dactylopius coccus]	None	Unclassified
MDJ-2_gene_1094	117	RDD34783.1	117	114/117(97.44)	3.32e-74	NAD(P)H-quinone oxidoreductase subunit 3 [Wolbachia endosymbiont of	118728	d__Bacteria;k__norank;p__Proteobacteria;c__Alphaproteobacteria;o__Rickettsiales;f__Anaplasmataceae;g__Wolbachia;s__Wolbachia_endosymbiont_of_Cylisticus_convexus
MDJ-2_gene_1096	373	AAX73214.1	881	141/430(32.79)	8.77e-38	cell surface antigen Sca12, partial [Rickettsia africae]	35788	d__Bacteria;k__norank;p__Proteobacteria;c__Alphaproteobacteria;o__Rickettsiales;f__Rickettsiaceae;g__Rickettsia;s__Rickettsia_africae
MDJ-2_gene_1101	230	AFA41078.1	234	75/215(34.88)	4.54e-23	50S ribosomal subunit protein L1 [Wigglesworthia glossinidia endosymbiont of Glossina morsitans morsitans (Yale	1142511	d__Bacteria;k__norank;p__Proteobacteria;c__Gammaproteobacteria;o__Enterobacterales;f__Erwiniaceae;g__Wigglesworthia;s__Wigglesworthia_glossinidia
MDJ-2_gene_1104	299	WP_097032089.1	394	240/298(80.54)	6.98e-176	elongation factor Tu [Sodalis endosymbiont of Henestaris halophilus]	1929246	d__Bacteria;k__norank;p__Proteobacteria;c__Gammaproteobacteria;o__Enterobacterales;f__Pectobacteriaceae;g__Sodalis;s__Sodalis_endosymbiont_of_Henestaris_halophilus
MDJ-2_gene_1108	159	KDB19169.1	157	132/155(85.16)	2.22e-94	hypothetical protein wGmm_0974 [Wolbachia endosymbiont of Glossina morsitans morsitans]	1150948	d__Bacteria;k__norank;p__Proteobacteria;c__Alphaproteobacteria;o__Rickettsiales;f__Anaplasmataceae;g__Wolbachia;s__Wolbachia_endosymbiont_of_Glossina_morsitans_morsitans
MDJ-2_gene_1109	154	WP_019236416.1	154	141/154(91.56)	4.89e-96	phage baseplate assembly protein V [Wolbachia pipientis]	955	d__Bacteria;k__norank;p__Proteobacteria;c__Alphaproteobacteria;o__Rickettsiales;f__Anaplasmataceae;g__Wolbachia;s__Wolbachia_pipientis
MDJ-2_gene_1110	84	WP_127464045.1	84	46/49(93.88)	4.80e-27	hypothetical protein [Wolbachia endosymbiont of Bemisia tabaci]	215173	d__Bacteria;k__norank;p__Proteobacteria;c__Alphaproteobacteria;o__Rickettsiales;f__Anaplasmataceae;g__Wolbachia;s__Wolbachia_endosymbiont_of_Bemisia_tabaci
MDJ-2_gene_1111	111	WP_077188131.1	111	103/111(92.79)	7.45e-66	baseplate assembly protein W [Wolbachia pipientis]	955	d__Bacteria;k__norank;p__Proteobacteria;c__Alphaproteobacteria;o__Rickettsiales;f__Anaplasmataceae;g__Wolbachia;s__Wolbachia_pipientis
MDJ-2_gene_1115	495	PCG11073.1	495	493/495(99.60)	0	UbiD family decarboxylase [Wolbachia pipientis]	None	Unclassified
MDJ-2_gene_1118	142	WP_096676744.1	142	139/142(97.89)	3.85e-94	6,7-dimethyl-8-ribityllumazine synthase [Wolbachia pipientis]	955	d__Bacteria;k__norank;p__Proteobacteria;c__Alphaproteobacteria;o__Rickettsiales;f__Anaplasmataceae;g__Wolbachia;s__Wolbachia_pipientis
MDJ-2_gene_1119	160	PBQ29282.1	160	160/160(100.00)	8.38e-109	transcription antitermination factor NusB [Wolbachia pipientis	2049495	d__Bacteria;k__norank;p__Proteobacteria;c__Alphaproteobacteria;o__Rickettsiales;f__Anaplasmataceae;g__Wolbachia;s__Wolbachia_pipientis
MDJ-2_gene_1122	153	AGJ99652.1	153	149/153(97.39)	3.09e-103	Deoxyuridine 5'-triphosphate nucleotidohydrolase [Wolbachia endosymbiont of Drosophila simulans wHa]	1236909	d__Bacteria;k__norank;p__Proteobacteria;c__Alphaproteobacteria;o__Rickettsiales;f__Anaplasmataceae;g__Wolbachia;s__Wolbachia_endosymbiont_of_Drosophila_simulans
MDJ-2_gene_1134	339	CAQ54539.1	339	338/339(99.71)	2.68e-244	Putative virulence factor RhuM protein [Wolbachia endosymbiont of Culex quinquefasciatus Pel]	570417	d__Bacteria;k__norank;p__Proteobacteria;c__Alphaproteobacteria;o__Rickettsiales;f__Anaplasmataceae;g__Wolbachia;s__Wolbachia_endosymbiont_of_Culex_quinquefasciatus
MDJ-2_gene_1135	163	WP_115955885.1	803	47/128(36.72)	2.67e-11	outer membrane protein assembly factor BamA [Candidatus Purcelliella pentastirinorum]	None	Unclassified
MDJ-2_gene_1136	307	AEO08593.1	800	75/227(33.04)	1.71e-11	outer membrane associated protein [Buchnera aphidicola str. Ak (Acyrthosiphon kondoi)]	1005090	d__Bacteria;k__norank;p__Proteobacteria;c__Gammaproteobacteria;o__Enterobacterales;f__Erwiniaceae;g__Buchnera;s__Buchnera_aphidicola
MDJ-2_gene_1137	121	BBA85059.1	930	67/122(54.92)	1.11e-29	DNA-directed DNA polymerase [endosymbiont of Rhynchophorus ferrugineus]	1972133	d__Bacteria;k__norank;p__Proteobacteria;c__Gammaproteobacteria;o__norank;f__norank;g__Candidatus_Nardonella;s__Candidatus_Nardonella_dryophthoridicola
MDJ-2_gene_1138	104	BBA85299.1	921	65/107(60.75)	5.91e-23	DNA polymerase III subunit alpha [endosymbiont of Sipalinus	1972134	d__Bacteria;k__norank;p__Proteobacteria;c__Gammaproteobacteria;o__norank;f__norank;g__Candidatus_Nardonella;s__endosymbiont_of_Sipalinus_gigas

MDJ-2_gene_1139	85	BBA84796.1	921	58/85(68.24)	3.57e-30	DNA-directed DNA polymerase [endosymbiont of Pachyrhynchus infernalis]	1971488	d__Bacteria;k__norank;p__Proteobacteria;c__Gammaproteobacteria;o__norank;f__norank;g__Candidatus_Nardonella;s__endosymbiont_of_Pachyrhynchus_infernalis
MDJ-2_gene_1147	207	BBA84670.1	206	138/207(66.67)	7.54e-84	30S ribosomal protein S4 [endosymbiont of Euscepes postfasciatus]	650377	d__Bacteria;k__norank;p__Proteobacteria;c__Gammaproteobacteria;o__norank;f__norank;g__norank;s__endosymbiont_of_Euscepes_postfasciatus
MDJ-2_gene_1148	323	BBA85241.1	329	168/323(52.01)	1.94e-101	DNA-directed RNA polymerase subunit alpha [endosymbiont of Sipalinus gigas]	1972134	d__Bacteria;k__norank;p__Proteobacteria;c__Gammaproteobacteria;o__norank;f__norank;g__Candidatus_Nardonella;s__endosymbiont_of_Sipalinus_gigas
MDJ-2_gene_1152	289	WP_019236939.1	289	287/289(99.31)	3.70e-194	SPFH domain-containing protein [Wolbachia pipientis]	953	d__Bacteria;k__norank;p__Proteobacteria;c__Alphaproteobacteria;o__Rickettsiales;f__Anaplasmataceae;g__Wolbachia;s__norank
MDJ-2_gene_1154	285	WP_017531719.1	689	174/259(67.18)	1.37e-104	hypothetical protein [Wolbachia endosymbiont of Diaphorina citri]	116598	d__Bacteria;k__norank;p__Proteobacteria;c__Alphaproteobacteria;o__Rickettsiales;f__Anaplasmataceae;g__Wolbachia;s__Wolbachia_endosymbiont_of_Diaphorina_citri
MDJ-2_gene_1155	420	WP_096616605.1	424	206/422(48.82)	4.22e-22	ankyrin repeat domain-containing protein [Wolbachia pipientis]	955	d__Bacteria;k__norank;p__Proteobacteria;c__Alphaproteobacteria;o__Rickettsiales;f__Anaplasmataceae;g__Wolbachia;s__Wolbachia_pipientis
MDJ-2_gene_1159	167	WP_127463623.1	170	150/169(88.76)	2.04e-95	phage tail protein [Wolbachia endosymbiont of Bemisia tabaci]	215173	d__Bacteria;k__norank;p__Proteobacteria;c__Alphaproteobacteria;o__Rickettsiales;f__Anaplasmataceae;g__Wolbachia;s__Wolbachia_endosymbiont_of_Bemisia_tabaci
MDJ-2_gene_1161	154	WP_064124760.1	154	142/154(92.21)	7.24e-98	phage baseplate assembly protein V [Wolbachia endosymbiont of Dactylopius coccus]	None	Unclassified
MDJ-2_gene_1163	111	OJH30454.1	111	108/111(97.30)	5.67e-70	Gene 25-like lysozyme [Armadillidium vulgare]	77039	d__Bacteria;k__norank;p__Proteobacteria;c__Alphaproteobacteria;o__Rickettsiales;f__Anaplasmataceae;g__Wolbachia;s__Wolbachia_endosymbiont_of_Armadillidium_vulgare
MDJ-2_gene_1165	217	BBA84779.1	539	103/206(50.00)	3.00e-49	methionine--tRNA ligase [endosymbiont of Pachyrhynchus infernalis]	1971488	d__Bacteria;k__norank;p__Proteobacteria;c__Gammaproteobacteria;o__norank;f__norank;g__Candidatus_Nardonella;s__endosymbiont_of_Pachyrhynchus_infernalis
MDJ-2_gene_1166	202	AAC97364.1	234	107/192(55.73)	7.86e-62	methionyl-tRNA synthetase, partial [Buchnera aphidicola]	9	d__Bacteria;k__norank;p__Proteobacteria;c__Gammaproteobacteria;o__Enterobacterales;f__Erwiniaceae;g__Buchnera;s__Buchnera_aphidicola
MDJ-2_gene_1168	683	WP_108784308.1	683	653/683(95.61)	0	hypothetical protein [Wolbachia endosymbiont of Bemisia tabaci]	215173	d__Bacteria;k__norank;p__Proteobacteria;c__Alphaproteobacteria;o__Rickettsiales;f__Anaplasmataceae;g__Wolbachia;s__Wolbachia_endosymbiont_of_Bemisia_tabaci
MDJ-2_gene_1172	89	PCG11431.1	318	77/89(86.52)	7.87e-37	hypothetical protein CO539_03250 [Wolbachia	None	Unclassified
MDJ-2_gene_1173	228	WP_010404795.1	228	222/228(97.37)	1.98e-154	outer membrane protein assembly factor BamD [Wolbachia endosymbiont of Nasonia vitripennis]	180837	d__Bacteria;k__norank;p__Proteobacteria;c__Alphaproteobacteria;o__Rickettsiales;f__Anaplasmataceae;g__Wolbachia;s__Wolbachia_endosymbiont_of_Nasonia_vitripennis
MDJ-2_gene_1174	246	AZU37749.1	246	244/246(99.19)	2.82e-172	transcriptional regulator [Wolbachia endosymbiont of	215173	d__Bacteria;k__norank;p__Proteobacteria;c__Alphaproteobacteria;o__Rickettsiales;f__Anaplasmataceae;g__Wolbachia;s__Wolbachia_endosymbiont_of_Bemisia_tabaci
MDJ-2_gene_1180	750	RDD34432.1	720	536/745(71.95)	0	ankyrin repeat domain-containing protein [Wolbachia endosymbiont of Cylisticus convexus]	118728	d__Bacteria;k__norank;p__Proteobacteria;c__Alphaproteobacteria;o__Rickettsiales;f__Anaplasmataceae;g__Wolbachia;s__Wolbachia_endosymbiont_of_Cylisticus_convexus
MDJ-2_gene_1185	331	WP_017532159.1	331	321/331(96.98)	1.07e-230	2-nitropropane dioxygenase [Wolbachia endosymbiont of Diaphorina citri]	116598	d__Bacteria;k__norank;p__Proteobacteria;c__Alphaproteobacteria;o__Rickettsiales;f__Anaplasmataceae;g__Wolbachia;s__Wolbachia_endosymbiont_of_Diaphorina_citri
MDJ-2_gene_1186	205	VAX76287.1	275	107/186(57.53)	6.68e-55	Ribosomal RNA small subunit methyltransferase I [Buchnera aphidicola (Cinara strobi)]	1921549	d__Bacteria;k__norank;p__Proteobacteria;c__Gammaproteobacteria;o__Enterobacterales;f__Erwiniaceae;g__Buchnera;s__Buchnera_aphidicola
MDJ-2_gene_1188	112	AWH90631.1	110	31/71(43.66)	1.24e-12	preprotein translocase subunit YajC [Buchnera aphidicola (Melanaphis sacchari)]	2173854	d__Bacteria;k__norank;p__Proteobacteria;c__Gammaproteobacteria;o__Enterobacterales;f__Erwiniaceae;g__Buchnera;s__Buchnera_aphidicola

MDJ-2_gene_1192	413	PBQ26871.1	413	407/413(98.55)	4.51e-294	IscS subfamily cysteine desulfurase [Wolbachia pipientis	2049495	d__Bacteria;k__norank;p__Proteobacteria;c__Alphaproteobacteria;o__Rickettsiales;f__Anaplasmataceae;g__Wolbachia;s__Wolbachia_pipientis
MDJ-2_gene_1194	478	AGJ98617.1	498	456/470(97.02)	0	Archaeal ATPase family protein [Wolbachia endosymbiont of Drosophila simulans wNo]	1236908	d__Bacteria;k__norank;p__Proteobacteria;c__Alphaproteobacteria;o__Rickettsiales;f__Anaplasmataceae;g__Wolbachia;s__Wolbachia_endosymbiont_of_Drosophila_simulans
MDJ-2_gene_1196	140	AVP40189.1	470	76/134(56.72)	3.06e-35	hypothetical protein vBEcoSSa179w3YLVW_00003 [Escherichia phage vB_EcoS Sa179lw]	2126819	d__Viruses;k__norank;p__Uroviricota;c__Caudoviricetes;o__Caudovirales;f__Siphoviridae;g__norank;s__Escherichia_phage_vB_EcoS_Sa179lw
MDJ-2_gene_1197	145	WP_064559194.1	144	106/145(73.10)	3.17e-60	hypothetical protein [Buttiauxella brennerae]	82988	d__Bacteria;k__norank;p__Proteobacteria;c__Gammaproteobacteria;o__Enterobacterales;f__Enterobacteriaceae;g__Buttiauxella;s__Buttiauxella_brennerae
MDJ-2_gene_1198	328	ORJ47853.1	343	234/343(68.22)	1.23e-162	encapsulating for peroxidase [Kluyvera intermedia]	61648	d__Bacteria;k__norank;p__Proteobacteria;c__Gammaproteobacteria;o__Enterobacterales;f__Enterobacteriaceae;g__Kluyvera;s__Kluyvera_intermedia
MDJ-2_gene_1203	96	PBQ26344.1	96	96/96(100.00)	3.15e-60	co-chaperone GroES [Wolbachia pipientis wAus]	2049495	d__Bacteria;k__norank;p__Proteobacteria;c__Alphaproteobacteria;o__Rickettsiales;f__Anaplasmataceae;g__Wolbachia;s__Wolbachia_pipientis
MDJ-2_gene_1204	265	PBQ26345.1	265	263/265(99.25)	6.96e-187	glycine cleavage system protein T [Wolbachia pipientis wAus]	2049495	d__Bacteria;k__norank;p__Proteobacteria;c__Alphaproteobacteria;o__Rickettsiales;f__Anaplasmataceae;g__Wolbachia;s__Wolbachia_pipientis
MDJ-2_gene_1205	78	WP_064085373.1	81	68/68(100.00)	3.49e-36	IS200/IS605 family transposase [Wolbachia endosymbiont of Dactylopius coccus]	None	Unclassified
MDJ-2_gene_1206	70	WP_052264914.1	275	66/67(98.51)	5.22e-40	hypothetical protein, partial [Wolbachia endosymbiont of Operophtera brumata]	1620420	d__Bacteria;k__norank;p__Proteobacteria;c__Alphaproteobacteria;o__Rickettsiales;f__Anaplasmataceae;g__Wolbachia;s__Wolbachia_endosymbiont_of_Operophtera_brumata
MDJ-2_gene_1207	472	OJH31403.1	472	467/472(98.94)	0	Archaeal ATPase [Wolbachia endosymbiont of Armadillidium vulgare]	77039	d__Bacteria;k__norank;p__Proteobacteria;c__Alphaproteobacteria;o__Rickettsiales;f__Anaplasmataceae;g__Wolbachia;s__Wolbachia_endosymbiont_of_Armadillidium_vulgare
MDJ-2_gene_1209	476	PCG11601.1	476	474/476(99.58)	0	metalloprotease TldD [Wolbachia pipientis]	None	Unclassified
MDJ-2_gene_1215	308	POG49325.1	416	296/311(95.18)	3.41e-207	citrate (Si)-synthase [Wolbachia sp. wMel_AMD]	1912095	d__Bacteria;k__norank;p__Proteobacteria;c__Alphaproteobacteria;o__Rickettsiales;f__Anaplasmataceae;g__Wolbachia;s__Wolbachia_sp._wMel_AMD
MDJ-2_gene_1216	89	PBQ29142.1	89	89/89(100.00)	1.11e-50	30S ribosomal protein S20 [Wolbachia pipientis wAus]	2049495	d__Bacteria;k__norank;p__Proteobacteria;c__Alphaproteobacteria;o__Rickettsiales;f__Anaplasmataceae;g__Wolbachia;s__Wolbachia_pipientis
MDJ-2_gene_1217	195	WP_029237643.1	241	195/195(100.00)	4.25e-132	1-acyl-sn-glycerol-3-phosphate acyltransferase [Wolbachia endosymbiont of Nasonia vitripennis]	2640676	d__Bacteria;k__norank;p__Proteobacteria;c__Alphaproteobacteria;o__Rickettsiales;f__Anaplasmataceae;g__Wolbachia;s__norank
MDJ-2_gene_1219	280	WP_063631149.1	273	179/281(63.70)	3.84e-99	hypothetical protein [Wolbachia endosymbiont of Laodelphax striatella]	368602	d__Bacteria;k__norank;p__Proteobacteria;c__Alphaproteobacteria;o__Rickettsiales;f__Anaplasmataceae;g__Wolbachia;s__Wolbachia_endosymbiont_of_Laodelphax_striatellus
MDJ-2_gene_1220	163	CAQ54392.1	172	152/162(93.83)	9.60e-98	Putative transposase [Wolbachia endosymbiont of Culex quinquefasciatus Pel]	570417	d__Bacteria;k__norank;p__Proteobacteria;c__Alphaproteobacteria;o__Rickettsiales;f__Anaplasmataceae;g__Wolbachia;s__Wolbachia_endosymbiont_of_Culex_quinquefasciatus
MDJ-2_gene_1223	391	WP_010402333.1	433	328/392(83.67)	2.08e-227	molecular chaperone DnaJ [Wolbachia endosymbiont of Nasonia vitripennis]	180837	d__Bacteria;k__norank;p__Proteobacteria;c__Alphaproteobacteria;o__Rickettsiales;f__Anaplasmataceae;g__Wolbachia;s__Wolbachia_endosymbiont_of_Nasonia_vitripennis
MDJ-2_gene_1224	469	WP_108784574.1	475	453/469(96.59)	0	isocitrate dehydrogenase [Wolbachia endosymbiont of Bemisia tabaci]	215173	d__Bacteria;k__norank;p__Proteobacteria;c__Alphaproteobacteria;o__Rickettsiales;f__Anaplasmataceae;g__Wolbachia;s__Wolbachia_endosymbiont_of_Bemisia_tabaci
MDJ-2_gene_1226	120	ONI57840.1	123	116/119(97.48)	8.22e-77	helix-turn-helix family protein [Wolbachia pipientis wVitA]	1379786	d__Bacteria;k__norank;p__Proteobacteria;c__Alphaproteobacteria;o__Rickettsiales;f__Anaplasmataceae;g__Wolbachia;s__Wolbachia_pipientis

MDJ-2_gene_1227	110	AGJ99406.1	110	102/110(92.73)	1.63e-64	Putative transcriptional regulator [Wolbachia endosymbiont of Drosophila simulans wNo]	1236908	d__Bacteria;k__norank;p__Proteobacteria;c__Alphaproteobacteria;o__Rickettsiales;f__Anaplasmataceae;g__Wolbachia;s__Wolbachia_endosymbiont_of_Drosophila_simulans
MDJ-2_gene_1228	219	AGJ98929.1	219	209/219(95.43)	1.34e-143	DNA repair protein RadC 1 [Wolbachia endosymbiont of Drosophila simulans wNo]	1236908	d__Bacteria;k__norank;p__Proteobacteria;c__Alphaproteobacteria;o__Rickettsiales;f__Anaplasmataceae;g__Wolbachia;s__Wolbachia_endosymbiont_of_Drosophila_simulans
MDJ-2_gene_1230	213	WP_010401658.1	213	210/213(98.59)	9.96e-152	HAD family hydrolase [Wolbachia endosymbiont of Nasonia vitripennis]	180837	d__Bacteria;k__norank;p__Proteobacteria;c__Alphaproteobacteria;o__Rickettsiales;f__Anaplasmataceae;g__Wolbachia;s__Wolbachia_endosymbiont_of_Nasonia_vitripennis
MDJ-2_gene_1231	157	PBQ27239.1	157	149/157(94.90)	2.14e-96	hypothetical protein BTO27_03825 [Wolbachia	2049495	d__Bacteria;k__norank;p__Proteobacteria;c__Alphaproteobacteria;o__Rickettsiales;f__Anaplasmataceae;g__Wolbachia;s__Wolbachia_pipientis
MDJ-2_gene_1232	158	WP_114517623.1	158	150/158(94.94)	2.92e-99	bacterioferritin [Wolbachia endosymbiont of Cylisticus convexus]	118728	d__Bacteria;k__norank;p__Proteobacteria;c__Alphaproteobacteria;o__Rickettsiales;f__Anaplasmataceae;g__Wolbachia;s__Wolbachia_endosymbiont_of_Cylisticus_convexus
MDJ-2_gene_1236	152	PCG11169.1	152	151/152(99.34)	1.94e-101	molecular chaperone Hsc20 [Wolbachia pipientis]	None	Unclassified
MDJ-2_gene_1240	136	RDD33928.1	123	113/123(91.87)	9.85e-71	head decoration protein [Wolbachia endosymbiont of Cylisticus convexus]	118728	d__Bacteria;k__norank;p__Proteobacteria;c__Alphaproteobacteria;o__Rickettsiales;f__Anaplasmataceae;g__Wolbachia;s__Wolbachia_endosymbiont_of_Cylisticus_convexus
MDJ-2_gene_1243	288	WP_063630548.1	288	284/288(98.61)	2.22e-190	hypothetical protein [Wolbachia endosymbiont of Laodelphax striatella]	368602	d__Bacteria;k__norank;p__Proteobacteria;c__Alphaproteobacteria;o__Rickettsiales;f__Anaplasmataceae;g__Wolbachia;s__Wolbachia_endosymbiont_of_Laodelphax_striatellus
MDJ-2_gene_1244	188	WP_063630549.1	188	186/188(98.94)	6.35e-129	phosphoribosylglycinamide formyltransferase [Wolbachia endosymbiont of Laodelphax striatella]	368602	d__Bacteria;k__norank;p__Proteobacteria;c__Alphaproteobacteria;o__Rickettsiales;f__Anaplasmataceae;g__Wolbachia;s__Wolbachia_endosymbiont_of_Laodelphax_striatellus
MDJ-2_gene_1250	293	RKP09962.1	571	120/250(48.00)	3.44e-71	glycoside hydrolase [Thamnocephalis sphaerospora]	78915	d__Eukaryota;k__Fungi;p__Zoopagomycota;c__Zoopagomycetes;o__Zoopagales;f__Sigmoideomycetaceae;g__Thamnocephalis;s__Thamnocephalis_sphaerospora
MDJ-2_gene_1251	84	AYR02254.1	157	51/71(71.83)	1.22e-29	hypothetical protein [Escherichia phage C1]	2340716	d__Viruses;k__norank;p__Uroviricota;c__Caudoviricetes;o__Caudovirales;f__Siphoviridae;g__norank;s__Escherichia_phage_C1
MDJ-2_gene_1252	157	TCW00382.1	157	108/157(68.79)	2.29e-75	uncharacterized protein DUF1833 [Biostraticola tofi]	466109	d__Bacteria;k__norank;p__Proteobacteria;c__Gammaproteobacteria;o__Enterobacterales;f__Pectobacteriaceae;g__Biostraticola;s__Biostraticola_tofi
MDJ-2_gene_1253	130	WP_120131262.1	143	78/130(60.00)	1.33e-57	peptidoglycan endopeptidase [Rahnella woolbedingensis]	1510574	d__Bacteria;k__norank;p__Proteobacteria;c__Gammaproteobacteria;o__Enterobacterales;f__Yersiniaceae;g__Rahnella;s__Rahnella_woolbedingensis
MDJ-2_gene_1256	271	WP_114516977.1	265	235/265(88.68)	1.00e-167	baseplate assembly protein J [Wolbachia endosymbiont of Cylisticus convexus]	118728	d__Bacteria;k__norank;p__Proteobacteria;c__Alphaproteobacteria;o__Rickettsiales;f__Anaplasmataceae;g__Wolbachia;s__Wolbachia_endosymbiont_of_Cylisticus_convexus
MDJ-2_gene_1257	111	BAH22230.1	111	100/111(90.09)	6.37e-67	baseplate assembly protein GpW [Wolbachia endosymbiont of Cadra cautella]	190193	d__Bacteria;k__norank;p__Proteobacteria;c__Alphaproteobacteria;o__Rickettsiales;f__Anaplasmataceae;g__Wolbachia;s__Wolbachia_endosymbiont_of_Cadra_cautella
MDJ-2_gene_1259	111	PBQ27674.1	126	76/83(91.57)	3.49e-49	hypothetical protein BTO27_03020 [Wolbachia	2049495	d__Bacteria;k__norank;p__Proteobacteria;c__Alphaproteobacteria;o__Rickettsiales;f__Anaplasmataceae;g__Wolbachia;s__Wolbachia_pipientis
MDJ-2_gene_1260	217	WP_010403901.1	217	217/217(100.00)	9.60e-161	glutathione S-transferase family protein [Wolbachia endosymbiont of Nasonia vitripennis]	180837	d__Bacteria;k__norank;p__Proteobacteria;c__Alphaproteobacteria;o__Rickettsiales;f__Anaplasmataceae;g__Wolbachia;s__Wolbachia_endosymbiont_of_Nasonia_vitripennis
MDJ-2_gene_1261	225	PCG11922.1	225	222/225(98.67)	4.03e-157	O-methyltransferase [Wolbachia pipientis]	None	Unclassified
MDJ-2_gene_1270	140	AEH39755.1	595	52/142(36.62)	1.81e-16	GTP-binding elongation factor [Buchnera aphidicola (Cinara tujafilina)]	261317	d__Bacteria;k__norank;p__Proteobacteria;c__Gammaproteobacteria;o__Enterobacterales;f__Erwiniaceae;g__Buchnera;s__Buchnera_aphidicola

MDJ-2_gene_1271	262	BBA84784.1	602	134/263(50.95)	2.83e-68	elongation factor 4 [endosymbiont of Pachyrhynchus	1971488	d__Bacteria;k__norank;p__Proteobacteria;c__Gammaproteobacteria;o__norank;f__norank;g__Candidatus_Nardonella;s__endosymbiont_of_Pachyrhynchus_infernalis
MDJ-2_gene_1272	199	BBA84782.1	205	85/152(55.92)	8.12e-44	protein GrpE [endosymbiont of Pachyrhynchus infernalis]	1971488	d__Bacteria;k__norank;p__Proteobacteria;c__Gammaproteobacteria;o__norank;f__norank;g__Candidatus_Nardonella;s__endosymbiont_of_Pachyrhynchus_infernalis
MDJ-2_gene_1277	128	WP_064124661.1	128	123/128(96.09)	6.95e-79	30S ribosomal protein S11 [Wolbachia endosymbiont of Dactylopius coccus]	None	Unclassified
MDJ-2_gene_1278	372	WP_064085600.1	355	349/355(98.31)	4.41e-251	DNA-directed RNA polymerase subunit alpha [Wolbachia endosymbiont of Dactylopius coccus]	None	Unclassified
MDJ-2_gene_1279	142	PBQ28109.1	142	142/142(100.00)	4.50e-93	50S ribosomal protein L17 [Wolbachia pipientis wAus]	2049495	d__Bacteria;k__norank;p__Proteobacteria;c__Alphaproteobacteria;o__Rickettsiales;f__Anaplasmataceae;g__Wolbachia;s__Wolbachia_pipientis
MDJ-2_gene_1298	318	WP_064085574.1	318	312/318(98.11)	3.86e-206	hypothetical protein [Wolbachia endosymbiont of Dactylopius coccus]	None	Unclassified
MDJ-2_gene_1305	323	CAQ54264.1	323	318/323(98.45)	1.03e-233	glyceraldehyde 3-phosphate dehydrogenase [Wolbachia endosymbiont of Culex quinquefasciatus Pel]	570417	d__Bacteria;k__norank;p__Proteobacteria;c__Alphaproteobacteria;o__Rickettsiales;f__Anaplasmataceae;g__Wolbachia;s__Wolbachia_endosymbiont_of_Culex_quinquefasciatus
MDJ-2_gene_1308	404	BBA85323.1	436	231/406(56.90)	2.76e-143	ATP-dependent protease ATPase subunit HslU [endosymbiont of Sipalinus gigas]	1972134	d__Bacteria;k__norank;p__Proteobacteria;c__Gammaproteobacteria;o__norank;f__norank;g__Candidatus_Nardonella;s__endosymbiont_of_Sipalinus_gigas
MDJ-2_gene_1312	229	WP_064085243.1	235	200/201(99.50)	6.02e-135	uroporphyrinogen-III synthase [Wolbachia endosymbiont of Dactylopius coccus]	None	Unclassified
MDJ-2_gene_1320	388	WP_064085277.1	388	385/388(99.23)	9.56e-281	bifunctional 2-polyprenyl-6-hydroxyphenol methylase/3-demethylubiquinol 3-O-methyltransferase UbiG [Wolbachia endosymbiont of Dactylopius coccus]	None	Unclassified
MDJ-2_gene_1329	747	OJH31309.1	1214	589/748(78.74)	0	Phosphocholine transferase AnkX [Wolbachia endosymbiont of Armadillidium vulgare]	77039	d__Bacteria;k__norank;p__Proteobacteria;c__Alphaproteobacteria;o__Rickettsiales;f__Anaplasmataceae;g__Wolbachia;s__Wolbachia_endosymbiont_of_Armadillidium_vulgare
MDJ-2_gene_1332	70	Q7VQD9.1	85	34/60(56.67)	3.69e-16	RecName: Full=30S ribosomal protein S17	203907	d__Bacteria;k__norank;p__Proteobacteria;c__Gammaproteobacteria;o__Enterobacterales;f__Enterobacteriaceae;g__Candidatus_Blochmannia;s__Candidatus_Blochmannia_floridanus
MDJ-2_gene_1334	134	ADV33600.1	137	86/130(66.15)	1.00e-54	50S ribosomal protein L16 [Candidatus Blochmannia vafer str. BVAF]	859654	d__Bacteria;k__norank;p__Proteobacteria;c__Gammaproteobacteria;o__Enterobacterales;f__Enterobacteriaceae;g__Candidatus_Blochmannia;s__Candidatus_Blochmannia_vafer
MDJ-2_gene_1336	112	AMA64773.1	110	55/108(50.93)	5.19e-27	50S ribosomal protein L22 [Candidatus Arsenophonus lipoptenae]	634113	d__Bacteria;k__norank;p__Proteobacteria;c__Gammaproteobacteria;o__Enterobacterales;f__Morganellaceae;g__Arsenophonus;s__Candidatus_Arsenophonus_lipoptenae
MDJ-2_gene_1337	56	AEO08289.1	100	39/53(73.58)	2.94e-21	30S ribosomal protein S19 [Buchnera aphidicola str. Ua (Uroleucon ambrosiae)]	1005057	d__Bacteria;k__norank;p__Proteobacteria;c__Gammaproteobacteria;o__Enterobacterales;f__Erwiniaceae;g__Buchnera;s__Buchnera_aphidicola
MDJ-2_gene_1339	633	WP_010404130.1	633	606/633(95.73)	0	threonine--tRNA ligase [Wolbachia endosymbiont of Nasonia vitripennis]	180837	d__Bacteria;k__norank;p__Proteobacteria;c__Alphaproteobacteria;o__Rickettsiales;f__Anaplasmataceae;g__Wolbachia;s__Wolbachia_endosymbiont_of_Nasonia_vitripennis

MDJ-2_gene_1340	192	BBA85174.1	458	102/189(53.97)	9.98e-46	UDP-N-acetylmuramyl pentapeptide synthase [endosymbiont of Sipalinus gigas]	1972134	d__Bacteria;k__norank;p__Proteobacteria;c__Gammaproteobacteria;o__norank;f__norank;g__Candidatus_Nardonella;s__endosymbiont_of_Sipalinus_gigas
MDJ-2_gene_1341	225	BBA84763.1	457	122/225(54.22)	6.29e-65	UDP-N-acetylmuramoyl-tripeptide--D-alanyl-D-alanine ligase [endosymbiont of Pachyrhynchus infernalis]	1971488	d__Bacteria;k__norank;p__Proteobacteria;c__Gammaproteobacteria;o__norank;f__norank;g__Candidatus_Nardonella;s__endosymbiont_of_Pachyrhynchus_infernalis
MDJ-2_gene_1342	57	BBA84762.1	491	32/58(55.17)	3.56e-09	UDP-N-acetylmuramoyl-L-alanyl-D-glutamate--2 6-diaminopimelate ligase [endosymbiont of Pachyrhynchus	1971488	d__Bacteria;k__norank;p__Proteobacteria;c__Gammaproteobacteria;o__norank;f__norank;g__Candidatus_Nardonella;s__endosymbiont_of_Pachyrhynchus_infernalis
MDJ-2_gene_1343	121	BBA84762.1	491	67/129(51.94)	2.49e-26	UDP-N-acetylmuramoyl-L-alanyl-D-glutamate--2 6-diaminopimelate ligase [endosymbiont of Pachyrhynchus	1971488	d__Bacteria;k__norank;p__Proteobacteria;c__Gammaproteobacteria;o__norank;f__norank;g__Candidatus_Nardonella;s__endosymbiont_of_Pachyrhynchus_infernalis
MDJ-2_gene_1350	447	WP_065094739.1	447	443/447(99.11)	1.72e-297	MULTISPECIES: signal recognition particle protein	2640676	d__Bacteria;k__norank;p__Proteobacteria;c__Alphaproteobacteria;o__Rickettsiales;f__Anaplasmataceae;g__Wolbachia;s__norank
MDJ-2_gene_1352	489	ODV83828.1	56	22/54(40.74)	8.33e-06	hypothetical protein CANARDRAFT_202073, partial [[Candida] arabinofermentans NRRL YB-2248]	983967	d__Eukaryota;k__Fungi;p__Ascomycota;c__Saccharomycetes;o__Saccharomycetales;f__Pichiaceae;g__Ogataea;s__[Candida]_arabinofermentans
MDJ-2_gene_1357	109	BBA84726.1	223	60/100(60.00)	5.70e-33	30S ribosomal protein S2 [endosymbiont of Euscepes postfasciatus]	650377	d__Bacteria;k__norank;p__Proteobacteria;c__Gammaproteobacteria;o__norank;f__norank;g__norank;s__endosymbiont_of_Euscepes_postfasciatus
MDJ-2_gene_1358	69	BBA84801.1	224	34/69(49.28)	2.65e-10	30S ribosomal protein S2 [endosymbiont of Pachyrhynchus infernalis]	1971488	d__Bacteria;k__norank;p__Proteobacteria;c__Gammaproteobacteria;o__norank;f__norank;g__Candidatus_Nardonella;s__endosymbiont_of_Pachyrhynchus_infernalis
MDJ-2_gene_1359	221	AWI49719.1	261	131/219(59.82)	4.87e-84	methionine aminopeptidase [Buchnera aphidicola (Schizaphis graminum)]	98794	d__Bacteria;k__norank;p__Proteobacteria;c__Gammaproteobacteria;o__Enterobacterales;f__Erwiniaceae;g__Buchnera;s__Buchnera_aphidicola
MDJ-2_gene_1364	264	WP_129827269.1	285	223/264(84.47)	1.69e-155	IS3 family transposase [Wolbachia pipientis]	955	d__Bacteria;k__norank;p__Proteobacteria;c__Alphaproteobacteria;o__Rickettsiales;f__Anaplasmataceae;g__Wolbachia;s__Wolbachia_pipientis
MDJ-2_gene_1365	43	EAL59200.1	44	30/39(76.92)	4.73e-12	transposase [Wolbachia endosymbiont of Drosophila ananassae]	307502	d__Bacteria;k__norank;p__Proteobacteria;c__Alphaproteobacteria;o__Rickettsiales;f__Anaplasmataceae;g__Wolbachia;s__Wolbachia_endosymbiont_of_Drosophila_ananassae
MDJ-2_gene_1366	312	WP_114517191.1	390	271/308(87.99)	3.20e-172	hypothetical protein [Wolbachia endosymbiont of Cylisticus convexus]	118728	d__Bacteria;k__norank;p__Proteobacteria;c__Alphaproteobacteria;o__Rickettsiales;f__Anaplasmataceae;g__Wolbachia;s__Wolbachia_endosymbiont_of_Cylisticus_convexus
MDJ-2_gene_1368	60	WP_080600789.1	60	60/60(100.00)	2.16e-35	MULTISPECIES: hypothetical protein [Wolbachia]	953	d__Bacteria;k__norank;p__Proteobacteria;c__Alphaproteobacteria;o__Rickettsiales;f__Anaplasmataceae;g__Wolbachia;s__norank
MDJ-2_gene_1370	46	ADW80226.1	365	31/33(93.94)	4.00e-09	ISSod13 transposase [Wolbachia endosymbiont wVitB of Nasonia vitripennis phage WOVitB]	949127	d__Viruses;k__norank;p__Uroviricota;c__Caudoviricetes;o__Caudovirales;f__Myoviridae;g__norank;s__Wolbachia_endosymbiont_wVitB_of_Nasonia_vitripennis_phage_WOVitB
MDJ-2_gene_1371	293	ADW80226.1	365	235/247(95.14)	8.79e-166	ISSod13 transposase [Wolbachia endosymbiont wVitB of Nasonia vitripennis phage WOVitB]	949127	d__Viruses;k__norank;p__Uroviricota;c__Caudoviricetes;o__Caudovirales;f__Myoviridae;g__norank;s__Wolbachia_endosymbiont_wVitB_of_Nasonia_vitripennis_phage_WOVitB
MDJ-2_gene_1372	112	WP_081600829.1	157	89/104(85.58)	1.59e-54	hypothetical protein [Wolbachia endosymbiont of Drosophila simulans]	77038	d__Bacteria;k__norank;p__Proteobacteria;c__Alphaproteobacteria;o__Rickettsiales;f__Anaplasmataceae;g__Wolbachia;s__Wolbachia_endosymbiont_of_Drosophila_simulans

MDJ-2_gene_1374	434	BBA85381.1	516	210/439(47.84)	9.88e-113	ATP-dependent RNA helicase DeaD [endosymbiont of Sipalinus	1972134	d__Bacteria;k__norank;p__Proteobacteria;c__Gammaproteobacteria;o__norank;f__norank;g__Candidatus_Nardonella;s__endosymbiont_of_Sipalinus_gigas
MDJ-2_gene_1375	260	BBA85382.1	382	123/251(49.00)	3.49e-61	proline--tRNA ligase [endosymbiont of Sipalinus gigas]	1972134	d__Bacteria;k__norank;p__Proteobacteria;c__Gammaproteobacteria;o__norank;f__norank;g__Candidatus_Nardonella;s__endosymbiont_of_Sipalinus_gigas
MDJ-2_gene_1378	217	RUA07048.1	924	59/191(30.89)	1.49e-17	hypothetical protein DSY43_00675	1913989	d__Bacteria;k__norank;p__Proteobacteria;c__Gammaproteobacteria;o__norank;f__norank;g__norank;s__Gammaproteobacteria_bacterium
MDJ-2_gene_1381	77	WP_114517730.1	77	74/77(96.10)	2.01e-43	hypothetical protein [Wolbachia endosymbiont of Cylisticus convexus]	118728	d__Bacteria;k__norank;p__Proteobacteria;c__Alphaproteobacteria;o__Rickettsiales;f__Anaplasmataceae;g__Wolbachia;s__Wolbachia_endosymbiont_of_Cylisticus_convexus
MDJ-2_gene_1382	440	BAA89629.1	448	339/440(77.05)	1.21e-247	gp3 [Wolbachia phage WO]	112596	d__Viruses;k__norank;p__Uroviricota;c__Caudoviricetes;o__Caudovirales;f__Myoviridae;g__norank;s__Wolbachia_phage_WO
MDJ-2_gene_1384	143	WP_029237644.1	195	142/143(99.30)	3.40e-84	MULTISPECIES: NADH-quinone oxidoreductase subunit J [Wolbachia]	2640676	d__Bacteria;k__norank;p__Proteobacteria;c__Alphaproteobacteria;o__Rickettsiales;f__Anaplasmataceae;g__Wolbachia;s__norank
MDJ-2_gene_1386	453	WP_064085800.1	157	151/153(98.69)	6.35e-97	hypothetical protein [Wolbachia endosymbiont of Dactylopius coccus]	None	Unclassified
MDJ-2_gene_1387	258	OUW49260.1	1085	75/216(34.72)	7.67e-12	hypothetical protein CBD47_02085 [Synechococcus	1986595	d__Bacteria;k__norank;p__Cyanobacteria;c__norank;o__Synechococcales;f__Synechococcaceae;g__Synechococcus;s__Synechococcus_sp._TMED187
MDJ-2_gene_1389	104	BBA84792.1	426	51/104(49.04)	1.09e-17	histidine--tRNA ligase [endosymbiont of Pachyrhynchus infernalis]	1971488	d__Bacteria;k__norank;p__Proteobacteria;c__Gammaproteobacteria;o__norank;f__norank;g__Candidatus_Nardonella;s__endosymbiont_of_Pachyrhynchus_infernalis
MDJ-2_gene_1391	235	AFA41097.1	418	130/234(55.56)	1.32e-79	serine hydroxymethyltransferase [Wigglesworthia glossinidia endosymbiont of Glossina morsitans morsitans (Yale	1142511	d__Bacteria;k__norank;p__Proteobacteria;c__Gammaproteobacteria;o__Enterobacterales;f__Erwiniaceae;g__Wigglesworthia;s__Wigglesworthia_glossinidia
MDJ-2_gene_1392	180	ANF17051.1	420	114/174(65.52)	1.01e-73	serine hydroxymethyltransferase [Buchnera aphidicola (Schlechtendalia chinensis)]	118110	d__Bacteria;k__norank;p__Proteobacteria;c__Gammaproteobacteria;o__Enterobacterales;f__Erwiniaceae;g__Buchnera;s__Buchnera_aphidicola
MDJ-2_gene_1393	75	PIZ76646.1	440	35/74(47.30)	6.15e-13	UDP-N-acetylglucosamine 1-carboxyvinyltransferase [Candidatus Peregrinibacteria bacterium	1974774	d__Bacteria;k__norank;p__Candidatus_Peregrinibacteria;c__norank;o__norank;f__norank;g__norank;s__Candidatus_Peregrinibacteria_bacterium_CG_4_10_14_0_2_um_filter_38_24
MDJ-2_gene_1398	205	PBQ25778.1	209	203/205(99.02)	3.57e-142	oxidoreductase [Wolbachia pipientis wAus]	2049495	d__Bacteria;k__norank;p__Proteobacteria;c__Alphaproteobacteria;o__Rickettsiales;f__Anaplasmataceae;g__Wolbachia;s__Wolbachia_pipientis
MDJ-2_gene_1399	354	PBQ25777.1	375	350/354(98.87)	1.22e-239	peptidylprolyl isomerase [Wolbachia pipientis wAus]	2049495	d__Bacteria;k__norank;p__Proteobacteria;c__Alphaproteobacteria;o__Rickettsiales;f__Anaplasmataceae;g__Wolbachia;s__Wolbachia_pipientis
MDJ-2_gene_1401	125	PBQ26851.1	125	125/125(100.00)	3.35e-80	molecular chaperone DnaK [Wolbachia pipientis wAus]	2049495	d__Bacteria;k__norank;p__Proteobacteria;c__Alphaproteobacteria;o__Rickettsiales;f__Anaplasmataceae;g__Wolbachia;s__Wolbachia_pipientis
MDJ-2_gene_1402	215	PBQ26850.1	215	213/215(99.07)	5.86e-136	transcriptional regulator [Wolbachia pipientis wAus]	2049495	d__Bacteria;k__norank;p__Proteobacteria;c__Alphaproteobacteria;o__Rickettsiales;f__Anaplasmataceae;g__Wolbachia;s__Wolbachia_pipientis
MDJ-2_gene_1404	296	AGJ98777.1	333	244/264(92.42)	2.89e-107	Ankyrin repeat domain protein [Wolbachia endosymbiont of Drosophila simulans wNo]	1236908	d__Bacteria;k__norank;p__Proteobacteria;c__Alphaproteobacteria;o__Rickettsiales;f__Anaplasmataceae;g__Wolbachia;s__Wolbachia_endosymbiont_of_Drosophila_simulans
MDJ-2_gene_1405	395	WP_064085665.1	391	382/389(98.20)	3.80e-285	phage portal protein [Wolbachia endosymbiont of Dactylopius coccus]	None	Unclassified

MDJ-2_gene_1408	197	WP_064085882.1	197	193/197(97.97)	2.46e-135	YihA family ribosome biogenesis GTP-binding protein [Wolbachia endosymbiont of Dactylopius coccus]	2640676	d__Bacteria;k__norank;p__Proteobacteria;c__Alphaproteobacteria;o__Rickettsiales;f__Anaplasmataceae;g__Wolbachia;s__norank
MDJ-2_gene_1410	610	EAL60172.1	607	585/609(96.06)	0	Phage terminase large subunit (GpA) [Wolbachia endosymbiont of Drosophila simulans]	77038	d__Bacteria;k__norank;p__Proteobacteria;c__Alphaproteobacteria;o__Rickettsiales;f__Anaplasmataceae;g__Wolbachia;s__Wolbachia_endosymbiont_of_Drosophila_simulans
MDJ-2_gene_1411	71	AGJ99297.1	71	61/71(85.92)	1.33e-34	Putative phage head stabilizing protein GpW [Wolbachia endosymbiont of Drosophila simulans wNo]	1236908	d__Bacteria;k__norank;p__Proteobacteria;c__Alphaproteobacteria;o__Rickettsiales;f__Anaplasmataceae;g__Wolbachia;s__Wolbachia_endosymbiont_of_Drosophila_simulans
MDJ-2_gene_1414	245	WP_010403278.1	245	236/245(96.33)	7.72e-165	hypothetical protein [Wolbachia endosymbiont of Nasonia vitripennis]	180837	d__Bacteria;k__norank;p__Proteobacteria;c__Alphaproteobacteria;o__Rickettsiales;f__Anaplasmataceae;g__Wolbachia;s__Wolbachia_endosymbiont_of_Nasonia_vitripennis
MDJ-2_gene_1426	440	WP_102800868.1	440	401/440(91.14)	2.98e-299	IS4 family transposase [Dickeya zeae]	204037	d__Bacteria;k__norank;p__Proteobacteria;c__Gammaproteobacteria;o__Enterobacterales;f__Pectobacteriaceae;g__Dickeya;s__norank
MDJ-2_gene_1427	111	EIB99618.1	111	87/111(78.38)	1.36e-36	hypothetical protein S7A_13935 [Pantoea sp. Sc1]	593105	d__Bacteria;k__norank;p__Proteobacteria;c__Gammaproteobacteria;o__Enterobacterales;f__Erwiniaceae;g__Pantoea;s__Pantoea_sp._Sc1
MDJ-2_gene_1430	122	BBA84858.1	317	60/127(47.24)	1.66e-19	methionyl-tRNA formyltransferase [endosymbiont of Pachyrhynchus infernalis]	1971488	d__Bacteria;k__norank;p__Proteobacteria;c__Gammaproteobacteria;o__norank;f__norank;g__Candidatus_Nardonella;s__endosymbiont_of_Pachyrhynchus_infernalis
MDJ-2_gene_1431	149	CEN32005.1	147	88/148(59.46)	5.19e-49	Peptide deformylase [Candidatus Westeberhardia cardiocondylae]	1594731	d__Bacteria;k__norank;p__Proteobacteria;c__Gammaproteobacteria;o__Enterobacterales;f__Enterobacteriaceae;g__Candidatus_Westeberhardia;s__Candidatus_Westeberhardia_cardiocondylae
MDJ-2_gene_1432	226	BBA85236.1	275	113/225(50.22)	2.19e-56	shikimate dehydrogenase (NADP(+)) [endosymbiont of	1972134	d__Bacteria;k__norank;p__Proteobacteria;c__Gammaproteobacteria;o__norank;f__norank;g__Candidatus_Nardonella;s__endosymbiont_of_Sipalinus_gigas
MDJ-2_gene_1441	390	AGJ98699.1	390	385/390(98.72)	2.16e-278	Elongation factor Tu 1 [Wolbachia endosymbiont of Drosophila simulans wNo]	1236908	d__Bacteria;k__norank;p__Proteobacteria;c__Alphaproteobacteria;o__Rickettsiales;f__Anaplasmataceae;g__Wolbachia;s__Wolbachia_endosymbiont_of_Drosophila_simulans
MDJ-2_gene_1442	527	WP_019236839.1	527	518/527(98.29)	0	DNA polymerase III subunit gamma/tau [Wolbachia pipientis]	955	d__Bacteria;k__norank;p__Proteobacteria;c__Alphaproteobacteria;o__Rickettsiales;f__Anaplasmataceae;g__Wolbachia;s__Wolbachia_pipientis
MDJ-2_gene_1443	110	CAQ55046.1	110	109/110(99.09)	3.56e-68	conserved hypothetical protein [Wolbachia endosymbiont of Culex quinquefasciatus Pel]	570417	d__Bacteria;k__norank;p__Proteobacteria;c__Alphaproteobacteria;o__Rickettsiales;f__Anaplasmataceae;g__Wolbachia;s__Wolbachia_endosymbiont_of_Culex_quinquefasciatus
MDJ-2_gene_1444	445	WP_006317361.1	439	396/439(90.21)	5.08e-292	IS4 family transposase [Serratia plymuthica]	82996	d__Bacteria;k__norank;p__Proteobacteria;c__Gammaproteobacteria;o__Enterobacterales;f__Yersiniaceae;g__Serratia;s__Serratia_plymuthica
MDJ-2_gene_1445	111	WP_111139289.1	111	110/111(99.10)	7.43e-75	universal stress protein UspB [Pantoea sp. ARC607]	2027922	d__Bacteria;k__norank;p__Proteobacteria;c__Gammaproteobacteria;o__Enterobacterales;f__Erwiniaceae;g__Pantoea;s__Pantoea_sp._ARC607
MDJ-2_gene_1452	155	EEH12534.1	183	145/148(97.97)	9.91e-89	Major facilitator family transporter [Wolbachia endosymbiont of Muscidifurax	77037	d__Bacteria;k__norank;p__Proteobacteria;c__Alphaproteobacteria;o__Rickettsiales;f__Anaplasmataceae;g__Wolbachia;s__Wolbachia_endosymbiont_of_Muscidifurax_uniraptor
MDJ-2_gene_1453	435	WP_127463676.1	435	430/435(98.85)	8.74e-310	nucleotide sugar dehydrogenase [Wolbachia endosymbiont of Bemisia tabaci]	215173	d__Bacteria;k__norank;p__Proteobacteria;c__Alphaproteobacteria;o__Rickettsiales;f__Anaplasmataceae;g__Wolbachia;s__Wolbachia_endosymbiont_of_Bemisia_tabaci
MDJ-2_gene_1456	227	WP_114516975.1	227	197/227(86.78)	6.47e-139	hypothetical protein [Wolbachia endosymbiont of Cylisticus convexus]	118728	d__Bacteria;k__norank;p__Proteobacteria;c__Alphaproteobacteria;o__Rickettsiales;f__Anaplasmataceae;g__Wolbachia;s__Wolbachia_endosymbiont_of_Cylisticus_convexus

MDJ-2_gene_1467	287	AGJ98875.1	420	286/287(99.65)	3.81e-190	Pyruvate dehydrogenase complex, E2 component, dihydrolipoamide acetyltransferase [Wolbachia endosymbiont of Drosophila simulans wNo]	1236908	d__Bacteria;k__norank;p__Proteobacteria;c__Alphaproteobacteria;o__Rickettsiales;f__Anaplasmataceae;g__Wolbachia;s__Wolbachia_endosymbiont_of_Drosophila_simulans
MDJ-2_gene_1468	164	WP_108784089.1	164	161/164(98.17)	3.36e-101	hypothetical protein [Wolbachia endosymbiont of Bemisia tabaci]	215173	d__Bacteria;k__norank;p__Proteobacteria;c__Alphaproteobacteria;o__Rickettsiales;f__Anaplasmataceae;g__Wolbachia;s__Wolbachia_endosymbiont_of_Bemisia_tabaci
MDJ-2_gene_1470	469	WP_127463665.1	471	361/487(74.13)	5.03e-213	hypothetical protein [Wolbachia endosymbiont of Bemisia tabaci]	215173	d__Bacteria;k__norank;p__Proteobacteria;c__Alphaproteobacteria;o__Rickettsiales;f__Anaplasmataceae;g__Wolbachia;s__Wolbachia_endosymbiont_of_Bemisia_tabaci
MDJ-2_gene_1472	694	PBQ26533.1	2386	223/307(72.64)	8.72e-127	hypothetical protein BTO27_04595, partial	2049495	d__Bacteria;k__norank;p__Proteobacteria;c__Alphaproteobacteria;o__Rickettsiales;f__Anaplasmataceae;g__Wolbachia;s__Wolbachia_pipientis
MDJ-2_gene_1474	508	POG49803.1	508	463/508(91.14)	0	tRNA uridine-5-carboxymethylaminomethyl(34) synthesis GTPase MnmE [Wolbachia sp. wMel_AMD]	1912095	d__Bacteria;k__norank;p__Proteobacteria;c__Alphaproteobacteria;o__Rickettsiales;f__Anaplasmataceae;g__Wolbachia;s__Wolbachia_sp._wMel_AMD
MDJ-2_gene_1479	294	B3CNB0.1	297	294/294(100.00)	3.78e-213	RecName: Full=tRNA dimethylallyltransferase; AltName: Full=Dimethylallyl diphosphate:tRNA dimethylallyltransferase; Short=DMAPP:tRNA dimethylallyltransferase; Short=DMATase; AltName: Full=Isopentenyl-diphosphate:tRNA isopentenyltransferase; Short=IPP transferase; Short=IPPT;	570417	d__Bacteria;k__norank;p__Proteobacteria;c__Alphaproteobacteria;o__Rickettsiales;f__Anaplasmataceae;g__Wolbachia;s__Wolbachia_endosymbiont_of_Culex_quinquefasciatus
MDJ-2_gene_1489	288	BAO99810.1	295	250/289(86.51)	1.84e-170	putative transposase [Wolbachia endosymbiont of Cimex	246273	d__Bacteria;k__norank;p__Proteobacteria;c__Alphaproteobacteria;o__Rickettsiales;f__Anaplasmataceae;g__Wolbachia;s__Wolbachia_endosymbiont_of_Cimex_lectularius
MDJ-2_gene_1497	215	BBA84931.1	242	123/210(58.57)	4.97e-63	inner membrane protein YbhL [endosymbiont of Pachyrhynchus infernalis]	1971488	d__Bacteria;k__norank;p__Proteobacteria;c__Gammaproteobacteria;o__norank;f__norank;g__Candidatus_Nardonella;s__endosymbiont_of_Pachyrhynchus_infernalis
MDJ-2_gene_1498	48	WP_085112654.1	365	27/43(62.79)	2.89e-08	rod shape-determining protein RodA [Thermoanaerobacterium	1930849	d__Bacteria;k__norank;p__Firmicutes;c__Clostridia;o__Thermoanaerobacterales;f__Thermoanaerobacterales_Family_III._Incertae_Sedis;g__Thermoanaerobacterium;s__Thermoanaerobacterium_sp.
MDJ-2_gene_1502	50	AGJ99269.1	418	40/47(85.11)	7.63e-17	hypothetical protein wNo_08770 [Wolbachia endosymbiont of Drosophila simulans wNo]	1236908	d__Bacteria;k__norank;p__Proteobacteria;c__Alphaproteobacteria;o__Rickettsiales;f__Anaplasmataceae;g__Wolbachia;s__Wolbachia_endosymbiont_of_Drosophila_simulans
MDJ-2_gene_1504	83	WP_064085509.1	96	82/83(98.80)	3.58e-49	hypothetical protein [Wolbachia endosymbiont of Dactylopius coccus]	None	Unclassified
MDJ-2_gene_1507	99	PPI88051.1	554	62/98(63.27)	2.62e-30	glutamine--tRNA ligase [Pantoea sp. SoEE]	2044609	d__Bacteria;k__norank;p__Proteobacteria;c__Gammaproteobacteria;o__Enterobacterales;f__Erwiniaceae;g__Pantoea;s__Pantoea_sp._SoEE
MDJ-2_gene_1510	167	PBQ29118.1	167	166/167(99.40)	3.24e-114	protein-export chaperone SecB [Wolbachia pipientis wAus]	2049495	d__Bacteria;k__norank;p__Proteobacteria;c__Alphaproteobacteria;o__Rickettsiales;f__Anaplasmataceae;g__Wolbachia;s__Wolbachia_pipientis
MDJ-2_gene_1511	110	EAL60204.1	175	102/110(92.73)	8.61e-60	hypothetical protein WwSim0041 [Wolbachia endosymbiont of Drosophila simulans]	77038	d__Bacteria;k__norank;p__Proteobacteria;c__Alphaproteobacteria;o__Rickettsiales;f__Anaplasmataceae;g__Wolbachia;s__Wolbachia_endosymbiont_of_Drosophila_simulans
MDJ-2_gene_1513	146	BBA85287.1	148	97/146(66.44)	3.08e-59	3-dehydroquinate dehydratase [endosymbiont of Sipalinus gigas]	1972134	d__Bacteria;k__norank;p__Proteobacteria;c__Gammaproteobacteria;o__norank;f__norank;g__Candidatus_Nardonella;s__endosymbiont_of_Sipalinus_gigas

MDJ-2_gene_1516	339	BBA85345.1	346	195/340(57.35)	1.42e-119	tRNA N6-adenosine threonylcarbamoyltransferase [endosymbiont of Sipalinus gigas]	1972134	d__Bacteria;k__norank;p__Proteobacteria;c__Gammaproteobacteria;o__norank;f__norank;g__Candidatus_Nardonella;s__endosymbiont_of_Sipalinus_gigas
MDJ-2_gene_1517	74	BBA84909.1	56	37/55(67.27)	2.92e-16	30S ribosomal protein S21 [endosymbiont of Pachyrhynchus infernalis]	1971488	d__Bacteria;k__norank;p__Proteobacteria;c__Gammaproteobacteria;o__norank;f__norank;g__Candidatus_Nardonella;s__endosymbiont_of_Pachyrhynchus_infernalis
MDJ-2_gene_1539	473	KEL82257.1	1031	252/359(70.19)	2.32e-134	tape measure domain protein [Citrobacter freundii]	546	d__Bacteria;k__norank;p__Proteobacteria;c__Gammaproteobacteria;o__Enterobacterales;f__Enterobacteriaceae;g__Citrobacter;s__Citrobacter_freundii
MDJ-2_gene_1540	183	YP_008126234.1	235	95/165(57.58)	3.01e-59	hypothetical protein VPRG_00071 [Vibrio phage	754051	d__Viruses;k__norank;p__Uroviricota;c__Caudoviricetes;o__Caudovirales;f__Siphoviridae;g__Roufvirus;s__Vibrio_phage_pYD38-A
MDJ-2_gene_1546	126	CAQ54137.1	126	114/126(90.48)	2.25e-74	Putative transposase [Wolbachia endosymbiont of Culex quinquefasciatus Pel]	570417	d__Bacteria;k__norank;p__Proteobacteria;c__Alphaproteobacteria;o__Rickettsiales;f__Anaplasmataceae;g__Wolbachia;s__Wolbachia_endosymbiont_of_Culex_quinquefasciatus
MDJ-2_gene_1548	149	WP_127464320.1	151	112/152(73.68)	1.09e-58	hypothetical protein [Wolbachia endosymbiont of Bemisia tabaci]	None	Unclassified
MDJ-2_gene_1549	364	AGJ99465.1	425	325/359(90.53)	3.71e-186	Putative exonuclease family domain protein [Wolbachia endosymbiont of Drosophila	1236908	d__Bacteria;k__norank;p__Proteobacteria;c__Alphaproteobacteria;o__Rickettsiales;f__Anaplasmataceae;g__Wolbachia;s__Wolbachia_endosymbiont_of_Drosophila_simulans
MDJ-2_gene_1553	211	WP_041063190.1	421	103/210(49.05)	4.41e-51	UDP-N-acetylglucosamine 1-carboxyvinyltransferase [Candidatus Tachikawaea	1410383	d__Bacteria;k__norank;p__Proteobacteria;c__Gammaproteobacteria;o__Enterobacterales;f__Enterobacteriaceae;g__Candidatus_Tachikawaea;s__Candidatus_Tachikawaea_gelatinosa
MDJ-2_gene_1555	288	BBA85306.1	453	202/289(69.90)	2.66e-126	serine endoprotease [endosymbiont of Sipalinus gigas]	1972134	d__Bacteria;k__norank;p__Proteobacteria;c__Gammaproteobacteria;o__norank;f__norank;g__Candidatus_Nardonella;s__endosymbiont_of_Sipalinus_gigas
MDJ-2_gene_1556	242	PBQ26900.1	242	241/242(99.59)	9.17e-176	DNA repair protein RecO [Wolbachia pipientis wAus]	2049495	d__Bacteria;k__norank;p__Proteobacteria;c__Alphaproteobacteria;o__Rickettsiales;f__Anaplasmataceae;g__Wolbachia;s__Wolbachia_pipientis
MDJ-2_gene_1557	175	WP_010401890.1	175	175/175(100.00)	5.30e-117	hypothetical protein [Wolbachia endosymbiont of Nasonia vitripennis]	None	Unclassified
MDJ-2_gene_1558	97	WP_029237605.1	112	97/97(100.00)	4.14e-64	MULTISPECIES: outer membrane protein assembly factor BamE [Wolbachia]	180837	d__Bacteria;k__norank;p__Proteobacteria;c__Alphaproteobacteria;o__Rickettsiales;f__Anaplasmataceae;g__Wolbachia;s__Wolbachia_endosymbiont_of_Nasonia_vitripennis
MDJ-2_gene_1571	196	ORM55987.1	480	107/201(53.23)	1.38e-68	hypothetical protein HA41_00720 [Pantoea	472705	d__Bacteria;k__norank;p__Proteobacteria;c__Gammaproteobacteria;o__Enterobacterales;f__Erwiniaceae;g__Pantoea;s__Pantoea_conspicua
MDJ-2_gene_1572	354	WP_077056545.1	235	116/233(49.79)	7.22e-56	phage tail protein [Burkholderia cenocepacia]	95486	d__Bacteria;k__norank;p__Proteobacteria;c__Betaproteobacteria;o__Burkholderiales;f__Burkholderiaceae;g__Burkholderia;s__Burkholderia_cenocepacia
MDJ-2_gene_1573	52	AYR02252.1	828	30/51(58.82)	2.98e-12	hypothetical protein [Escherichia phage C1]	2340716	d__Viruses;k__norank;p__Uroviricota;c__Caudoviricetes;o__Caudovirales;f__Siphoviridae;g__norank;s__Escherichia_phage_C1
MDJ-2_gene_1580	264	WP_063630729.1	373	258/263(98.10)	6.59e-183	alpha/beta hydrolase [Wolbachia endosymbiont of Laodelphax striatella]	368602	d__Bacteria;k__norank;p__Proteobacteria;c__Alphaproteobacteria;o__Rickettsiales;f__Anaplasmataceae;g__Wolbachia;s__Wolbachia_endosymbiont_of_Laodelphax_striatellus
MDJ-2_gene_1581	84	WP_108784267.1	96	83/84(98.81)	4.03e-53	hypothetical protein [Wolbachia endosymbiont of Bemisia tabaci]	215173	d__Bacteria;k__norank;p__Proteobacteria;c__Alphaproteobacteria;o__Rickettsiales;f__Anaplasmataceae;g__Wolbachia;s__Wolbachia_endosymbiont_of_Bemisia_tabaci
MDJ-2_gene_1584	237	BBA85211.1	232	121/232(52.16)	1.41e-78	pseudouridine synthase [endosymbiont of Sipalinus gigas]	1972134	d__Bacteria;k__norank;p__Proteobacteria;c__Gammaproteobacteria;o__norank;f__norank;g__Candidatus_Nardonella;s__endosymbiont_of_Sipalinus_gigas
MDJ-2_gene_1585	179	BBA84875.1	190	97/174(55.75)	6.01e-48	shikimate kinase 1 [endosymbiont of Pachyrhynchus infernalis]	1971488	d__Bacteria;k__norank;p__Proteobacteria;c__Gammaproteobacteria;o__norank;f__norank;g__Candidatus_Nardonella;s__endosymbiont_of_Pachyrhynchus_infernalis
MDJ-2_gene_1586	121	EEG86269.1	145	54/116(46.55)	1.80e-24	3-dehydroquinate synthase [Proteus penneri ATCC 35198]	471881	d__Bacteria;k__norank;p__Proteobacteria;c__Gammaproteobacteria;o__Enterobacterales;f__Morganellaceae;g__Proteus;s__Proteus_penneri
MDJ-2_gene_1588	117	AFN42309.1	742	46/82(56.10)	3.26e-17	leucine-rich protein [Cotesia sesamiae Mombasa bracovirus]	452649	d__Viruses;k__norank;p__norank;c__norank;o__norank;f__Polydnaviridae;g__Bracovirus;s__Cotesia_sesamiae_bracovirus

MDJ-2_gene_1596	84	EEB55288.1	222	76/80(95.00)	1.16e-50	Phage terminase large subunit GpA [Wolbachia endosymbiont of Culex quinquefasciatus JHB]	569881	d__Bacteria;k__norank;p__Proteobacteria;c__Alphaproteobacteria;o__Rickettsiales;f__Anaplasmataceae;g__Wolbachia;s__Wolbachia_endosymbiont_of_Culex_quinquefasciatus
MDJ-2_gene_1606	389	WP_010403274.1	389	382/389(98.20)	1.47e-280	aspartate aminotransferase family protein [Wolbachia endosymbiont of Nasonia vitripennis]	2640676	d__Bacteria;k__norank;p__Proteobacteria;c__Alphaproteobacteria;o__Rickettsiales;f__Anaplasmataceae;g__Wolbachia;s__norank
MDJ-2_gene_1608	104	BBA85204.1	566	72/104(69.23)	4.35e-39	aspartyl-tRNA synthetase [endosymbiont of Sipalinus gigas]	1972134	d__Bacteria;k__norank;p__Proteobacteria;c__Gammaproteobacteria;o__norank;f__norank;g__Candidatus_Nardonella;s__endosymbiont_of_Sipalinus_gigas
MDJ-2_gene_1632	261	AGJ98964.1	261	259/261(99.23)	4.37e-188	Type IV secretion system protein VirB9, putative [Wolbachia endosymbiont of Drosophila simulans wNo]	1236908	d__Bacteria;k__norank;p__Proteobacteria;c__Alphaproteobacteria;o__Rickettsiales;f__Anaplasmataceae;g__Wolbachia;s__Wolbachia_endosymbiont_of_Drosophila_simulans
MDJ-2_gene_1633	355	BBA85162.1	485	174/313(55.59)	8.69e-98	lysine-tRNA ligase [endosymbiont of Sipalinus gigas]	1972134	d__Bacteria;k__norank;p__Proteobacteria;c__Gammaproteobacteria;o__norank;f__norank;g__Candidatus_Nardonella;s__endosymbiont_of_Sipalinus_gigas
MDJ-2_gene_1635	83	ARC54719.1	503	55/82(67.07)	8.04e-26	lysine--tRNA ligase [Candidatus Riesia sp. GBBU]	1719125	d__Bacteria;k__norank;p__Proteobacteria;c__Gammaproteobacteria;o__Enterobacterales;f__Enterobacteriaceae;g__Candidatus_Riesia;s__Candidatus_Riesia_sp._GBBU
MDJ-2_gene_1645	70	BBA84826.1	1328	54/70(77.14)	2.16e-26	DNA-directed RNA polymerase subunit beta [endosymbiont of Pachyrhynchus infernalis]	1971488	d__Bacteria;k__norank;p__Proteobacteria;c__Gammaproteobacteria;o__norank;f__norank;g__Candidatus_Nardonella;s__endosymbiont_of_Pachyrhynchus_infernalis
MDJ-2_gene_1646	497	ARC54912.1	1341	320/497(64.39)	1.24e-206	DNA-directed RNA polymerase subunit beta [Candidatus Riesia sp. GBBU]	1719125	d__Bacteria;k__norank;p__Proteobacteria;c__Gammaproteobacteria;o__Enterobacterales;f__Enterobacteriaceae;g__Candidatus_Riesia;s__Candidatus_Riesia_sp._GBBU
MDJ-2_gene_1652	100	RUM94951.1	904	28/83(33.73)	9.50e-08	hypothetical protein DSZ28_00550 [Thiothrix sp.]	1032	d__Bacteria;k__norank;p__Proteobacteria;c__Gammaproteobacteria;o__Thiotrichales;f__Thiotrichaceae;g__Thiothrix;s__Thiothrix_sp.
MDJ-2_gene_1654	84	WP_106481696.1	133	32/71(45.07)	3.52e-08	MULTISPECIES: fibronectin type III domain-containing protein [Butyricimonas]	574697	d__Bacteria;k__norank;p__Bacteroidetes;c__Bacteroidia;o__Bacteroidales;f__Odoribacteraceae;g__Butyricimonas;s__norank
MDJ-2_gene_1674	77	WP_045171667.1	225	27/67(40.30)	2.75e-06	hypothetical protein [Ehrlichia minasensis]	None	Unclassified
MDJ-2_gene_1687	422	BBA84814.1	746	210/402(52.24)	3.33e-107	penicillin-binding protein 1B [endosymbiont of Pachyrhynchus infernalis]	1971488	d__Bacteria;k__norank;p__Proteobacteria;c__Gammaproteobacteria;o__norank;f__norank;g__Candidatus_Nardonella;s__endosymbiont_of_Pachyrhynchus_infernalis
MDJ-2_gene_1693	187	WP_111140878.1	187	182/187(97.33)	4.46e-124	ADP compounds hydrolase NudE [Pantoea sp. ARC607]	2027922	d__Bacteria;k__norank;p__Proteobacteria;c__Gammaproteobacteria;o__Enterobacterales;f__Erwiniaceae;g__Pantoea;s__Pantoea_sp._ARC607
MDJ-2_gene_1701	133	ORX91783.1	138	33/62(53.23)	9.70e-15	hypothetical protein K493DRAFT_227257 [Basidiobolus meristosporus CBS	1314790	d__Eukaryota;k__Fungi;p__Zoopagomycota;c__Basidiobolomycetes;o__Basidiobolales;f__Basidiobolaceae;g__Basidiobolus;s__Basidiobolus_meristosporus
MDJ-2_gene_1704	290	WP_010407101.1	290	288/290(99.31)	1.31e-183	MULTISPECIES: protease modulator HflC [Wolbachia]	2640676	d__Bacteria;k__norank;p__Proteobacteria;c__Alphaproteobacteria;o__Rickettsiales;f__Anaplasmataceae;g__Wolbachia;s__norank
MDJ-2_gene_1724	121	BBA85329.1	179	70/121(57.85)	1.20e-44	inorganic pyrophosphatase [endosymbiont of Sipalinus gigas]	1972134	d__Bacteria;k__norank;p__Proteobacteria;c__Gammaproteobacteria;o__norank;f__norank;g__Candidatus_Nardonella;s__endosymbiont_of_Sipalinus_gigas
MDJ-2_gene_1725	46	WP_092009657.1	180	31/46(67.39)	2.52e-12	oligoribonuclease [Marinobacter daqiaonensis]	650891	d__Bacteria;k__norank;p__Proteobacteria;c__Gammaproteobacteria;o__Alteromonadales;f__Alteromonadaceae;g__Marinobacter;s__Marinobacter_daqiaonensis
MDJ-2_gene_1735	190	ORM55987.1	480	115/185(62.16)	3.42e-67	hypothetical protein HA41_00720 [Pantoea	472705	d__Bacteria;k__norank;p__Proteobacteria;c__Gammaproteobacteria;o__Enterobacterales;f__Erwiniaceae;g__Pantoea;s__Pantoea_conspicua
MDJ-2_gene_1752	152	WP_064085995.1	226	149/152(98.03)	3.73e-95	orotidine-5'-phosphate decarboxylase [Wolbachia endosymbiont of Dactylopius coccus]	None	Unclassified

MDJ-2_gene_1760	56	BBA85144.1	363	38/56(67.86)	9.61e-15	3-dehydroquinate synthase [endosymbiont of Rhynchophorus ferrugineus]	1972133	d__Bacteria;k__norank;p__Proteobacteria;c__Gammaproteobacteria;o__norank;f__norank;g__Candidatus_Nardonella;s__Candidatus_Nardonella_dryophthoridicola
MDJ-2_gene_1761	94	BBA84874.1	363	39/74(52.70)	8.31e-14	3-dehydroquinate synthase [endosymbiont of Pachyrhynchus infernalis]	1971488	d__Bacteria;k__norank;p__Proteobacteria;c__Gammaproteobacteria;o__norank;f__norank;g__Candidatus_Nardonella;s__endosymbiont_of_Pachyrhynchus_infernalis
MDJ-2_gene_1762	194	BBA84873.1	178	89/180(49.44)	9.48e-38	peptidyl-tRNA hydrolase [endosymbiont of Pachyrhynchus infernalis]	1971488	d__Bacteria;k__norank;p__Proteobacteria;c__Gammaproteobacteria;o__norank;f__norank;g__Candidatus_Nardonella;s__endosymbiont_of_Pachyrhynchus_infernalis
MDJ-2_gene_1765	520	RYA71946.1	283	52/132(39.39)	4.64e-22	hypothetical protein DD592_26380, partial [Enterobacter cloacae complex	2511993	d__Bacteria;k__norank;p__Proteobacteria;c__Gammaproteobacteria;o__Enterobacterales;f__Enterobacteriaceae;g__Enterobacter;s__Enterobacter_cloacae_complex_sp._2DZ2F20B
MDJ-2_gene_1769	130	VFP88475.1	130	75/130(57.69)	1.95e-40	30S ribosomal protein S9 [Buchnera aphidicola (Cinara	1660043	d__Bacteria;k__norank;p__Proteobacteria;c__Gammaproteobacteria;o__Enterobacterales;f__Erwiniaceae;g__Buchnera;s__Buchnera_aphidicola
MDJ-2_gene_1774	358	XP_025168656.1	1051	93/334(27.84)	5.04e-28	putative peptidase M16 [Rhizophagus irregularis DAOM 181602=DAOM 197198]	747089	d__Eukaryota;k__Fungi;p__Mucoromycota;c__Glomeromycetes;o__Glomerales;f__Glomeraceae;g__Rhizophagus;s__Rhizophagus_irregularis
MDJ-2_gene_1786	57	GBU08355.1	170	22/55(40.00)	5.06e-07	flavodoxin 2 [Bacteroidales bacterium]	2030927	d__Bacteria;k__norank;p__Bacteroidetes;c__Bacteroidia;o__Bacteroidales;f__norank;g__norank;s__Bacteroidales_bacterium
MDJ-2_gene_1799	176	PBQ25932.1	176	174/176(98.86)	1.78e-120	phage head-tail connector protein [Wolbachia pipientis wAus]	2049495	d__Bacteria;k__norank;p__Proteobacteria;c__Alphaproteobacteria;o__Rickettsiales;f__Anaplasmataceae;g__Wolbachia;s__Wolbachia_pipientis
MDJ-2_gene_1800	92	WP_019236305.1	92	89/92(96.74)	3.37e-51	hypothetical protein [Wolbachia pipientis]	953	d__Bacteria;k__norank;p__Proteobacteria;c__Alphaproteobacteria;o__Rickettsiales;f__Anaplasmataceae;g__Wolbachia;s__norank
MDJ-2_gene_1803	327	BBA85373.1	600	244/325(75.08)	5.13e-174	ATP-dependent zinc metalloprotease FtsH [endosymbiont of Sipalinus gigas]	1972134	d__Bacteria;k__norank;p__Proteobacteria;c__Gammaproteobacteria;o__norank;f__norank;g__Candidatus_Nardonella;s__endosymbiont_of_Sipalinus_gigas
MDJ-2_gene_1804	83	BAC24377.1	638	45/78(57.69)	2.51e-18	hflB [Wigglesworthia glossinidia endosymbiont of Glossina brevipalpis]	36870	d__Bacteria;k__norank;p__Proteobacteria;c__Gammaproteobacteria;o__Enterobacterales;f__Erwiniaceae;g__Wigglesworthia;s__Wigglesworthia_glossinidia
MDJ-2_gene_1805	122	WP_115956184.1	426	52/126(41.27)	2.45e-15	tyrosine--tRNA ligase [Candidatus Purcelliella	472834	d__Bacteria;k__norank;p__Proteobacteria;c__Gammaproteobacteria;o__Enterobacterales;f__Enterobacteriaceae;g__Candidatus_Purcelliella;s__Candidatus_Purcelliella_pentastirinorum
MDJ-2_gene_1806	129	WP_115956184.1	426	61/127(48.03)	1.36e-26	tyrosine--tRNA ligase [Candidatus Purcelliella	472834	d__Bacteria;k__norank;p__Proteobacteria;c__Gammaproteobacteria;o__Enterobacterales;f__Enterobacteriaceae;g__Candidatus_Purcelliella;s__Candidatus_Purcelliella_pentastirinorum
MDJ-2_gene_1808	105	PCG11783.1	105	105/105(100.00)	8.28e-67	cell division protein ZapA [Wolbachia pipientis]	None	Unclassified
MDJ-2_gene_1809	92	WP_010404234.1	92	92/92(100.00)	7.34e-55	MULTISPECIES: integration host factor subunit beta	180837	d__Bacteria;k__norank;p__Proteobacteria;c__Alphaproteobacteria;o__Rickettsiales;f__Anaplasmataceae;g__Wolbachia;s__Wolbachia_endosymbiont_of_Nasonia_vitripennis
MDJ-2_gene_1816	270	KJM47320.1	292	262/270(97.04)	7.48e-192	transposase [[Enterobacter] aerogenes]	548	d__Bacteria;k__norank;p__Proteobacteria;c__Gammaproteobacteria;o__Enterobacterales;f__Enterobacteriaceae;g__Klebsiella;s__Klebsiella_aerogenes
MDJ-2_gene_1817	132	VDY63472.1	131	128/131(97.71)	2.40e-83	IS2 repressor TnpA [Shimwellia blattae]	563	d__Bacteria;k__norank;p__Proteobacteria;c__Gammaproteobacteria;o__Enterobacterales;f__Enterobacteriaceae;g__Shimwellia;s__Shimwellia_blattae
MDJ-2_gene_1829	95	WP_096616756.1	95	94/95(98.95)	2.14e-62	hypothetical protein [Wolbachia pipientis]	955	d__Bacteria;k__norank;p__Proteobacteria;c__Alphaproteobacteria;o__Rickettsiales;f__Anaplasmataceae;g__Wolbachia;s__Wolbachia_pipientis
MDJ-2_gene_1833	313	AGJ98588.1	313	297/313(94.89)	2.01e-210	Surface antigen-related protein [Wolbachia endosymbiont of Drosophila simulans wNo]	1236908	d__Bacteria;k__norank;p__Proteobacteria;c__Alphaproteobacteria;o__Rickettsiales;f__Anaplasmataceae;g__Wolbachia;s__Wolbachia_endosymbiont_of_Drosophila_simulans
MDJ-2_gene_1835	301	EGY29296.1	529	109/280(38.93)	9.29e-63	Pao retrotransposon peptidase protein, partial [Candidatus Regiella insecticola 5.15]	1005043	d__Bacteria;k__norank;p__Proteobacteria;c__Gammaproteobacteria;o__Enterobacterales;f__Enterobacteriaceae;g__Candidatus_Regiella;s__Candidatus_Regiella_insecticola

MDJ-2_gene_1840	373	ORM75967.1	380	370/373(99.20)	4.68e-282	pyrroloquinoline quinone biosynthesis protein PqqE [Pantoea deleyi]	470932	d__Bacteria;k__norank;p__Proteobacteria;c__Gammaproteobacteria;o__Enterobacterales;f__Erwiniaceae;g__Pantoea;s__Pantoea_deleyi
MDJ-2_gene_1846	339	ORX56388.1	276	89/228(39.04)	5.95e-45	hypothetical protein DM01DRAFT_266689 [Hesseltinella vesiculosa]	101127	d__Eukaryota;k__Fungi;p__Mucoromycota;c__Mucoromycetes;o__Mucorales;f__Cunninghamellaceae;g__Hesseltinella;s__Hesseltinella_vesiculosa
MDJ-2_gene_1853	69	ORY57091.1	1763	50/59(84.75)	1.26e-27	DNA-directed RNA polymerase [Leucosporidium creatinivorum]	106004	d__Eukaryota;k__Fungi;p__Basidiomycota;c__Microbotryomycetes;o__Leucosporidiales;f__Leucosporidiaceae;g__Leucosporidium;s__Leucosporidium_creatinivorum
MDJ-2_gene_1857	409	YP_009179248.1	414	80/273(29.30)	9.77e-19	UDP-GlcNAcbeta GAL beta1,3 N-acetyl glucosaminyl transferase 3 [Elephant endotheliotropic herpesvirus 4]	548914	d__Viruses;k__norank;p__Peploviricota;c__Herviviricetes;o__Herpesvirales;f__Herpesviridae;g__Proboscivirus;s__Elephant_endotheliotropic_herpesvirus_4
MDJ-2_gene_1862	326	PCG11757.1	1023	302/325(92.92)	1.25e-209	glutamate synthase subunit beta [Wolbachia pipientis]	None	Unclassified
MDJ-2_gene_1863	300	BBA84929.1	594	154/308(50.00)	6.97e-87	penicillin-binding protein 2 [endosymbiont of Pachyrhynchus infernalis]	1971488	d__Bacteria;k__norank;p__Proteobacteria;c__Gammaproteobacteria;o__norank;f__norank;g__Candidatus_Nardonella;s__endosymbiont_of_Pachyrhynchus_infernalis
MDJ-2_gene_1866	54	ORX82756.1	876	26/52(50.00)	3.85e-08	hypothetical protein K493DRAFT_91114 [Basidiobolus meristosporus CBS	1314790	d__Eukaryota;k__Fungi;p__Zoopagomycota;c__Basidiobolomycetes;o__Basidiobolales;f__Basidiobolaceae;g__Basidiobolus;s__Basidiobolus_meristosporus
MDJ-2_gene_1867	77	BAP00050.1	126	77/77(100.00)	1.43e-48	nitrogen fixation protein NifU and related proteins [Wolbachia endosymbiont of Cimex	246273	d__Bacteria;k__norank;p__Proteobacteria;c__Alphaproteobacteria;o__Rickettsiales;f__Anaplasmataceae;g__Wolbachia;s__Wolbachia_endosymbiont_of_Cimex_lectularius
MDJ-2_gene_1868	303	WP_019236959.1	303	299/303(98.68)	2.01e-205	signal recognition particle-docking protein FtsY [Wolbachia	953	d__Bacteria;k__norank;p__Proteobacteria;c__Alphaproteobacteria;o__Rickettsiales;f__Anaplasmataceae;g__Wolbachia;s__norank
MDJ-2_gene_1873	457	RYE26614.1	1339	162/464(34.91)	2.71e-65	hypothetical protein EOP45_03685	2021370	d__Bacteria;k__norank;p__Bacteroidetes;c__Sphingobacteriia;o__Sphingobacteriales;f__Sphingobacteriaceae;g__norank;s__Sphingobacteriaceae_bacterium
MDJ-2_gene_1874	72	AVQ87988.1	97	60/72(83.33)	1.77e-35	co-chaperone GroES [Plesiomonas shigelloides]	703	d__Bacteria;k__norank;p__Proteobacteria;c__Gammaproteobacteria;o__Enterobacterales;f__norank;g__Plesiomonas;s__Plesiomonas_shigelloides
MDJ-2_gene_1885	487	AVJ51765.1	885	414/486(85.19)	0	structural protein containing EPS-depolymerase domain [Pantoea phage vB_PagS_Vid5]	2099652	d__Viruses;k__norank;p__Uroviricota;c__Caudoviricetes;o__Caudovirales;f__Siphoviridae;g__Vidquintavirus;s__Pantoea_virus_Vid5
MDJ-2_gene_1892	121	WP_053940029.1	122	67/119(56.30)	1.07e-33	50S ribosomal protein L7/L12 [Buchnera aphidicola]	9	d__Bacteria;k__norank;p__Proteobacteria;c__Gammaproteobacteria;o__Enterobacterales;f__Erwiniaceae;g__Buchnera;s__Buchnera_aphidicola
MDJ-2_gene_1893	184	AAA24579.1	188	103/183(56.28)	1.25e-60	RNA-polymerase beta subunit, partial [Escherichia coli]	562	d__Bacteria;k__norank;p__Proteobacteria;c__Gammaproteobacteria;o__Enterobacterales;f__Enterobacteriaceae;g__Escherichia;s__Escherichia_coli
MDJ-2_gene_1916	116	PBQ29271.1	116	116/116(100.00)	1.79e-73	ribosomal-binding protein A [Wolbachia pipientis wAus]	2049495	d__Bacteria;k__norank;p__Proteobacteria;c__Alphaproteobacteria;o__Rickettsiales;f__Anaplasmataceae;g__Wolbachia;s__Wolbachia_pipientis
MDJ-2_gene_1918	65	ORM76622.1	105	55/65(84.62)	2.08e-29	hypothetical protein HA42_18890 [Pantoea deleyi]	470932	d__Bacteria;k__norank;p__Proteobacteria;c__Gammaproteobacteria;o__Enterobacterales;f__Erwiniaceae;g__Pantoea;s__Pantoea_deleyi
MDJ-2_gene_1920	55	WP_114517957.1	121	48/51(94.12)	7.19e-28	transposase [Wolbachia endosymbiont of Cylisticus convexus]	118728	d__Bacteria;k__norank;p__Proteobacteria;c__Alphaproteobacteria;o__Rickettsiales;f__Anaplasmataceae;g__Wolbachia;s__Wolbachia_endosymbiont_of_Cylisticus_convexus
MDJ-2_gene_1922	323	WP_065684115.1	369	122/327(37.31)	2.65e-67	porin OmpC [Serratia sp. 14-2641]	1841657	d__Bacteria;k__norank;p__Proteobacteria;c__Gammaproteobacteria;o__Enterobacterales;f__Yersiniaceae;g__Serratia;s__Serratia_sp._14-2641
MDJ-2_gene_1923	399	BBA84961.1	473	200/411(48.66)	3.49e-98	tRNA modification GTPase MnmE [endosymbiont of Pachyrhynchus infernalis]	1971488	d__Bacteria;k__norank;p__Proteobacteria;c__Gammaproteobacteria;o__norank;f__norank;g__Candidatus_Nardonella;s__endosymbiont_of_Pachyrhynchus_infernalis
MDJ-2_gene_1930	172	WP_127463623.1	170	151/167(90.42)	4.31e-96	phage tail protein [Wolbachia endosymbiont of Bemisia tabaci]	215173	d__Bacteria;k__norank;p__Proteobacteria;c__Alphaproteobacteria;o__Rickettsiales;f__Anaplasmataceae;g__Wolbachia;s__Wolbachia_endosymbiont_of_Bemisia_tabaci

MDJ-2_gene_1932	130	EEB55297.1	201	127/130(97.69)	3.41e-91	putative phage related protein [Wolbachia endosymbiont of Culex quinquefasciatus JHB]	569881	d__Bacteria;k__norank;p__Proteobacteria;c__Alphaproteobacteria;o__Rickettsiales;f__Anaplasmataceae;g__Wolbachia;s__Wolbachia_endosymbiont_of_Culex_quinquefasciatus
MDJ-2_gene_1934	133	BAC24585.1	580	57/133(42.86)	3.26e-23	recJ [Wigglesworthia glossinidia endosymbiont of Glossina brevipalpis]	36870	d__Bacteria;k__norank;p__Proteobacteria;c__Gammaproteobacteria;o__Enterobacterales;f__Erwiniaceae;g__Wigglesworthia;s__Wigglesworthia_glossinidia
MDJ-2_gene_1935	110	BBA84756.1	580	55/107(51.40)	1.25e-22	single-stranded DNA-specific exonuclease [endosymbiont of Pachyrhynchus infernalis]	1971488	d__Bacteria;k__norank;p__Proteobacteria;c__Gammaproteobacteria;o__norank;f__norank;g__Candidatus_Nardonella;s__endosymbiont_of_Pachyrhynchus_infernalis
MDJ-2_gene_1944	236	WP_114517495.1	267	229/236(97.03)	6.43e-169	ATP-binding protein [Wolbachia endosymbiont of Cylisticus convexus]	118728	d__Bacteria;k__norank;p__Proteobacteria;c__Alphaproteobacteria;o__Rickettsiales;f__Anaplasmataceae;g__Wolbachia;s__Wolbachia_endosymbiont_of_Cylisticus_convexus
MDJ-2_gene_1949	54	WP_118714888.1	132	21/40(52.50)	8.56e-08	fibronectin type III domain-containing protein [Odoribacter sp. AF15-53]	2292236	d__Bacteria;k__norank;p__Bacteroidetes;c__Bacteroidia;o__Bacteroidales;f__Odoribacteraceae;g__Odoribacter;s__Odoribacter_sp._AF15-53
MDJ-2_gene_1953	271	EEB55406.1	268	216/273(79.12)	1.23e-148	putative phage related protein [Wolbachia endosymbiont of Culex quinquefasciatus JHB]	569881	d__Bacteria;k__norank;p__Proteobacteria;c__Alphaproteobacteria;o__Rickettsiales;f__Anaplasmataceae;g__Wolbachia;s__Wolbachia_endosymbiont_of_Culex_quinquefasciatus
MDJ-2_gene_1954	99	WP_114517449.1	163	82/84(97.62)	2.92e-50	hypothetical protein [Wolbachia endosymbiont of Cylisticus convexus]	118728	d__Bacteria;k__norank;p__Proteobacteria;c__Alphaproteobacteria;o__Rickettsiales;f__Anaplasmataceae;g__Wolbachia;s__Wolbachia_endosymbiont_of_Cylisticus_convexus
MDJ-2_gene_1958	71	ORY93052.1	345	27/75(36.00)	1.71e-06	hypothetical protein BCR43DRAFT_444305 [Syncephalastrum racemosum]	13706	d__Eukaryota;k__Fungi;p__Mucoromycota;c__Mucoromycetes;o__Mucorales;f__Syncephalastraceae;g__Syncephalastrum;s__Syncephalastrum_racemosum
MDJ-2_gene_1961	93	BBA84650.1	366	44/90(48.89)	8.96e-11	t-protein [endosymbiont of Euscepes postfasciatus]	650377	d__Bacteria;k__norank;p__Proteobacteria;c__Gammaproteobacteria;o__norank;f__norank;g__norank;s__endosymbiont_of_Euscepes_postfasciatus
MDJ-2_gene_1962	210	BBA84649.1	348	125/208(60.10)	4.46e-68	phospho-2-dehydro-3-deoxyheptonate aldolase [endosymbiont of Euscepes postfasciatus]	650377	d__Bacteria;k__norank;p__Proteobacteria;c__Gammaproteobacteria;o__norank;f__norank;g__norank;s__endosymbiont_of_Euscepes_postfasciatus
MDJ-2_gene_1963	104	CEN32277.1	348	54/88(61.36)	1.34e-27	Phospho-2-dehydro-3-deoxyheptonate aldolase, Tyr-sensitive [Candidatus Westeberhardia cardiocondylae]	1594731	d__Bacteria;k__norank;p__Proteobacteria;c__Gammaproteobacteria;o__Enterobacterales;f__Enterobacteriaceae;g__Candidatus_Westeberhardia;s__Candidatus_Westeberhardia_cardiocondylae
MDJ-2_gene_1972	212	ARC53187.1	441	107/207(51.69)	9.93e-55	hypothetical protein AOQ87_00520 [Candidatus	428411	d__Bacteria;k__norank;p__Proteobacteria;c__Gammaproteobacteria;o__Enterobacterales;f__Enterobacteriaceae;g__Candidatus_Riesia;s__Candidatus_Riesia_pediculischaeffi
MDJ-2_gene_1973	179	P59436.1	340	100/171(58.48)	1.38e-55	RecName: Full=Phospho-N-acetylmuramoyl-pentapeptide-transferase; AltName: Full=UDP-MurNAc-pentapeptide phosphotransferase	224915	d__Bacteria;k__norank;p__Proteobacteria;c__Gammaproteobacteria;o__Enterobacterales;f__Erwiniaceae;g__Buchnera;s__Buchnera_aphidicola
MDJ-2_gene_1990	339	WP_010403618.1	339	337/339(99.41)	1.17e-238	iron-sulfur cluster carrier protein ApbC [Wolbachia endosymbiont of Nasonia vitripennis]	180837	d__Bacteria;k__norank;p__Proteobacteria;c__Alphaproteobacteria;o__Rickettsiales;f__Anaplasmataceae;g__Wolbachia;s__Wolbachia_endosymbiont_of_Nasonia_vitripennis
MDJ-2_gene_1991	206	CAQ54811.1	206	196/206(95.15)	2.75e-141	3'-5' exonuclease [Wolbachia endosymbiont of Culex quinquefasciatus Pel]	570417	d__Bacteria;k__norank;p__Proteobacteria;c__Alphaproteobacteria;o__Rickettsiales;f__Anaplasmataceae;g__Wolbachia;s__Wolbachia_endosymbiont_of_Culex_quinquefasciatus
MDJ-2_gene_1993	216	WP_064085658.1	342	174/216(80.56)	2.15e-48	hypothetical protein [Wolbachia endosymbiont of Dactylopius coccus]	None	Unclassified

MDJ-2_gene_1994	113	ONI56112.1	115	87/115(75.65)	5.90e-53	transposase family protein [Wolbachia pipientis wUni]	1379787	d__Bacteria;k__norank;p__Proteobacteria;c__Alphaproteobacteria;o__Rickettsiales;f__Anaplasmataceae;g__Wolbachia;s__Wolbachia_pipientis
MDJ-2_gene_2003	104	AZU37918.1	104	102/104(98.08)	6.86e-61	50S ribosomal protein L28 [Wolbachia endosymbiont of Bemisia tabaci]	215173	d__Bacteria;k__norank;p__Proteobacteria;c__Alphaproteobacteria;o__Rickettsiales;f__Anaplasmataceae;g__Wolbachia;s__Wolbachia_endosymbiont_of_Bemisia_tabaci
MDJ-2_gene_2026	204	WP_081600833.1	293	195/198(98.48)	6.26e-137	alpha/beta hydrolase [Wolbachia endosymbiont of Drosophila simulans]	None	Unclassified
MDJ-2_gene_2029	358	WP_019078754.1	833	357/358(99.72)	1.63e-228	preprotein translocase subunit SecA, partial [Wolbachia endosymbiont of Culex pipiens	263438	d__Bacteria;k__norank;p__Proteobacteria;c__Alphaproteobacteria;o__Rickettsiales;f__Anaplasmataceae;g__Wolbachia;s__Wolbachia_endosymbiont_of_Culex_pipiens_molestus
MDJ-2_gene_2036	195	BBA85328.1	335	102/193(52.85)	4.20e-46	tryptophan--tRNA ligase [endosymbiont of Sipalinus gigas]	1972134	d__Bacteria;k__norank;p__Proteobacteria;c__Gammaproteobacteria;o__norank;f__norank;g__Candidatus_Nardonella;s__endosymbiont_of_Sipalinus_gigas
MDJ-2_gene_2037	50	BBA85032.1	57	33/51(64.71)	8.53e-12	50S ribosomal protein L33 [endosymbiont of Rhynchophorus ferrugineus]	1972133	d__Bacteria;k__norank;p__Proteobacteria;c__Gammaproteobacteria;o__norank;f__norank;g__Candidatus_Nardonella;s__Candidatus_Nardonella_dryophthoridicola
MDJ-2_gene_2040	105	APX64391.1	105	105/105(100.00)	4.98e-68	transposase protein (plasmid) [Acinetobacter schindleri]	108981	d__Bacteria;k__norank;p__Proteobacteria;c__Gammaproteobacteria;o__Pseudomonadales;f__Moraxellaceae;g__Acinetobacter;s__Acinetobacter_schindleri
MDJ-2_gene_2041	235	OOS32651.1	238	235/235(100.00)	1.11e-171	transposase [Acinetobacter baumannii]	470	d__Bacteria;k__norank;p__Proteobacteria;c__Gammaproteobacteria;o__Pseudomonadales;f__Moraxellaceae;g__Acinetobacter;s__Acinetobacter_baumannii
MDJ-2_gene_2044	395	WP_041581324.1	395	389/395(98.48)	1.52e-276	cysteine desulfurase [Wolbachia endosymbiont of Drosophila simulans]	77038	d__Bacteria;k__norank;p__Proteobacteria;c__Alphaproteobacteria;o__Rickettsiales;f__Anaplasmataceae;g__Wolbachia;s__Wolbachia_endosymbiont_of_Drosophila_simulans
MDJ-2_gene_2054	368	ORM72638.1	367	336/368(91.30)	3.89e-240	nickel/cobalt efflux protein RcnA [Pantoea deleyi]	470932	d__Bacteria;k__norank;p__Proteobacteria;c__Gammaproteobacteria;o__Enterobacterales;f__Erwiniaceae;g__Pantoea;s__Pantoea_deleyi
MDJ-2_gene_2055	45	WP_128087038.1	59	44/45(97.78)	2.35e-28	DUF1289 domain-containing protein [Pantoea deleyi]	470932	d__Bacteria;k__norank;p__Proteobacteria;c__Gammaproteobacteria;o__Enterobacterales;f__Erwiniaceae;g__Pantoea;s__Pantoea_deleyi
MDJ-2_gene_2056	160	WP_040754944.1	160	153/160(95.62)	1.68e-99	hypothetical protein [Wolbachia endosymbiont of Diaphorina citri]	None	Unclassified
MDJ-2_gene_2057	201	WP_063631133.1	201	197/201(98.01)	2.10e-138	tRNA (adenosine(37)-N6)-threonylcarbamoyltransferase complex dimerization subunit type 1 TsaB [Wolbachia endosymbiont of Laodelphax	368602	d__Bacteria;k__norank;p__Proteobacteria;c__Alphaproteobacteria;o__Rickettsiales;f__Anaplasmataceae;g__Wolbachia;s__Wolbachia_endosymbiont_of_Laodelphax_striatellus
MDJ-2_gene_2074	214	VFP81166.1	230	114/213(53.52)	8.77e-66	tRNA (guanine-N(1)-)-methyltransferase [Buchnera aphidicola (Cinara kochiana kochiana)]	2518976	d__Bacteria;k__norank;p__Proteobacteria;c__Gammaproteobacteria;o__Enterobacterales;f__Erwiniaceae;g__Buchnera;s__Buchnera_aphidicola
MDJ-2_gene_2077	158	WP_063630979.1	158	116/157(73.89)	1.58e-71	hypothetical protein [Wolbachia endosymbiont of Laodelphax striatella]	368602	d__Bacteria;k__norank;p__Proteobacteria;c__Alphaproteobacteria;o__Rickettsiales;f__Anaplasmataceae;g__Wolbachia;s__Wolbachia_endosymbiont_of_Laodelphax_striatellus
MDJ-2_gene_2078	131	AGK00238.1	122	81/119(68.07)	1.02e-43	hypothetical protein wHa_08150 [Wolbachia endosymbiont of Drosophila simulans wHa]	1236909	d__Bacteria;k__norank;p__Proteobacteria;c__Alphaproteobacteria;o__Rickettsiales;f__Anaplasmataceae;g__Wolbachia;s__Wolbachia_endosymbiont_of_Drosophila_simulans
MDJ-2_gene_2081	167	VFP79247.1	745	72/136(52.94)	2.40e-35	Translation initiation factor IF-2, isoform beta [Buchnera aphidicola (Cinara curtihirsuta)]	2518974	d__Bacteria;k__norank;p__Proteobacteria;c__Gammaproteobacteria;o__Enterobacterales;f__Erwiniaceae;g__Buchnera;s__Buchnera_aphidicola
MDJ-2_gene_2087	111	WP_081334500.1	938	39/101(38.61)	4.63e-14	hypothetical protein [Anaplasma phagocytophilum]	None	Unclassified

MDJ-2_gene_2094	159	BBA85226.1	158	81/154(52.60)	6.65e-45	crossover junction endodeoxyribonuclease RuvC [endosymbiont of Sipalinus gigas]	1972134	d__Bacteria;k__norank;p__Proteobacteria;c__Gammaproteobacteria;o__norank;f__norank;g__Candidatus_Nardonella;s__endosymbiont_of_Sipalinus_gigas
MDJ-2_gene_2095	144	WP_115955843.1	569	81/141(57.45)	2.89e-43	aspartate--tRNA ligase [Candidatus Purcelliella	472834	d__Bacteria;k__norank;p__Proteobacteria;c__Gammaproteobacteria;o__Enterobacterales;f__Enterobacteriaceae;g__Candidatus_Purcelliella;s__Candidatus_Purcelliella_pentastirinorum
MDJ-2_gene_2107	287	WP_010403990.1	298	239/276(86.59)	1.29e-152	hypothetical protein [Wolbachia endosymbiont of Nasonia vitripennis]	None	Unclassified
MDJ-2_gene_2108	94	WP_063630899.1	276	92/94(97.87)	1.07e-47	hypothetical protein [Wolbachia endosymbiont of Laodelphax striatella]	None	Unclassified
MDJ-2_gene_2112	278	BBA84948.1	349	119/266(44.74)	2.97e-50	GTPase Obg [endosymbiont of Pachyrhynchus infernalis]	1971488	d__Bacteria;k__norank;p__Proteobacteria;c__Gammaproteobacteria;o__norank;f__norank;g__Candidatus_Nardonella;s__endosymbiont_of_Pachyrhynchus_infernalis
MDJ-2_gene_2113	82	RAP29850.1	84	53/82(64.63)	4.14e-32	50S ribosomal protein L27 [Candidatus Marinamargulisbacteria	2184343	d__Bacteria;k__norank;p__Candidatus_Margulisbacteria;c__Candidatus_Marinamargulisbacteria;o__norank;f__norank;g__norank;s__Candidatus_Marinamargulisbacteria_bacterium_SCGC_AG-343-D04
MDJ-2_gene_2119	167	PBD15875.1	166	164/167(98.20)	2.47e-111	hypothetical protein CLD06_03670 [Wolbachia endosymbiont of Drosophila	2033655	d__Bacteria;k__norank;p__Proteobacteria;c__Alphaproteobacteria;o__Rickettsiales;f__Anaplasmataceae;g__Wolbachia;s__Wolbachia_endosymbiont_of_Drosophila_subpulchrella
MDJ-2_gene_2124	82	RYH01092.1	155	31/66(46.97)	6.00e-16	hypothetical protein EON65_48575 [archaeon]	1906665	d__Archaea;k__norank;p__norank;c__norank;o__norank;f__norank;g__norank;s__archaeon
MDJ-2_gene_2131	123	BAA89644.1	123	118/123(95.93)	2.11e-73	hypothetical protein [Wolbachia phage WO]	112596	d__Viruses;k__norank;p__Uroviricota;c__Caudoviricetes;o__Caudovirales;f__Myoviridae;g__norank;s__Wolbachia_phage_WO
MDJ-2_gene_2139	71	CEN32158.1	72	57/71(80.28)	3.35e-35	Translation initiation factor IF-1 [Candidatus Westeberhardia cardiocondylae]	1594731	d__Bacteria;k__norank;p__Proteobacteria;c__Gammaproteobacteria;o__Enterobacterales;f__Enterobacteriaceae;g__Candidatus_Westeberhardia;s__Candidatus_Westeberhardia_cardiocondylae
MDJ-2_gene_2141	336	WP_010304114.1	422	127/341(37.24)	2.07e-52	hypothetical protein [Candidatus Odyssella thessalonicensis]	84647	d__Bacteria;k__norank;p__Proteobacteria;c__Alphaproteobacteria;o__Holosporales;f__Candidatus_Paracaedibacteraceae;g__Candidatus_Odyssella;s__Candidatus_Odyssella_thessalonicensis
MDJ-2_gene_2143	99	WP_114517731.1	95	89/95(93.68)	8.18e-50	hypothetical protein [Wolbachia endosymbiont of Cylisticus convexus]	118728	d__Bacteria;k__norank;p__Proteobacteria;c__Alphaproteobacteria;o__Rickettsiales;f__Anaplasmataceae;g__Wolbachia;s__Wolbachia_endosymbiont_of_Cylisticus_convexus
MDJ-2_gene_2146	74	BAH22220.1	74	69/74(93.24)	1.06e-39	hypothetical protein [Wolbachia endosymbiont of Cadra cautella]	190193	d__Bacteria;k__norank;p__Proteobacteria;c__Alphaproteobacteria;o__Rickettsiales;f__Anaplasmataceae;g__Wolbachia;s__Wolbachia_endosymbiont_of_Cadra_cautella
MDJ-2_gene_2147	304	WP_065095279.1	605	297/304(97.70)	3.16e-216	MULTISPECIES: phage terminase large subunit family protein [Wolbachia]	2640676	d__Bacteria;k__norank;p__Proteobacteria;c__Alphaproteobacteria;o__Rickettsiales;f__Anaplasmataceae;g__Wolbachia;s__norank
MDJ-2_gene_2149	75	SCV03751.1	1132	29/68(42.65)	5.62e-09	LAME_0H12926g1_1 [Lachancea meyersii CBS 8951]	1266667	d__Eukaryota;k__Fungi;p__Ascomycota;c__Saccharomycetes;o__Saccharomycetales;f__Saccharomycetaceae;g__Lachancea;s__Lachancea_meyersii
MDJ-2_gene_2160	78	QAX91981.1	81	34/77(44.16)	9.65e-18	holin protein [Klebsiella phage YX3973]	2500166	d__Viruses;k__norank;p__Uroviricota;c__Caudoviricetes;o__Caudovirales;f__Siphoviridae;g__norank;s__Klebsiella_phage_YX3973
MDJ-2_gene_2161	113	SQH92791.1	124	57/109(52.29)	8.57e-29	Uncharacterised protein [Salmonella enterica subsp.	59202	d__Bacteria;k__norank;p__Proteobacteria;c__Gammaproteobacteria;o__Enterobacterales;f__Enterobacteriaceae;g__Salmonella;s__Salmonella_enterica
MDJ-2_gene_2162	90	QAY00901.1	91	41/89(46.07)	5.37e-15	hypothetical protein SeSz2_42 [Salmonella phage SeSz-2]	2419753	d__Viruses;k__norank;p__Uroviricota;c__Caudoviricetes;o__Caudovirales;f__Siphoviridae;g__norank;s__Salmonella_phage_SeSz-2
MDJ-2_gene_2163	61	RLR58005.1	68	53/59(89.83)	1.51e-30	hypothetical protein CKA49_36450 [Pseudomonas	287	d__Bacteria;k__norank;p__Proteobacteria;c__Gammaproteobacteria;o__Pseudomonadales;f__Pseudomonadaceae;g__Pseudomonas;s__Pseudomonas_aeruginosa
MDJ-2_gene_2169	56	AGJ99081.1	56	53/56(94.64)	6.85e-26	hypothetical protein wNo_06750 [Wolbachia endosymbiont of Drosophila simulans wNo]	1236908	d__Bacteria;k__norank;p__Proteobacteria;c__Alphaproteobacteria;o__Rickettsiales;f__Anaplasmataceae;g__Wolbachia;s__Wolbachia_endosymbiont_of_Drosophila_simulans

MDJ-2_gene_2171	323	CAQ55158.1	438	304/322(94.41)	2.50e-216	Hypothetical protein WP1050 [Wolbachia endosymbiont of Culex quinquefasciatus Pel]	570417	d__Bacteria;k__norank;p__Proteobacteria;c__Alphaproteobacteria;o__Rickettsiales;f__Anaplasmataceae;g__Wolbachia;s__Wolbachia_endosymbiont_of_Culex_quinquefasciatus
MDJ-2_gene_2184	84	WP_046289123.1	109	34/80(42.50)	4.82e-15	TusE/DsrC/DsvC family sulfur relay protein [Blochmannia endosymbiont of Polyrhachis	1505596	d__Bacteria;k__norank;p__Proteobacteria;c__Gammaproteobacteria;o__Enterobacterales;f__Enterobacteriaceae;g__Candidatus_Blochmannia;s__Blochmannia_endosymbiont_of_Polyrhachis_(Hedomyrma)_turneri
MDJ-2_gene_2187	179	AEO08038.1	185	65/177(36.72)	4.04e-18	ribosome recycling factor [Buchnera aphidicola str. Ua (Uroleucon ambrosiae)]	1005057	d__Bacteria;k__norank;p__Proteobacteria;c__Gammaproteobacteria;o__Enterobacterales;f__Erwiniaceae;g__Buchnera;s__Buchnera_aphidicola
MDJ-2_gene_2206	204	PCG11886.1	204	201/204(98.53)	8.22e-142	50S ribosomal protein L4 [Wolbachia pipientis]	None	Unclassified
MDJ-2_gene_2212	93	ATE85677.1	94	61/93(65.59)	2.12e-32	hypothetical protein Sf11_gp30 [Shigella phage Sf11 SMD-2017]	2282196	d__Viruses;k__norank;p__Uroviricota;c__Caudoviricetes;o__Caudovirales;f__Siphoviridae;g__norank;s__Shigella_phage_Sf11_SMD-2017
MDJ-2_gene_2213	152	ARK07762.1	146	78/148(52.70)	8.00e-49	hypothetical protein LPST10_00030 [Salmonella	1973454	d__Viruses;k__norank;p__Uroviricota;c__Caudoviricetes;o__Caudovirales;f__Siphoviridae;g__norank;s__Salmonella_phage_LPST10
MDJ-2_gene_2214	52	AVJ51765.1	885	41/52(78.85)	8.76e-21	structural protein containing EPS-depolymerase domain [Pantoea phage vB_PagS_Vid5]	2099652	d__Viruses;k__norank;p__Uroviricota;c__Caudoviricetes;o__Caudovirales;f__Siphoviridae;g__Vidquintavirus;s__Pantoea_virus_Vid5
MDJ-2_gene_2222	78	OAL53019.1	210	33/70(47.14)	1.42e-12	cysteine dioxygenase type I [Pyrenochaeta sp. DS3sAY3a]	765867	d__Eukaryota;k__Fungi;p__Ascomycota;c__Dothideomycetes;o__Pleosporales;f__Cucurbitariaceae;g__Pyrenochaeta;s__Pyrenochaeta_sp._DS3sAY3a
MDJ-2_gene_2231	186	OSY19256.1	647	86/309(27.83)	1.90e-19	hypothetical protein B0D75_01370 [Entomoplasmatales bacterium	None	Unclassified
MDJ-2_gene_2246	208	EEB56031.1	208	206/208(99.04)	6.01e-146	putative rare lipoprotein A [Wolbachia endosymbiont of Culex quinquefasciatus JHB]	569881	d__Bacteria;k__norank;p__Proteobacteria;c__Alphaproteobacteria;o__Rickettsiales;f__Anaplasmataceae;g__Wolbachia;s__Wolbachia_endosymbiont_of_Culex_quinquefasciatus
MDJ-2_gene_2270	91	ETW98372.1	393	37/87(42.53)	7.69e-18	hypothetical protein ETSY2_42930 [Candidatus	1429439	d__Bacteria;k__norank;p__Candidatus_Tectomicrobia;c__norank;o__norank;f__norank;g__Candidatus_Entotheonella;s__Candidatus_Entotheonella_gemina
MDJ-2_gene_2272	60	ETW98900.1	920	30/65(46.15)	8.37e-07	hypothetical protein ETSY2_42015 [Candidatus	1429439	d__Bacteria;k__norank;p__Candidatus_Tectomicrobia;c__norank;o__norank;f__norank;g__Candidatus_Entotheonella;s__Candidatus_Entotheonella_gemina
MDJ-2_gene_2274	52	EEB55878.1	46	25/28(89.29)	1.93e-07	hypothetical protein C1A_900 [Wolbachia endosymbiont of Culex quinquefasciatus JHB]	569881	d__Bacteria;k__norank;p__Proteobacteria;c__Alphaproteobacteria;o__Rickettsiales;f__Anaplasmataceae;g__Wolbachia;s__Wolbachia_endosymbiont_of_Culex_quinquefasciatus
MDJ-2_gene_2275	177	PBQ28192.1	177	173/177(97.74)	8.14e-122	peptidase [Wolbachia pipientis wAus]	2049495	d__Bacteria;k__norank;p__Proteobacteria;c__Alphaproteobacteria;o__Rickettsiales;f__Anaplasmataceae;g__Wolbachia;s__Wolbachia_pipientis
MDJ-2_gene_2280	246	BBA84656.1	351	153/244(62.70)	5.17e-91	peptide chain release factor 1 [endosymbiont of Euscepes postfasciatus]	650377	d__Bacteria;k__norank;p__Proteobacteria;c__Gammaproteobacteria;o__norank;f__norank;g__norank;s__endosymbiont_of_Euscepes_postfasciatus
MDJ-2_gene_2288	220	ORM82390.1	220	214/220(97.27)	6.00e-161	hypothetical protein HA42_08495 [Pantoea deleyi]	470932	d__Bacteria;k__norank;p__Proteobacteria;c__Gammaproteobacteria;o__Enterobacterales;f__Erwiniaceae;g__Pantoea;s__Pantoea_deleyi
MDJ-2_gene_2293	60	WP_122889988.1	226	46/49(93.88)	2.59e-20	hypothetical protein [[Curtobacterium] plantarum]	221276	d__Bacteria;k__norank;p__Proteobacteria;c__Gammaproteobacteria;o__Enterobacterales;f__Erwiniaceae;g__Pantoea;s__[Curtobacterium]_plantarum
MDJ-2_gene_2294	266	WP_126483532.1	370	264/266(99.25)	8.09e-193	IS3 family transposase [Serratia plymuthica]	82996	d__Bacteria;k__norank;p__Proteobacteria;c__Gammaproteobacteria;o__Enterobacterales;f__Yersiniaceae;g__Serratia;s__Serratia_plymuthica
MDJ-2_gene_2295	249	CEN32242.1	369	100/249(40.16)	1.48e-36	DNA polymerase III subunit beta [Candidatus Westeberhardia cardiocondylae]	1594731	d__Bacteria;k__norank;p__Proteobacteria;c__Gammaproteobacteria;o__Enterobacterales;f__Enterobacteriaceae;g__Candidatus_Westeberhardia;s__Candidatus_Westeberhardia_cardiocondylae
MDJ-2_gene_2296	227	BBA85262.1	275	152/228(66.67)	4.35e-92	50S ribosomal protein L2 [endosymbiont of Sipalinus gigas]	1972134	d__Bacteria;k__norank;p__Proteobacteria;c__Gammaproteobacteria;o__norank;f__norank;g__Candidatus_Nardonella;s__endosymbiont_of_Sipalinus_gigas

MDJ-2_gene_2297	78	PKK87587.1	95	28/65(43.08)	4.36e-09	50S ribosomal protein L23 [Tenericutes bacterium HGW-Tenericutes-8]	2013851	d__Bacteria;k__norank;p__Tenericutes;c__norank;o__norank;f__norank;g__norank;s__Tenericutes_bacterium_HGW-Tenericutes-8
MDJ-2_gene_2301	301	ORM86132.1	301	300/301(99.67)	2.21e-217	hypothetical protein HA42_01465 [Pantoea deleyi]	470932	d__Bacteria;k__norank;p__Proteobacteria;c__Gammaproteobacteria;o__Enterobacterales;f__Erwiniaceae;g__Pantoea;s__Pantoea_deleyi
MDJ-2_gene_2304	73	PAW33696.1	73	71/73(97.26)	1.77e-42	type II toxin-antitoxin system prevent-host-death family antitoxin [Pantoea vagans]	470934	d__Bacteria;k__norank;p__Proteobacteria;c__Gammaproteobacteria;o__Enterobacterales;f__Erwiniaceae;g__Pantoea;s__Pantoea_vagans
MDJ-2_gene_2305	126	PAW34156.1	127	117/123(95.12)	2.42e-76	type II toxin-antitoxin system death-on-curing family toxin [Pantoea vagans]	470934	d__Bacteria;k__norank;p__Proteobacteria;c__Gammaproteobacteria;o__Enterobacterales;f__Erwiniaceae;g__Pantoea;s__Pantoea_vagans
MDJ-2_gene_2316	203	WP_127463963.1	203	203/203(100.00)	1.16e-136	50S ribosomal protein L25/general stress protein Ctc [Wolbachia endosymbiont of	215173	d__Bacteria;k__norank;p__Proteobacteria;c__Alphaproteobacteria;o__Rickettsiales;f__Anaplasmataceae;g__Wolbachia;s__Wolbachia_endosymbiont_of_Bemisia_tabaci
MDJ-2_gene_2323	313	PBQ25023.1	293	164/317(51.74)	1.93e-74	hypothetical protein BTO27_05620 [Wolbachia	2049495	d__Bacteria;k__norank;p__Proteobacteria;c__Alphaproteobacteria;o__Rickettsiales;f__Anaplasmataceae;g__Wolbachia;s__Wolbachia_pipientis
MDJ-2_gene_2327	210	ORM82832.1	210	203/209(97.13)	2.82e-136	dTMP kinase [Pantoea deleyi]	470932	d__Bacteria;k__norank;p__Proteobacteria;c__Gammaproteobacteria;o__Enterobacterales;f__Erwiniaceae;g__Pantoea;s__Pantoea_deleyi
MDJ-2_gene_2345	99	WP_052883314.1	479	32/66(48.48)	3.87e-09	cysteine--tRNA ligase [Thermoproteus tenax]	2271	d__Archaea;k__norank;p__Crenarchaeota;c__Thermoprotei;o__Thermoproteales;f__Thermoproteaceae;g__Thermoproteus;s__Thermoproteus_tenax
MDJ-2_gene_2346	180	WP_041063308.1	467	90/164(54.88)	1.14e-49	cysteine--tRNA ligase [Candidatus Tachikawaea	1410383	d__Bacteria;k__norank;p__Proteobacteria;c__Gammaproteobacteria;o__Enterobacterales;f__Enterobacteriaceae;g__Candidatus_Tachikawaea;s__Candidatus_Tachikawaea_gelatinosa
MDJ-2_gene_2350	278	PBQ29178.1	278	276/278(99.28)	2.77e-204	transcription termination/antitermination factor NusG [Wolbachia pipientis	2049495	d__Bacteria;k__norank;p__Proteobacteria;c__Alphaproteobacteria;o__Rickettsiales;f__Anaplasmataceae;g__Wolbachia;s__Wolbachia_pipientis
MDJ-2_gene_2351	94	ORX81757.1	447	40/91(43.96)	3.62e-16	Actin/actin-like protein [Anaeromyces robustus]	1754192	d__Eukaryota;k__Fungi;p__Chytridiomycota;c__Neocallimastigomycetes;o__Neocallimastigales;f__Neocallimastigaceae;g__Anaeromyces;s__Anaeromyces_robustus
MDJ-2_gene_2367	77	RYA73290.1	513	26/50(52.00)	4.86e-10	hypothetical protein DD592_26090, partial [Enterobacter cloacae complex	2511993	d__Bacteria;k__norank;p__Proteobacteria;c__Gammaproteobacteria;o__Enterobacterales;f__Enterobacteriaceae;g__Enterobacter;s__Enterobacter_cloacae_complex_sp._2DZ2F20B
MDJ-2_gene_2393	123	AYR02306.1	318	69/122(56.56)	1.02e-40	putative head assembly protein [Escherichia phage C1]	2340716	d__Viruses;k__norank;p__Uroviricota;c__Caudoviricetes;o__Caudovirales;f__Siphoviridae;g__norank;s__Escherichia_phage_C1
MDJ-2_gene_2394	66	ARB12511.1	78	35/66(53.03)	2.85e-20	hypothetical protein BIS47_07 [Klebsiella phage	1907784	d__Viruses;k__norank;p__Uroviricota;c__Caudoviricetes;o__Caudovirales;f__Myoviridae;g__norank;s__Klebsiella_phage_vB_KpnM_BIS47
MDJ-2_gene_2395	75	WP_126956123.1	102	39/53(73.58)	6.08e-17	hypothetical protein [Citrobacter portucalensis]	1344959	d__Bacteria;k__norank;p__Proteobacteria;c__Gammaproteobacteria;o__Enterobacterales;f__Enterobacteriaceae;g__Citrobacter;s__norank
MDJ-2_gene_2396	361	WP_105099977.1	394	359/361(99.45)	3.90e-263	MULTISPECIES: elongation factor Tu [Pantoea]	53335	d__Bacteria;k__norank;p__Proteobacteria;c__Gammaproteobacteria;o__Enterobacterales;f__Erwiniaceae;g__Pantoea;s__norank
MDJ-2_gene_2397	44	RZK36074.1	620	25/44(56.82)	2.70e-06	hypothetical protein EOO61_10965, partial	1898978	d__Bacteria;k__norank;p__Bacteroidetes;c__Cytophagia;o__Cytophagales;f__Hymenobacteraceae;g__Hymenobacter;s__Hymenobacter_sp.
MDJ-2_gene_2421	61	BBA85128.1	398	29/61(47.54)	4.56e-07	threonine--tRNA ligase [endosymbiont of Rhynchophorus ferrugineus]	1972133	d__Bacteria;k__norank;p__Proteobacteria;c__Gammaproteobacteria;o__norank;f__norank;g__Candidatus_Nardonella;s__Candidatus_Nardonella_dryophthoridicola
MDJ-2_gene_2422	102	WP_080626648.1	404	65/99(65.66)	1.71e-37	threonine--tRNA ligase [Candidatus Riesia	428411	d__Bacteria;k__norank;p__Proteobacteria;c__Gammaproteobacteria;o__Enterobacterales;f__Enterobacteriaceae;g__Candidatus_Riesia;s__Candidatus_Riesia_pediculischaeffi
MDJ-2_gene_2430	266	WP_114517186.1	272	254/266(95.49)	3.60e-175	hypothetical protein [Wolbachia endosymbiont of Cylisticus convexus]	118728	d__Bacteria;k__norank;p__Proteobacteria;c__Alphaproteobacteria;o__Rickettsiales;f__Anaplasmataceae;g__Wolbachia;s__Wolbachia_endosymbiont_of_Cylisticus_convexus

MDJ-2_gene_2433	185	OUM69954.1	221	128/182(70.33)	5.07e-89	hypothetical protein PIROE2DRAFT_37820, partial [Piromyces sp. E2]	73868	d__Eukaryota;k__Fungi;p__Chytridiomycota;c__Neocallimastigomycetes;o__Neocallimastigales;f__Neocallimastigaceae;g__Piromyces;s__Piromyces_sp._E2
MDJ-2_gene_2437	150	OQV42861.1	150	138/150(92.00)	2.35e-92	hypothetical protein BZ160_01670 [Pantoea vagans]	470934	d__Bacteria;k__norank;p__Proteobacteria;c__Gammaproteobacteria;o__Enterobacterales;f__Erwiniaceae;g__Pantoea;s__Pantoea_vagans
MDJ-2_gene_2465	353	BBA84806.1	838	185/341(54.25)	3.18e-57	DNA gyrase subunit A [endosymbiont of Pachyrhynchus infernalis]	1971488	d__Bacteria;k__norank;p__Proteobacteria;c__Gammaproteobacteria;o__norank;f__norank;g__Candidatus_Nardonella;s__endosymbiont_of_Pachyrhynchus_infernalis
MDJ-2_gene_2467	227	SVK45669.1	141	80/137(58.39)	1.85e-45	Uncharacterised protein [Acinetobacter baumannii]	470	d__Bacteria;k__norank;p__Proteobacteria;c__Gammaproteobacteria;o__Pseudomonadales;f__Moraxellaceae;g__Acinetobacter;s__Acinetobacter_baumannii
MDJ-2_gene_2472	231	WP_072794668.1	59	37/59(62.71)	1.14e-14	hypothetical protein [Escherichia coli]	562	d__Bacteria;k__norank;p__Proteobacteria;c__Gammaproteobacteria;o__Enterobacterales;f__Enterobacteriaceae;g__Escherichia;s__Escherichia_coli
MDJ-2_gene_2475	181	PCG11812.1	176	167/181(92.27)	2.48e-101	DUF2497 domain-containing protein [Wolbachia pipientis]	None	Unclassified
MDJ-2_gene_2480	232	ORM81093.1	241	230/232(99.14)	3.33e-157	chromosome partitioning protein MukE [Pantoea deleyi]	470932	d__Bacteria;k__norank;p__Proteobacteria;c__Gammaproteobacteria;o__Enterobacterales;f__Erwiniaceae;g__Pantoea;s__Pantoea_deleyi
MDJ-2_gene_2484	49	CSC02183.1	49	28/49(57.14)	3.81e-09	Uncharacterised protein [Vibrio cholerae]	666	d__Bacteria;k__norank;p__Proteobacteria;c__Gammaproteobacteria;o__Vibrionales;f__Vibrionaceae;g__Vibrio;s__Vibrio_cholerae
MDJ-2_gene_2494	133	BBA84958.1	690	68/133(51.13)	3.82e-21	polyribonucleotide nucleotidyltransferase [endosymbiont of Pachyrhynchus infernalis]	1971488	d__Bacteria;k__norank;p__Proteobacteria;c__Gammaproteobacteria;o__norank;f__norank;g__Candidatus_Nardonella;s__endosymbiont_of_Pachyrhynchus_infernalis
MDJ-2_gene_2504	87	WP_123802026.1	87	83/87(95.40)	2.75e-50	XRE family transcriptional regulator [Pantoea sp. RIT388]	1869313	d__Bacteria;k__norank;p__Proteobacteria;c__Gammaproteobacteria;o__Enterobacterales;f__Erwiniaceae;g__Pantoea;s__Pantoea_sp._RIT388
MDJ-2_gene_2506	167	ORM81494.1	167	157/167(94.01)	7.91e-106	hypothetical protein HA42_10505 [Pantoea deleyi]	470932	d__Bacteria;k__norank;p__Proteobacteria;c__Gammaproteobacteria;o__Enterobacterales;f__Erwiniaceae;g__Pantoea;s__Pantoea_deleyi
MDJ-2_gene_2508	256	YP_009322787.1	312	206/256(80.47)	1.14e-148	exonuclease [Salmonella phage IME207]	1873985	d__Viruses;k__norank;p__Uroviricota;c__Caudoviricetes;o__Caudovirales;f__Siphoviridae;g__norank;s__Salmonella_phage_IME207
MDJ-2_gene_2514	164	RUM44803.1	821	57/158(36.08)	1.65e-16	hypothetical protein DSY80_04015 [Desulfocapsa	2066021	d__Bacteria;k__norank;p__Proteobacteria;c__Deltaproteobacteria;o__Desulfobacterales;f__Desulfobulbaceae;g__Desulfocapsa;s__Desulfocapsa_sp.
MDJ-2_gene_2518	107	WP_033758124.1	114	28/50(56.00)	1.30e-08	hypothetical protein [Pantoea agglomerans]	549	d__Bacteria;k__norank;p__Proteobacteria;c__Gammaproteobacteria;o__Enterobacterales;f__Erwiniaceae;g__Pantoea;s__Pantoea_agglomerans
MDJ-2_gene_2523	55	OLR87714.1	55	55/55(100.00)	9.76e-30	50S ribosomal protein L33 [Escherichia coli]	562	d__Bacteria;k__norank;p__Proteobacteria;c__Gammaproteobacteria;o__Enterobacterales;f__Enterobacteriaceae;g__Escherichia;s__Escherichia_coli
MDJ-2_gene_2549	215	YP_009322264.1	495	147/217(67.74)	2.72e-93	hypothetical protein [Salmonella phage 64795_sal3]	1813769	d__Viruses;k__norank;p__Uroviricota;c__Caudoviricetes;o__Caudovirales;f__Siphoviridae;g__norank;s__Salmonella_phage_64795_sal3
MDJ-2_gene_2550	123	YP_001742084.1	317	59/123(47.97)	2.56e-30	putative DNA primase [Salmonella phage Vi II-E1]	424716	d__Viruses;k__norank;p__Uroviricota;c__Caudoviricetes;o__Caudovirales;f__Siphoviridae;g__norank;s__Salmonella_phage_Vi_II-E1
MDJ-2_gene_2552	66	PBQ29156.1	66	65/66(98.48)	5.45e-34	30S ribosomal protein S21 [Wolbachia pipientis wAus]	2049495	d__Bacteria;k__norank;p__Proteobacteria;c__Alphaproteobacteria;o__Rickettsiales;f__Anaplasmataceae;g__Wolbachia;s__Wolbachia_pipientis
MDJ-2_gene_2555	125	RLT60446.1	490	121/124(97.58)	6.02e-87	phage terminase large subunit family protein, partial [Wolbachia endosymbiont of Drosophila ananassae]	307502	d__Bacteria;k__norank;p__Proteobacteria;c__Alphaproteobacteria;o__Rickettsiales;f__Anaplasmataceae;g__Wolbachia;s__Wolbachia_endosymbiont_of_Drosophila_ananassae
MDJ-2_gene_2557	161	WP_019078753.1	178	152/158(96.20)	4.01e-109	hypothetical protein, partial [Wolbachia endosymbiont of Culex pipiens molestus]	263438	d__Bacteria;k__norank;p__Proteobacteria;c__Alphaproteobacteria;o__Rickettsiales;f__Anaplasmataceae;g__Wolbachia;s__Wolbachia_endosymbiont_of_Culex_pipiens_molestus
MDJ-2_gene_2590	171	CCE77661.1	171	166/171(97.08)	1.47e-112	conserved membrane hypothetical protein (RDD domain) [Wolbachia pipientis wAlbB]	1116230	d__Bacteria;k__norank;p__Proteobacteria;c__Alphaproteobacteria;o__Rickettsiales;f__Anaplasmataceae;g__Wolbachia;s__Wolbachia_pipientis

MDJ-2_gene_2603	184	BBA84638.1	598	104/189(55.03)	3.23e-57	ribonuclease E [endosymbiont of Euscepes postfasciatus]	650377	d__Bacteria;k__norank;p__Proteobacteria;c__Gammaproteobacteria;o__norank;f__norank;g__norank;s__endosymbiont_of_Euscepes_postfasciatus
MDJ-2_gene_2604	105	BBA85320.1	569	68/105(64.76)	1.20e-35	ribonuclease E [endosymbiont of Sipalinus gigas]	1972134	d__Bacteria;k__norank;p__Proteobacteria;c__Gammaproteobacteria;o__norank;f__norank;g__Candidatus_Nardonella;s__endosymbiont_of_Sipalinus_gigas
MDJ-2_gene_2616	133	ORY72578.1	517	44/112(39.29)	1.01e-19	adenylate cyclase associated N terminal-domain-containing protein [Leucosporidium	106004	d__Eukaryota;k__Fungi;p__Basidiomycota;c__Microbotryomycetes;o__Leucosporidiales;f__Leucosporidiaceae;g__Leucosporidium;s__Leucosporidium_creatinivorum
MDJ-2_gene_2617	211	EEB55202.1	217	192/210(91.43)	2.24e-128	hypothetical protein C1A_5 [Wolbachia endosymbiont of Culex quinquefasciatus JHB]	569881	d__Bacteria;k__norank;p__Proteobacteria;c__Alphaproteobacteria;o__Rickettsiales;f__Anaplasmataceae;g__Wolbachia;s__Wolbachia_endosymbiont_of_Culex_quinquefasciatus
MDJ-2_gene_2618	83	EEB55201.1	89	75/82(91.46)	1.55e-44	hypothetical protein C1A_4 [Wolbachia endosymbiont of Culex quinquefasciatus JHB]	569881	d__Bacteria;k__norank;p__Proteobacteria;c__Alphaproteobacteria;o__Rickettsiales;f__Anaplasmataceae;g__Wolbachia;s__Wolbachia_endosymbiont_of_Culex_quinquefasciatus
MDJ-2_gene_2621	103	BBA85224.1	293	54/99(54.55)	1.82e-23	glycine--tRNA ligase alpha subunit [endosymbiont of	1972134	d__Bacteria;k__norank;p__Proteobacteria;c__Gammaproteobacteria;o__norank;f__norank;g__Candidatus_Nardonella;s__endosymbiont_of_Sipalinus_gigas
MDJ-2_gene_2622	93	WP_041062386.1	294	53/93(56.99)	4.28e-29	glycine--tRNA ligase subunit alpha [Candidatus Tachikawaea gelatinosa]	1410383	d__Bacteria;k__norank;p__Proteobacteria;c__Gammaproteobacteria;o__Enterobacterales;f__Enterobacteriaceae;g__Candidatus_Tachikawaea;s__Candidatus_Tachikawaea_gelatinosa
MDJ-2_gene_2630	54	VEB51556.1	112	50/54(92.59)	3.75e-28	DNA-directed RNA polymerase subunit beta' [Salmonella enterica subsp. enterica]	59201	d__Bacteria;k__norank;p__Proteobacteria;c__Gammaproteobacteria;o__Enterobacterales;f__Enterobacteriaceae;g__Salmonella;s__Salmonella_enterica
MDJ-2_gene_2631	106	SQI29818.1	170	49/106(46.23)	4.56e-23	DNA-directed RNA polymerase subunit beta' [Serratia plymuthica]	82996	d__Bacteria;k__norank;p__Proteobacteria;c__Gammaproteobacteria;o__Enterobacterales;f__Yersiniaceae;g__Serratia;s__Serratia_plymuthica
MDJ-2_gene_2632	47	BBA84826.1	1328	31/43(72.09)	4.84e-10	DNA-directed RNA polymerase subunit beta [endosymbiont of Pachyrhynchus infernalis]	1971488	d__Bacteria;k__norank;p__Proteobacteria;c__Gammaproteobacteria;o__norank;f__norank;g__Candidatus_Nardonella;s__endosymbiont_of_Pachyrhynchus_infernalis
MDJ-2_gene_2647	135	YP_009305131.1	681	50/122(40.98)	1.49e-28	glycoprotein precursor [Wuhan mosquito virus 1]	1608126	d__Viruses;k__Orthornavirae;p__Negarnaviricota;c__Ellioviricetes;o__Bunyavirales;f__Phasmaviridae;g__Orthophasmavirus;s__Wuhan_mosquito_orthophasmavirus_1
MDJ-2_gene_2656	298	KYG40015.1	345	47/134(35.07)	3.51e-17	hypothetical protein M433DRAFT_98540, partial [Acidomyces richmondensis	766039	d__Eukaryota;k__Fungi;p__Ascomycota;c__Dothideomycetes;o__Mycosphaerellales;f__Teratosphaeriaceae;g__Acidomyces;s__Acidomyces_sp._'richmondensis'
MDJ-2_gene_2657	46	BBA84864.1	339	32/42(76.19)	6.20e-11	phenylalanine--tRNA ligase alpha subunit [endosymbiont of Pachyrhynchus infernalis]	1971488	d__Bacteria;k__norank;p__Proteobacteria;c__Gammaproteobacteria;o__norank;f__norank;g__Candidatus_Nardonella;s__endosymbiont_of_Pachyrhynchus_infernalis
MDJ-2_gene_2658	114	BBA85232.1	334	34/74(45.95)	2.15e-09	phenylalanine--tRNA ligase alpha subunit [endosymbiont of Sipalinus gigas]	1972134	d__Bacteria;k__norank;p__Proteobacteria;c__Gammaproteobacteria;o__norank;f__norank;g__Candidatus_Nardonella;s__endosymbiont_of_Sipalinus_gigas
MDJ-2_gene_2674	269	RUM30450.1	946	61/238(25.63)	4.64e-13	hypothetical protein DSY42_04590 [Aquifex sp.]	313370	d__Bacteria;k__norank;p__Aquificae;c__Aquificae;o__Aquificales;f__Aquificaceae;g__Aquifex;s__Aquifex_sp.
MDJ-2_gene_2677	186	ORM81589.1	199	171/186(91.94)	4.16e-111	hypothetical protein HA42_10230 [Pantoea deleyi]	470932	d__Bacteria;k__norank;p__Proteobacteria;c__Gammaproteobacteria;o__Enterobacterales;f__Erwiniaceae;g__Pantoea;s__Pantoea_deleyi
MDJ-2_gene_2683	180	WP_031590436.1	179	157/180(87.22)	7.58e-101	type 1 fimbrial protein [Pantoea agglomerans]	549	d__Bacteria;k__norank;p__Proteobacteria;c__Gammaproteobacteria;o__Enterobacterales;f__Erwiniaceae;g__Pantoea;s__Pantoea_agglomerans
MDJ-2_gene_2705	96	ORM86083.1	96	91/96(94.79)	5.01e-66	hypothetical protein HA42_01190 [Pantoea deleyi]	470932	d__Bacteria;k__norank;p__Proteobacteria;c__Gammaproteobacteria;o__Enterobacterales;f__Erwiniaceae;g__Pantoea;s__Pantoea_deleyi
MDJ-2_gene_2717	178	BBA84796.1	921	118/177(66.67)	5.54e-73	DNA-directed DNA polymerase [endosymbiont of Pachyrhynchus infernalis]	1971488	d__Bacteria;k__norank;p__Proteobacteria;c__Gammaproteobacteria;o__norank;f__norank;g__Candidatus_Nardonella;s__endosymbiont_of_Pachyrhynchus_infernalis

MDJ-2_gene_2718	96	BBA85059.1	930	71/94(75.53)	3.24e-33	DNA-directed DNA polymerase [endosymbiont of Rhynchophorus ferrugineus]	1972133	d__Bacteria;k__norank;p__Proteobacteria;c__Gammaproteobacteria;o__norank;f__norank;g__Candidatus_Nardonella;s__Candidatus_Nardonella_dryophthoridicola
MDJ-2_gene_2731	50	PBQ28233.1	330	50/50(100.00)	1.50e-22	P-type DNA transfer ATPase VirB11 [Wolbachia pipientis	2049495	d__Bacteria;k__norank;p__Proteobacteria;c__Alphaproteobacteria;o__Rickettsiales;f__Anaplasmataceae;g__Wolbachia;s__Wolbachia_pipientis
MDJ-2_gene_2755	170	WP_108784286.1	211	127/176(72.16)	6.79e-69	hypothetical protein [Wolbachia endosymbiont of Bemisia tabaci]	215173	d__Bacteria;k__norank;p__Proteobacteria;c__Alphaproteobacteria;o__Rickettsiales;f__Anaplasmataceae;g__Wolbachia;s__Wolbachia_endosymbiont_of_Bemisia_tabaci
MDJ-2_gene_2761	104	SJZ30236.1	104	103/104(99.04)	9.35e-68	BolA protein [Pantoea agglomerans]	470933	d__Bacteria;k__norank;p__Proteobacteria;c__Gammaproteobacteria;o__Enterobacterales;f__Erwiniaceae;g__Pantoea;s__Pantoea_eucalypti
MDJ-2_gene_2770	82	RKP02297.1	336	55/81(67.90)	8.06e-34	hypothetical protein CXG81DRAFT_29547 [Caulochytrium protostelioides]	1555241	d__Eukaryota;k__Fungi;p__Chytridiomycota;c__Chytridiomycetes;o__Caulochytriales;f__Caulochytriaceae;g__Caulochytrium;s__Caulochytrium_protostelioides
MDJ-2_gene_2798	82	EAL58392.1	570	38/76(50.00)	2.11e-17	SD27140p [Wolbachia endosymbiont of Drosophila	307502	d__Bacteria;k__norank;p__Proteobacteria;c__Alphaproteobacteria;o__Rickettsiales;f__Anaplasmataceae;g__Wolbachia;s__Wolbachia_endosymbiont_of_Drosophila_ananassae
MDJ-2_gene_2804	243	ORM86534.1	243	241/243(99.18)	3.18e-172	16S rRNA (uracil(1498)-N(3))-methyltransferase [Pantoea	470932	d__Bacteria;k__norank;p__Proteobacteria;c__Gammaproteobacteria;o__Enterobacterales;f__Erwiniaceae;g__Pantoea;s__Pantoea_deleyi
MDJ-2_gene_2807	121	ORM80518.1	113	93/113(82.30)	1.47e-50	hypothetical protein HA42_14250 [Pantoea deleyi]	470932	d__Bacteria;k__norank;p__Proteobacteria;c__Gammaproteobacteria;o__Enterobacterales;f__Erwiniaceae;g__Pantoea;s__Pantoea_deleyi
MDJ-2_gene_2813	102	WP_127463985.1	125	101/102(99.02)	6.58e-56	succinate dehydrogenase, cytochrome b556 subunit [Wolbachia endosymbiont of Bemisia tabaci]	215173	d__Bacteria;k__norank;p__Proteobacteria;c__Alphaproteobacteria;o__Rickettsiales;f__Anaplasmataceae;g__Wolbachia;s__Wolbachia_endosymbiont_of_Bemisia_tabaci
MDJ-2_gene_2820	304	WP_041943676.1	641	229/308(74.35)	1.32e-135	molecular chaperone DnaK [Wigglesworthia glossinidia]	51229	d__Bacteria;k__norank;p__Proteobacteria;c__Gammaproteobacteria;o__Enterobacterales;f__Erwiniaceae;g__Wigglesworthia;s__Wigglesworthia_glossinidia
MDJ-2_gene_2832	316	KKZ05312.1	629	41/139(29.50)	8.79e-06	hypothetical protein YC68_23740, partial [Vibrio	670	d__Bacteria;k__norank;p__Proteobacteria;c__Gammaproteobacteria;o__Vibrionales;f__Vibrionaceae;g__Vibrio;s__Vibrio_parahaemolyticus
MDJ-2_gene_2835	66	QAX91985.1	66	39/66(59.09)	2.16e-22	holin [Klebsiella phage YX3973]	2500166	d__Viruses;k__norank;p__Uroviricota;c__Caudoviricetes;o__Caudovirales;f__Siphoviridae;g__norank;s__Klebsiella_phage_YX3973
MDJ-2_gene_2841	287	AGJ98490.1	822	283/287(98.61)	6.65e-194	Valyl-tRNA synthetase [Wolbachia endosymbiont of Drosophila simulans wNo]	1236908	d__Bacteria;k__norank;p__Proteobacteria;c__Alphaproteobacteria;o__Rickettsiales;f__Anaplasmataceae;g__Wolbachia;s__Wolbachia_endosymbiont_of_Drosophila_simulans
MDJ-2_gene_2851	79	PCG16852.1	409	78/79(98.73)	2.29e-40	lipoprotein-releasing system transmembrane subunit LolC [Wolbachia pipientis]	None	Unclassified
MDJ-2_gene_2856	308	WP_111139999.1	299	295/299(98.66)	3.66e-206	(2E,6E)-farnesyl diphosphate synthase [Pantoea sp. ARC607]	2027922	d__Bacteria;k__norank;p__Proteobacteria;c__Gammaproteobacteria;o__Enterobacterales;f__Erwiniaceae;g__Pantoea;s__Pantoea_sp._ARC607
MDJ-2_gene_2862	155	WP_108784109.1	488	113/145(77.93)	7.75e-71	hypothetical protein [Wolbachia endosymbiont of Bemisia tabaci]	215173	d__Bacteria;k__norank;p__Proteobacteria;c__Alphaproteobacteria;o__Rickettsiales;f__Anaplasmataceae;g__Wolbachia;s__Wolbachia_endosymbiont_of_Bemisia_tabaci
MDJ-2_gene_2863	132	WP_127464307.1	483	113/130(86.92)	3.43e-72	hypothetical protein [Wolbachia endosymbiont of Bemisia tabaci]	215173	d__Bacteria;k__norank;p__Proteobacteria;c__Alphaproteobacteria;o__Rickettsiales;f__Anaplasmataceae;g__Wolbachia;s__Wolbachia_endosymbiont_of_Bemisia_tabaci
MDJ-2_gene_2864	68	ORM78180.1	67	66/67(98.51)	1.42e-41	hemolysin [Pantoea deleyi]	470932	d__Bacteria;k__norank;p__Proteobacteria;c__Gammaproteobacteria;o__Enterobacterales;f__Erwiniaceae;g__Pantoea;s__Pantoea_deleyi
MDJ-2_gene_2875	114	PZL88815.1	114	113/114(99.12)	3.23e-75	RidA family protein [Pantoea sp. ARC270]	2027923	d__Bacteria;k__norank;p__Proteobacteria;c__Gammaproteobacteria;o__Enterobacterales;f__Erwiniaceae;g__Pantoea;s__Pantoea_sp._ARC270
MDJ-2_gene_2893	71	SJZ51006.1	71	71/71(100.00)	1.68e-37	osmotically inducible lipoprotein OsmB [Pantoea agglomerans]	470933	d__Bacteria;k__norank;p__Proteobacteria;c__Gammaproteobacteria;o__Enterobacterales;f__Erwiniaceae;g__Pantoea;s__Pantoea_eucalypti
MDJ-2_gene_2895	49	AEO08576.1	439	27/49(55.10)	4.61e-06	UDP-N-acetylmuramoylalanine-D-glutamate ligase [Buchnera aphidicola str. Ak (Acyrthosiphon kondoi)]	1005090	d__Bacteria;k__norank;p__Proteobacteria;c__Gammaproteobacteria;o__Enterobacterales;f__Erwiniaceae;g__Buchnera;s__Buchnera_aphidicola

MDJ-2_gene_2896	101	BBA84765.1	441	57/101(56.44)	1.06e-27	UDP-N-acetylmuramoylalanine--D-glutamate ligase [endosymbiont of Pachyrhynchus	1971488	d__Bacteria;k__norank;p__Proteobacteria;c__Gammaproteobacteria;o__norank;f__norank;g__Candidatus_Nardonella;s__endosymbiont_of_Pachyrhynchus_infernalis
MDJ-2_gene_2942	87	EZI32703.1	87	72/87(82.76)	2.76e-41	hypothetical protein BW31_03053 [Pantoea	549	d__Bacteria;k__norank;p__Proteobacteria;c__Gammaproteobacteria;o__Enterobacterales;f__Erwiniaceae;g__Pantoea;s__Pantoea_agglomerans
MDJ-2_gene_2963	63	ORM81596.1	63	55/63(87.30)	2.48e-31	hypothetical protein HA42_10270 [Pantoea deleyi]	470932	d__Bacteria;k__norank;p__Proteobacteria;c__Gammaproteobacteria;o__Enterobacterales;f__Erwiniaceae;g__Pantoea;s__Pantoea_deleyi
MDJ-2_gene_2971	147	ORM77230.1	117	114/117(97.44)	4.97e-77	hypothetical protein HA42_18070 [Pantoea deleyi]	470932	d__Bacteria;k__norank;p__Proteobacteria;c__Gammaproteobacteria;o__Enterobacterales;f__Erwiniaceae;g__Pantoea;s__Pantoea_deleyi
MDJ-2_gene_2986	99	SIR58666.1	98	98/98(100.00)	7.67e-60	DNA-binding protein Fis [Enterobacter kobei]	208224	d__Bacteria;k__norank;p__Proteobacteria;c__Gammaproteobacteria;o__Enterobacterales;f__Enterobacteriaceae;g__Enterobacter;s__Enterobacter_kobei
MDJ-2_gene_3000	81	RKP18789.1	153	53/76(69.74)	2.77e-29	Glyoxalase/Bleomycin resistance protein/Dihydroxybiphenyl dioxygenase [Rozella allomycis CSF55]	988480	d__Eukaryota;k__Fungi;p__Cryptomycota;c__norank;o__norank;f__norank;g__Rozella;s__Rozella_allomycis
MDJ-2_gene_3003	112	TCW44310.1	137	95/110(86.36)	1.66e-61	HxlR family transcriptional regulator [Phytobacter	395631	d__Bacteria;k__norank;p__Proteobacteria;c__Gammaproteobacteria;o__Enterobacterales;f__Enterobacteriaceae;g__Phytobacter;s__Phytobacter_diazotrophicus
MDJ-2_gene_3005	34	OGV03892.1	72	29/33(87.88)	7.13e-11	phosphoglycerate kinase, partial [Ignavibacteria bacterium RIFOXYB2_FULL_36_7]	1798447	d__Bacteria;k__norank;p__Ignavibacteriae;c__Ignavibacteria;o__norank;f__norank;g__norank;s__Ignavibacteria_bacterium_RIFOXYB2_FULL_36_7
MDJ-2_gene_3006	165	WP_095700281.1	306	76/103(73.79)	6.64e-41	NAD-dependent epimerase/dehydratase family protein [Pectobacterium polaris]	2042057	d__Bacteria;k__norank;p__Proteobacteria;c__Gammaproteobacteria;o__Enterobacterales;f__Pectobacteriaceae;g__Pectobacterium;s__Pectobacterium_polaris
MDJ-2_gene_3009	247	WP_107319856.1	3822	225/249(90.36)	1.04e-120	filamentous hemagglutinin N-terminal domain-containing protein [Pantoea vagans]	470934	d__Bacteria;k__norank;p__Proteobacteria;c__Gammaproteobacteria;o__Enterobacterales;f__Erwiniaceae;g__Pantoea;s__Pantoea_vagans
MDJ-2_gene_3015	61	OAQ29825.1	742	30/56(53.57)	1.72e-12	hypothetical protein K457DRAFT_137707 [Mortierella elongata AG-77]	1314771	d__Eukaryota;k__Fungi;p__Mucoromycota;c__Mortierellomycetes;o__Mortierellales;f__Mortierellaceae;g__Linnemannia;s__Linnemannia_elongata
MDJ-2_gene_3026	125	PIA15235.1	440	61/114(53.51)	2.01e-37	hypothetical protein COEREDRAFT_103061 [Coemansia reversa NRRL 1564]	763665	d__Eukaryota;k__Fungi;p__Zoopagomycota;c__Kickxellomycetes;o__Kickxellales;f__Kickxellaceae;g__Coemansia;s__Coemansia_reversa
MDJ-2_gene_3027	104	ORM79326.1	104	93/104(89.42)	4.33e-25	colicin V synthesis protein [Pantoea deleyi]	470932	d__Bacteria;k__norank;p__Proteobacteria;c__Gammaproteobacteria;o__Enterobacterales;f__Erwiniaceae;g__Pantoea;s__Pantoea_deleyi
MDJ-2_gene_3033	64	WP_111142243.1	64	64/64(100.00)	8.78e-39	hypothetical protein [Pantoea sp. ARC607]	2027922	d__Bacteria;k__norank;p__Proteobacteria;c__Gammaproteobacteria;o__Enterobacterales;f__Erwiniaceae;g__Pantoea;s__Pantoea_sp._ARC607
MDJ-2_gene_3039	107	XP_007382294.1	1620	76/106(71.70)	6.37e-49	hypothetical protein PUNSTDRAFT_125473 [Punctularia strigosozonata HHB-	741275	d__Eukaryota;k__Fungi;p__Basidiomycota;c__Agaricomycetes;o__Corticiales;f__Punctulariaceae;g__Punctularia;s__Punctularia_strigosozonata
MDJ-2_gene_3049	97	RDD34694.1	104	83/89(93.26)	1.21e-50	ankyrin repeat domain-containing protein [Wolbachia endosymbiont of Cylisticus convexus]	118728	d__Bacteria;k__norank;p__Proteobacteria;c__Alphaproteobacteria;o__Rickettsiales;f__Anaplasmataceae;g__Wolbachia;s__Wolbachia_endosymbiont_of_Cylisticus_convexus
MDJ-2_gene_3050	154	WP_065095079.1	160	128/151(84.77)	4.36e-59	MULTISPECIES: ankyrin repeat domain-containing protein [Wolbachia]	2640676	d__Bacteria;k__norank;p__Proteobacteria;c__Alphaproteobacteria;o__Rickettsiales;f__Anaplasmataceae;g__Wolbachia;s__norank
MDJ-2_gene_3059	226	ORM82562.1	226	224/225(99.56)	2.41e-154	two-component system response regulator BasR [Pantoea deleyi]	470932	d__Bacteria;k__norank;p__Proteobacteria;c__Gammaproteobacteria;o__Enterobacterales;f__Erwiniaceae;g__Pantoea;s__Pantoea_deleyi
MDJ-2_gene_3068	76	ORM75644.1	76	71/76(93.42)	2.35e-46	cation transport regulator [Pantoea deleyi]	470932	d__Bacteria;k__norank;p__Proteobacteria;c__Gammaproteobacteria;o__Enterobacterales;f__Erwiniaceae;g__Pantoea;s__Pantoea_deleyi

MDJ-2_gene_3094	112	RYA69285.1	118	39/101(38.61)	1.89e-13	hypothetical protein DD595_25480, partial [Enterobacter cloacae complex	2511990	d__Bacteria;k__norank;p__Proteobacteria;c__Gammaproteobacteria;o__Enterobacterales;f__Enterobacteriaceae;g__Enterobacter;s__Enterobacter_cloacae_complex_sp._4DZ3-17B2
MDJ-2_gene_3129	65	BBA85126.1	69	37/54(68.52)	1.22e-10	50S ribosomal protein L35 [endosymbiont of Rhynchophorus ferrugineus]	1972133	d__Bacteria;k__norank;p__Proteobacteria;c__Gammaproteobacteria;o__norank;f__norank;g__Candidatus_Nardonella;s__Candidatus_Nardonella_dryophthoridicola
MDJ-2_gene_3143	231	AGJ98981.1	231	220/231(95.24)	5.03e-154	putative methyltransferase [Wolbachia endosymbiont of Drosophila simulans wNo]	1236908	d__Bacteria;k__norank;p__Proteobacteria;c__Alphaproteobacteria;o__Rickettsiales;f__Anaplasmataceae;g__Wolbachia;s__Wolbachia_endosymbiont_of_Drosophila_simulans
MDJ-2_gene_3185	79	WP_110883467.1	655	41/68(60.29)	4.07e-13	beta-galactosidase, partial [Gammaproteobacteria bacterium 2W06]	None	Unclassified
MDJ-2_gene_3196	172	ORM86591.1	172	172/172(100.00)	4.41e-128	flavodoxin FldB [Pantoea deleyi]	470932	d__Bacteria;k__norank;p__Proteobacteria;c__Gammaproteobacteria;o__Enterobacterales;f__Erwiniaceae;g__Pantoea;s__Pantoea_deleyi
MDJ-2_gene_3217	69	AJQ52466.1	438	52/68(76.47)	7.51e-18	hypothetical protein UQ52_07485 (plasmid)	369822	d__Bacteria;k__norank;p__Proteobacteria;c__Alphaproteobacteria;o__Rickettsiales;f__Rickettsiaceae;g__Rickettsia;s__Rickettsia_raoultii
MDJ-2_gene_3220	84	OAQ32889.1	269	45/83(54.22)	1.56e-23	tetrapyrrole biosynthesis, porphobilinogen synthase [Mortierella elongata AG-77]	1314771	d__Eukaryota;k__Fungi;p__Mucoromycota;c__Mortierellomycetes;o__Mortierellales;f__Mortierellaceae;g__Linnemannia;s__Linnemannia_elongata
MDJ-2_gene_3234	92	EPJ11528.1	184	52/58(89.66)	9.99e-29	transposase-like protein, partial [Salmonella enterica subsp. enterica serovar Enteritidis str.	1192593	d__Bacteria;k__norank;p__Proteobacteria;c__Gammaproteobacteria;o__Enterobacterales;f__Enterobacteriaceae;g__Salmonella;s__Salmonella_enterica
MDJ-2_gene_3240	104	KIE64126.1	446	45/101(44.55)	4.78e-15	Preprotein translocase secY subunit [Candidatus Riesia pediculischaeffi PTSU]	1401651	d__Bacteria;k__norank;p__Proteobacteria;c__Gammaproteobacteria;o__Enterobacterales;f__Enterobacteriaceae;g__Candidatus_Riesia;s__Candidatus_Riesia_pediculischaeffi
MDJ-2_gene_3241	76	BBA85244.1	116	44/72(61.11)	3.63e-21	30S ribosomal protein S13 [endosymbiont of Sipalinus gigas]	1972134	d__Bacteria;k__norank;p__Proteobacteria;c__Gammaproteobacteria;o__norank;f__norank;g__Candidatus_Nardonella;s__endosymbiont_of_Sipalinus_gigas
MDJ-2_gene_3247	97	ORM82909.1	247	83/87(95.40)	2.07e-55	hypothetical protein HA42_07210 [Pantoea deleyi]	470932	d__Bacteria;k__norank;p__Proteobacteria;c__Gammaproteobacteria;o__Enterobacterales;f__Erwiniaceae;g__Pantoea;s__Pantoea_deleyi
MDJ-2_gene_3252	108	WP_046288717.1	123	73/108(67.59)	1.05e-44	50S ribosomal protein L14 [Blochmannia endosymbiont of Polyrhachis (Hedomyrma)	1505596	d__Bacteria;k__norank;p__Proteobacteria;c__Gammaproteobacteria;o__Enterobacterales;f__Enterobacteriaceae;g__Candidatus_Blochmannia;s__Blochmannia_endosymbiont_of_Polyrhachis_(Hedomyrma)_turneri
MDJ-2_gene_3253	112	WP_075432244.1	104	52/101(51.49)	8.99e-22	50S ribosomal protein L24 [Buchnera aphidicola]	9	d__Bacteria;k__norank;p__Proteobacteria;c__Gammaproteobacteria;o__Enterobacterales;f__Erwiniaceae;g__Buchnera;s__Buchnera_aphidicola
MDJ-2_gene_3313	193	ORM81041.1	191	191/191(100.00)	5.43e-132	hypothetical protein HA42_12045 [Pantoea deleyi]	470932	d__Bacteria;k__norank;p__Proteobacteria;c__Gammaproteobacteria;o__Enterobacterales;f__Erwiniaceae;g__Pantoea;s__Pantoea_deleyi
MDJ-2_gene_3322	115	OQV43680.1	115	115/115(100.00)	1.60e-71	succinate dehydrogenase, hydrophobic membrane anchor protein [Pantoea vagans]	470934	d__Bacteria;k__norank;p__Proteobacteria;c__Gammaproteobacteria;o__Enterobacterales;f__Erwiniaceae;g__Pantoea;s__Pantoea_vagans
MDJ-2_gene_3323	140	ADO08749.1	146	130/133(97.74)	1.11e-82	succinate dehydrogenase cytochrome b-556 subunit [Pantoea vagans C9-1]	712898	d__Bacteria;k__norank;p__Proteobacteria;c__Gammaproteobacteria;o__Enterobacterales;f__Erwiniaceae;g__Pantoea;s__Pantoea_vagans
MDJ-2_gene_3326	110	BBA85160.1	373	53/112(47.32)	1.95e-18	DNA polymerase III subunit beta [endosymbiont of Sipalinus gigas]	1972134	d__Bacteria;k__norank;p__Proteobacteria;c__Gammaproteobacteria;o__norank;f__norank;g__Candidatus_Nardonella;s__endosymbiont_of_Sipalinus_gigas
MDJ-2_gene_3331	181	SKA25826.1	181	181/181(100.00)	5.28e-129	transcription antitermination protein nusG [Pantoea	470933	d__Bacteria;k__norank;p__Proteobacteria;c__Gammaproteobacteria;o__Enterobacterales;f__Erwiniaceae;g__Pantoea;s__Pantoea_eucalypti
MDJ-2_gene_3334	130	OFI46749.1	288	87/129(67.44)	2.58e-54	hypothetical protein BG262_02830 [Floricoccus	1859475	d__Bacteria;k__norank;p__Firmicutes;c__Bacilli;o__Lactobacillales;f__Streptococcaceae;g__Floricoccus;s__Floricoccus_penangensis
MDJ-2_gene_3335	144	WP_109990913.1	184	89/144(61.81)	3.86e-48	hypothetical protein [Lactococcus lactis]	1358	d__Bacteria;k__norank;p__Firmicutes;c__Bacilli;o__Lactobacillales;f__Streptococcaceae;g__Lactococcus;s__Lactococcus_lactis

MDJ-2_gene_3337	145	WP_094119125.1	298	118/142(83.10)	1.02e-75	phage head morphogenesis protein [Pantoea conspicua]	472705	d__Bacteria;k__norank;p__Proteobacteria;c__Gammaproteobacteria;o__Enterobacterales;f__Erwiniaceae;g__Pantoea;s__Pantoea_conspicua
MDJ-2_gene_3340	162	WP_125711331.1	196	70/162(43.21)	1.39e-32	GNAT family acetyltransferase [Lactobacillus sp. M1575]	2486009	d__Bacteria;k__norank;p__Firmicutes;c__Bacilli;o__Lactobacillales;f__Lactobacillaceae;g__Companilactobacillus;s__Lactobacillus_zhongbaensis
MDJ-2_gene_3349	246	ORM82462.1	246	242/246(98.37)	2.05e-165	2-dehydro-3-deoxyphosphooctonate aldolase	470932	d__Bacteria;k__norank;p__Proteobacteria;c__Gammaproteobacteria;o__Enterobacterales;f__Erwiniaceae;g__Pantoea;s__Pantoea_deleyi
MDJ-2_gene_3375	134	WP_127110899.1	284	62/95(65.26)	1.82e-27	MULTISPECIES: hypothetical protein [Rhodobacteraceae]	2497945	d__Bacteria;k__norank;p__Proteobacteria;c__Alphaproteobacteria;o__Rhodobacterales;f__Rhodobacteraceae;g__Thalassobius;s__Thalassobius_sp._ZQ172
MDJ-2_gene_3395	158	WP_104085959.1	396	155/158(98.10)	9.48e-97	MULTISPECIES: SfnB family sulfur acquisition oxidoreductase	2630326	d__Bacteria;k__norank;p__Proteobacteria;c__Gammaproteobacteria;o__Enterobacterales;f__Erwiniaceae;g__Pantoea;s__norank
MDJ-2_gene_3396	104	WP_104085958.1	403	104/104(100.00)	3.60e-62	MULTISPECIES: SfnB family sulfur acquisition oxidoreductase	2630326	d__Bacteria;k__norank;p__Proteobacteria;c__Gammaproteobacteria;o__Enterobacterales;f__Erwiniaceae;g__Pantoea;s__norank
MDJ-2_gene_3400	100	GBC05036.1	420	35/98(35.71)	1.08e-06	hypothetical protein RclHR1_00060049 [Rhizophagus clarus]	94130	d__Eukaryota;k__Fungi;p__Mucoromycota;c__Glomeromycetes;o__Glomerales;f__Glomeraceae;g__Rhizophagus;s__Rhizophagus_clarus
MDJ-2_gene_3415	253	ORM86802.1	250	247/250(98.80)	2.34e-176	copper homeostasis protein CutC [Pantoea deleyi]	470932	d__Bacteria;k__norank;p__Proteobacteria;c__Gammaproteobacteria;o__Enterobacterales;f__Erwiniaceae;g__Pantoea;s__Pantoea_deleyi
MDJ-2_gene_3424	83	KTR90866.1	144	37/59(62.71)	7.48e-18	hypothetical protein SA2_09470 [Pantoea dispersa]	59814	d__Bacteria;k__norank;p__Proteobacteria;c__Gammaproteobacteria;o__Enterobacterales;f__Erwiniaceae;g__Pantoea;s__Pantoea_dispersa
MDJ-2_gene_3428	145	WP_115098343.1	141	69/132(52.27)	6.83e-40	recombination protein NinB [Pasteurella multocida]	747	d__Bacteria;k__norank;p__Proteobacteria;c__Gammaproteobacteria;o__Pasteurellales;f__Pasteurellaceae;g__Pasteurella;s__Pasteurella_multocida
MDJ-2_gene_3429	106	WP_105626981.1	445	73/99(73.74)	4.46e-41	DNA helicase [Cronobacter malonaticus]	413503	d__Bacteria;k__norank;p__Proteobacteria;c__Gammaproteobacteria;o__Enterobacterales;f__Enterobacteriaceae;g__Cronobacter;s__Cronobacter_malonaticus
MDJ-2_gene_3468	158	WP_114445228.1	154	79/152(51.97)	8.55e-47	HK97 gp10 family phage protein [Hafnia paralvei]	546367	d__Bacteria;k__norank;p__Proteobacteria;c__Gammaproteobacteria;o__Enterobacterales;f__Hafniaceae;g__Hafnia;s__Hafnia_paralvei
MDJ-2_gene_3469	53	ARK07759.1	113	33/52(63.46)	9.38e-17	hypothetical protein LPST10_00027 [Salmonella	1973454	d__Viruses;k__norank;p__Uroviricota;c__Caudoviricetes;o__Caudovirales;f__Siphoviridae;g__norank;s__Salmonella_phage_LPST10
MDJ-2_gene_3481	83	CUX95857.1	466	38/83(45.78)	1.96e-11	Asparagine--tRNA ligase [Candidatus Mikella endobia]	1778264	d__Bacteria;k__norank;p__Proteobacteria;c__Gammaproteobacteria;o__Enterobacterales;f__Enterobacteriaceae;g__Candidatus_Mikella;s__Candidatus_Mikella_endobia
MDJ-2_gene_3484	70	RUM30459.1	600	28/56(50.00)	2.94e-08	hypothetical protein DSY42_04585 [Aquifex sp.]	313370	d__Bacteria;k__norank;p__Aquificae;c__Aquificae;o__Aquificales;f__Aquificaceae;g__Aquifex;s__Aquifex_sp.
MDJ-2_gene_3488	260	BBA85134.1	362	76/136(55.88)	2.50e-36	membrane-bound lytic murein transglycosylase C [endosymbiont of Rhynchophorus ferrugineus]	1972133	d__Bacteria;k__norank;p__Proteobacteria;c__Gammaproteobacteria;o__norank;f__norank;g__Candidatus_Nardonella;s__Candidatus_Nardonella_dryophthoridicola
MDJ-2_gene_3490	260	OMH84444.1	1167	63/214(29.44)	3.62e-14	Retrovirus-related Pol polyprotein from transposon [Zancudomyces culisetae]	1213189	d__Eukaryota;k__Fungi;p__Zoopagomycota;c__Harpellomycetes;o__Harpellales;f__Legeriomycetaceae;g__Zancudomyces;s__Zancudomyces_culisetae
MDJ-2_gene_3493	180	ORM75717.1	175	157/167(94.01)	9.52e-110	glycosyltransferase [Pantoea deleyi]	470932	d__Bacteria;k__norank;p__Proteobacteria;c__Gammaproteobacteria;o__Enterobacterales;f__Erwiniaceae;g__Pantoea;s__Pantoea_deleyi
MDJ-2_gene_3512	56	BBA84936.1	319	33/57(57.89)	3.00e-09	tRNA dimethylallyltransferase [endosymbiont of Pachyrhynchus infernalis]	1971488	d__Bacteria;k__norank;p__Proteobacteria;c__Gammaproteobacteria;o__norank;f__norank;g__Candidatus_Nardonella;s__endosymbiont_of_Pachyrhynchus_infernalis
MDJ-2_gene_3556	254	RYE27129.1	1122	98/253(38.74)	4.85e-50	hypothetical protein EOP45_02210	2021370	d__Bacteria;k__norank;p__Bacteroidetes;c__Sphingobacteriia;o__Sphingobacteriales;f__Sphingobacteriaceae;g__norank;s__Sphingobacteriaceae_bacterium
MDJ-2_gene_3564	80	WP_045140620.1	92	36/45(80.00)	3.08e-14	hypothetical protein [Pantoea agglomerans]	None	Unclassified
MDJ-2_gene_3574	243	RUM30001.1	189	66/183(36.07)	5.17e-29	hypothetical protein DSY42_05315 [Aquifex sp.]	313370	d__Bacteria;k__norank;p__Aquificae;c__Aquificae;o__Aquificales;f__Aquificaceae;g__Aquifex;s__Aquifex_sp.
MDJ-2_gene_3579	70	SKA80418.1	70	70/70(100.00)	2.11e-44	cold shock protein (beta-ribbon, CspA family) [Pantoea ananatis]	553	d__Bacteria;k__norank;p__Proteobacteria;c__Gammaproteobacteria;o__Enterobacterales;f__Erwiniaceae;g__Pantoea;s__Pantoea_ananatis

MDJ-2_gene_3591	112	AAX14415.1	172	104/112(92.86)	6.49e-71	GTP-binding protein YchF [Wolbachia endosymbiont of Drosophila mojavensis]	307503	d__Bacteria;k__norank;p__Proteobacteria;c__Alphaproteobacteria;o__Rickettsiales;f__Anaplasmataceae;g__Wolbachia;s__Wolbachia_endosymbiont_of_Drosophila_mojavensis
MDJ-2_gene_3634	59	ORM51312.1	59	59/59(100.00)	1.13e-32	50S ribosomal protein L30 [Pantoea conspicua]	472705	d__Bacteria;k__norank;p__Proteobacteria;c__Gammaproteobacteria;o__Enterobacterales;f__Erwiniaceae;g__Pantoea;s__Pantoea_conspicua
MDJ-2_gene_3638	104	ORM86298.1	104	102/104(98.08)	3.14e-66	cation tolerance protein CutA [Pantoea deleyi]	470932	d__Bacteria;k__norank;p__Proteobacteria;c__Gammaproteobacteria;o__Enterobacterales;f__Erwiniaceae;g__Pantoea;s__Pantoea_deleyi
MDJ-2_gene_3647	67	WP_114516972.1	67	66/67(98.51)	1.16e-42	hypothetical protein [Wolbachia endosymbiont of Cylisticus convexus]	118728	d__Bacteria;k__norank;p__Proteobacteria;c__Alphaproteobacteria;o__Rickettsiales;f__Anaplasmataceae;g__Wolbachia;s__Wolbachia_endosymbiont_of_Cylisticus_convexus
MDJ-2_gene_3673	99	WP_019236496.1	386	92/99(92.93)	8.50e-61	hypothetical protein [Wolbachia pipientis]	955	d__Bacteria;k__norank;p__Proteobacteria;c__Alphaproteobacteria;o__Rickettsiales;f__Anaplasmataceae;g__Wolbachia;s__Wolbachia_pipientis
MDJ-2_gene_3722	70	PJF18596.1	986	36/62(58.06)	3.62e-15	Oxysterol-binding C2F12.05c-like protein [Paramicrosporidium saccamoebae]	1246581	d__Eukaryota;k__Fungi;p__Cryptomycota;c__norank;o__norank;f__norank;g__Paramicrosporidium;s__Paramicrosporidium_saccamoebae
MDJ-2_gene_3766	47	ORY81083.1	1400	29/34(85.29)	6.85e-13	hypothetical protein LY90DRAFT_500175 [Neocallimastix californiae]	1754190	d__Eukaryota;k__Fungi;p__Chytridiomycota;c__Neocallimastigomycetes;o__Neocallimastigales;f__Neocallimastigaceae;g__Neocallimastix;s__Neocallimastix_californiae
MDJ-2_gene_3771	145	WP_122123645.1	528	45/141(31.91)	2.40e-08	carboxylesterase family protein [Commensalibacter sp. AMU001]	None	Unclassified
MDJ-2_gene_3823	183	ORM85297.1	183	180/183(98.36)	2.07e-136	N-acetyl-anhydromuranmyl-L-alanine amidase [Pantoea deleyi]	470932	d__Bacteria;k__norank;p__Proteobacteria;c__Gammaproteobacteria;o__Enterobacterales;f__Erwiniaceae;g__Pantoea;s__Pantoea_deleyi
MDJ-2_gene_3827	95	WP_095706374.1	106	69/77(89.61)	4.98e-43	hypothetical protein [Pantoea vagans]	None	Unclassified
MDJ-2_gene_3851	71	ORY52597.1	1688	32/60(53.33)	1.09e-15	hypothetical protein BCR33DRAFT_711869 [Rhizoclosmatium globosum]	329046	d__Eukaryota;k__Fungi;p__Chytridiomycota;c__Chytridiomycetes;o__Chytridiales;f__Chytriomycetaceae;g__Rhizoclosmatium;s__Rhizoclosmatium_globosum
MDJ-2_gene_3877	84	ORM84517.1	84	84/84(100.00)	7.11e-56	glutaredoxin 3 [Pantoea deleyi]	470932	d__Bacteria;k__norank;p__Proteobacteria;c__Gammaproteobacteria;o__Enterobacterales;f__Erwiniaceae;g__Pantoea;s__Pantoea_deleyi
MDJ-2_gene_3881	150	WP_111139729.1	128	109/131(83.21)	2.12e-72	DUF2531 family protein [Pantoea sp. ARC607]	2027922	d__Bacteria;k__norank;p__Proteobacteria;c__Gammaproteobacteria;o__Enterobacterales;f__Erwiniaceae;g__Pantoea;s__Pantoea_sp._ARC607
MDJ-2_gene_3883	88	OUW46808.1	1040	37/64(57.81)	4.35e-13	hypothetical protein CBD41_02085 [bacterium	1986654	d__Bacteria;k__norank;p__norank;c__norank;o__norank;f__norank;g__norank;s__bacterium_TMED181
MDJ-2_gene_3884	228	AXC43037.1	495	200/228(87.72)	1.38e-136	hypothetical protein CPT_Skate_079 [Salmonella	2234035	d__Viruses;k__norank;p__Uroviricota;c__Caudoviricetes;o__Caudovirales;f__Siphoviridae;g__norank;s__Salmonella_phage_Skate
MDJ-2_gene_3885	101	ORM84122.1	84	84/84(100.00)	7.44e-49	transcriptional regulator [Pantoea deleyi]	470932	d__Bacteria;k__norank;p__Proteobacteria;c__Gammaproteobacteria;o__Enterobacterales;f__Erwiniaceae;g__Pantoea;s__Pantoea_deleyi
MDJ-2_gene_3888	132	OAA62703.1	1932	41/75(54.67)	8.43e-22	DNA/RNA helicase [Sporothrix insectorum RCEF 264]	1081102	d__Eukaryota;k__Fungi;p__Ascomycota;c__Sordariomycetes;o__Ophiostomatales;f__Ophiostomataceae;g__Sporothrix;s__Sporothrix_insectorum
MDJ-2_gene_3907	66	WP_111140598.1	66	65/66(98.48)	2.47e-39	MULTISPECIES: YaeP family protein [Pantoea]	53335	d__Bacteria;k__norank;p__Proteobacteria;c__Gammaproteobacteria;o__Enterobacterales;f__Erwiniaceae;g__Pantoea;s__norank
MDJ-2_gene_3916	53	TCZ28779.1	91	50/57(87.72)	1.33e-26	type II toxin-antitoxin system RelE/ParE family toxin [Pantoea agglomerans]	549	d__Bacteria;k__norank;p__Proteobacteria;c__Gammaproteobacteria;o__Enterobacterales;f__Erwiniaceae;g__Pantoea;s__Pantoea_agglomerans
MDJ-2_gene_3946	127	WP_031592017.1	94	84/91(92.31)	2.03e-57	transcriptional regulator [Pantoea agglomerans]	549	d__Bacteria;k__norank;p__Proteobacteria;c__Gammaproteobacteria;o__Enterobacterales;f__Erwiniaceae;g__Pantoea;s__Pantoea_agglomerans
MDJ-2_gene_3948	200	PBQ30164.1	519	192/194(98.97)	2.11e-120	cation:proton antiporter [Wolbachia pipientis wAus]	2049495	d__Bacteria;k__norank;p__Proteobacteria;c__Alphaproteobacteria;o__Rickettsiales;f__Anaplasmataceae;g__Wolbachia;s__Wolbachia_pipientis
MDJ-2_gene_3993	84	ORM85055.1	79	79/79(100.00)	9.13e-49	virulence protein MsgA [Pantoea deleyi]	470932	d__Bacteria;k__norank;p__Proteobacteria;c__Gammaproteobacteria;o__Enterobacterales;f__Erwiniaceae;g__Pantoea;s__Pantoea_deleyi

MDJ-2_gene_3994	88	ORM85056.1	88	88/88(100.00)	2.62e-53	hypothetical protein HA42_03275 [Pantoea deleyi]	470932	d__Bacteria;k__norank;p__Proteobacteria;c__Gammaproteobacteria;o__Enterobacterales;f__Erwiniaceae;g__Pantoea;s__Pantoea_deleyi
MDJ-2_gene_4005	47	WP_111140320.1	588	43/47(91.49)	9.43e-21	bifunctional UDP-sugar hydrolase/5'-nucleotidase [Pantoea sp. ARC607]	2027922	d__Bacteria;k__norank;p__Proteobacteria;c__Gammaproteobacteria;o__Enterobacterales;f__Erwiniaceae;g__Pantoea;s__Pantoea_sp._ARC607
MDJ-2_gene_4016	191	ORM76845.1	184	175/184(95.11)	5.33e-130	GNAT family N-acetyltransferase [Pantoea deleyi]	470932	d__Bacteria;k__norank;p__Proteobacteria;c__Gammaproteobacteria;o__Enterobacterales;f__Erwiniaceae;g__Pantoea;s__Pantoea_deleyi
MDJ-2_gene_4020	39	SUB14837.1	163	39/39(100.00)	9.45e-19	Oligoribonuclease [Pantoea agglomerans]	549	d__Bacteria;k__norank;p__Proteobacteria;c__Gammaproteobacteria;o__Enterobacterales;f__Erwiniaceae;g__Pantoea;s__Pantoea_agglomerans
MDJ-2_gene_4033	201	BBA84809.1	254	70/175(40.00)	1.05e-24	cell shape-determining protein MreC [endosymbiont of Pachyrhynchus infernalis]	1971488	d__Bacteria;k__norank;p__Proteobacteria;c__Gammaproteobacteria;o__norank;f__norank;g__Candidatus_Nardonella;s__endosymbiont_of_Pachyrhynchus_infernalis
MDJ-2_gene_4037	109	CQD11609.1	221	105/109(96.33)	1.04e-72	Phage related DNA methylase [Wolbachia endosymbiont wPip_Mol of Culex molestus]	1402163	d__Bacteria;k__norank;p__Proteobacteria;c__Alphaproteobacteria;o__Rickettsiales;f__Anaplasmataceae;g__Wolbachia;s__Wolbachia_endosymbiont_of_Culex_molestus
MDJ-2_gene_4041	71	WP_129913657.1	171	32/36(88.89)	4.90e-14	hypothetical protein, partial [Gordonia sp. AMA120]	None	Unclassified
MDJ-2_gene_4055	157	ORM82378.1	157	156/157(99.36)	9.13e-116	NUDIX hydrolase [Pantoea deleyi]	470932	d__Bacteria;k__norank;p__Proteobacteria;c__Gammaproteobacteria;o__Enterobacterales;f__Erwiniaceae;g__Pantoea;s__Pantoea_deleyi
MDJ-2_gene_4072	99	XP_009266447.1	1491	33/85(38.82)	4.95e-13	5'-3' exoribonuclease 1 [Wallemia	1299270	d__Eukaryota;k__Fungi;p__Basidiomycota;c__Wallemiomycetes;o__Wallemiales;f__Wallemiaceae;g__Wallemia;s__Wallemia_ichthyophaga
MDJ-2_gene_4096	213	CRK85544.1	676	88/212(41.51)	2.81e-36	DNA ligase [Candidatus Providencia siddallii]	1715285	d__Bacteria;k__norank;p__Proteobacteria;c__Gammaproteobacteria;o__Enterobacterales;f__Morganellaceae;g__Providencia;s__Candidatus_Providencia_siddallii
MDJ-2_gene_4101	107	OOZ43285.1	212	40/87(45.98)	1.06e-17	hypothetical protein BOW38_12490, partial [Solemya	2340	d__Bacteria;k__norank;p__Proteobacteria;c__Gammaproteobacteria;o__norank;f__norank;g__norank;s__Solemya_velum_gill_symbiont
MDJ-2_gene_4111	124	YP_001967397.1	124	112/124(90.32)	9.25e-73	hypothetical protein RM_p17 (plasmid) [Rickettsia monacensis]	None	Unclassified
MDJ-2_gene_4129	73	WP_120633295.1	233	23/47(48.94)	6.78e-07	glutathione S-transferase family protein [Ruegeria sp. EL01]	2107578	d__Bacteria;k__norank;p__Proteobacteria;c__Alphaproteobacteria;o__Rhodobacterales;f__Rhodobacteraceae;g__Ruegeria;s__Ruegeria_sp._EL01
MDJ-2_gene_4132	100	ORM84157.1	100	89/100(89.00)	2.01e-58	hypothetical protein HA42_05225 [Pantoea deleyi]	470932	d__Bacteria;k__norank;p__Proteobacteria;c__Gammaproteobacteria;o__Enterobacterales;f__Erwiniaceae;g__Pantoea;s__Pantoea_deleyi
MDJ-2_gene_4140	85	WP_081600764.1	211	78/78(100.00)	6.16e-51	hypothetical protein [Wolbachia endosymbiont of Drosophila simulans]	None	Unclassified
MDJ-2_gene_4143	119	WP_130616993.1	128	51/116(43.97)	3.40e-15	hypothetical protein [endosymbiont of Sipalinus gigas]	1972134	d__Bacteria;k__norank;p__Proteobacteria;c__Gammaproteobacteria;o__norank;f__norank;g__Candidatus_Nardonella;s__endosymbiont_of_Sipalinus_gigas
MDJ-2_gene_4148	97	ORM78936.1	97	91/97(93.81)	1.82e-55	hypothetical protein HA42_16085 [Pantoea deleyi]	470932	d__Bacteria;k__norank;p__Proteobacteria;c__Gammaproteobacteria;o__Enterobacterales;f__Erwiniaceae;g__Pantoea;s__Pantoea_deleyi
MDJ-2_gene_4155	111	RLI64914.1	235	66/103(64.08)	2.09e-33	ribose 5-phosphate isomerase A [Candidatus Lokiarchaeota archaeon]	2053489	d__Archaea;k__norank;p__Candidatus_Lokiarchaeota;c__norank;o__norank;f__norank;g__norank;s__Candidatus_Lokiarchaeota_archaeon
MDJ-2_gene_4167	61	CUJ22168.1	101	33/50(66.00)	9.81e-12	Uncharacterised protein [Achromobacter sp. 2789STDY5608608]	72557	d__Bacteria;k__norank;p__Proteobacteria;c__Betaproteobacteria;o__Burkholderiales;f__Alcaligenaceae;g__Achromobacter;s__Achromobacter_ruhlandii
MDJ-2_gene_4168	132	OXH76782.1	341	115/126(91.27)	8.41e-65	hypothetical protein CBI57_21860, partial [Pantoea	549	d__Bacteria;k__norank;p__Proteobacteria;c__Gammaproteobacteria;o__Enterobacterales;f__Erwiniaceae;g__Pantoea;s__Pantoea_agglomerans
MDJ-2_gene_4178	205	AXC43032.1	649	174/206(84.47)	3.00e-124	ATP-dependent helicase [Salmonella phage Skate]	2234035	d__Viruses;k__norank;p__Uroviricota;c__Caudoviricetes;o__Caudovirales;f__Siphoviridae;g__norank;s__Salmonella_phage_Skate
MDJ-2_gene_4182	95	RUS25234.1	289	36/89(40.45)	1.65e-08	hypothetical protein BC938DRAFT_472447, partial [Jimgerdemannia flammicorona]	994334	d__Eukaryota;k__Fungi;p__Mucoromycota;c__Endogonomycetes;o__Endogonales;f__Endogonaceae;g__Jimgerdemannia;s__Jimgerdemannia_flammicorona

MDJ-2_gene_4212	139	AJQ52466.1	438	36/67(53.73)	1.07e-06	hypothetical protein UQ52_07485 (plasmid)	369822	d__Bacteria;k__norank;p__Proteobacteria;c__Alphaproteobacteria;o__Rickettsiales;f__Rickettsiaceae;g__Rickettsia;s__Rickettsia_raoultii
MDJ-2_gene_4240	84	OZJ03452.1	702	31/58(53.45)	7.91e-09	hypothetical protein BZG36_02736 [Bifiguratus	1938954	d__Eukaryota;k__Fungi;p__Mucoromycota;c__Endogonomycetes;o__Endogonales;f__norank;g__Bifiguratus;s__Bifiguratus_adelaidae
MDJ-2_gene_4241	58	WP_109990919.1	234	52/58(89.66)	8.54e-29	hypothetical protein [Lactococcus lactis]	1358	d__Bacteria;k__norank;p__Firmicutes;c__Bacilli;o__Lactobacillales;f__Streptococcaceae;g__Lactococcus;s__Lactococcus_lactis
MDJ-2_gene_4242	126	WP_109990918.1	438	119/126(94.44)	4.51e-81	hypothetical protein [Lactococcus lactis]	1358	d__Bacteria;k__norank;p__Firmicutes;c__Bacilli;o__Lactobacillales;f__Streptococcaceae;g__Lactococcus;s__Lactococcus_lactis
MDJ-2_gene_4247	156	STD18135.1	168	82/151(54.30)	1.56e-54	DNA-binding ATP-dependent protease La [Enterobacter	61645	d__Bacteria;k__norank;p__Proteobacteria;c__Gammaproteobacteria;o__Enterobacterales;f__Enterobacteriaceae;g__Enterobacter;s__Enterobacter_asburiae
MDJ-2_gene_4249	52	WP_083198511.1	123	40/52(76.92)	3.54e-18	MULTISPECIES: hypothetical protein [unclassified Rickettsia]	114295	d__Bacteria;k__norank;p__Proteobacteria;c__Alphaproteobacteria;o__Rickettsiales;f__Rickettsiaceae;g__Rickettsia;s__norank
MDJ-2_gene_4279	139	KJW03805.1	214	135/139(97.12)	4.23e-88	parB/RepB/Spo0J family partition domain protein [Rickettsia argasii T170-B]	1268837	d__Bacteria;k__norank;p__Proteobacteria;c__Alphaproteobacteria;o__Rickettsiales;f__Rickettsiaceae;g__Rickettsia;s__Rickettsia_argasii
MDJ-3_gene_8	518	PBQ29190.1	518	510/518(98.46)	0	penicillin-binding protein [Wolbachia pipientis wAus]	2049495	d__Bacteria;k__norank;p__Proteobacteria;c__Alphaproteobacteria;o__Rickettsiales;f__Anaplasmataceae;g__Wolbachia;s__Wolbachia_pipientis
MDJ-3_gene_23	758	OAM06293.1	758	752/758(99.21)	0	polyribonucleotide nucleotidyltransferase [Wolbachia endosymbiont of Dactylopius coccus]	1605993	d__Bacteria;k__norank;p__Proteobacteria;c__Alphaproteobacteria;o__Rickettsiales;f__Anaplasmataceae;g__Wolbachia;s__Wolbachia_endosymbiont_of_Dactylopius_coccus
MDJ-3_gene_30	672	RUP42080.1	672	458/671(68.26)	0	DUF4815 domain-containing protein [Acinetobacter sp.]	472	d__Bacteria;k__norank;p__Proteobacteria;c__Gammaproteobacteria;o__Pseudomonadales;f__Moraxellaceae;g__Acinetobacter;s__Acinetobacter_sp.
MDJ-3_gene_35	116	WP_114517903.1	150	107/116(92.24)	2.71e-57	hypothetical protein [Wolbachia endosymbiont of Cylisticus convexus]	118728	d__Bacteria;k__norank;p__Proteobacteria;c__Alphaproteobacteria;o__Rickettsiales;f__Anaplasmataceae;g__Wolbachia;s__Wolbachia_endosymbiont_of_Cylisticus_convexus
MDJ-3_gene_47	252	OJH31014.1	252	250/252(99.21)	2.90e-168	hypothetical protein Wxf_00388 [Wolbachia endosymbiont of Armadillidium vulgare]	77039	d__Bacteria;k__norank;p__Proteobacteria;c__Alphaproteobacteria;o__Rickettsiales;f__Anaplasmataceae;g__Wolbachia;s__Wolbachia_endosymbiont_of_Armadillidium_vulgare
MDJ-3_gene_49	483	PBQ29230.1	483	475/483(98.34)	0	secretion system protein [Wolbachia pipientis wAus]	2049495	d__Bacteria;k__norank;p__Proteobacteria;c__Alphaproteobacteria;o__Rickettsiales;f__Anaplasmataceae;g__Wolbachia;s__Wolbachia_pipientis
MDJ-3_gene_59	333	WP_108784303.1	454	281/385(72.99)	6.42e-181	ankyrin repeat domain-containing protein [Wolbachia endosymbiont of Bemisia tabaci]	215173	d__Bacteria;k__norank;p__Proteobacteria;c__Alphaproteobacteria;o__Rickettsiales;f__Anaplasmataceae;g__Wolbachia;s__Wolbachia_endosymbiont_of_Bemisia_tabaci
MDJ-3_gene_61	101	WP_065106842.1	328	95/101(94.06)	2.03e-58	aspartate--tRNA ligase, partial [Wolbachia endosymbiont of Nomada ferruginata]	1854761	d__Bacteria;k__norank;p__Proteobacteria;c__Alphaproteobacteria;o__Rickettsiales;f__Anaplasmataceae;g__Wolbachia;s__Wolbachia_endosymbiont_of_Nomada_ferruginata
MDJ-3_gene_67	312	WP_129335241.1	178	53/107(49.53)	6.03e-28	transposase [Enterobacter cloacae complex sp. 2DZ2F20B]	None	Unclassified
MDJ-3_gene_114	146	WP_114517846.1	146	136/146(93.15)	1.13e-88	DUF2924 domain-containing protein [Wolbachia endosymbiont of Cylisticus convexus]	118728	d__Bacteria;k__norank;p__Proteobacteria;c__Alphaproteobacteria;o__Rickettsiales;f__Anaplasmataceae;g__Wolbachia;s__Wolbachia_endosymbiont_of_Cylisticus_convexus
MDJ-3_gene_117	388	WP_064124695.1	387	350/386(90.67)	1.04e-261	phage tail protein [Wolbachia endosymbiont of Dactylopius coccus]	None	Unclassified
MDJ-3_gene_118	249	WP_019236525.1	249	246/249(98.80)	1.76e-172	hypothetical protein [Wolbachia pipientis]	953	d__Bacteria;k__norank;p__Proteobacteria;c__Alphaproteobacteria;o__Rickettsiales;f__Anaplasmataceae;g__Wolbachia;s__norank
MDJ-3_gene_163	242	CAQ54575.1	99	77/79(97.47)	3.46e-42	Hypothetical protein WP0467 [Wolbachia endosymbiont of Culex quinquefasciatus Pel]	570417	d__Bacteria;k__norank;p__Proteobacteria;c__Alphaproteobacteria;o__Rickettsiales;f__Anaplasmataceae;g__Wolbachia;s__Wolbachia_endosymbiont_of_Culex_quinquefasciatus

MDJ-3_gene_168	299	WP_064085375.1	299	299/299(100.00)	4.28e-214	methionyl-tRNA formyltransferase [Wolbachia endosymbiont of Dactylopius	None	Unclassified
MDJ-3_gene_197	275	WP_063630842.1	250	68/89(76.40)	5.57e-33	ankyrin repeat domain-containing protein [Wolbachia endosymbiont of Laodelphax striatella]	368602	d__Bacteria;k__norank;p__Proteobacteria;c__Alphaproteobacteria;o__Rickettsiales;f__Anaplasmataceae;g__Wolbachia;s__Wolbachia_endosymbiont_of_Laodelphax_striatellus
MDJ-3_gene_210	281	WP_064085438.1	271	264/271(97.42)	2.63e-183	30S ribosomal protein S2 [Wolbachia endosymbiont of Dactylopius coccus]	None	Unclassified
MDJ-3_gene_211	281	WP_010407216.1	281	278/281(98.93)	1.83e-190	MULTISPECIES: elongation factor Ts [Wolbachia]	2640676	d__Bacteria;k__norank;p__Proteobacteria;c__Alphaproteobacteria;o__Rickettsiales;f__Anaplasmataceae;g__Wolbachia;s__norank
MDJ-3_gene_214	89	OCA05991.1	92	85/89(95.51)	7.30e-52	hypothetical protein wTpre_312 [Wolbachia endosymbiont of Trichogramma pretiosum]	125593	d__Bacteria;k__norank;p__Proteobacteria;c__Alphaproteobacteria;o__Rickettsiales;f__Anaplasmataceae;g__Wolbachia;s__Wolbachia_endosymbiont_of_Trichogramma_pretiosum
MDJ-3_gene_242	399	WP_082861179.1	2839	396/399(99.25)	2.53e-248	DNA-directed RNA polymerase subunit beta/beta' [Wolbachia endosymbiont of Dactylopius coccus]	None	Unclassified
MDJ-3_gene_253	145	PBQ26888.1	184	143/144(99.31)	6.34e-78	hypothetical protein BTO27_04095 [Wolbachia	2049495	d__Bacteria;k__norank;p__Proteobacteria;c__Alphaproteobacteria;o__Rickettsiales;f__Anaplasmataceae;g__Wolbachia;s__Wolbachia_pipientis
MDJ-3_gene_267	352	WP_077188265.1	343	331/341(97.07)	1.51e-227	S49 family peptidase [Wolbachia pipientis]	None	Unclassified
MDJ-3_gene_277	298	WP_039120656.1	1331	130/285(45.61)	1.78e-85	hypothetical protein [Flavobacterium sp. JRM]	None	Unclassified
MDJ-3_gene_278	222	RLJ22758.1	810	93/226(41.15)	5.54e-49	hypothetical protein DJ031_00290, partial [bacterium endosymbiont of Escarpia	1221601	d__Bacteria;k__norank;p__norank;c__norank;o__norank;f__norank;g__norank;s__bacterium_endosymbiont_of_Escarpia_laminata
MDJ-3_gene_296	223	PCG11082.1	408	223/223(100.00)	4.77e-159	cytochrome b [Wolbachia	None	Unclassified
MDJ-3_gene_300	191	WP_095742649.1	191	158/191(82.72)	1.95e-104	hypothetical protein [Wolbachia endosymbiont of Drosophila simulans]	953	d__Bacteria;k__norank;p__Proteobacteria;c__Alphaproteobacteria;o__Rickettsiales;f__Anaplasmataceae;g__Wolbachia;s__norank
MDJ-3_gene_312	284	RUA07230.1	1765	104/260(40.00)	2.27e-52	hypothetical protein DSY43_00310	1913989	d__Bacteria;k__norank;p__Proteobacteria;c__Gammaproteobacteria;o__norank;f__norank;g__norank;s__Gammaproteobacteria_bacterium
MDJ-3_gene_322	558	WP_108784013.1	476	342/559(61.18)	1.08e-187	hypothetical protein [Wolbachia endosymbiont of Bemisia tabaci]	215173	d__Bacteria;k__norank;p__Proteobacteria;c__Alphaproteobacteria;o__Rickettsiales;f__Anaplasmataceae;g__Wolbachia;s__Wolbachia_endosymbiont_of_Bemisia_tabaci
MDJ-3_gene_324	516	OAM03955.1	596	437/456(95.83)	2.75e-314	hypothetical protein TV41_04265 [Wolbachia endosymbiont of Dactylopius coccus]	1605993	d__Bacteria;k__norank;p__Proteobacteria;c__Alphaproteobacteria;o__Rickettsiales;f__Anaplasmataceae;g__Wolbachia;s__Wolbachia_endosymbiont_of_Dactylopius_coccus
MDJ-3_gene_325	353	OJH30288.1	199	87/117(74.36)	1.25e-42	hypothetical protein Wxf_03222 [Armadillidium vulgare]	77039	d__Bacteria;k__norank;p__Proteobacteria;c__Alphaproteobacteria;o__Rickettsiales;f__Anaplasmataceae;g__Wolbachia;s__Wolbachia_endosymbiont_of_Armadillidium_vulgare
MDJ-3_gene_326	97	RDD34750.1	123	86/95(90.53)	8.68e-57	recombinase family protein [Wolbachia endosymbiont of Cylisticus convexus]	118728	d__Bacteria;k__norank;p__Proteobacteria;c__Alphaproteobacteria;o__Rickettsiales;f__Anaplasmataceae;g__Wolbachia;s__Wolbachia_endosymbiont_of_Cylisticus_convexus
MDJ-3_gene_327	102	PCG18184.1	102	102/102(100.00)	7.79e-57	NADH-quinone oxidoreductase subunit NuoK [Wolbachia	None	Unclassified
MDJ-3_gene_369	152	AGJ99347.1	193	129/152(84.87)	3.57e-81	hypothetical protein wNo_09710 [Wolbachia endosymbiont of Drosophila simulans wNo]	1236908	d__Bacteria;k__norank;p__Proteobacteria;c__Alphaproteobacteria;o__Rickettsiales;f__Anaplasmataceae;g__Wolbachia;s__Wolbachia_endosymbiont_of_Drosophila_simulans

MDJ-3_gene_381	798	WP_065095485.1	779	697/797(87.45)	0	MULTISPECIES: phenylalanine--tRNA ligase subunit beta [Wolbachia]	2640676	d__Bacteria;k__norank;p__Proteobacteria;c__Alphaproteobacteria;o__Rickettsiales;f__Anaplasmataceae;g__Wolbachia;s__norank
MDJ-3_gene_384	333	EAL58392.1	570	143/329(43.47)	7.37e-92	SD27140p [Wolbachia endosymbiont of Drosophila	307502	d__Bacteria;k__norank;p__Proteobacteria;c__Alphaproteobacteria;o__Rickettsiales;f__Anaplasmataceae;g__Wolbachia;s__Wolbachia_endosymbiont_of_Drosophila_ananassae
MDJ-3_gene_396	61	RDD34526.1	95	52/61(85.25)	2.28e-30	hypothetical protein Wcon_01372 [Wolbachia endosymbiont of Cylisticus convexus]	118728	d__Bacteria;k__norank;p__Proteobacteria;c__Alphaproteobacteria;o__Rickettsiales;f__Anaplasmataceae;g__Wolbachia;s__Wolbachia_endosymbiont_of_Cylisticus_convexus
MDJ-3_gene_397	87	CCE77113.1	91	84/86(97.67)	6.51e-52	conserved hypothetical protein [Wolbachia pipientis wAlbB]	1116230	d__Bacteria;k__norank;p__Proteobacteria;c__Alphaproteobacteria;o__Rickettsiales;f__Anaplasmataceae;g__Wolbachia;s__Wolbachia_pipientis
MDJ-3_gene_413	474	CEI94880.1	868	54/157(34.39)	3.86e-10	hypothetical protein RMCBS344292_09084 [Rhizopus microsporus]	58291	d__Eukaryota;k__Fungi;p__Mucoromycota;c__Mucoromycetes;o__Mucorales;f__Rhizopodaceae;g__Rhizopus;s__Rhizopus_microsporus
MDJ-3_gene_426	422	WP_064125546.1	637	416/422(98.58)	2.40e-300	DNA helicase II [Wolbachia endosymbiont of Dactylopius coccus]	None	Unclassified
MDJ-3_gene_451	336	WP_010403280.1	336	334/336(99.40)	6.51e-239	tRNA (adenosine(37)-N6)-threonylcarbamoyltransferase complex transferase subunit TsaD [Wolbachia endosymbiont of Nasonia vitripennis]	2640676	d__Bacteria;k__norank;p__Proteobacteria;c__Alphaproteobacteria;o__Rickettsiales;f__Anaplasmataceae;g__Wolbachia;s__norank
MDJ-3_gene_467	590	CAQ55328.1	658	520/653(79.63)	9.67e-202	ankyrin repeat domain protein [Wolbachia endosymbiont of Culex quinquefasciatus Pel]	570417	d__Bacteria;k__norank;p__Proteobacteria;c__Alphaproteobacteria;o__Rickettsiales;f__Anaplasmataceae;g__Wolbachia;s__Wolbachia_endosymbiont_of_Culex_quinquefasciatus
MDJ-3_gene_478	113	RLJ22807.1	885	55/112(49.11)	2.80e-29	hypothetical protein DJ031_00235 [bacterium endosymbiont of Escarpia	1221601	d__Bacteria;k__norank;p__norank;c__norank;o__norank;f__norank;g__norank;s__bacterium_endosymbiont_of_Escarpia_laminata
MDJ-3_gene_482	90	XP_025379919.1	2290	48/83(57.83)	1.98e-21	hypothetical protein FA10DRAFT_3405 [Acaromyces ingoldii]	215250	d__Eukaryota;k__Fungi;p__Basidiomycota;c__Exobasidiomycetes;o__Exobasidiales;f__Cryptobasidiaceae;g__Acaromyces;s__Acaromyces_ingoldii
MDJ-3_gene_488	390	WP_019236337.1	507	380/390(97.44)	6.70e-274	hypothetical protein [Wolbachia pipientis]	955	d__Bacteria;k__norank;p__Proteobacteria;c__Alphaproteobacteria;o__Rickettsiales;f__Anaplasmataceae;g__Wolbachia;s__Wolbachia_pipientis
MDJ-3_gene_491	142	WP_127463574.1	142	141/142(99.30)	2.14e-91	protoporphyrinogen oxidase HemJ [Wolbachia endosymbiont of Bemisia tabaci]	215173	d__Bacteria;k__norank;p__Proteobacteria;c__Alphaproteobacteria;o__Rickettsiales;f__Anaplasmataceae;g__Wolbachia;s__Wolbachia_endosymbiont_of_Bemisia_tabaci
MDJ-3_gene_521	219	WP_064124803.1	216	206/218(94.50)	3.39e-134	Tim44 domain-containing protein [Wolbachia endosymbiont of Dactylopius coccus]	None	Unclassified
MDJ-3_gene_525	538	CAQ54727.1	529	529/529(100.00)	0	glycosyl transferase, group 2 family protein [Wolbachia endosymbiont of Culex	570417	d__Bacteria;k__norank;p__Proteobacteria;c__Alphaproteobacteria;o__Rickettsiales;f__Anaplasmataceae;g__Wolbachia;s__Wolbachia_endosymbiont_of_Culex_quinquefasciatus
MDJ-3_gene_531	349	CCE77805.1	816	348/349(99.71)	3.50e-226	putative multidrug efflux transporter (fragment) [Wolbachia pipientis wAlbB]	1116230	d__Bacteria;k__norank;p__Proteobacteria;c__Alphaproteobacteria;o__Rickettsiales;f__Anaplasmataceae;g__Wolbachia;s__Wolbachia_pipientis
MDJ-3_gene_571	348	AGJ99822.1	344	317/342(92.69)	6.20e-217	Putative minor capsid protein C [Wolbachia endosymbiont of Drosophila simulans wHa]	1236909	d__Bacteria;k__norank;p__Proteobacteria;c__Alphaproteobacteria;o__Rickettsiales;f__Anaplasmataceae;g__Wolbachia;s__Wolbachia_endosymbiont_of_Drosophila_simulans
MDJ-3_gene_572	124	OJH31474.1	123	106/124(85.48)	8.87e-64	hypothetical protein Wxf_00865 [Wolbachia endosymbiont of Armadillidium vulgare]	77039	d__Bacteria;k__norank;p__Proteobacteria;c__Alphaproteobacteria;o__Rickettsiales;f__Anaplasmataceae;g__Wolbachia;s__Wolbachia_endosymbiont_of_Armadillidium_vulgare

MDJ-3_gene_607	291	PCG11342.1	291	287/291(98.63)	2.72e-197	ABC transporter substrate-binding protein [Wolbachia	None	Unclassified
MDJ-3_gene_609	721	WP_113446645.1	542	51/196(26.02)	1.71e-11	hypothetical protein [Escherichia coli]	None	Unclassified
MDJ-3_gene_612	98	PBQ30185.1	98	97/98(98.98)	1.05e-59	DNA-binding protein [Wolbachia pipientis wAus]	2049495	d__Bacteria;k__norank;p__Proteobacteria;c__Alphaproteobacteria;o__Rickettsiales;f__Anaplasmataceae;g__Wolbachia;s__Wolbachia_pipientis
MDJ-3_gene_613	130	PBQ30186.1	130	128/130(98.46)	9.08e-85	DNA-directed RNA polymerase subunit omega [Wolbachia pipientis wAus]	2049495	d__Bacteria;k__norank;p__Proteobacteria;c__Alphaproteobacteria;o__Rickettsiales;f__Anaplasmataceae;g__Wolbachia;s__Wolbachia_pipientis
MDJ-3_gene_614	90	PBQ30188.1	90	89/90(98.89)	2.92e-51	cation:proton antiporter [Wolbachia pipientis wAus]	2049495	d__Bacteria;k__norank;p__Proteobacteria;c__Alphaproteobacteria;o__Rickettsiales;f__Anaplasmataceae;g__Wolbachia;s__Wolbachia_pipientis
MDJ-3_gene_618	748	AGJ99411.1	1002	503/611(82.32)	4.94e-314	Ankyrin repeat domain protein [Wolbachia endosymbiont of Drosophila simulans wNo]	1236908	d__Bacteria;k__norank;p__Proteobacteria;c__Alphaproteobacteria;o__Rickettsiales;f__Anaplasmataceae;g__Wolbachia;s__Wolbachia_endosymbiont_of_Drosophila_simulans
MDJ-3_gene_620	274	WP_064085604.1	274	270/274(98.54)	8.52e-197	50S ribosomal protein L2 [Wolbachia endosymbiont of Dactylopius coccus]	None	Unclassified
MDJ-3_gene_627	329	WP_063630930.1	409	326/329(99.09)	2.60e-215	lipoprotein-releasing ABC transporter permease subunit [Wolbachia endosymbiont of Laodelphax striatella]	368602	d__Bacteria;k__norank;p__Proteobacteria;c__Alphaproteobacteria;o__Rickettsiales;f__Anaplasmataceae;g__Wolbachia;s__Wolbachia_endosymbiont_of_Laodelphax_striatellus
MDJ-3_gene_628	243	PBQ29131.1	243	238/243(97.94)	9.80e-167	hypothetical protein BTO27_01385 [Wolbachia	2049495	d__Bacteria;k__norank;p__Proteobacteria;c__Alphaproteobacteria;o__Rickettsiales;f__Anaplasmataceae;g__Wolbachia;s__Wolbachia_pipientis
MDJ-3_gene_635	125	PCG11867.1	122	115/121(95.04)	1.97e-76	30S ribosomal protein S13 [Wolbachia pipientis]	None	Unclassified
MDJ-3_gene_636	278	AGJ99991.1	909	191/320(59.69)	5.57e-40	Ankyrin repeat domain protein [Wolbachia endosymbiont of Drosophila simulans wHa]	1236909	d__Bacteria;k__norank;p__Proteobacteria;c__Alphaproteobacteria;o__Rickettsiales;f__Anaplasmataceae;g__Wolbachia;s__Wolbachia_endosymbiont_of_Drosophila_simulans
MDJ-3_gene_645	386	RUA05525.1	964	131/383(34.20)	2.20e-48	hypothetical protein DSY43_04445	1913989	d__Bacteria;k__norank;p__Proteobacteria;c__Gammaproteobacteria;o__norank;f__norank;g__norank;s__Gammaproteobacteria_bacterium
MDJ-3_gene_668	295	WP_029237698.1	295	294/295(99.66)	2.34e-200	4-hydroxybenzoate octaprenyltransferase [Wolbachia endosymbiont of Nasonia vitripennis]	180837	d__Bacteria;k__norank;p__Proteobacteria;c__Alphaproteobacteria;o__Rickettsiales;f__Anaplasmataceae;g__Wolbachia;s__Wolbachia_endosymbiont_of_Nasonia_vitripennis
MDJ-3_gene_673	121	CAQ55344.1	121	121/121(100.00)	1.86e-57	preprotein translocase, SecG subunit [Wolbachia endosymbiont of Culex	570417	d__Bacteria;k__norank;p__Proteobacteria;c__Alphaproteobacteria;o__Rickettsiales;f__Anaplasmataceae;g__Wolbachia;s__Wolbachia_endosymbiont_of_Culex_quinquefasciatus
MDJ-3_gene_674	174	WP_010404888.1	174	170/174(97.70)	9.21e-122	MULTISPECIES: gamma carbonic anhydrase family protein [Wolbachia]	180837	d__Bacteria;k__norank;p__Proteobacteria;c__Alphaproteobacteria;o__Rickettsiales;f__Anaplasmataceae;g__Wolbachia;s__Wolbachia_endosymbiont_of_Nasonia_vitripennis
MDJ-3_gene_680	315	WP_019078827.1	888	27/33(81.82)	3.09e-06	MULTISPECIES: hypothetical protein [Wolbachia]	2640676	d__Bacteria;k__norank;p__Proteobacteria;c__Alphaproteobacteria;o__Rickettsiales;f__Anaplasmataceae;g__Wolbachia;s__norank
MDJ-3_gene_683	51	WP_114517903.1	150	31/32(96.88)	1.38e-11	hypothetical protein [Wolbachia endosymbiont of Cylisticus convexus]	118728	d__Bacteria;k__norank;p__Proteobacteria;c__Alphaproteobacteria;o__Rickettsiales;f__Anaplasmataceae;g__Wolbachia;s__Wolbachia_endosymbiont_of_Cylisticus_convexus
MDJ-3_gene_714	118	PCG11796.1	923	111/115(96.52)	2.52e-59	hypothetical protein CO539_01055 [Wolbachia	None	Unclassified
MDJ-3_gene_719	282	RYX83067.1	249	63/223(28.25)	5.90e-23	hypothetical protein EON73_04015 [bacterium]	1869227	d__Bacteria;k__norank;p__norank;c__norank;o__norank;f__norank;g__norank;s__bacterium

MDJ-3_gene_731	146	CCE77944.1	241	134/136(98.53)	8.43e-89	Mannose-1-phosphate guanylyltransferase (fragment) [Wolbachia pipientis wAlbB]	1116230	d__Bacteria;k__norank;p__Proteobacteria;c__Alphaproteobacteria;o__Rickettsiales;f__Anaplasmataceae;g__Wolbachia;s__Wolbachia_pipientis
MDJ-3_gene_732	409	AGJ99039.1	411	361/407(88.70)	1.04e-254	hypothetical protein wNo_06280 [Wolbachia endosymbiont of Drosophila simulans wNo]	1236908	d__Bacteria;k__norank;p__Proteobacteria;c__Alphaproteobacteria;o__Rickettsiales;f__Anaplasmataceae;g__Wolbachia;s__Wolbachia_endosymbiont_of_Drosophila_simulans
MDJ-3_gene_740	298	EAL58741.1	1120	120/294(40.82)	1.64e-62	SD27140p [Wolbachia endosymbiont of Drosophila	307502	d__Bacteria;k__norank;p__Proteobacteria;c__Alphaproteobacteria;o__Rickettsiales;f__Anaplasmataceae;g__Wolbachia;s__Wolbachia_endosymbiont_of_Drosophila_ananassae
MDJ-3_gene_746	158	WP_041581447.1	158	142/158(89.87)	2.14e-101	hypothetical protein [Wolbachia endosymbiont of Drosophila simulans]	77038	d__Bacteria;k__norank;p__Proteobacteria;c__Alphaproteobacteria;o__Rickettsiales;f__Anaplasmataceae;g__Wolbachia;s__Wolbachia_endosymbiont_of_Drosophila_simulans
MDJ-3_gene_747	154	WP_019236416.1	154	142/154(92.21)	1.99e-95	phage baseplate assembly protein V [Wolbachia pipientis]	955	d__Bacteria;k__norank;p__Proteobacteria;c__Alphaproteobacteria;o__Rickettsiales;f__Anaplasmataceae;g__Wolbachia;s__Wolbachia_pipientis
MDJ-3_gene_771	302	PCG11279.1	306	281/306(91.83)	1.97e-185	transposase [Wolbachia pipientis]	None	Unclassified
MDJ-3_gene_801	299	WP_065095187.1	304	278/304(91.45)	3.14e-183	MULTISPECIES: helix-turn-helix domain-containing protein [Wolbachia]	2640676	d__Bacteria;k__norank;p__Proteobacteria;c__Alphaproteobacteria;o__Rickettsiales;f__Anaplasmataceae;g__Wolbachia;s__norank
MDJ-3_gene_804	502	WP_010402890.1	532	499/502(99.40)	0	MULTISPECIES: iron ABC transporter permease [Wolbachia]	180837	d__Bacteria;k__norank;p__Proteobacteria;c__Alphaproteobacteria;o__Rickettsiales;f__Anaplasmataceae;g__Wolbachia;s__Wolbachia_endosymbiont_of_Nasonia_vitripennis
MDJ-3_gene_815	505	WP_010404940.1	503	499/503(99.20)	0	TrbI/VirB10 family protein [Wolbachia endosymbiont of Nasonia vitripennis]	180837	d__Bacteria;k__norank;p__Proteobacteria;c__Alphaproteobacteria;o__Rickettsiales;f__Anaplasmataceae;g__Wolbachia;s__Wolbachia_endosymbiont_of_Nasonia_vitripennis
MDJ-3_gene_829	154	PCG11892.1	155	122/144(84.72)	1.43e-70	hypothetical protein CO539_00630 [Wolbachia	None	Unclassified
MDJ-3_gene_841	458	WP_010403545.1	458	457/458(99.78)	0	UDP-N-acetylmuramoyl-tripeptide--D-alanyl-D-alanine ligase [Wolbachia endosymbiont of Nasonia vitripennis]	180837	d__Bacteria;k__norank;p__Proteobacteria;c__Alphaproteobacteria;o__Rickettsiales;f__Anaplasmataceae;g__Wolbachia;s__Wolbachia_endosymbiont_of_Nasonia_vitripennis
MDJ-3_gene_906	87	BAA89641.1	260	85/87(97.70)	3.46e-51	gp17 [Wolbachia phage WO]	112596	d__Viruses;k__norank;p__Uroviricota;c__Caudoviricetes;o__Caudovirales;f__Myoviridae;g__norank;s__Wolbachia_phage_WO
MDJ-3_gene_913	432	WP_017532641.1	432	418/431(96.98)	4.45e-274	sodium:proton antiporter [Wolbachia endosymbiont of	116598	d__Bacteria;k__norank;p__Proteobacteria;c__Alphaproteobacteria;o__Rickettsiales;f__Anaplasmataceae;g__Wolbachia;s__Wolbachia_endosymbiont_of_Diaphorina_citri
MDJ-3_gene_919	178	XP_001729030.1	563	86/159(54.09)	1.28e-44	hypothetical protein MGL_3818 [Malassezia globosa CBS 7966]	425265	d__Eukaryota;k__Fungi;p__Basidiomycota;c__Malasseziomycetes;o__Malasseziales;f__Malasseziaceae;g__Malassezia;s__Malassezia_globosa
MDJ-3_gene_937	332	WP_064085384.1	332	327/332(98.49)	1.42e-240	tryptophan--tRNA ligase [Wolbachia endosymbiont of Dactylopius coccus]	None	Unclassified
MDJ-3_gene_947	455	PXF62377.1	965	71/275(25.82)	5.59e-20	hypothetical protein DL796_11835, partial [Kangiella	796379	d__Bacteria;k__norank;p__Proteobacteria;c__Gammaproteobacteria;o__Oceanospirillales;f__Kangiellaceae;g__Kangiella;s__Kangiella_spongicola
MDJ-3_gene_972	183	WP_010403418.1	183	182/183(99.45)	9.47e-133	MULTISPECIES: 5-formyltetrahydrofolate cyclo-ligase [Wolbachia]	2640676	d__Bacteria;k__norank;p__Proteobacteria;c__Alphaproteobacteria;o__Rickettsiales;f__Anaplasmataceae;g__Wolbachia;s__norank
MDJ-3_gene_997	113	OAQ22199.1	321	41/109(37.61)	7.60e-10	hypothetical protein K457DRAFT_26290 [Mortierella elongata AG-77]	1314771	d__Eukaryota;k__Fungi;p__Mucoromycota;c__Mortierellomycetes;o__Mortierellales;f__Mortierellaceae;g__Linnemannia;s__Linnemannia_elongata
MDJ-3_gene_1019	86	KNZ48147.1	483	36/65(55.38)	5.32e-16	exodeoxyribonuclease V [Puccinia sorghi]	27349	d__Eukaryota;k__Fungi;p__Basidiomycota;c__Pucciniomycetes;o__Pucciniales;f__Pucciniaceae;g__Puccinia;s__Puccinia_sorghi
MDJ-3_gene_1025	368	XP_023471058.1	390	191/301(63.46)	1.66e-136	Pkinase-domain-containing protein [Rhizopus microsporus	1340429	d__Eukaryota;k__Fungi;p__Mucoromycota;c__Mucoromycetes;o__Mucorales;f__Rhizopodaceae;g__Rhizopus;s__Rhizopus_microsporus

MDJ-3_gene_1114	316	RYE27181.1	1800	87/314(27.71)	1.31e-11	DUF1759 domain-containing protein [Sphingobacteriaceae	2021370	d__Bacteria;k__norank;p__Bacteroidetes;c__Sphingobacteriia;o__Sphingobacteriales;f__Sphingobacteriaceae;g__norank;s__Sphingobacteriaceae_bacterium
MDJ-3_gene_1116	505	AGJ99991.1	909	334/521(64.11)	1.67e-188	Ankyrin repeat domain protein [Wolbachia endosymbiont of Drosophila simulans wHa]	1236909	d__Bacteria;k__norank;p__Proteobacteria;c__Alphaproteobacteria;o__Rickettsiales;f__Anaplasmataceae;g__Wolbachia;s__Wolbachia_endosymbiont_of_Drosophila_simulans
MDJ-3_gene_1164	58	KNE88261.1	117	36/57(63.16)	1.48e-15	hypothetical protein PSTG_18343, partial [Puccinia striiformis f. sp. tritici PST-78]	1165861	d__Eukaryota;k__Fungi;p__Basidiomycota;c__Pucciniomycetes;o__Pucciniales;f__Pucciniaceae;g__Puccinia;s__Puccinia_striiformis
MDJ-3_gene_1193	125	CAD83497.1	267	54/125(43.20)	1.10e-25	exodeoxyribonuclease III [Candidatus Blochmannia	203907	d__Bacteria;k__norank;p__Proteobacteria;c__Gammaproteobacteria;o__Enterobacterales;f__Enterobacteriaceae;g__Candidatus_Blochmannia;s__Candidatus_Blochmannia_floridanus
MDJ-3_gene_1198	75	WP_019236863.1	75	74/75(98.67)	2.79e-40	hypothetical protein [Wolbachia pipientis]	None	Unclassified
MDJ-3_gene_1207	90	WP_010402759.1	92	87/90(96.67)	8.21e-56	MULTISPECIES: 30S ribosomal protein S18 [Wolbachia]	180837	d__Bacteria;k__norank;p__Proteobacteria;c__Alphaproteobacteria;o__Rickettsiales;f__Anaplasmataceae;g__Wolbachia;s__Wolbachia_endosymbiont_of_Nasonia_vitripennis
MDJ-3_gene_1210	231	BBA85203.1	236	115/224(51.34)	1.06e-54	ribonuclease 3 [endosymbiont of Sipalinus gigas]	1972134	d__Bacteria;k__norank;p__Proteobacteria;c__Gammaproteobacteria;o__norank;f__norank;g__Candidatus_Nardonella;s__endosymbiont_of_Sipalinus_gigas
MDJ-3_gene_1239	452	PCG11212.1	446	443/446(99.33)	9.38e-303	DNA recombination protein RmuC [Wolbachia pipientis]	None	Unclassified
MDJ-3_gene_1297	82	Q89AU9.1	939	40/81(49.38)	1.10e-14	RecName: Full=Isoleucine--tRNA ligase; AltName: Full=Isoleucyl-tRNA synthetase;	224915	d__Bacteria;k__norank;p__Proteobacteria;c__Gammaproteobacteria;o__Enterobacterales;f__Erwiniaceae;g__Buchnera;s__Buchnera_aphidicola
MDJ-3_gene_1313	130	PCG11165.1	133	128/130(98.46)	3.36e-83	cytochrome c maturation protein CcmE [Wolbachia pipientis]	None	Unclassified
MDJ-3_gene_1324	228	QBK47218.1	683	92/228(40.35)	6.20e-52	glycoprotein precursor [Anopheles triannulatus	2546222	d__Viruses;k__Orthornavirae;p__Negarnaviricota;c__Ellioviricetes;o__Bunyavirales;f__Phasmaviridae;g__Orthophasmavirus;s__Anopheles_orthophasmavirus
MDJ-3_gene_1346	351	RYE04584.1	799	94/338(27.81)	8.68e-23	reverse transcriptase family protein, partial [Rickettsiaceae	2023360	d__Bacteria;k__norank;p__Proteobacteria;c__Alphaproteobacteria;o__Rickettsiales;f__Rickettsiaceae;g__norank;s__Rickettsiaceae_bacterium
MDJ-3_gene_1348	318	WP_115955873.1	505	176/306(57.52)	2.67e-110	leucyl aminopeptidase [Candidatus Purcelliella	472834	d__Bacteria;k__norank;p__Proteobacteria;c__Gammaproteobacteria;o__Enterobacterales;f__Enterobacteriaceae;g__Candidatus_Purcelliella;s__Candidatus_Purcelliella_pentastirinorum
MDJ-3_gene_1362	86	GAF42065.1	252	33/65(50.77)	1.32e-09	short-chain dehydrogenase/reductase SDR [Lactobacillus composti DSM 18527 = JCM 14202]	1423734	d__Bacteria;k__norank;p__Firmicutes;c__Bacilli;o__Lactobacillales;f__Lactobacillaceae;g__Agrilactobacillus;s__Agrilactobacillus_composti
MDJ-3_gene_1366	218	PCG11020.1	202	195/201(97.01)	1.78e-146	superoxide dismutase [Wolbachia pipientis]	None	Unclassified
MDJ-3_gene_1382	71	QBB83608.1	774	66/71(92.96)	1.61e-31	ankyrin repeat domain-containing protein [Wolbachia pipientis	1116230	d__Bacteria;k__norank;p__Proteobacteria;c__Alphaproteobacteria;o__Rickettsiales;f__Anaplasmataceae;g__Wolbachia;s__Wolbachia_pipientis
MDJ-3_gene_1388	408	QBB83290.1	411	389/408(95.34)	6.65e-276	MFS transporter [Wolbachia pipientis wAlbB]	1116230	d__Bacteria;k__norank;p__Proteobacteria;c__Alphaproteobacteria;o__Rickettsiales;f__Anaplasmataceae;g__Wolbachia;s__Wolbachia_pipientis
MDJ-3_gene_1437	400	AGJ98858.1	400	385/400(96.25)	1.33e-268	kap family p-loop domain protein [Wolbachia endosymbiont of Drosophila simulans wNo]	1236908	d__Bacteria;k__norank;p__Proteobacteria;c__Alphaproteobacteria;o__Rickettsiales;f__Anaplasmataceae;g__Wolbachia;s__Wolbachia_endosymbiont_of_Drosophila_simulans
MDJ-3_gene_1462	54	POY71877.1	882	28/49(57.14)	3.53e-10	hypothetical protein BMF94_5238 [Rhodotorula	741276	d__Eukaryota;k__Fungi;p__Basidiomycota;c__Microbotryomycetes;o__Sporidiobolales;f__Sporidiobolaceae;g__Rhodotorula;s__Rhodotorula_taiwanensis
MDJ-3_gene_1483	125	WP_026092685.1	214	123/125(98.40)	1.38e-80	tRNA (guanosine(46)-N7)-methyltransferase TrmB [Wolbachia endosymbiont of	116598	d__Bacteria;k__norank;p__Proteobacteria;c__Alphaproteobacteria;o__Rickettsiales;f__Anaplasmataceae;g__Wolbachia;s__Wolbachia_endosymbiont_of_Diaphorina_citri
MDJ-3_gene_1485	311	AHK60937.1	218	72/206(34.95)	8.02e-19	nucleocapsid protein, partial [Karimabad virus]	415382	d__Viruses;k__Orthornavirae;p__Negarnaviricota;c__Ellioviricetes;o__Bunyavirales;f__Phenuiviridae;g__Phlebovirus;s__Karimabad_phlebovirus
MDJ-3_gene_1503	113	RVD81571.1	392	34/56(60.71)	1.24e-12	hypothetical protein DFL_009429 [Duddingtonia flagrans]	97331	d__Eukaryota;k__Fungi;p__Ascomycota;c__Orbiliomycetes;o__Orbiliales;f__Orbiliaceae;g__Arthrobotrys;s__Arthrobotrys_flagrans

MDJ-3_gene_1509	157	WP_017532311.1	163	150/155(96.77)	7.55e-80	hypothetical protein [Wolbachia endosymbiont of Diaphorina citri]	116598	d__Bacteria;k__norank;p__Proteobacteria;c__Alphaproteobacteria;o__Rickettsiales;f__Anaplasmataceae;g__Wolbachia;s__Wolbachia_endosymbiont_of_Diaphorina_citri
MDJ-3_gene_1524	399	WP_127464277.1	773	397/399(99.50)	2.31e-265	translation initiation factor IF-2 [Wolbachia endosymbiont of Bemisia tabaci]	215173	d__Bacteria;k__norank;p__Proteobacteria;c__Alphaproteobacteria;o__Rickettsiales;f__Anaplasmataceae;g__Wolbachia;s__Wolbachia_endosymbiont_of_Bemisia_tabaci
MDJ-3_gene_1526	325	WP_064085646.1	325	323/324(99.69)	1.27e-219	NAD(P)H-dependent glycerol-3-phosphate dehydrogenase [Wolbachia endosymbiont of Dactylopius coccus]	None	Unclassified
MDJ-3_gene_1535	271	WP_064085492.1	386	269/271(99.26)	2.54e-185	ADP-forming succinate--CoA ligase subunit beta [Wolbachia endosymbiont of Dactylopius coccus]	None	Unclassified
MDJ-3_gene_1537	133	WP_010401557.1	133	133/133(100.00)	2.68e-83	MULTISPECIES: cytochrome c-type biogenesis protein CcmH [Wolbachia]	2640676	d__Bacteria;k__norank;p__Proteobacteria;c__Alphaproteobacteria;o__Rickettsiales;f__Anaplasmataceae;g__Wolbachia;s__norank
MDJ-3_gene_1544	65	OCA06014.1	108	63/65(96.92)	4.00e-39	hypothetical protein wTpre_335 [Wolbachia endosymbiont of Trichogramma pretiosum]	125593	d__Bacteria;k__norank;p__Proteobacteria;c__Alphaproteobacteria;o__Rickettsiales;f__Anaplasmataceae;g__Wolbachia;s__Wolbachia_endosymbiont_of_Trichogramma_pretiosum
MDJ-3_gene_1562	116	WP_108997718.1	113	64/96(66.67)	1.30e-38	hypothetical protein [Escherichia coli]	None	Unclassified
MDJ-3_gene_1596	113	BBA84648.1	105	46/101(45.54)	1.75e-14	50S ribosomal protein L19 [endosymbiont of Euscepes postfasciatus]	650377	d__Bacteria;k__norank;p__Proteobacteria;c__Gammaproteobacteria;o__norank;f__norank;g__norank;s__endosymbiont_of_Euscepes_postfasciatus
MDJ-3_gene_1600	232	WP_114516906.1	232	218/232(93.97)	1.14e-155	alpha/beta hydrolase [Wolbachia endosymbiont of Cylisticus convexus]	118728	d__Bacteria;k__norank;p__Proteobacteria;c__Alphaproteobacteria;o__Rickettsiales;f__Anaplasmataceae;g__Wolbachia;s__Wolbachia_endosymbiont_of_Cylisticus_convexus
MDJ-3_gene_1622	217	WP_100182205.1	1089	57/202(28.22)	1.82e-06	hypothetical protein [Candidatus Nitrosotenuis aquarius]	1846278	d__Archaea;k__norank;p__Thaumarchaeota;c__norank;o__norank;f__norank;g__Candidatus_Nitrosotenuis;s__Candidatus_Nitrosotenuis_aquarius
MDJ-3_gene_1643	51	WP_129321181.1	354	26/46(56.52)	2.85e-10	DUF4817 domain-containing protein [Enterobacter cloacae complex sp. 4DZ3-17B2]	2511990	d__Bacteria;k__norank;p__Proteobacteria;c__Gammaproteobacteria;o__Enterobacterales;f__Enterobacteriaceae;g__Enterobacter;s__Enterobacter_cloacae_complex_sp._4DZ3-17B2
MDJ-3_gene_1650	64	WP_063630532.1	64	59/60(98.33)	1.65e-34	hypothetical protein [Wolbachia endosymbiont of Laodelphax striatella]	2640676	d__Bacteria;k__norank;p__Proteobacteria;c__Alphaproteobacteria;o__Rickettsiales;f__Anaplasmataceae;g__Wolbachia;s__norank
MDJ-3_gene_1652	51	WP_075607020.1	57	42/48(87.50)	3.85e-24	DNA repair protein [Enterobacter hormaechei]	None	Unclassified
MDJ-3_gene_1668	135	RKP09056.1	53	32/52(61.54)	2.56e-17	hypothetical protein THASP1DRAFT_6229, partial [Thamnocephalis sphaerospora]	78915	d__Eukaryota;k__Fungi;p__Zoopagomycota;c__Zoopagomycetes;o__Zoopagales;f__Sigmoideomycetaceae;g__Thamnocephalis;s__Thamnocephalis_sphaerospora
MDJ-3_gene_1705	395	PBQ26533.1	2386	177/206(85.92)	4.49e-102	hypothetical protein BTO27_04595, partial	2049495	d__Bacteria;k__norank;p__Proteobacteria;c__Alphaproteobacteria;o__Rickettsiales;f__Anaplasmataceae;g__Wolbachia;s__Wolbachia_pipientis
MDJ-3_gene_1706	188	AGJ99286.1	446	148/178(83.15)	4.83e-91	hypothetical protein wNo_08960 [Wolbachia endosymbiont of Drosophila simulans wNo]	1236908	d__Bacteria;k__norank;p__Proteobacteria;c__Alphaproteobacteria;o__Rickettsiales;f__Anaplasmataceae;g__Wolbachia;s__Wolbachia_endosymbiont_of_Drosophila_simulans
MDJ-3_gene_1721	180	CQD05446.1	571	174/180(96.67)	6.47e-117	collagen triple helix repeat protein [Wolbachia endosymbiont wPip_Mol of Culex molestus]	1402163	d__Bacteria;k__norank;p__Proteobacteria;c__Alphaproteobacteria;o__Rickettsiales;f__Anaplasmataceae;g__Wolbachia;s__Wolbachia_endosymbiont_of_Culex_molestus

MDJ-3_gene_1741	223	OSY17940.1	248	72/184(39.13)	5.04e-28	hypothetical protein B0D75_04510, partial [Entomoplasmatales bacterium	None	Unclassified
MDJ-3_gene_1748	201	WP_129335249.1	124	27/69(39.13)	2.30e-07	hypothetical protein [Enterobacter cloacae complex sp. 2DZ2F20B]	2511993	d__Bacteria;k__norank;p__Proteobacteria;c__Gammaproteobacteria;o__Enterobacterales;f__Enterobacteriaceae;g__Enterobacter;s__Enterobacter_cloacae_complex_sp._2DZ2F20B
MDJ-3_gene_1756	102	CQD11292.1	245	99/102(97.06)	3.71e-69	Putative phage related protein [Wolbachia endosymbiont wPip_Mol of Culex molestus]	1402163	d__Bacteria;k__norank;p__Proteobacteria;c__Alphaproteobacteria;o__Rickettsiales;f__Anaplasmataceae;g__Wolbachia;s__Wolbachia_endosymbiont_of_Culex_molestus
MDJ-3_gene_1757	105	WP_114517895.1	105	100/105(95.24)	8.28e-67	hypothetical protein [Wolbachia endosymbiont of Cylisticus convexus]	118728	d__Bacteria;k__norank;p__Proteobacteria;c__Alphaproteobacteria;o__Rickettsiales;f__Anaplasmataceae;g__Wolbachia;s__Wolbachia_endosymbiont_of_Cylisticus_convexus
MDJ-3_gene_1808	152	AOZ94782.1	276	24/61(39.34)	1.66e-07	hypothetical protein LPB68_17980 [Paenibacillus	1763538	d__Bacteria;k__norank;p__Firmicutes;c__Bacilli;o__Bacillales;f__Paenibacillaceae;g__Paenibacillus;s__Paenibacillus_crassostreae
MDJ-3_gene_1846	123	RYE19971.1	148	50/123(40.65)	7.92e-19	DUF4371 domain-containing protein [Sphingobacteriaceae	2021370	d__Bacteria;k__norank;p__Bacteroidetes;c__Sphingobacteriia;o__Sphingobacteriales;f__Sphingobacteriaceae;g__norank;s__Sphingobacteriaceae_bacterium
MDJ-3_gene_1879	259	WP_127464248.1	261	249/261(95.40)	7.39e-175	signal peptidase I [Wolbachia endosymbiont of Bemisia tabaci]	215173	d__Bacteria;k__norank;p__Proteobacteria;c__Alphaproteobacteria;o__Rickettsiales;f__Anaplasmataceae;g__Wolbachia;s__Wolbachia_endosymbiont_of_Bemisia_tabaci
MDJ-3_gene_1919	292	OJH31055.1	280	185/276(67.03)	1.39e-118	Ankyrin repeats (3 copies) [Wolbachia endosymbiont of Armadillidium vulgare]	77039	d__Bacteria;k__norank;p__Proteobacteria;c__Alphaproteobacteria;o__Rickettsiales;f__Anaplasmataceae;g__Wolbachia;s__Wolbachia_endosymbiont_of_Armadillidium_vulgare
MDJ-3_gene_1923	92	XP_019037277.1	1386	29/78(37.18)	3.17e-09	hypothetical protein WICANDRAFT_33802 [Wickerhamomyces anomalus NRRL Y-366-8]	683960	d__Eukaryota;k__Fungi;p__Ascomycota;c__Saccharomycetes;o__Saccharomycetales;f__Phaffomycetaceae;g__Wickerhamomyces;s__Wickerhamomyces_anomalus
MDJ-3_gene_1931	90	PCG11782.1	88	87/88(98.86)	1.11e-59	succinate dehydrogenase assembly factor 2 [Wolbachia	None	Unclassified
MDJ-3_gene_1945	202	CQD10618.1	976	66/152(43.42)	1.12e-24	Ankyrin repeat domain protein [Wolbachia endosymbiont wPip_Mol of Culex molestus]	1402163	d__Bacteria;k__norank;p__Proteobacteria;c__Alphaproteobacteria;o__Rickettsiales;f__Anaplasmataceae;g__Wolbachia;s__Wolbachia_endosymbiont_of_Culex_molestus
MDJ-3_gene_1949	118	PBD15875.1	166	80/84(95.24)	8.02e-49	hypothetical protein CLD06_03670 [Wolbachia endosymbiont of Drosophila	2033655	d__Bacteria;k__norank;p__Proteobacteria;c__Alphaproteobacteria;o__Rickettsiales;f__Anaplasmataceae;g__Wolbachia;s__Wolbachia_endosymbiont_of_Drosophila_subpulchrella
MDJ-3_gene_1960	102	OPY05021.1	85	43/81(53.09)	7.59e-17	hypothetical protein A4E61_00470	1811701	d__Bacteria;k__norank;p__Proteobacteria;c__Deltaproteobacteria;o__Syntrophobacterales;f__Syntrophorhabdaceae;g__Syntrophorhabdus;s__Syntrophorhabdus_sp._PtaB.Bin184
MDJ-3_gene_2017	95	WP_046305176.1	355	69/95(72.63)	1.29e-38	chorismate synthase [Blochmannia endosymbiont of Camponotus (Colobopsis)	1505597	d__Bacteria;k__norank;p__Proteobacteria;c__Gammaproteobacteria;o__Enterobacterales;f__Enterobacteriaceae;g__Candidatus_Blochmannia;s__Blochmannia_endosymbiont_of_Camponotus_(Colobopsis)_obliquus
MDJ-3_gene_2040	172	ORX87269.1	541	89/171(52.05)	2.01e-57	PLP-dependent transferase [Basidiobolus meristosporus CBS 931.73]	1314790	d__Eukaryota;k__Fungi;p__Zoopagomycota;c__Basidiobolomycetes;o__Basidiobolales;f__Basidiobolaceae;g__Basidiobolus;s__Basidiobolus_meristosporus
MDJ-3_gene_2054	139	PHX64333.1	308	43/96(44.79)	1.03e-17	hypothetical protein CK551_01420	2026779	d__Bacteria;k__norank;p__Planctomycetes;c__Planctomycetia;o__Planctomycetales;f__Planctomycetaceae;g__norank;s__Planctomycetaceae_bacterium
MDJ-3_gene_2089	160	AAO52669.1	103	41/91(45.05)	4.08e-16	putative copia-type polyprotein [Aster yellows phytoplasma]	35779	d__Bacteria;k__norank;p__Tenericutes;c__Mollicutes;o__Acholeplasmatales;f__Acholeplasmataceae;g__Candidatus_Phytoplasma;s__Aster_yellows_phytoplasma
MDJ-3_gene_2103	47	WP_114598922.1	433	42/47(89.36)	1.11e-18	phosphopyruvate hydratase [Microbacterium arborescens]	33883	d__Bacteria;k__norank;p__Actinobacteria;c__Actinobacteria;o__Micrococcales;f__Microbacteriaceae;g__Microbacterium;s__Microbacterium_arborescens
MDJ-3_gene_2109	334	GBC44980.1	467	151/333(45.35)	2.02e-88	retrotransposable element [Rhizophagus irregularis DAOM 181602=DAOM 197198]	747089	d__Eukaryota;k__Fungi;p__Mucoromycota;c__Glomeromycetes;o__Glomerales;f__Glomeraceae;g__Rhizophagus;s__Rhizophagus_irregularis

MDJ-3_gene_2116	105	POG49816.1	111	56/71(78.87)	8.79e-36	hypothetical protein BK222_03825, partial	1912095	d__Bacteria;k__norank;p__Proteobacteria;c__Alphaproteobacteria;o__Rickettsiales;f__Anaplasmataceae;g__Wolbachia;s__Wolbachia_sp._wMel_AMD
MDJ-3_gene_2139	312	BBA85170.1	310	159/325(48.92)	6.96e-76	ribosomal RNA small subunit methyltransferase H [endosymbiont of Sipalinus gigas]	1972134	d__Bacteria;k__norank;p__Proteobacteria;c__Gammaproteobacteria;o__norank;f__norank;g__Candidatus_Nardonella;s__endosymbiont_of_Sipalinus_gigas
MDJ-3_gene_2160	99	PWS23173.1	190	66/94(70.21)	1.06e-41	50S ribosomal protein L22 [Enterococcus faecium]	1352	d__Bacteria;k__norank;p__Firmicutes;c__Bacilli;o__Lactobacillales;f__Enterococcaceae;g__Enterococcus;s__Enterococcus_faecium
MDJ-3_gene_2204	65	ORY04347.1	1393	37/61(60.66)	1.91e-14	RNA polymerase III largest subunit Rpc1 [Basidiobolus meristosporus CBS 931.73]	1314790	d__Eukaryota;k__Fungi;p__Zoopagomycota;c__Basidiobolomycetes;o__Basidiobolales;f__Basidiobolaceae;g__Basidiobolus;s__Basidiobolus_meristosporus
MDJ-3_gene_2206	94	BBA84869.1	170	50/94(53.19)	6.31e-25	uncharacterized protein NARPIN1_01370 [endosymbiont of Pachyrhynchus infernalis]	1971488	d__Bacteria;k__norank;p__Proteobacteria;c__Gammaproteobacteria;o__norank;f__norank;g__Candidatus_Nardonella;s__endosymbiont_of_Pachyrhynchus_infernalis
MDJ-3_gene_2241	280	WP_110883477.1	472	113/299(37.79)	8.37e-52	hypothetical protein [Gammaproteobacteria bacterium 2W06]	None	Unclassified
MDJ-3_gene_2250	115	RUM30001.1	189	39/81(48.15)	7.46e-12	hypothetical protein DSY42_05315 [Aquifex sp.]	313370	d__Bacteria;k__norank;p__Aquificae;c__Aquificae;o__Aquificales;f__Aquificaceae;g__Aquifex;s__Aquifex_sp.
MDJ-3_gene_2256	85	WP_127116310.1	188	68/72(94.44)	3.23e-41	hypothetical protein [Thalassobius sp. ZQ172]	2497945	d__Bacteria;k__norank;p__Proteobacteria;c__Alphaproteobacteria;o__Rhodobacterales;f__Rhodobacteraceae;g__Thalassobius;s__Thalassobius_sp._ZQ172
MDJ-3_gene_2280	197	BBA84756.1	580	114/195(58.46)	5.68e-56	single-stranded DNA-specific exonuclease [endosymbiont of Pachyrhynchus infernalis]	1971488	d__Bacteria;k__norank;p__Proteobacteria;c__Gammaproteobacteria;o__norank;f__norank;g__Candidatus_Nardonella;s__endosymbiont_of_Pachyrhynchus_infernalis
MDJ-3_gene_2284	313	BBA84806.1	838	232/308(75.32)	1.99e-152	DNA gyrase subunit A [endosymbiont of Pachyrhynchus infernalis]	1971488	d__Bacteria;k__norank;p__Proteobacteria;c__Gammaproteobacteria;o__norank;f__norank;g__Candidatus_Nardonella;s__endosymbiont_of_Pachyrhynchus_infernalis
MDJ-3_gene_2352	63	YP_009163846.1	272	29/57(50.88)	1.24e-10	ORF_085L [Scale drop disease virus]	1697349	d__Viruses;k__Bamfordvirae;p__Nucleocytoviricota;c__Megaviricetes;o__Pimascovirales;f__Iridoviridae;g__Megalocytivirus;s__Scale_drop_disease_virus
MDJ-3_gene_2384	116	CZR24135.1	70	35/67(52.24)	2.75e-07	Uncharacterised protein [Legionella pneumophila]	446	d__Bacteria;k__norank;p__Proteobacteria;c__Gammaproteobacteria;o__Legionellales;f__Legionellaceae;g__Legionella;s__Legionella_pneumophila
MDJ-3_gene_2393	81	RUM45879.1	275	33/81(40.74)	3.19e-07	hypothetical protein DSY80_02865 [Desulfocapsa	2066021	d__Bacteria;k__norank;p__Proteobacteria;c__Deltaproteobacteria;o__Desulfobacterales;f__Desulfobulbaceae;g__Desulfocapsa;s__Desulfocapsa_sp.
MDJ-3_gene_2399	185	WP_117281898.1	63	25/62(40.32)	6.00e-07	hypothetical protein [Pasteurella multocida]	747	d__Bacteria;k__norank;p__Proteobacteria;c__Gammaproteobacteria;o__Pasteurellales;f__Pasteurellaceae;g__Pasteurella;s__Pasteurella_multocida
MDJ-3_gene_2427	83	BBA85173.1	492	50/78(64.10)	7.04e-20	UDP-N-acetylmuramyl tripeptide synthase [endosymbiont of Sipalinus gigas]	1972134	d__Bacteria;k__norank;p__Proteobacteria;c__Gammaproteobacteria;o__norank;f__norank;g__Candidatus_Nardonella;s__endosymbiont_of_Sipalinus_gigas
MDJ-3_gene_2435	77	RKP27179.1	423	29/65(44.62)	9.72e-08	tyrosine aminotransferase [Syncephalis pseudoplumigaleata]	1712513	d__Eukaryota;k__Fungi;p__Zoopagomycota;c__Zoopagomycetes;o__Zoopagales;f__Piptocephalidaceae;g__Syncephalis;s__Syncephalis_pseudoplumigaleata
MDJ-3_gene_2441	117	CDR79362.1	142	100/110(90.91)	1.41e-69	Putative transposase [Wolbachia endosymbiont of Drosophila simulans wAu]	225364	d__Bacteria;k__norank;p__Proteobacteria;c__Alphaproteobacteria;o__Rickettsiales;f__Anaplasmataceae;g__Wolbachia;s__Wolbachia_endosymbiont_of_Drosophila_simulans
MDJ-3_gene_2485	54	ONI56875.1	130	53/54(98.15)	6.26e-28	elongation factor Tu domain protein [Wolbachia pipientis	1379787	d__Bacteria;k__norank;p__Proteobacteria;c__Alphaproteobacteria;o__Rickettsiales;f__Anaplasmataceae;g__Wolbachia;s__Wolbachia_pipientis
MDJ-3_gene_2501	70	RUP43916.1	643	20/31(64.52)	6.73e-07	hypothetical protein BC936DRAFT_136548 [Jimgerdemannia flammicorona]	994334	d__Eukaryota;k__Fungi;p__Mucoromycota;c__Endogonomycetes;o__Endogonales;f__Endogonaceae;g__Jimgerdemannia;s__Jimgerdemannia_flammicorona
MDJ-3_gene_2510	154	WP_108784295.1	202	150/154(97.40)	1.91e-106	ParB/RepB/Spo0J family partition protein [Wolbachia endosymbiont of Bemisia tabaci]	215173	d__Bacteria;k__norank;p__Proteobacteria;c__Alphaproteobacteria;o__Rickettsiales;f__Anaplasmataceae;g__Wolbachia;s__Wolbachia_endosymbiont_of_Bemisia_tabaci

MDJ-3_gene_2530	117	OSY19287.1	671	36/108(33.33)	9.50e-06	hypothetical protein B0D75_01270, partial [Entomoplasmatales bacterium	None	Unclassified
MDJ-3_gene_2543	103	CDH57538.1	1506	40/89(44.94)	3.09e-18	phd transcription factor [Lichtheimia corymbifera	1263082	d__Eukaryota;k__Fungi;p__Mucoromycota;c__Mucoromycetes;o__Mucorales;f__Lichtheimiaceae;g__Lichtheimia;s__Lichtheimia_corymbifera
MDJ-3_gene_2578	109	KNE87445.1	122	54/93(58.06)	8.23e-35	hypothetical protein PSTG_19170, partial [Puccinia striiformis f. sp. tritici PST-78]	1165861	d__Eukaryota;k__Fungi;p__Basidiomycota;c__Pucciniomycetes;o__Pucciniales;f__Pucciniaceae;g__Puccinia;s__Puccinia_striiformis
MDJ-3_gene_2598	45	WP_082860893.1	95	40/45(88.89)	3.58e-18	hypothetical protein [Wolbachia endosymbiont of Dactylopius coccus]	None	Unclassified
MDJ-3_gene_2621	131	YP_008126221.1	641	94/131(71.76)	1.41e-60	helicase [Vibrio phage pYD38-A]	754051	d__Viruses;k__norank;p__Uroviricota;c__Caudoviricetes;o__Caudovirales;f__Siphoviridae;g__Rofvirus;s__Vibrio_phage_pYD38-A
MDJ-3_gene_2622	138	AXQ70383.1	138	92/138(66.67)	3.43e-61	hypothetical protein vst472_66 [Salmonella virus VSt472]	2301723	d__Viruses;k__norank;p__Uroviricota;c__Caudoviricetes;o__Caudovirales;f__Siphoviridae;g__norank;s__Salmonella_virus_VSt472
MDJ-3_gene_2639	55	ABV69055.1	366	27/35(77.14)	1.00e-07	casein kinase I [Phanerochaete chrysosporium]	5306	d__Eukaryota;k__Fungi;p__Basidiomycota;c__Agaricomycetes;o__Polyporales;f__Phanerochaetaceae;g__Phanerochaete;s__Phanerochaete_chrysosporium
MDJ-3_gene_2642	51	AGJ98484.1	157	40/44(90.91)	1.38e-18	hypothetical protein wNo_00230 [Wolbachia endosymbiont of Drosophila simulans wNo]	1236908	d__Bacteria;k__norank;p__Proteobacteria;c__Alphaproteobacteria;o__Rickettsiales;f__Anaplasmataceae;g__Wolbachia;s__Wolbachia_endosymbiont_of_Drosophila_simulans
MDJ-3_gene_2643	124	AGJ98484.1	157	58/80(72.50)	1.32e-27	hypothetical protein wNo_00230 [Wolbachia endosymbiont of Drosophila simulans wNo]	1236908	d__Bacteria;k__norank;p__Proteobacteria;c__Alphaproteobacteria;o__Rickettsiales;f__Anaplasmataceae;g__Wolbachia;s__Wolbachia_endosymbiont_of_Drosophila_simulans
MDJ-3_gene_2660	269	AJG39062.1	634	88/238(36.97)	4.15e-39	putative nucleoprotein [Wenzhou Crab Virus 2]	1608092	d__Viruses;k__Orthornavirae;p__Negarnaviricota;c__Monjiviricetes;o__Jingchuvirales;f__Chuviridae;g__Mivirus;s__Crab_mivirus
MDJ-3_gene_2671	154	BBA84822.1	179	93/147(63.27)	6.18e-56	transcription termination/antitermination protein NusG [endosymbiont of Pachyrhynchus infernalis]	1971488	d__Bacteria;k__norank;p__Proteobacteria;c__Gammaproteobacteria;o__norank;f__norank;g__Candidatus_Nardonella;s__endosymbiont_of_Pachyrhynchus_infernalis
MDJ-3_gene_2729	71	XP_003038223.1	1101	37/67(55.22)	7.05e-15	hypothetical protein SCHCODRAFT_230914 [Schizophyllum commune H4-8]	578458	d__Eukaryota;k__Fungi;p__Basidiomycota;c__Agaricomycetes;o__Agaricales;f__Schizophyllaceae;g__Schizophyllum;s__Schizophyllum_commune
MDJ-3_gene_2749	105	ANZ22430.1	753	47/106(44.34)	2.53e-21	penicillin-binding protein 1B [Buchnera aphidicola (Diuraphis noxia)]	118101	d__Bacteria;k__norank;p__Proteobacteria;c__Gammaproteobacteria;o__Enterobacterales;f__Erwiniaceae;g__Buchnera;s__Buchnera_aphidicola
MDJ-3_gene_2767	93	BBA84783.1	219	57/90(63.33)	5.61e-30	uracil-DNA glycosylase [endosymbiont of Pachyrhynchus infernalis]	1971488	d__Bacteria;k__norank;p__Proteobacteria;c__Gammaproteobacteria;o__norank;f__norank;g__Candidatus_Nardonella;s__endosymbiont_of_Pachyrhynchus_infernalis
MDJ-3_gene_2790	58	WP_114598922.1	433	46/51(90.20)	5.93e-23	phosphopyruvate hydratase [Microbacterium arborescens]	33883	d__Bacteria;k__norank;p__Actinobacteria;c__Actinobacteria;o__Micrococcales;f__Microbacteriaceae;g__Microbacterium;s__Microbacterium_arborescens
MDJ-3_gene_2796	84	OOH88455.1	86	45/78(57.69)	1.46e-26	hypothetical protein BMT63_10100, partial	562	d__Bacteria;k__norank;p__Proteobacteria;c__Gammaproteobacteria;o__Enterobacterales;f__Enterobacteriaceae;g__Escherichia;s__Escherichia_coli
MDJ-3_gene_2832	173	WP_019236703.1	244	81/83(97.59)	3.83e-46	hypothetical protein [Wolbachia pipientis]	955	d__Bacteria;k__norank;p__Proteobacteria;c__Alphaproteobacteria;o__Rickettsiales;f__Anaplasmataceae;g__Wolbachia;s__Wolbachia_pipientis
MDJ-3_gene_2835	112	ADV33788.1	366	69/109(63.30)	6.87e-37	tRNA-specific 2-thiouridylase mnmA [Candidatus Blochmannia vafer str. BVAF]	859654	d__Bacteria;k__norank;p__Proteobacteria;c__Gammaproteobacteria;o__Enterobacterales;f__Enterobacteriaceae;g__Candidatus_Blochmannia;s__Candidatus_Blochmannia_vafer
MDJ-3_gene_2840	127	WP_111138353.1	79	79/79(100.00)	5.20e-48	transcriptional regulator [Pantoea sp. ARC607]	2027922	d__Bacteria;k__norank;p__Proteobacteria;c__Gammaproteobacteria;o__Enterobacterales;f__Erwiniaceae;g__Pantoea;s__Pantoea_sp._ARC607

MDJ-3_gene_2841	84	ORM85237.1	68	28/29(96.55)	2.30e-08	hypothetical protein HA42_03100 [Pantoea deleyi]	470932	d__Bacteria;k__norank;p__Proteobacteria;c__Gammaproteobacteria;o__Enterobacterales;f__Erwiniaceae;g__Pantoea;s__Pantoea_deleyi
MDJ-3_gene_2870	194	PKY57110.1	841	69/184(37.50)	1.06e-30	hypothetical protein RhiirA4_509111 [Rhizophagus	588596	d__Eukaryota;k__Fungi;p__Mucoromycota;c__Glomeromycetes;o__Glomerales;f__Glomeraceae;g__Rhizophagus;s__Rhizophagus_irregularis
MDJ-3_gene_2890	99	WP_019236592.1	99	96/99(96.97)	6.77e-61	transposase [Wolbachia pipientis]	955	d__Bacteria;k__norank;p__Proteobacteria;c__Alphaproteobacteria;o__Rickettsiales;f__Anaplasmataceae;g__Wolbachia;s__Wolbachia_pipientis
MDJ-3_gene_2924	58	PBQ26112.1	423	57/57(100.00)	5.52e-29	MFS transporter [Wolbachia pipientis wAus]	2049495	d__Bacteria;k__norank;p__Proteobacteria;c__Alphaproteobacteria;o__Rickettsiales;f__Anaplasmataceae;g__Wolbachia;s__Wolbachia_pipientis
MDJ-3_gene_2981	54	XP_018285873.1	469	36/53(67.92)	8.22e-12	hypothetical protein PHYBLDRAFT_68202 [Phycomyces blakesleeanus	763407	d__Eukaryota;k__Fungi;p__Mucoromycota;c__Mucoromycetes;o__Mucorales;f__Phycomycetaceae;g__Phycomyces;s__Phycomyces_blakesleeanus
MDJ-3_gene_2983	237	XP_002911998.1	1229	56/203(27.59)	6.55e-06	hypothetical protein CC1G_14036 [Coprinopsis	240176	d__Eukaryota;k__Fungi;p__Basidiomycota;c__Agaricomycetes;o__Agaricales;f__Psathyrellaceae;g__Coprinopsis;s__Coprinopsis_cinerea
MDJ-3_gene_2987	173	PCG11588.1	282	139/148(93.92)	1.14e-81	hypothetical protein CO539_02330 [Wolbachia	None	Unclassified
MDJ-3_gene_2992	204	WP_111141786.1	205	187/204(91.67)	7.53e-136	malonate decarboxylase holo-ACP synthase [Pantoea sp.	2027922	d__Bacteria;k__norank;p__Proteobacteria;c__Gammaproteobacteria;o__Enterobacterales;f__Erwiniaceae;g__Pantoea;s__Pantoea_sp._ARC607
MDJ-3_gene_3056	236	WP_029237671.1	260	235/236(99.58)	1.24e-153	MULTISPECIES: ABC transporter permease [Wolbachia]	2640676	d__Bacteria;k__norank;p__Proteobacteria;c__Alphaproteobacteria;o__Rickettsiales;f__Anaplasmataceae;g__Wolbachia;s__norank
MDJ-3_gene_3089	78	SJZ34956.1	78	78/78(100.00)	6.18e-43	acyl carrier protein [Pantoea agglomerans]	470933	d__Bacteria;k__norank;p__Proteobacteria;c__Gammaproteobacteria;o__Enterobacterales;f__Erwiniaceae;g__Pantoea;s__Pantoea_eucalypti
MDJ-3_gene_3090	89	VEB07380.1	77	43/56(76.79)	5.96e-21	Uncharacterised protein [Klebsiella pneumoniae]	573	d__Bacteria;k__norank;p__Proteobacteria;c__Gammaproteobacteria;o__Enterobacterales;f__Enterobacteriaceae;g__Klebsiella;s__Klebsiella_pneumoniae
MDJ-3_gene_3127	65	KNE54947.1	1184	36/65(55.38)	8.73e-11	hypothetical protein AMAG_00888 [Allomyces macrogynus ATCC 38327]	578462	d__Eukaryota;k__Fungi;p__Blastocladiomycota;c__Blastocladiomycetes;o__Blastocladiales;f__Blastocladiaceae;g__Allomyces;s__Allomyces_macrogynus
MDJ-3_gene_3146	237	RYE19449.1	394	112/235(47.66)	4.31e-70	hypothetical protein EOP45_12640	2021370	d__Bacteria;k__norank;p__Bacteroidetes;c__Sphingobacteriia;o__Sphingobacteriales;f__Sphingobacteriaceae;g__norank;s__Sphingobacteriaceae_bacterium
MDJ-3_gene_3153	84	RIB08986.1	519	41/62(66.13)	3.31e-21	P-loop containing nucleoside triphosphate hydrolase protein [Gigaspora rosea]	44941	d__Eukaryota;k__Fungi;p__Mucoromycota;c__Glomeromycetes;o__Diversisporales;f__Gigasporaceae;g__Gigaspora;s__Gigaspora_rosea
MDJ-3_gene_3165	185	WP_111140279.1	185	183/185(98.92)	5.47e-133	RNA 2',3'-cyclic phosphodiesterase [Pantoea sp. ARC607]	2027922	d__Bacteria;k__norank;p__Proteobacteria;c__Gammaproteobacteria;o__Enterobacterales;f__Erwiniaceae;g__Pantoea;s__Pantoea_sp._ARC607
MDJ-3_gene_3173	234	AYR02321.1	648	176/240(73.33)	2.14e-119	putative ATP-dependent helicase [Escherichia phage C1]	2340716	d__Viruses;k__norank;p__Uroviricota;c__Caudoviricetes;o__Caudovirales;f__Siphoviridae;g__norank;s__Escherichia_phage_C1
MDJ-3_gene_3187	97	WP_120787132.1	492	69/85(81.18)	1.28e-40	aldehyde dehydrogenase family protein [Macrococcus	None	Unclassified
MDJ-3_gene_3210	52	RDD34189.1	57	24/29(82.76)	2.46e-07	Transposase [Wolbachia endosymbiont of Cylisticus convexus]	118728	d__Bacteria;k__norank;p__Proteobacteria;c__Alphaproteobacteria;o__Rickettsiales;f__Anaplasmataceae;g__Wolbachia;s__Wolbachia_endosymbiont_of_Cylisticus_convexus
MDJ-3_gene_3212	56	CDR78751.1	130	53/55(96.36)	3.39e-28	IS5 family transposase [Wolbachia endosymbiont of Drosophila simulans wAu]	225364	d__Bacteria;k__norank;p__Proteobacteria;c__Alphaproteobacteria;o__Rickettsiales;f__Anaplasmataceae;g__Wolbachia;s__Wolbachia_endosymbiont_of_Drosophila_simulans
MDJ-3_gene_3213	130	WP_010403981.1	293	130/130(100.00)	1.75e-83	succinate--CoA ligase subunit alpha [Wolbachia endosymbiont of Nasonia vitripennis]	180837	d__Bacteria;k__norank;p__Proteobacteria;c__Alphaproteobacteria;o__Rickettsiales;f__Anaplasmataceae;g__Wolbachia;s__Wolbachia_endosymbiont_of_Nasonia_vitripennis

MDJ-3_gene_3214	100	WP_010403984.1	386	100/100(100.00)	1.99e-56	ADP-forming succinate--CoA ligase subunit beta [Wolbachia endosymbiont of Nasonia vitripennis]	180837	d__Bacteria;k__norank;p__Proteobacteria;c__Alphaproteobacteria;o__Rickettsiales;f__Anaplasmataceae;g__Wolbachia;s__Wolbachia_endosymbiont_of_Nasonia_vitripennis
MDJ-3_gene_3222	127	ORM76261.1	77	37/77(48.05)	1.00e-08	hypothetical protein HA42_19280 [Pantoea deleyi]	470932	d__Bacteria;k__norank;p__Proteobacteria;c__Gammaproteobacteria;o__Enterobacterales;f__Erwiniaceae;g__Pantoea;s__Pantoea_deleyi
MDJ-3_gene_3234	102	OSY19256.1	647	45/100(45.00)	3.41e-22	hypothetical protein B0D75_01370 [Entomoplasmatales bacterium	None	Unclassified
MDJ-3_gene_3235	106	OSY19256.1	647	43/97(44.33)	8.42e-18	hypothetical protein B0D75_01370 [Entomoplasmatales bacterium	None	Unclassified
MDJ-3_gene_3242	56	OLN14819.1	102	44/56(78.57)	1.52e-28	hypothetical protein BUE68_12295, partial	1717	d__Bacteria;k__norank;p__Actinobacteria;c__Actinobacteria;o__Corynebacteriales;f__Corynebacteriaceae;g__Corynebacterium;s__Corynebacterium_diphtheriae
MDJ-3_gene_3246	87	PBQ27682.1	355	87/87(100.00)	6.70e-49	recombinase RecA [Wolbachia pipientis wAus]	2049495	d__Bacteria;k__norank;p__Proteobacteria;c__Alphaproteobacteria;o__Rickettsiales;f__Anaplasmataceae;g__Wolbachia;s__Wolbachia_pipientis
MDJ-3_gene_3249	88	XP_016609722.1	223	35/54(64.81)	1.49e-15	hypothetical protein SPPG_03478 [Spizellomyces punctatus DAOM BR117]	645134	d__Eukaryota;k__Fungi;p__Chytridiomycota;c__Chytridiomycetes;o__Spizellomycetales;f__Spizellomycetaceae;g__Spizellomyces;s__Spizellomyces_punctatus
MDJ-3_gene_3257	51	OJH30293.1	55	30/38(78.95)	1.52e-14	hypothetical protein Wxf_03227 [Armadillidium vulgare]	77039	d__Bacteria;k__norank;p__Proteobacteria;c__Alphaproteobacteria;o__Rickettsiales;f__Anaplasmataceae;g__Wolbachia;s__Wolbachia_endosymbiont_of_Armadillidium_vulgare
MDJ-3_gene_3258	51	PBQ25409.1	80	30/39(76.92)	8.55e-09	hypothetical protein BTO27_05365, partial	2049495	d__Bacteria;k__norank;p__Proteobacteria;c__Alphaproteobacteria;o__Rickettsiales;f__Anaplasmataceae;g__Wolbachia;s__Wolbachia_pipientis
MDJ-3_gene_3259	76	OUW42441.1	1084	21/36(58.33)	5.69e-06	hypothetical protein CBD41_09080, partial	1986654	d__Bacteria;k__norank;p__norank;c__norank;o__norank;f__norank;g__norank;s__bacterium_TMED181
MDJ-3_gene_3273	180	CAQ54157.1	290	169/180(93.89)	1.13e-111	transposase [Wolbachia endosymbiont of Culex quinquefasciatus Pel]	570417	d__Bacteria;k__norank;p__Proteobacteria;c__Alphaproteobacteria;o__Rickettsiales;f__Anaplasmataceae;g__Wolbachia;s__Wolbachia_endosymbiont_of_Culex_quinquefasciatus
MDJ-3_gene_3294	162	AGJ98566.1	344	124/148(83.78)	3.55e-76	Minor capsid protein C, putative [Wolbachia endosymbiont of Drosophila simulans wNo]	1236908	d__Bacteria;k__norank;p__Proteobacteria;c__Alphaproteobacteria;o__Rickettsiales;f__Anaplasmataceae;g__Wolbachia;s__Wolbachia_endosymbiont_of_Drosophila_simulans
MDJ-3_gene_3331	115	YP_001967400.1	168	100/115(86.96)	7.97e-65	hypothetical protein RM_p20 (plasmid) [Rickettsia monacensis]	None	Unclassified
MDJ-3_gene_3375	189	WP_084883551.1	280	148/189(78.31)	3.28e-104	DUF3289 family protein [Pantoea septica]	472695	d__Bacteria;k__norank;p__Proteobacteria;c__Gammaproteobacteria;o__Enterobacterales;f__Erwiniaceae;g__Pantoea;s__Pantoea_septica
MDJ-3_gene_3383	157	WP_047039870.1	995	106/116(91.38)	4.84e-59	Tn3 family transposase [Klebsiella aerogenes]	548	d__Bacteria;k__norank;p__Proteobacteria;c__Gammaproteobacteria;o__Enterobacterales;f__Enterobacteriaceae;g__Klebsiella;s__Klebsiella_aerogenes
MDJ-3_gene_3384	53	ORX70058.1	1058	26/40(65.00)	1.33e-10	hypothetical protein DL89DRAFT_151663 [Linderina pennispora]	61395	d__Eukaryota;k__Fungi;p__Zoopagomycota;c__Kickxellomycetes;o__Kickxellales;f__Kickxellaceae;g__Linderina;s__Linderina_pennispora
MDJ-3_gene_3399	81	ORY85885.1	3997	24/64(37.50)	6.50e-08	hypothetical protein LY90DRAFT_498993 [Neocallimastix californiae]	1754190	d__Eukaryota;k__Fungi;p__Chytridiomycota;c__Neocallimastigomycetes;o__Neocallimastigales;f__Neocallimastigaceae;g__Neocallimastix;s__Neocallimastix_californiae
MDJ-3_gene_3464	88	WP_111138833.1	83	70/83(84.34)	5.57e-45	bacteriocin immunity protein [Pantoea sp. ARC607]	2027922	d__Bacteria;k__norank;p__Proteobacteria;c__Gammaproteobacteria;o__Enterobacterales;f__Erwiniaceae;g__Pantoea;s__Pantoea_sp._ARC607
MDJ-3_gene_3467	82	RIB26048.1	734	27/69(39.13)	2.78e-06	Muniscin C-terminal mu homology domain-containing protein [Gigaspora rosea]	44941	d__Eukaryota;k__Fungi;p__Mucoromycota;c__Glomeromycetes;o__Diversisporales;f__Gigasporaceae;g__Gigaspora;s__Gigaspora_rosea
PB1_gene_2	97	ORM86357.1	137	97/97(100.00)	7.64e-63	excinuclease ABC subunit C [Pantoea deleyi]	470932	d__Bacteria;k__norank;p__Proteobacteria;c__Gammaproteobacteria;o__Enterobacterales;f__Erwiniaceae;g__Pantoea;s__Pantoea_deleyi

PB1_gene_3	168	ORM86356.1	168	162/168(96.43)	1.67e-112	GNAT family N-acetyltransferase [Pantoea deleyi]	470932	d__Bacteria;k__norank;p__Proteobacteria;c__Gammaproteobacteria;o__Enterobacterales;f__Erwiniaceae;g__Pantoea;s__Pantoea_deleyi
PB1_gene_4	176	ORM86365.1	174	169/174(97.13)	4.39e-116	hypothetical protein HA42_01110 [Pantoea deleyi]	470932	d__Bacteria;k__norank;p__Proteobacteria;c__Gammaproteobacteria;o__Enterobacterales;f__Erwiniaceae;g__Pantoea;s__Pantoea_deleyi
PB1_gene_5	331	ORM86355.1	331	329/331(99.40)	4.60e-241	protease [Pantoea deleyi]	470932	d__Bacteria;k__norank;p__Proteobacteria;c__Gammaproteobacteria;o__Enterobacterales;f__Erwiniaceae;g__Pantoea;s__Pantoea_deleyi
PB1_gene_6	294	ORM86354.1	292	285/292(97.60)	1.82e-212	U32 family peptidase [Pantoea deleyi]	470932	d__Bacteria;k__norank;p__Proteobacteria;c__Gammaproteobacteria;o__Enterobacterales;f__Erwiniaceae;g__Pantoea;s__Pantoea_deleyi
PB1_gene_7	336	ORM86353.1	336	331/336(98.51)	6.51e-239	hypothetical protein HA42_01095 [Pantoea deleyi]	470932	d__Bacteria;k__norank;p__Proteobacteria;c__Gammaproteobacteria;o__Enterobacterales;f__Erwiniaceae;g__Pantoea;s__Pantoea_deleyi
PB1_gene_8	88	ORM86352.1	306	59/72(81.94)	7.37e-31	AraC family transcriptional regulator [Pantoea deleyi]	470932	d__Bacteria;k__norank;p__Proteobacteria;c__Gammaproteobacteria;o__Enterobacterales;f__Erwiniaceae;g__Pantoea;s__Pantoea_deleyi
PB1_gene_9	82	WP_111139574.1	248	74/87(85.06)	2.52e-36	glucose 1-dehydrogenase [Pantoea sp. ARC607]	2027922	d__Bacteria;k__norank;p__Proteobacteria;c__Gammaproteobacteria;o__Enterobacterales;f__Erwiniaceae;g__Pantoea;s__Pantoea_sp._ARC607
PB1_gene_10	630	WP_111139575.1	630	620/630(98.41)	0	DEAD/DEAH family ATP-dependent RNA helicase [Pantoea	2027922	d__Bacteria;k__norank;p__Proteobacteria;c__Gammaproteobacteria;o__Enterobacterales;f__Erwiniaceae;g__Pantoea;s__Pantoea_sp._ARC607
PB1_gene_11	294	ORM86350.1	294	293/294(99.66)	3.25e-211	lipoprotein NlpI [Pantoea deleyi]	470932	d__Bacteria;k__norank;p__Proteobacteria;c__Gammaproteobacteria;o__Enterobacterales;f__Erwiniaceae;g__Pantoea;s__Pantoea_deleyi
PB1_gene_12	734	ADO11799.1	740	725/734(98.77)	0	polyribonucleotide nucleotidyltransferase [Pantoea vagans C9-1]	712898	d__Bacteria;k__norank;p__Proteobacteria;c__Gammaproteobacteria;o__Enterobacterales;f__Erwiniaceae;g__Pantoea;s__Pantoea_vagans
PB1_gene_13	89	SJZ87594.1	89	89/89(100.00)	4.01e-53	SSU ribosomal protein S15P [Pantoea agglomerans]	470933	d__Bacteria;k__norank;p__Proteobacteria;c__Gammaproteobacteria;o__Enterobacterales;f__Erwiniaceae;g__Pantoea;s__Pantoea_eucalypti
PB1_gene_14	314	WP_111139578.1	314	310/314(98.73)	2.81e-222	tRNA pseudouridine(55) synthase TruB [Pantoea sp. ARC607]	2027922	d__Bacteria;k__norank;p__Proteobacteria;c__Gammaproteobacteria;o__Enterobacterales;f__Erwiniaceae;g__Pantoea;s__Pantoea_sp._ARC607
PB1_gene_15	136	WP_111139579.1	135	135/135(100.00)	4.10e-86	30S ribosome-binding factor RbfA [Pantoea sp. ARC607]	2027922	d__Bacteria;k__norank;p__Proteobacteria;c__Gammaproteobacteria;o__Enterobacterales;f__Erwiniaceae;g__Pantoea;s__Pantoea_sp._ARC607
PB1_gene_18	195	WP_111139745.1	247	174/246(70.73)	2.39e-74	Sec-independent protein translocase subunit TatB [Pantoea	2027922	d__Bacteria;k__norank;p__Proteobacteria;c__Gammaproteobacteria;o__Enterobacterales;f__Erwiniaceae;g__Pantoea;s__Pantoea_sp._ARC607
PB1_gene_19	252	WP_111139744.1	252	251/252(99.60)	2.04e-168	Sec-independent protein translocase subunit TatC [Pantoea	2027922	d__Bacteria;k__norank;p__Proteobacteria;c__Gammaproteobacteria;o__Enterobacterales;f__Erwiniaceae;g__Pantoea;s__Pantoea_sp._ARC607
PB1_gene_20	260	WP_111139743.1	260	258/260(99.23)	1.72e-189	3'-5' ssDNA/RNA exonuclease TatD [Pantoea sp. ARC607]	2027922	d__Bacteria;k__norank;p__Proteobacteria;c__Gammaproteobacteria;o__Enterobacterales;f__Erwiniaceae;g__Pantoea;s__Pantoea_sp._ARC607
PB1_gene_21	162	ORM78269.1	162	162/162(100.00)	8.69e-112	transcriptional activator RfaH [Pantoea deleyi]	470932	d__Bacteria;k__norank;p__Proteobacteria;c__Gammaproteobacteria;o__Enterobacterales;f__Erwiniaceae;g__Pantoea;s__Pantoea_deleyi
PB1_gene_22	494	ORM78270.1	494	494/494(100.00)	0	3-octaprenyl-4-hydroxybenzoate decarboxylase [Pantoea deleyi]	470932	d__Bacteria;k__norank;p__Proteobacteria;c__Gammaproteobacteria;o__Enterobacterales;f__Erwiniaceae;g__Pantoea;s__Pantoea_deleyi
PB1_gene_23	233	WP_111139740.1	233	232/233(99.57)	8.43e-163	NAD(P)H-flavin reductase [Pantoea sp. ARC607]	2027922	d__Bacteria;k__norank;p__Proteobacteria;c__Gammaproteobacteria;o__Enterobacterales;f__Erwiniaceae;g__Pantoea;s__Pantoea_sp._ARC607
PB1_gene_24	387	ORM78272.1	387	381/387(98.45)	1.24e-271	3-ketoacyl-CoA thiolase [Pantoea deleyi]	470932	d__Bacteria;k__norank;p__Proteobacteria;c__Gammaproteobacteria;o__Enterobacterales;f__Erwiniaceae;g__Pantoea;s__Pantoea_deleyi
PB1_gene_25	727	WP_111139738.1	727	711/727(97.80)	0	fatty acid oxidation complex subunit alpha FadB [Pantoea sp.	2027922	d__Bacteria;k__norank;p__Proteobacteria;c__Gammaproteobacteria;o__Enterobacterales;f__Erwiniaceae;g__Pantoea;s__Pantoea_sp._ARC607
PB1_gene_26	443	ORM78273.1	443	440/443(99.32)	0	Xaa-Pro dipeptidase [Pantoea deleyi]	470932	d__Bacteria;k__norank;p__Proteobacteria;c__Gammaproteobacteria;o__Enterobacterales;f__Erwiniaceae;g__Pantoea;s__Pantoea_deleyi
PB1_gene_27	205	ORM78274.1	205	203/205(99.02)	1.08e-142	YigZ family protein [Pantoea deleyi]	470932	d__Bacteria;k__norank;p__Proteobacteria;c__Gammaproteobacteria;o__Enterobacterales;f__Erwiniaceae;g__Pantoea;s__Pantoea_deleyi
PB1_gene_28	483	ORM78275.1	483	483/483(100.00)	0	potassium transporter [Pantoea deleyi]	470932	d__Bacteria;k__norank;p__Proteobacteria;c__Gammaproteobacteria;o__Enterobacterales;f__Erwiniaceae;g__Pantoea;s__Pantoea_deleyi

PB1_gene_29	177	WP_111139734.1	177	176/177(99.44)	1.30e-127	menaquinone-dependent protoporphyrinogen IX dehydrogenase [Pantoea sp. ARC607]	2027922	d__Bacteria;k__norank;p__Proteobacteria;c__Gammaproteobacteria;o__Enterobacterales;f__Erwiniaceae;g__Pantoea;s__Pantoea_sp._ARC607
PB1_gene_32	300	ORM75512.1	300	290/300(96.67)	1.99e-199	malonate decarboxylase subunit epsilon [Pantoea deleyi]	470932	d__Bacteria;k__norank;p__Proteobacteria;c__Gammaproteobacteria;o__Enterobacterales;f__Erwiniaceae;g__Pantoea;s__Pantoea_deleyi
PB1_gene_33	311	ORM75513.1	311	299/311(96.14)	2.98e-211	LysR family transcriptional regulator [Pantoea deleyi]	470932	d__Bacteria;k__norank;p__Proteobacteria;c__Gammaproteobacteria;o__Enterobacterales;f__Erwiniaceae;g__Pantoea;s__Pantoea_deleyi
PB1_gene_34	815	WP_111141780.1	815	810/815(99.39)	0	glycogen phosphorylase [Pantoea sp. ARC607]	2027922	d__Bacteria;k__norank;p__Proteobacteria;c__Gammaproteobacteria;o__Enterobacterales;f__Erwiniaceae;g__Pantoea;s__Pantoea_sp._ARC607
PB1_gene_35	477	ORM80106.1	477	476/477(99.79)	0	starch synthase [Pantoea deleyi]	470932	d__Bacteria;k__norank;p__Proteobacteria;c__Gammaproteobacteria;o__Enterobacterales;f__Erwiniaceae;g__Pantoea;s__Pantoea_deleyi
PB1_gene_36	420	ORM80107.1	430	420/420(100.00)	1.23e-313	glucose-1-phosphate adenylyltransferase [Pantoea	470932	d__Bacteria;k__norank;p__Proteobacteria;c__Gammaproteobacteria;o__Enterobacterales;f__Erwiniaceae;g__Pantoea;s__Pantoea_deleyi
PB1_gene_39	239	ORM86318.1	239	235/239(98.33)	1.07e-168	monofunctional biosynthetic peptidoglycan transglycosylase [Pantoea deleyi]	470932	d__Bacteria;k__norank;p__Proteobacteria;c__Gammaproteobacteria;o__Enterobacterales;f__Erwiniaceae;g__Pantoea;s__Pantoea_deleyi
PB1_gene_40	90	ORM86319.1	90	87/90(96.67)	1.24e-52	phosphohistidinoprotein-hexose phosphotransferase [Pantoea	470932	d__Bacteria;k__norank;p__Proteobacteria;c__Gammaproteobacteria;o__Enterobacterales;f__Erwiniaceae;g__Pantoea;s__Pantoea_deleyi
PB1_gene_41	284	SJZ88496.1	284	284/284(100.00)	2.10e-202	UPF0042 nucleotide-binding protein [Pantoea agglomerans]	470933	d__Bacteria;k__norank;p__Proteobacteria;c__Gammaproteobacteria;o__Enterobacterales;f__Erwiniaceae;g__Pantoea;s__Pantoea_eucalypti
PB1_gene_42	159	SJZ88481.1	159	159/159(100.00)	2.14e-106	PTS IIA-like nitrogen-regulatory protein PtsN [Pantoea	470933	d__Bacteria;k__norank;p__Proteobacteria;c__Gammaproteobacteria;o__Enterobacterales;f__Erwiniaceae;g__Pantoea;s__Pantoea_eucalypti
PB1_gene_43	95	ORM86322.1	95	95/95(100.00)	8.73e-62	ribosomal subunit interface protein [Pantoea deleyi]	470932	d__Bacteria;k__norank;p__Proteobacteria;c__Gammaproteobacteria;o__Enterobacterales;f__Erwiniaceae;g__Pantoea;s__Pantoea_deleyi
PB1_gene_44	477	ORM86323.1	477	472/477(98.95)	4.64e-285	RNA polymerase factor sigma-54 [Pantoea deleyi]	470932	d__Bacteria;k__norank;p__Proteobacteria;c__Gammaproteobacteria;o__Enterobacterales;f__Erwiniaceae;g__Pantoea;s__Pantoea_deleyi
PB1_gene_45	241	PAW34510.1	241	241/241(100.00)	1.39e-165	ABC transporter ATP-binding protein [Pantoea vagans]	470934	d__Bacteria;k__norank;p__Proteobacteria;c__Gammaproteobacteria;o__Enterobacterales;f__Erwiniaceae;g__Pantoea;s__Pantoea_vagans
PB1_gene_46	179	ORM86325.1	183	179/179(100.00)	1.58e-105	lipopolysaccharide ABC transporter substrate-binding protein LptA [Pantoea deleyi]	470932	d__Bacteria;k__norank;p__Proteobacteria;c__Gammaproteobacteria;o__Enterobacterales;f__Erwiniaceae;g__Pantoea;s__Pantoea_deleyi
PB1_gene_47	190	WP_111139600.1	190	186/190(97.89)	1.81e-129	LPS export ABC transporter periplasmic protein LptC [Pantoea sp. ARC607]	2027922	d__Bacteria;k__norank;p__Proteobacteria;c__Gammaproteobacteria;o__Enterobacterales;f__Erwiniaceae;g__Pantoea;s__Pantoea_sp._ARC607
PB1_gene_48	188	SJZ88367.1	188	186/188(98.94)	2.30e-131	3-deoxy-D-manno-octulosonate 8-phosphate phosphatase (KDO 8-P phosphatase) [Pantoea	470933	d__Bacteria;k__norank;p__Proteobacteria;c__Gammaproteobacteria;o__Enterobacterales;f__Erwiniaceae;g__Pantoea;s__Pantoea_eucalypti
PB1_gene_51	682	ORM78084.1	682	681/682(99.85)	0	potassium-transporting ATPase subunit B [Pantoea deleyi]	470932	d__Bacteria;k__norank;p__Proteobacteria;c__Gammaproteobacteria;o__Enterobacterales;f__Erwiniaceae;g__Pantoea;s__Pantoea_deleyi
PB1_gene_52	562	ORM78083.1	562	556/562(98.93)	0	potassium-transporting ATPase subunit KdpA [Pantoea deleyi]	470932	d__Bacteria;k__norank;p__Proteobacteria;c__Gammaproteobacteria;o__Enterobacterales;f__Erwiniaceae;g__Pantoea;s__Pantoea_deleyi
PB1_gene_54	384	ORM78081.1	388	379/384(98.70)	2.00e-281	hypothetical protein HA42_17030 [Pantoea deleyi]	470932	d__Bacteria;k__norank;p__Proteobacteria;c__Gammaproteobacteria;o__Enterobacterales;f__Erwiniaceae;g__Pantoea;s__Pantoea_deleyi
PB1_gene_55	176	WP_090966972.1	166	154/166(92.77)	2.41e-109	GNAT family N-acetyltransferase [Pantoea sp. OV426]	1881036	d__Bacteria;k__norank;p__Proteobacteria;c__Gammaproteobacteria;o__Enterobacterales;f__Erwiniaceae;g__Pantoea;s__Pantoea_sp._OV426
PB1_gene_56	610	ORM78079.1	610	604/610(99.02)	0	multidrug ABC transporter ATP-binding protein [Pantoea deleyi]	470932	d__Bacteria;k__norank;p__Proteobacteria;c__Gammaproteobacteria;o__Enterobacterales;f__Erwiniaceae;g__Pantoea;s__Pantoea_deleyi
PB1_gene_59	272	ORM80377.1	272	272/272(100.00)	2.70e-183	undecaprenyl-diphosphatase [Pantoea deleyi]	470932	d__Bacteria;k__norank;p__Proteobacteria;c__Gammaproteobacteria;o__Enterobacterales;f__Erwiniaceae;g__Pantoea;s__Pantoea_deleyi

PB1_gene_61	201	ORM80379.1	201	199/201(99.00)	1.80e-139	glycerol-3-phosphate acyltransferase [Pantoea deleyi]	470932	d__Bacteria;k__norank;p__Proteobacteria;c__Gammaproteobacteria;o__Enterobacterales;f__Erwiniaceae;g__Pantoea;s__Pantoea_deleyi
PB1_gene_62	337	ORM80380.1	337	335/337(99.41)	2.56e-241	tRNA (adenosine(37)-N6)-threonylcarbamoyltransferase complex transferase subunit TsaD [Pantoea deleyi]	470932	d__Bacteria;k__norank;p__Proteobacteria;c__Gammaproteobacteria;o__Enterobacterales;f__Erwiniaceae;g__Pantoea;s__Pantoea_deleyi
PB1_gene_63	107	GBU11804.1	116	89/107(83.18)	4.38e-49	hypothetical protein AwEntero_04050 [Enterobacterales bacterium]	1993870	d__Bacteria;k__norank;p__Proteobacteria;c__Gammaproteobacteria;o__Enterobacterales;f__norank;g__norank;s__Enterobacterales_bacterium
PB1_gene_64	581	WP_111142201.1	581	576/581(99.14)	0	DNA primase [Pantoea sp. ARC607]	2027922	d__Bacteria;k__norank;p__Proteobacteria;c__Gammaproteobacteria;o__Enterobacterales;f__Erwiniaceae;g__Pantoea;s__Pantoea_sp._ARC607
PB1_gene_67	288	WP_111140874.1	288	286/288(99.31)	3.78e-207	Hsp33 family molecular chaperone HslO [Pantoea sp.	2027922	d__Bacteria;k__norank;p__Proteobacteria;c__Gammaproteobacteria;o__Enterobacterales;f__Erwiniaceae;g__Pantoea;s__Pantoea_sp._ARC607
PB1_gene_68	133	ORM80870.1	133	129/133(96.99)	3.80e-83	heat-shock protein [Pantoea deleyi]	470932	d__Bacteria;k__norank;p__Proteobacteria;c__Gammaproteobacteria;o__Enterobacterales;f__Erwiniaceae;g__Pantoea;s__Pantoea_deleyi
PB1_gene_69	215	ORM80871.1	215	205/215(95.35)	4.93e-153	GMP/IMP nucleotidase [Pantoea deleyi]	470932	d__Bacteria;k__norank;p__Proteobacteria;c__Gammaproteobacteria;o__Enterobacterales;f__Erwiniaceae;g__Pantoea;s__Pantoea_deleyi
PB1_gene_73	406	WP_111138342.1	400	400/400(100.00)	1.91e-290	ParA family protein [Pantoea sp. ARC607]	53335	d__Bacteria;k__norank;p__Proteobacteria;c__Gammaproteobacteria;o__Enterobacterales;f__Erwiniaceae;g__Pantoea;s__norank
PB1_gene_77	448	ORM83033.1	448	443/448(98.88)	9.37e-316	RIP metalloprotease RseP [Pantoea deleyi]	470932	d__Bacteria;k__norank;p__Proteobacteria;c__Gammaproteobacteria;o__Enterobacterales;f__Erwiniaceae;g__Pantoea;s__Pantoea_deleyi
PB1_gene_78	803	ORM83034.1	803	797/803(99.25)	0	outer membrane protein assembly factor BamA [Pantoea deleyi]	470932	d__Bacteria;k__norank;p__Proteobacteria;c__Gammaproteobacteria;o__Enterobacterales;f__Erwiniaceae;g__Pantoea;s__Pantoea_deleyi
PB1_gene_79	165	SJZ79644.1	165	164/164(100.00)	1.65e-97	periplasmic chaperone for outer membrane proteins Skp [Pantoea agglomerans]	470933	d__Bacteria;k__norank;p__Proteobacteria;c__Gammaproteobacteria;o__Enterobacterales;f__Erwiniaceae;g__Pantoea;s__Pantoea_eucalypti
PB1_gene_80	341	ORM83036.1	341	338/341(99.12)	8.96e-189	UDP-3-O-(3-hydroxymyristoyl)glucosamine N-acyltransferase [Pantoea deleyi]	470932	d__Bacteria;k__norank;p__Proteobacteria;c__Gammaproteobacteria;o__Enterobacterales;f__Erwiniaceae;g__Pantoea;s__Pantoea_deleyi
PB1_gene_81	151	SJZ79591.1	151	150/151(99.34)	1.87e-103	3-hydroxyacyl-[acyl-carrier-protein] dehydratase [Pantoea	470933	d__Bacteria;k__norank;p__Proteobacteria;c__Gammaproteobacteria;o__Enterobacterales;f__Erwiniaceae;g__Pantoea;s__Pantoea_eucalypti
PB1_gene_82	262	ORM83038.1	262	261/261(100.00)	4.17e-182	acyl-[acyl-carrier-protein]--UDP-N-acetylglucosamine O-acyltransferase [Pantoea deleyi]	470932	d__Bacteria;k__norank;p__Proteobacteria;c__Gammaproteobacteria;o__Enterobacterales;f__Erwiniaceae;g__Pantoea;s__Pantoea_deleyi
PB1_gene_84	431	ORM78613.1	431	431/431(100.00)	2.45e-298	phosphopyruvate hydratase [Pantoea deleyi]	470932	d__Bacteria;k__norank;p__Proteobacteria;c__Gammaproteobacteria;o__Enterobacterales;f__Erwiniaceae;g__Pantoea;s__Pantoea_deleyi
PB1_gene_85	545	ORM78612.1	545	545/545(100.00)	0	CTP synthase [Pantoea deleyi]	470932	d__Bacteria;k__norank;p__Proteobacteria;c__Gammaproteobacteria;o__Enterobacterales;f__Erwiniaceae;g__Pantoea;s__Pantoea_deleyi
PB1_gene_86	262	ORM78611.1	262	256/261(98.08)	2.94e-182	nucleoside triphosphate pyrophosphohydrolase [Pantoea deleyi]	470932	d__Bacteria;k__norank;p__Proteobacteria;c__Gammaproteobacteria;o__Enterobacterales;f__Erwiniaceae;g__Pantoea;s__Pantoea_deleyi
PB1_gene_87	743	ORM78610.1	743	741/743(99.73)	0	GTP diphosphokinase [Pantoea deleyi]	470932	d__Bacteria;k__norank;p__Proteobacteria;c__Gammaproteobacteria;o__Enterobacterales;f__Erwiniaceae;g__Pantoea;s__Pantoea_deleyi
PB1_gene_88	438	ORM78609.1	438	430/438(98.17)	0	23S rRNA (uracil(1939)-C(5))-methyltransferase [Pantoea	470932	d__Bacteria;k__norank;p__Proteobacteria;c__Gammaproteobacteria;o__Enterobacterales;f__Erwiniaceae;g__Pantoea;s__Pantoea_deleyi
PB1_gene_90	244	ORM75431.1	241	231/241(95.85)	1.67e-128	type IV pilus biogenesis/stability protein PilW [Pantoea deleyi]	470932	d__Bacteria;k__norank;p__Proteobacteria;c__Gammaproteobacteria;o__Enterobacterales;f__Erwiniaceae;g__Pantoea;s__Pantoea_deleyi
PB1_gene_91	336	WP_111139150.1	337	288/354(81.36)	4.77e-170	cytoskeleton protein RodZ [Pantoea sp. ARC607]	2027922	d__Bacteria;k__norank;p__Proteobacteria;c__Gammaproteobacteria;o__Enterobacterales;f__Erwiniaceae;g__Pantoea;s__Pantoea_sp._ARC607

PB1_gene_92	378	ORM81004.1	373	372/373(99.73)	2.27e-259	4-hydroxy-3-methylbut-2-en-1-yl diphosphate synthase [Pantoea deleyi]	470932	d__Bacteria;k__norank;p__Proteobacteria;c__Gammaproteobacteria;o__Enterobacterales;f__Erwiniaceae;g__Pantoea;s__Pantoea_deleyi
PB1_gene_93	424	ORM81005.1	426	421/424(99.29)	1.55e-307	histidine--tRNA ligase [Pantoea deleyi]	470932	d__Bacteria;k__norank;p__Proteobacteria;c__Gammaproteobacteria;o__Enterobacterales;f__Erwiniaceae;g__Pantoea;s__Pantoea_deleyi
PB1_gene_94	205	ORM81006.1	205	203/205(99.02)	3.47e-139	hypothetical protein HA42_12165 [Pantoea deleyi]	470932	d__Bacteria;k__norank;p__Proteobacteria;c__Gammaproteobacteria;o__Enterobacterales;f__Erwiniaceae;g__Pantoea;s__Pantoea_deleyi
PB1_gene_95	393	ORM81007.1	393	392/393(99.75)	1.80e-283	outer membrane protein assembly factor BamB [Pantoea deleyi]	470932	d__Bacteria;k__norank;p__Proteobacteria;c__Gammaproteobacteria;o__Enterobacterales;f__Erwiniaceae;g__Pantoea;s__Pantoea_deleyi
PB1_gene_96	496	SJZ70607.1	496	490/496(98.79)	0	GTP-binding protein [Pantoea agglomerans]	470933	d__Bacteria;k__norank;p__Proteobacteria;c__Gammaproteobacteria;o__Enterobacterales;f__Erwiniaceae;g__Pantoea;s__Pantoea_eucalypti
PB1_gene_99	125	ORM83979.1	125	124/125(99.20)	8.88e-85	Co2+/Mg2+ efflux protein ApaG [Pantoea deleyi]	470932	d__Bacteria;k__norank;p__Proteobacteria;c__Gammaproteobacteria;o__Enterobacterales;f__Erwiniaceae;g__Pantoea;s__Pantoea_deleyi
PB1_gene_100	273	ORM83978.1	273	272/273(99.63)	9.20e-196	16S rRNA (adenine(1518)-N(6)/adenine(1519)-N(6))-dimethyltransferase [Pantoea	470932	d__Bacteria;k__norank;p__Proteobacteria;c__Gammaproteobacteria;o__Enterobacterales;f__Erwiniaceae;g__Pantoea;s__Pantoea_deleyi
PB1_gene_101	330	ORM84012.1	330	327/330(99.09)	6.48e-236	4-hydroxythreonine-4-phosphate dehydrogenase PdxA [Pantoea deleyi]	470932	d__Bacteria;k__norank;p__Proteobacteria;c__Gammaproteobacteria;o__Enterobacterales;f__Erwiniaceae;g__Pantoea;s__Pantoea_deleyi
PB1_gene_102	431	ORM83977.1	431	429/431(99.54)	2.10e-299	peptidylprolyl isomerase SurA [Pantoea deleyi]	470932	d__Bacteria;k__norank;p__Proteobacteria;c__Gammaproteobacteria;o__Enterobacterales;f__Erwiniaceae;g__Pantoea;s__Pantoea_deleyi
PB1_gene_103	800	ORM83976.1	788	779/788(98.86)	0	LPS assembly protein LptD [Pantoea deleyi]	470932	d__Bacteria;k__norank;p__Proteobacteria;c__Gammaproteobacteria;o__Enterobacterales;f__Erwiniaceae;g__Pantoea;s__Pantoea_deleyi
PB1_gene_104	269	ORM83975.1	269	268/269(99.63)	6.86e-180	molecular chaperone DjlA [Pantoea deleyi]	470932	d__Bacteria;k__norank;p__Proteobacteria;c__Gammaproteobacteria;o__Enterobacterales;f__Erwiniaceae;g__Pantoea;s__Pantoea_deleyi
PB1_gene_107	170	ORM81273.1	170	170/170(100.00)	1.12e-111	peptide deformylase [Pantoea deleyi]	470932	d__Bacteria;k__norank;p__Proteobacteria;c__Gammaproteobacteria;o__Enterobacterales;f__Erwiniaceae;g__Pantoea;s__Pantoea_deleyi
PB1_gene_108	314	ORM81272.1	314	307/314(97.77)	2.40e-223	methionyl-tRNA formyltransferase [Pantoea	470932	d__Bacteria;k__norank;p__Proteobacteria;c__Gammaproteobacteria;o__Enterobacterales;f__Erwiniaceae;g__Pantoea;s__Pantoea_deleyi
PB1_gene_109	433	ORM81271.1	433	422/433(97.46)	6.40e-311	16S rRNA (cytosine(967)-C(5))-methyltransferase [Pantoea	470932	d__Bacteria;k__norank;p__Proteobacteria;c__Gammaproteobacteria;o__Enterobacterales;f__Erwiniaceae;g__Pantoea;s__Pantoea_deleyi
PB1_gene_110	458	ORM81270.1	458	458/458(100.00)	0	Trk system potassium transport protein TrkA [Pantoea deleyi]	470932	d__Bacteria;k__norank;p__Proteobacteria;c__Gammaproteobacteria;o__Enterobacterales;f__Erwiniaceae;g__Pantoea;s__Pantoea_deleyi
PB1_gene_111	158	ORM81269.1	136	136/136(100.00)	1.03e-85	large-conductance mechanosensitive channel	470932	d__Bacteria;k__norank;p__Proteobacteria;c__Gammaproteobacteria;o__Enterobacterales;f__Erwiniaceae;g__Pantoea;s__Pantoea_deleyi
PB1_gene_112	69	ORM81268.1	69	68/69(98.55)	3.16e-41	hypothetical protein HA42_11415 [Pantoea deleyi]	470932	d__Bacteria;k__norank;p__Proteobacteria;c__Gammaproteobacteria;o__Enterobacterales;f__Erwiniaceae;g__Pantoea;s__Pantoea_deleyi
PB1_gene_113	138	ORM81267.1	138	137/138(99.28)	1.65e-93	zinc-responsive transcriptional regulator [Pantoea deleyi]	470932	d__Bacteria;k__norank;p__Proteobacteria;c__Gammaproteobacteria;o__Enterobacterales;f__Erwiniaceae;g__Pantoea;s__Pantoea_deleyi
PB1_gene_114	123	ORM81266.1	123	120/123(97.56)	3.65e-83	hypothetical protein HA42_11405 [Pantoea deleyi]	470932	d__Bacteria;k__norank;p__Proteobacteria;c__Gammaproteobacteria;o__Enterobacterales;f__Erwiniaceae;g__Pantoea;s__Pantoea_deleyi
PB1_gene_115	128	OON35514.1	128	128/128(100.00)	3.58e-81	50S ribosomal protein L17 [Izhakiella australiensis]	1926881	d__Bacteria;k__norank;p__Proteobacteria;c__Gammaproteobacteria;o__Enterobacterales;f__Erwiniaceae;g__Izhakiella;s__Izhakiella_australiensis
PB1_gene_117	293	ORM80833.1	293	293/293(100.00)	6.51e-206	magnesium/cobalt efflux protein [Pantoea deleyi]	470932	d__Bacteria;k__norank;p__Proteobacteria;c__Gammaproteobacteria;o__Enterobacterales;f__Erwiniaceae;g__Pantoea;s__Pantoea_deleyi
PB1_gene_118	157	WP_111142185.1	157	157/157(100.00)	1.46e-112	rRNA maturation RNase YbeY [Pantoea sp. ARC607]	2027922	d__Bacteria;k__norank;p__Proteobacteria;c__Gammaproteobacteria;o__Enterobacterales;f__Erwiniaceae;g__Pantoea;s__Pantoea_sp._ARC607
PB1_gene_119	353	ORM80835.1	353	351/353(99.43)	2.90e-246	nucleoside triphosphate hydrolase [Pantoea deleyi]	470932	d__Bacteria;k__norank;p__Proteobacteria;c__Gammaproteobacteria;o__Enterobacterales;f__Erwiniaceae;g__Pantoea;s__Pantoea_deleyi

PB1_gene_120	474	ORM80836.1	474	473/474(99.79)	0	tRNA (N6-isopentenyl adenosine(37)-C2)-methylthiotransferase MiaB [Pantoea deleyi]	470932	d__Bacteria;k__norank;p__Proteobacteria;c__Gammaproteobacteria;o__Enterobacterales;f__Erwiniaceae;g__Pantoea;s__Pantoea_deleyi
PB1_gene_121	390	ORM80837.1	390	383/390(98.21)	2.46e-284	2-octaprenyl-3-methyl-6-methoxy-1,4-benzoquinol hydroxylase [Pantoea deleyi]	470932	d__Bacteria;k__norank;p__Proteobacteria;c__Gammaproteobacteria;o__Enterobacterales;f__Erwiniaceae;g__Pantoea;s__Pantoea_deleyi
PB1_gene_124	345	ERM08342.1	345	338/345(97.97)	8.26e-242	transcriptional regulator [Pantoea agglomerans Tx10]	1332072	d__Bacteria;k__norank;p__Proteobacteria;c__Gammaproteobacteria;o__Enterobacterales;f__Erwiniaceae;g__Pantoea;s__Pantoea_agglomerans
PB1_gene_125	462	ORM76685.1	462	451/462(97.62)	0	MFS transporter [Pantoea deleyi]	470932	d__Bacteria;k__norank;p__Proteobacteria;c__Gammaproteobacteria;o__Enterobacterales;f__Erwiniaceae;g__Pantoea;s__Pantoea_deleyi
PB1_gene_126	206	WP_111138487.1	184	176/184(95.65)	2.03e-122	histidine phosphatase family protein [Pantoea sp. ARC607]	2027922	d__Bacteria;k__norank;p__Proteobacteria;c__Gammaproteobacteria;o__Enterobacterales;f__Erwiniaceae;g__Pantoea;s__Pantoea_sp._ARC607
PB1_gene_127	321	WP_111138488.1	321	314/321(97.82)	1.53e-234	WD40 repeat domain-containing protein [Pantoea sp. ARC607]	2027922	d__Bacteria;k__norank;p__Proteobacteria;c__Gammaproteobacteria;o__Enterobacterales;f__Erwiniaceae;g__Pantoea;s__Pantoea_sp._ARC607
PB1_gene_128	313	ORM75327.1	337	305/313(97.44)	9.40e-224	mannosyltransferase [Pantoea deleyi]	470932	d__Bacteria;k__norank;p__Proteobacteria;c__Gammaproteobacteria;o__Enterobacterales;f__Erwiniaceae;g__Pantoea;s__Pantoea_deleyi
PB1_gene_129	306	WP_111138556.1	305	294/305(96.39)	1.18e-212	LysR family transcriptional regulator [Pantoea sp. ARC607]	2027922	d__Bacteria;k__norank;p__Proteobacteria;c__Gammaproteobacteria;o__Enterobacterales;f__Erwiniaceae;g__Pantoea;s__Pantoea_sp._ARC607
PB1_gene_130	486	WP_111140942.1	486	472/486(97.12)	0	ATPase RavA stimulator ViaA [Pantoea sp. ARC607]	2027922	d__Bacteria;k__norank;p__Proteobacteria;c__Gammaproteobacteria;o__Enterobacterales;f__Erwiniaceae;g__Pantoea;s__Pantoea_sp._ARC607
PB1_gene_131	498	ORM85489.1	498	487/498(97.79)	0	ATPase RavA [Pantoea deleyi]	470932	d__Bacteria;k__norank;p__Proteobacteria;c__Gammaproteobacteria;o__Enterobacterales;f__Erwiniaceae;g__Pantoea;s__Pantoea_deleyi
PB1_gene_132	622	ORM85449.1	622	621/622(99.84)	0	potassium transporter Kup [Pantoea deleyi]	470932	d__Bacteria;k__norank;p__Proteobacteria;c__Gammaproteobacteria;o__Enterobacterales;f__Erwiniaceae;g__Pantoea;s__Pantoea_deleyi
PB1_gene_133	443	WP_111140940.1	443	437/443(98.65)	3.44e-311	two-component system sensor histidine kinase QseC [Pantoea sp. ARC607]	2027922	d__Bacteria;k__norank;p__Proteobacteria;c__Gammaproteobacteria;o__Enterobacterales;f__Erwiniaceae;g__Pantoea;s__Pantoea_sp._ARC607
PB1_gene_134	225	WP_111140939.1	223	217/223(97.31)	1.25e-155	response regulator [Pantoea sp. ARC607]	2027922	d__Bacteria;k__norank;p__Proteobacteria;c__Gammaproteobacteria;o__Enterobacterales;f__Erwiniaceae;g__Pantoea;s__Pantoea_sp._ARC607
PB1_gene_136	158	ORM72663.1	149	134/149(89.93)	1.98e-95	GNAT family N-acetyltransferase [Pantoea deleyi]	470932	d__Bacteria;k__norank;p__Proteobacteria;c__Gammaproteobacteria;o__Enterobacterales;f__Erwiniaceae;g__Pantoea;s__Pantoea_deleyi
PB1_gene_137	263	ORM72630.1	261	230/254(90.55)	3.11e-168	hypothetical protein HA42_22200 [Pantoea deleyi]	470932	d__Bacteria;k__norank;p__Proteobacteria;c__Gammaproteobacteria;o__Enterobacterales;f__Erwiniaceae;g__Pantoea;s__Pantoea_deleyi
PB1_gene_138	652	WP_111138952.1	550	531/550(96.55)	3.26e-285	HAMP domain-containing protein [Pantoea sp. ARC607]	2027922	d__Bacteria;k__norank;p__Proteobacteria;c__Gammaproteobacteria;o__Enterobacterales;f__Erwiniaceae;g__Pantoea;s__Pantoea_sp._ARC607
PB1_gene_139	89	ORM61343.1	130	56/72(77.78)	1.91e-34	amino acid-binding protein [Pantoea rodasii]	1076549	d__Bacteria;k__norank;p__Proteobacteria;c__Gammaproteobacteria;o__Enterobacterales;f__Erwiniaceae;g__Pantoea;s__Pantoea_rodasii
PB1_gene_140	418	WP_111138951.1	418	401/418(95.93)	9.09e-276	MFS transporter [Pantoea sp. ARC607]	2027922	d__Bacteria;k__norank;p__Proteobacteria;c__Gammaproteobacteria;o__Enterobacterales;f__Erwiniaceae;g__Pantoea;s__Pantoea_sp._ARC607
PB1_gene_142	150	AIX48840.1	291	117/143(81.82)	4.07e-75	phage head morphogenesis protein [Pantoea sp. PSNIH1]	1484158	d__Bacteria;k__norank;p__Proteobacteria;c__Gammaproteobacteria;o__Enterobacterales;f__Erwiniaceae;g__Pantoea;s__Pantoea_sp._PSNIH1
PB1_gene_145	261	ORM84155.1	292	253/261(96.93)	1.40e-180	LysR family transcriptional regulator [Pantoea deleyi]	470932	d__Bacteria;k__norank;p__Proteobacteria;c__Gammaproteobacteria;o__Enterobacterales;f__Erwiniaceae;g__Pantoea;s__Pantoea_deleyi
PB1_gene_148	471	ORM84158.1	471	464/471(98.51)	0	GABA permease [Pantoea deleyi]	470932	d__Bacteria;k__norank;p__Proteobacteria;c__Gammaproteobacteria;o__Enterobacterales;f__Erwiniaceae;g__Pantoea;s__Pantoea_deleyi
PB1_gene_149	428	WP_111139558.1	428	424/428(99.07)	6.31e-304	4-aminobutyrate--2-oxoglutarate transaminase [Pantoea sp.	2027922	d__Bacteria;k__norank;p__Proteobacteria;c__Gammaproteobacteria;o__Enterobacterales;f__Erwiniaceae;g__Pantoea;s__Pantoea_sp._ARC607
PB1_gene_154	506	ORM83852.1	506	505/505(100.00)	0	NADH-quinone oxidoreductase subunit M [Pantoea deleyi]	470932	d__Bacteria;k__norank;p__Proteobacteria;c__Gammaproteobacteria;o__Enterobacterales;f__Erwiniaceae;g__Pantoea;s__Pantoea_deleyi

PB1_gene_156	611	ORM83853.1	611	608/611(99.51)	0	NADH-quinone oxidoreductase subunit L [Pantoea deleyi]	470932	d__Bacteria;k__norank;p__Proteobacteria;c__Gammaproteobacteria;o__Enterobacterales;f__Erwiniaceae;g__Pantoea;s__Pantoea_deleyi
PB1_gene_157	100	SJZ65828.1	100	100/100(100.00)	1.31e-54	NADH dehydrogenase subunit K [Pantoea agglomerans]	470933	d__Bacteria;k__norank;p__Proteobacteria;c__Gammaproteobacteria;o__Enterobacterales;f__Erwiniaceae;g__Pantoea;s__Pantoea_eucalypti
PB1_gene_158	183	ORM83855.1	183	183/183(100.00)	1.88e-114	NADH:ubiquinone oxidoreductase subunit J [Pantoea	470932	d__Bacteria;k__norank;p__Proteobacteria;c__Gammaproteobacteria;o__Enterobacterales;f__Erwiniaceae;g__Pantoea;s__Pantoea_deleyi
PB1_gene_159	180	ORM83856.1	180	180/180(100.00)	1.98e-119	NADH-quinone oxidoreductase subunit I [Pantoea deleyi]	470932	d__Bacteria;k__norank;p__Proteobacteria;c__Gammaproteobacteria;o__Enterobacterales;f__Erwiniaceae;g__Pantoea;s__Pantoea_deleyi
PB1_gene_160	325	ORM83857.1	325	325/325(100.00)	8.15e-232	NADH-quinone oxidoreductase subunit H [Pantoea deleyi]	470932	d__Bacteria;k__norank;p__Proteobacteria;c__Gammaproteobacteria;o__Enterobacterales;f__Erwiniaceae;g__Pantoea;s__Pantoea_deleyi
PB1_gene_163	339	WP_111139637.1	339	321/339(94.69)	1.12e-236	isopenicillin N synthase family oxygenase [Pantoea sp. ARC607]	2027922	d__Bacteria;k__norank;p__Proteobacteria;c__Gammaproteobacteria;o__Enterobacterales;f__Erwiniaceae;g__Pantoea;s__Pantoea_sp._ARC607
PB1_gene_164	268	WP_111139638.1	267	265/267(99.25)	1.34e-183	ABC transporter substrate-binding protein [Pantoea sp.	2027922	d__Bacteria;k__norank;p__Proteobacteria;c__Gammaproteobacteria;o__Enterobacterales;f__Erwiniaceae;g__Pantoea;s__Pantoea_sp._ARC607
PB1_gene_165	262	WP_111139639.1	262	249/262(95.04)	5.36e-169	ATP-binding cassette domain-containing protein [Pantoea sp. ARC607]	2027922	d__Bacteria;k__norank;p__Proteobacteria;c__Gammaproteobacteria;o__Enterobacterales;f__Erwiniaceae;g__Pantoea;s__Pantoea_sp._ARC607
PB1_gene_166	219	WP_098050175.1	222	212/219(96.80)	1.26e-135	ABC transporter permease [Pantoea agglomerans]	None	Unclassified
PB1_gene_167	294	ORM79689.1	296	294/294(100.00)	1.35e-199	maltose transporter permease [Pantoea deleyi]	470932	d__Bacteria;k__norank;p__Proteobacteria;c__Gammaproteobacteria;o__Enterobacterales;f__Erwiniaceae;g__Pantoea;s__Pantoea_deleyi
PB1_gene_170	775	ORM76788.1	784	756/775(97.55)	0	quinoprotein [Pantoea deleyi]	470932	d__Bacteria;k__norank;p__Proteobacteria;c__Gammaproteobacteria;o__Enterobacterales;f__Erwiniaceae;g__Pantoea;s__Pantoea_deleyi
PB1_gene_171	326	ORM76787.1	326	321/326(98.47)	9.99e-238	hypothetical protein HA42_18705 [Pantoea deleyi]	470932	d__Bacteria;k__norank;p__Proteobacteria;c__Gammaproteobacteria;o__Enterobacterales;f__Erwiniaceae;g__Pantoea;s__Pantoea_deleyi
PB1_gene_172	346	WP_111140368.1	346	341/346(98.55)	7.33e-241	ABC transporter substrate-binding protein [Pantoea sp.	2027922	d__Bacteria;k__norank;p__Proteobacteria;c__Gammaproteobacteria;o__Enterobacterales;f__Erwiniaceae;g__Pantoea;s__Pantoea_sp._ARC607
PB1_gene_176	89	ORM80861.1	89	88/89(98.88)	2.32e-52	hypothetical protein HA42_12590 [Pantoea deleyi]	470932	d__Bacteria;k__norank;p__Proteobacteria;c__Gammaproteobacteria;o__Enterobacterales;f__Erwiniaceae;g__Pantoea;s__Pantoea_deleyi
PB1_gene_177	258	ORM80860.1	258	253/258(98.06)	1.25e-181	pimeloyl-[acyl-carrier protein] methyl ester esterase [Pantoea	470932	d__Bacteria;k__norank;p__Proteobacteria;c__Gammaproteobacteria;o__Enterobacterales;f__Erwiniaceae;g__Pantoea;s__Pantoea_deleyi
PB1_gene_178	214	ORM80859.1	226	206/214(96.26)	4.07e-152	DNA utilization protein GntX [Pantoea deleyi]	470932	d__Bacteria;k__norank;p__Proteobacteria;c__Gammaproteobacteria;o__Enterobacterales;f__Erwiniaceae;g__Pantoea;s__Pantoea_deleyi
PB1_gene_179	191	ORM80858.1	191	191/191(100.00)	2.21e-135	Fe-S biogenesis protein NfuA [Pantoea deleyi]	470932	d__Bacteria;k__norank;p__Proteobacteria;c__Gammaproteobacteria;o__Enterobacterales;f__Erwiniaceae;g__Pantoea;s__Pantoea_deleyi
PB1_gene_182	1035	WP_125289964.1	1035	802/1035(77.49)	0	putative selenate reductase subunit YgfK [Erwinia sp. 198]	2022746	d__Bacteria;k__norank;p__Proteobacteria;c__Gammaproteobacteria;o__Enterobacterales;f__Erwiniaceae;g__Erwinia;s__Erwinia_sp._198
PB1_gene_183	270	WP_103058950.1	283	173/256(67.58)	5.95e-115	XdhC family protein [Mixta theicola]	1458355	d__Bacteria;k__norank;p__Proteobacteria;c__Gammaproteobacteria;o__Enterobacterales;f__Erwiniaceae;g__Mixta;s__Mixta_theicola
PB1_gene_184	310	WP_125289963.1	310	247/310(79.68)	6.50e-171	carbamate kinase [Erwinia sp. 198]	2022746	d__Bacteria;k__norank;p__Proteobacteria;c__Gammaproteobacteria;o__Enterobacterales;f__Erwiniaceae;g__Erwinia;s__Erwinia_sp._198
PB1_gene_187	300	WP_111138297.1	300	297/300(99.00)	1.84e-229	4-deoxy-4-formamido-L-arabinose-phosphoundecaprenol deformylase [Pantoea sp.	2027922	d__Bacteria;k__norank;p__Proteobacteria;c__Gammaproteobacteria;o__Enterobacterales;f__Erwiniaceae;g__Pantoea;s__Pantoea_sp._ARC607
PB1_gene_188	555	WP_111138298.1	555	517/555(93.15)	0	lipid IV(A) 4-amino-4-deoxy-L-arabinosyltransferase [Pantoea sp. ARC607]	2027922	d__Bacteria;k__norank;p__Proteobacteria;c__Gammaproteobacteria;o__Enterobacterales;f__Erwiniaceae;g__Pantoea;s__Pantoea_sp._ARC607
PB1_gene_189	107	ORM79331.1	106	102/106(96.23)	1.60e-58	4-amino-4-deoxy-L-arabinose-phospho-UDP flippase [Pantoea deleyi]	470932	d__Bacteria;k__norank;p__Proteobacteria;c__Gammaproteobacteria;o__Enterobacterales;f__Erwiniaceae;g__Pantoea;s__Pantoea_deleyi

PB1_gene_190	121	ORM79323.1	121	121/121(100.00)	1.97e-77	4-amino-4-deoxy-L-arabinose-phospho-UDP flippase [Pantoea deleyi]	470932	d__Bacteria;k__norank;p__Proteobacteria;c__Gammaproteobacteria;o__Enterobacterales;f__Erwiniaceae;g__Pantoea;s__Pantoea_deleyi
PB1_gene_191	508	ORM79324.1	508	496/508(97.64)	0	cell filamentation protein Fic [Pantoea deleyi]	470932	d__Bacteria;k__norank;p__Proteobacteria;c__Gammaproteobacteria;o__Enterobacterales;f__Erwiniaceae;g__Pantoea;s__Pantoea_deleyi
PB1_gene_193	191	PZL85810.1	191	191/191(100.00)	2.48e-132	bifunctional murein DD-endopeptidase/murein LD-carboxypeptidase [Pantoea sp. ARC270]	2027923	d__Bacteria;k__norank;p__Proteobacteria;c__Gammaproteobacteria;o__Enterobacterales;f__Erwiniaceae;g__Pantoea;s__Pantoea_sp._ARC270
PB1_gene_194	235	ORM81389.1	235	232/235(98.72)	1.02e-164	hypothetical protein HA42_11010 [Pantoea deleyi]	470932	d__Bacteria;k__norank;p__Proteobacteria;c__Gammaproteobacteria;o__Enterobacterales;f__Erwiniaceae;g__Pantoea;s__Pantoea_deleyi
PB1_gene_195	323	WP_111138746.1	322	308/322(95.65)	4.35e-228	GTP-binding protein [Pantoea sp. ARC607]	2027922	d__Bacteria;k__norank;p__Proteobacteria;c__Gammaproteobacteria;o__Enterobacterales;f__Erwiniaceae;g__Pantoea;s__Pantoea_sp._ARC607
PB1_gene_196	190	ORM75533.1	190	190/190(100.00)	1.55e-130	elongation factor P-like protein YeiP [Pantoea deleyi]	470932	d__Bacteria;k__norank;p__Proteobacteria;c__Gammaproteobacteria;o__Enterobacterales;f__Erwiniaceae;g__Pantoea;s__Pantoea_deleyi
PB1_gene_197	62	ORM75534.1	84	60/62(96.77)	1.66e-37	hypothetical protein HA42_20430 [Pantoea deleyi]	470932	d__Bacteria;k__norank;p__Proteobacteria;c__Gammaproteobacteria;o__Enterobacterales;f__Erwiniaceae;g__Pantoea;s__Pantoea_deleyi
PB1_gene_198	435	WP_111138749.1	376	373/376(99.20)	1.36e-255	fused PTS fructose transporter subunit IIA/HPr protein [Pantoea sp. ARC607]	2027922	d__Bacteria;k__norank;p__Proteobacteria;c__Gammaproteobacteria;o__Enterobacterales;f__Erwiniaceae;g__Pantoea;s__Pantoea_sp._ARC607
PB1_gene_199	312	ORM75536.1	312	312/312(100.00)	6.15e-225	1-phosphofructokinase [Pantoea deleyi]	470932	d__Bacteria;k__norank;p__Proteobacteria;c__Gammaproteobacteria;o__Enterobacterales;f__Erwiniaceae;g__Pantoea;s__Pantoea_deleyi
PB1_gene_202	481	ORM83377.1	481	478/481(99.38)	0	metalloprotease TldD [Pantoea deleyi]	470932	d__Bacteria;k__norank;p__Proteobacteria;c__Gammaproteobacteria;o__Enterobacterales;f__Erwiniaceae;g__Pantoea;s__Pantoea_deleyi
PB1_gene_203	1268	WP_111139671.1	1275	1213/1268(95.66)	0	AsmA2 domain-containing protein [Pantoea sp. ARC607]	2027922	d__Bacteria;k__norank;p__Proteobacteria;c__Gammaproteobacteria;o__Enterobacterales;f__Erwiniaceae;g__Pantoea;s__Pantoea_sp._ARC607
PB1_gene_206	399	ORM80429.1	399	394/399(98.75)	1.24e-293	S-methyl-5-thioribose kinase [Pantoea deleyi]	470932	d__Bacteria;k__norank;p__Proteobacteria;c__Gammaproteobacteria;o__Enterobacterales;f__Erwiniaceae;g__Pantoea;s__Pantoea_deleyi
PB1_gene_207	340	WP_115758903.1	340	320/340(94.12)	3.41e-227	S-methyl-5-thioribose-1-phosphate isomerase [Pantoea	53335	d__Bacteria;k__norank;p__Proteobacteria;c__Gammaproteobacteria;o__Enterobacterales;f__Erwiniaceae;g__Pantoea;s__norank
PB1_gene_208	180	SJZ29947.1	180	179/180(99.44)	5.30e-133	1,2-dihydroxy-3-keto-5-methylthiopentene dioxygenase [Pantoea agglomerans]	470933	d__Bacteria;k__norank;p__Proteobacteria;c__Gammaproteobacteria;o__Enterobacterales;f__Erwiniaceae;g__Pantoea;s__Pantoea_eucalypti
PB1_gene_209	227	ORM75993.1	227	218/227(96.04)	7.47e-154	2,3-diketo-5-methylthio-1-phosphopentane phosphatase [Pantoea deleyi]	470932	d__Bacteria;k__norank;p__Proteobacteria;c__Gammaproteobacteria;o__Enterobacterales;f__Erwiniaceae;g__Pantoea;s__Pantoea_deleyi
PB1_gene_213	230	WP_111138438.1	213	199/213(93.43)	5.16e-138	DUF1109 domain-containing protein [Pantoea sp. ARC607]	2027922	d__Bacteria;k__norank;p__Proteobacteria;c__Gammaproteobacteria;o__Enterobacterales;f__Erwiniaceae;g__Pantoea;s__Pantoea_sp._ARC607
PB1_gene_214	170	ORM80590.1	183	161/170(94.71)	2.12e-103	RNA polymerase subunit sigma-70 [Pantoea deleyi]	470932	d__Bacteria;k__norank;p__Proteobacteria;c__Gammaproteobacteria;o__Enterobacterales;f__Erwiniaceae;g__Pantoea;s__Pantoea_deleyi
PB1_gene_215	413	ORM80589.1	413	406/412(98.54)	4.15e-274	cytochrome C biogenesis protein [Pantoea deleyi]	470932	d__Bacteria;k__norank;p__Proteobacteria;c__Gammaproteobacteria;o__Enterobacterales;f__Erwiniaceae;g__Pantoea;s__Pantoea_deleyi
PB1_gene_217	402	WP_111138433.1	402	397/402(98.76)	1.75e-290	DUF4432 family protein [Pantoea sp. ARC607]	2027922	d__Bacteria;k__norank;p__Proteobacteria;c__Gammaproteobacteria;o__Enterobacterales;f__Erwiniaceae;g__Pantoea;s__Pantoea_sp._ARC607
PB1_gene_218	155	WP_111138432.1	169	149/155(96.13)	3.37e-102	DUF3237 family protein [Pantoea sp. ARC607]	2027922	d__Bacteria;k__norank;p__Proteobacteria;c__Gammaproteobacteria;o__Enterobacterales;f__Erwiniaceae;g__Pantoea;s__Pantoea_sp._ARC607
PB1_gene_221	133	ORM86310.1	133	133/133(100.00)	1.81e-90	hypothetical protein HA42_00820 [Pantoea deleyi]	470932	d__Bacteria;k__norank;p__Proteobacteria;c__Gammaproteobacteria;o__Enterobacterales;f__Erwiniaceae;g__Pantoea;s__Pantoea_deleyi
PB1_gene_222	387	EFM20180.1	387	377/387(97.42)	4.70e-276	AFG1-family ATPase [Pantoea sp. aB]	517433	d__Bacteria;k__norank;p__Proteobacteria;c__Gammaproteobacteria;o__Enterobacterales;f__Erwiniaceae;g__Pantoea;s__Pantoea_sp._aB

PB1_gene_223	142	ORM86311.1	142	142/142(100.00)	2.51e-99	50S ribosomal protein L13 [Pantoea deleyi]	470932	d__Bacteria;k__norank;p__Proteobacteria;c__Gammaproteobacteria;o__Enterobacterales;f__Erwiniaceae;g__Pantoea;s__Pantoea_deleyi
PB1_gene_224	130	SJZ88743.1	130	130/130(100.00)	6.14e-83	SSU ribosomal protein S9P [Pantoea agglomerans]	470933	d__Bacteria;k__norank;p__Proteobacteria;c__Gammaproteobacteria;o__Enterobacterales;f__Erwiniaceae;g__Pantoea;s__Pantoea_eucalypti
PB1_gene_225	213	OQV39861.1	213	213/213(100.00)	9.56e-150	stringent starvation protein A [Pantoea vagans]	470934	d__Bacteria;k__norank;p__Proteobacteria;c__Gammaproteobacteria;o__Enterobacterales;f__Erwiniaceae;g__Pantoea;s__Pantoea_vagans
PB1_gene_226	161	ORM86314.1	161	147/161(91.30)	3.00e-98	ClpXP protease specificity-enhancing factor [Pantoea deleyi]	470932	d__Bacteria;k__norank;p__Proteobacteria;c__Gammaproteobacteria;o__Enterobacterales;f__Erwiniaceae;g__Pantoea;s__Pantoea_deleyi
PB1_gene_229	211	WP_090962688.1	211	210/211(99.53)	4.06e-150	carbonic anhydrase [Pantoea sp. OV426]	1881036	d__Bacteria;k__norank;p__Proteobacteria;c__Gammaproteobacteria;o__Enterobacterales;f__Erwiniaceae;g__Pantoea;s__Pantoea_sp._OV426
PB1_gene_230	151	ORM81997.1	149	148/149(99.33)	5.20e-105	cytoplasmic protein [Pantoea deleyi]	470932	d__Bacteria;k__norank;p__Proteobacteria;c__Gammaproteobacteria;o__Enterobacterales;f__Erwiniaceae;g__Pantoea;s__Pantoea_deleyi
PB1_gene_231	301	WP_111140550.1	301	298/301(99.00)	5.27e-200	glutathione ABC transporter permease GsiD [Pantoea sp. ARC607]	2027922	d__Bacteria;k__norank;p__Proteobacteria;c__Gammaproteobacteria;o__Enterobacterales;f__Erwiniaceae;g__Pantoea;s__Pantoea_sp._ARC607
PB1_gene_232	309	WP_111140551.1	309	308/309(99.68)	1.21e-209	glutathione ABC transporter permease GsiC [Pantoea sp.	2027922	d__Bacteria;k__norank;p__Proteobacteria;c__Gammaproteobacteria;o__Enterobacterales;f__Erwiniaceae;g__Pantoea;s__Pantoea_sp._ARC607
PB1_gene_233	510	ORM81993.1	510	501/510(98.24)	0	glutathione ABC transporter substrate-binding protein GsiB [Pantoea deleyi]	470932	d__Bacteria;k__norank;p__Proteobacteria;c__Gammaproteobacteria;o__Enterobacterales;f__Erwiniaceae;g__Pantoea;s__Pantoea_deleyi
PB1_gene_236	237	SKA14850.1	237	234/237(98.73)	2.59e-159	branched-chain amino acid transport system ATP-binding protein [Pantoea agglomerans]	470933	d__Bacteria;k__norank;p__Proteobacteria;c__Gammaproteobacteria;o__Enterobacterales;f__Erwiniaceae;g__Pantoea;s__Pantoea_eucalypti
PB1_gene_237	255	ORM80405.1	255	253/255(99.22)	3.17e-178	high-affinity branched-chain amino acid ABC transporter ATP-binding protein LivG	470932	d__Bacteria;k__norank;p__Proteobacteria;c__Gammaproteobacteria;o__Enterobacterales;f__Erwiniaceae;g__Pantoea;s__Pantoea_deleyi
PB1_gene_238	423	ORM80406.1	423	422/423(99.76)	9.04e-290	high-affinity branched-chain amino acid ABC transporter permease LivM [Pantoea deleyi]	470932	d__Bacteria;k__norank;p__Proteobacteria;c__Gammaproteobacteria;o__Enterobacterales;f__Erwiniaceae;g__Pantoea;s__Pantoea_deleyi
PB1_gene_239	308	ORM80407.1	308	308/308(100.00)	3.83e-185	branched-chain amino acid ABC transporter permease LivH [Pantoea deleyi]	470932	d__Bacteria;k__norank;p__Proteobacteria;c__Gammaproteobacteria;o__Enterobacterales;f__Erwiniaceae;g__Pantoea;s__Pantoea_deleyi
PB1_gene_240	370	WP_111140406.1	370	369/370(99.73)	1.60e-263	branched-chain amino acid ABC transporter substrate-binding protein [Pantoea sp. ARC607]	2027922	d__Bacteria;k__norank;p__Proteobacteria;c__Gammaproteobacteria;o__Enterobacterales;f__Erwiniaceae;g__Pantoea;s__Pantoea_sp._ARC607
PB1_gene_243	280	ORM82634.1	280	277/280(98.93)	5.59e-205	23S rRNA (adenine(2030)-N(6))-methyltransferase RlmJ [Pantoea deleyi]	470932	d__Bacteria;k__norank;p__Proteobacteria;c__Gammaproteobacteria;o__Enterobacterales;f__Erwiniaceae;g__Pantoea;s__Pantoea_deleyi
PB1_gene_244	272	WP_086906636.1	272	231/272(84.93)	1.25e-156	CPBP family intramembrane metalloprotease [Pantoea agglomerans]	549	d__Bacteria;k__norank;p__Proteobacteria;c__Gammaproteobacteria;o__Enterobacterales;f__Erwiniaceae;g__Pantoea;s__Pantoea_agglomerans
PB1_gene_245	303	ORM75390.1	303	295/303(97.36)	7.41e-217	ribonuclease Z [Pantoea deleyi]	470932	d__Bacteria;k__norank;p__Proteobacteria;c__Gammaproteobacteria;o__Enterobacterales;f__Erwiniaceae;g__Pantoea;s__Pantoea_deleyi
PB1_gene_246	493	WP_111139297.1	450	445/450(98.89)	0	glutathione-disulfide reductase [Pantoea sp. ARC607]	2027922	d__Bacteria;k__norank;p__Proteobacteria;c__Gammaproteobacteria;o__Enterobacterales;f__Erwiniaceae;g__Pantoea;s__Pantoea_sp._ARC607
PB1_gene_249	438	ORM80984.1	436	432/436(99.08)	0	alcohol dehydrogenase [Pantoea deleyi]	470932	d__Bacteria;k__norank;p__Proteobacteria;c__Gammaproteobacteria;o__Enterobacterales;f__Erwiniaceae;g__Pantoea;s__Pantoea_deleyi
PB1_gene_250	594	ORM80981.1	594	588/594(98.99)	0	GMC family oxidoreductase [Pantoea deleyi]	470932	d__Bacteria;k__norank;p__Proteobacteria;c__Gammaproteobacteria;o__Enterobacterales;f__Erwiniaceae;g__Pantoea;s__Pantoea_deleyi

PB1_gene_251	244	WP_111141273.1	244	240/244(98.36)	9.85e-172	gluconate 2-dehydrogenase subunit 3 family protein [Pantoea sp. ARC607]	2027922	d__Bacteria;k__norank;p__Proteobacteria;c__Gammaproteobacteria;o__Enterobacterales;f__Erwiniaceae;g__Pantoea;s__Pantoea_sp._ARC607
PB1_gene_252	413	ORM76004.1	413	411/413(99.52)	1.64e-296	microcin B17 transporter [Pantoea deleyi]	470932	d__Bacteria;k__norank;p__Proteobacteria;c__Gammaproteobacteria;o__Enterobacterales;f__Erwiniaceae;g__Pantoea;s__Pantoea_deleyi
PB1_gene_253	69	SJZ90619.1	69	69/69(100.00)	6.96e-36	Protein of unknown function [Pantoea agglomerans]	470933	d__Bacteria;k__norank;p__Proteobacteria;c__Gammaproteobacteria;o__Enterobacterales;f__Erwiniaceae;g__Pantoea;s__Pantoea_eucalypti
PB1_gene_255	313	WP_111140476.1	309	305/309(98.71)	3.56e-219	DNA replication terminus site-binding protein [Pantoea sp. ARC607]	2027922	d__Bacteria;k__norank;p__Proteobacteria;c__Gammaproteobacteria;o__Enterobacterales;f__Erwiniaceae;g__Pantoea;s__Pantoea_sp._ARC607
PB1_gene_256	465	ORM80748.1	465	461/465(99.14)	0	fumarate hydratase, class II [Pantoea deleyi]	470932	d__Bacteria;k__norank;p__Proteobacteria;c__Gammaproteobacteria;o__Enterobacterales;f__Erwiniaceae;g__Pantoea;s__Pantoea_deleyi
PB1_gene_257	391	ORM80747.1	391	368/391(94.12)	7.68e-268	mannose-6-phosphate isomerase [Pantoea deleyi]	470932	d__Bacteria;k__norank;p__Proteobacteria;c__Gammaproteobacteria;o__Enterobacterales;f__Erwiniaceae;g__Pantoea;s__Pantoea_deleyi
PB1_gene_262	307	ORM82729.1	307	303/307(98.70)	2.41e-224	lipid A biosynthesis palmitoleoyl acyltransferase [Pantoea deleyi]	470932	d__Bacteria;k__norank;p__Proteobacteria;c__Gammaproteobacteria;o__Enterobacterales;f__Erwiniaceae;g__Pantoea;s__Pantoea_deleyi
PB1_gene_264	154	WP_082804157.1	209	106/149(71.14)	3.79e-73	DUF943 family protein [Erwinia sp. ErVv1]	1603299	d__Bacteria;k__norank;p__Proteobacteria;c__Gammaproteobacteria;o__Enterobacterales;f__Erwiniaceae;g__Erwinia;s__Erwinia_sp._ErVv1
PB1_gene_265	153	WP_124234229.1	154	118/153(77.12)	1.05e-83	DUF943 family protein [Erwinia psidii]	69224	d__Bacteria;k__norank;p__Proteobacteria;c__Gammaproteobacteria;o__Enterobacterales;f__Erwiniaceae;g__Erwinia;s__Erwinia_psidii
PB1_gene_266	266	WP_084883551.1	280	211/263(80.23)	3.94e-158	DUF3289 family protein [Pantoea septica]	472695	d__Bacteria;k__norank;p__Proteobacteria;c__Gammaproteobacteria;o__Enterobacterales;f__Erwiniaceae;g__Pantoea;s__Pantoea_septica
PB1_gene_267	51	VCW25745.1	54	39/51(76.47)	5.56e-19	Putative transposase (identified by ISEscan HMM) [Klebsiella pneumoniae]	244366	d__Bacteria;k__norank;p__Proteobacteria;c__Gammaproteobacteria;o__Enterobacterales;f__Enterobacteriaceae;g__Klebsiella;s__Klebsiella_variicola
PB1_gene_268	99	AKA39112.1	99	95/99(95.96)	6.03e-55	transposase [Yersinia ruckeri]	29486	d__Bacteria;k__norank;p__Proteobacteria;c__Gammaproteobacteria;o__Enterobacterales;f__Yersiniaceae;g__Yersinia;s__Yersinia_ruckeri
PB1_gene_272	112	ORM72609.1	112	102/112(91.07)	5.64e-66	hypothetical protein HA42_22050 [Pantoea deleyi]	470932	d__Bacteria;k__norank;p__Proteobacteria;c__Gammaproteobacteria;o__Enterobacterales;f__Erwiniaceae;g__Pantoea;s__Pantoea_deleyi
PB1_gene_273	157	ORM72610.1	182	154/157(98.09)	8.85e-106	DNA-binding protein [Pantoea deleyi]	470932	d__Bacteria;k__norank;p__Proteobacteria;c__Gammaproteobacteria;o__Enterobacterales;f__Erwiniaceae;g__Pantoea;s__Pantoea_deleyi
PB1_gene_274	240	ORM72613.1	240	230/240(95.83)	3.04e-164	thiamine biosynthesis protein ThiJ [Pantoea deleyi]	470932	d__Bacteria;k__norank;p__Proteobacteria;c__Gammaproteobacteria;o__Enterobacterales;f__Erwiniaceae;g__Pantoea;s__Pantoea_deleyi
PB1_gene_275	395	ORM72614.1	395	384/395(97.22)	1.36e-279	N-acyl-L-amino acid amidohydrolase [Pantoea deleyi]	470932	d__Bacteria;k__norank;p__Proteobacteria;c__Gammaproteobacteria;o__Enterobacterales;f__Erwiniaceae;g__Pantoea;s__Pantoea_deleyi
PB1_gene_277	241	ADI78091.1	241	237/241(98.34)	6.91e-166	Putative ABC transport system, permease protein (plasmid) [Pantoea vagans C9-1]	712898	d__Bacteria;k__norank;p__Proteobacteria;c__Gammaproteobacteria;o__Enterobacterales;f__Erwiniaceae;g__Pantoea;s__Pantoea_vagans
PB1_gene_280	330	WP_111138923.1	300	291/300(97.00)	7.04e-210	LysR family transcriptional regulator [Pantoea sp. ARC607]	2027922	d__Bacteria;k__norank;p__Proteobacteria;c__Gammaproteobacteria;o__Enterobacterales;f__Erwiniaceae;g__Pantoea;s__Pantoea_sp._ARC607
PB1_gene_281	249	ORM72602.1	249	244/249(97.99)	2.69e-167	short-chain dehydrogenase [Pantoea deleyi]	470932	d__Bacteria;k__norank;p__Proteobacteria;c__Gammaproteobacteria;o__Enterobacterales;f__Erwiniaceae;g__Pantoea;s__Pantoea_deleyi
PB1_gene_282	432	ORM72603.1	432	420/432(97.22)	3.51e-303	MFS transporter permease [Pantoea deleyi]	470932	d__Bacteria;k__norank;p__Proteobacteria;c__Gammaproteobacteria;o__Enterobacterales;f__Erwiniaceae;g__Pantoea;s__Pantoea_deleyi
PB1_gene_283	280	WP_111138926.1	280	277/280(98.93)	8.89e-202	transketolase [Pantoea sp. ARC607]	2027922	d__Bacteria;k__norank;p__Proteobacteria;c__Gammaproteobacteria;o__Enterobacterales;f__Erwiniaceae;g__Pantoea;s__Pantoea_sp._ARC607
PB1_gene_284	336	ORM72604.1	336	324/335(96.72)	2.33e-232	transketolase [Pantoea deleyi]	470932	d__Bacteria;k__norank;p__Proteobacteria;c__Gammaproteobacteria;o__Enterobacterales;f__Erwiniaceae;g__Pantoea;s__Pantoea_deleyi
PB1_gene_285	950	ORM77127.1	950	943/950(99.26)	0	two-component system sensor histidine kinase/response regulator [Pantoea deleyi]	470932	d__Bacteria;k__norank;p__Proteobacteria;c__Gammaproteobacteria;o__Enterobacterales;f__Erwiniaceae;g__Pantoea;s__Pantoea_deleyi

PB1_gene_286	216	WP_111140094.1	216	216/216(100.00)	2.24e-145	transcriptional regulator RcsB [Pantoea sp. ARC607]	53335	d__Bacteria;k__norank;p__Proteobacteria;c__Gammaproteobacteria;o__Enterobacterales;f__Erwiniaceae;g__Pantoea;s__norank
PB1_gene_289	526	WP_111139160.1	526	523/526(99.43)	0	glutamine-hydrolyzing GMP synthase [Pantoea sp. ARC607]	2027922	d__Bacteria;k__norank;p__Proteobacteria;c__Gammaproteobacteria;o__Enterobacterales;f__Erwiniaceae;g__Pantoea;s__Pantoea_sp._ARC607
PB1_gene_290	257	WP_111139161.1	257	249/257(96.89)	2.83e-182	DUF3750 domain-containing protein [Pantoea sp. ARC607]	2027922	d__Bacteria;k__norank;p__Proteobacteria;c__Gammaproteobacteria;o__Enterobacterales;f__Erwiniaceae;g__Pantoea;s__Pantoea_sp._ARC607
PB1_gene_291	98	KDA92620.1	98	80/98(81.63)	1.49e-50	hypothetical protein T296_20160 [Pantoea agglomerans Eh318]	1408177	d__Bacteria;k__norank;p__Proteobacteria;c__Gammaproteobacteria;o__Enterobacterales;f__Erwiniaceae;g__Pantoea;s__Pantoea_agglomerans
PB1_gene_292	81	RZK09419.1	81	78/81(96.30)	9.88e-57	hypothetical protein EOO84_01105 [Pantoea sp.]	69393	d__Bacteria;k__norank;p__Proteobacteria;c__Gammaproteobacteria;o__Enterobacterales;f__Erwiniaceae;g__Pantoea;s__Pantoea_sp.
PB1_gene_293	172	ORM81016.1	165	161/165(97.58)	9.81e-110	DNA-binding protein [Pantoea deleyi]	470932	d__Bacteria;k__norank;p__Proteobacteria;c__Gammaproteobacteria;o__Enterobacterales;f__Erwiniaceae;g__Pantoea;s__Pantoea_deleyi
PB1_gene_295	428	ORM79488.1	427	423/427(99.06)	4.58e-283	uracil permease [Pantoea deleyi]	470932	d__Bacteria;k__norank;p__Proteobacteria;c__Gammaproteobacteria;o__Enterobacterales;f__Erwiniaceae;g__Pantoea;s__Pantoea_deleyi
PB1_gene_298	487	WP_111139326.1	487	483/487(99.18)	0	M48 family peptidase [Pantoea sp. ARC607]	2027922	d__Bacteria;k__norank;p__Proteobacteria;c__Gammaproteobacteria;o__Enterobacterales;f__Erwiniaceae;g__Pantoea;s__Pantoea_sp._ARC607
PB1_gene_299	201	ORM79484.1	201	200/201(99.50)	2.66e-141	sulfur reduction protein DsrE [Pantoea deleyi]	470932	d__Bacteria;k__norank;p__Proteobacteria;c__Gammaproteobacteria;o__Enterobacterales;f__Erwiniaceae;g__Pantoea;s__Pantoea_deleyi
PB1_gene_300	202	ORM79483.1	202	201/202(99.50)	1.42e-141	sulfur reduction protein DsrE [Pantoea deleyi]	470932	d__Bacteria;k__norank;p__Proteobacteria;c__Gammaproteobacteria;o__Enterobacterales;f__Erwiniaceae;g__Pantoea;s__Pantoea_deleyi
PB1_gene_302	296	ORM82055.1	296	293/296(98.99)	1.85e-202	cytidine deaminase [Pantoea deleyi]	470932	d__Bacteria;k__norank;p__Proteobacteria;c__Gammaproteobacteria;o__Enterobacterales;f__Erwiniaceae;g__Pantoea;s__Pantoea_deleyi
PB1_gene_303	231	ORM82071.1	230	228/230(99.13)	9.64e-145	CidB/LrgB family autolysis modulator [Pantoea deleyi]	470932	d__Bacteria;k__norank;p__Proteobacteria;c__Gammaproteobacteria;o__Enterobacterales;f__Erwiniaceae;g__Pantoea;s__Pantoea_deleyi
PB1_gene_304	130	ORM82056.1	130	128/130(98.46)	8.39e-81	murein hydrolase regulator LrgA [Pantoea deleyi]	470932	d__Bacteria;k__norank;p__Proteobacteria;c__Gammaproteobacteria;o__Enterobacterales;f__Erwiniaceae;g__Pantoea;s__Pantoea_deleyi
PB1_gene_308	255	WP_111139449.1	257	247/255(96.86)	2.61e-182	cyclic-guanylate-specific phosphodiesterase [Pantoea sp. ARC607]	2027922	d__Bacteria;k__norank;p__Proteobacteria;c__Gammaproteobacteria;o__Enterobacterales;f__Erwiniaceae;g__Pantoea;s__Pantoea_sp._ARC607
PB1_gene_309	686	WP_111139450.1	686	672/686(97.96)	0	AsmA family protein [Pantoea sp. ARC607]	2027922	d__Bacteria;k__norank;p__Proteobacteria;c__Gammaproteobacteria;o__Enterobacterales;f__Erwiniaceae;g__Pantoea;s__Pantoea_sp._ARC607
PB1_gene_310	93	ORM81459.1	93	91/93(97.85)	4.21e-59	peptidylprolyl isomerase [Pantoea deleyi]	470932	d__Bacteria;k__norank;p__Proteobacteria;c__Gammaproteobacteria;o__Enterobacterales;f__Erwiniaceae;g__Pantoea;s__Pantoea_deleyi
PB1_gene_311	673	ORM81458.1	673	670/672(99.70)	0	ATP-dependent DNA helicase Rep [Pantoea deleyi]	470932	d__Bacteria;k__norank;p__Proteobacteria;c__Gammaproteobacteria;o__Enterobacterales;f__Erwiniaceae;g__Pantoea;s__Pantoea_deleyi
PB1_gene_313	72	OLU26611.1	72	72/72(100.00)	1.71e-44	translation initiation factor IF-1 [Klebsiella michiganensis]	1134687	d__Bacteria;k__norank;p__Proteobacteria;c__Gammaproteobacteria;o__Enterobacterales;f__Enterobacteriaceae;g__Klebsiella;s__Klebsiella_michiganensis
PB1_gene_314	213	ORM76076.1	230	211/213(99.06)	2.17e-159	leucyl/phenylalanyl-tRNA--protein transferase [Pantoea	470932	d__Bacteria;k__norank;p__Proteobacteria;c__Gammaproteobacteria;o__Enterobacterales;f__Erwiniaceae;g__Pantoea;s__Pantoea_deleyi
PB1_gene_315	576	WP_111138824.1	576	542/576(94.10)	0	cysteine/glutathione ABC transporter ATP-binding protein/permease CydC [Pantoea sp. ARC607]	2027922	d__Bacteria;k__norank;p__Proteobacteria;c__Gammaproteobacteria;o__Enterobacterales;f__Erwiniaceae;g__Pantoea;s__Pantoea_sp._ARC607
PB1_gene_316	589	ORM75879.1	589	560/589(95.08)	0	cysteine/glutathione ABC transporter permease/ATP-binding protein CydD [Pantoea	470932	d__Bacteria;k__norank;p__Proteobacteria;c__Gammaproteobacteria;o__Enterobacterales;f__Erwiniaceae;g__Pantoea;s__Pantoea_deleyi
PB1_gene_319	877	ORM86849.1	877	870/877(99.20)	0	hypothetical protein HA42_00290 [Pantoea deleyi]	470932	d__Bacteria;k__norank;p__Proteobacteria;c__Gammaproteobacteria;o__Enterobacterales;f__Erwiniaceae;g__Pantoea;s__Pantoea_deleyi
PB1_gene_320	414	WP_111138848.1	414	407/414(98.31)	8.66e-288	paraquat-inducible protein A [Pantoea sp. ARC607]	2027922	d__Bacteria;k__norank;p__Proteobacteria;c__Gammaproteobacteria;o__Enterobacterales;f__Erwiniaceae;g__Pantoea;s__Pantoea_sp._ARC607

PB1_gene_323	118	SKA63958.1	118	118/118(100.00)	8.12e-71	LSU ribosomal protein L20P [Pantoea ananatis]	553	d__Bacteria;k__norank;p__Proteobacteria;c__Gammaproteobacteria;o__Enterobacterales;f__Erwiniaceae;g__Pantoea;s__Pantoea_ananatis
PB1_gene_324	327	ORM79536.1	327	327/327(100.00)	8.87e-237	phenylalanine--tRNA ligase subunit alpha [Pantoea deleyi]	470932	d__Bacteria;k__norank;p__Proteobacteria;c__Gammaproteobacteria;o__Enterobacterales;f__Erwiniaceae;g__Pantoea;s__Pantoea_deleyi
PB1_gene_325	795	ORM79537.1	795	790/795(99.37)	0	phenylalanine--tRNA ligase subunit beta [Pantoea deleyi]	470932	d__Bacteria;k__norank;p__Proteobacteria;c__Gammaproteobacteria;o__Enterobacterales;f__Erwiniaceae;g__Pantoea;s__Pantoea_deleyi
PB1_gene_326	100	SJZ43234.1	100	100/100(100.00)	3.20e-64	integration host factor subunit alpha [Pantoea agglomerans]	470933	d__Bacteria;k__norank;p__Proteobacteria;c__Gammaproteobacteria;o__Enterobacterales;f__Erwiniaceae;g__Pantoea;s__Pantoea_eucalypti
PB1_gene_327	327	WP_111139112.1	327	313/327(95.72)	6.91e-209	vitamin B12 ABC transporter permease BtuC [Pantoea sp. ARC607]	2027922	d__Bacteria;k__norank;p__Proteobacteria;c__Gammaproteobacteria;o__Enterobacterales;f__Erwiniaceae;g__Pantoea;s__Pantoea_sp._ARC607
PB1_gene_328	109	SLO32440.1	121	48/89(53.93)	4.80e-19	Uncharacterised protein [Klebsiella pneumoniae]	573	d__Bacteria;k__norank;p__Proteobacteria;c__Gammaproteobacteria;o__Enterobacterales;f__Enterobacteriaceae;g__Klebsiella;s__Klebsiella_pneumoniae
PB1_gene_330	49	WP_128083924.1	62	46/49(93.88)	1.11e-21	hypothetical protein [Pantoea deleyi]	470932	d__Bacteria;k__norank;p__Proteobacteria;c__Gammaproteobacteria;o__Enterobacterales;f__Erwiniaceae;g__Pantoea;s__Pantoea_deleyi
PB1_gene_331	219	ORM86551.1	219	216/219(98.63)	7.51e-150	ribose 5-phosphate isomerase A [Pantoea deleyi]	470932	d__Bacteria;k__norank;p__Proteobacteria;c__Gammaproteobacteria;o__Enterobacterales;f__Erwiniaceae;g__Pantoea;s__Pantoea_deleyi
PB1_gene_332	301	ORM86550.1	301	301/301(100.00)	1.05e-215	transcriptional regulator ArgP [Pantoea deleyi]	470932	d__Bacteria;k__norank;p__Proteobacteria;c__Gammaproteobacteria;o__Enterobacterales;f__Erwiniaceae;g__Pantoea;s__Pantoea_deleyi
PB1_gene_333	241	WP_111140640.1	241	238/241(98.76)	5.22e-161	oxidative stress defense protein [Pantoea sp. ARC607]	2027922	d__Bacteria;k__norank;p__Proteobacteria;c__Gammaproteobacteria;o__Enterobacterales;f__Erwiniaceae;g__Pantoea;s__Pantoea_sp._ARC607
PB1_gene_334	207	ORM86548.1	207	202/207(97.58)	1.21e-140	arginine transporter [Pantoea deleyi]	470932	d__Bacteria;k__norank;p__Proteobacteria;c__Gammaproteobacteria;o__Enterobacterales;f__Erwiniaceae;g__Pantoea;s__Pantoea_deleyi
PB1_gene_335	293	ORM86547.1	293	291/293(99.32)	9.12e-181	mechanosensitive ion channel protein MscS [Pantoea deleyi]	470932	d__Bacteria;k__norank;p__Proteobacteria;c__Gammaproteobacteria;o__Enterobacterales;f__Erwiniaceae;g__Pantoea;s__Pantoea_deleyi
PB1_gene_338	162	ORM82569.1	165	162/162(100.00)	2.01e-104	chemotaxis protein CheW [Pantoea deleyi]	470932	d__Bacteria;k__norank;p__Proteobacteria;c__Gammaproteobacteria;o__Enterobacterales;f__Erwiniaceae;g__Pantoea;s__Pantoea_deleyi
PB1_gene_339	557	ORM82570.1	557	530/557(95.15)	3.78e-313	methyl-accepting chemotaxis protein II [Pantoea deleyi]	470932	d__Bacteria;k__norank;p__Proteobacteria;c__Gammaproteobacteria;o__Enterobacterales;f__Erwiniaceae;g__Pantoea;s__Pantoea_deleyi
PB1_gene_340	520	ORM82571.1	520	513/520(98.65)	4.79e-295	methyl-accepting chemotaxis protein II [Pantoea deleyi]	470932	d__Bacteria;k__norank;p__Proteobacteria;c__Gammaproteobacteria;o__Enterobacterales;f__Erwiniaceae;g__Pantoea;s__Pantoea_deleyi
PB1_gene_343	449	WP_031590443.1	449	335/449(74.61)	2.29e-257	glycosyl transferase [Pantoea agglomerans]	549	d__Bacteria;k__norank;p__Proteobacteria;c__Gammaproteobacteria;o__Enterobacterales;f__Erwiniaceae;g__Pantoea;s__Pantoea_agglomerans
PB1_gene_344	327	WP_031590442.1	327	252/327(77.06)	8.14e-178	fimbrial protein [Pantoea agglomerans]	549	d__Bacteria;k__norank;p__Proteobacteria;c__Gammaproteobacteria;o__Enterobacterales;f__Erwiniaceae;g__Pantoea;s__Pantoea_agglomerans
PB1_gene_345	360	WP_031590441.1	360	292/360(81.11)	6.77e-205	membrane protein [Pantoea agglomerans]	549	d__Bacteria;k__norank;p__Proteobacteria;c__Gammaproteobacteria;o__Enterobacterales;f__Erwiniaceae;g__Pantoea;s__Pantoea_agglomerans
PB1_gene_346	336	WP_031590440.1	337	298/334(89.22)	3.77e-220	type VI secretion system baseplate subunit TssG [Pantoea	549	d__Bacteria;k__norank;p__Proteobacteria;c__Gammaproteobacteria;o__Enterobacterales;f__Erwiniaceae;g__Pantoea;s__Pantoea_agglomerans
PB1_gene_348	241	WP_111140889.1	240	228/241(94.61)	5.25e-131	DUF2076 domain-containing protein [Pantoea sp. ARC607]	2027922	d__Bacteria;k__norank;p__Proteobacteria;c__Gammaproteobacteria;o__Enterobacterales;f__Erwiniaceae;g__Pantoea;s__Pantoea_sp._ARC607
PB1_gene_349	151	ORM75331.1	151	149/151(98.68)	2.18e-102	hypothetical protein HA42_21005 [Pantoea deleyi]	470932	d__Bacteria;k__norank;p__Proteobacteria;c__Gammaproteobacteria;o__Enterobacterales;f__Erwiniaceae;g__Pantoea;s__Pantoea_deleyi
PB1_gene_350	272	ORM75332.1	270	267/270(98.89)	4.26e-175	pyrroline-5-carboxylate reductase [Pantoea deleyi]	470932	d__Bacteria;k__norank;p__Proteobacteria;c__Gammaproteobacteria;o__Enterobacterales;f__Erwiniaceae;g__Pantoea;s__Pantoea_deleyi
PB1_gene_351	508	WP_111140892.1	508	486/508(95.67)	0	pyridine nucleotide-disulfide oxidoreductase [Pantoea sp. ARC607]	2027922	d__Bacteria;k__norank;p__Proteobacteria;c__Gammaproteobacteria;o__Enterobacterales;f__Erwiniaceae;g__Pantoea;s__Pantoea_sp._ARC607
PB1_gene_353	100	WP_111140893.1	100	65/65(100.00)	6.04e-35	phosphate starvation-inducible protein [Pantoea sp. ARC607]	2027922	d__Bacteria;k__norank;p__Proteobacteria;c__Gammaproteobacteria;o__Enterobacterales;f__Erwiniaceae;g__Pantoea;s__Pantoea_sp._ARC607

PB1_gene_354	82	ORM74636.1	82	76/82(92.68)	2.86e-43	hypothetical protein HA42_21430 [Pantoea deleyi]	470932	d__Bacteria;k__norank;p__Proteobacteria;c__Gammaproteobacteria;o__Enterobacterales;f__Erwiniaceae;g__Pantoea;s__Pantoea_deleyi
PB1_gene_357	433	WP_111142048.1	433	422/433(97.46)	1.76e-308	isocitrate lyase [Pantoea sp. ARC607]	2027922	d__Bacteria;k__norank;p__Proteobacteria;c__Gammaproteobacteria;o__Enterobacterales;f__Erwiniaceae;g__Pantoea;s__Pantoea_sp._ARC607
PB1_gene_358	532	WP_111142047.1	532	508/532(95.49)	0	malate synthase A [Pantoea sp. ARC607]	2027922	d__Bacteria;k__norank;p__Proteobacteria;c__Gammaproteobacteria;o__Enterobacterales;f__Erwiniaceae;g__Pantoea;s__Pantoea_sp._ARC607
PB1_gene_359	309	OQV39999.1	309	303/309(98.06)	9.14e-230	homoserine O-succinyltransferase [Pantoea vagans]	470934	d__Bacteria;k__norank;p__Proteobacteria;c__Gammaproteobacteria;o__Enterobacterales;f__Erwiniaceae;g__Pantoea;s__Pantoea_vagans
PB1_gene_361	153	AVV38770.1	154	151/153(98.69)	7.53e-102	SoxR-reducing system protein RseC [Pantoea vagans]	470934	d__Bacteria;k__norank;p__Proteobacteria;c__Gammaproteobacteria;o__Enterobacterales;f__Erwiniaceae;g__Pantoea;s__Pantoea_vagans
PB1_gene_362	318	WP_111139115.1	318	313/318(98.43)	4.66e-223	sigma-E factor regulatory protein RseB [Pantoea sp. ARC607]	2027922	d__Bacteria;k__norank;p__Proteobacteria;c__Gammaproteobacteria;o__Enterobacterales;f__Erwiniaceae;g__Pantoea;s__Pantoea_sp._ARC607
PB1_gene_363	217	ORM84909.1	217	215/217(99.08)	3.73e-149	anti-sigma factor [Pantoea deleyi]	470932	d__Bacteria;k__norank;p__Proteobacteria;c__Gammaproteobacteria;o__Enterobacterales;f__Erwiniaceae;g__Pantoea;s__Pantoea_deleyi
PB1_gene_364	191	ORM84930.1	191	191/191(100.00)	3.95e-129	RNA polymerase sigma factor RpoE [Pantoea deleyi]	470932	d__Bacteria;k__norank;p__Proteobacteria;c__Gammaproteobacteria;o__Enterobacterales;f__Erwiniaceae;g__Pantoea;s__Pantoea_deleyi
PB1_gene_365	540	ORM84908.1	540	534/540(98.89)	0	L-aspartate oxidase [Pantoea deleyi]	470932	d__Bacteria;k__norank;p__Proteobacteria;c__Gammaproteobacteria;o__Enterobacterales;f__Erwiniaceae;g__Pantoea;s__Pantoea_deleyi
PB1_gene_367	189	KVD77998.1	193	98/194(50.52)	1.41e-44	hypothetical protein WS62_29675 [Burkholderia sp.	1637860	d__Bacteria;k__norank;p__Proteobacteria;c__Betaproteobacteria;o__Burkholderiales;f__Burkholderiaceae;g__Burkholderia;s__Burkholderia_sp._ABCPW_14
PB1_gene_368	155	WP_112291289.1	238	48/151(31.79)	1.91e-13	hypothetical protein [Rahnella sp. AN3-3W3]	1610578	d__Bacteria;k__norank;p__Proteobacteria;c__Gammaproteobacteria;o__Enterobacterales;f__Yersiniaceae;g__Rahnella;s__Rahnella_sp._AN3-3W3
PB1_gene_369	175	WP_111139616.1	175	172/175(98.29)	2.26e-127	beta-carotene hydroxylase [Pantoea sp. ARC607]	2027922	d__Bacteria;k__norank;p__Proteobacteria;c__Gammaproteobacteria;o__Enterobacterales;f__Erwiniaceae;g__Pantoea;s__Pantoea_sp._ARC607
PB1_gene_370	296	WP_111139617.1	309	289/296(97.64)	1.89e-205	phytoene/squalene synthase family protein [Pantoea sp.	2027922	d__Bacteria;k__norank;p__Proteobacteria;c__Gammaproteobacteria;o__Enterobacterales;f__Erwiniaceae;g__Pantoea;s__Pantoea_sp._ARC607
PB1_gene_372	560	ORM84009.1	639	558/560(99.64)	0	murein transglycosylase [Pantoea deleyi]	470932	d__Bacteria;k__norank;p__Proteobacteria;c__Gammaproteobacteria;o__Enterobacterales;f__Erwiniaceae;g__Pantoea;s__Pantoea_deleyi
PB1_gene_373	106	WP_111141374.1	106	105/106(99.06)	1.32e-68	trp operon repressor [Pantoea sp. ARC607]	2027922	d__Bacteria;k__norank;p__Proteobacteria;c__Gammaproteobacteria;o__Enterobacterales;f__Erwiniaceae;g__Pantoea;s__Pantoea_sp._ARC607
PB1_gene_374	172	ORM84007.1	172	171/172(99.42)	1.79e-118	inosine/xanthosine triphosphatase [Pantoea deleyi]	470932	d__Bacteria;k__norank;p__Proteobacteria;c__Gammaproteobacteria;o__Enterobacterales;f__Erwiniaceae;g__Pantoea;s__Pantoea_deleyi
PB1_gene_375	215	ORM84006.1	215	213/215(99.07)	1.30e-148	phosphoglycerate mutase [Pantoea deleyi]	470932	d__Bacteria;k__norank;p__Proteobacteria;c__Gammaproteobacteria;o__Enterobacterales;f__Erwiniaceae;g__Pantoea;s__Pantoea_deleyi
PB1_gene_376	293	ORM84005.1	293	289/293(98.63)	1.09e-213	MDR efflux pump AcrAB transcriptional activator RobA [Pantoea deleyi]	470932	d__Bacteria;k__norank;p__Proteobacteria;c__Gammaproteobacteria;o__Enterobacterales;f__Erwiniaceae;g__Pantoea;s__Pantoea_deleyi
PB1_gene_377	157	WP_111141370.1	157	155/157(98.73)	2.15e-105	protein CreA [Pantoea sp. ARC607]	2027922	d__Bacteria;k__norank;p__Proteobacteria;c__Gammaproteobacteria;o__Enterobacterales;f__Erwiniaceae;g__Pantoea;s__Pantoea_sp._ARC607
PB1_gene_380	207	WP_111141119.1	207	207/207(100.00)	2.11e-159	glutathione S-transferase [Pantoea sp. ARC607]	2027922	d__Bacteria;k__norank;p__Proteobacteria;c__Gammaproteobacteria;o__Enterobacterales;f__Erwiniaceae;g__Pantoea;s__Pantoea_sp._ARC607
PB1_gene_381	495	ORM82133.1	495	483/495(97.58)	0	GntR family transcriptional regulator [Pantoea deleyi]	470932	d__Bacteria;k__norank;p__Proteobacteria;c__Gammaproteobacteria;o__Enterobacterales;f__Erwiniaceae;g__Pantoea;s__Pantoea_deleyi
PB1_gene_382	196	ORM82134.1	195	194/195(99.49)	1.01e-131	hypothetical protein HA42_09020 [Pantoea deleyi]	470932	d__Bacteria;k__norank;p__Proteobacteria;c__Gammaproteobacteria;o__Enterobacterales;f__Erwiniaceae;g__Pantoea;s__Pantoea_deleyi
PB1_gene_383	537	WP_111141126.1	537	530/537(98.70)	0	malate dehydrogenase (quinone) [Pantoea sp. ARC607]	2027922	d__Bacteria;k__norank;p__Proteobacteria;c__Gammaproteobacteria;o__Enterobacterales;f__Erwiniaceae;g__Pantoea;s__Pantoea_sp._ARC607
PB1_gene_385	418	ORM76465.1	418	417/418(99.76)	1.54e-283	polysaccharide biosynthesis protein [Pantoea deleyi]	470932	d__Bacteria;k__norank;p__Proteobacteria;c__Gammaproteobacteria;o__Enterobacterales;f__Erwiniaceae;g__Pantoea;s__Pantoea_deleyi
PB1_gene_386	131	ORM76464.1	131	131/131(100.00)	1.19e-85	transposase [Pantoea deleyi]	470932	d__Bacteria;k__norank;p__Proteobacteria;c__Gammaproteobacteria;o__Enterobacterales;f__Erwiniaceae;g__Pantoea;s__Pantoea_deleyi

PB1_gene_387	319	ORM76463.1	319	310/319(97.18)	9.88e-230	hypothetical protein HA42_19060 [Pantoea deleyi]	470932	d__Bacteria;k__norank;p__Proteobacteria;c__Gammaproteobacteria;o__Enterobacterales;f__Erwiniaceae;g__Pantoea;s__Pantoea_deleyi
PB1_gene_388	357	WP_111140210.1	275	263/273(96.34)	4.68e-193	AraC family transcriptional regulator [Pantoea sp. ARC607]	2027922	d__Bacteria;k__norank;p__Proteobacteria;c__Gammaproteobacteria;o__Enterobacterales;f__Erwiniaceae;g__Pantoea;s__Pantoea_sp._ARC607
PB1_gene_392	188	SUX29127.1	2219	58/170(34.12)	4.46e-15	Putative NAD(+)--arginine ADP-ribosyltransferase Vis precursor [Cedecea davisae]	158484	d__Bacteria;k__norank;p__Proteobacteria;c__Gammaproteobacteria;o__Enterobacterales;f__Enterobacteriaceae;g__Cedecea;s__Cedecea_davisae
PB1_gene_396	123	ORM83608.1	136	57/73(78.08)	6.59e-26	transcriptional regulator [Pantoea deleyi]	470932	d__Bacteria;k__norank;p__Proteobacteria;c__Gammaproteobacteria;o__Enterobacterales;f__Erwiniaceae;g__Pantoea;s__Pantoea_deleyi
PB1_gene_399	202	WP_111142078.1	202	200/202(99.01)	4.10e-150	bifunctional nicotinamidase/pyrazinamidase [Pantoea sp. ARC607]	2027922	d__Bacteria;k__norank;p__Proteobacteria;c__Gammaproteobacteria;o__Enterobacterales;f__Erwiniaceae;g__Pantoea;s__Pantoea_sp._ARC607
PB1_gene_400	337	WP_111142077.1	337	334/337(99.11)	8.57e-240	asparaginase [Pantoea sp. ARC607]	2027922	d__Bacteria;k__norank;p__Proteobacteria;c__Gammaproteobacteria;o__Enterobacterales;f__Erwiniaceae;g__Pantoea;s__Pantoea_sp._ARC607
PB1_gene_401	620	ORM81301.1	620	616/620(99.35)	0	signal peptide peptidase SppA [Pantoea deleyi]	470932	d__Bacteria;k__norank;p__Proteobacteria;c__Gammaproteobacteria;o__Enterobacterales;f__Erwiniaceae;g__Pantoea;s__Pantoea_deleyi
PB1_gene_402	183	ORM81300.1	183	182/183(99.45)	6.16e-129	NAD(P)H nitroreductase [Pantoea deleyi]	470932	d__Bacteria;k__norank;p__Proteobacteria;c__Gammaproteobacteria;o__Enterobacterales;f__Erwiniaceae;g__Pantoea;s__Pantoea_deleyi
PB1_gene_404	358	WP_029882953.1	401	320/401(79.80)	1.16e-226	ISL3 family transposase [Enterobacter sp. T1-1]	1449966	d__Bacteria;k__norank;p__Proteobacteria;c__Gammaproteobacteria;o__Enterobacterales;f__Enterobacteriaceae;g__Enterobacter;s__Enterobacter_sp._T1-1
PB1_gene_405	415	ORM82843.1	415	414/415(99.76)	4.05e-298	NADH dehydrogenase [Pantoea deleyi]	470932	d__Bacteria;k__norank;p__Proteobacteria;c__Gammaproteobacteria;o__Enterobacterales;f__Erwiniaceae;g__Pantoea;s__Pantoea_deleyi
PB1_gene_406	180	ORM82842.1	180	173/180(96.11)	4.89e-129	hypothetical protein HA42_07545 [Pantoea deleyi]	470932	d__Bacteria;k__norank;p__Proteobacteria;c__Gammaproteobacteria;o__Enterobacterales;f__Erwiniaceae;g__Pantoea;s__Pantoea_deleyi
PB1_gene_407	346	WP_111138660.1	346	340/346(98.27)	7.98e-245	beta-N-acetylhexosaminidase [Pantoea sp. ARC607]	2027922	d__Bacteria;k__norank;p__Proteobacteria;c__Gammaproteobacteria;o__Enterobacterales;f__Erwiniaceae;g__Pantoea;s__Pantoea_sp._ARC607
PB1_gene_410	784	WP_090392817.1	784	771/784(98.34)	0	type VI secretion system tip protein VgrG [Enterobacter sp.	1566251	d__Bacteria;k__norank;p__Proteobacteria;c__Gammaproteobacteria;o__Enterobacterales;f__Enterobacteriaceae;g__Enterobacter;s__Enterobacter_sp._NFIX58
PB1_gene_412	241	ERM10996.1	241	241/241(100.00)	5.97e-176	arginine ABC transporter ATP-binding protein [Pantoea agglomerans Tx10]	1332072	d__Bacteria;k__norank;p__Proteobacteria;c__Gammaproteobacteria;o__Enterobacterales;f__Erwiniaceae;g__Pantoea;s__Pantoea_agglomerans
PB1_gene_413	366	WP_111139685.1	366	362/366(98.91)	8.83e-259	amino acid ABC transporter permease [Pantoea sp. ARC607]	2027922	d__Bacteria;k__norank;p__Proteobacteria;c__Gammaproteobacteria;o__Enterobacterales;f__Erwiniaceae;g__Pantoea;s__Pantoea_sp._ARC607
PB1_gene_414	392	ORM83391.1	392	390/392(99.49)	1.40e-275	amino acid ABC transporter permease [Pantoea deleyi]	470932	d__Bacteria;k__norank;p__Proteobacteria;c__Gammaproteobacteria;o__Enterobacterales;f__Erwiniaceae;g__Pantoea;s__Pantoea_deleyi
PB1_gene_415	341	WP_111139683.1	341	339/341(99.41)	1.09e-244	amino acid ABC transporter substrate-binding protein [Pantoea sp. ARC607]	2027922	d__Bacteria;k__norank;p__Proteobacteria;c__Gammaproteobacteria;o__Enterobacterales;f__Erwiniaceae;g__Pantoea;s__Pantoea_sp._ARC607
PB1_gene_420	194	WP_111140166.1	194	182/194(93.81)	5.56e-126	cytochrome b/b6 domain-containing protein [Pantoea sp.	2027922	d__Bacteria;k__norank;p__Proteobacteria;c__Gammaproteobacteria;o__Enterobacterales;f__Erwiniaceae;g__Pantoea;s__Pantoea_sp._ARC607
PB1_gene_421	216	ORM83588.1	216	209/216(96.76)	1.59e-154	hypothetical protein HA42_05940 [Pantoea deleyi]	470932	d__Bacteria;k__norank;p__Proteobacteria;c__Gammaproteobacteria;o__Enterobacterales;f__Erwiniaceae;g__Pantoea;s__Pantoea_deleyi
PB1_gene_422	478	WP_111140168.1	478	455/478(95.19)	0	AMP-dependent synthetase [Pantoea sp. ARC607]	2027922	d__Bacteria;k__norank;p__Proteobacteria;c__Gammaproteobacteria;o__Enterobacterales;f__Erwiniaceae;g__Pantoea;s__Pantoea_sp._ARC607
PB1_gene_423	221	WP_111140169.1	222	219/221(99.10)	5.94e-155	biliverdin-producing heme oxygenase [Pantoea sp. ARC607]	2027922	d__Bacteria;k__norank;p__Proteobacteria;c__Gammaproteobacteria;o__Enterobacterales;f__Erwiniaceae;g__Pantoea;s__Pantoea_sp._ARC607
PB1_gene_426	324	WP_111141064.1	324	320/324(98.77)	2.07e-229	glyoxylate/hydroxypyruvate reductase GhrB [Pantoea sp. ARC607]	2027922	d__Bacteria;k__norank;p__Proteobacteria;c__Gammaproteobacteria;o__Enterobacterales;f__Erwiniaceae;g__Pantoea;s__Pantoea_sp._ARC607
PB1_gene_427	416	WP_111141062.1	416	414/416(99.52)	9.75e-311	valine--pyruvate transaminase [Pantoea sp. ARC607]	2027922	d__Bacteria;k__norank;p__Proteobacteria;c__Gammaproteobacteria;o__Enterobacterales;f__Erwiniaceae;g__Pantoea;s__Pantoea_sp._ARC607

PB1_gene_428	483	ORM85486.1	483	478/483(98.96)	0	GntR family transcriptional regulator [Pantoea deleyi]	470932	d__Bacteria;k__norank;p__Proteobacteria;c__Gammaproteobacteria;o__Enterobacterales;f__Erwiniaceae;g__Pantoea;s__Pantoea_deleyi
PB1_gene_431	1136	ORM82821.1	1139	1015/1139(89.11)	0	ribonuclease E [Pantoea deleyi]	470932	d__Bacteria;k__norank;p__Proteobacteria;c__Gammaproteobacteria;o__Enterobacterales;f__Erwiniaceae;g__Pantoea;s__Pantoea_deleyi
PB1_gene_435	419	WP_111138277.1	419	362/419(86.40)	1.23e-239	HlyD family efflux transporter periplasmic adaptor subunit [Pantoea sp. ARC607]	2027922	d__Bacteria;k__norank;p__Proteobacteria;c__Gammaproteobacteria;o__Enterobacterales;f__Erwiniaceae;g__Pantoea;s__Pantoea_sp._ARC607
PB1_gene_436	720	ORM81587.1	720	695/720(96.53)	0	ABC transporter [Pantoea deleyi]	470932	d__Bacteria;k__norank;p__Proteobacteria;c__Gammaproteobacteria;o__Enterobacterales;f__Erwiniaceae;g__Pantoea;s__Pantoea_deleyi
PB1_gene_437	145	ORM81586.1	145	124/145(85.52)	8.63e-88	hypothetical protein HA42_10210 [Pantoea deleyi]	470932	d__Bacteria;k__norank;p__Proteobacteria;c__Gammaproteobacteria;o__Enterobacterales;f__Erwiniaceae;g__Pantoea;s__Pantoea_deleyi
PB1_gene_438	306	ORM72653.1	306	293/306(95.75)	2.36e-210	AraC family transcriptional regulator [Pantoea deleyi]	470932	d__Bacteria;k__norank;p__Proteobacteria;c__Gammaproteobacteria;o__Enterobacterales;f__Erwiniaceae;g__Pantoea;s__Pantoea_deleyi
PB1_gene_439	121	ORM72654.1	277	112/276(40.58)	3.82e-43	short-chain dehydrogenase/reductase [Pantoea deleyi]	470932	d__Bacteria;k__norank;p__Proteobacteria;c__Gammaproteobacteria;o__Enterobacterales;f__Erwiniaceae;g__Pantoea;s__Pantoea_deleyi
PB1_gene_440	316	WP_111141996.1	316	303/316(95.89)	1.08e-211	hydroxyacid dehydrogenase [Pantoea sp. ARC607]	2027922	d__Bacteria;k__norank;p__Proteobacteria;c__Gammaproteobacteria;o__Enterobacterales;f__Erwiniaceae;g__Pantoea;s__Pantoea_sp._ARC607
PB1_gene_441	230	WP_111141997.1	230	228/230(99.13)	1.63e-154	RraA family protein [Pantoea sp. ARC607]	53335	d__Bacteria;k__norank;p__Proteobacteria;c__Gammaproteobacteria;o__Enterobacterales;f__Erwiniaceae;g__Pantoea;s__norank
PB1_gene_446	559	ADO11617.1	559	547/559(97.85)	0	Glucose-6-phosphate isomerase [Pantoea vagans C9-1]	712898	d__Bacteria;k__norank;p__Proteobacteria;c__Gammaproteobacteria;o__Enterobacterales;f__Erwiniaceae;g__Pantoea;s__Pantoea_vagans
PB1_gene_447	121	ORM82911.1	85	62/62(100.00)	8.27e-26	hypothetical protein HA42_07220 [Pantoea deleyi]	470932	d__Bacteria;k__norank;p__Proteobacteria;c__Gammaproteobacteria;o__Enterobacterales;f__Erwiniaceae;g__Pantoea;s__Pantoea_deleyi
PB1_gene_448	222	ORM82910.1	215	210/215(97.67)	2.99e-149	hypothetical protein HA42_07215 [Pantoea deleyi]	470932	d__Bacteria;k__norank;p__Proteobacteria;c__Gammaproteobacteria;o__Enterobacterales;f__Erwiniaceae;g__Pantoea;s__Pantoea_deleyi
PB1_gene_452	248	ORM84530.1	248	246/248(99.19)	1.07e-177	ferredoxin--NADP(+) reductase [Pantoea deleyi]	470932	d__Bacteria;k__norank;p__Proteobacteria;c__Gammaproteobacteria;o__Enterobacterales;f__Erwiniaceae;g__Pantoea;s__Pantoea_deleyi
PB1_gene_453	144	WP_111140702.1	144	139/144(96.53)	3.16e-103	DUF805 domain-containing protein [Pantoea sp. ARC607]	2027922	d__Bacteria;k__norank;p__Proteobacteria;c__Gammaproteobacteria;o__Enterobacterales;f__Erwiniaceae;g__Pantoea;s__Pantoea_sp._ARC607
PB1_gene_454	229	ORM84549.1	196	194/196(98.98)	3.52e-134	hypothetical protein HA42_04280 [Pantoea deleyi]	470932	d__Bacteria;k__norank;p__Proteobacteria;c__Gammaproteobacteria;o__Enterobacterales;f__Erwiniaceae;g__Pantoea;s__Pantoea_deleyi
PB1_gene_455	249	WP_111140703.1	255	247/249(99.20)	1.66e-167	triose-phosphate isomerase [Pantoea sp. ARC607]	2027922	d__Bacteria;k__norank;p__Proteobacteria;c__Gammaproteobacteria;o__Enterobacterales;f__Erwiniaceae;g__Pantoea;s__Pantoea_sp._ARC607
PB1_gene_456	257	WP_111140704.1	257	237/253(93.68)	1.73e-167	CDP-diacylglycerol diphosphatase [Pantoea sp.	2027922	d__Bacteria;k__norank;p__Proteobacteria;c__Gammaproteobacteria;o__Enterobacterales;f__Erwiniaceae;g__Pantoea;s__Pantoea_sp._ARC607
PB1_gene_457	329	WP_111140705.1	329	326/329(99.09)	5.36e-239	sulfate ABC transporter substrate-binding protein [Pantoea sp. ARC607]	2027922	d__Bacteria;k__norank;p__Proteobacteria;c__Gammaproteobacteria;o__Enterobacterales;f__Erwiniaceae;g__Pantoea;s__Pantoea_sp._ARC607
PB1_gene_459	338	WP_105591716.1	338	320/338(94.67)	6.86e-226	ABC transporter permease [Pantoea coffeiphila]	1465635	d__Bacteria;k__norank;p__Proteobacteria;c__Gammaproteobacteria;o__Enterobacterales;f__Erwiniaceae;g__Pantoea;s__Pantoea_coffeiphila
PB1_gene_460	296	WP_105591715.1	297	265/295(89.83)	9.58e-187	D,D-dipeptide ABC transporter permease [Pantoea coffeiphila]	1465635	d__Bacteria;k__norank;p__Proteobacteria;c__Gammaproteobacteria;o__Enterobacterales;f__Erwiniaceae;g__Pantoea;s__Pantoea_coffeiphila
PB1_gene_461	328	WP_038017269.1	328	257/328(78.35)	2.90e-183	ABC transporter ATP-binding protein [Tatumella morbirosei]	642227	d__Bacteria;k__norank;p__Proteobacteria;c__Gammaproteobacteria;o__Enterobacterales;f__Erwiniaceae;g__Tatumella;s__Tatumella_morbirosei
PB1_gene_462	316	WP_123333693.1	317	254/310(81.94)	2.02e-175	ABC transporter ATP-binding protein [Erwinia sp. JUb26]	2485126	d__Bacteria;k__norank;p__Proteobacteria;c__Gammaproteobacteria;o__Enterobacterales;f__Erwiniaceae;g__Erwinia;s__Erwinia_sp._JUb26
PB1_gene_465	243	WP_105099003.1	243	230/243(94.65)	1.90e-164	peptidase [Pantoea ananatis]	53335	d__Bacteria;k__norank;p__Proteobacteria;c__Gammaproteobacteria;o__Enterobacterales;f__Erwiniaceae;g__Pantoea;s__norank
PB1_gene_466	265	WP_129954669.1	264	251/264(95.08)	3.88e-186	MULTISPECIES: transglutaminase family protein	2635087	d__Bacteria;k__norank;p__Proteobacteria;c__Gammaproteobacteria;o__Enterobacterales;f__Yersiniaceae;g__Rahnella;s__norank

PB1_gene_467	313	WP_104924302.1	313	294/313(93.93)	1.65e-209	alpha-E domain-containing protein [Rahnella sp. ERMR1:05]	1805933	d__Bacteria;k__norank;p__Proteobacteria;c__Gammaproteobacteria;o__Enterobacterales;f__Yersiniaceae;g__Rahnella;s__Rahnella_sp._ERMR1:05
PB1_gene_470	145	ORM79515.1	145	142/145(97.93)	1.23e-96	peptidoglycan-binding protein LysM [Pantoea deleyi]	470932	d__Bacteria;k__norank;p__Proteobacteria;c__Gammaproteobacteria;o__Enterobacterales;f__Erwiniaceae;g__Pantoea;s__Pantoea_deleyi
PB1_gene_471	192	ORM79514.1	192	186/192(96.88)	1.13e-124	hypothetical protein HA42_15430 [Pantoea deleyi]	470932	d__Bacteria;k__norank;p__Proteobacteria;c__Gammaproteobacteria;o__Enterobacterales;f__Erwiniaceae;g__Pantoea;s__Pantoea_deleyi
PB1_gene_472	322	ORM79513.1	322	320/322(99.38)	4.91e-211	protein-export membrane protein SecF [Pantoea deleyi]	470932	d__Bacteria;k__norank;p__Proteobacteria;c__Gammaproteobacteria;o__Enterobacterales;f__Erwiniaceae;g__Pantoea;s__Pantoea_deleyi
PB1_gene_473	604	WP_111140009.1	615	601/604(99.50)	0	protein translocase subunit SecD [Pantoea sp. ARC607]	2027922	d__Bacteria;k__norank;p__Proteobacteria;c__Gammaproteobacteria;o__Enterobacterales;f__Erwiniaceae;g__Pantoea;s__Pantoea_sp._ARC607
PB1_gene_474	110	ORM79511.1	110	110/110(100.00)	4.17e-67	preprotein translocase subunit YajC [Pantoea deleyi]	470932	d__Bacteria;k__norank;p__Proteobacteria;c__Gammaproteobacteria;o__Enterobacterales;f__Erwiniaceae;g__Pantoea;s__Pantoea_deleyi
PB1_gene_477	330	ORM80767.1	330	330/330(100.00)	2.14e-225	RNA polymerase sigma factor RpoS [Pantoea deleyi]	470932	d__Bacteria;k__norank;p__Proteobacteria;c__Gammaproteobacteria;o__Enterobacterales;f__Erwiniaceae;g__Pantoea;s__Pantoea_deleyi
PB1_gene_478	405	WP_111142180.1	366	354/366(96.72)	7.74e-238	murein hydrolase activator NlpD [Pantoea sp. ARC607]	2027922	d__Bacteria;k__norank;p__Proteobacteria;c__Gammaproteobacteria;o__Enterobacterales;f__Erwiniaceae;g__Pantoea;s__Pantoea_sp._ARC607
PB1_gene_479	208	ORM80765.1	208	207/208(99.52)	1.04e-146	protein-L-isoaspartate O-methyltransferase [Pantoea	470932	d__Bacteria;k__norank;p__Proteobacteria;c__Gammaproteobacteria;o__Enterobacterales;f__Erwiniaceae;g__Pantoea;s__Pantoea_deleyi
PB1_gene_480	253	WP_111141649.1	253	250/253(98.81)	1.71e-181	5'/3'-nucleotidase SurE [Pantoea sp. ARC607]	2027922	d__Bacteria;k__norank;p__Proteobacteria;c__Gammaproteobacteria;o__Enterobacterales;f__Erwiniaceae;g__Pantoea;s__Pantoea_sp._ARC607
PB1_gene_482	504	ORM85223.1	504	490/504(97.22)	0	porin [Pantoea deleyi]	470932	d__Bacteria;k__norank;p__Proteobacteria;c__Gammaproteobacteria;o__Enterobacterales;f__Erwiniaceae;g__Pantoea;s__Pantoea_deleyi
PB1_gene_483	456	WP_111138346.1	456	452/456(99.12)	2.76e-315	PTS sucrose transporter subunit IIBC [Pantoea sp. ARC607]	2027922	d__Bacteria;k__norank;p__Proteobacteria;c__Gammaproteobacteria;o__Enterobacterales;f__Erwiniaceae;g__Pantoea;s__Pantoea_sp._ARC607
PB1_gene_484	469	ORM85225.1	469	447/469(95.31)	0	glycosyl hydrolase family 32 [Pantoea deleyi]	470932	d__Bacteria;k__norank;p__Proteobacteria;c__Gammaproteobacteria;o__Enterobacterales;f__Erwiniaceae;g__Pantoea;s__Pantoea_deleyi
PB1_gene_489	425	ORM81309.1	425	425/425(100.00)	1.83e-290	hypothetical protein HA42_11210 [Pantoea deleyi]	470932	d__Bacteria;k__norank;p__Proteobacteria;c__Gammaproteobacteria;o__Enterobacterales;f__Erwiniaceae;g__Pantoea;s__Pantoea_deleyi
PB1_gene_490	644	WP_111141829.1	644	644/644(100.00)	0	PrkA family serine protein kinase [Pantoea sp. ARC607]	53335	d__Bacteria;k__norank;p__Proteobacteria;c__Gammaproteobacteria;o__Enterobacterales;f__Erwiniaceae;g__Pantoea;s__norank
PB1_gene_494	748	ORM86581.1	748	744/748(99.47)	0	phosphoenolpyruvate--protein phosphotransferase PtsP [Pantoea deleyi]	470932	d__Bacteria;k__norank;p__Proteobacteria;c__Gammaproteobacteria;o__Enterobacterales;f__Erwiniaceae;g__Pantoea;s__Pantoea_deleyi
PB1_gene_495	175	ORM86580.1	175	174/175(99.43)	8.52e-132	RNA pyrophosphohydrolase [Pantoea deleyi]	470932	d__Bacteria;k__norank;p__Proteobacteria;c__Gammaproteobacteria;o__Enterobacterales;f__Erwiniaceae;g__Pantoea;s__Pantoea_deleyi
PB1_gene_496	225	ORM86579.1	225	222/225(98.67)	8.82e-161	DNA mismatch repair endonuclease MutH [Pantoea	470932	d__Bacteria;k__norank;p__Proteobacteria;c__Gammaproteobacteria;o__Enterobacterales;f__Erwiniaceae;g__Pantoea;s__Pantoea_deleyi
PB1_gene_499	377	WP_111142156.1	377	369/377(97.88)	5.20e-268	glycosyltransferase family 1 protein [Pantoea sp. ARC607]	2027922	d__Bacteria;k__norank;p__Proteobacteria;c__Gammaproteobacteria;o__Enterobacterales;f__Erwiniaceae;g__Pantoea;s__Pantoea_sp._ARC607
PB1_gene_500	424	ORM84503.1	424	421/424(99.29)	2.90e-307	3-deoxy-D-manno-octulosonic acid transferase [Pantoea deleyi]	470932	d__Bacteria;k__norank;p__Proteobacteria;c__Gammaproteobacteria;o__Enterobacterales;f__Erwiniaceae;g__Pantoea;s__Pantoea_deleyi
PB1_gene_501	258	WP_111142158.1	258	251/258(97.29)	1.07e-182	glycosyltransferase family 2 protein [Pantoea sp. ARC607]	2027922	d__Bacteria;k__norank;p__Proteobacteria;c__Gammaproteobacteria;o__Enterobacterales;f__Erwiniaceae;g__Pantoea;s__Pantoea_sp._ARC607
PB1_gene_502	159	ORM84501.1	159	156/159(98.11)	2.81e-102	pantetheine-phosphate adenylyltransferase [Pantoea	470932	d__Bacteria;k__norank;p__Proteobacteria;c__Gammaproteobacteria;o__Enterobacterales;f__Erwiniaceae;g__Pantoea;s__Pantoea_deleyi
PB1_gene_505	281	ARF48494.1	276	265/276(96.01)	3.96e-202	gluconolactonase [Pantoea stewartii subsp. stewartii DC283]	660596	d__Bacteria;k__norank;p__Proteobacteria;c__Gammaproteobacteria;o__Enterobacterales;f__Erwiniaceae;g__Pantoea;s__Pantoea_stewartii
PB1_gene_506	366	WP_039339010.1	366	349/366(95.36)	3.30e-254	sugar ABC transporter substrate-binding protein [Pantoea	66269	d__Bacteria;k__norank;p__Proteobacteria;c__Gammaproteobacteria;o__Enterobacterales;f__Erwiniaceae;g__Pantoea;s__Pantoea_stewartii
PB1_gene_507	366	ARF48492.1	366	360/366(98.36)	7.62e-244	ABC transporter permease [Pantoea stewartii subsp. stewartii	660596	d__Bacteria;k__norank;p__Proteobacteria;c__Gammaproteobacteria;o__Enterobacterales;f__Erwiniaceae;g__Pantoea;s__Pantoea_stewartii

PB1_gene_508	508	PXV76867.1	508	488/508(96.06)	0	monosaccharide ABC transporter ATP-binding protein (CUT2 family) [Pantoea sp. PNA 03-3]	2135460	d__Bacteria;k__norank;p__Proteobacteria;c__Gammaproteobacteria;o__Enterobacterales;f__Erwiniaceae;g__Pantoea;s__Pantoea_sp._PNA_03-3
PB1_gene_511	506	ORM82120.1	506	492/506(97.23)	0	ATP-dependent protease [Pantoea deleyi]	470932	d__Bacteria;k__norank;p__Proteobacteria;c__Gammaproteobacteria;o__Enterobacterales;f__Erwiniaceae;g__Pantoea;s__Pantoea_deleyi
PB1_gene_512	112	WP_111141094.1	112	112/112(100.00)	8.34e-68	DUF413 domain-containing protein [Pantoea sp. ARC607]	2027922	d__Bacteria;k__norank;p__Proteobacteria;c__Gammaproteobacteria;o__Enterobacterales;f__Erwiniaceae;g__Pantoea;s__Pantoea_sp._ARC607
PB1_gene_513	277	ORM82118.1	277	275/277(99.28)	5.55e-199	transcriptional regulator HdfR [Pantoea deleyi]	470932	d__Bacteria;k__norank;p__Proteobacteria;c__Gammaproteobacteria;o__Enterobacterales;f__Erwiniaceae;g__Pantoea;s__Pantoea_deleyi
PB1_gene_515	842	WP_111139045.1	841	796/842(94.54)	0	malto-oligosyltrehalose synthase [Pantoea sp. ARC607]	2027922	d__Bacteria;k__norank;p__Proteobacteria;c__Gammaproteobacteria;o__Enterobacterales;f__Erwiniaceae;g__Pantoea;s__Pantoea_sp._ARC607
PB1_gene_516	594	WP_111139044.1	594	565/594(95.12)	0	malto-oligosyltrehalose trehalohydrolase [Pantoea sp. ARC607]	2027922	d__Bacteria;k__norank;p__Proteobacteria;c__Gammaproteobacteria;o__Enterobacterales;f__Erwiniaceae;g__Pantoea;s__Pantoea_sp._ARC607
PB1_gene_520	438	WP_095706964.1	438	434/438(99.09)	0	4-aminobutyrate--2-oxoglutarate transaminase [Pantoea vagans]	470934	d__Bacteria;k__norank;p__Proteobacteria;c__Gammaproteobacteria;o__Enterobacterales;f__Erwiniaceae;g__Pantoea;s__Pantoea_vagans
PB1_gene_521	170	OQV41658.1	170	167/170(98.24)	8.22e-114	AsnC family transcriptional regulator [Pantoea vagans]	470934	d__Bacteria;k__norank;p__Proteobacteria;c__Gammaproteobacteria;o__Enterobacterales;f__Erwiniaceae;g__Pantoea;s__Pantoea_vagans
PB1_gene_522	148	WP_090959810.1	148	139/148(93.92)	2.57e-104	N-acetyltransferase [Pantoea sp. OV426]	1881036	d__Bacteria;k__norank;p__Proteobacteria;c__Gammaproteobacteria;o__Enterobacterales;f__Erwiniaceae;g__Pantoea;s__Pantoea_sp._OV426
PB1_gene_526	227	ORM82651.1	227	218/227(96.04)	3.81e-147	GntR family transcriptional regulator [Pantoea deleyi]	470932	d__Bacteria;k__norank;p__Proteobacteria;c__Gammaproteobacteria;o__Enterobacterales;f__Erwiniaceae;g__Pantoea;s__Pantoea_deleyi
PB1_gene_527	351	ORM82652.1	351	350/351(99.72)	3.16e-217	pheromone autoinducer 2 transporter [Pantoea deleyi]	470932	d__Bacteria;k__norank;p__Proteobacteria;c__Gammaproteobacteria;o__Enterobacterales;f__Erwiniaceae;g__Pantoea;s__Pantoea_deleyi
PB1_gene_528	407	RAH28316.1	407	386/403(95.78)	7.63e-267	MFS transporter [Pantoea agglomerans]	549	d__Bacteria;k__norank;p__Proteobacteria;c__Gammaproteobacteria;o__Enterobacterales;f__Erwiniaceae;g__Pantoea;s__Pantoea_agglomerans
PB1_gene_530	556	ORM77373.1	556	553/556(99.46)	0	ECF transporter S component [Pantoea deleyi]	470932	d__Bacteria;k__norank;p__Proteobacteria;c__Gammaproteobacteria;o__Enterobacterales;f__Erwiniaceae;g__Pantoea;s__Pantoea_deleyi
PB1_gene_531	242	ORM77374.1	242	239/242(98.76)	1.40e-170	DNA-binding response regulator [Pantoea deleyi]	470932	d__Bacteria;k__norank;p__Proteobacteria;c__Gammaproteobacteria;o__Enterobacterales;f__Erwiniaceae;g__Pantoea;s__Pantoea_deleyi
PB1_gene_532	321	ORM77375.1	321	321/321(100.00)	1.47e-232	glucokinase [Pantoea deleyi]	470932	d__Bacteria;k__norank;p__Proteobacteria;c__Gammaproteobacteria;o__Enterobacterales;f__Erwiniaceae;g__Pantoea;s__Pantoea_deleyi
PB1_gene_539	473	WP_077188542.1	470	438/469(93.39)	0	phage portal protein [Wolbachia pipientis]	955	d__Bacteria;k__norank;p__Proteobacteria;c__Alphaproteobacteria;o__Rickettsiales;f__Anaplasmataceae;g__Wolbachia;s__Wolbachia_pipientis
PB1_gene_542	232	WP_039339015.1	232	221/232(95.26)	1.51e-160	L-ribulose-5-phosphate 4-epimerase [Pantoea stewartii]	66269	d__Bacteria;k__norank;p__Proteobacteria;c__Gammaproteobacteria;o__Enterobacterales;f__Erwiniaceae;g__Pantoea;s__Pantoea_stewartii
PB1_gene_543	286	ARF48500.1	286	271/286(94.76)	9.17e-198	xylulose 5-phosphate 3-epimerase [Pantoea stewartii subsp. stewartii DC283]	660596	d__Bacteria;k__norank;p__Proteobacteria;c__Gammaproteobacteria;o__Enterobacterales;f__Erwiniaceae;g__Pantoea;s__Pantoea_stewartii
PB1_gene_544	218	WP_110268059.1	218	206/218(94.50)	1.24e-143	3-keto-L-gulonate-6-phosphate decarboxylase UlaD [Pantoea sp. PNA 03-3]	53335	d__Bacteria;k__norank;p__Proteobacteria;c__Gammaproteobacteria;o__Enterobacterales;f__Erwiniaceae;g__Pantoea;s__norank
PB1_gene_545	493	KKW51655.1	493	453/492(92.07)	0	xylulose kinase [Pantoea ananatis]	553	d__Bacteria;k__norank;p__Proteobacteria;c__Gammaproteobacteria;o__Enterobacterales;f__Erwiniaceae;g__Pantoea;s__Pantoea_ananatis
PB1_gene_547	252	BAH22221.1	475	238/250(95.20)	6.52e-171	phage portal protein, lambda family [Wolbachia endosymbiont of Cadra cautella]	190193	d__Bacteria;k__norank;p__Proteobacteria;c__Alphaproteobacteria;o__Rickettsiales;f__Anaplasmataceae;g__Wolbachia;s__Wolbachia_endosymbiont_of_Cadra_cautella
PB1_gene_548	120	WP_019236401.1	345	102/110(92.73)	7.84e-68	phage portal protein [Wolbachia pipientis]	955	d__Bacteria;k__norank;p__Proteobacteria;c__Alphaproteobacteria;o__Rickettsiales;f__Anaplasmataceae;g__Wolbachia;s__Wolbachia_pipientis

PB1_gene_550	123	WP_114517792.1	123	115/121(95.04)	4.97e-72	head decoration protein [Wolbachia endosymbiont of Cylisticus convexus]	118728	d__Bacteria;k__norank;p__Proteobacteria;c__Alphaproteobacteria;o__Rickettsiales;f__Anaplasmataceae;g__Wolbachia;s__Wolbachia_endosymbiont_of_Cylisticus_convexus
PB1_gene_551	334	BAH22224.1	334	325/334(97.31)	6.06e-243	hypothetical protein [Wolbachia endosymbiont of Cadra cautella]	190193	d__Bacteria;k__norank;p__Proteobacteria;c__Alphaproteobacteria;o__Rickettsiales;f__Anaplasmataceae;g__Wolbachia;s__Wolbachia_endosymbiont_of_Cadra_cautella
PB1_gene_557	370	ORM83992.1	392	368/370(99.46)	5.91e-237	Na(+)/H(+) antiporter NhaA [Pantoea deleyi]	470932	d__Bacteria;k__norank;p__Proteobacteria;c__Gammaproteobacteria;o__Enterobacterales;f__Erwiniaceae;g__Pantoea;s__Pantoea_deleyi
PB1_gene_558	381	ORM83993.1	381	381/381(100.00)	1.17e-234	molecular chaperone DnaJ [Pantoea deleyi]	470932	d__Bacteria;k__norank;p__Proteobacteria;c__Gammaproteobacteria;o__Enterobacterales;f__Erwiniaceae;g__Pantoea;s__Pantoea_deleyi
PB1_gene_559	636	PZL84788.1	636	634/636(99.69)	0	molecular chaperone DnaK [Pantoea sp. ARC270]	2027923	d__Bacteria;k__norank;p__Proteobacteria;c__Gammaproteobacteria;o__Enterobacterales;f__Erwiniaceae;g__Pantoea;s__Pantoea_sp._ARC270
PB1_gene_563	107	ORM81916.1	107	102/107(95.33)	1.26e-62	hypothetical protein HA42_09545 [Pantoea deleyi]	470932	d__Bacteria;k__norank;p__Proteobacteria;c__Gammaproteobacteria;o__Enterobacterales;f__Erwiniaceae;g__Pantoea;s__Pantoea_deleyi
PB1_gene_564	344	WP_111140545.1	344	338/344(98.26)	5.16e-240	L-rhamnose/proton symporter RhaT [Pantoea sp. ARC607]	2027922	d__Bacteria;k__norank;p__Proteobacteria;c__Gammaproteobacteria;o__Enterobacterales;f__Erwiniaceae;g__Pantoea;s__Pantoea_sp._ARC607
PB1_gene_565	288	WP_111140544.1	288	272/287(94.77)	1.42e-202	HTH-type transcriptional activator RhaR [Pantoea sp.	2027922	d__Bacteria;k__norank;p__Proteobacteria;c__Gammaproteobacteria;o__Enterobacterales;f__Erwiniaceae;g__Pantoea;s__Pantoea_sp._ARC607
PB1_gene_566	273	WP_111140543.1	273	266/273(97.44)	1.42e-199	HTH-type transcriptional activator RhaS [Pantoea sp.	2027922	d__Bacteria;k__norank;p__Proteobacteria;c__Gammaproteobacteria;o__Enterobacterales;f__Erwiniaceae;g__Pantoea;s__Pantoea_sp._ARC607
PB1_gene_569	307	ORM77220.1	307	305/307(99.35)	1.39e-223	formate dehydrogenase accessory protein FdhE [Pantoea deleyi]	470932	d__Bacteria;k__norank;p__Proteobacteria;c__Gammaproteobacteria;o__Enterobacterales;f__Erwiniaceae;g__Pantoea;s__Pantoea_deleyi
PB1_gene_570	203	WP_111141321.1	203	198/203(97.54)	1.72e-138	glutathione S-transferase [Pantoea sp. ARC607]	2027922	d__Bacteria;k__norank;p__Proteobacteria;c__Gammaproteobacteria;o__Enterobacterales;f__Erwiniaceae;g__Pantoea;s__Pantoea_sp._ARC607
PB1_gene_575	107	WP_083070435.1	107	81/107(75.70)	4.91e-51	hypothetical protein [Pantoea vagans]	470934	d__Bacteria;k__norank;p__Proteobacteria;c__Gammaproteobacteria;o__Enterobacterales;f__Erwiniaceae;g__Pantoea;s__Pantoea_vagans
PB1_gene_576	115	WP_073970898.1	115	112/115(97.39)	2.85e-83	hypothetical protein [Pantoea agglomerans]	549	d__Bacteria;k__norank;p__Proteobacteria;c__Gammaproteobacteria;o__Enterobacterales;f__Erwiniaceae;g__Pantoea;s__Pantoea_agglomerans
PB1_gene_578	444	ORM86343.1	444	444/444(100.00)	0	phosphoglucosamine mutase [Pantoea deleyi]	470932	d__Bacteria;k__norank;p__Proteobacteria;c__Gammaproteobacteria;o__Enterobacterales;f__Erwiniaceae;g__Pantoea;s__Pantoea_deleyi
PB1_gene_579	277	WP_111139585.1	277	275/277(99.28)	2.83e-192	dihydropteroate synthase [Pantoea sp. ARC607]	2027922	d__Bacteria;k__norank;p__Proteobacteria;c__Gammaproteobacteria;o__Enterobacterales;f__Erwiniaceae;g__Pantoea;s__Pantoea_sp._ARC607
PB1_gene_580	641	WP_111139586.1	641	640/641(99.84)	0	ATP-dependent zinc metalloprotease FtsH [Pantoea sp.	53335	d__Bacteria;k__norank;p__Proteobacteria;c__Gammaproteobacteria;o__Enterobacterales;f__Erwiniaceae;g__Pantoea;s__norank
PB1_gene_581	226	KEY41744.1	223	218/223(97.76)	7.55e-155	ribosomal RNA large subunit methyltransferase J RrmJ [Pantoea agglomerans]	549	d__Bacteria;k__norank;p__Proteobacteria;c__Gammaproteobacteria;o__Enterobacterales;f__Erwiniaceae;g__Pantoea;s__Pantoea_agglomerans
PB1_gene_583	346	ORM82049.1	346	345/346(99.71)	1.80e-241	DNA-binding transcriptional regulator GalS [Pantoea deleyi]	470932	d__Bacteria;k__norank;p__Proteobacteria;c__Gammaproteobacteria;o__Enterobacterales;f__Erwiniaceae;g__Pantoea;s__Pantoea_deleyi
PB1_gene_584	386	ORM82048.1	386	374/386(96.89)	3.19e-278	hypothetical protein HA42_09080 [Pantoea deleyi]	470932	d__Bacteria;k__norank;p__Proteobacteria;c__Gammaproteobacteria;o__Enterobacterales;f__Erwiniaceae;g__Pantoea;s__Pantoea_deleyi
PB1_gene_585	221	ORM82047.1	221	221/221(100.00)	8.76e-150	GTP cyclohydrolase I FolE [Pantoea deleyi]	470932	d__Bacteria;k__norank;p__Proteobacteria;c__Gammaproteobacteria;o__Enterobacterales;f__Erwiniaceae;g__Pantoea;s__Pantoea_deleyi
PB1_gene_588	523	SUB08731.1	523	512/523(97.90)	0	Aspartate ammonia-lyase [Pantoea agglomerans]	549	d__Bacteria;k__norank;p__Proteobacteria;c__Gammaproteobacteria;o__Enterobacterales;f__Erwiniaceae;g__Pantoea;s__Pantoea_agglomerans
PB1_gene_589	153	WP_111142142.1	153	149/152(98.03)	3.73e-95	FxsA family protein [Pantoea sp. ARC607]	2027922	d__Bacteria;k__norank;p__Proteobacteria;c__Gammaproteobacteria;o__Enterobacterales;f__Erwiniaceae;g__Pantoea;s__Pantoea_sp._ARC607
PB1_gene_590	97	SJZ89177.1	97	97/97(100.00)	1.33e-57	chaperonin GroES [Pantoea agglomerans]	470933	d__Bacteria;k__norank;p__Proteobacteria;c__Gammaproteobacteria;o__Enterobacterales;f__Erwiniaceae;g__Pantoea;s__Pantoea_eucalypti
PB1_gene_594	155	WP_111139565.1	155	152/155(98.06)	1.30e-114	anaerobic ribonucleoside-triphosphate reductase-activating protein [Pantoea sp. ARC607]	2027922	d__Bacteria;k__norank;p__Proteobacteria;c__Gammaproteobacteria;o__Enterobacterales;f__Erwiniaceae;g__Pantoea;s__Pantoea_sp._ARC607

PB1_gene_595	711	ORM75337.1	712	698/712(98.03)	0	anaerobic ribonucleoside-triphosphate reductase [Pantoea deleyi]	470932	d__Bacteria;k__norank;p__Proteobacteria;c__Gammaproteobacteria;o__Enterobacterales;f__Erwiniaceae;g__Pantoea;s__Pantoea_deleyi
PB1_gene_596	294	PAW34451.1	333	294/294(100.00)	1.18e-211	HTH-type transcriptional regulator GalR [Pantoea vagans]	470934	d__Bacteria;k__norank;p__Proteobacteria;c__Gammaproteobacteria;o__Enterobacterales;f__Erwiniaceae;g__Pantoea;s__Pantoea_vagans
PB1_gene_599	278	WP_111138429.1	278	263/278(94.60)	6.63e-187	type 1 glutamine amidotransferase domain-containing protein [Pantoea sp.	2027922	d__Bacteria;k__norank;p__Proteobacteria;c__Gammaproteobacteria;o__Enterobacterales;f__Erwiniaceae;g__Pantoea;s__Pantoea_sp._ARC607
PB1_gene_600	329	ORM76429.1	329	316/329(96.05)	1.92e-232	alcohol dehydrogenase [Pantoea deleyi]	470932	d__Bacteria;k__norank;p__Proteobacteria;c__Gammaproteobacteria;o__Enterobacterales;f__Erwiniaceae;g__Pantoea;s__Pantoea_deleyi
PB1_gene_601	165	TCV01278.1	195	119/162(73.46)	3.71e-80	nicotinamidase-related amidase [Samsonia erythrinae]	160434	d__Bacteria;k__norank;p__Proteobacteria;c__Gammaproteobacteria;o__Enterobacterales;f__Pectobacteriaceae;g__Samsonia;s__Samsonia_erythrinae
PB1_gene_602	313	CNM01793.1	494	200/317(63.09)	3.05e-139	Uncharacterised protein [Yersinia intermedia]	631	d__Bacteria;k__norank;p__Proteobacteria;c__Gammaproteobacteria;o__Enterobacterales;f__Yersiniaceae;g__Yersinia;s__Yersinia_intermedia
PB1_gene_605	339	VDY58686.1	341	205/341(60.12)	7.37e-138	WavE lipopolysaccharide synthesis [Klebsiella pneumoniae]	573	d__Bacteria;k__norank;p__Proteobacteria;c__Gammaproteobacteria;o__Enterobacterales;f__Enterobacteriaceae;g__Klebsiella;s__Klebsiella_pneumoniae
PB1_gene_606	254	WP_108452537.1	271	176/245(71.84)	1.94e-125	glycosyl transferase family 2 [Klebsiella pneumoniae]	573	d__Bacteria;k__norank;p__Proteobacteria;c__Gammaproteobacteria;o__Enterobacterales;f__Enterobacteriaceae;g__Klebsiella;s__Klebsiella_pneumoniae
PB1_gene_607	121	WP_108452536.1	119	77/116(66.38)	1.62e-46	hypothetical protein [Klebsiella pneumoniae]	573	d__Bacteria;k__norank;p__Proteobacteria;c__Gammaproteobacteria;o__Enterobacterales;f__Enterobacteriaceae;g__Klebsiella;s__Klebsiella_pneumoniae
PB1_gene_608	553	WP_108452535.1	579	272/547(49.73)	2.11e-152	hypothetical protein [Klebsiella pneumoniae]	573	d__Bacteria;k__norank;p__Proteobacteria;c__Gammaproteobacteria;o__Enterobacterales;f__Enterobacteriaceae;g__Klebsiella;s__Klebsiella_pneumoniae
PB1_gene_610	205	ORM85452.1	205	201/205(98.05)	1.31e-143	16S rRNA (guanine(527)-N(7))-methyltransferase [Pantoea	470932	d__Bacteria;k__norank;p__Proteobacteria;c__Gammaproteobacteria;o__Enterobacterales;f__Erwiniaceae;g__Pantoea;s__Pantoea_deleyi
PB1_gene_611	629	WP_111140946.1	629	622/629(98.89)	0	tRNA uridine-5-carboxymethylaminomethyl(34) synthesis enzyme MnmG [Pantoea sp. ARC607]	2027922	d__Bacteria;k__norank;p__Proteobacteria;c__Gammaproteobacteria;o__Enterobacterales;f__Erwiniaceae;g__Pantoea;s__Pantoea_sp._ARC607
PB1_gene_612	146	WP_111140945.1	146	141/146(96.58)	5.64e-98	FMN-binding protein MioC [Pantoea sp. ARC607]	2027922	d__Bacteria;k__norank;p__Proteobacteria;c__Gammaproteobacteria;o__Enterobacterales;f__Erwiniaceae;g__Pantoea;s__Pantoea_sp._ARC607
PB1_gene_613	155	ORM85451.1	153	153/153(100.00)	1.94e-102	transcriptional regulator AsnC [Pantoea deleyi]	470932	d__Bacteria;k__norank;p__Proteobacteria;c__Gammaproteobacteria;o__Enterobacterales;f__Erwiniaceae;g__Pantoea;s__Pantoea_deleyi
PB1_gene_619	436	WP_111139905.1	436	425/436(97.48)	8.67e-306	inorganic triphosphatase [Pantoea sp. ARC607]	2027922	d__Bacteria;k__norank;p__Proteobacteria;c__Gammaproteobacteria;o__Enterobacterales;f__Erwiniaceae;g__Pantoea;s__Pantoea_sp._ARC607
PB1_gene_620	950	WP_111139904.1	950	922/950(97.05)	0	bifunctional [glutamate--ammonia ligase]-adenylyl-L-tyrosine phosphorylase/[glutamate--ammonia-ligase] adenylyltransferase [Pantoea sp.	2027922	d__Bacteria;k__norank;p__Proteobacteria;c__Gammaproteobacteria;o__Enterobacterales;f__Erwiniaceae;g__Pantoea;s__Pantoea_sp._ARC607
PB1_gene_622	83	OQV39213.1	83	82/82(100.00)	5.25e-53	sulfurtransferase TusA [Pantoea vagans]	470934	d__Bacteria;k__norank;p__Proteobacteria;c__Gammaproteobacteria;o__Enterobacterales;f__Erwiniaceae;g__Pantoea;s__Pantoea_vagans
PB1_gene_623	224	WP_111141893.1	221	214/216(99.07)	2.43e-150	7-cyano-7-deazaguanine/7-aminomethyl-7-deazaguanine transporter [Pantoea sp. ARC607]	2027922	d__Bacteria;k__norank;p__Proteobacteria;c__Gammaproteobacteria;o__Enterobacterales;f__Erwiniaceae;g__Pantoea;s__Pantoea_sp._ARC607
PB1_gene_624	184	ORM82661.1	184	180/184(97.83)	5.42e-119	hypothetical protein HA42_07930 [Pantoea deleyi]	470932	d__Bacteria;k__norank;p__Proteobacteria;c__Gammaproteobacteria;o__Enterobacterales;f__Erwiniaceae;g__Pantoea;s__Pantoea_deleyi
PB1_gene_625	298	WP_111141889.1	298	284/298(95.30)	4.62e-213	myo-inosose-2 dehydratase [Pantoea sp. ARC607]	2027922	d__Bacteria;k__norank;p__Proteobacteria;c__Gammaproteobacteria;o__Enterobacterales;f__Erwiniaceae;g__Pantoea;s__Pantoea_sp._ARC607
PB1_gene_628	348	ORM78712.1	348	342/348(98.28)	4.94e-265	galactose-1-phosphate uridylyltransferase [Pantoea	470932	d__Bacteria;k__norank;p__Proteobacteria;c__Gammaproteobacteria;o__Enterobacterales;f__Erwiniaceae;g__Pantoea;s__Pantoea_deleyi

PB1_gene_629	382	WP_111141956.1	382	374/382(97.91)	3.98e-270	galactokinase [Pantoea sp. ARC607]	2027922	d__Bacteria;k__norank;p__Proteobacteria;c__Gammaproteobacteria;o__Enterobacterales;f__Erwiniaceae;g__Pantoea;s__Pantoea_sp._ARC607
PB1_gene_630	344	WP_111141955.1	344	331/344(96.22)	8.78e-257	galactose-1-epimerase [Pantoea sp. ARC607]	2027922	d__Bacteria;k__norank;p__Proteobacteria;c__Gammaproteobacteria;o__Enterobacterales;f__Erwiniaceae;g__Pantoea;s__Pantoea_sp._ARC607
PB1_gene_631	250	PZL88446.1	250	250/250(100.00)	5.99e-185	2,3-diphosphoglycerate-dependent phosphoglycerate mutase [Pantoea sp. ARC270]	2027923	d__Bacteria;k__norank;p__Proteobacteria;c__Gammaproteobacteria;o__Enterobacterales;f__Erwiniaceae;g__Pantoea;s__Pantoea_sp._ARC270
PB1_gene_634	64	ORM77282.1	64	61/64(95.31)	4.75e-34	hypothetical protein HA42_17995 [Pantoea deleyi]	470932	d__Bacteria;k__norank;p__Proteobacteria;c__Gammaproteobacteria;o__Enterobacterales;f__Erwiniaceae;g__Pantoea;s__Pantoea_deleyi
PB1_gene_635	289	ORM77283.1	289	280/289(96.89)	1.22e-208	AraC family transcriptional regulator [Pantoea deleyi]	470932	d__Bacteria;k__norank;p__Proteobacteria;c__Gammaproteobacteria;o__Enterobacterales;f__Erwiniaceae;g__Pantoea;s__Pantoea_deleyi
PB1_gene_636	55	WP_111140746.1	55	54/55(98.18)	3.98e-29	DUF2474 domain-containing protein [Pantoea sp. ARC607]	2027922	d__Bacteria;k__norank;p__Proteobacteria;c__Gammaproteobacteria;o__Enterobacterales;f__Erwiniaceae;g__Pantoea;s__Pantoea_sp._ARC607
PB1_gene_637	334	ORM77285.1	334	334/334(100.00)	2.11e-243	cytochrome d ubiquinol oxidase subunit II [Pantoea deleyi]	470932	d__Bacteria;k__norank;p__Proteobacteria;c__Gammaproteobacteria;o__Enterobacterales;f__Erwiniaceae;g__Pantoea;s__Pantoea_deleyi
PB1_gene_638	470	WP_111140748.1	470	467/470(99.36)	0	cytochrome ubiquinol oxidase subunit I [Pantoea sp. ARC607]	2027922	d__Bacteria;k__norank;p__Proteobacteria;c__Gammaproteobacteria;o__Enterobacterales;f__Erwiniaceae;g__Pantoea;s__Pantoea_sp._ARC607
PB1_gene_639	465	ORM85459.1	465	465/465(100.00)	0	F0F1 ATP synthase subunit beta [Pantoea deleyi]	470932	d__Bacteria;k__norank;p__Proteobacteria;c__Gammaproteobacteria;o__Enterobacterales;f__Erwiniaceae;g__Pantoea;s__Pantoea_deleyi
PB1_gene_640	288	ORM85458.1	288	288/288(100.00)	5.30e-198	F0F1 ATP synthase subunit gamma [Pantoea deleyi]	470932	d__Bacteria;k__norank;p__Proteobacteria;c__Gammaproteobacteria;o__Enterobacterales;f__Erwiniaceae;g__Pantoea;s__Pantoea_deleyi
PB1_gene_641	513	SKA11249.1	513	512/513(99.81)	0	F-type H+-transporting ATPase subunit alpha [Pantoea	470933	d__Bacteria;k__norank;p__Proteobacteria;c__Gammaproteobacteria;o__Enterobacterales;f__Erwiniaceae;g__Pantoea;s__Pantoea_eucalypti
PB1_gene_642	177	ORM85457.1	177	177/177(100.00)	8.42e-115	F0F1 ATP synthase subunit delta [Pantoea deleyi]	470932	d__Bacteria;k__norank;p__Proteobacteria;c__Gammaproteobacteria;o__Enterobacterales;f__Erwiniaceae;g__Pantoea;s__Pantoea_deleyi
PB1_gene_645	414	ORM76199.1	414	413/414(99.76)	3.44e-294	N-acetylmuramoyl-L-alanine amidase [Pantoea deleyi]	470932	d__Bacteria;k__norank;p__Proteobacteria;c__Gammaproteobacteria;o__Enterobacterales;f__Erwiniaceae;g__Pantoea;s__Pantoea_deleyi
PB1_gene_647	444	ORM76202.1	442	441/442(99.77)	1.90e-315	amino-acid N-acetyltransferase [Pantoea deleyi]	470932	d__Bacteria;k__norank;p__Proteobacteria;c__Gammaproteobacteria;o__Enterobacterales;f__Erwiniaceae;g__Pantoea;s__Pantoea_deleyi
PB1_gene_650	242	WP_111141441.1	242	229/242(94.63)	4.75e-153	nitrate reductase molybdenum cofactor assembly chaperone [Pantoea sp. ARC607]	2027922	d__Bacteria;k__norank;p__Proteobacteria;c__Gammaproteobacteria;o__Enterobacterales;f__Erwiniaceae;g__Pantoea;s__Pantoea_sp._ARC607
PB1_gene_651	225	WP_111141440.1	225	222/225(98.67)	5.72e-157	respiratory nitrate reductase subunit gamma [Pantoea sp.	2027922	d__Bacteria;k__norank;p__Proteobacteria;c__Gammaproteobacteria;o__Enterobacterales;f__Erwiniaceae;g__Pantoea;s__Pantoea_sp._ARC607
PB1_gene_652	360	ORM77519.1	360	360/360(100.00)	1.95e-268	murein transglycosylase B [Pantoea deleyi]	470932	d__Bacteria;k__norank;p__Proteobacteria;c__Gammaproteobacteria;o__Enterobacterales;f__Erwiniaceae;g__Pantoea;s__Pantoea_deleyi
PB1_gene_653	111	KDA92082.1	111	110/111(99.10)	1.89e-77	hypothetical protein T296_23060 [Pantoea agglomerans Eh318]	1408177	d__Bacteria;k__norank;p__Proteobacteria;c__Gammaproteobacteria;o__Enterobacterales;f__Erwiniaceae;g__Pantoea;s__Pantoea_agglomerans
PB1_gene_654	259	WP_111141445.1	258	241/257(93.77)	2.31e-175	trans-aconitate 2-methyltransferase [Pantoea sp.	2027922	d__Bacteria;k__norank;p__Proteobacteria;c__Gammaproteobacteria;o__Enterobacterales;f__Erwiniaceae;g__Pantoea;s__Pantoea_sp._ARC607
PB1_gene_657	201	ORM83396.1	200	196/199(98.49)	2.34e-128	sulfoxide reductase heme-binding subunit YedZ [Pantoea deleyi]	470932	d__Bacteria;k__norank;p__Proteobacteria;c__Gammaproteobacteria;o__Enterobacterales;f__Erwiniaceae;g__Pantoea;s__Pantoea_deleyi
PB1_gene_658	334	ORM83383.1	334	331/334(99.10)	2.05e-250	mononuclear molybdenum enzyme YedY [Pantoea deleyi]	470932	d__Bacteria;k__norank;p__Proteobacteria;c__Gammaproteobacteria;o__Enterobacterales;f__Erwiniaceae;g__Pantoea;s__Pantoea_deleyi
PB1_gene_659	649	ORM83382.1	649	641/649(98.77)	0	RNase E specificity factor CsrD [Pantoea deleyi]	470932	d__Bacteria;k__norank;p__Proteobacteria;c__Gammaproteobacteria;o__Enterobacterales;f__Erwiniaceae;g__Pantoea;s__Pantoea_deleyi
PB1_gene_660	312	KPA04729.1	287	271/286(94.76)	3.09e-187	Beta-glucoside operon antiterminator (plasmid) [Pantoea	549	d__Bacteria;k__norank;p__Proteobacteria;c__Gammaproteobacteria;o__Enterobacterales;f__Erwiniaceae;g__Pantoea;s__Pantoea_agglomerans
PB1_gene_661	621	WP_090965345.1	619	538/621(86.63)	0	PTS beta-glucoside transporter subunit IIABC [Pantoea sp.	1881036	d__Bacteria;k__norank;p__Proteobacteria;c__Gammaproteobacteria;o__Enterobacterales;f__Erwiniaceae;g__Pantoea;s__Pantoea_sp._OV426

PB1_gene_662	464	WP_061060771.1	464	427/463(92.22)	0	6-phospho-beta-glucosidase [Pantoea vagans]	470934	d__Bacteria;k__norank;p__Proteobacteria;c__Gammaproteobacteria;o__Enterobacterales;f__Erwiniaceae;g__Pantoea;s__Pantoea_vagans
PB1_gene_664	298	ORM77222.1	298	291/298(97.65)	3.05e-227	formate dehydrogenase subunit beta [Pantoea deleyi]	470932	d__Bacteria;k__norank;p__Proteobacteria;c__Gammaproteobacteria;o__Enterobacterales;f__Erwiniaceae;g__Pantoea;s__Pantoea_deleyi
PB1_gene_665	1016	WP_128086547.1	1016	999/1016(98.33)	0	formate dehydrogenase-N subunit alpha [Pantoea deleyi]	470932	d__Bacteria;k__norank;p__Proteobacteria;c__Gammaproteobacteria;o__Enterobacterales;f__Erwiniaceae;g__Pantoea;s__Pantoea_deleyi
PB1_gene_666	235	WP_088900992.1	235	161/231(69.70)	4.56e-113	molecular chaperone [Pantoea sp. AMG 501]	2008894	d__Bacteria;k__norank;p__Proteobacteria;c__Gammaproteobacteria;o__Enterobacterales;f__Erwiniaceae;g__Pantoea;s__Pantoea_sp._AMG_501
PB1_gene_667	439	WP_088900991.1	441	387/435(88.97)	1.30e-270	adhesin [Pantoea sp. AMG 501]	2008894	d__Bacteria;k__norank;p__Proteobacteria;c__Gammaproteobacteria;o__Enterobacterales;f__Erwiniaceae;g__Pantoea;s__Pantoea_sp._AMG_501
PB1_gene_670	306	ORM85283.1	306	295/306(96.41)	6.21e-206	D-alanine--D-alanine ligase [Pantoea deleyi]	470932	d__Bacteria;k__norank;p__Proteobacteria;c__Gammaproteobacteria;o__Enterobacterales;f__Erwiniaceae;g__Pantoea;s__Pantoea_deleyi
PB1_gene_671	279	ORM85284.1	279	278/279(99.64)	1.53e-197	cell division protein FtsQ [Pantoea deleyi]	470932	d__Bacteria;k__norank;p__Proteobacteria;c__Gammaproteobacteria;o__Enterobacterales;f__Erwiniaceae;g__Pantoea;s__Pantoea_deleyi
PB1_gene_672	418	SJZ81890.1	418	418/418(100.00)	6.79e-303	cell division protein FtsA [Pantoea agglomerans]	470933	d__Bacteria;k__norank;p__Proteobacteria;c__Gammaproteobacteria;o__Enterobacterales;f__Erwiniaceae;g__Pantoea;s__Pantoea_eucalypti
PB1_gene_673	384	ORM85285.1	384	384/384(100.00)	4.27e-266	cell division protein FtsZ [Pantoea deleyi]	470932	d__Bacteria;k__norank;p__Proteobacteria;c__Gammaproteobacteria;o__Enterobacterales;f__Erwiniaceae;g__Pantoea;s__Pantoea_deleyi
PB1_gene_676	314	ORM82394.1	314	310/314(98.73)	5.43e-220	transketolase [Pantoea deleyi]	470932	d__Bacteria;k__norank;p__Proteobacteria;c__Gammaproteobacteria;o__Enterobacterales;f__Erwiniaceae;g__Pantoea;s__Pantoea_deleyi
PB1_gene_677	276	ORM82393.1	276	271/276(98.19)	8.52e-198	transketolase [Pantoea deleyi]	470932	d__Bacteria;k__norank;p__Proteobacteria;c__Gammaproteobacteria;o__Enterobacterales;f__Erwiniaceae;g__Pantoea;s__Pantoea_deleyi
PB1_gene_678	318	ORM82392.1	318	314/318(98.74)	1.49e-219	DNA-binding transcriptional regulator [Pantoea deleyi]	470932	d__Bacteria;k__norank;p__Proteobacteria;c__Gammaproteobacteria;o__Enterobacterales;f__Erwiniaceae;g__Pantoea;s__Pantoea_deleyi
PB1_gene_679	163	ADO09191.1	163	162/163(99.39)	3.14e-110	putative membrane-bound lytic murein transglycosylase E [Pantoea vagans C9-1]	712898	d__Bacteria;k__norank;p__Proteobacteria;c__Gammaproteobacteria;o__Enterobacterales;f__Erwiniaceae;g__Pantoea;s__Pantoea_vagans
PB1_gene_681	414	WP_111138673.1	414	409/414(98.79)	2.43e-278	lipoprotein-releasing ABC transporter permease subunit LolE [Pantoea sp. ARC607]	2027922	d__Bacteria;k__norank;p__Proteobacteria;c__Gammaproteobacteria;o__Enterobacterales;f__Erwiniaceae;g__Pantoea;s__Pantoea_sp._ARC607
PB1_gene_682	234	ORM82364.1	234	232/234(99.15)	1.97e-157	lipoprotein ABC transporter ATP-binding protein LolD [Pantoea deleyi]	470932	d__Bacteria;k__norank;p__Proteobacteria;c__Gammaproteobacteria;o__Enterobacterales;f__Erwiniaceae;g__Pantoea;s__Pantoea_deleyi
PB1_gene_683	399	WP_111138671.1	399	394/399(98.75)	1.37e-265	lipoprotein-releasing ABC transporter permease subunit LolC [Pantoea sp. ARC607]	2027922	d__Bacteria;k__norank;p__Proteobacteria;c__Gammaproteobacteria;o__Enterobacterales;f__Erwiniaceae;g__Pantoea;s__Pantoea_sp._ARC607
PB1_gene_684	75	ORM82362.1	69	65/69(94.20)	9.98e-42	hypothetical protein HA42_08340 [Pantoea deleyi]	470932	d__Bacteria;k__norank;p__Proteobacteria;c__Gammaproteobacteria;o__Enterobacterales;f__Erwiniaceae;g__Pantoea;s__Pantoea_deleyi
PB1_gene_688	285	ORM85207.1	266	254/266(95.49)	7.80e-194	MBL fold metallo-hydrolase [Pantoea deleyi]	470932	d__Bacteria;k__norank;p__Proteobacteria;c__Gammaproteobacteria;o__Enterobacterales;f__Erwiniaceae;g__Pantoea;s__Pantoea_deleyi
PB1_gene_689	371	WP_111138327.1	372	357/371(96.23)	9.57e-243	metallophosphoesterase [Pantoea sp. ARC607]	2027922	d__Bacteria;k__norank;p__Proteobacteria;c__Gammaproteobacteria;o__Enterobacterales;f__Erwiniaceae;g__Pantoea;s__Pantoea_sp._ARC607
PB1_gene_691	564	ORM85857.1	564	558/564(98.94)	0	Kef family K(+) transporter [Pantoea deleyi]	470932	d__Bacteria;k__norank;p__Proteobacteria;c__Gammaproteobacteria;o__Enterobacterales;f__Erwiniaceae;g__Pantoea;s__Pantoea_deleyi
PB1_gene_692	433	WP_111140318.1	433	426/433(98.38)	0	inosine/guanosine kinase [Pantoea sp. ARC607]	2027922	d__Bacteria;k__norank;p__Proteobacteria;c__Gammaproteobacteria;o__Enterobacterales;f__Erwiniaceae;g__Pantoea;s__Pantoea_sp._ARC607
PB1_gene_693	319	ORM85859.1	319	319/319(100.00)	5.78e-238	ferrochelatase [Pantoea deleyi]	470932	d__Bacteria;k__norank;p__Proteobacteria;c__Gammaproteobacteria;o__Enterobacterales;f__Erwiniaceae;g__Pantoea;s__Pantoea_deleyi
PB1_gene_696	322	ORM82646.1	322	315/322(97.83)	1.46e-228	diguanylate cyclase [Pantoea deleyi]	470932	d__Bacteria;k__norank;p__Proteobacteria;c__Gammaproteobacteria;o__Enterobacterales;f__Erwiniaceae;g__Pantoea;s__Pantoea_deleyi

PB1_gene_697	327	ORM82647.1	325	324/325(99.69)	3.86e-226	delta-aminolevulinic acid dehydratase [Pantoea deleyi]	470932	d__Bacteria;k__norank;p__Proteobacteria;c__Gammaproteobacteria;o__Enterobacterales;f__Erwiniaceae;g__Pantoea;s__Pantoea_deleyi
PB1_gene_698	501	WP_111139276.1	501	492/501(98.20)	0	glycine betaine/L-proline transporter ProP [Pantoea sp.	2027922	d__Bacteria;k__norank;p__Proteobacteria;c__Gammaproteobacteria;o__Enterobacterales;f__Erwiniaceae;g__Pantoea;s__Pantoea_sp._ARC607
PB1_gene_709	235	CVD85396.1	381	128/234(54.70)	3.91e-58	Uncharacterised protein [Serratia marcescens]	615	d__Bacteria;k__norank;p__Proteobacteria;c__Gammaproteobacteria;o__Enterobacterales;f__Yersiniaceae;g__Serratia;s__Serratia_marcescens
PB1_gene_711	60	AJZ89769.1	114	29/44(65.91)	5.38e-08	hypothetical protein VW41_12350 [Klebsiella	1134687	d__Bacteria;k__norank;p__Proteobacteria;c__Gammaproteobacteria;o__Enterobacterales;f__Enterobacteriaceae;g__Klebsiella;s__Klebsiella_michiganensis
PB1_gene_715	588	ORM85275.1	588	588/588(100.00)	0	peptidoglycan glycosyltransferase FtsI [Pantoea deleyi]	470932	d__Bacteria;k__norank;p__Proteobacteria;c__Gammaproteobacteria;o__Enterobacterales;f__Erwiniaceae;g__Pantoea;s__Pantoea_deleyi
PB1_gene_716	106	SJZ82074.1	106	106/106(100.00)	5.81e-63	cell division protein FtsL [Pantoea agglomerans]	470933	d__Bacteria;k__norank;p__Proteobacteria;c__Gammaproteobacteria;o__Enterobacterales;f__Erwiniaceae;g__Pantoea;s__Pantoea_eucalypti
PB1_gene_717	313	WP_111139400.1	313	307/313(98.08)	3.79e-215	16S rRNA (cytosine(1402)-N(4))-methyltransferase [Pantoea sp. ARC607]	2027922	d__Bacteria;k__norank;p__Proteobacteria;c__Gammaproteobacteria;o__Enterobacterales;f__Erwiniaceae;g__Pantoea;s__Pantoea_sp._ARC607
PB1_gene_718	152	ORM85273.1	152	152/152(100.00)	2.35e-102	cell division/cell wall cluster transcriptional repressor MraZ [Pantoea deleyi]	470932	d__Bacteria;k__norank;p__Proteobacteria;c__Gammaproteobacteria;o__Enterobacterales;f__Erwiniaceae;g__Pantoea;s__Pantoea_deleyi
PB1_gene_720	274	RWS72644.1	278	263/273(96.34)	1.24e-190	Cof-type HAD-IIB family hydrolase [Enterobacter cloacae]	550	d__Bacteria;k__norank;p__Proteobacteria;c__Gammaproteobacteria;o__Enterobacterales;f__Enterobacteriaceae;g__Enterobacter;s__Enterobacter_cloacae
PB1_gene_721	77	KPA08544.1	99	67/72(93.06)	2.72e-43	hypothetical protein PAP10c_0583 [Pantoea	549	d__Bacteria;k__norank;p__Proteobacteria;c__Gammaproteobacteria;o__Enterobacterales;f__Erwiniaceae;g__Pantoea;s__Pantoea_agglomerans
PB1_gene_722	333	ORM76133.1	333	328/333(98.50)	3.67e-248	6-phosphogluconolactonase [Pantoea deleyi]	470932	d__Bacteria;k__norank;p__Proteobacteria;c__Gammaproteobacteria;o__Enterobacterales;f__Erwiniaceae;g__Pantoea;s__Pantoea_deleyi
PB1_gene_723	158	ORM76132.1	158	156/158(98.73)	1.64e-114	kinase inhibitor [Pantoea deleyi]	470932	d__Bacteria;k__norank;p__Proteobacteria;c__Gammaproteobacteria;o__Enterobacterales;f__Erwiniaceae;g__Pantoea;s__Pantoea_deleyi
PB1_gene_724	439	ORM76131.1	434	427/434(98.39)	0	adenosylmethionine--8-amino-7-oxononanoate transaminase [Pantoea deleyi]	470932	d__Bacteria;k__norank;p__Proteobacteria;c__Gammaproteobacteria;o__Enterobacterales;f__Erwiniaceae;g__Pantoea;s__Pantoea_deleyi
PB1_gene_725	310	WP_059307811.1	305	248/305(81.31)	1.79e-176	LysR family transcriptional regulator [Leclercia	83655	d__Bacteria;k__norank;p__Proteobacteria;c__Gammaproteobacteria;o__Enterobacterales;f__Enterobacteriaceae;g__Leclercia;s__Leclercia_adecarboxylata
PB1_gene_726	217	WP_059307810.1	217	185/217(85.25)	9.09e-125	SDR family NAD(P)-dependent oxidoreductase [Leclercia adecarboxylata]	83655	d__Bacteria;k__norank;p__Proteobacteria;c__Gammaproteobacteria;o__Enterobacterales;f__Enterobacteriaceae;g__Leclercia;s__Leclercia_adecarboxylata
PB1_gene_727	290	WP_130016394.1	292	253/289(87.54)	7.14e-177	LysR family transcriptional regulator [Serratia liquefaciens]	614	d__Bacteria;k__norank;p__Proteobacteria;c__Gammaproteobacteria;o__Enterobacterales;f__Yersiniaceae;g__Serratia;s__Serratia_liquefaciens
PB1_gene_728	144	WP_024906932.1	306	120/144(83.33)	5.82e-79	NAD-dependent epimerase/dehydratase family protein [Enterobacter asburiae]	61645	d__Bacteria;k__norank;p__Proteobacteria;c__Gammaproteobacteria;o__Enterobacterales;f__Enterobacteriaceae;g__Enterobacter;s__Enterobacter_asburiae
PB1_gene_731	483	ORM86561.1	479	473/479(98.75)	0	6-phospho-beta-glucosidase [Pantoea deleyi]	470932	d__Bacteria;k__norank;p__Proteobacteria;c__Gammaproteobacteria;o__Enterobacterales;f__Erwiniaceae;g__Pantoea;s__Pantoea_deleyi
PB1_gene_732	238	ORM86562.1	238	235/238(98.74)	3.30e-167	transcriptional regulator [Pantoea deleyi]	470932	d__Bacteria;k__norank;p__Proteobacteria;c__Gammaproteobacteria;o__Enterobacterales;f__Erwiniaceae;g__Pantoea;s__Pantoea_deleyi
PB1_gene_733	223	ORM86563.1	221	218/221(98.64)	1.26e-154	hemolysin III family protein [Pantoea deleyi]	470932	d__Bacteria;k__norank;p__Proteobacteria;c__Gammaproteobacteria;o__Enterobacterales;f__Erwiniaceae;g__Pantoea;s__Pantoea_deleyi
PB1_gene_735	491	ORM82304.1	491	478/491(97.35)	0	SulP family transporter [Pantoea deleyi]	470932	d__Bacteria;k__norank;p__Proteobacteria;c__Gammaproteobacteria;o__Enterobacterales;f__Erwiniaceae;g__Pantoea;s__Pantoea_deleyi
PB1_gene_736	211	WP_111141347.1	211	205/211(97.16)	2.08e-143	carbonic anhydrase [Pantoea sp. ARC607]	2027922	d__Bacteria;k__norank;p__Proteobacteria;c__Gammaproteobacteria;o__Enterobacterales;f__Erwiniaceae;g__Pantoea;s__Pantoea_sp._ARC607
PB1_gene_737	270	ORM82306.1	269	268/269(99.63)	9.58e-194	5'-nucleotidase, lipoprotein e(P4) family [Pantoea deleyi]	470932	d__Bacteria;k__norank;p__Proteobacteria;c__Gammaproteobacteria;o__Enterobacterales;f__Erwiniaceae;g__Pantoea;s__Pantoea_deleyi

PB1_gene_738	242	WP_098052322.1	235	212/235(90.21)	9.46e-156	laccase domain-containing protein [Pantoea agglomerans]	None	Unclassified
PB1_gene_740	109	ORM86841.1	109	95/109(87.16)	4.16e-53	multidrug transporter subunit MdtI [Pantoea deleyi]	470932	d__Bacteria;k__norank;p__Proteobacteria;c__Gammaproteobacteria;o__Enterobacterales;f__Erwiniaceae;g__Pantoea;s__Pantoea_deleyi
PB1_gene_741	380	ORM86840.1	380	376/380(98.95)	2.99e-264	acyltransferase [Pantoea deleyi]	470932	d__Bacteria;k__norank;p__Proteobacteria;c__Gammaproteobacteria;o__Enterobacterales;f__Erwiniaceae;g__Pantoea;s__Pantoea_deleyi
PB1_gene_742	86	WP_111138836.1	86	83/86(96.51)	2.27e-53	hypothetical protein [Pantoea sp. ARC607]	2027922	d__Bacteria;k__norank;p__Proteobacteria;c__Gammaproteobacteria;o__Enterobacterales;f__Erwiniaceae;g__Pantoea;s__Pantoea_sp._ARC607
PB1_gene_744	45	PXW18960.1	45	42/45(93.33)	1.48e-20	hypothetical protein BY447_0522 [Pantoea sp. JKS000250]	1938795	d__Bacteria;k__norank;p__Proteobacteria;c__Gammaproteobacteria;o__Enterobacterales;f__Erwiniaceae;g__Pantoea;s__Pantoea_sp._JKS000250
PB1_gene_747	181	ORM82471.1	181	179/181(98.90)	1.24e-118	hypothetical protein HA42_08205 [Pantoea deleyi]	470932	d__Bacteria;k__norank;p__Proteobacteria;c__Gammaproteobacteria;o__Enterobacterales;f__Erwiniaceae;g__Pantoea;s__Pantoea_deleyi
PB1_gene_748	450	WP_111139266.1	450	446/450(99.11)	0	UDP-N-acetylmuramate:L-alanyl-gamma-D-glutamyl-meso-diaminopimelate ligase [Pantoea sp. ARC607]	2027922	d__Bacteria;k__norank;p__Proteobacteria;c__Gammaproteobacteria;o__Enterobacterales;f__Erwiniaceae;g__Pantoea;s__Pantoea_sp._ARC607
PB1_gene_749	335	ORM82473.1	335	335/335(100.00)	6.10e-248	fructose-bisphosphatase [Pantoea deleyi]	470932	d__Bacteria;k__norank;p__Proteobacteria;c__Gammaproteobacteria;o__Enterobacterales;f__Erwiniaceae;g__Pantoea;s__Pantoea_deleyi
PB1_gene_752	241	ORM86092.1	252	238/241(98.76)	2.10e-174	phosphatidylglycerophosphatase B [Pantoea deleyi]	470932	d__Bacteria;k__norank;p__Proteobacteria;c__Gammaproteobacteria;o__Enterobacterales;f__Erwiniaceae;g__Pantoea;s__Pantoea_deleyi
PB1_gene_753	210	KDA93170.1	227	208/210(99.05)	3.30e-148	GTP cyclohydrolase II [Pantoea agglomerans Eh318]	1408177	d__Bacteria;k__norank;p__Proteobacteria;c__Gammaproteobacteria;o__Enterobacterales;f__Erwiniaceae;g__Pantoea;s__Pantoea_agglomerans
PB1_gene_757	339	ORM82831.1	339	334/339(98.53)	2.75e-237	cell division protein YceG [Pantoea deleyi]	470932	d__Bacteria;k__norank;p__Proteobacteria;c__Gammaproteobacteria;o__Enterobacterales;f__Erwiniaceae;g__Pantoea;s__Pantoea_deleyi
PB1_gene_758	271	ORM82830.1	271	265/271(97.79)	4.02e-189	aminodeoxychorismate lyase [Pantoea deleyi]	470932	d__Bacteria;k__norank;p__Proteobacteria;c__Gammaproteobacteria;o__Enterobacterales;f__Erwiniaceae;g__Pantoea;s__Pantoea_deleyi
PB1_gene_759	401	ORM82829.1	413	399/401(99.50)	9.96e-288	beta-ketoacyl-[acyl-carrier-protein] synthase II [Pantoea	470932	d__Bacteria;k__norank;p__Proteobacteria;c__Gammaproteobacteria;o__Enterobacterales;f__Erwiniaceae;g__Pantoea;s__Pantoea_deleyi
PB1_gene_762	448	ORM80481.1	448	443/448(98.88)	0	glucarate dehydratase [Pantoea deleyi]	470932	d__Bacteria;k__norank;p__Proteobacteria;c__Gammaproteobacteria;o__Enterobacterales;f__Erwiniaceae;g__Pantoea;s__Pantoea_deleyi
PB1_gene_763	453	ORM80482.1	453	448/453(98.90)	0	glucarate transporter [Pantoea deleyi]	470932	d__Bacteria;k__norank;p__Proteobacteria;c__Gammaproteobacteria;o__Enterobacterales;f__Erwiniaceae;g__Pantoea;s__Pantoea_deleyi
PB1_gene_767	534	WP_111140395.1	583	528/534(98.88)	0	gamma-glutamyltransferase [Pantoea sp. ARC607]	2027922	d__Bacteria;k__norank;p__Proteobacteria;c__Gammaproteobacteria;o__Enterobacterales;f__Erwiniaceae;g__Pantoea;s__Pantoea_sp._ARC607
PB1_gene_768	202	WP_111140411.1	175	162/175(92.57)	2.15e-114	GNAT family N-acetyltransferase [Pantoea sp. ARC607]	2027922	d__Bacteria;k__norank;p__Proteobacteria;c__Gammaproteobacteria;o__Enterobacterales;f__Erwiniaceae;g__Pantoea;s__Pantoea_sp._ARC607
PB1_gene_769	231	ORM80116.1	231	230/231(99.57)	9.93e-170	quercetin 2,3-dioxygenase [Pantoea deleyi]	470932	d__Bacteria;k__norank;p__Proteobacteria;c__Gammaproteobacteria;o__Enterobacterales;f__Erwiniaceae;g__Pantoea;s__Pantoea_deleyi
PB1_gene_770	331	ORM80115.1	331	329/331(99.40)	6.44e-232	transcriptional regulator [Pantoea deleyi]	470932	d__Bacteria;k__norank;p__Proteobacteria;c__Gammaproteobacteria;o__Enterobacterales;f__Erwiniaceae;g__Pantoea;s__Pantoea_deleyi
PB1_gene_772	386	ORM82207.1	386	383/386(99.22)	2.40e-298	hypothetical protein HA42_08885 [Pantoea deleyi]	470932	d__Bacteria;k__norank;p__Proteobacteria;c__Gammaproteobacteria;o__Enterobacterales;f__Erwiniaceae;g__Pantoea;s__Pantoea_deleyi
PB1_gene_773	223	WP_111139898.1	223	220/223(98.65)	2.15e-151	DUF1190 family protein [Pantoea sp. ARC607]	2027922	d__Bacteria;k__norank;p__Proteobacteria;c__Gammaproteobacteria;o__Enterobacterales;f__Erwiniaceae;g__Pantoea;s__Pantoea_sp._ARC607
PB1_gene_774	617	ORM82205.1	488	458/488(93.85)	7.53e-302	outer membrane channel protein TolC [Pantoea deleyi]	470932	d__Bacteria;k__norank;p__Proteobacteria;c__Gammaproteobacteria;o__Enterobacterales;f__Erwiniaceae;g__Pantoea;s__Pantoea_deleyi
PB1_gene_777	662	ORM82061.1	662	626/662(94.56)	0	alpha-glucuronidase [Pantoea deleyi]	470932	d__Bacteria;k__norank;p__Proteobacteria;c__Gammaproteobacteria;o__Enterobacterales;f__Erwiniaceae;g__Pantoea;s__Pantoea_deleyi
PB1_gene_780	295	ERM10752.1	294	282/290(97.24)	6.90e-179	peptidyl-prolyl cis-trans isomerase [Pantoea agglomerans	1332072	d__Bacteria;k__norank;p__Proteobacteria;c__Gammaproteobacteria;o__Enterobacterales;f__Erwiniaceae;g__Pantoea;s__Pantoea_agglomerans

PB1_gene_781	72	ORM81815.1	72	69/72(95.83)	5.35e-39	lysis protein [Pantoea deleyi]	470932	d__Bacteria;k__norank;p__Proteobacteria;c__Gammaproteobacteria;o__Enterobacterales;f__Erwiniaceae;g__Pantoea;s__Pantoea_deleyi
PB1_gene_782	186	KTR47460.1	182	158/158(100.00)	4.44e-109	peptidylprolyl isomerase [Pantoea ananatis]	553	d__Bacteria;k__norank;p__Proteobacteria;c__Gammaproteobacteria;o__Enterobacterales;f__Erwiniaceae;g__Pantoea;s__Pantoea_ananatis
PB1_gene_783	64	OQV39106.1	64	64/64(100.00)	1.90e-42	hypothetical protein BZ160_17810 [Pantoea vagans]	470934	d__Bacteria;k__norank;p__Proteobacteria;c__Gammaproteobacteria;o__Enterobacterales;f__Erwiniaceae;g__Pantoea;s__Pantoea_vagans
PB1_gene_785	395	WP_111139915.1	395	382/395(96.71)	1.81e-252	MFS transporter [Pantoea sp. ARC607]	2027922	d__Bacteria;k__norank;p__Proteobacteria;c__Gammaproteobacteria;o__Enterobacterales;f__Erwiniaceae;g__Pantoea;s__Pantoea_sp._ARC607
PB1_gene_786	320	ORM81218.1	319	309/319(96.87)	3.30e-226	class 1b ribonucleoside-diphosphate reductase subunit beta [Pantoea deleyi]	470932	d__Bacteria;k__norank;p__Proteobacteria;c__Gammaproteobacteria;o__Enterobacterales;f__Erwiniaceae;g__Pantoea;s__Pantoea_deleyi
PB1_gene_794	199	PZL87905.1	199	194/199(97.49)	8.93e-139	16S rRNA (guanine(966)-N(2))-methyltransferase [Pantoea sp. ARC270]	2027923	d__Bacteria;k__norank;p__Proteobacteria;c__Gammaproteobacteria;o__Enterobacterales;f__Erwiniaceae;g__Pantoea;s__Pantoea_sp._ARC270
PB1_gene_795	81	PZL87904.1	90	81/81(100.00)	1.71e-52	DUF1145 domain-containing protein [Pantoea sp. ARC270]	2027923	d__Bacteria;k__norank;p__Proteobacteria;c__Gammaproteobacteria;o__Enterobacterales;f__Erwiniaceae;g__Pantoea;s__Pantoea_sp._ARC270
PB1_gene_796	132	WP_111141899.1	118	116/118(98.31)	1.50e-72	DUF2500 domain-containing protein [Pantoea sp. ARC607]	2027922	d__Bacteria;k__norank;p__Proteobacteria;c__Gammaproteobacteria;o__Enterobacterales;f__Erwiniaceae;g__Pantoea;s__Pantoea_sp._ARC607
PB1_gene_797	209	ORM80414.1	209	208/209(99.52)	5.55e-147	hypothetical protein HA42_14775 [Pantoea deleyi]	470932	d__Bacteria;k__norank;p__Proteobacteria;c__Gammaproteobacteria;o__Enterobacterales;f__Erwiniaceae;g__Pantoea;s__Pantoea_deleyi
PB1_gene_801	832	WP_063877279.1	815	569/807(70.51)	0	fimbrial assembly protein [Pantoea sp. OXWO6B1]	None	Unclassified
PB1_gene_806	406	WP_111139712.1	406	397/406(97.78)	2.80e-289	aspartate aminotransferase family protein [Pantoea sp. ARC607]	2027922	d__Bacteria;k__norank;p__Proteobacteria;c__Gammaproteobacteria;o__Enterobacterales;f__Erwiniaceae;g__Pantoea;s__Pantoea_sp._ARC607
PB1_gene_807	191	ORM81832.1	191	191/191(100.00)	2.30e-137	anthranilate/aminodeoxychorismate synthase component II [Pantoea deleyi]	470932	d__Bacteria;k__norank;p__Proteobacteria;c__Gammaproteobacteria;o__Enterobacterales;f__Erwiniaceae;g__Pantoea;s__Pantoea_deleyi
PB1_gene_808	197	ORM81833.1	197	191/197(96.95)	6.30e-138	cell filamentation protein Fic [Pantoea deleyi]	470932	d__Bacteria;k__norank;p__Proteobacteria;c__Gammaproteobacteria;o__Enterobacterales;f__Erwiniaceae;g__Pantoea;s__Pantoea_deleyi
PB1_gene_809	56	WP_128085332.1	56	55/55(100.00)	2.56e-30	DUF2559 family protein [Pantoea deleyi]	470932	d__Bacteria;k__norank;p__Proteobacteria;c__Gammaproteobacteria;o__Enterobacterales;f__Erwiniaceae;g__Pantoea;s__Pantoea_deleyi
PB1_gene_811	91	TCZ28779.1	91	87/91(95.60)	1.05e-58	type II toxin-antitoxin system RelE/ParE family toxin [Pantoea agglomerans]	549	d__Bacteria;k__norank;p__Proteobacteria;c__Gammaproteobacteria;o__Enterobacterales;f__Erwiniaceae;g__Pantoea;s__Pantoea_agglomerans
PB1_gene_812	286	EIC00064.1	286	280/286(97.90)	2.16e-196	hypothetical protein S7A_16170 [Pantoea sp. Sc1]	593105	d__Bacteria;k__norank;p__Proteobacteria;c__Gammaproteobacteria;o__Enterobacterales;f__Erwiniaceae;g__Pantoea;s__Pantoea_sp._Sc1
PB1_gene_813	474	WP_122889249.1	474	461/474(97.26)	3.54e-315	PTS fructose transporter subunit IIBC [[Curtobacterium]	221276	d__Bacteria;k__norank;p__Proteobacteria;c__Gammaproteobacteria;o__Enterobacterales;f__Erwiniaceae;g__Pantoea;s__[Curtobacterium]_plantarum
PB1_gene_814	157	WP_090964058.1	157	149/155(96.13)	2.71e-99	PTS sugar transporter subunit IIA [Pantoea sp. OV426]	1881036	d__Bacteria;k__norank;p__Proteobacteria;c__Gammaproteobacteria;o__Enterobacterales;f__Erwiniaceae;g__Pantoea;s__Pantoea_sp._OV426
PB1_gene_817	448	ORM83858.1	448	447/448(99.78)	0	NADH-quinone oxidoreductase subunit F [Pantoea deleyi]	470932	d__Bacteria;k__norank;p__Proteobacteria;c__Gammaproteobacteria;o__Enterobacterales;f__Erwiniaceae;g__Pantoea;s__Pantoea_deleyi
PB1_gene_818	171	ORM83859.1	171	170/171(99.42)	2.19e-123	NADH-quinone oxidoreductase subunit E [Pantoea deleyi]	470932	d__Bacteria;k__norank;p__Proteobacteria;c__Gammaproteobacteria;o__Enterobacterales;f__Erwiniaceae;g__Pantoea;s__Pantoea_deleyi
PB1_gene_819	599	WP_111140113.1	599	597/599(99.67)	0	NADH-quinone oxidoreductase subunit C/D [Pantoea sp.	2027922	d__Bacteria;k__norank;p__Proteobacteria;c__Gammaproteobacteria;o__Enterobacterales;f__Erwiniaceae;g__Pantoea;s__Pantoea_sp._ARC607
PB1_gene_821	475	WP_111140682.1	475	418/475(88.00)	9.80e-309	bifunctional 2-methylcitrate dehydratase/aconitate hydratase [Pantoea sp. ARC607]	2027922	d__Bacteria;k__norank;p__Proteobacteria;c__Gammaproteobacteria;o__Enterobacterales;f__Erwiniaceae;g__Pantoea;s__Pantoea_sp._ARC607
PB1_gene_823	295	WP_111208619.1	295	262/295(88.81)	8.00e-192	LysR family transcriptional regulator [Pantoea sp. ARC270]	2630326	d__Bacteria;k__norank;p__Proteobacteria;c__Gammaproteobacteria;o__Enterobacterales;f__Erwiniaceae;g__Pantoea;s__norank

PB1_gene_825	835	WP_111140277.1	841	830/836(99.28)	0	bifunctional glycosyl transferase/transpeptidase [Pantoea sp. ARC607]	2027922	d__Bacteria;k__norank;p__Proteobacteria;c__Gammaproteobacteria;o__Enterobacterales;f__Erwiniaceae;g__Pantoea;s__Pantoea_sp._ARC607
PB1_gene_828	146	ORM80793.1	146	136/146(93.15)	9.36e-97	hypothetical protein HA42_12980 [Pantoea deleyi]	470932	d__Bacteria;k__norank;p__Proteobacteria;c__Gammaproteobacteria;o__Enterobacterales;f__Erwiniaceae;g__Pantoea;s__Pantoea_deleyi
PB1_gene_829	124	OQV43052.1	124	124/124(100.00)	6.03e-87	Fe-S cluster assembly scaffold SufA [Pantoea vagans]	470934	d__Bacteria;k__norank;p__Proteobacteria;c__Gammaproteobacteria;o__Enterobacterales;f__Erwiniaceae;g__Pantoea;s__Pantoea_vagans
PB1_gene_832	324	SKA24045.1	324	311/324(95.99)	3.48e-237	ADP-L-glycero-D-manno-heptose 6-epimerase [Pantoea	470933	d__Bacteria;k__norank;p__Proteobacteria;c__Gammaproteobacteria;o__Enterobacterales;f__Erwiniaceae;g__Pantoea;s__Pantoea_eucalypti
PB1_gene_833	351	WP_086906675.1	351	343/351(97.72)	2.07e-254	ADP-heptose--LPS heptosyltransferase RfaF [Pantoea agglomerans]	549	d__Bacteria;k__norank;p__Proteobacteria;c__Gammaproteobacteria;o__Enterobacterales;f__Erwiniaceae;g__Pantoea;s__Pantoea_agglomerans
PB1_gene_834	325	ORM84509.1	325	318/325(97.85)	8.86e-236	lipopolysaccharide heptosyltransferase 1 [Pantoea deleyi]	470932	d__Bacteria;k__norank;p__Proteobacteria;c__Gammaproteobacteria;o__Enterobacterales;f__Erwiniaceae;g__Pantoea;s__Pantoea_deleyi
PB1_gene_835	162	ORM84142.1	154	152/154(98.70)	1.49e-108	(2Fe-2S)-binding protein [Pantoea deleyi]	470932	d__Bacteria;k__norank;p__Proteobacteria;c__Gammaproteobacteria;o__Enterobacterales;f__Erwiniaceae;g__Pantoea;s__Pantoea_deleyi
PB1_gene_836	330	ORM84143.1	330	318/330(96.36)	1.75e-224	FAD-binding molybdopterin dehydrogenase [Pantoea deleyi]	470932	d__Bacteria;k__norank;p__Proteobacteria;c__Gammaproteobacteria;o__Enterobacterales;f__Erwiniaceae;g__Pantoea;s__Pantoea_deleyi
PB1_gene_837	764	ORM84144.1	764	747/764(97.77)	0	oxidoreductase [Pantoea deleyi]	470932	d__Bacteria;k__norank;p__Proteobacteria;c__Gammaproteobacteria;o__Enterobacterales;f__Erwiniaceae;g__Pantoea;s__Pantoea_deleyi
PB1_gene_839	389	PAW35901.1	389	368/389(94.60)	1.33e-251	secretion protein HlyD [Pantoea vagans]	470934	d__Bacteria;k__norank;p__Proteobacteria;c__Gammaproteobacteria;o__Enterobacterales;f__Erwiniaceae;g__Pantoea;s__Pantoea_vagans
PB1_gene_842	1024	WP_111139182.1	1024	1008/1023(98.53)	0	multidrug transporter subunit MdtC [Pantoea sp. ARC607]	2027922	d__Bacteria;k__norank;p__Proteobacteria;c__Gammaproteobacteria;o__Enterobacterales;f__Erwiniaceae;g__Pantoea;s__Pantoea_sp._ARC607
PB1_gene_845	126	ORM81413.1	126	114/126(90.48)	2.25e-74	hypothetical protein HA42_10785 [Pantoea deleyi]	470932	d__Bacteria;k__norank;p__Proteobacteria;c__Gammaproteobacteria;o__Enterobacterales;f__Erwiniaceae;g__Pantoea;s__Pantoea_deleyi
PB1_gene_846	774	WP_111138308.1	774	709/774(91.60)	0	Fe(2+) transporter permease subunit FeoB [Pantoea sp.	2027922	d__Bacteria;k__norank;p__Proteobacteria;c__Gammaproteobacteria;o__Enterobacterales;f__Erwiniaceae;g__Pantoea;s__Pantoea_sp._ARC607
PB1_gene_849	347	WP_111138620.1	347	338/347(97.41)	4.34e-254	dihydroorotase [Pantoea sp. ARC607]	2027922	d__Bacteria;k__norank;p__Proteobacteria;c__Gammaproteobacteria;o__Enterobacterales;f__Erwiniaceae;g__Pantoea;s__Pantoea_sp._ARC607
PB1_gene_850	187	ORM81048.1	187	185/187(98.93)	1.09e-124	hypothetical protein HA42_12080 [Pantoea deleyi]	470932	d__Bacteria;k__norank;p__Proteobacteria;c__Gammaproteobacteria;o__Enterobacterales;f__Erwiniaceae;g__Pantoea;s__Pantoea_deleyi
PB1_gene_851	215	ORM81049.1	215	210/215(97.67)	1.93e-150	glutaredoxin, GrxB family [Pantoea deleyi]	470932	d__Bacteria;k__norank;p__Proteobacteria;c__Gammaproteobacteria;o__Enterobacterales;f__Erwiniaceae;g__Pantoea;s__Pantoea_deleyi
PB1_gene_852	197	ORM81050.1	197	197/197(100.00)	4.45e-147	ribosomal protein S5 alanine N-acetyltransferase [Pantoea deleyi]	470932	d__Bacteria;k__norank;p__Proteobacteria;c__Gammaproteobacteria;o__Enterobacterales;f__Erwiniaceae;g__Pantoea;s__Pantoea_deleyi
PB1_gene_853	212	ORM81051.1	212	203/212(95.75)	2.59e-133	hypothetical protein HA42_12095 [Pantoea deleyi]	470932	d__Bacteria;k__norank;p__Proteobacteria;c__Gammaproteobacteria;o__Enterobacterales;f__Erwiniaceae;g__Pantoea;s__Pantoea_deleyi
PB1_gene_855	97	WP_089561849.1	97	85/97(87.63)	4.64e-49	MULTISPECIES: citrate lyase acyl carrier protein [Pantoea]	53335	d__Bacteria;k__norank;p__Proteobacteria;c__Gammaproteobacteria;o__Enterobacterales;f__Erwiniaceae;g__Pantoea;s__norank
PB1_gene_856	291	WP_089561850.1	291	270/291(92.78)	1.04e-185	MULTISPECIES: citrate (pro-3S)-lyase subunit beta [Pantoea]	53335	d__Bacteria;k__norank;p__Proteobacteria;c__Gammaproteobacteria;o__Enterobacterales;f__Erwiniaceae;g__Pantoea;s__norank
PB1_gene_857	505	WP_063163583.1	505	431/505(85.35)	2.21e-311	citrate lyase subunit alpha [Enterobacter cloacae]	158836	d__Bacteria;k__norank;p__Proteobacteria;c__Gammaproteobacteria;o__Enterobacterales;f__Enterobacteriaceae;g__Enterobacter;s__Enterobacter_hormaechei
PB1_gene_858	186	WP_096927671.1	179	141/179(78.77)	7.40e-105	citrate lyase holo-[acyl-carrier protein] synthase [Enterobacter cloacae]	550	d__Bacteria;k__norank;p__Proteobacteria;c__Gammaproteobacteria;o__Enterobacterales;f__Enterobacteriaceae;g__Enterobacter;s__Enterobacter_cloacae
PB1_gene_860	333	ORM86136.1	332	332/332(100.00)	2.83e-229	anthranilate phosphoribosyltransferase [Pantoea deleyi]	470932	d__Bacteria;k__norank;p__Proteobacteria;c__Gammaproteobacteria;o__Enterobacterales;f__Erwiniaceae;g__Pantoea;s__Pantoea_deleyi

PB1_gene_861	193	OXH80311.1	193	191/193(98.96)	8.99e-136	glutamine amidotransferase [Pantoea agglomerans]	549	d__Bacteria;k__norank;p__Proteobacteria;c__Gammaproteobacteria;o__Enterobacterales;f__Erwiniaceae;g__Pantoea;s__Pantoea_agglomerans
PB1_gene_862	521	WP_111140063.1	521	516/521(99.04)	0	anthranilate synthase component 1 [Pantoea sp. ARC607]	2027922	d__Bacteria;k__norank;p__Proteobacteria;c__Gammaproteobacteria;o__Enterobacterales;f__Erwiniaceae;g__Pantoea;s__Pantoea_sp._ARC607
PB1_gene_864	651	WP_111139348.1	666	645/651(99.08)	0	transketolase [Pantoea sp. ARC607]	2027922	d__Bacteria;k__norank;p__Proteobacteria;c__Gammaproteobacteria;o__Enterobacterales;f__Erwiniaceae;g__Pantoea;s__Pantoea_sp._ARC607
PB1_gene_865	316	ORM76912.1	316	314/316(99.37)	5.06e-226	transaldolase [Pantoea deleyi]	470932	d__Bacteria;k__norank;p__Proteobacteria;c__Gammaproteobacteria;o__Enterobacterales;f__Erwiniaceae;g__Pantoea;s__Pantoea_deleyi
PB1_gene_868	330	WP_111139758.1	330	327/330(99.09)	2.79e-246	lysophospholipase L2 [Pantoea sp. ARC607]	2027922	d__Bacteria;k__norank;p__Proteobacteria;c__Gammaproteobacteria;o__Enterobacterales;f__Erwiniaceae;g__Pantoea;s__Pantoea_sp._ARC607
PB1_gene_869	207	WP_111139759.1	207	207/207(100.00)	2.73e-137	homoserine/homoserine lactone efflux protein [Pantoea sp.	2027922	d__Bacteria;k__norank;p__Proteobacteria;c__Gammaproteobacteria;o__Enterobacterales;f__Erwiniaceae;g__Pantoea;s__Pantoea_sp._ARC607
PB1_gene_870	220	WP_108452542.1	223	148/221(66.97)	2.14e-95	HAD family hydrolase [Klebsiella pneumoniae]	573	d__Bacteria;k__norank;p__Proteobacteria;c__Gammaproteobacteria;o__Enterobacterales;f__Enterobacteriaceae;g__Klebsiella;s__Klebsiella_pneumoniae
PB1_gene_871	210	WP_108452541.1	210	130/210(61.90)	1.97e-90	hypothetical protein [Klebsiella pneumoniae]	573	d__Bacteria;k__norank;p__Proteobacteria;c__Gammaproteobacteria;o__Enterobacterales;f__Enterobacteriaceae;g__Klebsiella;s__Klebsiella_pneumoniae
PB1_gene_876	686	WP_111139648.1	691	634/685(92.55)	0	4-alpha-glucanotransferase [Pantoea sp. ARC607]	2027922	d__Bacteria;k__norank;p__Proteobacteria;c__Gammaproteobacteria;o__Enterobacterales;f__Erwiniaceae;g__Pantoea;s__Pantoea_sp._ARC607
PB1_gene_879	130	WP_111139835.1	130	128/130(98.46)	2.31e-78	hypothetical protein [Pantoea sp. ARC607]	2027922	d__Bacteria;k__norank;p__Proteobacteria;c__Gammaproteobacteria;o__Enterobacterales;f__Erwiniaceae;g__Pantoea;s__Pantoea_sp._ARC607
PB1_gene_880	453	WP_111139836.1	453	449/453(99.12)	1.81e-302	ATP-dependent RNA helicase RhlE [Pantoea sp. ARC607]	2027922	d__Bacteria;k__norank;p__Proteobacteria;c__Gammaproteobacteria;o__Enterobacterales;f__Erwiniaceae;g__Pantoea;s__Pantoea_sp._ARC607
PB1_gene_881	316	WP_111139837.1	316	310/316(98.10)	5.06e-226	tRNA dihydrouridine(16) synthase DusC [Pantoea sp.	2027922	d__Bacteria;k__norank;p__Proteobacteria;c__Gammaproteobacteria;o__Enterobacterales;f__Erwiniaceae;g__Pantoea;s__Pantoea_sp._ARC607
PB1_gene_882	186	EOB12939.1	165	56/132(42.42)	3.64e-16	hypothetical protein NBO_303g0001 [Nosema	578461	d__Eukaryota;k__Fungi;p__Microsporidia;c__norank;o__norank;f__Nosematidae;g__Nosema;s__Nosema_bombycis
PB1_gene_884	437	WP_111138258.1	437	432/436(99.08)	1.03e-293	citrate transporter [Pantoea sp. ARC607]	2027922	d__Bacteria;k__norank;p__Proteobacteria;c__Gammaproteobacteria;o__Enterobacterales;f__Erwiniaceae;g__Pantoea;s__Pantoea_sp._ARC607
PB1_gene_887	167	ELP25208.1	167	164/167(98.20)	1.54e-112	tRNA proofreading protein [Pantoea agglomerans 299R]	1261128	d__Bacteria;k__norank;p__Proteobacteria;c__Gammaproteobacteria;o__Enterobacterales;f__Erwiniaceae;g__Pantoea;s__Pantoea_agglomerans
PB1_gene_888	264	ORM76683.1	264	258/264(97.73)	8.17e-190	hydroxamate siderophore iron reductase FhuF [Pantoea deleyi]	470932	d__Bacteria;k__norank;p__Proteobacteria;c__Gammaproteobacteria;o__Enterobacterales;f__Erwiniaceae;g__Pantoea;s__Pantoea_deleyi
PB1_gene_889	365	ORM76682.1	365	360/365(98.63)	3.98e-250	diguanylate cyclase [Pantoea deleyi]	470932	d__Bacteria;k__norank;p__Proteobacteria;c__Gammaproteobacteria;o__Enterobacterales;f__Erwiniaceae;g__Pantoea;s__Pantoea_deleyi
PB1_gene_890	94	ORM76681.1	94	87/94(92.55)	5.97e-55	hypothetical protein HA42_18810 [Pantoea deleyi]	470932	d__Bacteria;k__norank;p__Proteobacteria;c__Gammaproteobacteria;o__Enterobacterales;f__Erwiniaceae;g__Pantoea;s__Pantoea_deleyi
PB1_gene_892	159	ORM81979.1	159	159/159(100.00)	1.34e-109	sensory transduction regulator [Pantoea deleyi]	470932	d__Bacteria;k__norank;p__Proteobacteria;c__Gammaproteobacteria;o__Enterobacterales;f__Erwiniaceae;g__Pantoea;s__Pantoea_deleyi
PB1_gene_893	372	ORM81996.1	369	368/369(99.73)	9.40e-270	spermidine/putrescine ABC transporter substrate-binding protein PotF [Pantoea deleyi]	470932	d__Bacteria;k__norank;p__Proteobacteria;c__Gammaproteobacteria;o__Enterobacterales;f__Erwiniaceae;g__Pantoea;s__Pantoea_deleyi
PB1_gene_894	377	ORM81978.1	377	375/377(99.47)	6.89e-273	putrescine transporter ATP-binding subunit [Pantoea deleyi]	470932	d__Bacteria;k__norank;p__Proteobacteria;c__Gammaproteobacteria;o__Enterobacterales;f__Erwiniaceae;g__Pantoea;s__Pantoea_deleyi
PB1_gene_897	495	ORM80700.1	495	485/495(97.98)	0	peptidase M32 [Pantoea deleyi]	470932	d__Bacteria;k__norank;p__Proteobacteria;c__Gammaproteobacteria;o__Enterobacterales;f__Erwiniaceae;g__Pantoea;s__Pantoea_deleyi
PB1_gene_898	433	ORM80699.1	433	427/433(98.61)	7.16e-308	two-component system sensor histidine kinase RstB [Pantoea deleyi]	470932	d__Bacteria;k__norank;p__Proteobacteria;c__Gammaproteobacteria;o__Enterobacterales;f__Erwiniaceae;g__Pantoea;s__Pantoea_deleyi
PB1_gene_899	241	ORM80698.1	241	241/241(100.00)	3.06e-169	two-component system response regulator RstA [Pantoea deleyi]	470932	d__Bacteria;k__norank;p__Proteobacteria;c__Gammaproteobacteria;o__Enterobacterales;f__Erwiniaceae;g__Pantoea;s__Pantoea_deleyi

PB1_gene_901	329	SKA25394.1	329	329/329(100.00)	7.83e-232	DNA-directed RNA polymerase subunit alpha [Pantoea	470933	d__Bacteria;k__norank;p__Proteobacteria;c__Gammaproteobacteria;o__Enterobacterales;f__Erwiniaceae;g__Pantoea;s__Pantoea_eucalypti
PB1_gene_902	206	SKA25385.1	206	206/206(100.00)	1.31e-139	SSU ribosomal protein S4P [Pantoea agglomerans]	470933	d__Bacteria;k__norank;p__Proteobacteria;c__Gammaproteobacteria;o__Enterobacterales;f__Erwiniaceae;g__Pantoea;s__Pantoea_eucalypti
PB1_gene_903	129	OON35511.1	129	129/129(100.00)	2.15e-87	30S ribosomal protein S11 [Izhakiella australiensis]	1926881	d__Bacteria;k__norank;p__Proteobacteria;c__Gammaproteobacteria;o__Enterobacterales;f__Erwiniaceae;g__Izhakiella;s__Izhakiella_australiensis
PB1_gene_904	118	SKA25368.1	118	117/117(100.00)	8.78e-75	SSU ribosomal protein S13P [Pantoea agglomerans]	470933	d__Bacteria;k__norank;p__Proteobacteria;c__Gammaproteobacteria;o__Enterobacterales;f__Erwiniaceae;g__Pantoea;s__Pantoea_eucalypti
PB1_gene_905	443	SKA25350.1	443	443/443(100.00)	9.86e-311	protein translocase subunit secY/sec61 alpha [Pantoea agglomerans]	470933	d__Bacteria;k__norank;p__Proteobacteria;c__Gammaproteobacteria;o__Enterobacterales;f__Erwiniaceae;g__Pantoea;s__Pantoea_eucalypti
PB1_gene_909	305	ORM84449.1	305	302/304(99.34)	4.33e-201	nitrogen assimilation transcriptional regulator [Pantoea	470932	d__Bacteria;k__norank;p__Proteobacteria;c__Gammaproteobacteria;o__Enterobacterales;f__Erwiniaceae;g__Pantoea;s__Pantoea_deleyi
PB1_gene_910	293	ORM86853.1	293	292/293(99.66)	3.18e-197	zinc metalloprotease HtpX [Pantoea deleyi]	470932	d__Bacteria;k__norank;p__Proteobacteria;c__Gammaproteobacteria;o__Enterobacterales;f__Erwiniaceae;g__Pantoea;s__Pantoea_deleyi
PB1_gene_911	679	ORM86852.1	679	675/679(99.41)	0	C-terminal processing peptidase [Pantoea deleyi]	470932	d__Bacteria;k__norank;p__Proteobacteria;c__Gammaproteobacteria;o__Enterobacterales;f__Erwiniaceae;g__Pantoea;s__Pantoea_deleyi
PB1_gene_912	233	PZL88796.1	233	221/233(94.85)	8.93e-133	RNA chaperone ProQ [Pantoea sp. ARC270]	2027923	d__Bacteria;k__norank;p__Proteobacteria;c__Gammaproteobacteria;o__Enterobacterales;f__Erwiniaceae;g__Pantoea;s__Pantoea_sp._ARC270
PB1_gene_915	294	WP_111141540.1	294	283/294(96.26)	1.34e-194	phosphonate ABC transporter, permease protein PhnE [Pantoea sp. ARC607]	2027922	d__Bacteria;k__norank;p__Proteobacteria;c__Gammaproteobacteria;o__Enterobacterales;f__Erwiniaceae;g__Pantoea;s__Pantoea_sp._ARC607
PB1_gene_916	867	WP_111141542.1	867	834/866(96.30)	0	response regulator [Pantoea sp. ARC607]	2027922	d__Bacteria;k__norank;p__Proteobacteria;c__Gammaproteobacteria;o__Enterobacterales;f__Erwiniaceae;g__Pantoea;s__Pantoea_sp._ARC607
PB1_gene_919	506	ORM85471.1	506	498/506(98.42)	0	xylose ABC transporter ATP-binding protein [Pantoea deleyi]	470932	d__Bacteria;k__norank;p__Proteobacteria;c__Gammaproteobacteria;o__Enterobacterales;f__Erwiniaceae;g__Pantoea;s__Pantoea_deleyi
PB1_gene_920	330	WP_111141404.1	330	327/330(99.09)	2.14e-225	D-xylose ABC transporter substrate-binding protein [Pantoea sp. ARC607]	2027922	d__Bacteria;k__norank;p__Proteobacteria;c__Gammaproteobacteria;o__Enterobacterales;f__Erwiniaceae;g__Pantoea;s__Pantoea_sp._ARC607
PB1_gene_921	331	SSK75150.1	291	146/263(55.51)	1.36e-94	Uncharacterised protein [Klebsiella pneumoniae]	573	d__Bacteria;k__norank;p__Proteobacteria;c__Gammaproteobacteria;o__Enterobacterales;f__Enterobacteriaceae;g__Klebsiella;s__Klebsiella_pneumoniae
PB1_gene_925	91	PQL29545.1	90	59/89(66.29)	9.00e-35	hypothetical protein C5L22_02520 [Pantoea ananatis]	553	d__Bacteria;k__norank;p__Proteobacteria;c__Gammaproteobacteria;o__Enterobacterales;f__Erwiniaceae;g__Pantoea;s__Pantoea_ananatis
PB1_gene_926	260	WP_111140688.1	260	239/258(92.64)	2.48e-173	alpha/beta fold hydrolase [Pantoea sp. ARC607]	2027922	d__Bacteria;k__norank;p__Proteobacteria;c__Gammaproteobacteria;o__Enterobacterales;f__Erwiniaceae;g__Pantoea;s__Pantoea_sp._ARC607
PB1_gene_927	193	WP_111140687.1	193	144/191(75.39)	8.90e-95	peptidylprolyl isomerase [Pantoea sp. ARC607]	2027922	d__Bacteria;k__norank;p__Proteobacteria;c__Gammaproteobacteria;o__Enterobacterales;f__Erwiniaceae;g__Pantoea;s__Pantoea_sp._ARC607
PB1_gene_928	108	WP_111140686.1	108	79/108(73.15)	2.50e-49	hypothetical protein [Pantoea sp. ARC607]	2027922	d__Bacteria;k__norank;p__Proteobacteria;c__Gammaproteobacteria;o__Enterobacterales;f__Erwiniaceae;g__Pantoea;s__Pantoea_sp._ARC607
PB1_gene_929	286	WP_111208618.1	278	224/275(81.45)	2.40e-166	MBL fold metallo-hydrolase [Pantoea sp. ARC270]	2630326	d__Bacteria;k__norank;p__Proteobacteria;c__Gammaproteobacteria;o__Enterobacterales;f__Erwiniaceae;g__Pantoea;s__norank
PB1_gene_932	241	ORM84118.1	241	232/241(96.27)	1.67e-157	oxidoreductase [Pantoea deleyi]	470932	d__Bacteria;k__norank;p__Proteobacteria;c__Gammaproteobacteria;o__Enterobacterales;f__Erwiniaceae;g__Pantoea;s__Pantoea_deleyi
PB1_gene_933	263	ORM84117.1	263	261/263(99.24)	3.97e-176	hypothetical protein HA42_04990 [Pantoea deleyi]	470932	d__Bacteria;k__norank;p__Proteobacteria;c__Gammaproteobacteria;o__Enterobacterales;f__Erwiniaceae;g__Pantoea;s__Pantoea_deleyi
PB1_gene_934	109	WP_111138907.1	109	106/109(97.25)	1.34e-76	hypothetical protein [Pantoea sp. ARC607]	2027922	d__Bacteria;k__norank;p__Proteobacteria;c__Gammaproteobacteria;o__Enterobacterales;f__Erwiniaceae;g__Pantoea;s__Pantoea_sp._ARC607
PB1_gene_935	263	ORM84115.1	230	226/230(98.26)	1.83e-155	aspartate/glutamate racemase [Pantoea deleyi]	470932	d__Bacteria;k__norank;p__Proteobacteria;c__Gammaproteobacteria;o__Enterobacterales;f__Erwiniaceae;g__Pantoea;s__Pantoea_deleyi
PB1_gene_936	106	WP_111138909.1	106	105/106(99.06)	3.92e-70	hypothetical protein [Pantoea sp. ARC607]	2027922	d__Bacteria;k__norank;p__Proteobacteria;c__Gammaproteobacteria;o__Enterobacterales;f__Erwiniaceae;g__Pantoea;s__Pantoea_sp._ARC607

PB1_gene_937	246	WP_111139462.1	246	239/246(97.15)	9.45e-171	lipopolysaccharide N-acetylmannosaminouronosyltransferase [Pantoea sp. ARC607]	2027922	d__Bacteria;k__norank;p__Proteobacteria;c__Gammaproteobacteria;o__Enterobacterales;f__Erwiniaceae;g__Pantoea;s__Pantoea_sp._ARC607
PB1_gene_938	444	ORM81447.1	447	438/444(98.65)	1.25e-312	enterobacterial common antigen polymerase [Pantoea deleyi]	470932	d__Bacteria;k__norank;p__Proteobacteria;c__Gammaproteobacteria;o__Enterobacterales;f__Erwiniaceae;g__Pantoea;s__Pantoea_deleyi
PB1_gene_939	355	ORM81448.1	355	343/355(96.62)	3.30e-253	4-alpha-L-fucosyltransferase [Pantoea deleyi]	470932	d__Bacteria;k__norank;p__Proteobacteria;c__Gammaproteobacteria;o__Enterobacterales;f__Erwiniaceae;g__Pantoea;s__Pantoea_deleyi
PB1_gene_941	1137	WP_083351058.1	1160	729/1143(63.78)	0	hypothetical protein [Lonsdalea quercina]	None	Unclassified
PB1_gene_943	372	ORM77532.1	372	370/372(99.46)	1.92e-256	ROK family protein [Pantoea deleyi]	470932	d__Bacteria;k__norank;p__Proteobacteria;c__Gammaproteobacteria;o__Enterobacterales;f__Erwiniaceae;g__Pantoea;s__Pantoea_deleyi
PB1_gene_944	294	WP_111141329.1	294	277/292(94.86)	6.79e-188	N-acetylglucosamine kinase [Pantoea sp. ARC607]	2027922	d__Bacteria;k__norank;p__Proteobacteria;c__Gammaproteobacteria;o__Enterobacterales;f__Erwiniaceae;g__Pantoea;s__Pantoea_sp._ARC607
PB1_gene_945	410	WP_111141328.1	385	379/385(98.44)	2.07e-280	Gfo/Idh/MocA family oxidoreductase [Pantoea sp. ARC607]	2027922	d__Bacteria;k__norank;p__Proteobacteria;c__Gammaproteobacteria;o__Enterobacterales;f__Erwiniaceae;g__Pantoea;s__Pantoea_sp._ARC607
PB1_gene_947	241	ORM83029.1	241	241/241(100.00)	7.20e-168	UMP kinase [Pantoea deleyi]	470932	d__Bacteria;k__norank;p__Proteobacteria;c__Gammaproteobacteria;o__Enterobacterales;f__Erwiniaceae;g__Pantoea;s__Pantoea_deleyi
PB1_gene_948	217	SUB08384.1	217	214/217(98.62)	8.08e-144	Ribosome-releasing factor [Pantoea agglomerans]	549	d__Bacteria;k__norank;p__Proteobacteria;c__Gammaproteobacteria;o__Enterobacterales;f__Erwiniaceae;g__Pantoea;s__Pantoea_agglomerans
PB1_gene_949	398	WP_111140583.1	398	395/398(99.25)	3.18e-275	1-deoxy-D-xylulose-5-phosphate reductoisomerase [Pantoea sp. ARC607]	2027922	d__Bacteria;k__norank;p__Proteobacteria;c__Gammaproteobacteria;o__Enterobacterales;f__Erwiniaceae;g__Pantoea;s__Pantoea_sp._ARC607
PB1_gene_950	250	ORM83031.1	250	250/250(100.00)	1.77e-177	(2E,6E)-farnesyl- diphosphate-specific ditrans,polycis-undecaprenyl-diphosphate synthase [Pantoea deleyi]	470932	d__Bacteria;k__norank;p__Proteobacteria;c__Gammaproteobacteria;o__Enterobacterales;f__Erwiniaceae;g__Pantoea;s__Pantoea_deleyi
PB1_gene_951	416	ORM82930.1	416	408/416(98.08)	1.57e-291	conjugal transfer protein TraF [Pantoea deleyi]	470932	d__Bacteria;k__norank;p__Proteobacteria;c__Gammaproteobacteria;o__Enterobacterales;f__Erwiniaceae;g__Pantoea;s__Pantoea_deleyi
PB1_gene_952	330	ORM82931.1	330	324/330(98.18)	6.04e-241	tRNA dihydrouridine(20/20a) synthase DusA [Pantoea deleyi]	470932	d__Bacteria;k__norank;p__Proteobacteria;c__Gammaproteobacteria;o__Enterobacterales;f__Erwiniaceae;g__Pantoea;s__Pantoea_deleyi
PB1_gene_954	274	WP_111139104.1	274	272/274(99.27)	1.93e-193	ammonia-dependent NAD(+) synthetase [Pantoea sp. ARC607]	2027922	d__Bacteria;k__norank;p__Proteobacteria;c__Gammaproteobacteria;o__Enterobacterales;f__Erwiniaceae;g__Pantoea;s__Pantoea_sp._ARC607
PB1_gene_955	296	ORM77076.1	296	283/296(95.61)	1.30e-202	aromatic amino acid transporter [Pantoea deleyi]	470932	d__Bacteria;k__norank;p__Proteobacteria;c__Gammaproteobacteria;o__Enterobacterales;f__Erwiniaceae;g__Pantoea;s__Pantoea_deleyi
PB1_gene_956	280	ORM77075.1	286	277/280(98.93)	6.49e-210	endonuclease [Pantoea deleyi]	470932	d__Bacteria;k__norank;p__Proteobacteria;c__Gammaproteobacteria;o__Enterobacterales;f__Erwiniaceae;g__Pantoea;s__Pantoea_deleyi
PB1_gene_957	172	ORM77074.1	164	151/165(91.52)	3.75e-91	ATP-independent periplasmic protein-refolding chaperone [Pantoea deleyi]	470932	d__Bacteria;k__norank;p__Proteobacteria;c__Gammaproteobacteria;o__Enterobacterales;f__Erwiniaceae;g__Pantoea;s__Pantoea_deleyi
PB1_gene_958	140	PZL97547.1	136	127/136(93.38)	8.62e-87	succinylglutamate desuccinylase, partial [Pantoea sp. ARC607]	2027922	d__Bacteria;k__norank;p__Proteobacteria;c__Gammaproteobacteria;o__Enterobacterales;f__Erwiniaceae;g__Pantoea;s__Pantoea_sp._ARC607
PB1_gene_960	357	ORM85220.1	357	353/357(98.88)	2.29e-245	replication protein RepA [Pantoea deleyi]	470932	d__Bacteria;k__norank;p__Proteobacteria;c__Gammaproteobacteria;o__Enterobacterales;f__Erwiniaceae;g__Pantoea;s__Pantoea_deleyi
PB1_gene_961	585	WP_111139447.1	586	553/585(94.53)	0	lipid A ABC transporter ATP-binding protein/permease MsbA [Pantoea sp. ARC607]	2027922	d__Bacteria;k__norank;p__Proteobacteria;c__Gammaproteobacteria;o__Enterobacterales;f__Erwiniaceae;g__Pantoea;s__Pantoea_sp._ARC607
PB1_gene_962	326	ORM78626.1	290	290/290(100.00)	2.33e-216	pectin acetylesterase [Pantoea deleyi]	470932	d__Bacteria;k__norank;p__Proteobacteria;c__Gammaproteobacteria;o__Enterobacterales;f__Erwiniaceae;g__Pantoea;s__Pantoea_deleyi
PB1_gene_965	60	WP_111141249.1	60	55/59(93.22)	3.08e-35	DUF1127 domain-containing protein [Pantoea sp. ARC607]	2027922	d__Bacteria;k__norank;p__Proteobacteria;c__Gammaproteobacteria;o__Enterobacterales;f__Erwiniaceae;g__Pantoea;s__Pantoea_sp._ARC607

PB1_gene_968	509	PZL87453.1	469	469/469(100.00)	0	glutamate--ammonia ligase [Pantoea sp. ARC270]	2027923	d__Bacteria;k__norank;p__Proteobacteria;c__Gammaproteobacteria;o__Enterobacterales;f__Erwiniaceae;g__Pantoea;s__Pantoea_sp._ARC270
PB1_gene_971	257	ORM83876.1	257	255/257(99.22)	1.51e-177	histidine/lysine/arginine/ornithine ABC transporter ATP-binding protein [Pantoea deleyi]	470932	d__Bacteria;k__norank;p__Proteobacteria;c__Gammaproteobacteria;o__Enterobacterales;f__Erwiniaceae;g__Pantoea;s__Pantoea_deleyi
PB1_gene_972	238	ORM83877.1	238	236/238(99.16)	5.04e-162	amino acid ABC transporter permease [Pantoea deleyi]	470932	d__Bacteria;k__norank;p__Proteobacteria;c__Gammaproteobacteria;o__Enterobacterales;f__Erwiniaceae;g__Pantoea;s__Pantoea_deleyi
PB1_gene_973	229	ORM83878.1	229	226/229(98.69)	6.13e-154	amino acid ABC transporter permease [Pantoea deleyi]	470932	d__Bacteria;k__norank;p__Proteobacteria;c__Gammaproteobacteria;o__Enterobacterales;f__Erwiniaceae;g__Pantoea;s__Pantoea_deleyi
PB1_gene_974	260	ORM83879.1	260	257/260(98.85)	3.43e-180	histidine ABC transporter substrate-binding protein HisJ	470932	d__Bacteria;k__norank;p__Proteobacteria;c__Gammaproteobacteria;o__Enterobacterales;f__Erwiniaceae;g__Pantoea;s__Pantoea_deleyi
PB1_gene_976	476	ORM80805.1	476	469/475(98.74)	0	6-phospho-beta-glucosidase [Pantoea deleyi]	470932	d__Bacteria;k__norank;p__Proteobacteria;c__Gammaproteobacteria;o__Enterobacterales;f__Erwiniaceae;g__Pantoea;s__Pantoea_deleyi
PB1_gene_977	482	ORM80806.1	482	460/481(95.63)	1.45e-309	PTS cellobiose/arbutin/salicin transporter subunit IIBC [Pantoea deleyi]	470932	d__Bacteria;k__norank;p__Proteobacteria;c__Gammaproteobacteria;o__Enterobacterales;f__Erwiniaceae;g__Pantoea;s__Pantoea_deleyi
PB1_gene_980	327	WP_090966667.1	327	318/327(97.25)	4.72e-232	transcriptional regulator RbsR [Pantoea sp. OV426]	1881036	d__Bacteria;k__norank;p__Proteobacteria;c__Gammaproteobacteria;o__Enterobacterales;f__Erwiniaceae;g__Pantoea;s__Pantoea_sp._OV426
PB1_gene_981	309	ORM85442.1	309	302/308(98.05)	5.28e-204	ribokinase [Pantoea deleyi]	470932	d__Bacteria;k__norank;p__Proteobacteria;c__Gammaproteobacteria;o__Enterobacterales;f__Erwiniaceae;g__Pantoea;s__Pantoea_deleyi
PB1_gene_982	291	WP_111140934.1	291	290/291(99.66)	9.10e-196	ribose ABC transporter substrate-binding protein RbsB [Pantoea sp. ARC607]	2027922	d__Bacteria;k__norank;p__Proteobacteria;c__Gammaproteobacteria;o__Enterobacterales;f__Erwiniaceae;g__Pantoea;s__Pantoea_sp._ARC607
PB1_gene_985	271	ORM77537.1	271	271/271(100.00)	5.88e-182	ABC transporter permease [Pantoea deleyi]	470932	d__Bacteria;k__norank;p__Proteobacteria;c__Gammaproteobacteria;o__Enterobacterales;f__Erwiniaceae;g__Pantoea;s__Pantoea_deleyi
PB1_gene_986	444	ORM77539.1	443	430/443(97.07)	0	hypothetical protein HA42_17665 [Pantoea deleyi]	470932	d__Bacteria;k__norank;p__Proteobacteria;c__Gammaproteobacteria;o__Enterobacterales;f__Erwiniaceae;g__Pantoea;s__Pantoea_deleyi
PB1_gene_987	356	ORM77538.1	356	351/356(98.60)	4.40e-263	tartrate dehydrogenase [Pantoea deleyi]	470932	d__Bacteria;k__norank;p__Proteobacteria;c__Gammaproteobacteria;o__Enterobacterales;f__Erwiniaceae;g__Pantoea;s__Pantoea_deleyi
PB1_gene_993	155	ORM79534.1	183	155/155(100.00)	1.91e-102	translation initiation factor IF-3 [Pantoea deleyi]	470932	d__Bacteria;k__norank;p__Proteobacteria;c__Gammaproteobacteria;o__Enterobacterales;f__Erwiniaceae;g__Pantoea;s__Pantoea_deleyi
PB1_gene_994	642	ORM79533.1	642	642/642(100.00)	0	threonine--tRNA ligase [Pantoea deleyi]	470932	d__Bacteria;k__norank;p__Proteobacteria;c__Gammaproteobacteria;o__Enterobacterales;f__Erwiniaceae;g__Pantoea;s__Pantoea_deleyi
PB1_gene_995	253	WP_111139093.1	253	253/253(100.00)	1.11e-177	DUF481 domain-containing protein [Pantoea sp. ARC607]	2027922	d__Bacteria;k__norank;p__Proteobacteria;c__Gammaproteobacteria;o__Enterobacterales;f__Erwiniaceae;g__Pantoea;s__Pantoea_sp._ARC607
PB1_gene_996	366	ORM81490.1	366	360/366(98.36)	4.77e-263	oxidoreductase [Pantoea deleyi]	470932	d__Bacteria;k__norank;p__Proteobacteria;c__Gammaproteobacteria;o__Enterobacterales;f__Erwiniaceae;g__Pantoea;s__Pantoea_deleyi
PB1_gene_997	172	WP_128085482.1	172	162/172(94.19)	2.32e-105	glutamate racemase [Pantoea deleyi]	470932	d__Bacteria;k__norank;p__Proteobacteria;c__Gammaproteobacteria;o__Enterobacterales;f__Erwiniaceae;g__Pantoea;s__Pantoea_deleyi
PB1_gene_998	133	ORM81488.1	133	125/132(94.70)	3.26e-93	prolyl endopeptidase [Pantoea deleyi]	470932	d__Bacteria;k__norank;p__Proteobacteria;c__Gammaproteobacteria;o__Enterobacterales;f__Erwiniaceae;g__Pantoea;s__Pantoea_deleyi
PB1_gene_999	343	ORM81487.1	343	339/343(98.83)	4.51e-254	NADP-dependent oxidoreductase [Pantoea deleyi]	470932	d__Bacteria;k__norank;p__Proteobacteria;c__Gammaproteobacteria;o__Enterobacterales;f__Erwiniaceae;g__Pantoea;s__Pantoea_deleyi
PB1_gene_1002	304	ORM84470.1	304	297/304(97.70)	2.36e-209	2-dehydropantoate 2-reductase [Pantoea deleyi]	470932	d__Bacteria;k__norank;p__Proteobacteria;c__Gammaproteobacteria;o__Enterobacterales;f__Erwiniaceae;g__Pantoea;s__Pantoea_deleyi
PB1_gene_1003	340	WP_111142098.1	340	336/340(98.82)	6.35e-248	LLM class flavin-dependent oxidoreductase [Pantoea sp. ARC607]	2027922	d__Bacteria;k__norank;p__Proteobacteria;c__Gammaproteobacteria;o__Enterobacterales;f__Erwiniaceae;g__Pantoea;s__Pantoea_sp._ARC607
PB1_gene_1006	71	SKA23660.1	71	71/71(100.00)	1.20e-48	large subunit ribosomal protein L31 [Pantoea agglomerans]	470933	d__Bacteria;k__norank;p__Proteobacteria;c__Gammaproteobacteria;o__Enterobacterales;f__Erwiniaceae;g__Pantoea;s__Pantoea_eucalypti

PB1_gene_1007	105	OQV38294.1	105	105/105(100.00)	6.29e-71	transcriptional repressor protein MetJ [Pantoea vagans]	470934	d__Bacteria;k__norank;p__Proteobacteria;c__Gammaproteobacteria;o__Enterobacterales;f__Erwiniaceae;g__Pantoea;s__Pantoea_vagans
PB1_gene_1008	386	WP_111140420.1	386	384/386(99.48)	8.40e-274	cystathionine gamma-synthase [Pantoea sp. ARC607]	2027922	d__Bacteria;k__norank;p__Proteobacteria;c__Gammaproteobacteria;o__Enterobacterales;f__Erwiniaceae;g__Pantoea;s__Pantoea_sp._ARC607
PB1_gene_1012	156	WP_111140711.1	157	152/156(97.44)	7.49e-108	tRNA (uridine(34)/cytosine(34)/5-carboxymethylaminomethyluridine(34)-2'-O)-	2027922	d__Bacteria;k__norank;p__Proteobacteria;c__Gammaproteobacteria;o__Enterobacterales;f__Erwiniaceae;g__Pantoea;s__Pantoea_sp._ARC607
PB1_gene_1013	459	ORM84522.1	459	459/459(100.00)	0	two-component system sensor histidine kinase CpxA [Pantoea deleyi]	470932	d__Bacteria;k__norank;p__Proteobacteria;c__Gammaproteobacteria;o__Enterobacterales;f__Erwiniaceae;g__Pantoea;s__Pantoea_deleyi
PB1_gene_1014	232	ORM84523.1	232	232/232(100.00)	5.49e-163	DNA-binding response regulator [Pantoea deleyi]	470932	d__Bacteria;k__norank;p__Proteobacteria;c__Gammaproteobacteria;o__Enterobacterales;f__Erwiniaceae;g__Pantoea;s__Pantoea_deleyi
PB1_gene_1015	169	WP_111140708.1	169	166/169(98.22)	2.95e-102	stress adaptor protein CpxP [Pantoea sp. ARC607]	2027922	d__Bacteria;k__norank;p__Proteobacteria;c__Gammaproteobacteria;o__Enterobacterales;f__Erwiniaceae;g__Pantoea;s__Pantoea_sp._ARC607
PB1_gene_1017	396	ORM79687.1	396	389/396(98.23)	1.79e-280	maltose ABC transporter substrate-binding protein MalE [Pantoea deleyi]	470932	d__Bacteria;k__norank;p__Proteobacteria;c__Gammaproteobacteria;o__Enterobacterales;f__Erwiniaceae;g__Pantoea;s__Pantoea_deleyi
PB1_gene_1018	369	OXH78388.1	369	365/369(98.92)	3.20e-258	ABC transporter ATP-binding protein [Pantoea agglomerans]	549	d__Bacteria;k__norank;p__Proteobacteria;c__Gammaproteobacteria;o__Enterobacterales;f__Erwiniaceae;g__Pantoea;s__Pantoea_agglomerans
PB1_gene_1022	405	WP_125289961.1	404	355/402(88.31)	7.83e-277	YgeY family selenium metabolism-linked hydrolase	2022746	d__Bacteria;k__norank;p__Proteobacteria;c__Gammaproteobacteria;o__Enterobacterales;f__Erwiniaceae;g__Erwinia;s__Erwinia_sp._198
PB1_gene_1023	401	EXU76828.1	403	302/397(76.07)	1.32e-223	diaminopropionate ammonia-lyase [Erwinia mallotivora]	69222	d__Bacteria;k__norank;p__Proteobacteria;c__Gammaproteobacteria;o__Enterobacterales;f__Erwiniaceae;g__Erwinia;s__Erwinia_mallotivora
PB1_gene_1026	324	ORM86819.1	324	322/324(99.38)	9.28e-242	lipid A biosynthesis (KDO)2-(lauroyl)-lipid IVA acyltransferase [Pantoea deleyi]	470932	d__Bacteria;k__norank;p__Proteobacteria;c__Gammaproteobacteria;o__Enterobacterales;f__Erwiniaceae;g__Pantoea;s__Pantoea_deleyi
PB1_gene_1027	422	ORM86818.1	443	418/422(99.05)	3.41e-301	murein DD-endopeptidase MepM [Pantoea deleyi]	470932	d__Bacteria;k__norank;p__Proteobacteria;c__Gammaproteobacteria;o__Enterobacterales;f__Erwiniaceae;g__Pantoea;s__Pantoea_deleyi
PB1_gene_1033	220	ORM82379.1	220	217/219(99.09)	1.25e-153	23S rRNA pseudouridylate synthase [Pantoea deleyi]	470932	d__Bacteria;k__norank;p__Proteobacteria;c__Gammaproteobacteria;o__Enterobacterales;f__Erwiniaceae;g__Pantoea;s__Pantoea_deleyi
PB1_gene_1034	416	SJZ35560.1	416	415/416(99.76)	4.44e-307	isocitrate dehydrogenase (NADP) [Pantoea agglomerans]	470933	d__Bacteria;k__norank;p__Proteobacteria;c__Gammaproteobacteria;o__Enterobacterales;f__Erwiniaceae;g__Pantoea;s__Pantoea_eucalypti
PB1_gene_1035	65	ORM82381.1	65	56/64(87.50)	2.84e-31	hypothetical protein HA42_08435 [Pantoea deleyi]	470932	d__Bacteria;k__norank;p__Proteobacteria;c__Gammaproteobacteria;o__Enterobacterales;f__Erwiniaceae;g__Pantoea;s__Pantoea_deleyi
PB1_gene_1037	431	ORM81138.1	431	431/431(100.00)	9.57e-208	Tol-Pal system beta propeller repeat protein TolB [Pantoea	470932	d__Bacteria;k__norank;p__Proteobacteria;c__Gammaproteobacteria;o__Enterobacterales;f__Erwiniaceae;g__Pantoea;s__Pantoea_deleyi
PB1_gene_1038	441	WP_098051192.1	441	422/440(95.91)	2.38e-102	cell envelope integrity protein TolA [Pantoea agglomerans]	470934	d__Bacteria;k__norank;p__Proteobacteria;c__Gammaproteobacteria;o__Enterobacterales;f__Erwiniaceae;g__Pantoea;s__Pantoea_vagans
PB1_gene_1039	143	ORM81140.1	142	142/142(100.00)	2.23e-91	protein TolR [Pantoea deleyi]	470932	d__Bacteria;k__norank;p__Proteobacteria;c__Gammaproteobacteria;o__Enterobacterales;f__Erwiniaceae;g__Pantoea;s__Pantoea_deleyi
PB1_gene_1042	144	ORM84898.1	144	144/144(100.00)	1.62e-96	ubiquinone-binding protein [Pantoea deleyi]	470932	d__Bacteria;k__norank;p__Proteobacteria;c__Gammaproteobacteria;o__Enterobacterales;f__Erwiniaceae;g__Pantoea;s__Pantoea_deleyi
PB1_gene_1043	97	ORM84899.1	97	94/96(97.92)	3.14e-56	RnfH family protein [Pantoea deleyi]	470932	d__Bacteria;k__norank;p__Proteobacteria;c__Gammaproteobacteria;o__Enterobacterales;f__Erwiniaceae;g__Pantoea;s__Pantoea_deleyi
PB1_gene_1044	115	ORM84900.1	115	115/115(100.00)	5.14e-77	outer membrane protein assembly factor BamE [Pantoea deleyi]	470932	d__Bacteria;k__norank;p__Proteobacteria;c__Gammaproteobacteria;o__Enterobacterales;f__Erwiniaceae;g__Pantoea;s__Pantoea_deleyi
PB1_gene_1045	553	ORM84901.1	553	550/553(99.46)	0	DNA repair protein RecN [Pantoea deleyi]	470932	d__Bacteria;k__norank;p__Proteobacteria;c__Gammaproteobacteria;o__Enterobacterales;f__Erwiniaceae;g__Pantoea;s__Pantoea_deleyi
PB1_gene_1048	225	ORM80436.1	225	222/225(98.67)	1.30e-162	6-phosphogluconate phosphatase [Pantoea deleyi]	470932	d__Bacteria;k__norank;p__Proteobacteria;c__Gammaproteobacteria;o__Enterobacterales;f__Erwiniaceae;g__Pantoea;s__Pantoea_deleyi

PB1_gene_1049	356	ORM80441.1	331	326/331(98.49)	1.72e-238	acetyltransferase [Pantoea deleyi]	470932	d__Bacteria;k__norank;p__Proteobacteria;c__Gammaproteobacteria;o__Enterobacterales;f__Erwiniaceae;g__Pantoea;s__Pantoea_deleyi
PB1_gene_1050	303	SKA09616.1	303	303/303(100.00)	9.49e-229	glycyl-tRNA synthetase alpha chain [Pantoea agglomerans]	470933	d__Bacteria;k__norank;p__Proteobacteria;c__Gammaproteobacteria;o__Enterobacterales;f__Erwiniaceae;g__Pantoea;s__Pantoea_eucalypti
PB1_gene_1055	283	ORM81308.1	283	274/283(96.82)	1.03e-197	hypothetical protein HA42_11195 [Pantoea deleyi]	470932	d__Bacteria;k__norank;p__Proteobacteria;c__Gammaproteobacteria;o__Enterobacterales;f__Erwiniaceae;g__Pantoea;s__Pantoea_deleyi
PB1_gene_1056	299	ORM81307.1	299	288/299(96.32)	2.12e-214	D-hexose-6-phosphate mutarotase [Pantoea deleyi]	470932	d__Bacteria;k__norank;p__Proteobacteria;c__Gammaproteobacteria;o__Enterobacterales;f__Erwiniaceae;g__Pantoea;s__Pantoea_deleyi
PB1_gene_1057	332	PAW36370.1	332	331/332(99.70)	9.22e-237	aldehyde dehydrogenase [Pantoea vagans]	470934	d__Bacteria;k__norank;p__Proteobacteria;c__Gammaproteobacteria;o__Enterobacterales;f__Erwiniaceae;g__Pantoea;s__Pantoea_vagans
PB1_gene_1058	136	ORM81305.1	136	133/136(97.79)	5.57e-100	peptide-methionine (R)-S-oxide reductase [Pantoea deleyi]	470932	d__Bacteria;k__norank;p__Proteobacteria;c__Gammaproteobacteria;o__Enterobacterales;f__Erwiniaceae;g__Pantoea;s__Pantoea_deleyi
PB1_gene_1061	238	WP_111138986.1	238	232/238(97.48)	6.95e-169	UDP-2,3-diacylglucosamine diphosphatase [Pantoea sp. ARC607]	2027922	d__Bacteria;k__norank;p__Proteobacteria;c__Gammaproteobacteria;o__Enterobacterales;f__Erwiniaceae;g__Pantoea;s__Pantoea_sp._ARC607
PB1_gene_1062	164	ORM85843.1	164	164/164(100.00)	9.37e-117	peptidylprolyl isomerase [Pantoea deleyi]	470932	d__Bacteria;k__norank;p__Proteobacteria;c__Gammaproteobacteria;o__Enterobacterales;f__Erwiniaceae;g__Pantoea;s__Pantoea_deleyi
PB1_gene_1063	461	WP_111138988.1	461	459/461(99.57)	0	cysteine--tRNA ligase [Pantoea sp. ARC607]	2027922	d__Bacteria;k__norank;p__Proteobacteria;c__Gammaproteobacteria;o__Enterobacterales;f__Erwiniaceae;g__Pantoea;s__Pantoea_sp._ARC607
PB1_gene_1064	70	ORM85841.1	70	70/70(100.00)	3.51e-43	ribosome-associated protein [Pantoea deleyi]	470932	d__Bacteria;k__norank;p__Proteobacteria;c__Gammaproteobacteria;o__Enterobacterales;f__Erwiniaceae;g__Pantoea;s__Pantoea_deleyi
PB1_gene_1067	520	ORM86079.1	514	506/513(98.64)	0	methyl-accepting chemotaxis protein [Pantoea deleyi]	470932	d__Bacteria;k__norank;p__Proteobacteria;c__Gammaproteobacteria;o__Enterobacterales;f__Erwiniaceae;g__Pantoea;s__Pantoea_deleyi
PB1_gene_1068	528	ORM86080.1	528	522/528(98.86)	0	PTS maltose transporter subunit IICB [Pantoea deleyi]	470932	d__Bacteria;k__norank;p__Proteobacteria;c__Gammaproteobacteria;o__Enterobacterales;f__Erwiniaceae;g__Pantoea;s__Pantoea_deleyi
PB1_gene_1069	900	AVV38818.1	900	836/900(92.89)	0	magnesium-translocating P-type ATPase [Pantoea vagans]	470934	d__Bacteria;k__norank;p__Proteobacteria;c__Gammaproteobacteria;o__Enterobacterales;f__Erwiniaceae;g__Pantoea;s__Pantoea_vagans
PB1_gene_1073	107	ORM81895.1	107	107/107(100.00)	8.20e-68	hypothetical protein HA42_09425 [Pantoea deleyi]	470932	d__Bacteria;k__norank;p__Proteobacteria;c__Gammaproteobacteria;o__Enterobacterales;f__Erwiniaceae;g__Pantoea;s__Pantoea_deleyi
PB1_gene_1074	98	ORM81896.1	98	94/98(95.92)	1.38e-55	hypothetical protein HA42_09430 [Pantoea deleyi]	470932	d__Bacteria;k__norank;p__Proteobacteria;c__Gammaproteobacteria;o__Enterobacterales;f__Erwiniaceae;g__Pantoea;s__Pantoea_deleyi
PB1_gene_1075	469	ORM81897.1	488	463/469(98.72)	0	EmrB/QacA family drug resistance transporter [Pantoea	470932	d__Bacteria;k__norank;p__Proteobacteria;c__Gammaproteobacteria;o__Enterobacterales;f__Erwiniaceae;g__Pantoea;s__Pantoea_deleyi
PB1_gene_1076	238	ORM81898.1	238	233/238(97.90)	4.08e-152	glycine/betaine ABC transporter permease [Pantoea deleyi]	470932	d__Bacteria;k__norank;p__Proteobacteria;c__Gammaproteobacteria;o__Enterobacterales;f__Erwiniaceae;g__Pantoea;s__Pantoea_deleyi
PB1_gene_1078	785	WP_111139385.1	786	775/785(98.73)	0	DNA polymerase II [Pantoea sp. ARC607]	2027922	d__Bacteria;k__norank;p__Proteobacteria;c__Gammaproteobacteria;o__Enterobacterales;f__Erwiniaceae;g__Pantoea;s__Pantoea_sp._ARC607
PB1_gene_1079	256	WP_111139386.1	254	253/254(99.61)	2.27e-187	DedA family protein [Pantoea sp. ARC607]	53335	d__Bacteria;k__norank;p__Proteobacteria;c__Gammaproteobacteria;o__Enterobacterales;f__Erwiniaceae;g__Pantoea;s__norank
PB1_gene_1082	208	ORM83040.1	208	205/208(98.56)	3.74e-140	ribonuclease HII [Pantoea deleyi]	470932	d__Bacteria;k__norank;p__Proteobacteria;c__Gammaproteobacteria;o__Enterobacterales;f__Erwiniaceae;g__Pantoea;s__Pantoea_deleyi
PB1_gene_1084	292	ORM81581.1	303	249/292(85.27)	8.25e-170	transcriptional regulator [Pantoea deleyi]	470932	d__Bacteria;k__norank;p__Proteobacteria;c__Gammaproteobacteria;o__Enterobacterales;f__Erwiniaceae;g__Pantoea;s__Pantoea_deleyi
PB1_gene_1085	163	ORM81582.1	163	147/162(90.74)	4.25e-99	hypothetical protein HA42_10190 [Pantoea deleyi]	470932	d__Bacteria;k__norank;p__Proteobacteria;c__Gammaproteobacteria;o__Enterobacterales;f__Erwiniaceae;g__Pantoea;s__Pantoea_deleyi
PB1_gene_1086	160	WP_111138273.1	160	125/157(79.62)	5.93e-84	hypothetical protein [Pantoea sp. ARC607]	2027922	d__Bacteria;k__norank;p__Proteobacteria;c__Gammaproteobacteria;o__Enterobacterales;f__Erwiniaceae;g__Pantoea;s__Pantoea_sp._ARC607
PB1_gene_1087	298	ORM81584.1	299	276/300(92.00)	1.14e-195	hypothetical protein HA42_10200 [Pantoea deleyi]	470932	d__Bacteria;k__norank;p__Proteobacteria;c__Gammaproteobacteria;o__Enterobacterales;f__Erwiniaceae;g__Pantoea;s__Pantoea_deleyi
PB1_gene_1090	99	EIB96402.1	112	49/109(44.95)	1.97e-16	hypothetical protein S7A_20099 [Pantoea sp. Sc1]	593105	d__Bacteria;k__norank;p__Proteobacteria;c__Gammaproteobacteria;o__Enterobacterales;f__Erwiniaceae;g__Pantoea;s__Pantoea_sp._Sc1

PB1_gene_1091	302	ORM80637.1	322	299/302(99.01)	3.33e-206	virulence factor VirK [Pantoea deleyi]	470932	d__Bacteria;k__norank;p__Proteobacteria;c__Gammaproteobacteria;o__Enterobacterales;f__Erwiniaceae;g__Pantoea;s__Pantoea_deleyi
PB1_gene_1092	120	ORM80636.1	120	119/120(99.17)	1.90e-70	hypothetical protein HA42_13745 [Pantoea deleyi]	470932	d__Bacteria;k__norank;p__Proteobacteria;c__Gammaproteobacteria;o__Enterobacterales;f__Erwiniaceae;g__Pantoea;s__Pantoea_deleyi
PB1_gene_1093	200	ERM09364.1	198	191/198(96.46)	5.36e-131	mannitol repressor protein [Pantoea agglomerans Tx10]	1332072	d__Bacteria;k__norank;p__Proteobacteria;c__Gammaproteobacteria;o__Enterobacterales;f__Erwiniaceae;g__Pantoea;s__Pantoea_agglomerans
PB1_gene_1094	382	ORM80634.1	382	378/382(98.95)	6.31e-267	mannitol-1-phosphate 5-dehydrogenase [Pantoea deleyi]	470932	d__Bacteria;k__norank;p__Proteobacteria;c__Gammaproteobacteria;o__Enterobacterales;f__Erwiniaceae;g__Pantoea;s__Pantoea_deleyi
PB1_gene_1097	83	ORM78521.1	83	80/82(97.56)	3.44e-49	hypothetical protein HA42_16580 [Pantoea deleyi]	470932	d__Bacteria;k__norank;p__Proteobacteria;c__Gammaproteobacteria;o__Enterobacterales;f__Erwiniaceae;g__Pantoea;s__Pantoea_deleyi
PB1_gene_1098	105	WP_111140898.1	105	96/105(91.43)	3.51e-59	hypothetical protein [Pantoea sp. ARC607]	2027922	d__Bacteria;k__norank;p__Proteobacteria;c__Gammaproteobacteria;o__Enterobacterales;f__Erwiniaceae;g__Pantoea;s__Pantoea_sp._ARC607
PB1_gene_1099	100	ORM78520.1	100	95/100(95.00)	1.59e-64	hypothetical protein HA42_16570 [Pantoea deleyi]	470932	d__Bacteria;k__norank;p__Proteobacteria;c__Gammaproteobacteria;o__Enterobacterales;f__Erwiniaceae;g__Pantoea;s__Pantoea_deleyi
PB1_gene_1102	58	SKA22706.1	58	58/58(100.00)	1.54e-14	hypothetical protein SAMN03097723_3694 [Pantoea agglomerans]	470933	d__Bacteria;k__norank;p__Proteobacteria;c__Gammaproteobacteria;o__Enterobacterales;f__Erwiniaceae;g__Pantoea;s__Pantoea_eucalypti
PB1_gene_1103	115	PAW31292.1	115	115/115(100.00)	3.47e-84	HNH endonuclease [Pantoea vagans]	470934	d__Bacteria;k__norank;p__Proteobacteria;c__Gammaproteobacteria;o__Enterobacterales;f__Erwiniaceae;g__Pantoea;s__Pantoea_vagans
PB1_gene_1104	112	ORM76315.1	112	109/112(97.32)	6.07e-79	cupin [Pantoea deleyi]	470932	d__Bacteria;k__norank;p__Proteobacteria;c__Gammaproteobacteria;o__Enterobacterales;f__Erwiniaceae;g__Pantoea;s__Pantoea_deleyi
PB1_gene_1107	95	CAX61230.1	95	93/95(97.89)	3.30e-57	IS1400 transposase A [Erwinia billingiae Eb661]	634500	d__Bacteria;k__norank;p__Proteobacteria;c__Gammaproteobacteria;o__Enterobacterales;f__Erwiniaceae;g__Erwinia;s__Erwinia_billingiae
PB1_gene_1110	247	ORM75660.1	247	240/247(97.17)	4.03e-177	Nif3-like dinuclear metal center protein [Pantoea deleyi]	470932	d__Bacteria;k__norank;p__Proteobacteria;c__Gammaproteobacteria;o__Enterobacterales;f__Erwiniaceae;g__Pantoea;s__Pantoea_deleyi
PB1_gene_1111	474	ORM75659.1	474	459/474(96.84)	0	deoxyribodipyrimidine photo-lyase [Pantoea deleyi]	470932	d__Bacteria;k__norank;p__Proteobacteria;c__Gammaproteobacteria;o__Enterobacterales;f__Erwiniaceae;g__Pantoea;s__Pantoea_deleyi
PB1_gene_1112	318	ORM75658.1	318	316/318(99.37)	3.92e-240	hypothetical protein HA42_20155 [Pantoea deleyi]	470932	d__Bacteria;k__norank;p__Proteobacteria;c__Gammaproteobacteria;o__Enterobacterales;f__Erwiniaceae;g__Pantoea;s__Pantoea_deleyi
PB1_gene_1114	215	ORM86124.1	215	213/215(99.07)	7.76e-141	hypothetical protein HA42_01425 [Pantoea deleyi]	470932	d__Bacteria;k__norank;p__Proteobacteria;c__Gammaproteobacteria;o__Enterobacterales;f__Erwiniaceae;g__Pantoea;s__Pantoea_deleyi
PB1_gene_1117	50	OQV43811.1	50	48/50(96.00)	6.30e-24	DUF2496 domain-containing protein [Pantoea vagans]	470934	d__Bacteria;k__norank;p__Proteobacteria;c__Gammaproteobacteria;o__Enterobacterales;f__Erwiniaceae;g__Pantoea;s__Pantoea_vagans
PB1_gene_1118	81	ORM77997.1	81	81/81(100.00)	1.13e-44	hypothetical protein HA42_17135 [Pantoea deleyi]	470932	d__Bacteria;k__norank;p__Proteobacteria;c__Gammaproteobacteria;o__Enterobacterales;f__Erwiniaceae;g__Pantoea;s__Pantoea_deleyi
PB1_gene_1119	176	ORM77998.1	176	171/176(97.16)	1.89e-106	PriC [Pantoea deleyi]	470932	d__Bacteria;k__norank;p__Proteobacteria;c__Gammaproteobacteria;o__Enterobacterales;f__Erwiniaceae;g__Pantoea;s__Pantoea_deleyi
PB1_gene_1120	129	WP_111140325.1	129	128/129(99.22)	5.70e-83	DUF454 family protein [Pantoea sp. ARC607]	2027922	d__Bacteria;k__norank;p__Proteobacteria;c__Gammaproteobacteria;o__Enterobacterales;f__Erwiniaceae;g__Pantoea;s__Pantoea_sp._ARC607
PB1_gene_1121	197	ELP25679.1	197	187/197(94.92)	6.24e-129	Adenine phosphoribosyltransferase	1261128	d__Bacteria;k__norank;p__Proteobacteria;c__Gammaproteobacteria;o__Enterobacterales;f__Erwiniaceae;g__Pantoea;s__Pantoea_agglomerans
PB1_gene_1123	1183	WP_101241016.1	2357	683/1236(55.26)	0	autotransporter outer membrane beta-barrel domain-containing protein [Enterobacterales bacterium CwR94]	None	Unclassified
PB1_gene_1126	250	ORM80839.1	250	249/250(99.60)	1.52e-178	UMP phosphatase [Pantoea deleyi]	470932	d__Bacteria;k__norank;p__Proteobacteria;c__Gammaproteobacteria;o__Enterobacterales;f__Erwiniaceae;g__Pantoea;s__Pantoea_deleyi
PB1_gene_1127	406	WP_111141043.1	406	404/406(99.51)	1.89e-287	ROK family transcriptional regulator [Pantoea sp. ARC607]	2027922	d__Bacteria;k__norank;p__Proteobacteria;c__Gammaproteobacteria;o__Enterobacterales;f__Erwiniaceae;g__Pantoea;s__Pantoea_sp._ARC607
PB1_gene_1130	104	WP_111138699.1	109	98/104(94.23)	5.23e-66	hypothetical protein [Pantoea sp. ARC607]	2027922	d__Bacteria;k__norank;p__Proteobacteria;c__Gammaproteobacteria;o__Enterobacterales;f__Erwiniaceae;g__Pantoea;s__Pantoea_sp._ARC607

PB1_gene_1131	60	ORM82387.1	59	59/59(100.00)	4.65e-30	hypothetical protein HA42_08480 [Pantoea deleyi]	470932	d__Bacteria;k__norank;p__Proteobacteria;c__Gammaproteobacteria;o__Enterobacterales;f__Erwiniaceae;g__Pantoea;s__Pantoea_deleyi
PB1_gene_1132	127	ORM82388.1	127	120/127(94.49)	7.84e-80	hypothetical protein HA42_08485 [Pantoea deleyi]	470932	d__Bacteria;k__norank;p__Proteobacteria;c__Gammaproteobacteria;o__Enterobacterales;f__Erwiniaceae;g__Pantoea;s__Pantoea_deleyi
PB1_gene_1133	137	WP_111138702.1	137	125/137(91.24)	5.33e-85	GNAT family N-acetyltransferase [Pantoea sp. ARC607]	2027922	d__Bacteria;k__norank;p__Proteobacteria;c__Gammaproteobacteria;o__Enterobacterales;f__Erwiniaceae;g__Pantoea;s__Pantoea_sp._ARC607
PB1_gene_1137	72	SKA16054.1	72	70/72(97.22)	4.05e-43	hypothetical protein SAMN03097723_3391 [Pantoea agglomerans]	470933	d__Bacteria;k__norank;p__Proteobacteria;c__Gammaproteobacteria;o__Enterobacterales;f__Erwiniaceae;g__Pantoea;s__Pantoea_eucalypti
PB1_gene_1138	349	WP_111139708.1	338	328/338(97.04)	1.86e-251	hydrolase [Pantoea sp. ARC607]	2027922	d__Bacteria;k__norank;p__Proteobacteria;c__Gammaproteobacteria;o__Enterobacterales;f__Erwiniaceae;g__Pantoea;s__Pantoea_sp._ARC607
PB1_gene_1139	192	ORM81826.1	200	189/192(98.44)	8.05e-129	alcohol dehydrogenase [Pantoea deleyi]	470932	d__Bacteria;k__norank;p__Proteobacteria;c__Gammaproteobacteria;o__Enterobacterales;f__Erwiniaceae;g__Pantoea;s__Pantoea_deleyi
PB1_gene_1140	326	ORM81825.1	326	325/326(99.69)	1.25e-231	cytochrome-c peroxidase [Pantoea deleyi]	470932	d__Bacteria;k__norank;p__Proteobacteria;c__Gammaproteobacteria;o__Enterobacterales;f__Erwiniaceae;g__Pantoea;s__Pantoea_deleyi
PB1_gene_1141	89	ADO11043.1	71	68/71(95.77)	1.38e-43	Nitrite reductase [NAD(P)H] [Pantoea vagans C9-1]	712898	d__Bacteria;k__norank;p__Proteobacteria;c__Gammaproteobacteria;o__Enterobacterales;f__Erwiniaceae;g__Pantoea;s__Pantoea_vagans
PB1_gene_1142	157	ORM81239.1	157	154/157(98.09)	1.58e-107	bacterioferritin [Pantoea deleyi]	470932	d__Bacteria;k__norank;p__Proteobacteria;c__Gammaproteobacteria;o__Enterobacterales;f__Erwiniaceae;g__Pantoea;s__Pantoea_deleyi
PB1_gene_1143	304	ORM81240.1	257	223/257(86.77)	2.31e-159	peptidase A24 [Pantoea deleyi]	470932	d__Bacteria;k__norank;p__Proteobacteria;c__Gammaproteobacteria;o__Enterobacterales;f__Erwiniaceae;g__Pantoea;s__Pantoea_deleyi
PB1_gene_1144	103	APT79325.1	103	103/103(100.00)	3.41e-65	SSU ribosomal protein S10p (S20e) [Salmonella enterica subsp. enterica serovar Cerro]	340188	d__Bacteria;k__norank;p__Proteobacteria;c__Gammaproteobacteria;o__Enterobacterales;f__Enterobacteriaceae;g__Salmonella;s__Salmonella_enterica
PB1_gene_1145	209	SKA25149.1	209	209/209(100.00)	1.78e-143	LSU ribosomal protein L3P [Pantoea agglomerans]	470933	d__Bacteria;k__norank;p__Proteobacteria;c__Gammaproteobacteria;o__Enterobacterales;f__Erwiniaceae;g__Pantoea;s__Pantoea_eucalypti
PB1_gene_1146	201	ORM81242.1	201	201/201(100.00)	3.08e-131	50S ribosomal protein L4 [Pantoea deleyi]	470932	d__Bacteria;k__norank;p__Proteobacteria;c__Gammaproteobacteria;o__Enterobacterales;f__Erwiniaceae;g__Pantoea;s__Pantoea_deleyi
PB1_gene_1148	286	ORM74869.1	286	284/286(99.30)	3.63e-204	LysR family transcriptional regulator [Pantoea deleyi]	470932	d__Bacteria;k__norank;p__Proteobacteria;c__Gammaproteobacteria;o__Enterobacterales;f__Erwiniaceae;g__Pantoea;s__Pantoea_deleyi
PB1_gene_1149	462	WP_111140908.1	463	450/463(97.19)	0	twin-arginine translocation signal domain-containing protein [Pantoea sp. ARC607]	2027922	d__Bacteria;k__norank;p__Proteobacteria;c__Gammaproteobacteria;o__Enterobacterales;f__Erwiniaceae;g__Pantoea;s__Pantoea_sp._ARC607
PB1_gene_1152	94	EIB98172.1	94	94/94(100.00)	4.70e-61	50S ribosomal protein L25 [Pantoea sp. Sc1]	593105	d__Bacteria;k__norank;p__Proteobacteria;c__Gammaproteobacteria;o__Enterobacterales;f__Erwiniaceae;g__Pantoea;s__Pantoea_sp._Sc1
PB1_gene_1153	616	ERM08910.1	616	596/616(96.75)	0	hypothetical protein L584_02085 [Pantoea agglomerans Tx10]	1332072	d__Bacteria;k__norank;p__Proteobacteria;c__Gammaproteobacteria;o__Enterobacterales;f__Erwiniaceae;g__Pantoea;s__Pantoea_agglomerans
PB1_gene_1154	239	WP_111138737.1	239	237/239(99.16)	1.11e-170	16S rRNA pseudouridine(516) synthase RsuA [Pantoea sp. ARC607]	2027922	d__Bacteria;k__norank;p__Proteobacteria;c__Gammaproteobacteria;o__Enterobacterales;f__Erwiniaceae;g__Pantoea;s__Pantoea_sp._ARC607
PB1_gene_1158	426	WP_111139212.1	426	424/426(99.53)	1.27e-302	GTPase HflX [Pantoea sp. ARC607]	2027922	d__Bacteria;k__norank;p__Proteobacteria;c__Gammaproteobacteria;o__Enterobacterales;f__Erwiniaceae;g__Pantoea;s__Pantoea_sp._ARC607
PB1_gene_1159	103	ORM79175.1	103	103/103(100.00)	2.22e-52	RNA chaperone Hfq [Pantoea deleyi]	470932	d__Bacteria;k__norank;p__Proteobacteria;c__Gammaproteobacteria;o__Enterobacterales;f__Erwiniaceae;g__Pantoea;s__Pantoea_deleyi
PB1_gene_1162	293	ORM80982.1	288	282/288(97.92)	5.08e-220	nucleoside-specific channel-forming protein Tsx [Pantoea	470932	d__Bacteria;k__norank;p__Proteobacteria;c__Gammaproteobacteria;o__Enterobacterales;f__Erwiniaceae;g__Pantoea;s__Pantoea_deleyi
PB1_gene_1163	144	ORM80976.1	145	140/142(98.59)	1.10e-101	hypothetical protein HA42_12320 [Pantoea deleyi]	470932	d__Bacteria;k__norank;p__Proteobacteria;c__Gammaproteobacteria;o__Enterobacterales;f__Erwiniaceae;g__Pantoea;s__Pantoea_deleyi
PB1_gene_1164	307	ORM80977.1	298	291/298(97.65)	1.61e-204	LysR family transcriptional regulator [Pantoea deleyi]	470932	d__Bacteria;k__norank;p__Proteobacteria;c__Gammaproteobacteria;o__Enterobacterales;f__Erwiniaceae;g__Pantoea;s__Pantoea_deleyi

PB1_gene_1167	463	ORM79524.1	463	461/463(99.57)	3.03e-306	L-cystine transporter tcyP [Pantoea deleyi]	470932	d__Bacteria;k__norank;p__Proteobacteria;c__Gammaproteobacteria;o__Enterobacterales;f__Erwiniaceae;g__Pantoea;s__Pantoea_deleyi
PB1_gene_1168	122	PAW36762.1	129	111/129(86.05)	4.26e-74	hypothetical protein CIL06_14050 [Pantoea vagans]	470934	d__Bacteria;k__norank;p__Proteobacteria;c__Gammaproteobacteria;o__Enterobacterales;f__Erwiniaceae;g__Pantoea;s__Pantoea_vagans
PB1_gene_1169	116	ORM77078.1	111	111/111(100.00)	3.19e-75	transcriptional regulator [Pantoea deleyi]	470932	d__Bacteria;k__norank;p__Proteobacteria;c__Gammaproteobacteria;o__Enterobacterales;f__Erwiniaceae;g__Pantoea;s__Pantoea_deleyi
PB1_gene_1171	572	WP_111140600.1	572	564/572(98.60)	0	proline--tRNA ligase [Pantoea sp. ARC607]	2027922	d__Bacteria;k__norank;p__Proteobacteria;c__Gammaproteobacteria;o__Enterobacterales;f__Erwiniaceae;g__Pantoea;s__Pantoea_sp._ARC607
PB1_gene_1172	234	ORM83048.1	234	230/234(98.29)	5.49e-164	tRNA (N6-threonylcarbamoyladenosine(37)-N6)-methyltransferase TrmO [Pantoea deleyi]	470932	d__Bacteria;k__norank;p__Proteobacteria;c__Gammaproteobacteria;o__Enterobacterales;f__Erwiniaceae;g__Pantoea;s__Pantoea_deleyi
PB1_gene_1173	132	ORM83049.1	132	122/132(92.42)	1.14e-79	Rcs stress response system protein RcsF [Pantoea deleyi]	470932	d__Bacteria;k__norank;p__Proteobacteria;c__Gammaproteobacteria;o__Enterobacterales;f__Erwiniaceae;g__Pantoea;s__Pantoea_deleyi
PB1_gene_1176	138	WP_123346412.1	138	87/138(63.04)	5.44e-51	MULTISPECIES: hypothetical protein [Enterobacter]	2608935	d__Bacteria;k__norank;p__Proteobacteria;c__Gammaproteobacteria;o__Enterobacterales;f__Enterobacteriaceae;g__Enterobacter;s__norank
PB1_gene_1177	872	WP_123346410.1	870	464/873(53.15)	1.16e-312	MULTISPECIES: hypothetical protein [Enterobacter]	2608935	d__Bacteria;k__norank;p__Proteobacteria;c__Gammaproteobacteria;o__Enterobacterales;f__Enterobacteriaceae;g__Enterobacter;s__norank
PB1_gene_1180	763	WP_111139441.1	763	748/763(98.03)	0	cellulose biosynthesis cyclic di-GMP-binding regulatory protein BcsB [Pantoea sp. ARC607]	2027922	d__Bacteria;k__norank;p__Proteobacteria;c__Gammaproteobacteria;o__Enterobacterales;f__Erwiniaceae;g__Pantoea;s__Pantoea_sp._ARC607
PB1_gene_1182	730	ORM85248.1	708	695/708(98.16)	0	diguanylate cyclase [Pantoea deleyi]	470932	d__Bacteria;k__norank;p__Proteobacteria;c__Gammaproteobacteria;o__Enterobacterales;f__Erwiniaceae;g__Pantoea;s__Pantoea_deleyi
PB1_gene_1183	190	WP_111138355.1	188	175/189(92.59)	1.23e-122	N-acetyltransferase [Pantoea sp. ARC607]	2027922	d__Bacteria;k__norank;p__Proteobacteria;c__Gammaproteobacteria;o__Enterobacterales;f__Erwiniaceae;g__Pantoea;s__Pantoea_sp._ARC607
PB1_gene_1184	137	YP_003710356.1	131	60/110(54.55)	8.27e-27	hypothetical protein (plasmid) [Pantoea agglomerans]	None	Unclassified
PB1_gene_1186	234	ORM80940.1	234	234/234(100.00)	2.58e-153	50S ribosomal protein L1 [Pantoea deleyi]	470932	d__Bacteria;k__norank;p__Proteobacteria;c__Gammaproteobacteria;o__Enterobacterales;f__Erwiniaceae;g__Pantoea;s__Pantoea_deleyi
PB1_gene_1187	142	SKA25815.1	142	142/142(100.00)	4.50e-93	LSU ribosomal protein L11P [Pantoea agglomerans]	470933	d__Bacteria;k__norank;p__Proteobacteria;c__Gammaproteobacteria;o__Enterobacterales;f__Erwiniaceae;g__Pantoea;s__Pantoea_eucalypti
PB1_gene_1189	127	SKA25838.1	127	126/127(99.21)	3.45e-74	protein translocase subunit secE/sec61 gamma [Pantoea agglomerans]	470933	d__Bacteria;k__norank;p__Proteobacteria;c__Gammaproteobacteria;o__Enterobacterales;f__Erwiniaceae;g__Pantoea;s__Pantoea_eucalypti
PB1_gene_1192	455	WP_031590424.1	455	390/455(85.71)	6.67e-283	type VI secretion system protein TssL [Pantoea agglomerans]	549	d__Bacteria;k__norank;p__Proteobacteria;c__Gammaproteobacteria;o__Enterobacterales;f__Erwiniaceae;g__Pantoea;s__Pantoea_agglomerans
PB1_gene_1193	449	WP_031590425.1	448	418/449(93.10)	4.85e-307	type VI secretion system baseplate subunit TssK [Pantoea	549	d__Bacteria;k__norank;p__Proteobacteria;c__Gammaproteobacteria;o__Enterobacterales;f__Erwiniaceae;g__Pantoea;s__Pantoea_agglomerans
PB1_gene_1196	293	WP_111140274.1	292	280/291(96.22)	9.97e-203	Fe(3+)-hydroxamate ABC transporter substrate-binding protein FhuD [Pantoea sp.	2027922	d__Bacteria;k__norank;p__Proteobacteria;c__Gammaproteobacteria;o__Enterobacterales;f__Erwiniaceae;g__Pantoea;s__Pantoea_sp._ARC607
PB1_gene_1197	264	ORM76234.1	264	257/264(97.35)	8.77e-185	iron-hydroxamate transporter ATP-binding subunit [Pantoea	470932	d__Bacteria;k__norank;p__Proteobacteria;c__Gammaproteobacteria;o__Enterobacterales;f__Erwiniaceae;g__Pantoea;s__Pantoea_deleyi
PB1_gene_1200	110	ORM85864.1	110	110/110(100.00)	2.15e-69	YbaB/EbfC family nucleoid-associated protein [Pantoea	470932	d__Bacteria;k__norank;p__Proteobacteria;c__Gammaproteobacteria;o__Enterobacterales;f__Erwiniaceae;g__Pantoea;s__Pantoea_deleyi
PB1_gene_1201	201	WP_111140312.1	201	200/201(99.50)	6.53e-142	recombination protein RecR [Pantoea sp. ARC607]	2027922	d__Bacteria;k__norank;p__Proteobacteria;c__Gammaproteobacteria;o__Enterobacterales;f__Erwiniaceae;g__Pantoea;s__Pantoea_sp._ARC607
PB1_gene_1202	624	ORM85862.1	624	624/624(100.00)	0	molecular chaperone HtpG [Pantoea deleyi]	470932	d__Bacteria;k__norank;p__Proteobacteria;c__Gammaproteobacteria;o__Enterobacterales;f__Erwiniaceae;g__Pantoea;s__Pantoea_deleyi
PB1_gene_1204	523	ORM86030.1	523	510/523(97.51)	0	ABC transporter substrate-binding protein [Pantoea deleyi]	470932	d__Bacteria;k__norank;p__Proteobacteria;c__Gammaproteobacteria;o__Enterobacterales;f__Erwiniaceae;g__Pantoea;s__Pantoea_deleyi

PB1_gene_1205	500	WP_111138525.1	500	482/500(96.40)	0	DUF853 domain-containing protein [Pantoea sp. ARC607]	2027922	d__Bacteria;k__norank;p__Proteobacteria;c__Gammaproteobacteria;o__Enterobacterales;f__Erwiniaceae;g__Pantoea;s__Pantoea_sp._ARC607
PB1_gene_1207	263	ORM80718.1	263	253/261(96.93)	8.47e-187	N-formylglutamate deformylase [Pantoea deleyi]	470932	d__Bacteria;k__norank;p__Proteobacteria;c__Gammaproteobacteria;o__Enterobacterales;f__Erwiniaceae;g__Pantoea;s__Pantoea_deleyi
PB1_gene_1208	405	WP_111140573.1	405	394/405(97.28)	2.59e-289	imidazolonepropionase [Pantoea sp. ARC607]	2027922	d__Bacteria;k__norank;p__Proteobacteria;c__Gammaproteobacteria;o__Enterobacterales;f__Erwiniaceae;g__Pantoea;s__Pantoea_sp._ARC607
PB1_gene_1212	332	WP_096835027.1	332	315/332(94.88)	6.23e-235	MULTISPECIES: integrase [Klebsiella]	570	d__Bacteria;k__norank;p__Proteobacteria;c__Gammaproteobacteria;o__Enterobacterales;f__Enterobacteriaceae;g__Klebsiella;s__norank
PB1_gene_1214	148	WP_110332058.1	161	142/148(95.95)	1.03e-106	DUF2778 domain-containing protein [Pantoea sp. JKS000250]	1938795	d__Bacteria;k__norank;p__Proteobacteria;c__Gammaproteobacteria;o__Enterobacterales;f__Erwiniaceae;g__Pantoea;s__Pantoea_sp._JKS000250
PB1_gene_1215	75	WP_110332057.1	75	57/75(76.00)	1.10e-28	hypothetical protein [Pantoea sp. JKS000250]	1938795	d__Bacteria;k__norank;p__Proteobacteria;c__Gammaproteobacteria;o__Enterobacterales;f__Erwiniaceae;g__Pantoea;s__Pantoea_sp._JKS000250
PB1_gene_1218	386	ORM85490.1	377	358/376(95.21)	6.03e-258	ROK family protein [Pantoea deleyi]	470932	d__Bacteria;k__norank;p__Proteobacteria;c__Gammaproteobacteria;o__Enterobacterales;f__Erwiniaceae;g__Pantoea;s__Pantoea_deleyi
PB1_gene_1219	246	ORM85474.1	246	245/246(99.59)	4.68e-171	DNA replication protein DnaC [Pantoea deleyi]	470932	d__Bacteria;k__norank;p__Proteobacteria;c__Gammaproteobacteria;o__Enterobacterales;f__Erwiniaceae;g__Pantoea;s__Pantoea_deleyi
PB1_gene_1220	178	ORM85475.1	178	176/178(98.88)	2.25e-124	primosomal protein DnaI [Pantoea deleyi]	470932	d__Bacteria;k__norank;p__Proteobacteria;c__Gammaproteobacteria;o__Enterobacterales;f__Erwiniaceae;g__Pantoea;s__Pantoea_deleyi
PB1_gene_1221	225	ORM85476.1	226	216/226(95.58)	1.54e-143	hypothetical protein HA42_02590 [Pantoea deleyi]	470932	d__Bacteria;k__norank;p__Proteobacteria;c__Gammaproteobacteria;o__Enterobacterales;f__Erwiniaceae;g__Pantoea;s__Pantoea_deleyi
PB1_gene_1224	439	ORM79504.1	439	436/439(99.32)	2.03e-301	branched-chain amino acid transport system II carrier protein [Pantoea deleyi]	470932	d__Bacteria;k__norank;p__Proteobacteria;c__Gammaproteobacteria;o__Enterobacterales;f__Erwiniaceae;g__Pantoea;s__Pantoea_deleyi
PB1_gene_1225	368	ORM79503.1	368	357/368(97.01)	1.37e-263	5-methyltetrahydropteroyltriglutamate--homocysteine S-methyltransferase [Pantoea	470932	d__Bacteria;k__norank;p__Proteobacteria;c__Gammaproteobacteria;o__Enterobacterales;f__Erwiniaceae;g__Pantoea;s__Pantoea_deleyi
PB1_gene_1227	406	ORM81128.1	406	404/406(99.51)	6.19e-277	dihydrolipoamide succinyltransferase [Pantoea	470932	d__Bacteria;k__norank;p__Proteobacteria;c__Gammaproteobacteria;o__Enterobacterales;f__Erwiniaceae;g__Pantoea;s__Pantoea_deleyi
PB1_gene_1228	388	SJZ31344.1	388	388/388(100.00)	2.52e-276	succinyl-CoA synthetase (ADP-forming) beta subunit [Pantoea agglomerans]	470933	d__Bacteria;k__norank;p__Proteobacteria;c__Gammaproteobacteria;o__Enterobacterales;f__Erwiniaceae;g__Pantoea;s__Pantoea_eucalypti
PB1_gene_1229	291	ORM81130.1	291	291/291(100.00)	5.57e-206	succinate--CoA ligase subunit alpha [Pantoea deleyi]	470932	d__Bacteria;k__norank;p__Proteobacteria;c__Gammaproteobacteria;o__Enterobacterales;f__Erwiniaceae;g__Pantoea;s__Pantoea_deleyi
PB1_gene_1230	797	ORM82062.1	797	763/796(95.85)	0	beta-glucosidase [Pantoea deleyi]	470932	d__Bacteria;k__norank;p__Proteobacteria;c__Gammaproteobacteria;o__Enterobacterales;f__Erwiniaceae;g__Pantoea;s__Pantoea_deleyi
PB1_gene_1232	422	WP_111142187.1	507	419/422(99.29)	1.77e-305	apolipoprotein N-acyltransferase [Pantoea sp. ARC607]	2027922	d__Bacteria;k__norank;p__Proteobacteria;c__Gammaproteobacteria;o__Enterobacterales;f__Erwiniaceae;g__Pantoea;s__Pantoea_sp._ARC607
PB1_gene_1233	298	ORM79037.1	298	298/298(100.00)	7.98e-214	glutamate/aspartate ABC transporter substrate-binding protein [Pantoea deleyi]	470932	d__Bacteria;k__norank;p__Proteobacteria;c__Gammaproteobacteria;o__Enterobacterales;f__Erwiniaceae;g__Pantoea;s__Pantoea_deleyi
PB1_gene_1236	66	PAW34714.1	66	65/66(98.48)	6.85e-37	hypothetical protein CIL06_02845 [Pantoea vagans]	470934	d__Bacteria;k__norank;p__Proteobacteria;c__Gammaproteobacteria;o__Enterobacterales;f__Erwiniaceae;g__Pantoea;s__Pantoea_vagans
PB1_gene_1237	852	ORM81444.1	852	849/852(99.65)	0	adenylate cyclase [Pantoea deleyi]	470932	d__Bacteria;k__norank;p__Proteobacteria;c__Gammaproteobacteria;o__Enterobacterales;f__Erwiniaceae;g__Pantoea;s__Pantoea_deleyi
PB1_gene_1241	430	WP_111138819.1	430	430/430(100.00)	1.19e-309	serine--tRNA ligase [Pantoea sp. ARC607]	2027922	d__Bacteria;k__norank;p__Proteobacteria;c__Gammaproteobacteria;o__Enterobacterales;f__Erwiniaceae;g__Pantoea;s__Pantoea_sp._ARC607
PB1_gene_1242	383	WP_111138818.1	383	377/383(98.43)	8.52e-261	MFS transporter [Pantoea sp. ARC607]	2027922	d__Bacteria;k__norank;p__Proteobacteria;c__Gammaproteobacteria;o__Enterobacterales;f__Erwiniaceae;g__Pantoea;s__Pantoea_sp._ARC607
PB1_gene_1246	65	ORM82642.1	65	65/65(100.00)	1.84e-36	hypothetical protein HA42_07810 [Pantoea deleyi]	470932	d__Bacteria;k__norank;p__Proteobacteria;c__Gammaproteobacteria;o__Enterobacterales;f__Erwiniaceae;g__Pantoea;s__Pantoea_deleyi

PB1_gene_1247	269	WP_111139284.1	275	264/268(98.51)	2.90e-187	hypothetical protein [Pantoea sp. ARC607]	2027922	d__Bacteria;k__norank;p__Proteobacteria;c__Gammaproteobacteria;o__Enterobacterales;f__Erwiniaceae;g__Pantoea;s__Pantoea_sp._ARC607
PB1_gene_1248	225	WP_111139283.1	225	208/225(92.44)	3.98e-148	SDR family oxidoreductase [Pantoea sp. ARC607]	2027922	d__Bacteria;k__norank;p__Proteobacteria;c__Gammaproteobacteria;o__Enterobacterales;f__Erwiniaceae;g__Pantoea;s__Pantoea_sp._ARC607
PB1_gene_1251	319	WP_111139185.1	319	302/319(94.67)	1.66e-212	aldo/keto reductase [Pantoea sp. ARC607]	2027922	d__Bacteria;k__norank;p__Proteobacteria;c__Gammaproteobacteria;o__Enterobacterales;f__Erwiniaceae;g__Pantoea;s__Pantoea_sp._ARC607
PB1_gene_1252	404	ORM76524.1	411	390/404(96.53)	7.86e-267	multidrug transporter subunit MdtA [Pantoea deleyi]	470932	d__Bacteria;k__norank;p__Proteobacteria;c__Gammaproteobacteria;o__Enterobacterales;f__Erwiniaceae;g__Pantoea;s__Pantoea_deleyi
PB1_gene_1254	104	SKA25264.1	104	104/104(100.00)	1.05e-64	LSU ribosomal protein L24P [Pantoea agglomerans]	470933	d__Bacteria;k__norank;p__Proteobacteria;c__Gammaproteobacteria;o__Enterobacterales;f__Erwiniaceae;g__Pantoea;s__Pantoea_eucalypti
PB1_gene_1255	179	SKA25268.1	179	179/179(100.00)	1.99e-123	LSU ribosomal protein L5P [Pantoea agglomerans]	470933	d__Bacteria;k__norank;p__Proteobacteria;c__Gammaproteobacteria;o__Enterobacterales;f__Erwiniaceae;g__Pantoea;s__Pantoea_eucalypti
PB1_gene_1256	101	SKA25280.1	101	100/101(99.01)	8.14e-63	SSU ribosomal protein S14P [Pantoea agglomerans]	470933	d__Bacteria;k__norank;p__Proteobacteria;c__Gammaproteobacteria;o__Enterobacterales;f__Erwiniaceae;g__Pantoea;s__Pantoea_eucalypti
PB1_gene_1257	128	SKA25291.1	130	128/128(100.00)	3.98e-83	SSU ribosomal protein S8P [Pantoea agglomerans]	470933	d__Bacteria;k__norank;p__Proteobacteria;c__Gammaproteobacteria;o__Enterobacterales;f__Erwiniaceae;g__Pantoea;s__Pantoea_eucalypti
PB1_gene_1258	177	SKA25303.1	177	177/177(100.00)	7.82e-120	LSU ribosomal protein L6P [Pantoea agglomerans]	470933	d__Bacteria;k__norank;p__Proteobacteria;c__Gammaproteobacteria;o__Enterobacterales;f__Erwiniaceae;g__Pantoea;s__Pantoea_eucalypti
PB1_gene_1259	117	SKA25311.1	117	117/117(100.00)	3.74e-71	LSU ribosomal protein L18P [Pantoea agglomerans]	470933	d__Bacteria;k__norank;p__Proteobacteria;c__Gammaproteobacteria;o__Enterobacterales;f__Erwiniaceae;g__Pantoea;s__Pantoea_eucalypti
PB1_gene_1260	166	SKA25319.1	166	166/166(100.00)	3.24e-109	SSU ribosomal protein S5P [Pantoea agglomerans]	470933	d__Bacteria;k__norank;p__Proteobacteria;c__Gammaproteobacteria;o__Enterobacterales;f__Erwiniaceae;g__Pantoea;s__Pantoea_eucalypti
PB1_gene_1264	223	ORM80469.1	223	221/223(99.10)	8.74e-151	DNA-binding response regulator [Pantoea deleyi]	470932	d__Bacteria;k__norank;p__Proteobacteria;c__Gammaproteobacteria;o__Enterobacterales;f__Erwiniaceae;g__Pantoea;s__Pantoea_deleyi
PB1_gene_1265	462	ORM80470.1	462	455/462(98.48)	0	histidine kinase [Pantoea deleyi]	470932	d__Bacteria;k__norank;p__Proteobacteria;c__Gammaproteobacteria;o__Enterobacterales;f__Erwiniaceae;g__Pantoea;s__Pantoea_deleyi
PB1_gene_1266	299	ORM80471.1	299	292/299(97.66)	1.88e-192	multidrug DMT transporter [Pantoea deleyi]	470932	d__Bacteria;k__norank;p__Proteobacteria;c__Gammaproteobacteria;o__Enterobacterales;f__Erwiniaceae;g__Pantoea;s__Pantoea_deleyi
PB1_gene_1269	215	OQV42845.1	215	213/215(99.07)	1.01e-136	choline ABC transporter permease [Pantoea vagans]	470934	d__Bacteria;k__norank;p__Proteobacteria;c__Gammaproteobacteria;o__Enterobacterales;f__Erwiniaceae;g__Pantoea;s__Pantoea_vagans
PB1_gene_1270	378	WP_033732728.1	378	377/378(99.74)	2.60e-273	osmoprotectant ABC transporter ATP-binding protein OsmV [Pantoea vagans]	470934	d__Bacteria;k__norank;p__Proteobacteria;c__Gammaproteobacteria;o__Enterobacterales;f__Erwiniaceae;g__Pantoea;s__Pantoea_vagans
PB1_gene_1273	225	WP_111138944.1	225	206/225(91.56)	2.57e-144	anti-sigma factor [Pantoea sp. ARC607]	2027922	d__Bacteria;k__norank;p__Proteobacteria;c__Gammaproteobacteria;o__Enterobacterales;f__Erwiniaceae;g__Pantoea;s__Pantoea_sp._ARC607
PB1_gene_1274	182	ORM72618.1	182	180/182(98.90)	1.77e-132	RNA polymerase subunit sigma [Pantoea deleyi]	470932	d__Bacteria;k__norank;p__Proteobacteria;c__Gammaproteobacteria;o__Enterobacterales;f__Erwiniaceae;g__Pantoea;s__Pantoea_deleyi
PB1_gene_1275	191	ORM72617.1	186	185/186(99.46)	1.24e-124	fasciclin [Pantoea deleyi]	470932	d__Bacteria;k__norank;p__Proteobacteria;c__Gammaproteobacteria;o__Enterobacterales;f__Erwiniaceae;g__Pantoea;s__Pantoea_deleyi
PB1_gene_1277	362	ORM83600.1	362	332/362(91.71)	2.89e-230	hypothetical protein HA42_06015 [Pantoea deleyi]	470932	d__Bacteria;k__norank;p__Proteobacteria;c__Gammaproteobacteria;o__Enterobacterales;f__Erwiniaceae;g__Pantoea;s__Pantoea_deleyi
PB1_gene_1278	231	ORM83601.1	231	208/231(90.04)	1.12e-141	DNA-binding response regulator [Pantoea deleyi]	470932	d__Bacteria;k__norank;p__Proteobacteria;c__Gammaproteobacteria;o__Enterobacterales;f__Erwiniaceae;g__Pantoea;s__Pantoea_deleyi
PB1_gene_1281	106	ORM78816.1	129	105/106(99.06)	3.24e-67	hypothetical protein HA42_16280 [Pantoea deleyi]	470932	d__Bacteria;k__norank;p__Proteobacteria;c__Gammaproteobacteria;o__Enterobacterales;f__Erwiniaceae;g__Pantoea;s__Pantoea_deleyi
PB1_gene_1282	138	PZL84907.1	138	138/138(100.00)	3.34e-93	heat shock protein IbpA [Pantoea sp. ARC270]	2027923	d__Bacteria;k__norank;p__Proteobacteria;c__Gammaproteobacteria;o__Enterobacterales;f__Erwiniaceae;g__Pantoea;s__Pantoea_sp._ARC270
PB1_gene_1283	303	ORM78815.1	302	299/302(99.01)	9.82e-199	O-acetylserine/cysteine exporter [Pantoea deleyi]	470932	d__Bacteria;k__norank;p__Proteobacteria;c__Gammaproteobacteria;o__Enterobacterales;f__Erwiniaceae;g__Pantoea;s__Pantoea_deleyi
PB1_gene_1286	366	ORM86019.1	366	362/365(99.18)	9.28e-245	lipopolysaccharide ABC transporter permease LptF	470932	d__Bacteria;k__norank;p__Proteobacteria;c__Gammaproteobacteria;o__Enterobacterales;f__Erwiniaceae;g__Pantoea;s__Pantoea_deleyi

PB1_gene_1287	503	WP_058957611.1	503	502/503(99.80)	0	leucyl aminopeptidase [Type-F symbiont of Plautia stali]	549	d__Bacteria;k__norank;p__Proteobacteria;c__Gammaproteobacteria;o__Enterobacterales;f__Erwiniaceae;g__Pantoea;s__Pantoea_agglomerans
PB1_gene_1290	978	WP_031590409.1	981	720/981(73.39)	0	autotransporter outer membrane beta-barrel domain-containing protein [Pantoea agglomerans]	549	d__Bacteria;k__norank;p__Proteobacteria;c__Gammaproteobacteria;o__Enterobacterales;f__Erwiniaceae;g__Pantoea;s__Pantoea_agglomerans
PB1_gene_1291	557	SJZ32712.1	557	556/557(99.82)	0	SSU ribosomal protein S1P [Pantoea agglomerans]	470933	d__Bacteria;k__norank;p__Proteobacteria;c__Gammaproteobacteria;o__Enterobacterales;f__Erwiniaceae;g__Pantoea;s__Pantoea_eucalypti
PB1_gene_1292	228	ORM80781.1	228	228/228(100.00)	8.41e-156	cytidylate kinase [Pantoea deleyi]	470932	d__Bacteria;k__norank;p__Proteobacteria;c__Gammaproteobacteria;o__Enterobacterales;f__Erwiniaceae;g__Pantoea;s__Pantoea_deleyi
PB1_gene_1295	483	ORM78836.1	482	466/481(96.88)	0	altronate oxidoreductase [Pantoea deleyi]	470932	d__Bacteria;k__norank;p__Proteobacteria;c__Gammaproteobacteria;o__Enterobacterales;f__Erwiniaceae;g__Pantoea;s__Pantoea_deleyi
PB1_gene_1301	511	WP_111139913.1	511	508/511(99.41)	0	multidrug efflux MFS transporter subunit EmrB [Pantoea sp.	2027922	d__Bacteria;k__norank;p__Proteobacteria;c__Gammaproteobacteria;o__Enterobacterales;f__Erwiniaceae;g__Pantoea;s__Pantoea_sp._ARC607
PB1_gene_1308	957	ORM86559.1	957	952/957(99.48)	0	glycine dehydrogenase (aminomethyl-transferring) [Pantoea deleyi]	470932	d__Bacteria;k__norank;p__Proteobacteria;c__Gammaproteobacteria;o__Enterobacterales;f__Erwiniaceae;g__Pantoea;s__Pantoea_deleyi
PB1_gene_1309	139	ORM86558.1	128	128/128(100.00)	1.74e-84	glycine cleavage system protein H [Pantoea deleyi]	470932	d__Bacteria;k__norank;p__Proteobacteria;c__Gammaproteobacteria;o__Enterobacterales;f__Erwiniaceae;g__Pantoea;s__Pantoea_deleyi
PB1_gene_1311	360	ORM79154.1	324	318/324(98.15)	3.50e-235	diguanylate cyclase [Pantoea deleyi]	470932	d__Bacteria;k__norank;p__Proteobacteria;c__Gammaproteobacteria;o__Enterobacterales;f__Erwiniaceae;g__Pantoea;s__Pantoea_deleyi
PB1_gene_1312	191	ORM75580.1	191	189/191(98.95)	6.09e-133	transcriptional regulator [Pantoea deleyi]	470932	d__Bacteria;k__norank;p__Proteobacteria;c__Gammaproteobacteria;o__Enterobacterales;f__Erwiniaceae;g__Pantoea;s__Pantoea_deleyi
PB1_gene_1315	298	EIB97875.1	298	291/298(97.65)	1.01e-216	D-allose kinase [Pantoea sp. Sc1]	593105	d__Bacteria;k__norank;p__Proteobacteria;c__Gammaproteobacteria;o__Enterobacterales;f__Erwiniaceae;g__Pantoea;s__Pantoea_sp._Sc1
PB1_gene_1316	219	WP_111139944.1	230	218/219(99.54)	7.63e-157	ribulose-phosphate 3-epimerase [Pantoea sp. ARC607]	2027922	d__Bacteria;k__norank;p__Proteobacteria;c__Gammaproteobacteria;o__Enterobacterales;f__Erwiniaceae;g__Pantoea;s__Pantoea_sp._ARC607
PB1_gene_1317	350	ORM84928.1	349	347/349(99.43)	6.37e-230	sugar ABC transporter permease [Pantoea deleyi]	470932	d__Bacteria;k__norank;p__Proteobacteria;c__Gammaproteobacteria;o__Enterobacterales;f__Erwiniaceae;g__Pantoea;s__Pantoea_deleyi
PB1_gene_1320	168	ORM80917.1	168	167/168(99.40)	7.35e-116	ferritin [Pantoea deleyi]	470932	d__Bacteria;k__norank;p__Proteobacteria;c__Gammaproteobacteria;o__Enterobacterales;f__Erwiniaceae;g__Pantoea;s__Pantoea_deleyi
PB1_gene_1323	279	ORM82657.1	279	279/279(100.00)	3.70e-189	Fe-S cluster assembly protein HesB [Pantoea deleyi]	470932	d__Bacteria;k__norank;p__Proteobacteria;c__Gammaproteobacteria;o__Enterobacterales;f__Erwiniaceae;g__Pantoea;s__Pantoea_deleyi
PB1_gene_1324	289	WP_111141946.1	293	287/289(99.31)	8.09e-199	4-hydroxybenzoate octaprenyltransferase [Pantoea sp. ARC607]	2027922	d__Bacteria;k__norank;p__Proteobacteria;c__Gammaproteobacteria;o__Enterobacterales;f__Erwiniaceae;g__Pantoea;s__Pantoea_sp._ARC607
PB1_gene_1325	170	ORM82922.1	170	168/170(98.82)	1.60e-120	chorismate lyase [Pantoea deleyi]	470932	d__Bacteria;k__norank;p__Proteobacteria;c__Gammaproteobacteria;o__Enterobacterales;f__Erwiniaceae;g__Pantoea;s__Pantoea_deleyi
PB1_gene_1328	222	WP_111139066.1	222	219/222(98.65)	2.16e-155	ribonuclease T [Pantoea sp. ARC607]	2027922	d__Bacteria;k__norank;p__Proteobacteria;c__Gammaproteobacteria;o__Enterobacterales;f__Erwiniaceae;g__Pantoea;s__Pantoea_sp._ARC607
PB1_gene_1329	135	ORM79307.1	135	135/135(100.00)	7.64e-93	lactoylglutathione lyase [Pantoea deleyi]	470932	d__Bacteria;k__norank;p__Proteobacteria;c__Gammaproteobacteria;o__Enterobacterales;f__Erwiniaceae;g__Pantoea;s__Pantoea_deleyi
PB1_gene_1330	365	WP_111139064.1	365	359/365(98.36)	3.94e-266	alkene reductase [Pantoea sp. ARC607]	2027922	d__Bacteria;k__norank;p__Proteobacteria;c__Gammaproteobacteria;o__Enterobacterales;f__Erwiniaceae;g__Pantoea;s__Pantoea_sp._ARC607
PB1_gene_1331	198	ORM79305.1	198	193/197(97.97)	2.74e-128	TetR family transcriptional regulator [Pantoea deleyi]	470932	d__Bacteria;k__norank;p__Proteobacteria;c__Gammaproteobacteria;o__Enterobacterales;f__Erwiniaceae;g__Pantoea;s__Pantoea_deleyi
PB1_gene_1335	534	WP_111141175.1	532	510/534(95.51)	0	MFS transporter [Pantoea sp. ARC607]	2027922	d__Bacteria;k__norank;p__Proteobacteria;c__Gammaproteobacteria;o__Enterobacterales;f__Erwiniaceae;g__Pantoea;s__Pantoea_sp._ARC607
PB1_gene_1337	457	WP_111142238.1	457	441/457(96.50)	0	NAD-dependent succinate-semialdehyde dehydrogenase [Pantoea sp. ARC607]	2027922	d__Bacteria;k__norank;p__Proteobacteria;c__Gammaproteobacteria;o__Enterobacterales;f__Erwiniaceae;g__Pantoea;s__Pantoea_sp._ARC607

PB1_gene_1338	76	ORM84695.1	76	74/76(97.37)	6.76e-46	hypothetical protein HA42_03920 [Pantoea deleyi]	470932	d__Bacteria;k__norank;p__Proteobacteria;c__Gammaproteobacteria;o__Enterobacterales;f__Erwiniaceae;g__Pantoea;s__Pantoea_deleyi
PB1_gene_1340	325	WP_083070371.1	551	321/325(98.77)	5.32e-215	cation acetate symporter [Pantoea vagans]	53335	d__Bacteria;k__norank;p__Proteobacteria;c__Gammaproteobacteria;o__Enterobacterales;f__Erwiniaceae;g__Pantoea;s__norank
PB1_gene_1341	109	ORM77791.1	109	109/109(100.00)	1.64e-68	hypothetical protein HA42_17325 [Pantoea deleyi]	470932	d__Bacteria;k__norank;p__Proteobacteria;c__Gammaproteobacteria;o__Enterobacterales;f__Erwiniaceae;g__Pantoea;s__Pantoea_deleyi
PB1_gene_1342	651	WP_095706360.1	651	640/651(98.31)	0	MULTISPECIES: acetate--CoA ligase [Pantoea]	53335	d__Bacteria;k__norank;p__Proteobacteria;c__Gammaproteobacteria;o__Enterobacterales;f__Erwiniaceae;g__Pantoea;s__norank
PB1_gene_1344	160	ORM75003.1	164	152/157(96.82)	1.93e-99	MarR family transcriptional regulator [Pantoea deleyi]	470932	d__Bacteria;k__norank;p__Proteobacteria;c__Gammaproteobacteria;o__Enterobacterales;f__Erwiniaceae;g__Pantoea;s__Pantoea_deleyi
PB1_gene_1345	58	ORM75002.1	77	55/58(94.83)	4.89e-30	hypothetical protein HA42_21250 [Pantoea deleyi]	470932	d__Bacteria;k__norank;p__Proteobacteria;c__Gammaproteobacteria;o__Enterobacterales;f__Erwiniaceae;g__Pantoea;s__Pantoea_deleyi
PB1_gene_1346	494	ORM80460.1	477	467/477(97.90)	0	invasin [Pantoea deleyi]	470932	d__Bacteria;k__norank;p__Proteobacteria;c__Gammaproteobacteria;o__Enterobacterales;f__Erwiniaceae;g__Pantoea;s__Pantoea_deleyi
PB1_gene_1349	405	WP_086906652.1	405	403/405(99.51)	1.02e-295	argininosuccinate synthase [Pantoea agglomerans]	549	d__Bacteria;k__norank;p__Proteobacteria;c__Gammaproteobacteria;o__Enterobacterales;f__Erwiniaceae;g__Pantoea;s__Pantoea_agglomerans
PB1_gene_1350	258	ORM80357.1	258	258/258(100.00)	7.21e-181	acetylglutamate kinase [Pantoea deleyi]	470932	d__Bacteria;k__norank;p__Proteobacteria;c__Gammaproteobacteria;o__Enterobacterales;f__Erwiniaceae;g__Pantoea;s__Pantoea_deleyi
PB1_gene_1351	334	ORM80367.1	334	329/334(98.50)	5.81e-241	N-acetyl-gamma-glutamyl-phosphate reductase [Pantoea	470932	d__Bacteria;k__norank;p__Proteobacteria;c__Gammaproteobacteria;o__Enterobacterales;f__Erwiniaceae;g__Pantoea;s__Pantoea_deleyi
PB1_gene_1353	450	WP_111139187.1	450	440/450(97.78)	0	molecular chaperone [Pantoea sp. ARC607]	2027922	d__Bacteria;k__norank;p__Proteobacteria;c__Gammaproteobacteria;o__Enterobacterales;f__Erwiniaceae;g__Pantoea;s__Pantoea_sp._ARC607
PB1_gene_1356	239	OXH78032.1	239	237/239(99.16)	4.91e-174	tRNA (guanosine(46)-N7)-methyltransferase TrmB [Pantoea agglomerans]	549	d__Bacteria;k__norank;p__Proteobacteria;c__Gammaproteobacteria;o__Enterobacterales;f__Erwiniaceae;g__Pantoea;s__Pantoea_agglomerans
PB1_gene_1357	108	ORM76861.1	108	108/108(100.00)	7.23e-76	hypothetical protein HA42_18575 [Pantoea deleyi]	470932	d__Bacteria;k__norank;p__Proteobacteria;c__Gammaproteobacteria;o__Enterobacterales;f__Erwiniaceae;g__Pantoea;s__Pantoea_deleyi
PB1_gene_1358	238	ORM76862.1	238	236/238(99.16)	1.19e-160	hypothetical protein HA42_18580 [Pantoea deleyi]	470932	d__Bacteria;k__norank;p__Proteobacteria;c__Gammaproteobacteria;o__Enterobacterales;f__Erwiniaceae;g__Pantoea;s__Pantoea_deleyi
PB1_gene_1360	928	ORM82747.1	928	919/928(99.03)	0	DNA polymerase I [Pantoea deleyi]	470932	d__Bacteria;k__norank;p__Proteobacteria;c__Gammaproteobacteria;o__Enterobacterales;f__Erwiniaceae;g__Pantoea;s__Pantoea_deleyi
PB1_gene_1361	160	WP_111141304.1	154	153/154(99.35)	7.91e-99	MarR family transcriptional regulator [Pantoea sp. ARC607]	2027922	d__Bacteria;k__norank;p__Proteobacteria;c__Gammaproteobacteria;o__Enterobacterales;f__Erwiniaceae;g__Pantoea;s__Pantoea_sp._ARC607
PB1_gene_1362	142	ORM80627.1	142	140/142(98.59)	1.70e-97	organic hydroperoxide resistance protein [Pantoea deleyi]	470932	d__Bacteria;k__norank;p__Proteobacteria;c__Gammaproteobacteria;o__Enterobacterales;f__Erwiniaceae;g__Pantoea;s__Pantoea_deleyi
PB1_gene_1363	563	ORM80626.1	563	559/563(99.29)	0	phosphoethanolamine transferase [Pantoea deleyi]	470932	d__Bacteria;k__norank;p__Proteobacteria;c__Gammaproteobacteria;o__Enterobacterales;f__Erwiniaceae;g__Pantoea;s__Pantoea_deleyi
PB1_gene_1365	551	WP_111140905.1	551	547/551(99.27)	0	L-lactate permease [Pantoea sp. ARC607]	2027922	d__Bacteria;k__norank;p__Proteobacteria;c__Gammaproteobacteria;o__Enterobacterales;f__Erwiniaceae;g__Pantoea;s__Pantoea_sp._ARC607
PB1_gene_1368	285	PZL96649.1	431	283/285(99.30)	9.45e-200	cellulose biosynthesis cyclic di-GMP-binding regulatory protein BcsB, partial [Pantoea sp.	2027922	d__Bacteria;k__norank;p__Proteobacteria;c__Gammaproteobacteria;o__Enterobacterales;f__Erwiniaceae;g__Pantoea;s__Pantoea_sp._ARC607
PB1_gene_1371	353	WP_094110180.1	315	228/316(72.15)	1.31e-159	hypothetical protein [Lonsdalea iberica]	None	Unclassified
PB1_gene_1372	184	SEA22738.1	185	114/183(62.30)	5.32e-85	hypothetical protein SAMN02982996_01176 [Lonsdalea quercina]	71657	d__Bacteria;k__norank;p__Proteobacteria;c__Gammaproteobacteria;o__Enterobacterales;f__Pectobacteriaceae;g__Lonsdalea;s__Lonsdalea_quercina
PB1_gene_1373	214	WP_063877196.1	211	154/210(73.33)	2.08e-121	hypothetical protein [Pantoea sp. OXWO6B1]	1835724	d__Bacteria;k__norank;p__Proteobacteria;c__Gammaproteobacteria;o__Enterobacterales;f__Erwiniaceae;g__Pantoea;s__Pantoea_sp._OXWO6B1
PB1_gene_1375	458	ORM76623.1	451	448/451(99.33)	0	phosphatidylserine synthase [Pantoea deleyi]	470932	d__Bacteria;k__norank;p__Proteobacteria;c__Gammaproteobacteria;o__Enterobacterales;f__Erwiniaceae;g__Pantoea;s__Pantoea_deleyi

PB1_gene_1377	429	WP_111142178.1	429	424/429(98.83)	5.37e-301	MFS transporter [Pantoea sp. ARC607]	2027922	d__Bacteria;k__norank;p__Proteobacteria;c__Gammaproteobacteria;o__Enterobacterales;f__Erwiniaceae;g__Pantoea;s__Pantoea_sp._ARC607
PB1_gene_1379	303	WP_111139799.1	300	262/303(86.47)	1.38e-177	LysR family transcriptional regulator [Pantoea sp. ARC607]	2027922	d__Bacteria;k__norank;p__Proteobacteria;c__Gammaproteobacteria;o__Enterobacterales;f__Erwiniaceae;g__Pantoea;s__Pantoea_sp._ARC607
PB1_gene_1380	579	ORM86799.1	576	573/576(99.48)	0	arginine--tRNA ligase [Pantoea deleyi]	470932	d__Bacteria;k__norank;p__Proteobacteria;c__Gammaproteobacteria;o__Enterobacterales;f__Erwiniaceae;g__Pantoea;s__Pantoea_deleyi
PB1_gene_1383	480	WP_111141402.1	480	467/480(97.29)	0	xylulokinase [Pantoea sp. ARC607]	2027922	d__Bacteria;k__norank;p__Proteobacteria;c__Gammaproteobacteria;o__Enterobacterales;f__Erwiniaceae;g__Pantoea;s__Pantoea_sp._ARC607
PB1_gene_1384	413	ORM85468.1	413	406/413(98.31)	2.29e-287	MFS transporter [Pantoea deleyi]	470932	d__Bacteria;k__norank;p__Proteobacteria;c__Gammaproteobacteria;o__Enterobacterales;f__Erwiniaceae;g__Pantoea;s__Pantoea_deleyi
PB1_gene_1385	244	OQV39567.1	244	244/244(100.00)	3.30e-170	phosphate transport system regulator PhoU [Pantoea vagans]	470934	d__Bacteria;k__norank;p__Proteobacteria;c__Gammaproteobacteria;o__Enterobacterales;f__Erwiniaceae;g__Pantoea;s__Pantoea_vagans
PB1_gene_1386	257	WP_111141400.1	257	256/257(99.61)	2.95e-184	phosphate ABC transporter ATP-binding protein PstB [Pantoea sp. ARC607]	2027922	d__Bacteria;k__norank;p__Proteobacteria;c__Gammaproteobacteria;o__Enterobacterales;f__Erwiniaceae;g__Pantoea;s__Pantoea_sp._ARC607
PB1_gene_1387	296	ORM85465.1	296	294/296(99.32)	4.36e-201	phosphate ABC transporter, permease protein PstA [Pantoea deleyi]	470932	d__Bacteria;k__norank;p__Proteobacteria;c__Gammaproteobacteria;o__Enterobacterales;f__Erwiniaceae;g__Pantoea;s__Pantoea_deleyi
PB1_gene_1394	151	ORM74992.1	151	151/151(100.00)	6.26e-102	RNA polymerase-binding protein DksA [Pantoea deleyi]	470932	d__Bacteria;k__norank;p__Proteobacteria;c__Gammaproteobacteria;o__Enterobacterales;f__Erwiniaceae;g__Pantoea;s__Pantoea_deleyi
PB1_gene_1395	308	WP_111140282.1	308	295/308(95.78)	5.41e-217	tRNA glutamyl-Q(34) synthetase GluQRS [Pantoea sp. ARC607]	2027922	d__Bacteria;k__norank;p__Proteobacteria;c__Gammaproteobacteria;o__Enterobacterales;f__Erwiniaceae;g__Pantoea;s__Pantoea_sp._ARC607
PB1_gene_1398	220	ORM82190.1	220	220/220(100.00)	1.07e-154	hypothetical protein HA42_08800 [Pantoea deleyi]	470932	d__Bacteria;k__norank;p__Proteobacteria;c__Gammaproteobacteria;o__Enterobacterales;f__Erwiniaceae;g__Pantoea;s__Pantoea_deleyi
PB1_gene_1399	396	ORM82189.1	396	390/396(98.48)	1.71e-278	cystathionine beta-lyase [Pantoea deleyi]	470932	d__Bacteria;k__norank;p__Proteobacteria;c__Gammaproteobacteria;o__Enterobacterales;f__Erwiniaceae;g__Pantoea;s__Pantoea_deleyi
PB1_gene_1402	143	ORM84516.1	143	141/143(98.60)	1.34e-90	rhodanese-like domain-containing protein [Pantoea deleyi]	470932	d__Bacteria;k__norank;p__Proteobacteria;c__Gammaproteobacteria;o__Enterobacterales;f__Erwiniaceae;g__Pantoea;s__Pantoea_deleyi
PB1_gene_1404	156	ORM84518.1	156	156/156(100.00)	2.52e-108	protein-export chaperone SecB [Pantoea deleyi]	470932	d__Bacteria;k__norank;p__Proteobacteria;c__Gammaproteobacteria;o__Enterobacterales;f__Erwiniaceae;g__Pantoea;s__Pantoea_deleyi
PB1_gene_1405	338	ORM84519.1	339	335/338(99.11)	1.53e-236	glycerol-3-phosphate dehydrogenase [Pantoea deleyi]	470932	d__Bacteria;k__norank;p__Proteobacteria;c__Gammaproteobacteria;o__Enterobacterales;f__Erwiniaceae;g__Pantoea;s__Pantoea_deleyi
PB1_gene_1408	775	WP_064085965.1	693	625/648(96.45)	0	hypothetical protein [Wolbachia endosymbiont of Dactylopius coccus]	None	Unclassified
PB1_gene_1411	82	ORM85059.1	82	78/82(95.12)	4.55e-49	hypothetical protein HA42_03295 [Pantoea deleyi]	470932	d__Bacteria;k__norank;p__Proteobacteria;c__Gammaproteobacteria;o__Enterobacterales;f__Erwiniaceae;g__Pantoea;s__Pantoea_deleyi
PB1_gene_1412	66	ORM85060.1	66	66/66(100.00)	5.00e-39	hypothetical protein HA42_03300 [Pantoea deleyi]	470932	d__Bacteria;k__norank;p__Proteobacteria;c__Gammaproteobacteria;o__Enterobacterales;f__Erwiniaceae;g__Pantoea;s__Pantoea_deleyi
PB1_gene_1413	113	ORM85061.1	113	104/113(92.04)	5.19e-67	hypothetical protein HA42_03305 [Pantoea deleyi]	470932	d__Bacteria;k__norank;p__Proteobacteria;c__Gammaproteobacteria;o__Enterobacterales;f__Erwiniaceae;g__Pantoea;s__Pantoea_deleyi
PB1_gene_1414	103	ORM85062.1	103	103/103(100.00)	6.38e-61	flagellar hook-basal body complex protein FliE [Pantoea	470932	d__Bacteria;k__norank;p__Proteobacteria;c__Gammaproteobacteria;o__Enterobacterales;f__Erwiniaceae;g__Pantoea;s__Pantoea_deleyi
PB1_gene_1417	67	STV90272.1	114	59/67(88.06)	9.24e-33	Urea carboxylase [Klebsiella oxytoca]	1134687	d__Bacteria;k__norank;p__Proteobacteria;c__Gammaproteobacteria;o__Enterobacterales;f__Enterobacteriaceae;g__Klebsiella;s__Klebsiella_michiganensis
PB1_gene_1418	211	ORM76225.1	211	208/211(98.58)	4.97e-160	urea carboxylase [Pantoea deleyi]	470932	d__Bacteria;k__norank;p__Proteobacteria;c__Gammaproteobacteria;o__Enterobacterales;f__Erwiniaceae;g__Pantoea;s__Pantoea_deleyi
PB1_gene_1419	242	WP_111138423.1	242	232/242(95.87)	1.99e-170	urea carboxylase-associated family protein [Pantoea sp.	2027922	d__Bacteria;k__norank;p__Proteobacteria;c__Gammaproteobacteria;o__Enterobacterales;f__Erwiniaceae;g__Pantoea;s__Pantoea_sp._ARC607
PB1_gene_1420	261	ORM76227.1	261	253/261(96.93)	7.79e-182	lauroyl acyltransferase [Pantoea deleyi]	470932	d__Bacteria;k__norank;p__Proteobacteria;c__Gammaproteobacteria;o__Enterobacterales;f__Erwiniaceae;g__Pantoea;s__Pantoea_deleyi

PB1_gene_1421	271	WP_111138421.1	271	270/271(99.63)	6.86e-181	ABC transporter permease [Pantoea sp. ARC607]	2027922	d__Bacteria;k__norank;p__Proteobacteria;c__Gammaproteobacteria;o__Enterobacterales;f__Erwiniaceae;g__Pantoea;s__Pantoea_sp._ARC607
PB1_gene_1422	75	SJZ44221.1	79	54/54(100.00)	3.97e-34	hypothetical protein SAMN03097723_0943 [Pantoea agglomerans]	470933	d__Bacteria;k__norank;p__Proteobacteria;c__Gammaproteobacteria;o__Enterobacterales;f__Erwiniaceae;g__Pantoea;s__Pantoea_eucalypti
PB1_gene_1423	305	WP_111139061.1	298	288/298(96.64)	1.02e-211	aldo/keto reductase family oxidoreductase [Pantoea sp. ARC607]	2027922	d__Bacteria;k__norank;p__Proteobacteria;c__Gammaproteobacteria;o__Enterobacterales;f__Erwiniaceae;g__Pantoea;s__Pantoea_sp._ARC607
PB1_gene_1424	172	WP_111139060.1	172	166/172(96.51)	4.22e-117	superoxide dismutase [Cu-Zn] SodC2 [Pantoea sp. ARC607]	2027922	d__Bacteria;k__norank;p__Proteobacteria;c__Gammaproteobacteria;o__Enterobacterales;f__Erwiniaceae;g__Pantoea;s__Pantoea_sp._ARC607
PB1_gene_1428	405	ORM85212.1	405	384/404(95.05)	3.17e-274	diguanylate phosphodiesterase [Pantoea deleyi]	470932	d__Bacteria;k__norank;p__Proteobacteria;c__Gammaproteobacteria;o__Enterobacterales;f__Erwiniaceae;g__Pantoea;s__Pantoea_deleyi
PB1_gene_1429	77	ORM85211.1	77	74/77(96.10)	1.07e-47	DNA polymerase V subunit [Pantoea deleyi]	470932	d__Bacteria;k__norank;p__Proteobacteria;c__Gammaproteobacteria;o__Enterobacterales;f__Erwiniaceae;g__Pantoea;s__Pantoea_deleyi
PB1_gene_1430	109	ORM85210.1	110	108/110(98.18)	5.91e-69	hypothetical protein HA42_02950 [Pantoea deleyi]	470932	d__Bacteria;k__norank;p__Proteobacteria;c__Gammaproteobacteria;o__Enterobacterales;f__Erwiniaceae;g__Pantoea;s__Pantoea_deleyi
PB1_gene_1432	567	ORM80601.1	567	557/567(98.24)	0	D-lactate dehydrogenase [Pantoea deleyi]	470932	d__Bacteria;k__norank;p__Proteobacteria;c__Gammaproteobacteria;o__Enterobacterales;f__Erwiniaceae;g__Pantoea;s__Pantoea_deleyi
PB1_gene_1434	104	ORM81911.1	104	99/103(96.12)	2.79e-69	L-rhamnose mutarotase [Pantoea deleyi]	470932	d__Bacteria;k__norank;p__Proteobacteria;c__Gammaproteobacteria;o__Enterobacterales;f__Erwiniaceae;g__Pantoea;s__Pantoea_deleyi
PB1_gene_1435	280	WP_111140540.1	280	268/280(95.71)	1.72e-199	rhamnulose-1-phosphate aldolase [Pantoea sp. ARC607]	2027922	d__Bacteria;k__norank;p__Proteobacteria;c__Gammaproteobacteria;o__Enterobacterales;f__Erwiniaceae;g__Pantoea;s__Pantoea_sp._ARC607
PB1_gene_1436	418	ORM81913.1	418	411/418(98.33)	8.99e-308	L-rhamnose isomerase [Pantoea deleyi]	470932	d__Bacteria;k__norank;p__Proteobacteria;c__Gammaproteobacteria;o__Enterobacterales;f__Erwiniaceae;g__Pantoea;s__Pantoea_deleyi
PB1_gene_1438	400	ORM79312.1	400	395/400(98.75)	1.80e-266	Bcr/CflA family multidrug efflux transporter [Pantoea deleyi]	470932	d__Bacteria;k__norank;p__Proteobacteria;c__Gammaproteobacteria;o__Enterobacterales;f__Erwiniaceae;g__Pantoea;s__Pantoea_deleyi
PB1_gene_1439	382	ORM79313.1	382	370/382(96.86)	3.00e-290	cyclopropane-fatty-acyl-phospholipid synthase [Pantoea deleyi]	470932	d__Bacteria;k__norank;p__Proteobacteria;c__Gammaproteobacteria;o__Enterobacterales;f__Erwiniaceae;g__Pantoea;s__Pantoea_deleyi
PB1_gene_1442	280	ORM84128.1	280	268/280(95.71)	1.99e-173	iron ABC transporter permease [Pantoea deleyi]	470932	d__Bacteria;k__norank;p__Proteobacteria;c__Gammaproteobacteria;o__Enterobacterales;f__Erwiniaceae;g__Pantoea;s__Pantoea_deleyi
PB1_gene_1443	115	WP_046103193.1	144	61/80(76.25)	9.91e-35	hypothetical protein [Pantoea anthophila]	470931	d__Bacteria;k__norank;p__Proteobacteria;c__Gammaproteobacteria;o__Enterobacterales;f__Erwiniaceae;g__Pantoea;s__Pantoea_anthophila
PB1_gene_1444	243	EFM17733.1	242	186/243(76.54)	5.00e-130	hypothetical protein PanABDRAFT_4244 [Pantoea sp. aB]	517433	d__Bacteria;k__norank;p__Proteobacteria;c__Gammaproteobacteria;o__Enterobacterales;f__Erwiniaceae;g__Pantoea;s__Pantoea_sp._aB
PB1_gene_1445	139	EFM17732.1	139	112/139(80.58)	8.92e-69	hypothetical protein PanABDRAFT_4243 [Pantoea sp. aB]	517433	d__Bacteria;k__norank;p__Proteobacteria;c__Gammaproteobacteria;o__Enterobacterales;f__Erwiniaceae;g__Pantoea;s__Pantoea_sp._aB
PB1_gene_1447	255	ORM75372.1	267	254/255(99.61)	3.62e-180	cellulose synthase operon protein YhjQ [Pantoea deleyi]	470932	d__Bacteria;k__norank;p__Proteobacteria;c__Gammaproteobacteria;o__Enterobacterales;f__Erwiniaceae;g__Pantoea;s__Pantoea_deleyi
PB1_gene_1448	703	ORM75371.1	703	697/703(99.15)	0	cellulose synthase catalytic subunit (UDP-forming) [Pantoea	470932	d__Bacteria;k__norank;p__Proteobacteria;c__Gammaproteobacteria;o__Enterobacterales;f__Erwiniaceae;g__Pantoea;s__Pantoea_deleyi
PB1_gene_1451	156	WP_111140976.1	156	150/156(96.15)	8.13e-105	GNAT family N-acetyltransferase [Pantoea sp. ARC607]	2027922	d__Bacteria;k__norank;p__Proteobacteria;c__Gammaproteobacteria;o__Enterobacterales;f__Erwiniaceae;g__Pantoea;s__Pantoea_sp._ARC607
PB1_gene_1452	287	ORM84150.1	287	284/287(98.95)	1.76e-191	23S rRNA pseudouridine synthase F [Pantoea deleyi]	470932	d__Bacteria;k__norank;p__Proteobacteria;c__Gammaproteobacteria;o__Enterobacterales;f__Erwiniaceae;g__Pantoea;s__Pantoea_deleyi
PB1_gene_1455	281	ORM80487.1	281	276/281(98.22)	2.19e-207	NADPH-dependent 7-cyano-7-deazaguanine reductase QueF [Pantoea deleyi]	470932	d__Bacteria;k__norank;p__Proteobacteria;c__Gammaproteobacteria;o__Enterobacterales;f__Erwiniaceae;g__Pantoea;s__Pantoea_deleyi

PB1_gene_1456	182	ORM80486.1	182	178/182(97.80)	2.50e-123	SecY-interacting protein [Pantoea deleyi]	470932	d__Bacteria;k__norank;p__Proteobacteria;c__Gammaproteobacteria;o__Enterobacterales;f__Erwiniaceae;g__Pantoea;s__Pantoea_deleyi
PB1_gene_1457	109	ORM80485.1	109	105/109(96.33)	2.07e-71	hypothetical protein HA42_14300 [Pantoea deleyi]	470932	d__Bacteria;k__norank;p__Proteobacteria;c__Gammaproteobacteria;o__Enterobacterales;f__Erwiniaceae;g__Pantoea;s__Pantoea_deleyi
PB1_gene_1460	88	WP_111142224.1	88	88/88(100.00)	4.32e-61	FAD assembly factor SdhE [Pantoea sp. ARC607]	2027922	d__Bacteria;k__norank;p__Proteobacteria;c__Gammaproteobacteria;o__Enterobacterales;f__Erwiniaceae;g__Pantoea;s__Pantoea_sp._ARC607
PB1_gene_1461	136	WP_111142223.1	136	125/136(91.91)	9.26e-90	hypothetical protein [Pantoea sp. ARC607]	2027922	d__Bacteria;k__norank;p__Proteobacteria;c__Gammaproteobacteria;o__Enterobacterales;f__Erwiniaceae;g__Pantoea;s__Pantoea_sp._ARC607
PB1_gene_1463	252	WP_099257873.1	271	229/252(90.87)	1.81e-155	hypothetical protein [Pantoea agglomerans]	549	d__Bacteria;k__norank;p__Proteobacteria;c__Gammaproteobacteria;o__Enterobacterales;f__Erwiniaceae;g__Pantoea;s__Pantoea_agglomerans
PB1_gene_1467	231	ORM81578.1	231	228/231(98.70)	1.34e-158	DNA-binding response regulator [Pantoea deleyi]	470932	d__Bacteria;k__norank;p__Proteobacteria;c__Gammaproteobacteria;o__Enterobacterales;f__Erwiniaceae;g__Pantoea;s__Pantoea_deleyi
PB1_gene_1468	389	ORM81577.1	389	379/389(97.43)	3.48e-263	two-component sensor histidine kinase [Pantoea deleyi]	470932	d__Bacteria;k__norank;p__Proteobacteria;c__Gammaproteobacteria;o__Enterobacterales;f__Erwiniaceae;g__Pantoea;s__Pantoea_deleyi
PB1_gene_1469	377	WP_111138266.1	377	348/376(92.55)	2.12e-235	efflux RND transporter periplasmic adaptor subunit [Pantoea sp. ARC607]	2027922	d__Bacteria;k__norank;p__Proteobacteria;c__Gammaproteobacteria;o__Enterobacterales;f__Erwiniaceae;g__Pantoea;s__Pantoea_sp._ARC607
PB1_gene_1471	978	WP_111141356.1	1215	782/978(79.96)	0	autotransporter outer membrane beta-barrel domain-containing protein [Pantoea sp. ARC607]	2027922	d__Bacteria;k__norank;p__Proteobacteria;c__Gammaproteobacteria;o__Enterobacterales;f__Erwiniaceae;g__Pantoea;s__Pantoea_sp._ARC607
PB1_gene_1473	408	ORM76354.1	408	397/407(97.54)	1.62e-289	peptidase T [Pantoea deleyi]	470932	d__Bacteria;k__norank;p__Proteobacteria;c__Gammaproteobacteria;o__Enterobacterales;f__Erwiniaceae;g__Pantoea;s__Pantoea_deleyi
PB1_gene_1476	321	ORM84912.1	324	321/321(100.00)	1.07e-237	S26 family signal peptidase [Pantoea deleyi]	470932	d__Bacteria;k__norank;p__Proteobacteria;c__Gammaproteobacteria;o__Enterobacterales;f__Erwiniaceae;g__Pantoea;s__Pantoea_deleyi
PB1_gene_1477	226	OQV42086.1	226	226/226(100.00)	1.52e-157	ribonuclease III [Pantoea vagans]	470934	d__Bacteria;k__norank;p__Proteobacteria;c__Gammaproteobacteria;o__Enterobacterales;f__Erwiniaceae;g__Pantoea;s__Pantoea_vagans
PB1_gene_1478	301	ORM84914.1	301	301/301(100.00)	3.67e-216	GTPase Era [Pantoea deleyi]	470932	d__Bacteria;k__norank;p__Proteobacteria;c__Gammaproteobacteria;o__Enterobacterales;f__Erwiniaceae;g__Pantoea;s__Pantoea_deleyi
PB1_gene_1481	374	ORM77453.1	374	373/374(99.73)	3.29e-274	S-(hydroxymethyl)glutathione dehydrogenase/class III alcohol dehydrogenase [Pantoea deleyi]	470932	d__Bacteria;k__norank;p__Proteobacteria;c__Gammaproteobacteria;o__Enterobacterales;f__Erwiniaceae;g__Pantoea;s__Pantoea_deleyi
PB1_gene_1482	303	WP_111138760.1	303	300/303(99.01)	1.43e-214	LysR family transcriptional regulator [Pantoea sp. ARC607]	2027922	d__Bacteria;k__norank;p__Proteobacteria;c__Gammaproteobacteria;o__Enterobacterales;f__Erwiniaceae;g__Pantoea;s__Pantoea_sp._ARC607
PB1_gene_1484	230	ORM78813.1	235	224/230(97.39)	5.93e-174	hypothetical protein HA42_16255 [Pantoea deleyi]	470932	d__Bacteria;k__norank;p__Proteobacteria;c__Gammaproteobacteria;o__Enterobacterales;f__Erwiniaceae;g__Pantoea;s__Pantoea_deleyi
PB1_gene_1485	424	WP_111141059.1	424	402/424(94.81)	5.76e-298	ATP-grasp domain-containing protein [Pantoea sp. ARC607]	2027922	d__Bacteria;k__norank;p__Proteobacteria;c__Gammaproteobacteria;o__Enterobacterales;f__Erwiniaceae;g__Pantoea;s__Pantoea_sp._ARC607
PB1_gene_1488	136	OON35495.1	136	136/136(100.00)	9.26e-90	50S ribosomal protein L16 [Izhakiella australiensis]	1926881	d__Bacteria;k__norank;p__Proteobacteria;c__Gammaproteobacteria;o__Enterobacterales;f__Erwiniaceae;g__Izhakiella;s__Izhakiella_australiensis
PB1_gene_1489	63	SKA25218.1	63	63/63(100.00)	3.01e-32	LSU ribosomal protein L29P [Pantoea agglomerans]	470933	d__Bacteria;k__norank;p__Proteobacteria;c__Gammaproteobacteria;o__Enterobacterales;f__Erwiniaceae;g__Pantoea;s__Pantoea_eucalypti
PB1_gene_1490	84	SKA25232.1	84	84/84(100.00)	6.86e-54	SSU ribosomal protein S17P [Pantoea agglomerans]	470933	d__Bacteria;k__norank;p__Proteobacteria;c__Gammaproteobacteria;o__Enterobacterales;f__Erwiniaceae;g__Pantoea;s__Pantoea_eucalypti
PB1_gene_1491	450	WP_033734118.1	450	425/448(94.87)	0	8-oxoguanine deaminase [Pantoea vagans]	470934	d__Bacteria;k__norank;p__Proteobacteria;c__Gammaproteobacteria;o__Enterobacterales;f__Erwiniaceae;g__Pantoea;s__Pantoea_vagans
PB1_gene_1492	123	SKA25253.1	123	123/123(100.00)	4.98e-81	LSU ribosomal protein L14P [Pantoea agglomerans]	470933	d__Bacteria;k__norank;p__Proteobacteria;c__Gammaproteobacteria;o__Enterobacterales;f__Erwiniaceae;g__Pantoea;s__Pantoea_eucalypti
PB1_gene_1494	282	ORM76910.1	282	277/281(98.58)	3.28e-189	N-acetylmuramoyl-L-alanine amidase [Pantoea deleyi]	470932	d__Bacteria;k__norank;p__Proteobacteria;c__Gammaproteobacteria;o__Enterobacterales;f__Erwiniaceae;g__Pantoea;s__Pantoea_deleyi
PB1_gene_1495	47	ORM76911.1	69	45/47(95.74)	7.08e-21	hypothetical protein HA42_18525 [Pantoea deleyi]	470932	d__Bacteria;k__norank;p__Proteobacteria;c__Gammaproteobacteria;o__Enterobacterales;f__Erwiniaceae;g__Pantoea;s__Pantoea_deleyi

PB1_gene_1496	199	KFC77274.1	195	165/195(84.62)	1.28e-112	hypothetical protein GEAM_4400 [Ewingella	910964	d__Bacteria;k__norank;p__Proteobacteria;c__Gammaproteobacteria;o__Enterobacterales;f__Yersiniaceae;g__Ewingella;s__Ewingella_americana
PB1_gene_1497	203	BAK12142.1	151	81/149(54.36)	9.76e-47	hypothetical protein PAJ_2062 [Pantoea ananatis AJ13355]	932677	d__Bacteria;k__norank;p__Proteobacteria;c__Gammaproteobacteria;o__Enterobacterales;f__Erwiniaceae;g__Pantoea;s__Pantoea_ananatis
PB1_gene_1499	348	ORM80796.1	348	342/348(98.28)	6.85e-247	3-deoxy-7-phosphoheptulonate synthase [Pantoea deleyi]	470932	d__Bacteria;k__norank;p__Proteobacteria;c__Gammaproteobacteria;o__Enterobacterales;f__Erwiniaceae;g__Pantoea;s__Pantoea_deleyi
PB1_gene_1500	483	WP_111139084.1	483	470/483(97.31)	0	YdiU family protein [Pantoea sp. ARC607]	2027922	d__Bacteria;k__norank;p__Proteobacteria;c__Gammaproteobacteria;o__Enterobacterales;f__Erwiniaceae;g__Pantoea;s__Pantoea_sp._ARC607
PB1_gene_1503	303	WP_049840378.1	298	170/292(58.22)	2.59e-116	alpha/beta hydrolase [Klebsiella sp. RIT-PI-d]	1681196	d__Bacteria;k__norank;p__Proteobacteria;c__Gammaproteobacteria;o__Enterobacterales;f__Enterobacteriaceae;g__Klebsiella;s__Klebsiella_sp._RIT-PI-d
PB1_gene_1504	248	WP_128085337.1	248	234/248(94.35)	1.32e-162	biotin--[acetyl-CoA-carboxylase] ligase [Pantoea deleyi]	None	Unclassified
PB1_gene_1508	183	ORM82301.1	183	173/182(95.05)	2.05e-118	hypothetical protein HA42_08725 [Pantoea deleyi]	470932	d__Bacteria;k__norank;p__Proteobacteria;c__Gammaproteobacteria;o__Enterobacterales;f__Erwiniaceae;g__Pantoea;s__Pantoea_deleyi
PB1_gene_1509	365	ORM82302.1	365	359/365(98.36)	1.19e-251	D-alanine--D-alanine ligase A [Pantoea deleyi]	470932	d__Bacteria;k__norank;p__Proteobacteria;c__Gammaproteobacteria;o__Enterobacterales;f__Erwiniaceae;g__Pantoea;s__Pantoea_deleyi
PB1_gene_1512	319	ORM85299.1	319	307/319(96.24)	7.95e-245	alpha-N-arabinofuranosidase [Pantoea deleyi]	470932	d__Bacteria;k__norank;p__Proteobacteria;c__Gammaproteobacteria;o__Enterobacterales;f__Erwiniaceae;g__Pantoea;s__Pantoea_deleyi
PB1_gene_1513	470	ORM85300.1	470	464/470(98.72)	0	permease [Pantoea deleyi]	470932	d__Bacteria;k__norank;p__Proteobacteria;c__Gammaproteobacteria;o__Enterobacterales;f__Erwiniaceae;g__Pantoea;s__Pantoea_deleyi
PB1_gene_1516	92	ORM84700.1	92	88/92(95.65)	1.32e-57	acylphosphatase [Pantoea deleyi]	470932	d__Bacteria;k__norank;p__Proteobacteria;c__Gammaproteobacteria;o__Enterobacterales;f__Erwiniaceae;g__Pantoea;s__Pantoea_deleyi
PB1_gene_1517	396	ORM84701.1	396	391/396(98.74)	1.23e-287	23S rRNA (cytosine(1962)-C(5))-methyltransferase [Pantoea	470932	d__Bacteria;k__norank;p__Proteobacteria;c__Gammaproteobacteria;o__Enterobacterales;f__Erwiniaceae;g__Pantoea;s__Pantoea_deleyi
PB1_gene_1518	105	ORM84702.1	105	105/105(100.00)	1.22e-68	heat-shock protein HspQ [Pantoea deleyi]	470932	d__Bacteria;k__norank;p__Proteobacteria;c__Gammaproteobacteria;o__Enterobacterales;f__Erwiniaceae;g__Pantoea;s__Pantoea_deleyi
PB1_gene_1519	137	WP_111141665.1	137	135/137(98.54)	1.21e-90	CoA-binding protein [Pantoea sp. ARC607]	2027922	d__Bacteria;k__norank;p__Proteobacteria;c__Gammaproteobacteria;o__Enterobacterales;f__Erwiniaceae;g__Pantoea;s__Pantoea_sp._ARC607
PB1_gene_1521	337	ORM81538.1	311	290/291(99.66)	7.90e-200	hypothetical protein HA42_10355 [Pantoea deleyi]	470932	d__Bacteria;k__norank;p__Proteobacteria;c__Gammaproteobacteria;o__Enterobacterales;f__Erwiniaceae;g__Pantoea;s__Pantoea_deleyi
PB1_gene_1522	461	WP_073534446.1	460	359/460(78.04)	3.34e-251	hypothetical protein [Serratia marcescens]	615	d__Bacteria;k__norank;p__Proteobacteria;c__Gammaproteobacteria;o__Enterobacterales;f__Yersiniaceae;g__Serratia;s__Serratia_marcescens
PB1_gene_1524	322	ORM85044.1	322	317/322(98.45)	9.55e-234	hypothetical protein HA42_03215 [Pantoea deleyi]	470932	d__Bacteria;k__norank;p__Proteobacteria;c__Gammaproteobacteria;o__Enterobacterales;f__Erwiniaceae;g__Pantoea;s__Pantoea_deleyi
PB1_gene_1529	526	ORM86863.1	526	523/526(99.43)	0	hypothetical protein HA42_00375 [Pantoea deleyi]	470932	d__Bacteria;k__norank;p__Proteobacteria;c__Gammaproteobacteria;o__Enterobacterales;f__Erwiniaceae;g__Pantoea;s__Pantoea_deleyi
PB1_gene_1532	343	WP_111142055.1	343	339/343(98.83)	1.33e-246	arginine N-succinyltransferase [Pantoea sp. ARC607]	2027922	d__Bacteria;k__norank;p__Proteobacteria;c__Gammaproteobacteria;o__Enterobacterales;f__Erwiniaceae;g__Pantoea;s__Pantoea_sp._ARC607
PB1_gene_1533	406	ORM75854.1	406	401/406(98.77)	8.73e-293	acetylornithine aminotransferase [Pantoea deleyi]	470932	d__Bacteria;k__norank;p__Proteobacteria;c__Gammaproteobacteria;o__Enterobacterales;f__Erwiniaceae;g__Pantoea;s__Pantoea_deleyi
PB1_gene_1536	317	WP_111139660.1	299	297/299(99.33)	5.81e-219	phosphatidylserine decarboxylase [Pantoea sp. ARC607]	2027922	d__Bacteria;k__norank;p__Proteobacteria;c__Gammaproteobacteria;o__Enterobacterales;f__Erwiniaceae;g__Pantoea;s__Pantoea_sp._ARC607
PB1_gene_1537	306	ORM86122.1	306	306/306(100.00)	7.25e-205	oligopeptide transporter permease [Pantoea deleyi]	470932	d__Bacteria;k__norank;p__Proteobacteria;c__Gammaproteobacteria;o__Enterobacterales;f__Erwiniaceae;g__Pantoea;s__Pantoea_deleyi
PB1_gene_1538	301	WP_033732832.1	302	300/301(99.67)	2.43e-203	oligopeptide ABC transporter permease OppC [Pantoea vagans]	470934	d__Bacteria;k__norank;p__Proteobacteria;c__Gammaproteobacteria;o__Enterobacterales;f__Erwiniaceae;g__Pantoea;s__Pantoea_vagans
PB1_gene_1539	337	WP_033732831.1	337	336/337(99.70)	1.48e-240	ABC transporter ATP-binding protein [Pantoea vagans]	53335	d__Bacteria;k__norank;p__Proteobacteria;c__Gammaproteobacteria;o__Enterobacterales;f__Erwiniaceae;g__Pantoea;s__norank
PB1_gene_1542	276	ORM84466.1	276	268/276(97.10)	1.49e-207	glycosyl transferase [Pantoea deleyi]	470932	d__Bacteria;k__norank;p__Proteobacteria;c__Gammaproteobacteria;o__Enterobacterales;f__Erwiniaceae;g__Pantoea;s__Pantoea_deleyi

PB1_gene_1545	275	ORM82202.1	275	269/275(97.82)	5.38e-205	3',5'-cyclic-AMP phosphodiesterase [Pantoea	470932	d__Bacteria;k__norank;p__Proteobacteria;c__Gammaproteobacteria;o__Enterobacterales;f__Erwiniaceae;g__Pantoea;s__Pantoea_deleyi
PB1_gene_1546	193	ORM82201.1	193	192/193(99.48)	1.18e-140	esterase YqiA [Pantoea deleyi]	470932	d__Bacteria;k__norank;p__Proteobacteria;c__Gammaproteobacteria;o__Enterobacterales;f__Erwiniaceae;g__Pantoea;s__Pantoea_deleyi
PB1_gene_1550	198	ORM81342.1	198	192/198(96.97)	6.27e-134	hypothetical protein HA42_11105 [Pantoea deleyi]	470932	d__Bacteria;k__norank;p__Proteobacteria;c__Gammaproteobacteria;o__Enterobacterales;f__Erwiniaceae;g__Pantoea;s__Pantoea_deleyi
PB1_gene_1551	166	ORM81343.1	166	164/166(98.80)	4.99e-113	type VI secretion system-associated lipoprotein [Pantoea	470932	d__Bacteria;k__norank;p__Proteobacteria;c__Gammaproteobacteria;o__Enterobacterales;f__Erwiniaceae;g__Pantoea;s__Pantoea_deleyi
PB1_gene_1552	448	ORM81348.1	446	445/446(99.78)	0	type VI secretion system-associated protein [Pantoea	470932	d__Bacteria;k__norank;p__Proteobacteria;c__Gammaproteobacteria;o__Enterobacterales;f__Erwiniaceae;g__Pantoea;s__Pantoea_deleyi
PB1_gene_1557	86	ORM78922.1	86	79/86(91.86)	1.48e-49	hypothetical protein HA42_16170 [Pantoea deleyi]	470932	d__Bacteria;k__norank;p__Proteobacteria;c__Gammaproteobacteria;o__Enterobacterales;f__Erwiniaceae;g__Pantoea;s__Pantoea_deleyi
PB1_gene_1558	218	ORM78923.1	218	216/218(99.08)	1.69e-141	methyltransferase [Pantoea deleyi]	470932	d__Bacteria;k__norank;p__Proteobacteria;c__Gammaproteobacteria;o__Enterobacterales;f__Erwiniaceae;g__Pantoea;s__Pantoea_deleyi
PB1_gene_1559	105	WP_111139493.1	91	90/91(98.90)	1.65e-43	DUF333 domain-containing protein [Pantoea sp. ARC607]	2027922	d__Bacteria;k__norank;p__Proteobacteria;c__Gammaproteobacteria;o__Enterobacterales;f__Erwiniaceae;g__Pantoea;s__Pantoea_sp._ARC607
PB1_gene_1562	334	ADO07899.1	334	323/334(96.71)	1.44e-225	putative sugar transferase (plasmid) [Pantoea vagans C9-1]	712898	d__Bacteria;k__norank;p__Proteobacteria;c__Gammaproteobacteria;o__Enterobacterales;f__Erwiniaceae;g__Pantoea;s__Pantoea_vagans
PB1_gene_1563	378	ORM78175.1	378	368/378(97.35)	2.79e-268	UDP-4-amino-4-deoxy-L-arabinose--oxoglutarate aminotransferase [Pantoea deleyi]	470932	d__Bacteria;k__norank;p__Proteobacteria;c__Gammaproteobacteria;o__Enterobacterales;f__Erwiniaceae;g__Pantoea;s__Pantoea_deleyi
PB1_gene_1564	536	ORM80625.1	536	536/536(100.00)	0	peptide ABC transporter substrate-binding protein	470932	d__Bacteria;k__norank;p__Proteobacteria;c__Gammaproteobacteria;o__Enterobacterales;f__Erwiniaceae;g__Pantoea;s__Pantoea_deleyi
PB1_gene_1569	348	WP_033732812.1	348	334/348(95.98)	2.31e-229	protease SohB [Pantoea vagans]	470934	d__Bacteria;k__norank;p__Proteobacteria;c__Gammaproteobacteria;o__Enterobacterales;f__Erwiniaceae;g__Pantoea;s__Pantoea_vagans
PB1_gene_1570	82	ORM86095.1	82	79/82(96.34)	4.55e-49	hypothetical protein HA42_01260 [Pantoea deleyi]	470932	d__Bacteria;k__norank;p__Proteobacteria;c__Gammaproteobacteria;o__Enterobacterales;f__Erwiniaceae;g__Pantoea;s__Pantoea_deleyi
PB1_gene_1573	75	WP_111139623.1	75	72/75(96.00)	1.09e-46	hypothetical protein [Pantoea sp. ARC607]	2027922	d__Bacteria;k__norank;p__Proteobacteria;c__Gammaproteobacteria;o__Enterobacterales;f__Erwiniaceae;g__Pantoea;s__Pantoea_sp._ARC607
PB1_gene_1574	207	ORM76797.1	176	169/176(96.02)	1.35e-114	hypothetical protein HA42_18665 [Pantoea deleyi]	470932	d__Bacteria;k__norank;p__Proteobacteria;c__Gammaproteobacteria;o__Enterobacterales;f__Erwiniaceae;g__Pantoea;s__Pantoea_deleyi
PB1_gene_1575	302	WP_111139621.1	302	300/302(99.34)	1.42e-209	polyprenyl synthetase family protein [Pantoea sp. ARC607]	2027922	d__Bacteria;k__norank;p__Proteobacteria;c__Gammaproteobacteria;o__Enterobacterales;f__Erwiniaceae;g__Pantoea;s__Pantoea_sp._ARC607
PB1_gene_1579	310	CAQ54569.1	303	282/308(91.56)	1.28e-180	hypothetical protein WP0461 [Wolbachia endosymbiont of Culex quinquefasciatus Pel]	570417	d__Bacteria;k__norank;p__Proteobacteria;c__Alphaproteobacteria;o__Rickettsiales;f__Anaplasmataceae;g__Wolbachia;s__Wolbachia_endosymbiont_of_Culex_quinquefasciatus
PB1_gene_1583	784	ORM83617.1	784	765/784(97.58)	0	transcriptional regulator [Pantoea deleyi]	470932	d__Bacteria;k__norank;p__Proteobacteria;c__Gammaproteobacteria;o__Enterobacterales;f__Erwiniaceae;g__Pantoea;s__Pantoea_deleyi
PB1_gene_1585	621	WP_111140000.1	621	618/621(99.52)	0	1-deoxy-D-xylulose-5-phosphate synthase [Pantoea sp. ARC607]	2027922	d__Bacteria;k__norank;p__Proteobacteria;c__Gammaproteobacteria;o__Enterobacterales;f__Erwiniaceae;g__Pantoea;s__Pantoea_sp._ARC607
PB1_gene_1586	324	ORM83158.1	324	315/324(97.22)	1.82e-223	oxidoreductase [Pantoea deleyi]	470932	d__Bacteria;k__norank;p__Proteobacteria;c__Gammaproteobacteria;o__Enterobacterales;f__Erwiniaceae;g__Pantoea;s__Pantoea_deleyi
PB1_gene_1589	246	WP_111141226.1	243	240/243(98.77)	1.40e-169	arginine ABC transporter substrate-binding protein [Pantoea sp. ARC607]	2027922	d__Bacteria;k__norank;p__Proteobacteria;c__Gammaproteobacteria;o__Enterobacterales;f__Erwiniaceae;g__Pantoea;s__Pantoea_sp._ARC607
PB1_gene_1590	275	KGD81584.1	259	255/258(98.84)	9.62e-177	arginine ABC transporter ATP-binding protein [Pantoea agglomerans]	549	d__Bacteria;k__norank;p__Proteobacteria;c__Gammaproteobacteria;o__Enterobacterales;f__Erwiniaceae;g__Pantoea;s__Pantoea_agglomerans
PB1_gene_1591	222	ORM81969.1	179	174/179(97.21)	3.23e-122	lipoprotein [Pantoea deleyi]	470932	d__Bacteria;k__norank;p__Proteobacteria;c__Gammaproteobacteria;o__Enterobacterales;f__Erwiniaceae;g__Pantoea;s__Pantoea_deleyi

PB1_gene_1594	296	ORM76580.1	296	296/296(100.00)	4.36e-201	antimicrobial peptide ABC transporter permease SapC [Pantoea deleyi]	470932	d__Bacteria;k__norank;p__Proteobacteria;c__Gammaproteobacteria;o__Enterobacterales;f__Erwiniaceae;g__Pantoea;s__Pantoea_deleyi
PB1_gene_1599	94	WP_120458417.1	96	41/75(54.67)	3.00e-21	hypothetical protein [Erwiniaceae bacterium IIIF1SW-P2]	2364647	d__Bacteria;k__norank;p__Proteobacteria;c__Gammaproteobacteria;o__Enterobacterales;f__Erwiniaceae;g__Kalamiella;s__Kalamiella_piersonii
PB1_gene_1600	200	OAD98032.1	173	121/176(68.75)	1.76e-68	hypothetical protein A6A26_24115 (plasmid)	1835724	d__Bacteria;k__norank;p__Proteobacteria;c__Gammaproteobacteria;o__Enterobacterales;f__Erwiniaceae;g__Pantoea;s__Pantoea_sp._OXWO6B1
PB1_gene_1602	328	ORM80622.1	328	327/328(99.70)	5.10e-232	dipeptide ABC transporter ATP-binding protein [Pantoea deleyi]	470932	d__Bacteria;k__norank;p__Proteobacteria;c__Gammaproteobacteria;o__Enterobacterales;f__Erwiniaceae;g__Pantoea;s__Pantoea_deleyi
PB1_gene_1603	300	ORM80623.1	300	299/300(99.67)	1.74e-193	dipeptide ABC transporter permease DppC [Pantoea deleyi]	470932	d__Bacteria;k__norank;p__Proteobacteria;c__Gammaproteobacteria;o__Enterobacterales;f__Erwiniaceae;g__Pantoea;s__Pantoea_deleyi
PB1_gene_1607	57	ORM84498.1	78	57/57(100.00)	4.27e-33	50S ribosomal protein L28 [Pantoea deleyi]	470932	d__Bacteria;k__norank;p__Proteobacteria;c__Gammaproteobacteria;o__Enterobacterales;f__Erwiniaceae;g__Pantoea;s__Pantoea_deleyi
PB1_gene_1608	218	WP_111141477.1	218	214/218(98.17)	1.91e-147	JAB domain-containing protein [Pantoea sp. ARC607]	2027922	d__Bacteria;k__norank;p__Proteobacteria;c__Gammaproteobacteria;o__Enterobacterales;f__Erwiniaceae;g__Pantoea;s__Pantoea_sp._ARC607
PB1_gene_1616	370	ORM85094.1	370	346/370(93.51)	3.44e-226	6-phosphogluconolactonase [Pantoea deleyi]	470932	d__Bacteria;k__norank;p__Proteobacteria;c__Gammaproteobacteria;o__Enterobacterales;f__Erwiniaceae;g__Pantoea;s__Pantoea_deleyi
PB1_gene_1617	469	ORM85095.1	469	468/469(99.79)	0	FMN/FAD transporter [Pantoea deleyi]	470932	d__Bacteria;k__norank;p__Proteobacteria;c__Gammaproteobacteria;o__Enterobacterales;f__Erwiniaceae;g__Pantoea;s__Pantoea_deleyi
PB1_gene_1619	59	ERM09161.1	658	38/59(64.41)	4.41e-18	DNA ligase [Pantoea agglomerans Tx10]	1332072	d__Bacteria;k__norank;p__Proteobacteria;c__Gammaproteobacteria;o__Enterobacterales;f__Erwiniaceae;g__Pantoea;s__Pantoea_agglomerans
PB1_gene_1620	638	WP_111141261.1	583	528/583(90.57)	0	NAD-dependent DNA ligase LigB [Pantoea sp. ARC607]	2027922	d__Bacteria;k__norank;p__Proteobacteria;c__Gammaproteobacteria;o__Enterobacterales;f__Erwiniaceae;g__Pantoea;s__Pantoea_sp._ARC607
PB1_gene_1623	342	ORM84512.1	342	337/342(98.54)	4.34e-256	L-threonine 3-dehydrogenase [Pantoea deleyi]	470932	d__Bacteria;k__norank;p__Proteobacteria;c__Gammaproteobacteria;o__Enterobacterales;f__Erwiniaceae;g__Pantoea;s__Pantoea_deleyi
PB1_gene_1624	303	WP_098052016.1	303	287/302(95.03)	1.54e-209	hypothetical protein [Pantoea agglomerans]	None	Unclassified
PB1_gene_1626	152	ORM82723.1	152	152/152(100.00)	4.23e-105	deoxyuridine 5'-triphosphate nucleotidohydrolase [Pantoea deleyi]	470932	d__Bacteria;k__norank;p__Proteobacteria;c__Gammaproteobacteria;o__Enterobacterales;f__Erwiniaceae;g__Pantoea;s__Pantoea_deleyi
PB1_gene_1627	198	ORM82724.1	198	197/198(99.49)	5.53e-128	nucleoid occlusion factor SlmA [Pantoea deleyi]	470932	d__Bacteria;k__norank;p__Proteobacteria;c__Gammaproteobacteria;o__Enterobacterales;f__Erwiniaceae;g__Pantoea;s__Pantoea_deleyi
PB1_gene_1628	213	ORM82725.1	213	213/213(100.00)	2.86e-151	orotate phosphoribosyltransferase [Pantoea deleyi]	470932	d__Bacteria;k__norank;p__Proteobacteria;c__Gammaproteobacteria;o__Enterobacterales;f__Erwiniaceae;g__Pantoea;s__Pantoea_deleyi
PB1_gene_1629	238	ORM82726.1	238	237/238(99.58)	2.05e-161	ribonuclease PH [Pantoea deleyi]	470932	d__Bacteria;k__norank;p__Proteobacteria;c__Gammaproteobacteria;o__Enterobacterales;f__Erwiniaceae;g__Pantoea;s__Pantoea_deleyi
PB1_gene_1630	86	KDA96513.1	41	32/35(91.43)	3.70e-13	hypothetical protein T296_00190 [Pantoea agglomerans Eh318]	1408177	d__Bacteria;k__norank;p__Proteobacteria;c__Gammaproteobacteria;o__Enterobacterales;f__Erwiniaceae;g__Pantoea;s__Pantoea_agglomerans
PB1_gene_1633	313	ORM78077.1	313	306/313(97.76)	3.44e-227	glutathione-dependent reductase [Pantoea deleyi]	470932	d__Bacteria;k__norank;p__Proteobacteria;c__Gammaproteobacteria;o__Enterobacterales;f__Erwiniaceae;g__Pantoea;s__Pantoea_deleyi
PB1_gene_1634	293	WP_111139313.1	301	290/293(98.98)	7.50e-207	LysR family transcriptional regulator [Pantoea sp. ARC607]	2027922	d__Bacteria;k__norank;p__Proteobacteria;c__Gammaproteobacteria;o__Enterobacterales;f__Erwiniaceae;g__Pantoea;s__Pantoea_sp._ARC607
PB1_gene_1637	408	ORM79851.1	408	398/408(97.55)	4.15e-292	hypothetical protein HA42_15140 [Pantoea deleyi]	470932	d__Bacteria;k__norank;p__Proteobacteria;c__Gammaproteobacteria;o__Enterobacterales;f__Erwiniaceae;g__Pantoea;s__Pantoea_deleyi
PB1_gene_1639	226	WP_111139550.1	226	220/226(97.35)	5.29e-158	type 1 glutamine amidotransferase domain-containing protein [Pantoea sp.	2027922	d__Bacteria;k__norank;p__Proteobacteria;c__Gammaproteobacteria;o__Enterobacterales;f__Erwiniaceae;g__Pantoea;s__Pantoea_sp._ARC607
PB1_gene_1640	174	WP_111139549.1	174	172/174(98.85)	9.97e-135	phenolic acid decarboxylase [Pantoea sp. ARC607]	2027922	d__Bacteria;k__norank;p__Proteobacteria;c__Gammaproteobacteria;o__Enterobacterales;f__Erwiniaceae;g__Pantoea;s__Pantoea_sp._ARC607
PB1_gene_1643	442	ORM77073.1	442	435/442(98.42)	0	N-succinylarginine dihydrolase [Pantoea deleyi]	470932	d__Bacteria;k__norank;p__Proteobacteria;c__Gammaproteobacteria;o__Enterobacterales;f__Erwiniaceae;g__Pantoea;s__Pantoea_deleyi

PB1_gene_1649	258	ORM85243.1	257	250/257(97.28)	3.55e-174	acetoin reductase [Pantoea deleyi]	470932	d__Bacteria;k__norank;p__Proteobacteria;c__Gammaproteobacteria;o__Enterobacterales;f__Erwiniaceae;g__Pantoea;s__Pantoea_deleyi
PB1_gene_1658	256	ORM80542.1	254	252/254(99.21)	9.52e-180	DNA-binding transcriptional repressor DeoR [Pantoea deleyi]	470932	d__Bacteria;k__norank;p__Proteobacteria;c__Gammaproteobacteria;o__Enterobacterales;f__Erwiniaceae;g__Pantoea;s__Pantoea_deleyi
PB1_gene_1659	552	ABU78569.1	566	355/544(65.26)	9.17e-249	hypothetical protein ESA_03348 [Cronobacter sakazakii ATCC BAA-894]	290339	d__Bacteria;k__norank;p__Proteobacteria;c__Gammaproteobacteria;o__Enterobacterales;f__Enterobacteriaceae;g__Cronobacter;s__Cronobacter_sakazakii
PB1_gene_1660	379	ORM81132.1	379	375/379(98.94)	3.28e-272	cytochrome d ubiquinol oxidase subunit II [Pantoea deleyi]	470932	d__Bacteria;k__norank;p__Proteobacteria;c__Gammaproteobacteria;o__Enterobacterales;f__Erwiniaceae;g__Pantoea;s__Pantoea_deleyi
PB1_gene_1661	523	ORM81131.1	523	517/523(98.85)	0	cytochrome d terminal oxidase subunit 1 [Pantoea deleyi]	470932	d__Bacteria;k__norank;p__Proteobacteria;c__Gammaproteobacteria;o__Enterobacterales;f__Erwiniaceae;g__Pantoea;s__Pantoea_deleyi
PB1_gene_1664	192	WP_111139844.1	192	189/191(98.95)	2.29e-133	DedA family protein [Pantoea sp. ARC607]	2027922	d__Bacteria;k__norank;p__Proteobacteria;c__Gammaproteobacteria;o__Enterobacterales;f__Erwiniaceae;g__Pantoea;s__Pantoea_sp._ARC607
PB1_gene_1668	299	WP_111140422.1	299	291/299(97.32)	1.18e-211	methylenetetrahydrofolate reductase [Pantoea sp. ARC607]	2027922	d__Bacteria;k__norank;p__Proteobacteria;c__Gammaproteobacteria;o__Enterobacterales;f__Erwiniaceae;g__Pantoea;s__Pantoea_sp._ARC607
PB1_gene_1671	79	SKA23729.1	79	79/79(100.00)	2.81e-44	cell division protein ZapB [Pantoea agglomerans]	470933	d__Bacteria;k__norank;p__Proteobacteria;c__Gammaproteobacteria;o__Enterobacterales;f__Erwiniaceae;g__Pantoea;s__Pantoea_eucalypti
PB1_gene_1672	195	SKA23721.1	195	193/195(98.97)	3.13e-137	regulator of ribonuclease activity A [Pantoea agglomerans]	470933	d__Bacteria;k__norank;p__Proteobacteria;c__Gammaproteobacteria;o__Enterobacterales;f__Erwiniaceae;g__Pantoea;s__Pantoea_eucalypti
PB1_gene_1675	228	ORM85848.1	228	227/228(99.56)	8.41e-156	ABC transporter ATP-binding protein [Pantoea deleyi]	470932	d__Bacteria;k__norank;p__Proteobacteria;c__Gammaproteobacteria;o__Enterobacterales;f__Erwiniaceae;g__Pantoea;s__Pantoea_deleyi
PB1_gene_1676	208	ORM85849.1	218	204/208(98.08)	1.43e-144	multifunctional acyl-CoA thioesterase I/protease I/lysophospholipase L1 [Pantoea deleyi]	470932	d__Bacteria;k__norank;p__Proteobacteria;c__Gammaproteobacteria;o__Enterobacterales;f__Erwiniaceae;g__Pantoea;s__Pantoea_deleyi
PB1_gene_1677	264	ORM85869.1	257	253/257(98.44)	2.97e-179	oxidoreductase [Pantoea deleyi]	470932	d__Bacteria;k__norank;p__Proteobacteria;c__Gammaproteobacteria;o__Enterobacterales;f__Erwiniaceae;g__Pantoea;s__Pantoea_deleyi
PB1_gene_1678	143	ADI78473.1	140	111/138(80.43)	8.48e-66	Transcriptional regulator hosA (plasmid) [Pantoea vagans C9-1]	712898	d__Bacteria;k__norank;p__Proteobacteria;c__Gammaproteobacteria;o__Enterobacterales;f__Erwiniaceae;g__Pantoea;s__Pantoea_vagans
PB1_gene_1679	189	WP_058957236.1	189	173/189(91.53)	2.76e-119	UbiX family flavin prenyltransferase [Type-F	1560358	d__Bacteria;k__norank;p__Proteobacteria;c__Gammaproteobacteria;o__Enterobacterales;f__Enterobacteriaceae;g__norank;s__Type-F_symbiont_of_Plautia_stali
PB1_gene_1680	475	WP_058957237.1	475	453/475(95.37)	0	UbiD family decarboxylase [Type-F symbiont of Plautia stali]	1560358	d__Bacteria;k__norank;p__Proteobacteria;c__Gammaproteobacteria;o__Enterobacterales;f__Enterobacteriaceae;g__norank;s__Type-F_symbiont_of_Plautia_stali
PB1_gene_1681	75	WP_058957238.1	75	67/75(89.33)	3.78e-47	hypothetical protein [Type-F symbiont of Plautia stali]	470934	d__Bacteria;k__norank;p__Proteobacteria;c__Gammaproteobacteria;o__Enterobacterales;f__Erwiniaceae;g__Pantoea;s__Pantoea_vagans
PB1_gene_1684	121	ORM81984.1	121	116/121(95.87)	4.65e-76	hypothetical protein HA42_09325 [Pantoea deleyi]	470932	d__Bacteria;k__norank;p__Proteobacteria;c__Gammaproteobacteria;o__Enterobacterales;f__Erwiniaceae;g__Pantoea;s__Pantoea_deleyi
PB1_gene_1685	87	WP_111141213.1	87	86/87(98.85)	1.85e-57	GrxA family glutaredoxin [Pantoea sp. ARC607]	2027922	d__Bacteria;k__norank;p__Proteobacteria;c__Gammaproteobacteria;o__Enterobacterales;f__Erwiniaceae;g__Pantoea;s__Pantoea_sp._ARC607
PB1_gene_1686	83	ORM81982.1	83	80/83(96.39)	1.64e-47	hypothetical protein HA42_09315 [Pantoea deleyi]	470932	d__Bacteria;k__norank;p__Proteobacteria;c__Gammaproteobacteria;o__Enterobacterales;f__Erwiniaceae;g__Pantoea;s__Pantoea_deleyi
PB1_gene_1687	240	WP_111141215.1	240	239/240(99.58)	8.80e-173	oxygen-insensitive NADPH nitroreductase [Pantoea sp.	2027922	d__Bacteria;k__norank;p__Proteobacteria;c__Gammaproteobacteria;o__Enterobacterales;f__Erwiniaceae;g__Pantoea;s__Pantoea_sp._ARC607
PB1_gene_1690	147	WP_111139217.1	147	146/147(99.32)	3.01e-98	HTH-type transcriptional repressor NsrR [Pantoea sp.	2027922	d__Bacteria;k__norank;p__Proteobacteria;c__Gammaproteobacteria;o__Enterobacterales;f__Erwiniaceae;g__Pantoea;s__Pantoea_sp._ARC607
PB1_gene_1692	544	ORM79493.1	544	535/544(98.35)	0	phosphate ABC transporter, permease protein PstA [Pantoea deleyi]	470932	d__Bacteria;k__norank;p__Proteobacteria;c__Gammaproteobacteria;o__Enterobacterales;f__Erwiniaceae;g__Pantoea;s__Pantoea_deleyi
PB1_gene_1699	237	WP_090118238.1	232	140/220(63.64)	1.06e-92	adenylyl cyclase [Kosakonia arachidis]	551989	d__Bacteria;k__norank;p__Proteobacteria;c__Gammaproteobacteria;o__Enterobacterales;f__Enterobacteriaceae;g__Kosakonia;s__Kosakonia_arachidis

PB1_gene_1700	89	ORM77025.1	89	78/89(87.64)	7.50e-49	hypothetical protein HA42_18380 [Pantoea deleyi]	470932	d__Bacteria;k__norank;p__Proteobacteria;c__Gammaproteobacteria;o__Enterobacterales;f__Erwiniaceae;g__Pantoea;s__Pantoea_deleyi
PB1_gene_1703	91	SKA24482.1	91	91/91(100.00)	6.58e-53	DNA-directed RNA polymerase subunit omega [Pantoea agglomerans]	470933	d__Bacteria;k__norank;p__Proteobacteria;c__Gammaproteobacteria;o__Enterobacterales;f__Erwiniaceae;g__Pantoea;s__Pantoea_eucalypti
PB1_gene_1704	702	WP_111141263.1	701	700/702(99.72)	0	bifunctional GTP diphosphokinase/guanosine-3',5'-bis pyrophosphate 3'-pyrophosphohydrolase [Pantoea sp. ARC607]	2027922	d__Bacteria;k__norank;p__Proteobacteria;c__Gammaproteobacteria;o__Enterobacterales;f__Erwiniaceae;g__Pantoea;s__Pantoea_sp._ARC607
PB1_gene_1707	355	ORM77521.1	355	354/355(99.72)	4.81e-246	recombinase RecA [Pantoea deleyi]	470932	d__Bacteria;k__norank;p__Proteobacteria;c__Gammaproteobacteria;o__Enterobacterales;f__Erwiniaceae;g__Pantoea;s__Pantoea_deleyi
PB1_gene_1709	118	AMO47505.1	123	61/92(66.30)	9.80e-34	hypothetical protein AKI40_1084 [Enterobacter sp. FY-07]	1692238	d__Bacteria;k__norank;p__Proteobacteria;c__Gammaproteobacteria;o__Enterobacterales;f__Enterobacteriaceae;g__Enterobacter;s__Enterobacter_sp._FY-07
PB1_gene_1712	237	ORM79157.1	237	236/237(99.58)	1.07e-167	purine-nucleoside phosphorylase [Pantoea deleyi]	470932	d__Bacteria;k__norank;p__Proteobacteria;c__Gammaproteobacteria;o__Enterobacterales;f__Erwiniaceae;g__Pantoea;s__Pantoea_deleyi
PB1_gene_1713	407	WP_111140758.1	407	405/407(99.51)	3.48e-304	phosphopentomutase [Pantoea sp. ARC607]	2027922	d__Bacteria;k__norank;p__Proteobacteria;c__Gammaproteobacteria;o__Enterobacterales;f__Erwiniaceae;g__Pantoea;s__Pantoea_sp._ARC607
PB1_gene_1716	351	ADO10676.1	351	347/351(98.86)	1.95e-243	Peptide chain release factor 2 (RF-2) [Pantoea vagans C9-1]	712898	d__Bacteria;k__norank;p__Proteobacteria;c__Gammaproteobacteria;o__Enterobacterales;f__Erwiniaceae;g__Pantoea;s__Pantoea_vagans
PB1_gene_1717	506	ORM86570.1	506	504/506(99.60)	0	lysine--tRNA ligase [Pantoea deleyi]	470932	d__Bacteria;k__norank;p__Proteobacteria;c__Gammaproteobacteria;o__Enterobacterales;f__Erwiniaceae;g__Pantoea;s__Pantoea_deleyi
PB1_gene_1719	575	WP_111139625.1	575	555/575(96.52)	0	DUF2534 family protein [Pantoea sp. ARC607]	2027922	d__Bacteria;k__norank;p__Proteobacteria;c__Gammaproteobacteria;o__Enterobacterales;f__Erwiniaceae;g__Pantoea;s__Pantoea_sp._ARC607
PB1_gene_1720	135	WP_128086809.1	136	126/136(92.65)	2.75e-84	DUF2946 domain-containing protein [Pantoea deleyi]	None	Unclassified
PB1_gene_1723	377	ORM81036.1	377	372/376(98.94)	2.93e-274	glucan biosynthesis protein C [Pantoea deleyi]	470932	d__Bacteria;k__norank;p__Proteobacteria;c__Gammaproteobacteria;o__Enterobacterales;f__Erwiniaceae;g__Pantoea;s__Pantoea_deleyi
PB1_gene_1725	412	ORM76965.1	411	409/411(99.51)	1.09e-307	aminotransferase V [Pantoea deleyi]	470932	d__Bacteria;k__norank;p__Proteobacteria;c__Gammaproteobacteria;o__Enterobacterales;f__Erwiniaceae;g__Pantoea;s__Pantoea_deleyi
PB1_gene_1728	120	SKA90121.1	120	120/120(100.00)	1.18e-64	large subunit ribosomal protein L7/L12 [Pantoea ananatis]	553	d__Bacteria;k__norank;p__Proteobacteria;c__Gammaproteobacteria;o__Enterobacterales;f__Erwiniaceae;g__Pantoea;s__Pantoea_ananatis
PB1_gene_1729	229	CSP90756.1	189	144/190(75.79)	1.16e-96	Uncharacterised protein [Shigella sonnei]	624	d__Bacteria;k__norank;p__Proteobacteria;c__Gammaproteobacteria;o__Enterobacterales;f__Enterobacteriaceae;g__Shigella;s__Shigella_sonnei
PB1_gene_1734	549	ORM75772.1	549	548/549(99.82)	0	Na+/H+ antiporter [Pantoea deleyi]	470932	d__Bacteria;k__norank;p__Proteobacteria;c__Gammaproteobacteria;o__Enterobacterales;f__Erwiniaceae;g__Pantoea;s__Pantoea_deleyi
PB1_gene_1736	727	WP_111140385.1	727	716/727(98.49)	0	1,4-alpha-glucan branching enzyme [Pantoea sp. ARC607]	2027922	d__Bacteria;k__norank;p__Proteobacteria;c__Gammaproteobacteria;o__Enterobacterales;f__Erwiniaceae;g__Pantoea;s__Pantoea_sp._ARC607
PB1_gene_1739	472	WP_111140861.1	471	467/471(99.15)	0	sodium:solute symporter family protein [Pantoea sp. ARC607]	2027922	d__Bacteria;k__norank;p__Proteobacteria;c__Gammaproteobacteria;o__Enterobacterales;f__Erwiniaceae;g__Pantoea;s__Pantoea_sp._ARC607
PB1_gene_1741	168	WP_010082221.1	397	164/168(97.62)	5.87e-111	hypothetical protein [Wolbachia endosymbiont of Drosophila simulans]	953	d__Bacteria;k__norank;p__Proteobacteria;c__Alphaproteobacteria;o__Rickettsiales;f__Anaplasmataceae;g__Wolbachia;s__norank
PB1_gene_1744	369	ORM80109.1	369	364/369(98.64)	1.96e-268	aspartate-semialdehyde dehydrogenase [Pantoea deleyi]	470932	d__Bacteria;k__norank;p__Proteobacteria;c__Gammaproteobacteria;o__Enterobacterales;f__Erwiniaceae;g__Pantoea;s__Pantoea_deleyi
PB1_gene_1745	197	OQV39183.1	197	197/197(100.00)	2.61e-121	antibiotic transporter [Pantoea vagans]	470934	d__Bacteria;k__norank;p__Proteobacteria;c__Gammaproteobacteria;o__Enterobacterales;f__Erwiniaceae;g__Pantoea;s__Pantoea_vagans
PB1_gene_1746	230	WP_089561845.1	230	206/229(89.96)	3.09e-143	MULTISPECIES: response regulator [Pantoea]	53335	d__Bacteria;k__norank;p__Proteobacteria;c__Gammaproteobacteria;o__Enterobacterales;f__Erwiniaceae;g__Pantoea;s__norank

PB1_gene_1749	337	SJZ62637.1	337	332/337(98.52)	4.82e-246	UDP-glucose 4-epimerase [Pantoea agglomerans]	470933	d__Bacteria;k__norank;p__Proteobacteria;c__Gammaproteobacteria;o__Enterobacterales;f__Erwiniaceae;g__Pantoea;s__Pantoea_eucalypti
PB1_gene_1757	77	ORM81311.1	77	76/77(98.70)	2.05e-54	hypothetical protein HA42_11220 [Pantoea deleyi]	470932	d__Bacteria;k__norank;p__Proteobacteria;c__Gammaproteobacteria;o__Enterobacterales;f__Erwiniaceae;g__Pantoea;s__Pantoea_deleyi
PB1_gene_1758	482	ORM81312.1	488	481/482(99.79)	0	UDP-phosphate galactose phosphotransferase [Pantoea	470932	d__Bacteria;k__norank;p__Proteobacteria;c__Gammaproteobacteria;o__Enterobacterales;f__Erwiniaceae;g__Pantoea;s__Pantoea_deleyi
PB1_gene_1760	168	WP_128086884.1	183	147/167(88.02)	2.77e-103	AAA family ATPase [Pantoea deleyi]	470932	d__Bacteria;k__norank;p__Proteobacteria;c__Gammaproteobacteria;o__Enterobacterales;f__Erwiniaceae;g__Pantoea;s__Pantoea_deleyi
PB1_gene_1761	209	WP_111142133.1	209	199/209(95.22)	3.81e-129	LysE family translocator [Pantoea sp. ARC607]	2027922	d__Bacteria;k__norank;p__Proteobacteria;c__Gammaproteobacteria;o__Enterobacterales;f__Erwiniaceae;g__Pantoea;s__Pantoea_sp._ARC607
PB1_gene_1762	343	ORM72658.1	343	341/343(99.42)	1.45e-250	5-methyltetrahydropteroyltriglutamate--homocysteine methyltransferase [Pantoea	470932	d__Bacteria;k__norank;p__Proteobacteria;c__Gammaproteobacteria;o__Enterobacterales;f__Erwiniaceae;g__Pantoea;s__Pantoea_deleyi
PB1_gene_1765	255	ORM85303.1	254	254/254(100.00)	5.22e-170	transcriptional regulator PdhR [Pantoea deleyi]	470932	d__Bacteria;k__norank;p__Proteobacteria;c__Gammaproteobacteria;o__Enterobacterales;f__Erwiniaceae;g__Pantoea;s__Pantoea_deleyi
PB1_gene_1766	68	WP_128084281.1	68	64/68(94.12)	3.21e-36	hypothetical protein [Pantoea deleyi]	470932	d__Bacteria;k__norank;p__Proteobacteria;c__Gammaproteobacteria;o__Enterobacterales;f__Erwiniaceae;g__Pantoea;s__Pantoea_deleyi
PB1_gene_1767	451	ORM85302.1	451	443/451(98.23)	3.72e-315	aromatic amino acid transporter AroP [Pantoea deleyi]	470932	d__Bacteria;k__norank;p__Proteobacteria;c__Gammaproteobacteria;o__Enterobacterales;f__Erwiniaceae;g__Pantoea;s__Pantoea_deleyi
PB1_gene_1769	132	ORM84120.1	132	123/132(93.18)	1.14e-88	germin [Pantoea deleyi]	470932	d__Bacteria;k__norank;p__Proteobacteria;c__Gammaproteobacteria;o__Enterobacterales;f__Erwiniaceae;g__Pantoea;s__Pantoea_deleyi
PB1_gene_1770	338	ORM84160.1	338	331/337(98.22)	1.87e-239	transcriptional regulator [Pantoea deleyi]	470932	d__Bacteria;k__norank;p__Proteobacteria;c__Gammaproteobacteria;o__Enterobacterales;f__Erwiniaceae;g__Pantoea;s__Pantoea_deleyi
PB1_gene_1771	64	ORM84121.1	64	62/64(96.88)	2.43e-36	hypothetical protein HA42_05015 [Pantoea deleyi]	470932	d__Bacteria;k__norank;p__Proteobacteria;c__Gammaproteobacteria;o__Enterobacterales;f__Erwiniaceae;g__Pantoea;s__Pantoea_deleyi
PB1_gene_1783	271	ORM75395.1	271	259/271(95.57)	2.95e-191	xylose isomerase [Pantoea deleyi]	470932	d__Bacteria;k__norank;p__Proteobacteria;c__Gammaproteobacteria;o__Enterobacterales;f__Erwiniaceae;g__Pantoea;s__Pantoea_deleyi
PB1_gene_1784	125	WP_111142119.1	125	125/125(100.00)	3.62e-84	fluoride efflux transporter CrcB [Pantoea sp. ARC607]	2027922	d__Bacteria;k__norank;p__Proteobacteria;c__Gammaproteobacteria;o__Enterobacterales;f__Erwiniaceae;g__Pantoea;s__Pantoea_sp._ARC607
PB1_gene_1785	306	ORM75397.1	307	293/304(96.38)	5.53e-207	nucleoside hydrolase [Pantoea deleyi]	470932	d__Bacteria;k__norank;p__Proteobacteria;c__Gammaproteobacteria;o__Enterobacterales;f__Erwiniaceae;g__Pantoea;s__Pantoea_deleyi
PB1_gene_1787	457	WP_111139169.1	457	456/457(99.78)	1.13e-268	exodeoxyribonuclease VII large subunit [Pantoea sp. ARC607]	2027922	d__Bacteria;k__norank;p__Proteobacteria;c__Gammaproteobacteria;o__Enterobacterales;f__Erwiniaceae;g__Pantoea;s__Pantoea_sp._ARC607
PB1_gene_1788	337	WP_111139158.1	337	329/337(97.63)	4.48e-251	peptidase M4 family protein [Pantoea sp. ARC607]	2027922	d__Bacteria;k__norank;p__Proteobacteria;c__Gammaproteobacteria;o__Enterobacterales;f__Erwiniaceae;g__Pantoea;s__Pantoea_sp._ARC607
PB1_gene_1791	317	WP_090963454.1	317	310/317(97.79)	3.78e-242	allantoinase PuuE [Pantoea sp. OV426]	1881036	d__Bacteria;k__norank;p__Proteobacteria;c__Gammaproteobacteria;o__Enterobacterales;f__Erwiniaceae;g__Pantoea;s__Pantoea_sp._OV426
PB1_gene_1792	126	OQV38455.1	126	123/126(97.62)	1.36e-84	DUF4440 domain-containing protein [Pantoea vagans]	470934	d__Bacteria;k__norank;p__Proteobacteria;c__Gammaproteobacteria;o__Enterobacterales;f__Erwiniaceae;g__Pantoea;s__Pantoea_vagans
PB1_gene_1795	319	ORM82818.1	319	318/319(99.69)	3.40e-221	flagellar rod assembly protein/muramidase FlgJ [Pantoea deleyi]	470932	d__Bacteria;k__norank;p__Proteobacteria;c__Gammaproteobacteria;o__Enterobacterales;f__Erwiniaceae;g__Pantoea;s__Pantoea_deleyi
PB1_gene_1798	400	WP_111140466.1	400	396/400(99.00)	2.78e-286	acetate kinase [Pantoea sp. ARC607]	2027922	d__Bacteria;k__norank;p__Proteobacteria;c__Gammaproteobacteria;o__Enterobacterales;f__Erwiniaceae;g__Pantoea;s__Pantoea_sp._ARC607
PB1_gene_1804	480	ORM80979.1	488	459/477(96.23)	0	amidohydrolase [Pantoea deleyi]	470932	d__Bacteria;k__norank;p__Proteobacteria;c__Gammaproteobacteria;o__Enterobacterales;f__Erwiniaceae;g__Pantoea;s__Pantoea_deleyi
PB1_gene_1806	72	SSL82769.1	111	38/66(57.58)	2.76e-16	Uncharacterised protein [Klebsiella pneumoniae]	573	d__Bacteria;k__norank;p__Proteobacteria;c__Gammaproteobacteria;o__Enterobacterales;f__Enterobacteriaceae;g__Klebsiella;s__Klebsiella_pneumoniae

PB1_gene_1808	347	ORM85463.1	347	344/347(99.14)	1.01e-243	phosphate ABC transporter substrate-binding protein PstS [Pantoea deleyi]	470932	d__Bacteria;k__norank;p__Proteobacteria;c__Gammaproteobacteria;o__Enterobacterales;f__Erwiniaceae;g__Pantoea;s__Pantoea_deleyi
PB1_gene_1811	207	ORM83722.1	207	203/207(98.07)	2.27e-145	lipoprotein localization factor LolB [Pantoea deleyi]	470932	d__Bacteria;k__norank;p__Proteobacteria;c__Gammaproteobacteria;o__Enterobacterales;f__Erwiniaceae;g__Pantoea;s__Pantoea_deleyi
PB1_gene_1812	418	ORM83721.1	418	416/418(99.52)	1.88e-284	glutamyl-tRNA reductase [Pantoea deleyi]	470932	d__Bacteria;k__norank;p__Proteobacteria;c__Gammaproteobacteria;o__Enterobacterales;f__Erwiniaceae;g__Pantoea;s__Pantoea_deleyi
PB1_gene_1816	256	ORM75325.1	256	248/256(96.88)	7.75e-175	molybdopterin-synthase adenylyltransferase MoeB [Pantoea deleyi]	470932	d__Bacteria;k__norank;p__Proteobacteria;c__Gammaproteobacteria;o__Enterobacterales;f__Erwiniaceae;g__Pantoea;s__Pantoea_deleyi
PB1_gene_1817	411	ORM75324.1	411	404/411(98.30)	1.32e-285	molybdopterin molybdotransferase [Pantoea	470932	d__Bacteria;k__norank;p__Proteobacteria;c__Gammaproteobacteria;o__Enterobacterales;f__Erwiniaceae;g__Pantoea;s__Pantoea_deleyi
PB1_gene_1820	106	WP_111138739.1	106	103/106(97.17)	1.13e-69	hypothetical protein [Pantoea sp. ARC607]	2027922	d__Bacteria;k__norank;p__Proteobacteria;c__Gammaproteobacteria;o__Enterobacterales;f__Erwiniaceae;g__Pantoea;s__Pantoea_sp._ARC607
PB1_gene_1821	534	WP_111138740.1	534	504/534(94.38)	0	microcin C ABC transporter ATP-binding protein YejF [Pantoea sp. ARC607]	2027922	d__Bacteria;k__norank;p__Proteobacteria;c__Gammaproteobacteria;o__Enterobacterales;f__Erwiniaceae;g__Pantoea;s__Pantoea_sp._ARC607
PB1_gene_1823	479	ORM80463.1	477	454/477(95.18)	0	GntR family transcriptional regulator [Pantoea deleyi]	470932	d__Bacteria;k__norank;p__Proteobacteria;c__Gammaproteobacteria;o__Enterobacterales;f__Erwiniaceae;g__Pantoea;s__Pantoea_deleyi
PB1_gene_1824	406	ORM80462.1	406	406/406(100.00)	1.19e-290	beta-ketoacyl-[acyl-carrier-protein] synthase I [Pantoea	470932	d__Bacteria;k__norank;p__Proteobacteria;c__Gammaproteobacteria;o__Enterobacterales;f__Erwiniaceae;g__Pantoea;s__Pantoea_deleyi
PB1_gene_1825	210	KDA94968.1	210	208/210(99.05)	2.76e-152	superoxide dismutase [Pantoea agglomerans Eh318]	1408177	d__Bacteria;k__norank;p__Proteobacteria;c__Gammaproteobacteria;o__Enterobacterales;f__Erwiniaceae;g__Pantoea;s__Pantoea_agglomerans
PB1_gene_1826	446	WP_111141314.1	446	439/446(98.43)	0	aspartate aminotransferase family protein [Pantoea sp. ARC607]	2027922	d__Bacteria;k__norank;p__Proteobacteria;c__Gammaproteobacteria;o__Enterobacterales;f__Erwiniaceae;g__Pantoea;s__Pantoea_sp._ARC607
PB1_gene_1828	661	ORM86084.1	661	659/661(99.70)	0	cyclic di-GMP phosphodiesterase [Pantoea deleyi]	470932	d__Bacteria;k__norank;p__Proteobacteria;c__Gammaproteobacteria;o__Enterobacterales;f__Erwiniaceae;g__Pantoea;s__Pantoea_deleyi
PB1_gene_1831	229	ORM80874.1	229	228/229(99.56)	1.93e-166	helix-turn-helix transcriptional regulator [Pantoea deleyi]	470932	d__Bacteria;k__norank;p__Proteobacteria;c__Gammaproteobacteria;o__Enterobacterales;f__Erwiniaceae;g__Pantoea;s__Pantoea_deleyi
PB1_gene_1832	136	ORM80875.1	136	135/136(99.26)	3.63e-96	GNAT family N-acetyltransferase [Pantoea deleyi]	470932	d__Bacteria;k__norank;p__Proteobacteria;c__Gammaproteobacteria;o__Enterobacterales;f__Erwiniaceae;g__Pantoea;s__Pantoea_deleyi
PB1_gene_1835	339	ORM86544.1	339	339/339(100.00)	7.67e-244	erythrose-4-phosphate dehydrogenase [Pantoea deleyi]	470932	d__Bacteria;k__norank;p__Proteobacteria;c__Gammaproteobacteria;o__Enterobacterales;f__Erwiniaceae;g__Pantoea;s__Pantoea_deleyi
PB1_gene_1838	638	WP_111142042.1	638	611/638(95.77)	0	ABC transporter ATP-binding protein [Pantoea sp. ARC607]	2027922	d__Bacteria;k__norank;p__Proteobacteria;c__Gammaproteobacteria;o__Enterobacterales;f__Erwiniaceae;g__Pantoea;s__Pantoea_sp._ARC607
PB1_gene_1842	417	WP_111138495.1	417	388/417(93.05)	2.87e-283	serine hydrolase [Pantoea sp. ARC607]	2027922	d__Bacteria;k__norank;p__Proteobacteria;c__Gammaproteobacteria;o__Enterobacterales;f__Erwiniaceae;g__Pantoea;s__Pantoea_sp._ARC607
PB1_gene_1844	299	WP_111140350.1	299	294/299(98.33)	1.32e-208	LysR family transcriptional regulator [Pantoea sp. ARC607]	2027922	d__Bacteria;k__norank;p__Proteobacteria;c__Gammaproteobacteria;o__Enterobacterales;f__Erwiniaceae;g__Pantoea;s__Pantoea_sp._ARC607
PB1_gene_1845	271	WP_128086463.1	269	255/268(95.15)	6.99e-169	extracellular solute-binding protein [Pantoea deleyi]	470932	d__Bacteria;k__norank;p__Proteobacteria;c__Gammaproteobacteria;o__Enterobacterales;f__Erwiniaceae;g__Pantoea;s__Pantoea_deleyi
PB1_gene_1847	63	ORM80730.1	63	63/63(100.00)	8.48e-41	hypothetical protein HA42_13395 [Pantoea deleyi]	470932	d__Bacteria;k__norank;p__Proteobacteria;c__Gammaproteobacteria;o__Enterobacterales;f__Erwiniaceae;g__Pantoea;s__Pantoea_deleyi
PB1_gene_1848	152	SJZ32092.1	152	152/152(100.00)	3.21e-100	iron (metal) dependent repressor, DtxR family [Pantoea	470933	d__Bacteria;k__norank;p__Proteobacteria;c__Gammaproteobacteria;o__Enterobacterales;f__Erwiniaceae;g__Pantoea;s__Pantoea_eucalypti
PB1_gene_1849	369	ORM80732.1	369	364/369(98.64)	1.49e-247	anion transporter [Pantoea deleyi]	470932	d__Bacteria;k__norank;p__Proteobacteria;c__Gammaproteobacteria;o__Enterobacterales;f__Erwiniaceae;g__Pantoea;s__Pantoea_deleyi
PB1_gene_1851	133	CRH39259.1	67	28/28(100.00)	6.59e-09	Uncharacterized protein {ECO:0000313|EMBL:CDH06504.1} [Pantoea ananatis]	553	d__Bacteria;k__norank;p__Proteobacteria;c__Gammaproteobacteria;o__Enterobacterales;f__Erwiniaceae;g__Pantoea;s__Pantoea_ananatis

PB1_gene_1852	262	ORM77229.1	262	261/262(99.62)	1.11e-186	formate/nitrite transporter [Pantoea deleyi]	470932	d__Bacteria;k__norank;p__Proteobacteria;c__Gammaproteobacteria;o__Enterobacterales;f__Erwiniaceae;g__Pantoea;s__Pantoea_deleyi
PB1_gene_1856	273	EFM18018.1	280	252/273(92.31)	1.13e-184	GCN5-related N-acetyltransferase [Pantoea sp. aB]	517433	d__Bacteria;k__norank;p__Proteobacteria;c__Gammaproteobacteria;o__Enterobacterales;f__Erwiniaceae;g__Pantoea;s__Pantoea_sp._aB
PB1_gene_1857	443	PQL29577.1	443	431/443(97.29)	0	FAD-binding oxidoreductase [Pantoea ananatis]	553	d__Bacteria;k__norank;p__Proteobacteria;c__Gammaproteobacteria;o__Enterobacterales;f__Erwiniaceae;g__Pantoea;s__Pantoea_ananatis
PB1_gene_1859	425	WP_111139718.1	425	414/425(97.41)	5.43e-308	cytosine deaminase [Pantoea sp. ARC607]	2027922	d__Bacteria;k__norank;p__Proteobacteria;c__Gammaproteobacteria;o__Enterobacterales;f__Erwiniaceae;g__Pantoea;s__Pantoea_sp._ARC607
PB1_gene_1860	392	ORM81835.1	392	389/392(99.23)	3.38e-267	MFS transporter TsgA [Pantoea deleyi]	470932	d__Bacteria;k__norank;p__Proteobacteria;c__Gammaproteobacteria;o__Enterobacterales;f__Erwiniaceae;g__Pantoea;s__Pantoea_deleyi
PB1_gene_1861	226	WP_090959832.1	228	220/224(98.21)	7.21e-161	cysteine hydrolase [Pantoea sp. OV426]	1881036	d__Bacteria;k__norank;p__Proteobacteria;c__Gammaproteobacteria;o__Enterobacterales;f__Erwiniaceae;g__Pantoea;s__Pantoea_sp._OV426
PB1_gene_1862	434	ORM79068.1	434	428/434(98.62)	4.58e-300	cobalamin biosynthesis protein [Pantoea deleyi]	470932	d__Bacteria;k__norank;p__Proteobacteria;c__Gammaproteobacteria;o__Enterobacterales;f__Erwiniaceae;g__Pantoea;s__Pantoea_deleyi
PB1_gene_1866	198	WP_111142114.1	196	194/196(98.98)	4.46e-138	glucose-1-phosphatase [Pantoea sp. ARC607]	2027922	d__Bacteria;k__norank;p__Proteobacteria;c__Gammaproteobacteria;o__Enterobacterales;f__Erwiniaceae;g__Pantoea;s__Pantoea_sp._ARC607
PB1_gene_1867	289	PEI04339.1	289	286/289(98.96)	3.12e-186	YihY/virulence factor BrkB family protein [Pantoea	549	d__Bacteria;k__norank;p__Proteobacteria;c__Gammaproteobacteria;o__Enterobacterales;f__Erwiniaceae;g__Pantoea;s__Pantoea_agglomerans
PB1_gene_1871	192	WP_111139345.1	192	184/192(95.83)	1.33e-132	GDP-mannose pyrophosphatase NudK [Pantoea sp. ARC607]	2027922	d__Bacteria;k__norank;p__Proteobacteria;c__Gammaproteobacteria;o__Enterobacterales;f__Erwiniaceae;g__Pantoea;s__Pantoea_sp._ARC607
PB1_gene_1873	95	SUB18855.1	389	94/95(98.95)	4.96e-46	Quinoprotein glucose dehydrogenase [Pantoea agglomerans]	549	d__Bacteria;k__norank;p__Proteobacteria;c__Gammaproteobacteria;o__Enterobacterales;f__Erwiniaceae;g__Pantoea;s__Pantoea_agglomerans
PB1_gene_1874	199	WP_111141879.1	219	198/199(99.50)	6.64e-141	hypothetical protein [Pantoea sp. ARC607]	2027922	d__Bacteria;k__norank;p__Proteobacteria;c__Gammaproteobacteria;o__Enterobacterales;f__Erwiniaceae;g__Pantoea;s__Pantoea_sp._ARC607
PB1_gene_1875	413	WP_124889614.1	413	391/413(94.67)	4.15e-274	MFS transporter [Pantoea agglomerans]	549	d__Bacteria;k__norank;p__Proteobacteria;c__Gammaproteobacteria;o__Enterobacterales;f__Erwiniaceae;g__Pantoea;s__Pantoea_agglomerans
PB1_gene_1877	896	WP_111139304.1	891	888/891(99.66)	0	two-component system sensor histidine kinase KdbD [Pantoea sp. ARC607]	53335	d__Bacteria;k__norank;p__Proteobacteria;c__Gammaproteobacteria;o__Enterobacterales;f__Erwiniaceae;g__Pantoea;s__norank
PB1_gene_1885	382	WP_111139463.1	376	374/375(99.73)	1.64e-272	UDP-N-acetylglucosamine 2-epimerase (non-hydrolyzing) [Pantoea sp. ARC607]	2027922	d__Bacteria;k__norank;p__Proteobacteria;c__Gammaproteobacteria;o__Enterobacterales;f__Erwiniaceae;g__Pantoea;s__Pantoea_sp._ARC607
PB1_gene_1887	337	ORM78375.1	337	335/337(99.41)	2.84e-183	hypothetical protein HA42_16805 [Pantoea deleyi]	470932	d__Bacteria;k__norank;p__Proteobacteria;c__Gammaproteobacteria;o__Enterobacterales;f__Erwiniaceae;g__Pantoea;s__Pantoea_deleyi
PB1_gene_1888	267	ORM78374.1	267	267/267(100.00)	2.53e-190	inositol monophosphatase [Pantoea deleyi]	470932	d__Bacteria;k__norank;p__Proteobacteria;c__Gammaproteobacteria;o__Enterobacterales;f__Erwiniaceae;g__Pantoea;s__Pantoea_deleyi
PB1_gene_1889	245	WP_111139140.1	245	243/245(99.18)	1.19e-168	tRNA (cytosine(32)/uridine(32)-2'-O)-methyltransferase TrmJ [Pantoea sp. ARC607]	2027922	d__Bacteria;k__norank;p__Proteobacteria;c__Gammaproteobacteria;o__Enterobacterales;f__Erwiniaceae;g__Pantoea;s__Pantoea_sp._ARC607
PB1_gene_1891	161	PNK70683.1	117	110/117(94.02)	1.61e-72	transcriptional regulator [Pantoea sp. FDAARGOS_194]	1975707	d__Bacteria;k__norank;p__Proteobacteria;c__Gammaproteobacteria;o__Enterobacterales;f__Erwiniaceae;g__Pantoea;s__Pantoea_sp._FDAARGOS_194
PB1_gene_1894	306	ORM52983.1	306	305/306(99.67)	2.52e-230	agmatinase [Pantoea conspicua]	472705	d__Bacteria;k__norank;p__Proteobacteria;c__Gammaproteobacteria;o__Enterobacterales;f__Erwiniaceae;g__Pantoea;s__Pantoea_conspicua
PB1_gene_1896	476	WP_111139886.1	476	472/476(99.16)	0	cell division protein FtsP [Pantoea sp. ARC607]	2027922	d__Bacteria;k__norank;p__Proteobacteria;c__Gammaproteobacteria;o__Enterobacterales;f__Erwiniaceae;g__Pantoea;s__Pantoea_sp._ARC607
PB1_gene_1897	274	ORM82192.1	274	271/274(98.91)	1.08e-199	2,5-didehydrogluconate reductase A [Pantoea deleyi]	470932	d__Bacteria;k__norank;p__Proteobacteria;c__Gammaproteobacteria;o__Enterobacterales;f__Erwiniaceae;g__Pantoea;s__Pantoea_deleyi
PB1_gene_1900	216	ORM80538.1	216	212/216(98.15)	2.92e-141	hypothetical protein HA42_14125 [Pantoea deleyi]	470932	d__Bacteria;k__norank;p__Proteobacteria;c__Gammaproteobacteria;o__Enterobacterales;f__Erwiniaceae;g__Pantoea;s__Pantoea_deleyi

PB1_gene_1901	325	WP_111138476.1	325	319/325(98.15)	6.98e-233	phosphoserine phosphatase [Pantoea sp. ARC607]	2027922	d__Bacteria;k__norank;p__Proteobacteria;c__Gammaproteobacteria;o__Enterobacterales;f__Erwiniaceae;g__Pantoea;s__Pantoea_sp._ARC607
PB1_gene_1906	210	SKA15994.1	210	210/210(100.00)	1.47e-147	CRP/FNR family transcriptional regulator, cyclic AMP receptor protein [Pantoea agglomerans]	470933	d__Bacteria;k__norank;p__Proteobacteria;c__Gammaproteobacteria;o__Enterobacterales;f__Erwiniaceae;g__Pantoea;s__Pantoea_eucalypti
PB1_gene_1907	141	PAW35170.1	134	134/134(100.00)	7.19e-88	hypothetical protein CIL06_05345 [Pantoea vagans]	470934	d__Bacteria;k__norank;p__Proteobacteria;c__Gammaproteobacteria;o__Enterobacterales;f__Erwiniaceae;g__Pantoea;s__Pantoea_vagans
PB1_gene_1910	352	ORM85281.1	352	347/352(98.58)	1.51e-252	undecaprenyldiphospho-muramoylpentapeptide beta-N-acetylglucosaminyltransferase [Pantoea deleyi]	470932	d__Bacteria;k__norank;p__Proteobacteria;c__Gammaproteobacteria;o__Enterobacterales;f__Erwiniaceae;g__Pantoea;s__Pantoea_deleyi
PB1_gene_1911	404	ORM85280.1	404	402/404(99.50)	7.03e-282	cell division protein FtsW [Pantoea deleyi]	470932	d__Bacteria;k__norank;p__Proteobacteria;c__Gammaproteobacteria;o__Enterobacterales;f__Erwiniaceae;g__Pantoea;s__Pantoea_deleyi
PB1_gene_1914	123	WP_110331776.1	123	104/123(84.55)	8.94e-75	hypothetical protein [Pantoea sp. JKS000250]	1938795	d__Bacteria;k__norank;p__Proteobacteria;c__Gammaproteobacteria;o__Enterobacterales;f__Erwiniaceae;g__Pantoea;s__Pantoea_sp._JKS000250
PB1_gene_1915	320	SJZ40819.1	320	316/320(98.75)	7.94e-216	hypothetical protein SAMN03097723_0822 [Pantoea agglomerans]	470933	d__Bacteria;k__norank;p__Proteobacteria;c__Gammaproteobacteria;o__Enterobacterales;f__Erwiniaceae;g__Pantoea;s__Pantoea_eucalypti
PB1_gene_1916	193	EFM19081.1	193	182/193(94.30)	8.43e-116	hypothetical protein PanABDRAFT_2676 [Pantoea sp. aB]	517433	d__Bacteria;k__norank;p__Proteobacteria;c__Gammaproteobacteria;o__Enterobacterales;f__Erwiniaceae;g__Pantoea;s__Pantoea_sp._aB
PB1_gene_1918	508	WP_111142248.1	508	505/508(99.41)	0	exopolyphosphatase [Pantoea sp. ARC607]	2027922	d__Bacteria;k__norank;p__Proteobacteria;c__Gammaproteobacteria;o__Enterobacterales;f__Erwiniaceae;g__Pantoea;s__Pantoea_sp._ARC607
PB1_gene_1919	218	WP_111142249.1	204	201/204(98.53)	1.64e-147	tellurite resistance methyltransferase TehB [Pantoea	2027922	d__Bacteria;k__norank;p__Proteobacteria;c__Gammaproteobacteria;o__Enterobacterales;f__Erwiniaceae;g__Pantoea;s__Pantoea_sp._ARC607
PB1_gene_1920	46	WP_111142250.1	61	46/46(100.00)	1.92e-21	DUF2633 family protein [Pantoea sp. ARC607]	2027922	d__Bacteria;k__norank;p__Proteobacteria;c__Gammaproteobacteria;o__Enterobacterales;f__Erwiniaceae;g__Pantoea;s__Pantoea_sp._ARC607
PB1_gene_1922	115	ORM74632.1	115	113/115(98.26)	5.14e-77	hydroxymyristoyl-ACP dehydratase [Pantoea deleyi]	470932	d__Bacteria;k__norank;p__Proteobacteria;c__Gammaproteobacteria;o__Enterobacterales;f__Erwiniaceae;g__Pantoea;s__Pantoea_deleyi
PB1_gene_1923	437	WP_111138595.1	439	407/437(93.14)	1.83e-308	acyl-CoA synthetase [Pantoea sp. ARC607]	2027922	d__Bacteria;k__norank;p__Proteobacteria;c__Gammaproteobacteria;o__Enterobacterales;f__Erwiniaceae;g__Pantoea;s__Pantoea_sp._ARC607
PB1_gene_1924	141	ORM75564.1	187	137/141(97.16)	1.27e-95	DNA gyrase subunit B [Pantoea deleyi]	470932	d__Bacteria;k__norank;p__Proteobacteria;c__Gammaproteobacteria;o__Enterobacterales;f__Erwiniaceae;g__Pantoea;s__Pantoea_deleyi
PB1_gene_1925	126	AWP34818.1	133	123/126(97.62)	1.16e-91	VOC family protein (plasmid) [Pantoea vagans]	470934	d__Bacteria;k__norank;p__Proteobacteria;c__Gammaproteobacteria;o__Enterobacterales;f__Erwiniaceae;g__Pantoea;s__Pantoea_vagans
PB1_gene_1926	395	WP_010671877.1	402	368/394(93.40)	3.49e-254	MFS transporter [Pantoea agglomerans]	549	d__Bacteria;k__norank;p__Proteobacteria;c__Gammaproteobacteria;o__Enterobacterales;f__Erwiniaceae;g__Pantoea;s__Pantoea_agglomerans
PB1_gene_1929	547	ORM84716.1	547	543/547(99.27)	0	paraquat-inducible protein B [Pantoea deleyi]	470932	d__Bacteria;k__norank;p__Proteobacteria;c__Gammaproteobacteria;o__Enterobacterales;f__Erwiniaceae;g__Pantoea;s__Pantoea_deleyi
PB1_gene_1934	100	WP_111140333.1	100	91/99(91.92)	4.56e-55	TonB-denpendent receptor [Pantoea sp. ARC607]	2027922	d__Bacteria;k__norank;p__Proteobacteria;c__Gammaproteobacteria;o__Enterobacterales;f__Erwiniaceae;g__Pantoea;s__Pantoea_sp._ARC607
PB1_gene_1939	367	WP_111142191.1	367	357/367(97.28)	1.11e-257	amidohydrolase [Pantoea sp. ARC607]	2027922	d__Bacteria;k__norank;p__Proteobacteria;c__Gammaproteobacteria;o__Enterobacterales;f__Erwiniaceae;g__Pantoea;s__Pantoea_sp._ARC607
PB1_gene_1940	158	ORM80919.1	158	153/158(96.84)	8.37e-99	hypothetical protein HA42_12540 [Pantoea deleyi]	470932	d__Bacteria;k__norank;p__Proteobacteria;c__Gammaproteobacteria;o__Enterobacterales;f__Erwiniaceae;g__Pantoea;s__Pantoea_deleyi
PB1_gene_1942	422	ORM75355.1	422	406/422(96.21)	4.93e-298	stationary phase inducible protein CsiE [Pantoea deleyi]	470932	d__Bacteria;k__norank;p__Proteobacteria;c__Gammaproteobacteria;o__Enterobacterales;f__Erwiniaceae;g__Pantoea;s__Pantoea_deleyi
PB1_gene_1943	382	WP_111139134.1	382	368/382(96.34)	2.36e-262	3-phenylpropionate MFS transporter [Pantoea sp. ARC607]	2027922	d__Bacteria;k__norank;p__Proteobacteria;c__Gammaproteobacteria;o__Enterobacterales;f__Erwiniaceae;g__Pantoea;s__Pantoea_sp._ARC607
PB1_gene_1944	584	ORM81378.1	584	580/584(99.32)	0	hypothetical protein HA42_10940 [Pantoea deleyi]	470932	d__Bacteria;k__norank;p__Proteobacteria;c__Gammaproteobacteria;o__Enterobacterales;f__Erwiniaceae;g__Pantoea;s__Pantoea_deleyi

PB1_gene_1945	81	SJZ64845.1	81	79/81(97.53)	3.36e-46	hypothetical protein SAMN03097723_1629 [Pantoea agglomerans]	470933	d__Bacteria;k__norank;p__Proteobacteria;c__Gammaproteobacteria;o__Enterobacterales;f__Erwiniaceae;g__Pantoea;s__Pantoea_eucalypti
PB1_gene_1947	288	ORM79064.1	237	230/237(97.05)	1.71e-159	hypothetical protein HA42_15950 [Pantoea deleyi]	470932	d__Bacteria;k__norank;p__Proteobacteria;c__Gammaproteobacteria;o__Enterobacterales;f__Erwiniaceae;g__Pantoea;s__Pantoea_deleyi
PB1_gene_1948	269	ORM79069.1	268	236/267(88.39)	5.70e-165	hypothetical protein HA42_15945 [Pantoea deleyi]	470932	d__Bacteria;k__norank;p__Proteobacteria;c__Gammaproteobacteria;o__Enterobacterales;f__Erwiniaceae;g__Pantoea;s__Pantoea_deleyi
PB1_gene_1951	337	WP_111140417.1	335	330/337(97.92)	9.26e-163	cell division protein FtsN [Pantoea sp. ARC607]	2027922	d__Bacteria;k__norank;p__Proteobacteria;c__Gammaproteobacteria;o__Enterobacterales;f__Erwiniaceae;g__Pantoea;s__Pantoea_sp._ARC607
PB1_gene_1954	293	WP_111141293.1	293	272/293(92.83)	6.14e-195	LysR family transcriptional regulator [Pantoea sp. ARC607]	2027922	d__Bacteria;k__norank;p__Proteobacteria;c__Gammaproteobacteria;o__Enterobacterales;f__Erwiniaceae;g__Pantoea;s__Pantoea_sp._ARC607
PB1_gene_1955	554	ORM75359.1	554	549/554(99.10)	6.63e-282	chemotaxis protein [Pantoea deleyi]	470932	d__Bacteria;k__norank;p__Proteobacteria;c__Gammaproteobacteria;o__Enterobacterales;f__Erwiniaceae;g__Pantoea;s__Pantoea_deleyi
PB1_gene_1961	438	KEY42032.1	438	418/438(95.43)	2.71e-310	hemL protein [Pantoea agglomerans]	549	d__Bacteria;k__norank;p__Proteobacteria;c__Gammaproteobacteria;o__Enterobacterales;f__Erwiniaceae;g__Pantoea;s__Pantoea_agglomerans
PB1_gene_1962	115	ORM83018.1	115	115/115(100.00)	8.88e-78	iron-sulfur cluster insertion protein ErpA [Pantoea deleyi]	470932	d__Bacteria;k__norank;p__Proteobacteria;c__Gammaproteobacteria;o__Enterobacterales;f__Erwiniaceae;g__Pantoea;s__Pantoea_deleyi
PB1_gene_1966	298	ORM80971.1	298	295/297(99.33)	1.42e-207	LysR family transcriptional regulator [Pantoea deleyi]	470932	d__Bacteria;k__norank;p__Proteobacteria;c__Gammaproteobacteria;o__Enterobacterales;f__Erwiniaceae;g__Pantoea;s__Pantoea_deleyi
PB1_gene_1967	401	ORM80970.1	401	386/401(96.26)	3.02e-270	MFS transporter [Pantoea deleyi]	470932	d__Bacteria;k__norank;p__Proteobacteria;c__Gammaproteobacteria;o__Enterobacterales;f__Erwiniaceae;g__Pantoea;s__Pantoea_deleyi
PB1_gene_1970	152	ORM86860.1	152	148/152(97.37)	2.09e-96	hypothetical protein HA42_00355 [Pantoea deleyi]	470932	d__Bacteria;k__norank;p__Proteobacteria;c__Gammaproteobacteria;o__Enterobacterales;f__Erwiniaceae;g__Pantoea;s__Pantoea_deleyi
PB1_gene_1971	281	ORM86861.1	281	280/281(99.64)	5.97e-196	PTS mannose transporter subunit IID [Pantoea deleyi]	470932	d__Bacteria;k__norank;p__Proteobacteria;c__Gammaproteobacteria;o__Enterobacterales;f__Erwiniaceae;g__Pantoea;s__Pantoea_deleyi
PB1_gene_1972	267	ORM86866.1	266	265/266(99.62)	1.20e-165	PTS mannose/fructose/sorbose transporter subunit IIC [Pantoea deleyi]	470932	d__Bacteria;k__norank;p__Proteobacteria;c__Gammaproteobacteria;o__Enterobacterales;f__Erwiniaceae;g__Pantoea;s__Pantoea_deleyi
PB1_gene_1976	540	WP_111139632.1	545	509/538(94.61)	0	DAK2 domain-containing protein [Pantoea sp. ARC607]	2027922	d__Bacteria;k__norank;p__Proteobacteria;c__Gammaproteobacteria;o__Enterobacterales;f__Erwiniaceae;g__Pantoea;s__Pantoea_sp._ARC607
PB1_gene_1977	235	ORM78408.1	235	232/235(98.72)	5.23e-158	hypothetical protein HA42_16700 [Pantoea deleyi]	470932	d__Bacteria;k__norank;p__Proteobacteria;c__Gammaproteobacteria;o__Enterobacterales;f__Erwiniaceae;g__Pantoea;s__Pantoea_deleyi
PB1_gene_1978	265	SFO18006.1	265	260/265(98.11)	9.75e-178	D-methionine transport system substrate-binding protein [Pantoea sp. OV426]	1881036	d__Bacteria;k__norank;p__Proteobacteria;c__Gammaproteobacteria;o__Enterobacterales;f__Erwiniaceae;g__Pantoea;s__Pantoea_sp._OV426
PB1_gene_1979	127	ORM78410.1	127	124/127(97.64)	1.03e-84	cupin [Pantoea deleyi]	470932	d__Bacteria;k__norank;p__Proteobacteria;c__Gammaproteobacteria;o__Enterobacterales;f__Erwiniaceae;g__Pantoea;s__Pantoea_deleyi
PB1_gene_1982	456	ORM72607.1	456	446/456(97.81)	0	succinate-semialdehyde dehydrogenase [Pantoea deleyi]	470932	d__Bacteria;k__norank;p__Proteobacteria;c__Gammaproteobacteria;o__Enterobacterales;f__Erwiniaceae;g__Pantoea;s__Pantoea_deleyi
PB1_gene_1985	294	ORM80506.1	294	292/294(99.32)	9.31e-211	cysteine synthase B [Pantoea deleyi]	470932	d__Bacteria;k__norank;p__Proteobacteria;c__Gammaproteobacteria;o__Enterobacterales;f__Erwiniaceae;g__Pantoea;s__Pantoea_deleyi
PB1_gene_1986	226	ORM80507.1	226	219/226(96.90)	1.56e-150	DNA-binding response regulator [Pantoea deleyi]	470932	d__Bacteria;k__norank;p__Proteobacteria;c__Gammaproteobacteria;o__Enterobacterales;f__Erwiniaceae;g__Pantoea;s__Pantoea_deleyi
PB1_gene_1988	51	SKA23874.1	51	29/29(100.00)	6.38e-11	hypothetical protein SAMN03097723_3771 [Pantoea agglomerans]	470933	d__Bacteria;k__norank;p__Proteobacteria;c__Gammaproteobacteria;o__Enterobacterales;f__Erwiniaceae;g__Pantoea;s__Pantoea_eucalypti
PB1_gene_1989	307	WP_111140707.1	300	297/300(99.00)	9.19e-200	Zn(2+)/Fe(2+)/Cd(2+) efflux CDF transporter FieF [Pantoea sp. ARC607]	2027922	d__Bacteria;k__norank;p__Proteobacteria;c__Gammaproteobacteria;o__Enterobacterales;f__Erwiniaceae;g__Pantoea;s__Pantoea_sp._ARC607
PB1_gene_1990	320	ORM84527.1	320	320/320(100.00)	2.74e-232	6-phosphofructokinase [Pantoea deleyi]	470932	d__Bacteria;k__norank;p__Proteobacteria;c__Gammaproteobacteria;o__Enterobacterales;f__Erwiniaceae;g__Pantoea;s__Pantoea_deleyi

PB1_gene_1994	194	WP_090392800.1	175	166/175(94.86)	1.50e-112	type VI secretion system lipoprotein TssJ [Enterobacter sp.	1566251	d__Bacteria;k__norank;p__Proteobacteria;c__Gammaproteobacteria;o__Enterobacterales;f__Enterobacteriaceae;g__Enterobacter;s__Enterobacter_sp._NFIX58
PB1_gene_1997	213	ORM82465.1	213	211/213(99.06)	5.48e-140	hypothetical protein HA42_08170 [Pantoea deleyi]	470932	d__Bacteria;k__norank;p__Proteobacteria;c__Gammaproteobacteria;o__Enterobacterales;f__Erwiniaceae;g__Pantoea;s__Pantoea_deleyi
PB1_gene_1998	376	ORM82464.1	376	364/376(96.81)	7.01e-261	dihydroorotase [Pantoea deleyi]	470932	d__Bacteria;k__norank;p__Proteobacteria;c__Gammaproteobacteria;o__Enterobacterales;f__Erwiniaceae;g__Pantoea;s__Pantoea_deleyi
PB1_gene_1999	64	BAH61390.1	367	30/59(50.85)	1.99e-07	hypothetical protein KP1_0516 [Klebsiella pneumoniae subsp. pneumoniae NTUH-K2044]	484021	d__Bacteria;k__norank;p__Proteobacteria;c__Gammaproteobacteria;o__Enterobacterales;f__Enterobacteriaceae;g__Klebsiella;s__Klebsiella_pneumoniae
PB1_gene_2001	284	WP_111140248.1	284	281/284(98.94)	1.12e-197	3-deoxy-8-phosphooctulonate synthase [Pantoea sp. ARC607]	2027922	d__Bacteria;k__norank;p__Proteobacteria;c__Gammaproteobacteria;o__Enterobacterales;f__Erwiniaceae;g__Pantoea;s__Pantoea_sp._ARC607
PB1_gene_2002	363	WP_111140247.1	363	361/363(99.45)	1.42e-242	sodium-potassium/proton antiporter ChaA [Pantoea sp.	2027922	d__Bacteria;k__norank;p__Proteobacteria;c__Gammaproteobacteria;o__Enterobacterales;f__Erwiniaceae;g__Pantoea;s__Pantoea_sp._ARC607
PB1_gene_2004	477	ORM85625.1	477	477/477(100.00)	0	UDP-phosphate galactose phosphotransferase [Pantoea	470932	d__Bacteria;k__norank;p__Proteobacteria;c__Gammaproteobacteria;o__Enterobacterales;f__Erwiniaceae;g__Pantoea;s__Pantoea_deleyi
PB1_gene_2006	50	WP_128087038.1	59	49/50(98.00)	1.50e-30	DUF1289 domain-containing protein [Pantoea deleyi]	470932	d__Bacteria;k__norank;p__Proteobacteria;c__Gammaproteobacteria;o__Enterobacterales;f__Erwiniaceae;g__Pantoea;s__Pantoea_deleyi
PB1_gene_2007	223	ORM72639.1	223	218/223(97.76)	1.11e-153	PhoP [Pantoea deleyi]	470932	d__Bacteria;k__norank;p__Proteobacteria;c__Gammaproteobacteria;o__Enterobacterales;f__Erwiniaceae;g__Pantoea;s__Pantoea_deleyi
PB1_gene_2010	100	WP_039660990.1	251	81/100(81.00)	2.82e-52	AraC family transcriptional regulator [Pantoea vagans]	53335	d__Bacteria;k__norank;p__Proteobacteria;c__Gammaproteobacteria;o__Enterobacterales;f__Erwiniaceae;g__Pantoea;s__norank
PB1_gene_2011	143	ORM75405.1	143	142/143(99.30)	2.81e-92	MarR family transcriptional regulator [Pantoea deleyi]	470932	d__Bacteria;k__norank;p__Proteobacteria;c__Gammaproteobacteria;o__Enterobacterales;f__Erwiniaceae;g__Pantoea;s__Pantoea_deleyi
PB1_gene_2015	386	WP_111139619.1	386	364/386(94.30)	3.42e-273	lycopene beta-cyclase CrtY [Pantoea sp. ARC607]	2027922	d__Bacteria;k__norank;p__Proteobacteria;c__Gammaproteobacteria;o__Enterobacterales;f__Erwiniaceae;g__Pantoea;s__Pantoea_sp._ARC607
PB1_gene_2019	610	ORM85209.1	552	523/552(94.75)	8.60e-313	methyl-accepting chemotaxis protein [Pantoea deleyi]	470932	d__Bacteria;k__norank;p__Proteobacteria;c__Gammaproteobacteria;o__Enterobacterales;f__Erwiniaceae;g__Pantoea;s__Pantoea_deleyi
PB1_gene_2020	143	SUB19213.1	217	125/143(87.41)	1.30e-79	POTRA domain, ShlB-type [Pantoea agglomerans]	549	d__Bacteria;k__norank;p__Proteobacteria;c__Gammaproteobacteria;o__Enterobacterales;f__Erwiniaceae;g__Pantoea;s__Pantoea_agglomerans
PB1_gene_2023	311	SJZ73758.1	311	299/311(96.14)	9.30e-215	homocysteine S-methyltransferase [Pantoea agglomerans]	470933	d__Bacteria;k__norank;p__Proteobacteria;c__Gammaproteobacteria;o__Enterobacterales;f__Erwiniaceae;g__Pantoea;s__Pantoea_eucalypti
PB1_gene_2024	139	ORM81204.1	139	139/139(100.00)	2.00e-99	hypothetical protein HA42_11520 [Pantoea deleyi]	470932	d__Bacteria;k__norank;p__Proteobacteria;c__Gammaproteobacteria;o__Enterobacterales;f__Erwiniaceae;g__Pantoea;s__Pantoea_deleyi
PB1_gene_2025	181	SJZ73729.1	181	175/181(96.69)	1.25e-127	diamine N-acetyltransferase [Pantoea agglomerans]	470933	d__Bacteria;k__norank;p__Proteobacteria;c__Gammaproteobacteria;o__Enterobacterales;f__Erwiniaceae;g__Pantoea;s__Pantoea_eucalypti
PB1_gene_2027	451	ORM86538.1	465	449/451(99.56)	2.71e-315	D-galactose transporter GalP [Pantoea deleyi]	470932	d__Bacteria;k__norank;p__Proteobacteria;c__Gammaproteobacteria;o__Enterobacterales;f__Erwiniaceae;g__Pantoea;s__Pantoea_deleyi
PB1_gene_2030	125	WP_122889979.1	125	121/124(97.58)	2.75e-79	peptidase [[Curtobacterium] plantarum]	221276	d__Bacteria;k__norank;p__Proteobacteria;c__Gammaproteobacteria;o__Enterobacterales;f__Erwiniaceae;g__Pantoea;s__[Curtobacterium]_plantarum
PB1_gene_2036	143	ORM86810.1	143	141/143(98.60)	1.63e-100	dihydroneopterin triphosphate diphosphatase [Pantoea deleyi]	470932	d__Bacteria;k__norank;p__Proteobacteria;c__Gammaproteobacteria;o__Enterobacterales;f__Erwiniaceae;g__Pantoea;s__Pantoea_deleyi
PB1_gene_2040	238	ORM81346.1	238	234/238(98.32)	1.31e-182	type VI secretion-associated protein [Pantoea deleyi]	470932	d__Bacteria;k__norank;p__Proteobacteria;c__Gammaproteobacteria;o__Enterobacterales;f__Erwiniaceae;g__Pantoea;s__Pantoea_deleyi
PB1_gene_2041	337	WP_111140785.1	337	322/337(95.55)	2.42e-230	type VI secretion system protein TssA [Pantoea sp. ARC607]	2027922	d__Bacteria;k__norank;p__Proteobacteria;c__Gammaproteobacteria;o__Enterobacterales;f__Erwiniaceae;g__Pantoea;s__Pantoea_sp._ARC607
PB1_gene_2044	245	WP_111139887.1	245	235/245(95.92)	1.82e-163	1-acylglycerol-3-phosphate O-acyltransferase [Pantoea sp. ARC607]	2027922	d__Bacteria;k__norank;p__Proteobacteria;c__Gammaproteobacteria;o__Enterobacterales;f__Erwiniaceae;g__Pantoea;s__Pantoea_sp._ARC607
PB1_gene_2047	160	ORM83981.1	160	159/160(99.38)	1.51e-111	dihydrofolate reductase [Pantoea deleyi]	470932	d__Bacteria;k__norank;p__Proteobacteria;c__Gammaproteobacteria;o__Enterobacterales;f__Erwiniaceae;g__Pantoea;s__Pantoea_deleyi

PB1_gene_2049	379	ORM83982.1	379	375/379(98.94)	1.87e-287	hypothetical protein HA42_05310 [Pantoea deleyi]	470932	d__Bacteria;k__norank;p__Proteobacteria;c__Gammaproteobacteria;o__Enterobacterales;f__Erwiniaceae;g__Pantoea;s__Pantoea_deleyi
PB1_gene_2054	304	ORM84126.1	304	283/304(93.09)	1.63e-200	iron ABC transporter substrate-binding protein [Pantoea deleyi]	470932	d__Bacteria;k__norank;p__Proteobacteria;c__Gammaproteobacteria;o__Enterobacterales;f__Erwiniaceae;g__Pantoea;s__Pantoea_deleyi
PB1_gene_2055	265	ORM84127.1	265	247/265(93.21)	1.17e-169	manganese/iron transporter ATP-binding protein [Pantoea deleyi]	470932	d__Bacteria;k__norank;p__Proteobacteria;c__Gammaproteobacteria;o__Enterobacterales;f__Erwiniaceae;g__Pantoea;s__Pantoea_deleyi
PB1_gene_2063	388	ORM83603.1	388	373/388(96.13)	1.15e-256	hypothetical protein HA42_06030 [Pantoea deleyi]	470932	d__Bacteria;k__norank;p__Proteobacteria;c__Gammaproteobacteria;o__Enterobacterales;f__Erwiniaceae;g__Pantoea;s__Pantoea_deleyi
PB1_gene_2066	381	WP_121554125.1	376	224/366(61.20)	2.02e-159	hypothetical protein [Gibbsiella quercinecans]	None	Unclassified
PB1_gene_2068	186	ORM76325.1	186	186/186(100.00)	6.63e-130	non-canonical purine NTP pyrophosphatase [Pantoea deleyi]	470932	d__Bacteria;k__norank;p__Proteobacteria;c__Gammaproteobacteria;o__Enterobacterales;f__Erwiniaceae;g__Pantoea;s__Pantoea_deleyi
PB1_gene_2071	448	WP_111139563.1	434	420/433(97.00)	9.67e-315	glycoside hydrolase family 10 protein [Pantoea sp. ARC607]	2027922	d__Bacteria;k__norank;p__Proteobacteria;c__Gammaproteobacteria;o__Enterobacterales;f__Erwiniaceae;g__Pantoea;s__Pantoea_sp._ARC607
PB1_gene_2075	183	ORM81819.1	183	183/183(100.00)	2.52e-137	glutathione-regulated potassium-efflux system ancillary protein KefG [Pantoea deleyi]	470932	d__Bacteria;k__norank;p__Proteobacteria;c__Gammaproteobacteria;o__Enterobacterales;f__Erwiniaceae;g__Pantoea;s__Pantoea_deleyi
PB1_gene_2077	520	ORM81387.1	520	506/520(97.31)	0	phage resistance protein [Pantoea deleyi]	470932	d__Bacteria;k__norank;p__Proteobacteria;c__Gammaproteobacteria;o__Enterobacterales;f__Erwiniaceae;g__Pantoea;s__Pantoea_deleyi
PB1_gene_2082	87	ORM51604.1	87	87/87(100.00)	7.27e-55	hypothetical protein HA39_21720 [Pantoea brenneri]	472694	d__Bacteria;k__norank;p__Proteobacteria;c__Gammaproteobacteria;o__Enterobacterales;f__Erwiniaceae;g__Pantoea;s__Pantoea_brenneri
PB1_gene_2085	156	ORM84013.1	156	156/156(100.00)	1.99e-105	peptidylprolyl isomerase [Pantoea deleyi]	470932	d__Bacteria;k__norank;p__Proteobacteria;c__Gammaproteobacteria;o__Enterobacterales;f__Erwiniaceae;g__Pantoea;s__Pantoea_deleyi
PB1_gene_2086	168	WP_111139369.1	168	168/168(100.00)	2.67e-118	lipoprotein signal peptidase [Pantoea sp. ARC607]	2027922	d__Bacteria;k__norank;p__Proteobacteria;c__Gammaproteobacteria;o__Enterobacterales;f__Erwiniaceae;g__Pantoea;s__Pantoea_sp._ARC607
PB1_gene_2089	621	ORM79177.1	621	616/621(99.19)	0	DNA mismatch repair protein MutL [Pantoea deleyi]	470932	d__Bacteria;k__norank;p__Proteobacteria;c__Gammaproteobacteria;o__Enterobacterales;f__Erwiniaceae;g__Pantoea;s__Pantoea_deleyi
PB1_gene_2092	452	WP_111139403.1	452	442/452(97.79)	2.43e-311	UDP-N-acetylmuramoyl-tripeptide--D-alanyl-D-alanine ligase [Pantoea sp. ARC607]	2027922	d__Bacteria;k__norank;p__Proteobacteria;c__Gammaproteobacteria;o__Enterobacterales;f__Erwiniaceae;g__Pantoea;s__Pantoea_sp._ARC607
PB1_gene_2096	341	WP_111139074.1	341	332/341(97.36)	1.07e-235	L,D-transpeptidase [Pantoea sp. ARC607]	2027922	d__Bacteria;k__norank;p__Proteobacteria;c__Gammaproteobacteria;o__Enterobacterales;f__Erwiniaceae;g__Pantoea;s__Pantoea_sp._ARC607
PB1_gene_2100	354	SJZ91362.1	354	351/354(99.15)	4.39e-262	uncharacterized zinc-type alcohol dehydrogenase-like protein [Pantoea agglomerans]	470933	d__Bacteria;k__norank;p__Proteobacteria;c__Gammaproteobacteria;o__Enterobacterales;f__Erwiniaceae;g__Pantoea;s__Pantoea_eucalypti
PB1_gene_2107	352	ORM81154.1	352	345/352(98.01)	1.84e-253	electron transport complex subunit RsxD [Pantoea deleyi]	470932	d__Bacteria;k__norank;p__Proteobacteria;c__Gammaproteobacteria;o__Enterobacterales;f__Erwiniaceae;g__Pantoea;s__Pantoea_deleyi
PB1_gene_2108	210	WP_111141129.1	210	204/210(97.14)	4.54e-142	electron transport complex subunit RsxG [Pantoea sp.	2027922	d__Bacteria;k__norank;p__Proteobacteria;c__Gammaproteobacteria;o__Enterobacterales;f__Erwiniaceae;g__Pantoea;s__Pantoea_sp._ARC607
PB1_gene_2111	161	ORM76469.1	161	161/161(100.00)	8.38e-114	redox-sensitive transcriptional activator SoxR [Pantoea deleyi]	470932	d__Bacteria;k__norank;p__Proteobacteria;c__Gammaproteobacteria;o__Enterobacterales;f__Erwiniaceae;g__Pantoea;s__Pantoea_deleyi
PB1_gene_2112	140	ORM76468.1	140	140/140(100.00)	6.96e-96	transcriptional regulator [Pantoea deleyi]	470932	d__Bacteria;k__norank;p__Proteobacteria;c__Gammaproteobacteria;o__Enterobacterales;f__Erwiniaceae;g__Pantoea;s__Pantoea_deleyi
PB1_gene_2113	93	ORM76467.1	93	93/93(100.00)	2.53e-60	hypothetical protein HA42_19080 [Pantoea deleyi]	470932	d__Bacteria;k__norank;p__Proteobacteria;c__Gammaproteobacteria;o__Enterobacterales;f__Erwiniaceae;g__Pantoea;s__Pantoea_deleyi
PB1_gene_2116	433	ORM84722.1	433	422/433(97.46)	4.24e-300	PST family transporter [Pantoea deleyi]	470932	d__Bacteria;k__norank;p__Proteobacteria;c__Gammaproteobacteria;o__Enterobacterales;f__Erwiniaceae;g__Pantoea;s__Pantoea_deleyi
PB1_gene_2117	331	ORM84723.1	326	324/326(99.39)	2.03e-236	glycosyl transferase [Pantoea deleyi]	470932	d__Bacteria;k__norank;p__Proteobacteria;c__Gammaproteobacteria;o__Enterobacterales;f__Erwiniaceae;g__Pantoea;s__Pantoea_deleyi
PB1_gene_2119	193	WP_111139889.1	193	190/193(98.45)	9.36e-138	flavodoxin family protein [Pantoea sp. ARC607]	2027922	d__Bacteria;k__norank;p__Proteobacteria;c__Gammaproteobacteria;o__Enterobacterales;f__Erwiniaceae;g__Pantoea;s__Pantoea_sp._ARC607

PB1_gene_2120	102	WP_111139890.1	102	102/102(100.00)	5.68e-68	antibiotic biosynthesis monooxygenase [Pantoea sp. ARC607]	2027922	d__Bacteria;k__norank;p__Proteobacteria;c__Gammaproteobacteria;o__Enterobacterales;f__Erwiniaceae;g__Pantoea;s__Pantoea_sp._ARC607
PB1_gene_2123	228	ORM81756.1	228	219/228(96.05)	2.18e-142	transporter [Pantoea deleyi]	470932	d__Bacteria;k__norank;p__Proteobacteria;c__Gammaproteobacteria;o__Enterobacterales;f__Erwiniaceae;g__Pantoea;s__Pantoea_deleyi
PB1_gene_2124	46	SFN30824.1	46	35/46(76.09)	2.98e-16	hypothetical protein SAMN05428971_0952 [Pantoea sp. OV426]	1881036	d__Bacteria;k__norank;p__Proteobacteria;c__Gammaproteobacteria;o__Enterobacterales;f__Erwiniaceae;g__Pantoea;s__Pantoea_sp._OV426
PB1_gene_2126	195	EXU74076.1	263	183/195(93.85)	7.94e-133	transposase [Erwinia mallotivora]	69222	d__Bacteria;k__norank;p__Proteobacteria;c__Gammaproteobacteria;o__Enterobacterales;f__Erwiniaceae;g__Erwinia;s__Erwinia_mallotivora
PB1_gene_2128	82	ORM81331.1	82	82/82(100.00)	1.30e-48	hypothetical protein HA42_11035 [Pantoea deleyi]	470932	d__Bacteria;k__norank;p__Proteobacteria;c__Gammaproteobacteria;o__Enterobacterales;f__Erwiniaceae;g__Pantoea;s__Pantoea_deleyi
PB1_gene_2129	248	ORM81347.1	244	236/244(96.72)	2.06e-165	flagellar brake protein [Pantoea deleyi]	470932	d__Bacteria;k__norank;p__Proteobacteria;c__Gammaproteobacteria;o__Enterobacterales;f__Erwiniaceae;g__Pantoea;s__Pantoea_deleyi
PB1_gene_2131	86	SJZ30372.1	86	85/86(98.84)	8.21e-56	large subunit ribosomal protein L31 [Pantoea agglomerans]	470933	d__Bacteria;k__norank;p__Proteobacteria;c__Gammaproteobacteria;o__Enterobacterales;f__Erwiniaceae;g__Pantoea;s__Pantoea_eucalypti
PB1_gene_2134	153	WP_111141672.1	146	144/146(98.63)	5.07e-96	YccF domain-containing protein [Pantoea sp. ARC607]	2027922	d__Bacteria;k__norank;p__Proteobacteria;c__Gammaproteobacteria;o__Enterobacterales;f__Erwiniaceae;g__Pantoea;s__Pantoea_sp._ARC607
PB1_gene_2135	136	WP_046102567.1	154	115/124(92.74)	2.29e-78	hypothetical protein [Pantoea anthophila]	470931	d__Bacteria;k__norank;p__Proteobacteria;c__Gammaproteobacteria;o__Enterobacterales;f__Erwiniaceae;g__Pantoea;s__Pantoea_anthophila
PB1_gene_2139	116	PCG11009.1	418	116/116(100.00)	9.54e-70	pyruvate dehydrogenase complex dihydrolipoamide acetyltransferase [Wolbachia	None	Unclassified
PB1_gene_2141	327	ORM80742.1	327	327/327(100.00)	4.87e-209	L-arabinose ABC transporter permease AraH [Pantoea deleyi]	470932	d__Bacteria;k__norank;p__Proteobacteria;c__Gammaproteobacteria;o__Enterobacterales;f__Erwiniaceae;g__Pantoea;s__Pantoea_deleyi
PB1_gene_2145	512	ORM81053.1	512	510/512(99.61)	0	murein biosynthesis integral membrane protein MurJ [Pantoea deleyi]	470932	d__Bacteria;k__norank;p__Proteobacteria;c__Gammaproteobacteria;o__Enterobacterales;f__Erwiniaceae;g__Pantoea;s__Pantoea_deleyi
PB1_gene_2146	283	ORM81052.1	306	274/283(96.82)	1.46e-198	virulence factor MviM [Pantoea deleyi]	470932	d__Bacteria;k__norank;p__Proteobacteria;c__Gammaproteobacteria;o__Enterobacterales;f__Erwiniaceae;g__Pantoea;s__Pantoea_deleyi
PB1_gene_2148	87	WP_077064457.1	87	79/87(90.80)	3.47e-53	PAAR domain-containing protein [Enterobacter chengduensis]	2494701	d__Bacteria;k__norank;p__Proteobacteria;c__Gammaproteobacteria;o__Enterobacterales;f__Enterobacteriaceae;g__Enterobacter;s__Enterobacter_chengduensis
PB1_gene_2149	401	WP_077064458.1	401	381/401(95.01)	1.59e-281	hypothetical protein [Enterobacter chengduensis]	2494701	d__Bacteria;k__norank;p__Proteobacteria;c__Gammaproteobacteria;o__Enterobacterales;f__Enterobacteriaceae;g__Enterobacter;s__Enterobacter_chengduensis
PB1_gene_2152	163	ADO09283.1	163	162/163(99.39)	8.02e-113	Putative type VI secretion system-associated hypothetical protein [Pantoea vagans C9-1]	712898	d__Bacteria;k__norank;p__Proteobacteria;c__Gammaproteobacteria;o__Enterobacterales;f__Erwiniaceae;g__Pantoea;s__Pantoea_vagans
PB1_gene_2153	405	ORM80454.1	405	404/405(99.75)	1.98e-293	hypothetical protein HA42_14490 [Pantoea deleyi]	470932	d__Bacteria;k__norank;p__Proteobacteria;c__Gammaproteobacteria;o__Enterobacterales;f__Erwiniaceae;g__Pantoea;s__Pantoea_deleyi
PB1_gene_2157	410	WP_111142016.1	410	402/410(98.05)	6.20e-263	serine/threonine transporter SstT [Pantoea sp. ARC607]	2027922	d__Bacteria;k__norank;p__Proteobacteria;c__Gammaproteobacteria;o__Enterobacterales;f__Erwiniaceae;g__Pantoea;s__Pantoea_sp._ARC607
PB1_gene_2158	334	WP_111142017.1	322	321/321(100.00)	1.83e-216	TerC family protein [Pantoea sp. ARC607]	2027922	d__Bacteria;k__norank;p__Proteobacteria;c__Gammaproteobacteria;o__Enterobacterales;f__Erwiniaceae;g__Pantoea;s__Pantoea_sp._ARC607
PB1_gene_2159	270	ORM79284.1	270	270/270(100.00)	5.07e-187	septum site-determining protein MinD [Pantoea deleyi]	470932	d__Bacteria;k__norank;p__Proteobacteria;c__Gammaproteobacteria;o__Enterobacterales;f__Erwiniaceae;g__Pantoea;s__Pantoea_deleyi
PB1_gene_2160	89	ORM79285.1	89	89/89(100.00)	2.06e-55	cell division topological specificity factor MinE [Pantoea	470932	d__Bacteria;k__norank;p__Proteobacteria;c__Gammaproteobacteria;o__Enterobacterales;f__Erwiniaceae;g__Pantoea;s__Pantoea_deleyi
PB1_gene_2161	373	WP_111138876.1	373	366/373(98.12)	4.63e-269	ribonuclease D [Pantoea sp. ARC607]	2027922	d__Bacteria;k__norank;p__Proteobacteria;c__Gammaproteobacteria;o__Enterobacterales;f__Erwiniaceae;g__Pantoea;s__Pantoea_sp._ARC607
PB1_gene_2166	384	ORM76166.1	384	363/384(94.53)	4.97e-265	class C beta-lactamase [Pantoea deleyi]	470932	d__Bacteria;k__norank;p__Proteobacteria;c__Gammaproteobacteria;o__Enterobacterales;f__Erwiniaceae;g__Pantoea;s__Pantoea_deleyi

PB1_gene_2169	302	WP_111141637.1	302	302/302(100.00)	1.10e-222	sulfate adenylyltransferase subunit CysD [Pantoea sp.	2027922	d__Bacteria;k__norank;p__Proteobacteria;c__Gammaproteobacteria;o__Enterobacterales;f__Erwiniaceae;g__Pantoea;s__Pantoea_sp._ARC607
PB1_gene_2172	142	ORM82727.1	142	134/142(94.37)	4.01e-96	hypothetical protein HA42_07595 [Pantoea deleyi]	470932	d__Bacteria;k__norank;p__Proteobacteria;c__Gammaproteobacteria;o__Enterobacterales;f__Erwiniaceae;g__Pantoea;s__Pantoea_deleyi
PB1_gene_2173	287	WP_111141469.1	287	283/287(98.61)	1.76e-191	YicC family protein [Pantoea sp. ARC607]	2027922	d__Bacteria;k__norank;p__Proteobacteria;c__Gammaproteobacteria;o__Enterobacterales;f__Erwiniaceae;g__Pantoea;s__Pantoea_sp._ARC607
PB1_gene_2175	256	ORM82636.1	256	242/253(95.65)	1.50e-172	16S rRNA (guanine(1516)-N(2))-methyltransferase [Pantoea	470932	d__Bacteria;k__norank;p__Proteobacteria;c__Gammaproteobacteria;o__Enterobacterales;f__Erwiniaceae;g__Pantoea;s__Pantoea_deleyi
PB1_gene_2176	87	SUB14524.1	80	38/78(48.72)	6.84e-13	Uncharacterised protein [Pantoea agglomerans]	549	d__Bacteria;k__norank;p__Proteobacteria;c__Gammaproteobacteria;o__Enterobacterales;f__Erwiniaceae;g__Pantoea;s__Pantoea_agglomerans
PB1_gene_2177	147	PAW35050.1	145	142/145(97.93)	7.75e-96	universal stress protein UspA [Pantoea vagans]	470934	d__Bacteria;k__norank;p__Proteobacteria;c__Gammaproteobacteria;o__Enterobacterales;f__Erwiniaceae;g__Pantoea;s__Pantoea_vagans
PB1_gene_2178	115	ORM75905.1	115	115/115(100.00)	4.95e-75	hypothetical protein HA42_19790 [Pantoea deleyi]	470932	d__Bacteria;k__norank;p__Proteobacteria;c__Gammaproteobacteria;o__Enterobacterales;f__Erwiniaceae;g__Pantoea;s__Pantoea_deleyi
PB1_gene_2179	536	WP_111140857.1	536	513/536(95.71)	0	multicopper oxidase CueO [Pantoea sp. ARC607]	2027922	d__Bacteria;k__norank;p__Proteobacteria;c__Gammaproteobacteria;o__Enterobacterales;f__Erwiniaceae;g__Pantoea;s__Pantoea_sp._ARC607
PB1_gene_2182	193	WP_111139190.1	193	192/193(99.48)	8.99e-136	dCTP deaminase [Pantoea sp. ARC607]	2027922	d__Bacteria;k__norank;p__Proteobacteria;c__Gammaproteobacteria;o__Enterobacterales;f__Erwiniaceae;g__Pantoea;s__Pantoea_sp._ARC607
PB1_gene_2183	213	SJZ63137.1	213	213/213(100.00)	2.25e-148	uridine kinase [Pantoea agglomerans]	470933	d__Bacteria;k__norank;p__Proteobacteria;c__Gammaproteobacteria;o__Enterobacterales;f__Erwiniaceae;g__Pantoea;s__Pantoea_eucalypti
PB1_gene_2186	260	PXW15947.1	260	229/260(88.08)	1.16e-162	NitT/TauT family transport system ATP-binding protein [Pantoea sp. JKS000250]	1938795	d__Bacteria;k__norank;p__Proteobacteria;c__Gammaproteobacteria;o__Enterobacterales;f__Erwiniaceae;g__Pantoea;s__Pantoea_sp._JKS000250
PB1_gene_2189	239	SKA15560.1	239	239/239(100.00)	6.17e-168	two-component system, OmpR family, phosphate regulon response regulator OmpR [Pantoea agglomerans]	470933	d__Bacteria;k__norank;p__Proteobacteria;c__Gammaproteobacteria;o__Enterobacterales;f__Erwiniaceae;g__Pantoea;s__Pantoea_eucalypti
PB1_gene_2191	335	WP_111140449.1	335	332/335(99.10)	5.93e-230	aspartate-semialdehyde dehydrogenase [Pantoea sp. ARC607]	2027922	d__Bacteria;k__norank;p__Proteobacteria;c__Gammaproteobacteria;o__Enterobacterales;f__Erwiniaceae;g__Pantoea;s__Pantoea_sp._ARC607
PB1_gene_2194	94	QAS63062.1	108	90/92(97.83)	2.10e-60	Hcp family T6SS protein CtsH1 [Klebsiella michiganensis]	1134687	d__Bacteria;k__norank;p__Proteobacteria;c__Gammaproteobacteria;o__Enterobacterales;f__Enterobacteriaceae;g__Klebsiella;s__Klebsiella_michiganensis
PB1_gene_2195	560	WP_090392821.1	560	550/560(98.21)	0	OmpA family protein [Enterobacter sp. NFIX58]	1566251	d__Bacteria;k__norank;p__Proteobacteria;c__Gammaproteobacteria;o__Enterobacterales;f__Enterobacteriaceae;g__Enterobacter;s__Enterobacter_sp._NFIX58
PB1_gene_2198	149	WP_111138978.1	148	140/148(94.59)	3.95e-97	NfeD family protein [Pantoea sp. ARC607]	2027922	d__Bacteria;k__norank;p__Proteobacteria;c__Gammaproteobacteria;o__Enterobacterales;f__Erwiniaceae;g__Pantoea;s__Pantoea_sp._ARC607
PB1_gene_2199	150	SUB18016.1	150	140/150(93.33)	2.98e-95	Copper export regulator [Pantoea agglomerans]	549	d__Bacteria;k__norank;p__Proteobacteria;c__Gammaproteobacteria;o__Enterobacterales;f__Erwiniaceae;g__Pantoea;s__Pantoea_agglomerans
PB1_gene_2200	249	SAW26952.1	239	46/101(45.54)	1.38e-15	Uncharacterised protein [Klebsiella pneumoniae]	573	d__Bacteria;k__norank;p__Proteobacteria;c__Gammaproteobacteria;o__Enterobacterales;f__Enterobacteriaceae;g__Klebsiella;s__Klebsiella_pneumoniae
PB1_gene_2206	610	WP_111138579.1	610	604/610(99.02)	0	excinuclease ABC subunit UvrC [Pantoea sp. ARC607]	2027922	d__Bacteria;k__norank;p__Proteobacteria;c__Gammaproteobacteria;o__Enterobacterales;f__Erwiniaceae;g__Pantoea;s__Pantoea_sp._ARC607
PB1_gene_2209	205	ORM82576.1	213	205/205(100.00)	7.66e-138	protein phosphatase CheZ [Pantoea deleyi]	470932	d__Bacteria;k__norank;p__Proteobacteria;c__Gammaproteobacteria;o__Enterobacterales;f__Erwiniaceae;g__Pantoea;s__Pantoea_deleyi
PB1_gene_2210	129	SJZ55609.1	129	129/129(100.00)	1.40e-83	two-component system, chemotaxis family, response regulator CheY [Pantoea	470933	d__Bacteria;k__norank;p__Proteobacteria;c__Gammaproteobacteria;o__Enterobacterales;f__Erwiniaceae;g__Pantoea;s__Pantoea_eucalypti
PB1_gene_2215	229	WP_111139812.1	229	228/229(99.56)	2.79e-150	molybdate ABC transporter permease subunit [Pantoea sp. ARC607]	2027922	d__Bacteria;k__norank;p__Proteobacteria;c__Gammaproteobacteria;o__Enterobacterales;f__Erwiniaceae;g__Pantoea;s__Pantoea_sp._ARC607

PB1_gene_2216	257	ORM78708.1	257	249/257(96.89)	8.97e-170	molybdate ABC transporter substrate-binding protein [Pantoea deleyi]	470932	d__Bacteria;k__norank;p__Proteobacteria;c__Gammaproteobacteria;o__Enterobacterales;f__Erwiniaceae;g__Pantoea;s__Pantoea_deleyi
PB1_gene_2217	80	ORM78709.1	80	79/80(98.75)	2.48e-43	hypothetical protein HA42_16385 [Pantoea deleyi]	470932	d__Bacteria;k__norank;p__Proteobacteria;c__Gammaproteobacteria;o__Enterobacterales;f__Erwiniaceae;g__Pantoea;s__Pantoea_deleyi
PB1_gene_2220	328	WP_111139389.1	328	322/327(98.47)	4.56e-235	thiamine ABC transporter substrate binding subunit [Pantoea sp. ARC607]	2027922	d__Bacteria;k__norank;p__Proteobacteria;c__Gammaproteobacteria;o__Enterobacterales;f__Erwiniaceae;g__Pantoea;s__Pantoea_sp._ARC607
PB1_gene_2226	173	ORM82746.1	173	170/173(98.27)	4.33e-106	GTPase-activating protein [Pantoea deleyi]	470932	d__Bacteria;k__norank;p__Proteobacteria;c__Gammaproteobacteria;o__Enterobacterales;f__Erwiniaceae;g__Pantoea;s__Pantoea_deleyi
PB1_gene_2227	199	WP_111141987.1	211	196/198(98.99)	1.26e-134	YihA family ribosome biogenesis GTP-binding protein [Pantoea sp. ARC607]	2027922	d__Bacteria;k__norank;p__Proteobacteria;c__Gammaproteobacteria;o__Enterobacterales;f__Erwiniaceae;g__Pantoea;s__Pantoea_sp._ARC607
PB1_gene_2229	263	ORM76431.1	257	239/257(93.00)	4.34e-174	arpA protein [Pantoea deleyi]	470932	d__Bacteria;k__norank;p__Proteobacteria;c__Gammaproteobacteria;o__Enterobacterales;f__Erwiniaceae;g__Pantoea;s__Pantoea_deleyi
PB1_gene_2232	309	WP_111139367.1	309	307/309(99.35)	1.10e-221	bifunctional riboflavin kinase/FAD synthetase [Pantoea	2027922	d__Bacteria;k__norank;p__Proteobacteria;c__Gammaproteobacteria;o__Enterobacterales;f__Erwiniaceae;g__Pantoea;s__Pantoea_sp._ARC607
PB1_gene_2233	61	SKA70446.1	87	61/61(100.00)	7.97e-34	SSU ribosomal protein S20P [Pantoea ananatis]	553	d__Bacteria;k__norank;p__Proteobacteria;c__Gammaproteobacteria;o__Enterobacterales;f__Erwiniaceae;g__Pantoea;s__Pantoea_ananatis
PB1_gene_2237	315	ORM82739.1	315	315/315(100.00)	2.63e-232	GNAT family N-acetyltransferase [Pantoea deleyi]	470932	d__Bacteria;k__norank;p__Proteobacteria;c__Gammaproteobacteria;o__Enterobacterales;f__Erwiniaceae;g__Pantoea;s__Pantoea_deleyi
PB1_gene_2238	364	WP_111139830.1	364	360/364(98.90)	2.64e-259	DUF1615 domain-containing protein [Pantoea sp. ARC607]	2027922	d__Bacteria;k__norank;p__Proteobacteria;c__Gammaproteobacteria;o__Enterobacterales;f__Erwiniaceae;g__Pantoea;s__Pantoea_sp._ARC607
PB1_gene_2239	235	WP_111139831.1	235	233/235(99.15)	2.78e-153	Bax inhibitor-1/YccA family protein [Pantoea sp. ARC607]	53335	d__Bacteria;k__norank;p__Proteobacteria;c__Gammaproteobacteria;o__Enterobacterales;f__Erwiniaceae;g__Pantoea;s__norank
PB1_gene_2242	602	WP_122292546.1	608	299/603(49.59)	8.30e-199	conjugal transfer protein [Pseudomonas syringae group genomosp. 3]	251701	d__Bacteria;k__norank;p__Proteobacteria;c__Gammaproteobacteria;o__Pseudomonadales;f__Pseudomonadaceae;g__Pseudomonas;s__Pseudomonas_syringae_group_genomosp._3
PB1_gene_2245	335	ORM75315.1	332	321/332(96.69)	3.95e-241	serine kinase, partial [Pantoea deleyi]	470932	d__Bacteria;k__norank;p__Proteobacteria;c__Gammaproteobacteria;o__Enterobacterales;f__Erwiniaceae;g__Pantoea;s__Pantoea_deleyi
PB1_gene_2249	307	ORM82914.1	307	304/307(99.02)	4.41e-211	bile acid:sodium symporter [Pantoea deleyi]	470932	d__Bacteria;k__norank;p__Proteobacteria;c__Gammaproteobacteria;o__Enterobacterales;f__Erwiniaceae;g__Pantoea;s__Pantoea_deleyi
PB1_gene_2256	230	ORM85065.1	230	223/230(96.96)	4.03e-139	flagellar assembly protein FliH [Pantoea deleyi]	470932	d__Bacteria;k__norank;p__Proteobacteria;c__Gammaproteobacteria;o__Enterobacterales;f__Erwiniaceae;g__Pantoea;s__Pantoea_deleyi
PB1_gene_2257	330	PZL95125.1	330	330/330(100.00)	2.39e-222	flagellar motor switch protein FliG [Pantoea sp. ARC607]	2027922	d__Bacteria;k__norank;p__Proteobacteria;c__Gammaproteobacteria;o__Enterobacterales;f__Erwiniaceae;g__Pantoea;s__Pantoea_sp._ARC607
PB1_gene_2260	242	ORM86807.1	242	242/242(100.00)	1.78e-173	carboxy-S-adenosyl-L-methionine synthase CmoA [Pantoea deleyi]	470932	d__Bacteria;k__norank;p__Proteobacteria;c__Gammaproteobacteria;o__Enterobacterales;f__Erwiniaceae;g__Pantoea;s__Pantoea_deleyi
PB1_gene_2261	131	ORM86808.1	131	130/131(99.24)	9.78e-85	hypothetical protein HA42_00055 [Pantoea deleyi]	470932	d__Bacteria;k__norank;p__Proteobacteria;c__Gammaproteobacteria;o__Enterobacterales;f__Erwiniaceae;g__Pantoea;s__Pantoea_deleyi
PB1_gene_2264	287	WP_089561847.1	287	247/287(86.06)	2.19e-185	MULTISPECIES: FAA hydrolase family protein [Pantoea]	53335	d__Bacteria;k__norank;p__Proteobacteria;c__Gammaproteobacteria;o__Enterobacterales;f__Erwiniaceae;g__Pantoea;s__norank
PB1_gene_2267	298	ORM85296.1	298	288/298(96.64)	6.93e-199	nicotinate-nucleotide diphosphorylase [Pantoea deleyi]	470932	d__Bacteria;k__norank;p__Proteobacteria;c__Gammaproteobacteria;o__Enterobacterales;f__Erwiniaceae;g__Pantoea;s__Pantoea_deleyi
PB1_gene_2268	151	WP_111139422.1	151	146/151(96.69)	3.91e-96	prepilin peptidase-dependent pilin [Pantoea sp. ARC607]	2027922	d__Bacteria;k__norank;p__Proteobacteria;c__Gammaproteobacteria;o__Enterobacterales;f__Erwiniaceae;g__Pantoea;s__Pantoea_sp._ARC607
PB1_gene_2269	78	ORM85294.1	461	74/78(94.87)	1.46e-39	type II secretion system protein GspE [Pantoea deleyi]	470932	d__Bacteria;k__norank;p__Proteobacteria;c__Gammaproteobacteria;o__Enterobacterales;f__Erwiniaceae;g__Pantoea;s__Pantoea_deleyi
PB1_gene_2271	317	WP_111139770.1	317	316/317(99.68)	1.38e-219	magnesium/cobalt transporter CorA [Pantoea sp. ARC607]	2027922	d__Bacteria;k__norank;p__Proteobacteria;c__Gammaproteobacteria;o__Enterobacterales;f__Erwiniaceae;g__Pantoea;s__Pantoea_sp._ARC607

PB1_gene_2274	427	ORM83999.1	427	423/427(99.06)	1.74e-305	threonine synthase [Pantoea deleyi]	470932	d__Bacteria;k__norank;p__Proteobacteria;c__Gammaproteobacteria;o__Enterobacterales;f__Erwiniaceae;g__Pantoea;s__Pantoea_deleyi
PB1_gene_2275	309	ORM84000.1	309	304/309(98.38)	4.00e-224	homoserine kinase [Pantoea deleyi]	470932	d__Bacteria;k__norank;p__Proteobacteria;c__Gammaproteobacteria;o__Enterobacterales;f__Erwiniaceae;g__Pantoea;s__Pantoea_deleyi
PB1_gene_2277	245	WP_111141193.1	234	228/234(97.44)	2.67e-167	phosphohydrolase [Pantoea sp. ARC607]	2027922	d__Bacteria;k__norank;p__Proteobacteria;c__Gammaproteobacteria;o__Enterobacterales;f__Erwiniaceae;g__Pantoea;s__Pantoea_sp._ARC607
PB1_gene_2278	93	WP_111141192.1	93	91/93(97.85)	8.88e-52	Arc family DNA-binding protein [Pantoea sp. ARC607]	2027922	d__Bacteria;k__norank;p__Proteobacteria;c__Gammaproteobacteria;o__Enterobacterales;f__Erwiniaceae;g__Pantoea;s__Pantoea_sp._ARC607
PB1_gene_2279	460	WP_111141191.1	461	431/459(93.90)	0	DNA (cytosine-5-)-methyltransferase [Pantoea sp.	2027922	d__Bacteria;k__norank;p__Proteobacteria;c__Gammaproteobacteria;o__Enterobacterales;f__Erwiniaceae;g__Pantoea;s__Pantoea_sp._ARC607
PB1_gene_2280	327	ORM78920.1	327	325/326(99.69)	4.22e-235	zinc transporter [Pantoea deleyi]	470932	d__Bacteria;k__norank;p__Proteobacteria;c__Gammaproteobacteria;o__Enterobacterales;f__Erwiniaceae;g__Pantoea;s__Pantoea_deleyi
PB1_gene_2284	263	ORM82834.1	263	261/263(99.24)	6.21e-189	metal-dependent hydrolase [Pantoea deleyi]	470932	d__Bacteria;k__norank;p__Proteobacteria;c__Gammaproteobacteria;o__Enterobacterales;f__Erwiniaceae;g__Pantoea;s__Pantoea_deleyi
PB1_gene_2287	219	ORM82644.1	219	218/219(99.54)	3.23e-169	hypothetical protein HA42_07830 [Pantoea deleyi]	470932	d__Bacteria;k__norank;p__Proteobacteria;c__Gammaproteobacteria;o__Enterobacterales;f__Erwiniaceae;g__Pantoea;s__Pantoea_deleyi
PB1_gene_2288	165	ORM82645.1	165	152/165(92.12)	1.05e-109	AraC family transcriptional regulator [Pantoea deleyi]	470932	d__Bacteria;k__norank;p__Proteobacteria;c__Gammaproteobacteria;o__Enterobacterales;f__Erwiniaceae;g__Pantoea;s__Pantoea_deleyi
PB1_gene_2291	179	ORM84712.1	179	176/179(98.32)	1.35e-121	Ter macrodomain-binding protein MatP [Pantoea deleyi]	470932	d__Bacteria;k__norank;p__Proteobacteria;c__Gammaproteobacteria;o__Enterobacterales;f__Erwiniaceae;g__Pantoea;s__Pantoea_deleyi
PB1_gene_2292	604	ORM84713.1	622	594/604(98.34)	0	Lon protease [Pantoea deleyi]	470932	d__Bacteria;k__norank;p__Proteobacteria;c__Gammaproteobacteria;o__Enterobacterales;f__Erwiniaceae;g__Pantoea;s__Pantoea_deleyi
PB1_gene_2294	428	ORM78370.1	428	425/428(99.30)	4.91e-315	aminopeptidase PepB [Pantoea deleyi]	470932	d__Bacteria;k__norank;p__Proteobacteria;c__Gammaproteobacteria;o__Enterobacterales;f__Erwiniaceae;g__Pantoea;s__Pantoea_deleyi
PB1_gene_2297	106	SJZ32500.1	106	106/106(100.00)	2.97e-74	ATP-dependent Clp protease adaptor protein ClpS [Pantoea agglomerans]	470933	d__Bacteria;k__norank;p__Proteobacteria;c__Gammaproteobacteria;o__Enterobacterales;f__Erwiniaceae;g__Pantoea;s__Pantoea_eucalypti
PB1_gene_2298	74	ORM76080.1	74	71/74(95.95)	2.12e-48	cold shock domain protein CspD [Pantoea deleyi]	470932	d__Bacteria;k__norank;p__Proteobacteria;c__Gammaproteobacteria;o__Enterobacterales;f__Erwiniaceae;g__Pantoea;s__Pantoea_deleyi
PB1_gene_2300	491	WP_111139774.1	491	490/491(99.80)	0	glucose-6-phosphate dehydrogenase [Pantoea sp. ARC607]	53335	d__Bacteria;k__norank;p__Proteobacteria;c__Gammaproteobacteria;o__Enterobacterales;f__Erwiniaceae;g__Pantoea;s__norank
PB1_gene_2301	212	ORM86823.1	212	207/211(98.10)	7.83e-144	keto-deoxy-phosphogluconate aldolase [Pantoea deleyi]	470932	d__Bacteria;k__norank;p__Proteobacteria;c__Gammaproteobacteria;o__Enterobacterales;f__Erwiniaceae;g__Pantoea;s__Pantoea_deleyi
PB1_gene_2304	308	ORM75590.1	308	304/308(98.70)	8.02e-219	phosphonate ABC transporter substrate-binding protein [Pantoea deleyi]	470932	d__Bacteria;k__norank;p__Proteobacteria;c__Gammaproteobacteria;o__Enterobacterales;f__Erwiniaceae;g__Pantoea;s__Pantoea_deleyi
PB1_gene_2305	275	ORM75589.1	275	268/275(97.45)	4.50e-188	phosphonate ABC transporter ATP-binding protein [Pantoea	470932	d__Bacteria;k__norank;p__Proteobacteria;c__Gammaproteobacteria;o__Enterobacterales;f__Erwiniaceae;g__Pantoea;s__Pantoea_deleyi
PB1_gene_2307	61	SJZ96244.1	61	61/61(100.00)	1.07e-31	carbon storage regulator, CsrA [Pantoea agglomerans]	470933	d__Bacteria;k__norank;p__Proteobacteria;c__Gammaproteobacteria;o__Enterobacterales;f__Erwiniaceae;g__Pantoea;s__Pantoea_eucalypti
PB1_gene_2309	594	ORM85218.1	594	585/594(98.48)	0	histidine kinase [Pantoea deleyi]	470932	d__Bacteria;k__norank;p__Proteobacteria;c__Gammaproteobacteria;o__Enterobacterales;f__Erwiniaceae;g__Pantoea;s__Pantoea_deleyi
PB1_gene_2312	156	SUB15665.1	139	96/101(95.05)	1.60e-64	Indole-3-pyruvate decarboxylase [Pantoea agglomerans]	549	d__Bacteria;k__norank;p__Proteobacteria;c__Gammaproteobacteria;o__Enterobacterales;f__Erwiniaceae;g__Pantoea;s__Pantoea_agglomerans
PB1_gene_2313	329	ORM77226.1	329	323/329(98.18)	1.72e-235	L-glyceraldehyde 3-phosphate reductase [Pantoea deleyi]	470932	d__Bacteria;k__norank;p__Proteobacteria;c__Gammaproteobacteria;o__Enterobacterales;f__Erwiniaceae;g__Pantoea;s__Pantoea_deleyi
PB1_gene_2314	133	PAW35799.1	134	112/134(83.58)	1.02e-46	DUF2502 domain-containing protein [Pantoea vagans]	470934	d__Bacteria;k__norank;p__Proteobacteria;c__Gammaproteobacteria;o__Enterobacterales;f__Erwiniaceae;g__Pantoea;s__Pantoea_vagans
PB1_gene_2316	244	WP_111140428.1	244	241/244(98.77)	1.30e-176	glutathione peroxidase [Pantoea sp. ARC607]	2027922	d__Bacteria;k__norank;p__Proteobacteria;c__Gammaproteobacteria;o__Enterobacterales;f__Erwiniaceae;g__Pantoea;s__Pantoea_sp._ARC607

PB1_gene_2319	334	ORM84507.1	334	321/334(96.11)	8.61e-243	sugar glycosyltransferase [Pantoea deleyi]	470932	d__Bacteria;k__norank;p__Proteobacteria;c__Gammaproteobacteria;o__Enterobacterales;f__Erwiniaceae;g__Pantoea;s__Pantoea_deleyi
PB1_gene_2322	60	OAM06476.1	56	53/54(98.15)	1.24e-29	transposase [Wolbachia endosymbiont of Dactylopius coccus]	1605993	d__Bacteria;k__norank;p__Proteobacteria;c__Alphaproteobacteria;o__Rickettsiales;f__Anaplasmataceae;g__Wolbachia;s__Wolbachia_endosymbiont_of_Dactylopius_coccus
PB1_gene_2325	261	ORM82551.1	494	258/261(98.85)	1.23e-175	sodium/proline symporter [Pantoea deleyi]	470932	d__Bacteria;k__norank;p__Proteobacteria;c__Gammaproteobacteria;o__Enterobacterales;f__Erwiniaceae;g__Pantoea;s__Pantoea_deleyi
PB1_gene_2328	234	WP_117343256.1	234	198/234(84.62)	1.37e-148	methyltransferase domain-containing protein [Citrobacter	544	d__Bacteria;k__norank;p__Proteobacteria;c__Gammaproteobacteria;o__Enterobacterales;f__Enterobacteriaceae;g__Citrobacter;s__norank
PB1_gene_2331	486	ORM82373.1	486	477/486(98.15)	0	sensor protein PhoQ [Pantoea deleyi]	470932	d__Bacteria;k__norank;p__Proteobacteria;c__Gammaproteobacteria;o__Enterobacterales;f__Erwiniaceae;g__Pantoea;s__Pantoea_deleyi
PB1_gene_2334	126	ORM80491.1	131	126/126(100.00)	5.39e-83	hypothetical protein HA42_14330 [Pantoea deleyi]	470932	d__Bacteria;k__norank;p__Proteobacteria;c__Gammaproteobacteria;o__Enterobacterales;f__Erwiniaceae;g__Pantoea;s__Pantoea_deleyi
PB1_gene_2335	366	ORM80490.1	366	364/366(99.45)	2.05e-273	23S rRNA (cytidine(2498)-2'-O)-methyltransferase RlmM [Pantoea deleyi]	470932	d__Bacteria;k__norank;p__Proteobacteria;c__Gammaproteobacteria;o__Enterobacterales;f__Erwiniaceae;g__Pantoea;s__Pantoea_deleyi
PB1_gene_2338	419	SKA08438.1	419	419/419(100.00)	1.36e-298	transcription termination factor Rho [Pantoea agglomerans]	470933	d__Bacteria;k__norank;p__Proteobacteria;c__Gammaproteobacteria;o__Enterobacterales;f__Erwiniaceae;g__Pantoea;s__Pantoea_eucalypti
PB1_gene_2339	110	SKA08448.1	110	110/110(100.00)	4.86e-75	thioredoxin [Pantoea agglomerans]	470933	d__Bacteria;k__norank;p__Proteobacteria;c__Gammaproteobacteria;o__Enterobacterales;f__Erwiniaceae;g__Pantoea;s__Pantoea_eucalypti
PB1_gene_2342	322	EZI32932.1	322	280/322(86.96)	1.32e-199	Thiazole biosynthesis protein ThiF [Pantoea agglomerans]	549	d__Bacteria;k__norank;p__Proteobacteria;c__Gammaproteobacteria;o__Enterobacterales;f__Erwiniaceae;g__Pantoea;s__Pantoea_agglomerans
PB1_gene_2343	137	ORM77023.1	137	130/137(94.89)	7.02e-90	ASCH domain-containing protein [Pantoea deleyi]	470932	d__Bacteria;k__norank;p__Proteobacteria;c__Gammaproteobacteria;o__Enterobacterales;f__Erwiniaceae;g__Pantoea;s__Pantoea_deleyi
PB1_gene_2346	122	OXH78948.1	122	122/122(100.00)	1.13e-72	diacylglycerol kinase [Pantoea agglomerans]	549	d__Bacteria;k__norank;p__Proteobacteria;c__Gammaproteobacteria;o__Enterobacterales;f__Erwiniaceae;g__Pantoea;s__Pantoea_agglomerans
PB1_gene_2347	204	ORM82926.1	204	204/204(100.00)	6.76e-141	repressor LexA [Pantoea deleyi]	470932	d__Bacteria;k__norank;p__Proteobacteria;c__Gammaproteobacteria;o__Enterobacterales;f__Erwiniaceae;g__Pantoea;s__Pantoea_deleyi
PB1_gene_2350	493	ORM85051.1	493	491/493(99.59)	0	alpha-amylase [Pantoea deleyi]	470932	d__Bacteria;k__norank;p__Proteobacteria;c__Gammaproteobacteria;o__Enterobacterales;f__Erwiniaceae;g__Pantoea;s__Pantoea_deleyi
PB1_gene_2351	156	WP_111139966.1	156	152/156(97.44)	3.45e-106	lipoprotein [Pantoea sp. ARC607]	2027922	d__Bacteria;k__norank;p__Proteobacteria;c__Gammaproteobacteria;o__Enterobacterales;f__Erwiniaceae;g__Pantoea;s__Pantoea_sp._ARC607
PB1_gene_2353	256	ORM77974.1	256	255/256(99.61)	5.45e-175	ABC transporter permease [Pantoea deleyi]	470932	d__Bacteria;k__norank;p__Proteobacteria;c__Gammaproteobacteria;o__Enterobacterales;f__Erwiniaceae;g__Pantoea;s__Pantoea_deleyi
PB1_gene_2354	309	ORM77973.1	309	308/309(99.68)	5.61e-215	ABC transporter [Pantoea deleyi]	470932	d__Bacteria;k__norank;p__Proteobacteria;c__Gammaproteobacteria;o__Enterobacterales;f__Erwiniaceae;g__Pantoea;s__Pantoea_deleyi
PB1_gene_2357	258	ORM81891.1	258	247/258(95.74)	5.52e-185	xanthine dehydrogenase accessory protein XdhC [Pantoea	470932	d__Bacteria;k__norank;p__Proteobacteria;c__Gammaproteobacteria;o__Enterobacterales;f__Erwiniaceae;g__Pantoea;s__Pantoea_deleyi
PB1_gene_2365	447	ORM80686.1	444	437/444(98.42)	6.22e-313	DUF1338 domain-containing protein, partial [Pantoea deleyi]	470932	d__Bacteria;k__norank;p__Proteobacteria;c__Gammaproteobacteria;o__Enterobacterales;f__Erwiniaceae;g__Pantoea;s__Pantoea_deleyi
PB1_gene_2368	439	ORM79155.1	439	430/439(97.95)	6.07e-303	pili assembly chaperone [Pantoea deleyi]	470932	d__Bacteria;k__norank;p__Proteobacteria;c__Gammaproteobacteria;o__Enterobacterales;f__Erwiniaceae;g__Pantoea;s__Pantoea_deleyi
PB1_gene_2371	205	WP_111141970.1	205	201/205(98.05)	1.47e-140	cytochrome c biogenesis heme-transporting ATPase CcmA [Pantoea sp. ARC607]	2027922	d__Bacteria;k__norank;p__Proteobacteria;c__Gammaproteobacteria;o__Enterobacterales;f__Erwiniaceae;g__Pantoea;s__Pantoea_sp._ARC607
PB1_gene_2372	219	WP_111141971.1	219	215/219(98.17)	3.02e-140	heme exporter protein CcmB [Pantoea sp. ARC607]	2027922	d__Bacteria;k__norank;p__Proteobacteria;c__Gammaproteobacteria;o__Enterobacterales;f__Erwiniaceae;g__Pantoea;s__Pantoea_sp._ARC607
PB1_gene_2373	246	ORM81540.1	246	242/245(98.78)	2.52e-175	heme ABC transporter permease [Pantoea deleyi]	470932	d__Bacteria;k__norank;p__Proteobacteria;c__Gammaproteobacteria;o__Enterobacterales;f__Erwiniaceae;g__Pantoea;s__Pantoea_deleyi
PB1_gene_2376	195	ORM83996.1	195	194/195(99.49)	6.06e-135	molybdopterin adenylyltransferase [Pantoea	470932	d__Bacteria;k__norank;p__Proteobacteria;c__Gammaproteobacteria;o__Enterobacterales;f__Erwiniaceae;g__Pantoea;s__Pantoea_deleyi

PB1_gene_2377	317	ORM83997.1	317	317/317(100.00)	3.54e-222	transaldolase [Pantoea deleyi]	470932	d__Bacteria;k__norank;p__Proteobacteria;c__Gammaproteobacteria;o__Enterobacterales;f__Erwiniaceae;g__Pantoea;s__Pantoea_deleyi
PB1_gene_2378	307	SJZ66137.1	307	307/307(100.00)	3.38e-215	DNA-binding transcriptional regulator, LysR family [Pantoea agglomerans]	470933	d__Bacteria;k__norank;p__Proteobacteria;c__Gammaproteobacteria;o__Enterobacterales;f__Erwiniaceae;g__Pantoea;s__Pantoea_eucalypti
PB1_gene_2379	129	CNV26202.1	86	44/81(54.32)	1.86e-24	Uncharacterised protein [Salmonella enterica subsp. enterica serovar	58097	d__Bacteria;k__norank;p__Proteobacteria;c__Gammaproteobacteria;o__Enterobacterales;f__Enterobacteriaceae;g__Salmonella;s__Salmonella_enterica
PB1_gene_2385	79	OON40272.1	79	79/79(100.00)	3.29e-43	ATP F0F1 synthase subunit C [Izhakiella australiensis]	1926881	d__Bacteria;k__norank;p__Proteobacteria;c__Gammaproteobacteria;o__Enterobacterales;f__Erwiniaceae;g__Izhakiella;s__Izhakiella_australiensis
PB1_gene_2386	272	WP_111140949.1	272	272/272(100.00)	7.21e-188	F0F1 ATP synthase subunit A [Pantoea sp. ARC607]	2027922	d__Bacteria;k__norank;p__Proteobacteria;c__Gammaproteobacteria;o__Enterobacterales;f__Erwiniaceae;g__Pantoea;s__Pantoea_sp._ARC607
PB1_gene_2387	126	ORM85453.1	126	125/126(99.21)	2.85e-77	ATP F0F1 synthase subunit I [Pantoea deleyi]	470932	d__Bacteria;k__norank;p__Proteobacteria;c__Gammaproteobacteria;o__Enterobacterales;f__Erwiniaceae;g__Pantoea;s__Pantoea_deleyi
PB1_gene_2391	210	CUQ60976.1	340	59/143(41.26)	4.62e-25	Uncharacterised protein [Flavonifractor plautii]	292800	d__Bacteria;k__norank;p__Firmicutes;c__Clostridia;o__Clostridiales;f__Ruminococcaceae;g__Flavonifractor;s__Flavonifractor_plautii
PB1_gene_2393	129	ORM83043.1	129	125/129(96.90)	3.05e-87	lyase [Pantoea deleyi]	470932	d__Bacteria;k__norank;p__Proteobacteria;c__Gammaproteobacteria;o__Enterobacterales;f__Erwiniaceae;g__Pantoea;s__Pantoea_deleyi
PB1_gene_2398	158	ORM83364.1	158	156/158(98.73)	2.41e-107	tRNA (adenosine(37)-N6)-threonylcarbamoyltransferase complex ATPase subunit type 1 TsaE [Pantoea deleyi]	470932	d__Bacteria;k__norank;p__Proteobacteria;c__Gammaproteobacteria;o__Enterobacterales;f__Erwiniaceae;g__Pantoea;s__Pantoea_deleyi
PB1_gene_2402	488	ORM77450.1	488	486/488(99.59)	0	lysine transporter [Pantoea deleyi]	470932	d__Bacteria;k__norank;p__Proteobacteria;c__Gammaproteobacteria;o__Enterobacterales;f__Erwiniaceae;g__Pantoea;s__Pantoea_deleyi
PB1_gene_2405	192	WP_111141812.1	203	187/193(96.89)	1.50e-136	cupin domain-containing protein [Pantoea sp. ARC607]	2027922	d__Bacteria;k__norank;p__Proteobacteria;c__Gammaproteobacteria;o__Enterobacterales;f__Erwiniaceae;g__Pantoea;s__Pantoea_sp._ARC607
PB1_gene_2407	130	OQV38822.1	130	130/130(100.00)	6.64e-87	hypothetical protein BZ160_19040 [Pantoea vagans]	470934	d__Bacteria;k__norank;p__Proteobacteria;c__Gammaproteobacteria;o__Enterobacterales;f__Erwiniaceae;g__Pantoea;s__Pantoea_vagans
PB1_gene_2408	281	ORM83053.1	274	274/274(100.00)	4.77e-173	2,3,4,5-tetrahydropyridine-2,6-dicarboxylate N-succinyltransferase [Pantoea	470932	d__Bacteria;k__norank;p__Proteobacteria;c__Gammaproteobacteria;o__Enterobacterales;f__Erwiniaceae;g__Pantoea;s__Pantoea_deleyi
PB1_gene_2411	80	ORM81597.1	80	73/78(93.59)	2.77e-49	hypothetical protein HA42_10275 [Pantoea deleyi]	470932	d__Bacteria;k__norank;p__Proteobacteria;c__Gammaproteobacteria;o__Enterobacterales;f__Erwiniaceae;g__Pantoea;s__Pantoea_deleyi
PB1_gene_2413	211	ORM81595.1	211	192/211(91.00)	1.97e-132	helix-turn-helix transcriptional regulator [Pantoea deleyi]	470932	d__Bacteria;k__norank;p__Proteobacteria;c__Gammaproteobacteria;o__Enterobacterales;f__Erwiniaceae;g__Pantoea;s__Pantoea_deleyi
PB1_gene_2415	395	WP_111141275.1	395	380/395(96.20)	9.38e-255	MFS transporter [Pantoea sp. ARC607]	2027922	d__Bacteria;k__norank;p__Proteobacteria;c__Gammaproteobacteria;o__Enterobacterales;f__Erwiniaceae;g__Pantoea;s__Pantoea_sp._ARC607
PB1_gene_2416	321	ORM76006.1	321	317/321(98.75)	3.04e-225	AraC family transcriptional regulator [Pantoea deleyi]	470932	d__Bacteria;k__norank;p__Proteobacteria;c__Gammaproteobacteria;o__Enterobacterales;f__Erwiniaceae;g__Pantoea;s__Pantoea_deleyi
PB1_gene_2417	664	ORM72642.1	664	655/664(98.64)	0	choline transporter [Pantoea deleyi]	470932	d__Bacteria;k__norank;p__Proteobacteria;c__Gammaproteobacteria;o__Enterobacterales;f__Erwiniaceae;g__Pantoea;s__Pantoea_deleyi
PB1_gene_2420	510	ORM80694.1	510	503/510(98.63)	0	NAD(P) transhydrogenase subunit alpha [Pantoea deleyi]	470932	d__Bacteria;k__norank;p__Proteobacteria;c__Gammaproteobacteria;o__Enterobacterales;f__Erwiniaceae;g__Pantoea;s__Pantoea_deleyi
PB1_gene_2422	222	ORM84445.1	222	214/222(96.40)	7.58e-165	haloacid dehalogenase, type II [Pantoea deleyi]	470932	d__Bacteria;k__norank;p__Proteobacteria;c__Gammaproteobacteria;o__Enterobacterales;f__Erwiniaceae;g__Pantoea;s__Pantoea_deleyi
PB1_gene_2425	408	WP_104189453.1	408	389/408(95.34)	4.26e-285	aminotransferase class I/II-fold pyridoxal phosphate-dependent enzyme [Pantoea sp. ICBG 828]	2071684	d__Bacteria;k__norank;p__Proteobacteria;c__Gammaproteobacteria;o__Enterobacterales;f__Erwiniaceae;g__Pantoea;s__Pantoea_sp._ICBG_828
PB1_gene_2428	346	ORM85292.1	346	342/346(98.84)	5.39e-243	GMP reductase [Pantoea deleyi]	470932	d__Bacteria;k__norank;p__Proteobacteria;c__Gammaproteobacteria;o__Enterobacterales;f__Erwiniaceae;g__Pantoea;s__Pantoea_deleyi

PB1_gene_2431	417	ORM78931.1	417	409/417(98.08)	3.82e-288	glutamate-5-semialdehyde dehydrogenase [Pantoea deleyi]	470932	d__Bacteria;k__norank;p__Proteobacteria;c__Gammaproteobacteria;o__Enterobacterales;f__Erwiniaceae;g__Pantoea;s__Pantoea_deleyi
PB1_gene_2435	284	ORM72598.1	284	279/284(98.24)	8.19e-200	oxidoreductase [Pantoea deleyi]	470932	d__Bacteria;k__norank;p__Proteobacteria;c__Gammaproteobacteria;o__Enterobacterales;f__Erwiniaceae;g__Pantoea;s__Pantoea_deleyi
PB1_gene_2438	381	ORM86536.1	381	370/381(97.11)	2.82e-274	glycosyl transferase [Pantoea deleyi]	470932	d__Bacteria;k__norank;p__Proteobacteria;c__Gammaproteobacteria;o__Enterobacterales;f__Erwiniaceae;g__Pantoea;s__Pantoea_deleyi
PB1_gene_2439	182	ORM86589.1	170	166/170(97.65)	1.77e-118	SprT family protein [Pantoea deleyi]	470932	d__Bacteria;k__norank;p__Proteobacteria;c__Gammaproteobacteria;o__Enterobacterales;f__Erwiniaceae;g__Pantoea;s__Pantoea_deleyi
PB1_gene_2440	127	OFC60740.1	232	59/117(50.43)	7.05e-27	hypothetical protein BBW68_14365 [Candidatus	252393	d__Bacteria;k__norank;p__Proteobacteria;c__Gammaproteobacteria;o__Enterobacterales;f__Erwiniaceae;g__Erwinia;s__Candidatus_Erwinia_dacicola
PB1_gene_2441	188	ORM86304.1	188	188/188(100.00)	1.19e-133	elongation factor P [Pantoea deleyi]	470932	d__Bacteria;k__norank;p__Proteobacteria;c__Gammaproteobacteria;o__Enterobacterales;f__Erwiniaceae;g__Pantoea;s__Pantoea_deleyi
PB1_gene_2442	43	ORM86305.1	43	43/43(100.00)	4.52e-21	EcnA [Pantoea deleyi]	470932	d__Bacteria;k__norank;p__Proteobacteria;c__Gammaproteobacteria;o__Enterobacterales;f__Erwiniaceae;g__Pantoea;s__Pantoea_deleyi
PB1_gene_2443	59	SUB08722.1	59	58/59(98.31)	1.06e-28	entericidin B membrane lipoprotein [Pantoea	549	d__Bacteria;k__norank;p__Proteobacteria;c__Gammaproteobacteria;o__Enterobacterales;f__Erwiniaceae;g__Pantoea;s__Pantoea_agglomerans
PB1_gene_2444	175	ORM86306.1	175	172/175(98.29)	1.12e-127	hypothetical protein HA42_00800 [Pantoea deleyi]	470932	d__Bacteria;k__norank;p__Proteobacteria;c__Gammaproteobacteria;o__Enterobacterales;f__Erwiniaceae;g__Pantoea;s__Pantoea_deleyi
PB1_gene_2448	460	WP_111209109.1	460	404/460(87.83)	2.81e-282	DHA2 family efflux MFS transporter permease subunit [Pantoea sp. ARC270]	53335	d__Bacteria;k__norank;p__Proteobacteria;c__Gammaproteobacteria;o__Enterobacterales;f__Erwiniaceae;g__Pantoea;s__norank
PB1_gene_2449	164	ORM85819.1	157	138/157(87.90)	3.47e-81	hypothetical protein HA42_01815 [Pantoea deleyi]	470932	d__Bacteria;k__norank;p__Proteobacteria;c__Gammaproteobacteria;o__Enterobacterales;f__Erwiniaceae;g__Pantoea;s__Pantoea_deleyi
PB1_gene_2450	279	ORM85818.1	278	270/278(97.12)	8.52e-197	sugar-phosphatase [Pantoea deleyi]	470932	d__Bacteria;k__norank;p__Proteobacteria;c__Gammaproteobacteria;o__Enterobacterales;f__Erwiniaceae;g__Pantoea;s__Pantoea_deleyi
PB1_gene_2453	199	ORM75454.1	199	195/199(97.99)	5.33e-131	FMN-dependent NADH-azoreductase [Pantoea deleyi]	470932	d__Bacteria;k__norank;p__Proteobacteria;c__Gammaproteobacteria;o__Enterobacterales;f__Erwiniaceae;g__Pantoea;s__Pantoea_deleyi
PB1_gene_2455	157	WP_111138460.1	157	151/157(96.18)	1.91e-99	OmpA family protein [Pantoea sp. ARC607]	2027922	d__Bacteria;k__norank;p__Proteobacteria;c__Gammaproteobacteria;o__Enterobacterales;f__Erwiniaceae;g__Pantoea;s__Pantoea_sp._ARC607
PB1_gene_2456	401	ORM83251.1	401	384/401(95.76)	2.89e-268	hypothetical protein HA42_06600 [Pantoea deleyi]	470932	d__Bacteria;k__norank;p__Proteobacteria;c__Gammaproteobacteria;o__Enterobacterales;f__Erwiniaceae;g__Pantoea;s__Pantoea_deleyi
PB1_gene_2457	186	ORM83250.1	186	164/186(88.17)	5.80e-115	hypothetical protein HA42_06595 [Pantoea deleyi]	470932	d__Bacteria;k__norank;p__Proteobacteria;c__Gammaproteobacteria;o__Enterobacterales;f__Erwiniaceae;g__Pantoea;s__Pantoea_deleyi
PB1_gene_2466	237	WP_090963824.1	226	205/226(90.71)	3.18e-146	pilus assembly protein PapD [Pantoea sp. OV426]	1881036	d__Bacteria;k__norank;p__Proteobacteria;c__Gammaproteobacteria;o__Enterobacterales;f__Erwiniaceae;g__Pantoea;s__Pantoea_sp._OV426
PB1_gene_2470	291	ORM80504.1	291	288/291(98.97)	1.47e-201	sulfate ABC transporter permease subunit CysW [Pantoea deleyi]	470932	d__Bacteria;k__norank;p__Proteobacteria;c__Gammaproteobacteria;o__Enterobacterales;f__Erwiniaceae;g__Pantoea;s__Pantoea_deleyi
PB1_gene_2473	127	ORM75488.1	127	108/127(85.04)	6.95e-74	hypothetical protein HA42_20540 [Pantoea deleyi]	470932	d__Bacteria;k__norank;p__Proteobacteria;c__Gammaproteobacteria;o__Enterobacterales;f__Erwiniaceae;g__Pantoea;s__Pantoea_deleyi
PB1_gene_2478	215	WP_128086410.1	215	212/215(98.60)	5.24e-139	two-component system response regulator NarL [Pantoea deleyi]	470932	d__Bacteria;k__norank;p__Proteobacteria;c__Gammaproteobacteria;o__Enterobacterales;f__Erwiniaceae;g__Pantoea;s__Pantoea_deleyi
PB1_gene_2482	150	ORM85231.1	232	138/149(92.62)	7.71e-91	methyltransferase [Pantoea deleyi]	470932	d__Bacteria;k__norank;p__Proteobacteria;c__Gammaproteobacteria;o__Enterobacterales;f__Erwiniaceae;g__Pantoea;s__Pantoea_deleyi
PB1_gene_2485	230	WP_109651999.1	226	177/226(78.32)	3.68e-118	fimbrial chaperone protein [Pantoea allii]	None	Unclassified
PB1_gene_2487	400	OQV40581.1	379	362/379(95.51)	1.41e-251	glycerate kinase [Pantoea vagans]	470934	d__Bacteria;k__norank;p__Proteobacteria;c__Gammaproteobacteria;o__Enterobacterales;f__Erwiniaceae;g__Pantoea;s__Pantoea_vagans
PB1_gene_2496	160	SJZ30312.1	180	153/159(96.23)	4.25e-102	Lrp/AsnC family transcriptional regulator [Pantoea agglomerans]	470933	d__Bacteria;k__norank;p__Proteobacteria;c__Gammaproteobacteria;o__Enterobacterales;f__Erwiniaceae;g__Pantoea;s__Pantoea_eucalypti
PB1_gene_2498	97	OQV39818.1	97	97/97(100.00)	1.96e-59	RNA-binding protein [Pantoea vagans]	470934	d__Bacteria;k__norank;p__Proteobacteria;c__Gammaproteobacteria;o__Enterobacterales;f__Erwiniaceae;g__Pantoea;s__Pantoea_vagans

PB1_gene_2499	158	ORM86340.1	158	158/158(100.00)	5.47e-104	transcription elongation factor GreA [Pantoea deleyi]	470932	d__Bacteria;k__norank;p__Proteobacteria;c__Gammaproteobacteria;o__Enterobacterales;f__Erwiniaceae;g__Pantoea;s__Pantoea_deleyi
PB1_gene_2503	327	WP_111140980.1	327	313/327(95.72)	2.80e-224	XdhC family protein [Pantoea sp. ARC607]	2027922	d__Bacteria;k__norank;p__Proteobacteria;c__Gammaproteobacteria;o__Enterobacterales;f__Erwiniaceae;g__Pantoea;s__Pantoea_sp._ARC607
PB1_gene_2509	115	ORM83591.1	115	109/115(94.78)	7.31e-68	hypothetical protein HA42_05960 [Pantoea deleyi]	470932	d__Bacteria;k__norank;p__Proteobacteria;c__Gammaproteobacteria;o__Enterobacterales;f__Erwiniaceae;g__Pantoea;s__Pantoea_deleyi
PB1_gene_2512	237	PQK91705.1	237	196/237(82.70)	4.21e-133	hypothetical protein CG432_01810 [Pantoea ananatis]	553	d__Bacteria;k__norank;p__Proteobacteria;c__Gammaproteobacteria;o__Enterobacterales;f__Erwiniaceae;g__Pantoea;s__Pantoea_ananatis
PB1_gene_2517	140	ADO08506.1	140	133/140(95.00)	6.44e-92	Uncharacterized protein ydeI precursor [Pantoea vagans C9-1]	712898	d__Bacteria;k__norank;p__Proteobacteria;c__Gammaproteobacteria;o__Enterobacterales;f__Erwiniaceae;g__Pantoea;s__Pantoea_vagans
PB1_gene_2518	355	ORM78944.1	320	319/320(99.69)	2.70e-223	chemotaxis protein CheV [Pantoea deleyi]	470932	d__Bacteria;k__norank;p__Proteobacteria;c__Gammaproteobacteria;o__Enterobacterales;f__Erwiniaceae;g__Pantoea;s__Pantoea_deleyi
PB1_gene_2519	44	PXW21322.1	49	37/49(75.51)	8.09e-14	hypothetical protein BY447_3018 [Pantoea sp. JKS000250]	1938795	d__Bacteria;k__norank;p__Proteobacteria;c__Gammaproteobacteria;o__Enterobacterales;f__Erwiniaceae;g__Pantoea;s__Pantoea_sp._JKS000250
PB1_gene_2521	433	ORM81315.1	433	432/433(99.77)	2.91e-315	D-amino acid dehydrogenase small subunit [Pantoea deleyi]	470932	d__Bacteria;k__norank;p__Proteobacteria;c__Gammaproteobacteria;o__Enterobacterales;f__Erwiniaceae;g__Pantoea;s__Pantoea_deleyi
PB1_gene_2523	203	ORM72662.1	202	185/202(91.58)	5.50e-128	thymidylate kinase [Pantoea deleyi]	470932	d__Bacteria;k__norank;p__Proteobacteria;c__Gammaproteobacteria;o__Enterobacterales;f__Erwiniaceae;g__Pantoea;s__Pantoea_deleyi
PB1_gene_2527	247	ORM85290.1	247	244/247(98.79)	9.90e-178	cell division protein ZapD [Pantoea deleyi]	470932	d__Bacteria;k__norank;p__Proteobacteria;c__Gammaproteobacteria;o__Enterobacterales;f__Erwiniaceae;g__Pantoea;s__Pantoea_deleyi
PB1_gene_2528	65	WP_111139416.1	65	64/65(98.46)	6.06e-44	DNA gyrase inhibitor YacG [Pantoea sp. ARC607]	2027922	d__Bacteria;k__norank;p__Proteobacteria;c__Gammaproteobacteria;o__Enterobacterales;f__Erwiniaceae;g__Pantoea;s__Pantoea_sp._ARC607
PB1_gene_2529	131	WP_111139415.1	131	129/131(98.47)	3.83e-91	8-oxo-dGTP diphosphatase MutT [Pantoea sp. ARC607]	2027922	d__Bacteria;k__norank;p__Proteobacteria;c__Gammaproteobacteria;o__Enterobacterales;f__Erwiniaceae;g__Pantoea;s__Pantoea_sp._ARC607
PB1_gene_2540	209	ORM84709.1	209	205/209(98.09)	2.54e-143	competence protein TfoX [Pantoea deleyi]	470932	d__Bacteria;k__norank;p__Proteobacteria;c__Gammaproteobacteria;o__Enterobacterales;f__Erwiniaceae;g__Pantoea;s__Pantoea_deleyi
PB1_gene_2542	532	ORM80631.1	532	522/532(98.12)	0	gamma-glutamyltransferase [Pantoea deleyi]	470932	d__Bacteria;k__norank;p__Proteobacteria;c__Gammaproteobacteria;o__Enterobacterales;f__Erwiniaceae;g__Pantoea;s__Pantoea_deleyi
PB1_gene_2545	419	WP_111138521.1	419	404/419(96.42)	1.80e-303	c-type cytochrome [Pantoea sp. ARC607]	2027922	d__Bacteria;k__norank;p__Proteobacteria;c__Gammaproteobacteria;o__Enterobacterales;f__Erwiniaceae;g__Pantoea;s__Pantoea_sp._ARC607
PB1_gene_2547	382	ORM75943.1	383	365/382(95.55)	2.62e-257	alcohol dehydrogenase [Pantoea deleyi]	470932	d__Bacteria;k__norank;p__Proteobacteria;c__Gammaproteobacteria;o__Enterobacterales;f__Erwiniaceae;g__Pantoea;s__Pantoea_deleyi
PB1_gene_2550	385	WP_111140579.1	385	375/385(97.40)	9.46e-275	amidohydrolase [Pantoea sp. ARC607]	2027922	d__Bacteria;k__norank;p__Proteobacteria;c__Gammaproteobacteria;o__Enterobacterales;f__Erwiniaceae;g__Pantoea;s__Pantoea_sp._ARC607
PB1_gene_2552	251	WP_111140647.1	251	249/251(99.20)	4.85e-171	metalloprotease [Pantoea sp. ARC607]	2027922	d__Bacteria;k__norank;p__Proteobacteria;c__Gammaproteobacteria;o__Enterobacterales;f__Erwiniaceae;g__Pantoea;s__Pantoea_sp._ARC607
PB1_gene_2557	68	ABU77897.1	545	34/61(55.74)	4.82e-13	hypothetical protein ESA_02657 [Cronobacter sakazakii ATCC BAA-894]	290339	d__Bacteria;k__norank;p__Proteobacteria;c__Gammaproteobacteria;o__Enterobacterales;f__Enterobacteriaceae;g__Cronobacter;s__Cronobacter_sakazakii
PB1_gene_2566	171	ORM80565.1	171	171/171(100.00)	6.27e-123	S-ribosylhomocysteine lyase [Pantoea deleyi]	470932	d__Bacteria;k__norank;p__Proteobacteria;c__Gammaproteobacteria;o__Enterobacterales;f__Erwiniaceae;g__Pantoea;s__Pantoea_deleyi
PB1_gene_2567	428	ORM80564.1	428	426/427(99.77)	1.77e-294	magnesium/cobalt efflux protein [Pantoea deleyi]	470932	d__Bacteria;k__norank;p__Proteobacteria;c__Gammaproteobacteria;o__Enterobacterales;f__Erwiniaceae;g__Pantoea;s__Pantoea_deleyi
PB1_gene_2569	73	ORM81411.1	73	72/73(98.63)	3.58e-42	hypothetical protein HA42_10775 [Pantoea deleyi]	470932	d__Bacteria;k__norank;p__Proteobacteria;c__Gammaproteobacteria;o__Enterobacterales;f__Erwiniaceae;g__Pantoea;s__Pantoea_deleyi
PB1_gene_2570	348	ORM81410.1	348	327/348(93.97)	5.30e-235	recombinase Cre [Pantoea deleyi]	470932	d__Bacteria;k__norank;p__Proteobacteria;c__Gammaproteobacteria;o__Enterobacterales;f__Erwiniaceae;g__Pantoea;s__Pantoea_deleyi
PB1_gene_2571	410	WP_111139220.1	542	400/410(97.56)	1.30e-294	isovaleryl-CoA dehydrogenase [Pantoea sp. ARC607]	2027922	d__Bacteria;k__norank;p__Proteobacteria;c__Gammaproteobacteria;o__Enterobacterales;f__Erwiniaceae;g__Pantoea;s__Pantoea_sp._ARC607

PB1_gene_2572	244	ORM79168.1	244	243/244(99.59)	3.85e-169	23S rRNA (guanosine(2251)-2'-O)-methyltransferase RlmB [Pantoea deleyi]	470932	d__Bacteria;k__norank;p__Proteobacteria;c__Gammaproteobacteria;o__Enterobacterales;f__Erwiniaceae;g__Pantoea;s__Pantoea_deleyi
PB1_gene_2575	189	WP_111140359.1	210	181/187(96.79)	1.19e-111	acetyltransferase [Pantoea sp. ARC607]	2027922	d__Bacteria;k__norank;p__Proteobacteria;c__Gammaproteobacteria;o__Enterobacterales;f__Erwiniaceae;g__Pantoea;s__Pantoea_sp._ARC607
PB1_gene_2576	87	WP_078804396.1	88	55/88(62.50)	4.27e-27	hypothetical protein [Pantoea agglomerans]	470933	d__Bacteria;k__norank;p__Proteobacteria;c__Gammaproteobacteria;o__Enterobacterales;f__Erwiniaceae;g__Pantoea;s__Pantoea_eucalypti
PB1_gene_2582	270	WP_122890008.1	270	259/270(95.93)	1.18e-176	DUF4225 domain-containing protein [[Curtobacterium]	221276	d__Bacteria;k__norank;p__Proteobacteria;c__Gammaproteobacteria;o__Enterobacterales;f__Erwiniaceae;g__Pantoea;s__[Curtobacterium]_plantarum
PB1_gene_2583	104	WP_122890009.1	106	101/103(98.06)	9.60e-66	hypothetical protein [[Curtobacterium] plantarum]	221276	d__Bacteria;k__norank;p__Proteobacteria;c__Gammaproteobacteria;o__Enterobacterales;f__Erwiniaceae;g__Pantoea;s__[Curtobacterium]_plantarum
PB1_gene_2591	645	ORM81332.1	699	636/645(98.60)	0	ferrioxamine B receptor [Pantoea deleyi]	470932	d__Bacteria;k__norank;p__Proteobacteria;c__Gammaproteobacteria;o__Enterobacterales;f__Erwiniaceae;g__Pantoea;s__Pantoea_deleyi
PB1_gene_2593	97	ORM80801.1	407	95/96(98.96)	5.22e-57	bifunctional cysteine desulfurase/selenocysteine lyase [Pantoea deleyi]	470932	d__Bacteria;k__norank;p__Proteobacteria;c__Gammaproteobacteria;o__Enterobacterales;f__Erwiniaceae;g__Pantoea;s__Pantoea_deleyi
PB1_gene_2594	426	ORM80789.1	426	420/426(98.59)	3.12e-303	FeS cluster assembly protein SufD [Pantoea deleyi]	470932	d__Bacteria;k__norank;p__Proteobacteria;c__Gammaproteobacteria;o__Enterobacterales;f__Erwiniaceae;g__Pantoea;s__Pantoea_deleyi
PB1_gene_2597	318	ORM83041.1	318	317/318(99.69)	8.41e-226	acetyl-CoA carboxylase carboxyltransferase subunit alpha [Pantoea deleyi]	470932	d__Bacteria;k__norank;p__Proteobacteria;c__Gammaproteobacteria;o__Enterobacterales;f__Erwiniaceae;g__Pantoea;s__Pantoea_deleyi
PB1_gene_2599	363	WP_111138404.1	360	352/360(97.78)	6.41e-252	sn-glycerol-3-phosphate ABC transporter ATP-binding protein UgpC [Pantoea sp. ARC607]	2027922	d__Bacteria;k__norank;p__Proteobacteria;c__Gammaproteobacteria;o__Enterobacterales;f__Erwiniaceae;g__Pantoea;s__Pantoea_sp._ARC607
PB1_gene_2601	81	ORM85816.1	81	80/81(98.77)	1.26e-50	hypothetical protein HA42_01795 [Pantoea deleyi]	470932	d__Bacteria;k__norank;p__Proteobacteria;c__Gammaproteobacteria;o__Enterobacterales;f__Erwiniaceae;g__Pantoea;s__Pantoea_deleyi

Notes: Query_ID: Unique identifier of the input query sequence. Query_length: Full-length of the query sequence (in nucleotides for DNA sequences). Subject_ID: Accession number or unique identifier of the matched database sequence (hit). Subject_length: Full-length of the subject sequence. Identity: Percentage of identical residues between the query and subject sequences over the aligned region (calculated as identical positions / alignment length × 100%). *E*_value (Expect value): The statistical significance of the alignment; the number of alignments with a similar score that would be expected to occur by chance in a database search. Lower values indicate higher confidence. Description: Functional annotation or brief textual description of the subject sequence. Taxonomy_ID: NCBI Taxonomy identifier (taxid) representing the taxonomic classification of the organism from which the subject sequence originates.

Table S6 Relative abundance of KEGG Level 1 pathways across geographic populations of *Lissorhoptrus oryzophilu* , normalized within each group

KEGG Level 1 ID	MDJ	PB	SC
Environmental Information Processing	0.493065710704574	0.311747217339046	0.485078910341464
Metabolism	0.16703905631336	0.360875123404667	0.168117792217108
Organismal Systems	0.12993667044917	0.0514208609303563	0.125829078447011
Genetic Information Processing	0.101781879620604	0.102500721058414	0.0734863743523562
Human Diseases	0.0616078070867553	0.0785312739980289	0.0893231000556599
Cellular Processes	0.0465688758255359	0.0949248032694877	0.0581647445864006

Note: Values represent the relative abundance of KEGG-annotated genes within each of the six Level 1 functional categories (i.e., Environmental Information Processing, Metabolism, etc.). Abundances were normalized within each population (MDJ, Mudanjiang, Heilongjiang; PB, Pingba, Guizhou; SC, Yibin, Sichuan). Within each population, the proportions collectively sum to 1.

Table S7 Relative abundance of genes assigned to 45 KEGG Level 2 pathways across geographic populations of *Lissorhoptrus oryzophilus*

KEGG Level 2 ID	MDJ	PB	SC
Aging	0.00240451783217465	0.00239292613282346	0.00216623767052518
Amino acid metabolism	0.0135820875467615	0.0297642126664381	0.0126751985061506
Biosynthesis of other secondary metabolites	0.00274869476958761	0.00622362519497465	0.00289427449767596
Cancers: Overview	0.00537908479556294	0.00516939211715647	0.00790816736793102
Cancers: Specific types	0.00202568549368357	0.00198014581790112	0.0033431098313672
Carbohydrate metabolism	0.0144680607142919	0.0388284781245931	0.0139454851965746
Cardiovascular diseases	0.00369593047567713	0.00309734889730214	0.00612112081714582
Cell growth and death	0.00430484807624687	0.00500962465081856	0.00409941799668282
Cell motility	0.00196107374272392	0.00903642837395304	0.00239719055274589
Cellular community - eukaryotes	0.00220360071522555	0.0014894242121145	0.00394439726999999
Cellular community - prokaryotes	0.00519019692672568	0.0170936983286967	0.00471080842702224
Circulatory system	0.00144525773514426	0.00113089983010045	0.00248876528674389
Development	0.00172456227364029	0.00115329261118016	0.00329346438357462
Digestive system	0.00088619224129545	0.000711562430203265	0.00159932085577686
Drug resistance: Antimicrobial	0.00317384479193512	0.00863244405298933	0.00240169578609987
Drug resistance: Antineoplastic	0.00169156938872326	0.00208418198334327	0.00176095098476114
Endocrine and metabolic diseases	0.00325658234921497	0.00300704010105173	0.00398481742046609
Endocrine system	0.00329742989314594	0.00408239611036814	0.00542044253846741
Energy metabolism	0.009898741574139	0.0224509224595889	0.00877966022392514
Environmental adaptation	0.00239818540445297	0.0029217645193191	0.0030479205055892
Excretory system	0.00072748471442943	0.000809647498232053	0.0014264748410245
Folding, sorting and degradation	0.00676589087218376	0.00913234735554137	0.00513946561767857
Glycan biosynthesis and metabolism	0.00480613124389257	0.00874028327592362	0.00381021499344971
Immune diseases	0.000376299296915768	0.00026319438368171	0.000457982609939928
Immune system	0.00265428693278679	0.00220519546102881	0.00457722335347673
Infectious diseases: Bacterial	0.00518517114149894	0.0079847482223502	0.00845209256900776
Infectious diseases: Parasitic	0.000592586348848187	0.000537484343313876	0.0010622610160423
Infectious diseases: Viral	0.00222267758812077	0.00157684833491085	0.00340276022126563
Lipid metabolism	0.0040404058989801	0.00873234189637096	0.00384662448756785
Membrane transport	0.147662027579681	0.0870860604841647	0.130617888583917
Metabolism of cofactors and vitamins	0.0109983568253695	0.0241618260941086	0.00986557905475451

Metabolism of other amino acids	0.00477378484232168	0.0100645739165551	0.00363733790989572
Metabolism of terpenoids and polyketides	0.00235143062983984	0.0050701478374483	0.00222540691073157
Nervous system	0.00215240619851796	0.00178996321185984	0.00376579858154685
Neurodegenerative diseases	0.00251275059726974	0.00244899041141992	0.0037095432428629
Nucleotide metabolism	0.00619538986660679	0.0153539250197327	0.00547988025925205
Replication and repair	0.0085258119453556	0.0105455597177322	0.00597919808082401
Sensory system	0.031670072583731	0.00723756913817556	0.0234676677728443
Signal transduction	0.0112760114374593	0.0233086414815117	0.0142635068350586
Signaling molecules and interaction	0.000515188255360735	0.000269689868133162	0.000631417151759387
Substance dependence	0.00111640964109151	0.000804817272245523	0.00198958070611452
Transcription	0.00183672539352961	0.00120419213283248	0.00106777045253965
Translation	0.0155714982503131	0.0151471895860429	0.00984870056168896
Transport and catabolism	0.00272773563378538	0.00271824357685575	0.00477261943342831
Xenobiotics biodegradation and metabolism	0.00175569036916862	0.00538372765692355	0.00160643461696141
Others	0.635251629172592	0.581162983207985	0.647914124017142

Note: Values represent the relative abundance of genes annotated to the 45 KEGG Level 2 pathways. MDJ, PB, and SC indicate the three geographic populations of *Lissorhoptrus oryzophilus* : MDJ, Mudanjiang (Heilongjiang); PB, Pingba (Guizhou); SC, Yibin (Sichuan).

Table S8 Relative abundance of eggNOG functional categories across geographic populations of *Lissorhoptrus oryzophilus* , normalized within each group

ID	Description	MDJ	PB	SC
S	Function unknown	0.327275142943613	0.289832398541346	0.375672151059203
U	Intracellular trafficking, secretion, and vesicular transport	0.250579170749986	0.0758048481038591	0.206912744714069
L	Replication, recombination and repair	0.0589331929461148	0.0568750602181354	0.0606408921466213
K	Transcription	0.0425341222243148	0.066444194111117	0.0582221846798111
E	Amino acid transport and metabolism	0.0357355475529703	0.0717220257406976	0.0357047773905622
J	Translation, ribosomal structure and biogenesis	0.0576559342253486	0.0466040218576003	0.0380332034412096
G	Carbohydrate transport and metabolism	0.027095254402451	0.0650754786974114	0.0278036323955648
M	Cell wall/membrane/envelope biogenesis	0.0289325738907326	0.0527582494526058	0.0231151199802555
P	Inorganic ion transport and metabolism	0.0241998951539233	0.0525135390677865	0.0236856273655569
C	Energy production and conversion	0.0297483242545857	0.0471219740582886	0.026106228046223
O	Posttranslational modification, protein turnover, chaperones	0.0341202441015726	0.0348509548258841	0.0306634469237142
T	Signal transduction mechanisms	0.016702023376291	0.0316528222848832	0.0200288977536942
H	Coenzyme transport and metabolism	0.0172419680776842	0.0275800987208282	0.0159309893505589
F	Nucleotide transport and metabolism	0.0140475158888588	0.0220921480477238	0.0123913887233978
I	Lipid transport and metabolism	0.011628917090478	0.0187022255549502	0.0118232270869829
D	Cell cycle control, cell division, chromosome partitioning	0.00775398143920999	0.010174566501079	0.0134542115090623
N	Cell motility	0.00354080793721674	0.0115284214368665	0.00361933270136764
Q	Secondary metabolites biosynthesis, transport and catabolism	0.00366632019202923	0.00824389718462423	0.00459669015473757
V	Defense mechanisms	0.00368141224834764	0.00842382191964768	0.00404603036642366
Z	Cytoskeleton	0.0029987529695634	0.00110079452720879	0.00464074185010215
B	Chromatin structure and dynamics	0.00155069209912162	0.000596085941718101	0.00219539292444734
A	RNA processing and modification	0.000378206235585956	0.000302373205738292	0.000713089436435023

Notes: ID: The single-letter code for each functional category in the eggNOG/COG classification system. Description: The corresponding functional definition. Relative abundance of eggNOG-annotated functional categories across geographic populations (MDJ, PB, SC). Abundances were normalized to 1 per sample to facilitate cross-population comparison of functional profiles. MDJ, PB, and SC indicate the three geographic populations of *Lissorhoptrus oryzophilus* : MDJ, Mudanjiang (Heilongjiang); PB, Pingba (Guizhou); SC, Yibin (Sichuan).

Table S9 Relative abundance of five CAZy classes across geographic populations of *Lissorhoptrus oryzophilus* , normalized within each group

ID	MDJ	PB	SC
GTs:GlycosylTransferases	0.476593270542413	0.418119451160732	0.494761523695746
GHs:Glycoside Hydrolases	0.393518863444537	0.436345212810011	0.373392651933628
CEs:Carbohydrate Esterases	0.0708507862783273	0.0557244146453114	0.0537626028502447
AAs:Auxiliary Activities	0.0337991057682011	0.0555110952690804	0.0511821430057788
CBMs:Carbohydrate-Binding Modules	0.0252379739665221	0.0342998261148652	0.0269010785146023

Notes: ID indicates the five primary CAZy classes (GH, GT, CE, CBM, AA). Values are the proportion of genes in each class relative to the total CAZy-annotated genes per sample; proportions within each sample sum to 1. MDJ, PB, and SC indicate the three geographic populations of *Lissorhoptrus oryzophilus* : MDJ, Mudanjiang (Heilongjiang); PB, Pingba (Guizhou); SC, Yibin (Sichuan).

Table S10 Relative abundance of all identified CAZy families across geographic populations of *Lissorhoptrus oryzophilus*

ID	MDJ	PB	SC	Description
AA3	0.000111089207974793	0.000619989145414633	0.000150759043455506	cellobiose dehydrogenase (EC 1.1.99.18); glucose 1-oxidase (EC 1.1.3.4); aryl alcohol oxidase (EC 1.1.3.7); alcohol oxidase (EC 1.1.3.13);
AA6	8.78136548149157e-06	0.000126536402011373	3.26973803773197e-05	1,4-benzoquinone reductase (EC. 1.6.5.6)
CBM48	8.95080015722789e-05	0.000461271685289172	9.64237793047814e-05	Modules of approx. 100 residues with glycogen-binding function, appended to GH13 modules. Also found in the beta subunit (glycogen-binding) of AMP-activated protein kinases (AMPK)
CE1	2.80266299267985e-05	0.000131680782688868	3.30978908391089e-05	acetyl xylan esterase (EC 3.1.1.72); cinnamoyl esterase (EC 3.1.1.-); feruloyl esterase (EC 3.1.1.73); carboxylesterase (EC 3.1.1.1); S-formylglutathione hydrolase (EC 3.1.2.12); diacylglycerol O-acyltransferase (EC 2.3.1.20); trehalose 6-O-mycolyltransferase (EC 2.3.1.122)
CE10	6.12815387223098e-05	0.000268783960819291	3.58373635570437e-05	arylesterase (EC 3.1.1.-); carboxyl esterase (EC 3.1.1.3); acetylcholinesterase (EC 3.1.1.7); cholinesterase (EC 3.1.1.8); sterol esterase (EC
CE11	3.83354878557829e-05	0.000151851190379202	2.27811077249339e-05	UDP-3-0-acyl N-acetylglucosamine deacetylase (EC 3.5.1.-).
CE4	9.2109994775478e-05	4.89929035797785e-05	6.81803229762237e-05	acetyl xylan esterase (EC 3.1.1.72); chitin deacetylase (EC 3.5.1.41); chitooligosaccharide deacetylase (EC 3.5.1.-); peptidoglycan GlcNAc deacetylase (EC 3.5.1.-); peptidoglycan N-acetylmuramic acid deacetylase (EC 3.5.1.-).
CE9	3.15229487355407e-05	0.000148085476344134	3.28090961697366e-05	N-acetylglucosamine 6-phosphate deacetylase (EC 3.5.1.25); N-acetylglucosamine 6-phosphate deacetylase (EC 3.5.1.80)
GH1	0.000123535346880838	0.000613223625679231	0.000119960961135793	β-glucosidase (EC 3.2.1.21); β-galactosidase (EC 3.2.1.23); β-mannosidase (EC 3.2.1.25); β-glucuronidase (EC 3.2.1.31); β-xylosidase (EC 3.2.1.37); β-D-fucosidase (EC 3.2.1.38); phlorizin hydrolase (EC 3.2.1.62); exo-β-1,4-glucanase (EC 3.2.1.74); 6-phospho-β-galactosidase (EC 3.2.1.85); 6-phospho-β-glucosidase (EC 3.2.1.86); strictosidine β-glucosidase (EC 3.2.1.105); lactase (EC 3.2.1.108); amygdalin β-glucosidase (EC 3.2.1.117); prunasin β-glucosidase (EC 3.2.1.118); vicianin hydrolase (EC 3.2.1.119); raucaffricine β-glucosidase (EC 3.2.1.125); thioglucosidase (EC 3.2.1.147); β-primeverosidase (EC 3.2.1.149); isoflavonoid 7-O-β-apiosyl-β-glucosidase (EC 3.2.1.161); ABA-specific β-glucosidase (EC
GH102	2.49077476694392e-05	0.000114122754184584	2.5250407681438e-05	peptidoglycan lytic transglycosylase (EC 3.2.1.-)
GH103	4.24549035929706e-05	0.000159468899247175	2.27496605814761e-05	peptidoglycan lytic transglycosylase (EC 3.2.1.-)
GH105	2.10866892011852e-05	0.000124958856945546	2.87707789617423e-05	unsaturated rhamnogalacturonyl hydrolase (EC 3.2.1.172); d-4,5-unsaturated β-glucuronyl hydrolase (EC 3.2.1.-); d-4,5-unsaturated α-
GH13_10	2.60856620053565e-05	0.000130114028001768	2.99878502472726e-05	α-amylase (EC 3.2.1.1); pullulanase (EC 3.2.1.41); cyclomaltodextrin glucanotransferase (EC 2.4.1.19); cyclomaltodextrinase (EC 3.2.1.54); trehalose-6-phosphate hydrolase (EC 3.2.1.93); oligo-α-glucosidase (EC 3.2.1.10); maltogenic amylase (EC 3.2.1.133); neopullulanase (EC 3.2.1.135); α-glucosidase (EC 3.2.1.20); maltotetraose-forming α-amylase (EC 3.2.1.60); isoamylase (EC 3.2.1.68); glucodextranase (EC 3.2.1.70); maltohexaose-forming α-amylase (EC 3.2.1.98); maltotriose-forming α-amylase (EC 3.2.1.116); branching enzyme (EC 2.4.1.18); trehalose synthase (EC 5.4.99.16); 4-α-glucanotransferase (EC 2.4.1.25); maltopentaose-forming α-amylase (EC 3.2.1.-) ; amylosucrase (EC 2.4.1.4) ; sucrose phosphorylase (EC 2.4.1.7); malto-oligosyltrehalose trehalohydrolase (EC 3.2.1.141); isomaltulose synthase (EC 5.4.99.11); malto-oligosyltrehalose
GH13_11	4.66791599879462e-05	0.000285696775717547	6.36686308676477e-05	α-amylase (EC 3.2.1.1); pullulanase (EC 3.2.1.41); cyclomaltodextrin glucanotransferase (EC 2.4.1.19); cyclomaltodextrinase (EC 3.2.1.54); trehalose-6-phosphate hydrolase (EC 3.2.1.93); oligo-α-glucosidase (EC 3.2.1.10); maltogenic amylase (EC 3.2.1.133); neopullulanase (EC 3.2.1.135); α-glucosidase (EC 3.2.1.20); maltotetraose-forming α-amylase (EC 3.2.1.60); isoamylase (EC 3.2.1.68); glucodextranase (EC 3.2.1.70); maltohexaose-forming α-amylase (EC 3.2.1.98); maltotriose-forming α-amylase (EC 3.2.1.116); branching enzyme (EC 2.4.1.18); trehalose synthase (EC 5.4.99.16); 4-α-glucanotransferase (EC 2.4.1.25); maltopentaose-forming α-amylase (EC 3.2.1.-) ; amylosucrase (EC 2.4.1.4) ; sucrose phosphorylase (EC 2.4.1.7); malto-oligosyltrehalose trehalohydrolase (EC 3.2.1.141); isomaltulose synthase (EC 5.4.99.11); malto-oligosyltrehalose
GH13_18	3.44677995953462e-05	0.000157423798072931	4.22917882834844e-05	α-amylase (EC 3.2.1.1); pullulanase (EC 3.2.1.41); cyclomaltodextrin glucanotransferase (EC 2.4.1.19); cyclomaltodextrinase (EC 3.2.1.54); trehalose-6-phosphate hydrolase (EC 3.2.1.93); oligo-α-glucosidase (EC 3.2.1.10); maltogenic amylase (EC 3.2.1.133); neopullulanase (EC 3.2.1.135); α-glucosidase (EC 3.2.1.20); maltotetraose-forming α-amylase (EC 3.2.1.60); isoamylase (EC 3.2.1.68); glucodextranase (EC 3.2.1.70); maltohexaose-forming α-amylase (EC 3.2.1.98); maltotriose-forming α-amylase (EC 3.2.1.116); branching enzyme (EC 2.4.1.18); trehalose synthase (EC 5.4.99.16); 4-α-glucanotransferase (EC 2.4.1.25); maltopentaose-forming α-amylase (EC 3.2.1.-) ; amylosucrase (EC 2.4.1.4) ; sucrose phosphorylase (EC 2.4.1.7); malto-oligosyltrehalose trehalohydrolase (EC 3.2.1.141); isomaltulose synthase (EC 5.4.99.11); malto-oligosyltrehalose
GH13_26	3.1381001835733e-05	0.000139689838533199	2.03646083839734e-05	α-amylase (EC 3.2.1.1); pullulanase (EC 3.2.1.41); cyclomaltodextrin glucanotransferase (EC 2.4.1.19); cyclomaltodextrinase (EC 3.2.1.54); trehalose-6-phosphate hydrolase (EC 3.2.1.93); oligo-α-glucosidase (EC 3.2.1.10); maltogenic amylase (EC 3.2.1.133); neopullulanase (EC 3.2.1.135); α-glucosidase (EC 3.2.1.20); maltotetraose-forming α-amylase (EC 3.2.1.60); isoamylase (EC 3.2.1.68); glucodextranase (EC 3.2.1.70); maltohexaose-forming α-amylase (EC 3.2.1.98); maltotriose-forming α-amylase (EC 3.2.1.116); branching enzyme (EC 2.4.1.18); trehalose synthase (EC 5.4.99.16); 4-α-glucanotransferase (EC 2.4.1.25); maltopentaose-forming α-amylase (EC 3.2.1.-) ; amylosucrase (EC 2.4.1.4) ; sucrose phosphorylase (EC 2.4.1.7); malto-oligosyltrehalose trehalohydrolase (EC 3.2.1.141); isomaltulose synthase (EC 5.4.99.11); malto-oligosyltrehalose
GH13_5	2.95640970089638e-05	0.000157608935869592	2.77453131579468e-05	α-amylase (EC 3.2.1.1); pullulanase (EC 3.2.1.41); cyclomaltodextrin glucanotransferase (EC 2.4.1.19); cyclomaltodextrinase (EC 3.2.1.54); trehalose-6-phosphate hydrolase (EC 3.2.1.93); oligo-α-glucosidase (EC 3.2.1.10); maltogenic amylase (EC 3.2.1.133); neopullulanase (EC 3.2.1.135); α-glucosidase (EC 3.2.1.20); maltotetraose-forming α-amylase (EC 3.2.1.60); isoamylase (EC 3.2.1.68); glucodextranase (EC 3.2.1.70); maltohexaose-forming α-amylase (EC 3.2.1.98); maltotriose-forming α-amylase (EC 3.2.1.116); branching enzyme (EC 2.4.1.18); trehalose synthase (EC 5.4.99.16); 4-α-glucanotransferase (EC 2.4.1.25); maltopentaose-forming α-amylase (EC 3.2.1.-) ; amylosucrase (EC 2.4.1.4) ; sucrose phosphorylase (EC 2.4.1.7); malto-oligosyltrehalose trehalohydrolase (EC 3.2.1.141); isomaltulose synthase (EC 5.4.99.11); malto-oligosyltrehalose

GH13_9	4.28288415843328e-05	0.000175574909571625	3.27551484371336e-05	α-amylase (EC 3.2.1.1); pullulanase (EC 3.2.1.41); cyclomaltodextrin glucanotransferase (EC 2.4.1.19); cyclomaltodextrinase (EC 3.2.1.54); trehalose-6-phosphate hydrolase (EC 3.2.1.93); oligo-α-glucosidase (EC 3.2.1.10); maltogenic amylase (EC 3.2.1.133); neopullulanase (EC 3.2.1.135); α-glucosidase (EC 3.2.1.20); maltotetraose-forming α-amylase (EC 3.2.1.60); isoamylase (EC 3.2.1.68); glucodextranase (EC 3.2.1.70); maltohexaose-forming α-amylase (EC 3.2.1.98); maltotriose-forming α-amylase (EC 3.2.1.116); branching enzyme (EC 2.4.1.18); trehalose synthase (EC 5.4.99.16); 4-α-glucanotransferase (EC 2.4.1.25); maltopentaose-forming α-amylase (EC 3.2.1.-) ; amylosucrase (EC 2.4.1.4) ; sucrose phosphorylase (EC 2.4.1.7); malto-oligosyltrehalose trehalohydrolase (EC 3.2.1.141); isomaltulose synthase (EC 5.4.99.11); malto-oligosyltrehalose
GH15	3.62233001328251e-05	0.000142316503498619	3.48906785982375e-05	glucoamylase (EC 3.2.1.3); glucodextranase (EC 3.2.1.70); α,α-trehalase (EC 3.2.1.28); dextran dextrinase (EC 2.4.1.2)
GH18	3.50100783604158e-05	0.000135063558408315	2.7949375446178e-05	chitinase (EC 3.2.1.14); lysozyme (EC 3.2.1.17); endo-β-N-acetylglucosaminidase (EC 3.2.1.96); peptidoglycan hydrolase with endo-β-N-acetylglucosaminidase specificity (EC 3.2.1.-); Nod factor hydrolase (EC 3.2.1.-); xylanase inhibitor; concanavalin B; narbonin
GH19	1.29931774171124e-05	8.56094066070306e-05	1.98137515657238e-05	chitinase (EC 3.2.1.14); lysozyme (EC 3.2.1.17)
GH2	2.74984179665012e-05	0.000131661853725563	2.49002694957156e-05	β-galactosidase (EC 3.2.1.23) ; β-mannosidase (EC 3.2.1.25); β-glucuronidase (EC 3.2.1.31); α-L-arabinofuranosidase (EC 3.2.1.55); mannosylglycoprotein endo-β-mannosidase (EC 3.2.1.152); exo-β-glucosaminidase (EC 3.2.1.165); α-L-arabinopyranosidase (EC 3.2.1.-); β-
GH20	2.28061492479109e-05	0.000167327243651761	3.32716531272632e-05	β-hexosaminidase (EC 3.2.1.52); lacto-N-biosidase (EC 3.2.1.140); β-1,6-N-acetylglucosaminidase) (EC 3.2.1.-); β-6-SO3-N-acetylglucosaminidase
GH23	0.000220346127521485	0.000710089948070026	0.000124577605588196	lysozyme type G (EC 3.2.1.17); peptidoglycan lyase (EC 4.2.2.n1) also known in the literature as peptidoglycan lytic transglycosylase; chitinase (EC
GH24	1.21250068915321e-05	0.000127688210354751	3.13853009966817e-05	lysozyme (EC 3.2.1.17)
GH28	2.24541230398329e-05	0.000157629886673282	2.69200314577253e-05	polygalacturonase (EC 3.2.1.15); α-L-rhamnosidase (EC 3.2.1.40); exo-polygalacturonase (EC 3.2.1.67); exo-polygalacturonosidase (EC 3.2.1.82); rhamnogalacturonase (EC 3.2.1.171); rhamnogalacturonan α-1,2-galacturonohydrolase (EC 3.2.1.173); endo-xylogalacturonan hydrolase (EC
GH3	0.000103402945323655	0.00042402793352999	9.75199753341564e-05	β-glucosidase (EC 3.2.1.21); xylan 1,4-β-xylosidase (EC 3.2.1.37); β-glucosylceramidase (EC 3.2.1.45); β-N-acetylhexosaminidase (EC 3.2.1.52); α-L-arabinofuranosidase (EC 3.2.1.55); glucan 1,3-β-glucosidase (EC 3.2.1.58); glucan 1,4-β-glucosidase (EC 3.2.1.74); isoprimeverose-producing oligoxyloglucan hydrolase (EC 3.2.1.120); coniferin β-glucosidase (EC 3.2.1.126); exo-1,3-1,4-glucanase (EC 3.2.1.-); β-N-acetylglucosaminide
GH31	2.56645047492898e-05	0.000138246318450782	2.29778714144193e-05	α-glucosidase (EC 3.2.1.20); α-galactosidase (EC 3.2.1.22); α-mannosidase (EC 3.2.1.24); α-1,3-glucosidase (EC 3.2.1.84); sucrase-isomaltase (EC 3.2.1.48) (EC 3.2.1.10); α-xylosidase (EC 3.2.1.177); α-glucan lyase (EC 4.2.2.13); isomaltosyltransferase (EC 2.4.1.-); oligosaccharide α-1,4-
GH32	3.90752240344107e-05	0.00014302508569648	1.58631830019625e-05	invertase (EC 3.2.1.26); endo-inulinase (EC 3.2.1.7); β-2,6-fructan 6-levanbiohydrolase (EC 3.2.1.64); endo-levanase (EC 3.2.1.65); exo-inulinase (EC 3.2.1.80); fructan β-(2,1)-fructosidase/1-exohydrolase (EC 3.2.1.153); fructan β-(2,6)-fructosidase/6-exohydrolase (EC 3.2.1.154); sucrose:sucrose 1-fructosyltransferase (EC 2.4.1.99); fructan:fructan 1-fructosyltransferase (EC 2.4.1.100); sucrose:fructan 6-fructosyltransferase (EC 2.4.1.10); fructan:fructan 6G-fructosyltransferase (EC 2.4.1.243); levan fructosyltransferase (EC 2.4.1.-); [retaining] sucrose:sucrose 6-
GH33	3.03583790928412e-05	0.000169518404940591	2.25222184603936e-05	sialidase or neuraminidase (EC 3.2.1.18); trans-sialidase (EC 2.4.1.-); anhydrosialidase (EC 4.2.2.15); Kdo hydrolase (EC 3.2.1.-); 2-keto-3-deoxynononic acid hydrolase / KDNase (EC 3.2.1.-)
GH37	0.000153774170041178	0.000314755020023173	0.000233888005508735	α,α-trehalase (EC 3.2.1.28).
GH43_26	3.40887178858897e-05	0.000165916157407933	3.26006222859177e-05	β-xylosidase (EC 3.2.1.37); α-L-arabinofuranosidase (EC 3.2.1.55); xylanase (EC 3.2.1.8); α-1,2-L-arabinofuranosidase (EC 3.2.1.-); exo-α-1,5-L-arabinofuranosidase (EC 3.2.1.-); [inverting] exo-α-1,5-L-arabinanase (EC 3.2.1.-); β-1,3-xylosidase (EC 3.2.1.-); [inverting] exo-α-1,5-L-arabinanase (EC 3.2.1.-); [inverting] endo-α-1,5-L-arabinanase (EC 3.2.1.99); exo-β-1,3-galactanase (EC 3.2.1.145); β-D-galactofuranosidase (EC
GH67	3.12770625740857e-05	0.000150705781973582	4.10314074618505e-05	α-glucuronidase (EC 3.2.1.139); xylan α-1,2-glucuronidase (EC 3.2.1.131)
GH73	4.9169007384931e-05	0.000237920463353412	6.37346974355816e-05	lysozyme (EC 3.2.1.17); mannosyl-glycoprotein endo-β-N-acetylglucosaminidase (EC 3.2.1.96); peptidoglycan hydrolase with endo-β-N-acetylglucosaminidase specificity (EC 3.2.1.-)
GH77	7.31660779267078e-05	0.00027558174667207	5.10556127577553e-05	amylomaltase or 4-α-glucanotransferase (EC 2.4.1.25)
GH8	4.32147381848556e-05	0.00013310146185748	1.99350160835039e-05	chitosanase (EC 3.2.1.132); cellulase (EC 3.2.1.4); licheninase (EC 3.2.1.73); endo-1,4-β-xylanase (EC 3.2.1.8); reducing-end-xylose releasing exo-
GT1	5.01140111899456e-05	0.00014549187227971	3.06426886100029e-05	UDP-glucuronosyltransferase (EC 2.4.1.17); zeatin O-β-xylosyltransferase (EC 2.4.2.40); 2-hydroxyacylsphingosine 1-β-galactosyltransferase (EC 2.4.1.45); N-acylsphingosine galactosyltransferase (EC 2.4.1.47); flavonol 3-O-glucosyltransferase (EC 2.4.1.91); anthocyanidin 3-O-glucosyltransferase (EC 2.4.1.115); sinapate 1-glucosyltransferase (EC 2.4.1.120); indole-3-acetate β-glucosyltransferase (EC 2.4.1.121); flavonol L-rhamnosyltransferase (EC 2.4.1.159); sterol glucosyltransferase (EC 2.4.1.173); UDP-Glc: 4-hydroxybenzoate 4-O-β-glucosyltransferase (EC 2.4.1.194); zeatin O-β-glucosyltransferase (EC 2.4.1.203); limonoid glucosyltransferase (EC 2.4.1.210); UDP-GlcA: baicalein 7-O-β-glucuronosyltransferase (EC 2.4.1.253); UDP-Glc: chalcone 4â€²-O-β-glucosyltransferase (EC 2.4.1.286); ecdysteroid UDP-glucosyltransferase (EC 2.4.1.-); salicylic acid β-glucosyltransferase (EC 2.4.1.-); anthocyanin 3-O-galactosyltransferase (EC 2.4.1.-); anthocyanin 5-O-glucosyltransferase
GT101	1.78303895694628e-05	0.000132149419150633	3.7706966032517e-05	glucosyltransferase (EC 2.4.1.-)
GT19	3.59565892226248e-05	0.000140412406448877	2.61965928061463e-05	lipid-A-disaccharide synthase (EC 2.4.1.182).
GT2	8.27924957234118e-05	0.000312862763278884	4.39337773670379e-05	cellulose synthase (EC 2.4.1.12); chitin synthase (EC 2.4.1.16); dolichyl-phosphate β-D-mannosyltransferase (EC 2.4.1.83); dolichyl-phosphate β-glucosyltransferase (EC 2.4.1.117); N-acetylglucosaminyltransferase (EC 2.4.1.-); N-acetylgalactosaminyltransferase (EC 2.4.1.-); hyaluronan synthase (EC 2.4.1.212); chitin oligosaccharide synthase (EC 2.4.1.-); β-1,3-glucan synthase (EC 2.4.1.34); β-1,4-mannan synthase (EC 2.4.1.-); β-mannosylphosphodecaprenol-mannooligosaccharide α-1,6-mannosyltransferase (EC 2.4.1.199); UDP-Galf: rhamnopyranosyl-N-acetylglucosaminyl-PP-decaprenol β-1,4/1,5-galactofuranosyltransferase (EC 2.4.1.287); UDP-Galf: galactofuranosyl-galactofuranosyl-rhamnosyl-N-acetylglucosaminyl-PP-decaprenol β-1,5/1,6-galactofuranosyltransferase (EC 2.4.1.288); dTDP-L-Rha: N-acetylglucosaminyl-PP-decaprenol α-1,3-

GT20	2.63012504863068e-05	0.000150517462971096	3.45226929769393e-05	α,α-trehalose-phosphate synthase [UDP-forming] (EC 2.4.1.15); Glucosylglycerol-phosphate synthase (EC 2.4.1.213); trehalose-6-P phosphatase (EC 3.1.3.12); [retaining] GDP-valeniol: validamine 7-phosphate valeniolyltransferase (EC 2.-.-.-)
GT26	2.27937122126812e-05	0.000147956052119509	1.5232489871508e-05	UDP-ManNAcA: β-N-acetyl mannosaminuronyltransferase (EC 2.4.1.-); UDP-ManNAc: β-N-acetyl-mannosaminyltransferase (EC 2.4.1.-); UDP-Glc: β-1,4-glucosyltransferase (EC 2.4.1.-); β-1,4-galactosyltransferase (EC 2.4.1.-)
GT28	0.000147606229602471	0.000256386017411772	8.17193244209415e-05	1,2-diacylglycerol 3-β-galactosyltransferase (EC 2.4.1.46); 1,2-diacylglycerol 3-β-glucosyltransferase (EC 2.4.1.157); UDP-GlcNAc: Und-PP-MurAc-pentapeptide β-N-acetylglucosaminyltransferase (EC 2.4.1.227); digalactosyldiacylglycerol synthase (EC 2.4.1.241)
GT30	2.0657456045767e-05	0.000168470274802969	3.28992976342622e-05	CMP-β-KDO: α-3-deoxy-D-manno-octulosonic-acid (KDO) transferase (EC 2.4.99.-).
GT31	0.000110251486927925	5.97003545449486e-05	0.000163882643261779	N-acetyllactosaminide β-1,3-N-acetylglucosaminyltransferase (EC 2.4.1.149); Glycoprotein-N-acetylgalactosamine 3-β-galactosyltransferase (EC 2.4.1.122); fucose-specific β-1,3-N-acetylglucosaminyltransferase (EC 2.4.1.-); globotriosylceramide β-1,3-GalNAc transferase (EC 2.4.1.79); chondroitin synthase (β-1,3-GlcUA and β-1,4-GalNAc transferase (EC 2.4.1.175); chondroitin β-1,3-glucuronyltransferase (EC 2.4.1.226); chondroitin β-1,4-N-acetylgalactosaminyltransferase (EC 2.4.1.-); UDP-Gal: β-galactosylxylosylprotein β-1,3-galactosyltransferase (EC 2.4.1.134);
GT35	9.6646974513074e-05	0.00049158534608307	0.000102381524483845	glycogen or starch phosphorylase (EC 2.4.1.1).
GT4	0.000244362722008928	0.00100150139220755	0.000281514010866094	sucrose synthase (EC 2.4.1.13); sucrose-phosphate synthase (EC 2.4.1.14); α-glucosyltransferase (EC 2.4.1.52); lipopolysaccharide N-acetylglucosaminyltransferase (EC 2.4.1.56); phosphatidylinositol α-mannosyltransferase (EC 2.4.1.57); GDP-Man: Man1GlcNAc2-PP-dolichol α-1,3-mannosyltransferase (EC 2.4.1.132); GDP-Man: Man3GlcNAc2-PP-dolichol/Man4GlcNAc2-PP-dolichol α-1,2-mannosyltransferase (EC 2.4.1.131); digalactosyldiacylglycerol synthase (EC 2.4.1.141); 1,2-diacylglycerol 3-glucosyltransferase (EC 2.4.1.157); diglucosyl diacylglycerol synthase (EC 2.4.1.208); trehalose phosphorylase (EC 2.4.1.231); NDP-Glc: α-glucose α-glucosyltransferase / α,α-trehalose synthase (EC 2.4.1.245); GDP-Man: Man2GlcNAc2-PP-dolichol α-1,6-mannosyltransferase (EC 2.4.1.257); UDP-GlcNAc: 2-deoxystreptamine α-N-acetylglucosaminyltransferase (EC 2.4.1.283); UDP-GlcNAc: ribostamycin α-N-acetylglucosaminyltransferase (EC 2.4.1.285); UDP-Gal α-galactosyltransferase (EC 2.4.1.-); UDP-Xyl α-xylosyltransferase (EC 2.4.2.-); UDP-GlcA α-glucuronyltransferase (EC 2.4.1.-); UDP-Glc α-
GT49	0.000191431419381705	7.93466947839227e-05	0.000269676263637639	β-1,3-N-acetylglucosaminyltransferase (EC 2.4.1.-).
GT5	3.83384185192943e-05	0.00016410105025348	3.71330275548345e-05	UDP-Glc: glycogen glucosyltransferase (EC 2.4.1.11); ADP-Glc: starch glucosyltransferase (EC 2.4.1.21); NDP-Glc: starch glucosyltransferase (EC 2.4.1.242); UDP-Glc: α-1,3-glucan synthase (EC 2.4.1.183) UDP-Glc: α-1,4-glucan synthase (EC 2.4.1.-)
GT51	0.000191121313653374	0.000593167081558382	0.000171138918514927	murein polymerase (EC 2.4.1.129).
GT56	2.90543240786105e-05	0.000148109522820299	2.36761193484075e-05	TDP-Fuc4NAc: lipid II Fuc4NAc transferase (EC 2.4.1.-)
GT60	2.58453233076425e-05	0.000105467545530141	2.40963570277139e-05	UDP-GlcNAc: polypeptide α-N-acetylglucosaminyltransferase (EC 2.4.1.-); UDP-GlcNAc: hydroxyproline polypeptide α-N-
GT8	7.80302100205791e-05	0.000310527080535371	7.86511703289273e-05	lipopolysaccharide α-1,3-galactosyltransferase (EC 2.4.1.44); UDP-Glc: (glucosyl)lipopolysaccharide α-1,2-glucosyltransferase (EC 2.4.1.-); lipopolysaccharide glucosyltransferase 1 (EC 2.4.1.58); glycogenin glucosyltransferase (EC 2.4.1.186); inositol 1-α-galactosyltransferase (galactinol synthase) (EC 2.4.1.123); homogalacturonan α-1,4-galacturonosyltransferase (EC 2.4.1.43); UDP-GlcA: xylan α-glucuronyltransferase (EC 2.4.1.-)
GT81	3.23869714739923e-05	0.000148963655459319	3.30149718007152e-05	NDP-Glc: glucosyl-3-phosphoglycerate synthase (EC 2.4.1.-); NDP-Man: mannosyl-3-phosphoglycerate synthase (EC 2.4.1.-); ADP-Glc: glucosyl-
GT83	3.28174351707695e-05	0.000124317461192854	4.78373580995897e-05	undecaprenyl phosphate-α-L-Ara4N: 4-amino-4-deoxy-β-L-arabinosyltransferase (EC 2.4.2.43); dodecaprenyl phosphate-β-galacturonic acid: lipopolysaccharide core α-galacturonosyl transferase (EC 2.4.1.-)
GT9	0.00021592813481175	0.000941929859652602	0.000237558798492608	lipopolysaccharide N-acetylglucosaminyltransferase (EC 2.4.1.56); heptosyltransferase (EC 2.4.-.-).
Others	0.996453439499898	0.98655177773367	0.996415616598701	Others

Notes: ID: the CAZy family identifier, consisting of a two-letter class code (AA, Auxiliary Activities; CBM, Carbohydrate-Binding Modules; CE, Carbohydrate Esterases; GH, Glycoside Hydrolases; GT, GlycosylTransferases) followed by a family number. Description: the functional definition and representative Enzyme Commission (EC) numbers for each family as curated in the CAZy database.Values represent the relative abundance of genes assigned to each family, calculated as the proportion of annotated genes in a given family relative to the total number of CAZy-annotated genes within each population. MDJ, PB, and SC indicate the three geographic populations of Lissorhoptrus oryzophilus: MDJ, Mudanjiang, Heilongjiang Province; PB, Pingba, Guizhou Province; SC, Yibin, Sichuan Province.

Table S11 Relative abundance of all identified Antimicrobial Resistance Orthology (ARO)-annotated genes across geographic populations of *Lissorhoptrus oryzophilus*

ID	MDJ	PB	SC	Description
ARO:3000024	1.7819793037622e-05	0.00021069270778722	1.60986097914563e-05	patA; PatA is an ABC transporter of Streptococcus pneumoniae that interacts with PatB to confer fluoroquinolone resistance.
ARO:3000027	1.73244018659675e-05	0.000128791719998217	2.84139936039577e-05	emrA; EmrA is a membrane fusion protein, providing an efflux pathway with EmrB and TolC between the inner and outer membranes of E. coli, a Gram-negative bacterium.
ARO:3000074	7.64991709984259e-05	0.000483435712945208	7.58118664540601e-05	emrB; emrB is a translocase in the emrB -TolC efflux protein in E. coli. It recognizes substrates including carbonyl cyanide m-chlorophenylhydrazone (CCCP), nalidixic acid, and thioloactomycin.
ARO:3000168	6.5778767370549e-05	0.000398374530410869	7.51024445161888e-05	tet(D); TetD is a tetracycline efflux pump found exclusively in Gram-negative bacteria.
ARO:3000190	2.29107428301461e-05	0.000141754632985094	2.25623049805057e-05	tetO; TetO is a ribosomal protection protein. It is associated with conjugative plasmids.
ARO:3000191	0.000126482808248991	0.000130040237018782	0.000107275327137461	tetQ; TetQ is a ribosomal protection protein. Its gene is associated with a conjugative transposon and has been found in both Gram-positive and Gram-negative bacteria.
ARO:3000193	0.00016922064040676	0.000274143116288854	0.000130063687341718	tetT; Tet(T) is a ribosomal protection protein of streptococci. It is similar to Tet(Q).
ARO:3000195	0.000207372026875501	1.04309601994306e-05	1.76362344848047e-05	tetB(P); TetB(P) is a tetracycline ribosomal protection protein found on the same operon as tetA(P), a tetracycline efflux protein.
ARO:3000206	1.6799083011023e-05	0.000116500856938582	4.10065211237551e-05	emrK; emrK is a membrane fusion protein that is a homolog of EmrA. Together with the inner membrane transporter EmrY and the outer membrane channel TolC, it mediates multidrug efflux.
ARO:3000216	2.86804125882469e-05	0.000152362544964327	3.30892094364237e-05	acrB; Protein subunit of AcrA-AcrB-TolC multidrug efflux complex. AcrB functions as a herterotrimer which forms the inner membrane component and is primarily responsible for substrate recognition and energy transduction by acting as a drug/proton antiporter.
ARO:3000237	1.77424096202873e-05	0.000154003213651967	3.64660051113046e-05	TolC; TolC is a protein subunit of many multidrug efflux complexes in Gram negative bacteria. It is an outer membrane efflux protein and is constitutively open. Regulation of efflux activity is often at its periplasmic entrance by other components of the efflux complex.
ARO:3000254	3.12222891960025e-05	0.000125300445111097	3.19526940505361e-05	emrY; emrY is a multidrug transport that moves substrates across the inner membrane of the Gram-negative E. coli. It is a homolog of emrB.
ARO:3000263	4.11790815172542e-05	0.000195562180779897	2.80783543816891e-05	marA; In the presence of antibiotic stress, E. coli overexpresses the global activator protein MarA, which besides inducing MDR efflux pump AcrAB, also down- regulates synthesis of the porin OmpF.
ARO:3000391	3.43684312146982e-05	0.000100187375519429	2.02284839824201e-05	norA; NorA is a multidrug efflux pump in Staphylococcus aureus that confers resistance to fluoroquinolones and other structurally unrelated antibiotics like acriflavine. It shares 30% similarity with NorA, and is a structural homolog of Bmr of Bacillus subtilis. It is regulated by arlRS and mgrA, the latter also known as
ARO:3000421	4.26759205079632e-05	0.00014141407720263	2.87817591819848e-05	norB; NorB is a multidrug efflux pump in Staphylococcus aureus that confers resistance to fluoroquinolones and other structurally unrelated antibiotics like tetracycline. It shares 30% similarity with NorB, and is a structural homolog of Blt of Bacillus subtilis. It is regulated by mgrA, also known as NorR.
ARO:3000491	2.77665355952729e-05	0.000146885129583452	2.83690932253373e-05	acrD; AcrD is an aminoglycoside efflux pump expressed in E. coli. Its expression can be induced by indole, and is regulated by baeRS and cpxAR.
ARO:3000501	0.000516856926915223	0.000266503295767356	0.000216613988720489	rpoB2; Due to gene duplication, the genomes of Nocardia species include both rifampin-sensitive beta-subunit of RNA polymerase (rpoB) and rifampin-resistant beta-subunit of RNA polymerase (rpoB2) genes, with ~88% similarity between the two gene products. Expression of the rpoB2 variant results in replacement of rifampin sensitivity with rifampin resistance.

ARO:3000504	1.22415882679032e-05	0.000128955205910797	3.43036115032634e-05	golS; GolS is a regulator activated by the presence of gold, and promotes the expression of the MdsABC
ARO:3000510	0.00025597796191923	0.000298502446187125	0.000150143707063031	Staphylococcus mupB conferring resistance to mupirocin; An alternative isoleucyl-tRNA synthetase conferring resistance to mupirocin.
ARO:3000516	3.6045582775465e-05	0.000117275931779368	3.43292349215037e-05	emrR; EmrR is a negative regulator for the EmrAB-TolC multidrug efflux pump in E. coli. Mutations lead to EmrAB-TolC overexpression.
ARO:3000518	3.56184122942381e-05	0.000104480732476548	4.72838507227503e-05	CRP; CRP is a global regulator that represses MdtEF multidrug efflux pump expression.
ARO:3000521	0.000130416298270401	0.00012083279921462	0.000100196966492758	Staphylococcus mupA conferring resistance to mupirocin; An alternative isoleucyl-tRNA synthetase conferring resistance to mupirocin.
ARO:3000535	0.000960713536729091	0.00407192636364941	0.00100812924739296	macB; MacB is an ATP-binding cassette (ABC) transporter that exports macrolides with 14- or 15-membered lactones. It forms an antibiotic efflux complex with MacA and TolC. macB corresponds to 1 locus in Pseudomonas aeruginosa PAO1 and 1 locus in Pseudomonas aeruginosa LESB58.
ARO:3000549	3.22436596876094e-05	0.000158457695931223	2.75968770729541e-05	adeS; AdeS is a sensor kinase in the AdeRS regulatory system of AdeABC. It is essential for AdeABC
ARO:3000553	9.16377200065307e-05	0.000453605458110439	0.000104172531616956	adeR; AdeR is a positive regulator of AdeABC efflux system. AdeR inactivation leads to susceptibility to aminoglycoside antibiotics.
ARO:3000556	3.20687430089325e-05	0.000190519311614089	4.06556557014058e-05	tet44; Tet44 is a tetracycline resistance gene found in Campylobacter fetus, and binds to the ribosome to confer antibiotic resistance as a ribosomal protection protein.
ARO:3000600	1.36254435067043e-05	0.000143239219333228	3.26533485883639e-05	Erm(34); ErmD confers MLSb phenotype.
ARO:3000620	0.000273064434422124	0.00130344161284828	0.000251469555145033	adeL; AdeL is a regulator of AdeFGH in Acinetobacter baumannii. AdeL mutations are associated with AdeFGH overexpression and multidrug resistance.
ARO:3000656	4.95856212986523e-05	0.00010130473132954	3.31408687321181e-05	AcrS; AcrS is a repressor of the AcrAB efflux complex and is associated with the expression of AcrEF. AcrS is believed to regulate a switch between AcrAB and AcrEF efflux.
ARO:3000676	7.38173110883418e-05	0.000394916472062277	0.000124942615112889	H-NS; H-NS is a histone-like protein involved in global gene regulation in Gram-negative bacteria. It is a repressor of the membrane fusion protein genes acrE, mdtE, and emrK as well as nearby genes of many RND-type multidrug exporters.
ARO:3000768	4.27410049352863e-05	6.63479224984582e-05	4.46026593042557e-05	abeS; AbeS in an efflux pump of the SMR family of transporters found in Acinetobacter baumannii.
ARO:3000774	3.75481318619197e-05	0.000141665203539495	3.36776717834834e-05	adeA; AdeA is the membrane fusion protein of the multidrug efflux complex AdeABC.
ARO:3000775	2.37102669457198e-05	0.000140224685925394	2.40869387267759e-05	adeB; AdeB is the multidrug transporter of the adeABC efflux system.
ARO:3000792	2.82572188521348e-05	0.000132551468803097	4.48259545011421e-05	mdtA; MdtA is the membrane fusion protein of the multidrug efflux complex mdtABC.
ARO:3000793	2.55884969381326e-05	0.000135930176043966	3.91969916895739e-05	mdtB; MdtB is a transporter that forms a heteromultimer complex with MdtC to form a multidrug transporter. MdtBC is part of the MdtABC-TolC efflux complex.
ARO:3000794	3.67965301063339e-05	0.000135098032639997	3.0341668780988e-05	mdtC; MdtC is a transporter that forms a heteromultimer complex with MdtB to form a multidrug transporter. MdtBC is part of the MdtABC-TolC efflux complex. In the absence of MdtB, MdtC can form a homomultimer complex that results in a functioning efflux complex with a narrower drug specificity. mdtC corresponds to 3 loci in Pseudomonas aeruginosa PAO1 (gene name: muxC/muxB) and 3 loci in
ARO:3000805	2.76092656860565e-05	0.000132961739929061	1.80781892754088e-05	OprN; OprN is the outer membrane channel component of the MexEF-OprN multidrug efflux complex.
ARO:3000808	0.000121644526561053	8.84275102530138e-05	8.64047431664031e-05	MexI; MexI is the inner membrane transporter of the efflux complex MexGHI-OpmD.
ARO:3000815	6.85989840294737e-05	0.000300750840640299	5.04092071667328e-05	mgrA; MgrA, also known as NorR, is a regulator for norA, norB, and tet38. It is a positive regulator for norA expression, but is a direct repressor for norB and an indirect repressor of tet38.
ARO:3000816	7.39817530242543e-05	0.000280180454769866	5.63382220055497e-05	mtrA; MtrA is a transcriptional activator of the MtrCDE multidrug efflux pump of Neisseria gonorrhoeae.

ARO:3000823	1.9738489592605e-05	0.000130715928814876	1.600135312659e-05	ramA; RamA (resistance antibiotic multiple) is a positive regulator of AcrAB-TolC and leads to high level multidrug resistance in Klebsiella pneumoniae, Salmonella enterica, and Enterobacter aerugenes, increasing the expression of both the mar operon as well as AcrAB. RamA also decreases OmpF expression.
ARO:3000826	3.45583672942633e-05	0.000120391504590995	2.84055689567942e-05	sdiA; SdiA is a cell division regulator that is also a positive regulator of AcrAB only when it's expressed from a plasmid. When the sdiA gene is on the chromosome, it has no effect on expression of acrAB
ARO:3000828	3.20793273927446e-05	0.000130132308820568	2.97138308532826e-05	baeR; BaeR is a response regulator that promotes the expression of MdtABC and AcrD efflux complexes.
ARO:3000829	9.77904245705496e-05	0.000406427281977157	7.33522796971238e-05	baeS; BaeS is a sensor kinase in the BaeSR regulatory system. While it phosphorylates BaeR to increase its activity, BaeS is not necessary for overexpressed BaeR to confer resistance.
ARO:3000830	0.000139627173748025	0.000747805215267451	0.000134351775046205	cpxA; CpxA is a membrane-localized sensor kinase that is activated by envelope stress. It starts a kinase cascade that activates CpxR, which promotes efflux complex expression.
ARO:3000832	6.03922111887819e-05	0.000402558731932108	8.74272396134889e-05	evgA; EvgA, when phosphorylated, is a positive regulator for efflux protein complexes emrKY and mdtEF. While usually phosphorylated in a EvgS dependent manner, it can be phosphorylated in the absence of EvgS when overexpressed.
ARO:3000833	0.000269435582020142	0.00102522774774299	0.000286110927459433	evgS; EvgS is a sensor protein that phosphorylates the regulatory protein EvgA. evgS corresponds to 1 locus in Pseudomonas aeruginosa PAO1 and 1 locus in Pseudomonas aeruginosa LESB58.
ARO:3000838	0.00023256278504672	0.000468078641743232	0.000159586104359636	arlR; ArlR is a response regulator that binds to the norA promoter to activate expression. ArlR must first be phosphorylated by ArlS.
ARO:3000839	2.65484473730169e-05	0.000168582734683516	3.26595865076226e-05	arlS; ArlS is a protein histidine kinase that phosphorylates ArlR, a promoter for norA expression.
ARO:3001209	0.000183677089003441	0.000349347631937933	0.000174107834229279	mecC; A foreign PBP2a acquired by lateral gene transfer that able to perform peptidoglycan synthesis in the presence of beta-lactams.
ARO:3001301	2.28340520817821e-05	0.000123472434679145	3.04956517587624e-05	RlmA(II); RlmA(II) is a methyltransferase found in Streptococcus pneumoniae and confers resistance to mycinamicin, tylosin and lincosamides. Specifically, this enzyme adds a methyl group to guanosine 748 (E. coli numbering) of 23S ribosomal RNA.
ARO:3001327	2.63379166791883e-05	0.000137939487022897	2.41569057369394e-05	MdtK; A multidrug and toxic compound extrusions (MATE) transporter conferring resistance to norfloxacin, doxorubicin and acriflavine.
ARO:3001328	5.98247403562564e-05	0.000296114798936067	7.56668315150979e-05	Escherichia coli mdfA; Multidrug efflux pump in E. coli. This multidrug efflux system was originally identified as the Cmr/CmlA chloramphenicol exporter.
ARO:3001329	5.11169562548503e-05	0.000270369313100472	4.94938515807772e-05	mdtG; The MdtG protein, also named YceE, appears to be a member of the major facilitator superfamily of transporters, and it has been reported, when overexpressed, to increase fosfomycin and deoxycholate resistances. mdtG is a member of the marA-soxS-rob regulon.
ARO:3002108	2.74959579568914e-05	0.00011405904155874	3.41941656870164e-05	CMY-95; CMY-95 is a beta-lactamase. From the Lahey list of CMY beta-lactamases.
ARO:3002117	2.42967360567452e-05	0.000125385643741731	3.18158239660163e-05	CMY-105; CMY-105 is a beta-lactamase. From the Lahey list of CMY beta-lactamases.
ARO:3002494	1.81771056522378e-05	0.000164966926337707	3.88525001880779e-05	SRT-2; SRT-2, isolated from Serratia marcescens, is a chromosomal beta-lactamase that confers resistance to
ARO:3002522	0.000190829597065946	0.00094223682462352	0.000209663865068094	novA; A type III ABC transporter, identified on the novobiocin biosynthetic gene cluster, involved in the transport and resistance of novobiocin.
ARO:3002570	2.84613581379238e-05	8.45294856089626e-05	1.91819084168433e-05	AAC(6')-Iz; AAC(6')-Iz is a chromosomal-encoded aminoglycoside acetyltransferase in S. maltophilia
ARO:3002591	2.66850564720022e-05	9.91266975614022e-05	2.52633203769285e-05	AAC(6')-Ib-Suzhou; AAC(6')-Ib-Suzhou is an aminoglycoside acetyltransferase in E. cloacae and K.
ARO:3002597	7.79864096439273e-05	5.96016461369156e-05	3.62139944319834e-05	AAC(6')-Ie-APH(2")-Ia; AAC(6')-Ie-APH(2")-Ia is an aminoglycoside acetyltransferase encoded by plasmids and transposons in S. aureus, E. faecalis, E. faecium and Staphylococcus warneri
ARO:3002681	7.20424263853827e-06	0.000120929203347155	2.31255897712174e-05	catB9; catB9 is a chromosome-encoded variant of the cat gene found in Vibrio cholerae

ARO:3002696	3.52341452734926e-05	0.000132256598631657	1.63812230041482e-05	cmlA6; cmlA6 is a plasmid-encoded chloramphenicol exporter that is found in Pseudomonas aeruginosa
ARO:3002698	3.92784619856243e-05	0.00013725205982024	2.84963288004367e-05	cmlB; cmlB is a plasmid-encoded chloramphenicol exporter that is found in Enterobacter aerogenes
ARO:3002700	6.04967831285831e-05	0.00026019823089998	5.8299714354452e-05	cmlv; cmlv is a chromosome-encoded chloramphenicol phoshotransferase that is found in Streptomyces
ARO:3002703	4.52519116738675e-05	0.000333021312247901	5.8088313180393e-05	cmx; cmx is a plasmid or transposon-encoded chloramphenicol exporter that is found in Corynebacterium striatum and Pseudomonas aeruginosa.
ARO:3002705	2.14843454745398e-05	0.0001222681402036	3.70366217411294e-05	floR; floR is a plasmid or chromosome-encoded chloramphenicol exporter that is found in Bordetella bronchiseptica, Escherichia coli, Klebsiella pneumoniae,\nSalmonella typhimurium DT104 and Vibrio
ARO:3002813	3.72372393482202e-05	0.000149550802846026	4.41248871116457e-05	lmrB; lmrB is a chromosomally-encoded efflux pump that confers resistance to lincosamides in Bacillus
ARO:3002815	2.01245732139289e-05	0.000122990672365947	4.22672738139582e-05	clbB; clbB is a plasmid-encoded cfr gene found in Bacillus brevis
ARO:3002827	6.3539919580347e-05	0.000160196262342728	6.73417518042636e-05	tlrC; tlrC is an ABC-F subfamily protein found in Streptomyces fradiae and confers resistance to mycinamicin, tylosin and lincosamides. tlrC is found in the tylosin biosynthetic cluster and is one mechanism by which S. fradiae protects itself from self-destruction when producing this macrolide.
ARO:3002830	2.56938536228344e-05	6.81325127990346e-05	1.1887337668173e-05	vgaALC; vgaALC is an ABC-F subfamily protein expressed in staphylococci that confers resistance to streptogramin A antibiotics and related compounds. It is associated with plasmid DNA.
ARO:3002874	1.86218650129444e-05	0.000112162926493149	1.44922742859913e-05	FosC2; FosC2 is an enzyme that phosphorylates fosfomycin to confer resistance in Escherichia coli
ARO:3002881	0.000245548921382653	0.000758273149032636	0.000225898798203998	lmrC; lmrC is an ABC-F subfamily protein that confers resistance to lincosamides in Streptomyces lincolnensis and Lactococcus lactis. It can dimerize with lmrD
ARO:3002892	2.77166213318073e-05	0.00014489549726916	1.57779770007467e-05	otr(B); otr(B) is a tetracycline resistance efflux pump found in Streptomyces rimosus
ARO:3002907	0.000104280591962475	8.60293764797196e-05	9.95336642134675e-05	vanE; VanE is a D-Ala-D-Ala ligase homolog that can synthesize D-Ala-D-Ser, an alternative substrate for peptidoglycan synthesis that reduces vancomycin binding affinity in Enterococcus faecalis
ARO:3002909	3.41440310523226e-05	0.000144074142189558	2.8678530784282e-05	vanG; VanG is a D-Ala-D-Ala ligase homolog that can synthesize D-Ala-D-Ser, an alternative substrate for peptidoglycan synthesis that reduces vancomycin binding affinity in Enterococcus faecalis
ARO:3002910	4.70785636828825e-05	0.000159654399701504	2.99856547121731e-05	vanL; VanL is a D-Ala-D-Ala ligase homolog that can synthesize D-Ala-D-Ser, an alternative substrate for peptidoglycan synthesis that reduces vancomycin binding affinity in Enterococcus faecalis
ARO:3002913	0.000128807160650833	7.00706085849014e-05	7.57634042940565e-05	vanO; VanO is a D-Ala-D-Ala ligase homolog that can synthesize D-Ala-D-Lac, an alternative substrate for peptidoglycan synthesis that reduces vancomycin binding affinity. It is associated with both vancomycin and teicoplanin resistance.
ARO:3002914	2.82412734990302e-05	0.000105457173019453	2.34560005931923e-05	vanJ; vanJ is a novel membrane protein that confers resistance to teicoplanin and its derivatives in Streptomyces coelicolor by recycling undecaprenol pyrophosphate during cell wall biosynthesis.
ARO:3002921	2.99133881817579e-05	0.000179935805154504	3.18162127779744e-05	vanRB; vanRB is a vanR variant found in the vanB gene cluster
ARO:3002924	2.33659414103754e-05	0.000141449422260312	1.73455746601671e-05	vanRE; vanRE is a vanR variant found in the vanE gene cluster
ARO:3002925	0.000134172596573843	0.000113590670526216	9.37642653918542e-05	vanRF; vanRF is a vanR variant found in the vanF gene cluster
ARO:3002930	3.40837109996415e-05	0.000134349236029095	3.07960828577316e-05	vanRO; vanRO is a vanR variant found in the vanO gene cluster
ARO:3002939	2.60941977154276e-05	0.000125973284699971	3.98561897818014e-05	vanSM; vanSM is a vanS variant found in the vanM gene cluster
ARO:3002941	2.6354252458627e-05	0.00013249435323465	4.33448770352237e-05	vanSO; vanSO is a vanS variant found in the vanO gene cluster
ARO:3002943	1.67583330245171e-05	0.000143541604262311	3.78310953847836e-05	vanHB; vanHB is a vanH variant in the vanB gene cluster
ARO:3002944	5.88465421486774e-05	0.000331170550994333	5.39745630269089e-05	vanHD; vanHD is a vanH variant in the vanD gene cluster
ARO:3002945	5.34971971462866e-05	0.000290216358828926	6.18709635237546e-05	vanHF; vanHF is a vanH variant in the vanF gene cluster
ARO:3002948	1.70989264588678e-05	0.000102447592312931	1.5825998356723e-05	vanHO; vanHO is a vanH variant in the vanO gene cluster
ARO:3002974	1.51721338230644e-05	0.000124857354554089	3.21666551371032e-05	vanTrL; vanTrL is a vanT variant found in the vanL gene cluster. vanTrL codes for the racemase component

ARO:3002985	9.80149187458055e-05	0.000413439429629869	0.000114076491828206	arnA; arnA modifies lipid A with 4-amino-4-deoxy-L-arabinose (Ara4N) which allows gram-negative bacteria to resist the antimicrobial activity of cationic antimicrobial peptides and antibiotics such as polymyxin. arnA is found in E. coli and P. aeruginosa.
ARO:3002986	1.92357561821674e-05	0.000175096375567352	1.36791310183894e-05	bacA; bacA is a gene that recycles undecaprenyl pyrophosphate during cell wall biosynthesis which confers resistance to bacitracin
ARO:3002987	0.000294564999614478	0.000795899367009899	0.000272688143751467	bcrA; bcrA is an ABC transporter found in Bacillus licheniformis that confers bacitracin resistance
ARO:3003017	0.000136006197460758	9.78117312288001e-05	0.000111531756548364	dfrA21; dfrA21 is an integron-encoded dihydrofolate reductase found in Salmonella enterica
ARO:3003046	2.56581157299979e-05	0.000149376490606189	3.28831679935271e-05	qacA; qacA is a subunit of the qac multidrug efflux pump
ARO:3003049	5.30542651360673e-05	0.000285968947368061	5.80504351419314e-05	rosB; rosB is part of an efflux pump/potassium antiporter system (RosAB) in Yersinia that confers resistance to cationic antimicrobial peptides such as polymyxin B.
ARO:3003057	2.86992172326909e-05	0.000107560080152063	2.78464346150399e-05	smeF; smeF is an outer membrane multidrug efflux protein of the smeDEF complex in Stenotrophomonas
ARO:3003066	1.75474291135785e-05	0.000145318005780889	1.9261452130749e-05	smeR; smeR is the responder component of a two component signal transduction system that includes smeS
ARO:3003105	2.58449447196405e-05	0.000175099703217051	2.14233619879871e-05	dfrA3; dfrA3 is an integron-encoded dihydrofolate reductase found in Escherichia coli
ARO:3003250	2.95682912229268e-05	0.000126729055272679	1.36085550325391e-05	bcrC; bcrC is an undecaprenyl pyrophosphate phosphatase that has a role in bacitracin resistance if it is overexpressed. It is isolated from Bacillus subtilis.
ARO:3003318	0.000192017550316304	0.000414476838170615	0.000128773979122965	Streptomyces rishiriensis parY mutant conferring resistance to aminocoumarin; point mutation on the Streptomyces rishiriensis parY resulting in aminocoumarin resistance
ARO:3003440	0.000122117045361085	1.23019966510582e-05	1.20680002676585e-05	mecB; A foreign PBP2 acquired by lateral gene transfer that able to perform peptidoglycan synthesis in the presence of beta-lactams.
ARO:3003548	2.58948967104897e-05	0.000145690677617784	4.67320720965913e-05	mdtN; Multidrug resistance efflux pump. Could be involved in resistance to puromycin, acriflavine and tetraphenylarsonium chloride.
ARO:3003576	9.49970906511698e-05	0.000469267863461306	0.000109857960742336	eptA; PmrC mediates the modification of Lipid A by the addition of 4-amino-4-deoxy-L-arabinose (L-Ara4N) and phosphoethanolamine, resulting in a less negative cell membrane and decreased binding of
ARO:3003578	8.83297979232977e-05	0.000258923013173956	6.9403177656105e-05	PmrF; PmrF is required for the synthesis and transfer of 4-amino-4-deoxy-L-arabinose (Ara4N) to Lipid A, which allows gram-negative bacteria to resist the antimicrobial activity of cationic antimicrobial peptides and antibiotics such as polymyxin. pmrF corresponds to 1 locus in Pseudomonas aeruginosa PAO1 and 1 locus in Pseudomonas aeruginosa LESB58.
ARO:3003583	2.80661396571389e-05	0.000158844094367987	3.54049375062244e-05	basS; Histidine protein kinase sensor Lipid A modification gene; part of a two-component system involved in polymyxin resistance that senses high extracellular Fe(2+)
ARO:3003665	5.13102305125453e-05	0.000272992624883544	3.19993315686238e-05	NmcR; NmcR is a homolog of the LysR regulator found in Enterobacter cloacae that contribute to the regulation of NmcA beta-lactamase
ARO:3003670	3.235409924828e-05	0.000127288693209263	1.10731241433602e-05	PEDO-1; PEDO-1 is a subclass B3 metallo-beta lactamase isolated from Pedobacter roseus exhibiting resistance to carbapenems.
ARO:3003680	3.87869300658472e-05	0.000135692889849809	2.92933391860619e-05	TriB; TriB is a membrane protein that is fused to TriA and both are required for the triclosan efflux pump function of TriABC-OpmH in P. aeruginosa.
ARO:3003681	3.64798658306702e-05	0.000141634785518072	2.98222242849736e-05	TriC; TriC is a resistance nodulation cell division (RND) transporter that is a part of TriABC-OpmH, a triclosan-specific efflux protein.
ARO:3003693	0.000107057468014642	7.69152246538226e-05	9.20361700163331e-05	MexK; mexK is the inner membrane resistance-nodulation-cell division (RND) transporter in the MexJK multidrug efflux protein.

ARO:3003728	3.35554703206762e-05	0.00015935047145982	4.09606561550168e-05	vanRI; VanRI is the regulatory transcriptional activator in the VanSR regulator within the VanI glycopeptide resistance gene cluster.
ARO:3003730	0.000355360217190546	0.000468155473042591	0.000222230883287724	Bifidobacterium ileS conferring resistance to mupirocin; Bifidobacteria have an intrinsically resistant form of ileS (isoleucyl-tRNA synthetase) that confers resistance to mupirocin.
ARO:3003746	9.68879518639997e-05	0.000424458339759134	9.00941725107772e-05	optrA; optrA encodes an ABC-F subfamily gene conferring resistance to oxazolidinones that was isolated from a plasmid in Enterococcus faecalis and Enterococcus faecium.
ARO:3003748	0.000304785271278282	0.00102357718089954	0.000326588302570195	oleC; oleC is an ABC transporter isolated from Streptomyces antibioticus and is involved in oleandomycin
ARO:3003749	1.9403291804733e-05	0.000140276506989846	3.25955355131925e-05	salA; salA is an ABC-F subfamily protein gene isolated from the chromosome of Staphylococcus sciuri conferring resistance to lincosamides and streptogramins.
ARO:3003772	1.75176824674996e-05	0.000129092439012422	2.26931859857044e-05	Brucella suis mprF; MprF is a integral membrane protein that modifies the negatively-charged phosphatidylglycerol on the membrane surface. This confers resistance to cationic peptides that disrupt the
ARO:3003785	0.000108750657802532	0.000109991007532306	9.33966575058467e-05	Chlamydia trachomatis intrinsic murA conferring resistance to fosfomycin; Chlamydia murA confers intrinsic resistance to fosfomycin. The presence of an aspartic acid residue in place of the critical cysteine at position 119 that enables fosfomycin binding is believed to be responsible for this intrinsic resistance.
ARO:3003801	0.000210174278660113	0.000356902035719518	0.00015797397695662	bcr-1; Transmembrane protein which expels bicyclomycin from the cell, leading to bicyclomycin resistance. Identified in Pseudomonas aeruginosa strains responsible for outbreaks in Brazil, often appearing with blaSPM-1, another bicyclomycin resistance gene
ARO:3003841	7.71625685450906e-05	0.000329283497493119	9.17875676259126e-05	kdpE; kdpE is a transcriptional activator that is part of the two-component system KdpD/KdpE that is studied for its regulatory role in potassium transport and has been identified as an adaptive regulator involved in the virulence and intracellular survival of pathogenic bacteria. kdpE regulates a range of virulence loci through
ARO:3003908	8.14139793193941e-05	0	2.05873999326905e-05	Erm(47); Chromosome-encoded gene conferring MLSB resistance. Identified from Helcococcus kunzii.
ARO:3003922	0.000133701996235223	0.000173836182783143	0.000106901677527681	oqxA; RND efflux pump conferring resistance to fluoroquinolone
ARO:3003923	2.02458610670283e-05	0.000150658029202447	3.26672650541206e-05	oqxB; RND efflux pump conferring resistance to fluoroquinolone
ARO:3003948	1.82013414635532e-05	0.00013911702212377	5.43601866773232e-05	efrA; efrA is a part of the EfrAB efflux pump, and both efrA and efrB are necessary to confer drug resistance.
ARO:3003950	0.000712419501880111	0.00205553721824654	0.00061944240132834	msbA; MsbA is a multidrug resistance transporter homolog from E. coli and belongs to a superfamily of transporters that contain an adenosine triphosphate (ATP) binding cassette (ABC) which is also called a nucleotide-binding domain (NBD). MsbA is a member of the MDR-ABC transporter group by sequence homology. MsbA transports lipid A, a major component of the bacterial outer cell membrane, and is the only bacterial ABC transporter that is essential for cell viability.
ARO:3003952	0.000116718038829975	0.000179182437198948	9.89727296375562e-05	YojI; YojI mediates resistance to the peptide antibiotic microcin J25 when it is expressed from a multicopy vector. YojI is capable of pumping out microcin molecules. The outer membrane protein TolC in addition to YojI is required for export of microcin J25 out of the cell. Microcin J25 is thus the first known substrate for
ARO:3003953	2.85223229036674e-05	0.000163512823121265	3.55470372258808e-05	hmrM; hmrM is a multidrug efflux pump belonging to the MATE family and functions as a Na+/drug
ARO:3003961	5.97029347892439e-05	0.000369541330392021	9.22393003530758e-05	farA; farA is the membrane fusion protein that is part of the farAB efflux pump.
ARO:3003962	4.04863533258078e-05	0.000126594747839624	2.92295707686153e-05	farB; farB is the cytoplasmic transporter protein that is part of the farAB efflux pump. farB corresponds to 3 loci in Pseudomonas aeruginosa PAO1 and 3 loci in Pseudomonas aeruginosa LESB58.
ARO:3003964	6.71329714349156e-05	0.000210307052576577	6.20134593240517e-05	hp1181; hp1181 is a translocase that is part of the MFS efflux pump family. It is found in H. pylori and has role in the active efflux of antibiotics.
ARO:3003969	2.57672832595173e-05	0.000195794518196888	3.03527831796893e-05	lmrP; lmrP is a proton motive force-dependent drug transporter that is part of the MFS efflux pump family.

ARO:3003980	0.000322952836517632	0.00163533805438107	0.000361472787747855	tetA(48); TetA(48) is a Tetracycline efflux pump described in Paenibacillus sp. LC231, a strain of Paenibacillus isolated from Lechuguilla Cave, NM, USA. Described by Pawlowski et al. 2016.
ARO:3003982	2.35741751203439e-05	0.000148555301581851	2.78067464421476e-05	LlmA 23S ribosomal RNA methyltransferase; Lincosamide (Clindamycin) resistant (putative) ribosomal methyltransferase related to the RlmK 23S rRNA methyltransferase COG family. Detected in Paenibacillus sp. LC231, an isolated strain of Paenibacillus found in Lechuguilla Cave, NM, USA. Described by
ARO:3003986	5.25913297477276e-05	0.000315129082002715	4.99567655127627e-05	TaeA; Pleuromutilin (Tiamulin) ABC efflux pump found in Paenibacillus sp. LC231, a strain of Paenibacillus isolated from Lechuguilla Cave, NM, USA. Confers resistance to pleuromutilin antibiotics. Described by
ARO:3003992	0.000145698255062948	0.00021966205789978	0.00012785561010207	rphB; rphB is a rifampin phosphotransferase protein found in Paenibacillus sp. LC231, a strain of Paenibacillus isolated from Lechuguilla Cave, NM, USA. Confers resistance to rifamycin antibiotics, specifically rifampin, through rifampin inactivation. Described by Pawlowski et al. 2016.
ARO:3004032	2.49719411707479e-05	0.000141946386787051	4.1608660259927e-05	tetA(46); tetA(46) is a subunit of tetAB(46), a heterodimeric ABC transporter, that is required for conferring tetracycline resistance in Streptococcus australis isolated from the oral cavity.
ARO:3004033	4.59100713018311e-05	0.000206109784411284	4.11406363808082e-05	tetB(46); tetB(46) is a subunit of tetAB(46), a heterodimeric ABC transporter, that is required for conferring tetracycline resistance in Streptococcus australis isolated from the oral cavity.
ARO:3004035	5.26788429098628e-05	0.000332703762286231	5.12350038590293e-05	tetA(60); tetA(60) is a subunit of tetAB(60), an ABC transporter that confers resistance to tetracycline and tigercycline identified by screening a human saliva metagenomic library in Escherichia coli, which is required
ARO:3004036	2.10732940460842e-05	0.000101230424638661	2.08394016558024e-05	tetB(60); tetB(60) is a subunit of tetAB(60), an ABC transporter that confers resistance to tetracycline and tigercycline identified by screening a human saliva metagenomic library in Escherichia coli, which is required
ARO:3004043	3.31837971882405e-05	0.000143922375852269	1.84633354601141e-05	Escherichia coli acrA; AcrA is a subunit of the AcrAB-TolC multidrug efflux system that in E. coli.
ARO:3004054	0.000213035202722099	0.000472932092780016	0.000158319176625503	Pseudomonas aeruginosa CpxR; CpxR is directly involved in activation of expression of RND efflux pump MexAB-OprM in P. aeruginosa. CpxR is required to enhance mexAB-oprM expression and drug resistance, in the absence of repressor MexR.
ARO:3004107	1.76893345747435e-05	0.000189264061033371	2.005969079679e-05	Pseudomonas aeruginosa soxR; SoxR is a redox-sensitive transcriptional activator that induces expression of a small regulon that includes the RND efflux pump-encoding operon mexGHI-opmD. SoxR was shown to be activated by pyocyanin.
ARO:3004122	0.000354006886239847	0.000614546822312465	0.000152372171983962	Klebsiella pneumoniae OmpK37; Klebsiella pneumoniae outer membrane porin protein. Is preferentially detected in porin-deficient strains. Functional characterization of this new porin revealed a narrower pore than those of porins OmpK35 and OmpK36, which did not allow penetration by certain 尾-lactams. Also, when a resistant strain expresses porin OmpK37 is less susceptible to cefotaxime and cefoxitin than when it is
ARO:3004185	1.68523454299315e-05	0.000160994165250807	4.58524542981055e-05	mecD; mecD is a PBP2a variant identified on a genomic resistance island in Macrococcus caseolyticus. MecD confers resistance to methicillin and other beta-lactam antibiotics through the production of an alternative low-affinity PBP. First described by Schwendener et al. 2017 and identified from canine and
ARO:3004361	0.000175388369090627	0.000294860598862391	0.000140835433511204	sul4; sul4 is a dihydropteroate synthase gene and mobile sulfonamide resistance gene shown to confer resistance when expressed in E. coli.
ARO:3004379	3.02828776259836e-05	0.000134567928670219	2.80870016921481e-05	QepA4; A plasmid-mediate quinolone efflux pump variant described in Escherichia coli. QepA4 confers resistance to quinolone and fluoroquinolone antibiotics by expulsion from the cell. Described by Manageiro
ARO:3004442	2.22768261102276e-05	0.000150979058130162	1.99470479048103e-05	tet(W/N/W); tet(W/N/W) is a mosaic tetracycline resistance gene and ribosomal protection protein.

ARO:3004480	0.000145075748373418	0.000156314272289618	5.43908502385796e-05	Bifidobacterium adolescentis rpoB mutants conferring resistance to rifampicin; Bifidobacterium are antibiotic resistant probiotics are prescribed to upkeep the population beneficial bacteria in the gut microbiome. However, horizontal gene transfer among gut microbes could create harmful antibiotic-resistant pathogenic bacteria, such as Mycobacterium tuberculosis. Lokesh et al. analyzed Bifidobacterium antitubercular drug resistance and mutations in rpo尾. They found that B. animalis, B. longum and B. adolescentis showed considerable resistance to pyrazinamide, isoniazid, and streptomycin, while B. adolescentis had mutations both in the rifampicin (RIF) pocket and in regions outside the pockets, and also
Others	0.987209213200817	0.959296959489259	0.988787135193931	Others

Notes: ID: ARO identifier. Description: the functional annotation and associated resistance phenotype as curated in the CARD database. Values represent the relative abundance of genes mapped to each ARO, calculated as the proportion of annotated genes relative to the total number of predicted protein-coding genes per population. MDJ, PB, and SC indicate the three geographic populations of *Lissorhoptrus oryzophilus* : MDJ, Mudanjiang (Heilongjiang); PB, Pingba (Guizhou); SC, Yibin (Sichuan).
